# Integrated multi-omics for rapid rare disease diagnosis on a national scale

**DOI:** 10.1038/s41591-023-02401-9

**Published:** 2023-06-08

**Authors:** Sebastian Lunke, Sophie E. Bouffler, Chirag V. Patel, Sarah A. Sandaradura, Meredith Wilson, Jason Pinner, Matthew F. Hunter, Christopher P. Barnett, Mathew Wallis, Benjamin Kamien, Tiong Y. Tan, Mary-Louise Freckmann, Belinda Chong, Dean Phelan, David Francis, Karin S. Kassahn, Thuong Ha, Song Gao, Peer Arts, Matilda R. Jackson, Hamish S. Scott, Stefanie Eggers, Simone Rowley, Kirsten Boggs, Ana Rakonjac, Gemma R. Brett, Michelle G. de Silva, Amanda Springer, Michelle Ward, Kirsty Stallard, Cas Simons, Thomas Conway, Andreas Halman, Nicole J. Van Bergen, Tim Sikora, Liana N. Semcesen, David A. Stroud, Alison G. Compton, David R. Thorburn, Katrina M. Bell, Simon Sadedin, Kathryn N. North, John Christodoulou, Zornitza Stark

**Affiliations:** 1 Victorian Clinical Genetics Services, Murdoch Children’s Research Institute, Melbourne, Victoria Australia; 2 Medicine, Dentistry and Health Sciences, University of Melbourne, Melbourne, Victoria Australia; 3 Australian Genomics, Melbourne, Victoria Australia; 4 Genetic Health Queensland, Royal Brisbane and Women’s Hospital, Brisbane, Queensland Australia; 5 Sydney Children’s Hospitals Network – Westmead, Sydney, New South Wales Australia; 6 Children’s Hospital Westmead Clinical School, University of Sydney, Sydney, New South Wales Australia; 7 Sydney Children’s Hospitals Network – Randwick, Sydney, New South Wales Australia; 8 Medicine and Health, University of New South Wales, Sydney, New South Wales Australia; 9 Monash Genetics, Monash Health, Melbourne, Victoria Australia; 10 Department of Paediatrics, Monash University, Melbourne, Victoria Australia; 11 Paediatric and Reproductive Genetics Unit, Women’s and Children’s Hospital, North Adelaide, South Australia Australia; 12 Department of Genetics and Molecular Pathology, SA Pathology, Adelaide, South Australia Australia; 13 Adelaide Medical School, The University of Adelaide, Adelaide, South Australia Australia; 14 Tasmanian Clinical Genetics Service, Tasmanian Health Service, Hobart, Tasmania Australia; 15 School of Medicine and Menzies Institute for Medical Research, University of Tasmania, Hobart, Tasmania Australia; 16 Genetic Services of Western Australia, Perth, Western Australia Australia; 17 Department of Clinical Genetics, The Canberra Hospital, Canberra, Australian Capital Territory Australia; 18 Centre for Cancer Biology, An alliance between SA Pathology and the University of South Australia, Adelaide, South Australia; 19 UniSA Clinical and Health Sciences, University of South Australia, Adelaide, South Australia Australia; 20 Murdoch Children’s Research Institute, Melbourne, Victoria Australia

**Keywords:** Diseases, Genetics research, Translational research

## Abstract

Critically ill infants and children with rare diseases need equitable access to rapid and accurate diagnosis to direct clinical management. Over 2 years, the Acute Care Genomics program provided whole-genome sequencing to 290 families whose critically ill infants and children were admitted to hospitals throughout Australia with suspected genetic conditions. The average time to result was 2.9 d and diagnostic yield was 47%. We performed additional bioinformatic analyses and transcriptome sequencing in all patients who remained undiagnosed. Long-read sequencing and functional assays, ranging from clinically accredited enzyme analysis to bespoke quantitative proteomics, were deployed in selected cases. This resulted in an additional 19 diagnoses and an overall diagnostic yield of 54%. Diagnostic variants ranged from structural chromosomal abnormalities through to an intronic retrotransposon, disrupting splicing. Critical care management changed in 120 diagnosed patients (77%). This included major impacts, such as informing precision treatments, surgical and transplant decisions and palliation, in 94 patients (60%). Our results provide preliminary evidence of the clinical utility of integrating multi-omic approaches into mainstream diagnostic practice to fully realize the potential of rare disease genomic testing in a timely manner.

## Abstract (web summary)

A report from the Australian Acute Care Genomics programme shows that the integration of rapid whole-genome sequencing and multi-omic analyses informs diagnoses and treatment decisions in a prospective cohort of 290 critically ill infants and children.

## Main

Genomic testing is transforming rare disease diagnosis. The unparalleled acceleration of rare disease gene discovery, dramatic reductions in the cost of genomic sequencing and government investments to drive clinical application have significantly improved both diagnostic rates and the timeliness of genetic diagnosis^1^. Genomic testing is around five times more likely to achieve a diagnosis than previous ‘gold standard’ tests such as chromosomal microarray^2^ and is increasingly delivered with rapid turnaround times to guide clinical management in real time^3–6^. The diagnostic and clinical utility, as well as cost-effectiveness of rapid genomic testing in critically ill infants and children with suspected genetic conditions are now well established, with over 30 studies totaling 2,000 patients published worldwide^5,7^. Multiple healthcare systems have funded this type of testing as standard of care^8^.

Targeted panels, exomes and genomes have all been used in rapid genomic testing programs, but as whole-genome sequencing (WGS) becomes increasingly deliverable at scale across healthcare systems^9,10^, it is expected to supersede other modalities. WGS can comprehensively assess multiple variant types, including structural and copy-number variants (CNVs), short tandem repeats (STRs) and mitochondrial variants, in a single test and the shorter sample preparation times will further decrease time to result. Despite these advantages, adoption is hampered by higher costs, at times immature analysis tools and lack of robust evidence for a significant increase in diagnostic yield^11,12^. In addition, the improved analytical performance of WGS and ever earlier test initiation driven by rapid diagnosis programs exacerbate existing interpretive challenges. Improvements in bioinformatic analysis and integration of multi-omic approaches will further optimize diagnostic performance, as there is a growing appreciation for the need to closely integrate discovery research with clinical testing to maximize diagnostic benefits for current and future patients^13^. However, these approaches rarely form part of current diagnostic practice and are typically the domain of specialized research programs, limiting access.

In the present study, we expanded our rapid genomic diagnosis program to a national scale in a prospectively ascertained cohort of critically ill infants and children with rare disease. In addition, we assessed the diagnostic performance of WGS and the impact of systematically integrating additional analysis types, transcriptome analysis and functional assays.

## Results

### Participant demographics and indications for testing

**Fig. 1:**
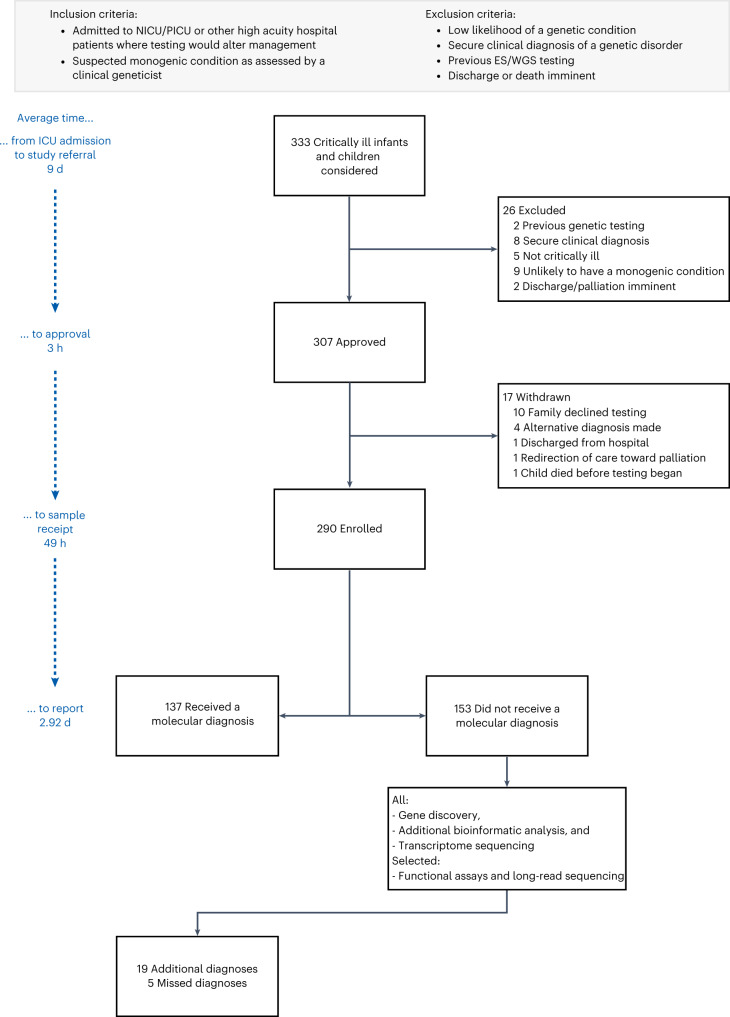
Recruitment workflow for the Acute Care Genomics program. Critically ill infants and children with suspected genetic conditions were proposed to a national panel of experts, with those approved undergoing ultra-rapid WGS. Additional bioinformatic analyses and transcriptome sequencing were performed in all undiagnosed patients. Long-read sequencing and functional assays were deployed in selected cases. ICU, intensive care unit.

**Fig. 2:**
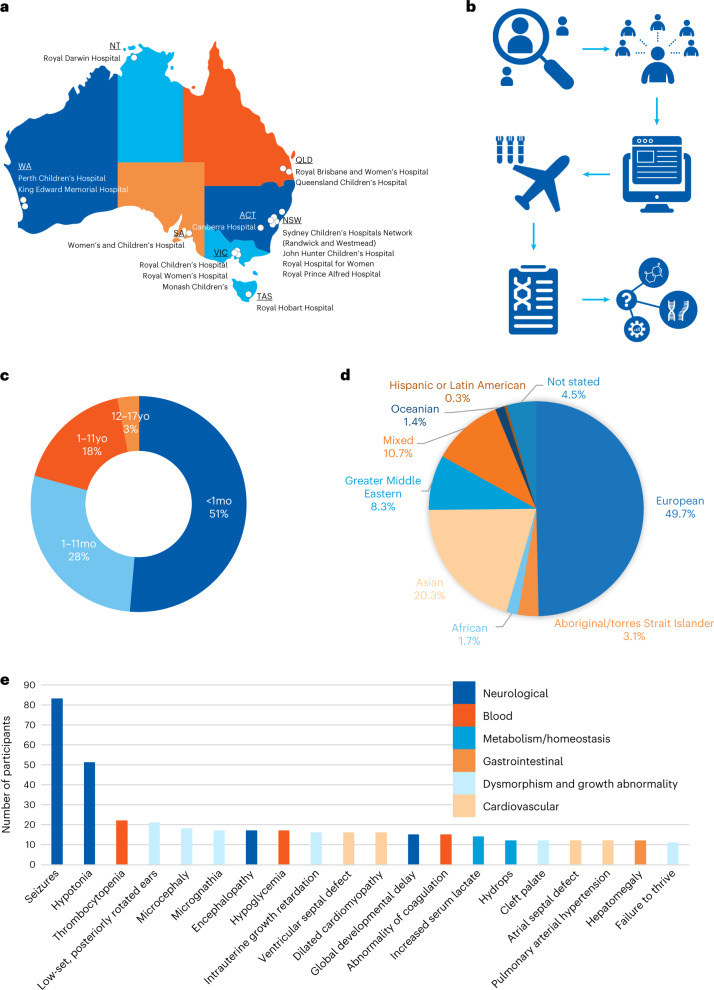
Patient recruitment and key characteristics. **a**, Recruitment sites. NT, Northern Territory; QLD, Queensland; WA, Western Australia; ACT, Australian Capital Territory; NSW, New South Wales; SA, South Australia; VIC, Victoria; TAS, Tasmania. **b**, Study workflow, including patient selection using guidelines and virtual expert panel; electronic resources to support test ordering and consent; sample shipping; diagnostic reporting; and extended analysis and multi-omic approaches in unsolved cases. **c**, Age of study participants. **d**, Ancestry/ethnicity of participants. **e**, Twenty most common HPO terms, coded by major groups.

A total of 333 infants and children were referred to the Acute Care Genomics program. The panel deemed 26 ineligible and a further 17 were withdrawn by the referring team after approval (Fig. 1). Of the 290 participants, 135 (47%) were karyotypically female; the median age was 29 d (range 0 d to 17 years); and 64% presented with symptoms at birth (Fig. 2c).

Trio analysis was performed in 273 families (94%), with the remaining 17 (6%) proceeding as duos due to parental unavailability. Patients admitted to neonatal intensive care units (NICUs) accounted for 47% of the cohort (135 of 290), 39% (112) were admitted to pediatric intensive care units (PICUs) and 15% (43) were other critically unwell hospital patients (for example, awaiting transplant). Genetic counselors and/or clinical geneticists provided pre-test counseling to 286 of 290 families (99%). The cohort was ethnically and clinically diverse, with 694 unique Human Phenotype Ontology (HPO) terms recorded at an average of five per patient (Fig. 2d,e)^14,15^. The commonest reasons for referral were neurological disorders (hypotonia and seizures) and complex multi-system disorders indicative of either syndromic or metabolic disease.

### Time to clinical WGS report

**Fig. 3:**
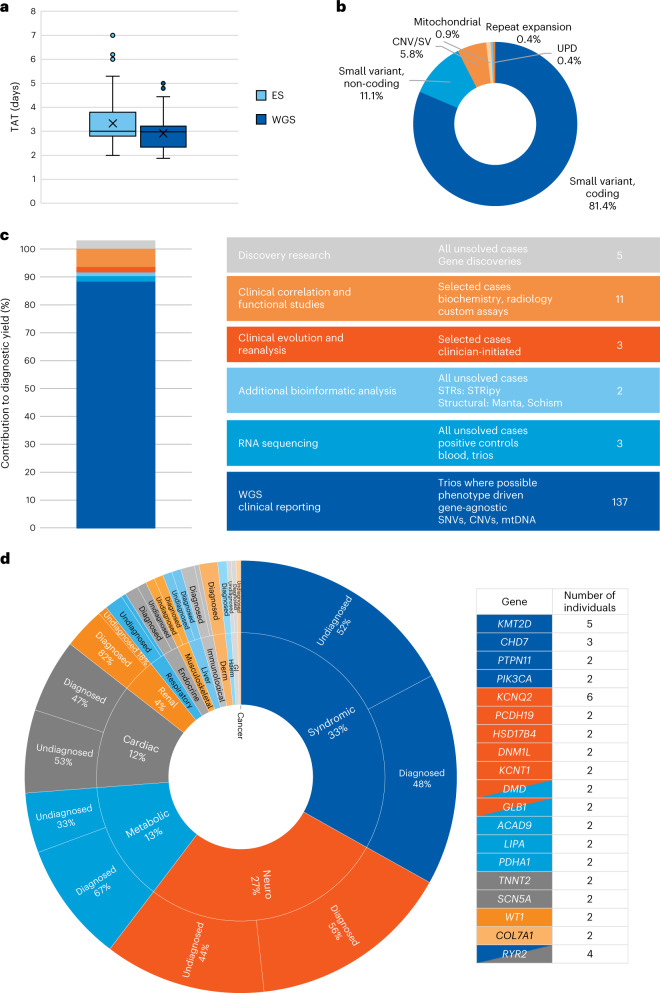
Summary of diagnostic outcomes. **a**, Time to clinical report for ultra-rapid WGS cohort (*n* = 290), compared to previous ultra-rapid ES cohort (*n* = 108). X represents the mean; central line represents the median; top and bottom edges of the boxes are the first and third quartiles; the whiskers show the minima to maxima no greater than 1.5× the interquartile range with remaining outliers plotted individually. TAT, turnaround time. **b**, Variant types detected by WGS. **c**, Incremental gain in diagnostic yield from extended analysis and multi-omic approaches. CNV, copy number variant; SV, structural variant; UPD, uniparental disomy. **d**, Sunburst representing the spectrum of diagnoses. Arranged by number of patients, clockwise, the inner ring represents the principal clinical presentation and the second ring represents diagnostic yield in each group. Genes responsible for diagnoses in multiple individuals represented in the adjacent table, color-coded by principal clinical presentation. Full names of each disorder and Online Mendelian Inheritance in Man numbers are included in Supplementary Tables 1–3.

The average laboratory turnaround time as measured from receipt of all samples to clinical report was 2.9 d (95% CI 2.85–2.99), with a fastest time to result of 45 h. All reports were issued within 5 calendar days of sample receipt (Fig. 3a).

### Diagnostic yield of standard WGS analysis

Of the 290 individuals tested, 137 were diagnosed during initial clinical analysis. The majority of diagnoses (127 of 137; 93%) were in genes contained in the virtual panels assigned by clinicians and 10 (7%) were from panel-agnostic analyses. Of note, eight diagnoses (6%) were due to parentally inherited variants in genes associated with dominant conditions (*SHH*, *FOXF1*, *SCN5A* X2, *FGFR3*, *RANBP2*, *BAG3* and *ENG*), where the parents were not recognized as affected before testing. Of these, five parents were subsequently recognized as clinically affected (*FGFR3*, *SCN5A* X2, *BAG3* and *ENG*), with important implications for further cascade testing within the family; one parent was found to be mosaic (*SHH*, 20% mosaicism). Parental transmission of *FOXF1* and *RANBP2* variants on the other hand, are thought to be explained by a parent-of-origin effect^16^ and incomplete penetrance^17^, respectively. Eight individuals received a partial diagnosis, whereas another five had a dual diagnosis (Supplementary Tables 1–3). Diagnostic yield was highest in those presenting with isolated renal, dermatological and hematological phenotypes and lowest in those with unexplained interstitial lung disease (Fig. 3d). Nineteen genes accounted for diagnoses in multiple individuals (Fig. 3d), with all other diagnoses being unique, highlighting the wide diversity of diagnoses encountered in this clinical setting.

### Diagnostic yield of extended bioinformatic analysis

A triplet repeat expansion in *DMPK* was identified using the STRipy tool^18^ in an infant with hypotonia (A0131129). STRipy detected an expanded allele predicted to contain over 200 repeats but was unable to accurately size the expansion. Clinically accredited testing confirmed the presence of a ~900 repeat allele in the proband, consistent with a diagnosis of congenital myotonic dystrophy and 48 repeats (premutation range) in the mother, who was not clinically affected. Paternal allele sizes were normal. Nanopore long-read sequencing in the proband indicated expansion size of 1,048 repeats.

**Fig. 4:**
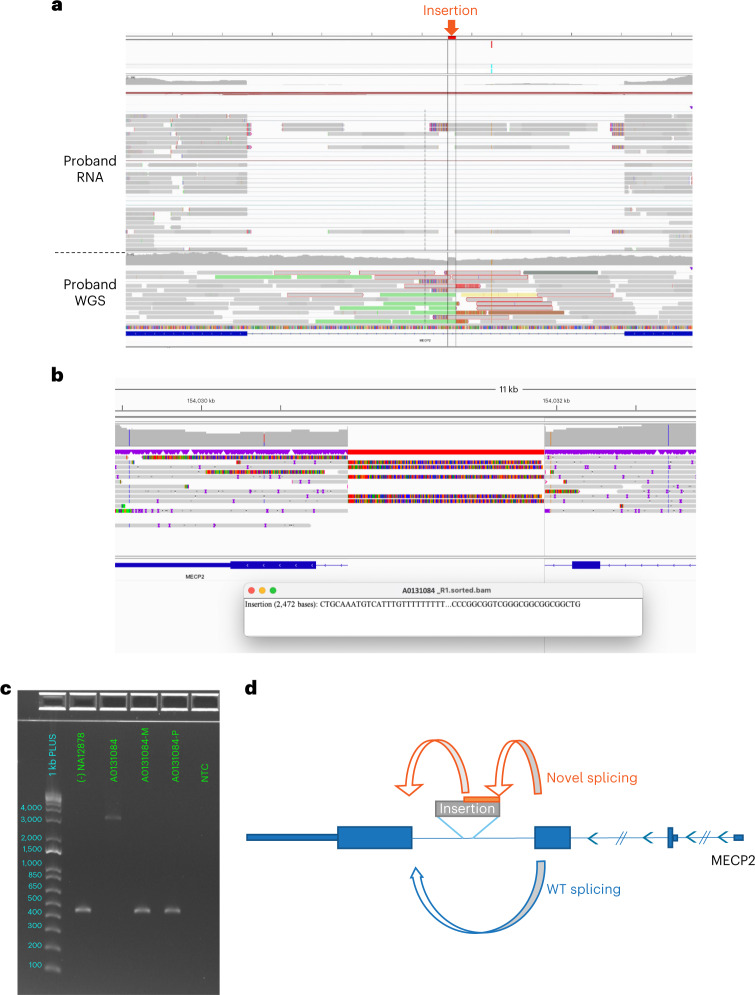
Identification and confirmation of intronic insertion variant, likely derived from an SVA retrotransposable element, in the last intron of *MECP2*. **a**, Integrated Genomics Viewer (IGV) proband RNA (top) and DNA (bottom) short-read sequencing data indicating the presence of a DNA insertion resulting in the inclusion of a pseudo-exon in the last intron of *MECP2*. **b**, Nanopore sequencing data demonstrating the insertion. **c**, PCR gel electrophoresis of relevant *MECP2* region in the proband (A0131084), parents (A0131084-M and A0131084-P) and a control sample (NA12878), consistent with an insertion of approximately 2.6 kb. This clinically accredited assay was performed once. **d**, Schematic of the observed splicing outcomes of *MECP2* in the proband with the presence of a transposon-derived pseudo-exon, residual canonical splicing, skipping of the penultimate exon and intronic read-through. WT, wild-type.

Analysis using Manta and Schism for complex structural variants indicated a large, up to 2.6-kb, intronic insertion variant, likely derived from a SINV-VNTR-Alus (SVA) retrotransposable element, in the last intron of *MECP2* in a male child with microcephaly, developmental delay and epileptic encephalopathy (A0131084). The presence and origin of the insertion were confirmed using Nanopore long-read sequencing, demonstrating that the full 2.5-kb SVA sequence is present in the last intron of *MECP2* (Fig. 4b). Testing of the proband and the parents using long-range PCR also confirmed this to be a de novo event. Manual analysis of RNA data determined that the intronic insertion disrupted normal splicing; however, an estimated 66% of wild-type *MECP2* splicing remained, consistent with reduced clinical severity. Of note, the overall expression of *MECP2* was not reduced, nor was our independent bioinformatic splicing analysis able to detect this event, highlighting the value of multiple approaches to identify pathogenic variants.

### Transcriptome yield

RNA-sequencing data were successfully generated from 335 individuals (115 patients and their parents), resulting in three new diagnoses.

In A0131122, presenting with features of skeletal dysplasia, a known pathogenic heterozygous promoter variant in *RMRP* was identified (NR_003051.3(*RMRP*):n.-5_-4insAACTACTCTGTGAAGCTGA), consistent with a diagnosis of cartilage-hair hypoplasia. This was confirmed to reduce expression of the affected allele in the proband and paternal sample. A second single-nucleotide insertion variant, initially of uncertain significance, was identified in the maternal allele (NR_003051.3(*RMRP*), n.93_94insA). Analysis of maternal RNA data showed this insertion conferred strong (>99%) preferential expression from the wild-type allele, indicating instability of the variant transcript. In combination with the paternal pathogenic variant and a 9.2-fold downregulation of *RMRP* in the proband, this was considered sufficient evidence to ascribe pathogenicity to the maternal insertion, securing the diagnosis.

In A0831013, presenting with neonatal hypotonia, a new 403-bp hemizygous, maternally inherited deletion spanning parts of the gene promoter and 5′ UTR of *MTM1* was identified, including the transcription start site (TSS) (NM_000252.2(*MTM1)*: c.-76_-11del), consistent with a diagnosis of myotubular myopathy. Analysis of RNA data confirmed reduction in *MTM1* expression in the male proband, effectively abolishing gene expression with no evidence of alternative TSS utilization.

The *GLB1* variant in A1031002 was a new homozygous exon 7 splice donor region variant (NM_000404.3(*GLB1)*: c.733+6 T > C) predicted to cause a splice defect, which would result in a premature stop codon and nonsense-mediated decay. Pathogenic variants in *GLB1* are associated with GM1 gangliosidosis; however, the patient’s phenotype was milder than expected. Analysis of RNA data indicated exon skipping with residual wild-type transcription in the proband, consistent with the milder clinical presentation. Confirmation of a pathogenic splice variant determined access to a clinical trial.

Confirmed splice variants in another seven samples were included as positive controls, with a splice defect confirmed in two of these, whereas five had insufficient expression in blood to be informative. This included the confirmation of an in-frame exon skipping event caused by the NM_005188.3(*CBL*): c.1096-2 A > T canonical splice variant, in a child with Moyamoya disease and developmental delay. Consistent with the known mechanism of disease^19,20^, this splice variant resulted in the upregulation of *CBL*, which was detected in our expression outlier analysis, highlighting the need to carefully assess upregulated outliers.

### Clinical and functional correlation

Thirty variants of uncertain significance (VUSs), deemed highly likely to be clinically relevant, were reported. One individual underwent specialized clinical re-review including international consultation to establish variant pathogenicity^21^. In ten individuals, additional functional studies were performed (Table 1). This consisted of a range of clinically accredited assays in seven individuals and bespoke research assays in three.

**Table 1:** Individuals with variants of uncertain significance (VUS) where additional functional validation resulted in variant reclassification

Study ID	Presenting clinical features	Gene variant(s), original classification	Additional studies	Final variant classification(s), time to report
Clinically accredited tests				
A3231005	Hydrops	*GLB1* c.130 G > T,p.(Asp44Tyr), VUS	Enzyme studies	LP 5 days
A0831018	Immunological dysfunction, bone marrow failure	*PNP* c.97 T > C, p.(Ser33Pro), VUS	Enzyme studies	LP 5 days
A3331002	Seizures	*HSD17B4* c.1132 G > A, p.(Gly378Arg), hmz, VUS	Very-long-chain fatty acids	LP 12 days
A2131008	Hydrops, dilated cardiomyopathy, thrombocytopenia	*TBX19* c.666-2 A > T, VUS	Endocrine testing	LP 17 days
A0831021	Lactic acidosis, central hypotonia	*GTPBP3* c.521 G > C, p.(Arg174Pro), hmz, VUS	Respiratory chain enzymes	LP 7 weeks
A0731002	Severe intrauterine growth restriction	*RNU4ATAC* n.18 G > A, VUS n.50 G > A, LP	Bone dysplasia specialist review of skeletal survey	LP, LP 2 months
A0431022	Neurological deterioration	*PDHA1* c.1045 G > A, p.(Ala349Thr), VUS	Enzyme studies	LP 4 months
Research assays				
A1431031	Severe lactic acidosis	*ACAD9* c.1636 G> A, p.(Val546Met), LP and c.1376_1381delins, p.(Lys459_Ser461delinsThrCys) VUS	Western blot^23^	P, LP 4 months
A1131048	Encephalopathic episode	*NUP214* c.112 C > T; p.(Arg38Cys) P and c.929 T > C; p.(Ile310Thr) VUS	Western blot, nuclear pore quantification, quantitative proteomics	P, LP 6 months
A0131063	Recurrent liver failure	*NBAS* c.2951 T > G, p.(Ile984Ser), VUS and c.406 A > G, p.(Arg136Gly), VUS	Western blot and p31 level^22^	P, LP 10 months

hmz, homozygous; P, pathogenic; LP, likely pathogenic.

#### NUP214

**Fig. 5:**
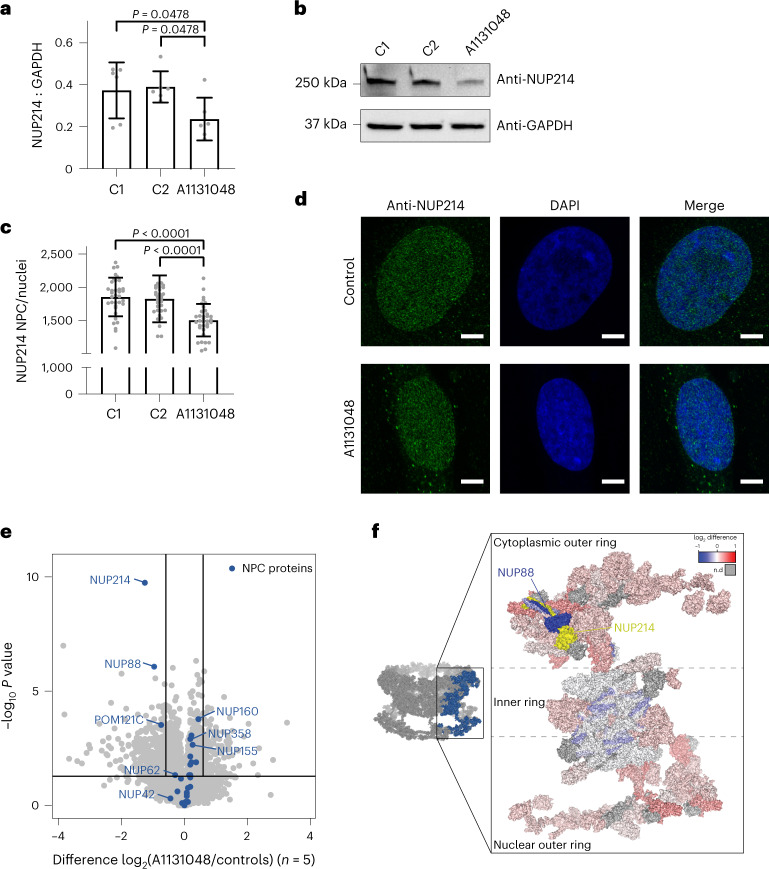
Decreased NUP214 steady-state levels and decreased NUP214-containing nuclear pore complex density in fibroblasts from A1131048. **a**, Densitometry shows significantly reduced NUP214 levels in fibroblasts compared to controls. *n* = 6 biological samples per cell line. Data represent mean ± s.d. One-way analysis of variance (ANOVA) with Holm–Sidak’s multiple comparisons test. **b**, Representative western blot of NUP214 in fibroblasts from A1131048 and two controls (C1 and C2). **c**, Quantification of NUP214-containing nuclear pore complex pore density (pores per nucleus) in fibroblasts from A1131048 compared to controls shows a significant decrease in fibroblasts from A1131048. *n* = 3 biological samples per cell line, from three independent experiments, represented is pooled data from from *n* = 35 for C1, *n* = 31 for C2 and *n* = 35 for A1131048. Data represent mean ± s.d. One-way ANOVA with Holm–Sidak’s multiple comparisons test. **d**, Compressed Z-stack representative images of NUP214 immunostaining (green spots) and nucleus (4,6-diamidino-2-phenylindole (DAPI); blue) in fibroblasts from A1131048 and controls. Scale bars, 5 μm. Images are representative from three experiments. **e**, Volcano plot showing protein abundances in the fibroblasts from A1131048 relative to healthy controls (*n* = 5). Nuclear pore complex (NPC) components are indicated in blue. The horizonal line represents *P* = 0.05 and the vertical lines represent fold changes of ±1.5. Data were derived from a two-sided Student’s *t*-test. No adjustments were made for multiple comparisons. **f**, Topographical heat map showing fold changes of NPC proteins identified by proteomics mapped onto the structure of the NPC cytosolic face (Protein Data Bank, 7TBL). Yellow indicates NUP214 subunit. Other subunits, including NUP88, which is coiled around NUP214, are colored according to their fold change relative to controls, as indicated in the inset; gray indicates no data. Source data

We identified *NUP214* biallelic variants in A1131048; one variant (NM_005085.3(*NUP214*): c.112 C > T; p.(Arg38Cys)) was previously reported and was classified as pathogenic^22^. The second variant (NM_005085.3(*NUP214*): c.929 T > C; p.(Ile310Thr)) was new, absent in gnomAD and predicted to be damaging by multiple in silico tools. Western blot of proteins from patient fibroblasts demonstrated a significant decrease (*P* < 0.05) in NUP214 levels compared to controls (Fig. 5a,b). Quantification of NUP214-containing pores in the nuclear region by super-resolution microscopy imaging showed a significant reduction (*P* < 0.0001) in the NUP214-containing nuclear pore density in the nuclear envelope compared to controls (Fig. 5c,d), confirming previous reports^22^. Quantitative proteomics confirmed decreased NUP214 protein levels (Fig. 5e and Supplementary Table 4) and revealed a decrease in two other nucleoporins; POM121C and NUP88, the latter a physical interactor of NUP214 within the human nuclear pore complex (Fig. 5f) and previously reported as reduced in patients with *NUP214* pathogenic variants^22^. Last, we observed a decrease in viability of patient fibroblasts after a 2-h heat shock, validating previous reports^22^, without changes in the apoptotic response to heat stress. These multiple lines of additional evidence to support pathogenicity of the *NUP214* variants in A1131048 were used to reclassify the second variant as likely pathogenic.

### Reanalysis

Three variants were reported as diagnostic following reanalysis prompted by evolving clinical features. A0731004 initially presented with multiple congenital anomalies, but subsequently developed seizures at 5 months of age, leading to the reporting of a de novo new missense variant in *GABRB3*. A0131108 initially presented in infancy with focal seizures, remained undiagnosed but developed nystagmus, resulting in the reporting of a pathogenic variant in *FRMD7*. Finally, infant A0131089 presented with neonatal seizures and had a pathogenic de novo variant identified in *KCNQ2*, as well as a maternally inherited variant of uncertain significance in *GABRG2*. The *GABRG2* variant was upgraded as pathogenic 2 years later based on family history, evolving clinical presentation and newly published data.

### Gene discovery

Ten gene candidates were submitted to GeneMatcher. Five matched to multiple other similarly affected individuals and are currently being pursued to establish gene–disease relationships.

### Diagnoses not obtained by WGS

Five individuals had uninformative WGS and were subsequently diagnosed through orthogonal genetic testing. In three, the causative variants were mosaic, one each in the *KCNT1*, *PIK3CA* and *IKBKG* genes. In each of these cases, the relevant variants were filtered from the analysis as failing quality parameters (the minimum variant allele threshold of ≥15% of reads with the variant). The *PIK3CA* variant was detected in a fibroblast sample (mosaicism level 21%), whereas the *KCNT1* variant was detected on exome sequencing (mosaicism level 16%). The mosaic *IKBKG* variant was detected using Sanger sequencing (mosaicism level not determined). Review of the available trio WGS data identified that the variant was maternally inherited, with the apparent mosaicism in the proband reflecting somatic reversion.

A further case was diagnosed by microarray with uniparental isodisomy of chromosome 15 (UPD), causing Prader–Willi syndrome. Notably, RNA-sequencing data were also able to identify this diagnosis based on the lack of expression of *SNHG14* and *SNRPN*, which are only expressed from the paternally inherited allele, illustrating the scope of RNA-sequencing data to identify dysregulation of imprinted genes. The final missed diagnosis had a polyalanine repeat expansion in the *PHOX2B* gene, a well-described variant causing congenital central hypoventilation syndrome, missed due to lack of read coverage in the responsible low-complexity gene region.

### Clinical utility

Of the 156 individuals diagnosed through WGS, 120 (77%) had changes in critical care management as reported by treating clinicians (Supplementary Tables 1 and 2). Many of these related to improvements in the overall process of care through rationalizing investigations, referrals and treatments. In 94 patients (60%), results had major implications for management, including informing precision treatments; surgical and transplant decisions; and palliation. Enzyme replacement therapy was commenced in three individuals and six infants were diagnosed with *KCNQ2*-related epileptic encephalopathy, informing choice of anti-epileptic agent. Ten diagnoses (6%) informed decisions regarding transplantation and 35 diagnoses (22%) formed part of decisions to redirect care toward palliation. As an example of a diagnosis with major implications for clinical management, A1131034 presented in adolescence with unexplained renal failure and was diagnosed with Frasier syndrome. This prompted further imaging, which revealed dysplastic gonads and identification of bilateral gonadoblastomas on biopsy. The individual underwent successful surgery to resect these.

## Discussion

In this prospectively ascertained national cohort, we demonstrate diagnostic benefits of using ultra-rapid WGS as a first-tier test, due to a combination of increased analytical performance and use of multi-omic approaches to improve variant interpretation. We also increased robustness of the national approach by implementing ultra-rapid data sharing, analysis and reporting in a second diagnostic laboratory, while expanding clinical recruitment to all Australian states and territories. We provide detailed data on diagnostic yields in different patient groups to guide future implementation.

Laboratory testing times for WGS were not significantly different from those in our previously described cohort using exome sequencing (ES)^3^, with an average time from sample receipt to report of 2.9 d (95% CI 2.85–2.99) for WGS and 3.1 (95% CI 2.98–3.21) for ES. While sample preparation times were shortened with the removal of the exome enrichment step, average bioinformatic processing time increased due to increased data volumes and addition of CNV calling, highlighting this as an area for future improvement. The range of variant types identified using WGS was much broader, including CNVs ranging in size from 57 kb to a ring chromosome, deep intronic and regulatory variants, mitochondrial DNA variants, STRs and a transposon insertion. Six diagnoses were due to a combination of a small coding variant with a CNV, further highlighting the utility of WGS to combine analysis for multiple variant types in a single test, shortening time to diagnosis.

It is also clear that the analytical potential of WGS currently outstrips our ability to robustly detect, validate and classify many variant types under accredited conditions, with additional testing modalities critical in demonstrating downstream effects. This was most notably exemplified by the male proband with atypical features of Rett syndrome caused by a retrotransposon insertion causing splice defect in *MECP2*. This diagnosis was made using a combination of extensive, cutting-edge bioinformatic analysis, trio RNA-sequencing and orthogonal validation by established molecular techniques.

Transcriptome analysis was also instrumental in securing three additional diagnoses. In these cases, both aberrant splicing and reduction of expression were identified, with a mixture of variant types, including non-canonical splice site, promoter deletion and expression changes in a noncoding gene. Availability of parental RNA data was critical for resolution in at least one case. We elected to perform RNA-sequencing using the original whole blood samples submitted for WGS to reduce burden on families and clinicians. The additional diagnostic yield obtained from RNA-sequencing in this cohort (2.5%) is lower than reported in other studies (7–36%) (refs. ^23–26^), which likely reflects the inclusion of all, rather than selected, unsolved patients and limitations of tissue-specific expression. Despite these challenges, our approach of using trio transcriptome analysis on RNA from blood samples has proven both feasible and useful and we will further explore implementation in parallel with ultra-rapid WGS.

The largest gain in diagnostic yield was from pursuing clinical and functional correlation of VUSs identified by WGS analysis. This included radiological, metabolic and immunological tests that are clinically available through to custom-designed studies, such as western blots, ultra-resolution immunofluorescent imaging and quantitative proteomics. Achieving timely and systematic functional validation remains challenging, with the range of assays currently clinically available focusing on specific metabolic and immunological disorders. As genome-wide tests become increasingly incorporated at the start of the diagnostic pathway, there will be a growing need for high-throughput, gene-agnostic functional assays to resolve VUSs. Such functional assays need to be designed so that the results can be incorporated into diagnostic reporting using established criteria^27^. Many of these assays will need to transition from research to clinical laboratories to scale up and ensure appropriate test funding, validity, reproducibility and timeliness of results.

There is ongoing debate about the relative advantages of clinically focused and gene-agnostic analysis approaches. We used a combination of the two, with 7% of diagnoses achieved outside of the virtual gene panels nominated by clinicians. By contrast, analysis outside of the ‘Mendeliome’ virtual gene panel did not reveal any additional diagnoses but yielded ten gene candidates, which were submitted to GeneMatcher^28^. We anticipate that several of these will be confirmed as disease genes, with the same approach applied in our previously published ES cohort (*n* = 108; 2018 to 2019) having yielded six gene discoveries to date^29–33^. Identifying gene candidates has traditionally been the domain of dedicated research programs^34–36^, with selected patients entering these at the end of the diagnostic trajectory. With genomic testing increasingly performed through clinical pathways, identifying gene candidates should form an integral part of diagnostic analysis. Several diagnostic laboratories have now reported their experience of systematically contributing to gene discovery^37–39^ and while this requires additional resourcing, wider implementation will both reduce the time to gene discovery and expand the effort.

We are aware of five diagnoses that were missed by WGS, highlighting some important limitations and the need to consider alternative testing modalities when clinical suspicion of a specific disorder is high. Three were due to mosaicism and while we have amended our practice to review known pathogenic variants failing quality checks, detection of mosaicism will continue to be challenging due to the relatively lower coverage of WGS compared to exome or targeted sequencing. This limitation is only likely to be overcome once reduction in the cost of WGS data generation allows deeper sequencing. Deeper sequencing of variants in low-complexity regions would also have improved the chance of detecting the missed polyalanine repeat variant in *PHOX2B*. The missed UPD, as well as the identification of an STR variant and a complex structural variant using unaccredited bioinformatic analysis in this cohort, highlight the need to continuously improve and expand the range of analysis tools used in the diagnostic setting. Some of these challenges may be overcome by increased use of long-read sequencing technology. While we used Nanopore as a post hoc validation tool, long-read sequencing as first-line diagnostic testing would have substantially reduced the time to diagnosis in both the repeat expansion and the transposon insertion cases and may have identified other complex variants. The feasibility of using long-read sequencing in the acute setting has recently been demonstrated and holds the promise of dramatically shortening turnaround times and potentially decentralizing sequencing and interpretation^6,40,41^. From a broader implementation perspective, the service delivery model in our study, which included patient selection by clinical geneticists and centralized sequencing, may only be applicable to a subset of healthcare systems. While a substantial body of work has now accumulated about the utility of rapid genomic testing in the acute setting, long-term outcome data are lacking and this remains a major limitation of this and other studies.

In this prospectively ascertained national cohort undergoing ultra-rapid WGS, we demonstrate scalability of the model and highlight gains in diagnostic yield by rapidly integrating genomic, transcriptomic and proteomic data. There is a need to systematically integrate these approaches as part of diagnostic pathways to fully realize the potential of genomic sequencing to alter outcomes in rare disease patients and families in a timely manner.

## Methods

### Ethics

The Australian Genomics Acute Care study has Human Research Ethics Committee approval (HREC/16/MH/251). Parents provided informed consent for participation in the study, following genetic counseling.

### Study design and participants

The Acute Care Genomics program is a national multi-site study delivering ultra-rapid genomic testing to critically ill pediatric patients with suspected genetic conditions. The study adopts a highly coordinated clinical and laboratory approach, informed by implementation science principles and theory^3,42^. Participants were recruited prospectively from a network of 17 hospitals, including all children’s hospitals in Australia, between January 2020 and April 2022. Patients were eligible if they were admitted to a participating NICU or PICU with a suspected monogenic condition as assessed by a clinical geneticist. Other hospital patients were considered if a rapid result was likely to alter management. Patients were ineligible if a monogenic etiology was considered unlikely; if a secure clinical diagnosis (such as Apert syndrome) was in place; if previous exome or genome testing had been performed; or if death or discharge were imminent (Fig. 1).

Electronic referrals were peer reviewed by a panel of study investigators for approval. Electronic test ordering and consent were used to collect clinical and other metadata in standardized formats, including HPO terms^15^, as well as to manage remote recruitment during COVID-19 restrictions. Patient sex was determined karyotypically. Parents self-reported ancestry and this was recorded by referring clinicians based on categories in the Human Ancestry Ontology^14^. Virtual gene panels to guide phenotype-driven analysis were assigned by requesting clinicians; all panels used in the study are publicly available from PanelApp Australia^43^ (https://panelapp.agha.umccr.org). Data were managed using REDCap, a secure, web-based application designed to support data capture for research studies^44^. Chromosomal microarray (SNP-CMA) was performed before enrollment if the likelihood of a chromosomal condition was considered high (for example, patients with multiple congenital abnormalities or isolated hypotonia to exclude Prader–Willi syndrome). Laboratory reports were issued to referring clinicians and were accompanied by plain language reports for families^45^. Additional findings unrelated to the reason for testing were not deliberately sought. A separate sub-study will explore a two-step model for offering these to families 3–6 months post-result.

### Genome sequencing, data analysis and interpretation

WGS data generation and clinical analysis was performed using diagnostically accredited methods by the Victorian Clinical Genetics Services (VCGS) in Melbourne, Australia or SA Pathology, South Australia, Australia. For samples processed by the VCGS, DNA was extracted manually from blood collected in EDTA vacutainers using the QIAamp DNA blood mini kit (QIAGEN). DNA quantity and quality were assessed using the Qubit dsDNA BR (broad-range) Assay kit (Thermo Fisher) and TapeStation genomic DNA kit (Agilent), respectively. Whole-genome DNA libraries were created using Nextera DNA Flex Library Prep kit/Illumina DNA prep kit (Illumina) followed by 2 × 150-bp paired-end DNA sequencing on a NovaSeq 6000 instrument (Illumina), variably using S2 or S4 flow cells. Targeted mean sequencing depth was 30×, with a minimum of 90% of bases sequenced to at least 10× for nuclear DNA (nDNA) and a minimum of 800× mean coverage for mitochondrial DNA (mtDNA).

Data were bioinformatically processed using commercially available and in-house analysis pipelines. Alignment to the reference genome (GRCh38) and calling of nuclear/germline DNA variants was performed using the Dragen v.3.3.7 (Illumina) workflow. Alignment to the revised Cambridge Reference Sequence (rCRS) mitochondrial genome (NC_012920.1) and calling of mitochondrial DNA variants was performed using an in-house analysis pipeline based on the Broad Institute best practice workflow (https://gatk.broadinstitute.org/hc/en-us/articles/4403870837275-Mitochondrial-short-variant-discovery-SNVs-Indels).

Automated sex determination, relatedness and contamination checks were performed using a combination of Somalier^46^ and in-house tools.

For nDNA, variant analysis and interpretation within the selected target regions (RefSeq genes ± 1 kb) was performed using the trio (where available) analysis approach in the Alissa Interpret software suite (Agilent). CNVs were screened for and interpreted using an internal CNV detection tool, CXGo^47^, incorporating four CNV detection tools (Delly, Lumpy, CNVNator and Canvas)^48–51^. For mtDNA variant interpretation, a custom in-house analysis pipeline was used to visualize large deletions and annotate the VCF file with variant information before manual filtering. An mtDNA variant was regarded as homoplasmic or apparently homoplasmic when it was present in at least 97%, respectively, of sequence reads aligned to the genomic position.

Genes with established disease association (Mendeliome) are considered during routine analysis. Curation of nDNA and CNV variants was phenotype-driven with pre-curated or custom gene lists used for variant prioritization (PanelApp Australia https://panelapp.agha.umccr.org/)^43^. Further analysis of all genes to identify research candidate genes was performed for undiagnosed cases (see below).

Classification of nDNA, CNVs and mtDNA variants was based on the appropriate American College of Medical Genetics and Genomics guidelines. Reported high-confidence small variants were generally not confirmed by an orthogonal method. All reported CNVs were orthogonally validated unless otherwise stated.

For South Australian patients with referral after May 2021, clinical data analysis was performed by a separate laboratory, SA Pathology, following data generation at VCGS. For these cases, relevant fastq and/or bam and vcf files were shared via BaseSpace (Illumina) followed by analysis and reporting using SA Pathology’s clinically accredited systems for small variants (SNVs and indels). Briefly, variant annotation was performed using the in-house VariantGrid v.3 interpretation support software. Rare inherited variants (consistent with Mendelian inheritance modes) and de novo variants were reviewed for overlap with the clinical presentation. In addition, phenotype-driven and virtual gene panel analyses were performed, independent of inheritance modes with the proband as singleton, focusing on genes most strongly associated with the clinical phenotypes. Frequency in population databases (gnomAD) and in-house database (VariantGrid) was used to prioritize or exclude variants for review. Variants were further prioritized based on in silico pathogenicity predictions, sequence conservation scores, protein function and expression and known disease associations. Results were integrated with copy-number and mtDNA analyses performed by VCGS. Candidate variants were reviewed in IGV. All reportable variants met quality criteria and were thus not confirmed by an orthogonal method.

### Extended bioinformatic analysis and long-read sequencing

Data from all undiagnosed cases were analyzed for STR expansions using STRipy^18^ and screened for balanced structural variants such as inversions and structural variants within 50 kb upstream and downstream of relevant genes using Manta^52^ and Schism (https://github.com/ssadedin/schism). Events were manually assessed to confirm inheritance along with plausible proximity to a clinically relevant gene. Findings were orthogonally validated using clinically accredited methods before reporting. A complex structural variant and a large repeat expansion were confirmed using long-read sequencing (Nanopore).

### Gene candidates

In all undiagnosed cases, analysis was expanded to variants in all genes, focusing on de novo and recessive high impact variants. Biologically plausible gene candidates, based on gene properties and/or literature research, were submitted to GeneMatcher^28^.

### Trio transcriptome sequencing, data analysis and interpretation

In all undiagnosed cases, RNA was extracted from EDTA blood using the Omega Bio-Tek EZNA DNA/RNA Isolation kit. The quantitative and qualitative analysis of the extracted RNA was performed using the Qubit RNA Assay kit (Thermo Fisher) TapeStation High Sensitivity RNA and standard RNA kits (Agilent). Libraries for RNA-sequencing were constructed using the Illumina TruSeq stranded total RNA gold kit (including probes for ribosomal and globin RNA transcription inhibition) with subsequent NovaSeq 6000 2 × 150-bp paired-end sequencing (Illumina), variably using S2 or S4 flow cells with an aim of ~80 million fragments (~160 M paired-end reads) per sample. To identify true outliers of gene expression, the Bioconductor package OUTRIDER^53^ was utilized. One hundred and seven of the probands were analyzed together, with parental samples analyzed separately. For quality control, samples with size factor <0.1 were removed from the analysis. Overall, 9,651 genes were filtered out due to zero counts (not expressed), which accounted for 16.2% of the original annotated genes from Gencode v.37. Genes with an adjusted *P* value < 0.1 per sample were interrogated, with a focus on downregulated genes.

### Nanopore sequencing

DNA for Nanopore sequencing was extracted from EDTA blood as described above (Genome sequencing). Data were generated on a Promethion using v.10.4.1 flow cells and chemistry, followed by alignment using Minimap2 before visualization in IGV.

### Long-range PCR

Primers were designed to bind in the intronic regions flanking the retrotransposon insertion (MECP2-int4-gDNA-Fwd: 5′-GCCTCTCCAAAGTTCAGCAAC-3′; MECP2-int4-gDNA-Rev: 5′-TGCCCTGAGTGGGAAGTTCT-3′). PCR was performed using PrimeStarGXL DNA Polymerase (Takara Bio) according to manufacturer’s instructions. Then, 50 ng genomic DNA from the proband (A0131084), both parents (A0131084-M and A0131084-P) and NA12878 (Coriell) was amplified, alongside a No Template Control reaction. Reactions were run on a 1% E-Gel EX (Thermo Fisher) and visualized using a GelDoc XR (Bio-Rad).

### Functional assays

VUSs related to phenotype and close to ‘likely pathogenic’ classification were reported and a pathway was sought to access functional studies and/or additional clinical correlation (such as imaging studies). Clinically accredited functional assays were used where possible, with some variants referred to research groups. Western blots were performed as previously described^54,55^.

### *NUP214* functional genomic analysis

#### Cell culture

Primary cultures of fibroblasts from individual A1131048 and two unrelated pediatric control fibroblasts (from a 2-month-old female and female <16 years old) were established from skin biopsies as previously described^56^. Fibroblasts were cultured in basal medium (high-glucose DMEM (Gibco) with 10% fetal bovine serum (Gibco), 100 U ml^−1^ penicillin and 100 µg ml^−1^ streptomycin) at 37 °C with 5% CO_2_. All fibroblast control cell lines were established in-house and were established from pediatric individuals without any suspected genetic disorders. All cell lines were mycoplasma negative and the *NUP214* genotype was validated by PCR and Sanger sequencing using the following primers: *NUP214* chr9:131127590c.112 C > T; p.(Arg38Cys); Fwd: GAGACAGACCTTGGTCTCAGTAA; Rev: AGCATGCCACCATACTCCTC and *NUP214* chr9:131134995c.929 T > C; p.(Ile310Thr); Fwd: CGGTTGATGGCCAATGTTTGT; Rev: CAAGGCATCTCAGCCTCCATT.

### Western blot

For denaturing gels, proteins from fibroblasts were extracted, 30 µg of proteins separated by SDS–PAGE and transferred to PVDF (Merck, cat. no. IPVH00010) as previously described^57^. Primary antibodies were specific to human NUP214 (cat. no. ab70497) and human GAPDH (cat. no. G9545, Sigma Aldrich; 1:10,000 dilution) and appropriate anti-rabbit horseradish peroxidase-conjugated antibodies (GE Healthcare; 1:5,000 dilution), enhanced chemiluminescence reagents (Bio-Rad) and Bio-Rad ChemiDoc were used to capture band intensity. Antibodies were validated by the manufacturer against a titrated amount of control human fibroblast lysate and used according to manufacturer’s protocols. Protein band intensities were quantified using Image Lab v.6.0 software.

### Immunofluorescence microscopy

Patient and control fibroblasts were fixed in 4% paraformaldehyde for 20 min and washed in PBST (PBS + 0.5% Triton X-100). Cells were blocked and permeabilized with 1% BSA in PBST for 1 h at room temperature. Cells were probed with primary antibody anti-NUP214 antibody (rabbit, cat. no. ab70497) diluted with 1% BSA in PBST (1:500) for 1 h at room temperature and washed in PBS. Secondary incubation was with Alexa Fluor 488-conjugated goat anti-rabbit antibody (Thermo Fisher Scientific, cat. no. A-11008) in 1% BSA in PBST (1:1,000 dilution) for 1 h at room temperature and washed in PBS. Coverslips were mounted on a microscopy slide using ProLongTM Gold Antifade with DAPI (Life Technologies).

### Quantitative mass spectrometry and data analysis

Fibroblast cellular pellets for the A1131048 patient and five control fibroblast cell lines were resuspended in 5% SDS and 50 mM triethylammonium bicarbonate. Total protein concentration for each sample was quantified with Pierce BCA protein assay kit (Thermo Fisher) and samples were aliquoted for 25 µg of protein in triplicates for the patient and single aliquots for each control cell line. Samples were processed using S-trap micro-spin columns according to the manufacturer’s instructions, with reduction and alkylation being performed with 40 mM chloroacetamide (Sigma) and 10 mM tri(2-carboxyethyl)phosphine hydrochloride (BondBreaker, Thermo Fisher). Protein digestion was performed at a 1:10 trypsin to protein ratio at 37 °C overnight and eluted peptides were dried down using a CentriVap Benchtop Vacuum Concentrator (Labconco). Samples were reconstituted in 45 µl 2% acetonitrile, 0.1% trifluoroacetic acid buffer and 2 µl each sample injected for liquid chromatography (LC)-tandem mass spectrometry (MS/MS). Data were acquired on an Orbitrap Eclipse mass spectrometer (Thermo Fisher) coupled with an Ultimate 3000 HPLC (Thermo Fisher) and NanoESI interface. The system was equipped with an Acclaim Pepmap nano-trap column (Dionex-C18, 75 µm × 2 cm) and an Acclaim Pepmap RSLC analytical column (Dionex-C18, 75 µm × 50 cm), running in a data-independent acquisition mode with a previously described method^58^. Raw files were processed using Spectronaut (v.16.2.220903.53000, Rubin) against a data-dependent-acquired peptide library generated from deeply fractionated control fibroblast samples containing 150,106 peptide precursors. Default Spectronaut BGS Factory search parameters were used with changes made to select ‘Exclude single hit proteins’ settings and de-select ‘Major Group Top N’ and ‘Minor Group Top N’ options, allowing all identified peptides to be considered for quantitation. Proteins were searched using the UniProt reviewed human canonical and isoform database (42,360 entries).

Data were imported into Perseus (v.1.6.15.0) (ref. ^59^) for processing where known contaminants were filtered for and all MS2 quantity levels were log_2_ transformed. The A1131048 patient and control groups were filtered for proteins having at least two valid values and a two-sided *t*-test was conducted. A volcano plot was generated using the scatter-plot function with significance set at ±1.5 fold change (log_2_ ± 0.585) and *P* value = 0.05 (−log_10_ = 1.301), with NPC components being manually annotated. Log_2_-transformed changes of NPC components were exported and the values were used to color chains representing specific subunits according to the fold change of the relevant protein on the cryoelectron microscopy-derived complex structure (Protein Data Bank accession code 7TBL) using the PyMOL Molecular Graphics System, v.1.7.2.1 (Schrödinger)^60,61^.

### Microscopy and analysis

For NUP214-containing nuclear pore density images were captured with a Zeiss LSM 900 confocal microscope with Airyscan 2 super-resolution imaging. NUP214 immunostained cells were captured with a ×63 oil immersion lens and Z-stacks with a series of 30 slices. For NUP214 quantification, Z-stack images were flattened in ImageJ using Z-Projection and ‘Maximum Projection’. Images were loaded in CellProfiler and a pipeline developed to mask the area of the nucleus to exclude cytoplasmic staining and to measure the number of ‘spots’ per nucleus. For nuclear morphology, cells were imaged with a Zeiss Axiovert microscope at ×20 magnification and nuclear morphology was scored as either normal or nuclei with dysmorphic morphology were classed as blebs, invagination, micro-nucleoli or herniated nuclei as previously described^62^ by blind assessment.

### Cellular stress and apoptosis

Apoptosis, viability and cytotoxicity were assessed after heat shock, with the ApoTox-Glo Triplex Assay kit (Promega, cat. no. G6320) according to previously described methods with minor modifications^22^. To expose cells to heat shock stress, 10,000 fibroblasts per well were first grown at 37 °C in 96-well ELISA microplates and allowed to attach overnight. The following day the growth medium was exchanged with pre-warmed medium and the cells were moved into a 43 °C incubator for 2 h of heat stress exposure. Cells were then returned to 37 °C to recover for various time points. To provide a positive control for the induction of apoptosis, 2.5 µM staurosporine was added to the growth medium for 6 h. To provide a positive control for cytotoxicity, 70 µM digitonin was added to the culture medium for 15 min. Fluorescence (viability (400Ex/505Em) and cytotoxicity (485Ex/520Em)) and luminescence were measured on a FLUOstar Optima microplate reader (BMG Labtech).

### Orthogonal tests

Data were collected from referring clinicians about any diagnoses achieved using alternative genetic testing modalities.

### Clinical utility of results

Data on changes in management following ultra-rapid WGS were collected from referring clinicians via a REDCap survey 3 months post-result using a structured data collection instrument (Supplementary Table 5). These are grouped into three main categories: targeted treatments; redirection of care toward palliation; targeted surveillance (investigations and subspecialist referrals aimed at known complications).

### Statistics and reproducibility

No statistical method was used to predetermine sample size. Sample size was determined by the available funding. No data were excluded from the analyses.

### Reporting summary

Further information on research design is available in the Nature Portfolio Reporting Summary linked to this article.

## Online content

Any methods, additional references, Nature Portfolio reporting summaries, source data, extended data, supplementary information, acknowledgements, peer review information; details of author contributions and competing interests; and statements of data and code availability are available at 10.1038/s41591-023-02401-9.

## Supplementary information

Supplementary Information Supplementary Tables 1–5.

Reporting Summary

## Data Availability

De-identified genomic and associated data from this study are available for ethically approved research. Data access requests are accepted via an online application form that will require approval from the Australian Genomics Data Access Committee. For access to the data, please email AG-datarequest@mcri.edu.au. Data access requests are reviewed by the committee once a month. Access to the data will require a Data Transfer Agreement; once signed, the data will be transferred to the requestor from the Australian Genomics’ Genomic Data Repository. All variants reported in this study have been deposited in ClinVar (SUB13026601, SCV003921769 to SCV003922018). The mass spectrometry proteomics data have been deposited to the ProteomeXchange Consortium via the PRIDE partner repository with the dataset identifier PXD042001. The web resources used are: ClinVar: https://www.ncbi.nlm.nih.gov/clinvar GATK: https://gatk.broadinstitute.org gnomAD: https://gnomad.broadinstitute.org HPO: https://hpo.jax.org/app The Human Ancestry Ontology: https://www.ebi.ac.uk/ols/ontologies/hancestro IGV: https://software.broadinstitute.org/software/igv NCBI RefSeq: https://www.ncbi.nlm.nih.gov/refseq Manta: https://github.com/Illumina/manta OMIM: https://omim.org PanelApp Australia: https://panelapp.agha.umccr.org/ Schism: https://github.com/ssadedin/schism STRipy: https://stripy.org UniProt: https://www.uniprot.org/ VariantGrid: https://variantgrid.com VEP: https://ensembl.org/info/docs/tools/vep.Source data are provided with this paper.

## Source data

Source Data Fig. 5 Unprocessed western blots.

## Code availability

Schism identifies new variation in short-read whole-genome data without imposing a preconceived model, by comparing raw reads in test samples to those sampled from large scale aggregated control sets. The Schism code used during the study is available on GitHub at https://github.com/ssadedin/schism. The VariantGrid code (www.variantgrid.com) used during this study is available for research use under business source license 1.1 on GitHub at https://github.com/SACGF/variantgrid.
