# Supplementary material for: Integrated multi-omics for rapid rare disease diagnosis on a national scale
Source: Nat Med. 2023 Jun 8;29(7):1681–91. doi: 10.1038/s41591-023-02401-9 (PMC10353936; doi:10.1038/s41591-023-02401-9)
Supplement: Supplementary file 1 — Supplementary Tables 1–5. [file 41591_2023_2401_MOESM1_ESM.pdf]

nature medicine

Article https://doi.org/10.1038/s41591-023-02401-9

# Integrated multi-omics for rapid rare disease diagnosis on a national scale

In the format provided by the authors and unedited

**Supplementary Table 1:** Patients receiving a diagnosis (including partial or dual diagnoses) as part of the study, including presenting features, molecular diagnosis, and clinician-reported clinical utility.

| **Study ID** | **Phenotype (HPO terms)** | **Gene name Transcript number** | **Variant(s)** | **Diagnosis (condition, OMIM#, *further detail regarding partial diagnosis** )** | **Changes in medication** | **Improved process of care** | **Surgical procedures** | **Palliation** |
|---|---|---|---|---|---|---|---|---|
| A0131064 | HP:0004430\|Severe combined immunodeficiency | *IL2RG* NM_000206.2 | c.865C>T, p.(Arg289*), Pathogenic | Severe combined immunodeficiency MIM#300400 | | Yes | Informed BMT | |
| A0131065 | HP:0001695\|Cardiac arrest HP:0005184\|Prolonged QTc interval HP:0005150\|Abnormal atrioventricular conduction | *SCN5A* NM_198056.2 | c.1231G>A, p.(Val411Met), Pathogenic | Long QT syndrome MIM#603830 | Started enteral Mexilitine/Propranolol. Ceased Lignocaine/Esmolol | | Permanent pacemaker inserted | |
| A0131066 | HP:0001250\|Seizures | *KCNQ2* NM_172107.3 | c.2330_2331dupCC, p.(Glu778Profs*153), Pathogenic | Epileptic encephalopathy, early infantile, 7 MIM#613720 | Influenced choice of AED (sodium channel blocker) Other AEDs discontinued | Investigations and referrals rationalised | | |
| A0131067 | HP:0000612\|Iris coloboma HP:0002871\|Central apnea HP:0001537\|Umbilical hernia | *PITX2* NC_000004.12 | g.110650730-112833790del, p.?, Likely pathogenic | Axenfeld-Rieger syndrome, type 1 MIM#180500 | | Additional referral to opthalmology | | |
| A0131068 | HP:0200134\|Epileptic encephalopathy HP:0000260\|Wide anterior fontanel | *KCNQ2* NM_172107.3 | c.1678C>T, p.(Arg560Trp), Pathogenic | Epileptic encephalopathy, early infantile, 7 MIM#613720 | Influenced choice of AED. Other AEDs discontinued | | | |
| A0131070 | HP:0000127\|Renal salt wasting HP:0001941\|Acidosis HP:0002153\|Hyperkalemia HP:0002902\|Hyponatremia | *SCNN1A* NM_001038.5 | c.148delG, p.(Glu50Serfs*33), Pathogenic | Pseudohypoaldosteronism, type I MIM#264350 | Sodium supplements commenced. Hydrocortisone/fludrocortisone ceased. | Additional referral to respiratory | | |

| **Study ID** | **Phenotype (HPO terms)** | **Gene name Transcript number** | **Variant(s)** | **Diagnosis (condition, OMIM#, *further detail regarding partial diagnosis** )** | **Changes in medication** | **Improved process of care** | **Surgical procedures** | **Palliation** |
|---|---|---|---|---|---|---|---|---|
| A0131071 | HP:0000825\|Hyperinsulinemic hypoglycemia HP:0001250\|Seizures HP:0002240\|Hepatomegaly | *ABCC8* NM_001287174.1 | c.3776dupT, p.(Val1260Glyfs*147), Pathogenic | Hyperinsulinemic hypoglycemia, familial, 1 MIM#256450 | Ceased trial with diazoxide as unlikely to be responsive | | | |
| A0131072 | HP:0008066\|Abnormal blistering of the skin | *COL7A1* NM_000094.3 | c.8245G>A, p.(Gly2749Arg), Pathogenic | Epidermolysis bullosa dystrophica, AR MIM#226600 | | | | Yes |
| A0131074 | HP:0007334\|Generalized tonic-clonic seizures with focal onset | *KCNT1* NM_020822.2 | c.1283G>A, p.(Arg428Gln), Pathogenic | Epileptic encephalopathy, early infantile, 14 MIM#614959 | Rationalised medications. Ceased biotin, folinate and pyridoxine | Additional referral to developmental medicine | | |
| A0131076 | HP:0002804\|Arthrogryposis multiplex congenita HP:0000175\|Cleft palate HP:0000278\|Retrognathia HP:0000047\|Hypospadias HP:0000023\|Inguinal hernia HP:0006808\|Cerebral hypomyelination HP:0002144\|Tethered cord | *ZC4H2* NM_018684.3 | c.592C>T, p.(Arg198Trp), Pathogenic | Wieacker-Wolff syndrome MIM#314580 | | | | Yes |
| A0131078 | HP:0001943\|Hypoglycemia HP:0001667\|Right ventricular hypertrophy HP:0002240\|Hepatomegaly HP:0001263\|Global developmental delay HP:0001508\|Failure to thrive | *AGL* NM_000642.2 | c.3179C>G, p.(Ser1060*), Pathogenic<br>c.1020delA, p.(Glu340Aspfs*9), Pathogenic | Glycogen storage disease III MIM#232400 | Informed dietary management plan | | | |

| Study ID | Phenotype (HPO terms) | Gene name Transcript number | Variant(s) | Diagnosis (condition, OMIM#, *further detail regarding partial diagnosis** ) | Changes in medication | Improved process of care | Surgical procedures | Palliation |
|---|---|---|---|---|---|---|---|---|
| A0131081 | HP:0031165\|Multifocal seizures HP:0002197\|Generalized-onset seizure HP:0200134\|Epileptic encephalopathy | *KCNQ2* NM_172107.3 | c.629G>A, p.(Arg210His), Pathogenic | Epileptic encephalopathy, early infantile, 7 MIM#613720 | Oxcarbazepine commenced and Keppra ceased | | | |
| A0131085 | HP:0040168\|Focal seizures, afebril HP:0032052\|Focal cortical dysplasia type IIa | *PCDH19* NM_001184880.1 | c.498C>G, p.(Tyr166*), Pathogenic | X-linked early infantile epileptic encephalopathy 9 MIM#300088 | | Further neuroimaging cancelled | Epilepsy surgery cancelled due to low chance of success in light of genetic diagnosis | |
| A0131087 | HP:0001250\|Seizures HP:0200134\|Epileptic encephalopathy | *KCNQ2* NM_172107.3 | c.793G>A, p.Ala265Thr, Pathogenic | Autosomal dominant early infantile epileptic encephalopathy 7 MIM#613720 | Trileptal started. Ceased topiramate, phenobarbitone. Ceased metabolic cocktail of medicines | Metabolics no longer required | | |
| A0131088 | HP:0011682\|Perimembranous ventricular septal defect HP:0005950\|Laryngeal web HP:0001537\|Umbilical hernia HP:0000368\|Low-set, posteriorly rotated ears HP:0000316\|Hypertelorism HP:0001511\|Intrauterine growth retardation HP:0000347\|Micrognathia | *KMT2D* NM_003482.3 | c.10615C>T, p.(Arg3539Trp), Likely pathogenic | KMT2D-related malformation disorder MIM#147920 | | | | |
| A0131090 | HP:0004308\|Ventricular arrhythmia HP:0001695\|Cardiac arrest | *SCN5A* NM_198056.2 | c.4477_4479delAAG, p.(Lys1493del), Pathogenic | Brugada syndrome MIM#601144 | Inform titration of beta blocker doses. Avoidance of Burgada-associated drugs and amiodarone. | | | |

| Study ID | Phenotype (HPO terms) | Gene name Transcript number | Variant(s) | Diagnosis (condition, OMIM#, *further detail regarding partial diagnosis** ) | Changes in medication | Improved process of care | Surgical procedures | Palliation |
|---|---|---|---|---|---|---|---|---|
| A0131091 | HP:0001644\|Dilated cardiomyopathy HP:0001723\|Restrictive cardiomyopathy HP:0005184\|Prolonged QTc interval HP:0001649\|Tachycardia | *TNNT2* NM_001276345.1 | c.451C>T, p.(Arg151Trp), Pathogenic | TNNT2 -related cardiomyopathies | | Listed for cardiac transplant | | |
| A0131092 | HP:0002803\|Congenital contracture HP:0008935\|Generalized neonatal hypotonia HP:0001315\|Reduced tendon reflexes HP:0003236\|Elevated serum creatine kinase | *EXOSC3* NM_016042.3 | c.703G>A, p.(Gly235Arg), Likely pathogenic<br>c.395A>C, p.(Asp132Ala), Pathogenic | Autosomal recessive pontocerebellar hypoplasia, type 1B MIM#614678 | | | | Yes |
| A0131095 | HP:0001250\|Seizures | *PCDH19* NM_001184880.1 | c.1682C>G, p.(Pro561Arg), Pathogenic | Developmental and epileptic encephalopathy 9 MIM#300088 | | Referral to a neurologist with a special interest in PCDH19 associated epilepsy | | |
| A0131100 | HP:0001644\|Dilated cardiomyopathy HP:0001511\|Intrauterine growth retardation HP:0001873\|Thrombocytopenia | Ring X chromosome 46,X,r(X)(p21.1q21.1)[39]/45,X[16] | c. ?, p.?, Pathogenic | Turner syndrome | | Referral to endocrinology, endocrine surveillance initiated | | |
| A0131101 | HP:0000452\|Choanal stenosis HP:0001631\|Atrial septal defect HP:0002566\|Intestinal malrotation HP:0008757\|Unilateral vocal cord paralysis HP:0000391\|Thickened helices | *RERE* NM_001042681.1 | c.4313_4318dupTCCACC, p.(Leu1438_His1439dup), Pathogenic | Neurodevelopmental disorder with or without anomalies of the brain, eye, or heart MIM#616975 | | | | |

| Study ID | Phenotype (HPO terms) | Gene name Transcript number | Variant(s) | Diagnosis (condition, OMIM#, *further detail regarding partial diagnosis** ) | Changes in medication | Improved process of care | Surgical procedures | Palliation |
|---|---|---|---|---|---|---|---|---|
| A0131106 | HP:0002902\|Hyponatremia HP:0000103\|Polyuria HP:0003113\|Hypochloremia HP:0200114\|Metabolic alkalosis HP:0001561\|Polyhydramnios | *SLC12A1* NM_000338.2 | c.1966C>T, p.(Gln656*), Pathogenic<br>c.1432G>A, p.(Gly478Arg), Pathogenic | Bartter syndrome, type 1 MIM#601678 | Informed fluid and medication management in context of additional acute kidney injury | | | |
| A0131115 | HP:0006566\|Neonatal cholestatic liver disease HP:0001028\|Hemangioma HP:0002908\|Conjugated hyperbilirubinemia HP:0001511\|Intrauterine growth retardation HP:0008619\|Bilateral sensorineural hearing impairment | *ABCD1* / *BCAP31* Partial deletion of Xq28 involving *ABCD1* and *BCAP31* | c. ?, p.?, Pathogenic | Contiguous Abcd1/Dxs1375e deletion syndrome (CADDS) MIM#300475 | | | Avoided liver biopsy | Yes |
| A0131118 | HP:0011398\|Central hypotonia HP:0002376\|Developmental regression HP:0012762\|Cerebral white matter atrophy HP:0100716\|Self-injurious behavior HP:0002072\|Chorea HP:0002451\|Limb dystonia HP:0001507\|Growth abnormality | *KCNQ5* NM_001160133.1 | c.545C>T, p.(Ala182Val), Likely pathogenic | KCNQ5-related neurodevelopmental delay MIM#617601 | | Additional referral to neurologist with neurogenetic interest, Metabolic medicine no longer required to investigate further | | |
| A0131121 | HP:0011398\|Central hypotonia HP:0001348\|Brisk reflexes | *THOC2* NM_001081550.1 | c.229C>T, p.(Arg77Cys), Pathogenic | THOC2-related neurodevelopmental disorder MIM#300957 | | | | |

| Study ID | Phenotype (HPO terms) | Gene name Transcript number | Variant(s) | Diagnosis (condition, OMIM#, *further detail regarding partial diagnosis** ) | Changes in medication | Improved process of care | Surgical procedures | Palliation |
|---|---|---|---|---|---|---|---|---|
| A0131123 | HP:0001943\|Hypoglycemia HP:0001297\|Stroke HP:0001250\|Seizures HP:0002151\|Increased serum lactate | *MT-TL1* NC_012920.1 | m.3243A>G, p.?, Pathogenic | Mitochondrial encephalomyopathy with lactic acidosis and stroke-like episodes (MELAS), maternally inherited diabetes and deafness, and progressive external ophthalmoplegia PMIDs:11571698;23355809 | Collected multiple sample types to investigate level of heteroplasmy | | | |
| A0131128 | HP:0001987\|Hyperammonemia HP:0002910\|Elevated hepatic transaminase HP:0001928\|Abnormality of coagulation HP:0005543\|Reduced protein C activity | *ASS1* NM_054012.3 | c.370G>A, p.(Asp124Asn), Pathogenic | Citrullinemia MIM#215700 | | | Informed decision to proceed with liver transplant | |
| A0131137 | HP:0030682\|Left ventricular noncompaction | *MYH7* NM_000257.2 | c.1573G>A, p.(Glu525Lys), Pathogenic | MYH7-related cardiomyopathy MIM#613426 | | Investigations and referrals rationalised | Informed decision for cardiac transplant | |
| A0131139 | HP:0007431\|Congenital ichthyosiform erythroderma HP:0001776\|Bilateral talipes equinovarus HP:0001188\|Hand clenching | *SPINK5* NM_006846.3 | c.1111C>T, p.(Arg371*), Pathogenic<br>c.81+2T>A, p.?, Pathogenic | Netherton syndrome MIM#256500 | | Avoided skin biopsy | | |

| **Study ID** | **Phenotype (HPO terms)** | **Gene name Transcript number** | **Variant(s)** | **Diagnosis (condition, OMIM#, *further detail regarding partial diagnosis** )** | **Changes in medication** | **Improved process of care** | **Surgical procedures** | **Palliation** |
|---|---|---|---|---|---|---|---|---|
| A0131140 | HP:0100519\|Anuria<br>HP:0001919\|Acute kidney injury<br>HP:0001562\|Oligohydramnios<br>HP:0005932\|Abnormal renal corticomedullary differentiation<br>HP:0002092\|Pulmonary arterial hypertension<br>HP:0002107\|Pneumothorax<br>HP:0002089\|Pulmonary hypoplasia<br>HP:0001714\|Ventricular hypertrophy<br>HP:0005144\|Ventricular septal hypertrophy<br>HP:0010468\|Aplasia/Hypoplasia of the testes<br>HP:0000047\|Hypospadias | *WT1*<br>NM_024426.3 | c.1385G>A, p.(Arg462Gln), Pathogenic | Denys-Drash syndrome MIM#194080 | | Chromosome breakage studies initiated to help clarify relevance of VUS | | Yes |

| **Study ID** | **Phenotype (HPO terms)** | **Gene name Transcript number** | **Variant(s)** | **Diagnosis (condition, OMIM#, *further detail regarding partial diagnosis** )** | **Changes in medication** | **Improved process of care** | **Surgical procedures** | **Palliation** |
|---|---|---|---|---|---|---|---|---|
| A0131143 | HP:0002419\|Molar tooth sign on MRI HP:0010627\|Anterior pituitary hypoplasia HP:0001998\|Neonatal hypoglycemia HP:0002151\|Increased serum lactate | *KIAA0753* NM_014804.2 | c.1546-3C>A, p.(Asp439Glyfs*6), Pathogenic<br>c.1891A>T, p.(Lys631*), Pathogenic<br>c.2333T>C, p.(Met778Thr), Likely pathogenic | Joubert syndrome 38 MIM#619476 | | | | |
| A0131144 | HP:0002028\|Chronic diarrhea | *SPINT2* NM_021102.3 | c.421C>G, p.(Pro141Ala), Likely pathogenic | Congenital secretory sodium diarrhea 3 MONDO:0010036 | | | | |
| A0131145 | HP:0100026\|Arteriovenous malformation HP:0002408\|Cerebral arteriovenous malformation HP:0001250\|Seizures | *ENG* NM_001114753.2 | c.1470dup, p.(Asp491Argfs*10), Pathogenic | Hereditary haemorrhagic telangiectasia, type 1 MIM#187300 | | Longterm HHT surveillance | | |
| A0131146 | HP:0031677\|Polymorphic ventricular tachycardia HP:0004756\|Ventricular tachycardia HP:0001688\|Sinus bradycardia HP:0001657\|Prolonged QT interval HP:0001664\|Torsade de pointes HP:0001663\|Ventricular fibrillation HP:0007018\|Attention deficit hyperactivity disorder | *RYR2* NM_001035.2 | c.506G>A, p.(Arg169Gln), Pathogenic | Catecholaminergic polymorphic ventricular tachycardia (CPVT) 1 MIM#604772 | Flecainide added to Nadolol | Electrophysiological study not necessary | Left cardiac sympathectomy performed and avoided ICD implantation | |

| Study ID | Phenotype (HPO terms) | Gene name Transcript number | Variant(s) | Diagnosis (condition, OMIM#, *further detail regarding partial diagnosis** ) | Changes in medication | Improved process of care | Surgical procedures | Palliation |
|---|---|---|---|---|---|---|---|---|
| A0131148 | HP:0007932\|Bilateral congenital mydriasis HP:0001643\|Patent ductus arteriosus HP:0001631\|Atrial septal defect HP:0001297\|Stroke HP:0009145\|Abnormal cerebral artery morphology HP:0002079\|Hypoplasia of the corpus callosum | *ACTA2* NM_001613.2 | c.535C>T, p.(Arg179Cys), Pathogenic | Multisystemic smooth muscle dysfunction syndrome MIM#613834 | | Baseline whole body MRA/MRV and additional referral to cardiology | | |
| A0131151 | HP:0011398\|Central hypotonia HP:0002093\|Respiratory insufficiency HP:0032407\|Bilateral perisylvian polymicrogyria HP:0002508\|Brainstem dysplasia HP:0001250\|Seizures HP:0001518\|Small for gestational age HP:0001558\|Decreased fetal movement HP:0000046\|Scrotal hypoplasia | *HSD17B4* NM_000414.3 | c.1517G>A, p.(Arg506His), Pathogenic<br>c.31dupG, p.(Val11Glyfs*30), Pathogenic | D-bifunctional protein deficiency MIM#261515 | | | | Yes |

| Study ID | Phenotype (HPO terms) | Gene name Transcript number | Variant(s) | Diagnosis (condition, OMIM#, *further detail regarding partial diagnosis** ) | Changes in medication | Improved process of care | Surgical procedures | Palliation |
|---|---|---|---|---|---|---|---|---|
| A0131152 | HP:0001601\|Laryngomalacia HP:0008738\|Partially duplicated kidney HP:0006989\|Dysplastic corpus callosum HP:0000028\|Cryptorchidism HP:0001631\|Atrial septal defect HP:0000776\|Congenital diaphragmatic hernia HP:0025386\|Bitemporal hollowing HP:0000431\|Wide nasal bridge HP:0000368\|Low-set, posteriorly rotated ears HP:0000998\|Hypertrichosis | *ARID1B* NM_020732.3 | c.2281G>C, p.(Gly761Arg), Likely pathogenic | Coffin -Siris syndrome 1 MIM#135900 | | | | |
| A0131153 | HP:0012622\|Chronic kidney disease HP:0000113\|Polycystic kidney dysplasia HP:0000093\|Proteinuria HP:0000105\|Enlarged kidney HP:0002902\|Hyponatremia HP:0003113\|Hypochloremia HP:0001508\|Failure to thrive HP:0001270\|Motor delay | *INVS* NM_014425.4 | c.2719C>T, p.(Arg907*), Pathogenic<br>c.3062_3063del, p.(Lys1021Serfs*20), Likely pathogenic | Infantile nephronophthisis 2 MIM#602088 | | | Avoided renal biopsy | |
| A0131154 | HP:0001695\|Cardiac arrest | *RYR2* NM_001035.2 | c.14297A>C, p.(Gln4766Pro), Likely pathogenic | Catecholaminergic polymorphic ventricular tachycardia (CPVT) 1 MIM#604772 | | Electrophysiological study not necessary | Avoided ICD implantation | |
| A0131155 | HP:0007506\|Congenital absence of skin of limbs HP:0008066\|Abnormal blistering of the skin | *COL7A1* NM_000094.3 | c.3840delC, p.(Gly1281Valfs*44), Pathogenic | Epidermolysis bullosa dystrophica MIM#226600 | | Avoided skin biopsy | | |

| **Study ID** | **Phenotype (HPO terms)** | **Gene name Transcript number** | **Variant(s)** | **Diagnosis (condition, OMIM#, *further detail regarding partial diagnosis** )** | **Changes in medication** | **Improved process of care** | **Surgical procedures** | **Palliation** |
|---|---|---|---|---|---|---|---|---|
| | HP:0200097\|Oral mucosal blisters HP:0031446\|Erosion of oral mucosa | | c.7474C>T, p.(Arg2492*), Pathogenic | | | | | |
| A0131157 | HP:0000452\|Choanal stenosis HP:0000237\|Small anterior fontanelle HP:0002007\|Frontal bossing HP:0430026\|Abnormality of the shape of the midface HP:0001629\|Ventricular septal defect | *FGFR3* NM_000142.4 | c.749C>G, p.(Pro250Arg), Pathogenic | Muenke syndrome MIM#602849 | | Referral to combined craniofacial/neurosurgery clinic | | |
| A0131158 | HP:0005684\|Distal arthrogryposis HP:0001631\|Atrial septal defect HP:0001274\|Agenesis of corpus callosum HP:0003316\|Butterfly vertebrae HP:0001884\|Talipes calcaneovalgus HP:0000003\|Multicystic kidney dysplasia HP:0000062\|Ambiguous genitalia HP:0000347\|Micrognathia HP:0000278\|Retrognathia HP:0000369\|Low-set ears HP:0000463\|Anteverted nares HP:0000506\|Telecanthus HP:0000431\|Wide nasal bridge HP:0010557\|Overlapping fingers HP:0000960\|Sacral dimple | *ALG3* NM_005787.5 | c.512G>A, p.(Arg171Gln), Pathogenic | Congenital disorder of glycosylation, type Id MIM#601110 | | Additional referral to metabolic medicine and transferrin isoforms ordered | | Yes |

| Study ID | Phenotype (HPO terms) | Gene name Transcript number | Variant(s) | Diagnosis (condition, OMIM#, *further detail regarding partial diagnosis** ) | Changes in medication | Improved process of care | Surgical procedures | Palliation |
|---|---|---|---|---|---|---|---|---|
| A0331029 | HP:0002023\|Anal atresia HP:0000825\|Hyperinsulinemic hypoglycemia HP:0000952\|Jaundice HP:0001905\|Congenital thrombocytopenia HP:0002901\|Hypocalcemia HP:0002119\|Ventriculomegaly HP:0002416\|Subependymal cysts HP:0000954\|Single transverse palmar crease HP:0031703\|Abnormal ear morphology HP:0002007\|Frontal bossing | *KMT2D* NM_003482.3 | c.3190del, p.(Val1064Serfs*55), Pathogenic | Kabuki syndrome 1 MIM#147920 | | Referral to immunology. | | |
| A0331032 | HP:0001508\|Failure to thrive HP:0011342\|Mild global developmental delay HP:0012469\|Infantile spasms HP:0000737\|Irritability HP:0000496\|Abnormality of eye movement | *UBA5* NM_024818.4 | c.280A>G, p.(Arg94Gly), Likely pathogenic<br>c.1111G>A, p.(Ala371Thr), Pathogenic | Developmental and epileptic encephalopathy 44 MIM#617132 | | | | Yes |

| **Study ID** | **Phenotype (HPO terms)** | **Gene name Transcript number** | **Variant(s)** | **Diagnosis (condition, OMIM#, *further detail regarding partial diagnosis** )** | **Changes in medication** | **Improved process of care** | **Surgical procedures** | **Palliation** |
|---|---|---|---|---|---|---|---|---|
| A0331033 | HP:0001684\|Secundum atrial septal defect HP:0011451\|Congenital microcephaly HP:0011667\|Bilateral superior vena cava with bridging vein HP:0000175\|Cleft palate HP:0002948\|Vertebral fusion HP:0000085\|Horseshoe kidney HP:0000800\|Cystic renal dysplasia HP:0008689\|Bilateral cryptorchidism HP:0000377\|Abnormality of the pinna HP:0045075\|Sparse eyebrow HP:0000455\|Broad nasal tip HP:0000309\|Abnormality of the midface HP:0000347\|Micrognathia HP:0001792\|Small nail | *KMT2D* NM_003482.3 | c.11119C>T, p.(Arg3707*), Pathogenic | Kabuki syndrome 1 MIM#147920 | | Additional referral to developmental paediatrics and cardiology. | | |
| A0331034 | HP:0001250\|Seizures HP:0000494\|Downslanted palpebral fissures HP:0200134\|Epileptic encephalopathy HP:0002376\|Developmental regression HP:0011343\|Moderate global developmental delay HP:0006863\|Severe expressive language delay | *NSD1* NM_022455.4 | c.4919G>A, p.(Cys1640Tyr), Pathogenic | Sotos syndrome 1 MIM#117550 | Rationalised AED medications. | Cardiac echo and renal ultrasound ordered | | |

| Study ID | Phenotype (HPO terms) | Gene name<br>Transcript number | Variant(s) | Diagnosis (condition, OMIM#, *further detail regarding partial diagnosis** ) | Changes in medication | Improved process of care | Surgical procedures | Palliation |
|---|---|---|---|---|---|---|---|---|
| A0331035 | HP:0002376\|Developmental regression HP:0001263\|Global developmental delay HP:0000505\|Visual impairment HP:0000365\|Hearing impairment HP:0001276\|Hypertonia HP:0001257\|Spasticity HP:0012171\|Stereotypical hand wringing HP:0002121\|Absence seizure HP:0012444\|Brain atrophy HP:0002415\|Leukodystrophy HP:0002463\|Language impairment | *PPT1*<br>NM_000310.3 | c.169dupA, p.(Met57Asnfs*45), Pathogenic<br>c.451C>T, p.(Arg151*), Pathogenic | Neuronal ceroid lipofuscinosis 1 MIM#256730 | | Avoided lumbar puncture and other investigations. | | Yes |
| A0331039 | HP:0032471\|Focal polymicrogyria HP:0001873\|Thrombocytopenia HP:0002092\|Pulmonary arterial hypertension HP:0007371\|Corpus callosum atrophy | *KDM5B*<br>NM_006618.4 | c.2117del, p.(Cys706Phefs*14), Pathogenic | KDM5B-related intellectual disability with dysmorphic features and KDM5B-related intellectual disability with incomplete penetrance MIM#618109;PMID:30409806 | | | | |
| A0331040 | HP:0007023\|Antenatal intracerebral hemorrhage HP:0001873\|Thrombocytopenia HP:0001875\|Neutropenia [illegible] | *MPL*<br>NM_005373.2 | c.712G>T, p.(Gly238Cys), Likely pathogenic | Amegakaryocytic thrombocytopenia MIM#604498 | | | Informed decision about BMT | |

| Study ID | Phenotype (HPO terms) | Gene name Transcript number | Variant(s) | Diagnosis (condition, OMIM#, *further detail regarding partial diagnosis** ) | Changes in medication | Improved process of care | Surgical procedures | Palliation |
|---|---|---|---|---|---|---|---|---|
| | HP:0005548 \| Megakaryocytopenia HP:0012143 \| Abnormal megakaryocyte morphology HP:0031385 \| Megakaryocyte nucleus hypolobulation | | c.305G>C, p.(Arg102Pro), Pathogenic | | | | | |
| A0331041 | HP:0012469 \| Infantile spasms HP:0200134 \| Epileptic encephalopathy HP:0000256 \| Macrocephaly HP:0001263 \| Global developmental delay HP:0002194 \| Delayed gross motor development HP:0000505 \| Visual impairment HP:0000365 \| Hearing impairment HP:0010846 \| EEG with persistent abnormal rhythmic activity HP:0001252 \| Muscular hypotonia HP:0001548 \| Overgrowth HP:0001357 \| Plagiocephaly HP:0000248 \| Brachycephaly HP:0002265 \| Large fleshy ears | *CHD3* NM_001005273.2 | c.2785C>T, p.(Leu929Phe), Pathogenic | Snijders Blok-Campeau syndrome MIM#618205 | | Echo, renal ultrasound, ophthalmology investigations ordered | | |
| A0331044 | HP:0007359 \| Focal-onset seizure HP:0002901 \| Hypocalcemia HP:0002917 \| Hypomagnesemia HP:0000836 \| Hyperthyroidism | *TRPM6* NM_017662.4 | c.841+1G>A, p.?, Pathogenic | Hypomagnesaemia 1 MIM#602014 | Treatment with magnesium supplements | | | |
| A0331045 | HP:0030280 \| Rib gap HP:0000201 \| Pierre-Robin sequence | *SNRPB* NM_003091.3 | c.155+301G>C, p.?, Pathogenic | Cerebrocostomandibular syndrome MIM#117650 | | | | |

| **Study ID** | **Phenotype (HPO terms)** | **Gene name Transcript number** | **Variant(s)** | **Diagnosis (condition, OMIM#, *further detail regarding partial diagnosis** )** | **Changes in medication** | **Improved process of care** | **Surgical procedures** | **Palliation** |
|---|---|---|---|---|---|---|---|---|
| A0331046 | HP:0011834\|Moyamoya phenomenon<br>HP:0002123\|Generalized myoclonic seizures | *CBL*<br>NM_005188.3 | c.1096-2A>T, p.?, Pathogenic | CBL-related disorder | | Echocardiogram | | |
| A0331047 | HP:0001290\|Generalized hypotonia<br>HP:0001265\|Hyporeflexia<br>HP:0002098\|Respiratory distress<br>HP:0040288\|Nasogastric tube feeding | *RYR1*<br>NM_000540.2 | c.5989G>A, p.(Glu1997Lys), Pathogenic | RYR1-related myopathy MONDO:0100150 | | Malignant hyperthermia precautions initiated with anaesthetics | | |
| A0331051 | HP:0001297\|Stroke | *ETHE1*<br>NM_014297.4 | c.131_132delAG, p.(Glu44Valfs*62), Pathogenic | Ethylmalonic encephalopathy MIM#602473 | Specially formulated parenteral nutrition and vitamins. Additional treatments to reduce the generation of toxins. | | | |
| A0431018 | HP:0002804\|Arthrogryposis multiplex congenita<br>HP:0002650\|Scoliosis<br>HP:0002827\|Hip dislocation<br>HP:0012469\|Infantile spasms<br>HP:0011968\|Feeding difficulties<br>HP:0001537\|Umbilical hernia<br>HP:0001263\|Global developmental delay | *SNAP25*<br>NM_130811.3 | c.589C>T, p.(Gln197*), Likely pathogenic | SNAP25-related disorder | | Nerve conduction studies initiated | | |

| Study ID | Phenotype (HPO terms) | Gene name Transcript number | Variant(s) | Diagnosis (condition, OMIM#, *further detail regarding partial diagnosis** ) | Changes in medication | Improved process of care | Surgical procedures | Palliation |
|---|---|---|---|---|---|---|---|---|
| A0431019 | HP:0010512\|Adrenal calcification HP:0001922\|Vacuolated lymphocytes HP:0003281\|Increased serum ferritin HP:0001928\|Abnormality of coagulation HP:0002240\|Hepatomegaly HP:0001744\|Splenomegaly HP:0001541\|Ascites HP:0001876\|Pancytopenia HP:0001954\|Recurrent fever HP:0002151\|Increased serum lactate | *LIPA* NM_000235.3 | c.524A>C, p.(Gln175Pro), Likely pathogenic | Wolman disease MIM#278000 | Sebelipase alfa enzyme therapy for Wolman Disease | | | |
| | | UPD10 | UPD10, Pathogenic | | | | | |
| A0431021 | HP:0003072\|Hypercalcemia HP:0000843\|Hyperparathyroidism HP:0000938\|Osteopenia HP:0002917\|Hypomagnesemia HP:0002900\|Hypokalemia | *CASR* NM_000388.3 | c.190A>G, p.(Asn64Asp), Likely pathogenic | Neonatal hyperparathyroidism MIM#239200 | Medication started based on result | Investigations and referrals rationalised | Avoided thyroid biopsy | |
| | | | c.101T>C, p.(Leu34Pro), Likely pathogenic | | | | | |
| A0431025 | HP:0001319\|Neonatal hypotonia HP:0002871\|Central apnea HP:0000278\|Retrognathia HP:0000237\|Small anterior fontanelle HP:0000193\|Bifid uvula HP:0001662\|Bradycardia HP:0001943\|Hypoglycemia | *KCNK9* NM_001282534.1 | c.706G>A, p.(Gly236Arg), Pathogenic | Birk-Barel syndrome MIM#612292 | | Sleep study ordered | | |
| A0431030 | HP:0005318\|Cerebral vasculitis HP:0002140\|Ischemic stroke HP:0000965\|Cutis marmorata | *ADA2* NM_001282225.2 | c.336C>G, p.(His112Gln), Pathogenic | Vasculitis due to ADA2 deficiency MONDO:0014306 | Added TNF-alpha inhibitor and reduced steroid | Reduced involement from cardiology | | |

| Study ID | Phenotype (HPO terms) | Gene name Transcript number | Variant(s) | Diagnosis (condition, OMIM#, *further detail regarding partial diagnosis** ) | Changes in medication | Improved process of care | Surgical procedures | Palliation |
|---|---|---|---|---|---|---|---|---|
| | HP:0000678\|Dental crowding HP:0000750\|Delayed speech and language development HP:0002236\|Frontal upsweep of hair HP:0025252\|Geographic tongue | | c.752C>T, p.(Pro251Leu), Pathogenic | | dose | | | |
| A0431032 | HP:0008905\|Rhizomelia HP:0001876\|Pancytopenia HP:0000050\|Hypoplastic male external genitalia HP:0009755\|Ankyloblepharon HP:0001643\|Patent ductus arteriosus HP:0001943\|Hypoglycemia HP:0002098\|Respiratory distress | *KAT6B* NM_012330.3 | c.5302C>T, p.(Gln1768*), Pathogenic | KAT6B-related disorder MIM#603736 | | Additional referral to audiology, ophthalmology, endocrinology, physiotherapy | | |
| A0431034 | HP:0011675\|Arrhythmia HP:0001663\|Ventricular fibrillation HP:0000717\|Autism HP:0010865\|Oppositional defiant disorder | *RYR2* NM_001035.2 | c.14311G>A, p.(Val4771Ile), Pathogenic | Catecholaminergic polymorphic ventricular tachycardia (CPVT) 1 MIM#604772 | | | Insertion of AICD | |
| A0731001 | HP:0002575\|Tracheo-oesophageal fistula HP:0002744\|Bilateral cleft lip and palate HP:0001629\|Ventricular septal defect HP:0001172\|Abnormal thumb morphology HP:0000394\|Lop ear | *CHD7* NM_017780.2 | c.4393C>T, p.(Arg1465*), Pathogenic | CHARGE syndrome MIM#214800 | | Additional referral to ophthalmologist and ENT | | |

| Study ID | Phenotype (HPO terms) | Gene name Transcript number | Variant(s) | Diagnosis (condition, OMIM#, *further detail regarding partial diagnosis** ) | Changes in medication | Improved process of care | Surgical procedures | Palliation |
|---|---|---|---|---|---|---|---|---|
| A0731003 | HP:0004383\|Hypoplastic left heart HP:0001321\|Cerebellar hypoplasia HP:0000218\|High palate HP:0009110\|Diaphragmatic eventration | *KMT2D* NM_003482.3 | c.7997_8003dup, p.(Met2669Profs*7), Pathogenic | Kabuki syndrome MIM#147920 | | | | Yes |
| A0731008 | HP:0004898\|Persistent lactic acidosis HP:0001511\|Intrauterine growth retardation HP:0001643\|Patent ductus arteriosus HP:0001998\|Neonatal hypoglycemia HP:0002490\|Increased CSF lactate HP:0001319\|Neonatal hypotonia | *ACAD9* NM_014049.4 | c.1169C>T, p.(Ala390Val), Likely pathogenic<br>c.1185_1188delTGAG, p.(Ser395Argfs*43), Pathogenic | Nuclear type 20 mitochondrial complex I deficiency MIM#611126 | | Referred to neurometabolic clinic | Avoided muscle and skin biopsy | |
| A0731009 | HP:0011342\|Mild global developmental delay HP:0002133\|Status epilepticus | *DNM1L* NM_012062.4 | c.1207C>T, p.(Arg403Cys), Pathogenic | Encephalopathy due to defective mitochondrial peroxisomal fission 1 MIM#614388 | | | | |
| A0731010 | HP:0001644\|Dilated cardiomyopathy HP:0001639\|Hypertrophic cardiomyopathy HP:0012817\|Noncompaction cardiomyopathy HP:0001698\|Pericardial effusion HP:0004979\|Metaphyseal sclerosis HP:0001873\|Thrombocytopenia | *DSP* NM_004415.2 | c.8077_8080delAAAG, p.(Lys2693Profs*3), Pathogenic | Dilated cardiomyopathy MIM#615821 | | Investigations and referral rationalised | | Yes |

| Study ID | Phenotype (HPO terms) | Gene name<br>Transcript number | Variant(s) | Diagnosis (condition, OMIM#, *further detail regarding partial diagnosis** ) | Changes in medication | Improved process of care | Surgical procedures | Palliation |
|---|---|---|---|---|---|---|---|---|
| A0731012 | HP:0200134\|Epileptic encephalopathy | *BSCL2*<br>NM_001122955.3 | c.446C>G, p.(Pro149Arg), Likely pathogenic | Developmental and epileptic encephalopathy, BSCL2-related, autosomal dominant. MONDO:0100062 | | | | Yes |
| A0831001 | HP:0200134\|Epileptic encephalopathy<br>HP:0001336\|Myoclonus | *STXBP1*<br>NM_003165.3 | c.1651C>T, p.(Arg551Cys), Pathogenic | STXBP1-related epileptic encephalopathy MIM#612164 | Ceased pyridoxine | Ceased metabolic investigations | | Yes |
| A0831016 | HP:0002133\|Status epilepticus<br>HP:0002194\|Delayed gross motor development HP:0010851\|EEG with burst suppression | *POLG*<br>NM_002693.2 | c.1399G>A, p.(Ala467Thr), Pathogenic<br>c.3538_3539dup TT, p.(Ala1182Valfs*13), Pathogenic | Mitochondrial DNA depletion syndrome 4A (Alpers type) MIM#203700 | Rationalised AED medications. | | | Yes |
| A0831017 | HP:0012622\|Chronic kidney disease<br>HP:0000822\|Hypertension<br>HP:0012593\|Nephrotic range proteinuria<br>HP:0002907\|Microscopic hematuria | *MYH9*<br>NM_002473.5 | c.2680G>A, p.(Glu894Lys), Likely pathogenic | Macrothrombocytopenia and granulocyte inclusions with or without nephritis or sensorineural hearing loss MIM#155100 | Commenced haemodialysis | | | |

| Study ID | Phenotype (HPO terms) | Gene name Transcript number | Variant(s) | Diagnosis (condition, OMIM#, *further detail regarding partial diagnosis** ) | Changes in medication | Improved process of care | Surgical procedures | Palliation |
|---|---|---|---|---|---|---|---|---|
| A0831022 | HP:0002803\|Congenital contracture HP:0002878\|Respiratory failure HP:0002104\|Apnea HP:0001662\|Bradycardia HP:0000347\|Micrognathia HP:0000369\|Low-set ears HP:0010851\|EEG with burst suppression HP:0002374\|Diminished movement | *SCN2A* NM_001040142.2 | c.1267G>C, p.(Val423Leu), Pathogenic | Developmental and epileptic encephalopathy 11 MIM#613721 | | | | Yes |
| A0831023 | HP:0002092\|Pulmonary arterial hypertension HP:0005162\|Left ventricular dysfunction HP:0001629\|Ventricular septal defect HP:0000260\|Wide anterior fontanel | *AFF4* NM_014423.4 | c.778A>G, p.(Met260Val), Likely pathogenic | CHOPS syndrome MIM#616368 | | | | Yes |
| A0831027 | HP:0000776\|Congenital diaphragmatic hernia HP:0008689\|Bilateral cryptorchidism HP:0000347\|Micrognathia HP:0000047\|Hypospadias HP:0000126\|Hydronephrosis HP:0001561\|Polyhydramnios HP:0010880\|Increased nuchal translucency | *PBX1* NM_002585.4 | c.700C>T, p.(Arg234Trp), Pathogenic | Congenital anomalies of kidney and urinary tract syndrome with or without hearing loss, abnormal ears, or developmental delay or CAKUTHED MIM#617641 | | | | Yes |

| Study ID | Phenotype (HPO terms) | Gene name Transcript number | Variant(s) | Diagnosis (condition, OMIM#, *further detail regarding partial diagnosis** ) | Changes in medication | Improved process of care | Surgical procedures | Palliation |
|---|---|---|---|---|---|---|---|---|
| A0831028 | HP:0100257\|Ectrodactyly HP:0010760\|Absent toe HP:0001274\|Agenesis of corpus callosum HP:0001629\|Ventricular septal defect HP:0001545\|Anteriorly placed anus HP:0001320\|Cerebellar vermis hypoplasia HP:0000278\|Retrognathia HP:0000494\|Downslanted palpebral fissures HP:0000369\|Low-set ears | *DDX3X* NM_001356.4 | c.1600C>T, p.(Arg534Cys), Pathogenic | Intellectual developmental disorder, Snijders Blok type syndrome MIM#300958 | | | | Yes |
| A1131007 | HP:0011398\|Central hypotonia HP:0001319\|Neonatal hypotonia HP:0001263\|Global developmental delay HP:0002871\|Central apnea HP:0032114\|Saccadic intrusion HP:0004305\|Involuntary movements HP:0100703\|Tongue thrusting HP:0001290\|Generalized hypotonia | *DNM1L* NM_012062.4 | c.115A>C, p.(Ser39Arg), Likely pathogenic | Encephalopathy, lethal, due to defective mitochondrial peroxisomal fission 1 MIM#614388 | | Cancelled investigations: ERG, VER, repeat MRI brain. Skin biopsy and fibroblast testing initiated | | Yes |
| A1131010 | HP:0002376\|Developmental regression HP:0001250\|Seizures | *PRRT2* NM_001256442.1 | c.1001T>A, p.(Ile334Asn), Likely pathogenic | Seizures MIM#605751 | | | | |
| A1131013 | HP:0001250\|Seizures HP:0200134\|Epileptic encephalopathy | *KCNQ2* NM_172107.3 | c.829A>G, p.(Thr277Ala), Pathogenic | Epileptic encephalopathy, early infantile MIM#613720 | Rationalised AED medications. | | | |

| **Study ID** | **Phenotype (HPO terms)** | **Gene name Transcript number** | **Variant(s)** | **Diagnosis (condition, OMIM#, *further detail regarding partial diagnosis** )** | **Changes in medication** | **Improved process of care** | **Surgical procedures** | **Palliation** |
|---|---|---|---|---|---|---|---|---|
| A1131014 | HP:0000252\|Microcephaly HP:0001339\|Lissencephaly HP:0001274\|Agenesis of corpus callosum HP:0001263\|Global developmental delay HP:0100704\|Cerebral visual impairment HP:0011476\|Profound sensorineural hearing impairment | *WDR81* NM_001163673.1 | c.234C>A, p.(Tyr78*), Pathogenic<br>c.1802G>T, p.(Gly601Val), Likely pathogenic | WDR81-related neurodevelopmental disorder | | | | Yes |
| A1131015 | HP:0001250\|Seizures HP:0001290\|Generalized hypotonia HP:0001662\|Bradycardia HP:0011968\|Feeding difficulties | *PURA* NM_005859.4 | c.228delC, p.(Asp76Glufs*2), Pathogenic | Mental retardation MIM#616158 | Rationalised AED medications. | Echocardiogram and renal ultrasound ordered | | |
| A1131017 | HP:0001943\|Hypoglycemia HP:0003128\|Lactic acidosis HP:0001399\|Hepatic failure HP:0001635\|Congestive heart failure HP:0001951\|Episodic ammonia intoxication | *TAFAZZIN* NM_000116.3 | c.532T>A, p.(Phe178Ile), Likely pathogenic | Barth syndrome MIM#302060 | | | | Yes |
| A1131021 | HP:0001263\|Global developmental delay HP:0001915\|Aplastic anemia HP:0001513\|Obesity HP:0000098\|Tall stature HP:0100543\|Cognitive impairment HP:0000391\|Thickened helices HP:0011228\|Horizontal eyebrow HP:0000256\|Macrocephaly | *GRIN1* NM_007327.3 | c.421G>A, p.(Val141Met), Likely pathogenic | Autosomal dominant neurodevelopmental disorder with or without hyperkinetic movements and seizures MIM#614254 | | | Informed BMT | |

| Study ID | Phenotype (HPO terms) | Gene name Transcript number | Variant(s) | Diagnosis (condition, OMIM#, *further detail regarding partial diagnosis** ) | Changes in medication | Improved process of care | Surgical procedures | Palliation |
|---|---|---|---|---|---|---|---|---|
| A1131024 | HP:0004713\|Reversible renal failure HP:0001878\|Hemolytic anemia HP:0005575\|Hemolytic-uremic syndrome | *CFH* operon NM_000186.4 | c. ?, p.?, Pathogenic | Atypical haemolytic uraemic syndrome (aHUS) MIM#612923;235400 | Established need for long term eculizumab. No ongoing need for plasma exchange | | Renal biopsy cancelled | |
| | | *CFI* NM_000204.3 | c.355G>A, p.(Gly119Arg), Pathogenic | | | | | |
| | | *CFTR* NM_000492.3 | c.1521_1523delCTT, p.(Phe508del), Pathogenic | Cystic fibrosis MIM# 219700 | | | | |
| A1131026 | HP:0002507\|Semilobar holoprosencephaly HP:0000252\|Microcephaly HP:0009099\|Median cleft palate HP:0100336\|Bilateral cleft lip HP:0000612\|Iris coloboma HP:0003228\|Hypernatremia HP:0000873\|Diabetes insipidus HP:0001250\|Seizures HP:0005285\|Absent nasal bridge | *SHH* NM_000193.3 | c.1-3225_300+2938del, p.?, Likely pathogenic | Holoprosencephaly 3 MIM#142945 | | | | |
| A1131027 | HP:0002049\|Proximal renal tubular acidosis HP:0001994\|Renal Fanconi syndrome HP:0002900\|Hypokalemia HP:0002148\|Hypophosphatemia HP:0001942\|Metabolic acidosis HP:0001824\|Weight loss | *CTNS* NC_000017.11 | g.3600934_3658165del, p.?, Pathogenic | Nephropathic cystinosis MIM#219800 | Cystagon- specific therapy started | Investigations and referrals rationalised | | |

| Study ID | Phenotype (HPO terms) | Gene name Transcript number | Variant(s) | Diagnosis (condition, OMIM#, *further detail regarding partial diagnosis** ) | Changes in medication | Improved process of care | Surgical procedures | Palliation |
|---|---|---|---|---|---|---|---|---|
| A1131028 | HP:0001298\|Encephalopathy HP:0001290\|Generalized hypotonia HP:0001284\|Areflexia HP:0000252\|Microcephaly HP:0001873\|Thrombocytopenia HP:0100502\|Vitamin B12 deficiency HP:0010919\|Abnormal circulating homocysteine concentration HP:0010901\|Abnormal circulating methionine concentration | *MTHFR* NM_005957.4 | c.1013T>C, p.(Met338Thr), Pathogenic<br>c.1699C>T, p.(Arg567*), Pathogenic | MTHFR deficiency MIM#236250 | S-adensyl methionine started. Vitamin B12 intramuscrular injection adjusted. | | | |
| A1131029 | HP:0001250\|Seizures HP:0002791\|Hypoventilation HP:0000639\|Nystagmus HP:0000273\|Facial grimacing HP:0012332\|Abnormal autonomic nervous system physiology | *WWOX* NM_016373.3 | c.982_998del, p.(Tyr328Glnfs*195), Pathogenic<br>c.606-1779_792-2745del, p.(Pro203_Arg264del), Pathogenic | Developmental and epileptic encephalopathy 28 MIM#616211 | Rationalised AED medications. | Investigations and referrals rationalised | | Yes |
| A1131032 | HP:0003774\|Stage 5 chronic kidney disease HP:0000100\|Nephrotic syndrome HP:0000112\|Nephropathy HP:0000822\|Hypertension HP:0001513\|Obesity HP:0000316\|Hypertelorism HP:0000486\|Strabismus | *ACTN4* NM_004924.5 | c.584G>T, p.(Gly195Val), Pathogenic | Glomerulosclerosis, focal segmental, 1 MIM#603278 | | | | |

| **Study ID** | **Phenotype (HPO terms)** | **Gene name Transcript number** | **Variant(s)** | **Diagnosis (condition, OMIM#, *further detail regarding partial diagnosis** )** | **Changes in medication** | **Improved process of care** | **Surgical procedures** | **Palliation** |
|---|---|---|---|---|---|---|---|---|
| A1131033 | HP:0002098\|Respiratory distress HP:0002789\|Tachypnea HP:0001371\|Flexion contracture HP:0011448\|Ankle clonus HP:0000851\|Congenital hypothyroidism HP:0000028\|Cryptorchidism HP:0000252\|Microcephaly HP:0001511\|Intrauterine growth retardation | *RAD51* NM_002875.4 | c.590C>T, p.(Thr197Ile), Likely pathogenic | Complementation group R fanconi anemia MIM#617244 | | Additional referral to haematology, ophthalmology, endocrinology | | |
| A1131034 | HP:0000093\|Proteinuria HP:0003259\|Elevated serum creatinine | *WT1* NM_024426.3 | c.1432+1G>A, p.?, Pathogenic | Frasier syndrome MIM#136680 | | Additional referral to endocrine, surgical, sex disorders multidisciplinary team. | Imaging of the pelvis that revealed dysplastic gonads, leading to gonad biopsy revealing bilateral gonadoblastomas followed by the bilateral gonadectomy. | |

| Study ID | Phenotype (HPO terms) | Gene name Transcript number | Variant(s) | Diagnosis (condition, OMIM#, *further detail regarding partial diagnosis** ) | Changes in medication | Improved process of care | Surgical procedures | Palliation |
|---|---|---|---|---|---|---|---|---|
| A1131035 | HP:0004383\|Hypoplastic left heart HP:0000252\|Microcephaly HP:0000175\|Cleft palate HP:0000085\|Horseshoe kidney HP:0006585\|Congenital pseudoarthrosis of the clavicle HP:0001374\|Congenital hip dislocation HP:0000316\|Hypertelorism HP:0000527\|Long eyelashes HP:0000219\|Thin upper lip vermilion HP:0010751\|Dimple chin HP:0000278\|Retrognathia HP:0000358\|Posteriorly rotated ears | *KMT2D* NM_003482.3 | c.10198C>T, p.(Gln3400*), Pathogenic | Kabuki syndrome 1 MIM#147920 | | | Decision to not pursue further surgery for complex CHD | Yes |
| A1131038 | HP:0031063\|Impaired feeding ability HP:0011471\|Gastrostomy tube feeding in infancy HP:0000252\|Microcephaly HP:0000347\|Micrognathia HP:0000047\|Hypospadias HP:0002092\|Pulmonary arterial hypertension HP:0001655\|Patent foramen ovale HP:0001290\|Generalized hypotonia HP:0001263\|Global developmental delay HP:0008527\|Congenital sensorineural hearing impairment | *SMS* NM_004595.4 | c.674T>C, p.(Val225Ala), Likely pathogenic | Snyder-Robinson type MIM#309583 | | | | |

| **Study ID** | **Phenotype (HPO terms)** | **Gene name Transcript number** | **Variant(s)** | **Diagnosis (condition, OMIM#, *further detail regarding partial diagnosis** )** | **Changes in medication** | **Improved process of care** | **Surgical procedures** | **Palliation** |
|---|---|---|---|---|---|---|---|---|
| A1131047 | HP:0012516\|Tetralogy of Fallot with pulmonary atresia HP:0012020\|Right aortic arch HP:0001338\|Partial agenesis of the corpus callosum HP:0001545\|Anteriorly placed anus HP:0001195\|Single umbilical artery | *PIK3CA* NM_006218.2 | c.263G>A, p.(Arg88Gln), Pathogenic | Megalencephaly-capillary malformation-polymicrogyria syndrome MIM#602501 | | Additional referrals to respiratory, ENT, general paediatrics. | | |
| A1131049 | HP:0002744\|Bilateral cleft lip and palate HP:0000589\|Coloboma HP:0031801\|Vocal cord dysfunction HP:0007209\|Facial paralysis HP:0001643\|Patent ductus arteriosus HP:0010946\|Dilatation of the renal pelvis HP:0007109\|Periventricular cysts | *CHD7* NM_017780.2 | c.7711_7718del, p.(Val2571Serfs*2), Pathogenic | CHARGE syndrome MIM#214800 | | | | |
| A1431023 | HP:0001880\|Eosinophilia HP:0001882\|Leukopenia HP:0001019\|Erythroderma | *RAG2* NM_001243786.1 | c.1247G>T, p.(Trp416Leu), Pathogenic | Omenn syndrome MIM#603554 | | | Informed BMT planning | |
| A1431025 | HP:0001298\|Encephalopathy HP:0001263\|Global developmental delay HP:0000486\|Strabismus HP:0000639\|Nystagmus HP:0001276\|Hypertonia HP:0002490\|Increased CSF lactate HP:0002151\|Increased serum lactate HP:0001875\|Neutropenia | *NDUFS4* NM_002495.3 | c.350+1G>T, p.?, Likely pathogenic | Mitochondrial complex I deficiency, nuclear type 1 MIM#252010 | | | | Yes |

| Study ID | Phenotype (HPO terms) | Gene name Transcript number | Variant(s) | Diagnosis (condition, OMIM#, *further detail regarding partial diagnosis** ) | Changes in medication | Improved process of care | Surgical procedures | Palliation |
|---|---|---|---|---|---|---|---|---|
| A1431029 | HP:0001723\|Restrictive cardiomyopathy HP:0001639\|Hypertrophic cardiomyopathy HP:0001695\|Cardiac arrest HP:0031295\|Left atrial enlargement HP:0030718\|Right atrial enlargement | *TNNT2* NM_000364.2 | c.310C>A, p.(Arg104Ser), Pathogenic | hypertrophic cardiomyopathy MIM#115195 | | | | |
| A1431032 | HP:0002605\|Hepatic necrosis HP:0001928\|Abnormality of coagulation HP:0001951\|Episodic ammonia intoxication | *OTC* NM_000531.5 | c.621C>A, p.(Ser207Arg), Likely pathogenic | Ornithine transcarbamylase deficiency MIM#311250 | | | Avoided liver biopsy, had liver transplant based on result - team worried if it wasn't OTC that liver transplant would not treat condition | |
| A1431033 | HP:0003074\|Hyperglycemia HP:0004388\|Microcolon HP:0001629\|Ventricular septal defect HP:0002151\|Increased serum lactate HP:0001518\|Small for gestational age HP:0000260\|Wide anterior fontanel HP:0001903\|Anemia | *PTF1A* NM_178161.2 | c.571C>A, p.(Pro191Thr), Likely pathogenic | Pancreatic agenesis 2 or pancreatic and cerebellar agenesis MIM#615953;609069 | Started on creon for pancreatic exocrine insufficiency. Insulin changed from temporary to permanent with view to progress to insulin pump | MRI brain to look for potential cerebella changes. Rationalised specialists involved in care. | | |
| A1431037 | HP:0001744\|Splenomegaly HP:0002240\|Hepatomegaly HP:0001873\|Thrombocytopenia HP:0001903\|Anemia HP:0002908\|Conjugated hyperbilirubinemia HP:0002098\|Respiratory distress | *GBA* NM_000157.3 | c.1342G>C, p.(Asp448His), Pathogenic<br>c.887G>A, p.(Arg296Gln), Pathogenic | Gaucher disease MIM#230800 | Enzyme replacement therapy. Ceased treatment for GALD | Investigations and referrals rationalised | | |

| **Study ID** | **Phenotype (HPO terms)** | **Gene name Transcript number** | **Variant(s)** | **Diagnosis (condition, OMIM#, *further detail regarding partial diagnosis** )** | **Changes in medication** | **Improved process of care** | **Surgical procedures** | **Palliation** |
|---|---|---|---|---|---|---|---|---|
| A1431041 | HP:0011664\|Left ventricular noncompaction cardiomyopathy HP:0001030\|Fragile skin | *CUL3* NM_003590.4 | c.1156_1159delinsTC, p.?, Likely pathogenic | CUL3-related neurodevelopmental disorder MIM#619239 | | | | |
| A1431047 | HP:0001639\|Hypertrophic cardiomyopathy HP:0004308\|Ventricular arrhythmia | *FHL1* NM_001159699.1 | c.613_614delGT, p.(Val205Tyrfs*4), Pathogenic | X-linked recessive Emery-Dreifuss muscular dystrophy 6, X-linked MIM#300696 | | Neuromuscular specialist review. Malignant hyperthermia precautions initiated with anaesthetics | Informed decision for cardiac transplant | |
| A1431050 | HP:0002721\|Immunodeficiency HP:0008777\|Abnormal vocal cord morphology HP:0005483\|Abnormal epiglottis morphology HP:0031703\|Abnormal ear morphology HP:0002901\|Hypocalcemia | *CHD7* NM_017780.2 | c.6371T>C, p.(Phe2124Ser), Likely pathogenic | CHARGE syndrome MIM#214800 | Medications rationalised | | | Yes |
| A1431053 | HP:0001250\|Seizures HP:0410263\|Brain imaging abnormality HP:0006965\|Acute necrotizing encephalopathy | *RANBP2* NM_006267.4 | c.1754C>T, p.(Thr585Met), Pathogenic | Acute infection-induced encephalopathy 3 MIM#608033 | Weaned off steroids | | | |
| A1431054 | HP:0001639\|Hypertrophic cardiomyopathy | *MRAS* NM_001085049.2 | c.212A>G, p.(Gln71Arg), Pathogenic | Noonan syndrome 11 MIM#618499 | | Additional referral to oncology and immunology | | |

| **Study ID** | **Phenotype (HPO terms)** | **Gene name Transcript number** | **Variant(s)** | **Diagnosis (condition, OMIM#, *further detail regarding partial diagnosis** )** | **Changes in medication** | **Improved process of care** | **Surgical procedures** | **Palliation** |
|---|---|---|---|---|---|---|---|---|
| A1431056 | HP:0011679\|Tetralogy of Fallot with pulmonary stenosis HP:0005164\|Dysplastic pulmonary valve HP:0001539\|Omphalocele HP:0000913\|Posterior rib fusion HP:0000086\|Ectopic kidney HP:0001338\|Partial agenesis of the corpus callosum HP:0000054\|Micropenis HP:0001545\|Anteriorly placed anus HP:0001727\|Thromboembolic stroke HP:0030242\|Portal vein thrombosis HP:0030283\|Partial absence of the septum pellucidum HP:0004691\|2-3 toe syndactyly | *CHD4* NM_001273.4 | c.3529C>T, p.(Arg1177Cys), Pathogenic | Sifrim-Hitz-Weiss syndrome MIM#617159 | | Additional referral for hearing and visual screen, re-referral to neurology and endocrinology. Suggested repeat gonadotrophin level testing | | |
| A1431057 | HP:0000252\|Microcephaly HP:0001250\|Seizures HP:0410263\|Brain imaging abnormality HP:0002828\|Multiple joint contractures HP:0009879\|Simplified gyral pattern HP:0000368\|Low-set, posteriorly rotated ears HP:0000426\|Prominent nasal bridge HP:0003186\|Inverted nipples HP:0000347\|Micrognathia | *ALG1* NM_019109.4 | c.773C>T, p.(Ser258Leu), Pathogenic<br>c.1187+3A>G, p.?, Pathogenic | Type Ik congenital disorder of glycosylation MIM#608540 | | Additional surveillance ordered, including coagulation, echocardiogram, abdominal ultrasound | | Yes |
| A1431058 | HP:0001644\|Dilated cardiomyopathy HP:0030149\|Cardiogenic shock | *BAG3* NM_004281.3 | c.367C>T, p.(Arg123*), Pathogenic | Dilated cardiomyopathy 1HH MIM#613881 | | | Informed decision for cardiac transplant | |

| Study ID | Phenotype (HPO terms) | Gene name Transcript number | Variant(s) | Diagnosis (condition, OMIM#, *further detail regarding partial diagnosis** ) | Changes in medication | Improved process of care | Surgical procedures | Palliation |
|---|---|---|---|---|---|---|---|---|
| A1431059 | HP:0001252 \| Muscular hypotonia HP:0002747 \| Respiratory insufficiency due to muscle weakness HP:0003236 \| Elevated serum creatine kinase HP:0001324 \| Muscle weakness HP:0001371 \| Flexion contracture | *LAMA2* NM_000426.3 | c.442delC, p.(Arg148Glyfs*24), Pathogenic<br>c.1207-1G>A, p.?, Likely pathogenic | Congenital merosin deficient muscular dystrophy MIM#607855 | Prednisolone started | Additional referral to neurogenetics. Investigations and referrals rationalised | | |
| A1431060 | HP:0200134 \| Epileptic encephalopathy HP:0010952 \| Mild fetal ventriculomegaly HP:0032144 \| Coffee ground vomitus HP:0005301 \| Persistent left superior vena cava | *ALDH7A1* NM_001182.4 | c.1061A>C, p.(Tyr354Ser), Pathogenic<br>c.1468delG, p.(Ala490Leufs*28), Pathogenic | Epilepsy, pyridoxine-dependent MIM#266100 | Rationalised AED medications. | Avoided extensive metabolic investigations including lumbar puncture | | |
| A1531005 | HP:0005671 \| Bilateral intracranial calcifications HP:0001873 \| Thrombocytopenia HP:0001511 \| Intrauterine growth retardation HP:0002514 \| Cerebral calcification | *SAMHD1* NM_015474.3 | c.968T>C, p.(Leu323Pro), Likely pathogenic | Aicardi-Goutières syndrome 5 MIM#612952 | | Avoided investigations including lumbar puncture | | Yes |
| A1531006 | HP:0001518 \| Small for gestational age HP:0002202 \| Pleural effusion HP:0012020 \| Right aortic arch HP:0001873 \| Thrombocytopenia HP:0002901 \| Hypocalcemia HP:0030283 \| Partial absence of the septum pellucidum HP:0006849 \| Hypodysplasia of the corpus callosum | *PTPN11* NM_002834.3 | c.1507G>A, p.(Gly503Arg), Pathogenic | Noonan syndrome MIM#163950 | | Additional referral to general paediatrics | | |

| Study ID | Phenotype (HPO terms) | Gene name Transcript number | Variant(s) | Diagnosis (condition, OMIM#, *further detail regarding partial diagnosis** ) | Changes in medication | Improved process of care | Surgical procedures | Palliation |
|---|---|---|---|---|---|---|---|---|
| A2131010 | HP:0200134\|Epileptic encephalopathy HP:0032407\|Bilateral perisylvian polymicrogyria HP:0011398\|Central hypotonia HP:0000316\|Hypertelorism HP:0000455\|Broad nasal tip | *SPTAN1* NM_001130438.2 | c.6619_6621del, p.(Glu2207del), Pathogenic | Developmental and epileptic encephalopathy 5 MIM#613477 | Influenced choice of AED. | | | |
| A2231002 | HP:0006846\|Acute encephalopathy HP:0001250\|Seizures HP:0011398\|Central hypotonia HP:0001048\|Cavernous hemangioma | *AMT* NM_000481.3 | c.1074_1076del, p.(Lys358del), Likely pathogenic<br>c.14dupT, p.(Ser6Lysfs*22), Pathogenic | Glycine encephalopathy MIM#605899 | | | | Yes |
| A2331003 | HP:0001789\|Hydrops fetalis HP:0000062\|Ambiguous genitalia HP:0004969\|Peripheral pulmonary artery stenosis | *PTPN11* NM_002834.3 | c.1505C>T, p.(Ser502Leu), Pathogenic | Noonan syndrome 1 MIM#163950 | | | | |
| A2331004 | HP:0004902\|Congenital lactic acidosis HP:0001290\|Generalized hypotonia HP:0012650\|Perisylvian polymicrogyria HP:0001928\|Abnormality of coagulation HP:0004305\|Involuntary movements | *LRPPRC* NM_133259.3<br>*LRPPRC* NC_000002.12 | c.2882T>C, p.(Leu961Pro), Likely pathogenic<br>g.43986415_44009956del, p.?, Pathogenic | Leigh syndrome, French-Canadian type MIM#220111 | | | | Yes |

| **Study ID** | **Phenotype (HPO terms)** | **Gene name Transcript number** | **Variant(s)** | **Diagnosis (condition, OMIM#, *further detail regarding partial diagnosis** )** | **Changes in medication** | **Improved process of care** | **Surgical procedures** | **Palliation** |
|---|---|---|---|---|---|---|---|---|
| A2831006 | HP:0001511\|Intrauterine growth retardation HP:0000347\|Micrognathia HP:0000369\|Low-set ears HP:0006610\|Wide intermamillary distance HP:0000954\|Single transverse palmar crease HP:0001838\|Rocker bottom foot | *DYRK1A* NM_001347721.1 | c.638-9_638-5delCTCTT, p.?, Pathogenic | DYRK1A-related intellectual disability syndrome, MONDO: 0013578 MIM#614104 | | | | |
| A2831008 | HP:0001541\|Ascites HP:0002098\|Respiratory distress HP:0001903\|Anemia HP:0001873\|Thrombocytopenia HP:0001433\|Hepatosplenomegaly HP:0001888\|Lymphopenia | *LIPA* NM_000235.3 | c.111+1G>A, p.?, Pathogenic<br>c.1055_1057delACG, p.(Asp352del), Likely pathogenic | Wolman disease MIM#278000 | Commenced enzyme replacement therapy for Lysosomal acid Lipase deficiency (Wolman disease) | | | |
| A3031007 | HP:0011398\|Central hypotonia HP:0000508\|Ptosis HP:0002033\|Poor suck | *UGP2* NM_001001521.1 | c.1A>G, p.(Met1?), Pathogenic | Developmental and epileptic encephalopathy 83 MIM#618744 | | Investigations rationalised, avoided muscle biopsy. Additional referral to neurology | | Yes |
| A3031008 | HP:0002092\|Pulmonary arterial hypertension HP:0001622\|Premature birth HP:0030918\|Low 1-minute APGAR score HP:0030919\|Low 5-minute APGAR score HP:0004887\|Respiratory failure requiring assisted ventilation HP:0002686\|Prenatal maternal abnormality | *FOXF1* NM_001451.2 | c.21delG, p.(Lys7Asnfs*63), Pathogenic | Alveolar capillary dysplasia with misalignment of pulmonary veins MIM#265380 | | | | Yes |

| **Study ID** | **Phenotype (HPO terms)** | **Gene name Transcript number** | **Variant(s)** | **Diagnosis (condition, OMIM#, *further detail regarding partial diagnosis** )** | **Changes in medication** | **Improved process of care** | **Surgical procedures** | **Palliation** |
|---|---|---|---|---|---|---|---|---|
| A3031010 | HP:0001789 \| Hydrops fetalis HP:0000369 \| Low-set ears HP:0000358 \| Posteriorly rotated ears HP:0000337 \| Broad forehead HP:0002202 \| Pleural effusion HP:0001541 \| Ascites | *RAF1* NM_001354689.1 | c.781C>T, p.(Pro261Ser), Pathogenic | Noonan syndrome MIM#611553 | | Additional referral to cardiology | | |
| A3031011 | HP:0001930 \| Nonspherocytic hemolytic anemia HP:0001789 \| Hydrops fetalis | *SLC4A1* NM_000342.3 | c.1199_1225del, p.(Ala400_Ala408del), Pathogenic | Ovalocytosis, SA type MIM#166900 | | | | |

| Study ID | Phenotype (HPO terms) | Gene name Transcript number | Variant(s) | Diagnosis (condition, OMIM#, *further detail regarding partial diagnosis** ) | Changes in medication | Improved process of care | Surgical procedures | Palliation |
|---|---|---|---|---|---|---|---|---|
| A3031012 | HP:0200128\|Biventricular hypertrophy HP:0001650\|Aortic valve stenosis HP:0000954\|Single transverse palmar crease HP:0000028\|Cryptorchidism | *RIT1* NM_006912.5 | c.270G>A, p.(Met90Ile), Pathogenic | Noonan syndrome 8 MIM#615355 | | Additional referral to cardiology | | |
| A3031013 | HP:0001298\|Encephalopathy HP:0410263\|Brain imaging abnormality HP:0003128\|Lactic acidosis HP:0001290\|Generalized hypotonia HP:0001250\|Seizures | *PDHA1* NM_000284.3 | c.483C>T, p.(Tyr161=), Pathogenic | Pyruvate dehydrogenase E1-alpha deficiency MIM#312170 | Commenced on high dose thiamine + ketogenic diet | | | Yes |
| A3231006 | HP:0008064\|Ichthyosis | *TGM1* NM_000359.2 | c.877-2A>G, p.?, Pathogenic<br>c.790C>T, p.(Arg264Trp), Pathogenic | Congenital ichthyosis 1 MIM#242300 | | Rationalised investigations. Avoided skin biopsy | | |
| A3331001 | HP:0001298\|Encephalopathy HP:0003201\|Rhabdomyolysis HP:0005184\|Prolonged QTc interval HP:0000821\|Hypothyroidism HP:0001263\|Global developmental delay HP:0001250\|Seizures HP:0001943\|Hypoglycemia | *TANGO2* NM_152906.6<br>*TANGO2* NC_000022.11 | c.256C>T, p.(Arg86*), Pathogenic<br>g.20041466_20075200del, p.?, Pathogenic | Metabolic encephalomyopathic crises, recurrent, with rhabdomyolysis, cardiac arrhythmias, and neurodegeneration MIM#616878 | Coenzyme Q started. Sick day management plan initiated. | | | |

| **Study ID** | **Phenotype (HPO terms)** | **Gene name Transcript number** | **Variant(s)** | **Diagnosis (condition, OMIM#, *further detail regarding partial diagnosis** )** | **Changes in medication** | **Improved process of care** | **Surgical procedures** | **Palliation** |
|---|---|---|---|---|---|---|---|---|
| **Partial diagnoses** | | | | | | | | |
| A0131112 | HP:0006530\|Interstitial pulmonary abnormality<br>HP:0002092\|Pulmonary arterial hypertension | *ADAMTS13*<br>NM_139025.4 | c.1169G>A, p.(Trp390*), Pathogenic<br><br>c.1705+1G>A, p.?, Likely pathogenic | Thrombotic thrombocytopenic purpura, hereditary MIM#274150<br>*Severity of clinical presentation not fully explained, required ECMO and subsequently died.* | | Additional referral to haematology, ADAMTS13 level investigated | | |
| A0131133 | HP:0001250\|Seizures<br>HP:0003201\|Rhabdomyolysis<br>HP:0000750\|Delayed speech and language development<br>HP:0002376\|Developmental regression<br>HP:0001298\|Encephalopathy<br>HP:0002910\|Elevated hepatic transaminase | *DMD*<br>NM_004006.2 | c.650-39498A>G, p.(Asp217Alafs*2), Pathogenic | Dystrophinopathy<br>*Did not explain rhabdomyolysis.* | Medications rationalised | Referral to neurology team as primary care team. Investigations and referrals rationalised. | | |

| Study ID | Phenotype (HPO terms) | Gene name Transcript number | Variant(s) | Diagnosis (condition, OMIM#, *further detail regarding partial diagnosis** ) | Changes in medication | Improved process of care | Surgical procedures | Palliation |
|---|---|---|---|---|---|---|---|---|
| A0331025 | HP:0002958\|Immune dysregulation HP:0012819\|Myocarditis HP:0003765\|Psoriasiform dermatitis HP:0012265\|Ciliary dyskinesia HP:0001696\|Situs inversus totalis HP:0002665\|Lymphoma HP:0002861\|Melanoma HP:0002671\|Basal cell carcinoma HP:0002860\|Squamous cell carcinoma | *DNAAF4* NM_130810.3 | c.784_893del, p.(Trp262Glnfs*16), Pathogenic | Primary ciliary dyskinesia, 25 MIM#615482 *Did not explain the reason for acute deterioration, myocarditis.* | | | | |
| A0431024 | HP:0001644\|Dilated cardiomyopathy HP:0005110\|Atrial fibrillation HP:0002342\|Intellectual disability, moderate HP:0011343\|Moderate global developmental delay | *RYR2* NM_001035.2 | c.14174A>G, p.(Tyr4725Cys), Pathogenic | Catecholaminergic polymorphic ventricular tachycardia (CPVT) 1 MIM#604772 *Did not explain intellectual disability.* | | | Insertion of AICD | |
| A1531007 | HP:0003026\|Short long bone HP:0007957\|Corneal opacity HP:0000023\|Inguinal hernia HP:0000811\|Abnormal external genitalia HP:0000271\|Abnormality of the face | *PROKR2* NM_144773.3 | c.58delC, p.(His20Metfs*24), Likely pathogenic | Hypogonadotropic hypogonadism 3 with or without anosmia MONDO:0009482 *Did not explain IUGR, corneal clouding and dysmorphic features.* | | Additional referral for endocrine review | | |

| Study ID | Phenotype (HPO terms) | Gene name<br>Transcript number | Variant(s) | Diagnosis (condition, OMIM#, ***further detail regarding partial diagnosis**** ) | Changes in medication | Improved process of care | Surgical procedures | Palliation |
|---|---|---|---|---|---|---|---|---|
| A2331002 | HP:0001250\|Seizures<br>HP:0006543\|Cardiorespiratory arrest | *DMD*<br>NM_004006.2 | c.7098+2804_7542+11514del, p.(Glu2367_Lys2514del), Pathogenic | X-linked muscle disorders referred to as dystrophinopathies in males, including Becker muscular dystrophy MIM#310200<br>*Did not explain seizures.* | | | | |

| Study ID | Phenotype (HPO terms) | Gene name Transcript number | Variant(s) | Diagnosis (condition, OMIM#, *further detail regarding partial diagnosis** ) | Changes in medication | Improved process of care | Surgical procedures | Palliation |
|---|---|---|---|---|---|---|---|---|
| **Dual diagnosis** | | | | | | | | |
| A0131156 | HP:0002133\|Status epilepticus HP:0001274\|Agenesis of corpus callosum HP:0001629\|Ventricular septal defect HP:0000175\|Cleft palate HP:0100542\|Abnormal localization of kidney HP:0002342\|Intellectual disability, moderate HP:0000252\|Microcephaly | *DOCK7* NM_001271999.1 | c.4243C>T, p.(Arg1415*), Pathogenic | Developmental and epileptic encephalopathy 23 MIM#615859 | | | | |
| | | | c.453dupA, p.(Gly152Argfs*8), Pathogenic | | | | | |
| | | *ARHGAP35* NM_004491.4 | c.2814dupT, p.(Lys939*), Pathogenic | neurodevelopmental disorder, ARHGAP35-related MONDO:0700092 | | | | |
| A0331057 | HP:0001263\|Global developmental delay HP:0002376\|Developmental regression HP:0001250\|Seizures HP:0001252\|Muscular hypotonia HP:0001298\|Encephalopathy | *COL4A1* NM_001845.5 | c.1702G>T, p.(Gly568Cys), Likely pathogenic | COL4A1-related cerebral small vessel disease MONDO:0018788 | | | | |
| | | *KCNT1* NM_020822.2 | See supp. table 3 | See supp. table 3 | | | | |

| **Study ID** | **Phenotype (HPO terms)** | **Gene name Transcript number** | **Variant(s)** | **Diagnosis (condition, OMIM#, *further detail regarding partial diagnosis** )** | **Changes in medication** | **Improved process of care** | **Surgical procedures** | **Palliation** |
|---|---|---|---|---|---|---|---|---|
| A0431020 | HP:0008677\|Congenital nephrotic syndrome HP:0011387\|Enlarged vestibular aqueduct HP:0008619\|Bilateral sensorineural hearing impairment HP:0025492\|Microcoria HP:0004713\|Reversible renal failure | *LAMB2* NM_002292.3 | c.2494C>T, p.(Gln832*), Pathogenic | Pierson syndrome MIM#609049 | | Thyroid surveillance | Avoided renal biopsy | |
| | | *SLC26A4* NM_000441.1 | c.1489G>A, p.(Gly497Ser), Pathogenic | Deafness with enlarged vestibular aqueduct and Pendred syndrome MIM#600791;274600 | | | | |
| A1131042 | HP:0001669\|Transposition of the great arteries HP:0001629\|Ventricular septal defect HP:0005148\|Pulmonary valve defects HP:0000776\|Congenital diaphragmatic hernia HP:0000047\|Hypospadias HP:0008064\|Ichthyosis HP:0000239\|Large fontanelles HP:0000175\|Cleft palate HP:0006530\|Interstitial pulmonary abnormality HP:0001508\|Failure to thrive HP:0003334\|Elevated circulating catecholamine level HP:0005328\|Progeroid facial appearance HP:0001507\|Growth abnormality | *FLG* NM_002016.1 | c.2929C>T, p.(Gln977*), Pathogenic | Ichthyosis vulgaris MIM#146700 | | | | |
| | | *GNB1* NM_002074.4 | c.230G>A, p.(Gly77Asp), Pathogenic | Intellectual disability 42 MIM#616973 | | | | |

| **Study ID** | **Phenotype (HPO terms)** | **Gene name Transcript number** | **Variant(s)** | **Diagnosis (condition, OMIM#, *further detail regarding partial diagnosis** )** | **Changes in medication** | **Improved process of care** | **Surgical procedures** | **Palliation** |
|---|---|---|---|---|---|---|---|---|
| A0131089 | HP:0200134\|Epileptic encephalopathy | *KCNQ2* NM_172107.3 | c.901G>A, p.(Gly301Ser), Pathogenic | Autosomal dominant febrile seizures, familial, 8 MIM#607681 | Commenced on oxcarbazepine and phenytoin. Vitamins and levetiracetam ceased. | | | |
| | | *GABRG2* NM_198903.2 | See supp. table 2 | See supp. table 2 | | | | |

* further detail regarding partial diagnoses inlcuded in *italics* for partial diagnoses only

**Supplementary Table 2:** Patients receiving a diagnosis (including partial or dual diagnoses) obtained through extended analysis and functional validation, including presenting features, molecular diagnosis and clinician-reported clinical utility.

| **Study ID** | **Phenotype (HPO terms)** | **Gene name Transcript number** | **Variant(s)** | **Diagnosis (condition, OMIM#, *further detail regarding partial diagnosis** )** | **Changes in medication** | **Improved process of care** | **Surgical procedures** | **Palliation** |
|---|---|---|---|---|---|---|---|---|
| A0131063 | HP:0006554\|Acute hepatic failure HP:0001928\|Abnormality of coagulation HP:0001943\|Hypoglycemia | *NBAS* NM_015909.3 | c.2951T>G, p.(Ile984Ser), Pathogenic | Infantile liver failure syndrome 2 MIM#616483 | | | | |
| | | | c.406A>G, p.(Arg136Gly), Likely pathogenic | | | | | |
| A0131122 | HP:0001511\|Intrauterine growth retardation HP:0008897\|Postnatal growth retardation HP:0001888\|Lymphopenia HP:0002028\|Chronic diarrhea HP:0001955\|Unexplained fevers HP:0001903\|Anemia HP:0008905\|Rhizomelia HP:0012385\|Camptodactyly HP:0007598\|Bilateral single transverse palmar creases HP:0100512\|Low levels of vitamin D | *RMRP* NR_003051.3 | n.-5_-4insAACTACTCTGTGAAGCTGA, p.?, Pathogenic | Cartilage-hair hypoplasia MIM#250250 | | | | |
| | | | n.93_94insA, p.?, Pathogenic | | | | | |

| **Study ID** | **Phenotype (HPO terms)** | **Gene name Transcript number** | **Variant(s)** | **Diagnosis (condition, OMIM#, *further detail regarding partial diagnosis** )** | **Changes in medication** | **Improved process of care** | **Surgical procedures** | **Palliation** |
|---|---|---|---|---|---|---|---|---|
| A0131129 | HP:0000369\|Low-set ears HP:0000347\|Micrognathia HP:0000278\|Retrognathia HP:0000316\|Hypertelorism HP:0010823\|Ridged cranial sutures HP:0011220\|Prominent forehead HP:0025386\|Bitemporal hollowing HP:0000767\|Pectus excavatum HP:0009017\|Loss of gluteal subcutaneous adipose tissue HP:0031139\|Frog-leg posture HP:0011398\|Central hypotonia HP:0012389\|Appendicular hypotonia HP:0001998\|Neonatal hypoglycemia | *DMPK* NM_004409.5 | Allele 1: 5 repeats Allele 2: ~900 repeats | Myotonic dystrophy 1 MIM#160900 | | | | |
| A0431022 | HP:0012751\|Abnormal basal ganglia MRI signal intensity HP:0002151\|Increased serum lactate HP:0002490\|Increased CSF lactate HP:0009830\|Peripheral neuropathy HP:0001298\|Encephalopathy HP:0009053\|Distal lower limb muscle weakness HP:0003236\|Elevated serum creatine kinase | *PDHA1* NM_000284.3 | c.1045G>A, p.(Ala349Thr), Likely pathogenic | pyruvate dehydrogenase E1-alpha deficiency MIM#312170 | | | | |

| Study ID | Phenotype (HPO terms) | Gene name Transcript number | Variant(s) | Diagnosis (condition, OMIM#, *further detail regarding partial diagnosis** ) | Changes in medication | Improved process of care | Surgical procedures | Palliation |
|---|---|---|---|---|---|---|---|---|
| A0731002 | HP:0008846\|Severe intrauterine growth retardation HP:0001838\|Rocker bottom foot HP:0006989\|Dysplastic corpus callosum HP:0010508\|Metatarsus valgus HP:0002908\|Conjugated hyperbilirubinemia HP:0007598\|Bilateral single transverse palmar creases HP:0004991\|Rhizomelic arm shortening HP:0000307\|Pointed chin | *RNU4ATAC* NR_023343.1 | n.18G>A, p.?, Likely pathogenic<br>n.50G>A, p.?, Likely pathogenic | Roifman syndrome MIM#616651 | | | | |
| A0731004 | HP:0000278\|Retrognathia HP:0001770\|Toe syndactyly HP:0001776\|Bilateral talipes equinovarus HP:0000175\|Cleft palate HP:0001188\|Hand clenching | *GABRB3* NM_000814.5 | c.929T>G, p.(Leu310Arg), Pathogenic | developmental and epileptic encephalopathy 43 MIM#617113 | | | | |
| A0831013 | HP:0003198\|Myopathy HP:0002878\|Respiratory failure HP:0000028\|Cryptorchidism | *MTM1* NM_000252.2 | c.-76_-11del, p.?, Pathogenic | Myotubular myopathy MIM#310400 | | | | |
| A0831018 | HP:0001876\|Pancytopenia HP:0003281\|Increased serum ferritin HP:0001945\|Fever HP:0001928\|Abnormality of coagulation HP:0011343\|Moderate global developmental delay | *PNP* NM_000270.3 | c.97T>C, p.(Ser33Pro), Likely pathogenic | immunodeficiency due to purine nucleoside phosphorylase deficiency MIM#613179 | | | Informed BMT | |
| A0831021 | HP:0004900\|Severe lactic acidosis HP:0011398\|Central hypotonia | *GTPBP3* NM_032620.4 | c.521G>C, p.(Arg174Pro), Likely pathogenic | Combined Oxidative Phosphorylation Deficiency 23 MIM#608536 | | Lumbar puncture not required | | Yes |

| Study ID | Phenotype (HPO terms) | Gene name Transcript number | Variant(s) | Diagnosis (condition, OMIM#, further detail regarding partial diagnosis* ) | Changes in medication | Improved process of care | Surgical procedures | Palliation |
|---|---|---|---|---|---|---|---|---|
| A1031002 | HP:0002376 \| Developmental regression HP:0001272 \| Cerebellar atrophy HP:0002061 \| Lower limb spasticity HP:0000750 \| Delayed speech and language development | *GLB1* NM_000404.3 | c.733+6T>C, p.?, Pathogenic | GM1-gangliosidosis MONDO:0018149 | Determined access to a trial | | | |
| A1431031 | HP:0002151 \| Increased serum lactate HP:0001942 \| Metabolic acidosis HP:0002033 \| Poor suck | *ACAD9* NM_014049.4 | c.1636G>A, p.(Val546Met), Pathogenic | Mitochondrial complex I deficiency, nuclear type 20 MIM#611126 | Mitochondrial enzymes started | Avoided liver and muscle biopsy | | |
| | | | c.1376_1381delins CCT, p.(Lys459_Ser461delinsThrCys), Likely pathogenic | | | | | |
| A2131008 | HP:0001873 \| Thrombocytopenia HP:0001928 \| Abnormality of coagulation HP:0001903 \| Anemia HP:0001643 \| Patent ductus arteriosus HP:0001644 \| Dilated cardiomyopathy HP:0000337 \| Broad forehead | *TBX19* NM_005149.2 | c.666-2A>T, p.?, Likely pathogenic | Adrenocorticotropic hormone deficiency MIM#201400 | Commenced hydrocortisone | | | |
| A3231005 | HP:0002202 \| Pleural effusion HP:0001922 \| Vacuolated lymphocytes HP:0002098 \| Respiratory distress HP:0000969 \| Edema HP:0000316 \| Hypertelorism | *GLB1* NM_000404.3 | c.130G>T, p.(Asp44Tyr), Likely pathogenic | GM1-gangliosidosis, type I MIM#230500 | | | | Yes |

| Study ID | Phenotype (HPO terms) | Gene name Transcript number | Variant(s) | Diagnosis (condition, OMIM#, *further detail regarding partial diagnosis** ) | Changes in medication | Improved process of care | Surgical procedures | Palliation |
|---|---|---|---|---|---|---|---|---|
| A3331002 | HP:0011398 \| Central hypotonia<br>HP:0001250 \| Seizures<br>HP:0012650 \| Perisylvian polymicrogyria<br>HP:0030001 \| Lagopthalmos<br>HP:0011648 \| Patent ductus arteriosus after birth at term | *HSD17B4*<br>NM_000414.3 | c.1132G>A, p.(Gly378Arg), Likely pathogenic | D-bifunctional protein deficiency MIM#261515 | | | | Yes |
| A0131089 | HP:0200134 \| Epileptic encephalopathy | *GABRG2*<br>NM_198903.2 | c.373C>T, p.(Arg125Cys), Pathogenic | febrile seizures, familial, 8 MIM#607681 | | | | |
| A1131048 | HP:0001250 \| Seizures<br>HP:0006846 \| Acute encephalopathy<br>HP:0004305 \| Involuntary movements<br>HP:0001266 \| Choreoathetosis<br>HP:0000639 \| Nystagmus<br>HP:0410263 \| Brain imaging abnormality | *NUP214*<br>NM_005085.3 | c.112C>T, p.(Arg38Cys), Pathogenic<br>c.929T>C, p.(Ile310Thr), Likely pathogenic | Susceptibility to infection-induced acute encephalopathy MIM#618426 | | | | |
| A0131084 | HP:0011343 \| Moderate global developmental delay<br>HP:0011398 \| Central hypotonia<br>HP:0002509 \| Limb hypertonia<br>HP:0200134 \| Epileptic encephalopathy<br>HP:0012450 \| Chronic constipation | *MECP2*<br>NC_000023.11 | g.154031853_154031868insSVA*, Likely pathogenic | Intellectual developmental disorder, X-linked, syndromic 13 MIM#300055 | | | | |

| Study ID | Phenotype (HPO terms) | Gene name Transcript number | Variant(s) | Diagnosis (condition, OMIM#, ***further detail regarding partial diagnosis**** ) | Changes in medication | Improved process of care | Surgical procedures | Palliation |
|---|---|---|---|---|---|---|---|---|
| **Partial diagnoses** | | | | | | | | |
| A0131108 | HP:0040168\|Focal seizures, afebril | *FRMD7* NM_194277.2 | c.781C>T, p.(Arg261*), Pathogenic | congenital nystagmus 1 MIM#310700 *Did not explain persistently raised lactate and cardiomyopathy.* | | | | |
| A0131105 | HP:0002151\|Increased serum lactate HP:0001714\|Ventricular hypertrophy HP:0001508\|Failure to thrive HP:0001254\|Lethargy HP:0011968\|Feeding difficulties HP:0008504\|Moderate sensorineural hearing impairment | *POLR1A* NM_015425.5 | c.190delT, p.(Cys64Alafs*42), Likely pathogenic | Acrofacial dysostosis,Cincinnati type MIM#616462 *Did not explain seizures.* | | | | |

* further detail regarding partial diagnoses inlcuded in italics for partial diagnoses only

^ dual diagnosis. Refer to Supplementary Table 1

**Supplementary Table 3:** Diagnoses missed by WGS analysis, including presenting features, molecular diagnosis and additional tests leading to diagnosis.

| **Study ID** | **Phenotype (HPO terms)** | **Gene name Transcript number** | **Variant(s)** | **Diagnosis (condition, OMIM#)** | **Additional tests leading to diagnosis** |
|---|---|---|---|---|---|
| A1131016 | HP:0011398\|Central hypotonia HP:0002104\|Apnea HP:0009916\|Anisocoria HP:0001250\|Seizures | *PHOX2B* | PolyA tract expansion, Pathogenic | Central hypoventilation syndrome, congenital, 1 MIM#209880 | PolyA tract expansion testing due to high level of clinical suspicion |
| A3031009 | HP:0001319\|Neonatal hypotonia HP:0001290\|Generalized hypotonia HP:0001315\|Reduced tendon reflexes HP:0000278\|Retrognathia HP:0001776\|Bilateral talipes equinovarus HP:0006380\|Knee flexion contracture HP:0003273\|Hip contracture HP:0002374\|Diminished movement HP:0030276\|Small scrotum HP:0008689\|Bilateral cryptorchidism HP:0002033\|Poor suck HP:0003236\|Elevated serum creatine kinase HP:0002151\|Increased serum lactate | UPD15 | UPD15, Pathogenic | Prader-Willi syndrome MIM#176270 | SNP-based chromosomal microarray initiated prior to ultra-rapid WGS. Result available after ultra-rapid WGS |
| A0331057^ | HP:0001263\|Global developmental delay HP:0002376\|Developmental regression HP:0001250\|Seizures HP:0001252\|Muscular hypotonia HP:0001298\|Encephalopathy | *KCNT1* NM_020822.2 | c.1420C>T, p.Arg474Cys, Pathogenic, Mosaicism level 16% | Epileptic encephalopathy, early infantile 14 MIM#614959 | Epilepsy gene panel initiated prior to ultra-rapid WGS. Result available after ultra-rapid WGS |
| A0131125 | HP:0011002\|Osteopetrosis HP:0004979\|Metaphyseal sclerosis HP:0003025\|Metaphyseal irregularity HP:0000256\|Macrocephaly HP:0410242\|Abnormal IgG level HP:0100327\|Cow milk allergy HP:0000964\|Eczema HP:0001051\|Seborrheic dermatitis HP:0100658\|Cellulitis HP:0002090\|Pneumonia HP:0001507\|Growth abnormality HP:0001744\|Splenomegaly | *IKBKG* NM_001099857.3 | c.1167dup, p.(Glu390Argfs*5), Pathogenic, Mosaicism level not determined | Anhidrotic (hydpohidrotic) ectodermal dysplasia with immune deficiency (EDA-ID) MIM#300291 | Sanger sequencing initiated due to high level of clinical suspicion |
| A3031015 | HP:0001355\|Megalencephaly HP:0002126\|Polymicrogyria HP:0006101\|Finger syndactyly HP:0001770\|Toe syndactyly HP:0000028\|Cryptorchidism | *PIK3CA* NM_006218.2 | c.1132T>C, p.(Cys378Arg), Pathogenic, Mosaicism level in fibroblasts 21% | Megalencephaly-capillary malformation-polymicrogyria syndrome MIM#602501 | Whole genome sequencing of fibroblast sample initiated due to high level of clinical suspicion |

^ dual diagnosis. Refer to Supplementary Table 1

**Supplementary Table 4:** Proteomics data for A1131048 and controls, including log2-transformed protein abundance intensities for all quantified cellular proteins in patient and control fibroblasts. Data was derived from a two-sided Student's T-test. No adjustments were made for multiple comparisons.

| Gene names | UniProt IDs | Protein Group P-value | Protein Group Q-value | Log2 Difference NUP214 vs Controls (n=5) | p<0.05 | -log10 p-value | T-test Test statistic | NPC Localization | Log2 MS2_quantity_NUP214_1 | Log2 MS2_quantity_NUP214_2 | Log2 MS2_quantity_NUP214_3 | Log2 MS2_quantity_Control_1 | Log2 MS2_quantity_Control_2 | Log2 MS2_quantity_Control_3 | Log2 MS2_quantity_Control_4 | Log2 MS2_quantity_Control_5 | Number Peptides for Quantitation NUP214_1 | Number Peptides for Quantitation NUP214_2 | Number Peptides for Quantitation NUP214_3 | Number Peptides for Quantitation Control_1 | Number Peptides for Quantitation Control_2 | Number Peptides for Quantitation Control_3 | Number Peptides for Quantitation Control_4 | Number Peptides for Quantitation Control_5 |
|---|---|---|---|---|---|---|---|---|---|---|---|---|---|---|---|---|---|---|---|---|---|---|---|---|
| NUP58 | Q9BVL2;Q9 | 5.51E-09 | 2.07E-09 | -0.124824 | | 1.20438 | -2.28392 | + | 15.7476 | 15.6448 | 15.7457 | 15.8009 | 15.9433 | 15.7599 | 15.7782 | 15.9054 | 8 | 8 | 8 | 8 | 8 | 8 | 8 | 8 |
| NUP54 | Q7Z3B4;Q7 | 4.57E-12 | 2.83E-12 | -0.0125263 | | 0.102512 | -0.27882 | + | 16.5611 | 16.5755 | 16.5711 | 16.6261 | 16.6588 | 16.4616 | 16.5699 | 16.5924 | 15 | 13 | 14 | 15 | 13 | 12 | 14 | 14 |
| NUP188 | Q5SRE5 | 3.03E-19 | 1.59E-18 | -0.00868797 | | 0.038868 | -0.11211 | + | 15.3671 | 15.3627 | 15.3385 | 15.5163 | 15.4087 | 15.1905 | 15.2781 | 15.4303 | 27 | 26 | 26 | 25 | 15 | 19 | 21 | 23 |
| NUP93 | Q8N1F7 | 3.39E-10 | 1.49E-10 | 0.0850255 | | 0.597883 | 1.26609 | + | 16.5259 | 16.542 | 16.5544 | 16.6333 | 16.4067 | 16.3576 | 16.3843 | 16.4967 | 35 | 36 | 35 | 32 | 28 | 34 | 30 | 34 |
| NUP205 | Q92621 | 4.22E-11 | 2.17E-11 | 0.166752 | + | 1.34154 | 2.51576 | + | 16.3578 | 16.3531 | 16.3672 | 16.3496 | 16.2188 | 16.0664 | 16.1074 | 16.2208 | 47 | 46 | 46 | 47 | 39 | 43 | 40 | 44 |
| NUP35 | Q8NFH5;Q8 | 5.31E-07 | 1.60E-07 | 0.19403 | | 1.23193 | 2.33022 | + | 16.347 | 16.4485 | 16.4104 | 16.3687 | 16.322 | 16.0852 | 16.0716 | 16.1922 | 6 | 6 | 6 | 6 | 4 | 6 | 6 | 6 |
| NUP155 | O75694 | 1.61E-13 | 1.45E-13 | 0.256724 | + | 2.6603 | 5.11624 | + | 16.3784 | 16.3604 | 16.3636 | 16.2359 | 16.0353 | 16.0806 | 16.0477 | 16.1543 | 35 | 35 | 32 | 28 | 28 | 28 | 32 | 28 |
| NUP62 | P37198 | 2.70E-16 | 5.43E-16 | -0.310585 | + | 1.35692 | -2.542 | + | 16.2034 | 16.0257 | 16.3947 | 16.6809 | 16.3146 | 16.4042 | 16.6542 | 16.5388 | 9 | 9 | 10 | 9 | 9 | 10 | 10 | 10 |
| RAE1 | P78406 | 5.77E-11 | 2.90E-11 | 0.1954 | + | 1.33841 | 2.51043 | + | 16.8659 | 16.9189 | 16.8585 | 16.8241 | 16.4772 | 16.7396 | 16.6812 | 16.7063 | 9 | 9 | 9 | 9 | 8 | 9 | 9 | 9 |
| NUP98 | P52948;P5 | 1.20E-16 | 2.79E-16 | 0.207187 | + | 1.9247 | 3.56236 | + | 16.0521 | 16.0124 | 16.0343 | 15.9891 | 15.8293 | 15.7741 | 15.7429 | 15.7934 | 40 | 41 | 40 | 41 | 35 | 38 | 40 | 39 |
| NDC1 | Q9BTX1;Q9 | 4.41E-05 | 1.09E-05 | -0.0599758 | | 0.144217 | -0.37943 | + | 14.3016 | 14.7102 | 14.4791 | 14.7342 | 14.6628 | 14.4084 | 14.2413 | 14.738 | 5 | 4 | 5 | 6 | 2 | 4 | 4 | 5 |
| AAAS | Q9NRG9 | 0.000637 | 0.000135 | 0.0806091 | | 0.531464 | 1.14942 | + | 15.9397 | 15.9781 | 15.911 | 15.9131 | 15.9817 | 15.684 | 15.9133 | 15.8196 | 9 | 8 | 7 | 7 | 6 | 7 | 6 | 7 |
| NUP214 | P35658;P3 | 9.52E-12 | 5.56E-12 | -1.2596 | + | 9.74081 | -84.7607 | + | 14.4143 | 14.4444 | 14.3994 | 15.6898 | 15.6837 | 15.6776 | 15.6474 | 15.6963 | 26 | 23 | 23 | 34 | 30 | 32 | 33 | 30 |
| NUP88 | Q99567 | 2.13E-14 | 2.45E-14 | -0.970154 | + | 6.06802 | -20.5849 | + | 15.1842 | 15.2048 | 15.2056 | 16.2373 | 16.236 | 16.0462 | 16.174 | 16.1482 | 15 | 16 | 16 | 17 | 16 | 16 | 16 | 17 |
| NUP42 | O15504;O1 | 0.012725 | 0.002156 | -0.45933 | | 0.340124 | -0.80579 | + | 12.2746 | 13.4058 | 13.6785 | 12.7003 | 14.3484 | NaN | 14.0252 | 13.2419 | 1 | 1 | 1 | 1 | 2 | NaN | 1 | 1 |
| GLE1 | Q53GS7;Q5 | 8.27E-06 | 2.19E-06 | 0.00680269 | | 0.023267 | 0.068209 | + | 14.2959 | 14.3165 | 14.1088 | 14.3166 | 14.4205 | 14.1222 | 14.0571 | 14.2518 | 6 | 4 | 6 | 5 | 5 | 4 | 3 | 5 |
| RANBP2 | P49792 | 1.12E-21 | 1.30E-20 | 0.179077 | + | 2.92836 | 5.77335 | + | 16.0739 | 16.1095 | 16.0686 | 15.9891 | 15.9064 | 15.8657 | 15.8735 | 15.89 | 81 | 84 | 80 | 79 | 61 | 73 | 72 | 69 |
| NUP50 | Q9UKX7;Q9 | 1.22E-15 | 2.04E-15 | -0.216297 | | 0.627896 | -1.31808 | + | 15.825 | 15.9149 | 15.8773 | 16.3573 | 16.0788 | 15.7546 | 15.8896 | 16.3632 | 9 | 10 | 10 | 9 | 8 | 8 | 9 | 10 |
| NUP153 | P49790 | 1.17E-17 | 3.63E-17 | 0.127825 | | 0.809049 | 1.62531 | + | 15.3985 | 15.3545 | 15.4318 | 15.3737 | 15.1021 | 15.1988 | 15.243 | 15.418 | 24 | 26 | 25 | 27 | 20 | 25 | 26 | 25 |
| TPR | P12270 | 5.90E-14 | 6.00E-14 | 0.234881 | + | 3.05096 | 6.09343 | + | 16.1989 | 16.1857 | 16.1819 | 16.0428 | 15.9506 | 15.889 | 15.8978 | 15.9896 | 80 | 79 | 80 | 75 | 61 | 72 | 68 | 75 |
| AHCTF1 | Q8WYP5;Q | 1.20E-10 | 5.71E-11 | 0.0510897 | | 0.190062 | 0.483973 | + | 14.4502 | 14.4205 | 14.4924 | 14.5142 | 14.4722 | 14.092 | 14.4769 | 14.4612 | 18 | 20 | 20 | 18 | 17 | 14 | 18 | 15 |
| NUP43 | Q8NFH3 | 1.21E-07 | 3.89E-08 | 0.0659728 | | 0.410935 | 0.930103 | + | 17.1149 | 17.203 | 17.1218 | 17.1315 | 17.1444 | 16.9065 | 17.0296 | 17.1911 | 4 | 4 | 4 | 4 | 4 | 4 | 4 | 4 |
| SEC13 | P55735 | 2.16E-05 | 5.50E-06 | 0.104283 | | 0.19354 | 0.49168 | + | 18.2246 | 18.4443 | 18.3627 | 18.4851 | 17.8112 | 18.6119 | 18.3461 | 17.9435 | 10 | 11 | 11 | 12 | 8 | 11 | 11 | 7 |
| NUP133 | Q8WUM0 | 4.09E-15 | 5.82E-15 | 0.178938 | + | 2.17645 | 4.05858 | + | 15.881 | 15.9577 | 15.9309 | 15.8491 | 15.6656 | 15.7661 | 15.7233 | 15.7172 | 33 | 32 | 32 | 31 | 25 | 28 | 31 | 26 |
| NUP107 | P57740 | 1.42E-13 | 1.30E-13 | 0.180313 | + | 1.77793 | 3.28704 | + | 15.8189 | 15.8774 | 15.8872 | 15.8193 | 15.7006 | 15.6647 | 15.6348 | 15.585 | 22 | 23 | 26 | 21 | 21 | 22 | 19 | 19 |
| NUP37 | Q8NFH4 | 5.95E-06 | 1.60E-06 | 0.183995 | | 0.836093 | 1.67053 | + | 16.4076 | 16.4869 | 16.4077 | 16.4722 | 15.9727 | 16.2823 | 16.2132 | 16.3099 | 9 | 9 | 9 | 9 | 6 | 8 | 8 | 7 |
| NUP85 | Q9BW27 | 5.24E-15 | 7.19E-15 | 0.372032 | + | 1.89186 | 3.49993 | + | 15.9597 | 16.04 | 16.0635 | 15.8037 | 15.3904 | 15.8002 | 15.5689 | 15.6819 | 18 | 18 | 18 | 16 | 14 | 12 | 13 | 14 |
| NUP160 | Q12769 | 5.66E-10 | 2.42E-10 | 0.456283 | + | 3.77042 | 8.26564 | + | 16.2546 | 16.247 | 16.2249 | 15.8997 | 15.8664 | 15.7544 | 15.6972 | 15.7116 | 25 | 25 | 25 | 23 | 21 | 23 | 20 | 21 |
| UCHL1 | P09936 | 6.23E-18 | 2.16E-17 | -3.84781 | + | 7.00265 | -29.5591 | | 15.8935 | 15.7365 | 16.1379 | 19.6308 | 19.9776 | 19.7153 | 19.6172 | 19.9114 | 13 | 13 | 11 | 15 | 16 | 16 | 15 | 16 |
| SYNPO2 | Q9UMS6 | 2.85E-10 | 1.27E-10 | -3.84589 | + | 3.97142 | -8.97697 | | 12.4583 | 12.0717 | 12.916 | 15.4013 | 17.1476 | 16.0438 | 16.4237 | 16.6231 | 2 | 2 | 4 | 12 | 15 | 14 | 15 | 15 |
| CALM1;CALM2;CALM | P0DP23;P0 | 3.69E-13 | 3.00E-13 | -3.69484 | + | 2.04481 | -3.79507 | | 15.2858 | 14.5512 | 15.6936 | 16.1831 | 19.4839 | 18.8802 | 20.2604 | 19.551 | 6 | 6 | 6 | 6 | 7 | 8 | 10 | 9 |
| CALML3 | P27482 | 0.000316 | 6.98E-05 | -3.40582 | + | 1.97326 | -3.65561 | | 16.181 | 15.1905 | 16.5549 | 16.8783 | 19.6815 | 19.5519 | 20.7987 | 19.9959 | 3 | 2 | 3 | 4 | 3 | 3 | 3 | 4 |
| CDKN2A | P42771;P4 | 0.00216 | 0.00042 | -3.28865 | + | 1.87541 | -3.74789 | | 11.6133 | NaN | 11.9908 | 14.9064 | 13.7993 | 14.3402 | 15.6184 | 16.7892 | 1 | NaN | 1 | 3 | 3 | 3 | 3 | 4 |
| LAMTOR5 | O43504 | 0.000518 | 0.000111 | -3.11816 | + | 2.28617 | -4.28556 | | 14.5778 | 14.1695 | 14.7374 | 15.491 | 18.1715 | 17.8521 | 18.4074 | 18.1433 | 1 | 1 | 2 | 2 | 2 | 3 | 3 | 3 |
| TMSB4X | P62328 | 4.08E-05 | 1.01E-05 | -3.05248 | + | 2.6809 | -5.1648 | | 14.3197 | 15.0827 | 15.4654 | 16.6089 | 17.7715 | 18.1794 | 18.9977 | 18.4846 | 6 | 5 | 5 | 7 | 7 | 7 | 7 | 7 |
| LIMCH1 | Q9UPQ0;Q | 6.34E-08 | 2.09E-08 | -3.01954 | + | 2.50591 | -4.76214 | | 12.3382 | 12.5149 | 12.6345 | 14.9025 | 14.9556 | 17.3815 | 15.3517 | 14.9858 | 1 | 1 | 3 | 12 | 7 | 24 | 11 | 8 |
| JPT1 | Q9UK76;Q9 | 5.53E-20 | 3.76E-19 | -2.72566 | + | 3.59998 | -7.70099 | | 13.7526 | 13.4662 | 14.0233 | 15.5522 | 16.7588 | 16.3506 | 16.7456 | 16.958 | 3 | 2 | 3 | 4 | 4 | 4 | 3 | 4 |
| ABRACL | Q9P1F3 | 0.000315 | 6.98E-05 | -2.65404 | + | 2.40856 | -4.5472 | | 18.2062 | 16.0689 | 17.6019 | 19.4207 | 20.0185 | 19.2614 | 20.4605 | 20.5707 | 2 | 1 | 2 | 2 | 2 | 2 | 2 | 2 |
| DKK1 | O94907 | 5.42E-08 | 1.80E-08 | -2.61908 | + | 2.82368 | -5.51001 | | 12.466 | 12.4601 | 12.6136 | 15.0882 | 14.3966 | 14.4833 | 16.367 | 15.3265 | 1 | 2 | 2 | 3 | 4 | 4 | 4 | 4 |
| RDH10 | Q8IZV5 | 0.000171 | 3.93E-05 | -2.56402 | | 0.901574 | -1.8382 | | 12.216 | NaN | 12.3242 | 15.6297 | 11.9292 | 16.8178 | 15.5592 | 14.235 | 2 | NaN | 1 | 7 | 2 | 8 | 8 | 5 |
| RPLP2 | P05387 | 6.15E-08 | 2.03E-08 | -2.47632 | + | 2.2814 | -4.27554 | | 17.6273 | 17.0501 | 18.3029 | 18.766 | 20.4636 | 19.8388 | 20.721 | 20.8927 | 7 | 7 | 8 | 9 | 10 | 10 | 10 | 10 |
| RPLP1 | P05386 | 0.001506 | 0.000303 | -2.44488 | + | 2.20202 | -4.11086 | | 17.8763 | 17.6891 | 18.6607 | 19.3424 | 20.0206 | 20.6954 | 20.7007 | 21.8422 | 2 | 2 | 2 | 2 | 2 | 2 | 3 | 3 |
| GLRX | P35754 | 0.000394 | 8.61E-05 | -2.41534 | + | 2.94715 | -5.82157 | | 14.3109 | 13.5345 | 14.3927 | 17.2101 | 16.3528 | 15.5561 | 16.6121 | 16.7422 | 1 | 1 | 1 | 1 | 1 | 1 | 1 | 1 |
| MCOLN1 | Q9GZU1 | 0.001363 | 0.000276 | -2.41011 | + | 2.59385 | -4.96178 | | 12.9633 | 13.3076 | 11.2446 | 15.2291 | 14.8329 | 14.7416 | 15.0792 | 14.6935 | 1 | 2 | 2 | 4 | 1 | 2 | 6 | 4 |
| MTPN | P58546 | 7.91E-24 | 1.88E-22 | -2.39589 | + | 1.79301 | -3.31491 | | 15.9907 | 15.1749 | 15.8215 | 16.0746 | 19.018 | 17.9583 | 18.5568 | 18.6838 | 4 | 5 | 5 | 5 | 4 | 5 | 6 | 6 |
| SULF1 | Q8IWU6 | 1.35E-09 | 5.48E-10 | -2.38118 | | 1.13849 | -2.17364 | | 15.4909 | 12.1796 | 11.8928 | 15.2558 | 15.4572 | 15.8903 | 17.2462 | 13.9953 | 1 | 3 | 1 | 9 | 8 | 10 | 11 | 5 |
| HACD1 | B0YJ81 | 0.00012 | 2.81E-05 | -2.32928 | + | 2.4936 | -4.7346 | | 11.5506 | 13.3308 | 13.2479 | 14.7932 | 15.0402 | 14.5224 | 15.6279 | 15.2116 | 2 | 2 | 2 | 1 | 2 | 1 | 2 | 1 |
| IGF2BP3 | O00425 | 6.54E-10 | 2.77E-10 | -2.32898 | + | 4.49233 | -11.0798 | | 14.4041 | 14.6759 | 14.5756 | 17.189 | 16.9784 | 16.3102 | 16.8752 | 17.0514 | 13 | 10 | 10 | 23 | 21 | 18 | 19 | 21 |
| ITGB3 | P05106 | 8.68E-17 | 2.08E-16 | -2.2171 | + | 3.36039 | -6.96214 | | 13.0604 | 12.67 | 12.9595 | 15.9144 | 14.5026 | 15.1546 | 15.0877 | 14.9092 | 3 | 4 | 3 | 14 | 4 | 11 | 11 | 10 |
| TUBA1C | Q9BQE3 | 6.14E-14 | 6.21E-14 | -2.17458 | + | 1.94915 | -3.60917 | | 20.7053 | 20.788 | 20.2721 | 21.9623 | 23.7324 | 21.4626 | 23.4235 | 23.2345 | 2 | 2 | 2 | 3 | 2 | 3 | 3 | 2 |
| SERPINF2 | P08697;P0 | 0.002173 | 0.000423 | -2.16284 | | 1.24565 | -2.35332 | | 14.6016 | 14.2957 | 14.6399 | 15.9647 | 15.3474 | 16.3657 | 19.3174 | 16.3809 | 1 | 1 | 1 | 2 | 2 | 2 | 2 | 2 |
| TMSB10 | P63313 | 7.76E-17 | 1.88E-16 | -2.13631 | + | 2.91969 | -5.75121 | | 15.4867 | 16.1758 | 16.1098 | 17.5214 | 18.3987 | 17.4189 | 18.2673 | 18.6958 | 4 | 4 | 4 | 4 | 4 | 5 | 4 | 4 |
| SRXN1 | Q9BYN0 | 0.000359 | 7.89E-05 | -2.05273 | + | 2.02153 | -3.74942 | | 11.7585 | 11.89 | 12.1356 | 14.3674 | 12.9145 | 13.0957 | 14.7127 | 14.8134 | 2 | 2 | 2 | 4 | 3 | 3 | 5 | 4 |
| SERPINB2 | P05120 | 2.67E-17 | 7.41E-17 | -2.03929 | + | 1.6548 | -3.06296 | | 15.0029 | 14.8559 | 14.9277 | 17.7181 | 15.7424 | 15.7539 | 17.745 | 17.881 | 10 | 11 | 11 | 17 | 8 | 12 | 17 | 17 |
| LOX | P28300 | 3.76E-11 | 1.95E-11 | -2.02108 | + | 5.78765 | -18.4576 | | 16.485 | 16.5169 | 16.5937 | 18.565 | 18.7316 | 18.3139 | 18.4387 | 18.7154 | 8 | 8 | 8 | 10 | 9 | 10 | 10 | 9 |
| S100A6 | P06703 | 0.000265 | 5.93E-05 | -2.01145 | + | 2.71679 | -5.25011 | | 19.3536 | 18.7277 | 19.9919 | 20.7533 | 21.4343 | 21.1913 | 21.4407 | 22.0264 | 7 | 6 | 7 | 7 | 7 | 7 | 7 | 7 |
| TMEM200A | Q86VY9 | 1.88E-16 | 4.09E-16 | -1.99045 | + | 1.70996 | -3.16261 | | 12.9729 | 13.0926 | 13.3279 | 16.9664 | 14.5636 | 14.948 | 14.6799 | 14.45 | 2 | 4 | 3 | 10 | 3 | 8 | 7 | 7 |
| RNF181 | Q9P0P0 | 7.41E-05 | 1.78E-05 | -1.96068 | + | 2.44029 | -4.61658 | | 14.8598 | 14.0584 | 15.1345 | 15.7209 | 17.0542 | 16.3885 | 16.9318 | 17.1293 | 4 | 2 | 4 | 4 | 4 | 4 | 4 | 4 |
| MYH14 | Q7Z406;Q7 | 0.000176 | 4.03E-05 | -1.94991 | + | 1.54907 | -2.87493 | | 18.5613 | 18.5951 | 16.4108 | 19.5459 | 21.0776 | 19.3779 | 19.5571 | 19.4696 | 2 | 3 | 2 | 3 | 2 | 3 | 3 | 3 |
| DBI | P07108 | 9.49E-09 | 3.46E-09 | -1.92583 | + | 1.66922 | -3.0889 | | 15.8581 | 15.2986 | 16.1811 | 16.0992 | 18.0646 | 17.4837 | 18.1745 | 18.7034 | 7 | 6 | 7 | 7 | 7 | 7 | 8 | 8 |
| SERPINC1 | P01008 | 0.000211 | 4.79E-05 | -1.89701 | + | 3.80784 | -8.39423 | | 16.882 | 16.7802 | 16.893 | 18.7438 | 18.0995 | 18.9817 | 18.925 | 18.9935 | 5 | 5 | 5 | 5 | 5 | 5 | 5 | 5 |
| OCC1 | Q8TAD7 | 7.72E-05 | 1.85E-05 | -1.87317 | + | 4.24959 | -10.0502 | | 15.9434 | 15.8834 | 16.1775 | 17.7497 | 17.8058 | 17.5395 | 17.9518 | 18.3262 | 2 | 2 | 2 | 3 | 2 | 2 | 3 | 3 |
| SLC7A11 | Q9UPY5 | 0.00344 | 0.000632 | -1.85316 | | 1.29571 | -2.56049 | | NaN | 13.2683 | 13.5125 | 15.0915 | 14.0452 | 15.0532 | 16.7313 | 15.2965 | NaN | 1 | 1 | 2 | 2 | 2 | 2 | 2 |
| USP53 | Q70EK8 | 0.000574 | 0.000123 | -1.84179 | + | 2.15041 | -4.00572 | | 13.475 | 13.5613 | 13.5671 | 16.6103 | 14.6281 | 14.854 | 15.3025 | 15.4862 | 2 | 2 | 3 | 7 | 3 | 3 | 2 | 2 |
| COLEC12 | Q5KU26 | 2.43E-05 | 6.13E-06 | -1.83476 | | 1.01861 | -1.9741 | | 15.1424 | 15.085 | 15.0737 | 16.8869 | 16.4214 | 15.8711 | 19.6183 | 15.878 | 9 | 10 | 10 | 16 | 10 | 11 | 2 | 9 |
| APOE | P02649 | 7.84E-07 | 2.31E-07 | -1.80156 | + | 2.63245 | -5.05112 | | 14.5838 | 14.1595 | 14.391 | 16.8551 | 15.9948 | 15.8222 | 15.5157 | 16.7106 | 1 | 2 | 1 | 3 | 2 | 2 | 2 | 3 |
| UQCRH | P07919 | 8.70E-05 | 2.07E-05 | -1.79932 | | 1.15751 | -2.20542 | | 14.5103 | 14.0215 | 15.791 | 15.7321 | 15.792 | 15.5876 | 17.6296 | 18.1266 | 2 | 1 | 2 | 2 | 2 | 2 | 2 | 2 |
| FAM171A1 | Q5VUB5 | 0.007373 | 0.001288 | -1.78625 | + | 3.0918 | -13.8805 | | NaN | 13.4907 | 13.4957 | 15.4719 | 15.1382 | NaN | 15.2283 | NaN | NaN | 2 | 3 | 3 | 2 | NaN | 3 | NaN |
| PDCD1LG2 | Q9BQ51 | 0.000479 | 0.000103 | -1.78238 | + | 2.65438 | -5.10237 | | 13.766 | 13.6516 | 14.0891 | 15.8154 | 16.1208 | 14.6508 | 15.6918 | 15.8109 | 1 | 3 | 1 | 3 | 2 | 3 | 3 | 3 |
| EFS | O43281 | 0.000137 | 3.18E-05 | -1.78131 | + | 1.39772 | -2.99802 | | NaN | 13.6643 | 13.8119 | NaN | 16.6464 | 15.2812 | 14.8012 | 15.3488 | NaN | 2 | 2 | NaN | 1 | 1 | 2 | 1 |
| S100A11 | P31949 | 9.96E-08 | 3.22E-08 | -1.67922 | + | 2.27951 | -4.27158 | | 18.2413 | 17.8499 | 18.5102 | 18.9423 | 19.9761 | 19.7303 | 20.1321 | 20.6173 | 7 | 6 | 7 | 6 | 7 | 7 | 7 | 7 |
| REEP4 | Q9H6H4 | 0.000384 | 8.39E-05 | -1.61929 | + | 1.69558 | -3.13652 | | 13.9559 | 14.7936 | 14.0033 | 15.4111 | 15.5223 | 15.7494 | 17.2772 | 15.3911 | 2 | 3 | 1 | 2 | 3 | 3 | 3 | 4 |
| TNFRSF11B | O00300 | 1.03E-05 | 2.69E-06 | -1.61298 | + | 2.05891 | -3.82286 | | 13.8673 | 14.0497 | 14.0452 | 15.4502 | 15.5153 | 14.7033 | 16.6693 | 15.6638 | 3 | 3 | 2 | 4 | 3 | 2 | 4 | 3 |
| NENF | Q9UMX5 | 1.72E-06 | 4.87E-07 | -1.60434 | + | 2.06663 | -3.83812 | | 15.5499 | 15.0416 | 15.7756 | 15.9353 | 17.1613 | 17.3885 | 17.5782 | 17.237 | 8 | 7 | 9 | 8 | 9 | 9 | 10 | 9 |
| IGFBP6 | P24592 | 0.000379 | 8.29E-05 | -1.60249 | + | 2.67973 | -5.16203 | | 14.0167 | 14.1229 | 13.3185 | 15.7486 | 15.5146 | 14.8981 | 15.8658 | 15.082 | 2 | 2 | 2 | 3 | 3 | 3 | 3 | 3 |
| CA12 | O43570;O4 | 6.78E-08 | 2.22E-08 | -1.58773 | + | 2.77084 | -6.11543 | | 13.5081 | 13.6644 | 13.9421 | 15.87 | 15.034 | 15.2556 | NaN | 15.0108 | 2 | 3 | 1 | 5 | 1 | 1 | NaN | 3 |
| RBPMS | Q93062;Q9 | 2.45E-11 | 1.32E-11 | -1.58431 | + | 2.29298 | -4.29988 | | 15.2119 | 14.7693 | 15.4711 | 16.6544 | 16.8002 | 15.9347 | 16.7615 | 17.5246 | 5 | 4 | 6 | 7 | 7 | 6 | 6 | 8 |

| Gene names | UniProt IDs | Protein Group P-value | Protein Group Q-value | Log2 Difference NUP214 vs Controls (n=5) | p<0.05 | -log10 p-value | T-test Test statistic | NPC Localization | Log2 MS2_quantity_NUP214_1 | Log2 MS2_quantity_NUP214_2 | Log2 MS2_quantity_NUP214_3 | Log2 MS2_quantity_Control_1 | Log2 MS2_quantity_Control_2 | Log2 MS2_quantity_Control_3 | Log2 MS2_quantity_Control_4 | Log2 MS2_quantity_Control_5 | Number Peptides for Quantitation NUP214_1 | Number Peptides for Quantitation NUP214_2 | Number Peptides for Quantitation NUP214_3 | Number Peptides for Quantitation Control_1 | Number Peptides for Quantitation Control_2 | Number Peptides for Quantitation Control_3 | Number Peptides for Quantitation Control_4 | Number Peptides for Quantitation Control_5 |
|---|---|---|---|---|---|---|---|---|---|---|---|---|---|---|---|---|---|---|---|---|---|---|---|---|
| DPYSL4 | O14531 | 0.003992 | 0.000725 | -1.56819 | + | 2.54233 | -9.02918 | | 15.3624 | 15.0092 | 15.1261 | 16.8822 | NaN | 16.586 | NaN | NaN | 1 | 1 | 2 | 3 | NaN | 1 | NaN | NaN |
| C6orf120 | Q7Z4R8 | 6.38E-05 | 1.55E-05 | -1.56073 | + | 2.36953 | -4.46275 | | 15.2565 | 14.8065 | 15.3329 | 15.7599 | 17.1259 | 16.6383 | 16.9627 | 16.9767 | 1 | 1 | 2 | 1 | 2 | 2 | 2 | 2 |
| CD99 | P14209;P1 | 1.90E-05 | 4.85E-06 | -1.54725 | + | 1.82602 | -3.37622 | | 14.3499 | 13.9262 | 14.6886 | 14.7744 | 15.5828 | 16.5639 | 16.02 | 16.4031 | 2 | 1 | 2 | 1 | 1 | 2 | 2 | 3 |
| SERPINE1 | P05121 | 1.70E-07 | 5.37E-08 | -1.52261 | + | 2.8767 | -5.64229 | | 15.8502 | 15.9312 | 15.8011 | 18.049 | 17.0871 | 16.8671 | 17.4913 | 17.4228 | 16 | 15 | 15 | 17 | 13 | 16 | 17 | 17 |
| SELENOS | Q9BQE4 | 0.000192 | 4.36E-05 | -1.52206 | + | 2.411 | -4.55251 | | 12.0968 | 12.5785 | 12.9269 | 14.3605 | 13.8727 | 13.4576 | 14.6895 | 13.9004 | 2 | 2 | 2 | 4 | 4 | 3 | 4 | 3 |
| CPNE1 | Q99829 | 4.82E-09 | 1.82E-09 | -1.51507 | + | 2.9517 | -5.83329 | | 17.6936 | 17.7128 | 17.6446 | 19.5033 | 19.1782 | 19.5548 | 19.286 | 18.4715 | 15 | 15 | 14 | 14 | 15 | 14 | 14 | 14 |
| RND3 | P61587 | 1.68E-08 | 5.95E-09 | -1.50359 | + | 3.17509 | -6.43105 | | 15.2413 | 15.3146 | 15.2755 | 16.9873 | 16.8871 | 16.1154 | 17.1206 | 16.7932 | 8 | 7 | 7 | 9 | 9 | 8 | 9 | 9 |
| APBB1IP | Q7Z5R6 | 1.23E-13 | 1.14E-13 | -1.50278 | + | 2.4433 | -4.62319 | | 14.2166 | 14.0651 | 14.2425 | 15.3526 | 16.5944 | 15.4367 | 15.2735 | 15.7305 | 8 | 8 | 7 | 9 | 13 | 9 | 10 | 11 |
| RANGRF | Q9HD47;Q | 0.000686 | 0.000145 | -1.49821 | | 1.0083 | -1.95696 | | 13.5498 | 11.6269 | 13.073 | 13.204 | 13.5247 | 13.7326 | 15.162 | 15.6171 | 1 | 1 | 1 | 1 | 1 | 1 | 1 | 1 |
| SLIT1 | O75093;O7 | 0.00341 | 0.000627 | -1.49384 | + | 3.9892 | -9.04241 | | 15.7855 | 16.0307 | 15.8479 | 17.1554 | 17.0427 | 17.5529 | 17.5557 | 17.6026 | 2 | 2 | 2 | 2 | 2 | 2 | 2 | 2 |
| KRT18 | P05783 | 1.71E-08 | 6.03E-09 | -1.48867 | + | 1.70101 | -3.14636 | | 14.3671 | 14.1848 | 14.2653 | 16.1352 | 14.3949 | 16.408 | 16.0262 | 15.8409 | 5 | 5 | 5 | 12 | 4 | 12 | 13 | 11 |
| SCIN | Q9Y6U3 | 1.06E-11 | 6.15E-12 | -1.48233 | + | 1.51658 | -2.8179 | | 15.4156 | 15.4349 | 15.5513 | 16.6229 | 16.8391 | 15.793 | 18.1798 | 17.3131 | 27 | 28 | 29 | 30 | 31 | 31 | 33 | 32 |
| S100A9 | P06702 | 0.004766 | 0.000855 | -1.47521 | | 1.05622 | -2.03659 | | 12.5221 | 12.3423 | 14.5562 | 14.871 | 13.8605 | 13.8504 | 14.592 | 15.9031 | 2 | 1 | 5 | 4 | 3 | 2 | 4 | 6 |
| HSPB6 | O14558 | 4.96E-08 | 1.66E-08 | -1.46319 | + | 1.82911 | -3.38199 | | 17.4768 | 16.8639 | 17.4248 | 17.7766 | 18.7321 | 18.6358 | 18.7389 | 19.7085 | 8 | 7 | 8 | 8 | 8 | 8 | 8 | 8 |
| SMURF2 | Q9HAU4 | 1.62E-18 | 6.61E-18 | -1.45354 | + | 1.86704 | -3.45307 | | 13.4102 | 13.4088 | 13.2665 | 15.2459 | 15.5999 | 13.764 | 14.5601 | 14.9069 | 10 | 9 | 11 | 22 | 18 | 7 | 13 | 18 |
| PEA15 | Q15121;Q1 | 2.06E-11 | 1.12E-11 | -1.45232 | + | 1.79617 | -3.32076 | | 17.2414 | 16.6399 | 17.9974 | 18.5625 | 18.244 | 18.293 | 19.0991 | 19.5275 | 6 | 6 | 7 | 8 | 7 | 7 | 7 | 8 |
| TMEM208 | Q9BTX3 | 0.004024 | 0.00073 | -1.45073 | + | 2.34528 | -14.8309 | | 12.664 | NaN | 12.8595 | NaN | NaN | 14.2157 | 14.2093 | NaN | 2 | NaN | 1 | NaN | NaN | 2 | 1 | NaN |
| DCBLD2 | Q96PD2;Q | 2.90E-15 | 4.33E-15 | -1.44679 | + | 1.74712 | -3.23042 | | 15.0663 | 14.8327 | 15.0169 | 16.957 | 16.4422 | 15.1311 | 16.6966 | 16.8668 | 6 | 6 | 5 | 8 | 5 | 5 | 8 | 7 |
| FTL | P02792 | 1.00E-11 | 5.83E-12 | -1.44311 | + | 1.3072 | -2.45738 | | 17.3512 | 16.996 | 17.6231 | 19.6659 | 18.0415 | 17.5112 | 19.0009 | 19.6131 | 9 | 9 | 9 | 9 | 9 | 9 | 9 | 9 |
| UBE2S | Q16763 | 2.67E-07 | 8.30E-08 | -1.44204 | + | 2.73197 | -5.28648 | | 14.2928 | 14.1394 | 14.5119 | 15.9204 | 15.8345 | 14.9997 | 15.8963 | 16.133 | 1 | 2 | 2 | 3 | 2 | 1 | 3 | 3 |
| MYL6 | P60660;P6 | 1.69E-05 | 4.32E-06 | -1.43142 | + | 1.83475 | -3.39252 | | 19.9176 | 19.5847 | 20.3924 | 20.3345 | 21.5797 | 21.2771 | 21.9065 | 21.8838 | 6 | 5 | 6 | 6 | 6 | 6 | 6 | 6 |
| SH3RF1 | Q7Z6J0 | 0.002267 | 0.000439 | -1.41641 | + | 2.38888 | -4.50451 | | 14.1355 | 14.4517 | 14.1274 | 15.5298 | 16.3524 | 14.9492 | 15.5885 | 15.8531 | 2 | 3 | 2 | 4 | 4 | 3 | 3 | 3 |
| SDC1 | P18827 | 8.63E-08 | 2.80E-08 | -1.41345 | + | 3.21166 | -6.53323 | | 13.7175 | 13.5698 | 13.6993 | 15.1594 | 15.2976 | 14.4853 | 15.0331 | 15.4026 | 1 | 1 | 1 | 1 | 2 | 1 | 1 | 2 |
| EPHB2 | P29323;P2 | 1.17E-11 | 6.68E-12 | -1.40723 | | 1.1246 | -2.24166 | | 14.5866 | 14.5806 | 14.282 | 17.4481 | NaN | 15.1577 | 15.3961 | 15.5594 | 4 | 5 | 4 | 9 | NaN | 6 | 3 | 6 |
| HMGA2 | P52926 | 0.003287 | 0.000607 | -1.40219 | + | 1.98808 | -3.68428 | | 14.6533 | 14.801 | 14.4897 | 16.2368 | 15.5497 | 15.3086 | 16.2706 | 16.8852 | 3 | 3 | 2 | 4 | 2 | 4 | 3 | 3 |
| CCND1 | P24385 | 2.03E-06 | 5.69E-07 | -1.40073 | + | 1.78281 | -3.29605 | | 15.5394 | 15.5007 | 15.4444 | 17.7184 | 16.9952 | 15.7975 | 16.7393 | 17.2274 | 7 | 7 | 7 | 11 | 7 | 6 | 7 | 10 |
| BID | P55957;P5 | 5.61E-07 | 1.68E-07 | -1.39936 | + | 2.05616 | -3.81744 | | 15.6448 | 15.1724 | 15.9754 | 16.7142 | 17.3486 | 16.1742 | 17.27 | 17.4774 | 4 | 4 | 4 | 4 | 4 | 4 | 4 | 4 |
| SGTB | Q96EQ0 | 1.72E-05 | 4.42E-06 | -1.39387 | + | 2.89662 | -5.69257 | | 15.0491 | 14.4795 | 15.4913 | 16.649 | 16.5093 | 16.1482 | 16.2554 | 16.4407 | 8 | 5 | 8 | 9 | 7 | 9 | 7 | 8 |
| CDH2 | P19022;P1 | 2.66E-13 | 2.26E-13 | -1.39128 | + | 2.64929 | -5.09044 | | 14.3592 | 14.3186 | 14.3591 | 15.536 | 15.0961 | 16.2233 | 16.125 | 15.7042 | 7 | 6 | 6 | 9 | 8 | 10 | 9 | 8 |
| ARL8A | Q96BM9 | 0.001589 | 0.000319 | -1.39078 | + | 3.28217 | -6.73386 | | 15.0746 | 15.4422 | 15.3358 | 16.3825 | 17.1509 | 16.6789 | 16.7797 | 16.3829 | 2 | 2 | 3 | 2 | 2 | 1 | 3 | 2 |
| HPCAL1 | P37235 | 8.72E-11 | 4.22E-11 | -1.38829 | + | 2.82262 | -5.5074 | | 16.3694 | 16.0158 | 16.5705 | 17.2382 | 18.0852 | 17.4057 | 18.0189 | 17.7863 | 11 | 12 | 14 | 12 | 15 | 15 | 15 | 15 |
| RACGAP1 | Q9H0H5 | 4.43E-11 | 2.27E-11 | -1.37794 | + | 2.08614 | -3.87683 | | 13.9933 | 13.846 | 13.9362 | 15.535 | 15.1648 | 14.3362 | 15.8423 | 15.6373 | 8 | 7 | 9 | 10 | 9 | 7 | 10 | 10 |
| PRKAG2 | Q9UGJ0;Q | 0.000179 | 4.09E-05 | -1.34269 | + | 1.42102 | -3.05265 | | 11.6809 | NaN | 12.3158 | 13.8042 | 13.1064 | 13.7438 | 12.7097 | NaN | 1 | NaN | 1 | 1 | 2 | 1 | 1 | NaN |
| NFU1 | Q9UMS0;Q | 1.58E-08 | 5.61E-09 | -1.34102 | + | 2.41641 | -4.5643 | | 14.8553 | 14.4544 | 15.1886 | 15.8031 | 16.3067 | 15.6785 | 16.414 | 16.6665 | 6 | 6 | 6 | 6 | 6 | 6 | 6 | 6 |
| LACC1 | Q8IV20 | 1.23E-06 | 3.53E-07 | -1.33109 | + | 2.44184 | -4.61999 | | 13.685 | 12.8588 | 13.516 | 14.4581 | 14.9764 | 15.1337 | 14.6291 | 14.2245 | 3 | 2 | 1 | 3 | 3 | 2 | 4 | 2 |
| ERH | P84090 | 3.75E-08 | 1.27E-08 | -1.33062 | + | 1.69971 | -3.14402 | | 16.9282 | 16.4512 | 17.1321 | 17.2338 | 18.2406 | 17.9022 | 18.4248 | 19.0376 | 6 | 6 | 6 | 6 | 6 | 6 | 6 | 6 |
| YAP1 | P46937;P4 | 9.62E-14 | 9.23E-14 | -1.32467 | + | 2.39393 | -4.51543 | | 15.0784 | 14.4772 | 15.5906 | 16.4068 | 16.6086 | 16.4643 | 15.8637 | 16.5236 | 11 | 9 | 13 | 12 | 14 | 13 | 14 | 13 |
| COX6B1 | P14854 | 0.001226 | 0.00025 | -1.31356 | + | 2.21731 | -4.1423 | | 16.2314 | 15.9158 | 16.4308 | 16.6833 | 17.4736 | 17.5725 | 17.9337 | 17.868 | 2 | 1 | 1 | 1 | 3 | 3 | 4 | 2 |
| NDFIP2 | Q9NV92 | 3.55E-06 | 9.76E-07 | -1.3035 | + | 2.4467 | -4.63066 | | 14.5365 | 14.4745 | 14.2048 | 16.1678 | 15.6637 | 14.996 | 15.6795 | 16.0368 | 2 | 2 | 2 | 2 | 1 | 1 | 2 | 3 |
| COX5A | P20674 | 3.78E-06 | 1.04E-06 | -1.30156 | + | 1.49481 | -2.77986 | | 16.6743 | 16.0624 | 16.7317 | 16.6433 | 17.7839 | 17.6527 | 18.342 | 18.5333 | 6 | 6 | 6 | 5 | 6 | 6 | 6 | 6 |
| CCNDBP1 | O95273;O | 0.002809 | 0.000528 | -1.29037 | + | 2.40353 | -4.53627 | | 14.0542 | 13.9805 | 14.4386 | 15.7642 | 14.6959 | 15.5671 | 15.7786 | 15.4348 | 1 | 2 | 1 | 3 | 2 | 3 | 2 | 2 |
| PLAT | P00750;P0 | 2.83E-07 | 8.77E-08 | -1.28522 | | 1.29593 | -2.5609 | | 12.8235 | 12.5849 | 12.8485 | 14.3173 | 14.5178 | 12.7861 | NaN | 14.5289 | 1 | 1 | 2 | 2 | 1 | 1 | NaN | 2 |
| ITGA2 | P17301 | 1.82E-11 | 1.01E-11 | -1.2837 | | 1.12702 | -2.1545 | | 16.4967 | 16.5376 | 16.5733 | 19.2354 | 16.8036 | 16.919 | 18.1639 | 17.9759 | 25 | 27 | 25 | 29 | 23 | 26 | 27 | 27 |
| CLEC2B | Q92478 | 4.11E-09 | 1.57E-09 | -1.28289 | + | 1.30639 | -2.456 | | 14.7404 | 14.6886 | 14.6083 | 17.4046 | 16.0324 | 15.2934 | 15.8324 | 15.2472 | 2 | 2 | 2 | 2 | 2 | 1 | 2 | 2 |
| PACSIN3 | Q9UKS6 | 4.40E-19 | 2.19E-18 | -1.27901 | + | 1.93255 | -3.57737 | | 14.0059 | 14.2563 | 13.9418 | 16.0943 | 15.7352 | 14.9816 | 14.6129 | 15.3112 | 5 | 6 | 5 | 7 | 5 | 5 | 5 | 6 |
| HNMT | P50135 | 0.000118 | 2.77E-05 | -1.27345 | + | 2.92406 | -6.61115 | | 13.4279 | 13.6983 | 13.5182 | NaN | 14.5092 | 15.0431 | 15.1213 | 14.6127 | 1 | 2 | 2 | NaN | 1 | 2 | 1 | 1 |
| TXNRD1 | Q16881;Q1 | 1.10E-15 | 1.87E-15 | -1.25919 | + | 2.64611 | -5.083 | | 17.5558 | 17.6094 | 17.5416 | 19.5074 | 18.5034 | 18.6821 | 18.9213 | 18.5264 | 21 | 20 | 21 | 26 | 25 | 25 | 24 | 26 |
| PAMR1 | Q6UXH9;Q | 1.14E-05 | 2.99E-06 | -1.25792 | + | 1.64656 | -3.04815 | | 14.9468 | 14.7249 | 14.6078 | 16.7047 | 16.4743 | 15.0809 | 16.2868 | 15.542 | 3 | 3 | 2 | 7 | 7 | 4 | 6 | 4 |
| RRM2 | P31350;P3 | 1.25E-18 | 5.39E-18 | -1.24996 | + | 1.35163 | -2.53297 | | 13.8954 | 13.7115 | 13.8956 | 15.379 | 15.1704 | 13.6636 | 15.424 | 15.7837 | 6 | 6 | 5 | 8 | 8 | 3 | 8 | 9 |
| ANXA3 | P12429 | 9.19E-05 | 2.19E-05 | -1.24478 | + | 1.67493 | -3.09919 | | 14.4038 | 14.2583 | 14.244 | 14.6054 | 15.9661 | 15.219 | 16.3382 | 15.6052 | 6 | 6 | 6 | 6 | 9 | 7 | 9 | 10 |
| HGFAC | Q04756 | 2.52E-06 | 7.02E-07 | -1.23875 | + | 2.70096 | -5.21235 | | 14.551 | 14.531 | 14.6704 | 16.0009 | 15.3352 | 15.4754 | 16.246 | 16.0567 | 3 | 3 | 3 | 4 | 1 | 2 | 4 | 4 |
| EXT1 | Q16394 | 6.24E-05 | 1.51E-05 | -1.23373 | + | 2.4117 | -4.55404 | | 13.5887 | 13.9775 | 13.7465 | 15.2487 | 14.9184 | 14.2929 | 15.3871 | 15.1761 | 3 | 1 | 3 | 7 | 3 | 5 | 5 | 4 |
| NCALD | P61601 | 2.60E-05 | 6.55E-06 | -1.23119 | | 1.25944 | -2.37656 | | 15.2127 | 14.6737 | 15.0828 | 16.7372 | 14.9019 | 16.1092 | 17.1386 | 16.2178 | 2 | 1 | 1 | 4 | 2 | 4 | 1 | 4 |
| CD59 | P13987;P1 | 1.45E-08 | 5.16E-09 | -1.23097 | + | 3.3754 | -7.00664 | | 19.2417 | 19.1001 | 19.4939 | 20.1498 | 20.3699 | 20.814 | 20.6664 | 20.5476 | 4 | 5 | 4 | 5 | 4 | 5 | 4 | 5 |
| CRIM1 | Q9NZV1 | 2.56E-24 | 7.58E-23 | -1.23039 | + | 2.24201 | -4.19336 | | 13.9855 | 13.9334 | 14.0531 | 15.2343 | 15.0781 | 14.4749 | 15.6706 | 15.6475 | 12 | 11 | 11 | 11 | 11 | 12 | 13 | 14 |
| FBN2 | P35556 | 2.14E-16 | 4.53E-16 | -1.22371 | + | 3.93128 | -8.8308 | | 15.5492 | 15.4911 | 15.6356 | 16.7507 | 16.9784 | 16.4134 | 16.8177 | 16.9515 | 29 | 28 | 28 | 37 | 32 | 29 | 34 | 33 |
| CLGN | O14967 | 5.18E-08 | 1.73E-08 | -1.21935 | | 1.07197 | -2.14547 | | 12.6717 | 14.6956 | 13.1486 | 15.2107 | 14.2249 | 14.5837 | NaN | 14.8793 | 2 | 2 | 2 | 12 | 3 | 7 | NaN | 10 |
| DCLK1 | O15075;O1 | 4.79E-09 | 1.81E-09 | -1.21826 | | 0.503326 | -1.09921 | | 13.3994 | 13.2189 | 13.4857 | 15.216 | 14.8612 | 15.9246 | 11.3431 | 15.5866 | 5 | 4 | 3 | 8 | 8 | 14 | 3 | 11 |
| MIF | P14174 | 1.76E-05 | 4.50E-06 | -1.21819 | + | 2.0518 | -3.80884 | | 20.8822 | 20.761 | 21.339 | 21.7213 | 22.2193 | 21.9295 | 22.1772 | 23.014 | 3 | 2 | 2 | 3 | 3 | 3 | 3 | 3 |
| ELOB | Q15370;Q1 | 8.25E-20 | 5.29E-19 | -1.21132 | + | 3.21678 | -6.54763 | | 17.0797 | 16.8072 | 17.2891 | 17.8595 | 18.2631 | 18.2474 | 18.5243 | 18.4557 | 7 | 7 | 8 | 8 | 7 | 8 | 8 | 8 |
| JCAD | Q9P266 | 7.01E-09 | 2.60E-09 | -1.20638 | + | 2.55214 | -4.86643 | | 14.1807 | 14.3846 | 14.418 | 15.9589 | 15.0266 | 15.9391 | 15.3258 | 15.4204 | 14 | 16 | 15 | 26 | 16 | 26 | 17 | 21 |
| TSPYL1 | Q9H0U9 | 0.006866 | 0.001204 | -1.20194 | + | 1.71491 | -3.17161 | | 14.9676 | 15.1014 | 14.6407 | 15.6452 | 16.5973 | 15.3713 | 16.0922 | 16.8198 | 2 | 3 | 4 | 4 | 4 | 3 | 3 | 4 |
| PLIN3 | O60664-3 | 0.027918 | 0.004597 | -1.19836 | | 0.731173 | -1.53361 | | 12.388 | 15.2925 | 14.302 | 15.3685 | 15.6053 | 14.3988 | NaN | 15.3975 | 1 | 2 | 2 | 1 | 1 | 1 | NaN | 1 |
| COX5B | P10606 | 0.000666 | 0.000141 | -1.19635 | | 1.22264 | -2.3146 | | 15.4571 | 15.1355 | 15.8199 | 16.1309 | 16.2379 | 16.0235 | 16.9287 | 18.0149 | 6 | 5 | 5 | 6 | 5 | 6 | 6 | 6 |
| HSPA2 | P54652 | 2.56E-24 | 7.58E-23 | -1.1906 | | 1.15427 | -2.2 | | 16.282 | 16.4015 | 16.3464 | 16.5567 | 17.8074 | 16.953 | 18.9162 | 17.4361 | 14 | 15 | 14 | 13 | 14 | 14 | 16 | 14 |
| ADAMTS1 | Q9UHI8 | 1.74E-06 | 4.92E-07 | -1.18756 | + | 1.6536 | -3.26984 | | NaN | 13.4458 | 12.8834 | 13.9785 | 15.0446 | 14.2016 | 14.5212 | 14.015 | NaN | 1 | 2 | 4 | 1 | 5 | 6 | 3 |
| ADAMTSL1 | Q8N6G6 | 9.41E-09 | 3.43E-09 | -1.18428 | + | 1.80325 | -3.33388 | | 13.7175 | 13.4526 | 13.8991 | 15.4456 | 15.0447 | 14.2136 | 14.3215 | 15.3447 | 1 | 1 | 1 | 4 | 1 | 1 | 1 | 3 |
| FKRP | Q9H9S5 | 0.007905 | 0.001376 | -1.17442 | + | 1.50777 | -2.97265 | | 14.987 | NaN | 15.8755 | 17.0665 | 16.1018 | 16.4809 | 17.0245 | 16.3547 | 3 | NaN | 4 | 7 | 3 | 3 | 3 | 4 |
| COX7A2 | P14406 | 1.21E-05 | 3.16E-06 | -1.17129 | | 1.12108 | -2.14459 | | 16.6435 | 16.3841 | 17.1442 | 17.8676 | 17.5253 | 16.8263 | 18.036 | 19.2209 | 2 | 2 | 2 | 2 | 2 | 2 | 2 | 2 |
| WDHD1 | O75717 | 6.17E-05 | 1.50E-05 | -1.16423 | + | 2.01017 | -3.72724 | | 13.6008 | 13.3746 | 13.5217 | 14.4779 | 15.2856 | 13.9003 | 14.7416 | 14.911 | 3 | 3 | 4 | 5 | 3 | 2 | 4 | 9 |
| PHLDA2 | Q53GA4 | 0.001098 | 0.000225 | -1.16407 | + | 1.30737 | -2.45767 | | 15.0852 | 15.102 | 15.3899 | 17.1302 | 16.5132 | 15.1142 | 16.8659 | 16.1586 | 2 | 2 | 2 | 2 | 2 | 1 | 2 | 2 |
| SVIL | O95425-4 | 1.32E-13 | 1.22E-13 | -1.16191 | + | 2.43637 | -4.60798 | | 13.7804 | 13.9868 | 13.9212 | 15.2138 | 15.6822 | 14.6766 | 15.0237 | 14.694 | 8 | 9 | 9 | 28 | 22 | 16 | 21 | 14 |
| VCAN | P13611 | 1.29E-09 | 5.28E-10 | -1.16178 | + | 2.28705 | -4.2874 | | 13.8313 | 13.8848 | 13.3212 | 14.9966 | 14.1782 | 14.8559 | 15.2414 | 14.9323 | 5 | 8 | 9 | 12 | 4 | 14 | 16 | 12 |
| ASB6 | Q9NWX5 | 0.000295 | 6.56E-05 | -1.16071 | + | 2.57379 | -4.91576 | | 13.8041 | 13.8812 | 13.8666 | 15.2584 | 15.1523 | 14.3323 | 15.0164 | 15.2974 | 1 | 1 | 1 | 3 | 2 | 1 | 2 | 3 |
| NDN | Q99608 | 0.00042 | 9.14E-05 | -1.1511 | + | 1.77679 | -4.85106 | | 12.9738 | NaN | 12.6731 | 14.2482 | 13.6875 | NaN | NaN | 13.9881 | 1 | NaN | 1 | 4 | 4 | NaN | NaN | 4 |
| F3 | P13726 | 0.00039 | 8.53E-05 | -1.14297 | + | 1.66286 | -3.07745 | | 14.3105 | 14.1498 | 14.1822 | 14.4599 | 15.109 | 15.5896 | 16.1298 | 15.4975 | 3 | 4 | 4 | 4 | 4 | 4 | 5 | 4 |
| MME | P08473 | 1.22E-13 | 1.13E-13 | -1.14196 | + | 2.35509 | -4.43176 | | 15.9252 | 15.9291 | 15.9288 | 17.6625 | 16.5395 | 17.224 | 17.1444 | 16.778 | 29 | 32 | 32 | 37 | 31 | 37 | 37 | 34 |
| CUTA | O60888;O6 | 1.23E-05 | 3.20E-06 | -1.13557 | + | 1.57665 | -2.92361 | | 15.9322 | 15.323 | 15.8342 | 15.995 | 16.4476 | 17.1811 | 17.0107 | 17.5256 | 2 | 2 | 2 | 2 | 2 | 2 | 2 | 3 |

| Gene names | UniProt IDs | Protein Group P-value | Protein Group Q-value | Log2 Difference NUP214 vs Controls (n=5) | p<0.05 | -log10 p-value | T-test Test statistic | NPC Localization | Log2 MS2_quantity_NUP214_1 | Log2 MS2_quantity_NUP214_2 | Log2 MS2_quantity_NUP214_3 | Log2 MS2_quantity_Control_1 | Log2 MS2_quantity_Control_2 | Log2 MS2_quantity_Control_3 | Log2 MS2_quantity_Control_4 | Log2 MS2_quantity_Control_5 | Number Peptides for Quantitation NUP214_1 | Number Peptides for Quantitation NUP214_2 | Number Peptides for Quantitation NUP214_3 | Number Peptides for Quantitation Control_1 | Number Peptides for Quantitation Control_2 | Number Peptides for Quantitation Control_3 | Number Peptides for Quantitation Control_4 | Number Peptides for Quantitation Control_5 |
|---|---|---|---|---|---|---|---|---|---|---|---|---|---|---|---|---|---|---|---|---|---|---|---|---|
| DYNLRB1 | Q9NP97 | 2.94E-06 | 8.14E-07 | -1.13536 | + | 2.47051 | -4.68326 | | 18.0726 | 17.9916 | 18.2814 | 18.8297 | 19.5132 | 18.8335 | 19.4136 | 19.6628 | 5 | 5 | 5 | 5 | 5 | 5 | 5 | 5 |
| TYMS | P04818 | 9.50E-13 | 6.92E-13 | -1.13138 | + | 1.58906 | -2.94559 | | 15.3841 | 15.2874 | 15.3118 | 16.8729 | 16.4048 | 15.3962 | 16.5841 | 17.0379 | 8 | 10 | 10 | 11 | 10 | 7 | 11 | 11 |
| SERPINB12 | Q96P63;Q | 0.001472 | 0.000297 | -1.11802 | + | 2.33038 | -4.37899 | | 14.0808 | 13.6742 | 14.6372 | 15.6205 | 15.1767 | 15.0551 | 14.9966 | 15.3951 | 3 | 1 | 5 | 7 | 5 | 3 | 6 | 6 |
| DAGLB | Q8NCG7;Q | 3.69E-15 | 5.35E-15 | -1.11681 | + | 3.65021 | -7.86388 | | 14.1215 | 14.3001 | 14.1033 | 15.2284 | 15.2645 | 14.9713 | 15.5725 | 15.4222 | 5 | 5 | 3 | 5 | 6 | 6 | 7 | 5 |
| AAMDC | Q9H7C9 | 1.22E-13 | 1.13E-13 | -1.11386 | + | 2.90694 | -5.71874 | | 15.3611 | 15.2787 | 15.5693 | 16.9439 | 16.6257 | 16.2233 | 16.2057 | 16.5857 | 7 | 6 | 6 | 7 | 8 | 8 | 7 | 7 |
| MDP1 | Q86V88;Q | 0.000616 | 0.000131 | -1.11254 | + | 3.54899 | -7.53856 | | 15.0444 | 15.209 | 15.2951 | 16.0744 | 16.212 | 16.1125 | 16.491 | 16.5871 | 1 | 1 | 1 | 1 | 1 | 1 | 1 | 1 |
| GNG12 | Q9UBI6 | 2.15E-14 | 2.47E-14 | -1.11115 | + | 1.98252 | -3.67351 | | 16.28 | 15.9967 | 16.3701 | 17.1441 | 16.9957 | 17.064 | 17.2458 | 18.184 | 5 | 5 | 6 | 6 | 6 | 6 | 6 | 6 |
| MKI67 | P46013 | 1.50E-07 | 4.75E-08 | -1.11077 | + | 1.76258 | -3.25878 | | 13.6996 | 13.7012 | 14.1813 | 15.0353 | 14.8793 | 14.1201 | 15.2924 | 15.5303 | 8 | 8 | 6 | 16 | 11 | 6 | 15 | 18 |
| DCD | P81605;P8 | 8.09E-05 | 1.94E-05 | -1.10978 | | 1.30099 | -2.44685 | | 14.2793 | 14.0102 | 14.8256 | 16.2159 | 15.5455 | 14.7164 | 16.1128 | 14.8168 | 2 | 2 | 3 | 3 | 3 | 2 | 3 | 2 |
| FAT1 | Q14517 | 0.00271 | 0.000513 | -1.10937 | + | 1.69694 | -3.13898 | | 13.7483 | 13.9893 | 13.7683 | 14.0189 | 15.1412 | 14.7656 | 15.5197 | 15.2778 | 10 | 10 | 10 | 10 | 20 | 21 | 34 | 27 |
| FGF2 | P09038-2 | 4.64E-06 | 1.26E-06 | -1.10729 | + | 3.38432 | -7.03319 | | 17.3979 | 17.3813 | 17.3382 | 18.2063 | 18.2896 | 18.6795 | 18.8254 | 18.398 | 7 | 6 | 6 | 7 | 7 | 8 | 8 | 8 |
| ABCB8 | Q9NUT2;Q | 0.00227 | 0.000439 | -1.10453 | | 0.991687 | -2.11484 | | 13.8901 | 13.8623 | 13.7657 | 13.9427 | 15.195 | 15.694 | NaN | NaN | 2 | 2 | 1 | 2 | 1 | 3 | NaN | NaN |
| CDKN1A | P38936 | 0.00014 | 3.26E-05 | -1.10415 | + | 1.93786 | -3.58752 | | 15.7931 | 15.9027 | 15.9142 | 17.3152 | 17.1655 | 16.0727 | 17.2661 | 17.0514 | 5 | 4 | 3 | 4 | 4 | 4 | 5 | 5 |
| FANCI | Q9NVI1 | 0.000139 | 3.24E-05 | -1.10162 | + | 2.03016 | -3.76631 | | 13.3641 | 13.6796 | 13.5938 | 14.6151 | 14.1866 | 15.3203 | 14.8946 | 14.2208 | 1 | 1 | 1 | 2 | 1 | 1 | 2 | 2 |
| NDOR1 | Q9UHB4;Q | 2.73E-07 | 8.47E-08 | -1.09756 | | 1.2654 | -2.38662 | | 14.5587 | 14.2279 | 14.439 | 14.7456 | 16.3788 | 14.8688 | 16.2373 | 15.3 | 4 | 4 | 5 | 3 | 3 | 3 | 2 | 2 |
| CIAO2B | Q9Y3D0 | 0.00059 | 0.000126 | -1.09658 | | 1.22961 | -2.32632 | | 15.4007 | 14.5949 | 15.504 | 15.1908 | 16.5507 | 15.9334 | 16.6755 | 16.9651 | 4 | 3 | 4 | 4 | 4 | 3 | 4 | 4 |
| AZGP1 | P25311 | 0.00324 | 0.000599 | -1.09112 | + | 2.1842 | -4.07439 | | 14.1748 | 14.2366 | 14.2906 | 16.0541 | 15.1717 | 14.844 | 15.2102 | 15.3456 | 1 | 2 | 3 | 4 | 2 | 3 | 3 | 3 |
| PRMT9 | Q6P2P2 | 0.012004 | 0.002041 | -1.09024 | | 0.540316 | -1.2877 | | 14.0959 | 13.7444 | NaN | NaN | 13.8588 | 16.1008 | 15.0715 | NaN | 3 | 4 | NaN | NaN | 2 | 1 | 1 | NaN |
| SELENOM | Q8WWX9 | 0.000204 | 4.63E-05 | -1.09014 | + | 1.48442 | -2.76176 | | 15.6546 | 15.6623 | 15.2954 | 16.1813 | 16.067 | 16.4368 | 16.7804 | 17.6725 | 2 | 3 | 3 | 2 | 2 | 4 | 4 | 4 |
| STC2 | O76061 | 0.003069 | 0.00057 | -1.08856 | | 1.12586 | -2.15256 | | 12.9837 | 13.1996 | 13.3135 | 13.6602 | 14.1166 | 13.3894 | 15.5146 | 14.5902 | 3 | 3 | 4 | 4 | 4 | 3 | 4 | 4 |
| S100A4 | P26447 | 9.90E-05 | 2.34E-05 | -1.08566 | | 0.936438 | -1.83761 | | 20.5554 | 19.979 | 20.8277 | 20.384 | 22.7453 | 20.8106 | 21.8751 | 21.8833 | 5 | 5 | 6 | 6 | 7 | 6 | 7 | 7 |
| SLC9A7 | Q96T83 | 5.52E-07 | 1.65E-07 | -1.08555 | + | 3.44357 | -7.21172 | | 14.8154 | 15.1075 | 14.9909 | 16.2429 | 16.2944 | 15.7389 | 16.0872 | 15.9205 | 3 | 3 | 2 | 3 | 3 | 3 | 3 | 3 |
| ELOC | Q15369 | 8.46E-12 | 4.98E-12 | -1.085 | + | 1.76339 | -3.26027 | | 17.1526 | 16.7715 | 17.3922 | 17.3149 | 18.6421 | 18.2262 | 18.3456 | 18.4232 | 6 | 6 | 6 | 6 | 6 | 6 | 6 | 6 |
| PFDN5 | Q99471 | 0.000742 | 0.000156 | -1.08056 | + | 1.39966 | -2.61519 | | 15.1955 | 14.4156 | 15.9994 | 16.0422 | 15.9836 | 16.1647 | 16.2378 | 16.992 | 6 | 6 | 6 | 6 | 6 | 6 | 6 | 6 |
| RRN3 | Q9NYV6;Q | 0.007567 | 0.001321 | -1.07971 | + | 1.88906 | -3.49464 | | 14.0642 | 14.7264 | 14.8706 | 15.7379 | 16.0515 | 15.0421 | 15.9562 | 15.3794 | 2 | 4 | 2 | 4 | 3 | 3 | 3 | 2 |
| CYB5A | P00167;P0 | 3.58E-07 | 1.10E-07 | -1.07912 | + | 1.65637 | -3.06578 | | 15.8368 | 15.3264 | 15.9242 | 16.1231 | 17.0797 | 16.2752 | 17.3705 | 17.0263 | 5 | 5 | 5 | 5 | 5 | 5 | 5 | 5 |
| KIF23 | Q02241;Q0 | 2.30E-09 | 9.05E-10 | -1.07435 | + | 1.89175 | -3.49972 | | 13.9267 | 13.8559 | 13.8307 | 15.1918 | 14.918 | 14.0707 | 15.3559 | 15.1907 | 5 | 8 | 5 | 11 | 7 | 8 | 11 | 10 |
| TNFAIP2 | Q03169 | 0.003647 | 0.000667 | -1.07426 | | 0.661619 | -1.37604 | | 14.2965 | 14.2707 | 14.2699 | 17.6429 | 14.5804 | 15.1975 | 14.8544 | 14.4912 | 1 | 1 | 3 | 1 | 1 | 3 | 1 | 1 |
| DMD | P11532;P1 | 0.002307 | 0.000445 | -1.06998 | + | 1.76211 | -4.79126 | | 14.272 | 13.9425 | 13.7584 | 15.2094 | NaN | 14.9125 | NaN | NaN | 3 | 3 | 3 | 2 | NaN | 3 | NaN | NaN |
| S100A10 | P60903 | 1.86E-09 | 7.40E-10 | -1.06666 | + | 1.65234 | -3.05853 | | 18.3572 | 17.8213 | 18.5443 | 18.4626 | 19.7294 | 19.5686 | 19.1504 | 19.6268 | 10 | 9 | 10 | 10 | 10 | 10 | 10 | 11 |
| NCOA4 | Q13772;Q1 | 0.000108 | 2.55E-05 | -1.06614 | + | 2.0214 | -3.74915 | | 14.4301 | 14.7441 | 14.7251 | 16.3952 | 15.459 | 15.174 | 15.6169 | 15.8511 | 2 | 4 | 2 | 5 | 2 | 2 | 3 | 3 |
| ATOX1 | O00244 | 3.08E-08 | 1.06E-08 | -1.06589 | + | 1.9105 | -3.5353 | | 16.4375 | 15.9585 | 16.4664 | 16.5944 | 17.3956 | 17.4334 | 17.4806 | 17.8629 | 6 | 6 | 7 | 7 | 7 | 7 | 8 | 7 |
| NDUFB11 | Q9NX14;Q | 0.000174 | 3.99E-05 | -1.06114 | + | 1.36957 | -2.56362 | | 15.3609 | 14.8094 | 15.5317 | 15.2759 | 16.1388 | 16.4655 | 16.948 | 16.6473 | 3 | 3 | 3 | 3 | 3 | 3 | 3 | 3 |
| APOB | P04114 | 3.61E-11 | 1.88E-11 | -1.05246 | + | 2.34438 | -4.40885 | | 16.3131 | 16.4393 | 16.4927 | 17.9912 | 17.4314 | 17.077 | 17.1097 | 17.7282 | 8 | 9 | 9 | 10 | 9 | 9 | 9 | 10 |
| PLXNA3 | P51805 | 4.23E-06 | 1.15E-06 | -1.05049 | + | 4.27289 | -10.1452 | | 13.7487 | 13.5879 | 13.7117 | 14.6832 | 14.9591 | 14.511 | 14.7915 | 14.7215 | 9 | 9 | 11 | 15 | 9 | 10 | 9 | 14 |
| PITPNC1 | Q9UKF7;Q | 0.001618 | 0.000324 | -1.04669 | + | 1.76992 | -3.27229 | | 14.3061 | 14.5472 | 14.6308 | 16.3553 | 15.2064 | 15.1068 | 15.7738 | 15.2648 | 5 | 4 | 4 | 7 | 3 | 4 | 4 | 4 |
| TMEM47 | Q9BQJ4 | 7.95E-16 | 1.40E-15 | -1.04635 | + | 1.80567 | -3.33837 | | 14.9168 | 14.7786 | 14.8585 | 15.9728 | 14.983 | 16.1236 | 16.2883 | 16.1206 | 2 | 2 | 2 | 3 | 3 | 3 | 3 | 3 |
| TSC22D2 | O75157;O7 | 1.25E-06 | 3.59E-07 | -1.04188 | + | 2.90635 | -5.71723 | | 15.4339 | 15.0976 | 15.1806 | 16.1469 | 16.5243 | 16.5224 | 15.8644 | 16.3382 | 5 | 5 | 6 | 7 | 6 | 7 | 6 | 7 |
| PKP4 | Q99569;Q | 0.000984 | 0.000203 | -1.04053 | + | 2.67466 | -5.15004 | | 13.9892 | 13.2262 | 13.6263 | 14.6739 | 14.6353 | 14.6965 | 14.3465 | 14.92 | 3 | 3 | 3 | 6 | 2 | 6 | 4 | 2 |
| S100A16 | Q96FQ6 | 1.76E-15 | 2.82E-15 | -1.03347 | + | 1.36188 | -2.55047 | | 15.453 | 15.0317 | 16.0795 | 16.3263 | 16.084 | 16.2446 | 16.6063 | 17.5132 | 5 | 5 | 5 | 5 | 5 | 5 | 5 | 5 |
| PTGS1 | P23219;P2 | 5.27E-07 | 1.58E-07 | -1.03145 | + | 2.93474 | -5.78971 | | 15.8136 | 15.8368 | 15.8032 | 16.4637 | 16.9297 | 16.7462 | 16.8218 | 17.2851 | 11 | 13 | 11 | 13 | 15 | 14 | 15 | 15 |
| FUCA1 | P04066 | 2.58E-10 | 1.16E-10 | -1.03139 | + | 2.31315 | -4.34243 | | 15.4906 | 15.6251 | 15.6711 | 16.1232 | 17.0422 | 16.3039 | 16.8613 | 16.8044 | 3 | 3 | 3 | 3 | 3 | 3 | 3 | 3 |
| CLIC3 | O95833 | 5.49E-08 | 1.83E-08 | -1.02947 | | 1.27112 | -2.39629 | | 13.7665 | 13.5377 | 14.1214 | 13.8411 | 15.0804 | 15.4187 | 14.4257 | 15.4242 | 4 | 4 | 5 | 3 | 2 | 5 | 5 | 5 |
| ACTBL2 | Q562R1 | 0.005422 | 0.000963 | -1.02783 | + | 1.76872 | -3.27008 | | 23.1797 | 23.7967 | 23.8903 | 24.5849 | 24.6036 | 24.0058 | 25.2583 | 24.7978 | 3 | 3 | 5 | 5 | 5 | 3 | 6 | 6 |
| KLHL11 | Q9NVR0 | 0.001718 | 0.000342 | -1.02595 | + | 2.68639 | -5.17779 | | 14.2374 | 13.9264 | 14.1545 | 14.9349 | 15.3263 | 14.677 | 15.3861 | 15.336 | 4 | 4 | 4 | 6 | 4 | 7 | 4 | 4 |
| COBLL1 | Q53SF7;Q5 | 5.68E-08 | 1.89E-08 | -1.02426 | | 0.907092 | -1.78883 | | 12.8029 | 14.8149 | 12.9246 | 15.4045 | 14.382 | 14.5549 | 14.4037 | 13.9468 | 1 | 1 | 1 | 5 | 1 | 2 | 4 | 3 |
| TK1 | P04183 | 1.03E-11 | 5.94E-12 | -1.0149 | | 0.978704 | -1.90781 | | 13.8726 | 14.1703 | 13.9472 | 15.561 | 15.2113 | 13.507 | 15.0394 | 15.7393 | 4 | 4 | 4 | 5 | 4 | 2 | 7 | 8 |
| OSCP1 | Q8WVF1;Q | 0.054439 | 0.008825 | -1.01265 | + | 2.21717 | -4.142 | | 13.6122 | 13.7687 | 14.0971 | 14.2942 | 15.2072 | 14.8505 | 15.1488 | 14.6924 | 1 | 2 | 2 | 2 | 1 | 2 | 3 | 3 |
| ATP6V1F | Q16864;Q1 | 1.51E-05 | 3.90E-06 | -1.00531 | + | 1.37529 | -2.5734 | | 15.9024 | 15.2065 | 15.749 | 16.0245 | 16.1969 | 16.3718 | 17.1592 | 17.3707 | 4 | 4 | 5 | 5 | 4 | 5 | 5 | 5 |
| STARD7 | Q9NQZ5 | 8.98E-12 | 5.26E-12 | -1.00189 | + | 2.22536 | -4.1589 | | 14.9545 | 14.5645 | 15.1503 | 16.0445 | 16.0144 | 15.2834 | 15.9825 | 16.1334 | 3 | 3 | 1 | 5 | 2 | 2 | 2 | 4 |
| ARG1 | P05089;P0 | 0.001981 | 0.000389 | -1.00003 | | 0.875268 | -1.73587 | | 14.4152 | 14.661 | 14.4526 | 16.2461 | 15.6976 | 13.8296 | 15.8065 | 15.9684 | 3 | 2 | 4 | 6 | 4 | 1 | 4 | 4 |
| PBK | Q96KB5;Q | 0.000186 | 4.24E-05 | -0.998085 | | 1.26683 | -2.38904 | | 14.2974 | 14.2047 | 13.992 | 15.763 | 15.0583 | 14.0432 | 15.2563 | 15.6931 | 4 | 3 | 3 | 7 | 6 | 3 | 8 | 8 |
| IQGAP3 | Q86VI3 | 3.75E-17 | 1.00E-16 | -0.996964 | + | 2.05317 | -3.81154 | | 14.5711 | 14.5916 | 14.5858 | 15.8114 | 15.6566 | 14.8598 | 15.5545 | 16.0167 | 31 | 30 | 25 | 39 | 35 | 25 | 34 | 40 |
| CYP1B1 | Q16678 | 3.86E-09 | 1.48E-09 | -0.995904 | | 1.11099 | -2.12777 | | 13.3538 | 13.5172 | 13.4837 | 14.9602 | 13.0846 | 14.9527 | 14.7053 | 14.5346 | 6 | 2 | 8 | 14 | 2 | 15 | 16 | 13 |
| MYL6B | P14649 | 8.42E-16 | 1.47E-15 | -0.988212 | + | 2.01066 | -3.7282 | | 19.9502 | 19.6272 | 20.2531 | 20.3519 | 20.8304 | 20.9373 | 21.17 | 21.3689 | 4 | 5 | 5 | 5 | 4 | 4 | 5 | 4 |
| PTX3 | P26022 | 8.84E-14 | 8.58E-14 | -0.987809 | + | 2.84621 | -5.56595 | | 15.495 | 15.5822 | 15.7155 | 16.6966 | 16.3196 | 17.021 | 16.5378 | 16.3517 | 8 | 8 | 8 | 9 | 8 | 9 | 10 | 10 |
| POLA1 | P09884 | 0.000336 | 7.40E-05 | -0.98755 | + | 3.23757 | -6.60638 | | 21.7393 | 21.5598 | 21.4674 | 22.5439 | 22.8175 | 22.2568 | 22.4806 | 22.783 | 3 | 2 | 4 | 5 | 2 | 2 | 2 | 2 |
| BRI3 | O95415 | 0.001497 | 0.000301 | -0.987233 | + | 2.16625 | -4.03782 | | 17.1186 | 17.1021 | 16.6829 | 17.743 | 18.368 | 17.4674 | 18.2661 | 17.9309 | 2 | 2 | 2 | 2 | 2 | 2 | 2 | 2 |
| JUN | P05412 | 1.04E-07 | 3.36E-08 | -0.9857 | | 1.26038 | -2.37815 | | 15.5295 | 15.5608 | 15.3949 | 17.0177 | 16.8241 | 15.4298 | 16.1233 | 17.0088 | 3 | 2 | 2 | 3 | 3 | 3 | 3 | 3 |
| EIF1 | P41567 | 3.58E-17 | 9.69E-17 | -0.983645 | + | 1.51393 | -2.81325 | | 16.7215 | 16.3039 | 16.868 | 16.8846 | 17.4526 | 17.4122 | 18.2069 | 18.1176 | 9 | 9 | 9 | 10 | 10 | 9 | 10 | 10 |
| IL17RA | Q96F46;Q | 1.50E-05 | 3.87E-06 | -0.983563 | + | 1.45159 | -2.70476 | | 12.9105 | 13.253 | 13.164 | 13.6334 | 14.6582 | 13.7708 | 13.5814 | 14.8198 | 2 | 2 | 1 | 3 | 4 | 2 | 1 | 3 |
| SH3BP5L | Q7L8J4 | 0.000502 | 0.000108 | -0.982268 | + | 1.40831 | -2.63006 | | 13.6168 | 13.6979 | 13.5817 | 14.943 | 15.0025 | 13.5217 | 14.6628 | 14.9419 | 3 | 4 | 3 | 5 | 4 | 4 | 4 | 6 |
| CCN2 | P29279 | 2.26E-10 | 1.03E-10 | -0.978664 | | 0.940019 | -1.84356 | | 16.1848 | 16.2508 | 16.2905 | 16.9277 | 15.84 | 17.7691 | 18.1287 | 17.438 | 10 | 10 | 11 | 12 | 9 | 14 | 14 | 12 |
| TSEN15 | Q8WW01 | 0.000384 | 8.40E-05 | -0.976821 | | 1.01908 | -1.97488 | | 14.1432 | 13.2784 | 13.9752 | 13.7069 | 14.5029 | 15.7216 | 15.2401 | 14.7072 | 2 | 1 | 1 | 1 | 1 | 2 | 2 | 2 |
| NCS1 | P62166 | 0.004885 | 0.000875 | -0.970931 | + | 2.30948 | -4.33466 | | 14.8368 | 14.3633 | 14.817 | 15.4944 | 16.0839 | 15.217 | 15.6273 | 15.7939 | 3 | 2 | 3 | 4 | 4 | 5 | 4 | 5 |
| USF2 | Q15853;Q1 | 0.002216 | 0.00043 | -0.969973 | + | 1.4726 | -2.7412 | | 14.0279 | 13.6718 | 13.7691 | 14.3753 | 15.7096 | 14.4149 | 15.0289 | 14.4358 | 2 | 3 | 3 | 2 | 2 | 1 | 1 | 2 |
| BOD1 | Q96IK1 | 2.01E-05 | 5.13E-06 | -0.968156 | + | 1.86339 | -3.44619 | | 14.042 | 13.7584 | 14.2125 | 14.4699 | 15.3084 | 14.5161 | 15.1979 | 15.37 | 2 | 2 | 3 | 5 | 4 | 3 | 3 | 4 |
| DYNLL1 | P63167 | 6.69E-10 | 2.82E-10 | -0.964895 | + | 1.35051 | -2.53105 | | 16.4016 | 16.0142 | 16.7807 | 17.2026 | 17.4279 | 16.5396 | 17.5017 | 18.1466 | 5 | 5 | 5 | 4 | 4 | 4 | 5 | 5 |
| BANF1 | O75531 | 2.17E-12 | 1.44E-12 | -0.964193 | | 0.865443 | -1.7195 | | 17.7121 | 17.1382 | 17.9792 | 17.1414 | 18.3871 | 18.8567 | 19.011 | 19.4739 | 4 | 4 | 4 | 4 | 4 | 4 | 4 | 4 |
| NEDD8 | Q15843 | 3.27E-08 | 1.12E-08 | -0.964132 | + | 2.27556 | -4.2633 | | 17.7109 | 17.4761 | 17.613 | 18.0511 | 18.7373 | 18.4101 | 18.5781 | 19.0442 | 4 | 4 | 4 | 4 | 4 | 4 | 4 | 4 |
| EEF1AKMT1 | Q8WVE0 | 0.011519 | 0.001961 | -0.962512 | | 0.624563 | -1.38825 | | 13.9182 | 14.294 | 14.1132 | NaN | 14.3374 | 16.4394 | 14.4362 | NaN | 1 | 1 | 2 | NaN | 1 | 1 | 1 | NaN |
| UGCG | Q16739 | 1.81E-06 | 5.10E-07 | -0.962241 | + | 1.98068 | -3.66995 | | 14.3996 | 14.3104 | 14.2395 | 15.8209 | 15.0206 | 14.7288 | 15.2355 | 15.5879 | 4 | 4 | 2 | 5 | 3 | 4 | 3 | 4 |
| FDX2 | Q6P4F2 | 9.20E-05 | 2.19E-05 | -0.95778 | + | 1.68953 | -3.12557 | | 14.2802 | 13.2545 | 14.1779 | 14.2956 | 14.9695 | 15.0606 | 15.0703 | 14.9139 | 1 | 1 | 2 | 2 | 1 | 2 | 1 | 1 |
| ATP5F1D | P30049 | 1.65E-09 | 6.61E-10 | -0.95758 | + | 1.79732 | -3.32289 | | 17.1011 | 16.9026 | 17.4288 | 17.6016 | 17.9067 | 17.8566 | 18.537 | 18.6071 | 3 | 3 | 3 | 3 | 3 | 3 | 3 | 4 |
| CRELD1 | Q96HD1 | 1.17E-05 | 3.05E-06 | -0.954072 | | 1.21818 | -2.30709 | | 14.5293 | 14.7322 | 14.6592 | 15.9552 | 14.8708 | 15.4933 | 16.5783 | 15.0741 | 6 | 5 | 7 | 7 | 6 | 8 | 9 | 6 |
| KIAA0754 | O94854 | 1.05E-19 | 6.42E-19 | -0.954003 | + | 3.76729 | -8.25497 | | 13.5526 | 13.7685 | 13.6592 | 14.8723 | 14.4791 | 14.4177 | 14.6713 | 14.6302 | 4 | 6 | 7 | 8 | 6 | 6 | 8 | 7 |
| LDLRAP1 | Q5SW96 | 0.00024 | 5.40E-05 | -0.9521 | + | 2.05708 | -4.16653 | | 13.7209 | 13.3869 | 14.2436 | NaN | 14.8124 | 14.5973 | 14.9213 | 14.6126 | 2 | 2 | 1 | NaN | 2 | 3 | 2 | 2 |
| FAM180A | Q6UWF9 | 1.21E-09 | 4.98E-10 | -0.950849 | | 0.850255 | -1.69417 | | 15.0161 | 14.7249 | 15.3183 | 16.775 | 16.0188 | 14.4912 | 15.8759 | 16.6921 | 3 | 3 | 4 | 4 | 4 | 2 | 3 | 4 |

| Gene names | UniProt IDs | Protein Group P-value | Protein Group Q-value | Log2 Difference NUP214 vs Controls (n=5) | p<0.05 | -log10 p-value | T-test Test statistic | NPC Localization | Log2 MS2_quantity_NUP214_1 | Log2 MS2_quantity_NUP214_2 | Log2 MS2_quantity_NUP214_3 | Log2 MS2_quantity_Control_1 | Log2 MS2_quantity_Control_2 | Log2 MS2_quantity_Control_3 | Log2 MS2_quantity_Control_4 | Log2 MS2_quantity_Control_5 | Number Peptides for Quantitation NUP214_1 | Number Peptides for Quantitation NUP214_2 | Number Peptides for Quantitation NUP214_3 | Number Peptides for Quantitation Control_1 | Number Peptides for Quantitation Control_2 | Number Peptides for Quantitation Control_3 | Number Peptides for Quantitation Control_4 | Number Peptides for Quantitation Control_5 |
|---|---|---|---|---|---|---|---|---|---|---|---|---|---|---|---|---|---|---|---|---|---|---|---|---|
| LOXL2 | Q9Y4K0 | 5.68E-14 | 5.79E-14 | -0.949201 | + | 1.6992 | -3.14308 | | 15.918 | 15.957 | 15.8735 | 16.7342 | 17.06 | 16.4657 | 17.6464 | 16.4207 | 18 | 19 | 18 | 19 | 21 | 20 | 21 | 18 |
| DSG1 | Q02413 | 1.34E-10 | 6.29E-11 | -0.946605 | + | 1.59987 | -2.9648 | | 14.6182 | 14.5152 | 14.5433 | 16.0561 | 15.3627 | 14.6627 | 15.6439 | 15.8024 | 5 | 5 | 5 | 10 | 5 | 5 | 6 | 5 |
| IGF2BP1 | Q9NZI8 | 6.89E-10 | 2.90E-10 | -0.945884 | | 1.07115 | -2.06142 | | 14.2071 | 14.2639 | 14.2484 | 15.1211 | 14.2039 | 14.8218 | 15.5221 | 16.2596 | 9 | 10 | 11 | 15 | 7 | 12 | 14 | 16 |
| POLR2I | P36954 | 1.13E-06 | 3.25E-07 | -0.944758 | + | 1.57906 | -2.92787 | | 15.0626 | 14.6046 | 15.5133 | 15.314 | 16.4974 | 15.9973 | 16.0152 | 16.2007 | 2 | 1 | 3 | 2 | 3 | 4 | 3 | 3 |
| ATP13A3 | Q9H7F0 | 6.96E-14 | 6.93E-14 | -0.944696 | + | 1.80638 | -3.33968 | | 14.1841 | 14.1697 | 14.3767 | 15.7809 | 15.3908 | 14.5139 | 15.03 | 15.2253 | 8 | 10 | 9 | 16 | 13 | 10 | 12 | 15 |
| STX3 | Q13277;Q1 | 7.82E-05 | 1.87E-05 | -0.938388 | + | 1.82247 | -3.36961 | | 14.1793 | 14.3164 | 13.9832 | 15.6661 | 15.2345 | 14.4442 | 15.2247 | 14.9206 | 4 | 4 | 2 | 3 | 3 | 2 | 3 | 3 |
| CALD1 | Q05682-5 | 0.000168 | 3.86E-05 | -0.935199 | + | 2.25109 | -4.21221 | | 17.3731 | 17.7182 | 17.8227 | 18.2054 | 18.2213 | 18.8394 | 18.8744 | 18.7256 | 3 | 4 | 4 | 3 | 3 | 4 | 4 | 4 |
| FMN2 | Q9NZ56 | 8.54E-18 | 2.80E-17 | -0.934178 | + | 3.41894 | -7.13707 | | 15.0988 | 15.1291 | 15.2246 | 16.3206 | 16.1412 | 15.7404 | 16.0598 | 16.163 | 14 | 18 | 18 | 21 | 16 | 19 | 17 | 19 |
| RAD23A | P54725;P5 | 4.42E-07 | 1.34E-07 | -0.933817 | + | 2.00589 | -3.71888 | | 16.1522 | 15.9028 | 16.5898 | 16.8401 | 16.7292 | 17.3301 | 17.5054 | 17.3389 | 4 | 6 | 5 | 6 | 6 | 6 | 6 | 6 |
| GEMIN6 | Q8WXD5 | 1.14E-12 | 8.16E-13 | -0.93299 | + | 3.11517 | -6.26631 | | 14.2716 | 13.9406 | 14.3451 | 14.8822 | 15.3569 | 15.03 | 15.0355 | 15.2892 | 3 | 2 | 3 | 2 | 2 | 3 | 3 | 3 |
| APMAP | Q9HDC9 | 8.67E-09 | 3.18E-09 | -0.931408 | + | 5.5514 | -16.8319 | | 18.0819 | 18.0641 | 18.0959 | 19.0095 | 19.1395 | 18.8867 | 19.0436 | 18.9808 | 16 | 16 | 16 | 17 | 16 | 16 | 17 | 17 |
| HECW2 | Q9P2P5 | 0.002865 | 0.000538 | -0.93056 | + | 1.90106 | -3.51736 | | 14.0897 | 14.2695 | 13.9898 | 14.983 | 15.0478 | 14.4502 | 15.6678 | 15.0857 | 3 | 4 | 4 | 7 | 3 | 4 | 6 | 4 |
| IFNGR1 | P15260 | 0.00038 | 8.32E-05 | -0.928527 | + | 2.29506 | -4.75782 | | 14.0071 | 13.9416 | 13.8768 | 14.9294 | 15.2121 | NaN | 14.9123 | 14.4277 | 2 | 2 | 2 | 3 | 2 | NaN | 3 | 3 |
| LTBP1 | Q14766;Q1 | 3.27E-07 | 1.01E-07 | -0.928255 | + | 1.33211 | -2.84679 | | 13.3319 | 13.902 | 13.4591 | 15.0425 | NaN | 14.2689 | NaN | 14.1664 | 3 | 5 | 3 | 12 | NaN | 8 | NaN | 6 |
| LIMK1 | P53667;P5 | 7.46E-12 | 4.45E-12 | -0.928013 | + | 1.70836 | -3.15971 | | 13.1909 | 13.7128 | 13.8076 | 14.9388 | 14.6023 | 14.553 | 14.6251 | 13.7731 | 6 | 6 | 7 | 6 | 5 | 8 | 5 | 5 |
| SIK3 | Q9Y2K2;Q9 | 0.003989 | 0.000724 | -0.922352 | + | 2.25881 | -7.2053 | | 14.5741 | 14.8178 | 14.7732 | NaN | 15.7566 | 15.5316 | NaN | NaN | 1 | 1 | 1 | NaN | 2 | 1 | NaN | NaN |
| TOM1L2 | Q6ZVM7 | 3.20E-14 | 3.49E-14 | -0.91393 | + | 2.88353 | -5.65948 | | 15.777 | 15.6432 | 15.8096 | 16.525 | 16.8877 | 16.2639 | 16.7375 | 16.8718 | 15 | 15 | 15 | 17 | 15 | 17 | 17 | 17 |
| HSPB7 | Q9UBY9 | 2.19E-10 | 1.00E-10 | -0.912948 | | 1.28727 | -2.4236 | | 17.8934 | 17.9032 | 17.9552 | 18.9047 | 18.2805 | 18.9179 | 18.2482 | 19.7998 | 9 | 9 | 9 | 9 | 9 | 9 | 9 | 9 |
| FTH1 | P02794 | 1.42E-14 | 1.73E-14 | -0.9114 | + | 1.69263 | -3.13117 | | 17.065 | 16.9005 | 17.3245 | 18.5326 | 17.6253 | 17.4558 | 18.0537 | 18.3728 | 12 | 12 | 11 | 11 | 11 | 11 | 11 | 12 |
| CLDN11 | O75508 | 4.87E-06 | 1.32E-06 | -0.908316 | | 0.366164 | -0.84528 | | 16.2446 | 16.0955 | 16.0067 | 17.9671 | 18.4434 | 17.5438 | 13.9045 | 17.2609 | 3 | 3 | 4 | 5 | 5 | 3 | 2 | 3 |
| CREB3L1 | Q96BA8 | 0.001164 | 0.000238 | -0.902957 | + | 2.83117 | -5.52858 | | 15.0744 | 15.1132 | 14.8841 | 15.7289 | 16.2948 | 15.6789 | 16.0902 | 15.8417 | 5 | 5 | 4 | 5 | 3 | 5 | 7 | 7 |
| ASB1 | Q9Y576 | 1.59E-06 | 4.51E-07 | -0.901094 | + | 2.77434 | -5.38894 | | 13.4361 | 13.5801 | 13.5543 | 14.8469 | 14.3925 | 14.1091 | 14.2847 | 14.4896 | 4 | 5 | 4 | 9 | 4 | 4 | 6 | 5 |
| EPS8L2 | Q9H6S3 | 9.05E-10 | 3.77E-10 | -0.898204 | + | 2.99587 | -5.94801 | | 15.6629 | 15.7537 | 15.8121 | 16.9717 | 16.7089 | 16.6669 | 16.2863 | 16.5718 | 12 | 16 | 14 | 19 | 13 | 19 | 15 | 17 |
| PPCDC | Q96CD2 | 0.000784 | 0.000164 | -0.88663 | | 1.13974 | -2.26947 | | 13.9186 | NaN | 13.9421 | 14.1759 | 15.1232 | 14.3294 | 15.1599 | 15.2964 | 3 | NaN | 1 | 3 | 3 | 1 | 3 | 3 |
| PRNP | P04156 | 3.45E-09 | 1.33E-09 | -0.883178 | | 1.03923 | -2.00835 | | 16.8428 | 16.8656 | 17.221 | 16.7813 | 18.1262 | 17.8934 | 18.7721 | 17.7254 | 2 | 2 | 2 | 2 | 2 | 2 | 2 | 2 |
| COMMD6 | Q7Z4G1;Q7 | 0.000214 | 4.84E-05 | -0.881303 | | 1.04482 | -2.01765 | | 14.6157 | 14.6311 | 15.0354 | 15.1195 | 14.7328 | 15.7383 | 16.1828 | 16.4368 | 2 | 2 | 3 | 3 | 3 | 2 | 2 | 3 |
| DGKQ | P52824 | 0.000498 | 0.000107 | -0.881107 | + | 1.82611 | -3.3764 | | 13.3677 | 13.0145 | 12.7355 | 14.1452 | 13.407 | 13.6309 | 14.1922 | 14.2263 | 2 | 3 | 3 | 2 | 3 | 2 | 3 | 2 |
| LYZ | P61626 | 0.005204 | 0.000927 | -0.8798 | | 0.346164 | -0.81784 | | 13.7336 | NaN | 14.2438 | 16.4857 | 14.7572 | 12.5866 | 15.2438 | 15.2691 | 2 | NaN | 3 | 3 | 3 | 2 | 3 | 3 |
| CNN1 | P51911 | 3.21E-10 | 1.41E-10 | -0.878073 | | 1.11323 | -2.1315 | | 16.3566 | 16.4092 | 16.3335 | 17.7791 | 16.2227 | 17.5111 | 16.8644 | 17.8451 | 9 | 10 | 10 | 10 | 9 | 10 | 10 | 10 |
| ALG3 | Q92685;Q9 | 0.002776 | 0.000523 | -0.876851 | + | 2.54618 | -4.85289 | | 15.7728 | 15.4793 | 15.7625 | 16.5384 | 16.4884 | 16.5277 | 16.9832 | 16.204 | 2 | 2 | 2 | 2 | 2 | 2 | 2 | 2 |
| HBA1 | P69905 | 4.55E-15 | 6.40E-15 | -0.876457 | + | 1.66662 | -3.08421 | | 15.8092 | 15.3002 | 15.9573 | 16.6964 | 16.7507 | 16.5694 | 15.8722 | 16.9381 | 5 | 5 | 5 | 4 | 5 | 4 | 5 | 5 |
| GAA | P10253 | 6.09E-12 | 3.70E-12 | -0.873785 | + | 2.0875 | -3.87953 | | 16.6639 | 16.6571 | 16.7967 | 17.0275 | 17.4077 | 17.7525 | 18.0001 | 17.7107 | 16 | 16 | 15 | 15 | 14 | 15 | 15 | 16 |
| RPS6KB2 | Q9UBS0 | 0.000444 | 9.63E-05 | -0.871003 | + | 2.07369 | -3.8521 | | 13.9887 | 13.6653 | 13.4158 | 14.5255 | 14.1985 | 14.8933 | 14.8809 | 14.3066 | 1 | 3 | 2 | 2 | 1 | 2 | 3 | 1 |
| ACSS3 | Q9H6R3 | 0.002601 | 0.000494 | -0.870827 | + | 2.58118 | -5.5433 | | 14.2355 | 14.288 | 14.0755 | NaN | 15.0933 | 14.8027 | 15.3989 | 14.9871 | 5 | 4 | 4 | NaN | 2 | 4 | 5 | 5 |
| PEX12 | O00623 | 0.014436 | 0.002436 | -0.865154 | | 0.717136 | -1.50846 | | 14.3042 | 14.7637 | NaN | 15.9768 | 16.3332 | 15.3113 | 14.5974 | 14.7768 | 3 | 3 | NaN | 3 | 3 | 2 | 1 | 2 |
| MAPK8 | P45983-3;I | 0.000672 | 0.000142 | -0.863593 | + | 2.42043 | -4.57309 | | 13.6935 | 14.1768 | 14.1827 | 14.643 | 14.9836 | 14.6235 | 15.2053 | 14.951 | 2 | 4 | 4 | 4 | 2 | 4 | 4 | 3 |
| IGSF8 | Q969P0;Q9 | 4.25E-10 | 1.84E-10 | -0.862146 | + | 2.02285 | -3.75199 | | 14.7845 | 14.6672 | 14.429 | 14.9717 | 15.9851 | 15.398 | 15.539 | 15.5513 | 3 | 2 | 2 | 4 | 4 | 3 | 4 | 4 |
| SRI | P30626;P3 | 2.83E-08 | 9.75E-09 | -0.861239 | + | 1.36808 | -2.56107 | | 17.4385 | 17.0548 | 17.5136 | 17.4052 | 18.333 | 17.9737 | 18.4494 | 18.8231 | 9 | 9 | 9 | 9 | 9 | 9 | 9 | 9 |
| SCARB1 | Q8WTV0;Q | 0.003545 | 0.00065 | -0.860354 | + | 1.53987 | -3.03701 | | 15.0457 | 14.7274 | 15.0161 | 15.3797 | NaN | 15.6948 | 15.6426 | 16.4434 | 2 | 2 | 2 | 2 | NaN | 1 | 1 | 1 |
| NAA40 | Q86UY6;Q8 | 7.52E-09 | 2.78E-09 | -0.858823 | + | 2.09801 | -3.90045 | | 15.5677 | 15.3625 | 15.5872 | 16.2127 | 16.0999 | 16.0703 | 16.5179 | 16.9224 | 3 | 2 | 3 | 3 | 1 | 3 | 2 | 2 |
| POLR3A | O14802 | 0.023438 | 0.003882 | -0.856698 | + | 1.40686 | -2.77383 | | 13.9398 | 13.956 | 14.2167 | NaN | 14.5155 | 14.9873 | 14.5002 | 15.5739 | 3 | 5 | 4 | NaN | 1 | 3 | 2 | 2 |
| CYB5B | O43169 | 2.00E-07 | 6.30E-08 | -0.85566 | + | 2.37915 | -4.48348 | | 17.7969 | 17.6565 | 18.0738 | 18.215 | 18.7473 | 18.8091 | 18.9579 | 18.761 | 4 | 4 | 4 | 4 | 4 | 4 | 4 | 4 |
| RBSN | Q9H1K0 | 0.003961 | 0.00072 | -0.85274 | | 1.26337 | -2.38321 | | 14.7355 | 14.6748 | 14.4716 | 15.2724 | 15.0609 | 15.1177 | 16.5051 | 15.444 | 4 | 4 | 5 | 4 | 3 | 4 | 2 | 3 |
| HMGA1 | P17096;P1 | 2.14E-09 | 8.44E-10 | -0.851751 | + | 2.09466 | -3.89378 | | 18.135 | 18.1462 | 18.1779 | 19.4452 | 19.0434 | 18.5906 | 18.6824 | 19.2626 | 7 | 7 | 7 | 8 | 7 | 7 | 7 | 7 |
| ZDHHC6 | Q9H6R6;Q9 | 0.004345 | 0.000784 | -0.850558 | + | 1.66306 | -3.0778 | | 13.6311 | 13.4895 | 13.314 | 14.2301 | 13.7926 | 15.0186 | 14.4319 | 14.1706 | 1 | 1 | 1 | 1 | 1 | 2 | 2 | 2 |
| TIMP3 | P35625 | 1.23E-09 | 5.04E-10 | -0.850546 | + | 1.67598 | -3.10109 | | 16.9127 | 16.9777 | 16.952 | 17.8095 | 17.4472 | 17.5774 | 18.5857 | 17.5702 | 9 | 8 | 9 | 9 | 8 | 9 | 9 | 9 |
| GPCPD1 | Q9NPB8 | 0.000503 | 0.000108 | -0.849825 | | 0.879087 | -1.88769 | | NaN | 15.0086 | 14.5398 | 15.7031 | NaN | 14.8118 | 16.1252 | 15.8561 | NaN | 3 | 2 | 5 | NaN | 4 | 2 | 1 |
| HGH1 | Q9BTY7 | 0.000202 | 4.59E-05 | -0.847163 | + | 1.87569 | -3.46936 | | 15.2457 | 14.9144 | 15.4983 | 15.844 | 16.2316 | 15.795 | 15.8476 | 16.615 | 6 | 5 | 6 | 7 | 7 | 7 | 6 | 7 |
| TOP2A | P11388;P1 | 1.63E-10 | 7.58E-11 | -0.846567 | + | 1.54723 | -2.87169 | | 14.2592 | 14.0618 | 14.0423 | 14.9588 | 15.0571 | 14.1504 | 15.3071 | 15.3648 | 11 | 9 | 12 | 15 | 13 | 9 | 18 | 21 |
| UBQLN4 | Q9NRR5 | 6.98E-06 | 1.86E-06 | -0.836698 | + | 2.38539 | -4.49695 | | 15.0818 | 14.6698 | 15.3635 | 15.5678 | 16.0062 | 15.8076 | 16.0078 | 15.9858 | 7 | 6 | 7 | 7 | 6 | 7 | 7 | 7 |
| POLR3F | Q9H1D9 | 0.001691 | 0.000337 | -0.835017 | + | 1.68321 | -3.11414 | | 14.0177 | 15.2165 | 14.6612 | 15.2392 | 15.4203 | 15.6203 | 15.4811 | 15.5732 | 2 | 3 | 2 | 1 | 2 | 3 | 1 | 2 |
| RPP14 | O95059 | 0.003261 | 0.000602 | -0.834041 | | 0.870495 | -1.72792 | | 13.7851 | 14.1027 | 13.9984 | 14.2209 | 15.3551 | 13.6687 | 15.3834 | 15.3523 | 3 | 4 | 3 | 3 | 2 | 1 | 2 | 2 |
| RFTN2 | Q52LD8 | 0.000385 | 8.42E-05 | -0.830248 | | 1.1673 | -2.79685 | | 13.6999 | 13.4755 | 13.6616 | NaN | NaN | 14.0628 | 14.8223 | NaN | 1 | 1 | 1 | NaN | NaN | 2 | 1 | NaN |
| ATP6V0D1 | P61421 | 8.24E-05 | 1.97E-05 | -0.830114 | + | 2.17465 | -4.05493 | | 17.7176 | 17.5456 | 18.1304 | 18.4531 | 18.667 | 18.2771 | 18.9637 | 18.779 | 8 | 9 | 9 | 9 | 9 | 9 | 8 | 9 |
| P3H2 | Q8IVL5 | 2.61E-07 | 8.13E-08 | -0.829913 | | 1.04458 | -2.0957 | | 13.3904 | 13.4425 | 13.3459 | 15.0474 | NaN | 14.214 | 14.2192 | 13.4107 | 9 | 6 | 10 | 12 | NaN | 10 | 9 | 6 |
| DNAJB4 | Q9UDY4 | 2.85E-13 | 2.40E-13 | -0.829836 | + | 1.4139 | -2.63969 | | 16.0524 | 16.035 | 15.9499 | 17.3746 | 17.0203 | 15.9746 | 16.8077 | 17.034 | 14 | 15 | 13 | 15 | 15 | 12 | 15 | 15 |
| CDK1 | P06493 | 1.01E-11 | 5.88E-12 | -0.827972 | + | 1.91271 | -3.53952 | | 15.2619 | 15.2889 | 15.1683 | 16.0551 | 16.1591 | 15.4112 | 16.3821 | 16.3309 | 11 | 8 | 10 | 12 | 11 | 10 | 14 | 13 |
| PLEKHM1 | Q9Y4G2 | 0.00984 | 0.00169 | -0.826682 | | 1.18554 | -2.25232 | | 13.7123 | 13.8674 | 13.7648 | 14.5485 | 14.2467 | 13.9943 | 15.6034 | 14.648 | 2 | 3 | 3 | 3 | 2 | 2 | 2 | 3 |
| OPHN1 | O60890 | 0.004207 | 0.000761 | -0.825653 | | 0.572111 | -1.24634 | | 15.1015 | 13.1889 | 14.8515 | 15.9156 | 14.184 | 15.4018 | NaN | 15.3237 | 3 | 2 | 2 | 4 | 1 | 2 | NaN | 3 |
| UHRF1 | Q96T88;Q9 | 6.33E-12 | 3.83E-12 | -0.824644 | | 0.898008 | -1.77372 | | 14.6174 | 14.6973 | 14.599 | 16.0027 | 15.3032 | 14.2016 | 15.6461 | 16.1592 | 7 | 8 | 8 | 11 | 7 | 4 | 12 | 10 |
| AMPH | P49418;P4 | 3.44E-07 | 1.06E-07 | -0.822208 | + | 2.17367 | -4.05291 | | 14.5375 | 14.6479 | 14.6781 | 14.8934 | 15.7739 | 15.4199 | 15.4934 | 15.6363 | 12 | 12 | 14 | 10 | 13 | 14 | 11 | 13 |
| AK6 | Q9Y3D8 | 0.000411 | 8.95E-05 | -0.820617 | + | 1.78713 | -3.30404 | | 15.2231 | 14.9212 | 15.6436 | 15.9277 | 16.2249 | 15.5807 | 16.3253 | 16.3577 | 4 | 4 | 4 | 4 | 3 | 3 | 4 | 4 |
| TPST1 | O60507 | 1.23E-07 | 3.93E-08 | -0.82034 | + | 3.52278 | -7.45617 | | 13.9463 | 14.0712 | 14.0603 | 14.8823 | 14.8649 | 15.0739 | 14.5761 | 14.8342 | 3 | 6 | 6 | 6 | 5 | 6 | 6 | 6 |
| ISCA2 | Q86U28 | 0.001254 | 0.000255 | -0.814904 | | 1.2862 | -2.42178 | | 14.5686 | 14.8049 | 14.6803 | 15.3752 | 16.3659 | 15.3332 | 14.8333 | 15.5899 | 3 | 2 | 3 | 3 | 2 | 2 | 2 | 3 |
| SGTA | O43765 | 1.51E-07 | 4.80E-08 | -0.814238 | + | 1.78821 | -3.30603 | | 16.9591 | 16.4542 | 17.4479 | 17.4727 | 17.9616 | 17.6288 | 17.7999 | 17.9767 | 10 | 9 | 11 | 11 | 11 | 11 | 10 | 11 |
| F2 | P00734 | 0.000185 | 4.23E-05 | -0.81236 | | 1.01812 | -1.97327 | | 14.1858 | 14.5993 | 14.564 | 15.6616 | 14.9632 | 15.3132 | 14.309 | 16.0631 | 2 | 2 | 3 | 4 | 3 | 5 | 3 | 4 |
| FGFR1 | P11362;P1 | 0.001776 | 0.000353 | -0.811949 | + | 1.5262 | -2.83476 | | 14.5221 | 14.3105 | 14.5951 | 15.4846 | 15.9433 | 14.6764 | 15.1041 | 15.231 | 5 | 6 | 5 | 6 | 2 | 5 | 4 | 6 |
| SERPINE2 | P07093 | 2.56E-24 | 7.58E-23 | -0.811576 | | 0.907452 | -1.78943 | | 17.9974 | 18.0069 | 17.9512 | 17.6253 | 18.4755 | 19.0438 | 19.4225 | 19.4166 | 20 | 18 | 21 | 19 | 21 | 21 | 21 | 21 |
| MFSD1 | Q9H3U5;Q | 8.37E-12 | 4.94E-12 | -0.809344 | + | 3.37864 | -7.01629 | | 16.5693 | 16.4985 | 16.5963 | 17.3775 | 17.266 | 17.3116 | 17.6802 | 17.1849 | 2 | 2 | 2 | 2 | 2 | 2 | 2 | 2 |
| BUD23 | O43709;O4 | 5.11E-09 | 1.93E-09 | -0.80647 | + | 1.73101 | -3.20095 | | 14.1323 | 14.2878 | 14.3052 | 15.138 | 15.6513 | 14.5669 | 15.1298 | 14.7551 | 3 | 2 | 3 | 2 | 2 | 3 | 4 | 3 |
| NCAPG2 | Q86XI2;Q8 | 0.001778 | 0.000353 | -0.804776 | | 0.959823 | -1.87646 | | 13.5718 | 13.4719 | 13.4656 | 14.7947 | 14.3589 | 13.0849 | 14.4358 | 14.8651 | 3 | 2 | 3 | 4 | 1 | 1 | 4 | 3 |
| POLR2L | P62875 | 0.000237 | 5.34E-05 | -0.80439 | | 0.783425 | -1.69844 | | 15.7902 | 15.7061 | 16.0724 | 16.9308 | NaN | NaN | 15.7631 | 17.288 | 1 | 2 | 2 | 2 | NaN | NaN | 2 | 2 |
| SDC2 | P34741 | 1.51E-05 | 3.90E-06 | -0.803477 | | 1.0137 | -1.96594 | | 12.7975 | 12.4149 | 12.8245 | 13.2621 | 12.7317 | 13.2403 | 14.5177 | 13.6604 | 2 | 2 | 4 | 2 | 1 | 2 | 4 | 3 |
| YIPF6 | Q96EC8 | 0.000328 | 7.25E-05 | -0.803218 | + | 1.96681 | -3.64314 | | 15.0033 | 14.9264 | 14.8136 | 15.1725 | 16.0458 | 15.6115 | 16.0528 | 15.7057 | 2 | 2 | 2 | 2 | 2 | 2 | 2 | 2 |
| SMTN | P53814-5;I | 9.70E-13 | 7.04E-13 | -0.802396 | | 1.29599 | -2.43836 | | 15.2589 | 15.1972 | 15.2395 | 16.4979 | 16.1852 | 15.3677 | 15.5441 | 16.5765 | 14 | 15 | 16 | 16 | 16 | 14 | 14 | 17 |
| ZNRD2 | O60232 | 1.41E-10 | 6.60E-11 | -0.802187 | | 1.13279 | -2.16413 | | 15.8069 | 15.2577 | 15.8769 | 15.8621 | 16.1674 | 16.2223 | 16.6518 | 17.3431 | 3 | 2 | 3 | 4 | 2 | 3 | 4 | 4 |
| GPR108 | Q9NPR9 | 0.01559 | 0.002619 | -0.801293 | | 1.00539 | -2.1429 | | 15.3096 | 15.3836 | 15.2588 | 15.7331 | NaN | NaN | 16.8628 | 15.7599 | 2 | 2 | 3 | 2 | NaN | NaN | 1 | 2 |
| PZP | P20742 | 9.62E-05 | 2.28E-05 | -0.801061 | | 0.482067 | -1.06091 | | 20.5515 | 20.6161 | 20.4854 | 21.1775 | 20.1021 | 20.8605 | 23.4796 | 21.1407 | 4 | 3 | 3 | 3 | 3 | 4 | 4 | 3 |

| Gene names | UniProt IDs | Protein Group P-value | Protein Group Q-value | Log2 Difference NUP214 vs Controls (n=5) | p<0.05 | -log10 p-value | T-test Test statistic | NPC Localization | Log2 MS2_quantity_NUP214_1 | Log2 MS2_quantity_NUP214_2 | Log2 MS2_quantity_NUP214_3 | Log2 MS2_quantity_Control_1 | Log2 MS2_quantity_Control_2 | Log2 MS2_quantity_Control_3 | Log2 MS2_quantity_Control_4 | Log2 MS2_quantity_Control_5 | Number Peptides for Quantitation NUP214_1 | Number Peptides for Quantitation NUP214_2 | Number Peptides for Quantitation NUP214_3 | Number Peptides for Quantitation Control_1 | Number Peptides for Quantitation Control_2 | Number Peptides for Quantitation Control_3 | Number Peptides for Quantitation Control_4 | Number Peptides for Quantitation Control_5 |
|---|---|---|---|---|---|---|---|---|---|---|---|---|---|---|---|---|---|---|---|---|---|---|---|---|
| KANK1 | Q14678;Q1 | 3.57E-17 | 9.69E-17 | -0.800217 | + | 2.43007 | -4.59415 | | 13.8112 | 13.8633 | 14.0866 | 14.5737 | 15.1418 | 14.504 | 14.8514 | 14.5321 | 7 | 10 | 6 | 11 | 9 | 11 | 10 | 9 |
| ATP8B1 | O43520 | 9.42E-12 | 5.50E-12 | -0.799324 | + | 1.32491 | -2.48745 | | 14.8309 | 14.6293 | 14.8335 | 16.2073 | 15.7585 | 14.8309 | 15.2473 | 15.7756 | 5 | 6 | 5 | 11 | 6 | 4 | 7 | 6 |
| USP11 | P51784 | 3.46E-06 | 9.52E-07 | -0.799132 | + | 4.3258 | -10.3637 | | 13.6786 | 13.7865 | 13.6811 | 14.578 | 14.4632 | 14.482 | 14.6849 | 14.3644 | 8 | 7 | 5 | 9 | 2 | 8 | 10 | 4 |
| STARD3NL | O95772;O9 | 1.61E-06 | 4.56E-07 | -0.796779 | + | 2.8771 | -5.64329 | | 15.208 | 15.5124 | 15.4022 | 15.9168 | 16.2353 | 16.1052 | 16.4872 | 16.1104 | 2 | 2 | 2 | 2 | 2 | 2 | 2 | 2 |
| COA6 | Q5JTJ3;Q5 | 1.11E-19 | 6.71E-19 | -0.795421 | | 1.08122 | -2.07819 | | 14.6863 | 14.3579 | 15.1661 | 15.5749 | 14.7851 | 15.1456 | 15.9968 | 16.1586 | 2 | 2 | 2 | 2 | 2 | 2 | 2 | 3 |
| CEMIP2 | Q9UHN6 | 5.24E-18 | 1.84E-17 | -0.794176 | + | 2.9557 | -5.84361 | | 14.2317 | 14.1684 | 14.1146 | 14.9886 | 15.2578 | 14.6273 | 14.9892 | 14.966 | 8 | 8 | 7 | 10 | 9 | 6 | 11 | 13 |
| USP28 | Q96RU2-2 | 3.49E-09 | 1.35E-09 | -0.793644 | + | 1.39034 | -2.59919 | | 15.0265 | 14.0813 | 14.0917 | 14.6618 | 15.0694 | 15.4144 | 15.5067 | 15.3151 | 6 | 4 | 6 | 4 | 3 | 4 | 4 | 3 |
| QPRT | Q15274 | 0.000264 | 5.90E-05 | -0.791893 | | 0.896032 | -1.77043 | | 15.1733 | 15.264 | 15.2055 | 14.7611 | 16.0717 | 16.2921 | 16.1283 | 16.7777 | 5 | 5 | 5 | 3 | 5 | 5 | 5 | 5 |
| CCN1 | O00622 | 2.45E-14 | 2.77E-14 | -0.790351 | + | 2.28406 | -4.28112 | | 15.6618 | 15.6006 | 15.6444 | 16.483 | 15.9554 | 16.3089 | 16.7254 | 16.657 | 11 | 11 | 11 | 11 | 11 | 12 | 11 | 12 |
| ENSA | O43768;O4 | 8.60E-09 | 3.15E-09 | -0.789147 | + | 1.67104 | -3.09218 | | 15.1282 | 14.9711 | 15.7511 | 16.2342 | 15.7404 | 15.7651 | 16.1582 | 16.4651 | 3 | 3 | 5 | 7 | 5 | 7 | 7 | 7 |
| CCDC115 | Q96NT0 | 0.003758 | 0.000686 | -0.786307 | + | 2.14779 | -4.00042 | | 15.156 | 15.1043 | 15.4468 | 16.006 | 16.2268 | 15.5044 | 16.2206 | 16.1521 | 2 | 2 | 2 | 2 | 2 | 2 | 2 | 2 |
| CHST3 | Q7LGC8 | 0.000203 | 4.61E-05 | -0.784822 | + | 1.8534 | -3.42744 | | 14.2066 | 14.2189 | 14.1077 | 14.6761 | 14.9328 | 14.6634 | 15.6024 | 14.9381 | 2 | 2 | 2 | 2 | 1 | 2 | 2 | 2 |
| HEG1 | Q9ULI3 | 3.77E-23 | 6.88E-22 | -0.784409 | + | 1.48764 | -2.76737 | | 16.8992 | 17.0586 | 17.1955 | 18.4182 | 17.5838 | 18.1071 | 17.2166 | 17.852 | 4 | 4 | 5 | 6 | 5 | 6 | 4 | 6 |
| NFASC | O94856-12 | 1.22E-11 | 7.00E-12 | -0.783437 | + | 2.32983 | -4.37784 | | 15.2627 | 15.1981 | 15.2195 | 15.9824 | 16.4123 | 15.647 | 15.8263 | 16.183 | 9 | 6 | 6 | 11 | 10 | 6 | 8 | 11 |
| SLC36A1 | Q7Z2H8;Q7 | 8.63E-11 | 4.19E-11 | -0.782483 | + | 1.68839 | -3.12352 | | 15.0705 | 14.9334 | 14.9914 | 15.7072 | 15.584 | 15.4896 | 16.5144 | 15.6094 | 1 | 1 | 1 | 1 | 1 | 1 | 2 | 1 |
| FAT4 | Q6V0I7;Q6 | 0.002449 | 0.000469 | -0.779411 | + | 2.2324 | -4.17346 | | 13.7015 | 13.9542 | 13.9351 | 14.3516 | 15.1207 | 14.7175 | 14.5048 | 14.5204 | 3 | 5 | 1 | 4 | 2 | 6 | 1 | 3 |
| BASP1 | P80723 | 1.58E-05 | 4.07E-06 | -0.778615 | + | 1.42918 | -2.66602 | | 16.8075 | 16.6037 | 17.3474 | 18.3376 | 17.451 | 17.8055 | 17.2782 | 17.6185 | 15 | 13 | 16 | 17 | 15 | 18 | 15 | 17 |
| SLC39A7 | Q92504 | 0.000909 | 0.000189 | -0.7782 | | 1.07465 | -2.06725 | | 13.3172 | 14.7305 | 14.6955 | 14.9549 | 14.8377 | 15.4905 | 14.8298 | 15.0168 | 2 | 1 | 2 | 2 | 1 | 2 | 1 | 2 |
| CDAN1 | Q8IWY9;Q8 | 1.27E-07 | 4.05E-08 | -0.777329 | + | 3.38021 | -7.02095 | | 14.1134 | 14.1375 | 14.1927 | 15.0582 | 14.8513 | 15.0138 | 14.6361 | 15.0667 | 4 | 4 | 3 | 2 | 3 | 3 | 1 | 3 |
| EPB41L3 | Q9Y2J2-2 | 1.47E-13 | 1.33E-13 | -0.77582 | | 1.06002 | -2.04292 | | 16.0666 | 16.0674 | 16.0645 | 17.5199 | 16.6907 | 17.4573 | 16.0815 | 16.4419 | 38 | 37 | 39 | 41 | 39 | 40 | 37 | 37 |
| PHYKPL | Q8IUZ5 | 0.002918 | 0.000546 | -0.775635 | + | 2.0681 | -3.84102 | | 13.8277 | 14.058 | 13.9636 | 14.3015 | 15.1901 | 14.7748 | 14.8022 | 14.5584 | 2 | 3 | 5 | 3 | 2 | 4 | 4 | 2 |
| STK17A | Q9UEE5 | 0.000185 | 4.22E-05 | -0.774383 | + | 1.80801 | -3.34272 | | 14.4727 | 14.5852 | 14.3506 | 15.0087 | 15.6411 | 14.7039 | 15.4242 | 15.4415 | 2 | 3 | 3 | 3 | 3 | 2 | 3 | 3 |
| TMEM41B | Q5BJD5 | 6.71E-05 | 1.62E-05 | -0.774124 | + | 2.2327 | -4.17407 | | 15.5759 | 15.5645 | 15.5397 | 16.7769 | 16.2154 | 16.0691 | 16.0765 | 16.5328 | 3 | 4 | 3 | 4 | 3 | 3 | 4 | 2 |
| MKLN1 | Q9UL63 | 0.00014 | 3.25E-05 | -0.770196 | + | 1.56046 | -2.895 | | 13.6444 | 14.2032 | 14.166 | 14.8197 | 14.9878 | 14.0965 | 15.0368 | 14.9328 | 6 | 7 | 6 | 11 | 7 | 2 | 7 | 8 |
| C8orf33 | Q9H7E9;Q9 | 7.80E-06 | 2.07E-06 | -0.769125 | | 0.647486 | -1.38323 | | NaN | 15.0339 | 13.5255 | 15.1809 | 15.6047 | 15.3269 | 14.8821 | 14.2494 | NaN | 2 | 2 | 3 | 3 | 3 | 2 | 3 |
| COL6A2 | P12110-2 | 3.87E-05 | 9.58E-06 | -0.765533 | + | 2.068 | -3.84082 | | 16.954 | 16.8701 | 16.8686 | 17.9941 | 17.9603 | 17.2546 | 17.3902 | 17.7163 | 2 | 2 | 2 | 2 | 2 | 2 | 2 | 2 |
| SLC17A5 | Q9NRA2 | 3.83E-08 | 1.30E-08 | -0.764888 | + | 2.08614 | -3.87682 | | 16.1861 | 16.1521 | 16.1072 | 16.6508 | 16.6342 | 16.9548 | 17.4472 | 16.8798 | 5 | 5 | 4 | 5 | 4 | 5 | 5 | 5 |
| MASP1 | P48740-2 | 5.98E-13 | 4.57E-13 | -0.764728 | | 0.207761 | -0.5229 | | 18.4535 | 12.4233 | 13.4533 | 15.2639 | 16.6259 | 15.7057 | 14.1893 | 15.9223 | 1 | 2 | 2 | 4 | 7 | 5 | 2 | 8 |
| LRRC8D | Q7L1W4 | 0.002356 | 0.000453 | -0.764109 | + | 2.0443 | -3.79407 | | 12.9411 | 13.3274 | 12.9124 | 14.1804 | 13.9404 | 13.4175 | 13.9349 | 13.6488 | 2 | 4 | 4 | 2 | 2 | 3 | 2 | 2 |
| CD9 | P21926 | 0.000107 | 2.52E-05 | -0.76268 | | 0.752435 | -1.53023 | | 16.8227 | 16.7161 | 16.5934 | 18.7883 | 16.6403 | 16.8996 | 17.443 | 17.5958 | 4 | 4 | 4 | 5 | 5 | 5 | 5 | 5 |
| PGRMC1 | O00264 | 6.54E-07 | 1.94E-07 | -0.761707 | + | 2.03239 | -3.77067 | | 18.6113 | 18.1988 | 19.0792 | 19.1964 | 19.3541 | 19.3744 | 19.5061 | 19.5262 | 10 | 10 | 10 | 10 | 10 | 10 | 10 | 10 |
| NT5E | P21589 | 3.55E-13 | 2.91E-13 | -0.76049 | + | 2.9106 | -5.72804 | | 18.7192 | 18.7422 | 18.6948 | 19.6576 | 19.5526 | 19.3257 | 19.1736 | 19.6867 | 24 | 26 | 25 | 26 | 26 | 24 | 27 | 26 |
| DPH2 | Q9BQC3;Q9 | 0.027172 | 0.00448 | -0.75944 | | 1.06745 | -2.05527 | | 14.6198 | 14.6885 | 14.3695 | 15.2417 | 14.504 | 15.5675 | 16.1605 | 15.12 | 2 | 2 | 2 | 1 | 2 | 3 | 2 | 2 |
| YIPF5 | Q969M3;Q | 0.001268 | 0.000258 | -0.757914 | + | 2.77115 | -5.38119 | | 16.6483 | 17.0942 | 17.0283 | 17.5748 | 17.62 | 17.859 | 17.8509 | 17.5028 | 2 | 2 | 2 | 2 | 2 | 2 | 2 | 2 |
| NUDT2 | P50583 | 2.97E-18 | 1.12E-17 | -0.757356 | + | 1.60201 | -2.96859 | | 15.6578 | 15.4007 | 15.7098 | 16.4791 | 15.992 | 15.8351 | 16.677 | 16.7508 | 7 | 8 | 8 | 8 | 5 | 7 | 8 | 8 |
| CPNE7 | Q9UBL6;Q9 | 5.96E-09 | 2.23E-09 | -0.75514 | | 0.984052 | -1.98629 | | 13.5951 | 13.4932 | 13.4715 | 14.7613 | 13.9742 | 13.5169 | 14.8481 | NaN | 4 | 6 | 4 | 7 | 5 | 4 | 9 | NaN |
| PYCARD | Q9ULZ3 | 4.57E-09 | 1.74E-09 | -0.754722 | | 0.905525 | -1.78622 | | 15.6749 | 14.9951 | 16.0113 | 16.3044 | 15.9886 | 15.6277 | 16.3984 | 17.2565 | 5 | 5 | 7 | 8 | 5 | 6 | 7 | 9 |
| LAGE3 | Q14657 | 0.001378 | 0.000278 | -0.753118 | | 1.24199 | -2.34715 | | 13.7553 | 13.8588 | 14.0594 | 14.2856 | 15.0423 | 14.0357 | 14.5473 | 15.3103 | 1 | 1 | 1 | 1 | 1 | 2 | 2 | 3 |
| SLC20A2 | Q08357 | 2.54E-12 | 1.67E-12 | -0.75278 | + | 2.41517 | -4.5616 | | 14.8699 | 14.9601 | 14.7966 | 15.8296 | 15.3685 | 15.2978 | 15.8271 | 15.8186 | 4 | 5 | 4 | 4 | 3 | 5 | 4 | 4 |
| POM121C | A8CG34 | 0.037157 | 0.006073 | -0.752378 | + | 3.55142 | -9.01184 | | 14.0852 | 14.0576 | 14.0788 | NaN | 14.9783 | 14.6409 | 14.8685 | 14.8173 | 2 | 6 | 4 | NaN | 2 | 5 | 5 | 4 |
| PFKFB3 | Q16875;Q1 | 0.002039 | 0.000399 | -0.749836 | + | 1.30477 | -2.45326 | | 13.0436 | 13.068 | 13.2667 | 14.1977 | 14.2774 | 13.0261 | 13.8252 | 14.0533 | 2 | 3 | 2 | 3 | 1 | 2 | 2 | 1 |
| APLP2 | Q06481;Q0 | 8.40E-12 | 4.95E-12 | -0.748926 | | 0.772409 | -1.56385 | | 14.6712 | 14.7378 | 14.779 | 16.5845 | 15.0098 | 14.7996 | 14.9201 | 16.0772 | 15 | 15 | 16 | 20 | 14 | 15 | 16 | 17 |
| FLNC | Q14315 | 1.96E-12 | 1.32E-12 | -0.746757 | + | 1.6639 | -3.07931 | | 19.973 | 20.0171 | 19.9774 | 21.2862 | 20.7524 | 20.3392 | 20.3464 | 20.9554 | 154 | 152 | 154 | 157 | 155 | 153 | 152 | 157 |
| PXDC1 | Q5TGL8 | 8.86E-14 | 8.58E-14 | -0.746275 | + | 2.26038 | -4.23158 | | 14.6454 | 14.9957 | 14.9644 | 15.2511 | 15.4757 | 15.9381 | 15.6687 | 15.7402 | 4 | 4 | 4 | 4 | 4 | 4 | 3 | 4 |
| ATF6B | Q99941;Q9 | 5.18E-10 | 2.22E-10 | -0.744651 | + | 1.42657 | -2.66151 | | 13.8131 | 13.5892 | 13.956 | 14.3409 | 14.8566 | 13.8643 | 15.0023 | 14.5897 | 3 | 3 | 5 | 5 | 4 | 2 | 3 | 3 |
| HDAC4 | P56524 | 0.010645 | 0.001821 | -0.744643 | | 0.876682 | -2.04943 | | 13.7222 | 13.1261 | 13.8886 | NaN | NaN | 14.6009 | NaN | 14.0463 | 4 | 4 | 2 | NaN | NaN | 2 | NaN | 2 |
| ENTPD4 | Q9Y227;Q9 | 0.005045 | 0.000901 | -0.742246 | + | 3.42672 | -7.16059 | | 14.2193 | 14.3935 | 14.6037 | 15.1007 | 15.3347 | 15.1342 | 15.0579 | 15.1112 | 4 | 2 | 3 | 2 | 4 | 3 | 3 | 2 |
| QSOX1 | O00391 | 4.53E-16 | 8.46E-16 | -0.741708 | + | 3.8039 | -8.38062 | | 14.9378 | 15.0456 | 14.9764 | 15.7084 | 15.8118 | 15.5423 | 15.9174 | 15.6616 | 10 | 10 | 11 | 15 | 9 | 13 | 15 | 13 |
| DOHH | Q9BU89 | 2.53E-12 | 1.66E-12 | -0.741615 | | 1.24096 | -2.34543 | | 15.616 | 15.0241 | 15.8537 | 15.6401 | 16.4022 | 15.9368 | 16.5433 | 16.6755 | 7 | 6 | 7 | 6 | 7 | 6 | 7 | 8 |
| IVNS1ABP | Q9Y6Y0 | 7.95E-05 | 1.90E-05 | -0.740687 | + | 2.05495 | -3.81505 | | 13.5341 | 13.5972 | 13.3856 | 13.7482 | 14.1926 | 14.5161 | 14.5232 | 14.2514 | 3 | 3 | 2 | 3 | 2 | 3 | 4 | 3 |
| CACNB3 | P54284;P5 | 4.26E-07 | 1.29E-07 | -0.740482 | | 1.0373 | -2.00515 | | 13.6483 | 13.3754 | 13.1247 | 15.0393 | 13.9022 | 13.6673 | 13.6346 | 14.3729 | 2 | 1 | 2 | 4 | 2 | 2 | 3 | 2 |
| C1QTNF5 | Q9BXJ0 | 0.000678 | 0.000143 | -0.740361 | + | 2.21814 | -4.14401 | | 14.792 | 15.135 | 14.7265 | 15.6081 | 15.7244 | 15.8515 | 15.1932 | 15.747 | 3 | 3 | 3 | 3 | 2 | 3 | 2 | 2 |
| TAMM41 | Q96BW9-3 | 4.60E-14 | 4.81E-14 | -0.738857 | + | 2.95767 | -5.84871 | | 13.4372 | 13.3517 | 13.4834 | 13.9113 | 14.2372 | 14.1351 | 14.0657 | 14.4654 | 4 | 3 | 5 | 3 | 3 | 5 | 3 | 2 |
| SFT2D3 | Q587I9 | 0.000337 | 7.42E-05 | -0.738709 | + | 2.10398 | -4.27903 | | 14.4166 | 14.7226 | 14.309 | 15.5409 | NaN | 14.9839 | 15.153 | 15.208 | 1 | 1 | 2 | 2 | NaN | 2 | 2 | 2 |
| CHAF1B | Q13112 | 5.14E-11 | 2.61E-11 | -0.734834 | | 0.579531 | -1.23409 | | 13.4153 | 14.0287 | 13.5469 | 13.8626 | 15.1579 | 12.9647 | 14.7502 | 15.2569 | 1 | 1 | 2 | 1 | 2 | 1 | 1 | 3 |
| FAM177A1 | Q8N128;Q8 | 2.17E-13 | 1.89E-13 | -0.732392 | + | 2.73237 | -5.28744 | | 15.9233 | 15.6472 | 15.9502 | 16.7482 | 16.585 | 16.2815 | 16.763 | 16.4855 | 5 | 5 | 5 | 6 | 5 | 5 | 6 | 5 |
| ANXA5 | P08758 | 2.96E-11 | 1.57E-11 | -0.731257 | + | 2.63286 | -5.05208 | | 22.5639 | 22.511 | 22.5081 | 23.429 | 23.5342 | 23.3053 | 22.9587 | 23.0673 | 26 | 26 | 27 | 26 | 26 | 26 | 27 | 27 |
| ZCCHC17 | Q9NP64;Q9 | 5.04E-05 | 1.24E-05 | -0.729495 | + | 2.2675 | -4.24644 | | 13.0195 | 13.7628 | 13.3686 | 13.9374 | 14.0958 | 14.1567 | 14.2625 | 14.1133 | 2 | 1 | 2 | 2 | 2 | 2 | 2 | 2 |
| SLC2A6 | Q9UGQ3;Q | 1.07E-06 | 3.09E-07 | -0.728273 | + | 1.48646 | -2.7653 | | 14.2954 | 14.2188 | 14.4031 | 15.7476 | 14.8577 | 14.6548 | 14.7725 | 15.1376 | 4 | 4 | 3 | 4 | 4 | 3 | 3 | 4 |
| LRRC8C | Q8TDW0 | 2.25E-05 | 5.72E-06 | -0.728144 | + | 2.61538 | -5.64326 | | 14.431 | 14.392 | 14.6832 | 15.4153 | 15.3256 | NaN | 15.019 | 15.1609 | 7 | 8 | 8 | 11 | 4 | NaN | 6 | 9 |
| TNRC6A | Q8NDV7;Q | 0.002552 | 0.000486 | -0.727219 | + | 1.95148 | -3.61364 | | 13.8269 | 13.6309 | 13.8622 | 14.8562 | 14.7247 | 14.2332 | 14.5969 | 14.0918 | 3 | 4 | 3 | 3 | 3 | 3 | 3 | 5 |
| RC3H1 | Q5TC82 | 0.000403 | 8.78E-05 | -0.726975 | + | 2.44981 | -4.63752 | | 14.227 | 14.6366 | 14.3132 | 14.885 | 15.4349 | 15.0782 | 14.9847 | 15.2134 | 2 | 2 | 4 | 2 | 2 | 3 | 4 | 2 |
| DHFR | P00374 | 0.002014 | 0.000395 | -0.726722 | | 1.10384 | -2.11586 | | 14.5988 | 14.6975 | 14.6206 | 15.6143 | 15.6478 | 14.4339 | 15.2258 | 15.9066 | 4 | 5 | 4 | 6 | 4 | 1 | 4 | 7 |
| SQOR | Q9Y6N5 | 4.22E-13 | 3.38E-13 | -0.723534 | + | 1.83216 | -3.38768 | | 16.7612 | 16.7997 | 16.7978 | 17.1696 | 18.1001 | 17.3472 | 17.5602 | 17.3719 | 26 | 27 | 25 | 27 | 28 | 28 | 28 | 27 |
| SLC29A1 | Q99808;Q9 | 0.000626 | 0.000133 | -0.723213 | | 0.436744 | -0.97808 | | 12.7414 | 13.3282 | 13.3704 | 15.938 | 12.8008 | 13.4089 | 13.3494 | 13.8523 | 1 | 2 | 2 | 2 | 1 | 1 | 1 | 1 |
| CHPF | Q8IZ52 | 0.000577 | 0.000123 | -0.720864 | + | 2.76982 | -5.37795 | | 14.535 | 14.5981 | 14.7166 | 15.2128 | 15.3494 | 15.2657 | 15.701 | 15.1583 | 7 | 6 | 7 | 9 | 9 | 8 | 11 | 8 |
| ATP11A | P98196 | 4.16E-05 | 1.03E-05 | -0.719066 | + | 2.83309 | -5.53333 | | 13.97 | 13.9898 | 13.9404 | 14.7571 | 15.007 | 14.6366 | 14.4164 | 14.6118 | 2 | 2 | 3 | 2 | 3 | 2 | 2 | 2 |
| TMBIM6 | P55061;P5 | 3.67E-05 | 9.10E-06 | -0.717595 | + | 4.32429 | -10.3574 | | 16.0259 | 16.0084 | 16.0444 | 16.7109 | 16.6793 | 16.686 | 16.9493 | 16.6935 | 3 | 3 | 3 | 3 | 3 | 3 | 3 | 3 |
| CNPY2 | Q9Y2B0 | 3.48E-08 | 1.19E-08 | -0.717176 | + | 1.44268 | -2.68934 | | 17.2446 | 16.818 | 17.5931 | 17.6113 | 17.6546 | 18.2042 | 18.4088 | 17.7997 | 12 | 12 | 12 | 12 | 12 | 12 | 12 | 12 |
| NDUFAF5 | Q5TEU4;Q5 | 7.82E-05 | 1.87E-05 | -0.716114 | + | 2.43359 | -4.60187 | | 15.3566 | 14.9715 | 15.453 | 16.0015 | 16.1953 | 15.6971 | 15.9064 | 16.0822 | 1 | 2 | 1 | 2 | 2 | 2 | 2 | 2 |
| OSBPL10 | Q9BXB5 | 0.002221 | 0.000431 | -0.714555 | + | 2.24792 | -4.20563 | | 13.706 | 13.9488 | 14.3166 | 14.4211 | 14.7951 | 14.8738 | 14.813 | 14.622 | 1 | 1 | 1 | 2 | 1 | 1 | 1 | 1 |
| RGS19 | P49795 | 0.011904 | 0.002024 | -0.713838 | + | 4.29095 | -10.2193 | | 15.1708 | 15.2114 | 15.21 | 15.9859 | 16.0452 | 15.7721 | 15.9418 | 15.8111 | 2 | 2 | 2 | 2 | 2 | 2 | 2 | 2 |
| CAPG | P40121 | 3.40E-14 | 3.68E-14 | -0.713813 | + | 2.24736 | -4.20448 | | 17.7362 | 17.7964 | 17.863 | 18.7317 | 18.4504 | 18.8712 | 18.2203 | 18.2883 | 14 | 12 | 13 | 13 | 14 | 14 | 12 | 15 |
| SH2D4A | Q9H788 | 1.79E-15 | 2.86E-15 | -0.71293 | + | 1.88569 | -3.48825 | | 14.6494 | 14.8201 | 14.661 | 15.3712 | 15.0743 | 15.9454 | 15.2088 | 15.5159 | 17 | 17 | 16 | 17 | 12 | 19 | 16 | 18 |
| COMMD8 | Q9NX08 | 0.000304 | 6.75E-05 | -0.71213 | | 0.871785 | -1.73006 | | 14.2248 | 13.8562 | 15.5822 | 15.2678 | 15.3816 | 14.9267 | 15.1545 | 15.6019 | 5 | 5 | 6 | 5 | 5 | 5 | 5 | 6 |
| FADD | Q13158 | 0.004259 | 0.00077 | -0.711204 | + | 1.86846 | -3.45574 | | 14.5344 | 14.5812 | 15.0093 | 15.3993 | 15.5811 | 14.9592 | 15.4206 | 15.7374 | 3 | 3 | 3 | 3 | 3 | 2 | 2 | 3 |
| ISG20L2 | Q9H9L3 | 0.000374 | 8.20E-05 | -0.71084 | + | 3.3185 | -6.83913 | | 14.2301 | 13.9334 | 14.0077 | 14.8334 | 14.6262 | 14.8533 | 14.9085 | 14.6182 | 2 | 3 | 3 | 2 | 2 | 3 | 2 | 2 |
| NMT2 | O60551 | 1.75E-15 | 2.81E-15 | -0.710472 | + | 3.58956 | -7.66756 | | 15.1333 | 15.0948 | 15.0685 | 16.0341 | 15.7381 | 15.6144 | 15.8278 | 15.8324 | 9 | 7 | 7 | 9 | 8 | 9 | 8 | 8 |

| Gene names | UniProt IDs | Protein Group P-value | Protein Group Q-value | Log2 Difference NUP214 vs Controls (n=5) | p<0.05 | -log10 p-value | T-test Test statistic | NPC Localization | Log2 MS2_quantity_NUP214_1 | Log2 MS2_quantity_NUP214_2 | Log2 MS2_quantity_NUP214_3 | Log2 MS2_quantity_Control_1 | Log2 MS2_quantity_Control_2 | Log2 MS2_quantity_Control_3 | Log2 MS2_quantity_Control_4 | Log2 MS2_quantity_Control_5 | Number Peptides for Quantitation NUP214_1 | Number Peptides for Quantitation NUP214_2 | Number Peptides for Quantitation NUP214_3 | Number Peptides for Quantitation Control_1 | Number Peptides for Quantitation Control_2 | Number Peptides for Quantitation Control_3 | Number Peptides for Quantitation Control_4 | Number Peptides for Quantitation Control_5 |
|---|---|---|---|---|---|---|---|---|---|---|---|---|---|---|---|---|---|---|---|---|---|---|---|---|
| CNST | Q6PJW8 | 4.92E-18 | 1.76E-17 | -0.70921 | + | 2.30234 | -4.31958 | | 14.5836 | 14.5311 | 14.6958 | 15.3146 | 15.6573 | 14.9025 | 15.3487 | 15.3405 | 6 | 6 | 7 | 7 | 9 | 6 | 7 | 8 |
| AKT1S1 | Q96B36 | 1.14E-05 | 2.98E-06 | -0.708665 | + | 2.83603 | -5.54062 | | 16.7808 | 16.467 | 16.9904 | 17.5121 | 17.6011 | 17.3926 | 17.4416 | 17.3264 | 4 | 4 | 5 | 5 | 4 | 4 | 5 | 5 |
| PAM | P19021 | 1.85E-15 | 2.94E-15 | -0.708559 | | 1.28105 | -2.41309 | | 15.1102 | 15.1502 | 15.1459 | 15.5898 | 15.408 | 15.9244 | 15.6379 | 16.6597 | 14 | 14 | 14 | 16 | 14 | 17 | 16 | 17 |
| FBLIM1 | Q8WUP2 | 1.17E-07 | 3.76E-08 | -0.707613 | + | 3.02961 | -6.03677 | | 16.6534 | 16.7278 | 16.672 | 17.5264 | 17.3365 | 17.502 | 17.5217 | 17.0734 | 10 | 11 | 12 | 12 | 9 | 13 | 11 | 12 |
| TMEM175 | Q9BSA9 | 0.000774 | 0.000162 | -0.706783 | + | 2.24517 | -4.19991 | | 13.4743 | 13.4077 | 13.693 | 13.9184 | 14.3085 | 14.1606 | 14.628 | 14.1432 | 3 | 3 | 3 | 4 | 4 | 3 | 4 | 3 |
| GLI2 | P10070;P1 | 0.001155 | 0.000236 | -0.706128 | + | 1.66648 | -3.6614 | | NaN | 14.6245 | 14.2849 | 14.9501 | NaN | 14.9986 | 15.3325 | 15.3621 | NaN | 3 | 1 | 3 | NaN | 2 | 2 | 1 |
| RUFY3 | Q7L099;Q7 | 0.000102 | 2.41E-05 | -0.70249 | + | 2.66403 | -5.12501 | | 15.0944 | 14.9791 | 15.0245 | 15.6383 | 15.8971 | 15.8533 | 15.9064 | 15.3806 | 3 | 6 | 4 | 6 | 5 | 6 | 6 | 4 |
| COPZ1 | P61923 | 4.08E-08 | 1.37E-08 | -0.700176 | + | 1.64716 | -3.04923 | | 18.2983 | 17.967 | 18.4146 | 18.6276 | 18.7769 | 18.6524 | 19.1537 | 19.4233 | 7 | 7 | 7 | 7 | 7 | 7 | 7 | 7 |
| OSTM1 | Q86WC4 | 0.001603 | 0.000321 | -0.698474 | | 0.896203 | -1.77072 | | 15.536 | 15.6063 | 14.5998 | 15.5135 | 15.2775 | 16.2681 | 16.5333 | 16.137 | 2 | 2 | 2 | 3 | 1 | 2 | 3 | 3 |
| MAP3K20 | Q9NYL2 | 0.000376 | 8.24E-05 | -0.696505 | + | 1.78531 | -3.30066 | | 13.3174 | 13.1853 | 13.3149 | 14.329 | 14.0915 | 13.4282 | 13.8389 | 14.1575 | 4 | 6 | 3 | 7 | 4 | 4 | 6 | 4 |
| CDC42EP2 | O14613 | 0.00173 | 0.000344 | -0.696369 | + | 2.2389 | -4.18692 | | 17.0153 | 16.8189 | 17.1794 | 17.7809 | 17.7178 | 17.8823 | 17.2721 | 17.8513 | 3 | 3 | 3 | 3 | 3 | 3 | 3 | 3 |
| STAMBPL1 | Q96FJ0;Q9 | 0.001905 | 0.000375 | -0.696186 | + | 1.65926 | -3.07097 | | 14.6092 | 14.9692 | 15.2611 | 16.1566 | 15.5128 | 15.3629 | 15.5807 | 15.6005 | 3 | 4 | 5 | 5 | 5 | 5 | 5 | 3 |
| A2M | P01023 | 0.0002 | 4.55E-05 | -0.696009 | | 0.426831 | -0.95972 | | 20.6433 | 20.616 | 20.6564 | 21.5246 | 19.9195 | 20.8987 | 23.2393 | 21.091 | 4 | 4 | 4 | 5 | 5 | 6 | 8 | 8 |
| STEAP1 | Q9UHE8 | 0.00606 | 0.001069 | -0.695451 | | 0.609838 | -1.28685 | | 12.8693 | 13.2512 | 13.4953 | 15.0188 | 12.6767 | 13.7268 | 13.674 | 14.4073 | 3 | 3 | 3 | 5 | 3 | 2 | 2 | 4 |
| TAF4 | O00268 | 0.012491 | 0.002119 | -0.695022 | | 0.829369 | -1.70911 | | 13.6222 | 13.8075 | 13.641 | 14.0616 | 15.3774 | 14.2562 | NaN | 13.8458 | 2 | 1 | 2 | 1 | 1 | 1 | NaN | 1 |
| DLGAP5 | Q15398;Q1 | 0.001861 | 0.000367 | -0.694883 | + | 1.86556 | -3.45028 | | 14.1286 | 13.7778 | 13.9192 | 14.8356 | 14.2657 | 14.4962 | 14.5209 | 15.0653 | 2 | 2 | 1 | 4 | 2 | 1 | 4 | 6 |
| BAIAP2L1 | Q9UHR4 | 0.004162 | 0.000754 | -0.694241 | + | 1.78693 | -3.30366 | | 13.2541 | 13.3829 | 12.9577 | 13.9581 | 14.2503 | 13.4247 | 14.0721 | 13.7571 | 1 | 2 | 1 | 4 | 4 | 3 | 4 | 4 |
| NDC80 | O14777 | 0.000229 | 5.16E-05 | -0.692851 | | 1.0031 | -1.94834 | | 14.7049 | 14.4695 | 14.5655 | 15.0142 | 16.2534 | 14.6957 | 15.0937 | 15.3072 | 11 | 11 | 9 | 11 | 5 | 6 | 8 | 7 |
| PIKFYVE | Q9Y2I7 | 7.25E-05 | 1.74E-05 | -0.691979 | + | 1.87116 | -3.46083 | | 13.987 | 13.2998 | 13.8669 | 14.3924 | 14.5639 | 14.4338 | 14.5969 | 14.0625 | 4 | 3 | 3 | 4 | 4 | 2 | 4 | 4 |
| TOR3A | Q9H497;Q | 0.000421 | 9.15E-05 | -0.691622 | + | 2.28474 | -4.28254 | | 14.2467 | 13.8305 | 14.4 | 14.6078 | 14.9252 | 14.8017 | 15.0802 | 14.8386 | 2 | 2 | 2 | 4 | 1 | 3 | 3 | 2 |
| TPBG | Q13641 | 1.21E-05 | 3.14E-06 | -0.689886 | + | 2.02116 | -3.74868 | | 17.0079 | 17.0567 | 17.1501 | 18.1433 | 17.6741 | 17.3746 | 17.9854 | 17.6298 | 7 | 5 | 6 | 7 | 6 | 8 | 8 | 7 |
| NEPRO | Q6NW34 | 0.001701 | 0.000339 | -0.689491 | + | 1.93118 | -3.57473 | | 15.4613 | 15.3922 | 15.4957 | 15.9294 | 16.5781 | 16.3879 | 15.9153 | 15.8854 | 4 | 2 | 5 | 4 | 3 | 2 | 4 | 2 |
| SERPINB7 | O75635;O | 0.000715 | 0.000151 | -0.688892 | | 1.15436 | -2.20015 | | 13.8585 | 13.8593 | 13.5809 | 13.9766 | 15.2148 | 14.0766 | 14.7244 | 14.2832 | 2 | 1 | 2 | 2 | 2 | 3 | 2 | 3 |
| PCBD1 | P61457 | 1.47E-06 | 4.19E-07 | -0.687724 | + | 1.67305 | -3.0958 | | 15.575 | 15.4355 | 15.9713 | 16.3637 | 15.8157 | 16.4074 | 16.6415 | 16.5133 | 4 | 4 | 4 | 5 | 5 | 4 | 4 | 4 |
| CIT | O14578 | 4.57E-05 | 1.13E-05 | -0.687246 | + | 2.01703 | -3.74062 | | 14.7247 | 14.7663 | 14.8316 | 15.1255 | 15.6527 | 15.1462 | 15.593 | 15.7898 | 15 | 13 | 13 | 18 | 18 | 5 | 13 | 20 |
| UFM1 | P61960 | 0.002014 | 0.000395 | -0.687174 | + | 2.39259 | -4.51252 | | 18.5073 | 18.2851 | 18.515 | 18.8608 | 19.3115 | 18.8899 | 19.3792 | 19.1736 | 3 | 2 | 4 | 4 | 2 | 4 | 4 | 4 |
| SLC52A2 | Q9HAB3 | 0.000519 | 0.000111 | -0.686233 | + | 2.07822 | -3.86108 | | 16.0656 | 15.5463 | 15.6507 | 16.1697 | 16.3586 | 16.3631 | 16.7695 | 16.5413 | 2 | 2 | 2 | 2 | 2 | 2 | 2 | 2 |
| LAMTOR3 | Q9UHA4;Q | 3.59E-08 | 1.22E-08 | -0.686144 | + | 2.54288 | -4.84541 | | 17.0203 | 17.1269 | 17.3877 | 17.6887 | 17.7402 | 17.7866 | 18.1767 | 17.9299 | 5 | 5 | 5 | 5 | 4 | 5 | 5 | 4 |
| TXNDC15 | Q96J42 | 2.33E-05 | 5.91E-06 | -0.684109 | + | 1.45313 | -2.70742 | | 15.2308 | 14.9546 | 15.0608 | 15.8744 | 15.9192 | 15.0865 | 16.1931 | 15.7576 | 5 | 4 | 4 | 4 | 4 | 4 | 5 | 5 |
| NPC2 | P61916 | 0.000174 | 3.99E-05 | -0.683012 | + | 1.88591 | -3.48867 | | 17.6101 | 17.5574 | 17.6987 | 18.0898 | 18.2766 | 18.0016 | 18.8356 | 18.3216 | 7 | 8 | 8 | 7 | 9 | 7 | 9 | 9 |
| LMBRD2 | Q68DH5 | 0.000635 | 0.000135 | -0.68196 | + | 2.25346 | -4.21715 | | 14.21 | 14.2284 | 14.0787 | 14.4504 | 14.7904 | 14.8617 | 15.1459 | 15.0233 | 2 | 2 | 2 | 3 | 1 | 2 | 5 | 2 |
| PUM2 | Q8TB72;Q8 | 7.83E-05 | 1.88E-05 | -0.681399 | + | 1.57799 | -2.92598 | | 15.0649 | 14.2347 | 14.8722 | 15.4339 | 15.7485 | 15.0707 | 15.3621 | 15.4115 | 3 | 4 | 4 | 4 | 2 | 5 | 4 | 3 |
| DDA1 | Q9BW61 | 0.000689 | 0.000145 | -0.680553 | | 1.12034 | -2.14336 | | 13.489 | 13.3047 | 13.5066 | 13.8445 | 13.8495 | 13.5414 | 14.5525 | 14.7822 | 2 | 1 | 2 | 2 | 2 | 2 | 2 | 2 |
| CEP170 | Q5SW79-2 | 0.000178 | 4.08E-05 | -0.679918 | | 0.892741 | -1.76496 | | 12.4635 | 12.2484 | 12.2719 | 13.9398 | 12.8984 | 13.0609 | 12.137 | 13.0033 | 1 | 1 | 3 | 3 | 1 | 1 | 1 | 2 |
| UBE2G2 | P60604;P6 | 1.38E-06 | 3.94E-07 | -0.677526 | + | 1.84662 | -3.41472 | | 15.3093 | 15.0587 | 15.4652 | 15.8384 | 15.7083 | 15.6773 | 16.2546 | 16.2976 | 4 | 4 | 4 | 4 | 3 | 4 | 3 | 4 |
| DCTPP1 | Q9H773 | 0.002953 | 0.000552 | -0.674935 | | 0.986271 | -1.92038 | | 14.8923 | 14.3015 | 14.8496 | 14.8552 | 15.1248 | 14.9479 | 15.766 | 16.0865 | 3 | 2 | 3 | 3 | 4 | 4 | 4 | 4 |
| HERC4 | Q5GLZ8;Q5 | 8.95E-20 | 5.55E-19 | -0.673774 | + | 1.62996 | -3.01843 | | 16.3312 | 16.3624 | 16.3634 | 16.607 | 17.5117 | 16.691 | 17.1632 | 17.1577 | 43 | 43 | 44 | 42 | 45 | 43 | 46 | 43 |
| SIPA1L1 | O43166-2; | 5.01E-15 | 6.92E-15 | -0.67275 | + | 1.48638 | -2.76517 | | 14.3058 | 14.2562 | 14.3007 | 15.6666 | 14.8678 | 14.625 | 14.7674 | 14.8748 | 21 | 21 | 19 | 32 | 16 | 25 | 25 | 25 |
| SYNPO | Q8N3V7;Q | 0.002051 | 0.000401 | -0.672324 | | 1.10029 | -2.10993 | | 14.4885 | 14.2483 | 14.1149 | 14.7065 | 14.4149 | 15.5495 | 15.4696 | 14.6405 | 1 | 3 | 3 | 2 | 2 | 4 | 1 | 1 |
| UBAP2 | Q5T6F2 | 2.97E-09 | 1.16E-09 | -0.671933 | + | 1.94333 | -3.598 | | 15.0339 | 15.0269 | 15.0703 | 15.9263 | 15.8431 | 15.1619 | 15.8032 | 15.8437 | 6 | 6 | 7 | 7 | 5 | 5 | 6 | 7 |
| ABT1 | Q9ULW3 | 0.000657 | 0.000139 | -0.668036 | | 0.725282 | -1.48438 | | 14.6207 | 15.1677 | 15.28 | 16.8587 | 15.0397 | 15.2937 | 15.4348 | 15.8273 | 2 | 1 | 3 | 2 | 1 | 1 | 2 | 2 |
| TXN2 | Q99757 | 2.42E-05 | 6.12E-06 | -0.667637 | + | 1.39622 | -2.60928 | | 15.2712 | 14.9941 | 15.4046 | 15.5599 | 15.6565 | 15.5857 | 16.2488 | 16.4038 | 5 | 3 | 5 | 4 | 5 | 5 | 4 | 5 |
| MORF4L2 | Q15014 | 0.002487 | 0.000475 | -0.667619 | + | 1.65314 | -3.05997 | | 13.3224 | 13.5655 | 13.7918 | 13.896 | 14.2393 | 14.0148 | 14.747 | 14.2405 | 2 | 2 | 3 | 4 | 3 | 1 | 3 | 3 |
| B4GALT4 | O60513 | 3.50E-11 | 1.83E-11 | -0.667587 | | 0.798168 | -1.60709 | | 13.5395 | 13.6304 | 13.6796 | 14.891 | 13.7887 | 14.4229 | 14.9549 | 13.3632 | 2 | 2 | 2 | 3 | 3 | 3 | 4 | 3 |
| GSTM4 | Q03013;Q | 3.37E-09 | 1.30E-09 | -0.666913 | | 0.672363 | -1.39442 | | 16.668 | 16.5739 | 16.7587 | 16.1295 | 17.9235 | 17.7797 | 16.896 | 17.9401 | 7 | 9 | 9 | 6 | 9 | 9 | 7 | 10 |
| KCNMA1 | Q12791-2; | 5.21E-06 | 1.41E-06 | -0.665893 | + | 2.9589 | -5.85188 | | 15.3548 | 15.3425 | 15.3803 | 15.7169 | 16.0938 | 16.1214 | 16.2099 | 15.9834 | 9 | 8 | 7 | 9 | 10 | 11 | 12 | 14 |
| MAPRE3 | Q9UPY8 | 0.000413 | 8.99E-05 | -0.665365 | + | 3.83815 | -8.49965 | | 16.062 | 16.0017 | 15.8022 | 16.5648 | 16.68 | 16.4922 | 16.7052 | 16.6613 | 7 | 8 | 8 | 10 | 8 | 9 | 8 | 9 |
| BOLA2 | Q9H3K6 | 2.85E-06 | 7.90E-07 | -0.664908 | + | 1.44258 | -2.68916 | | 17.6025 | 16.9271 | 17.565 | 17.6883 | 17.9799 | 17.7942 | 18.2238 | 18.4627 | 4 | 4 | 4 | 4 | 4 | 4 | 4 | 4 |
| TPM1 | P09493-5 | 0.000754 | 0.000158 | -0.663318 | + | 1.62452 | -3.00871 | | 16.6241 | 16.6555 | 16.5732 | 17.4021 | 17.4445 | 17.7425 | 16.8358 | 16.9797 | 2 | 2 | 2 | 2 | 2 | 2 | 2 | 2 |
| CEP55 | Q53EZ4 | 0.002738 | 0.000517 | -0.662118 | + | 1.31721 | -2.47437 | | 14.1007 | 14.5642 | 13.8942 | 15.0787 | 14.2793 | 14.7155 | 14.9054 | 15.2634 | 5 | 5 | 6 | 9 | 4 | 4 | 6 | 7 |
| PSMD10 | O75832 | 8.83E-15 | 1.13E-14 | -0.656956 | + | 1.41284 | -2.63787 | | 15.0356 | 14.8239 | 15.2928 | 15.2488 | 15.9177 | 15.3347 | 16.0054 | 16.032 | 8 | 9 | 11 | 9 | 8 | 10 | 10 | 9 |
| CARD6 | Q9BX69 | 6.78E-05 | 1.64E-05 | -0.655282 | | 0.690115 | -1.45998 | | 13.0491 | 12.9394 | 13.046 | 14.2929 | 12.8073 | 14.3131 | NaN | 13.2538 | 1 | 1 | 2 | 2 | 2 | 2 | NaN | 1 |
| COPZ2 | Q9P299 | 0.000125 | 2.92E-05 | -0.655264 | + | 2.07755 | -3.85975 | | 17.3168 | 17.243 | 17.6304 | 17.8237 | 17.8868 | 18.1978 | 18.4085 | 17.9431 | 7 | 7 | 7 | 7 | 6 | 7 | 7 | 7 |
| PISD | Q9UG56;Q | 0.000358 | 7.86E-05 | -0.655184 | + | 1.7138 | -3.1696 | | 16.0986 | 16.174 | 16.1776 | 17.2629 | 16.4964 | 16.4251 | 16.9038 | 16.938 | 3 | 3 | 3 | 3 | 4 | 4 | 4 | 4 |
| GYG1 | P46976;P4 | 9.06E-10 | 3.77E-10 | -0.655126 | + | 2.25576 | -4.22194 | | 16.4231 | 16.4942 | 16.3752 | 17.5231 | 17.0209 | 16.8757 | 17.0794 | 16.9308 | 7 | 8 | 8 | 10 | 8 | 8 | 10 | 10 |
| ERCC1 | P07992 | 0.00049 | 0.000106 | -0.654628 | | 0.625888 | -1.31461 | | 13.6994 | 11.8714 | 13.8305 | 13.8163 | 13.6178 | 13.3591 | 13.9942 | 14.1545 | 3 | 2 | 2 | 2 | 3 | 2 | 2 | 3 |
| UAP1 | Q16222;Q1 | 5.46E-17 | 1.39E-16 | -0.654325 | + | 1.65314 | -3.05996 | | 17.6294 | 17.5666 | 17.5484 | 18.7219 | 18.2101 | 17.7329 | 18.1571 | 18.357 | 22 | 22 | 21 | 25 | 23 | 24 | 24 | 23 |
| TTC39C | Q8N584 | 5.62E-20 | 3.77E-19 | -0.653932 | + | 4.06981 | -9.34431 | | 14.3753 | 14.4535 | 14.3868 | 15.0486 | 15.0776 | 14.9138 | 15.2291 | 15.0265 | 8 | 9 | 8 | 11 | 9 | 8 | 7 | 10 |
| PRIM2 | P49643 | 9.92E-07 | 2.88E-07 | -0.653126 | | 1.06245 | -2.04696 | | 14.1752 | 14.131 | 14.0198 | 15.0714 | 14.9579 | 13.8201 | 14.8891 | 15.0705 | 3 | 3 | 4 | 6 | 4 | 3 | 6 | 5 |
| CTSB | P07858 | 8.13E-12 | 4.81E-12 | -0.652823 | + | 1.6334 | -3.02458 | | 19.7184 | 19.7499 | 19.7174 | 19.8723 | 20.3124 | 20.3571 | 20.8794 | 20.4856 | 17 | 17 | 17 | 17 | 17 | 17 | 17 | 18 |
| SUMF2 | Q8NBJ7 | 1.36E-07 | 4.34E-08 | -0.648396 | + | 1.77004 | -3.27252 | | 17.3558 | 17.2869 | 17.3108 | 17.9147 | 17.5371 | 18.4386 | 18.0913 | 17.8495 | 10 | 10 | 10 | 10 | 10 | 10 | 11 | 10 |
| FBLN1 | P23142 | 3.23E-15 | 4.76E-15 | -0.648117 | | 0.828731 | -1.65823 | | 16.3424 | 16.4149 | 16.3394 | 17.5921 | 17.0555 | 17.4764 | 15.9345 | 17.0101 | 8 | 10 | 9 | 13 | 9 | 11 | 8 | 11 |
| TFB2M | Q9H5Q4 | 0.001595 | 0.00032 | -0.647992 | + | 2.69342 | -5.19445 | | 13.6786 | 13.7842 | 13.7415 | 14.226 | 14.4869 | 14.168 | 14.355 | 14.6779 | 6 | 5 | 6 | 7 | 3 | 5 | 6 | 5 |
| DDX58 | O95786 | 0.000984 | 0.000203 | -0.647829 | + | 2.46057 | -4.66126 | | 14.278 | 14.0671 | 14.0579 | 14.8621 | 14.8966 | 14.4264 | 14.9795 | 14.7461 | 6 | 9 | 9 | 13 | 7 | 4 | 5 | 7 |
| IGF2R | P11717 | 3.62E-17 | 9.75E-17 | -0.647683 | | 1.06156 | -2.04547 | | 15.9936 | 15.9709 | 15.9677 | 17.2844 | 16.786 | 15.8223 | 16.7223 | 16.5105 | 67 | 70 | 68 | 70 | 63 | 63 | 69 | 66 |
| TMEM159 | Q96B96;Q | 0.002885 | 0.000541 | -0.647322 | | 0.492825 | -1.09989 | | 14.857 | 15.352 | 15.2445 | 14.8725 | NaN | 17.164 | 15.4928 | 15.6647 | 2 | 2 | 2 | 1 | NaN | 1 | 1 | 2 |
| CAV2 | P51636 | 7.83E-07 | 2.31E-07 | -0.645684 | + | 1.5353 | -2.85072 | | 16.8723 | 16.9682 | 17.2464 | 17.2288 | 17.7158 | 17.7325 | 18.1928 | 17.5032 | 5 | 5 | 5 | 5 | 5 | 5 | 5 | 5 |
| HINT2 | Q9BX68 | 0.000113 | 2.65E-05 | -0.64555 | | 0.595822 | -1.2625 | | 15.5749 | 14.6463 | 15.473 | 14.5891 | 15.8516 | 15.9611 | 16.4678 | 16.5151 | 5 | 5 | 5 | 5 | 5 | 5 | 5 | 5 |
| TYW1 | Q9NV66 | 0.001318 | 0.000267 | -0.645495 | + | 2.38554 | -4.49727 | | 13.3635 | 13.1881 | 13.2796 | 13.6664 | 13.9654 | 13.6969 | 14.114 | 14.1702 | 1 | 2 | 2 | 2 | 2 | 1 | 2 | 1 |
| MARCKSL1 | P49006 | 3.49E-06 | 9.60E-07 | -0.645489 | | 1.02789 | -1.9895 | | 15.2845 | 15.1203 | 15.6741 | 16.4023 | 16.3578 | 15.7163 | 15.2499 | 16.2992 | 2 | 2 | 3 | 3 | 3 | 3 | 3 | 3 |
| P2RX4 | Q99571;Q | 5.38E-06 | 1.45E-06 | -0.644871 | + | 2.49085 | -4.72848 | | 14.6853 | 14.7158 | 14.6942 | 15.2415 | 15.5788 | 15.3172 | 15.5507 | 15.0283 | 7 | 6 | 7 | 5 | 6 | 6 | 7 | 7 |
| MOXD1 | Q6UVY6 | 5.36E-13 | 4.15E-13 | -0.644525 | + | 2.58558 | -4.94277 | | 16.58 | 16.62 | 16.6743 | 17.4506 | 17.3043 | 17.1933 | 16.9373 | 17.4609 | 16 | 15 | 16 | 16 | 15 | 16 | 15 | 16 |
| MGAT5 | Q09328 | 0.051808 | 0.008406 | -0.644135 | | 0.788402 | -1.70822 | | 13.1349 | 13.075 | 12.8613 | 13.7208 | 14.2768 | 13.006 | NaN | NaN | 1 | 2 | 1 | 2 | 1 | 1 | NaN | NaN |
| DPY19L3 | Q6ZPD9 | 0.002187 | 0.000425 | -0.644003 | + | 2.85666 | -5.59204 | | 14.4552 | 14.3124 | 14.6023 | 15.0889 | 14.8696 | 15.2163 | 15.0372 | 15.2911 | 2 | 2 | 2 | 2 | 1 | 2 | 2 | 2 |
| DHPS | P49366 | 4.34E-08 | 1.46E-08 | -0.64259 | | 1.15551 | -2.20208 | | 14.4875 | 14.2081 | 14.2602 | 15.344 | 15.2891 | 14.4867 | 14.3921 | 15.294 | 4 | 6 | 5 | 7 | 3 | 3 | 5 | 5 |
| HSPA1L | P34931 | 1.77E-09 | 7.06E-10 | -0.642247 | + | 1.9119 | -3.53796 | | 19.0646 | 18.9631 | 18.8216 | 19.424 | 20.0571 | 19.295 | 19.5281 | 19.6558 | 4 | 5 | 5 | 5 | 4 | 5 | 5 | 5 |
| WIPF2 | Q8TF74 | 3.65E-05 | 9.07E-06 | -0.640301 | + | 1.83985 | -3.40205 | | 13.9002 | 13.863 | 13.8262 | 14.1004 | 14.5835 | 14.5371 | 14.3448 | 14.9513 | 6 | 5 | 7 | 6 | 6 | 7 | 7 | 9 |
| INTS12 | Q96CB8 | 0.006312 | 0.001111 | -0.640253 | | 0.375378 | -0.8951 | | 15.1401 | 15.2408 | 14.9741 | 15.0113 | NaN | 17.1801 | NaN | 15.0844 | 2 | 2 | 2 | 1 | NaN | 1 | NaN | 1 |

| Gene names | UniProt IDs | Protein Group P-value | Protein Group Q-value | Log2 Difference NUP214 vs Controls (n=5) | p<0.05 | -log10 p-value | T-test Test statistic | NPC Localization | Log2 MS2_quantity_NUP214_1 | Log2 MS2_quantity_NUP214_2 | Log2 MS2_quantity_NUP214_3 | Log2 MS2_quantity_Control_1 | Log2 MS2_quantity_Control_2 | Log2 MS2_quantity_Control_3 | Log2 MS2_quantity_Control_4 | Log2 MS2_quantity_Control_5 | Number Peptides for Quantitation NUP214_1 | Number Peptides for Quantitation NUP214_2 | Number Peptides for Quantitation NUP214_3 | Number Peptides for Quantitation Control_1 | Number Peptides for Quantitation Control_2 | Number Peptides for Quantitation Control_3 | Number Peptides for Quantitation Control_4 | Number Peptides for Quantitation Control_5 |
|---|---|---|---|---|---|---|---|---|---|---|---|---|---|---|---|---|---|---|---|---|---|---|---|---|
| AVL9 | Q8NBF6;Q8 | 0.000824 | 0.000172 | -0.639792 | + | 1.69997 | -3.14448 | | 14.6786 | 14.6062 | 14.56 | 15.405 | 15.6833 | 14.776 | 15.1096 | 15.2996 | 5 | 5 | 5 | 7 | 4 | 5 | 5 | 5 |
| PPP2R5B | Q15173;Q1 | 0.002566 | 0.000489 | -0.63947 | + | 2.04164 | -3.78883 | | 15.3148 | 15.541 | 15.6005 | 16.0071 | 16.5524 | 15.8642 | 16.1684 | 16.0324 | 2 | 3 | 5 | 5 | 4 | 4 | 5 | 4 |
| RB1 | P06400 | 7.93E-14 | 7.77E-14 | -0.639209 | + | 1.59173 | -2.95034 | | 14.0969 | 14.1607 | 14.0903 | 14.9193 | 14.5873 | 14.2001 | 14.9811 | 15.0881 | 6 | 8 | 7 | 8 | 4 | 5 | 6 | 8 |
| FOSL1 | P15407 | 5.82E-16 | 1.05E-15 | -0.639057 | | 0.543794 | -1.17127 | | 15.3945 | 15.4521 | 15.6007 | 16.6462 | 15.8656 | 14.6268 | 16.7368 | 16.7321 | 3 | 2 | 3 | 3 | 3 | 1 | 3 | 3 |
| CAPNS1 | P04632 | 3.36E-13 | 2.77E-13 | -0.63802 | + | 2.56135 | -4.88737 | | 19.692 | 19.5015 | 19.8181 | 20.0758 | 20.5842 | 20.34 | 20.3304 | 20.2123 | 11 | 11 | 12 | 13 | 12 | 12 | 13 | 13 |
| PTPN21 | Q16825 | 0.004834 | 0.000866 | -0.637786 | + | 1.55594 | -2.88704 | | 16.5208 | 16.327 | 16.3026 | 17.1532 | 17.3055 | 16.4229 | 16.9581 | 17.2667 | 3 | 3 | 3 | 3 | 2 | 4 | 2 | 3 |
| ARPP19 | P56211;P5 | 0.000754 | 0.000158 | -0.63765 | | 0.417534 | -0.94243 | | 15.2254 | 15.0265 | 15.6999 | 16.7342 | 14.8797 | 14.7838 | 16.0967 | 17.28 | 3 | 3 | 4 | 3 | 3 | 3 | 4 | 3 |
| RTL8C | A6ZKI3 | 0.000492 | 0.000106 | -0.636436 | + | 1.63418 | -3.02598 | | 15.9127 | 15.5718 | 15.9121 | 16.5856 | 16.4855 | 15.9923 | 16.2655 | 16.8478 | 5 | 6 | 5 | 5 | 5 | 4 | 7 | 6 |
| POLR1F | Q3B726 | 0.001248 | 0.000254 | -0.636436 | + | 1.69632 | -3.13786 | | 14.467 | 14.3618 | 14.3521 | 15.0402 | 15.4705 | 14.5593 | 14.9009 | 15.1794 | 2 | 2 | 2 | 2 | 2 | 2 | 2 | 3 |
| TRPC4AP | Q8TEL6;Q8 | 0.008278 | 0.001435 | -0.636325 | + | 1.31603 | -2.59908 | | 14.3949 | 14.3757 | NaN | 15.3632 | 14.6091 | 14.7454 | 15.1475 | 15.243 | 1 | 2 | NaN | 2 | 2 | 2 | 2 | 2 |
| SAMD9L | Q8IVG5 | 3.35E-05 | 8.36E-06 | -0.635749 | | 1.284 | -2.41807 | | 14.2362 | 14.1628 | 13.898 | 14.3291 | 14.3601 | 15.2191 | 15.1387 | 14.6266 | 5 | 4 | 1 | 3 | 3 | 5 | 5 | 2 |
| KCT2 | Q8NC54 | 0.000535 | 0.000115 | -0.635671 | + | 2.45911 | -4.65802 | | 15.5335 | 15.4014 | 15.6722 | 16.1605 | 16.0659 | 15.9583 | 16.1602 | 16.512 | 2 | 2 | 2 | 2 | 2 | 2 | 2 | 2 |
| TMCO4 | Q5TGY1;Q5 | 2.40E-07 | 7.50E-08 | -0.634251 | + | 1.38739 | -2.59413 | | 15.1445 | 14.7248 | 15.0228 | 15.6429 | 15.0393 | 15.5733 | 15.6244 | 16.1116 | 2 | 2 | 2 | 3 | 2 | 2 | 3 | 2 |
| CEP131 | Q9UPN4;Q | 2.15E-10 | 9.79E-11 | -0.634063 | + | 1.54583 | -2.86924 | | 14.0243 | 13.8215 | 14.028 | 14.7826 | 14.6653 | 14.0806 | 14.4095 | 15.022 | 3 | 3 | 3 | 3 | 3 | 1 | 2 | 3 |
| RAI14 | Q9P0K7-4 | 3.16E-13 | 2.63E-13 | -0.633175 | + | 4.13076 | -9.57851 | | 16.0686 | 16.0792 | 16.0721 | 16.835 | 16.7388 | 16.7597 | 16.5456 | 16.6533 | 43 | 45 | 43 | 48 | 44 | 45 | 45 | 46 |
| RABIF | P47224 | 0.001542 | 0.000309 | -0.632911 | + | 2.1939 | -4.09422 | | 16.6043 | 16.5055 | 16.786 | 17.2526 | 17.551 | 16.8931 | 17.2727 | 17.3547 | 2 | 2 | 3 | 2 | 2 | 3 | 2 | 2 |
| NMRAL1 | Q9HBL8 | 8.77E-06 | 2.32E-06 | -0.632096 | + | 1.53994 | -2.85887 | | 14.3068 | 14.3197 | 14.2626 | 14.2845 | 15.0016 | 15.0104 | 15.2036 | 15.1423 | 5 | 6 | 4 | 6 | 5 | 8 | 8 | 6 |
| UBQLN2 | Q9UHD9 | 4.40E-20 | 3.16E-19 | -0.631606 | + | 3.01258 | -5.99185 | | 15.9028 | 15.6381 | 15.9823 | 16.4142 | 16.4225 | 16.5494 | 16.3342 | 16.6431 | 6 | 5 | 7 | 6 | 6 | 7 | 5 | 6 |
| WWC2 | Q6AWC2 | 0.000901 | 0.000187 | -0.630127 | + | 4.40387 | -10.694 | | 14.4196 | 14.3295 | 14.471 | 15.033 | 14.9708 | 15.1577 | 14.9461 | 15.0767 | 6 | 2 | 3 | 8 | 5 | 4 | 6 | 5 |
| GINM1 | Q9NU53 | 0.000696 | 0.000147 | -0.628479 | | 1.11743 | -2.22851 | | 13.3402 | 13.5142 | 14.2731 | 14.5397 | 14.0987 | NaN | 14.5693 | 14.1429 | 3 | 2 | 1 | 3 | 1 | NaN | 2 | 2 |
| C6orf47 | O95873 | 0.003055 | 0.000568 | -0.627479 | | 0.820401 | -1.64431 | | 15.2341 | 15.2459 | 15.5129 | 16.9496 | 15.2457 | 16.0849 | 15.765 | 15.7469 | 3 | 2 | 3 | 2 | 1 | 3 | 2 | 3 |
| ATP7A | Q04656;Q0 | 0.006694 | 0.001175 | -0.627474 | | 1.23914 | -2.34235 | | 13.3932 | 13.4572 | 12.4913 | 13.4239 | 13.9158 | 13.9294 | 13.8865 | 13.5513 | 3 | 2 | 2 | 2 | 1 | 3 | 5 | 2 |
| GMFG | O60234 | 0.001615 | 0.000323 | -0.627426 | + | 2.5769 | -4.92289 | | 16.6783 | 16.4991 | 16.793 | 17.4688 | 17.2342 | 16.9897 | 17.4015 | 17.327 | 2 | 2 | 2 | 3 | 2 | 2 | 3 | 2 |
| KPNA2 | P52292 | 1.97E-18 | 7.86E-18 | -0.626642 | | 1.14394 | -2.18275 | | 17.1296 | 17.081 | 17.0778 | 17.8985 | 18.005 | 16.9131 | 17.6779 | 18.1195 | 14 | 14 | 15 | 15 | 14 | 14 | 15 | 15 |
| AGFG2 | O95081 | 0.005526 | 0.000979 | -0.626024 | + | 1.41264 | -2.63752 | | 15.0746 | 15.1092 | 14.9943 | 16.1657 | 15.6298 | 15.4392 | 15.1983 | 15.9939 | 3 | 3 | 2 | 3 | 2 | 3 | 3 | 3 |
| LARP4 | Q71RC2;Q7 | 2.67E-16 | 5.42E-16 | -0.625976 | + | 2.03988 | -3.78539 | | 15.2436 | 15.3187 | 15.1937 | 16.1034 | 16.0721 | 15.4983 | 15.676 | 16.0401 | 12 | 12 | 11 | 15 | 11 | 10 | 13 | 14 |
| UCK2 | Q9BZX2 | 7.61E-13 | 5.66E-13 | -0.62567 | + | 2.14697 | -3.99876 | | 15.5832 | 15.48 | 15.496 | 16.0542 | 16.3009 | 15.9075 | 15.9449 | 16.5195 | 5 | 6 | 6 | 7 | 8 | 6 | 7 | 7 |
| WNT5A | P41221;P4 | 4.25E-11 | 2.18E-11 | -0.624777 | | 0.379987 | -0.8717 | | 14.4481 | 14.3401 | 14.4726 | 13.9869 | 14.3667 | 15.8079 | 16.7835 | 14.2801 | 7 | 7 | 8 | 8 | 8 | 9 | 9 | 5 |
| SLC4A7 | Q9Y6M7-1 | 1.95E-10 | 8.96E-11 | -0.624708 | + | 1.30624 | -2.45574 | | 13.6803 | 13.5958 | 13.5955 | 13.8287 | 14.1153 | 14.5063 | 14.8541 | 13.9385 | 8 | 6 | 8 | 9 | 7 | 11 | 11 | 9 |
| ATG4A | Q8WYN0;Q | 0.015495 | 0.002605 | -0.62316 | | 0.853182 | -1.83615 | | 13.0497 | 12.8093 | 13.0978 | 13.9042 | NaN | 12.9549 | NaN | 13.9671 | 2 | 2 | 2 | 2 | NaN | 2 | NaN | 1 |
| ANKRD13D | Q6ZTN6-3 | 0.00332 | 0.000612 | -0.621618 | + | 1.76871 | -3.27006 | | 17.4938 | 17.4482 | 17.4538 | 18.289 | 18.4477 | 17.6608 | 17.8712 | 18.1657 | 2 | 3 | 4 | 5 | 4 | 4 | 4 | 2 |
| GPR176 | Q14439;Q1 | 9.82E-11 | 4.71E-11 | -0.621515 | + | 5.29897 | -15.2476 | | 15.7839 | 15.6763 | 15.6551 | 16.3448 | 16.3694 | 16.2649 | 16.2871 | 16.3669 | 6 | 7 | 5 | 5 | 6 | 6 | 6 | 7 |
| DCAF16 | Q9NXF7 | 3.94E-06 | 1.08E-06 | -0.621236 | | 0.838017 | -1.67375 | | 12.9308 | 13.6865 | 13.8973 | 13.5406 | 14.7897 | 13.6942 | 14.2805 | 14.3256 | 3 | 3 | 3 | 3 | 2 | 3 | 3 | 3 |
| PBLD | P30039 | 8.38E-05 | 2.00E-05 | -0.620213 | + | 1.97999 | -3.66862 | | 14.4606 | 14.4944 | 14.3306 | 14.6205 | 15.1333 | 14.9398 | 15.3038 | 15.2464 | 6 | 6 | 7 | 10 | 8 | 10 | 10 | 11 |
| UPP1 | Q16831 | 6.24E-14 | 6.30E-14 | -0.619552 | | 0.486629 | -1.06915 | | 14.0761 | 14.09 | 14.3011 | 14.6332 | 13.5198 | 15.0753 | 16.1776 | 14.4704 | 5 | 6 | 6 | 4 | 3 | 7 | 8 | 4 |
| NRBP1 | Q9UHY1 | 8.72E-09 | 3.19E-09 | -0.61948 | + | 2.27324 | -4.25843 | | 16.0477 | 15.9911 | 16.014 | 16.5974 | 16.9772 | 16.6529 | 16.2933 | 16.6647 | 15 | 16 | 15 | 14 | 12 | 14 | 13 | 15 |
| POLG | P54098 | 0.003842 | 0.0007 | -0.618588 | | 0.378586 | -0.93621 | | 14.0939 | NaN | 14.2255 | 14.1968 | NaN | 14.3424 | NaN | 15.7956 | 3 | NaN | 3 | 2 | NaN | 1 | NaN | 1 |
| DCP1A | Q9NPI6;Q9 | 2.53E-06 | 7.03E-07 | -0.617898 | | 0.587665 | -1.24829 | | 14.1121 | 13.9158 | 14.1587 | 14.4581 | 16.1429 | 14.1843 | 14.2335 | 14.3818 | 5 | 6 | 6 | 4 | 2 | 4 | 4 | 5 |
| TTF2 | Q9UNY4 | 0.001879 | 0.00037 | -0.617603 | | 0.502493 | -1.11792 | | 11.9823 | 12.3985 | 12.298 | 13.5928 | 11.6622 | NaN | 13.5454 | 12.5751 | 1 | 2 | 2 | 3 | 1 | NaN | 2 | 2 |
| INA | Q16352 | 2.62E-12 | 1.71E-12 | -0.616616 | | 0.855523 | -1.70296 | | 14.7266 | 14.6823 | 14.6869 | 15.3595 | 15.4012 | 14.602 | 14.9799 | 16.2335 | 8 | 8 | 6 | 9 | 9 | 6 | 7 | 11 |
| ST7 | Q9NRC1 | 0.029452 | 0.004843 | -0.615731 | | 0.764952 | -1.55131 | | 14.1683 | 14.8109 | 14.4163 | 14.5953 | 16.1646 | 15.0239 | 14.7755 | 14.8453 | 3 | 1 | 1 | 1 | 2 | 2 | 3 | 1 |
| GK | P32189;P3 | 0.000226 | 5.10E-05 | -0.615022 | | 0.7814 | -1.57896 | | 14.8925 | 14.8606 | 14.8684 | 16.5108 | 15.4786 | 14.8228 | 15.0346 | 15.5975 | 7 | 6 | 7 | 10 | 5 | 8 | 5 | 6 |
| CLDND1 | Q9NY35;Q9 | 0.000239 | 5.38E-05 | -0.614464 | + | 3.63693 | -7.82053 | | 14.2583 | 14.4231 | 14.4644 | 15.03 | 14.8678 | 15.0624 | 15.1186 | 14.9031 | 3 | 2 | 2 | 2 | 1 | 2 | 2 | 2 |
| ATP8B2 | P98198;P9 | 0.001733 | 0.000345 | -0.614382 | + | 3.89752 | -8.7095 | | 13.9311 | 14.1219 | 13.8288 | 14.5468 | 14.6161 | 14.5166 | 14.6467 | 14.5486 | 5 | 4 | 6 | 6 | 5 | 5 | 4 | 6 |
| PSTPIP2 | Q9H939 | 0.001132 | 0.000232 | -0.613097 | + | 1.98262 | -3.6737 | | 13.6934 | 13.7117 | 13.6948 | 14.5577 | 14.5686 | 13.898 | 14.1929 | 14.3482 | 2 | 3 | 4 | 4 | 2 | 1 | 2 | 2 |
| ANKRD52 | Q8NB46 | 7.77E-11 | 3.80E-11 | -0.612473 | + | 1.81693 | -3.3593 | | 13.8585 | 13.5859 | 13.8685 | 14.6155 | 13.9104 | 14.3335 | 14.5351 | 14.5227 | 5 | 4 | 4 | 4 | 3 | 3 | 5 | 4 |
| TRIM11 | Q96F44;Q9 | 4.21E-05 | 1.04E-05 | -0.612354 | | 0.927683 | -1.82306 | | 14.7634 | 14.2265 | 14.3249 | 15.6323 | 15.5775 | 14.4625 | 14.7246 | 14.8563 | 2 | 2 | 2 | 3 | 1 | 2 | 3 | 3 |
| TTC13 | Q8NBP0 | 0.001441 | 0.000291 | -0.610811 | + | 1.59991 | -2.96486 | | 14.4271 | 14.5233 | 14.4871 | 14.5619 | 15.3487 | 15.1974 | 15.3971 | 14.9448 | 4 | 4 | 3 | 5 | 5 | 5 | 4 | 4 |
| DERL1 | Q9BUN8 | 0.00043 | 9.34E-05 | -0.610764 | + | 2.54064 | -4.84035 | | 17.4814 | 17.689 | 17.5453 | 18.168 | 17.8942 | 18.3238 | 18.4051 | 18.1222 | 6 | 6 | 5 | 6 | 5 | 6 | 7 | 6 |
| DAB2 | P98082 | 3.61E-16 | 6.94E-16 | -0.609657 | + | 2.09396 | -3.89238 | | 17.4067 | 17.3672 | 17.374 | 17.9953 | 18.3123 | 18.0958 | 17.5906 | 17.9675 | 25 | 25 | 25 | 25 | 25 | 26 | 23 | 25 |
| SPTY2D1 | Q68D10;Q6 | 1.52E-05 | 3.91E-06 | -0.608754 | + | 1.54143 | -2.86149 | | 14.2326 | 14.5861 | 14.3381 | 15.2069 | 14.4284 | 14.9968 | 15.2598 | 15.0799 | 3 | 1 | 3 | 4 | 3 | 3 | 3 | 4 |
| EHD1 | Q9H4M9 | 1.02E-16 | 2.42E-16 | -0.608425 | + | 2.58304 | -4.93695 | | 18.0303 | 18.0051 | 17.9386 | 18.5879 | 18.879 | 18.4175 | 18.3969 | 18.7175 | 25 | 26 | 25 | 25 | 27 | 26 | 25 | 26 |
| UXS1 | Q8NBZ7;Q8 | 9.19E-05 | 2.19E-05 | -0.60756 | + | 2.24217 | -4.19369 | | 14.4449 | 14.4775 | 14.51 | 15.3144 | 15.2717 | 14.7033 | 15.0572 | 15.0783 | 4 | 5 | 6 | 7 | 4 | 4 | 6 | 5 |
| MRAS | O14807 | 2.03E-08 | 7.08E-09 | -0.607387 | + | 4.3861 | -10.618 | | 15.5869 | 15.773 | 15.7348 | 16.3428 | 16.2155 | 16.351 | 16.2552 | 16.3638 | 3 | 4 | 4 | 5 | 3 | 4 | 3 | 4 |
| TARBP2 | Q15633;Q1 | 0.001675 | 0.000334 | -0.607225 | + | 3.00107 | -5.96162 | | 14.2216 | 14.0423 | 13.9993 | 14.6518 | 14.9447 | 14.5447 | 14.6823 | 14.6511 | 2 | 4 | 3 | 4 | 2 | 1 | 5 | 3 |
| AP5S1 | Q9NUS5 | 0.004605 | 0.000828 | -0.606047 | + | 1.87665 | -3.47119 | | 14.3643 | 13.9403 | 14.3854 | 14.9299 | 15.0437 | 14.4567 | 14.9698 | 14.7802 | 2 | 3 | 3 | 2 | 2 | 2 | 3 | 3 |
| SNX19 | Q92543;Q9 | 0.002355 | 0.000453 | -0.60602 | + | 1.53047 | -3.01811 | | 13.6346 | 13.5161 | 13.3762 | 14.3918 | NaN | 14.0553 | 14.3292 | 13.6837 | 3 | 3 | 3 | 3 | NaN | 4 | 3 | 3 |
| SCRN1 | Q12765;Q1 | 7.83E-11 | 3.82E-11 | -0.60483 | + | 2.93663 | -5.79454 | | 17.59 | 17.655 | 17.6327 | 18.5199 | 18.2533 | 18.1726 | 18.0843 | 18.1237 | 19 | 17 | 19 | 19 | 17 | 20 | 19 | 17 |
| CDYL | Q9Y232;Q9 | 0.00201 | 0.000394 | -0.604309 | + | 2.20339 | -4.11366 | | 13.4813 | 13.3388 | 13.3135 | 13.7049 | 14.2612 | 13.7725 | 14.1412 | 14.0311 | 2 | 2 | 2 | 2 | 1 | 2 | 3 | 3 |
| NDUFA5 | Q16718 | 7.60E-15 | 9.90E-15 | -0.604283 | | 1.10646 | -2.12021 | | 15.6231 | 15.3549 | 15.9307 | 16.05 | 16.0309 | 15.9229 | 16.2063 | 16.9925 | 5 | 4 | 6 | 5 | 4 | 5 | 6 | 6 |
| DNAJC16 | Q9Y2G8 | 0.000115 | 2.69E-05 | -0.604099 | + | 3.22908 | -6.58234 | | 16.7157 | 16.8598 | 16.7941 | 17.408 | 17.5339 | 17.1875 | 17.3187 | 17.5217 | 4 | 5 | 6 | 6 | 6 | 5 | 6 | 7 |
| UCHL3 | P15374 | 5.43E-13 | 4.20E-13 | -0.603954 | + | 1.63987 | -3.03616 | | 16.3177 | 15.8287 | 16.4438 | 16.3716 | 16.9563 | 16.9078 | 16.8976 | 16.8703 | 14 | 13 | 14 | 12 | 14 | 13 | 13 | 14 |
| MYO5A | Q9Y4I1;Q9 | 5.81E-15 | 7.84E-15 | -0.602645 | + | 3.32387 | -6.85479 | | 16.1347 | 16.1733 | 16.1002 | 16.8322 | 16.9235 | 16.5535 | 16.655 | 16.7293 | 40 | 41 | 39 | 49 | 37 | 40 | 38 | 42 |
| DNMBP | Q6XZF7 | 7.00E-12 | 4.20E-12 | -0.601876 | + | 2.13896 | -3.98259 | | 15.3368 | 15.2864 | 15.2084 | 16.0673 | 16.1652 | 15.528 | 15.803 | 15.8319 | 18 | 18 | 20 | 27 | 23 | 23 | 23 | 21 |
| MFSD8 | Q8NHS3 | 0.000237 | 5.34E-05 | -0.60118 | + | 2.00756 | -3.72214 | | 14.5431 | 14.7272 | 14.6751 | 15.1471 | 14.8897 | 15.2861 | 15.6098 | 15.3155 | 2 | 2 | 2 | 4 | 1 | 4 | 3 | 3 |
| SLC38A2 | Q96QD8 | 9.79E-10 | 4.06E-10 | -0.600599 | + | 1.79402 | -3.31677 | | 17.7562 | 17.693 | 17.6847 | 18.3162 | 17.9821 | 18.3404 | 18.786 | 18.1347 | 5 | 5 | 5 | 5 | 5 | 5 | 5 | 5 |
| GALM | Q96C23 | 0.001158 | 0.000237 | -0.599586 | + | 1.64838 | -3.05143 | | 16.1009 | 16.0337 | 15.9864 | 16.3486 | 17.1776 | 16.5271 | 16.7 | 16.4464 | 6 | 7 | 4 | 7 | 7 | 7 | 8 | 5 |
| CSGALNACT2 | Q8N6G5 | 0.000326 | 7.19E-05 | -0.598119 | | 1.02405 | -1.98312 | | 14.49 | 14.5869 | 14.4711 | 14.9093 | 15.5109 | 14.7785 | 15.7748 | 14.5972 | 1 | 3 | 2 | 2 | 1 | 1 | 3 | 1 |
| MCAT | Q8IVS2 | 0.002534 | 0.000483 | -0.597922 | | 0.775934 | -1.81281 | | 14.1044 | 14.5609 | 14.5365 | 14.6381 | 15.3589 | NaN | NaN | NaN | 4 | 4 | 3 | 2 | 1 | NaN | NaN | NaN |
| TCF20 | Q9UGU0;Q | 0.00095 | 0.000197 | -0.597881 | + | 2.51957 | -4.7928 | | 13.2239 | 13.3626 | 13.3016 | 13.9237 | 13.7535 | 13.6268 | 14.1159 | 14.0494 | 2 | 4 | 4 | 5 | 2 | 2 | 2 | 4 |
| PODXL | O00592;O0 | 6.02E-09 | 2.25E-09 | -0.596393 | | 0.770622 | -1.56085 | | 16.5354 | 16.5594 | 16.5856 | 17.9958 | 16.8391 | 16.4687 | 16.8217 | 17.6574 | 9 | 10 | 10 | 11 | 9 | 10 | 9 | 9 |
| PKM | P14618-2 | 1.24E-09 | 5.08E-10 | -0.595606 | + | 2.75451 | -5.34083 | | 19.9103 | 19.9885 | 19.9474 | 20.6846 | 20.7126 | 20.3265 | 20.6356 | 20.3624 | 5 | 5 | 5 | 5 | 5 | 5 | 5 | 5 |
| HSPG2 | P98160 | 1.74E-13 | 1.55E-13 | -0.595516 | + | 1.8607 | -3.44114 | | 15.0604 | 14.9791 | 15.0125 | 15.7671 | 15.5739 | 15.53 | 15.2087 | 15.9844 | 41 | 46 | 40 | 51 | 40 | 49 | 40 | 47 |
| PKN1 | Q16512;Q1 | 9.91E-08 | 3.20E-08 | -0.595319 | + | 2.11918 | -3.9428 | | 15.507 | 15.3129 | 15.3867 | 16.0883 | 16.1071 | 16.1768 | 16.0453 | 15.5702 | 11 | 9 | 11 | 14 | 9 | 15 | 15 | 9 |
| SCAMP3 | O14828 | 1.62E-07 | 5.14E-08 | -0.59529 | + | 1.91717 | -3.54801 | | 17.5204 | 17.4937 | 17.4973 | 17.8524 | 18.0261 | 17.886 | 18.5426 | 18.1883 | 8 | 7 | 7 | 8 | 7 | 8 | 8 | 8 |
| UHRF1BP1L | A0JNW5 | 1.36E-20 | 1.18E-19 | -0.595048 | + | 2.49791 | -4.74423 | | 14.1727 | 14.0226 | 14.0095 | 14.8631 | 14.3844 | 14.5257 | 14.7856 | 14.758 | 5 | 6 | 7 | 11 | 5 | 8 | 9 | 8 |
| TOM1 | O60784 | 7.85E-13 | 5.81E-13 | -0.594628 | + | 1.72027 | -3.18138 | | 17.1677 | 16.9944 | 17.2364 | 17.5311 | 17.6933 | 18.1418 | 17.894 | 17.3771 | 16 | 17 | 18 | 17 | 17 | 18 | 17 | 17 |
| COL11A1 | P12107;P1 | 0.001794 | 0.000356 | -0.594144 | | 0.565695 | -1.2346 | | 13.3186 | 13.519 | 13.7817 | NaN | 14.6943 | 14.6843 | 14.1458 | 13.0113 | 2 | 1 | 1 | NaN | 1 | 5 | 5 | 1 |

| Gene names | UniProt IDs | Protein Group P-value | Protein Group Q-value | Log2 Difference NUP214 vs Controls (n=5) | p<0.05 | -log10 p-value | T-test Test statistic | NPC Localization | Log2 MS2_quantity_NUP214_1 | Log2 MS2_quantity_NUP214_2 | Log2 MS2_quantity_NUP214_3 | Log2 MS2_quantity_Control_1 | Log2 MS2_quantity_Control_2 | Log2 MS2_quantity_Control_3 | Log2 MS2_quantity_Control_4 | Log2 MS2_quantity_Control_5 | Number Peptides for Quantitation NUP214_1 | Number Peptides for Quantitation NUP214_2 | Number Peptides for Quantitation NUP214_3 | Number Peptides for Quantitation Control_1 | Number Peptides for Quantitation Control_2 | Number Peptides for Quantitation Control_3 | Number Peptides for Quantitation Control_4 | Number Peptides for Quantitation Control_5 |
|---|---|---|---|---|---|---|---|---|---|---|---|---|---|---|---|---|---|---|---|---|---|---|---|---|
| ABCD4 | O14678 | 0.002845 | 0.000535 | -0.593793 | | 1.07958 | -2.15933 | | 14.3926 | 14.5394 | 14.3318 | 14.4114 | NaN | 15.0174 | 15.5159 | 15.1155 | 2 | 1 | 1 | 3 | NaN | 2 | 4 | 2 |
| SLC30A6 | Q6NXT4;Q6 | 2.64E-16 | 5.38E-16 | -0.593673 | + | 2.46091 | -4.66201 | | 14.3404 | 14.4871 | 14.3864 | 14.7329 | 14.9191 | 14.941 | 15.2711 | 15.1276 | 5 | 4 | 6 | 5 | 3 | 5 | 5 | 6 |
| ATP6V0A1 | Q93050;Q9 | 1.03E-13 | 9.82E-14 | -0.593603 | + | 2.51585 | -4.78444 | | 16.6048 | 16.5208 | 16.5774 | 17.3344 | 16.9868 | 16.9743 | 17.4234 | 17.0876 | 16 | 16 | 16 | 17 | 16 | 17 | 17 | 17 |
| SKP1 | P63208 | 1.94E-06 | 5.45E-07 | -0.592926 | | 0.932653 | -1.83132 | | 17.9095 | 17.1592 | 18.6349 | 18.444 | 18.3627 | 18.3565 | 18.6363 | 18.6714 | 10 | 9 | 8 | 8 | 8 | 7 | 10 | 9 |
| CPEB2 | Q7Z5Q1;Q7 | 0.004033 | 0.000731 | -0.592613 | + | 1.55466 | -2.88478 | | 12.5773 | 12.5577 | 12.2687 | 13.0407 | 12.8904 | 12.696 | 13.5604 | 13.1149 | 1 | 2 | 1 | 3 | 2 | 1 | 1 | 3 |
| NBEAL1 | Q6ZS30 | 8.83E-09 | 3.23E-09 | -0.590512 | + | 2.74462 | -5.31694 | | 13.6295 | 13.8228 | 13.9155 | 14.5854 | 14.3014 | 14.3463 | 14.1876 | 14.4783 | 8 | 7 | 6 | 8 | 4 | 7 | 7 | 8 |
| WDR35 | Q9P2L0;Q9 | 0.005909 | 0.001044 | -0.589899 | + | 1.38418 | -2.58863 | | 14.6052 | 14.7084 | 15.3085 | 15.3666 | 15.9181 | 15.191 | 15.4535 | 15.3904 | 3 | 3 | 2 | 2 | 2 | 2 | 2 | 1 |
| TSC22D4 | Q9Y3Q8 | 6.78E-09 | 2.52E-09 | -0.589772 | + | 1.90623 | -3.52718 | | 15.1589 | 14.9801 | 15.0579 | 15.9144 | 15.7589 | 15.4908 | 15.2615 | 15.8514 | 9 | 7 | 9 | 9 | 10 | 11 | 6 | 9 |
| ITGBL1 | O95965 | 5.34E-13 | 4.14E-13 | -0.589388 | | 1.21264 | -2.29779 | | 14.3555 | 14.5658 | 14.4748 | 14.6075 | 14.9842 | 15.6579 | 15.2818 | 14.7424 | 5 | 6 | 4 | 5 | 6 | 6 | 7 | 5 |
| TMEM199 | Q8N511 | 0.000169 | 3.88E-05 | -0.589376 | + | 2.68025 | -5.16326 | | 14.5502 | 14.5635 | 14.4369 | 15.1247 | 15.2124 | 14.782 | 15.1946 | 15.2174 | 4 | 4 | 3 | 6 | 5 | 3 | 5 | 4 |
| SMC5 | Q8IY18 | 0.004914 | 0.00088 | -0.589112 | | 0.348367 | -0.82223 | | 14.2152 | 14.1966 | 14.0903 | 14.6392 | 13.361 | 16.3146 | NaN | 14.7111 | 4 | 4 | 7 | 3 | 2 | 1 | NaN | 3 |
| ZNHIT2 | Q9UHR6 | 0.001982 | 0.000389 | -0.58688 | | 0.383376 | -0.9114 | | 14.7902 | 14.4524 | 14.9111 | 15.2236 | 16.4328 | NaN | 14.258 | NaN | 2 | 3 | 2 | 2 | 1 | NaN | 1 | NaN |
| PRPS1 | P60891 | 2.00E-14 | 2.32E-14 | -0.585313 | + | 1.45175 | -2.70503 | | 18.2691 | 18.3033 | 18.2394 | 19.258 | 18.7509 | 18.9697 | 18.2936 | 19.0073 | 17 | 17 | 17 | 16 | 17 | 17 | 15 | 17 |
| RALGPS2 | Q86X27;Q8 | 3.48E-14 | 3.76E-14 | -0.582531 | + | 2.31303 | -4.34219 | | 14.8089 | 14.8265 | 14.8428 | 15.4619 | 15.7308 | 15.11 | 15.4093 | 15.3308 | 9 | 11 | 9 | 13 | 11 | 11 | 13 | 12 |
| SCYL3 | Q8IZE3;Q8I | 1.12E-05 | 2.93E-06 | -0.582057 | | 1.2266 | -2.32126 | | 13.9286 | 13.8804 | 13.8627 | 14.4188 | 15.0453 | 14.0308 | 14.7307 | 14.1375 | 3 | 3 | 4 | 3 | 4 | 3 | 2 | 3 |
| DSC1 | Q08554;Q0 | 4.13E-09 | 1.58E-09 | -0.580965 | | 0.778618 | -1.57428 | | 14.3165 | 14.4789 | 14.3389 | 15.6049 | 14.8025 | 13.996 | 15.0634 | 15.3286 | 2 | 2 | 3 | 4 | 2 | 1 | 3 | 4 |
| TLCD3A | Q8TBR7;Q8 | 0.007745 | 0.00135 | -0.580623 | + | 3.10727 | -6.24484 | | 15.2973 | 15.4428 | 15.3687 | 15.7788 | 16.145 | 16.0509 | 15.916 | 15.8605 | 2 | 2 | 2 | 2 | 2 | 2 | 3 | 2 |
| DICER1 | Q9UPY3 | 0.000702 | 0.000148 | -0.579153 | + | 2.52842 | -4.81274 | | 13.9746 | 13.9311 | 13.832 | 14.5935 | 14.4549 | 14.644 | 14.5996 | 14.1666 | 7 | 6 | 4 | 7 | 4 | 6 | 6 | 4 |
| NDUFA4 | O00483 | 1.92E-06 | 5.39E-07 | -0.578531 | + | 1.46871 | -2.73444 | | 18.1825 | 17.77 | 18.434 | 18.5671 | 18.566 | 18.5289 | 18.7121 | 19.1627 | 4 | 4 | 4 | 4 | 4 | 4 | 4 | 4 |
| LSM1 | O15116 | 0.001767 | 0.000351 | -0.578418 | | 0.792134 | -1.59697 | | 16.9595 | 16.6791 | 17.1893 | 17.1518 | 17.6602 | 16.8543 | 17.5553 | 18.3835 | 2 | 2 | 2 | 3 | 3 | 3 | 3 | 4 |
| HPF1 | Q9NWY4 | 0.000747 | 0.000157 | -0.575383 | + | 2.20078 | -4.10831 | | 13.6865 | 13.8291 | 13.706 | 14.6103 | 14.3057 | 14.108 | 14.0831 | 14.4725 | 6 | 7 | 6 | 5 | 5 | 5 | 5 | 7 |
| CD44 | P16070;P1 | 3.20E-06 | 8.82E-07 | -0.575056 | + | 1.93192 | -3.57615 | | 20.4778 | 20.4593 | 20.4536 | 21.4471 | 21.1099 | 20.7119 | 20.9703 | 20.9539 | 9 | 9 | 9 | 9 | 9 | 9 | 9 | 9 |
| HAX1 | O00165;O0 | 6.66E-05 | 1.61E-05 | -0.574709 | + | 1.77135 | -3.27492 | | 15.6328 | 15.6792 | 15.7468 | 15.7924 | 16.4069 | 16.2511 | 16.2796 | 16.5749 | 3 | 4 | 3 | 3 | 2 | 3 | 2 | 2 |
| RBIS | Q8N0T1 | 0.001065 | 0.000219 | -0.572969 | | 0.518414 | -1.18079 | | 12.9744 | NaN | 13.8585 | 14.0867 | 14.4821 | 13.2261 | NaN | 14.1629 | 2 | NaN | 1 | 1 | 2 | 1 | NaN | 2 |
| BLOC1S4 | Q9NUP1 | 1.39E-06 | 3.96E-07 | -0.570223 | + | 1.66207 | -3.07603 | | 15.7943 | 15.6955 | 15.9715 | 15.8841 | 16.5307 | 16.6407 | 16.4167 | 16.4809 | 3 | 3 | 3 | 3 | 2 | 3 | 3 | 2 |
| CAVIN2 | O95810 | 6.27E-07 | 1.86E-07 | -0.569699 | | 0.234613 | -0.58063 | | 15.3706 | 15.425 | 15.4835 | 13.3383 | 16.3745 | 16.764 | 17.7226 | 15.7808 | 7 | 6 | 7 | 3 | 8 | 8 | 8 | 5 |
| ZBTB21 | Q9ULJ3;Q9 | 5.32E-21 | 5.22E-20 | -0.569379 | | 0.675313 | -1.39945 | | 13.8465 | 13.8249 | 12.3124 | 13.9172 | 13.9328 | 13.4239 | 14.1367 | 14.0759 | 2 | 2 | 2 | 2 | 2 | 1 | 2 | 2 |
| TECPR2 | O15040;O1 | 0.002811 | 0.000529 | -0.569355 | + | 1.77701 | -3.28536 | | 14.7896 | 15.1491 | 15.045 | 15.376 | 15.8655 | 15.2218 | 15.6748 | 15.6814 | 6 | 7 | 7 | 7 | 3 | 6 | 5 | 5 |
| FBXW8 | Q8N3Y1 | 2.64E-10 | 1.18E-10 | -0.568454 | + | 4.32895 | -10.3769 | | 14.2018 | 14.2261 | 14.2863 | 14.7302 | 14.9034 | 14.8321 | 14.7027 | 14.8642 | 8 | 8 | 7 | 7 | 5 | 8 | 7 | 6 |
| HBD;HBB | P02042;P6 | 6.87E-05 | 1.66E-05 | -0.565905 | + | 1.99997 | -3.70736 | | 17.3488 | 16.839 | 17.0842 | 17.835 | 17.4088 | 17.5769 | 17.6276 | 17.8347 | 2 | 2 | 2 | 2 | 2 | 2 | 2 | 2 |
| | Q6ZSR9 | 0.000145 | 3.37E-05 | -0.565206 | + | 3.12839 | -6.3024 | | 17.4078 | 17.2515 | 17.2302 | 18.0461 | 17.9256 | 17.7037 | 17.7688 | 17.8643 | 6 | 5 | 6 | 7 | 6 | 6 | 6 | 7 |
| CEP170 | Q5SW79 | 1.51E-21 | 1.65E-20 | -0.564935 | | 0.890016 | -1.76042 | | 15.6773 | 15.733 | 15.7078 | 17.1748 | 16.1013 | 15.952 | 15.815 | 16.3118 | 46 | 43 | 45 | 53 | 45 | 46 | 46 | 50 |
| CHEK1 | O14757;O1 | 0.001481 | 0.000298 | -0.564106 | | 0.619468 | -1.37832 | | 13.7734 | NaN | 13.6249 | 14.8267 | 13.7391 | NaN | 13.8633 | 14.6241 | 1 | 0 | 2 | 3 | 2 | NaN | 2 | 2 |
| RPA3 | P35244 | 3.57E-05 | 8.88E-06 | -0.56402 | | 1.00275 | -1.94774 | | 16.1117 | 15.8052 | 16.1738 | 16.0538 | 16.4233 | 16.3322 | 17.1106 | 17.0512 | 4 | 4 | 4 | 4 | 4 | 4 | 4 | 4 |
| ERCC4 | Q92889 | 0.00097 | 0.000201 | -0.564017 | + | 1.48398 | -2.761 | | 13.8594 | 13.9744 | 13.9833 | 14.1928 | 14.8544 | 14.1287 | 14.8188 | 14.5206 | 4 | 7 | 6 | 4 | 3 | 2 | 4 | 6 |
| TANC2 | Q9HCD6;Q | 6.99E-11 | 3.46E-11 | -0.563989 | + | 2.71712 | -5.25091 | | 14.3985 | 14.4559 | 14.4438 | 15.0119 | 15.2207 | 14.7196 | 15.0192 | 15.0123 | 9 | 6 | 6 | 11 | 6 | 3 | 6 | 8 |
| RAPGEF1 | Q13905;Q1 | 0.001241 | 0.000253 | -0.563584 | | 0.950522 | -1.92598 | | 14.5053 | 14.466 | 14.6415 | 15.2299 | 15.7378 | NaN | 14.6843 | 14.7527 | 5 | 4 | 4 | 6 | 4 | NaN | 3 | 3 |
| DUSP3 | P51452 | 7.58E-07 | 2.24E-07 | -0.563299 | | 1.05494 | -2.03447 | | 17.6935 | 17.5742 | 17.6172 | 18.5079 | 18.4358 | 17.3944 | 18.1836 | 18.4363 | 7 | 7 | 7 | 7 | 7 | 7 | 7 | 7 |
| SH3BGRL | O75368 | 8.32E-14 | 8.11E-14 | -0.562976 | | 1.06266 | -2.04731 | | 19.0078 | 18.4758 | 19.1149 | 18.8175 | 19.5202 | 19.3165 | 19.8476 | 19.6439 | 9 | 9 | 9 | 9 | 9 | 9 | 9 | 9 |
| GFPT1 | Q06210;Q0 | 6.28E-11 | 3.13E-11 | -0.561768 | + | 3.45128 | -7.23522 | | 18.0548 | 18.0618 | 18.0407 | 18.4988 | 18.5806 | 18.6946 | 18.7974 | 18.4997 | 30 | 30 | 30 | 30 | 28 | 30 | 30 | 29 |
| RHOB | P62745 | 0.000996 | 0.000205 | -0.561593 | + | 1.56084 | -3.07938 | | 14.6444 | 14.4366 | 14.4887 | 14.7826 | NaN | 15.1802 | 15.4527 | 14.9237 | 2 | 2 | 3 | 2 | NaN | 2 | 3 | 2 |
| MOCOS | Q96EN8 | 8.51E-05 | 2.03E-05 | -0.560096 | | 0.931511 | -1.99275 | | 14.4172 | 14.6977 | 14.3853 | NaN | 14.6487 | 15.5496 | 14.9823 | NaN | 6 | 3 | 4 | NaN | 1 | 7 | 2 | NaN |
| GANC | Q8TET4 | 1.17E-06 | 3.37E-07 | -0.558857 | | 0.935185 | -1.89844 | | 14.7125 | 14.2575 | 14.5921 | 14.4491 | 15.3436 | 15.4834 | 15.0423 | NaN | 1 | 1 | 1 | 1 | 1 | 2 | 1 | NaN |
| ECD | O95905;O9 | 4.49E-09 | 1.71E-09 | -0.558093 | | 1.10213 | -2.11301 | | 14.603 | 14.3438 | 13.8604 | 15.3189 | 14.5158 | 14.6492 | 14.5707 | 15.0812 | 3 | 6 | 6 | 8 | 4 | 6 | 5 | 7 |
| CDH13 | P55290;P5 | 0.000202 | 4.58E-05 | -0.557665 | | 0.609673 | -1.28656 | | 14.6085 | 15.0952 | 14.9516 | 14.9501 | 15.3386 | 16.5153 | 15.6905 | 14.7194 | 5 | 3 | 4 | 2 | 2 | 4 | 4 | 3 |
| BCL2L1 | Q07817;Q0 | 0.000123 | 2.87E-05 | -0.557525 | + | 2.56991 | -4.90689 | | 16.5281 | 16.3669 | 16.5282 | 16.9352 | 17.2848 | 16.8125 | 17.0164 | 17.1107 | 3 | 2 | 3 | 3 | 2 | 2 | 3 | 3 |
| CCNT1 | O60563 | 0.001824 | 0.000361 | -0.557359 | + | 2.41198 | -4.55466 | | 13.8046 | 13.7919 | 13.5638 | 14.2921 | 14.3525 | 13.9694 | 14.4467 | 14.3264 | 2 | 3 | 3 | 4 | 3 | 3 | 3 | 4 |
| IGF1R | P08069 | 0.001832 | 0.000362 | -0.557218 | | 1.22122 | -2.42041 | | 14.5169 | 14.2036 | 14.3527 | 14.8802 | NaN | 14.512 | 15.4038 | 14.8639 | 4 | 3 | 5 | 4 | NaN | 4 | 1 | 2 |
| SLC12A6 | Q9UHW9;Q | 1.31E-06 | 3.74E-07 | -0.556138 | + | 1.89689 | -3.50946 | | 13.5954 | 13.6646 | 13.3079 | 14.2937 | 14.1339 | 13.7312 | 14.2553 | 13.9799 | 4 | 5 | 4 | 4 | 4 | 2 | 5 | 4 |
| KCTD5 | Q9NXV2 | 3.68E-20 | 2.74E-19 | -0.555136 | + | 2.36257 | -4.44779 | | 15.0548 | 15.0781 | 15.0887 | 15.709 | 15.7457 | 15.2718 | 15.6252 | 15.7934 | 3 | 3 | 3 | 3 | 3 | 2 | 3 | 3 |
| ARL2BP | Q9Y2Y0;Q9 | 0.002647 | 0.000502 | -0.554873 | + | 1.69733 | -3.13969 | | 16.2271 | 16.2138 | 16.2648 | 16.5146 | 16.8768 | 16.4431 | 17.0213 | 17.0946 | 4 | 2 | 4 | 3 | 4 | 3 | 5 | 5 |
| TUBB2A | Q13885 | 1.62E-09 | 6.49E-10 | -0.553651 | | 0.647041 | -1.35104 | | 19.4519 | 18.9772 | 19.4057 | 19.41 | 20.9504 | 19.2794 | 19.6986 | 19.8212 | 2 | 2 | 2 | 2 | 2 | 2 | 2 | 2 |
| ATP5ME | P56385 | 7.08E-11 | 3.49E-11 | -0.552961 | | 0.38715 | -0.88531 | | 17.3841 | 16.9899 | 17.7309 | 17.9557 | 17.4633 | 16.6735 | 18.068 | 19.4459 | 5 | 5 | 5 | 5 | 5 | 5 | 5 | 5 |
| NSRP1 | Q9H0G5 | 1.31E-07 | 4.19E-08 | -0.552482 | + | 2.34622 | -4.41277 | | 12.946 | 13.18 | 13.2262 | 13.5446 | 13.8868 | 13.4758 | 13.8341 | 13.608 | 3 | 3 | 4 | 6 | 3 | 3 | 2 | 4 |
| CBLB | Q13191 | 0.000508 | 0.000109 | -0.552352 | + | 1.58602 | -2.94022 | | 14.7525 | 14.6203 | 14.8327 | 15.0329 | 15.2725 | 15.7665 | 15.3526 | 15.0132 | 6 | 8 | 7 | 6 | 7 | 10 | 6 | 7 |
| TNFRSF10B | O14763;O1 | 3.07E-08 | 1.05E-08 | -0.552331 | + | 1.61098 | -2.98456 | | 14.6702 | 14.5742 | 14.2184 | 14.9487 | 15.2325 | 14.6327 | 15.1093 | 15.2764 | 5 | 4 | 5 | 7 | 3 | 5 | 6 | 5 |
| YRDC | Q86U90 | 0.000252 | 5.66E-05 | -0.55211 | + | 1.37802 | -2.57807 | | 15.5045 | 15.5733 | 15.3797 | 16.2704 | 16.4028 | 15.5275 | 15.8441 | 16.145 | 3 | 3 | 4 | 4 | 2 | 3 | 4 | 6 |
| PAAF1 | Q9BRP4 | 0.000101 | 2.39E-05 | -0.551683 | + | 2.16058 | -4.02633 | | 15.4479 | 15.4205 | 15.3497 | 16.054 | 16.2883 | 15.9482 | 15.7603 | 15.7376 | 8 | 8 | 5 | 9 | 6 | 8 | 5 | 8 |
| CD68 | P34810 | 0.00219 | 0.000425 | -0.551214 | + | 3.04197 | -6.06952 | | 17.0383 | 16.9597 | 16.993 | 17.6768 | 17.5454 | 17.4182 | 17.718 | 17.3827 | 1 | 1 | 1 | 2 | 1 | 1 | 1 | 2 |
| RRM2B | Q7LG56 | 2.91E-05 | 7.29E-06 | -0.551118 | + | 2.08092 | -3.86644 | | 16.0778 | 16.1667 | 16.2738 | 16.3649 | 16.7153 | 16.8498 | 16.9776 | 16.7119 | 8 | 8 | 9 | 9 | 9 | 8 | 9 | 9 |
| MAN2B1 | O00754;O0 | 2.40E-14 | 2.72E-14 | -0.550875 | + | 1.78378 | -3.29784 | | 16.0034 | 16.255 | 16.1747 | 16.6233 | 17.1277 | 16.6744 | 16.6489 | 16.4018 | 18 | 17 | 17 | 18 | 18 | 19 | 18 | 19 |
| FUCA2 | Q9BTY2 | 1.45E-05 | 3.75E-06 | -0.55085 | + | 2.18898 | -4.08416 | | 14.4944 | 14.4887 | 14.6235 | 14.7796 | 15.2917 | 14.9325 | 15.2282 | 15.2002 | 6 | 8 | 8 | 4 | 8 | 5 | 8 | 7 |
| UTP11 | Q9Y3A2 | 0.003609 | 0.00066 | -0.549976 | + | 2.12027 | -3.945 | | 13.6372 | 13.8074 | 13.6156 | 14.3611 | 14.1875 | 14.0002 | 14.5516 | 14.083 | 5 | 6 | 6 | 4 | 3 | 2 | 3 | 5 |
| SP3 | Q02447;Q0 | 0.003799 | 0.000693 | -0.549128 | | 0.907596 | -1.78967 | | 12.3346 | 13.0906 | 12.9472 | 13.3803 | 14.0182 | 12.9401 | 13.3598 | 13.0012 | 3 | 3 | 4 | 4 | 3 | 3 | 2 | 3 |
| CDR2 | Q01850 | 0.002563 | 0.000488 | -0.549077 | + | 3.05322 | -6.09946 | | 15.0895 | 15.1389 | 15.156 | 15.6336 | 15.5318 | 15.8682 | 15.7972 | 15.5554 | 2 | 3 | 2 | 1 | 2 | 2 | 3 | 3 |
| PIEZO1 | Q92508 | 2.59E-13 | 2.21E-13 | -0.547853 | + | 3.33847 | -6.89756 | | 15.169 | 15.1135 | 15.1697 | 15.7437 | 15.5702 | 15.5632 | 15.7443 | 15.8716 | 19 | 21 | 21 | 23 | 20 | 21 | 22 | 24 |
| VTI1B | Q9UEU0 | 6.64E-10 | 2.80E-10 | -0.547551 | + | 2.32786 | -4.37363 | | 15.7079 | 15.8111 | 15.7646 | 16.454 | 16.1819 | 16.0514 | 16.5679 | 16.2887 | 8 | 8 | 8 | 8 | 7 | 7 | 7 | 7 |
| NID1 | P14543;P1 | 0.00456 | 0.00082 | -0.547483 | | 1.08826 | -2.0899 | | 14.5706 | 14.3018 | 14.4021 | 14.6716 | 15.0483 | 14.4815 | 15.5967 | 15.0634 | 5 | 4 | 4 | 3 | 1 | 4 | 6 | 4 |
| LIG1 | P18858;P1 | 3.75E-06 | 1.03E-06 | -0.546751 | | 0.637277 | -1.33425 | | 15.2012 | 15.1602 | 14.4159 | 15.795 | 15.8831 | 14.4933 | 15.2517 | 15.9395 | 4 | 4 | 4 | 4 | 4 | 4 | 7 | 6 |
| C12orf29 | Q8N999;Q8 | 3.07E-08 | 1.05E-08 | -0.546671 | + | 2.11393 | -3.93228 | | 13.9291 | 13.8513 | 13.9778 | 14.7258 | 14.6822 | 14.4099 | 14.2118 | 14.3007 | 2 | 2 | 2 | 4 | 3 | 3 | 2 | 3 |
| ATP6AP1 | Q15904 | 1.99E-10 | 9.12E-11 | -0.546248 | + | 3.21386 | -6.53942 | | 16.9896 | 17.0296 | 17.0634 | 17.5606 | 17.5978 | 17.4347 | 17.7919 | 17.4839 | 10 | 9 | 9 | 10 | 8 | 9 | 9 | 9 |
| CHP1 | Q99653 | 1.03E-06 | 2.99E-07 | -0.545922 | + | 1.7849 | -3.29991 | | 16.1582 | 15.863 | 16.4243 | 16.549 | 16.5805 | 16.5333 | 16.8753 | 16.934 | 9 | 8 | 9 | 10 | 9 | 9 | 9 | 9 |
| TSR3 | Q9UJK0 | 1.77E-05 | 4.52E-06 | -0.545651 | + | 2.84207 | -5.55564 | | 14.7541 | 14.6012 | 14.8171 | 15.3273 | 15.3674 | 15.0653 | 15.1762 | 15.4127 | 3 | 2 | 3 | 3 | 4 | 2 | 3 | 3 |
| BCL2L13 | Q9BXK5 | 2.34E-12 | 1.54E-12 | -0.544763 | + | 2.64503 | -5.08048 | | 15.7959 | 15.7334 | 15.7438 | 16.266 | 16.4696 | 16.0112 | 16.3762 | 16.3895 | 11 | 11 | 11 | 11 | 6 | 9 | 10 | 11 |
| MROH1 | Q8NDA8 | 3.84E-14 | 4.10E-14 | -0.544694 | + | 1.5306 | -2.84247 | | 14.7979 | 14.8817 | 14.931 | 15.3717 | 15.6594 | 14.8822 | 15.6238 | 15.5374 | 19 | 17 | 21 | 17 | 15 | 15 | 19 | 19 |
| ADAM15 | Q13444;Q1 | 0.020011 | 0.003331 | -0.544117 | | 0.796174 | -1.64983 | | 13.4909 | 13.1295 | 13.6042 | 14.236 | NaN | 14.1539 | 13.1756 | 14.2438 | 2 | 2 | 1 | 4 | NaN | 3 | 2 | 2 |
| LSM7 | Q9UK45 | 0.002668 | 0.000505 | -0.543753 | | 0.839692 | -1.67654 | | 16.905 | 16.2655 | 16.8307 | 16.3623 | 17.3924 | 17.2802 | 17.4749 | 17.5444 | 2 | 2 | 2 | 2 | 2 | 2 | 2 | 2 |
| CRTC1 | Q6UUV9;Q | 3.13E-05 | 7.83E-06 | -0.542973 | + | 2.88639 | -5.6667 | | 15.4334 | 15.1659 | 15.3931 | 15.7817 | 15.9228 | 15.9635 | 15.7061 | 15.9948 | 3 | 3 | 3 | 2 | 1 | 3 | 3 | 3 |

| Gene names | UniProt IDs | Protein Group P-value | Protein Group Q-value | Log2 Difference NUP214 vs Controls (n=5) | p<0.05 | -log10 p-value | T-test Test statistic | NPC Localization | Log2 MS2_quantity_ NUP214_1 | Log2 MS2_quantity_ NUP214_2 | Log2 MS2_quantity_ NUP214_3 | Log2 MS2_quantity_ Control_1 | Log2 MS2_quantity_ Control_2 | Log2 MS2_quantity_ Control_3 | Log2 MS2_quantity_ Control_4 | Log2 MS2_quantity_ Control_5 | Number Peptides for Quantitation NUP214_1 | Number Peptides for Quantitation NUP214_2 | Number Peptides for Quantitation NUP214_3 | Number Peptides for Quantitation Control_1 | Number Peptides for Quantitation Control_2 | Number Peptides for Quantitation Control_3 | Number Peptides for Quantitation Control_4 | Number Peptides for Quantitation Control_5 |
|---|---|---|---|---|---|---|---|---|---|---|---|---|---|---|---|---|---|---|---|---|---|---|---|---|
| RAB11FIP2 | Q7L804;Q7 | 0.000247 | 5.55E-05 | -0.542832 | | 0.892392 | -1.76438 | | 15.3076 | 15.1677 | 14.9854 | 15.3657 | 16.191 | 15.8125 | 15.0059 | 16.1068 | 2 | 2 | 1 | 2 | 2 | 2 | 1 | 2 |
| DNMT1 | P26358 | 4.29E-15 | 6.07E-15 | -0.541742 | | 0.809726 | -1.62645 | | 15.4341 | 15.3637 | 15.344 | 16.4273 | 15.8046 | 15.0453 | 15.9605 | 16.3741 | 27 | 31 | 27 | 39 | 33 | 26 | 34 | 37 |
| FAH | P16930 | 6.42E-13 | 4.87E-13 | -0.541281 | + | 2.616 | -5.01291 | | 16.4557 | 16.4027 | 16.3875 | 16.9417 | 17.1658 | 17.0617 | 16.9266 | 16.687 | 11 | 10 | 9 | 11 | 10 | 11 | 11 | 12 |
| CISD3 | P0C7P0 | 0.00218 | 0.000424 | -0.541133 | | 0.607573 | -1.28292 | | 14.2595 | 14.2611 | 14.6742 | 15.9049 | 15.2434 | 14.9166 | 14.5225 | 14.1097 | 2 | 2 | 2 | 2 | 2 | 2 | 2 | 2 |
| FEZ2 | Q9UHY8 | 1.22E-12 | 8.62E-13 | -0.540777 | + | 1.34021 | -2.51349 | | 15.0505 | 14.5344 | 14.8107 | 15.2906 | 14.8735 | 15.625 | 15.2804 | 15.6271 | 5 | 4 | 4 | 5 | 4 | 6 | 4 | 5 |
| TMEM185B | Q9H7F4 | 0.005998 | 0.001059 | -0.540294 | + | 1.70384 | -3.15151 | | 13.5422 | 13.4559 | 13.7458 | 14.2873 | 14.011 | 13.8023 | 14.0194 | 14.488 | 2 | 2 | 1 | 4 | 3 | 2 | 1 | 3 |
| CEMIP | Q8WUJ3 | 4.20E-15 | 5.96E-15 | -0.539733 | | 0.536631 | -1.15859 | | 15.5531 | 15.6759 | 15.5515 | 17.3564 | 16.1331 | 16.1139 | 15.2071 | 15.8555 | 40 | 43 | 41 | 51 | 39 | 40 | 37 | 41 |
| USP22 | Q9UPT9;Q9 | 0.002856 | 0.000536 | -0.539571 | | 1.30013 | -2.44539 | | 15.1799 | 15.0722 | 15.261 | 15.56 | 16.0378 | 15.4216 | 15.3729 | 16.1605 | 2 | 2 | 1 | 2 | 1 | 2 | 2 | 2 |
| RANBP10 | Q6VN20;Q | 0.001178 | 0.00024 | -0.537018 | + | 1.33762 | -2.50909 | | 13.6261 | 13.1953 | 13.58 | 14.1722 | 13.9938 | 14.2986 | 13.473 | 14.0833 | 2 | 2 | 4 | 2 | 1 | 2 | 2 | 3 |
| CCDC92 | Q53HC0;Q | 0.003635 | 0.000665 | -0.536998 | | 0.645231 | -1.34793 | | 14.711 | 14.8857 | 15.0198 | 15.8825 | 15.9285 | 14.383 | 15.1176 | 15.7342 | 4 | 7 | 5 | 7 | 5 | 3 | 5 | 6 |
| IDUA | P35475 | 1.80E-06 | 5.09E-07 | -0.535323 | | 1.06735 | -2.0551 | | 15.4896 | 15.4709 | 15.4738 | 15.7273 | 15.9223 | 16.3128 | 16.591 | 15.5137 | 12 | 12 | 12 | 13 | 12 | 14 | 14 | 12 |
| CRYBG3 | Q68DQ2 | 0.003519 | 0.000645 | -0.534726 | | 1.24182 | -2.34686 | | 14.4964 | 14.2927 | 14.3312 | 15.0259 | 15.4552 | 14.7611 | 14.435 | 14.8636 | 8 | 8 | 6 | 7 | 5 | 7 | 6 | 6 |
| ARL6 | Q9H0F7;Q9 | 0.000306 | 6.78E-05 | -0.534314 | + | 3.15363 | -6.37167 | | 14.7564 | 14.8952 | 14.8388 | 15.1608 | 15.5024 | 15.4018 | 15.438 | 15.3193 | 2 | 3 | 4 | 3 | 2 | 2 | 2 | 3 |
| LASP1 | Q14847;Q1 | 1.12E-11 | 6.42E-12 | -0.533 | + | 3.49634 | -7.37382 | | 19.9961 | 19.8989 | 19.8472 | 20.361 | 20.6201 | 20.3997 | 20.3683 | 20.4863 | 18 | 18 | 19 | 19 | 19 | 18 | 19 | 19 |
| ATP5MF | P56134-2; | 1.04E-09 | 4.30E-10 | -0.532762 | | 0.662149 | -1.37695 | | 16.5729 | 15.6177 | 15.9132 | 16.5483 | 17.335 | 15.835 | 16.3596 | 16.759 | 2 | 2 | 2 | 2 | 2 | 1 | 1 | 2 |
| FHL1 | Q13642-1 | 6.29E-11 | 3.14E-11 | -0.532313 | | 0.570286 | -1.21791 | | 18.946 | 19.0088 | 18.9581 | 18.486 | 20.0168 | 19.7711 | 19.0093 | 20.2331 | 3 | 3 | 3 | 3 | 3 | 3 | 3 | 3 |
| SLC8A1 | P32418-2 | 1.01E-09 | 4.20E-10 | -0.532211 | + | 2.18753 | -4.0812 | | 14.8163 | 14.8396 | 14.6642 | 15.015 | 15.3219 | 15.4095 | 15.5685 | 15.213 | 6 | 5 | 6 | 6 | 6 | 9 | 8 | 6 |
| ABLIM3 | O94929-2; | 4.21E-14 | 4.46E-14 | -0.53135 | + | 1.49992 | -2.78877 | | 14.7236 | 14.7685 | 14.7133 | 15.4609 | 15.6108 | 14.9107 | 14.9437 | 15.4063 | 7 | 9 | 7 | 10 | 7 | 5 | 5 | 9 |
| DDX21 | Q9NR30 | 3.58E-20 | 2.68E-19 | -0.531039 | + | 2.11552 | -3.93547 | | 16.251 | 16.3475 | 16.3082 | 17.0155 | 16.6433 | 16.5466 | 17.0318 | 16.9292 | 33 | 35 | 32 | 38 | 33 | 33 | 35 | 36 |
| MAP2K3 | P46734;P4 | 4.08E-12 | 2.55E-12 | -0.53044 | | 1.20643 | -2.28737 | | 17.2249 | 17.2742 | 17.2522 | 18.3163 | 17.7338 | 17.4701 | 17.3739 | 18.0104 | 16 | 16 | 16 | 16 | 16 | 16 | 16 | 16 |
| NFKBIB | Q15653 | 0.001242 | 0.000253 | -0.528863 | + | 2.4273 | -4.5881 | | 14.1215 | 14.058 | 14.5089 | 14.75 | 14.6362 | 14.8508 | 14.8324 | 14.7224 | 3 | 4 | 3 | 3 | 3 | 3 | 3 | 3 |
| POMGNT1 | Q8WZA1;Q | 0.001604 | 0.000321 | -0.528256 | + | 1.9203 | -3.55397 | | 15.1995 | 15.145 | 14.8701 | 15.9241 | 15.4011 | 15.4282 | 15.6972 | 15.5484 | 2 | 3 | 3 | 4 | 3 | 4 | 5 | 4 |
| ZGPAT | Q8N5A5-2 | 1.24E-07 | 3.96E-08 | -0.527532 | + | 1.66751 | -3.08582 | | 14.364 | 14.2156 | 14.4207 | 14.7251 | 15.1976 | 14.4663 | 14.9605 | 14.9554 | 2 | 3 | 2 | 4 | 2 | 3 | 2 | 3 |
| VMA21 | Q3ZAQ7 | 2.56E-24 | 7.58E-23 | -0.526198 | + | 2.05516 | -3.81547 | | 17.3654 | 17.0265 | 17.2191 | 17.8541 | 17.7526 | 17.4229 | 17.6809 | 17.9387 | 2 | 2 | 2 | 2 | 2 | 2 | 2 | 2 |
| FMNL2 | Q96PY5;Q9 | 0.001661 | 0.000332 | -0.525653 | + | 1.54056 | -3.0384 | | 14.6987 | 14.3885 | 14.7191 | 15.4894 | NaN | 15.0983 | 14.9389 | 14.9843 | 8 | 5 | 8 | 9 | NaN | 2 | 5 | 4 |
| BSDC1 | Q9NW68;Q | 8.84E-06 | 2.33E-06 | -0.52482 | + | 2.27394 | -4.2599 | | 14.6766 | 14.7365 | 15.0603 | 15.1778 | 15.3154 | 15.2536 | 15.4844 | 15.5152 | 5 | 4 | 5 | 6 | 4 | 6 | 7 | 6 |
| SLC39A1 | Q9NY26;Q9 | 0.002346 | 0.000452 | -0.524227 | + | 1.77334 | -3.5237 | | 14.4593 | 14.6772 | 14.4915 | 15.1299 | NaN | 14.8939 | 14.8748 | 15.3689 | 2 | 2 | 1 | 2 | NaN | 1 | 1 | 2 |
| TRAM1 | Q15629;Q1 | 1.63E-08 | 5.78E-09 | -0.523963 | + | 2.69252 | -5.19231 | | 17.167 | 17.077 | 17.1022 | 17.6201 | 17.5223 | 17.6456 | 17.9138 | 17.495 | 7 | 7 | 7 | 6 | 6 | 6 | 7 | 6 |
| PSMG4 | Q5JS54;Q5 | 2.60E-08 | 8.98E-09 | -0.523371 | | 0.748646 | -1.52384 | | 15.2377 | 14.8447 | 15.1754 | 15.0443 | 14.9958 | 15.8723 | 15.9026 | 16.2315 | 4 | 4 | 4 | 3 | 4 | 4 | 4 | 4 |
| KIF1C | O43896 | 8.18E-05 | 1.96E-05 | -0.523036 | + | 2.79764 | -5.44589 | | 15.0223 | 15.0715 | 15.1197 | 15.8161 | 15.6066 | 15.4106 | 15.6544 | 15.4834 | 12 | 12 | 14 | 14 | 8 | 14 | 9 | 10 |
| TRAFD1 | O14545 | 0.000416 | 9.06E-05 | -0.522477 | + | 1.53904 | -2.85729 | | 13.6104 | 13.4962 | 13.6034 | 13.5991 | 14.1694 | 14.092 | 14.4269 | 14.1748 | 6 | 6 | 9 | 7 | 2 | 7 | 5 | 5 |
| ZFAND3 | Q9H8U3 | 0.006085 | 0.001073 | -0.522344 | + | 1.812 | -3.35013 | | 13.2176 | 13.4049 | 12.8937 | 13.6412 | 13.8809 | 13.4661 | 13.5909 | 13.8927 | 2 | 1 | 1 | 1 | 1 | 1 | 1 | 1 |
| GLA | P06280 | 0.000713 | 0.00015 | -0.521991 | | 1.17596 | -2.23628 | | 15.5285 | 15.7703 | 15.8281 | 16.3298 | 16.4312 | 15.6018 | 16.5724 | 16.2196 | 3 | 3 | 4 | 2 | 2 | 2 | 4 | 3 |
| MAD2L1 | Q13257 | 8.31E-07 | 2.44E-07 | -0.521695 | | 1.15356 | -2.19882 | | 15.4808 | 15.6112 | 15.786 | 16.3765 | 15.9475 | 15.5738 | 16.3308 | 16.5097 | 7 | 6 | 6 | 6 | 7 | 7 | 7 | 7 |
| MMADHC | Q9H3L0 | 4.12E-08 | 1.39E-08 | -0.521544 | + | 4.2893 | -10.2125 | | 14.4758 | 14.4412 | 14.5569 | 14.9438 | 15.1106 | 14.9749 | 15.0744 | 14.9605 | 6 | 4 | 3 | 3 | 3 | 4 | 4 | 3 |
| RANGAP1 | P46060 | 5.27E-13 | 4.09E-13 | -0.52024 | | 1.18619 | -2.25342 | | 16.6819 | 16.7259 | 16.715 | 17.7216 | 17.1645 | 16.6671 | 17.175 | 17.411 | 25 | 25 | 25 | 27 | 25 | 24 | 27 | 27 |
| CLTB | P09497-2 | 2.44E-22 | 3.36E-21 | -0.520131 | + | 2.25816 | -4.22695 | | 17.3085 | 17.3442 | 17.5036 | 18.0909 | 17.6925 | 17.8699 | 18.1186 | 17.7559 | 13 | 12 | 12 | 12 | 11 | 13 | 13 | 12 |
| ATG12 | O94817 | 0.005162 | 0.000921 | -0.519297 | + | 1.85087 | -3.42268 | | 15.0254 | 14.8679 | 15.0181 | 15.4154 | 15.4215 | 15.5361 | 15.8743 | 15.2017 | 2 | 3 | 3 | 3 | 1 | 2 | 3 | 2 |
| TTC9C | Q8N5M4 | 1.32E-14 | 1.62E-14 | -0.51912 | + | 1.4431 | -2.69007 | | 16.2362 | 16.0612 | 16.6625 | 16.9718 | 16.5833 | 16.6147 | 16.8828 | 17.1429 | 3 | 2 | 3 | 4 | 2 | 2 | 2 | 3 |
| POLR3B | Q9NW08 | 0.017361 | 0.002903 | -0.518957 | | 0.839205 | -1.67573 | | 14.6079 | 13.689 | 14.7673 | 14.9709 | 14.3198 | 15.0892 | 14.9248 | 15.0637 | 2 | 2 | 2 | 4 | 1 | 4 | 5 | 4 |
| MRPL50 | Q8N5N7 | 0.000495 | 0.000107 | -0.518369 | + | 1.63128 | -3.02079 | | 15.8379 | 15.6988 | 16.0475 | 16.1366 | 16.5065 | 16.2558 | 16.2259 | 16.774 | 6 | 6 | 6 | 5 | 7 | 6 | 4 | 6 |
| SH3BP4 | Q9P0V3 | 1.58E-12 | 1.09E-12 | -0.517922 | + | 4.34207 | -10.4318 | | 15.6625 | 15.6509 | 15.599 | 16.0812 | 16.1435 | 16.2749 | 16.1884 | 16.0889 | 33 | 33 | 33 | 32 | 29 | 33 | 31 | 32 |
| GPATCH8 | Q9UKJ3 | 0.000364 | 7.98E-05 | -0.516952 | | 0.766533 | -1.55397 | | 14.4033 | 14.5672 | 14.6607 | 14.7112 | 15.9278 | 14.7718 | 15.2834 | 14.6092 | 5 | 4 | 4 | 2 | 1 | 4 | 3 | 4 |
| SYNGR1 | O43759;O4 | 8.81E-09 | 3.22E-09 | -0.516892 | | 0.511289 | -1.11347 | | 16.0947 | 15.614 | 15.8814 | 15.9559 | 16.2519 | 15.4596 | 17.4158 | 16.8182 | 2 | 2 | 2 | 2 | 1 | 1 | 1 | 2 |
| NUDT15 | Q9NV35 | 4.19E-13 | 3.36E-13 | -0.516758 | + | 1.8546 | -3.42968 | | 13.9098 | 13.9549 | 14.248 | 14.7194 | 14.6918 | 14.6611 | 14.5025 | 14.1969 | 5 | 4 | 4 | 5 | 2 | 4 | 4 | 2 |
| HPS5 | Q9UPZ3 | 2.35E-10 | 1.06E-10 | -0.516343 | + | 2.68026 | -5.16329 | | 14.5615 | 14.5326 | 14.4538 | 14.8988 | 15.115 | 15.0124 | 15.2661 | 14.8692 | 10 | 8 | 10 | 13 | 9 | 8 | 13 | 7 |
| FAM160B2 | Q86V87 | 6.94E-19 | 3.14E-18 | -0.516212 | | 1.0174 | -1.97208 | | 14.5054 | 14.6391 | 14.7128 | 14.9845 | 15.902 | 15.0111 | 14.8589 | 14.9201 | 4 | 4 | 3 | 4 | 3 | 3 | 4 | 4 |
| TOR1B | O14657 | 1.44E-12 | 1.00E-12 | -0.515797 | + | 1.6891 | -3.1248 | | 16.5615 | 16.5451 | 16.5456 | 17.2377 | 17.0687 | 16.5946 | 17.1461 | 17.2856 | 8 | 9 | 9 | 9 | 9 | 9 | 9 | 8 |
| NAT1 | P18440 | 0.001258 | 0.000256 | -0.515449 | + | 3.54175 | -7.51572 | | 15.7586 | 15.8025 | 15.88 | 16.1547 | 16.4353 | 16.3301 | 16.3891 | 16.3368 | 3 | 2 | 3 | 2 | 2 | 3 | 4 | 3 |
| SERPINB8 | P50452 | 7.31E-20 | 4.73E-19 | -0.515349 | + | 2.29817 | -4.3108 | | 17.5777 | 17.636 | 17.6215 | 18.0315 | 18.0716 | 18.2735 | 18.3797 | 17.8791 | 15 | 15 | 15 | 16 | 12 | 17 | 15 | 15 |
| RFLNB | Q8N5W9 | 2.34E-17 | 6.68E-17 | -0.515015 | | 1.00208 | -1.94663 | | 15.9569 | 15.8485 | 15.8645 | 16.72 | 16.0602 | 16.9314 | 16.4412 | 15.872 | 4 | 4 | 4 | 4 | 4 | 4 | 5 | 4 |
| PDPK1 | O15530;O1 | 1.45E-13 | 1.32E-13 | -0.514557 | + | 3.53698 | -7.50071 | | 14.5114 | 14.6042 | 14.675 | 15.0738 | 15.2601 | 14.9893 | 15.0949 | 15.1389 | 13 | 12 | 13 | 12 | 7 | 13 | 12 | 13 |
| APBB1 | O00213;O0 | 0.000267 | 5.96E-05 | -0.51206 | | 0.672172 | -1.39409 | | 14.7263 | 14.7177 | 14.7559 | 15.9874 | 15.222 | 14.3358 | 15.0954 | 15.5861 | 4 | 4 | 5 | 4 | 3 | 3 | 3 | 3 |
| SAMD4A | Q9UPU9 | 0.000203 | 4.61E-05 | -0.511784 | + | 3.09107 | -6.201 | | 14.9012 | 15.0305 | 14.794 | 15.6156 | 15.3587 | 15.3822 | 15.3544 | 15.3911 | 5 | 6 | 5 | 6 | 4 | 5 | 7 | 7 |
| TMEM230 | Q96A57;Q9 | 8.84E-07 | 2.59E-07 | -0.511674 | + | 2.75881 | -5.35124 | | 16.0582 | 15.9704 | 15.9696 | 16.3092 | 16.6046 | 16.6675 | 16.5932 | 16.3809 | 3 | 3 | 3 | 3 | 2 | 3 | 3 | 3 |
| ZBTB8OS | Q8IWT0 | 2.68E-11 | 1.43E-11 | -0.511657 | + | 1.97346 | -3.65599 | | 16.5781 | 16.1594 | 16.4708 | 16.6329 | 17.0866 | 16.8915 | 17.0423 | 16.9188 | 2 | 1 | 2 | 1 | 1 | 2 | 2 | 1 |
| HECTD4 | Q9Y4D8;Q9 | 0.0002 | 4.55E-05 | -0.511163 | | 1.18107 | -2.24485 | | 14.4316 | 14.3478 | 14.2493 | 14.897 | 14.7413 | 14.7583 | 15.4512 | 14.4224 | 5 | 4 | 6 | 6 | 2 | 3 | 3 | 4 |
| PRSS23 | O95084;O9 | 0.000504 | 0.000108 | -0.510749 | | 0.465909 | -1.04939 | | 14.8149 | 14.8253 | 14.8324 | NaN | 14.6045 | 15.737 | 16.2995 | 14.6988 | 1 | 1 | 2 | NaN | 1 | 3 | 4 | 2 |
| CASKIN2 | Q8WXE0;Q | 0.000312 | 6.90E-05 | -0.510506 | + | 1.95676 | -3.62379 | | 13.985 | 13.8601 | 13.9154 | 14.3074 | 14.6871 | 14.1352 | 14.6422 | 14.3816 | 5 | 4 | 3 | 3 | 3 | 3 | 3 | 2 |
| PDE4DIP | Q5VU43-13 | 7.31E-10 | 3.07E-10 | -0.509137 | + | 3.18077 | -6.44685 | | 14.4243 | 14.446 | 14.5311 | 15.0113 | 15.1723 | 14.8396 | 14.9068 | 14.9513 | 11 | 11 | 12 | 14 | 10 | 12 | 12 | 11 |
| COMMD7 | Q86VX2;Q8 | 5.16E-06 | 1.39E-06 | -0.508923 | + | 1.56361 | -2.90057 | | 14.8687 | 14.3846 | 14.8292 | 15.0337 | 15.3498 | 14.901 | 15.304 | 15.427 | 4 | 4 | 4 | 4 | 4 | 5 | 5 | 4 |
| PRRC2B | Q5JSZ5 | 0.000557 | 0.000119 | -0.508867 | + | 2.56133 | -4.88732 | | 13.7827 | 13.9176 | 13.9667 | 14.4331 | 14.5958 | 14.1466 | 14.4007 | 14.4133 | 9 | 11 | 12 | 16 | 9 | 9 | 13 | 12 |
| PYGL | P06737;P0 | 3.40E-15 | 4.98E-15 | -0.508537 | | 0.518854 | -1.12698 | | 16.7635 | 16.7864 | 16.8053 | 16.1385 | 17.5095 | 17.3105 | 18.2463 | 17.2631 | 38 | 33 | 35 | 30 | 33 | 38 | 38 | 37 |
| GPC1 | P35052 | 1.06E-09 | 4.39E-10 | -0.508073 | | 1.0891 | -2.09131 | | 15.9873 | 16.038 | 16.0952 | 16.4905 | 16.5285 | 16.0284 | 17.1677 | 16.5263 | 7 | 8 | 8 | 7 | 8 | 7 | 12 | 9 |
| SLC35E1 | Q96K37 | 2.68E-05 | 6.73E-06 | -0.507753 | + | 2.46958 | -4.68118 | | 15.4122 | 15.5508 | 15.6584 | 16.0726 | 15.9949 | 15.8037 | 16.1589 | 16.2108 | 4 | 4 | 4 | 4 | 4 | 4 | 5 | 5 |
| MINDY2 | Q8NBR6;Q8 | 4.19E-08 | 1.41E-08 | -0.507293 | + | 1.85824 | -3.43653 | | 13.8391 | 13.8397 | 13.9505 | 14.7329 | 14.0603 | 14.3288 | 14.3394 | 14.4572 | 3 | 3 | 4 | 4 | 2 | 4 | 4 | 5 |
| CSTF2 | P33240;P3 | 5.40E-08 | 1.80E-08 | -0.506403 | | 0.944908 | -1.85168 | | 15.9332 | 15.0546 | 16.2091 | 16.206 | 16.4314 | 16.0408 | 16.1231 | 16.3924 | 5 | 4 | 5 | 5 | 4 | 5 | 5 | 5 |
| EPDR1 | Q9UM22 | 2.70E-05 | 6.78E-06 | -0.506139 | | 0.627763 | -1.34758 | | 16.7273 | 16.7799 | 16.7935 | NaN | 18.1919 | 16.8189 | 17.1958 | 16.8856 | 6 | 5 | 6 | NaN | 5 | 5 | 5 | 5 |
| UGGT2 | Q9NYU1 | 5.95E-14 | 6.04E-14 | -0.505593 | + | 1.44049 | -2.68556 | | 15.8092 | 15.7558 | 15.7855 | 15.9195 | 16.3213 | 16.2874 | 16.7766 | 16.1408 | 19 | 18 | 18 | 21 | 16 | 19 | 25 | 21 |
| TOMM22 | Q9NS69 | 2.00E-05 | 5.10E-06 | -0.505528 | | 0.591516 | -1.25501 | | 14.6932 | 14.2966 | 14.6118 | 14.5523 | 14.8454 | 14.6958 | 14.9115 | 16.1919 | 2 | 2 | 2 | 2 | 2 | 2 | 2 | 2 |
| IFT122 | Q9HBG6;Q | 0.00288 | 0.00054 | -0.505359 | | 0.832154 | -1.71409 | | 14.3629 | 14.1047 | 14.0799 | NaN | 15.1565 | 15.0229 | 14.4292 | 14.1427 | 5 | 5 | 3 | NaN | 1 | 3 | 4 | 2 |
| SPTLC2 | O15270 | 5.21E-14 | 5.37E-14 | -0.504881 | + | 1.4084 | -2.63022 | | 15.8072 | 15.914 | 15.8713 | 16.6877 | 16.469 | 15.8461 | 16.3257 | 16.5168 | 16 | 16 | 14 | 16 | 16 | 16 | 14 | 18 |
| KATNAL1 | Q9BW62 | 0.00061 | 0.00013 | -0.50466 | + | 2.5117 | -4.77512 | | 15.116 | 15.1019 | 15.2551 | 15.6165 | 15.6307 | 15.5771 | 15.9531 | 15.5343 | 5 | 5 | 6 | 6 | 5 | 6 | 7 | 7 |
| KBTBD4 | Q9NVX7;Q9 | 0.00921 | 0.001587 | -0.504418 | | 1.08941 | -2.09181 | | 13.2384 | 14.1844 | 13.5204 | 14.1551 | 14.4296 | 13.9266 | 13.9618 | 14.2877 | 3 | 4 | 3 | 3 | 3 | 2 | 2 | 3 |
| STAM | Q92783;Q9 | 2.97E-08 | 1.02E-08 | -0.503708 | + | 2.77533 | -5.39136 | | 16.1134 | 16.0973 | 16.0784 | 16.5001 | 16.5563 | 16.5315 | 16.8771 | 16.5352 | 14 | 13 | 13 | 13 | 11 | 12 | 14 | 12 |
| MED24 | O75448;O7 | 0.012344 | 0.002096 | -0.503539 | + | 2.40895 | -4.54806 | | 14.067 | 13.9324 | 13.7731 | 14.3999 | 14.5924 | 14.4698 | 14.1818 | 14.4947 | 5 | 7 | 5 | 5 | 3 | 2 | 3 | 4 |
| RCN2 | Q14257;Q1 | 1.53E-09 | 6.16E-10 | -0.503293 | + | 3.35174 | -6.9366 | | 16.4757 | 16.4146 | 16.6236 | 16.988 | 17.0968 | 17.0374 | 17.0639 | 16.8536 | 11 | 12 | 13 | 12 | 12 | 13 | 12 | 12 |
| BAG4 | O95429;O9 | 3.14E-08 | 1.07E-08 | -0.503225 | + | 1.88505 | -3.48704 | | 14.3999 | 14.1118 | 14.4765 | 14.7898 | 15.0402 | 14.5197 | 14.8496 | 14.9639 | 3 | 2 | 2 | 3 | 3 | 3 | 2 | 3 |

| Gene names | UniProt IDs | Protein Group P-value | Protein Group Q-value | Log2 Difference NUP214 vs Controls (n=5) | p<0.05 | -log10 p-value | T-test Test statistic | NPC Localization | Log2 MS2_quantity_NUP214_1 | Log2 MS2_quantity_NUP214_2 | Log2 MS2_quantity_NUP214_3 | Log2 MS2_quantity_Control_1 | Log2 MS2_quantity_Control_2 | Log2 MS2_quantity_Control_3 | Log2 MS2_quantity_Control_4 | Log2 MS2_quantity_Control_5 | Number Peptides for Quantitation NUP214_1 | Number Peptides for Quantitation NUP214_2 | Number Peptides for Quantitation NUP214_3 | Number Peptides for Quantitation Control_1 | Number Peptides for Quantitation Control_2 | Number Peptides for Quantitation Control_3 | Number Peptides for Quantitation Control_4 | Number Peptides for Quantitation Control_5 |
|---|---|---|---|---|---|---|---|---|---|---|---|---|---|---|---|---|---|---|---|---|---|---|---|---|
| CDK7 | P50613 | 3.34E-06 | 9.20E-07 | -0.502532 | + | 3.13851 | -6.33009 | | 15.9628 | 15.8501 | 15.8216 | 16.4264 | 16.5019 | 16.1821 | 16.3574 | 16.4355 | 4 | 4 | 5 | 5 | 5 | 4 | 4 | 4 |
| CDK4 | P11802 | 4.21E-13 | 3.37E-13 | -0.501464 | + | 2.05691 | -3.8189 | | 18.2165 | 18.209 | 18.2452 | 18.8545 | 18.6686 | 18.3729 | 18.9368 | 18.7922 | 12 | 12 | 11 | 12 | 11 | 11 | 11 | 11 |
| C7orf25 | Q9BPX7 | 0.000223 | 5.03E-05 | -0.500968 | + | 2.64673 | -5.08446 | | 15.0065 | 15.1287 | 15.003 | 15.5902 | 15.6077 | 15.3297 | 15.4628 | 15.7448 | 3 | 4 | 3 | 3 | 2 | 3 | 3 | 2 |
| ZMAT2 | Q96NC0 | 0.002719 | 0.000514 | -0.499972 | | 0.575408 | -1.22688 | | 13.7745 | 14.0104 | 14.1945 | 13.8989 | 15.6281 | 14.457 | 14.3107 | 14.1708 | 2 | 2 | 2 | 2 | 1 | 2 | 1 | 1 |
| ERGIC1 | Q969X5;Q9 | 3.52E-07 | 1.08E-07 | -0.499594 | + | 2.93 | -5.77756 | | 18.665 | 18.7464 | 18.6605 | 19.3725 | 19.2918 | 19.074 | 19.0418 | 19.1711 | 9 | 9 | 9 | 9 | 9 | 9 | 8 | 9 |
| TMEM189 | A5PLL7;A5 | 0.000171 | 3.93E-05 | -0.499478 | | 1.12441 | -2.15014 | | 13.8201 | 13.7312 | 13.854 | 14.3325 | 13.9424 | 14.9035 | 14.3496 | 13.9783 | 1 | 1 | 1 | 1 | 1 | 2 | 1 | 1 |
| PRDX5 | P30044-2 | 1.73E-05 | 4.43E-06 | -0.498608 | | 1.027 | -1.98803 | | 20.0312 | 19.732 | 20.2179 | 19.9194 | 20.5015 | 20.4629 | 20.992 | 20.5855 | 11 | 11 | 11 | 11 | 12 | 12 | 12 | 12 |
| SMPD1 | P17405;P1 | 2.90E-05 | 7.25E-06 | -0.498428 | + | 2.16811 | -4.0416 | | 16.5395 | 16.5708 | 16.5745 | 17.212 | 16.8103 | 16.8598 | 17.2233 | 17.1948 | 9 | 9 | 10 | 9 | 8 | 9 | 10 | 9 |
| UBQLN1 | Q9UMX0;Q | 1.13E-17 | 3.54E-17 | -0.498392 | + | 2.51787 | -4.78897 | | 18.0274 | 17.8461 | 18.1925 | 18.3222 | 18.6548 | 18.4961 | 18.5629 | 18.5659 | 9 | 9 | 9 | 9 | 9 | 10 | 10 | 9 |
| CUL4B | Q13620;Q1 | 2.29E-13 | 1.97E-13 | -0.497928 | | 1.14166 | -2.17893 | | 16.8749 | 16.9717 | 16.9489 | 16.9576 | 17.5788 | 17.3095 | 17.9864 | 17.3165 | 41 | 42 | 40 | 41 | 37 | 40 | 44 | 39 |
| DUSP11 | O75319 | 0.00043 | 9.33E-05 | -0.497314 | | 0.524177 | -1.13647 | | 14.1433 | 13.8848 | 14.1026 | 14.4109 | 13.9799 | 13.9902 | 15.7575 | 14.5659 | 2 | 2 | 2 | 2 | 1 | 1 | 2 | 1 |
| ISCA1 | Q9BUE6;Q9 | 0.000104 | 2.45E-05 | -0.496254 | + | 3.48112 | -7.32676 | | 13.9617 | 14.0084 | 14.0435 | 14.4349 | 14.4345 | 14.4737 | 14.6946 | 14.4661 | 2 | 1 | 2 | 2 | 1 | 2 | 2 | 1 |
| UBL4A | P11441 | 1.38E-06 | 3.94E-07 | -0.495962 | | 0.825891 | -1.65349 | | 15.3144 | 14.8206 | 15.5505 | 15.8206 | 15.9247 | 14.9844 | 15.8064 | 16.0862 | 6 | 3 | 5 | 6 | 5 | 4 | 4 | 7 |
| SAAL1 | Q96ER3 | 0.000506 | 0.000109 | -0.495953 | | 0.807819 | -1.62325 | | 14.5967 | 14.3493 | 14.465 | 14.6684 | 15.5297 | 14.277 | 15.0224 | 15.3339 | 3 | 2 | 2 | 2 | 2 | 2 | 2 | 2 |
| MBOAT7 | Q96N66 | 2.12E-05 | 5.39E-06 | -0.49568 | | 1.2504 | -2.36133 | | 17.6068 | 17.5307 | 17.5583 | 18.2425 | 17.9521 | 17.4955 | 18.3654 | 18.2492 | 10 | 9 | 10 | 10 | 10 | 10 | 10 | 10 |
| BUD13 | Q9BRD0 | 0.020746 | 0.003449 | -0.495169 | | 0.284472 | -0.68412 | | 14.5864 | 14.6171 | 14.4019 | 14.8823 | 16.3874 | 13.3066 | 15.9583 | 14.617 | 3 | 2 | 2 | 1 | 1 | 1 | 1 | 1 |
| PPHLN1 | Q8NEY8-2;( | 2.10E-07 | 6.58E-08 | -0.495134 | | 0.878763 | -1.74169 | | 14.9419 | 14.8628 | 14.964 | 15.1476 | 15.8665 | 15.8408 | 14.7501 | 15.4852 | 5 | 4 | 4 | 3 | 4 | 2 | 2 | 3 |
| TCIRG1 | Q13488 | 1.42E-16 | 3.22E-16 | -0.494081 | + | 4.28107 | -10.1787 | | 17.0125 | 17.1112 | 17.078 | 17.4345 | 17.6095 | 17.5828 | 17.6119 | 17.5679 | 18 | 18 | 18 | 18 | 18 | 18 | 18 | 18 |
| MPZL1 | O95297;O9 | 3.56E-06 | 9.78E-07 | -0.493554 | | 1.15729 | -2.20504 | | 16.6719 | 16.5696 | 16.5755 | 17.7297 | 16.8047 | 16.8741 | 16.9545 | 17.1332 | 6 | 6 | 6 | 6 | 5 | 6 | 6 | 6 |
| TMEM131 | Q92545 | 1.75E-11 | 9.71E-12 | -0.492332 | + | 1.45865 | -2.71699 | | 14.951 | 15.075 | 14.9324 | 15.0007 | 15.8053 | 15.4553 | 15.6232 | 15.5078 | 7 | 4 | 7 | 7 | 5 | 7 | 6 | 6 |
| ACOT11 | Q8WXI4;Q8 | 0.002921 | 0.000546 | -0.491776 | + | 1.66482 | -3.08097 | | 14.3866 | 14.2942 | 14.376 | 14.9707 | 14.6018 | 14.8225 | 14.5988 | 15.2264 | 2 | 2 | 1 | 2 | 1 | 2 | 1 | 2 |
| IFIT3 | O14879 | 2.90E-14 | 3.21E-14 | -0.491173 | + | 1.62869 | -3.01617 | | 15.7659 | 15.9841 | 16.1101 | 16.2802 | 16.7373 | 16.2054 | 16.3244 | 16.6755 | 7 | 7 | 6 | 6 | 8 | 5 | 7 | 8 |
| AMOTL2 | Q9Y2J4;Q9 | 5.42E-08 | 1.80E-08 | -0.49039 | + | 1.69845 | -3.14172 | | 13.1487 | 13.4666 | 13.1609 | 13.3698 | 13.8946 | 13.9153 | 13.8675 | 13.6983 | 3 | 4 | 4 | 4 | 3 | 2 | 4 | 4 |
| ZFP36L2 | P47974 | 2.07E-10 | 9.47E-11 | -0.489682 | | 1.03051 | -1.99386 | | 15.6378 | 15.6985 | 15.6472 | 16.5778 | 16.3043 | 15.7475 | 15.6801 | 16.4446 | 2 | 2 | 3 | 3 | 2 | 2 | 2 | 2 |
| PDZD11 | Q5EBL8;Q5 | 8.10E-07 | 2.38E-07 | -0.489639 | + | 1.38273 | -2.58615 | | 14.7497 | 14.5042 | 15.0015 | 15.0489 | 15.3249 | 14.8871 | 15.4367 | 15.5096 | 3 | 4 | 4 | 3 | 3 | 4 | 3 | 4 |
| CSTB | P04080 | 2.95E-06 | 8.16E-07 | -0.489019 | + | 1.3256 | -2.48863 | | 19.9908 | 19.6581 | 20.1188 | 20.211 | 20.4283 | 20.0494 | 20.7016 | 20.6676 | 5 | 5 | 5 | 5 | 5 | 5 | 5 | 5 |
| MGAT4B | Q9UQ53;Q | 3.03E-12 | 1.95E-12 | -0.488832 | + | 2.51654 | -4.78598 | | 15.0322 | 15.0861 | 15.0565 | 15.7702 | 15.6568 | 15.3391 | 15.4459 | 15.5236 | 4 | 5 | 4 | 8 | 4 | 6 | 6 | 7 |
| CMPK1 | P30085 | 3.20E-10 | 1.41E-10 | -0.488653 | + | 3.09734 | -6.21793 | | 19.0576 | 19.0832 | 19.0257 | 19.3999 | 19.4581 | 19.5 | 19.6784 | 19.6843 | 12 | 12 | 12 | 12 | 12 | 12 | 12 | 13 |
| NQO2 | P16083 | 2.51E-09 | 9.84E-10 | -0.488127 | | 0.635271 | -1.33079 | | 15.8419 | 15.8531 | 15.8085 | 16.1212 | 17.0881 | 16.6928 | 16.2508 | 15.4602 | 6 | 6 | 6 | 6 | 7 | 8 | 8 | 4 |
| RASSF8 | Q8NHQ8;Q | 0.003364 | 0.000619 | -0.487908 | + | 2.32317 | -4.36368 | | 13.9979 | 13.9676 | 13.9068 | 14.7258 | 14.3142 | 14.5376 | 14.287 | 14.3623 | 5 | 5 | 5 | 4 | 6 | 4 | 6 | 6 |
| WDR44 | Q5JSH3 | 2.74E-12 | 1.78E-12 | -0.487346 | + | 2.53275 | -4.8225 | | 15.1094 | 15.0797 | 15.1297 | 15.8732 | 15.4413 | 15.5068 | 15.5294 | 15.6175 | 28 | 31 | 30 | 32 | 27 | 29 | 32 | 29 |
| TMEM59 | Q9BXS4 | 1.02E-08 | 3.71E-09 | -0.486795 | | 0.610578 | -1.28813 | | 15.5827 | 15.7098 | 15.7859 | 16.9358 | 15.4266 | 15.6331 | 16.4335 | 16.4689 | 6 | 6 | 5 | 7 | 5 | 6 | 6 | 6 |
| GMFB | P60983 | 0.000393 | 8.59E-05 | -0.48645 | + | 1.54709 | -2.87145 | | 17.0158 | 16.8881 | 17.2895 | 17.6748 | 17.6615 | 17.1181 | 17.6043 | 17.6958 | 6 | 6 | 6 | 7 | 8 | 6 | 7 | 8 |
| GGA3 | Q9NZ52;Q9 | 1.40E-15 | 2.30E-15 | -0.485291 | + | 1.5152 | -2.81549 | | 14.3249 | 14.313 | 14.3112 | 14.8894 | 15.2512 | 14.4998 | 14.6315 | 14.7363 | 6 | 7 | 6 | 6 | 5 | 5 | 6 | 7 |
| ITPA | Q9BY32 | 4.65E-13 | 3.67E-13 | -0.485134 | | 0.512316 | -1.11531 | | 16.1922 | 15.9778 | 16.0593 | 15.2911 | 16.66 | 16.9743 | 16.8342 | 17.0481 | 5 | 5 | 6 | 5 | 5 | 7 | 6 | 6 |
| TMEM65 | Q6PI78 | 6.80E-19 | 3.09E-18 | -0.484374 | + | 1.39258 | -2.60303 | | 16.0231 | 16.1372 | 15.9475 | 16.8669 | 16.2851 | 16.6762 | 16.126 | 16.6472 | 5 | 5 | 6 | 6 | 5 | 6 | 5 | 6 |
| ENTPD5 | O75356 | 0.002051 | 0.000401 | -0.484032 | + | 1.46513 | -2.72824 | | 13.777 | 13.9993 | 13.7633 | 14.2035 | 14.5514 | 14.2522 | 14.6749 | 13.9709 | 5 | 4 | 5 | 4 | 3 | 3 | 2 | 3 |
| UBL7 | Q96S82 | 0.005337 | 0.00095 | -0.484005 | | 0.653224 | -1.39359 | | 15.5121 | NaN | 15.2374 | 15.4687 | 15.2673 | 16.2062 | 16.165 | 16.1865 | 3 | NaN | 3 | 3 | 5 | 5 | 5 | 4 |
| MACROH2A2 | Q9P0M6 | 9.34E-07 | 2.73E-07 | -0.483954 | + | 1.553 | -2.88186 | | 17.1452 | 17.1303 | 17.203 | 17.563 | 17.6382 | 18.0125 | 17.7583 | 17.2454 | 8 | 8 | 10 | 8 | 9 | 12 | 12 | 8 |
| PIN4 | Q9Y237;Q9 | 2.54E-21 | 2.72E-20 | -0.483043 | + | 1.49489 | -2.77999 | | 16.1204 | 16.0331 | 16.1005 | 16.181 | 16.8925 | 16.7966 | 16.4002 | 16.568 | 5 | 5 | 5 | 5 | 3 | 5 | 5 | 5 |
| DIEXF | Q68CQ4 | 0.000185 | 4.22E-05 | -0.482574 | + | 2.13225 | -3.96907 | | 14.9081 | 15.1877 | 15.0123 | 15.5912 | 15.6191 | 15.2339 | 15.6833 | 15.4654 | 8 | 9 | 11 | 11 | 9 | 8 | 10 | 9 |
| LRCH2 | Q5VUJ6;Q5 | 0.000179 | 4.09E-05 | -0.481885 | | 0.723829 | -1.48192 | | 14.3315 | 14.0739 | 14.116 | 15.3174 | 14.7768 | 14.4323 | 13.8798 | 14.8723 | 4 | 2 | 3 | 4 | 4 | 3 | 3 | 3 |
| KEAP1 | Q14145 | 3.26E-08 | 1.11E-08 | -0.481186 | + | 1.57946 | -2.92859 | | 14.652 | 14.376 | 14.5254 | 15.0511 | 15.1331 | 14.5539 | 15.0472 | 15.2097 | 7 | 9 | 10 | 11 | 9 | 9 | 8 | 9 |
| SYDE1 | Q6ZW31 | 8.86E-05 | 2.11E-05 | -0.479785 | + | 1.64055 | -3.03739 | | 15.2073 | 15.2945 | 15.2729 | 15.6897 | 16.0679 | 15.4052 | 15.6003 | 15.9269 | 12 | 11 | 10 | 11 | 9 | 9 | 9 | 11 |
| GABARAPL2 | P60520 | 4.99E-10 | 2.15E-10 | -0.478025 | + | 1.6984 | -3.14164 | | 16.5585 | 16.4836 | 16.6109 | 17.4013 | 16.9082 | 16.7438 | 16.9534 | 17.1385 | 6 | 6 | 6 | 7 | 6 | 7 | 7 | 6 |
| BORCS6 | Q96GS4 | 0.002072 | 0.000405 | -0.477792 | + | 2.05441 | -3.81399 | | 14.9376 | 14.8891 | 15.2712 | 15.3425 | 15.385 | 15.5023 | 15.6786 | 15.6436 | 4 | 3 | 6 | 5 | 3 | 6 | 5 | 6 |
| ALDH1A3 | P47895 | 2.56E-24 | 7.58E-23 | -0.476982 | | 0.20571 | -0.51843 | | 14.9949 | 15.0697 | 15.2055 | 17.5339 | 15.5577 | 16.5293 | 13.6362 | 14.5781 | 6 | 5 | 2 | 23 | 18 | 22 | 4 | 5 |
| GID4 | Q8IVV7 | 1.94E-09 | 7.71E-10 | -0.476609 | | 0.984008 | -1.91662 | | 13.8986 | 13.8156 | 13.1717 | 13.8562 | 14.5144 | 13.7638 | 14.1045 | 14.2872 | 2 | 2 | 2 | 1 | 2 | 1 | 1 | 2 |
| SLC44A2 | Q8IWA5;Q8 | 7.78E-10 | 3.26E-10 | -0.474714 | | 1.14399 | -2.18283 | | 16.8254 | 16.6828 | 16.7513 | 16.8801 | 17.1165 | 17.0068 | 17.8007 | 17.3352 | 10 | 8 | 11 | 8 | 9 | 10 | 9 | 9 |
| LMBRD1 | Q9NUN5;Q | 0.002789 | 0.000525 | -0.473457 | | 0.832479 | -1.6645 | | 14.8853 | 14.7865 | 14.8382 | 15.3751 | 15.7174 | 14.8154 | 15.8164 | 14.8262 | 2 | 2 | 2 | 2 | 2 | 2 | 2 | 2 |
| COL4A1 | P02462 | 1.53E-08 | 5.44E-09 | -0.473065 | | 0.642015 | -1.3424 | | 14.6978 | 14.7407 | 14.7168 | 15.2799 | 14.323 | 15.6206 | 15.8076 | 14.9264 | 5 | 3 | 4 | 4 | 2 | 4 | 3 | 4 |
| SEC11C | Q9BY50 | 0.000427 | 9.27E-05 | -0.473049 | + | 2.22288 | -4.15378 | | 15.2567 | 15.0973 | 15.1189 | 15.6943 | 15.4487 | 15.4478 | 15.8678 | 15.6949 | 3 | 3 | 3 | 2 | 3 | 4 | 3 | 2 |
| GLIPR1 | P48060 | 1.72E-11 | 9.58E-12 | -0.472898 | | 1.21896 | -2.3084 | | 15.1622 | 15.1728 | 15.1338 | 15.4753 | 15.8711 | 15.6269 | 15.1472 | 16.0252 | 5 | 5 | 5 | 4 | 4 | 4 | 4 | 5 |
| AFAP1 | Q8N556;Q8 | 1.32E-21 | 1.49E-20 | -0.472747 | + | 1.76175 | -3.25725 | | 15.7503 | 15.7648 | 15.7652 | 15.9783 | 15.9998 | 16.2584 | 16.536 | 16.3915 | 14 | 15 | 14 | 14 | 13 | 15 | 14 | 15 |
| PPP2R2D | Q66LE6 | 6.33E-08 | 2.08E-08 | -0.472615 | | 1.29909 | -2.44362 | | 15.2067 | 15.0944 | 15.0605 | 15.4713 | 15.667 | 15.3312 | 16.117 | 15.3791 | 2 | 2 | 3 | 2 | 2 | 2 | 3 | 3 |
| TUBB3 | Q13509 | 9.14E-09 | 3.34E-09 | -0.472457 | + | 1.974 | -3.65704 | | 20.1155 | 20.2253 | 20.1678 | 20.887 | 20.6087 | 20.5043 | 20.3826 | 20.8274 | 9 | 9 | 9 | 9 | 9 | 8 | 9 | 9 |
| NOA1 | Q8NC60 | 0.002823 | 0.000531 | -0.47215 | + | 1.5265 | -2.83527 | | 15.1321 | 15.2045 | 14.949 | 15.7637 | 15.9148 | 15.3496 | 15.311 | 15.4976 | 3 | 2 | 3 | 2 | 2 | 2 | 3 | 2 |
| SLC2A10 | O95528 | 0.002433 | 0.000466 | -0.471739 | + | 2.99207 | -5.93806 | | 16.3562 | 16.185 | 16.4397 | 16.8106 | 16.9593 | 16.7289 | 16.7714 | 16.7231 | 3 | 3 | 3 | 3 | 3 | 2 | 3 | 2 |
| NAA20 | P61599 | 8.86E-08 | 2.87E-08 | -0.471684 | + | 1.54349 | -2.86512 | | 15.4785 | 15.1774 | 15.6 | 15.7798 | 16.1407 | 15.5721 | 15.8842 | 16.0747 | 4 | 4 | 4 | 4 | 3 | 4 | 4 | 4 |
| TMUB1 | Q9BVT8 | 0.000461 | 9.97E-05 | -0.470591 | + | 2.06505 | -3.83499 | | 13.6566 | 13.5999 | 13.6473 | 13.8352 | 13.9441 | 14.254 | 14.1887 | 14.3041 | 3 | 3 | 2 | 3 | 2 | 3 | 3 | 3 |
| CLIP1 | P30622-2 | 2.55E-13 | 2.18E-13 | -0.470414 | + | 2.28163 | -4.27601 | | 16.0622 | 16.0353 | 16.0401 | 16.646 | 16.4544 | 16.4767 | 16.7399 | 16.2643 | 57 | 63 | 62 | 64 | 54 | 64 | 67 | 64 |
| MAGED1 | Q9Y5V3;Q9 | 1.45E-09 | 5.85E-10 | -0.470282 | + | 1.90792 | -3.5304 | | 15.1933 | 15.1669 | 15.1612 | 15.784 | 15.4134 | 15.4491 | 15.9409 | 15.6331 | 6 | 6 | 7 | 9 | 6 | 5 | 6 | 8 |
| DCP1B | Q8IZD4 | 0.00033 | 7.28E-05 | -0.470198 | + | 1.71507 | -3.17191 | | 14.578 | 14.8526 | 14.9707 | 15.1599 | 15.5859 | 15.086 | 15.3609 | 15.1604 | 3 | 4 | 5 | 3 | 2 | 4 | 3 | 5 |
| COTL1 | Q14019 | 2.34E-08 | 8.12E-09 | -0.469227 | + | 1.65439 | -3.06221 | | 19.2258 | 19.2051 | 19.2189 | 20.1302 | 19.6624 | 19.5303 | 19.496 | 19.6102 | 14 | 14 | 14 | 15 | 14 | 14 | 13 | 14 |
| PTRHD1 | Q6GMV3 | 1.24E-10 | 5.86E-11 | -0.468512 | | 0.389965 | -0.89064 | | 14.5605 | 13.9333 | 14.3918 | 14.2764 | 13.9295 | 14.4541 | 15.0585 | 16.1002 | 1 | 2 | 3 | 4 | 2 | 3 | 5 | 6 |
| ITGB1 | P05556 | 4.49E-12 | 2.78E-12 | -0.46847 | + | 1.53865 | -2.85661 | | 19.7202 | 19.7405 | 19.6955 | 20.5677 | 20.2286 | 19.8633 | 19.9877 | 20.2887 | 28 | 27 | 27 | 27 | 28 | 27 | 28 | 28 |
| APBB2 | Q92870;Q9 | 0.000212 | 4.80E-05 | -0.468405 | | 1.00556 | -1.95241 | | 14.6678 | 15.2053 | 15.3123 | 15.4666 | 15.9751 | 15.6571 | 15.4519 | 15.1004 | 2 | 4 | 4 | 5 | 4 | 4 | 5 | 3 |
| ARSB | P15848 | 1.95E-06 | 5.48E-07 | -0.468164 | | 0.867694 | -1.72325 | | 16.4898 | 16.531 | 16.3907 | 16.5231 | 16.6642 | 17.6899 | 16.8544 | 16.9616 | 10 | 10 | 10 | 11 | 10 | 13 | 13 | 13 |
| METTL9 | Q9H1A3 | 2.45E-07 | 7.64E-08 | -0.467987 | + | 1.47535 | -2.74597 | | 15.3013 | 15.3417 | 15.418 | 16.0685 | 15.5905 | 15.472 | 16.1055 | 15.8718 | 5 | 5 | 5 | 5 | 5 | 5 | 5 | 5 |
| HYI | Q5T013 | 0.000188 | 4.28E-05 | -0.467837 | + | 1.85889 | -3.43775 | | 15.4111 | 15.2876 | 15.6139 | 16.098 | 15.9433 | 15.6524 | 15.7565 | 16.0767 | 4 | 4 | 6 | 4 | 4 | 4 | 5 | 3 |
| RHOBTB3 | O94955 | 1.75E-06 | 4.94E-07 | -0.467505 | | 1.1125 | -2.21947 | | 14.2373 | 14.3581 | 14.136 | 15.1 | 14.8685 | 14.5622 | NaN | 14.3144 | 7 | 6 | 6 | 8 | 5 | 7 | NaN | 5 |
| TECPR1 | Q7Z6L1;Q7 | 0.002519 | 0.000481 | -0.467345 | | 0.817199 | -1.68738 | | 14.457 | 14.8025 | 14.6624 | 15.0434 | NaN | 15.1729 | 15.6503 | 14.5652 | 4 | 4 | 4 | 4 | NaN | 5 | 2 | 4 |
| VANGL1 | Q8TAA9;Q8 | 6.85E-07 | 2.03E-07 | -0.464955 | | 1.09319 | -2.09811 | | 13.51 | 13.769 | 13.7819 | 14.6758 | 13.8125 | 14.2673 | 13.8325 | 14.1715 | 5 | 5 | 5 | 7 | 5 | 5 | 6 | 5 |
| ITM2B | Q9Y287 | 2.40E-12 | 1.58E-12 | -0.464828 | | 1.09806 | -2.10623 | | 16.2052 | 16.3664 | 16.4268 | 16.9362 | 16.4922 | 16.3614 | 16.9632 | 17.2352 | 4 | 5 | 5 | 6 | 4 | 5 | 6 | 6 |
| MCRIP1 | C9JLW8 | 1.42E-05 | 3.66E-06 | -0.464637 | | 0.426665 | -0.95942 | | 15.7286 | 15.6542 | 16.2122 | 16.7916 | 15.7881 | 15.2982 | 16.5196 | 17.2508 | 4 | 3 | 5 | 6 | 4 | 3 | 6 | 6 |
| GALNT11 | Q8NCW6 | 0.037404 | 0.006112 | -0.464296 | | 1.0811 | -2.07799 | | 15.0095 | 14.6096 | 14.9458 | 15.444 | 15.5937 | 14.8635 | 15.6384 | 15.0567 | 2 | 1 | 2 | 2 | 1 | 2 | 2 | 1 |
| NTM | Q9P121;Q9 | 0.002365 | 0.000455 | -0.464291 | | 0.558353 | -1.19695 | | 14.5687 | 14.6711 | 14.6648 | 14.7987 | 16.232 | 14.9465 | 14.9299 | 14.5887 | 1 | 1 | 1 | 2 | 2 | 1 | 1 | 2 |
| MFSD12 | Q6NUT3;Q( | 0.016743 | 0.002804 | -0.463588 | + | 1.35677 | -2.54174 | | 13.9481 | 14.2072 | 14.197 | 14.2535 | 14.6363 | 14.6979 | 14.971 | 14.3463 | 2 | 2 | 2 | 1 | 2 | 2 | 2 | 2 |

| Gene names | UniProt IDs | Protein Group P-value | Protein Group Q-value | Log2 Difference NUP214 vs Controls (n=5) | p<0.05 | -log10 p-value | T-test Test statistic | NPC Localization | Log2 MS2_quantity_NUP214_1 | Log2 MS2_quantity_NUP214_2 | Log2 MS2_quantity_NUP214_3 | Log2 MS2_quantity_Control_1 | Log2 MS2_quantity_Control_2 | Log2 MS2_quantity_Control_3 | Log2 MS2_quantity_Control_4 | Log2 MS2_quantity_Control_5 | Number Peptides for Quantitation NUP214_1 | Number Peptides for Quantitation NUP214_2 | Number Peptides for Quantitation NUP214_3 | Number Peptides for Quantitation Control_1 | Number Peptides for Quantitation Control_2 | Number Peptides for Quantitation Control_3 | Number Peptides for Quantitation Control_4 | Number Peptides for Quantitation Control_5 |
|---|---|---|---|---|---|---|---|---|---|---|---|---|---|---|---|---|---|---|---|---|---|---|---|---|
| LYPLA2 | O95372 | 6.19E-13 | 4.72E-13 | -0.463426 | + | 1.9707 | -3.65067 | | 15.6491 | 15.3589 | 15.7671 | 15.945 | 15.9261 | 15.9995 | 16.1083 | 16.2967 | 7 | 7 | 7 | 7 | 8 | 7 | 8 | 8 |
| EXTL3 | O43909 | 0.001872 | 0.000369 | -0.462982 | | 1.13838 | -2.17346 | | 14.497 | 14.85 | 14.681 | 14.9512 | 15.1801 | 14.6815 | 15.5397 | 15.3424 | 1 | 1 | 2 | 2 | 1 | 1 | 2 | 2 |
| RIPOR1 | Q6ZS17;Q6 | 4.02E-11 | 2.07E-11 | -0.462982 | + | 2.07705 | -3.85875 | | 15.4367 | 15.5049 | 15.4867 | 16.1132 | 15.9678 | 15.6484 | 15.8417 | 16.1244 | 21 | 22 | 20 | 24 | 23 | 20 | 26 | 24 |
| CASTOR2 | A6NHX0 | 0.002206 | 0.000428 | -0.462524 | + | 1.4338 | -2.67399 | | 15.4107 | 15.404 | 15.2427 | 15.8693 | 16.1613 | 15.466 | 15.5989 | 15.9796 | 6 | 7 | 7 | 7 | 7 | 6 | 6 | 7 |
| HSPB8 | Q9UJY1 | 8.18E-13 | 6.02E-13 | -0.461162 | | 1.10658 | -2.12042 | | 16.3599 | 16.2885 | 16.4662 | 16.6947 | 16.4362 | 17.3532 | 16.6542 | 17.0251 | 6 | 7 | 9 | 8 | 6 | 8 | 6 | 8 |
| NEXN | Q0ZGT2 | 6.42E-18 | 2.21E-17 | -0.45976 | | 0.721482 | -1.47795 | | 15.6935 | 15.7252 | 15.7757 | 16.5851 | 15.3006 | 16.371 | 16.5145 | 16.1848 | 21 | 22 | 21 | 26 | 19 | 24 | 26 | 25 |
| CCPG1 | Q9ULG6;Q9 | 6.41E-19 | 2.96E-18 | -0.459423 | | 1.15347 | -2.19867 | | 14.2702 | 14.2577 | 14.298 | 14.5719 | 14.3712 | 14.6285 | 15.2963 | 14.8058 | 6 | 6 | 7 | 8 | 4 | 7 | 11 | 8 |
| LAMP2 | P13473 | 0.001151 | 0.000236 | -0.459237 | | 1.30041 | -2.44586 | | 19.7558 | 19.8873 | 19.972 | 20.1621 | 20.0936 | 20.4419 | 20.8193 | 20.1379 | 8 | 8 | 7 | 8 | 8 | 7 | 7 | 8 |
| NCEH1 | Q6PIU2 | 2.47E-06 | 6.89E-07 | -0.458493 | | 0.927486 | -1.82273 | | 17.2133 | 17.1221 | 17.1601 | 17.8648 | 18.1611 | 17.1589 | 17.2483 | 17.6852 | 14 | 15 | 15 | 15 | 14 | 13 | 13 | 14 |
| CHMP2B | Q9UQN3 | 1.02E-14 | 1.29E-14 | -0.458308 | | 1.09763 | -2.1055 | | 16.383 | 16.0584 | 16.241 | 17.1877 | 16.4384 | 16.7264 | 16.2915 | 16.7848 | 3 | 4 | 2 | 2 | 3 | 4 | 2 | 4 |
| COMMD5 | Q9GZQ3 | 1.87E-07 | 5.89E-08 | -0.458179 | | 1.08617 | -2.08643 | | 15.8187 | 15.5966 | 15.9981 | 15.8451 | 16.2303 | 16.0541 | 16.7059 | 16.4778 | 8 | 7 | 8 | 8 | 8 | 8 | 8 | 8 |
| RPSA | P08865 | 6.87E-12 | 4.13E-12 | -0.457691 | + | 2.77417 | -5.38854 | | 21.1257 | 20.9721 | 21.2921 | 21.4493 | 21.5619 | 21.6702 | 21.6304 | 21.6266 | 14 | 14 | 14 | 13 | 13 | 14 | 14 | 14 |
| SEPTIN10 | Q9P0V9;Q9 | 4.55E-20 | 3.20E-19 | -0.457184 | | 1.15334 | -2.19844 | | 14.9804 | 14.9295 | 14.9077 | 15.9624 | 15.0979 | 15.1275 | 15.3655 | 15.4285 | 8 | 9 | 8 | 9 | 6 | 9 | 9 | 9 |
| MTRR | Q9UBK8;Q9 | 1.64E-12 | 1.12E-12 | -0.457084 | + | 1.47786 | -2.75033 | | 14.1558 | 14.1044 | 14.0864 | 14.8064 | 14.4629 | 14.1616 | 14.5911 | 14.8412 | 5 | 8 | 5 | 9 | 5 | 6 | 5 | 5 |
| TAX1BP1 | Q86VP1;Q8 | 6.21E-09 | 2.32E-09 | -0.456859 | + | 1.55021 | -2.87693 | | 15.1315 | 15.1787 | 15.1873 | 16.0642 | 15.4234 | 15.4146 | 15.5648 | 15.6463 | 9 | 11 | 11 | 13 | 7 | 9 | 11 | 12 |
| FAIM | Q9NVQ4 | 0.000109 | 2.56E-05 | -0.456799 | + | 3.3451 | -6.91704 | | 15.2834 | 15.2998 | 15.2521 | 15.7351 | 15.5966 | 15.6734 | 15.7869 | 15.8841 | 2 | 2 | 3 | 2 | 2 | 2 | 2 | 2 |
| CLPTM1 | O96005;O9 | 9.29E-09 | 3.39E-09 | -0.456684 | + | 2.31371 | -4.34363 | | 16.5422 | 16.5147 | 16.4844 | 17.1717 | 16.9341 | 16.6977 | 17.0061 | 17.0428 | 11 | 12 | 11 | 12 | 12 | 11 | 11 | 11 |
| BIN3 | Q9NQY0 | 4.04E-06 | 1.10E-06 | -0.456026 | | 1.25904 | -2.37589 | | 15.5287 | 15.5546 | 15.4909 | 16.3172 | 16.2734 | 15.5385 | 15.8722 | 15.9023 | 4 | 5 | 5 | 7 | 6 | 6 | 6 | 6 |
| FZD6 | O60353;O6 | 0.004467 | 0.000805 | -0.455948 | | 0.767567 | -1.55571 | | 14.4457 | 14.7167 | 14.7036 | 15.4903 | 14.9832 | 14.639 | 14.6187 | 15.6585 | 2 | 2 | 3 | 4 | 3 | 3 | 4 | 4 |
| RIF1 | Q5UIP0;Q5 | 0.002101 | 0.00041 | -0.455886 | | 0.72297 | -1.48047 | | 13.774 | 14.2288 | 13.9601 | 14.8126 | 14.6534 | 14.0279 | 13.8161 | 14.9077 | 2 | 4 | 2 | 4 | 1 | 1 | 4 | 5 |
| TMBIM1 | Q969X1 | 9.87E-06 | 2.59E-06 | -0.455476 | + | 1.31287 | -2.467 | | 17.843 | 18.0419 | 17.9282 | 18.8041 | 18.2773 | 18.1975 | 18.6062 | 18.0807 | 2 | 2 | 2 | 2 | 2 | 2 | 2 | 2 |
| PTAR1 | Q7Z6K3 | 4.62E-11 | 2.36E-11 | -0.45464 | + | 3.10576 | -6.24074 | | 15.0791 | 15.1444 | 15.1276 | 15.6302 | 15.6542 | 15.3837 | 15.6684 | 15.5219 | 6 | 6 | 9 | 7 | 6 | 6 | 7 | 7 |
| PCDHGA5 | Q9Y5G8 | 2.41E-08 | 8.34E-09 | -0.454566 | + | 1.31179 | -2.46516 | | 16.0148 | 15.9192 | 16.0788 | 16.5955 | 16.755 | 15.968 | 16.3864 | 16.5893 | 2 | 3 | 4 | 4 | 4 | 4 | 5 | 4 |
| HEATR5A | Q86XA9 | 2.56E-24 | 7.58E-23 | -0.454486 | | 1.00039 | -1.94383 | | 14.703 | 14.6542 | 14.6845 | 14.6482 | 15.0043 | 15.4687 | 15.5996 | 14.9545 | 18 | 19 | 23 | 23 | 15 | 27 | 26 | 19 |
| UTP4 | Q969X6 | 5.82E-10 | 2.48E-10 | -0.454112 | + | 2.38345 | -4.49276 | | 15.4018 | 15.2533 | 15.3273 | 15.8247 | 15.8829 | 15.5135 | 15.765 | 15.9218 | 10 | 9 | 11 | 10 | 10 | 11 | 12 | 10 |
| PHYH | O14832 | 4.66E-08 | 1.56E-08 | -0.453869 | | 0.580196 | -1.23525 | | 14.542 | 15.2891 | 14.3153 | 14.9736 | 15.4235 | 14.4106 | 15.714 | 15.3251 | 4 | 2 | 3 | 2 | 4 | 1 | 3 | 4 |
| FHL1 | Q13642 | 1.44E-13 | 1.31E-13 | -0.453578 | | 0.466299 | -1.03228 | | 18.5936 | 18.6626 | 18.6242 | 18.0517 | 19.5726 | 19.3506 | 18.5968 | 19.8302 | 14 | 13 | 14 | 13 | 14 | 15 | 12 | 15 |
| ITPRID2 | P28290;P2 | 1.38E-06 | 3.94E-07 | -0.4533 | + | 2.42719 | -4.58786 | | 15.052 | 15.1492 | 15.2017 | 15.7152 | 15.6404 | 15.3597 | 15.7257 | 15.4969 | 11 | 7 | 4 | 10 | 7 | 4 | 12 | 9 |
| SGSH | P51688 | 3.01E-11 | 1.59E-11 | -0.452609 | + | 2.0864 | -3.87734 | | 16.8062 | 16.7607 | 16.8248 | 16.9669 | 17.2605 | 17.2806 | 17.5135 | 17.2276 | 15 | 14 | 14 | 14 | 15 | 14 | 13 | 14 |
| ZFAND6 | Q6FIF0;Q6I | 2.61E-11 | 1.40E-11 | -0.452444 | | 0.787729 | -1.58958 | | 14.8009 | 14.9491 | 14.512 | 15.7023 | 15.3451 | 14.4757 | 15.1878 | 15.3211 | 2 | 2 | 2 | 3 | 2 | 2 | 2 | 2 |
| ALPK2 | Q86TB3 | 0.00349 | 0.00064 | -0.452283 | | 0.521378 | -1.15299 | | 13.3451 | 12.5532 | 13.3457 | 13.2541 | NaN | 14.0949 | 12.9092 | 13.8763 | 2 | 2 | 1 | 2 | NaN | 1 | 5 | 1 |
| MRGPRF | Q96AM1 | 2.00E-06 | 5.63E-07 | -0.451722 | | 1.15872 | -2.20744 | | 17.0717 | 17.0033 | 16.8814 | 17.5421 | 17.6913 | 16.8579 | 17.4546 | 17.6398 | 2 | 2 | 2 | 2 | 2 | 2 | 2 | 2 |
| KLHL9 | Q9P2J3 | 8.92E-12 | 5.23E-12 | -0.45042 | + | 1.86097 | -3.44165 | | 14.5827 | 14.5659 | 14.4896 | 15.2654 | 15.0397 | 14.6704 | 14.9437 | 15.0631 | 6 | 6 | 5 | 6 | 4 | 4 | 3 | 3 |
| STBD1 | O95210 | 1.05E-14 | 1.32E-14 | -0.449884 | | 1.1175 | -2.13863 | | 15.4599 | 15.5494 | 15.4627 | 16.485 | 15.7841 | 15.7175 | 15.6253 | 16.091 | 7 | 9 | 7 | 10 | 7 | 8 | 8 | 8 |
| FCHSD1 | Q86WN1 | 0.000218 | 4.93E-05 | -0.449763 | | 1.21804 | -2.30686 | | 15.0484 | 15.0359 | 15.0889 | 15.5269 | 15.9869 | 15.2715 | 15.1463 | 15.6059 | 4 | 5 | 6 | 5 | 6 | 6 | 3 | 5 |
| SLC33A1 | O00400 | 2.48E-13 | 2.12E-13 | -0.449099 | + | 3.61134 | -7.73757 | | 15.9393 | 16.0299 | 15.9296 | 16.4088 | 16.3124 | 16.4234 | 16.5554 | 16.3768 | 8 | 7 | 8 | 9 | 6 | 8 | 10 | 8 |
| GLMP | Q8WWB7;( | 5.69E-07 | 1.70E-07 | -0.448609 | + | 1.87171 | -3.46186 | | 17.579 | 17.9552 | 17.9004 | 18.1439 | 18.2498 | 18.2387 | 18.5357 | 18.1325 | 3 | 3 | 3 | 3 | 3 | 3 | 3 | 3 |
| DCTN5 | Q9BTE1;Q9 | 1.74E-07 | 5.50E-08 | -0.448433 | + | 3.58431 | -7.65076 | | 14.7774 | 14.7619 | 14.7098 | 15.1394 | 15.1826 | 15.0991 | 15.3465 | 15.2233 | 2 | 2 | 2 | 1 | 2 | 1 | 1 | 1 |
| CCNK | O75909;O7 | 0.00042 | 9.14E-05 | -0.448092 | + | 2.4201 | -4.57236 | | 14.1366 | 14.0656 | 14.2392 | 14.3968 | 14.6844 | 14.4674 | 14.7366 | 14.6909 | 5 | 4 | 5 | 4 | 5 | 3 | 4 | 5 |
| NFAT5 | O94916;O9 | 3.42E-07 | 1.05E-07 | -0.447253 | + | 1.35763 | -2.54321 | | 13.9698 | 14.0003 | 13.9227 | 14.4517 | 14.6351 | 13.9851 | 14.7145 | 14.2713 | 6 | 6 | 6 | 6 | 6 | 5 | 6 | 4 |
| SLC4A2 | P04920;P0 | 1.50E-10 | 7.00E-11 | -0.446938 | + | 1.51876 | -2.82171 | | 14.5361 | 14.2166 | 14.3645 | 14.9916 | 14.5883 | 14.6076 | 15.1371 | 14.772 | 3 | 5 | 4 | 7 | 2 | 3 | 5 | 7 |
| MID1 | O15344 | 7.78E-14 | 7.66E-14 | -0.446438 | + | 1.36812 | -2.56114 | | 15.0681 | 14.9909 | 14.9208 | 15.2268 | 15.9115 | 15.4932 | 15.3593 | 15.2079 | 8 | 6 | 4 | 5 | 4 | 5 | 7 | 4 |
| SGK3 | Q96BR1;Q9 | 6.63E-11 | 3.29E-11 | -0.446417 | | 0.925032 | -1.81866 | | 14.0879 | 13.9579 | 14.091 | 14.3582 | 15.02 | 13.9279 | 14.4412 | 14.7129 | 3 | 3 | 3 | 3 | 3 | 2 | 3 | 3 |
| WASHC3 | Q9Y3C0 | 1.17E-09 | 4.82E-10 | -0.446075 | | 1.1483 | -2.19003 | | 15.9962 | 15.7823 | 16.1913 | 16.1214 | 16.1798 | 16.8839 | 16.5692 | 16.4258 | 5 | 5 | 5 | 5 | 3 | 5 | 5 | 5 |
| AKAP13 | Q12802 | 1.21E-09 | 4.96E-10 | -0.445586 | + | 2.03499 | -3.77578 | | 14.9285 | 14.9047 | 14.8731 | 15.3048 | 15.6095 | 15.0667 | 15.4227 | 15.3347 | 20 | 20 | 23 | 21 | 16 | 21 | 21 | 20 |
| UCKL1 | Q9NWZ5 | 8.46E-05 | 2.02E-05 | -0.445426 | + | 2.81235 | -5.48205 | | 15.1928 | 15.4129 | 15.3547 | 15.7918 | 15.9334 | 15.6462 | 15.694 | 15.7625 | 7 | 7 | 6 | 6 | 6 | 6 | 6 | 7 |
| FAM20B | O75063 | 2.58E-10 | 1.16E-10 | -0.445062 | + | 4.07988 | -9.38266 | | 15.5365 | 15.5932 | 15.5917 | 16.091 | 15.9027 | 15.983 | 16.0531 | 16.0645 | 5 | 6 | 8 | 6 | 6 | 6 | 7 | 8 |
| CALU | O43852-2;( | 5.81E-13 | 4.47E-13 | -0.444664 | | 0.5845 | -1.24277 | | 16.8967 | 16.9158 | 16.9434 | 17.1284 | 16.439 | 17.982 | 17.6145 | 17.6527 | 3 | 3 | 3 | 3 | 3 | 3 | 4 | 3 |
| TXN | P10599 | 1.22E-06 | 3.52E-07 | -0.443659 | + | 1.68307 | -3.11389 | | 20.3985 | 20.4974 | 20.4052 | 20.9658 | 20.5948 | 20.7444 | 21.2189 | 20.8628 | 7 | 7 | 7 | 7 | 8 | 8 | 8 | 8 |
| TVP23C;TVP23B | Q96ET8-3;( | 0.017875 | 0.002987 | -0.44362 | | 0.586465 | -1.2462 | | 13.9172 | 14.397 | 15.1047 | 14.9242 | 15.0826 | 14.4838 | 15.5274 | 14.5649 | 1 | 2 | 1 | 2 | 1 | 1 | 1 | 1 |
| CYP2U1 | Q7Z449 | 1.76E-05 | 4.50E-06 | -0.442806 | | 0.990322 | -1.92711 | | 14.9322 | 15.0172 | 15.0507 | 15.1542 | 15.7821 | 15.537 | 15.7998 | 14.941 | 3 | 3 | 3 | 3 | 3 | 3 | 3 | 3 |
| CKAP4 | Q07065 | 1.48E-08 | 5.28E-09 | -0.442746 | + | 2.13737 | -3.97939 | | 20.6715 | 20.6496 | 20.6369 | 20.9401 | 21.3495 | 20.9483 | 21.2362 | 21.0031 | 44 | 43 | 45 | 45 | 45 | 45 | 45 | 45 |
| ADCY6 | O43306;O4 | 0.030761 | 0.005052 | -0.441803 | | 0.61005 | -1.35997 | | NaN | 13.4589 | 14.0094 | 14.2874 | 14.6485 | 13.9351 | NaN | 13.8329 | NaN | 1 | 1 | 1 | 1 | 1 | NaN | 1 |
| MEX3D | Q86XN8;Q8 | 0.003619 | 0.000662 | -0.441802 | | 0.800876 | -1.61162 | | 13.6394 | 14.3765 | 14.3346 | 14.6695 | 15.0788 | 14.3459 | 14.1409 | 14.558 | 2 | 3 | 3 | 3 | 2 | 3 | 5 | 2 |
| DBN1 | Q16643;Q1 | 1.92E-17 | 5.58E-17 | -0.441542 | + | 1.49617 | -2.78223 | | 18.2924 | 18.3588 | 18.343 | 19.1885 | 18.6609 | 18.7852 | 18.4639 | 18.7661 | 25 | 23 | 24 | 25 | 23 | 26 | 24 | 25 |
| N6AMT1 | Q9Y5N5 | 0.001473 | 0.000297 | -0.441538 | | 0.897619 | -1.77307 | | 16.2001 | 15.9389 | 16.1876 | 16.0559 | 16.718 | 16.2841 | 16.5875 | 17.1065 | 2 | 2 | 2 | 2 | 2 | 2 | 2 | 2 |
| LAMB2 | P55268 | 7.86E-16 | 1.39E-15 | -0.441291 | | 0.99541 | -1.93556 | | 16.1646 | 16.1795 | 16.1629 | 15.9938 | 16.9072 | 16.8632 | 16.802 | 16.4853 | 40 | 39 | 42 | 40 | 39 | 42 | 43 | 40 |
| AK3 | Q9UIJ7 | 3.29E-10 | 1.44E-10 | -0.440313 | + | 3.08956 | -6.19692 | | 17.0329 | 16.9108 | 16.9748 | 17.3994 | 17.3871 | 17.311 | 17.6024 | 17.3659 | 19 | 19 | 19 | 20 | 19 | 18 | 19 | 19 |
| EFEMP2 | O95967 | 1.62E-10 | 7.55E-11 | -0.440287 | | 1.05025 | -2.02666 | | 14.4696 | 14.4494 | 14.4719 | 14.8352 | 14.7873 | 14.4991 | 15.4942 | 14.9037 | 3 | 3 | 3 | 4 | 4 | 5 | 3 | 4 |
| SPR | P35270 | 4.49E-07 | 1.36E-07 | -0.440121 | + | 2.60169 | -4.97985 | | 16.3489 | 16.3013 | 16.3885 | 16.9323 | 16.8487 | 16.6174 | 16.6451 | 16.8883 | 8 | 8 | 8 | 9 | 8 | 7 | 7 | 8 |
| SLC25A4 | P12235 | 3.98E-17 | 1.06E-16 | -0.439924 | + | 1.66563 | -3.08244 | | 16.8958 | 16.9394 | 16.9819 | 17.6734 | 17.0249 | 17.3235 | 17.3936 | 17.4794 | 5 | 4 | 5 | 5 | 5 | 5 | 5 | 5 |
| LSM12 | Q3MHD2;Q | 2.92E-08 | 1.01E-08 | -0.439511 | + | 2.0482 | -3.80175 | | 16.9718 | 17.0175 | 17.1472 | 17.5651 | 17.5177 | 17.2023 | 17.6964 | 17.4434 | 4 | 4 | 4 | 4 | 4 | 4 | 4 | 4 |
| CLCC1 | Q96S66 | 5.02E-12 | 3.08E-12 | -0.439303 | + | 3.4674 | -7.28456 | | 15.6745 | 15.7335 | 15.7526 | 16.1647 | 16.1268 | 16.0345 | 16.3038 | 16.1677 | 16 | 16 | 16 | 17 | 14 | 17 | 17 | 17 |
| TXNDC12 | O95881 | 1.64E-08 | 5.80E-09 | -0.438765 | | 1.19881 | -2.27458 | | 18.0486 | 17.6885 | 18.3353 | 18.2669 | 18.1777 | 18.6474 | 18.6845 | 18.5381 | 6 | 7 | 6 | 6 | 6 | 7 | 7 | 7 |
| FHL2 | Q14192 | 9.81E-09 | 3.57E-09 | -0.438273 | | 1.06907 | -2.05797 | | 18.8842 | 18.9073 | 18.8658 | 18.9434 | 19.2476 | 19.8479 | 19.493 | 19.0882 | 19 | 21 | 21 | 20 | 20 | 21 | 20 | 20 |
| ZDHHC3 | Q9NYG2;Q9 | 0.000159 | 3.66E-05 | -0.438235 | + | 1.35369 | -2.53649 | | 14.7062 | 14.8859 | 14.9943 | 15.2224 | 15.7282 | 15.0044 | 15.3609 | 15.1859 | 2 | 2 | 2 | 2 | 2 | 2 | 2 | 2 |
| ISYNA1 | Q9NPH2;Q9 | 8.38E-05 | 2.00E-05 | -0.438021 | | 0.544142 | -1.17188 | | 15.3466 | 15.363 | 15.4436 | 16.0466 | 16.1363 | 15.0539 | 16.5703 | 15.305 | 6 | 5 | 4 | 5 | 5 | 4 | 6 | 4 |
| EHBP1L1 | Q8N3D4 | 2.38E-15 | 3.66E-15 | -0.437109 | + | 2.22461 | -4.15735 | | 15.5277 | 15.4381 | 15.4677 | 16.1179 | 15.9793 | 15.8755 | 15.6471 | 15.9548 | 24 | 27 | 26 | 29 | 27 | 28 | 24 | 26 |
| HSF1 | Q00613;Q0 | 0.000582 | 0.000124 | -0.436875 | + | 1.80526 | -3.33762 | | 16.2131 | 15.9385 | 16.1782 | 16.7157 | 16.68 | 16.4995 | 16.6027 | 16.2362 | 3 | 2 | 3 | 2 | 2 | 2 | 2 | 2 |
| RAPGEF6 | Q8TEU7;Q8 | 2.35E-13 | 2.02E-13 | -0.435879 | + | 1.31652 | -2.4732 | | 14.3348 | 14.2829 | 14.3093 | 14.5321 | 14.6346 | 14.8576 | 14.4925 | 15.2078 | 9 | 6 | 4 | 6 | 3 | 2 | 4 | 4 |
| MED15 | Q96RN5;Q9 | 1.31E-05 | 3.41E-06 | -0.435854 | + | 1.65175 | -3.05748 | | 15.0505 | 14.7256 | 14.7995 | 15.298 | 15.307 | 14.9527 | 15.4756 | 15.4386 | 2 | 4 | 4 | 4 | 2 | 4 | 4 | 4 |
| RSPRY1 | Q96DX4 | 2.39E-11 | 1.29E-11 | -0.434363 | + | 1.36746 | -2.56 | | 14.7704 | 14.5298 | 14.5264 | 14.6715 | 15.2825 | 14.8679 | 15.2719 | 15.1224 | 4 | 4 | 3 | 4 | 4 | 4 | 3 | 4 |
| FKBP11 | Q9NYL4;Q9 | 6.42E-06 | 1.72E-06 | -0.434019 | | 0.767998 | -1.55643 | | 17.5201 | 17.5085 | 17.4464 | 17.6121 | 17.5202 | 18.1673 | 18.6316 | 17.6972 | 6 | 6 | 6 | 6 | 5 | 6 | 6 | 6 |
| CHURC1 | Q8WUH1;Q | 0.005065 | 0.000905 | -0.432535 | + | 1.68649 | -3.12006 | | 16.1718 | 15.9375 | 16.2783 | 16.3549 | 16.3604 | 16.5985 | 16.7768 | 16.7182 | 2 | 2 | 2 | 2 | 2 | 2 | 2 | 2 |
| FTSJ3 | Q8IY81 | 2.91E-12 | 1.88E-12 | -0.432362 | + | 3.05814 | -6.11258 | | 14.9154 | 15.0882 | 14.7684 | 15.3213 | 15.3979 | 15.3567 | 15.385 | 15.3209 | 13 | 16 | 16 | 15 | 13 | 15 | 14 | 12 |
| SPRYD7 | Q5W111 | 4.76E-06 | 1.29E-06 | -0.432344 | + | 2.53047 | -4.81736 | | 15.9732 | 16.0913 | 16.0495 | 16.6062 | 16.3723 | 16.4577 | 16.6234 | 16.292 | 5 | 5 | 5 | 5 | 5 | 5 | 5 | 5 |
| TANGO2 | Q6ICL3;Q6 | 9.53E-06 | 2.51E-06 | -0.432294 | + | 1.92269 | -3.55853 | | 14.9677 | 15.1301 | 14.8322 | 15.5578 | 15.135 | 15.4228 | 15.3698 | 15.5594 | 4 | 3 | 3 | 4 | 4 | 5 | 5 | 4 |
| GPAM | Q9HCL2 | 3.98E-06 | 1.09E-06 | -0.4322 | + | 1.92272 | -3.55858 | | 14.6279 | 14.8111 | 15.0382 | 15.433 | 15.1085 | 15.1701 | 15.386 | 15.1921 | 5 | 4 | 5 | 5 | 4 | 6 | 4 | 5 |

| Gene names | UniProt IDs | Protein Group P-value | Protein Group Q-value | Log2 Difference NUP214 vs Controls (n=5) | p<0.05 | -log10 p-value | T-test Test statistic | NPC Localization | Log2 MS2_quantity_ NUP214_1 | Log2 MS2_quantity_ NUP214_2 | Log2 MS2_quantity_ NUP214_3 | Log2 MS2_quantity_ Control_1 | Log2 MS2_quantity_ Control_2 | Log2 MS2_quantity_ Control_3 | Log2 MS2_quantity_ Control_4 | Log2 MS2_quantity_ Control_5 | Number Peptides for Quantitation NUP214_1 | Number Peptides for Quantitation NUP214_2 | Number Peptides for Quantitation NUP214_3 | Number Peptides for Quantitation Control_1 | Number Peptides for Quantitation Control_2 | Number Peptides for Quantitation Control_3 | Number Peptides for Quantitation Control_4 | Number Peptides for Quantitation Control_5 |
|---|---|---|---|---|---|---|---|---|---|---|---|---|---|---|---|---|---|---|---|---|---|---|---|---|
| MCEE | Q96PE7 | 0.000455 | 9.83E-05 | -0.431713 | + | 1.56599 | -2.90477 | | 15.8836 | 15.8866 | 16.026 | 15.9797 | 16.5581 | 16.562 | 16.2865 | 16.4325 | 2 | 3 | 3 | 2 | 2 | 3 | 3 | 3 |
| CALD1 | Q05682;Q( | 1.60E-15 | 2.58E-15 | -0.431563 | | 1.15738 | -2.20521 | | 19.4033 | 19.407 | 19.4332 | 19.3341 | 19.9485 | 20.0474 | 20.1688 | 19.7316 | 56 | 56 | 59 | 56 | 56 | 60 | 59 | 59 |
| UBAC2 | Q8NBM4 | 0.001168 | 0.000239 | -0.430786 | + | 1.67957 | -3.10757 | | 15.6527 | 15.5013 | 15.6155 | 15.9556 | 16.3339 | 15.7145 | 15.9947 | 16.1044 | 4 | 5 | 4 | 2 | 2 | 3 | 4 | 3 |
| SCAMP4 | Q969E2;Q9 | 0.003333 | 0.000614 | -0.430691 | | 0.739975 | -1.50921 | | 16.9065 | 17.4564 | 17.2969 | 17.7427 | 17.5033 | 17.7551 | 18.2234 | 17.0286 | 3 | 3 | 3 | 3 | 3 | 3 | 3 | 3 |
| LSM3 | P62310 | 8.02E-11 | 3.91E-11 | -0.430657 | | 0.603909 | -1.27656 | | 17.7353 | 17.0503 | 17.8168 | 17.659 | 17.6116 | 17.7386 | 18.0435 | 18.7712 | 2 | 2 | 2 | 2 | 2 | 2 | 2 | 2 |
| FBXL12 | Q9NXK8;Q9 | 0.001149 | 0.000235 | -0.43054 | | 0.630192 | -1.35197 | | 14.197 | 13.9574 | 14.0876 | 15.0041 | 14.5569 | NaN | 13.7674 | 14.7165 | 3 | 3 | 4 | 4 | 3 | NaN | 1 | 2 |
| VIM | P08670 | 4.37E-14 | 4.60E-14 | -0.43042 | + | 1.58159 | -2.93236 | | 25.0979 | 25.1242 | 25.0882 | 25.3995 | 25.9491 | 25.5352 | 25.3174 | 25.468 | 70 | 71 | 71 | 71 | 71 | 71 | 72 | 72 |
| NEK3 | P51956;P5 | 0.001791 | 0.000355 | -0.430266 | | 1.04148 | -2.0121 | | 13.2124 | 13.4301 | 13.2145 | 13.5037 | 13.966 | 13.2598 | 13.7249 | 14.1252 | 3 | 4 | 4 | 3 | 2 | 3 | 2 | 3 |
| FBXO28 | Q9NVF7 | 0.000819 | 0.000171 | -0.430127 | | 0.777625 | -1.57262 | | 14.019 | 13.7973 | 14.0103 | 14.4238 | 14.8516 | 13.6278 | 14.4948 | 14.4636 | 3 | 3 | 2 | 3 | 4 | 1 | 5 | 4 |
| TTPAL | Q9BTX7 | 6.86E-17 | 1.71E-16 | -0.429957 | + | 1.88536 | -3.48762 | | 15.1538 | 15.1142 | 15.0446 | 15.8049 | 15.4504 | 15.4048 | 15.3235 | 15.6872 | 8 | 7 | 7 | 7 | 7 | 7 | 7 | 7 |
| RIPK3 | Q9Y572 | 0.015247 | 0.002565 | -0.429944 | | 0.380088 | -0.88479 | | 14.5734 | 14.3343 | 14.64 | 15.4541 | NaN | 13.7806 | 15.0094 | 15.5392 | 3 | 2 | 2 | 3 | NaN | 1 | 2 | 2 |
| TTC17 | Q96AE7 | 2.57E-10 | 1.16E-10 | -0.429814 | + | 1.40205 | -2.6193 | | 14.528 | 14.3284 | 14.464 | 14.5161 | 14.9439 | 14.8879 | 15.2427 | 14.7593 | 5 | 5 | 5 | 3 | 4 | 2 | 5 | 5 |
| MAP2K1 | Q02750 | 9.62E-13 | 6.99E-13 | -0.428903 | + | 2.34429 | -4.40865 | | 17.2071 | 17.2519 | 17.218 | 17.8994 | 17.7143 | 17.4769 | 17.5572 | 17.6252 | 17 | 15 | 16 | 15 | 15 | 14 | 14 | 15 |
| ITGA6 | P23229-2;I | 1.87E-12 | 1.26E-12 | -0.428433 | | 0.345524 | -0.80541 | | 15.9804 | 15.9696 | 15.9558 | 17.7856 | 16.3602 | 15.3063 | 16.1445 | 16.3884 | 21 | 24 | 22 | 27 | 23 | 22 | 23 | 23 |
| ZYX | Q15942 | 3.69E-11 | 1.92E-11 | -0.428309 | + | 1.44663 | -2.69617 | | 19.4088 | 19.4135 | 19.4586 | 20.1689 | 19.9504 | 19.8017 | 19.4442 | 19.9112 | 25 | 26 | 26 | 26 | 25 | 26 | 26 | 26 |
| PCDHGC3 | Q9UN70 | 0.002674 | 0.000506 | -0.428201 | | 0.734015 | -1.49915 | | 15.9082 | 15.7723 | 15.7224 | 16.7065 | 16.4713 | 15.5853 | 15.8789 | 16.504 | 4 | 3 | 3 | 3 | 3 | 2 | 3 | 3 |
| DSP | P15924 | 5.87E-15 | 7.91E-15 | -0.428164 | | 0.81149 | -1.6294 | | 16.1371 | 16.1576 | 16.2243 | 16.7813 | 17.2287 | 16.44 | 16.0418 | 16.514 | 129 | 130 | 132 | 137 | 135 | 127 | 118 | 130 |
| FBXW11 | Q9UKB1;Q9 | 4.04E-06 | 1.10E-06 | -0.427306 | | 1.21074 | -2.2946 | | 14.8357 | 14.5848 | 14.7957 | 15.3865 | 15.4683 | 14.7058 | 15.139 | 15.1305 | 8 | 5 | 4 | 8 | 4 | 7 | 8 | 6 |
| MTG1 | Q9BT17 | 0.003121 | 0.000579 | -0.427152 | | 0.813361 | -1.75737 | | 13.824 | 14.014 | 14.3218 | NaN | 14.8603 | 14.2138 | NaN | 14.3671 | 2 | 2 | 2 | NaN | 1 | 1 | NaN | 1 |
| STOML1 | Q9UBI4;Q9 | 1.74E-05 | 4.47E-06 | -0.427044 | | 1.17563 | -2.23573 | | 15.1616 | 15.2921 | 15.3543 | 15.9431 | 15.8109 | 15.1559 | 15.853 | 15.7192 | 4 | 4 | 3 | 4 | 2 | 4 | 2 | 5 |
| SLC25A16 | P16260 | 0.002379 | 0.000457 | -0.426781 | + | 2.26624 | -4.2438 | | 14.9245 | 14.945 | 14.813 | 15.2704 | 15.0833 | 15.3226 | 15.5065 | 15.422 | 5 | 6 | 5 | 5 | 3 | 4 | 4 | 6 |
| FOXRED1 | Q96CU9;Q9 | 0.003322 | 0.000612 | -0.426483 | + | 1.71163 | -3.16565 | | 13.7509 | 13.9213 | 13.9221 | 13.9494 | 14.3933 | 14.5137 | 14.3624 | 14.2375 | 3 | 3 | 2 | 2 | 2 | 3 | 3 | 3 |
| CASP14 | P31944 | 7.85E-09 | 2.89E-09 | -0.426456 | | 0.351576 | -0.81716 | | 13.2236 | 13.4193 | 14.1955 | 15.0393 | 14.2578 | 12.8253 | 13.9531 | 14.1208 | 5 | 3 | 5 | 9 | 6 | 3 | 5 | 4 |
| ITGA5 | P08648 | 2.45E-14 | 2.77E-14 | -0.42632 | + | 1.35301 | -2.53532 | | 17.6186 | 17.6525 | 17.6626 | 18.5011 | 17.8102 | 17.8506 | 18.0121 | 18.1804 | 18 | 19 | 18 | 18 | 18 | 18 | 18 | 18 |
| DPP3 | Q9NY33 | 8.95E-14 | 8.66E-14 | -0.426219 | + | 3.31621 | -6.83244 | | 17.3534 | 17.3245 | 17.3228 | 17.6689 | 17.8677 | 17.6357 | 17.8463 | 17.7803 | 22 | 22 | 22 | 22 | 23 | 23 | 23 | 23 |
| LRP10 | Q7Z4F1 | 5.89E-07 | 1.76E-07 | -0.425747 | | 0.624536 | -1.31228 | | 17.0347 | 16.9797 | 16.9605 | 18.077 | 17.1824 | 16.6554 | 17.4229 | 17.7493 | 6 | 6 | 6 | 6 | 6 | 6 | 6 | 6 |
| CYB5R1 | Q9UHQ9 | 7.04E-09 | 2.61E-09 | -0.425668 | + | 1.9346 | -3.58128 | | 17.1444 | 17.1833 | 17.2332 | 17.4861 | 17.5371 | 17.4994 | 17.9583 | 17.5822 | 14 | 15 | 13 | 15 | 15 | 15 | 14 | 15 |
| PCNX3 | Q9H6A9 | 0.000393 | 8.59E-05 | -0.425604 | | 1.07264 | -2.06391 | | 13.495 | 13.6188 | 13.5314 | 14.2025 | 14.1725 | 13.3906 | 13.938 | 14.1664 | 4 | 3 | 2 | 3 | 3 | 3 | 1 | 2 |
| KDELR1 | P24390;P2 | 9.61E-08 | 3.11E-08 | -0.42542 | | 0.933249 | -1.83231 | | 18.875 | 18.6816 | 18.9932 | 18.8624 | 19.0509 | 19.3241 | 19.8529 | 19.2866 | 2 | 2 | 2 | 2 | 2 | 2 | 2 | 2 |
| DOCK5 | Q9H7D0 | 5.83E-16 | 1.05E-15 | -0.425258 | | 1.23052 | -2.32785 | | 15.4689 | 15.498 | 15.485 | 15.8518 | 15.9502 | 15.4996 | 16.3584 | 15.8863 | 39 | 45 | 42 | 39 | 36 | 39 | 43 | 36 |
| PIK3CD | O00329 | 2.67E-06 | 7.41E-07 | -0.425045 | | 1.28355 | -2.41731 | | 13.7318 | 13.7152 | 13.82 | 14.496 | 14.1609 | 14.3959 | 14.1055 | 13.7452 | 4 | 2 | 2 | 2 | 3 | 2 | 2 | 2 |
| RTN4 | Q9NQC3 | 9.24E-11 | 4.46E-11 | -0.424031 | | 0.730244 | -1.49277 | | 20.1664 | 20.2205 | 20.2663 | 21.0878 | 20.6155 | 19.847 | 20.8538 | 20.8046 | 15 | 13 | 16 | 17 | 10 | 13 | 15 | 15 |
| HELZ | P42694 | 3.43E-11 | 1.80E-11 | -0.423889 | + | 3.02409 | -6.02218 | | 14.4576 | 14.3693 | 14.5199 | 14.7105 | 14.9808 | 14.9399 | 14.8373 | 14.8956 | 9 | 11 | 6 | 9 | 7 | 10 | 9 | 6 |
| CSPG4 | Q6UVK1 | 1.75E-10 | 8.12E-11 | -0.423495 | | 0.758574 | -1.54058 | | 16.5661 | 16.4498 | 16.2764 | 16.9348 | 17.4635 | 16.2758 | 16.5875 | 17.0096 | 17 | 17 | 18 | 21 | 18 | 12 | 16 | 18 |
| NUF2 | Q9BZD4 | 0.000144 | 3.35E-05 | -0.423035 | | 1.20264 | -2.281 | | 13.9653 | 14.0551 | 14.0677 | 14.5054 | 14.1992 | 14.1167 | 14.5508 | 14.8899 | 6 | 6 | 6 | 5 | 4 | 2 | 6 | 5 |
| DIABLO | Q9NR28;Q9 | 6.20E-16 | 1.12E-15 | -0.422959 | | 0.477879 | -1.05332 | | 15.2124 | 14.5907 | 16.1478 | 15.8928 | 15.3174 | 15.4055 | 15.8311 | 16.253 | 8 | 8 | 7 | 7 | 7 | 7 | 8 | 8 |
| EPG5 | Q9HCE0 | 2.56E-24 | 7.58E-23 | -0.422831 | + | 2.37118 | -4.46631 | | 15.33 | 15.3945 | 15.3779 | 16.056 | 15.687 | 15.6866 | 15.8101 | 15.7117 | 52 | 50 | 46 | 56 | 41 | 51 | 46 | 51 |
| DLC1 | Q96QB1-6 | 0.000475 | 0.000103 | -0.422673 | + | 1.94871 | -3.60832 | | 14.5483 | 14.5099 | 14.5395 | 14.658 | 14.9286 | 15.2034 | 14.9896 | 14.9966 | 8 | 6 | 7 | 10 | 7 | 10 | 10 | 10 |
| PRKAB2 | O43741 | 0.000686 | 0.000145 | -0.422622 | + | 1.35157 | -2.53288 | | 15.0532 | 14.9511 | 14.8525 | 14.947 | 15.5978 | 15.6097 | 15.4084 | 15.3115 | 7 | 7 | 8 | 7 | 3 | 6 | 7 | 6 |
| CLMP | Q9H6B4 | 1.81E-09 | 7.23E-10 | -0.422605 | | 0.962207 | -1.88042 | | 15.3793 | 15.6021 | 15.6286 | 16.5636 | 15.6362 | 15.8665 | 15.7332 | 15.9969 | 8 | 9 | 10 | 9 | 9 | 8 | 8 | 9 |
| TMEM70 | Q9BUB7 | 1.15E-06 | 3.31E-07 | -0.422514 | + | 1.78941 | -3.30825 | | 15.2607 | 15.5819 | 15.5758 | 16.0597 | 15.961 | 15.6183 | 15.977 | 15.8605 | 4 | 4 | 4 | 4 | 1 | 4 | 3 | 4 |
| CXorf56 | Q9H5V9;Q9 | 0.001056 | 0.000217 | -0.422049 | + | 1.44344 | -3.10571 | | 13.1004 | 12.9921 | 13.3207 | 13.417 | 13.741 | NaN | NaN | 13.5214 | 2 | 2 | 2 | 2 | 1 | NaN | NaN | 2 |
| MDC1 | Q14676;Q1 | 2.45E-08 | 8.48E-09 | -0.42203 | | 0.928896 | -1.82508 | | 14.0461 | 13.8618 | 14.0481 | 14.6613 | 14.3283 | 13.8647 | 14.3212 | 14.8612 | 6 | 6 | 7 | 8 | 5 | 6 | 7 | 6 |
| BMPR1A | P36894 | 5.56E-14 | 5.69E-14 | -0.421868 | | 0.664689 | -1.3813 | | 13.9509 | 13.8887 | 13.7364 | 14.8279 | 14.3447 | 13.5102 | 14.1199 | 14.6 | 2 | 1 | 3 | 3 | 2 | 1 | 1 | 1 |
| PEX14 | O75381;O7 | 4.41E-14 | 4.64E-14 | -0.421859 | + | 2.61654 | -5.01417 | | 15.8154 | 15.8592 | 16.0284 | 16.4175 | 16.3056 | 16.1281 | 16.3923 | 16.3708 | 7 | 7 | 8 | 8 | 8 | 8 | 8 | 8 |
| KLC2 | Q9H0B6 | 9.46E-13 | 6.90E-13 | -0.421727 | + | 1.72203 | -3.18458 | | 16.362 | 16.4382 | 16.3742 | 16.8539 | 16.8971 | 16.5223 | 17.1047 | 16.6879 | 13 | 12 | 9 | 13 | 10 | 11 | 12 | 11 |
| AP1S2 | P56377 | 1.02E-09 | 4.21E-10 | -0.421446 | + | 1.31628 | -2.47279 | | 17.6729 | 17.6795 | 17.6204 | 18.3916 | 18.1497 | 17.6256 | 18.1977 | 18.0305 | 6 | 6 | 6 | 6 | 5 | 6 | 6 | 6 |
| GSTT1 | P30711 | 1.06E-06 | 3.07E-07 | -0.421012 | | 0.571596 | -1.2202 | | 15.593 | 15.5346 | 15.7525 | 16.1805 | 15.6698 | 15.266 | 16.6477 | 16.4747 | 9 | 6 | 8 | 9 | 5 | 4 | 9 | 10 |
| TMOD2 | Q9NZR1 | 1.01E-05 | 2.65E-06 | -0.420832 | | 0.806442 | -1.62095 | | 15.7935 | 15.8145 | 15.7737 | 16.7188 | 16.3084 | 15.9239 | 15.6353 | 16.4873 | 9 | 8 | 9 | 10 | 8 | 9 | 8 | 9 |
| PSMF1 | Q92530 | 1.61E-07 | 5.11E-08 | -0.420632 | | 1.14099 | -2.17782 | | 16.2082 | 15.9119 | 16.6792 | 16.6003 | 16.8465 | 16.6099 | 16.4876 | 16.8909 | 7 | 5 | 7 | 8 | 7 | 8 | 8 | 7 |
| RRBP1 | Q9P2E9-3 | 1.48E-20 | 1.25E-19 | -0.420239 | + | 2.66652 | -5.13087 | | 15.8465 | 16.0838 | 15.8887 | 16.2029 | 16.492 | 16.3442 | 16.3916 | 16.3689 | 2 | 3 | 2 | 3 | 3 | 3 | 3 | 2 |
| GRB2 | P62993 | 5.34E-06 | 1.44E-06 | -0.419921 | + | 2.19032 | -4.08689 | | 16.8084 | 16.7345 | 16.706 | 17.2755 | 17.322 | 16.9023 | 17.1139 | 17.2343 | 14 | 13 | 14 | 14 | 11 | 13 | 13 | 12 |
| TSEN2 | Q8NCE0;Q8 | 0.000408 | 8.90E-05 | -0.419719 | + | 2.7634 | -5.36235 | | 16.3959 | 16.4351 | 16.4089 | 16.6059 | 16.929 | 16.8801 | 16.9029 | 16.8474 | 1 | 1 | 1 | 2 | 1 | 1 | 1 | 1 |
| CYLD | Q9NQC7;Q | 0.003918 | 0.000712 | -0.419662 | | 0.842179 | -1.68069 | | 14.4538 | 14.7912 | 14.4234 | 14.8382 | 15.4664 | 14.5544 | 14.7113 | 15.3089 | 7 | 5 | 8 | 4 | 9 | 5 | 6 | 6 |
| HK2 | P52789 | 3.48E-16 | 6.72E-16 | -0.419272 | | 1.05725 | -2.03831 | | 15.6695 | 15.7341 | 15.6499 | 16.6808 | 16.1234 | 15.858 | 16.0198 | 15.8368 | 31 | 30 | 32 | 32 | 28 | 31 | 31 | 31 |
| VAT1 | Q99536 | 6.60E-16 | 1.18E-15 | -0.419204 | + | 1.50912 | -2.80485 | | 20.6408 | 20.6153 | 20.6241 | 21.1057 | 21.0422 | 21.3745 | 21.0343 | 20.6728 | 21 | 21 | 21 | 21 | 22 | 21 | 21 | 20 |
| CEP97 | Q8IW35;Q8 | 5.08E-05 | 1.25E-05 | -0.418759 | | 1.08908 | -2.09127 | | 15.0183 | 15.0479 | 15.2734 | 15.3271 | 15.8241 | 15.142 | 15.8918 | 15.4748 | 5 | 5 | 4 | 4 | 3 | 4 | 6 | 5 |
| ACLY | P53396 | 3.39E-11 | 1.78E-11 | -0.418644 | + | 3.87833 | -8.64117 | | 19.5122 | 19.5158 | 19.4921 | 19.9712 | 19.8702 | 19.8921 | 19.8493 | 20.0439 | 59 | 60 | 58 | 60 | 58 | 59 | 60 | 60 |
| GRAMD2B | Q96HH9 | 3.27E-10 | 1.44E-10 | -0.418206 | + | 1.32397 | -2.48586 | | 14.7484 | 14.8189 | 14.7657 | 15.1682 | 15.1974 | 15.4807 | 15.383 | 14.75 | 8 | 7 | 8 | 9 | 7 | 9 | 9 | 5 |
| RDH13 | Q8NBN7 | 0.005756 | 0.001017 | -0.418057 | | 0.672589 | -1.42847 | | 16.1519 | 16.0578 | 16.226 | 16.381 | 17.2847 | 16.1916 | NaN | 16.3959 | 3 | 3 | 3 | 1 | 3 | 3 | NaN | 3 |
| SYNJ2 | O15056;O1 | 6.45E-15 | 8.63E-15 | -0.417923 | + | 1.49846 | -2.78622 | | 15.3672 | 15.3065 | 15.2717 | 15.9236 | 15.3946 | 15.9431 | 15.8626 | 15.5415 | 22 | 22 | 23 | 24 | 21 | 24 | 25 | 21 |
| LZTS2 | Q9BRK4 | 0.035519 | 0.005808 | -0.417779 | | 0.44757 | -1.08638 | | 12.7978 | 12.8285 | 12.1094 | 12.6788 | 13.314 | NaN | NaN | NaN | 2 | 2 | 3 | 1 | 1 | NaN | NaN | NaN |
| MNAT1 | P51948 | 0.000153 | 3.54E-05 | -0.417069 | + | 1.89638 | -3.5085 | | 14.836 | 15.1624 | 15.1441 | 15.4253 | 15.2411 | 15.5304 | 15.6538 | 15.4722 | 4 | 3 | 3 | 5 | 3 | 4 | 3 | 4 |
| ALCAM | Q13740;Q1 | 5.80E-13 | 4.46E-13 | -0.41705 | + | 1.89185 | -3.49992 | | 17.5171 | 17.6228 | 17.5005 | 18.1891 | 17.8485 | 18.0517 | 18.0376 | 17.6923 | 18 | 19 | 19 | 20 | 16 | 20 | 20 | 21 |
| BAZ1A | Q9NRL2 | 0.002258 | 0.000437 | -0.415854 | | 0.692362 | -1.42852 | | 14.4134 | 13.2727 | 14.1469 | 14.5682 | 13.9364 | 14.447 | 14.3893 | 14.4601 | 2 | 2 | 3 | 7 | 1 | 2 | 2 | 4 |
| KIF3B | O15066 | 0.000161 | 3.72E-05 | -0.415754 | + | 2.7521 | -5.335 | | 14.664 | 14.7843 | 14.6016 | 14.8991 | 15.1325 | 15.143 | 15.1763 | 15.1446 | 12 | 14 | 11 | 9 | 9 | 11 | 11 | 11 |
| TMED8 | Q6PL24 | 0.000479 | 0.000103 | -0.414944 | + | 1.85615 | -3.43259 | | 14.541 | 14.7341 | 14.5827 | 15.1063 | 14.9049 | 14.8723 | 15.333 | 14.9546 | 2 | 2 | 3 | 3 | 2 | 3 | 2 | 3 |
| ADAL | Q6DHV7;Q | 0.00311 | 0.000577 | -0.414589 | | 1.19846 | -2.27399 | | 13.598 | 13.5724 | 13.8392 | 13.7633 | 14.1736 | 14.4276 | 14.2474 | 13.8102 | 3 | 3 | 3 | 2 | 3 | 3 | 4 | 3 |
| BMS1 | Q14692 | 1.41E-09 | 5.72E-10 | -0.414387 | + | 3.20378 | -6.5111 | | 17.0538 | 17.1431 | 17.0381 | 17.4855 | 17.5369 | 17.5452 | 17.5713 | 17.3247 | 12 | 12 | 14 | 12 | 9 | 12 | 13 | 12 |
| SLC35A2 | P78381;P7 | 0.00344 | 0.000632 | -0.413975 | + | 1.37294 | -2.56939 | | 14.6412 | 14.7474 | 14.5295 | 15.1819 | 15.1368 | 14.5912 | 15.1782 | 15.1785 | 2 | 1 | 1 | 1 | 1 | 1 | 1 | 1 |
| CDC34 | P49427 | 0.000974 | 0.000201 | -0.413934 | + | 2.47515 | -4.69355 | | 15.6254 | 15.7713 | 15.7733 | 16.2287 | 16.2531 | 15.9587 | 16.0254 | 16.2206 | 3 | 4 | 4 | 4 | 4 | 4 | 4 | 4 |
| STARD13 | Q9Y3M8;Q9 | 2.72E-16 | 5.44E-16 | -0.413822 | | 0.469382 | -1.03789 | | 14.8253 | 14.7021 | 14.9025 | 14.1318 | 15.7461 | 15.0798 | 15.6978 | 15.4634 | 8 | 8 | 9 | 4 | 4 | 5 | 5 | 6 |
| CDK17 | Q00537;Q( | 3.10E-07 | 9.56E-08 | -0.413762 | | 0.808133 | -1.62378 | | 15.0065 | 14.8494 | 14.8884 | 15.868 | 15.4806 | 15.3594 | 14.7018 | 15.2329 | 4 | 3 | 4 | 9 | 5 | 5 | 5 | 7 |
| HEBP1 | Q9NRV9 | 2.54E-08 | 8.80E-09 | -0.413267 | + | 1.6668 | -3.08453 | | 17.931 | 17.9339 | 17.9335 | 18.6692 | 18.2895 | 18.3861 | 18.344 | 18.0416 | 10 | 10 | 9 | 10 | 10 | 9 | 10 | 10 |
| SIN3B | O75182;O7 | 1.45E-08 | 5.18E-09 | -0.413165 | | 1.04521 | -2.09684 | | 14.5306 | 14.5996 | 13.9988 | 14.5984 | NaN | 14.65 | 15.0094 | 14.9003 | 3 | 3 | 2 | 1 | NaN | 1 | 2 | 2 |
| BCAR1 | P56945 | 2.56E-24 | 7.58E-23 | -0.413162 | + | 1.51497 | -2.81508 | | 15.8896 | 15.951 | 15.9566 | 16.549 | 16.4636 | 15.9298 | 16.3338 | 16.4516 | 18 | 18 | 18 | 18 | 16 | 20 | 17 | 17 |
| FXR2 | P51116 | 4.50E-12 | 2.78E-12 | -0.413127 | + | 3.29587 | -6.7734 | | 15.7038 | 15.7287 | 15.7026 | 16.1507 | 16.1394 | 15.9512 | 16.1638 | 16.219 | 15 | 14 | 16 | 15 | 14 | 17 | 16 | 15 |
| DNLZ | Q5SXM8 | 0.003482 | 0.000639 | -0.413105 | | 0.545408 | -1.17412 | | 14.3878 | 13.5165 | 13.7568 | 13.6033 | 14.9224 | 14.5982 | 14.1649 | 14.212 | 1 | 1 | 2 | 2 | 1 | 1 | 2 | 2 |

| Gene names | UniProt IDs | Protein Group P-value | Protein Group Q-value | Log2 Difference NUP214 vs Controls (n=5) | p<0.05 | -log10 p-value | T-test Test statistic | NPC Localization | Log2 MS2_quantity_ NUP214_1 | Log2 MS2_quantity_ NUP214_2 | Log2 MS2_quantity_ NUP214_3 | Log2 MS2_quantity_ Control_1 | Log2 MS2_quantity_ Control_2 | Log2 MS2_quantity_ Control_3 | Log2 MS2_quantity_ Control_4 | Log2 MS2_quantity_ Control_5 | Number Peptides for Quantitation NUP214_1 | Number Peptides for Quantitation NUP214_2 | Number Peptides for Quantitation NUP214_3 | Number Peptides for Quantitation Control_1 | Number Peptides for Quantitation Control_2 | Number Peptides for Quantitation Control_3 | Number Peptides for Quantitation Control_4 | Number Peptides for Quantitation Control_5 |
|---|---|---|---|---|---|---|---|---|---|---|---|---|---|---|---|---|---|---|---|---|---|---|---|---|
| PRIM1 | P49642 | 1.51E-05 | 3.90E-06 | -0.411767 | | 0.371649 | -0.85579 | | 13.569 | 13.6143 | 13.6403 | 14.3949 | 14.4485 | 12.5861 | 14.2243 | 14.4443 | 1 | 2 | 2 | 3 | 2 | 1 | 2 | 3 |
| SPPL2A | Q8TCT8 | 0.000296 | 6.58E-05 | -0.411592 | | 1.0917 | -2.09562 | | 18.0767 | 17.9864 | 18.1057 | 18.3043 | 18.184 | 18.3265 | 19.0128 | 18.5116 | 2 | 2 | 2 | 2 | 2 | 2 | 2 | 2 |
| UBAC1 | Q9BSL1 | 2.37E-13 | 2.03E-13 | -0.41132 | | 0.593438 | -1.25835 | | 15.0622 | 14.9906 | 14.856 | 15.0603 | 16.3094 | 15.1265 | 14.9945 | 15.4138 | 3 | 4 | 3 | 3 | 3 | 4 | 3 | 2 |
| AMFR | Q9UKV5 | 8.38E-08 | 2.72E-08 | -0.410954 | + | 1.44715 | -2.69708 | | 15.7596 | 15.6965 | 15.6875 | 16.1894 | 16.3396 | 15.6884 | 16.1574 | 16.2526 | 4 | 6 | 4 | 6 | 4 | 5 | 6 | 7 |
| ANO10 | Q9NW15 | 8.49E-15 | 1.09E-14 | -0.410868 | + | 1.57965 | -2.92893 | | 16.4682 | 16.4917 | 16.5075 | 16.8341 | 16.7783 | 16.6651 | 17.2801 | 16.9423 | 16 | 17 | 18 | 16 | 16 | 17 | 18 | 17 |
| SERINC1 | Q9NRX5 | 8.05E-16 | 1.41E-15 | -0.41024 | + | 1.35521 | -2.53908 | | 17.1411 | 17.1822 | 17.2448 | 17.9724 | 17.5324 | 17.2225 | 17.6388 | 17.6319 | 4 | 4 | 4 | 4 | 3 | 4 | 4 | 4 |
| SQSTM1 | Q13501 | 4.47E-20 | 3.17E-19 | -0.410232 | | 0.864063 | -1.7172 | | 17.7443 | 17.7099 | 17.669 | 18.7294 | 18.1292 | 17.7508 | 17.7691 | 18.2112 | 13 | 13 | 13 | 13 | 13 | 13 | 13 | 13 |
| STEAP3 | Q658P3;Q6 | 1.92E-07 | 6.04E-08 | -0.410098 | | 0.597312 | -1.2651 | | 16.1776 | 16.1841 | 16.1775 | 17.2689 | 16.8699 | 16.104 | 15.9694 | 16.737 | 11 | 11 | 11 | 11 | 10 | 11 | 11 | 11 |
| APH1A | Q96BI3;Q9 | 2.61E-08 | 9.02E-09 | -0.410096 | + | 1.55765 | -2.89005 | | 15.0593 | 15.2396 | 15.1733 | 15.5577 | 15.4336 | 15.4305 | 15.9664 | 15.4493 | 2 | 2 | 2 | 2 | 2 | 2 | 2 | 2 |
| TFG | Q92734;Q9 | 1.10E-08 | 3.98E-09 | -0.409884 | + | 4.00995 | -9.1193 | | 18.1138 | 17.9579 | 18.0693 | 18.4186 | 18.4612 | 18.5112 | 18.4977 | 18.3958 | 10 | 7 | 10 | 8 | 10 | 9 | 10 | 8 |
| BAG6 | P46379;P4 | 8.46E-11 | 4.11E-11 | -0.409785 | + | 1.8152 | -3.35607 | | 16.5344 | 16.5365 | 16.7666 | 17.1129 | 17.2547 | 16.792 | 16.8967 | 17.0552 | 24 | 23 | 23 | 26 | 23 | 23 | 25 | 24 |
| EVI5 | O60447;O6 | 0.000418 | 9.11E-05 | -0.408738 | | 0.55354 | -1.18847 | | 13.2037 | 13.6008 | 13.6201 | 14.2726 | 14.0756 | 13.8379 | 14.2818 | 12.9501 | 2 | 2 | 2 | 3 | 1 | 4 | 2 | 1 |
| SSH3 | Q8TE77;Q8 | 0.00084 | 0.000175 | -0.408717 | + | 1.59617 | -2.95821 | | 15.237 | 15.457 | 15.4518 | 15.4721 | 16.0249 | 15.7972 | 15.9388 | 15.7204 | 5 | 6 | 5 | 7 | 5 | 6 | 6 | 7 |
| RHBDD1 | Q8TEB9 | 1.74E-05 | 4.46E-06 | -0.408556 | + | 1.32231 | -2.48303 | | 16.0758 | 15.9582 | 15.6762 | 15.9911 | 16.5902 | 16.1646 | 16.4142 | 16.3996 | 4 | 4 | 4 | 4 | 4 | 4 | 4 | 4 |
| NACC1 | Q96RE7 | 3.44E-10 | 1.51E-10 | -0.408008 | | 1.15496 | -2.20115 | | 15.6105 | 15.7142 | 15.6069 | 16.2321 | 16.2497 | 15.5507 | 15.9574 | 16.2695 | 6 | 7 | 5 | 5 | 4 | 4 | 5 | 5 |
| ABCA1 | O95477 | 0.001245 | 0.000253 | -0.407968 | + | 2.20456 | -4.11608 | | 13.684 | 13.4235 | 13.6584 | 14.0192 | 14.1377 | 14.0895 | 13.8078 | 13.9288 | 2 | 3 | 4 | 5 | 6 | 5 | 5 | 3 |
| SLC35A4 | L0R6Q1 | 0.002871 | 0.000538 | -0.407855 | | 0.293041 | -0.70148 | | 14.4629 | 14.5836 | 14.8911 | 15.2904 | 14.6011 | 13.6368 | 15.642 | 16.0983 | 2 | 1 | 1 | 3 | 3 | 2 | 3 | 3 |
| EPS8 | Q12929 | 5.99E-14 | 6.07E-14 | -0.407832 | + | 3.01028 | -5.98578 | | 16.4919 | 16.5147 | 16.5528 | 17.1072 | 16.8271 | 16.8433 | 16.9098 | 16.9509 | 26 | 26 | 26 | 28 | 28 | 28 | 29 | 29 |
| HEBP2 | Q9Y5Z4 | 2.18E-06 | 6.12E-07 | -0.40762 | + | 1.63508 | -3.0276 | | 17.9674 | 17.9053 | 18.0432 | 18.3233 | 18.1968 | 18.4904 | 18.7057 | 18.1817 | 8 | 8 | 7 | 7 | 7 | 8 | 8 | 8 |
| PPP1R12C | Q9BZL4 | 2.98E-13 | 2.49E-13 | -0.407608 | + | 5.27256 | -15.0904 | | 15.5854 | 15.5491 | 15.5871 | 16.0214 | 16.0194 | 15.9502 | 15.9251 | 15.9911 | 23 | 23 | 23 | 24 | 22 | 25 | 24 | 23 |
| RABAC1 | Q9UI14 | 4.60E-20 | 3.21E-19 | -0.407194 | | 1.28755 | -2.42407 | | 16.3835 | 16.1111 | 16.2057 | 16.3838 | 16.8126 | 16.4584 | 17.0138 | 16.5346 | 3 | 4 | 4 | 4 | 4 | 4 | 4 | 3 |
| BAG3 | O95817 | 1.04E-12 | 7.50E-13 | -0.407153 | + | 1.3879 | -2.59502 | | 16.4007 | 16.446 | 16.5743 | 17.2666 | 16.5981 | 16.8983 | 16.7102 | 16.9309 | 24 | 25 | 24 | 25 | 20 | 24 | 25 | 25 |
| ZFYVE19 | Q96K21;Q9 | 1.42E-06 | 4.07E-07 | -0.407097 | + | 3.3182 | -6.83824 | | 14.6461 | 14.7588 | 14.5802 | 15.0865 | 15.1049 | 15.0076 | 15.1678 | 14.9772 | 5 | 5 | 4 | 4 | 2 | 5 | 5 | 4 |
| CPPED1 | Q9BRF8 | 3.21E-08 | 1.10E-08 | -0.406995 | | 1.23042 | -2.32768 | | 17.9034 | 17.9149 | 17.8779 | 17.9192 | 18.6567 | 18.1102 | 18.368 | 18.4747 | 9 | 9 | 8 | 9 | 9 | 10 | 10 | 9 |
| DST | Q03001 | 6.67E-19 | 3.04E-18 | -0.406984 | + | 1.36549 | -2.55665 | | 15.4658 | 15.4619 | 15.4624 | 15.7975 | 16.1325 | 15.4546 | 16.0628 | 15.9045 | 219 | 215 | 219 | 222 | 209 | 199 | 233 | 219 |
| ERLEC1 | Q96DZ1;Q9 | 5.61E-09 | 2.11E-09 | -0.406711 | + | 1.59315 | -2.95285 | | 15.6681 | 15.6256 | 15.6256 | 15.865 | 16.0749 | 16.0385 | 16.4146 | 15.8393 | 16 | 14 | 13 | 14 | 11 | 15 | 17 | 13 |
| KIAA1191 | Q96A73;Q9 | 8.96E-07 | 2.62E-07 | -0.406602 | | 0.528048 | -1.14335 | | 13.8282 | 13.8058 | 13.5864 | 14.1049 | 14.3944 | 14.1812 | 13.2222 | 14.8309 | 4 | 4 | 3 | 4 | 3 | 3 | 2 | 3 |
| G6PC3 | Q9BUM1 | 7.44E-12 | 4.45E-12 | -0.406105 | | 0.68153 | -1.41006 | | 16.1121 | 16.5597 | 16.5608 | 17.1261 | 16.5244 | 17.2206 | 17.0352 | 16.1785 | 2 | 2 | 2 | 2 | 2 | 2 | 2 | 1 |
| BICD2 | Q8TD16;Q8 | 6.86E-11 | 3.40E-11 | -0.405841 | + | 1.61586 | -2.99325 | | 15.3203 | 15.2913 | 15.3271 | 15.9987 | 15.6917 | 15.38 | 15.6965 | 15.8269 | 21 | 19 | 19 | 23 | 20 | 19 | 22 | 23 |
| PSME4 | Q14997 | 2.61E-13 | 2.22E-13 | -0.405695 | + | 2.74862 | -5.3266 | | 14.5066 | 14.605 | 14.5768 | 15.0829 | 15.0967 | 14.8055 | 14.9497 | 14.9077 | 20 | 21 | 19 | 24 | 17 | 18 | 19 | 24 |
| UBE2J1 | Q9Y385 | 0.000204 | 4.63E-05 | -0.404498 | + | 1.49475 | -2.77975 | | 14.8127 | 14.7512 | 14.6575 | 15.1255 | 14.7674 | 15.1347 | 15.3444 | 15.3528 | 4 | 5 | 4 | 5 | 3 | 5 | 5 | 5 |
| PDLIM4 | P50479 | 7.00E-12 | 4.20E-12 | -0.403708 | + | 2.26503 | -4.24128 | | 18.5754 | 18.5666 | 18.5993 | 18.8972 | 18.8472 | 19.2318 | 19.0542 | 18.8902 | 15 | 14 | 14 | 15 | 15 | 14 | 15 | 16 |
| HYPK | Q9NX55 | 2.46E-05 | 6.20E-06 | -0.403702 | | 0.345459 | -0.80529 | | 15.7573 | 15.2896 | 16.5592 | 17.029 | 15.2968 | 15.9061 | 16.2706 | 16.8593 | 5 | 4 | 6 | 4 | 4 | 5 | 5 | 5 |
| TGFB1 | P01137 | 9.37E-05 | 2.23E-05 | -0.403648 | | 0.84767 | -1.68986 | | 15.1172 | 15.1902 | 15.3824 | 15.6928 | 15.2961 | 15.4064 | 16.2785 | 15.494 | 6 | 8 | 6 | 6 | 5 | 4 | 6 | 5 |
| UNC119 | Q13432 | 2.58E-11 | 1.39E-11 | -0.403331 | + | 1.74004 | -3.21746 | | 15.8719 | 15.7956 | 15.769 | 16.3498 | 16.391 | 15.9274 | 16.0649 | 16.3444 | 4 | 6 | 6 | 5 | 4 | 4 | 5 | 5 |
| ARHGAP18 | Q8N392 | 3.68E-18 | 1.35E-17 | -0.402681 | | 0.790064 | -1.5935 | | 15.902 | 15.9059 | 15.9237 | 17.0521 | 16.2591 | 15.9938 | 16.1145 | 16.1466 | 24 | 24 | 24 | 26 | 23 | 24 | 27 | 25 |
| SNX16 | P57768 | 9.57E-18 | 3.10E-17 | -0.402507 | | 1.01428 | -1.9669 | | 14.5936 | 14.6379 | 14.7067 | 14.8892 | 15.0627 | 14.6612 | 15.5861 | 15.0436 | 3 | 4 | 3 | 3 | 4 | 3 | 4 | 5 |
| POLR1D | P0DPB6 | 0.000164 | 3.77E-05 | -0.402224 | | 0.904162 | -1.78396 | | 15.7632 | 15.4648 | 15.784 | 15.6505 | 16.5678 | 15.9338 | 16.2839 | 15.9284 | 3 | 2 | 3 | 4 | 2 | 4 | 2 | 3 |
| SYT11 | Q9BT88 | 2.60E-05 | 6.55E-06 | -0.402137 | | 0.793817 | -1.59979 | | 15.4323 | 15.4222 | 15.4049 | 16.3891 | 15.669 | 15.2311 | 15.93 | 15.8904 | 4 | 5 | 4 | 5 | 4 | 3 | 4 | 4 |
| LAMC1 | P11047 | 1.18E-14 | 1.47E-14 | -0.400514 | | 1.1226 | -2.14712 | | 16.4523 | 16.47 | 16.432 | 16.3942 | 16.9286 | 17.0115 | 17.213 | 16.7125 | 46 | 48 | 44 | 42 | 43 | 48 | 48 | 47 |
| PSMA5 | P28066 | 6.12E-09 | 2.29E-09 | -0.400169 | + | 1.31962 | -2.47846 | | 18.5317 | 18.3697 | 18.5829 | 18.49 | 18.8192 | 18.9997 | 19.1756 | 18.9902 | 14 | 13 | 13 | 13 | 14 | 13 | 13 | 14 |
| MARF1 | Q9Y4F3;Q9 | 0.000176 | 4.03E-05 | -0.399888 | | 1.00706 | -2.02779 | | 13.7664 | 14.1959 | 14.2771 | 14.6494 | 14.6703 | 14.1373 | NaN | 14.4618 | 1 | 1 | 1 | 1 | 1 | 1 | NaN | 1 |
| TGFBR2 | P37173;P3 | 2.72E-15 | 4.11E-15 | -0.399813 | | 0.669798 | -1.39003 | | 15.4445 | 15.197 | 15.3655 | 16.3301 | 15.2885 | 15.5896 | 15.3321 | 16.1372 | 8 | 7 | 8 | 9 | 4 | 7 | 6 | 8 |
| NPC1 | O15118 | 1.37E-08 | 4.91E-09 | -0.399511 | + | 3.92212 | -8.79775 | | 16.7776 | 16.7383 | 16.7114 | 17.2142 | 17.0727 | 17.2239 | 17.1173 | 17.0816 | 14 | 13 | 14 | 14 | 13 | 14 | 14 | 14 |
| IFIT1 | P09914;P0 | 5.80E-07 | 1.73E-07 | -0.398046 | + | 1.40936 | -2.63187 | | 14.9413 | 14.9996 | 15.0476 | 15.0643 | 15.6752 | 15.2084 | 15.4817 | 15.5414 | 11 | 11 | 10 | 11 | 10 | 11 | 14 | 12 |
| PPAN | Q9NQ55;Q9 | 1.73E-08 | 6.10E-09 | -0.397804 | + | 2.79745 | -5.44542 | | 14.5314 | 14.7063 | 14.5745 | 15.1587 | 14.9319 | 14.8976 | 14.9725 | 15.0488 | 5 | 6 | 5 | 5 | 4 | 5 | 6 | 6 |
| MMTAG2 | Q9BU76;Q9 | 0.001479 | 0.000298 | -0.397632 | | 0.7281 | -1.48915 | | 13.6593 | 13.7671 | 13.5746 | 14.583 | 13.5591 | 13.8206 | 13.886 | 14.4743 | 1 | 2 | 2 | 3 | 1 | 1 | 2 | 2 |
| GALNT4 | Q8N4A0 | 0.000422 | 9.18E-05 | -0.397559 | | 1.12383 | -2.14918 | | 14.5853 | 14.6491 | 14.7287 | 15.3425 | 15.4019 | 14.7016 | 14.9445 | 14.869 | 4 | 5 | 5 | 7 | 4 | 4 | 6 | 4 |
| SLC16A7 | O60669 | 2.87E-06 | 7.94E-07 | -0.397539 | | 1.23511 | -2.33557 | | 15.8141 | 15.6725 | 15.6078 | 15.8171 | 16.5049 | 15.8701 | 16.1833 | 16.1028 | 3 | 4 | 4 | 3 | 2 | 4 | 3 | 3 |
| N4BP1 | O75113 | 8.53E-06 | 2.26E-06 | -0.39726 | | 1.2157 | -2.30294 | | 14.2277 | 14.2069 | 14.4306 | 14.7938 | 14.7986 | 14.1961 | 14.8648 | 14.7751 | 8 | 7 | 6 | 7 | 5 | 6 | 6 | 7 |
| NFATC2IP | Q8NCF5;Q8 | 0.00112 | 0.00023 | -0.396397 | | 0.888228 | -1.81432 | | 14.1614 | 14.7494 | 14.4803 | 14.4649 | 14.9359 | NaN | 14.9128 | 15.1267 | 1 | 1 | 1 | 1 | 1 | NaN | 1 | 1 |
| DNAJB12 | Q9NXW2 | 1.28E-09 | 5.23E-10 | -0.396312 | + | 1.52715 | -2.83642 | | 14.4787 | 14.4808 | 14.4504 | 15.073 | 14.838 | 14.4852 | 15.0374 | 14.8978 | 9 | 10 | 9 | 9 | 10 | 10 | 10 | 10 |
| RPS19BP1 | Q86WX3 | 0.000516 | 0.000111 | -0.396236 | + | 1.80344 | -3.33423 | | 14.9391 | 14.996 | 14.931 | 15.3656 | 15.5645 | 15.1727 | 15.1305 | 15.5247 | 2 | 3 | 3 | 3 | 2 | 2 | 2 | 4 |
| TPM3 | P06753-2 | 6.61E-13 | 4.99E-13 | -0.396129 | + | 2.64063 | -5.07019 | | 19.6482 | 19.5647 | 19.5612 | 20.091 | 20.1348 | 19.883 | 19.8443 | 19.9845 | 24 | 24 | 24 | 24 | 24 | 24 | 24 | 24 |
| UFD1 | Q92890;Q9 | 2.21E-13 | 1.91E-13 | -0.395971 | + | 2.53039 | -4.81718 | | 17.122 | 17.2267 | 17.1063 | 17.6692 | 17.7004 | 17.4031 | 17.4836 | 17.4819 | 11 | 11 | 12 | 11 | 11 | 11 | 11 | 11 |
| RASAL2 | Q9UJF2-2 | 1.29E-11 | 7.34E-12 | -0.395915 | + | 1.83865 | -3.3998 | | 14.7869 | 14.7365 | 14.9265 | 15.3946 | 15.2008 | 14.9243 | 15.2013 | 15.3418 | 15 | 14 | 16 | 18 | 12 | 13 | 14 | 12 |
| HSPA4L | O95757 | 6.07E-11 | 3.04E-11 | -0.395426 | + | 1.84854 | -3.41831 | | 15.7842 | 15.7387 | 15.7871 | 15.8689 | 16.1094 | 16.3403 | 16.3306 | 16.1779 | 13 | 13 | 10 | 10 | 15 | 15 | 14 | 16 |
| TKFC | Q3LXA3 | 1.65E-12 | 1.13E-12 | -0.395335 | + | 1.55511 | -2.88557 | | 16.4388 | 16.4098 | 16.4048 | 16.5333 | 16.7237 | 17.111 | 16.9771 | 16.7205 | 17 | 15 | 16 | 17 | 13 | 16 | 17 | 15 |
| C6orf89 | Q6UWU4;Q | 0.001155 | 0.000236 | -0.395155 | + | 1.6022 | -2.96894 | | 14.717 | 14.4645 | 14.6572 | 15.1325 | 15.298 | 14.8123 | 14.9356 | 14.862 | 4 | 3 | 4 | 5 | 2 | 4 | 3 | 4 |
| CD63 | P08962;P0 | 1.69E-10 | 7.82E-11 | -0.39486 | + | 1.57494 | -2.92059 | | 20.6001 | 20.5152 | 20.4498 | 21.0694 | 20.5465 | 20.9851 | 21.0863 | 20.8955 | 5 | 5 | 5 | 5 | 5 | 5 | 5 | 5 |
| CES2 | O00748;O0 | 1.13E-11 | 6.50E-12 | -0.394423 | | 0.844986 | -1.68538 | | 14.8165 | 14.9683 | 14.8224 | 14.8083 | 15.7204 | 15.6021 | 15.0133 | 15.1733 | 3 | 5 | 6 | 6 | 6 | 7 | 8 | 8 |
| DDX49 | Q9Y6V7 | 0.002073 | 0.000405 | -0.394318 | + | 1.49748 | -2.78452 | | 14.6376 | 14.4035 | 14.4124 | 14.8321 | 14.989 | 14.8976 | 14.5441 | 15.1312 | 5 | 5 | 4 | 3 | 4 | 3 | 2 | 6 |
| TAPT1 | Q6NXT6 | 5.25E-13 | 4.09E-13 | -0.394291 | + | 1.90365 | -3.52228 | | 14.9449 | 14.8793 | 14.8844 | 15.3138 | 15.0831 | 15.2126 | 15.5886 | 15.2877 | 5 | 5 | 5 | 5 | 4 | 4 | 4 | 5 |
| POFUT2 | Q9Y2G5 | 2.45E-16 | 5.05E-16 | -0.393525 | + | 3.25173 | -6.64666 | | 16.8987 | 16.9443 | 17.0183 | 17.4513 | 17.2423 | 17.4187 | 17.3488 | 17.2753 | 14 | 16 | 15 | 14 | 14 | 15 | 16 | 17 |
| LMO7 | Q8WWI1-3 | 7.50E-13 | 5.59E-13 | -0.392346 | + | 1.88211 | -3.48148 | | 17.877 | 17.8662 | 17.8535 | 18.3444 | 18.1876 | 18.0104 | 18.5188 | 18.2284 | 75 | 75 | 76 | 74 | 70 | 74 | 74 | 75 |
| NDEL1 | Q9GZM8;Q | 0.00035 | 7.71E-05 | -0.392263 | + | 1.85106 | -3.42305 | | 14.5975 | 14.3227 | 14.4353 | 14.9214 | 15.013 | 14.5723 | 14.8356 | 14.8781 | 1 | 3 | 2 | 3 | 2 | 2 | 3 | 2 |
| ERCC5 | P28715 | 0.007582 | 0.001323 | -0.392091 | | 0.837803 | -1.67339 | | 14.2537 | 13.3699 | 13.9792 | 14.3951 | 14.5605 | 14.0639 | 14.2666 | 14.0125 | 3 | 5 | 5 | 3 | 3 | 5 | 4 | 3 |
| PLEC | Q15149-6 | 3.15E-07 | 9.70E-08 | -0.391816 | | 0.991598 | -1.92923 | | 14.5416 | 15.0825 | 14.7384 | 15.4351 | 15.1058 | 15.499 | 15.0151 | 14.8416 | 2 | 2 | 2 | 2 | 2 | 2 | 2 | 2 |
| GBE1 | Q04446 | 1.41E-09 | 5.71E-10 | -0.391556 | + | 1.50719 | -2.80147 | | 18.3339 | 18.3524 | 18.317 | 18.9272 | 18.6695 | 18.974 | 18.3939 | 18.6653 | 31 | 31 | 30 | 31 | 30 | 31 | 31 | 31 |
| ATXN2 | Q99700;Q9 | 5.57E-14 | 5.70E-14 | -0.391336 | + | 2.45693 | -4.65321 | | 15.6303 | 15.6832 | 15.6427 | 16.2614 | 16.0215 | 15.8791 | 15.992 | 16.063 | 18 | 21 | 21 | 22 | 17 | 22 | 20 | 18 |
| DPF3 | Q92784 | 0.003282 | 0.000606 | -0.391245 | | 0.587157 | -1.24741 | | 13.2777 | 12.9745 | 13.1409 | 13.7728 | 14.0813 | 12.94 | 13.8089 | 13.0083 | 3 | 4 | 3 | 3 | 3 | 2 | 2 | 2 |
| C3orf38 | Q5JPI3;Q5J | 0.00026 | 5.82E-05 | -0.390853 | | 0.919701 | -1.8098 | | 13.9978 | 13.7567 | 13.902 | 14.2686 | 13.7967 | 14.238 | 14.7897 | 14.2887 | 2 | 4 | 3 | 4 | 3 | 3 | 3 | 2 |
| SPC24 | Q8NBT2 | 2.49E-11 | 1.34E-11 | -0.390674 | | 0.270063 | -0.65467 | | 14.6705 | 13.7954 | 15.6441 | 15.6673 | 14.754 | 13.9593 | 15.264 | 15.8255 | 3 | 2 | 3 | 3 | 3 | 2 | 3 | 3 |
| NRP1 | O14786 | 1.53E-12 | 1.06E-12 | -0.390617 | | 1.18649 | -2.25393 | | 15.6481 | 15.713 | 15.7726 | 16.2863 | 16.1106 | 15.7057 | 16.4468 | 15.9599 | 18 | 19 | 16 | 17 | 14 | 16 | 19 | 15 |
| CALCOCO1 | Q9P1Z2;Q9 | 0.001722 | 0.000343 | -0.390362 | + | 2.24822 | -4.20625 | | 14.8213 | 14.8726 | 14.8979 | 15.2262 | 15.1973 | 15.0421 | 15.4196 | 15.3864 | 7 | 7 | 7 | 8 | 4 | 6 | 7 | 7 |
| WDR37 | Q9Y2I8;Q9 | 2.79E-14 | 3.11E-14 | -0.389332 | | 0.86983 | -1.72681 | | 15.6539 | 15.5171 | 15.5821 | 15.5776 | 16.4776 | 15.99 | 16.1796 | 15.6436 | 5 | 5 | 6 | 6 | 3 | 5 | 7 | 4 |
| CLPTM1L | Q96KA5;Q9 | 6.11E-10 | 2.60E-10 | -0.389109 | + | 1.88089 | -3.47919 | | 16.5069 | 16.6255 | 16.4502 | 16.9181 | 16.8381 | 16.7013 | 17.1841 | 16.9417 | 11 | 11 | 10 | 11 | 9 | 10 | 11 | 11 |
| HTRA2 | O43464 | 5.13E-08 | 1.71E-08 | -0.388857 | + | 3.88174 | -8.65327 | | 15.7206 | 15.7515 | 15.6486 | 16.1117 | 16.0475 | 16.1523 | 16.1581 | 16.0093 | 7 | 7 | 7 | 7 | 6 | 7 | 7 | 7 |

| Gene names | UniProt IDs | Protein Group P-value | Protein Group Q-value | Log2 Difference NUP214 vs Controls (n=5) | p<0.05 | -log10 p-value | T-test Test statistic | NPC Localization | Log2 MS2_quantity_ NUP214_1 | Log2 MS2_quantity_ NUP214_2 | Log2 MS2_quantity_ NUP214_3 | Log2 MS2_quantity_ Control_1 | Log2 MS2_quantity_ Control_2 | Log2 MS2_quantity_ Control_3 | Log2 MS2_quantity_ Control_4 | Log2 MS2_quantity_ Control_5 | Number Peptides for Quantitation NUP214_1 | Number Peptides for Quantitation NUP214_2 | Number Peptides for Quantitation NUP214_3 | Number Peptides for Quantitation Control_1 | Number Peptides for Quantitation Control_2 | Number Peptides for Quantitation Control_3 | Number Peptides for Quantitation Control_4 | Number Peptides for Quantitation Control_5 |
|---|---|---|---|---|---|---|---|---|---|---|---|---|---|---|---|---|---|---|---|---|---|---|---|---|
| EFCAB14 | O75071 | 7.56E-05 | 1.82E-05 | -0.388818 | | 0.818098 | -1.64046 | | 14.5082 | 14.858 | 14.5015 | 14.7463 | 15.6166 | 14.805 | 14.7702 | 15.1188 | 3 | 2 | 2 | 4 | 3 | 2 | 2 | 3 |
| TP53BP2 | Q13625;Q1 | 6.65E-06 | 1.78E-06 | -0.388699 | + | 1.73548 | -3.20912 | | 15.3344 | 15.2356 | 15.3746 | 15.8888 | 15.8664 | 15.3999 | 15.7011 | 15.6616 | 12 | 12 | 10 | 14 | 12 | 8 | 11 | 12 |
| NOM1 | Q5C9Z4 | 2.30E-06 | 6.44E-07 | -0.388516 | + | 1.35611 | -2.54061 | | 13.8408 | 13.7555 | 13.6904 | 14.5315 | 14.1778 | 13.9227 | 13.9233 | 14.1984 | 4 | 6 | 4 | 5 | 3 | 3 | 5 | 4 |
| ZNF318 | Q5VUA4 | 0.003404 | 0.000626 | -0.388354 | + | 1.35187 | -2.53338 | | 14.0867 | 14.3302 | 14.3231 | 14.5672 | 14.4051 | 14.5766 | 14.5878 | 15.0385 | 6 | 4 | 5 | 3 | 3 | 3 | 3 | 2 |
| MAFF | Q9ULX9 | 0.019723 | 0.003286 | -0.388158 | | 0.595749 | -1.28947 | | 14.0229 | 14.474 | 14.2958 | 14.7303 | 14.9443 | NaN | 13.9606 | 14.9743 | 2 | 1 | 2 | 3 | 1 | NaN | 2 | 2 |
| PDLIM5 | Q96HC4 | 9.83E-11 | 4.71E-11 | -0.387469 | + | 1.30518 | -2.45395 | | 17.4031 | 17.5033 | 17.4505 | 18.2198 | 17.7313 | 17.9949 | 17.6719 | 17.5809 | 25 | 24 | 24 | 26 | 24 | 24 | 23 | 25 |
| AGPAT5 | Q9NUQ2 | 0.002635 | 0.0005 | -0.387 | | 0.784224 | -1.5837 | | 13.1859 | 13.5012 | 12.9922 | 13.4448 | 13.4063 | 13.8623 | 13.2305 | 14.1232 | 4 | 3 | 2 | 2 | 1 | 1 | 3 | 3 |
| ACOX1 | Q15067;Q1 | 2.56E-24 | 7.58E-23 | -0.386811 | + | 1.32485 | -2.48736 | | 16.0397 | 16.0548 | 16.0696 | 16.7795 | 16.3124 | 16.1394 | 16.3379 | 16.6383 | 19 | 17 | 20 | 18 | 20 | 15 | 18 | 19 |
| STS | P08842 | 0.000187 | 4.28E-05 | -0.386603 | | 0.637468 | -1.33457 | | 15.1134 | 15.1741 | 15.1566 | 15.3954 | 15.6631 | 15.4681 | 14.9029 | 16.2436 | 8 | 9 | 8 | 9 | 8 | 8 | 5 | 10 |
| CHMP7 | Q8WUX9 | 1.01E-11 | 5.88E-12 | -0.386487 | + | 1.46887 | -2.73472 | | 14.8354 | 14.8845 | 14.7722 | 15.32 | 15.4848 | 14.8526 | 15.2545 | 15.1739 | 6 | 6 | 6 | 7 | 5 | 5 | 6 | 5 |
| CUEDC1 | Q9NWM3 | 0.007711 | 0.001344 | -0.386376 | | 1.14909 | -2.19134 | | 14.8885 | 15.076 | 15.2479 | 15.793 | 15.2314 | 15.5233 | 15.1439 | 15.5943 | 2 | 3 | 3 | 2 | 3 | 2 | 2 | 2 |
| AHR | P35869 | 1.12E-06 | 3.24E-07 | -0.385891 | | 1.08126 | -2.07825 | | 13.9194 | 14.0043 | 13.973 | 14.2025 | 14.4533 | 13.8856 | 14.6471 | 14.5688 | 4 | 5 | 5 | 5 | 4 | 3 | 6 | 3 |
| GAPDH | P04406 | 1.10E-11 | 6.34E-12 | -0.385826 | | 0.876556 | -1.73801 | | 23.486 | 23.4326 | 23.3842 | 23.6781 | 24.428 | 23.6516 | 23.463 | 23.8798 | 24 | 24 | 26 | 25 | 25 | 26 | 23 | 26 |
| XYLB | O75191 | 2.85E-11 | 1.52E-11 | -0.385138 | | 0.70498 | -1.44997 | | 14.7082 | 14.5844 | 14.6348 | 14.9784 | 15.1943 | 15.2777 | 15.403 | 14.2845 | 4 | 3 | 3 | 4 | 4 | 6 | 7 | 3 |
| SAC3D1 | A6NKF1 | 0.01011 | 0.001733 | -0.385005 | | 0.916513 | -1.8045 | | 15.5319 | 16.2154 | 16.1935 | 16.7495 | 16.4018 | 16.2036 | 16.2645 | 16.2069 | 3 | 4 | 2 | 2 | 2 | 1 | 3 | 3 |
| SIL1 | Q9H173 | 5.09E-18 | 1.80E-17 | -0.384818 | | 0.945114 | -1.85202 | | 15.6949 | 15.7801 | 15.7416 | 15.759 | 16.2806 | 15.8225 | 16.6064 | 16.1499 | 13 | 14 | 14 | 15 | 13 | 14 | 16 | 14 |
| CDK5RAP1 | Q96SZ6;Q9 | 0.014318 | 0.002417 | -0.384568 | | 0.423013 | -0.99151 | | 13.4082 | NaN | 12.7882 | 13.2263 | NaN | 13.0453 | 13.593 | 14.0666 | 1 | NaN | 2 | 2 | NaN | 1 | 2 | 2 |
| LGALS3BP | Q08380 | 0.000491 | 0.000106 | -0.384504 | | 0.595024 | -1.26112 | | 14.9472 | 14.7596 | 14.8887 | 15.192 | 15.3941 | 15.5733 | 15.6825 | 14.4064 | 1 | 1 | 2 | 2 | 2 | 3 | 3 | 1 |
| VPS37A | Q8NEZ2 | 1.15E-10 | 5.46E-11 | -0.384058 | + | 2.5565 | -4.87632 | | 14.7053 | 14.694 | 14.7696 | 15.0505 | 15.1566 | 15.0116 | 15.3106 | 15.0058 | 8 | 9 | 8 | 10 | 7 | 8 | 8 | 8 |
| SRCAP | Q6ZRS2;Q6 | 0.000943 | 0.000195 | -0.383675 | | 0.812815 | -1.63162 | | 15.5596 | 15.509 | 15.4069 | 15.5872 | 16.0756 | 15.3941 | 15.9448 | 16.3758 | 5 | 4 | 4 | 3 | 2 | 3 | 2 | 2 |
| CDR2L | Q86X02 | 7.94E-10 | 3.32E-10 | -0.383631 | + | 1.56847 | -2.90915 | | 15.2263 | 15.2944 | 15.3104 | 15.6788 | 15.3686 | 15.5317 | 15.9261 | 15.7981 | 5 | 4 | 5 | 6 | 4 | 8 | 8 | 7 |
| MAP1LC3B2;MAP1LC | A6NCE7;Q9 | 3.18E-05 | 7.94E-06 | -0.383188 | | 0.981384 | -1.91226 | | 17.7342 | 17.4307 | 17.4291 | 17.7169 | 18.0718 | 17.8599 | 17.5628 | 18.3611 | 2 | 2 | 2 | 2 | 3 | 2 | 2 | 2 |
| LGMN | Q99538;Q9 | 1.57E-09 | 6.34E-10 | -0.382649 | | 1.11874 | -2.14068 | | 16.4401 | 15.953 | 16.2848 | 16.4499 | 16.5727 | 16.4025 | 17.0168 | 16.6012 | 4 | 4 | 4 | 4 | 3 | 5 | 5 | 4 |
| PABPC4 | Q13310-2; | 2.43E-20 | 1.92E-19 | -0.382039 | + | 2.56929 | -4.90549 | | 16.9621 | 17.0468 | 17.0125 | 17.4286 | 17.2967 | 17.4289 | 17.5584 | 17.2333 | 17 | 18 | 18 | 17 | 17 | 18 | 18 | 18 |
| SLC38A10 | Q9HBR0 | 3.59E-14 | 3.87E-14 | -0.381416 | | 0.95479 | -1.8681 | | 14.958 | 15.0225 | 15.0572 | 15.0938 | 15.2078 | 15.6194 | 15.881 | 15.1679 | 13 | 12 | 11 | 12 | 8 | 13 | 16 | 8 |
| INPP5B | P32019;P3 | 0.004429 | 0.000799 | -0.381389 | | 0.410193 | -0.96572 | | 11.9603 | 12.5942 | 11.2943 | NaN | 12.4089 | 12.0899 | NaN | 12.4941 | 2 | 2 | 2 | NaN | 1 | 1 | NaN | 3 |
| GM2A | P17900 | 0.000289 | 6.42E-05 | -0.381322 | | 0.972219 | -1.89704 | | 16.4243 | 16.1694 | 16.5467 | 16.5306 | 16.5554 | 16.5241 | 17.0762 | 17.121 | 6 | 6 | 5 | 4 | 6 | 5 | 6 | 6 |
| CASC3 | O15234 | 2.35E-19 | 1.30E-18 | -0.38098 | | 0.65843 | -1.37058 | | 13.512 | 13.0912 | 13.6516 | 14.389 | 13.9557 | 13.2629 | 13.6017 | 13.7871 | 1 | 3 | 1 | 1 | 2 | 1 | 2 | 2 |
| NUTF2 | P61970 | 8.48E-12 | 4.99E-12 | -0.38081 | | 0.787533 | -1.58925 | | 18.674 | 18.3464 | 18.6989 | 18.4435 | 18.7882 | 18.9412 | 19.1513 | 19.4453 | 6 | 5 | 5 | 5 | 5 | 5 | 5 | 5 |
| TMX2 | Q9Y320 | 7.01E-11 | 3.46E-11 | -0.380069 | + | 3.24042 | -6.61447 | | 16.2839 | 16.2743 | 16.2565 | 16.5661 | 16.7756 | 16.5777 | 16.7326 | 16.6062 | 13 | 12 | 12 | 12 | 12 | 13 | 12 | 13 |
| ZBTB7A | O95365 | 2.76E-13 | 2.33E-13 | -0.3793 | | 1.24649 | -2.35474 | | 14.3131 | 14.1353 | 14.3265 | 14.9084 | 14.8537 | 14.2659 | 14.5338 | 14.6261 | 4 | 3 | 5 | 5 | 3 | 4 | 3 | 3 |
| RBPJ | Q06330;Q0 | 4.06E-08 | 1.37E-08 | -0.379075 | | 0.470468 | -1.03987 | | 15.2947 | 15.4118 | 15.3566 | 16.3072 | 15.995 | 15.2438 | 14.9317 | 16.1895 | 12 | 12 | 13 | 13 | 10 | 8 | 7 | 13 |
| AXL | P30530 | 1.04E-06 | 3.02E-07 | -0.378771 | | 0.771114 | -1.56167 | | 16.2081 | 16.3165 | 16.3026 | 17.2025 | 16.4652 | 16.1577 | 16.547 | 16.9001 | 11 | 12 | 12 | 11 | 10 | 7 | 10 | 13 |
| IKBKG | Q9Y6K9;Q9 | 5.98E-11 | 3.00E-11 | -0.377885 | + | 1.33935 | -2.51203 | | 15.8571 | 15.9819 | 15.8651 | 16.6376 | 16.3612 | 16.1106 | 15.9946 | 16.2922 | 9 | 8 | 8 | 9 | 9 | 9 | 9 | 9 |
| FCGRT | P55899 | 0.001414 | 0.000285 | -0.377497 | | 0.414892 | -0.9375 | | 14.5265 | 14.5109 | 14.3429 | 15.9633 | 14.488 | 14.9445 | 14.3167 | 14.4754 | 3 | 3 | 2 | 2 | 1 | 3 | 2 | 3 |
| WDR4 | P57081;P5 | 0.000338 | 7.46E-05 | -0.377259 | + | 2.40184 | -4.5326 | | 14.9782 | 14.9312 | 14.9681 | 15.341 | 15.4129 | 15.0955 | 15.421 | 15.4116 | 2 | 2 | 3 | 1 | 2 | 3 | 3 | 2 |
| SLC3A2 | P08195;P0 | 2.68E-11 | 1.43E-11 | -0.376769 | | 0.304493 | -0.7245 | | 18.3286 | 18.3526 | 18.3297 | 17.4966 | 18.6348 | 18.9168 | 19.9369 | 18.5835 | 21 | 21 | 21 | 19 | 19 | 22 | 22 | 21 |
| MALSU1 | Q96EH3 | 0.003893 | 0.000708 | -0.376473 | | 0.324895 | -0.76502 | | 13.9833 | 13.7599 | 13.9195 | 14.1142 | 15.0499 | 12.9242 | 14.5293 | 14.7028 | 1 | 1 | 2 | 2 | 1 | 2 | 1 | 2 |
| LEO1 | Q8WVC0 | 0.00027 | 6.02E-05 | -0.376447 | + | 2.02865 | -3.76335 | | 14.7494 | 14.7478 | 14.7343 | 15.1855 | 15.0663 | 14.86 | 15.1879 | 15.3017 | 6 | 6 | 7 | 6 | 5 | 5 | 6 | 6 |
| DNAJC5 | Q9H3Z4;Q9 | 5.93E-13 | 4.54E-13 | -0.376101 | + | 1.48889 | -2.76953 | | 14.0395 | 14.0644 | 14.161 | 14.7372 | 14.1395 | 14.5062 | 14.563 | 14.3761 | 3 | 4 | 4 | 4 | 3 | 4 | 4 | 3 |
| TGOLN2 | O43493;O4 | 2.05E-09 | 8.13E-10 | -0.375995 | + | 1.58333 | -2.93544 | | 15.0627 | 15.1994 | 15.2288 | 15.8214 | 15.2689 | 15.6031 | 15.5708 | 15.434 | 7 | 9 | 7 | 9 | 5 | 11 | 6 | 7 |
| DAAM1 | Q9Y4D1;Q9 | 9.23E-06 | 2.43E-06 | -0.375839 | | 0.765095 | -1.59429 | | 14.3713 | 14.3241 | 14.4125 | 14.6323 | NaN | 15.1179 | 14.2364 | 14.9939 | 3 | 4 | 4 | 3 | NaN | 5 | 2 | 2 |
| RBKS | Q9H477;Q9 | 0.000354 | 7.78E-05 | -0.3757 | | 1.09891 | -2.10764 | | 14.7905 | 14.8417 | 14.9979 | 14.85 | 15.1924 | 15.6612 | 15.2799 | 15.2784 | 1 | 1 | 1 | 2 | 1 | 2 | 1 | 1 |
| GTF2A2 | P52657 | 1.39E-07 | 4.43E-08 | -0.375637 | | 0.597231 | -1.26496 | | 16.3378 | 16.0845 | 16.3286 | 16.4789 | 16.3505 | 16.2221 | 16.6215 | 17.4567 | 2 | 1 | 2 | 2 | 1 | 2 | 2 | 1 |
| ANKRD13A | Q8IZ07 | 4.78E-13 | 3.75E-13 | -0.375438 | + | 1.53761 | -2.85478 | | 16.1569 | 16.153 | 16.2101 | 16.78 | 16.6988 | 16.3318 | 16.2993 | 16.634 | 15 | 16 | 17 | 18 | 16 | 16 | 15 | 16 |
| PAFAH1B2 | P68402 | 2.38E-06 | 6.63E-07 | -0.374469 | + | 1.71822 | -3.17765 | | 18.9242 | 18.7362 | 18.9084 | 19.0194 | 19.4415 | 19.2083 | 19.0928 | 19.3916 | 9 | 8 | 9 | 8 | 9 | 8 | 8 | 8 |
| RTN3 | O95197;O9 | 4.37E-09 | 1.67E-09 | -0.374025 | + | 1.96182 | -3.63353 | | 17.4521 | 17.5398 | 17.4418 | 18.1061 | 17.7098 | 17.7331 | 17.9403 | 17.7703 | 6 | 4 | 4 | 6 | 3 | 3 | 3 | 4 |
| UBAP1 | Q9NZ09;Q9 | 5.01E-05 | 1.23E-05 | -0.373562 | + | 1.8574 | -3.43494 | | 15.5508 | 15.2984 | 15.598 | 15.6326 | 16.0246 | 15.888 | 15.8954 | 15.8393 | 5 | 4 | 4 | 6 | 3 | 5 | 4 | 6 |
| NECTIN3 | Q9NQS3 | 4.02E-05 | 9.94E-06 | -0.373336 | + | 2.86257 | -5.60681 | | 14.5283 | 14.6931 | 14.6602 | 15.1045 | 14.9226 | 14.8842 | 15.0364 | 15.0551 | 4 | 3 | 4 | 4 | 5 | 4 | 4 | 4 |
| ARFIP1 | P53367 | 1.90E-05 | 4.85E-06 | -0.373327 | + | 1.60485 | -2.97364 | | 18.6431 | 18.4876 | 18.5644 | 18.5996 | 19.1242 | 18.9683 | 19.0609 | 18.9387 | 1 | 1 | 1 | 1 | 2 | 2 | 2 | 2 |
| ANKLE2 | Q86XL3 | 4.39E-14 | 4.62E-14 | -0.372711 | + | 2.81311 | -5.48392 | | 15.2927 | 15.3217 | 15.2655 | 15.6236 | 15.7816 | 15.5342 | 15.6055 | 15.7851 | 18 | 22 | 20 | 23 | 20 | 21 | 22 | 19 |
| HSDL1 | Q3SXM5 | 1.37E-10 | 6.44E-11 | -0.372677 | + | 1.38169 | -2.58437 | | 15.4247 | 15.5879 | 15.6828 | 15.975 | 16.2807 | 15.6706 | 15.9213 | 15.8415 | 7 | 8 | 7 | 6 | 4 | 7 | 6 | 8 |
| B3GAT3 | O94766 | 0.000278 | 6.19E-05 | -0.372489 | | 0.939463 | -1.84264 | | 16.6592 | 16.7111 | 16.6453 | 17.3449 | 17.3291 | 16.5133 | 17.0521 | 16.9823 | 4 | 4 | 5 | 7 | 6 | 4 | 5 | 5 |
| LOXL1 | Q08397 | 4.89E-09 | 1.85E-09 | -0.37214 | | 0.529413 | -1.14578 | | 15.3937 | 15.2307 | 15.4713 | 15.8195 | 16.404 | 15.391 | 15.0339 | 16.0385 | 3 | 3 | 3 | 3 | 3 | 3 | 3 | 3 |
| ZPR1 | O75312 | 7.35E-11 | 3.61E-11 | -0.372094 | + | 3.17855 | -6.44066 | | 15.8791 | 15.7713 | 15.8308 | 16.2871 | 16.1062 | 16.2426 | 16.1002 | 16.2597 | 15 | 17 | 15 | 16 | 13 | 16 | 16 | 15 |
| IRF2BP1 | Q8IU81 | 0.002736 | 0.000517 | -0.371675 | + | 2.65422 | -5.10198 | | 14.0377 | 14.0096 | 14.1195 | 14.2947 | 14.5731 | 14.3352 | 14.5038 | 14.4296 | 5 | 5 | 5 | 4 | 2 | 3 | 4 | 5 |
| NPEPPS | P55786 | 2.48E-13 | 2.12E-13 | -0.371534 | + | 5.99508 | -20.0096 | | 18.4105 | 18.4194 | 18.422 | 18.7505 | 18.8223 | 18.7758 | 18.8189 | 18.7768 | 40 | 39 | 40 | 41 | 39 | 40 | 40 | 39 |
| SLC35B2 | Q8TB61;Q8 | 1.71E-09 | 6.83E-10 | -0.37032 | + | 1.78941 | -3.30825 | | 16.9704 | 16.8611 | 16.9145 | 17.333 | 17.2815 | 16.9861 | 17.4851 | 17.3426 | 8 | 9 | 8 | 10 | 8 | 9 | 10 | 9 |
| MTMR12 | Q9C0I1 | 6.37E-05 | 1.54E-05 | -0.369121 | | 1.06185 | -2.04596 | | 14.4184 | 14.3072 | 14.289 | 14.4758 | 14.8699 | 15.1468 | 14.4369 | 14.6071 | 5 | 6 | 6 | 6 | 2 | 3 | 4 | 4 |
| RABGGTA | Q92696 | 6.53E-16 | 1.17E-15 | -0.369052 | + | 2.15928 | -4.02369 | | 16.1116 | 16.158 | 16.1045 | 16.3666 | 16.4146 | 16.5027 | 16.7521 | 16.4327 | 16 | 18 | 14 | 16 | 15 | 16 | 17 | 15 |
| KIF15 | Q9NS87;Q9 | 2.86E-13 | 2.41E-13 | -0.367484 | + | 1.86008 | -3.43997 | | 15.4118 | 15.4296 | 15.3965 | 15.7803 | 15.7389 | 15.6736 | 15.6261 | 16.0819 | 6 | 6 | 8 | 11 | 5 | 3 | 7 | 3 |
| LAPTM4A | Q15012 | 0.001852 | 0.000366 | -0.367108 | | 0.545441 | -1.17418 | | 15.9905 | 15.8491 | 16.1942 | 17.07 | 16.0309 | 15.8703 | 16.1698 | 16.7508 | 1 | 1 | 1 | 1 | 1 | 1 | 1 | 1 |
| GMEB1 | Q9Y692;Q9 | 0.008188 | 0.001421 | -0.366767 | | 0.696467 | -1.4355 | | 15.7863 | 16.0785 | 15.3302 | 16.507 | 15.6995 | 16.0592 | 15.8665 | 16.3602 | 2 | 2 | 2 | 1 | 2 | 1 | 1 | 2 |
| CANT1 | Q8WVQ1;C | 1.46E-06 | 4.17E-07 | -0.366625 | + | 1.69919 | -3.14307 | | 14.3591 | 14.4701 | 14.2264 | 14.8974 | 14.5342 | 14.556 | 14.8941 | 14.7107 | 10 | 9 | 9 | 10 | 6 | 6 | 8 | 8 |
| LPCAT1 | Q8NF37 | 0.000205 | 4.66E-05 | -0.366317 | | 0.699608 | -1.44084 | | 16.4721 | 16.2784 | 16.3457 | 17.4254 | 16.5574 | 16.3872 | 16.4679 | 16.8209 | 5 | 6 | 7 | 11 | 3 | 7 | 6 | 9 |
| PGS1 | Q32NB8;Q3 | 0.000139 | 3.23E-05 | -0.365937 | | 1.02673 | -1.98758 | | 15.1163 | 15.5272 | 14.8338 | 15.4083 | 15.6064 | 15.2982 | 15.7829 | 15.5295 | 2 | 2 | 3 | 2 | 4 | 2 | 2 | 3 |
| TYW5 | A2RUC4 | 0.001481 | 0.000298 | -0.364616 | + | 2.00142 | -3.71019 | | 14.3945 | 14.5238 | 14.3216 | 14.6715 | 14.7498 | 14.9722 | 14.8823 | 14.6138 | 3 | 5 | 6 | 5 | 4 | 4 | 4 | 3 |
| SLC30A7 | Q8NEW0 | 6.34E-06 | 1.70E-06 | -0.364208 | + | 1.47792 | -2.75045 | | 17.5941 | 17.5916 | 17.4812 | 17.6685 | 17.8114 | 18.0983 | 18.1918 | 17.8292 | 4 | 4 | 4 | 4 | 4 | 4 | 4 | 4 |
| BMP2K | Q9NSY1 | 3.96E-08 | 1.34E-08 | -0.364197 | + | 1.97176 | -3.6527 | | 16.0129 | 15.908 | 15.9257 | 16.1639 | 16.4139 | 16.1109 | 16.4173 | 16.4593 | 13 | 14 | 13 | 12 | 11 | 14 | 13 | 14 |
| SDAD1 | Q9NVU7 | 2.30E-14 | 2.62E-14 | -0.36404 | + | 2.85246 | -5.58152 | | 14.654 | 14.6827 | 14.5031 | 14.8816 | 14.9203 | 14.9518 | 15.0787 | 15.0541 | 8 | 8 | 8 | 7 | 6 | 8 | 6 | 8 |
| STX12 | Q86Y82 | 7.39E-07 | 2.18E-07 | -0.363608 | + | 1.86408 | -3.44749 | | 17.7581 | 17.6932 | 17.7755 | 18.3551 | 17.884 | 18.0171 | 18.1487 | 18.1244 | 10 | 10 | 10 | 10 | 10 | 10 | 10 | 10 |
| LAMTOR1 | Q6IAA8 | 1.77E-14 | 2.10E-14 | -0.363587 | + | 1.3958 | -2.60856 | | 17.784 | 17.9032 | 17.96 | 18.0758 | 18.2725 | 17.9821 | 18.55 | 18.3494 | 7 | 7 | 7 | 7 | 7 | 7 | 7 | 7 |
| HAGH | Q16775;Q1 | 1.32E-05 | 3.43E-06 | -0.362613 | + | 2.63995 | -5.06861 | | 16.1047 | 16.1016 | 16.0474 | 16.4142 | 16.5759 | 16.2958 | 16.3932 | 16.5568 | 8 | 8 | 8 | 8 | 7 | 7 | 8 | 7 |
| CRYAB | P02511 | 2.27E-07 | 7.10E-08 | -0.362483 | | 0.836786 | -1.67169 | | 18.7048 | 18.6782 | 18.7738 | 18.8919 | 19.0574 | 18.8926 | 18.8519 | 19.7133 | 13 | 12 | 14 | 11 | 14 | 14 | 12 | 14 |
| BECN1 | Q14457 | 0.001623 | 0.000325 | -0.362458 | + | 1.37132 | -2.56661 | | 13.6106 | 13.9319 | 14.1338 | 14.053 | 14.3884 | 14.2143 | 14.4064 | 14.2107 | 3 | 3 | 3 | 3 | 3 | 3 | 3 | 2 |
| ORMDL1 | Q9P0S3 | 2.35E-13 | 2.02E-13 | -0.362349 | | 0.828096 | -1.65717 | | 16.2545 | 16.1252 | 16.1446 | 16.8239 | 16.5528 | 16.0042 | 16.3923 | 16.9122 | 4 | 4 | 4 | 4 | 4 | 4 | 4 | 4 |
| THTPA | Q9BU02 | 0.000536 | 0.000115 | -0.361785 | + | 1.6628 | -3.07734 | | 16.5805 | 16.2298 | 16.6741 | 16.6764 | 16.8724 | 16.8909 | 16.8802 | 16.963 | 6 | 5 | 5 | 5 | 2 | 5 | 5 | 4 |
| FBXW2 | Q9UKT8 | 0.000264 | 5.90E-05 | -0.361215 | | 1.20744 | -2.39472 | | NaN | 13.8671 | 13.8934 | 13.9949 | 14.2735 | 14.0791 | 14.3914 | 14.4684 | NaN | 2 | 3 | 2 | 1 | 3 | 3 | 1 |

| Gene names | UniProt IDs | Protein Group P-value | Protein Group Q-value | Log2 Difference NUP214 vs Controls (n=5) | p<0.05 | -log10 p-value | T-test Test statistic | NPC Localization | Log2 MS2_quantity_NUP214_1 | Log2 MS2_quantity_NUP214_2 | Log2 MS2_quantity_NUP214_3 | Log2 MS2_quantity_Control_1 | Log2 MS2_quantity_Control_2 | Log2 MS2_quantity_Control_3 | Log2 MS2_quantity_Control_4 | Log2 MS2_quantity_Control_5 | Number Peptides for Quantitation NUP214_1 | Number Peptides for Quantitation NUP214_2 | Number Peptides for Quantitation NUP214_3 | Number Peptides for Quantitation Control_1 | Number Peptides for Quantitation Control_2 | Number Peptides for Quantitation Control_3 | Number Peptides for Quantitation Control_4 | Number Peptides for Quantitation Control_5 |
|---|---|---|---|---|---|---|---|---|---|---|---|---|---|---|---|---|---|---|---|---|---|---|---|---|
| PDCD6 | O75340 | 7.98E-22 | 9.72E-21 | -0.361036 | + | 1.69583 | -3.13697 | | 17.8902 | 17.7752 | 18.0024 | 18.2766 | 17.9478 | 18.3083 | 18.3155 | 18.4032 | 7 | 7 | 7 | 7 | 7 | 7 | 7 | 7 |
| FARP1 | Q9Y4F1;Q9 | 1.42E-23 | 3.17E-22 | -0.360397 | + | 1.77359 | -3.27905 | | 16.6533 | 16.6456 | 16.5983 | 16.7402 | 17.1449 | 17.0348 | 17.1699 | 16.8743 | 32 | 33 | 34 | 34 | 36 | 36 | 35 | 35 |
| EEF1A2 | Q05639 | 0.000393 | 8.59E-05 | -0.360118 | + | 1.48324 | -2.7597 | | 17.2798 | 17.5226 | 17.5749 | 17.9693 | 17.8724 | 17.896 | 17.8682 | 17.4901 | 2 | 2 | 2 | 2 | 2 | 2 | 2 | 2 |
| ERGIC3 | Q9Y282;Q9 | 5.87E-11 | 2.95E-11 | -0.360079 | + | 1.82868 | -3.38119 | | 15.3882 | 15.425 | 15.4213 | 15.5987 | 15.7219 | 15.6422 | 16.0363 | 15.8587 | 8 | 9 | 9 | 9 | 8 | 8 | 9 | 8 |
| NOTCH3 | Q9UM47 | 0.000181 | 4.14E-05 | -0.359954 | | 0.499147 | -1.0917 | | 14.2583 | 14.1186 | 14.1139 | 14.9396 | 14.7763 | 13.5676 | 14.7608 | 14.5736 | 6 | 5 | 5 | 2 | 4 | 3 | 6 | 5 |
| FASTKD2 | Q9NYY8 | 7.39E-07 | 2.18E-07 | -0.359583 | | 0.563015 | -1.20515 | | 14.7464 | 14.8249 | 14.7524 | 15.4975 | 15.3196 | 14.2587 | 15.2209 | 15.3738 | 7 | 6 | 6 | 7 | 5 | 6 | 8 | 6 |
| PRPS2 | P11908;P1 | 8.52E-07 | 2.50E-07 | -0.359357 | + | 2.28351 | -4.27995 | | 15.3261 | 15.5579 | 15.4937 | 15.9391 | 15.8656 | 15.8789 | 15.7511 | 15.6582 | 5 | 4 | 4 | 5 | 5 | 5 | 4 | 5 |
| TOMM40L | Q969M1;Q | 0.006284 | 0.001107 | -0.358294 | | 0.687991 | -1.42107 | | 14.6023 | 14.6939 | 14.4424 | 15.0176 | 15.6011 | 14.7049 | 14.8439 | 14.5215 | 2 | 2 | 2 | 1 | 1 | 1 | 2 | 1 |
| ISOC2 | Q96AB3;Q9 | 2.27E-12 | 1.50E-12 | -0.35829 | | 1.03501 | -2.00134 | | 15.4912 | 15.116 | 15.4247 | 15.3453 | 15.651 | 15.8728 | 15.6053 | 16.0367 | 2 | 2 | 2 | 2 | 2 | 2 | 2 | 2 |
| SURF1 | Q15526;Q1 | 0.000577 | 0.000123 | -0.357928 | | 0.728424 | -1.48969 | | 16.0383 | 15.9385 | 15.4802 | 16.7015 | 15.9921 | 15.7806 | 16.2516 | 16.1589 | 4 | 4 | 4 | 3 | 2 | 1 | 3 | 3 |
| CPNE8 | Q86YQ8 | 0.000871 | 0.000181 | -0.357503 | | 0.880694 | -1.7449 | | 19.3525 | 19.2429 | 19.3698 | 19.6859 | 20.0348 | 19.1455 | 19.6295 | 19.9005 | 3 | 2 | 3 | 1 | 3 | 3 | 3 | 1 |
| UTP3 | Q9NQZ2 | 6.54E-12 | 3.95E-12 | -0.357327 | + | 2.19775 | -4.1021 | | 14.6211 | 14.5334 | 14.6279 | 15.0631 | 14.7779 | 14.8826 | 15.1262 | 14.9077 | 7 | 7 | 7 | 8 | 7 | 7 | 6 | 6 |
| EIF4A2 | Q14240;Q1 | 1.56E-10 | 7.26E-11 | -0.357275 | + | 2.57553 | -4.91974 | | 17.9953 | 18.0587 | 17.9866 | 18.2298 | 18.2979 | 18.3515 | 18.529 | 18.4458 | 10 | 11 | 11 | 11 | 10 | 10 | 12 | 11 |
| HIF1AN | Q9NWT6 | 2.79E-06 | 7.73E-07 | -0.356889 | + | 1.35025 | -2.53062 | | 15.623 | 15.6277 | 15.5914 | 16.2512 | 15.8366 | 15.6418 | 16.0377 | 16.0874 | 4 | 5 | 5 | 5 | 4 | 5 | 4 | 5 |
| PEF1 | Q9UBV8 | 7.01E-11 | 3.46E-11 | -0.356578 | | 0.719342 | -1.47432 | | 17.2513 | 17.0336 | 17.3703 | 17.0753 | 17.4086 | 17.4602 | 18.0124 | 17.9184 | 5 | 4 | 5 | 5 | 5 | 5 | 5 | 5 |
| SUMO3 | P55854;P5 | 0.000162 | 3.73E-05 | -0.356446 | + | 2.11707 | -3.93857 | | 19.0656 | 18.9055 | 19.1989 | 19.5148 | 19.5445 | 19.3031 | 19.3224 | 19.3808 | 3 | 3 | 3 | 3 | 3 | 3 | 3 | 3 |
| WIPI2 | Q9Y4P8;Q9 | 4.00E-07 | 1.22E-07 | -0.356134 | | 1.28446 | -2.41884 | | 15.3447 | 15.4731 | 15.4838 | 16.1899 | 15.6255 | 15.8048 | 15.5831 | 15.7468 | 8 | 9 | 8 | 10 | 7 | 10 | 8 | 9 |
| ANXA2 | P07355 | 2.56E-24 | 7.58E-23 | -0.355839 | | 1.22405 | -2.31697 | | 23.1251 | 23.1166 | 23.0898 | 23.763 | 23.6474 | 23.502 | 23.136 | 23.2834 | 50 | 50 | 49 | 47 | 49 | 49 | 48 | 49 |
| TAF6 | P49848;P4 | 0.001061 | 0.000218 | -0.355784 | | 0.655326 | -1.36526 | | 13.3705 | 13.5142 | 13.5476 | 13.8528 | 14.4901 | 13.3674 | 13.922 | 13.5339 | 3 | 3 | 2 | 2 | 3 | 2 | 4 | 5 |
| C5orf24 | Q7Z6I8 | 3.33E-10 | 1.46E-10 | -0.355759 | | 1.175 | -2.23469 | | 13.7456 | 13.6194 | 13.9551 | 13.99 | 14.475 | 13.8386 | 14.1903 | 14.1519 | 3 | 3 | 2 | 1 | 2 | 3 | 2 | 1 |
| ENAH | Q8N8S7;Q8 | 3.18E-09 | 1.23E-09 | -0.355323 | | 1.07901 | -2.0745 | | 16.8923 | 16.894 | 16.9178 | 17.6615 | 16.8574 | 17.303 | 17.1934 | 17.2681 | 16 | 16 | 15 | 16 | 15 | 17 | 15 | 16 |
| FAM172A | Q8WUF8 | 4.47E-09 | 1.70E-09 | -0.355225 | + | 1.75378 | -3.24262 | | 15.2539 | 15.1377 | 15.2471 | 15.3159 | 15.7583 | 15.5728 | 15.7087 | 15.4849 | 7 | 7 | 7 | 8 | 6 | 7 | 7 | 7 |
| TRIOBP | Q9H2D6-5 | 7.62E-22 | 9.41E-21 | -0.354805 | + | 1.79735 | -3.32294 | | 16.4856 | 16.5022 | 16.5022 | 16.6204 | 17.087 | 16.9326 | 16.8756 | 16.7417 | 30 | 34 | 32 | 31 | 29 | 32 | 29 | 31 |
| MRPL49 | Q13405 | 2.52E-06 | 7.03E-07 | -0.354572 | | 0.490297 | -1.07577 | | 15.5033 | 15.039 | 15.8537 | 16.0933 | 15.6406 | 15.0838 | 16.0123 | 16.2697 | 6 | 4 | 6 | 7 | 5 | 6 | 6 | 7 |
| PIK3R1 | P27986;P2 | 7.47E-15 | 9.77E-15 | -0.354242 | | 1.15064 | -2.19394 | | 17.8392 | 17.7976 | 17.8269 | 18.1461 | 18.5747 | 17.9422 | 17.9223 | 18.292 | 13 | 14 | 14 | 17 | 14 | 12 | 13 | 16 |
| SPCS1 | Q9Y6A9 | 2.38E-06 | 6.63E-07 | -0.35423 | + | 2.14955 | -4.00398 | | 16.2727 | 16.4393 | 16.4856 | 16.9238 | 16.5988 | 16.7184 | 16.8288 | 16.6973 | 2 | 2 | 2 | 2 | 2 | 2 | 2 | 2 |
| HELZ2 | Q9BYK8 | 0.002625 | 0.000499 | -0.35406 | | 1.1948 | -2.26785 | | 14.0621 | 14.365 | 14.2604 | 14.9002 | 14.7727 | 14.4577 | 14.4268 | 14.359 | 7 | 7 | 7 | 8 | 6 | 6 | 3 | 6 |
| GLO1 | Q04760;Q0 | 1.64E-11 | 9.15E-12 | -0.353566 | | 1.04593 | -2.01949 | | 18.7031 | 18.4604 | 18.7612 | 18.5306 | 19.1732 | 19.1956 | 19.0027 | 19.0735 | 11 | 10 | 12 | 11 | 11 | 12 | 12 | 11 |
| CLUH | O75153 | 2.56E-24 | 7.58E-23 | -0.353548 | | 0.930941 | -1.82848 | | 15.433 | 15.4579 | 15.4854 | 16.1337 | 16.023 | 15.3272 | 15.6564 | 15.9212 | 32 | 32 | 30 | 31 | 23 | 29 | 27 | 33 |
| CERS5 | Q8N5B7;Q8 | 2.92E-05 | 7.32E-06 | -0.35331 | | 1.24405 | -2.35063 | | 14.5031 | 14.507 | 14.4324 | 15.0239 | 14.4068 | 14.8689 | 14.8642 | 15.0069 | 3 | 4 | 4 | 2 | 3 | 2 | 3 | 2 |
| BCAT1 | P54687 | 5.03E-18 | 1.79E-17 | -0.353236 | | 0.416519 | -0.94054 | | 17.4746 | 17.4269 | 17.4585 | 18.5227 | 17.4441 | 18.0823 | 18.0694 | 16.9142 | 11 | 11 | 12 | 12 | 11 | 11 | 12 | 11 |
| ZC3H7B | Q9UGR2 | 1.06E-13 | 1.00E-13 | -0.352766 | + | 1.55203 | -2.88014 | | 15.4274 | 15.3706 | 15.3882 | 16.0273 | 15.8197 | 15.4661 | 15.7467 | 15.6812 | 13 | 12 | 14 | 14 | 13 | 12 | 13 | 13 |
| CHRAC1 | Q9NRG0 | 5.48E-10 | 2.34E-10 | -0.352533 | | 0.738854 | -1.50732 | | 15.0775 | 15.0194 | 15.0506 | 15.5936 | 15.893 | 14.8701 | 15.1821 | 15.4696 | 2 | 3 | 2 | 3 | 3 | 2 | 2 | 3 |
| MAP7D1 | Q3KQU3 | 2.63E-15 | 3.99E-15 | -0.352075 | + | 1.71711 | -3.17562 | | 16.7584 | 16.701 | 16.695 | 17.2731 | 17.1089 | 16.8775 | 16.8802 | 17.2113 | 19 | 19 | 17 | 18 | 15 | 17 | 16 | 18 |
| SLC26A11 | Q86WA9 | 0.005231 | 0.000932 | -0.352018 | | 0.847074 | -1.68886 | | 15.3298 | 15.0528 | 15.3269 | 15.2921 | 16.0631 | 15.3086 | 15.7835 | 15.4953 | 3 | 3 | 3 | 3 | 2 | 3 | 4 | 3 |
| TRABD | Q9H4I3 | 6.68E-08 | 2.19E-08 | -0.35186 | | 1.18432 | -2.25028 | | 16.6256 | 16.2951 | 16.5087 | 17.0696 | 16.9228 | 16.4708 | 16.9481 | 16.7305 | 2 | 2 | 3 | 4 | 3 | 2 | 3 | 3 |
| MAP2K7 | O14733-3 | 0.002499 | 0.000477 | -0.351716 | + | 1.36907 | -2.56276 | | 15.111 | 14.7827 | 14.9406 | 15.2829 | 15.5399 | 15.3969 | 15.0011 | 15.2617 | 4 | 5 | 5 | 4 | 3 | 2 | 3 | 3 |
| RALGAPB | Q86X10;Q8 | 0.000974 | 0.000201 | -0.350178 | + | 2.35768 | -4.4373 | | 14.5039 | 14.5436 | 14.775 | 15.0198 | 15.0359 | 14.9098 | 14.8385 | 14.9845 | 4 | 4 | 2 | 5 | 2 | 4 | 3 | 3 |
| RMC1 | Q96DM3 | 6.31E-14 | 6.36E-14 | -0.349898 | + | 2.06398 | -3.83287 | | 15.0469 | 14.9891 | 14.9858 | 15.2901 | 15.2545 | 15.3749 | 15.6127 | 15.2537 | 6 | 7 | 8 | 8 | 6 | 7 | 10 | 8 |
| ANXA1 | P04083 | 8.26E-07 | 2.43E-07 | -0.349787 | + | 1.42885 | -2.66546 | | 21.7845 | 21.7835 | 21.7767 | 22.127 | 22.4315 | 21.8161 | 22.1861 | 22.0964 | 33 | 33 | 33 | 32 | 34 | 33 | 32 | 33 |
| CAMKK2 | Q96RR4;Q9 | 0.000111 | 2.60E-05 | -0.349029 | + | 2.11577 | -3.93598 | | 14.6931 | 15.0249 | 14.8993 | 15.2288 | 15.3689 | 15.1593 | 15.2093 | 15.1408 | 5 | 6 | 10 | 8 | 6 | 9 | 6 | 4 |
| MOV10 | Q9HCE1 | 1.15E-08 | 4.14E-09 | -0.348944 | | 1.06131 | -2.04507 | | 15.3949 | 15.4843 | 15.4393 | 16.0164 | 16.0048 | 15.3297 | 15.7113 | 15.8801 | 29 | 28 | 28 | 33 | 26 | 24 | 24 | 28 |
| FRA10AC1 | Q70Z53;Q7 | 0.002345 | 0.000452 | -0.348897 | | 0.277978 | -0.67906 | | 12.8998 | NaN | 13.576 | 14.1168 | 12.4688 | 13.7815 | 13.8569 | 13.7101 | 4 | NaN | 1 | 3 | 2 | 2 | 1 | 1 |
| LTBP3 | Q9NS15 | 0.002753 | 0.000519 | -0.348779 | | 0.709877 | -1.45828 | | 13.8865 | 14.1259 | 14.0177 | 13.9699 | 14.8381 | 14.7056 | 14.0454 | 14.235 | 3 | 6 | 4 | 5 | 4 | 4 | 2 | 4 |
| MADD | Q8WXG6-2 | 0.00148 | 0.000298 | -0.348684 | + | 1.8273 | -3.37861 | | 14.3963 | 14.4646 | 14.3133 | 14.6236 | 14.8026 | 14.5296 | 14.9484 | 14.7964 | 11 | 10 | 10 | 10 | 7 | 8 | 7 | 9 |
| ANXA11 | P50995;P5 | 1.32E-13 | 1.22E-13 | -0.348501 | + | 1.31325 | -2.46764 | | 19.2288 | 19.2518 | 19.2389 | 19.9712 | 19.6255 | 19.5557 | 19.3971 | 19.3921 | 24 | 25 | 24 | 25 | 25 | 25 | 24 | 24 |
| KCTD12 | Q96CX2 | 2.50E-05 | 6.30E-06 | -0.34821 | | 0.347962 | -0.81015 | | 18.529 | 18.5469 | 18.5952 | 20.1659 | 18.3315 | 18.6857 | 18.6684 | 18.6746 | 13 | 13 | 13 | 13 | 12 | 12 | 12 | 12 |
| OXNAD1 | Q96HP4 | 0.001792 | 0.000355 | -0.348144 | | 1.13974 | -2.17572 | | 16.9837 | 16.6795 | 16.3282 | 17.0376 | 17.1386 | 17.0576 | 16.7818 | 17.0442 | 3 | 3 | 2 | 2 | 1 | 2 | 2 | 3 |
| ANKIB1 | Q9P2G1 | 0.000997 | 0.000206 | -0.347739 | + | 2.86765 | -5.61954 | | 14.0179 | 14.1024 | 13.9552 | 14.2746 | 14.336 | 14.3574 | 14.3791 | 14.5173 | 5 | 7 | 8 | 6 | 6 | 7 | 7 | 4 |
| STX8 | Q9UNK0 | 2.77E-12 | 1.80E-12 | -0.347589 | + | 1.3554 | -2.5394 | | 16.1491 | 16.1381 | 16.1396 | 16.4936 | 16.4725 | 16.1371 | 16.77 | 16.576 | 7 | 7 | 8 | 8 | 7 | 7 | 8 | 8 |
| IREB2 | P48200 | 3.74E-10 | 1.63E-10 | -0.347313 | + | 3.09641 | -6.21543 | | 14.9514 | 14.9628 | 15.0639 | 15.4638 | 15.3295 | 15.3097 | 15.3605 | 15.2366 | 4 | 9 | 7 | 9 | 6 | 7 | 10 | 6 |
| OGFOD1 | Q8N543 | 0.000107 | 2.52E-05 | -0.347226 | | 1.24511 | -2.35241 | | 13.939 | 13.9922 | 14.0379 | 14.4807 | 14.5471 | 13.9216 | 14.3452 | 14.3898 | 10 | 10 | 12 | 12 | 5 | 10 | 9 | 10 |
| ATP11B | Q9Y2G3 | 0.000486 | 0.000105 | -0.347146 | + | 1.99133 | -3.69059 | | 15.2939 | 15.3386 | 15.3683 | 15.9 | 15.5581 | 15.7674 | 15.6573 | 15.5209 | 4 | 7 | 7 | 10 | 4 | 11 | 7 | 9 |
| EED | O75530;O7 | 2.44E-05 | 6.16E-06 | -0.346987 | + | 1.38749 | -2.59431 | | 14.4106 | 14.6904 | 14.7164 | 15.0598 | 14.9436 | 14.6273 | 15.0542 | 15.0792 | 3 | 4 | 6 | 5 | 3 | 2 | 3 | 3 |
| NECAP1 | Q8NC96 | 2.45E-15 | 3.76E-15 | -0.346906 | | 0.942097 | -1.84701 | | 15.5796 | 15.5119 | 15.6475 | 16.4463 | 15.6236 | 15.7734 | 15.8847 | 15.9048 | 6 | 5 | 3 | 5 | 4 | 5 | 4 | 5 |
| MPP5 | Q8N3R9 | 4.67E-06 | 1.27E-06 | -0.346731 | + | 1.67109 | -3.09227 | | 15.3614 | 15.3009 | 15.2664 | 15.7667 | 15.6704 | 15.4835 | 15.8966 | 15.4644 | 14 | 14 | 14 | 15 | 9 | 14 | 14 | 11 |
| TRIM13 | O60858;O6 | 2.65E-14 | 2.97E-14 | -0.34642 | | 0.515714 | -1.12138 | | 12.5282 | 12.2975 | 12.347 | 12.9212 | 11.8801 | 13.1854 | 13.007 | 12.6928 | 2 | 1 | 1 | 2 | 1 | 2 | 1 | 1 |
| TNIP1 | Q15025;Q1 | 3.32E-06 | 9.13E-07 | -0.34626 | | 0.553279 | -1.18801 | | 14.4906 | 14.5869 | 14.5197 | 14.4971 | 15.4143 | 14.4931 | 14.5803 | 15.4084 | 4 | 5 | 6 | 5 | 4 | 5 | 2 | 2 |
| SCARF2 | Q96GP6;Q9 | 6.73E-08 | 2.21E-08 | -0.346181 | | 1.09834 | -2.10669 | | 14.5678 | 14.5904 | 14.3597 | 15.2898 | 14.7896 | 14.5883 | 14.8069 | 14.7862 | 6 | 6 | 8 | 7 | 6 | 5 | 8 | 4 |
| PACS1 | Q6VY07 | 5.82E-13 | 4.47E-13 | -0.345799 | + | 1.96678 | -3.64309 | | 16.0553 | 16.0737 | 16.0461 | 16.5232 | 16.3036 | 16.6108 | 16.226 | 16.3571 | 24 | 25 | 24 | 25 | 21 | 28 | 25 | 25 |
| FAM160A2 | Q8N612;Q8 | 0.001479 | 0.000298 | -0.345662 | | 0.858679 | -1.70822 | | 14.6168 | 15.0101 | 14.8856 | 14.9608 | 15.6996 | 15.2224 | 15.0735 | 14.9594 | 2 | 3 | 4 | 5 | 2 | 4 | 3 | 4 |
| LPAR1 | Q92633;Q9 | 2.15E-08 | 7.50E-09 | -0.345447 | | 0.372157 | -0.85677 | | 15.9007 | 15.8698 | 15.8209 | 17.0923 | 16.208 | 16.5762 | 15.3284 | 15.8414 | 3 | 3 | 3 | 3 | 3 | 3 | 3 | 3 |
| TOLLIP | Q9H0E2 | 0.000294 | 6.52E-05 | -0.345343 | + | 2.37765 | -4.48023 | | 17.4777 | 17.3949 | 17.4495 | 17.7828 | 17.6588 | 17.9546 | 17.8617 | 17.6723 | 10 | 9 | 9 | 9 | 9 | 9 | 9 | 9 |
| KCTD9 | Q7L273 | 7.24E-05 | 1.74E-05 | -0.344349 | | 0.659123 | -1.37177 | | 15.1154 | 15.1528 | 14.9562 | 15.6055 | 16.0614 | 15.1505 | 15.2134 | 15.065 | 5 | 4 | 6 | 6 | 3 | 3 | 4 | 6 |
| PON2 | Q15165;Q1 | 1.63E-13 | 1.46E-13 | -0.344088 | | 0.930375 | -1.82753 | | 17.7213 | 17.6806 | 17.7402 | 18.2524 | 18.4217 | 17.6113 | 17.8997 | 18.1055 | 4 | 5 | 4 | 6 | 4 | 5 | 5 | 5 |
| FAM3C | Q92520 | 1.74E-13 | 1.55E-13 | -0.344072 | | 1.26227 | -2.38134 | | 17.2579 | 17.2761 | 17.2029 | 17.8845 | 17.4434 | 17.6058 | 17.2731 | 17.7418 | 12 | 11 | 12 | 12 | 10 | 12 | 11 | 12 |
| TXNDC9 | O14530 | 3.21E-12 | 2.05E-12 | -0.343734 | | 0.863902 | -1.71693 | | 15.1156 | 15.1585 | 15.0426 | 15.885 | 15.344 | 14.9939 | 15.3935 | 15.63 | 7 | 7 | 7 | 10 | 6 | 7 | 7 | 7 |
| SH3BGRL3 | Q9H299 | 3.43E-06 | 9.45E-07 | -0.343607 | | 0.324645 | -0.76453 | | 19.8209 | 19.7195 | 20.0638 | 20.3542 | 19.653 | 19.3882 | 20.3672 | 21.2957 | 5 | 4 | 3 | 4 | 3 | 3 | 3 | 5 |
| KIAA1217 | Q5T5P2 | 6.61E-05 | 1.60E-05 | -0.343516 | | 0.558056 | -1.19643 | | 14.9099 | 14.8769 | 14.887 | 15.9272 | 15.2197 | 14.8957 | 14.6948 | 15.4365 | 19 | 17 | 20 | 26 | 17 | 10 | 14 | 18 |
| CLP1 | Q92989 | 0.002352 | 0.000453 | -0.343488 | | 0.693904 | -1.43114 | | 14.7649 | 14.8662 | 14.6372 | 14.5464 | 15.0552 | 15.6265 | 15.2643 | 15.0056 | 4 | 4 | 4 | 4 | 3 | 2 | 4 | 4 |
| CAVIN1 | Q6NZI2 | 6.19E-17 | 1.55E-16 | -0.343418 | | 0.96565 | -1.88614 | | 20.6304 | 20.6021 | 20.5971 | 20.4993 | 21.3511 | 21.0286 | 20.9757 | 20.9116 | 20 | 21 | 22 | 20 | 22 | 20 | 21 | 20 |
| CHD8 | Q9HCK8;Q9 | 7.60E-05 | 1.83E-05 | -0.343288 | + | 1.37397 | -2.57115 | | 14.2687 | 14.7133 | 14.4303 | 14.9201 | 14.8421 | 14.539 | 14.8555 | 14.9136 | 4 | 3 | 2 | 5 | 2 | 4 | 4 | 4 |
| APOOL | Q6UXV4 | 2.18E-13 | 1.89E-13 | -0.34322 | + | 1.65995 | -3.0722 | | 15.9902 | 16.1635 | 16.0325 | 16.3053 | 16.3936 | 16.6518 | 16.4863 | 16.1894 | 11 | 11 | 12 | 11 | 11 | 13 | 12 | 11 |
| CD46 | P15529;P1 | 1.43E-05 | 3.69E-06 | -0.343219 | | 0.638772 | -1.33682 | | 15.5314 | 15.4882 | 15.0798 | 16.3222 | 15.619 | 15.3657 | 15.3974 | 15.8441 | 2 | 2 | 2 | 2 | 2 | 2 | 2 | 1 |
| ACSF2 | Q96CM8;Q | 2.79E-10 | 1.24E-10 | -0.343011 | | 0.776307 | -1.5704 | | 13.893 | 14.2693 | 14.1213 | 14.4355 | 14.8405 | 14.4609 | 13.8999 | 14.5509 | 9 | 10 | 9 | 9 | 9 | 8 | 7 | 10 |
| RAB11FIP5 | Q9BXF6 | 9.86E-14 | 9.42E-14 | -0.341894 | | 0.973287 | -1.89882 | | 16.8815 | 16.9542 | 17.0033 | 17.7652 | 17.1337 | 17.059 | 17.0853 | 17.398 | 28 | 26 | 25 | 26 | 23 | 26 | 27 | 26 |
| RRM1 | P23921 | 4.45E-17 | 1.16E-16 | -0.341814 | | 0.693042 | -1.42967 | | 16.3888 | 16.4187 | 16.3701 | 17.0702 | 16.5767 | 16.1065 | 16.8925 | 17.0259 | 23 | 23 | 22 | 22 | 21 | 22 | 22 | 23 |
| LRRFIP2 | Q9Y608-4 | 1.93E-11 | 1.06E-11 | -0.341692 | | 1.21047 | -2.29415 | | 16.249 | 16.2761 | 16.2032 | 16.6451 | 16.8417 | 16.1723 | 16.5951 | 16.668 | 16 | 18 | 19 | 22 | 18 | 18 | 17 | 18 |

| Gene names | UniProt IDs | Protein Group P-value | Protein Group Q-value | Log2 Difference NUP214 vs Controls (n=5) | p<0.05 | -log10 p-value | T-test Test statistic | NPC Localization | Log2 MS2_quantity_NUP214_1 | Log2 MS2_quantity_NUP214_2 | Log2 MS2_quantity_NUP214_3 | Log2 MS2_quantity_Control_1 | Log2 MS2_quantity_Control_2 | Log2 MS2_quantity_Control_3 | Log2 MS2_quantity_Control_4 | Log2 MS2_quantity_Control_5 | Number Peptides for Quantitation NUP214_1 | Number Peptides for Quantitation NUP214_2 | Number Peptides for Quantitation NUP214_3 | Number Peptides for Quantitation Control_1 | Number Peptides for Quantitation Control_2 | Number Peptides for Quantitation Control_3 | Number Peptides for Quantitation Control_4 | Number Peptides for Quantitation Control_5 |
|---|---|---|---|---|---|---|---|---|---|---|---|---|---|---|---|---|---|---|---|---|---|---|---|---|
| RER1 | O15258 | 1.20E-08 | 4.32E-09 | -0.341492 | + | 2.29787 | -4.31017 | | 18.6272 | 18.5924 | 18.6065 | 18.8692 | 18.8434 | 18.923 | 19.1764 | 18.939 | 7 | 7 | 7 | 7 | 7 | 7 | 7 | 7 |
| DNAJB14 | Q8TBM8 | 0.003745 | 0.000684 | -0.341311 | + | 1.72759 | -3.19472 | | 13.9788 | 14.2711 | 14.2115 | 14.3788 | 14.6676 | 14.3236 | 14.5784 | 14.5272 | 2 | 3 | 2 | 5 | 1 | 2 | 3 | 3 |
| DLG1 | Q12959;Q1 | 2.40E-16 | 4.97E-16 | -0.340996 | | 1.02138 | -1.97869 | | 15.2201 | 15.2899 | 15.1925 | 15.1994 | 15.6904 | 15.8167 | 15.8256 | 15.3437 | 20 | 20 | 21 | 19 | 17 | 20 | 17 | 16 |
| HACE1 | Q8IYU2 | 0.000846 | 0.000176 | -0.340982 | | 1.15072 | -2.19408 | | 13.8089 | 13.9957 | 13.6583 | 14.1066 | 14.3346 | 14.0345 | 14.4546 | 13.8794 | 2 | 2 | 3 | 3 | 1 | 2 | 3 | 2 |
| VTI1A | Q96AJ9;Q9 | 6.73E-05 | 1.63E-05 | -0.340833 | | 0.999141 | -1.94175 | | 14.8557 | 14.8822 | 14.8534 | 15.3864 | 15.1985 | 14.7505 | 15.5283 | 15.1594 | 5 | 5 | 5 | 6 | 4 | 5 | 5 | 6 |
| HRAS | P01112 | 5.11E-13 | 3.99E-13 | -0.340106 | + | 2.73556 | -5.29511 | | 17.0749 | 17.2403 | 17.0574 | 17.4589 | 17.4761 | 17.4866 | 17.5617 | 17.3383 | 2 | 2 | 2 | 2 | 2 | 2 | 2 | 2 |
| TMED4 | Q7Z7H5;Q7 | 2.67E-07 | 8.30E-08 | -0.339581 | + | 2.9109 | -5.7288 | | 17.1524 | 17.258 | 17.2716 | 17.532 | 17.4935 | 17.5883 | 17.7108 | 17.5099 | 6 | 6 | 7 | 6 | 6 | 6 | 6 | 6 |
| ERBIN | Q96RT1;Q9 | 7.06E-18 | 2.37E-17 | -0.339566 | + | 1.88725 | -3.49121 | | 15.7732 | 15.7592 | 15.8161 | 15.9827 | 16.3566 | 16.0156 | 16.0314 | 16.2254 | 35 | 36 | 36 | 34 | 33 | 34 | 35 | 34 |
| SESN2 | P58004 | 0.002463 | 0.000471 | -0.338559 | | 0.719472 | -1.47454 | | 14.898 | 14.7101 | 14.7861 | 14.7856 | 14.9063 | 15.642 | 15.4399 | 14.9093 | 3 | 4 | 6 | 4 | 2 | 4 | 5 | 4 |
| OSTC | Q9NRP0;Q9 | 0.00018 | 4.11E-05 | -0.338295 | | 0.609006 | -1.2854 | | 19.7747 | 19.5966 | 19.8311 | 19.3939 | 20.1315 | 20.3475 | 20.5189 | 19.9705 | 3 | 3 | 3 | 3 | 3 | 3 | 3 | 3 |
| LMF1 | Q96S06 | 3.63E-05 | 9.01E-06 | -0.338022 | | 0.701284 | -1.44369 | | 16.1639 | 15.7215 | 15.8316 | 15.6692 | 16.4274 | 16.5506 | 16.4422 | 16.129 | 2 | 2 | 2 | 2 | 2 | 2 | 2 | 2 |
| YIPF4 | Q9BSR8 | 0.00084 | 0.000175 | -0.337324 | + | 1.84597 | -3.41351 | | 17.654 | 17.6521 | 17.5296 | 17.9449 | 17.8297 | 17.785 | 18.1853 | 18.0012 | 2 | 2 | 2 | 2 | 2 | 2 | 2 | 2 |
| SLC30A5 | Q8TAD4 | 0.000274 | 6.09E-05 | -0.337256 | + | 1.34388 | -2.51974 | | 14.5098 | 14.475 | 14.5152 | 14.5702 | 14.7281 | 14.8593 | 15.1789 | 14.85 | 7 | 7 | 6 | 5 | 5 | 5 | 6 | 6 |
| PPM1F | P49593 | 1.37E-12 | 9.54E-13 | -0.336928 | + | 2.64693 | -5.08491 | | 17.1976 | 17.1999 | 17.1431 | 17.5373 | 17.6437 | 17.4811 | 17.3534 | 17.5702 | 12 | 12 | 13 | 13 | 12 | 13 | 11 | 12 |
| G3BP2 | Q9UN86 | 1.92E-13 | 1.69E-13 | -0.336913 | + | 2.12171 | -3.94787 | | 16.774 | 16.8808 | 16.8197 | 17.3972 | 17.0523 | 17.0769 | 17.1602 | 17.1221 | 16 | 15 | 15 | 17 | 13 | 16 | 16 | 16 |
| PLA2G15 | Q8NCC3 | 4.32E-05 | 1.07E-05 | -0.336716 | | 0.668099 | -1.38713 | | 16.0953 | 16.0613 | 16.0379 | 16.7202 | 16.6537 | 15.7064 | 16.5143 | 16.4131 | 8 | 7 | 7 | 9 | 7 | 7 | 7 | 8 |
| NOP14 | P78316 | 5.99E-05 | 1.46E-05 | -0.336274 | + | 1.45053 | -2.70292 | | 15.1381 | 15.3561 | 15.1484 | 15.7118 | 15.2275 | 15.659 | 15.5904 | 15.5638 | 9 | 10 | 10 | 13 | 9 | 13 | 9 | 11 |
| ERCC6 | Q03468 | 3.51E-05 | 8.74E-06 | -0.336067 | | 0.705666 | -1.45113 | | 14.831 | 14.4795 | 14.858 | 14.5655 | 15.1093 | 15.0165 | 15.5742 | 15.029 | 3 | 2 | 3 | 2 | 2 | 2 | 3 | 3 |
| RIPK2 | O43353 | 0.001706 | 0.00034 | -0.335931 | | 0.670363 | -1.391 | | 14.5145 | 14.4919 | 14.3057 | 14.4054 | 15.2603 | 14.3239 | 14.9591 | 14.9179 | 6 | 7 | 6 | 6 | 9 | 7 | 9 | 8 |
| CAV1 | Q03135-2 | 7.43E-07 | 2.19E-07 | -0.335925 | | 0.662063 | -1.3768 | | 20.4164 | 20.4245 | 20.4701 | 20.2097 | 21.2991 | 20.695 | 21.0054 | 20.6554 | 9 | 9 | 9 | 9 | 9 | 9 | 9 | 9 |
| FKBP5 | Q13451 | 2.39E-14 | 2.70E-14 | -0.335921 | | 0.370439 | -0.91838 | | 13.6925 | 13.7252 | 13.679 | 14.525 | NaN | NaN | NaN | 13.5446 | 5 | 2 | 4 | 8 | NaN | NaN | NaN | 3 |
| PTPRF | P10586;P1 | 1.01E-16 | 2.40E-16 | -0.335885 | | 0.32523 | -0.76568 | | 14.3004 | 14.193 | 14.2899 | 15.7471 | 14.8306 | 14.3678 | 13.844 | 14.1956 | 3 | 4 | 4 | 8 | 4 | 3 | 4 | 3 |
| MTHFR | P42898;P4 | 0.000368 | 8.07E-05 | -0.335589 | | 1.21343 | -2.29913 | | 14.1991 | 14.1405 | 14.337 | 14.7102 | 14.8505 | 14.4872 | 14.2348 | 14.5229 | 3 | 5 | 7 | 4 | 7 | 6 | 4 | 5 |
| GFPT2 | O94808 | 7.65E-23 | 1.33E-21 | -0.334714 | | 0.733544 | -1.49835 | | 16.6787 | 16.7518 | 16.6814 | 17.3693 | 16.6536 | 17.5015 | 16.797 | 16.8722 | 23 | 25 | 23 | 28 | 19 | 27 | 22 | 19 |
| CUX1 | P39880-9 | 9.96E-15 | 1.27E-14 | -0.334594 | | 1.28625 | -2.42188 | | 14.547 | 14.7437 | 14.772 | 15.0675 | 15.1707 | 14.866 | 15.2636 | 14.743 | 4 | 3 | 3 | 3 | 3 | 2 | 3 | 2 |
| DOCK10 | Q96BY6;Q9 | 3.13E-13 | 2.61E-13 | -0.33417 | + | 1.4486 | -2.69957 | | 15.1989 | 15.1995 | 15.2247 | 15.5607 | 15.8628 | 15.3922 | 15.5654 | 15.3283 | 39 | 42 | 41 | 41 | 35 | 38 | 44 | 36 |
| RNF126 | Q9BV68 | 2.40E-07 | 7.50E-08 | -0.334018 | + | 1.90185 | -3.51887 | | 15.7939 | 15.7057 | 15.8623 | 16.0589 | 16.3636 | 16.1334 | 15.9625 | 16.0881 | 5 | 6 | 5 | 5 | 5 | 5 | 6 | 6 |
| ACYP1 | P07311 | 4.61E-13 | 3.64E-13 | -0.333909 | | 0.853771 | -1.70004 | | 15.9377 | 15.808 | 15.9656 | 16.4198 | 16.3872 | 15.6755 | 16.2486 | 16.4572 | 4 | 4 | 4 | 3 | 3 | 4 | 5 | 4 |
| TNFAIP8L3 | Q5GJ75 | 0.000949 | 0.000196 | -0.333903 | | 0.434183 | -0.97334 | | 13.4818 | 13.4299 | 13.6364 | 14.7949 | 13.8177 | 13.3169 | 13.5008 | 13.8193 | 4 | 3 | 3 | 3 | 1 | 1 | 1 | 2 |
| BCAR3 | O75815 | 2.25E-10 | 1.02E-10 | -0.333808 | | 0.629307 | -1.32051 | | 15.1419 | 15.1685 | 15.1881 | 15.6228 | 15.8777 | 14.9149 | 15.2149 | 15.8694 | 17 | 18 | 15 | 21 | 16 | 15 | 13 | 17 |
| IFT57 | Q9NWB7 | 0.015223 | 0.002562 | -0.333331 | | 1.07059 | -2.27774 | | 13.8424 | 14.103 | 14.0057 | 14.1054 | NaN | 14.5382 | 14.3075 | NaN | 3 | 3 | 2 | 2 | NaN | 3 | 1 | NaN |
| ANKRD54 | Q6NXT1 | 7.80E-14 | 7.68E-14 | -0.333322 | | 0.786343 | -1.58726 | | 14.5701 | 14.0994 | 13.7523 | 14.4525 | 14.6896 | 14.1639 | 14.6003 | 14.4634 | 4 | 4 | 3 | 3 | 3 | 2 | 2 | 3 |
| HSPA13 | P48723 | 2.68E-16 | 5.42E-16 | -0.332934 | | 1.02386 | -1.98281 | | 16.2209 | 16.1148 | 16.0878 | 16.0892 | 16.7448 | 16.3876 | 16.7451 | 16.404 | 10 | 12 | 10 | 10 | 9 | 9 | 11 | 9 |
| CLCN3 | P51790;P5 | 1.18E-06 | 3.39E-07 | -0.332641 | + | 2.14423 | -3.99323 | | 14.9951 | 15.3021 | 15.1677 | 15.5009 | 15.4861 | 15.4703 | 15.6133 | 15.3675 | 6 | 6 | 7 | 7 | 5 | 7 | 8 | 6 |
| GGA1 | Q9UJY5;Q9 | 2.95E-10 | 1.31E-10 | -0.331859 | + | 1.47919 | -2.75265 | | 15.5755 | 15.6131 | 15.4748 | 16.0522 | 16.0237 | 15.6181 | 15.7377 | 15.9999 | 8 | 9 | 10 | 9 | 8 | 8 | 8 | 10 |
| MYO9B | Q13459 | 1.62E-14 | 1.93E-14 | -0.331189 | + | 2.09575 | -3.89596 | | 15.6213 | 15.5898 | 15.6092 | 16.1032 | 15.9608 | 15.8974 | 15.722 | 16.0062 | 57 | 55 | 56 | 58 | 51 | 58 | 56 | 59 |
| ACYP2 | P14621 | 0.002542 | 0.000485 | -0.331168 | | 0.808555 | -1.62449 | | 15.7464 | 15.7096 | 15.8711 | 15.8582 | 15.7409 | 16.0678 | 16.579 | 16.2885 | 3 | 3 | 3 | 3 | 3 | 3 | 3 | 3 |
| TAB2 | Q9NYJ8 | 9.77E-12 | 5.69E-12 | -0.330509 | + | 1.61457 | -2.99096 | | 14.7398 | 14.7282 | 14.7879 | 14.8185 | 15.3228 | 15.0544 | 15.0546 | 15.1621 | 5 | 4 | 5 | 4 | 4 | 4 | 4 | 4 |
| SPECC1L | Q69YQ0;Q6 | 2.38E-10 | 1.08E-10 | -0.33035 | + | 3.35807 | -6.95527 | | 16.9554 | 16.9016 | 16.9516 | 17.2553 | 17.3045 | 17.3586 | 17.1502 | 17.2641 | 21 | 22 | 22 | 20 | 19 | 22 | 22 | 23 |
| RPL27A | P46776 | 5.12E-09 | 1.93E-09 | -0.330242 | | 0.440886 | -0.98572 | | 21.6413 | 21.5154 | 21.5842 | 21.691 | 22.8167 | 21.8374 | 21.2941 | 21.9134 | 6 | 6 | 6 | 6 | 6 | 6 | 6 | 6 |
| SMG9 | Q9H0W8 | 3.04E-09 | 1.18E-09 | -0.330068 | + | 1.91663 | -3.54698 | | 15.2804 | 15.4554 | 15.3619 | 15.5505 | 15.8039 | 15.8624 | 15.7129 | 15.5502 | 7 | 7 | 6 | 6 | 8 | 7 | 7 | 5 |
| URGCP | Q8TCY9;Q8 | 0.002081 | 0.000406 | -0.329567 | | 1.23574 | -2.33664 | | 15.1517 | 15.2992 | 15.2676 | 15.714 | 15.6309 | 15.1634 | 15.7042 | 15.6328 | 7 | 7 | 10 | 7 | 6 | 5 | 8 | 8 |
| ABCF3 | Q9NUQ8;Q | 1.63E-10 | 7.58E-11 | -0.329439 | + | 3.06408 | -6.12845 | | 16.2791 | 16.3243 | 16.2646 | 16.7297 | 16.6466 | 16.5007 | 16.6491 | 16.5677 | 16 | 18 | 17 | 20 | 14 | 19 | 17 | 16 |
| TIMP1 | P01033 | 1.85E-14 | 2.17E-14 | -0.329415 | | 0.441866 | -0.98752 | | 17.653 | 17.6945 | 17.6062 | 17.6731 | 17.7193 | 17.7847 | 18.9771 | 17.7489 | 6 | 6 | 6 | 6 | 6 | 6 | 6 | 6 |
| MORF4L1 | Q9UBU8-2 | 0.00108 | 0.000222 | -0.32904 | + | 1.56422 | -2.90164 | | 14.6467 | 14.6079 | 14.8224 | 15.1435 | 14.7915 | 14.8806 | 15.1395 | 15.1518 | 7 | 7 | 7 | 7 | 5 | 5 | 7 | 6 |
| TANGO6 | Q9C0B7 | 7.75E-18 | 2.57E-17 | -0.32839 | | 1.30013 | -2.44538 | | 14.7977 | 14.678 | 14.9198 | 15.1093 | 15.4349 | 14.8468 | 15.1206 | 15.1229 | 7 | 5 | 8 | 6 | 4 | 4 | 4 | 6 |
| AKAP9 | Q99996;Q9 | 8.62E-05 | 2.05E-05 | -0.327466 | + | 3.43582 | -7.18817 | | 14.4971 | 14.4127 | 14.4192 | 14.8495 | 14.7142 | 14.776 | 14.8238 | 14.6889 | 19 | 23 | 27 | 29 | 11 | 23 | 25 | 26 |
| SLC7A5 | Q01650 | 3.60E-06 | 9.88E-07 | -0.327198 | | 0.17844 | -0.458 | | 17.3013 | 17.0362 | 17.1178 | 15.6097 | 17.5115 | 17.7942 | 18.93 | 17.5494 | 5 | 5 | 5 | 5 | 5 | 5 | 6 | 5 |
| PUDP | Q08623;Q0 | 2.65E-11 | 1.42E-11 | -0.327173 | | 0.883174 | -1.74903 | | 15.6632 | 15.6598 | 15.8388 | 15.845 | 16.2888 | 16.1828 | 15.6146 | 16.3078 | 6 | 5 | 6 | 5 | 4 | 5 | 2 | 5 |
| LPP | Q93052 | 2.75E-11 | 1.47E-11 | -0.32701 | + | 2.14594 | -3.99668 | | 17.4266 | 17.3938 | 17.42 | 17.7802 | 17.7394 | 17.9469 | 17.6015 | 17.6345 | 26 | 27 | 26 | 25 | 26 | 27 | 24 | 26 |
| RHOC | P08134 | 3.90E-05 | 9.65E-06 | -0.326978 | | 0.843626 | -1.68311 | | 19.5612 | 19.0896 | 19.4656 | 19.7695 | 20.0795 | 19.3376 | 19.5652 | 19.7438 | 3 | 3 | 2 | 3 | 2 | 3 | 2 | 3 |
| HSPA5 | P11021 | 2.34E-09 | 9.20E-10 | -0.326945 | + | 2.49491 | -4.73754 | | 21.2539 | 21.2435 | 21.2483 | 21.3878 | 21.7007 | 21.611 | 21.6113 | 21.5668 | 50 | 49 | 50 | 48 | 49 | 50 | 50 | 49 |
| NECAP2 | Q9NVZ3;Q9 | 2.21E-06 | 6.20E-07 | -0.326567 | | 1.02464 | -1.98411 | | 17.8358 | 18.0112 | 18.0442 | 18.6737 | 17.9573 | 18.3833 | 18.2562 | 18.1809 | 4 | 4 | 4 | 5 | 5 | 5 | 5 | 5 |
| CTSA | P10619;P1 | 1.83E-10 | 8.46E-11 | -0.326381 | | 0.825248 | -1.65241 | | 18.1748 | 18.2057 | 18.2051 | 18.1713 | 18.4846 | 18.4995 | 19.0653 | 18.387 | 10 | 10 | 12 | 11 | 12 | 11 | 11 | 11 |
| GCLC | P48506 | 4.00E-09 | 1.53E-09 | -0.325503 | | 0.626169 | -1.3151 | | 15.3557 | 15.3956 | 15.4831 | 15.1186 | 15.6446 | 15.8161 | 16.2579 | 15.8476 | 8 | 9 | 8 | 8 | 10 | 8 | 10 | 9 |
| TMEM184C | Q9NVA4 | 4.40E-05 | 1.09E-05 | -0.325491 | + | 1.58393 | -2.93651 | | 16.0258 | 15.954 | 16.0659 | 16.2912 | 16.0711 | 16.3321 | 16.5427 | 16.4664 | 1 | 1 | 1 | 1 | 1 | 1 | 2 | 1 |
| TMEM30A | Q9NV96 | 0.00059 | 0.000126 | -0.325343 | | 1.16779 | -2.22262 | | 16.6813 | 16.6348 | 16.7427 | 17.4252 | 16.9212 | 16.8176 | 16.8764 | 17.0177 | 7 | 7 | 7 | 7 | 7 | 6 | 8 | 7 |
| AMPD3 | Q01432;Q0 | 0.000272 | 6.06E-05 | -0.325261 | | 1.26865 | -2.39212 | | 14.0879 | 14.4949 | 14.0736 | 14.7514 | 14.619 | 14.4541 | 14.352 | 14.5439 | 3 | 3 | 3 | 4 | 3 | 3 | 3 | 4 |
| ARF4 | P18085 | 0.000267 | 5.97E-05 | -0.32518 | | 1.18849 | -2.25727 | | 21.3451 | 21.401 | 21.4618 | 21.8082 | 21.3508 | 21.8575 | 21.9651 | 21.6576 | 6 | 6 | 6 | 6 | 6 | 6 | 6 | 6 |
| PSME3IP1 | Q9GZU8 | 8.65E-05 | 2.06E-05 | -0.324981 | | 0.9811 | -1.91179 | | 15.3165 | 15.2239 | 15.476 | 15.699 | 15.8327 | 15.2125 | 15.6678 | 15.9068 | 6 | 7 | 7 | 8 | 5 | 6 | 5 | 7 |
| LARP4B | Q92615 | 3.70E-11 | 1.92E-11 | -0.324291 | + | 1.74353 | -3.22385 | | 15.8527 | 15.8935 | 15.8543 | 16.298 | 16.3502 | 15.9181 | 16.1677 | 16.2216 | 14 | 11 | 12 | 13 | 12 | 11 | 13 | 13 |
| SYNRG | Q9UMZ2;Q | 1.00E-06 | 2.91E-07 | -0.324272 | + | 1.44661 | -2.69614 | | 14.2664 | 14.3223 | 14.2291 | 14.8336 | 14.7326 | 14.5248 | 14.3171 | 14.5763 | 6 | 8 | 10 | 9 | 6 | 7 | 6 | 6 |
| APRT | P07741 | 1.40E-11 | 7.91E-12 | -0.324262 | + | 1.91665 | -3.54702 | | 18.5599 | 18.3736 | 18.4977 | 18.6957 | 18.9165 | 18.6665 | 18.7508 | 18.9771 | 11 | 11 | 13 | 11 | 11 | 11 | 12 | 10 |
| NDFIP1 | Q9BT67 | 1.28E-12 | 9.04E-13 | -0.323499 | | 0.718471 | -1.47285 | | 15.7289 | 15.7384 | 15.668 | 16.2759 | 16.0586 | 15.4019 | 16.1423 | 16.2976 | 5 | 5 | 5 | 5 | 5 | 4 | 6 | 5 |
| SPATS2L | Q9NUQ6 | 4.63E-11 | 2.36E-11 | -0.323083 | + | 2.68909 | -5.18418 | | 16.0357 | 16.0544 | 16.0223 | 16.5018 | 16.264 | 16.2519 | 16.389 | 16.3961 | 23 | 22 | 23 | 23 | 20 | 23 | 24 | 22 |
| SLC35F6 | Q8N357 | 0.000109 | 2.57E-05 | -0.322969 | | 0.931056 | -1.82867 | | 16.6886 | 16.4737 | 16.3713 | 16.5455 | 16.7734 | 16.8484 | 17.2802 | 16.7233 | 3 | 3 | 3 | 3 | 3 | 3 | 3 | 3 |
| SNX9 | Q9Y5X1 | 1.41E-23 | 3.17E-22 | -0.322736 | | 0.633112 | -1.32707 | | 17.968 | 17.9816 | 17.99 | 18.7116 | 17.6984 | 18.642 | 18.291 | 18.1702 | 19 | 21 | 19 | 19 | 17 | 20 | 21 | 18 |
| GOLM2 | Q6P4E1;Q6 | 2.78E-15 | 4.19E-15 | -0.322555 | + | 1.65921 | -3.07087 | | 15.1485 | 15.2181 | 15.2301 | 15.3469 | 15.5137 | 15.6642 | 15.7264 | 15.3561 | 10 | 12 | 12 | 12 | 8 | 9 | 12 | 10 |
| DNAJB11 | Q9UBS4 | 1.18E-10 | 5.61E-11 | -0.322256 | + | 1.33644 | -2.50707 | | 17.1541 | 17.1545 | 17.2161 | 17.2641 | 17.5538 | 17.4517 | 17.8307 | 17.3855 | 10 | 10 | 10 | 10 | 10 | 10 | 11 | 9 |
| PPP1CC | P36873;P3 | 0.00203 | 0.000398 | -0.321592 | | 1.24801 | -2.3573 | | 18.9023 | 18.7983 | 18.7538 | 19.2368 | 19.3983 | 18.8356 | 18.9963 | 19.2314 | 2 | 2 | 2 | 2 | 2 | 2 | 3 | 2 |
| ZDHHC5 | Q9C0B5;Q9 | 4.85E-12 | 2.98E-12 | -0.321503 | | 1.25011 | -2.36084 | | 15.059 | 14.9587 | 15.0216 | 15.4207 | 14.9601 | 15.4914 | 15.5066 | 15.2941 | 5 | 4 | 4 | 4 | 4 | 5 | 4 | 3 |
| MAP3K20 | Q9NYL2-2 | 1.61E-17 | 4.77E-17 | -0.321182 | | 0.41787 | -0.94306 | | 16.1259 | 16.1895 | 16.1392 | 17.2762 | 16.4983 | 16.0754 | 15.8066 | 16.7071 | 12 | 10 | 12 | 13 | 10 | 11 | 11 | 11 |
| ADGRL2 | O95490;O9 | 0.000476 | 0.000103 | -0.321148 | | 0.43318 | -0.97149 | | 14.1567 | 14.5492 | 15.1014 | 15.4558 | 15.3078 | 14.6033 | 14.8097 | 14.4414 | 2 | 1 | 1 | 2 | 1 | 1 | 3 | 1 |
| MACF1 | Q9UPN3-2 | 2.18E-18 | 8.65E-18 | -0.320876 | + | 1.90332 | -3.52166 | | 15.9475 | 15.9527 | 15.9509 | 16.5377 | 16.1863 | 16.1762 | 16.2625 | 16.1935 | 172 | 180 | 182 | 191 | 150 | 173 | 176 | 171 |
| MICAL3 | Q7RTP6 | 9.85E-07 | 2.86E-07 | -0.320858 | + | 1.41105 | -2.63478 | | 14.823 | 14.7856 | 14.8311 | 15.0235 | 15.4867 | 14.9967 | 15.0332 | 15.1303 | 15 | 17 | 18 | 18 | 11 | 17 | 16 | 16 |
| RAB28 | P51157;P5 | 0.005293 | 0.000942 | -0.320729 | | 0.557327 | -1.21925 | | 13.4745 | 13.8829 | 13.8875 | 13.8942 | 13.7271 | 14.0113 | 14.6434 | NaN | 3 | 3 | 3 | 1 | 2 | 3 | 2 | NaN |
| ZFYVE16 | Q7Z3T8 | 3.14E-10 | 1.39E-10 | -0.32056 | + | 2.13862 | -3.98191 | | 14.8596 | 14.8298 | 14.8134 | 15.1465 | 15.0662 | 15.0266 | 15.3725 | 15.1624 | 9 | 9 | 12 | 10 | 8 | 8 | 10 | 10 |
| SOD2 | P04179 | 1.87E-07 | 5.89E-08 | -0.320547 | | 0.651868 | -1.35932 | | 18.4204 | 18.3685 | 18.5173 | 18.5335 | 18.47 | 19.223 | 18.4133 | 19.1399 | 12 | 12 | 11 | 11 | 10 | 12 | 11 | 11 |

| Gene names | UniProt IDs | Protein Group P-value | Protein Group Q-value | Log2 Difference NUP214 vs Controls (n=5) | p<0.05 | -log10 p-value | T-test Test statistic | NPC Localization | Log2 MS2_quantity_NUP214_1 | Log2 MS2_quantity_NUP214_2 | Log2 MS2_quantity_NUP214_3 | Log2 MS2_quantity_Control_1 | Log2 MS2_quantity_Control_2 | Log2 MS2_quantity_Control_3 | Log2 MS2_quantity_Control_4 | Log2 MS2_quantity_Control_5 | Number Peptides for Quantitation NUP214_1 | Number Peptides for Quantitation NUP214_2 | Number Peptides for Quantitation NUP214_3 | Number Peptides for Quantitation Control_1 | Number Peptides for Quantitation Control_2 | Number Peptides for Quantitation Control_3 | Number Peptides for Quantitation Control_4 | Number Peptides for Quantitation Control_5 |
|---|---|---|---|---|---|---|---|---|---|---|---|---|---|---|---|---|---|---|---|---|---|---|---|---|
| HSPB11 | Q9Y547 | 8.50E-09 | 3.12E-09 | -0.319807 | + | 3.4413 | -7.2048 | | 15.8624 | 15.9587 | 15.906 | 16.2452 | 16.3008 | 16.1207 | 16.2293 | 16.2482 | 4 | 5 | 4 | 4 | 3 | 4 | 4 | 3 |
| FILIP1L | Q4L180;Q4 | 2.83E-05 | 7.10E-06 | -0.319784 | | 0.738418 | -1.50658 | | 14.7556 | 14.643 | 14.5796 | 15.5751 | 14.9323 | 14.6575 | 14.8194 | 14.9116 | 14 | 15 | 14 | 17 | 13 | 12 | 16 | 14 |
| FUT4 | P22083 | 0.002751 | 0.000519 | -0.319461 | | 0.489824 | -1.12448 | | 13.3189 | 13.391 | NaN | 13.9729 | NaN | 13.6577 | 13.9201 | 13.147 | 2 | 2 | NaN | 2 | NaN | 1 | 2 | 1 |
| RTN2 | O75298;O7 | 0.009804 | 0.001684 | -0.319401 | | 0.930241 | -1.82731 | | 13.8725 | 14.2329 | 14.1786 | 14.5305 | 14.3603 | 14.0557 | 14.7614 | 14.3624 | 2 | 2 | 1 | 4 | 1 | 1 | 2 | 2 |
| NHLRC3 | Q5JS37 | 7.53E-07 | 2.22E-07 | -0.319351 | | 0.843591 | -1.68305 | | 14.9662 | 14.9612 | 14.9511 | 14.9019 | 15.2353 | 15.4447 | 15.7232 | 15.0892 | 3 | 4 | 4 | 3 | 4 | 3 | 4 | 5 |
| WASF1 | Q92558 | 0.001225 | 0.00025 | -0.319338 | + | 1.34764 | -2.52617 | | 14.6193 | 14.4609 | 14.3905 | 14.5377 | 15.0312 | 14.8209 | 14.71 | 14.9482 | 5 | 4 | 4 | 5 | 3 | 4 | 3 | 4 |
| TGFBR3 | Q03167;Q0 | 0.001159 | 0.000237 | -0.319232 | | 0.228185 | -0.57322 | | 14.3071 | 14.0909 | 14.1512 | 15.066 | 14.0419 | 13.4263 | NaN | 15.475 | 2 | 2 | 2 | 2 | 2 | 1 | NaN | 2 |
| PTDSS2 | Q9BVG9 | 0.000237 | 5.34E-05 | -0.318235 | + | 1.84257 | -3.40714 | | 16.1147 | 16.2046 | 15.8831 | 16.4724 | 16.4773 | 16.2213 | 16.385 | 16.3725 | 3 | 3 | 3 | 3 | 2 | 3 | 5 | 3 |
| NUFIP2 | Q7Z417 | 9.91E-11 | 4.75E-11 | -0.318213 | + | 1.76723 | -3.26734 | | 15.6773 | 15.4752 | 15.6314 | 15.8966 | 15.6993 | 15.9831 | 16.0944 | 15.8909 | 15 | 14 | 15 | 14 | 12 | 13 | 15 | 14 |
| LURAP1L | Q8IV03 | 0.005585 | 0.000989 | -0.31796 | | 0.187548 | -0.47838 | | 14.4092 | 15.305 | 14.1717 | 13.8274 | 14.7757 | 16.4524 | 15.423 | 14.2543 | 1 | 1 | 3 | 2 | 1 | 3 | 3 | 2 |
| RILPL1 | Q5EBL4 | 2.20E-08 | 7.65E-09 | -0.317748 | + | 1.33575 | -2.50591 | | 15.4678 | 15.4809 | 15.5047 | 15.8611 | 15.5545 | 15.8473 | 16.101 | 15.6471 | 13 | 14 | 13 | 14 | 10 | 14 | 14 | 13 |
| SHMT2 | P34897;P3 | 3.60E-09 | 1.38E-09 | -0.317728 | | 1.03649 | -2.00379 | | 18.4346 | 18.4563 | 18.3626 | 18.5729 | 18.5504 | 19.1934 | 18.7088 | 18.6522 | 25 | 25 | 26 | 25 | 27 | 26 | 26 | 26 |
| EIF3F | O00303 | 1.61E-09 | 6.47E-10 | -0.317554 | + | 1.59427 | -2.95485 | | 18.2733 | 18.0748 | 18.2388 | 18.4877 | 18.7135 | 18.2917 | 18.4456 | 18.6274 | 14 | 15 | 15 | 15 | 15 | 15 | 15 | 15 |
| OCRL | Q01968;Q0 | 6.33E-11 | 3.15E-11 | -0.317384 | + | 4.10387 | -9.47458 | | 15.1812 | 15.0903 | 15.1308 | 15.4577 | 15.3854 | 15.4684 | 15.5107 | 15.4351 | 14 | 17 | 18 | 16 | 14 | 16 | 15 | 16 |
| MIB1 | Q86YT6 | 0.000173 | 3.96E-05 | -0.317174 | + | 2.72149 | -5.26134 | | 13.9032 | 13.7344 | 13.7745 | 14.0126 | 14.237 | 14.1265 | 14.1168 | 14.1131 | 5 | 5 | 4 | 7 | 5 | 3 | 6 | 7 |
| RAB43 | Q86YS6 | 1.98E-08 | 6.94E-09 | -0.317142 | | 0.865746 | -1.72 | | 15.5542 | 15.5845 | 15.5117 | 16.0463 | 16.06 | 15.3436 | 15.8319 | 16.0545 | 5 | 6 | 7 | 7 | 6 | 5 | 7 | 6 |
| FITM2 | Q8N6M3 | 0.001094 | 0.000224 | -0.316935 | + | 1.31585 | -2.47205 | | 16.1933 | 16.2669 | 16.3346 | 16.4013 | 16.5785 | 16.3904 | 16.9021 | 16.6372 | 4 | 4 | 4 | 3 | 3 | 4 | 4 | 3 |
| RNGTT | O60942;O6 | 0.000212 | 4.80E-05 | -0.316863 | + | 1.49972 | -2.78843 | | 14.4457 | 14.3621 | 14.241 | 14.6159 | 14.5494 | 14.6071 | 14.9781 | 14.5818 | 4 | 6 | 5 | 6 | 2 | 4 | 4 | 4 |
| SPIRE1 | Q08AE8;Q0 | 0.00497 | 0.000889 | -0.316686 | | 0.946 | -1.8535 | | 14.1113 | 14.3226 | 13.971 | 14.5667 | 14.6158 | 14.1683 | 14.7232 | 14.1841 | 3 | 3 | 3 | 3 | 3 | 3 | 2 | 3 |
| RBMS2 | Q15434 | 0.000594 | 0.000126 | -0.316674 | | 0.955879 | -1.86991 | | 16.8143 | 16.7235 | 16.8145 | 16.8037 | 17.3885 | 16.8028 | 17.3254 | 17.1834 | 3 | 3 | 2 | 3 | 3 | 3 | 3 | 3 |
| RAB23 | Q9ULC3 | 2.72E-20 | 2.14E-19 | -0.316418 | + | 2.68125 | -5.16561 | | 16.0171 | 16.0269 | 16.0565 | 16.2178 | 16.4772 | 16.4143 | 16.3501 | 16.2901 | 12 | 12 | 12 | 12 | 11 | 12 | 12 | 12 |
| NAGK | Q9UJ70;Q9 | 6.19E-19 | 2.89E-18 | -0.316252 | + | 3.42468 | -7.1544 | | 18.7722 | 18.7303 | 18.702 | 18.963 | 19.0453 | 19.1107 | 19.1297 | 19.0067 | 19 | 18 | 19 | 18 | 18 | 18 | 18 | 18 |
| GIPC1 | O14908 | 1.20E-17 | 3.71E-17 | -0.316054 | | 1.13347 | -2.16525 | | 16.7251 | 16.7706 | 16.7967 | 17.4431 | 16.7872 | 16.9896 | 17.0159 | 17.165 | 16 | 15 | 15 | 16 | 13 | 14 | 14 | 15 |
| HM13 | Q8TCT9;Q8 | 3.75E-05 | 9.31E-06 | -0.315146 | + | 2.57656 | -4.9221 | | 18.9699 | 18.8698 | 18.9214 | 19.2176 | 19.2014 | 19.0945 | 19.3082 | 19.3558 | 10 | 10 | 10 | 10 | 11 | 10 | 11 | 10 |
| ACOT8 | O14734 | 0.000792 | 0.000166 | -0.315037 | + | 2.32955 | -4.37722 | | 16.7267 | 16.7957 | 16.689 | 16.8941 | 17.1602 | 17.1431 | 17.0894 | 16.974 | 7 | 7 | 6 | 6 | 6 | 6 | 7 | 6 |
| TRPM4 | Q8TD43;Q8 | 9.53E-11 | 4.58E-11 | -0.314967 | | 1.25796 | -2.37407 | | 14.8626 | 14.8186 | 14.7986 | 15.2179 | 15.2386 | 14.9295 | 14.9012 | 15.4206 | 9 | 9 | 11 | 11 | 8 | 10 | 8 | 11 |
| CAPRIN1 | Q14444;Q1 | 4.57E-14 | 4.79E-14 | -0.314531 | | 1.1888 | -2.25779 | | 17.7475 | 17.7096 | 17.7663 | 18.3372 | 18.0271 | 17.7305 | 17.9729 | 18.2106 | 16 | 13 | 13 | 13 | 14 | 14 | 14 | 15 |
| ITGA1 | P56199 | 3.93E-11 | 2.03E-11 | -0.314337 | | 0.253854 | -0.62111 | | 15.5195 | 15.7135 | 15.6415 | 14.7685 | 15.8034 | 16.4436 | 17.01 | 15.6705 | 18 | 19 | 17 | 15 | 15 | 19 | 20 | 17 |
| SPATA20 | Q8TB22;Q8 | 0.001597 | 0.00032 | -0.314331 | | 0.74366 | -1.55596 | | 14.4198 | 14.0818 | 13.9262 | 14.5309 | 14.0894 | NaN | 14.7431 | 14.4644 | 7 | 7 | 8 | 10 | 4 | NaN | 7 | 6 |
| INPPL1 | O15357 | 2.81E-12 | 1.81E-12 | -0.314266 | + | 3.23592 | -6.60171 | | 15.4395 | 15.4071 | 15.4212 | 15.6467 | 15.7959 | 15.7149 | 15.8391 | 15.6877 | 29 | 26 | 25 | 26 | 26 | 22 | 27 | 27 |
| SRGAP1 | Q7Z6B7 | 1.90E-18 | 7.66E-18 | -0.313568 | | 0.795345 | -1.60235 | | 15.5826 | 15.5658 | 15.6247 | 16.3139 | 15.93 | 15.8971 | 15.3999 | 15.9822 | 32 | 33 | 33 | 35 | 31 | 33 | 30 | 32 |
| YPEL5 | P62699 | 1.91E-12 | 1.29E-12 | -0.313448 | + | 1.4724 | -2.74084 | | 15.7574 | 15.7269 | 15.6851 | 15.881 | 16.2921 | 15.8204 | 16.1225 | 16.0668 | 5 | 5 | 5 | 5 | 5 | 4 | 4 | 5 |
| ITIH3 | Q06033;Q0 | 3.34E-09 | 1.29E-09 | -0.313358 | | 0.232859 | -0.57691 | | 18.9167 | 19.0733 | 19.0064 | 20.2453 | 18.3479 | 18.3854 | 19.4842 | 20.0981 | 7 | 7 | 7 | 7 | 5 | 6 | 7 | 7 |
| FKBP14 | Q9NWM8 | 3.29E-13 | 2.72E-13 | -0.313289 | + | 1.62453 | -3.00873 | | 16.3936 | 16.3304 | 16.3133 | 16.4769 | 16.5639 | 16.7761 | 16.8967 | 16.5817 | 6 | 6 | 6 | 6 | 6 | 6 | 6 | 6 |
| HAT1 | O14929 | 1.81E-12 | 1.23E-12 | -0.313105 | | 0.742248 | -1.51305 | | 15.8037 | 15.8787 | 15.824 | 16.5019 | 16.0462 | 15.6019 | 16.2755 | 16.3174 | 5 | 5 | 6 | 6 | 5 | 6 | 6 | 6 |
| ORC2 | Q13416 | 0.005767 | 0.001019 | -0.313008 | | 0.730574 | -1.49333 | | 13.7046 | 13.3238 | 13.3501 | 13.6296 | 14.2903 | 13.4471 | 13.6837 | 13.8119 | 2 | 3 | 3 | 5 | 3 | 1 | 3 | 1 |
| PSEN1 | P49768;P4 | 4.29E-08 | 1.44E-08 | -0.312869 | | 1.15312 | -2.19807 | | 15.9481 | 15.9238 | 15.9358 | 15.9353 | 16.3804 | 16.0634 | 16.5039 | 16.3609 | 3 | 3 | 3 | 4 | 4 | 3 | 3 | 4 |
| FABP3 | P05413 | 1.82E-14 | 2.14E-14 | -0.312736 | | 0.279074 | -0.67313 | | 17.3314 | 17.3629 | 17.2384 | 16.9012 | 16.9252 | 17.4612 | 18.6706 | 18.16 | 10 | 9 | 9 | 8 | 8 | 9 | 11 | 9 |
| POLE3 | Q9NRF9 | 1.02E-10 | 4.88E-11 | -0.311888 | | 0.956284 | -1.87058 | | 16.3983 | 16.4131 | 16.3955 | 16.9059 | 16.6936 | 16.2397 | 16.9118 | 16.82 | 3 | 3 | 3 | 3 | 2 | 4 | 3 | 3 |
| FCSK | Q8N0W3;Q | 0.00106 | 0.000218 | -0.311562 | | 0.160263 | -0.42692 | | 13.7938 | 14.572 | 14.3152 | NaN | 15.8143 | 13.4318 | NaN | 14.3695 | 4 | 5 | 5 | NaN | 2 | 2 | NaN | 1 |
| ARFIP2 | P53365;P5 | 3.72E-12 | 2.34E-12 | -0.311451 | + | 1.78888 | -3.30727 | | 15.4363 | 15.4415 | 15.4976 | 15.5262 | 15.7738 | 15.8061 | 15.9612 | 15.7823 | 5 | 6 | 6 | 6 | 4 | 6 | 7 | 6 |
| TSPO | P30536 | 0.000882 | 0.000183 | -0.311344 | + | 1.76238 | -3.25843 | | 18.919 | 19.0192 | 19.1937 | 19.3117 | 19.218 | 19.4017 | 19.5492 | 19.2959 | 2 | 2 | 2 | 2 | 2 | 2 | 2 | 2 |
| MAGED2 | Q9UNF1 | 9.33E-07 | 2.72E-07 | -0.311231 | + | 2.46242 | -4.66534 | | 17.3105 | 17.3158 | 17.2615 | 17.5649 | 17.4385 | 17.6389 | 17.7188 | 17.6748 | 20 | 20 | 20 | 20 | 20 | 20 | 21 | 21 |
| TPM2 | P07951-2 | 2.31E-13 | 1.99E-13 | -0.311113 | | 1.00874 | -1.9577 | | 18.2739 | 18.3854 | 18.3292 | 18.3106 | 18.57 | 18.9406 | 18.5052 | 18.8767 | 20 | 21 | 21 | 20 | 20 | 20 | 20 | 22 |
| NOMO2 | Q5JPE7;Q5 | 1.96E-14 | 2.27E-14 | -0.311089 | + | 3.35669 | -6.95121 | | 17.4004 | 17.481 | 17.4382 | 17.7513 | 17.7473 | 17.6514 | 17.8474 | 17.7575 | 34 | 37 | 36 | 36 | 37 | 37 | 37 | 35 |
| RAB4B | P61018;P6 | 1.35E-10 | 6.37E-11 | -0.31108 | + | 2.74856 | -5.32643 | | 16.3154 | 16.2559 | 16.3394 | 16.7764 | 16.5594 | 16.5471 | 16.5874 | 16.603 | 4 | 5 | 4 | 6 | 4 | 5 | 4 | 5 |
| ATP6V1D | Q9Y5K8 | 2.71E-09 | 1.06E-09 | -0.311004 | + | 1.82576 | -3.37573 | | 16.5024 | 16.4787 | 16.3978 | 16.6934 | 16.6287 | 16.7421 | 17.0207 | 16.7683 | 11 | 13 | 12 | 13 | 9 | 12 | 12 | 11 |
| NTAQ1 | Q96HA8;Q9 | 0.011285 | 0.001924 | -0.310945 | | 0.799364 | -1.65552 | | 14.7113 | 15.008 | 14.8934 | 15.1163 | 15.3912 | 15.4255 | 14.7944 | NaN | 1 | 2 | 2 | 1 | 1 | 1 | 1 | NaN |
| POLR1E | Q9GZS1;Q9 | 0.002256 | 0.000437 | -0.310705 | | 0.811451 | -1.67711 | | 14.8452 | 14.7692 | 14.6163 | 14.6849 | NaN | 14.9926 | 15.1397 | 15.3999 | 2 | 5 | 5 | 4 | NaN | 2 | 3 | 2 |
| LARP6 | Q9BRS8 | 7.18E-16 | 1.28E-15 | -0.310541 | | 1.17172 | -2.22918 | | 14.9854 | 14.929 | 15.0031 | 15.2909 | 15.2619 | 15.6432 | 15.2211 | 14.998 | 12 | 13 | 11 | 12 | 9 | 11 | 10 | 9 |
| ERLIN2 | O94905 | 6.06E-11 | 3.03E-11 | -0.310442 | + | 4.59316 | -11.5352 | | 17.9205 | 17.8899 | 17.885 | 18.2341 | 18.1819 | 18.1793 | 18.273 | 18.1762 | 21 | 21 | 22 | 21 | 21 | 22 | 20 | 22 |
| SPARC | P09486 | 1.71E-13 | 1.53E-13 | -0.310438 | | 0.23038 | -0.57163 | | 19.1425 | 19.082 | 19.2584 | 18.5539 | 19.1032 | 20.355 | 20.5278 | 18.8173 | 11 | 12 | 12 | 11 | 10 | 12 | 12 | 11 |
| EEF1B2 | P24534 | 1.03E-12 | 7.42E-13 | -0.310429 | + | 1.69072 | -3.12772 | | 19.5681 | 19.3092 | 19.6123 | 19.6264 | 19.7973 | 19.9603 | 19.8116 | 19.8391 | 11 | 11 | 11 | 11 | 11 | 11 | 11 | 11 |
| RIN3 | Q8TB24 | 0.000325 | 7.18E-05 | -0.310038 | | 0.663767 | -1.37972 | | 13.9902 | 14.4051 | 14.4836 | 14.9271 | 14.5535 | 14.7497 | 14.0685 | 14.7162 | 5 | 4 | 5 | 6 | 3 | 6 | 5 | 5 |
| EXT2 | Q93063;Q9 | 4.69E-15 | 6.57E-15 | -0.309736 | | 0.789839 | -1.59312 | | 15.4649 | 15.488 | 15.417 | 15.701 | 16.0228 | 15.2417 | 16.0371 | 15.8293 | 12 | 10 | 10 | 11 | 11 | 10 | 12 | 10 |
| NAA30 | Q147X3 | 2.31E-05 | 5.85E-06 | -0.309469 | + | 1.50341 | -2.79487 | | 16.0543 | 15.9248 | 15.9794 | 16.1564 | 16.5137 | 16.136 | 16.466 | 16.2061 | 6 | 6 | 7 | 7 | 4 | 6 | 4 | 6 |
| AP3M2 | P53677 | 1.05E-17 | 3.34E-17 | -0.309047 | + | 2.45865 | -4.65702 | | 16.317 | 16.2817 | 16.1639 | 16.5866 | 16.5569 | 16.6392 | 16.4027 | 16.6308 | 10 | 10 | 10 | 11 | 10 | 10 | 11 | 10 |
| LPCAT4 | Q643R3 | 0.00133 | 0.00027 | -0.308552 | | 0.773522 | -1.56572 | | 16.7396 | 16.6461 | 16.6584 | 17.2558 | 17.4069 | 16.7344 | 16.6525 | 16.9 | 2 | 2 | 2 | 2 | 2 | 2 | 2 | 2 |
| WWP2 | O00308 | 0.006087 | 0.001073 | -0.308358 | | 1.02485 | -1.98445 | | 15.7654 | 15.7861 | 15.7543 | 16.2992 | 15.9767 | 15.9843 | 15.7441 | 16.3807 | 6 | 5 | 6 | 6 | 4 | 2 | 3 | 3 |
| DFFA | O00273 | 5.06E-09 | 1.91E-09 | -0.308285 | | 1.12811 | -2.15632 | | 16.3122 | 16.3068 | 16.3204 | 16.8846 | 16.6621 | 16.2738 | 16.507 | 16.7796 | 9 | 9 | 9 | 10 | 9 | 9 | 9 | 10 |
| DROSHA | Q9NRR4;Q9 | 0.027899 | 0.004595 | -0.308258 | | 0.936821 | -1.83825 | | 14.2735 | 14.2852 | 14.8984 | 14.7828 | 15.0084 | 14.7018 | 14.7364 | 14.7402 | 2 | 1 | 1 | 1 | 2 | 1 | 2 | 1 |
| LMAN2L | Q9H0V9;Q9 | 6.53E-11 | 3.24E-11 | -0.30823 | | 1.21418 | -2.30037 | | 16.3853 | 16.5621 | 16.5094 | 16.7531 | 16.9019 | 16.4751 | 17.0592 | 16.7798 | 10 | 10 | 10 | 11 | 9 | 10 | 9 | 9 |
| EOGT | Q5NDL2 | 7.46E-12 | 4.45E-12 | -0.308118 | | 0.638495 | -1.33634 | | 15.6272 | 15.5367 | 15.6832 | 16.5448 | 15.9722 | 15.528 | 15.7366 | 15.8377 | 8 | 8 | 7 | 10 | 6 | 6 | 6 | 9 |
| SUMO2 | P61956;P6 | 1.02E-09 | 4.24E-10 | -0.307949 | + | 2.6361 | -5.05964 | | 15.7378 | 15.5839 | 15.7216 | 16.0576 | 16.0137 | 15.8461 | 16 | 16.028 | 2 | 2 | 2 | 2 | 2 | 2 | 2 | 2 |
| PLEC | Q15149-4 | 0.000103 | 2.43E-05 | -0.307485 | | 0.314605 | -0.74466 | | 19.035 | 19.0232 | 18.9983 | 18.7618 | 19.6406 | 19.5669 | 18.482 | 20.1803 | 2 | 2 | 2 | 2 | 2 | 2 | 2 | 2 |
| BTN2A1 | Q7KYR7;Q7 | 3.68E-07 | 1.13E-07 | -0.30747 | | 1.10388 | -2.11591 | | 14.4653 | 14.6308 | 14.6057 | 14.6546 | 14.6796 | 14.8591 | 15.2351 | 14.9452 | 7 | 7 | 6 | 7 | 5 | 5 | 8 | 6 |
| WLS | Q5T9L3;Q5 | 1.31E-09 | 5.36E-10 | -0.307373 | | 0.281729 | -0.67854 | | 15.1642 | 15.2104 | 15.2955 | 16.7597 | 14.7633 | 15.0915 | 15.5789 | 15.4604 | 8 | 8 | 7 | 10 | 6 | 8 | 8 | 7 |
| ADSS2 | P30520 | 5.30E-13 | 4.11E-13 | -0.307228 | + | 1.63106 | -3.02041 | | 17.7103 | 17.7781 | 17.7315 | 17.9427 | 18.2803 | 17.9273 | 18.1738 | 17.9119 | 20 | 19 | 20 | 19 | 20 | 20 | 21 | 19 |
| LMOD1 | P29536;P2 | 3.13E-11 | 1.65E-11 | -0.307086 | | 0.769573 | -1.55908 | | 16.4077 | 16.4204 | 16.3687 | 16.4405 | 16.8318 | 16.8943 | 16.2868 | 17.0768 | 14 | 14 | 16 | 15 | 13 | 15 | 15 | 15 |
| KCTD10 | Q9H3F6 | 5.47E-10 | 2.34E-10 | -0.306991 | | 1.05111 | -2.02809 | | 15.1248 | 15.0921 | 15.0356 | 15.6819 | 15.498 | 15.1172 | 15.1356 | 15.523 | 11 | 9 | 12 | 12 | 9 | 10 | 11 | 11 |
| PRUNE1 | Q86TP1 | 0.001796 | 0.000356 | -0.306987 | | 0.457288 | -1.01583 | | 14.2595 | 13.9712 | 14.1628 | 14.7344 | 13.69 | 14.721 | 14.8709 | 14.1743 | 3 | 3 | 3 | 4 | 1 | 5 | 3 | 3 |
| SLFN11 | Q7Z7L1 | 1.05E-06 | 3.03E-07 | -0.30695 | | 0.474566 | -1.04731 | | 14.8605 | 14.9333 | 14.5621 | 15.19 | 15.8479 | 14.8301 | 14.6165 | 14.9767 | 6 | 8 | 8 | 6 | 1 | 4 | 2 | 5 |
| UBE2V1 | Q13404;Q1 | 0.000267 | 5.97E-05 | -0.306816 | + | 3.5552 | -7.55819 | | 19.9176 | 19.9232 | 20.0262 | 20.2859 | 20.3015 | 20.1984 | 20.3128 | 20.2139 | 6 | 6 | 6 | 6 | 6 | 6 | 6 | 6 |
| HGSNAT | Q68CP4;Q6 | 1.85E-11 | 1.02E-11 | -0.306404 | | 1.2328 | -2.33167 | | 15.675 | 15.6021 | 15.9148 | 16.0218 | 15.7257 | 16.0883 | 16.2137 | 16.1357 | 5 | 4 | 5 | 2 | 2 | 5 | 5 | 2 |
| GNAI1 | P63096;P6 | 1.77E-07 | 5.58E-08 | -0.306043 | | 0.728273 | -1.48944 | | 16.2623 | 16.0998 | 16.0204 | 16.4955 | 16.0997 | 16.4405 | 16.9495 | 16.1826 | 5 | 5 | 5 | 5 | 5 | 5 | 5 | 5 |
| RAB32 | Q13637 | 1.78E-12 | 1.21E-12 | -0.30586 | | 1.04391 | -2.01613 | | 17.6808 | 17.6971 | 17.7777 | 18.1331 | 18.3186 | 17.636 | 17.9716 | 18.0626 | 15 | 15 | 14 | 13 | 15 | 14 | 13 | 13 |
| HSPH1 | Q92598;Q9 | 3.86E-16 | 7.36E-16 | -0.305846 | | 1.229 | -2.32529 | | 18.3473 | 18.3704 | 18.3479 | 18.7778 | 18.6204 | 18.2983 | 18.8607 | 18.7481 | 43 | 42 | 42 | 42 | 42 | 43 | 41 | 41 |
| GATAD2A | Q86YP4;Q8 | 0.000124 | 2.89E-05 | -0.305591 | | 0.649961 | -1.35605 | | 14.3804 | 14.4366 | 14.3585 | 15.1176 | 14.8053 | 14.1791 | 14.4567 | 14.9284 | 8 | 9 | 8 | 11 | 5 | 7 | 6 | 9 |

| Gene names | UniProt IDs | Protein Group P-value | Protein Group Q-value | Log2 Difference NUP214 vs Controls (n=5) | p<0.05 | -log10 p-value | T-test Test statistic | NPC Localization | Log2 MS2_quantity_NUP214_1 | Log2 MS2_quantity_NUP214_2 | Log2 MS2_quantity_NUP214_3 | Log2 MS2_quantity_Control_1 | Log2 MS2_quantity_Control_2 | Log2 MS2_quantity_Control_3 | Log2 MS2_quantity_Control_4 | Log2 MS2_quantity_Control_5 | Number Peptides for Quantitation NUP214_1 | Number Peptides for Quantitation NUP214_2 | Number Peptides for Quantitation NUP214_3 | Number Peptides for Quantitation Control_1 | Number Peptides for Quantitation Control_2 | Number Peptides for Quantitation Control_3 | Number Peptides for Quantitation Control_4 | Number Peptides for Quantitation Control_5 |
|---|---|---|---|---|---|---|---|---|---|---|---|---|---|---|---|---|---|---|---|---|---|---|---|---|
| SLC25A46 | Q96AG3 | 0.000251 | 5.63E-05 | -0.304032 | + | 3.09171 | -6.20273 | | 15.3023 | 15.2095 | 15.3418 | 15.5145 | 15.6923 | 15.5744 | 15.6058 | 15.5559 | 5 | 7 | 5 | 4 | 2 | 5 | 5 | 5 |
| PKD2 | Q13563;Q1 | 2.37E-14 | 2.69E-14 | -0.303743 | + | 1.60838 | -2.97993 | | 15.4133 | 15.3646 | 15.4333 | 15.7159 | 15.7243 | 15.6398 | 15.9617 | 15.4957 | 10 | 12 | 14 | 14 | 13 | 14 | 15 | 14 |
| SLC25A17 | O43808 | 0.002724 | 0.000515 | -0.303638 | | 0.587051 | -1.24722 | | 13.068 | 12.8984 | 13.6407 | 13.6269 | 13.2408 | 13.86 | 13.1443 | 13.658 | 3 | 2 | 3 | 3 | 2 | 2 | 2 | 2 |
| TNFRSF1A | P19438 | 7.28E-05 | 1.75E-05 | -0.303427 | | 0.527676 | -1.14269 | | 14.9982 | 14.8762 | 15.2856 | 16.0619 | 15.2166 | 15.0235 | 15.0827 | 15.3993 | 3 | 2 | 3 | 3 | 4 | 3 | 3 | 3 |
| CIP2A | Q8TCG1 | 3.02E-05 | 7.56E-06 | -0.303324 | | 0.590562 | -1.25334 | | 14.6644 | 14.6522 | 14.5684 | 15.2466 | 14.9969 | 14.2419 | 14.9717 | 15.2013 | 12 | 16 | 13 | 15 | 6 | 7 | 9 | 14 |
| YJU2 | Q9BW85 | 0.000681 | 0.000144 | -0.303096 | | 0.384342 | -0.87998 | | 13.8004 | 14.6511 | 14.335 | 14.8588 | 14.4698 | 14.1547 | 15.254 | 14.0892 | 3 | 3 | 3 | 3 | 1 | 2 | 2 | 3 |
| CCDC9B | Q6ZUT6;Q6 | 1.42E-06 | 4.04E-07 | -0.302807 | | 0.458876 | -1.01873 | | 14.1236 | 13.937 | 14.1712 | 13.6783 | 14.3942 | 14.1553 | 14.8805 | 14.7921 | 4 | 4 | 4 | 5 | 3 | 4 | 5 | 5 |
| UBE2A | P49459 | 2.26E-07 | 7.09E-08 | -0.302337 | | 0.565121 | -1.20885 | | 16.6525 | 16.8458 | 16.9507 | 17.495 | 17.1873 | 17.0438 | 17.4013 | 16.4659 | 3 | 2 | 2 | 2 | 3 | 2 | 2 | 2 |
| USP10 | Q14694 | 3.06E-08 | 1.05E-08 | -0.302268 | | 0.920132 | -1.81051 | | 16.262 | 16.2385 | 16.2076 | 16.9207 | 16.567 | 16.227 | 16.3107 | 16.6661 | 16 | 16 | 16 | 17 | 16 | 17 | 19 | 16 |
| SEC24A | O95486 | 2.13E-13 | 1.86E-13 | -0.301705 | + | 3.48066 | -7.32535 | | 17.002 | 16.9676 | 16.9501 | 17.1917 | 17.3197 | 17.2595 | 17.3613 | 17.2424 | 22 | 23 | 23 | 23 | 20 | 23 | 22 | 23 |
| ENPP2 | Q13822;Q1 | 3.48E-07 | 1.07E-07 | -0.301484 | | 0.199066 | -0.50387 | | 13.9151 | 13.7774 | 13.9144 | 15.7613 | 14.3081 | 13.064 | 13.7053 | 14.0135 | 8 | 10 | 8 | 13 | 6 | 5 | 6 | 6 |
| UBE2N | P61088 | 0.000902 | 0.000187 | -0.300985 | + | 1.43518 | -2.67638 | | 19.3267 | 19.1472 | 19.3317 | 19.5995 | 19.3159 | 19.4845 | 19.7161 | 19.7316 | 11 | 9 | 11 | 11 | 11 | 10 | 10 | 10 |
| RPS6KB1 | P23443;P2 | 2.11E-06 | 5.92E-07 | -0.300853 | | 1.2673 | -2.38984 | | 15.5875 | 15.6489 | 15.7146 | 15.6742 | 15.832 | 16.1698 | 15.9513 | 16.1287 | 4 | 5 | 5 | 4 | 3 | 4 | 4 | 4 |
| PTPN14 | Q15678 | 2.99E-10 | 1.33E-10 | -0.300105 | + | 1.35481 | -2.53839 | | 15.3059 | 15.2554 | 15.2741 | 15.6202 | 15.8379 | 15.3155 | 15.6519 | 15.4673 | 29 | 27 | 27 | 27 | 24 | 26 | 25 | 25 |
| CLU | P10909;P1 | 0.001026 | 0.000211 | -0.300097 | | 0.410287 | -0.92889 | | 14.646 | 14.4907 | 14.6027 | 14.8867 | 14.8964 | 15.7508 | 14.3954 | 14.4702 | 3 | 3 | 3 | 3 | 3 | 4 | 3 | 3 |
| CD47 | Q08722;Q0 | 6.93E-07 | 2.05E-07 | -0.299806 | + | 2.46632 | -4.67397 | | 18.8839 | 18.9141 | 18.9193 | 19.3903 | 19.1685 | 19.1292 | 19.141 | 19.199 | 3 | 3 | 3 | 3 | 3 | 3 | 3 | 3 |
| CACNA2D1 | P54289;P5 | 5.88E-13 | 4.51E-13 | -0.299622 | + | 1.44895 | -2.70019 | | 16.18 | 16.197 | 16.0968 | 16.4578 | 16.5749 | 16.5233 | 16.5872 | 16.1447 | 19 | 19 | 19 | 20 | 19 | 18 | 21 | 18 |
| MB21D2 | Q8IYB1 | 1.75E-15 | 2.81E-15 | -0.299577 | + | 1.69024 | -3.12686 | | 15.7218 | 15.6004 | 15.5919 | 15.9637 | 15.9088 | 15.7134 | 16.1381 | 15.9641 | 11 | 10 | 12 | 9 | 11 | 10 | 12 | 11 |
| CASP8 | Q14790-9 | 2.57E-10 | 1.16E-10 | -0.299515 | + | 1.56583 | -2.90449 | | 16.853 | 16.8236 | 16.8934 | 17.2066 | 17.3283 | 17.2953 | 16.965 | 16.9855 | 7 | 7 | 7 | 9 | 6 | 8 | 8 | 8 |
| SESTD1 | Q86VW0 | 0.000121 | 2.83E-05 | -0.299341 | | 0.452306 | -1.00671 | | 14.5706 | 14.4978 | 14.4665 | 15.4768 | 14.3177 | 15.1659 | 14.4108 | 14.6838 | 2 | 4 | 4 | 7 | 3 | 8 | 3 | 5 |
| PGLS | O95336 | 4.45E-11 | 2.28E-11 | -0.299059 | | 0.713273 | -1.46404 | | 19.3974 | 19.0775 | 19.397 | 19.0867 | 19.6558 | 19.5066 | 19.8188 | 19.8805 | 12 | 11 | 11 | 11 | 11 | 11 | 11 | 11 |
| SLC38A9 | Q8NBW4;C | 0.000521 | 0.000112 | -0.298807 | | 1.14367 | -2.18229 | | 12.9047 | 13.1635 | 13.1174 | 13.3058 | 13.0368 | 13.5544 | 13.5249 | 13.3816 | 2 | 2 | 2 | 2 | 2 | 3 | 3 | 3 |
| CLASP1 | Q7Z460;Q7 | 2.18E-10 | 9.94E-11 | -0.298688 | + | 1.98952 | -3.68708 | | 14.9045 | 14.9946 | 14.9568 | 15.3517 | 15.3024 | 15.032 | 15.2251 | 15.3422 | 38 | 40 | 40 | 40 | 27 | 37 | 37 | 39 |
| TPRG1L | Q5T0D9 | 5.56E-15 | 7.56E-15 | -0.298109 | | 1.17737 | -2.23864 | | 16.1001 | 16.0941 | 16.0679 | 16.5928 | 16.5227 | 16.0168 | 16.4187 | 16.3764 | 7 | 7 | 7 | 8 | 7 | 7 | 8 | 8 |
| PTTG1IP | P53801 | 1.73E-08 | 6.10E-09 | -0.297842 | | 1.05311 | -2.03143 | | 20.246 | 19.8774 | 20.1537 | 20.2405 | 20.6095 | 20.5252 | 20.1168 | 20.4592 | 3 | 3 | 3 | 3 | 3 | 3 | 3 | 3 |
| JUP | P14923 | 7.52E-05 | 1.81E-05 | -0.297601 | | 0.383344 | -0.87809 | | 15.9195 | 15.7023 | 16.2405 | 16.8083 | 16.3916 | 15.3702 | 16.2394 | 16.4491 | 14 | 16 | 18 | 18 | 14 | 11 | 14 | 17 |
| STX7 | O15400;O1 | 1.61E-07 | 5.11E-08 | -0.297321 | + | 1.49044 | -2.77224 | | 17.9454 | 17.8848 | 18.0086 | 18.3046 | 18.3865 | 18.0211 | 18.4077 | 18.0981 | 9 | 10 | 8 | 8 | 8 | 9 | 9 | 9 |
| AASDHPPT | Q9NRN7 | 2.06E-08 | 7.18E-09 | -0.29723 | + | 2.79382 | -5.43653 | | 16.51 | 16.559 | 16.6209 | 16.8037 | 17.005 | 16.8398 | 16.8457 | 16.8086 | 8 | 8 | 8 | 8 | 8 | 8 | 9 | 8 |
| NAA25 | Q14CX7 | 1.09E-05 | 2.86E-06 | -0.297221 | + | 2.60882 | -4.99631 | | 15.4371 | 15.4508 | 15.4815 | 15.8652 | 15.829 | 15.632 | 15.7645 | 15.6778 | 20 | 19 | 20 | 21 | 17 | 18 | 21 | 16 |
| EIF4A1 | P60842 | 2.54E-09 | 9.95E-10 | -0.297012 | + | 2.26644 | -4.24421 | | 20.7731 | 20.8492 | 20.7763 | 21.274 | 21.0648 | 21.0333 | 20.9795 | 21.1311 | 25 | 25 | 26 | 25 | 24 | 25 | 25 | 25 |
| CEP170B | Q9Y4F5;Q9 | 1.47E-13 | 1.33E-13 | -0.297001 | + | 1.6987 | -3.14219 | | 14.6405 | 14.5508 | 14.7439 | 14.9001 | 15.0577 | 14.8372 | 14.7915 | 15.1238 | 11 | 11 | 9 | 18 | 8 | 10 | 16 | 13 |
| FBLN5 | Q9UBX5 | 1.08E-05 | 2.83E-06 | -0.297 | + | 1.52384 | -2.83061 | | 14.5024 | 14.5639 | 14.398 | 14.8924 | 14.5868 | 14.6298 | 14.8608 | 14.9558 | 4 | 3 | 3 | 3 | 3 | 5 | 5 | 7 |
| TRIM22 | Q8IYM9 | 9.96E-05 | 2.36E-05 | -0.296964 | | 1.02972 | -1.99256 | | 18.2337 | 18.3092 | 18.2939 | 18.2307 | 18.9187 | 18.6585 | 18.5342 | 18.5374 | 10 | 10 | 9 | 9 | 9 | 10 | 10 | 10 |
| RAPGEF2 | Q9Y4G8 | 4.11E-05 | 1.02E-05 | -0.296911 | | 0.829814 | -1.66004 | | 14.896 | 14.8641 | 14.686 | 14.9428 | 15.6266 | 14.9828 | 15.0275 | 14.9819 | 8 | 6 | 5 | 6 | 4 | 7 | 5 | 7 |
| GPX8 | Q8TED1 | 6.09E-07 | 1.81E-07 | -0.296713 | | 0.838762 | -1.67499 | | 18.6015 | 18.6294 | 18.6328 | 18.7147 | 18.7735 | 19.1194 | 19.3367 | 18.6453 | 8 | 9 | 9 | 9 | 9 | 9 | 8 | 8 |
| TAX1BP3 | O14907 | 0.000186 | 4.25E-05 | -0.296229 | | 1.25212 | -2.36422 | | 19.4399 | 19.1155 | 19.4604 | 19.5516 | 19.4167 | 19.7042 | 19.663 | 19.8389 | 3 | 3 | 3 | 3 | 3 | 3 | 3 | 3 |
| OCIAD1 | Q9NX40 | 4.21E-12 | 2.62E-12 | -0.296029 | + | 2.16699 | -4.03934 | | 17.1741 | 17.1738 | 17.1536 | 17.4614 | 17.5088 | 17.2542 | 17.5653 | 17.5261 | 12 | 11 | 11 | 11 | 11 | 12 | 11 | 11 |
| PLEKHM2 | Q8IWE5 | 1.61E-07 | 5.11E-08 | -0.295886 | + | 2.01143 | -3.7297 | | 17.5407 | 17.5494 | 17.7519 | 17.9806 | 17.9596 | 17.7998 | 18.011 | 17.7985 | 7 | 6 | 4 | 6 | 6 | 6 | 5 | 5 |
| PNPLA2 | Q96AD5 | 0.027468 | 0.004526 | -0.295609 | + | 1.36571 | -2.55703 | | 14.5562 | 14.3316 | 14.4417 | 14.6601 | 14.6187 | 15.0246 | 14.5977 | 14.7927 | 3 | 4 | 2 | 4 | 2 | 3 | 3 | 2 |
| TMEM87B | Q96K49;Q9 | 1.52E-09 | 6.13E-10 | -0.295564 | + | 1.5725 | -2.91627 | | 15.9917 | 15.6937 | 15.8166 | 16.0764 | 16.2066 | 16.1365 | 16.2894 | 15.9389 | 4 | 4 | 4 | 5 | 5 | 5 | 5 | 5 |
| OTUB1 | Q96FW1 | 1.02E-13 | 9.76E-14 | -0.295115 | | 1.06272 | -2.0474 | | 18.1276 | 17.9082 | 18.438 | 18.4761 | 18.263 | 18.3622 | 18.6653 | 18.4988 | 11 | 12 | 12 | 12 | 11 | 11 | 11 | 11 |
| RABGGTB | P53611 | 0.000241 | 5.41E-05 | -0.295056 | + | 1.73511 | -3.20845 | | 16.2841 | 16.2112 | 16.3925 | 16.4713 | 16.6256 | 16.5805 | 16.8095 | 16.468 | 3 | 4 | 4 | 5 | 3 | 5 | 5 | 5 |
| SOWAHC | Q53LP3 | 1.13E-14 | 1.41E-14 | -0.294683 | + | 1.71215 | -3.16661 | | 14.4794 | 14.3497 | 14.4127 | 14.6099 | 14.5424 | 14.883 | 14.6614 | 14.8464 | 5 | 4 | 3 | 4 | 3 | 5 | 4 | 4 |
| STXBP5 | Q5T5C0;Q5 | 5.73E-13 | 4.41E-13 | -0.29453 | + | 1.55948 | -2.89328 | | 16.0124 | 15.9919 | 15.9508 | 16.2505 | 16.4351 | 16.3655 | 16.346 | 16.0007 | 16 | 17 | 17 | 17 | 16 | 16 | 18 | 17 |
| CCDC127 | Q96BQ5 | 0.004456 | 0.000803 | -0.294519 | | 0.374325 | -0.86091 | | 14.2889 | 14.9444 | 14.8037 | 15.4379 | 14.5082 | 14.3181 | 15.3286 | 15.2747 | 2 | 2 | 2 | 3 | 2 | 1 | 2 | 3 |
| GSDME | O60443 | 1.99E-09 | 7.89E-10 | -0.294367 | | 0.788843 | -1.59145 | | 16.9176 | 16.884 | 16.8331 | 16.7982 | 17.2017 | 17.6193 | 17.2553 | 16.9886 | 18 | 16 | 18 | 17 | 19 | 19 | 17 | 16 |
| MBNL2 | Q5VZF2;Q5 | 1.70E-10 | 7.90E-11 | -0.294208 | | 0.424569 | -0.95552 | | 16.4008 | 16.4397 | 16.5365 | 17.0734 | 15.933 | 17.2653 | 16.6492 | 16.8451 | 3 | 3 | 3 | 4 | 3 | 4 | 4 | 3 |
| SETD3 | Q86TU7 | 4.84E-09 | 1.83E-09 | -0.293596 | + | 2.07773 | -3.8601 | | 16.0683 | 16.0518 | 16.0005 | 16.3177 | 16.3002 | 16.1787 | 16.5261 | 16.3462 | 19 | 18 | 18 | 18 | 15 | 18 | 18 | 20 |
| GOPC | Q9HD26;Q | 1.02E-15 | 1.74E-15 | -0.293393 | | 0.943919 | -1.85004 | | 16.1691 | 16.2555 | 16.1583 | 16.3521 | 16.2355 | 16.831 | 16.7054 | 16.3144 | 19 | 18 | 18 | 18 | 19 | 19 | 20 | 19 |
| BET1 | O15155 | 0.000147 | 3.40E-05 | -0.293314 | | 0.150609 | -0.39436 | | 15.7403 | 16.1908 | 16.3325 | 16.1351 | 18.4092 | 15.306 | 15.5517 | 16.5039 | 2 | 2 | 2 | 2 | 1 | 2 | 2 | 2 |
| ABHD12 | Q8N2K0;Q8 | 9.30E-11 | 4.49E-11 | -0.293296 | | 0.91821 | -1.80732 | | 16.1557 | 16.247 | 16.2453 | 16.4521 | 16.8201 | 16.1105 | 16.4785 | 16.6852 | 13 | 13 | 12 | 13 | 14 | 13 | 13 | 13 |
| WDSUB1 | Q8N9V3 | 2.65E-10 | 1.19E-10 | -0.293218 | + | 1.36746 | -2.56002 | | 14.2088 | 14.1699 | 14.1248 | 14.2338 | 14.7158 | 14.5803 | 14.3526 | 14.4226 | 5 | 6 | 6 | 6 | 4 | 5 | 5 | 5 |
| ENDOG | Q14249 | 3.94E-06 | 1.08E-06 | -0.292836 | | 0.802808 | -1.61486 | | 15.6822 | 15.6751 | 15.686 | 16.0488 | 16.291 | 15.5129 | 15.8522 | 16.1648 | 6 | 7 | 7 | 6 | 6 | 5 | 6 | 6 |
| PCBP2 | Q15366-4; | 0.002162 | 0.000421 | -0.292658 | | 0.81513 | -1.63549 | | 17.6787 | 17.696 | 18.3379 | 18.368 | 18.2154 | 18.0861 | 18.029 | 18.2859 | 2 | 2 | 2 | 2 | 2 | 2 | 2 | 2 |
| RPL27 | P61353 | 3.45E-11 | 1.81E-11 | -0.292451 | + | 2.752 | -5.33475 | | 19.9002 | 20.0581 | 19.9113 | 20.1471 | 20.2803 | 20.3312 | 20.2496 | 20.2365 | 9 | 9 | 9 | 7 | 8 | 9 | 8 | 8 |
| MSRA | Q9UJ68;Q9 | 1.18E-08 | 4.25E-09 | -0.292386 | | 1.29784 | -2.44151 | | 15.061 | 15.0847 | 15.1228 | 15.7087 | 15.2184 | 15.4233 | 15.2396 | 15.3194 | 5 | 5 | 5 | 5 | 4 | 4 | 4 | 4 |
| USP46 | P62068;P6 | 0.003113 | 0.000578 | -0.291774 | + | 1.30942 | -2.46114 | | 14.3943 | 14.4276 | 14.5386 | 15.0473 | 14.8205 | 14.5921 | 14.6584 | 14.608 | 1 | 3 | 3 | 2 | 2 | 3 | 3 | 3 |
| EIF4G1 | Q04637-8 | 1.63E-22 | 2.42E-21 | -0.291437 | + | 1.72814 | -3.19573 | | 17.9759 | 17.9578 | 18.0137 | 18.5008 | 18.1253 | 18.1528 | 18.2577 | 18.3328 | 54 | 51 | 52 | 51 | 49 | 49 | 52 | 50 |
| CYTH2 | Q99418;Q9 | 0.000427 | 9.27E-05 | -0.290746 | + | 2.27129 | -4.25437 | | 17.6938 | 17.4909 | 17.5361 | 17.8996 | 17.9971 | 17.8164 | 17.7771 | 17.8316 | 2 | 4 | 4 | 4 | 3 | 2 | 3 | 2 |
| RBM6 | P78332 | 1.60E-12 | 1.10E-12 | -0.290701 | | 1.0982 | -2.10645 | | 14.509 | 14.3494 | 14.5235 | 14.5709 | 15.0595 | 14.5359 | 14.8895 | 14.7008 | 7 | 8 | 8 | 7 | 6 | 6 | 5 | 6 |
| MGAT1 | P26572 | 1.65E-06 | 4.68E-07 | -0.290658 | | 1.27158 | -2.39707 | | 16.7685 | 16.8355 | 16.7576 | 17.0792 | 17.3165 | 16.7607 | 17.1422 | 17.0904 | 13 | 13 | 13 | 12 | 11 | 14 | 13 | 13 |
| MAP4K5 | Q9Y4K4 | 2.56E-24 | 7.58E-23 | -0.290558 | + | 1.31617 | -2.4726 | | 15.4596 | 15.5239 | 15.5178 | 15.4989 | 16.0464 | 15.775 | 15.8353 | 15.7993 | 21 | 21 | 21 | 21 | 18 | 20 | 20 | 21 |
| SEL1L | Q9UBV2 | 2.33E-14 | 2.65E-14 | -0.290469 | | 1.23728 | -2.33923 | | 15.4787 | 15.5116 | 15.518 | 15.5721 | 15.8637 | 15.7811 | 16.1041 | 15.6451 | 13 | 15 | 14 | 13 | 12 | 15 | 13 | 13 |
| UHRF2 | Q96PU4 | 0.000985 | 0.000203 | -0.290339 | | 1.12271 | -2.1473 | | 14.2474 | 13.8879 | 13.8719 | 14.1959 | 14.4692 | 14.1047 | 14.217 | 14.4769 | 5 | 5 | 5 | 6 | 4 | 6 | 5 | 5 |
| DDX51 | Q8N8A6 | 0.000323 | 7.14E-05 | -0.290269 | | 1.05184 | -2.02931 | | 13.6538 | 14.1202 | 13.947 | 14.2425 | 14.2152 | 14.3338 | 14.295 | 13.8999 | 4 | 5 | 4 | 3 | 2 | 4 | 5 | 3 |
| LPXN | O60711;O6 | 1.40E-16 | 3.19E-16 | -0.290245 | | 0.365455 | -0.84392 | | 16.3758 | 16.3563 | 16.34 | 17.2911 | 16.3483 | 17.1189 | 15.8712 | 16.6085 | 12 | 12 | 12 | 12 | 12 | 12 | 10 | 12 |
| TSPAN6 | O43657 | 3.94E-15 | 5.65E-15 | -0.290084 | | 0.619948 | -1.30435 | | 15.005 | 15.1395 | 15.1843 | 14.9271 | 15.2883 | 15.7012 | 15.8346 | 15.2472 | 5 | 4 | 5 | 5 | 4 | 6 | 5 | 5 |
| SWAP70 | Q9UH65 | 9.08E-12 | 5.31E-12 | -0.289717 | | 0.953624 | -1.86616 | | 16.7243 | 16.7501 | 16.6915 | 16.6258 | 17.0881 | 17.2647 | 17.1966 | 16.8833 | 34 | 35 | 34 | 34 | 32 | 35 | 38 | 36 |
| VPS4A | Q9UN37 | 2.09E-08 | 7.30E-09 | -0.289563 | + | 2.09231 | -3.88909 | | 16.5361 | 16.6287 | 16.5253 | 16.9496 | 16.9756 | 16.7117 | 16.7486 | 16.8791 | 13 | 13 | 14 | 13 | 12 | 12 | 11 | 13 |
| KATNB1 | Q9BVA0 | 1.10E-06 | 3.19E-07 | -0.289231 | | 1.08066 | -2.07724 | | 14.079 | 14.051 | 13.9252 | 14.0874 | 14.2951 | 14.4809 | 14.5866 | 14.0881 | 7 | 8 | 6 | 6 | 8 | 4 | 10 | 7 |
| PHLDB2 | Q86SQ0 | 2.74E-13 | 2.31E-13 | -0.289142 | | 1.19143 | -2.26221 | | 15.5903 | 15.6287 | 15.6564 | 16.1816 | 15.6126 | 16.0372 | 15.8744 | 15.8656 | 16 | 15 | 16 | 18 | 10 | 15 | 15 | 14 |
| BAG2 | O95816 | 2.53E-06 | 7.05E-07 | -0.288726 | + | 1.73912 | -3.21577 | | 18.5058 | 18.5372 | 18.582 | 18.8716 | 19.0289 | 18.8077 | 18.6156 | 18.8281 | 11 | 11 | 11 | 12 | 12 | 11 | 12 | 12 |
| CHST12 | Q9NRB3 | 0.010227 | 0.001752 | -0.288663 | | 1.18675 | -2.25436 | | 15.0749 | 15.0389 | 14.8732 | 15.4869 | 15.4884 | 15.0395 | 15.2562 | 15.1507 | 1 | 1 | 1 | 1 | 1 | 1 | 1 | 1 |
| ACOX3 | O15254 | 2.86E-13 | 2.40E-13 | -0.288653 | | 1.17259 | -2.23065 | | 15.4004 | 15.3183 | 15.324 | 15.5265 | 15.7656 | 15.9471 | 15.4254 | 15.5165 | 15 | 13 | 14 | 16 | 12 | 15 | 14 | 14 |
| TLE1;TLE4;TLE4;TLE4; | Q04724;Q0 | 2.36E-06 | 6.60E-07 | -0.287539 | | 0.941518 | -1.84605 | | 15.3599 | 15.422 | 15.4641 | 15.7214 | 15.6401 | 15.4426 | 16.1282 | 15.5823 | 6 | 7 | 7 | 5 | 6 | 7 | 7 | 5 |
| CD46 | P15529-2; | 7.62E-07 | 2.24E-07 | -0.287528 | | 0.553828 | -1.18898 | | 15.3992 | 15.4434 | 15.5018 | 16.4448 | 15.4633 | 15.5987 | 15.509 | 15.6624 | 6 | 6 | 5 | 7 | 5 | 5 | 5 | 6 |
| DGKA | P23743 | 6.23E-09 | 2.32E-09 | -0.287462 | + | 1.97398 | -3.65699 | | 15.8224 | 15.8598 | 15.7888 | 16.2301 | 16.2716 | 15.9928 | 16.0375 | 16.0236 | 21 | 19 | 20 | 22 | 19 | 18 | 21 | 21 |
| ELOVL1 | Q9BW60 | 1.42E-13 | 1.29E-13 | -0.287226 | | 0.682691 | -1.41204 | | 16.2253 | 15.879 | 15.9721 | 16.5203 | 16.5155 | 15.8394 | 16.1308 | 16.5576 | 3 | 3 | 3 | 3 | 3 | 3 | 3 | 3 |

| Gene names | UniProt IDs | Protein Group P-value | Protein Group Q-value | Log2 Difference NUP214 vs Controls (n=5) | p<0.05 | -log10 p-value | T-test Test statistic | NPC Localization | Log2 MS2_quantity_NUP214_1 | Log2 MS2_quantity_NUP214_2 | Log2 MS2_quantity_NUP214_3 | Log2 MS2_quantity_Control_1 | Log2 MS2_quantity_Control_2 | Log2 MS2_quantity_Control_3 | Log2 MS2_quantity_Control_4 | Log2 MS2_quantity_Control_5 | Number Peptides for Quantitation NUP214_1 | Number Peptides for Quantitation NUP214_2 | Number Peptides for Quantitation NUP214_3 | Number Peptides for Quantitation Control_1 | Number Peptides for Quantitation Control_2 | Number Peptides for Quantitation Control_3 | Number Peptides for Quantitation Control_4 | Number Peptides for Quantitation Control_5 |
|---|---|---|---|---|---|---|---|---|---|---|---|---|---|---|---|---|---|---|---|---|---|---|---|---|
| ITPR3 | Q14573 | 3.57E-19 | 1.83E-18 | -0.287118 | | 1.02292 | -1.98125 | | 15.5057 | 15.4954 | 15.5115 | 15.8032 | 16.1932 | 15.5622 | 15.6494 | 15.7485 | 41 | 42 | 42 | 47 | 34 | 40 | 33 | 39 |
| PDZRN3 | Q9UPQ7 | 3.58E-08 | 1.22E-08 | -0.286428 | | 0.316206 | -0.74784 | | 14.8492 | 14.8167 | 14.857 | 14.5614 | 15.9744 | 14.4107 | 15.3064 | 15.384 | 10 | 11 | 11 | 10 | 10 | 8 | 13 | 10 |
| ATP6V1H | Q9UI12;Q9 | 2.62E-10 | 1.18E-10 | -0.286399 | + | 2.58891 | -4.95041 | | 16.6197 | 16.6835 | 16.6844 | 16.9526 | 16.8796 | 17.0012 | 17.0724 | 16.8389 | 16 | 15 | 15 | 16 | 14 | 16 | 16 | 15 |
| PLEKHJ1 | Q9NW61 | 0.022984 | 0.003809 | -0.285944 | | 0.985808 | -2.31748 | | 14.4744 | 14.4999 | NaN | 14.9396 | 14.7703 | NaN | NaN | 14.6095 | 1 | 1 | NaN | 2 | 1 | NaN | NaN | 1 |
| TRPT1 | Q86TN4;Q8 | 0.000324 | 7.16E-05 | -0.285779 | | 0.624105 | -1.31153 | | 14.1883 | 14.3325 | 14.1743 | 14.7993 | 14.811 | 13.9492 | 14.391 | 14.6369 | 1 | 3 | 3 | 4 | 3 | 1 | 1 | 2 |
| MVP | Q14764 | 2.52E-17 | 7.11E-17 | -0.285732 | | 1.2088 | -2.29134 | | 19.7601 | 19.773 | 19.7385 | 20.3325 | 20.183 | 19.8867 | 19.8389 | 19.9734 | 53 | 53 | 54 | 54 | 52 | 53 | 53 | 53 |
| NKRF | O15226;O1 | 5.26E-07 | 1.58E-07 | -0.285583 | + | 1.73873 | -3.21506 | | 14.815 | 14.9433 | 14.7956 | 15.0772 | 15.0651 | 15.3772 | 15.1241 | 15.0408 | 6 | 4 | 5 | 6 | 4 | 5 | 7 | 5 |
| EIF1AD | Q8N9N8 | 4.35E-06 | 1.18E-06 | -0.285131 | + | 2.44649 | -4.63021 | | 14.9858 | 15.0573 | 15.1616 | 15.3417 | 15.4597 | 15.3857 | 15.3468 | 15.2329 | 4 | 4 | 4 | 5 | 3 | 5 | 5 | 4 |
| LRRC42 | Q9Y546 | 0.000853 | 0.000178 | -0.285053 | | 0.587819 | -1.27502 | | 12.8434 | 13.0784 | 12.4859 | 13.3279 | NaN | 12.7124 | 13.0088 | 13.3013 | 2 | 4 | 3 | 3 | NaN | 3 | 3 | 3 |
| AP4M1 | O00189 | 3.20E-08 | 1.09E-08 | -0.284813 | | 1.22087 | -2.31161 | | 14.9614 | 14.7364 | 14.8655 | 14.9557 | 15.4296 | 15.2212 | 15.0077 | 15.082 | 3 | 5 | 5 | 5 | 4 | 6 | 4 | 5 |
| TMEM115 | Q12893 | 0.000175 | 4.01E-05 | -0.284254 | + | 1.69051 | -3.12735 | | 15.2172 | 15.3762 | 15.3828 | 15.4812 | 15.8089 | 15.5214 | 15.6933 | 15.5435 | 4 | 4 | 4 | 4 | 4 | 4 | 4 | 4 |
| ATP1B3 | P54709 | 2.13E-05 | 5.41E-06 | -0.284104 | + | 1.41139 | -2.63536 | | 18.1041 | 18.0477 | 18.0157 | 18.5927 | 18.2916 | 18.1058 | 18.3038 | 18.4059 | 11 | 11 | 11 | 11 | 11 | 11 | 11 | 11 |
| TXNDC11 | Q6PKC3;Q6 | 0.026328 | 0.004345 | -0.284052 | | 1.02299 | -1.98136 | | 14.0097 | 13.9736 | 14.2285 | 14.5144 | 14.0949 | 14.6273 | 14.1944 | 14.3424 | 3 | 3 | 3 | 3 | 3 | 2 | 3 | 3 |
| TPM2 | P07951 | 0.000334 | 7.37E-05 | -0.283899 | | 0.490396 | -1.07595 | | 15.8736 | 15.9572 | 15.908 | 15.5974 | 16.1752 | 16.835 | 16.109 | 16.2674 | 4 | 3 | 3 | 3 | 3 | 3 | 4 | 3 |
| DNTTIP2 | Q5QJE6 | 9.55E-08 | 3.09E-08 | -0.283809 | + | 1.3532 | -2.53565 | | 14.4254 | 14.5302 | 14.4921 | 14.9313 | 14.8259 | 14.4678 | 14.7238 | 14.8831 | 11 | 13 | 13 | 14 | 9 | 12 | 11 | 13 |
| CSNK1G3 | Q9Y6M4;Q | 0.00255 | 0.000486 | -0.283809 | + | 1.31333 | -2.46778 | | 14.8811 | 15.1747 | 15.106 | 15.5986 | 15.3412 | 15.2502 | 15.1778 | 15.3209 | 4 | 4 | 7 | 8 | 4 | 6 | 7 | 6 |
| ARHGAP21 | Q5T5U3 | 7.57E-13 | 5.64E-13 | -0.283779 | | 1.05082 | -2.02761 | | 14.2978 | 14.1826 | 14.1455 | 14.7488 | 14.7351 | 14.3201 | 14.3232 | 14.3349 | 15 | 15 | 19 | 16 | 12 | 12 | 13 | 13 |
| PGD | P52209;P5 | 2.38E-11 | 1.29E-11 | -0.283493 | | 0.415425 | -0.93849 | | 19.0555 | 19.0863 | 19.035 | 19.9722 | 19.6326 | 18.632 | 19.3318 | 19.1434 | 29 | 29 | 29 | 29 | 29 | 29 | 29 | 28 |
| TDP2 | O95551;O9 | 0.00494 | 0.000883 | -0.283324 | | 1.16531 | -2.21847 | | 14.8922 | 14.4248 | 14.7167 | 14.9771 | 15.0899 | 14.8292 | 14.8176 | 15.0921 | 2 | 4 | 4 | 4 | 4 | 2 | 3 | 4 |
| RPL28 | P46779 | 9.62E-06 | 2.53E-06 | -0.282964 | + | 1.83687 | -3.39648 | | 18.7557 | 18.7637 | 18.6259 | 18.7863 | 19.115 | 19.0788 | 19.0274 | 18.9828 | 11 | 11 | 11 | 11 | 12 | 12 | 12 | 12 |
| B4GALT7 | Q9UBV7 | 0.000434 | 9.42E-05 | -0.282654 | + | 2.02373 | -3.75371 | | 15.9862 | 16.051 | 15.8836 | 16.2523 | 16.4037 | 16.0929 | 16.2873 | 16.2452 | 3 | 3 | 2 | 3 | 2 | 3 | 3 | 3 |
| ATP2C1 | P98194-3 | 1.98E-16 | 4.26E-16 | -0.282591 | | 0.966424 | -1.88742 | | 15.6154 | 15.666 | 15.6404 | 16.1939 | 16.008 | 15.5155 | 15.9112 | 15.9873 | 22 | 22 | 19 | 26 | 22 | 20 | 23 | 20 |
| HEATR5B | Q9P2D3 | 9.21E-07 | 2.69E-07 | -0.282123 | | 0.828694 | -1.65817 | | 14.1179 | 14.1203 | 14.0557 | 14.4539 | 14.7178 | 14.1051 | 14.0683 | 14.5552 | 14 | 15 | 14 | 16 | 9 | 11 | 8 | 17 |
| GNL2 | Q13823 | 2.10E-05 | 5.35E-06 | -0.281833 | + | 1.74576 | -3.22793 | | 14.6159 | 14.6503 | 14.5497 | 14.9809 | 14.8877 | 14.6419 | 14.9728 | 14.9524 | 8 | 9 | 9 | 11 | 7 | 10 | 10 | 10 |
| ARMC5 | Q96C12;Q9 | 4.33E-09 | 1.65E-09 | -0.281715 | | 0.73929 | -1.50806 | | 14.4136 | 14.6484 | 14.0873 | 14.7052 | 14.8745 | 14.4482 | 14.9134 | 14.3829 | 3 | 3 | 3 | 3 | 2 | 2 | 2 | 3 |
| TSPAN13 | O95857 | 2.37E-05 | 5.99E-06 | -0.28167 | | 0.314467 | -0.74438 | | 16.0141 | 16.0759 | 15.9645 | 16.9612 | 16.545 | 15.4122 | 15.8912 | 16.6898 | 2 | 2 | 2 | 3 | 2 | 2 | 2 | 3 |
| AGAP3 | Q96P47;Q9 | 2.89E-15 | 4.32E-15 | -0.281501 | | 0.953768 | -1.8664 | | 15.1449 | 15.1588 | 15.1016 | 15.567 | 15.7277 | 15.099 | 15.2361 | 15.4533 | 7 | 5 | 6 | 5 | 5 | 4 | 5 | 4 |
| UBE2H | P62256 | 4.74E-16 | 8.80E-16 | -0.281399 | + | 3.31858 | -6.83935 | | 16.1911 | 16.2211 | 16.2606 | 16.5894 | 16.453 | 16.5578 | 16.4821 | 16.446 | 6 | 7 | 7 | 6 | 6 | 7 | 7 | 7 |
| SUPT7L | O94864;O9 | 1.39E-13 | 1.27E-13 | -0.28132 | | 1.20139 | -2.2789 | | 13.9072 | 13.8126 | 14.1671 | 14.1277 | 14.4046 | 14.0861 | 14.4294 | 14.1703 | 1 | 2 | 2 | 2 | 2 | 2 | 2 | 2 |
| C1GALT1 | Q9NS00 | 0.000555 | 0.000119 | -0.2809 | + | 1.33312 | -2.50143 | | 15.3367 | 15.1948 | 15.1314 | 15.2903 | 15.6999 | 15.3536 | 15.5642 | 15.6014 | 6 | 5 | 6 | 3 | 6 | 4 | 4 | 5 |
| BTBD1 | Q9H0C5;Q9 | 0.000189 | 4.30E-05 | -0.280739 | | 0.344277 | -0.80299 | | 14.7185 | 14.2471 | 14.6386 | 15.3432 | 15.4094 | 14.3507 | 14.7911 | 14.183 | 3 | 4 | 3 | 3 | 1 | 1 | 1 | 1 |
| ITFG1 | Q8TB96 | 1.98E-11 | 1.09E-11 | -0.280659 | | 0.868785 | -1.72507 | | 15.6791 | 15.632 | 15.4886 | 16.3093 | 15.7235 | 15.6204 | 15.8442 | 15.9053 | 6 | 7 | 6 | 7 | 6 | 7 | 6 | 5 |
| NADSYN1 | Q6IA69 | 5.53E-13 | 4.27E-13 | -0.280213 | | 0.959268 | -1.87554 | | 15.2439 | 15.1028 | 15.1483 | 15.3924 | 15.1046 | 15.7693 | 15.5651 | 15.3947 | 10 | 9 | 9 | 9 | 8 | 10 | 10 | 9 |
| DNAJC3 | Q13217 | 2.55E-17 | 7.16E-17 | -0.280025 | + | 1.55695 | -2.88881 | | 16.5009 | 16.4764 | 16.5125 | 16.9217 | 16.6659 | 16.5783 | 16.9559 | 16.7613 | 23 | 20 | 21 | 20 | 18 | 20 | 21 | 22 |
| RNF41 | Q9H4P4;Q9 | 0.00233 | 0.000449 | -0.279688 | | 0.707328 | -1.45395 | | 14.8686 | 14.7139 | 14.9062 | 15.1506 | 15.6286 | 14.8095 | 14.9853 | 14.972 | 1 | 2 | 2 | 2 | 1 | 1 | 1 | 2 |
| CYB5R3 | P00387;P0 | 1.78E-15 | 2.85E-15 | -0.279512 | + | 1.8078 | -3.34231 | | 19.7755 | 19.9017 | 19.8906 | 20.0451 | 20.0669 | 20.2544 | 20.2988 | 20.0121 | 20 | 21 | 20 | 21 | 18 | 21 | 21 | 20 |
| SLC12A4 | Q9UP95;Q9 | 2.58E-15 | 3.93E-15 | -0.279468 | + | 1.86072 | -3.44119 | | 16.6401 | 16.6547 | 16.622 | 16.7969 | 16.8104 | 16.9551 | 17.1316 | 16.898 | 25 | 24 | 26 | 25 | 24 | 24 | 23 | 24 |
| PXN | P49023 | 2.40E-13 | 2.05E-13 | -0.279196 | + | 2.35729 | -4.43646 | | 16.9949 | 16.9886 | 16.9395 | 17.3592 | 17.3064 | 17.1897 | 17.1057 | 17.3066 | 16 | 17 | 16 | 16 | 16 | 16 | 17 | 17 |
| RAB5C | P51148;P5 | 0.000104 | 2.47E-05 | -0.279102 | | 1.15314 | -2.19811 | | 19.1787 | 19.4112 | 19.3009 | 19.6231 | 19.7675 | 19.2456 | 19.5905 | 19.6536 | 6 | 6 | 6 | 7 | 6 | 6 | 7 | 7 |
| SCARB2 | Q14108 | 6.50E-08 | 2.14E-08 | -0.27895 | | 1.16301 | -2.21462 | | 19.291 | 19.2592 | 19.3483 | 19.4636 | 19.3073 | 19.7976 | 19.7754 | 19.5483 | 11 | 11 | 11 | 10 | 10 | 11 | 11 | 11 |
| AGTRAP | Q6RW13 | 0.000426 | 9.26E-05 | -0.278882 | | 0.507505 | -1.1067 | | 16.2576 | 16.2059 | 16.3291 | 15.8808 | 16.5162 | 16.9429 | 16.8689 | 16.5067 | 3 | 3 | 4 | 3 | 4 | 3 | 3 | 3 |
| BNIP3L | O60238;O6 | 1.06E-08 | 3.83E-09 | -0.278737 | | 1.08007 | -2.07627 | | 15.0365 | 14.7792 | 15.1157 | 15.4627 | 15.2016 | 15.4143 | 14.9963 | 15.2043 | 3 | 3 | 4 | 3 | 3 | 4 | 5 | 3 |
| PDIA6 | Q15084;Q1 | 3.54E-10 | 1.55E-10 | -0.278663 | + | 1.3324 | -2.50019 | | 21.2098 | 21.1894 | 21.1922 | 21.3472 | 21.8007 | 21.3556 | 21.4342 | 21.4412 | 22 | 21 | 21 | 21 | 21 | 21 | 21 | 22 |
| KLHDC2 | Q9Y2U9;Q9 | 3.53E-05 | 8.78E-06 | -0.278562 | + | 1.79765 | -3.3235 | | 14.8991 | 14.7465 | 14.8506 | 15.1059 | 15.1039 | 15.0248 | 15.3247 | 14.9939 | 1 | 1 | 1 | 1 | 1 | 1 | 1 | 2 |
| C9orf78 | Q9NZ63 | 0.000488 | 0.000105 | -0.278545 | | 0.627313 | -1.31707 | | 15.2772 | 15.207 | 15.4123 | 15.9763 | 15.8176 | 15.086 | 15.4255 | 15.5815 | 4 | 3 | 4 | 4 | 3 | 2 | 4 | 2 |
| MPRIP | Q6WCQ1;Q | 7.54E-12 | 4.49E-12 | -0.27849 | + | 1.62602 | -3.01138 | | 16.2253 | 16.3191 | 16.266 | 16.7748 | 16.3881 | 16.61 | 16.4461 | 16.5241 | 44 | 43 | 43 | 45 | 42 | 45 | 40 | 44 |
| TPM1 | P09493-7 | 2.93E-12 | 1.89E-12 | -0.27833 | | 0.467793 | -1.035 | | 18.249 | 18.2584 | 18.0734 | 18.1074 | 17.8843 | 18.8494 | 18.814 | 18.7046 | 12 | 13 | 12 | 11 | 10 | 15 | 13 | 12 |
| FRYL | O94915 | 1.60E-11 | 8.94E-12 | -0.278084 | | 1.02452 | -1.9839 | | 15.0475 | 15.0663 | 15.0793 | 15.5285 | 15.5403 | 15.0039 | 15.195 | 15.4444 | 32 | 31 | 35 | 38 | 25 | 25 | 28 | 33 |
| JMJD6 | Q6NYC1;Q6 | 3.29E-12 | 2.10E-12 | -0.278067 | + | 1.5013 | -2.79119 | | 15.6799 | 15.7422 | 15.8828 | 16.2484 | 16.1402 | 15.8613 | 15.994 | 15.9878 | 10 | 9 | 10 | 11 | 8 | 10 | 7 | 8 |
| MSRB2 | Q9Y3D2 | 1.06E-06 | 3.07E-07 | -0.27804 | + | 1.94473 | -3.60068 | | 15.4917 | 15.489 | 15.5655 | 15.6269 | 15.9386 | 15.8889 | 15.7202 | 15.7927 | 4 | 2 | 4 | 4 | 2 | 4 | 3 | 3 |
| ADH5 | P11766 | 2.22E-11 | 1.21E-11 | -0.277739 | + | 1.60609 | -2.97585 | | 19.1239 | 19.2362 | 19.2271 | 19.5401 | 19.2554 | 19.6534 | 19.5114 | 19.4071 | 19 | 19 | 18 | 18 | 17 | 19 | 19 | 19 |
| IP6K1 | Q92551 | 5.94E-08 | 1.97E-08 | -0.277275 | + | 2.9798 | -5.90607 | | 14.3008 | 14.2572 | 14.3031 | 14.4338 | 14.5731 | 14.5741 | 14.618 | 14.6225 | 5 | 6 | 6 | 5 | 3 | 5 | 5 | 5 |
| RAB13 | P51153 | 1.47E-08 | 5.24E-09 | -0.277253 | + | 1.33065 | -2.49722 | | 16.9018 | 16.964 | 16.9002 | 17.1365 | 17.47 | 17.2188 | 17.2131 | 16.9579 | 8 | 8 | 8 | 7 | 6 | 7 | 7 | 7 |
| WDR47 | O94967;O9 | 7.56E-17 | 1.84E-16 | -0.276846 | | 1.10426 | -2.11655 | | 14.6148 | 14.594 | 14.7158 | 15.1966 | 14.8892 | 14.6 | 14.9216 | 14.9844 | 11 | 12 | 12 | 15 | 11 | 10 | 12 | 8 |
| FBXO21 | O94952;O9 | 3.42E-05 | 8.52E-06 | -0.276464 | + | 1.96427 | -3.63826 | | 15.167 | 15.2536 | 15.0397 | 15.2761 | 15.536 | 15.3992 | 15.4311 | 15.507 | 2 | 2 | 2 | 1 | 1 | 2 | 2 | 2 |
| STK24 | Q9Y6E0-2 | 6.70E-14 | 6.71E-14 | -0.276175 | + | 4.75754 | -12.3149 | | 16.2917 | 16.2613 | 16.2477 | 16.6007 | 16.5292 | 16.5465 | 16.5208 | 16.5182 | 18 | 19 | 18 | 20 | 18 | 19 | 17 | 18 |
| AAK1 | Q2M2I8 | 5.32E-11 | 2.69E-11 | -0.276123 | + | 1.78776 | -3.3052 | | 16.4642 | 16.4414 | 16.4755 | 16.9474 | 16.7301 | 16.5593 | 16.7513 | 16.6943 | 16 | 15 | 17 | 18 | 16 | 19 | 18 | 20 |
| PTK2 | Q05397;Q0 | 1.79E-17 | 5.26E-17 | -0.275918 | + | 1.3576 | -2.54317 | | 16.0273 | 16.0062 | 16.0277 | 16.5499 | 16.3635 | 16.2519 | 16.0506 | 16.2655 | 16 | 14 | 16 | 16 | 14 | 14 | 11 | 14 |
| DPP8 | Q6V1X1;Q6 | 4.88E-09 | 1.85E-09 | -0.275888 | | 1.17803 | -2.23974 | | 14.7656 | 14.8768 | 14.8963 | 14.9432 | 15.4181 | 15.1585 | 15.1639 | 14.927 | 7 | 6 | 6 | 6 | 5 | 7 | 7 | 5 |
| E2F4 | Q16254 | 0.0028 | 0.000527 | -0.275884 | | 0.936567 | -1.83782 | | 15.2072 | 15.2432 | 15.2425 | 15.5913 | 15.8613 | 15.2075 | 15.3357 | 15.5384 | 1 | 2 | 3 | 2 | 2 | 1 | 2 | 3 |
| TXNL4B | Q9NX01 | 0.000952 | 0.000197 | -0.275777 | | 0.485667 | -1.06742 | | 15.1123 | 14.4828 | 14.7137 | 14.5933 | 15.2576 | 14.8566 | 14.967 | 15.5522 | 1 | 1 | 1 | 1 | 1 | 1 | 1 | 1 |
| MACF1 | Q9UPN3-5 | 7.44E-10 | 3.12E-10 | -0.275631 | | 0.812461 | -1.63102 | | 15.2308 | 15.24 | 15.234 | 15.9253 | 15.6705 | 15.2242 | 15.3626 | 15.37 | 34 | 34 | 34 | 46 | 33 | 32 | 39 | 41 |
| TDRD7 | Q8NHU6 | 1.87E-06 | 5.26E-07 | -0.27547 | | 0.561114 | -1.2018 | | 14.2119 | 14.1415 | 14.2966 | 14.8947 | 14.8763 | 14.0509 | 14.2329 | 14.4059 | 14 | 12 | 16 | 16 | 13 | 12 | 13 | 14 |
| EFHD2 | Q96C19 | 1.62E-05 | 4.16E-06 | -0.274577 | | 0.564416 | -1.20761 | | 17.565 | 17.5573 | 17.5895 | 18.4309 | 17.7371 | 17.5648 | 17.4934 | 17.9996 | 12 | 12 | 12 | 12 | 12 | 12 | 12 | 12 |
| TAOK2 | Q9UL54-2 | 0.002275 | 0.00044 | -0.274262 | | 0.38454 | -0.89348 | | 15.3243 | 15.2549 | 15.2706 | 15.9976 | 14.8074 | 15.7289 | NaN | 15.6963 | 2 | 2 | 2 | 2 | 2 | 2 | NaN | 2 |
| EHD2 | Q9NZN4 | 1.99E-08 | 6.97E-09 | -0.274243 | + | 1.36803 | -2.56098 | | 19.7801 | 19.8063 | 19.7814 | 19.8558 | 20.3007 | 20.1924 | 19.9886 | 19.98 | 32 | 31 | 32 | 31 | 32 | 32 | 32 | 33 |
| HOMER3 | Q9NSC5;Q9 | 6.14E-12 | 3.73E-12 | -0.274198 | + | 1.71867 | -3.17846 | | 15.3543 | 15.3536 | 15.3177 | 15.6964 | 15.72 | 15.3727 | 15.5977 | 15.6937 | 11 | 12 | 11 | 12 | 12 | 10 | 12 | 13 |
| WFS1 | O76024 | 7.57E-15 | 9.86E-15 | -0.273805 | | 0.817078 | -1.63875 | | 16.2543 | 16.2775 | 16.2855 | 16.6171 | 16.676 | 16.1752 | 16.3673 | 16.8955 | 15 | 14 | 14 | 15 | 13 | 14 | 16 | 15 |
| CLNS1A | P54105 | 3.37E-12 | 2.15E-12 | -0.273569 | | 0.903217 | -1.78239 | | 17.117 | 17.0807 | 17.2468 | 17.7632 | 17.2199 | 17.5687 | 17.1565 | 17.4002 | 6 | 6 | 6 | 5 | 5 | 5 | 4 | 4 |
| PSMB5 | P28074 | 0.001945 | 0.000382 | -0.272941 | + | 2.54597 | -4.85242 | | 18.8469 | 18.8323 | 18.8119 | 18.9623 | 19.1185 | 19.1573 | 19.2077 | 19.0709 | 3 | 3 | 2 | 2 | 2 | 2 | 2 | 2 |
| LZTR1 | Q8N653 | 9.47E-13 | 6.91E-13 | -0.27292 | + | 2.47315 | -4.68911 | | 16.9039 | 16.8622 | 16.8698 | 17.2677 | 17.1734 | 17.0106 | 17.1125 | 17.1936 | 8 | 8 | 9 | 9 | 8 | 8 | 6 | 7 |
| ABHD14B | Q96IU4 | 6.96E-12 | 4.18E-12 | -0.272912 | | 0.5511 | -1.18417 | | 18.6061 | 18.4392 | 18.5362 | 18.2746 | 18.8767 | 19.2601 | 19.0064 | 18.5826 | 7 | 7 | 7 | 7 | 7 | 7 | 7 | 7 |
| EIF5A2 | Q9GZV4 | 3.80E-08 | 1.29E-08 | -0.272884 | | 0.216072 | -0.54093 | | 13.7519 | 13.682 | 13.426 | 15.167 | 12.8896 | 13.5223 | 13.8164 | 14.069 | 1 | 1 | 1 | 2 | 1 | 1 | 1 | 1 |
| C2orf69 | Q8N8R5 | 0.004266 | 0.000771 | -0.27282 | | 0.365461 | -0.84394 | | 14.7367 | 15.4392 | 14.9939 | 15.4106 | 16.0985 | 14.8381 | 15.248 | 15.052 | 4 | 3 | 4 | 3 | 2 | 2 | 4 | 3 |
| PDCL3 | Q9H2J4 | 3.32E-08 | 1.13E-08 | -0.272579 | | 0.797437 | -1.60586 | | 15.7067 | 15.7903 | 15.7443 | 16.2173 | 15.5769 | 15.8928 | 16.2147 | 16.1968 | 11 | 10 | 11 | 11 | 10 | 11 | 11 | 10 |
| FBXO45 | P0C2W1 | 0.002607 | 0.000495 | -0.272361 | | 0.864739 | -1.71832 | | 14.2826 | 14.5777 | 14.3874 | 14.8679 | 14.453 | 15.0199 | 14.5831 | 14.5173 | 2 | 2 | 1 | 2 | 2 | 2 | 2 | 2 |
| HSPA1A;HSPA1B | P0DMV8;P | 1.94E-11 | 1.07E-11 | -0.272078 | | 1.22355 | -2.31613 | | 20.0608 | 20.0717 | 20.0719 | 20.6106 | 20.4236 | 20.0845 | 20.3407 | 20.2417 | 33 | 34 | 32 | 35 | 34 | 33 | 33 | 34 |
| PROCR | Q9UNN8 | 1.45E-15 | 2.38E-15 | -0.272012 | | 0.262377 | -0.63881 | | 17.5456 | 17.562 | 17.3995 | 18.5203 | 17.3488 | 18.5668 | 17.0778 | 17.3582 | 2 | 2 | 2 | 2 | 2 | 2 | 2 | 2 |

| Gene names | UniProt IDs | Protein Group P-value | Protein Group Q-value | Log2 Difference NUP214 vs Controls (n=5) | p<0.05 | -log10 p-value | T-test Test statistic | NPC Localization | Log2 MS2_quantity_NUP214_1 | Log2 MS2_quantity_NUP214_2 | Log2 MS2_quantity_NUP214_3 | Log2 MS2_quantity_Control_1 | Log2 MS2_quantity_Control_2 | Log2 MS2_quantity_Control_3 | Log2 MS2_quantity_Control_4 | Log2 MS2_quantity_Control_5 | Number Peptides for Quantitation NUP214_1 | Number Peptides for Quantitation NUP214_2 | Number Peptides for Quantitation NUP214_3 | Number Peptides for Quantitation Control_1 | Number Peptides for Quantitation Control_2 | Number Peptides for Quantitation Control_3 | Number Peptides for Quantitation Control_4 | Number Peptides for Quantitation Control_5 |
|---|---|---|---|---|---|---|---|---|---|---|---|---|---|---|---|---|---|---|---|---|---|---|---|---|
| PPP1R18 | Q6NYC8 | 4.65E-12 | 2.87E-12 | -0.271897 | + | 2.07104 | -3.84685 | | 17.1655 | 17.1895 | 17.2884 | 17.6246 | 17.5182 | 17.4524 | 17.3256 | 17.5107 | 23 | 23 | 23 | 23 | 24 | 23 | 23 | 23 |
| STAU2 | Q9NUL3;Q | 3.02E-12 | 1.94E-12 | -0.271868 | | 1.19008 | -2.25994 | | 16.3329 | 16.411 | 16.4787 | 16.9632 | 16.6307 | 16.5152 | 16.5029 | 16.785 | 9 | 9 | 10 | 12 | 9 | 11 | 8 | 9 |
| TEX2 | Q8IWB9;Q | 0.000577 | 0.000123 | -0.271782 | | 0.885461 | -1.75284 | | 15.9995 | 16.0039 | 15.9527 | 16.6486 | 15.9403 | 16.2141 | 16.1611 | 16.3216 | 12 | 11 | 8 | 12 | 11 | 9 | 11 | 11 |
| GALNT7 | Q86SF2 | 3.72E-06 | 1.02E-06 | -0.27155 | | 0.517644 | -1.12483 | | 15.0369 | 15.0315 | 14.9917 | 15.9554 | 15.3593 | 14.9128 | 15.074 | 15.1562 | 10 | 10 | 9 | 12 | 9 | 8 | 8 | 9 |
| ERAL1 | O75616 | 0.002354 | 0.000453 | -0.271355 | | 0.490464 | -1.07607 | | 15.1621 | 15.1445 | 14.7927 | 15.3445 | 15.8204 | 14.895 | 14.9293 | 15.5332 | 3 | 4 | 4 | 6 | 2 | 3 | 4 | 5 |
| EPB41L5 | Q9HCM4;Q | 0.00385 | 0.000701 | -0.271342 | | 0.638645 | -1.3366 | | 14.1488 | 14.0179 | 13.9941 | 14.8718 | 14.2037 | 14.1279 | 14.0222 | 14.3991 | 2 | 2 | 1 | 3 | 2 | 2 | 1 | 2 |
| RAD23B | P54727 | 1.51E-14 | 1.83E-14 | -0.271271 | | 0.84674 | -1.68831 | | 18.9409 | 18.8534 | 18.9894 | 19.0562 | 19.532 | 19.2907 | 18.8329 | 19.284 | 18 | 19 | 18 | 17 | 17 | 19 | 17 | 19 |
| COX15 | Q7KZN9;Q | 2.03E-08 | 7.07E-09 | -0.271267 | | 0.730248 | -1.49278 | | 17.3576 | 17.2224 | 17.2726 | 17.9705 | 17.2899 | 17.3307 | 17.4115 | 17.7749 | 5 | 4 | 4 | 6 | 5 | 5 | 5 | 6 |
| LARP1 | Q6PKG0 | 2.22E-11 | 1.21E-11 | -0.271108 | + | 2.80275 | -5.45843 | | 16.2219 | 16.2226 | 16.235 | 16.5773 | 16.3754 | 16.5172 | 16.5625 | 16.4556 | 28 | 29 | 29 | 29 | 27 | 28 | 32 | 30 |
| NACA | E9PAV3;E9 | 1.50E-08 | 5.34E-09 | -0.271021 | + | 3.67397 | -7.94194 | | 20.159 | 20.2164 | 20.1702 | 20.5326 | 20.4072 | 20.4204 | 20.4226 | 20.4818 | 6 | 6 | 6 | 6 | 6 | 6 | 6 | 6 |
| TANK | Q92844 | 0.001885 | 0.000371 | -0.270939 | | 0.680329 | -1.40802 | | 14.4819 | 14.2957 | 14.251 | 14.8803 | 14.4846 | 14.9992 | 14.4352 | 14.2697 | 3 | 4 | 4 | 4 | 3 | 4 | 3 | 3 |
| MAST2 | Q6P0Q8 | 0.005535 | 0.000981 | -0.27062 | | 0.757595 | -1.53893 | | 14.7083 | 14.4892 | 14.8673 | 14.8967 | 15.3241 | 14.9311 | 14.5977 | 15.0448 | 4 | 4 | 3 | 6 | 4 | 5 | 4 | 5 |
| HINT1 | P49773 | 1.12E-09 | 4.63E-10 | -0.270281 | + | 1.6251 | -3.00974 | | 18.3727 | 18.3157 | 18.4164 | 18.4773 | 18.4878 | 18.7113 | 18.7966 | 18.7198 | 9 | 9 | 9 | 9 | 9 | 9 | 9 | 9 |
| KIFBP | Q96EK5 | 3.11E-07 | 9.60E-08 | -0.270275 | + | 1.48069 | -2.75527 | | 16.1499 | 16.2154 | 16.1986 | 16.6135 | 16.549 | 16.3297 | 16.2411 | 16.5578 | 20 | 20 | 21 | 21 | 20 | 21 | 22 | 20 |
| NDUFV3 | P56181-2 | 5.08E-17 | 1.30E-16 | -0.269741 | + | 3.43621 | -7.18935 | | 15.5017 | 15.5785 | 15.5328 | 15.8694 | 15.8485 | 15.7829 | 15.8106 | 15.7255 | 12 | 11 | 10 | 11 | 12 | 12 | 11 | 11 |
| ZDHHC17 | Q8IUH5 | 0.01584 | 0.002659 | -0.2697 | | 0.774352 | -1.61084 | | 15.1957 | 15.6319 | 15.0188 | 15.4003 | NaN | 15.6514 | 15.6366 | 15.5189 | 2 | 1 | 1 | 3 | NaN | 2 | 3 | 2 |
| MSN | P26038 | 1.10E-14 | 1.38E-14 | -0.269381 | | 0.954716 | -1.86797 | | 19.8953 | 19.8215 | 19.798 | 20.3371 | 20.183 | 20.0967 | 19.7085 | 20.2128 | 43 | 46 | 44 | 45 | 45 | 46 | 44 | 47 |
| HSPA8 | P11142 | 3.01E-14 | 3.31E-14 | -0.269085 | + | 1.80031 | -3.32842 | | 21.643 | 21.6632 | 21.6097 | 22.0114 | 22.0279 | 21.713 | 21.8278 | 21.9585 | 39 | 38 | 37 | 38 | 38 | 36 | 36 | 36 |
| MTCH1 | Q9NZJ7;Q9 | 8.36E-09 | 3.07E-09 | -0.269004 | | 0.801784 | -1.61315 | | 16.7915 | 16.9475 | 16.801 | 17.3097 | 16.9323 | 17.3934 | 16.7396 | 17.2035 | 12 | 13 | 12 | 13 | 11 | 11 | 10 | 12 |
| NR2C2AP | Q86WQ0;Q | 1.49E-15 | 2.43E-15 | -0.268902 | | 0.921478 | -1.81275 | | 14.9532 | 15.0118 | 15.2614 | 15.3261 | 15.6252 | 15.0935 | 15.1776 | 15.4995 | 4 | 4 | 5 | 5 | 3 | 3 | 5 | 4 |
| PPP1R2 | P41236 | 5.69E-08 | 1.89E-08 | -0.268713 | | 1.01108 | -1.96159 | | 15.4069 | 14.9953 | 15.5844 | 15.5377 | 15.5704 | 15.7072 | 15.6634 | 15.5092 | 7 | 6 | 6 | 7 | 7 | 7 | 8 | 8 |
| PHAX | Q9H814 | 2.31E-11 | 1.26E-11 | -0.268412 | + | 1.66136 | -3.07475 | | 14.8684 | 14.8482 | 14.9406 | 15.2809 | 15.144 | 14.916 | 15.1959 | 15.234 | 8 | 8 | 9 | 7 | 8 | 7 | 8 | 7 |
| TBC1D10B | Q4KMP7 | 5.34E-10 | 2.29E-10 | -0.268407 | + | 1.54004 | -2.85905 | | 14.7257 | 14.7212 | 14.6293 | 15.1342 | 14.7882 | 15.0289 | 14.811 | 15.0401 | 12 | 14 | 11 | 12 | 10 | 12 | 12 | 12 |
| TBC1D10A | Q9BXI6;Q9 | 1.04E-08 | 3.77E-09 | -0.267865 | + | 1.55516 | -2.88565 | | 14.2095 | 14.0047 | 14.191 | 14.184 | 14.4628 | 14.5394 | 14.435 | 14.3933 | 7 | 5 | 7 | 6 | 4 | 6 | 6 | 6 |
| KCTD15 | Q96SI1 | 0.008038 | 0.001396 | -0.267798 | | 0.165595 | -0.47272 | | NaN | 15.1292 | 15.2527 | 14.8956 | NaN | NaN | 16.0219 | NaN | NaN | 3 | 2 | 1 | NaN | NaN | 2 | NaN |
| ANKRD50 | Q9ULJ7 | 0.002238 | 0.000434 | -0.267634 | | 0.43388 | -0.97278 | | 14.1563 | 14.3901 | 14.0462 | 14.5782 | 13.7007 | 14.511 | 14.8286 | 14.7071 | 3 | 3 | 3 | 6 | 3 | 3 | 4 | 4 |
| PANX1 | Q96RD7;Q | 0.000553 | 0.000118 | -0.267012 | | 0.598102 | -1.26647 | | 16.4952 | 16.333 | 16.3291 | 17.1979 | 16.5876 | 16.4422 | 16.2944 | 16.7415 | 4 | 4 | 4 | 4 | 3 | 4 | 3 | 4 |
| PI4K2A | Q9BTU6 | 1.55E-15 | 2.52E-15 | -0.266982 | + | 1.77347 | -3.27882 | | 16.3042 | 16.3372 | 16.38 | 16.5003 | 16.7113 | 16.4353 | 16.7421 | 16.6483 | 22 | 22 | 22 | 20 | 20 | 21 | 22 | 21 |
| NCSTN | Q92542;Q | 3.76E-05 | 9.32E-06 | -0.266678 | | 1.06413 | -2.04975 | | 18.3086 | 18.1914 | 18.227 | 18.3708 | 18.5595 | 18.2374 | 18.7964 | 18.5811 | 9 | 9 | 9 | 9 | 9 | 9 | 9 | 9 |
| GBA | P04062;P0 | 9.20E-13 | 6.72E-13 | -0.266544 | | 0.682376 | -1.41151 | | 18.041 | 18.0869 | 18.0475 | 17.9593 | 18.1125 | 18.4792 | 18.7687 | 18.3053 | 21 | 20 | 20 | 19 | 19 | 20 | 21 | 20 |
| PI4KB | Q9UBF8;Q | 3.57E-11 | 1.86E-11 | -0.266398 | | 1.14799 | -2.18951 | | 14.0533 | 14.2067 | 14.314 | 14.6744 | 14.59 | 14.2204 | 14.3508 | 14.4531 | 10 | 8 | 7 | 9 | 7 | 7 | 9 | 7 |
| PARG | Q86W56;Q | 0.000157 | 3.62E-05 | -0.266297 | | 0.75922 | -1.54166 | | 14.9082 | 14.8995 | 14.3772 | 14.8972 | 15.3205 | 15.0245 | 14.8844 | 14.8463 | 8 | 7 | 9 | 8 | 5 | 6 | 5 | 9 |
| MEAF6 | Q9HAF1;Q | 0.005525 | 0.000979 | -0.265937 | | 0.868078 | -1.72389 | | 14.9834 | 14.8565 | 14.9076 | 15.0866 | 15.3927 | 14.9421 | 14.9783 | 15.5092 | 2 | 3 | 2 | 2 | 2 | 1 | 1 | 2 |
| MYOF | Q9NZM1;Q | 3.98E-13 | 3.21E-13 | -0.265932 | | 1.27788 | -2.40771 | | 19.256 | 19.2825 | 19.2434 | 19.4845 | 19.5723 | 19.4023 | 19.8212 | 19.3525 | 125 | 128 | 126 | 125 | 122 | 126 | 128 | 125 |
| NAXE | Q8NCW5;Q | 1.24E-12 | 8.77E-13 | -0.265725 | | 1.26966 | -2.39383 | | 17.6483 | 17.5135 | 17.6884 | 17.6329 | 17.9996 | 17.8591 | 18.0891 | 17.8316 | 8 | 7 | 8 | 8 | 8 | 8 | 9 | 8 |
| TMEM179B | Q7Z7N9 | 0.00036 | 7.89E-05 | -0.265569 | | 0.362136 | -0.83754 | | 16.3761 | 15.7804 | 16.0269 | 15.9535 | 16.0461 | 16.6274 | 17.0423 | 15.9642 | 1 | 2 | 2 | 1 | 2 | 2 | 2 | 2 |
| LDLR | P01130;P0 | 5.69E-15 | 7.71E-15 | -0.265554 | | 0.417222 | -0.94185 | | 15.8019 | 15.7794 | 15.7206 | 16.5946 | 16.0764 | 15.746 | 15.4044 | 16.3429 | 12 | 12 | 13 | 12 | 13 | 11 | 11 | 13 |
| CNOT8 | Q9UFF9;Q | 0.002912 | 0.000545 | -0.264771 | | 0.761779 | -1.65597 | | 14.1201 | 13.9955 | 14.4915 | 14.5535 | NaN | 14.4912 | 14.3567 | NaN | 1 | 2 | 1 | 2 | NaN | 1 | 1 | NaN |
| PIP4P2 | Q8N4L2 | 4.82E-11 | 2.46E-11 | -0.264373 | | 1.26611 | -2.38783 | | 16.2806 | 16.2158 | 16.3256 | 16.6864 | 16.5757 | 16.2565 | 16.6971 | 16.4761 | 4 | 4 | 4 | 4 | 4 | 4 | 4 | 4 |
| IFRD2 | Q12894;Q | 6.71E-06 | 1.79E-06 | -0.264202 | | 1.1183 | -2.13996 | | 14.5186 | 14.1604 | 14.4699 | 14.6374 | 14.7616 | 14.6167 | 14.8089 | 14.4112 | 3 | 3 | 3 | 4 | 3 | 4 | 3 | 3 |
| R3HCC1 | Q9Y3T6;Q9 | 0.004188 | 0.000758 | -0.264059 | + | 1.79729 | -3.32284 | | 15.6369 | 15.5839 | 15.62 | 15.821 | 16.0941 | 15.9049 | 15.8101 | 15.7584 | 2 | 2 | 2 | 3 | 2 | 1 | 2 | 2 |
| CLEC11A | Q9Y240 | 1.08E-05 | 2.81E-06 | -0.264039 | | 0.449471 | -1.00151 | | 14.8743 | 14.824 | 14.8886 | 14.8902 | 15.3661 | 14.5273 | 15.6793 | 15.1688 | 5 | 6 | 5 | 4 | 4 | 4 | 6 | 4 |
| CTTNBP2NL | Q9P2B4 | 5.15E-12 | 3.16E-12 | -0.263958 | + | 1.59882 | -2.96293 | | 15.5371 | 15.5826 | 15.5398 | 16.0279 | 15.7181 | 15.8499 | 15.6395 | 15.8501 | 17 | 17 | 14 | 17 | 17 | 16 | 17 | 17 |
| WNK1 | Q9H4A3;Q | 6.71E-13 | 5.05E-13 | -0.263529 | + | 1.88657 | -3.48992 | | 15.961 | 16.0014 | 15.9734 | 16.4618 | 16.1939 | 16.1593 | 16.2289 | 16.1668 | 24 | 23 | 24 | 27 | 21 | 22 | 22 | 21 |
| SLC12A2 | P55011 | 9.98E-12 | 5.80E-12 | -0.263354 | | 0.811411 | -1.62927 | | 15.9326 | 16.0838 | 15.9946 | 16.5729 | 15.8756 | 16.4228 | 16.2986 | 16.1653 | 16 | 17 | 17 | 17 | 10 | 15 | 13 | 14 |
| RASA2 | Q15283;Q | 5.40E-08 | 1.80E-08 | -0.263271 | + | 1.69541 | -3.13622 | | 14.5966 | 14.6626 | 14.5701 | 15.09 | 14.876 | 14.7226 | 14.8745 | 14.802 | 7 | 10 | 10 | 12 | 9 | 11 | 9 | 11 |
| ERGIC2 | Q96RQ1 | 1.98E-08 | 6.92E-09 | -0.263012 | + | 1.66163 | -3.07523 | | 15.7284 | 15.6648 | 15.789 | 15.9964 | 16.0293 | 15.7714 | 16.1478 | 16.0073 | 7 | 8 | 9 | 8 | 8 | 9 | 8 | 9 |
| HERC2 | O95714 | 1.35E-15 | 2.24E-15 | -0.262916 | | 0.546944 | -1.17684 | | 13.9326 | 13.674 | 13.7604 | 14.1965 | 14.4055 | 13.4414 | 14.0746 | 14.1305 | 10 | 10 | 11 | 11 | 8 | 5 | 8 | 11 |
| PRKG1 | Q13976 | 1.31E-11 | 7.45E-12 | -0.262693 | | 0.584422 | -1.24263 | | 16.8778 | 16.8684 | 16.8526 | 16.8966 | 16.9194 | 17.514 | 17.5142 | 16.8006 | 23 | 23 | 23 | 22 | 21 | 23 | 24 | 23 |
| MIB2 | Q96AX9;Q | 3.23E-10 | 1.42E-10 | -0.262612 | | 1.13232 | -2.16335 | | 14.259 | 14.5183 | 14.188 | 14.533 | 14.7224 | 14.7027 | 14.3259 | 14.6377 | 4 | 5 | 5 | 5 | 7 | 6 | 5 | 5 |
| NKIRAS1 | Q9NYS0 | 0.003161 | 0.000586 | -0.262547 | | 1.11546 | -2.13522 | | 15.5536 | 15.6818 | 15.5775 | 15.9073 | 15.8947 | 15.9965 | 15.5209 | 16.0148 | 2 | 2 | 4 | 3 | 2 | 3 | 2 | 2 |
| MFF | Q9GZY8 | 3.53E-08 | 1.20E-08 | -0.262381 | | 1.11475 | -2.13403 | | 14.9151 | 14.8064 | 14.9797 | 15.4646 | 15.0081 | 15.09 | 14.9999 | 15.2514 | 8 | 7 | 8 | 9 | 7 | 7 | 8 | 9 |
| MYDGF | Q969H8 | 1.47E-06 | 4.20E-07 | -0.262213 | | 0.581084 | -1.2368 | | 20.0673 | 20.1014 | 20.2158 | 20.0987 | 20.2069 | 20.4158 | 20.9772 | 20.2033 | 6 | 6 | 6 | 6 | 6 | 6 | 6 | 6 |
| RNF25 | Q96BH1 | 0.000897 | 0.000186 | -0.262186 | + | 1.64314 | -3.04203 | | 15.6373 | 15.5548 | 15.4755 | 15.8125 | 15.7029 | 15.6806 | 15.8974 | 15.9969 | 6 | 6 | 6 | 6 | 5 | 6 | 6 | 5 |
| SEC11A | P67812 | 3.77E-08 | 1.28E-08 | -0.262062 | + | 2.45527 | -4.64954 | | 18.8147 | 18.7163 | 18.7165 | 18.9895 | 18.8989 | 18.9739 | 19.09 | 19.104 | 6 | 6 | 7 | 7 | 7 | 7 | 7 | 7 |
| SMARCAL1 | Q9NZC9 | 1.87E-08 | 6.56E-09 | -0.26189 | + | 1.89847 | -3.51246 | | 13.6131 | 13.6651 | 13.6809 | 13.9862 | 13.8951 | 13.7811 | 14.085 | 13.8271 | 6 | 8 | 8 | 5 | 3 | 5 | 8 | 4 |
| LYPLAL1 | Q5VWZ2 | 0.001891 | 0.000373 | -0.261467 | | 0.605775 | -1.2798 | | 15.7814 | 15.8406 | 15.6466 | 15.6411 | 16.4177 | 16.0433 | 16.2635 | 15.7228 | 5 | 6 | 4 | 5 | 6 | 6 | 6 | 6 |
| PDCD10 | Q9BUL8 | 0.000451 | 9.76E-05 | -0.261172 | + | 1.36471 | -2.55531 | | 17.132 | 17.2835 | 17.2678 | 17.6454 | 17.5035 | 17.2296 | 17.4704 | 17.5956 | 9 | 10 | 10 | 10 | 10 | 9 | 10 | 10 |
| MAVS | Q7Z434 | 7.15E-06 | 1.91E-06 | -0.261064 | + | 1.43159 | -2.67018 | | 16.6519 | 16.6255 | 16.697 | 17.1479 | 16.7385 | 16.8535 | 17.016 | 16.8401 | 8 | 8 | 10 | 8 | 10 | 8 | 8 | 8 |
| BAD | Q92934 | 0.002967 | 0.000554 | -0.260412 | | 0.624257 | -1.31179 | | 15.0537 | 14.8572 | 15.0107 | 15.0201 | 14.7791 | 15.5603 | 15.3627 | 15.4492 | 5 | 5 | 4 | 5 | 6 | 4 | 5 | 6 |
| CARMIL1 | Q5VZK9 | 0.011501 | 0.001959 | -0.260203 | | 0.68529 | -1.45132 | | 14.4847 | 14.6501 | 14.621 | 14.7932 | 15.2331 | 14.837 | 14.5185 | NaN | 2 | 3 | 2 | 2 | 1 | 4 | 3 | NaN |
| FARS2 | O95363 | 0.002163 | 0.000421 | -0.260083 | | 0.624811 | -1.31275 | | 15.1943 | 15.4206 | 15.2322 | 15.4376 | 16.1033 | 15.283 | 15.4425 | 15.4458 | 2 | 4 | 4 | 3 | 2 | 5 | 4 | 5 |
| WDR41 | Q9HAD4 | 9.01E-13 | 6.60E-13 | -0.26008 | + | 1.61464 | -2.99108 | | 14.6838 | 14.6455 | 14.6406 | 15.1292 | 14.9444 | 14.8409 | 14.7377 | 14.9314 | 5 | 7 | 6 | 6 | 3 | 4 | 4 | 4 |
| MRM3 | Q9HC36 | 0.004266 | 0.000771 | -0.2598 | | 0.554127 | -1.18951 | | 16.8388 | 16.6924 | 16.487 | 16.5896 | 16.9693 | 16.7792 | 16.826 | 17.4986 | 3 | 3 | 2 | 4 | 2 | 3 | 2 | 2 |
| SCO1 | O75880 | 4.48E-09 | 1.71E-09 | -0.259501 | + | 1.81622 | -3.35797 | | 15.6675 | 15.781 | 15.8848 | 15.9001 | 16.1743 | 16.022 | 15.992 | 16.098 | 3 | 5 | 4 | 4 | 3 | 3 | 4 | 4 |
| THBS3 | P49746 | 0.000745 | 0.000157 | -0.258855 | | 0.376151 | -0.86439 | | 14.7085 | 14.7715 | 14.6447 | 14.9457 | 15.1697 | 14.1339 | 15.4551 | 15.1311 | 4 | 4 | 3 | 4 | 3 | 4 | 5 | 5 |
| SNX14 | Q9Y5W7;Q | 6.87E-05 | 1.66E-05 | -0.258608 | + | 1.4135 | -2.639 | | 14.9128 | 14.5128 | 14.8857 | 15.0912 | 15.0423 | 14.9883 | 14.9817 | 15.0418 | 4 | 6 | 6 | 3 | 4 | 5 | 5 | 4 |
| NAPG | Q99747 | 2.00E-15 | 3.14E-15 | -0.258493 | + | 1.75989 | -3.25385 | | 16.4515 | 16.3733 | 16.4838 | 16.6958 | 16.5588 | 16.6096 | 16.8902 | 16.7192 | 14 | 14 | 14 | 14 | 12 | 13 | 14 | 13 |
| NDUFB3 | O43676 | 0.002323 | 0.000448 | -0.258288 | + | 2.72513 | -5.27006 | | 17.0366 | 17.0036 | 17.0185 | 17.2564 | 17.3339 | 17.2834 | 17.3626 | 17.1527 | 4 | 4 | 4 | 4 | 4 | 4 | 4 | 4 |
| SRFBP1 | Q8NEF9 | 0.005918 | 0.001045 | -0.258245 | + | 1.99944 | -3.70635 | | 14.6686 | 14.6633 | 14.5635 | 15.023 | 14.9115 | 14.7315 | 14.8456 | 14.9386 | 3 | 4 | 4 | 6 | 4 | 3 | 4 | 3 |
| SLAIN2 | Q9P270 | 3.80E-13 | 3.08E-13 | -0.257742 | + | 1.40059 | -2.6168 | | 15.819 | 15.7302 | 15.9157 | 16.1936 | 16.2057 | 15.8738 | 15.9618 | 16.162 | 13 | 14 | 17 | 13 | 12 | 14 | 14 | 13 |
| TRIP10 | Q15642 | 7.34E-12 | 4.39E-12 | -0.257322 | | 0.483014 | -1.06262 | | 15.6726 | 15.7547 | 15.699 | 16.4217 | 15.5782 | 16.3639 | 15.6076 | 15.8591 | 13 | 13 | 14 | 18 | 10 | 12 | 12 | 11 |
| TRIM27 | P14373;P1 | 1.34E-08 | 4.81E-09 | -0.256427 | + | 1.87616 | -3.47025 | | 14.5868 | 14.6148 | 14.4609 | 14.7211 | 14.8372 | 14.9326 | 14.8875 | 14.6747 | 2 | 2 | 2 | 2 | 2 | 2 | 3 | 2 |
| GRN | P28799 | 6.56E-12 | 3.96E-12 | -0.256256 | | 0.682519 | -1.41175 | | 16.5953 | 16.6695 | 16.7055 | 16.5347 | 16.8007 | 16.7848 | 17.2408 | 17.2041 | 11 | 12 | 11 | 8 | 10 | 11 | 11 | 9 |
| RAB5B | P61020 | 2.33E-12 | 1.54E-12 | -0.25623 | + | 2.0507 | -3.80666 | | 19.4598 | 19.5524 | 19.5105 | 19.8869 | 19.7817 | 19.5959 | 19.7408 | 19.8138 | 9 | 8 | 8 | 8 | 8 | 8 | 8 | 8 |
| MICAL2 | O94851-4 | 6.31E-15 | 8.46E-15 | -0.256227 | | 0.588184 | -1.2492 | | 15.4821 | 15.496 | 15.5127 | 16.3114 | 15.5664 | 15.8595 | 15.4931 | 15.5354 | 22 | 25 | 25 | 28 | 21 | 27 | 25 | 26 |
| SHKBP1 | Q8TBC3 | 2.17E-12 | 1.44E-12 | -0.255892 | | 0.523239 | -1.1348 | | 14.5142 | 14.6479 | 14.7009 | 15.2515 | 14.937 | 14.326 | 14.7122 | 15.1579 | 2 | 2 | 2 | 3 | 3 | 2 | 3 | 2 |
| ROMO1 | P60602 | 0.002636 | 0.0005 | -0.255815 | | 1.07694 | -2.07106 | | 17.0664 | 17.0113 | 17.0005 | 17.5501 | 17.4291 | 17.0842 | 17.0931 | 17.2531 | 2 | 2 | 2 | 2 | 2 | 2 | 2 | 2 |

| Gene names | UniProt IDs | Protein Group P-value | Protein Group Q-value | Log2 Difference NUP214 vs Controls (n=5) | p<0.05 | -log10 p-value | T-test Test statistic | NPC Localization | Log2 MS2_quantity_NUP214_1 | Log2 MS2_quantity_NUP214_2 | Log2 MS2_quantity_NUP214_3 | Log2 MS2_quantity_Control_1 | Log2 MS2_quantity_Control_2 | Log2 MS2_quantity_Control_3 | Log2 MS2_quantity_Control_4 | Log2 MS2_quantity_Control_5 | Number Peptides for Quantitation NUP214_1 | Number Peptides for Quantitation NUP214_2 | Number Peptides for Quantitation NUP214_3 | Number Peptides for Quantitation Control_1 | Number Peptides for Quantitation Control_2 | Number Peptides for Quantitation Control_3 | Number Peptides for Quantitation Control_4 | Number Peptides for Quantitation Control_5 |
|---|---|---|---|---|---|---|---|---|---|---|---|---|---|---|---|---|---|---|---|---|---|---|---|---|
| STK11IP | Q8N1F8 | 1.53E-09 | 6.16E-10 | -0.255747 | | 0.965102 | -1.88522 | | 14.8142 | 14.6829 | 14.9135 | 14.8975 | 14.9606 | 15.0926 | 15.4152 | 14.9305 | 6 | 9 | 10 | 6 | 9 | 9 | 9 | 10 |
| CAMLG | P49069 | 6.69E-07 | 1.98E-07 | -0.25544 | + | 1.63695 | -3.03093 | | 15.7626 | 15.8317 | 15.7594 | 15.9403 | 15.8645 | 16.0609 | 16.2062 | 16.1281 | 2 | 1 | 1 | 1 | 1 | 1 | 1 | 1 |
| SHPRH | Q149N8;Q | 0.009306 | 0.001603 | -0.255374 | | 0.423454 | -0.95345 | | 17.499 | 17.1887 | 17.5344 | 17.7497 | 18.226 | 17.3202 | 17.859 | 17.1588 | 1 | 2 | 1 | 1 | 2 | 1 | 2 | 1 |
| ARHGAP17 | Q68EM7 | 1.02E-12 | 7.40E-13 | -0.254951 | + | 2.7603 | -5.35483 | | 16.8153 | 16.816 | 16.8034 | 17.1858 | 16.968 | 17.0469 | 17.0893 | 17.0426 | 16 | 18 | 17 | 18 | 15 | 17 | 19 | 15 |
| STRN3 | Q13033-2 | 2.24E-07 | 7.01E-08 | -0.254548 | + | 2.31131 | -4.33855 | | 16.4229 | 16.4352 | 16.3862 | 16.6846 | 16.7316 | 16.6657 | 16.7561 | 16.5088 | 14 | 15 | 13 | 14 | 14 | 13 | 15 | 13 |
| MTR | Q99707 | 2.77E-11 | 1.47E-11 | -0.254382 | + | 3.30227 | -6.79194 | | 14.8739 | 14.9522 | 14.9539 | 15.2267 | 15.1249 | 15.1469 | 15.2492 | 15.1575 | 21 | 21 | 24 | 19 | 17 | 19 | 20 | 18 |
| HBS1L | Q9Y450 | 1.45E-21 | 1.62E-20 | -0.254325 | + | 2.5512 | -4.86429 | | 15.8983 | 15.9328 | 15.952 | 16.2413 | 16.192 | 16.0506 | 16.2699 | 16.1564 | 26 | 28 | 26 | 27 | 22 | 26 | 29 | 25 |
| RBM34 | P42696 | 2.26E-06 | 6.33E-07 | -0.254187 | + | 1.41318 | -2.63845 | | 14.7963 | 14.6992 | 14.7575 | 14.8275 | 15.2126 | 14.9815 | 15.1127 | 14.8915 | 6 | 6 | 6 | 6 | 5 | 5 | 6 | 6 |
| TWSG1 | Q9GZX9 | 2.72E-17 | 7.55E-17 | -0.253963 | | 0.558433 | -1.19709 | | 16.0649 | 16.0094 | 16.0376 | 16.8014 | 16.1746 | 15.8172 | 16.2927 | 16.3703 | 4 | 4 | 4 | 4 | 4 | 4 | 4 | 4 |
| STX6 | O43752 | 2.35E-06 | 6.56E-07 | -0.253509 | | 1.09164 | -2.09552 | | 15.3637 | 15.496 | 15.577 | 15.9921 | 15.8519 | 15.5217 | 15.6242 | 15.6722 | 5 | 4 | 5 | 7 | 4 | 6 | 5 | 5 |
| SPTLC1 | O15269 | 1.39E-18 | 5.82E-18 | -0.253485 | | 1.20063 | -2.27763 | | 16.9582 | 16.9567 | 16.8688 | 17.3771 | 17.3102 | 17.1044 | 16.9116 | 17.2037 | 16 | 16 | 17 | 17 | 16 | 17 | 17 | 17 |
| CTTN | Q14247 | 2.78E-12 | 1.80E-12 | -0.253108 | | 1.14131 | -2.17834 | | 18.8468 | 18.8387 | 18.8146 | 19.2561 | 19.2495 | 18.9749 | 18.8041 | 19.1479 | 30 | 30 | 29 | 31 | 30 | 31 | 29 | 31 |
| SLC4A1AP | Q9BWU0 | 5.79E-09 | 2.17E-09 | -0.252501 | | 1.21327 | -2.29885 | | 14.9361 | 15.0407 | 14.9827 | 15.3051 | 15.2792 | 14.9405 | 15.2438 | 15.4264 | 9 | 10 | 10 | 10 | 9 | 7 | 11 | 9 |
| NBR1 | Q14596;Q1 | 0.000146 | 3.38E-05 | -0.252436 | | 0.367069 | -0.84702 | | 15.6237 | 15.488 | 15.7385 | 16.1877 | 15.085 | 15.6846 | 16.1396 | 16.2491 | 2 | 5 | 3 | 4 | 3 | 4 | 4 | 4 |
| DMXL1 | Q9Y485 | 0.002882 | 0.00054 | -0.252351 | + | 1.77086 | -3.27402 | | 15.8734 | 15.8741 | 15.9663 | 16.159 | 16.0451 | 16.3581 | 16.1557 | 16.0667 | 6 | 12 | 11 | 5 | 2 | 8 | 3 | 3 |
| BPTF | Q12830;Q1 | 0.008115 | 0.001409 | -0.252142 | | 0.831009 | -1.66204 | | 14.7053 | 14.9441 | 14.7048 | 14.6645 | 15.2853 | 15.0842 | 15.1626 | 14.9879 | 4 | 6 | 6 | 4 | 4 | 4 | 6 | 4 |
| ATP6V1C1 | P21283 | 6.15E-24 | 1.55E-22 | -0.251879 | + | 3.07534 | -6.15864 | | 16.459 | 16.498 | 16.4977 | 16.8151 | 16.698 | 16.6825 | 16.8037 | 16.6845 | 17 | 18 | 19 | 18 | 16 | 18 | 19 | 18 |
| SLC30A1 | Q9Y6M5 | 3.31E-15 | 4.85E-15 | -0.25158 | | 0.630605 | -1.32275 | | 15.5124 | 15.6772 | 15.6186 | 16.2808 | 15.5306 | 16.0274 | 15.578 | 15.8548 | 8 | 9 | 9 | 10 | 5 | 10 | 6 | 9 |
| CRYZL1 | O95825 | 1.56E-07 | 4.94E-08 | -0.251538 | + | 1.45572 | -2.71191 | | 14.9022 | 14.9938 | 14.9531 | 14.9894 | 15.2848 | 15.3973 | 15.1662 | 15.1686 | 10 | 10 | 10 | 9 | 7 | 10 | 10 | 8 |
| UNG | P13051;P1 | 2.08E-07 | 6.54E-08 | -0.25127 | | 0.605497 | -1.27932 | | 14.7034 | 14.5226 | 14.5985 | 15.0536 | 14.4019 | 15.2021 | 14.6619 | 14.9776 | 2 | 3 | 4 | 4 | 1 | 3 | 2 | 3 |
| DHRS7 | Q9Y394 | 1.47E-08 | 5.22E-09 | -0.250676 | + | 1.3655 | -2.55665 | | 17.3833 | 17.4076 | 17.3413 | 17.699 | 17.6574 | 17.4759 | 17.8484 | 17.4598 | 12 | 13 | 13 | 12 | 12 | 12 | 12 | 12 |
| ATL1 | Q8WXF7;Q | 1.09E-07 | 3.52E-08 | -0.250439 | | 0.397481 | -0.90484 | | 15.5238 | 15.5093 | 15.4794 | 16.4847 | 15.7221 | 15.735 | 15.196 | 15.6352 | 15 | 15 | 15 | 15 | 13 | 12 | 12 | 15 |
| PDCD2 | Q16342;Q1 | 4.28E-05 | 1.06E-05 | -0.249688 | | 0.300641 | -0.72589 | | 13.706 | 13.7371 | 13.9874 | 14.0102 | NaN | 13.3173 | 14.2336 | 14.6783 | 3 | 4 | 3 | 3 | NaN | 2 | 2 | 2 |
| SMARCD1 | Q96GM5 | 0.000761 | 0.00016 | -0.249029 | | 0.624644 | -1.34193 | | 14.8769 | 14.756 | 14.7897 | 15.0858 | 14.6141 | NaN | 15.3156 | 15.2106 | 4 | 5 | 4 | 6 | 6 | NaN | 4 | 6 |
| GJC1 | P36383 | 1.70E-07 | 5.39E-08 | -0.248699 | | 0.218848 | -0.54692 | | 15.4525 | 15.3258 | 15.021 | 16.2175 | 15.5648 | 14.3192 | 15.4181 | 16.0559 | 2 | 2 | 2 | 2 | 2 | 2 | 2 | 2 |
| KCMF1 | Q9P0J7 | 7.44E-12 | 4.44E-12 | -0.248582 | + | 1.34461 | -2.52099 | | 15.883 | 15.9456 | 15.8934 | 16.2174 | 15.9611 | 16.058 | 16.3921 | 16.1508 | 5 | 4 | 4 | 4 | 4 | 5 | 4 | 5 |
| PLD2 | O14939;O1 | 0.028722 | 0.004727 | -0.248245 | | 0.757748 | -1.53918 | | 12.7629 | 13.1125 | 13.2382 | 13.2383 | 13.6394 | 13.1248 | 13.2766 | 13.1515 | 3 | 3 | 3 | 3 | 3 | 2 | 3 | 3 |
| BRD2 | P25440;P2 | 0.002198 | 0.000427 | -0.24795 | | 0.453023 | -1.00802 | | 14.3761 | 14.2364 | 14.1158 | 14.5704 | 14.5813 | 13.7898 | 14.7494 | 14.7628 | 6 | 5 | 4 | 6 | 3 | 2 | 5 | 3 |
| DYNC1I2 | Q13409-2; | 6.30E-22 | 7.95E-21 | -0.247925 | + | 2.51592 | -4.78458 | | 17.4619 | 17.4596 | 17.45 | 17.7147 | 17.8188 | 17.5791 | 17.7311 | 17.6816 | 25 | 24 | 25 | 27 | 25 | 24 | 26 | 25 |
| CRABP2 | P29373 | 9.65E-07 | 2.81E-07 | -0.247882 | | 0.249137 | -0.61125 | | 17.9238 | 17.9795 | 17.9331 | 17.4142 | 17.7207 | 19.1021 | 18.623 | 18.1067 | 14 | 14 | 15 | 14 | 13 | 14 | 14 | 14 |
| SMYD2 | Q9NRG4 | 2.09E-09 | 8.25E-10 | -0.247739 | | 0.514206 | -1.11869 | | 14.1368 | 13.9359 | 13.6177 | 14.6773 | 14.0031 | 14.0449 | 13.8278 | 14.1693 | 3 | 5 | 3 | 6 | 4 | 4 | 4 | 4 |
| FBN1 | P35555 | 2.72E-19 | 1.48E-18 | -0.247097 | | 0.381441 | -0.87447 | | 15.9716 | 15.9614 | 15.9436 | 15.5528 | 16.0131 | 16.5846 | 16.7409 | 16.1384 | 54 | 51 | 55 | 51 | 51 | 57 | 55 | 58 |
| UTP6 | Q9NYH9 | 2.49E-07 | 7.75E-08 | -0.247045 | + | 1.91247 | -3.53905 | | 14.4907 | 14.6148 | 14.4999 | 14.8078 | 14.9222 | 14.6325 | 14.8095 | 14.7391 | 14 | 12 | 13 | 15 | 8 | 12 | 13 | 12 |
| PCYOX1 | Q9UHG3 | 4.08E-15 | 5.82E-15 | -0.247026 | | 1.06737 | -2.05513 | | 18.6527 | 18.6538 | 18.5955 | 18.5562 | 19.0329 | 18.8943 | 19.0579 | 18.8638 | 19 | 19 | 20 | 19 | 20 | 19 | 20 | 20 |
| SBNO1 | A3KN83;A3 | 0.004619 | 0.00083 | -0.246888 | | 0.595425 | -1.28888 | | 14.8047 | 14.8859 | 14.8307 | 15.0689 | 14.6405 | NaN | 15.2818 | 15.358 | 2 | 3 | 3 | 3 | 2 | NaN | 7 | 2 |
| METAP2 | P50579-2 | 0.02117 | 0.003517 | -0.246872 | | 0.460048 | -1.02087 | | 12.7642 | 12.3504 | 12.234 | 12.2873 | 12.7422 | 12.3976 | 13.1385 | 12.9164 | 2 | 2 | 2 | 1 | 2 | 2 | 2 | 1 |
| LGALS8 | O00214;O0 | 5.71E-12 | 3.48E-12 | -0.246743 | + | 1.35361 | -2.53636 | | 15.6131 | 15.6733 | 15.5623 | 16.0863 | 15.7252 | 15.6977 | 15.9245 | 15.8812 | 6 | 7 | 6 | 8 | 5 | 8 | 6 | 7 |
| MPP6 | Q9NZW5 | 1.89E-05 | 4.82E-06 | -0.246727 | | 0.724025 | -1.48225 | | 14.9688 | 14.9953 | 14.9922 | 15.0647 | 14.9455 | 15.6528 | 15.3573 | 15.1404 | 11 | 13 | 14 | 13 | 9 | 13 | 14 | 11 |
| TAF7 | Q15545 | 3.47E-06 | 9.56E-07 | -0.246496 | | 0.852608 | -1.6981 | | 14.4554 | 14.8099 | 14.6399 | 15.252 | 14.7996 | 14.7472 | 14.8166 | 14.7924 | 5 | 5 | 4 | 6 | 4 | 4 | 7 | 6 |
| CDV3 | Q9UKY7 | 3.05E-08 | 1.05E-08 | -0.246472 | | 0.502409 | -1.09756 | | 17.1555 | 16.9857 | 17.3928 | 17.9214 | 17.0327 | 17.6122 | 17.329 | 17.2271 | 11 | 12 | 13 | 13 | 9 | 12 | 12 | 13 |
| TCP11L1 | Q9NUJ3 | 3.84E-09 | 1.47E-09 | -0.246329 | + | 1.89267 | -3.50147 | | 16.5557 | 16.5076 | 16.4666 | 16.5938 | 16.7496 | 16.7277 | 16.8058 | 16.9046 | 8 | 9 | 10 | 8 | 8 | 10 | 13 | 10 |
| TOR1AIP2 | Q8NFQ8 | 7.83E-18 | 2.60E-17 | -0.24625 | + | 1.75362 | -3.24233 | | 15.6685 | 15.6847 | 15.9008 | 16.078 | 15.9764 | 15.8598 | 16.0031 | 16.0706 | 13 | 12 | 13 | 15 | 11 | 14 | 14 | 13 |
| SYNC | Q9H7C4-2 | 2.11E-12 | 1.41E-12 | -0.246142 | + | 1.99444 | -3.69663 | | 16.8712 | 16.765 | 16.8304 | 17.2023 | 17.0606 | 17.0471 | 16.9154 | 17.1163 | 16 | 19 | 16 | 19 | 16 | 16 | 17 | 16 |
| TENT5A | Q96IP4;Q9 | 2.31E-06 | 6.47E-07 | -0.245394 | | 0.198691 | -0.50304 | | 14.479 | 14.2728 | 14.3385 | 13.5639 | 14.5048 | 14.6679 | 15.8447 | 14.4629 | 3 | 4 | 4 | 3 | 3 | 3 | 4 | 4 |
| CEP250 | Q9BV73;Q9 | 0.005361 | 0.000953 | -0.245304 | | 1.1911 | -2.26165 | | 13.9393 | 13.6463 | 13.9097 | 14.0913 | 13.8841 | 13.9903 | 14.201 | 14.2184 | 9 | 6 | 5 | 9 | 5 | 4 | 3 | 5 |
| ARMCX1 | Q9P291 | 1.09E-09 | 4.50E-10 | -0.245267 | | 0.827602 | -1.65635 | | 16.008 | 16.0073 | 15.9337 | 15.9106 | 16.1849 | 16.2518 | 16.6004 | 16.1936 | 13 | 13 | 14 | 13 | 13 | 12 | 13 | 13 |
| CBWD2 | Q8IUF1 | 0.001233 | 0.000251 | -0.245077 | | 0.722798 | -1.48017 | | 14.6484 | 14.4051 | 14.4103 | 14.434 | 15.0563 | 14.6915 | 14.5493 | 14.934 | 6 | 8 | 5 | 6 | 4 | 7 | 6 | 4 |
| HAS2 | Q92819 | 0.000113 | 2.66E-05 | -0.245042 | | 0.183069 | -0.46839 | | 14.6994 | 14.7632 | 14.8532 | 16.2577 | 15.2342 | 14.1355 | 14.2103 | 15.2471 | 5 | 5 | 3 | 8 | 3 | 1 | 2 | 4 |
| PLIN3 | O60664 | 3.91E-09 | 1.50E-09 | -0.244619 | + | 1.88565 | -3.48817 | | 20.0343 | 20.0671 | 20.0231 | 20.2742 | 20.4898 | 20.2291 | 20.2064 | 20.2309 | 21 | 22 | 22 | 23 | 22 | 22 | 22 | 22 |
| SGMS1 | Q86VZ5 | 0.002302 | 0.000444 | -0.244587 | | 0.321367 | -0.75806 | | 14.3795 | 14.1547 | 14.1861 | 15.1968 | 13.7618 | 14.3382 | 14.3658 | 14.7609 | 3 | 2 | 3 | 4 | 3 | 4 | 3 | 3 |
| PHKG2 | P15735;P1 | 3.22E-06 | 8.88E-07 | -0.244552 | | 1.07995 | -2.07607 | | 14.326 | 14.4525 | 14.6763 | 14.5769 | 14.8103 | 14.7037 | 14.6081 | 14.9485 | 2 | 3 | 3 | 2 | 2 | 2 | 3 | 3 |
| CTU2 | Q2VPK5 | 0.001296 | 0.000263 | -0.244549 | | 0.839274 | -1.67585 | | 13.9106 | 14.4233 | 14.1777 | 14.3329 | 14.1983 | 14.4931 | 14.6348 | 14.4163 | 5 | 4 | 5 | 3 | 3 | 4 | 3 | 4 |
| SEPTIN8 | Q92599;Q9 | 2.56E-24 | 7.58E-23 | -0.244548 | | 1.10692 | -2.12098 | | 15.7754 | 15.7761 | 15.7593 | 16.2643 | 15.9659 | 15.7833 | 15.9065 | 16.1541 | 8 | 8 | 8 | 8 | 7 | 8 | 8 | 8 |
| CCDC86 | Q9H6F5 | 0.000393 | 8.59E-05 | -0.244521 | + | 1.57999 | -2.92953 | | 14.8795 | 14.8277 | 14.7401 | 15.2242 | 15.0754 | 14.8665 | 15.0247 | 15.1107 | 6 | 5 | 6 | 6 | 5 | 4 | 6 | 6 |
| PAK1 | Q13153 | 5.34E-11 | 2.70E-11 | -0.244481 | | 0.868514 | -1.72461 | | 16.5891 | 16.6167 | 16.6397 | 17.0648 | 17.0644 | 16.5959 | 16.6126 | 16.9606 | 11 | 14 | 11 | 11 | 12 | 11 | 12 | 11 |
| PLIN2 | Q99541 | 0.000238 | 5.36E-05 | -0.244457 | | 0.220195 | -0.55582 | | 13.9601 | 14.3508 | 14.2727 | 14.8056 | NaN | 13.4284 | 15.0846 | 14.4374 | 1 | 4 | 1 | 6 | NaN | 4 | 4 | 5 |
| PID1 | Q7Z2X4;Q7 | 1.85E-05 | 4.73E-06 | -0.244109 | | 0.281361 | -0.6778 | | 13.4672 | 13.596 | 13.5672 | 14.6279 | 13.1147 | 13.5516 | 14.1634 | 13.4804 | 5 | 5 | 5 | 4 | 1 | 4 | 4 | 3 |
| TFDP1 | Q14186 | 0.002901 | 0.000543 | -0.243908 | | 0.620771 | -1.30577 | | 14.4748 | 14.693 | 14.606 | 15.1032 | 14.8258 | 14.6495 | 14.4385 | 15.1587 | 1 | 1 | 2 | 1 | 2 | 1 | 1 | 1 |
| KCTD21 | Q4G0X4 | 0.002747 | 0.000518 | -0.243898 | | 0.189772 | -0.48333 | | 15.5566 | 15.647 | 15.1021 | 15.5768 | 14.4763 | 15.8189 | 16.7806 | 15.7431 | 2 | 1 | 3 | 4 | 2 | 2 | 3 | 2 |
| HSPE1 | P61604 | 4.35E-13 | 3.47E-13 | -0.243773 | + | 1.79113 | -3.31144 | | 19.8648 | 19.7659 | 19.8883 | 20.2289 | 20.0938 | 19.9467 | 19.9953 | 20.1526 | 10 | 10 | 10 | 10 | 10 | 10 | 10 | 10 |
| DUS3L | Q96G46 | 6.62E-10 | 2.80E-10 | -0.243744 | + | 1.77108 | -3.27441 | | 14.2847 | 14.304 | 14.2168 | 14.3388 | 14.6514 | 14.4734 | 14.5015 | 14.5963 | 7 | 8 | 9 | 8 | 6 | 7 | 8 | 5 |
| RNF146 | Q9NTX7;Q9 | 8.15E-11 | 3.96E-11 | -0.243375 | | 0.798025 | -1.60685 | | 13.4896 | 13.5302 | 13.5304 | 13.6502 | 14.1292 | 13.4467 | 13.7201 | 13.8544 | 3 | 4 | 3 | 3 | 1 | 3 | 3 | 3 |
| NADK | O95544 | 1.75E-05 | 4.49E-06 | -0.243344 | | 0.533676 | -1.15335 | | 15.4967 | 15.2261 | 15.1715 | 15.7141 | 15.6178 | 15.591 | 14.9694 | 15.815 | 3 | 4 | 4 | 4 | 4 | 2 | 4 | 4 |
| HACD3 | Q9P035 | 2.26E-11 | 1.23E-11 | -0.242953 | + | 1.40757 | -2.6288 | | 17.9063 | 17.8525 | 17.8025 | 17.9662 | 18.3435 | 17.9936 | 18.0583 | 18.1221 | 11 | 11 | 11 | 11 | 11 | 11 | 11 | 11 |
| PACSIN2 | Q9UNF0;Q9 | 1.95E-15 | 3.07E-15 | -0.242934 | | 0.337243 | -0.78927 | | 17.1477 | 17.2116 | 17.1301 | 18.2301 | 17.3582 | 17.2171 | 16.8186 | 17.4063 | 16 | 16 | 16 | 16 | 14 | 16 | 16 | 16 |
| SCYL2 | Q6P3W7 | 5.04E-15 | 6.95E-15 | -0.242861 | + | 1.47315 | -2.74215 | | 15.4585 | 15.5265 | 15.4925 | 15.9251 | 15.7692 | 15.52 | 15.7659 | 15.6967 | 13 | 15 | 14 | 14 | 14 | 13 | 15 | 13 |
| HSP90AB4P | Q58FF6 | 0.000569 | 0.000121 | -0.242754 | | 0.531237 | -1.14902 | | 18.2337 | 18.4947 | 18.6178 | 19.2134 | 18.3244 | 18.5686 | 18.7101 | 18.6408 | 2 | 3 | 3 | 2 | 3 | 2 | 2 | 2 |
| MGRN1 | O60291;O6 | 4.84E-06 | 1.31E-06 | -0.242716 | | 1.07783 | -2.07253 | | 15.2043 | 15.1381 | 15.138 | 15.605 | 15.1541 | 15.2604 | 15.4249 | 15.5697 | 10 | 10 | 11 | 11 | 8 | 9 | 10 | 11 |
| CSDE1 | O75534;O7 | 1.03E-17 | 3.30E-17 | -0.242579 | + | 1.50671 | -2.80063 | | 16.953 | 16.9485 | 16.9403 | 17.2675 | 17.1522 | 16.9577 | 17.3335 | 17.2382 | 37 | 40 | 36 | 39 | 36 | 38 | 40 | 39 |
| CAPZA1 | P52907 | 1.05E-10 | 5.02E-11 | -0.242408 | + | 3.78769 | -8.32479 | | 19.5796 | 19.5699 | 19.5976 | 19.8551 | 19.8788 | 19.7821 | 19.7678 | 19.8401 | 13 | 13 | 13 | 13 | 13 | 13 | 13 | 13 |
| PLEKHA1 | Q9HB21;Q9 | 0.000686 | 0.000145 | -0.242311 | | 0.994682 | -1.93435 | | 13.8983 | 13.7043 | 13.4881 | 13.6965 | 14.0813 | 13.9972 | 13.8938 | 14.0274 | 2 | 2 | 2 | 2 | 3 | 2 | 4 | 2 |
| HGS | O14964 | 5.97E-13 | 4.56E-13 | -0.241787 | + | 3.24207 | -6.61917 | | 17.1578 | 17.1723 | 17.2112 | 17.5083 | 17.3957 | 17.3785 | 17.4552 | 17.3734 | 22 | 22 | 22 | 21 | 19 | 20 | 21 | 20 |
| CDC123 | O75794 | 7.20E-08 | 2.36E-08 | -0.241508 | + | 1.36964 | -2.56373 | | 15.2454 | 15.2656 | 15.2529 | 15.7106 | 15.3407 | 15.382 | 15.4372 | 15.6102 | 6 | 6 | 7 | 6 | 5 | 7 | 8 | 6 |
| SPAG9 | O60271 | 2.27E-17 | 6.50E-17 | -0.241328 | | 1.04663 | -2.02065 | | 16.7756 | 16.8287 | 16.8374 | 17.3824 | 16.8846 | 16.9731 | 17.0973 | 16.9388 | 52 | 50 | 53 | 55 | 48 | 51 | 54 | 51 |
| TSC1 | Q92574 | 2.22E-09 | 8.74E-10 | -0.24082 | + | 1.88174 | -3.48078 | | 15.0544 | 14.9031 | 14.9085 | 15.3569 | 15.1888 | 15.0854 | 15.1801 | 15.1696 | 9 | 9 | 9 | 11 | 7 | 9 | 9 | 10 |
| SEC24B | O95487;O9 | 9.87E-11 | 4.73E-11 | -0.240525 | + | 2.80742 | -5.46992 | | 17.2105 | 17.229 | 17.2124 | 17.4897 | 17.4539 | 17.3388 | 17.5364 | 17.4704 | 17 | 18 | 18 | 17 | 18 | 19 | 18 | 18 |
| UPF3A | Q9H1J1 | 0.002461 | 0.000471 | -0.23985 | | 0.329924 | -0.78534 | | 13.9502 | 13.8501 | 13.9629 | 14.6342 | 14.1366 | 13.4535 | 14.4194 | NaN | 2 | 2 | 2 | 2 | 1 | 1 | 1 | NaN |
| AAR2 | Q9Y312 | 7.32E-08 | 2.39E-08 | -0.239832 | + | 3.48902 | -7.35116 | | 14.8318 | 14.764 | 14.8666 | 15.0976 | 14.9975 | 15.0885 | 15.0454 | 15.0741 | 7 | 5 | 7 | 6 | 4 | 7 | 6 | 6 |

| Gene names | UniProt IDs | Protein Group P-value | Protein Group Q-value | Log2 Difference NUP214 vs Controls (n=5) | p<0.05 | -log10 p-value | T-test Test statistic | NPC Localization | Log2 MS2_quantity_NUP214_1 | Log2 MS2_quantity_NUP214_2 | Log2 MS2_quantity_NUP214_3 | Log2 MS2_quantity_Control_1 | Log2 MS2_quantity_Control_2 | Log2 MS2_quantity_Control_3 | Log2 MS2_quantity_Control_4 | Log2 MS2_quantity_Control_5 | Number Peptides for Quantitation NUP214_1 | Number Peptides for Quantitation NUP214_2 | Number Peptides for Quantitation NUP214_3 | Number Peptides for Quantitation Control_1 | Number Peptides for Quantitation Control_2 | Number Peptides for Quantitation Control_3 | Number Peptides for Quantitation Control_4 | Number Peptides for Quantitation Control_5 |
|---|---|---|---|---|---|---|---|---|---|---|---|---|---|---|---|---|---|---|---|---|---|---|---|---|
| TBC1D24 | Q9ULP9;Q | 0.001136 | 0.000233 | -0.239536 | + | 1.97196 | -3.65309 | | 15.4068 | 15.3641 | 15.4538 | 15.4726 | 15.7541 | 15.6898 | 15.6715 | 15.6509 | 12 | 11 | 12 | 11 | 10 | 13 | 13 | 10 |
| RNF5 | Q99942 | 3.41E-11 | 1.78E-11 | -0.239063 | | 0.23331 | -0.57787 | | 14.2351 | 14.0412 | 13.6608 | 14.1275 | 14.9224 | 14.3755 | 13.1512 | 14.5137 | 1 | 1 | 1 | 1 | 2 | 1 | 1 | 1 |
| IKBIP | Q70UQ0 | 1.22E-12 | 8.63E-13 | -0.238971 | | 0.825406 | -1.65268 | | 18.5493 | 18.5861 | 18.568 | 18.6078 | 18.8618 | 18.5439 | 19.1518 | 18.8687 | 20 | 19 | 19 | 19 | 18 | 18 | 19 | 19 |
| ZNF729 | A6NN14 | 0.00295 | 0.000551 | -0.238932 | | 0.376555 | -0.86517 | | 15.022 | 15.1533 | 14.2359 | 14.5494 | 15.2029 | 14.9562 | 15.2281 | 15.2768 | 3 | 3 | 2 | 3 | 2 | 2 | 3 | 2 |
| UNC45A | Q9H3U1 | 3.17E-16 | 6.21E-16 | -0.238333 | | 1.20371 | -2.28281 | | 17.0281 | 17.0137 | 17.0083 | 17.4992 | 17.2639 | 17.0388 | 17.1491 | 17.3241 | 34 | 35 | 35 | 37 | 30 | 34 | 32 | 35 |
| UVRAG | Q9P2Y5 | 0.001056 | 0.000217 | -0.238265 | + | 1.58237 | -2.93375 | | 16.6514 | 16.6916 | 16.5916 | 16.8921 | 17.0967 | 16.8689 | 16.8071 | 16.7509 | 8 | 9 | 7 | 10 | 6 | 7 | 7 | 7 |
| CHUK | O15111 | 1.74E-10 | 8.04E-11 | -0.237775 | + | 1.71384 | -3.16967 | | 15.3509 | 15.3129 | 15.3053 | 15.4659 | 15.7226 | 15.411 | 15.625 | 15.5795 | 14 | 12 | 15 | 13 | 13 | 14 | 13 | 14 |
| DR1 | Q01658 | 1.25E-07 | 3.98E-08 | -0.23727 | | 0.327576 | -0.7703 | | 16.7809 | 16.4942 | 17.4136 | 17.2246 | 17.0689 | 16.497 | 17.3357 | 17.5413 | 6 | 5 | 6 | 5 | 6 | 5 | 6 | 6 |
| NISCH | Q9Y2I1 | 4.20E-13 | 3.37E-13 | -0.237087 | + | 1.32949 | -2.49525 | | 15.1478 | 15.1428 | 15.1589 | 15.3908 | 15.5634 | 15.2562 | 15.5233 | 15.2009 | 11 | 12 | 10 | 12 | 12 | 10 | 10 | 9 |
| PELO | Q9BRX2 | 1.58E-12 | 1.08E-12 | -0.236256 | + | 2.8289 | -5.52294 | | 16.8768 | 16.8532 | 16.8026 | 17.1685 | 17.0655 | 17.0165 | 17.1289 | 17.0229 | 14 | 14 | 14 | 14 | 14 | 14 | 14 | 14 |
| PPP2R2A | P63151 | 1.71E-08 | 6.05E-09 | -0.23617 | + | 2.12662 | -3.95775 | | 17.0704 | 17.0706 | 17.0504 | 17.424 | 17.1539 | 17.2726 | 17.3011 | 17.348 | 14 | 14 | 14 | 15 | 13 | 14 | 15 | 14 |
| CSNK1E | P49674 | 1.14E-14 | 1.42E-14 | -0.235901 | | 1.1998 | -2.27623 | | 15.865 | 15.9305 | 15.8305 | 16.2078 | 16.2623 | 15.824 | 16.1419 | 16.1201 | 8 | 10 | 11 | 10 | 9 | 7 | 10 | 9 |
| PEX3 | P56589 | 0.000351 | 7.73E-05 | -0.235794 | | 1.13875 | -2.17407 | | 16.2767 | 16.1583 | 16.3827 | 16.6608 | 16.2406 | 16.5726 | 16.5921 | 16.4759 | 4 | 3 | 4 | 3 | 3 | 4 | 4 | 5 |
| ARHGEF40 | Q8TER5 | 6.07E-15 | 8.16E-15 | -0.235542 | + | 3.59682 | -7.69086 | | 15.5437 | 15.5508 | 15.6008 | 15.839 | 15.8358 | 15.7746 | 15.7316 | 15.8223 | 25 | 23 | 25 | 27 | 21 | 26 | 27 | 26 |
| CDS2 | O95674 | 8.27E-05 | 1.98E-05 | -0.235512 | | 0.966539 | -1.88761 | | 15.0944 | 15.2179 | 15.1384 | 15.5551 | 15.1898 | 15.365 | 15.6316 | 15.1873 | 5 | 6 | 5 | 6 | 2 | 5 | 6 | 6 |
| DNAJB1 | P25685 | 7.65E-08 | 2.50E-08 | -0.235279 | | 1.17706 | -2.23812 | | 17.2087 | 17.22 | 17.1763 | 17.5295 | 17.1727 | 17.3745 | 17.6365 | 17.4716 | 18 | 18 | 16 | 17 | 16 | 17 | 17 | 17 |
| SOGA1 | O94964-2 | 0.003032 | 0.000565 | -0.235131 | | 0.286245 | -0.68773 | | 14.8831 | 14.701 | 14.8343 | 15.4182 | 15.8479 | 14.509 | 14.6483 | 14.7829 | 5 | 6 | 8 | 8 | 5 | 7 | 7 | 9 |
| NPLOC4 | Q8TAT6;Q8 | 5.32E-14 | 5.47E-14 | -0.235043 | + | 2.2197 | -4.14722 | | 16.6031 | 16.533 | 16.6465 | 16.98 | 16.7759 | 16.7963 | 16.7763 | 16.8178 | 20 | 18 | 19 | 19 | 19 | 20 | 19 | 20 |
| HERC1 | Q15751 | 0.002365 | 0.000455 | -0.234991 | | 0.837379 | -1.67268 | | 13.5575 | 13.8837 | 13.7809 | 13.9296 | 13.7699 | 14.0917 | 14.2665 | 13.821 | 8 | 10 | 6 | 9 | 7 | 9 | 7 | 10 |
| ACTB | P60709 | 1.90E-12 | 1.28E-12 | -0.234925 | | 0.481245 | -1.05942 | | 25.4805 | 25.3083 | 25.2554 | 25.268 | 26.0921 | 25.6067 | 25.2069 | 25.7413 | 35 | 35 | 35 | 34 | 35 | 34 | 33 | 35 |
| BTF3 | P20290 | 7.57E-05 | 1.82E-05 | -0.234855 | | 0.971414 | -1.89571 | | 17.5996 | 17.7221 | 17.591 | 18.1341 | 18.0042 | 17.6629 | 17.693 | 17.868 | 7 | 8 | 7 | 6 | 7 | 8 | 7 | 7 |
| STAM2 | O75886 | 5.22E-13 | 4.07E-13 | -0.234835 | + | 2.57808 | -4.92558 | | 16.5818 | 16.6259 | 16.6185 | 16.9168 | 16.9252 | 16.8193 | 16.7375 | 16.8191 | 14 | 13 | 13 | 14 | 12 | 13 | 13 | 12 |
| ARHGAP35 | Q9NRY4 | 2.88E-15 | 4.32E-15 | -0.234615 | + | 1.30132 | -2.4474 | | 15.6729 | 15.7435 | 15.7062 | 16.1571 | 15.925 | 15.7108 | 15.9461 | 15.9716 | 37 | 38 | 38 | 38 | 31 | 33 | 37 | 36 |
| ABRAXAS2 | Q15018 | 2.79E-09 | 1.09E-09 | -0.234591 | | 1.12067 | -2.1439 | | 15.7319 | 15.755 | 15.7168 | 15.6818 | 16.0531 | 16.1163 | 16.1 | 15.8946 | 11 | 11 | 11 | 10 | 11 | 10 | 12 | 9 |
| SRPK1 | Q96SB4;Q9 | 6.67E-11 | 3.31E-11 | -0.234203 | | 0.85939 | -1.70941 | | 15.1717 | 15.1697 | 15.2288 | 15.597 | 15.5151 | 15.0604 | 15.3451 | 15.6036 | 7 | 9 | 8 | 8 | 8 | 8 | 8 | 8 |
| KIF7 | Q2M1P5 | 6.40E-10 | 2.71E-10 | -0.234162 | | 0.48972 | -1.07473 | | 14.6134 | 14.6164 | 14.4355 | 14.4436 | 14.8713 | 14.6235 | 14.6387 | 15.3691 | 7 | 8 | 6 | 8 | 4 | 6 | 5 | 5 |
| PHKB | Q93100-3; | 8.28E-07 | 2.43E-07 | -0.234015 | | 0.86416 | -1.71736 | | 14.9015 | 15.1713 | 15.116 | 14.9826 | 15.2395 | 15.3058 | 15.4868 | 15.4702 | 11 | 11 | 12 | 8 | 12 | 12 | 11 | 10 |
| PTRH2 | Q9Y3E5 | 9.95E-09 | 3.62E-09 | -0.233796 | + | 2.06312 | -3.83117 | | 17.2757 | 17.2706 | 17.3009 | 17.6193 | 17.6023 | 17.3665 | 17.5048 | 17.4881 | 7 | 7 | 7 | 7 | 7 | 7 | 7 | 7 |
| SHFL | Q9NUL5 | 2.32E-06 | 6.49E-07 | -0.233773 | | 1.14675 | -2.18744 | | 15.1299 | 15.0437 | 15.013 | 15.1676 | 15.3295 | 15.4583 | 15.4558 | 15.0687 | 8 | 8 | 9 | 8 | 7 | 7 | 9 | 8 |
| RPS6KA3 | P51812 | 1.30E-11 | 7.40E-12 | -0.233681 | + | 1.47535 | -2.74597 | | 15.8598 | 15.8859 | 15.8423 | 15.9437 | 16.2094 | 16.2611 | 15.965 | 16.1027 | 25 | 27 | 26 | 26 | 23 | 27 | 28 | 26 |
| TPRKB | Q9Y3C4;Q9 | 1.01E-11 | 5.88E-12 | -0.233429 | | 0.503415 | -1.09937 | | 15.2262 | 15.0087 | 15.4791 | 15.347 | 15.507 | 15.2758 | 16.0015 | 15.2257 | 3 | 2 | 4 | 2 | 2 | 2 | 2 | 3 |
| PANK2 | Q9BZ23;Q9 | 1.61E-16 | 3.59E-16 | -0.233302 | + | 1.59226 | -2.95128 | | 14.7789 | 14.7641 | 14.7532 | 14.7724 | 15.0545 | 15.0696 | 15.1017 | 14.9953 | 6 | 7 | 6 | 6 | 5 | 5 | 7 | 6 |
| ARL8B | Q9NVJ2 | 1.96E-07 | 6.17E-08 | -0.232881 | | 1.0787 | -2.07399 | | 18.5441 | 18.5968 | 18.5021 | 18.8089 | 18.5138 | 18.9282 | 18.9664 | 18.6854 | 7 | 7 | 8 | 7 | 7 | 8 | 8 | 8 |
| UBE2Q1 | Q7Z7E8 | 0.001795 | 0.000356 | -0.232778 | + | 1.96122 | -3.63237 | | 14.6042 | 14.5406 | 14.5515 | 14.6869 | 14.8466 | 14.6846 | 14.8686 | 14.9045 | 5 | 4 | 5 | 5 | 5 | 5 | 6 | 4 |
| TMEM245 | Q9H330 | 5.60E-15 | 7.59E-15 | -0.232451 | + | 1.73806 | -3.21384 | | 15.2807 | 15.1017 | 15.1829 | 15.3651 | 15.3741 | 15.3059 | 15.5471 | 15.5122 | 3 | 4 | 5 | 4 | 3 | 3 | 4 | 3 |
| MARK2 | Q7KZI7;Q7 | 2.22E-12 | 1.47E-12 | -0.232226 | + | 1.72465 | -3.18935 | | 14.8715 | 14.9332 | 14.9675 | 15.2291 | 15.0439 | 15.1485 | 15.3138 | 15.0463 | 10 | 10 | 10 | 10 | 7 | 10 | 9 | 8 |
| EIF6 | P56537 | 0.000842 | 0.000175 | -0.232134 | + | 1.64357 | -3.0428 | | 19.1977 | 19.141 | 19.1794 | 19.3852 | 19.6069 | 19.3924 | 19.2567 | 19.3828 | 9 | 9 | 9 | 9 | 7 | 8 | 8 | 8 |
| ARHGDIA | P52565 | 1.37E-13 | 1.26E-13 | -0.231659 | | 0.997649 | -1.93928 | | 20.6591 | 20.6802 | 20.6581 | 21.0199 | 21.1781 | 20.7935 | 20.8218 | 20.674 | 12 | 12 | 12 | 11 | 12 | 12 | 12 | 12 |
| BRAP | Q7Z569 | 1.26E-05 | 3.26E-06 | -0.231646 | | 0.510461 | -1.13274 | | 13.3698 | 14.1475 | 13.6495 | 13.7954 | NaN | 13.9364 | 14.1018 | 13.982 | 3 | 3 | 3 | 3 | NaN | 4 | 3 | 2 |
| EHMT2 | Q96KQ7;Q9 | 0.002526 | 0.000482 | -0.231637 | | 0.706766 | -1.48987 | | 13.6065 | 14.0565 | 13.8495 | 13.917 | 14.0073 | NaN | 14.3433 | 14.009 | 2 | 5 | 4 | 5 | 4 | NaN | 4 | 3 |
| MAPRE1 | Q15691 | 6.63E-13 | 5.00E-13 | -0.231598 | | 0.875285 | -1.7359 | | 17.8511 | 17.8464 | 17.8719 | 18.2653 | 18.2022 | 17.7063 | 18.0803 | 18.1862 | 13 | 13 | 12 | 13 | 12 | 12 | 13 | 13 |
| DVL1 | O14640-2 | 9.49E-10 | 3.94E-10 | -0.231211 | | 1.26836 | -2.39163 | | 14.8884 | 14.7101 | 14.8405 | 15.278 | 15.0793 | 14.9716 | 14.8841 | 15.008 | 5 | 6 | 4 | 9 | 5 | 6 | 4 | 6 |
| CREG1 | O75629 | 9.53E-07 | 2.77E-07 | -0.231179 | | 0.282415 | -0.67994 | | 15.8125 | 15.7032 | 15.1849 | 15.7155 | 15.1778 | 15.6556 | 16.6122 | 15.8291 | 2 | 1 | 2 | 2 | 1 | 2 | 3 | 1 |
| UBL5 | Q9BZL1 | 5.79E-09 | 2.17E-09 | -0.231175 | | 0.625198 | -1.31342 | | 16.7542 | 16.8275 | 16.8879 | 17.2463 | 17.2283 | 16.5465 | 17.0783 | 17.1724 | 4 | 4 | 4 | 4 | 4 | 3 | 4 | 4 |
| PSMB5 | P28074-3 | 1.81E-08 | 6.38E-09 | -0.230714 | + | 1.90274 | -3.52056 | | 19.1309 | 19.1527 | 19.1172 | 19.2357 | 19.2934 | 19.4314 | 19.5106 | 19.3505 | 7 | 7 | 7 | 7 | 7 | 7 | 9 | 7 |
| FKBP4 | Q02790 | 8.17E-21 | 7.57E-20 | -0.230647 | | 0.982841 | -1.91468 | | 17.2374 | 17.3512 | 17.3094 | 17.8185 | 17.3328 | 17.3563 | 17.5489 | 17.5935 | 30 | 32 | 30 | 32 | 26 | 30 | 32 | 31 |
| WDR6 | Q9NNW5 | 2.18E-11 | 1.19E-11 | -0.230594 | + | 2.2179 | -4.14352 | | 15.5643 | 15.6706 | 15.6505 | 15.7757 | 15.9174 | 15.8289 | 15.9752 | 15.798 | 12 | 12 | 12 | 10 | 7 | 8 | 10 | 10 |
| DYNC1LI1 | Q9Y6G9 | 1.84E-13 | 1.63E-13 | -0.230578 | | 1.06509 | -2.05134 | | 17.4827 | 17.5802 | 17.5863 | 17.7685 | 17.9726 | 17.4879 | 17.8931 | 17.7793 | 23 | 22 | 22 | 22 | 21 | 21 | 23 | 22 |
| NF2 | P35240;P3 | 3.30E-13 | 2.72E-13 | -0.230423 | + | 1.63277 | -3.02345 | | 15.6144 | 15.582 | 15.5624 | 15.9822 | 15.7963 | 15.745 | 15.6609 | 15.8989 | 18 | 18 | 18 | 17 | 16 | 18 | 17 | 17 |
| EIF4B | P23588 | 3.51E-10 | 1.54E-10 | -0.230405 | + | 2.13958 | -3.98383 | | 17.2517 | 17.3864 | 17.3958 | 17.6664 | 17.492 | 17.631 | 17.5887 | 17.497 | 19 | 19 | 21 | 20 | 18 | 21 | 20 | 20 |
| LRCH4 | O75427 | 0.000493 | 0.000106 | -0.229922 | | 0.697752 | -1.43769 | | 14.5668 | 14.5414 | 14.5071 | 14.893 | 14.9967 | 14.3517 | 14.6542 | 14.9462 | 8 | 6 | 7 | 7 | 8 | 5 | 8 | 8 |
| PTP4A1 | Q93096 | 0.002356 | 0.000453 | -0.229491 | | 0.186131 | -0.48009 | | 14.027 | 15.5964 | 14.575 | 15.1728 | NaN | 15.5146 | 14.7384 | 14.4234 | 1 | 1 | 3 | 3 | NaN | 2 | 3 | 1 |
| ACTR1A | P61163 | 1.43E-05 | 3.70E-06 | -0.229325 | + | 1.3962 | -2.60925 | | 19.9331 | 19.9881 | 20.0044 | 20.3874 | 20.3161 | 20.0298 | 20.1285 | 20.1609 | 8 | 8 | 9 | 8 | 8 | 8 | 8 | 8 |
| WDR45B | Q5MNZ6 | 0.000381 | 8.33E-05 | -0.228976 | | 0.936175 | -1.83717 | | 14.7565 | 15.0617 | 15.0487 | 15.2811 | 14.9244 | 15.3766 | 15.192 | 15.1489 | 4 | 5 | 4 | 4 | 3 | 4 | 4 | 4 |
| SYNE2 | Q8WXH0;C | 0.004978 | 0.00089 | -0.228768 | | 0.773203 | -1.56519 | | 14.1706 | 13.8984 | 14.1378 | 14.1891 | 14.6243 | 14.0932 | 14.4249 | 14.1571 | 4 | 4 | 5 | 4 | 1 | 3 | 5 | 4 |
| PPIC | P45877 | 0.002705 | 0.000512 | -0.228459 | | 0.312462 | -0.7404 | | 18.893 | 19.045 | 19.0058 | 18.6538 | 18.9987 | 19.752 | 19.7613 | 18.8829 | 3 | 4 | 3 | 3 | 3 | 4 | 3 | 3 |
| HSD17B7 | P56937;P5 | 2.73E-08 | 9.43E-09 | -0.228455 | | 0.388751 | -0.88835 | | 14.8314 | 14.8818 | 14.5945 | 15.4738 | 15.3363 | 14.4765 | 14.7079 | 14.994 | 2 | 4 | 2 | 4 | 2 | 2 | 2 | 3 |
| STXBP1 | P61764 | 3.69E-11 | 1.92E-11 | -0.228413 | | 1.0337 | -1.99917 | | 16.2693 | 16.3687 | 16.3447 | 16.8821 | 16.3992 | 16.5152 | 16.4738 | 16.5098 | 23 | 21 | 24 | 24 | 23 | 23 | 24 | 24 |
| ATP9A | O75110 | 8.35E-05 | 1.99E-05 | -0.228133 | | 0.563104 | -1.2053 | | 15.0337 | 15.0473 | 15.1242 | 15.8255 | 15.0758 | 15.0966 | 15.133 | 15.3517 | 12 | 12 | 11 | 14 | 10 | 11 | 11 | 13 |
| PIN1 | Q13526 | 5.30E-09 | 2.00E-09 | -0.228001 | | 0.808492 | -1.62438 | | 17.0257 | 17.0143 | 16.8875 | 17.307 | 17.5195 | 16.9587 | 17.2275 | 17.0066 | 8 | 8 | 8 | 8 | 8 | 8 | 8 | 8 |
| CLINT1 | Q14677;Q1 | 5.54E-10 | 2.36E-10 | -0.227973 | + | 1.35788 | -2.54364 | | 15.8567 | 15.9126 | 15.9424 | 16.2951 | 15.9225 | 16.0794 | 16.2463 | 16.1161 | 18 | 18 | 18 | 18 | 15 | 19 | 16 | 16 |
| MTHFSD | Q2M296-3, | 0.018258 | 0.00305 | -0.22764 | | 0.190531 | -0.49002 | | 16.0485 | 16.2182 | 15.8715 | 15.7617 | 16.2081 | 17.386 | 15.7389 | NaN | 2 | 2 | 4 | 2 | 1 | 1 | 2 | NaN |
| CAMK2D | Q13557;Q1 | 6.36E-14 | 6.41E-14 | -0.227419 | | 1.25632 | -2.3713 | | 18.5928 | 18.5458 | 18.5851 | 18.9862 | 18.9188 | 18.5758 | 18.7463 | 18.7828 | 15 | 15 | 15 | 14 | 15 | 15 | 13 | 14 |
| PTPN1 | P18031 | 6.73E-13 | 5.06E-13 | -0.227197 | | 1.02294 | -1.98128 | | 16.6077 | 16.6621 | 16.6838 | 17.1274 | 16.6488 | 16.8131 | 17.0128 | 16.79 | 19 | 19 | 19 | 18 | 16 | 18 | 19 | 19 |
| NOP53 | Q9NZM5 | 1.05E-06 | 3.04E-07 | -0.226849 | | 0.971523 | -1.89589 | | 15.164 | 15.2425 | 15.145 | 15.2484 | 15.1757 | 15.4231 | 15.6068 | 15.5993 | 5 | 5 | 6 | 6 | 5 | 6 | 6 | 6 |
| TSG101 | Q99816 | 3.31E-09 | 1.28E-09 | -0.226825 | + | 1.77457 | -3.28086 | | 16.9448 | 16.9909 | 17.0046 | 17.2875 | 17.0411 | 17.1371 | 17.3059 | 17.263 | 14 | 12 | 14 | 15 | 12 | 13 | 15 | 13 |
| AP3S1 | Q92572 | 3.24E-10 | 1.43E-10 | -0.226704 | | 1.03254 | -1.99724 | | 17.8531 | 17.6011 | 17.8959 | 17.9417 | 18.1352 | 17.7722 | 18.1217 | 18.0797 | 8 | 9 | 9 | 9 | 8 | 8 | 9 | 9 |
| CDK16 | Q00536;Q0 | 3.84E-21 | 3.93E-20 | -0.226681 | + | 2.65599 | -5.10615 | | 15.3291 | 15.1712 | 15.2125 | 15.4416 | 15.5244 | 15.4136 | 15.4396 | 15.5023 | 9 | 10 | 9 | 6 | 8 | 10 | 8 | 10 |
| VAT1L | Q9HCJ6 | 1.25E-10 | 5.93E-11 | -0.226673 | | 0.172979 | -0.44568 | | 15.1552 | 15.1817 | 15.1083 | 16.821 | 14.7713 | 14.9302 | 15.4723 | 14.8804 | 10 | 11 | 11 | 15 | 8 | 10 | 11 | 11 |
| DUT | P33316 | 3.51E-10 | 1.54E-10 | -0.226418 | | 0.447823 | -0.99848 | | 18.185 | 18.1952 | 18.239 | 18.8481 | 18.1156 | 17.9725 | 18.4995 | 18.7283 | 9 | 8 | 8 | 8 | 8 | 7 | 8 | 8 |
| IKBIP | Q70UQ0-4 | 2.14E-10 | 9.77E-11 | -0.226298 | | 0.772792 | -1.56449 | | 17.6686 | 17.6952 | 17.6756 | 17.7242 | 17.8706 | 17.7126 | 18.31 | 17.9129 | 20 | 20 | 19 | 19 | 18 | 19 | 21 | 18 |
| EIF3L | Q9Y262 | 7.04E-11 | 3.47E-11 | -0.225771 | + | 1.3795 | -2.58061 | | 18.3339 | 18.3972 | 18.3485 | 18.6363 | 18.709 | 18.3346 | 18.6151 | 18.633 | 25 | 25 | 25 | 25 | 25 | 24 | 25 | 26 |
| NEU1 | Q99519 | 3.31E-11 | 1.74E-11 | -0.225367 | + | 1.50745 | -2.80193 | | 16.5147 | 16.5864 | 16.6277 | 16.714 | 16.8755 | 16.6794 | 16.9899 | 16.7493 | 9 | 9 | 9 | 9 | 7 | 8 | 9 | 9 |
| TRMT1 | Q9NXH9;Q | 1.10E-06 | 3.19E-07 | -0.22526 | | 0.947922 | -1.85669 | | 15.3662 | 15.4601 | 15.4197 | 15.9546 | 15.694 | 15.4215 | 15.5852 | 15.5476 | 8 | 9 | 8 | 11 | 7 | 10 | 10 | 9 |
| PI4K2B | Q8TCG2 | 0.000161 | 3.71E-05 | -0.224926 | + | 2.94509 | -5.81628 | | 14.8915 | 14.9094 | 14.8013 | 15.1072 | 15.1134 | 15.0268 | 15.1557 | 15.0586 | 5 | 7 | 6 | 6 | 3 | 7 | 6 | 5 |
| CD248 | Q9HCU0 | 1.57E-06 | 4.47E-07 | -0.224773 | | 0.646652 | -1.35037 | | 16.3247 | 16.2817 | 16.2936 | 16.0803 | 16.6845 | 16.4446 | 16.7927 | 16.6218 | 11 | 11 | 12 | 11 | 12 | 11 | 11 | 12 |
| SOD1 | P00441 | 8.11E-09 | 2.98E-09 | -0.224741 | | 0.331148 | -0.77733 | | 20.3893 | 20.1206 | 20.1552 | 19.8615 | 21.1764 | 20.2985 | 20.4287 | 20.4672 | 9 | 9 | 9 | 8 | 9 | 9 | 9 | 9 |
| NSMCE2 | Q96MF7 | 4.41E-06 | 1.20E-06 | -0.224641 | | 0.423424 | -0.96866 | | 13.7805 | 13.9644 | 14.0269 | NaN | 14.6969 | 13.8717 | 13.9292 | 14.0964 | 2 | 2 | 2 | NaN | 1 | 3 | 2 | 2 |

| Gene names | UniProt IDs | Protein Group P-value | Protein Group Q-value | Log2 Difference NUP214 vs Controls (n=5) | p<0.05 | -log10 p-value | T-test Test statistic | NPC Localization | Log2 MS2_quantity_ NUP214_1 | Log2 MS2_quantity_ NUP214_2 | Log2 MS2_quantity_ NUP214_3 | Log2 MS2_quantity_ Control_1 | Log2 MS2_quantity_ Control_2 | Log2 MS2_quantity_ Control_3 | Log2 MS2_quantity_ Control_4 | Log2 MS2_quantity_ Control_5 | Number Peptides for Quantitation NUP214_1 | Number Peptides for Quantitation NUP214_2 | Number Peptides for Quantitation NUP214_3 | Number Peptides for Quantitation Control_1 | Number Peptides for Quantitation Control_2 | Number Peptides for Quantitation Control_3 | Number Peptides for Quantitation Control_4 | Number Peptides for Quantitation Control_5 |
|---|---|---|---|---|---|---|---|---|---|---|---|---|---|---|---|---|---|---|---|---|---|---|---|---|
| POP1 | Q99575 | 1.81E-16 | 3.97E-16 | -0.224593 | | 0.598923 | -1.2679 | | 15.0224 | 14.9524 | 14.9481 | 15.6274 | 15.1822 | 14.9124 | 14.9418 | 15.3308 | 18 | 17 | 18 | 18 | 14 | 16 | 14 | 16 |
| VDAC2 | P45880 | 1.02E-15 | 1.74E-15 | -0.224174 | + | 1.80138 | -3.33042 | | 19.6217 | 19.6438 | 19.641 | 19.7695 | 20.05 | 19.783 | 19.8567 | 19.8392 | 13 | 14 | 14 | 14 | 14 | 14 | 13 | 13 |
| IRAK4 | Q9NWZ3 | 1.99E-07 | 6.25E-08 | -0.224143 | | 1.1269 | -2.15429 | | 16.8678 | 16.9422 | 16.8613 | 17.3522 | 17.1696 | 16.9965 | 16.9067 | 17.148 | 8 | 9 | 10 | 10 | 8 | 8 | 6 | 8 |
| RAB5A | P20339;P2 | 2.65E-05 | 6.65E-06 | -0.224093 | | 1.14323 | -2.18156 | | 18.7544 | 18.8846 | 18.7724 | 18.8552 | 19.1509 | 19.0346 | 19.2267 | 18.8721 | 4 | 4 | 5 | 5 | 5 | 4 | 4 | 4 |
| PPP3R1 | P63098 | 3.86E-14 | 4.12E-14 | -0.22395 | | 0.303566 | -0.72264 | | 15.035 | 14.7768 | 14.8789 | 14.2153 | 15.2577 | 15.4161 | 15.4235 | 15.2917 | 5 | 5 | 6 | 4 | 5 | 6 | 6 | 6 |
| GDAP2 | Q9NXN4;Q | 2.58E-07 | 8.03E-08 | -0.223929 | | 0.884006 | -1.75042 | | 14.7906 | 14.7527 | 14.756 | 14.7748 | 14.9817 | 15.037 | 15.3228 | 14.8355 | 6 | 6 | 6 | 6 | 6 | 7 | 7 | 5 |
| ALG13 | Q9NP73-2 | 0.000262 | 5.87E-05 | -0.223826 | | 0.947305 | -1.85566 | | 15.8526 | 15.9019 | 16.0448 | 16.0156 | 16.0042 | 16.1094 | 16.4689 | 16.1866 | 6 | 4 | 4 | 5 | 5 | 5 | 4 | 5 |
| MPP1 | Q00013;Q | 1.86E-12 | 1.26E-12 | -0.22368 | | 0.831856 | -1.71356 | | 17.0911 | 16.9792 | 16.9254 | 16.9574 | NaN | 17.2924 | 17.1823 | 17.4569 | 5 | 5 | 4 | 4 | NaN | 5 | 3 | 2 |
| PNPLA6 | Q8IY17;Q8 | 1.38E-09 | 5.62E-10 | -0.223642 | + | 1.64303 | -3.04183 | | 16.2081 | 16.2357 | 16.1895 | 16.3744 | 16.5393 | 16.4879 | 16.5232 | 16.249 | 21 | 23 | 22 | 26 | 20 | 24 | 24 | 23 |
| STRN4 | Q9NRL3;Q | 1.32E-22 | 2.06E-21 | -0.223642 | + | 2.83799 | -5.5455 | | 15.787 | 15.7801 | 15.8161 | 15.9319 | 16.0166 | 16.0247 | 16.117 | 16 | 19 | 17 | 17 | 16 | 16 | 17 | 18 | 16 |
| GLS | O94925 | 1.03E-12 | 7.42E-13 | -0.223132 | | 0.754722 | -1.53409 | | 18.3805 | 18.4234 | 18.3817 | 18.6016 | 18.3066 | 18.9216 | 18.7843 | 18.4775 | 29 | 29 | 28 | 28 | 28 | 28 | 29 | 29 |
| LIMA1 | Q9UHB6 | 2.12E-14 | 2.44E-14 | -0.22298 | | 0.536664 | -1.15865 | | 18.0603 | 18.0381 | 18.0788 | 18.1597 | 18.5398 | 18.5508 | 17.7795 | 18.3804 | 44 | 45 | 44 | 44 | 41 | 44 | 44 | 45 |
| FDX1 | P10109 | 0.009392 | 0.001617 | -0.222925 | | 0.992058 | -1.92999 | | 15.8543 | 15.5774 | 15.647 | 15.6967 | 16.134 | 15.8239 | 15.9517 | 15.9728 | 2 | 2 | 2 | 2 | 1 | 3 | 2 | 2 |
| GOLIM4 | O00461 | 1.10E-11 | 6.32E-12 | -0.222876 | + | 1.35863 | -2.54492 | | 15.6056 | 15.7211 | 15.8006 | 16.112 | 15.8212 | 15.9023 | 15.8104 | 16.0139 | 21 | 20 | 21 | 21 | 18 | 20 | 21 | 21 |
| ABHD17B | Q5VST6;Q5 | 2.00E-18 | 7.97E-18 | -0.22261 | | 0.438565 | -0.98144 | | 14.2499 | 14.3643 | 14.3115 | 15.1444 | 14.5642 | 14.1231 | 14.4203 | 14.4039 | 5 | 5 | 6 | 7 | 3 | 5 | 5 | 5 |
| RELCH | Q9P260-2 | 7.48E-12 | 4.45E-12 | -0.222415 | + | 1.78569 | -3.30137 | | 16.2174 | 16.2852 | 16.3115 | 16.3804 | 16.5405 | 16.4351 | 16.6564 | 16.4564 | 3 | 3 | 3 | 3 | 3 | 3 | 3 | 3 |
| SYNM | O15061 | 0.003147 | 0.000584 | -0.222226 | | 0.809055 | -1.62532 | | 14.5829 | 14.7626 | 14.7642 | 15.0678 | 15.0897 | 15.093 | 14.6672 | 14.7097 | 11 | 10 | 10 | 10 | 11 | 11 | 9 | 11 |
| SSR4 | P51571 | 3.90E-07 | 1.19E-07 | -0.221801 | + | 1.37199 | -2.56776 | | 19.8508 | 19.8875 | 19.9165 | 19.9396 | 20.2668 | 20.1754 | 20.1799 | 19.9721 | 6 | 6 | 6 | 6 | 6 | 6 | 6 | 6 |
| LDHA | P00338 | 8.60E-22 | 1.03E-20 | -0.221243 | | 0.840125 | -1.67727 | | 22.033 | 22.084 | 22.0842 | 22.4232 | 22.5448 | 22.1571 | 21.9866 | 22.3299 | 29 | 30 | 30 | 31 | 31 | 30 | 30 | 30 |
| SSR1 | P43307 | 1.83E-12 | 1.24E-12 | -0.221223 | | 1.11078 | -2.12742 | | 20.2828 | 20.3329 | 20.3616 | 20.3514 | 20.5298 | 20.7467 | 20.6948 | 20.4122 | 6 | 6 | 6 | 4 | 4 | 6 | 5 | 5 |
| AKAP10 | O43572 | 0.00035 | 7.70E-05 | -0.221114 | + | 2.02738 | -3.76086 | | 14.8824 | 14.9711 | 15.066 | 15.1222 | 15.1892 | 15.2646 | 15.2747 | 15.1208 | 7 | 7 | 7 | 7 | 5 | 5 | 6 | 5 |
| SPCS3 | P61009 | 7.74E-09 | 2.85E-09 | -0.220926 | + | 1.79545 | -3.31943 | | 18.294 | 18.3207 | 18.3147 | 18.4967 | 18.426 | 18.443 | 18.6887 | 18.5991 | 5 | 5 | 5 | 5 | 5 | 5 | 5 | 5 |
| RELCH | Q9P260 | 6.21E-13 | 4.73E-13 | -0.220898 | | 1.22761 | -2.32295 | | 15.6335 | 15.6545 | 15.7659 | 15.7807 | 16.0233 | 15.8923 | 16.0914 | 15.7399 | 25 | 23 | 24 | 24 | 22 | 23 | 20 | 22 |
| CNOT4 | O95628-10 | 1.27E-05 | 3.30E-06 | -0.220833 | | 1.06405 | -2.04961 | | 14.7514 | 14.5557 | 14.5848 | 14.9851 | 14.9179 | 14.5951 | 14.783 | 14.9764 | 7 | 6 | 6 | 7 | 6 | 5 | 5 | 7 |
| PRDX6 | P30041 | 1.01E-08 | 3.66E-09 | -0.220566 | | 0.166724 | -0.43147 | | 21.5063 | 21.4541 | 21.5374 | 20.6601 | 21.9825 | 21.745 | 22.9561 | 21.2556 | 24 | 23 | 25 | 24 | 24 | 24 | 25 | 23 |
| UBE4B | O95155;O9 | 1.39E-10 | 6.51E-11 | -0.220553 | + | 3.00123 | -5.96204 | | 15.6 | 15.6358 | 15.7028 | 15.878 | 15.9056 | 15.7819 | 15.9 | 15.8684 | 25 | 24 | 24 | 24 | 16 | 23 | 21 | 22 |
| UTP20 | O75691 | 1.93E-10 | 8.87E-11 | -0.220409 | + | 2.89986 | -5.70078 | | 14.6387 | 14.7023 | 14.5778 | 14.9387 | 14.8449 | 14.81 | 14.8581 | 14.8482 | 21 | 23 | 24 | 28 | 22 | 25 | 29 | 24 |
| RICTOR | Q6R327;Q6 | 2.37E-15 | 3.65E-15 | -0.220085 | + | 1.42878 | -2.66534 | | 14.5207 | 14.5404 | 14.4492 | 14.6199 | 14.7016 | 14.6353 | 14.9534 | 14.7074 | 12 | 12 | 12 | 11 | 9 | 7 | 14 | 6 |
| STAU1 | O95793;O9 | 1.03E-13 | 9.80E-14 | -0.219957 | + | 2.13147 | -3.9675 | | 16.6525 | 16.6495 | 16.6381 | 16.9324 | 16.9798 | 16.7491 | 16.8073 | 16.8649 | 21 | 21 | 21 | 22 | 15 | 21 | 22 | 19 |
| GAN | Q9H2C0 | 1.89E-11 | 1.04E-11 | -0.219717 | | 0.813428 | -1.63264 | | 14.8035 | 14.7916 | 14.817 | 14.7538 | 15.1637 | 15.2913 | 15.0772 | 14.8327 | 6 | 6 | 5 | 5 | 5 | 5 | 5 | 4 |
| EHMT1 | Q9H9B1 | 1.30E-05 | 3.36E-06 | -0.219522 | | 1.00088 | -1.94464 | | 13.6085 | 13.6895 | 13.4246 | 13.8601 | 13.9167 | 13.5944 | 13.9524 | 13.645 | 3 | 5 | 5 | 5 | 2 | 3 | 4 | 2 |
| REXO4 | Q9GZR2 | 2.61E-05 | 6.55E-06 | -0.219337 | | 0.858833 | -1.70848 | | 14.0422 | 14.3221 | 14.2388 | 14.6738 | 14.5727 | 14.2512 | 14.3058 | 14.2983 | 7 | 9 | 8 | 6 | 6 | 7 | 4 | 5 |
| FASTKD5 | Q7L8L6 | 0.000128 | 2.99E-05 | -0.219138 | | 0.76707 | -1.55487 | | 15.8012 | 15.7315 | 15.585 | 15.8578 | 15.8533 | 15.742 | 15.8578 | 16.3142 | 2 | 2 | 1 | 2 | 1 | 1 | 2 | 2 |
| SHROOM3 | Q8TF72 | 7.23E-12 | 4.34E-12 | -0.219038 | | 0.33173 | -0.77847 | | 14.6686 | 14.6351 | 14.5525 | 14.0092 | 15.0438 | 15.0894 | 14.9172 | 15.1292 | 17 | 16 | 17 | 9 | 9 | 16 | 12 | 15 |
| TRMT5 | Q32P41 | 2.64E-11 | 1.41E-11 | -0.218978 | | 0.933125 | -1.83211 | | 14.4151 | 14.5883 | 14.6763 | 14.7065 | 14.6465 | 14.929 | 14.6079 | 15.0045 | 11 | 8 | 11 | 10 | 4 | 11 | 6 | 6 |
| PAK2 | Q13177 | 1.64E-14 | 1.95E-14 | -0.218418 | | 1.18155 | -2.24565 | | 17.3705 | 17.4872 | 17.4324 | 17.8789 | 17.5781 | 17.5716 | 17.48 | 17.7338 | 21 | 20 | 22 | 20 | 20 | 21 | 21 | 22 |
| NAMPT | P43490 | 3.10E-13 | 2.59E-13 | -0.218083 | | 0.779426 | -1.57564 | | 17.3466 | 17.4052 | 17.3922 | 17.8749 | 17.7094 | 17.2914 | 17.4428 | 17.6787 | 19 | 20 | 20 | 21 | 20 | 20 | 20 | 21 |
| LRRC8A | Q8IWT6 | 9.50E-05 | 2.25E-05 | -0.217783 | | 0.388367 | -0.88762 | | 15.823 | 15.7502 | 15.7459 | 16.4218 | 15.8031 | 15.3792 | 16.1025 | 16.2475 | 9 | 8 | 12 | 12 | 7 | 7 | 8 | 11 |
| CBFB | Q13951-2 | 0.001639 | 0.000327 | -0.217767 | | 0.843954 | -1.68366 | | 16.347 | 16.2644 | 16.084 | 16.6428 | 16.2734 | 16.4486 | 16.2372 | 16.6459 | 1 | 1 | 1 | 1 | 1 | 1 | 1 | 1 |
| ZC3H7A | Q8IWR0 | 8.82E-06 | 2.33E-06 | -0.217657 | + | 1.9238 | -3.56065 | | 14.9985 | 15.0785 | 15.0934 | 15.3645 | 15.3601 | 15.149 | 15.2026 | 15.2962 | 16 | 17 | 17 | 18 | 16 | 16 | 15 | 15 |
| JPT2 | Q9H910;Q9 | 3.87E-14 | 4.13E-14 | -0.217637 | | 0.201258 | -0.50868 | | 17.2468 | 16.3844 | 17.0825 | 17.4313 | 17.1514 | 16.0963 | 17.1091 | 17.8229 | 6 | 6 | 6 | 6 | 6 | 6 | 6 | 6 |
| DHRS7B | Q6IAN0 | 5.90E-13 | 4.52E-13 | -0.217429 | | 0.784542 | -1.58423 | | 15.1211 | 15.0887 | 15.1558 | 15.0524 | 15.2281 | 15.568 | 15.2685 | 15.5794 | 11 | 10 | 11 | 9 | 9 | 11 | 12 | 11 |
| VAPB | O95292 | 8.73E-14 | 8.48E-14 | -0.217199 | + | 2.01696 | -3.74049 | | 16.7143 | 16.743 | 16.7195 | 17.0423 | 16.8577 | 16.8369 | 17.0382 | 16.9388 | 8 | 9 | 8 | 8 | 8 | 8 | 8 | 9 |
| PDE12 | Q6L8Q7 | 1.12E-09 | 4.63E-10 | -0.217054 | + | 1.32466 | -2.48704 | | 16.6626 | 16.6914 | 16.6264 | 16.9485 | 17.0228 | 16.6558 | 16.8153 | 16.9435 | 11 | 12 | 12 | 13 | 11 | 12 | 13 | 12 |
| DDI2 | Q5TDH0;Q5 | 7.12E-11 | 3.50E-11 | -0.216864 | | 0.839861 | -1.67682 | | 15.5156 | 15.4941 | 15.5251 | 15.4972 | 15.6767 | 16.0634 | 15.7973 | 15.6077 | 6 | 7 | 7 | 7 | 6 | 7 | 7 | 6 |
| NEGR1 | Q7Z3B1;Q7 | 0.002029 | 0.000398 | -0.216346 | | 0.198632 | -0.50291 | | 14.8773 | 14.5505 | 14.7999 | 13.8555 | 15.7837 | 15.2528 | 15.0917 | 14.8111 | 1 | 1 | 2 | 1 | 1 | 1 | 1 | 1 |
| DIMT1 | Q9UNQ2 | 3.42E-15 | 4.99E-15 | -0.216325 | + | 1.41695 | -2.64494 | | 15.4059 | 15.4875 | 15.5136 | 15.6634 | 15.6264 | 15.5287 | 15.7255 | 15.8825 | 11 | 11 | 11 | 11 | 10 | 11 | 11 | 10 |
| SLC25A11 | Q02978 | 3.30E-09 | 1.28E-09 | -0.216143 | + | 1.44711 | -2.69701 | | 17.8972 | 17.9855 | 17.9213 | 17.9521 | 18.1522 | 18.2347 | 18.2945 | 18.1207 | 15 | 15 | 15 | 15 | 15 | 15 | 15 | 15 |
| RPS27 | P42677 | 0.000224 | 5.05E-05 | -0.216079 | | 1.26375 | -2.38385 | | 18.3572 | 18.3707 | 18.4344 | 18.8347 | 18.5717 | 18.448 | 18.6503 | 18.5129 | 3 | 3 | 3 | 3 | 3 | 3 | 3 | 3 |
| PSAP | P07602;P0 | 4.65E-12 | 2.87E-12 | -0.216071 | | 0.733608 | -1.49846 | | 16.1915 | 16.0624 | 16.2875 | 16.2202 | 16.2728 | 16.5017 | 16.7509 | 16.2373 | 16 | 14 | 16 | 17 | 14 | 18 | 18 | 16 |
| PDIA4 | P13667 | 1.44E-08 | 5.13E-09 | -0.216029 | + | 1.58176 | -2.93266 | | 19.106 | 19.1301 | 19.0911 | 19.1864 | 19.3913 | 19.2167 | 19.3503 | 19.4808 | 53 | 52 | 52 | 51 | 52 | 53 | 53 | 53 |
| PHF1 | O43189;O4 | 3.94E-10 | 1.72E-10 | -0.215604 | + | 1.71625 | -3.17405 | | 13.9024 | 13.8631 | 13.8247 | 14.1667 | 14.1255 | 13.9936 | 14.1783 | 13.9311 | 2 | 1 | 1 | 2 | 3 | 1 | 2 | 1 |
| PTGR2 | Q8N8N7 | 0.002158 | 0.00042 | -0.215351 | | 0.229705 | -0.57019 | | 15.3424 | 15.5189 | 15.9456 | 15.6973 | 14.8837 | 16.1156 | 16.4705 | 15.9211 | 2 | 3 | 4 | 3 | 2 | 3 | 1 | 2 |
| RIOX2 | Q8IUF8;Q8 | 5.15E-07 | 1.55E-07 | -0.215254 | | 0.736761 | -1.50379 | | 14.1691 | 14.2782 | 14.356 | 14.6012 | 14.1354 | 14.3588 | 14.6552 | 14.6646 | 6 | 5 | 6 | 5 | 3 | 5 | 3 | 6 |
| MITD1 | Q8WV92 | 0.002197 | 0.000426 | -0.215207 | | 0.626139 | -1.34464 | | 14.5865 | 14.7593 | 14.8309 | 14.6517 | NaN | 14.8346 | 15.2251 | 15.0517 | 2 | 2 | 2 | 3 | NaN | 2 | 2 | 2 |
| GIGYF1 | O75420 | 0.000619 | 0.000131 | -0.215114 | | 0.296593 | -0.70864 | | 13.5476 | 13.6296 | 13.6874 | 13.7686 | 14.635 | 13.4546 | 13.364 | 13.961 | 3 | 3 | 2 | 3 | 1 | 1 | 2 | 1 |
| TRAPPC3 | O43617;O4 | 2.24E-05 | 5.68E-06 | -0.215073 | | 0.331116 | -0.77726 | | 17.2287 | 17.0451 | 17.4789 | 17.1692 | 17.6488 | 16.9294 | 17.5216 | 18.061 | 6 | 5 | 5 | 5 | 5 | 5 | 7 | 7 |
| TBL3 | Q12788 | 3.89E-10 | 1.70E-10 | -0.21506 | + | 1.3183 | -2.47622 | | 15.8451 | 15.7514 | 15.8005 | 16.1115 | 16.0969 | 15.7947 | 15.9465 | 16.1209 | 18 | 17 | 16 | 18 | 14 | 15 | 19 | 18 |
| RABGEF1 | Q9UJ41;Q9 | 3.04E-08 | 1.04E-08 | -0.214606 | + | 2.20071 | -4.10817 | | 16.3657 | 16.3747 | 16.4137 | 16.7399 | 16.5844 | 16.5821 | 16.5854 | 16.5048 | 12 | 11 | 12 | 12 | 11 | 12 | 12 | 12 |
| ATP6V1A | P38606 | 2.80E-16 | 5.57E-16 | -0.214216 | + | 1.55995 | -2.89411 | | 18.1164 | 18.1335 | 18.1396 | 18.364 | 18.2149 | 18.3823 | 18.5215 | 18.2376 | 34 | 34 | 34 | 34 | 32 | 34 | 32 | 35 |
| AKT3 | Q9Y243;Q9 | 1.35E-08 | 4.82E-09 | -0.213975 | | 0.95509 | -1.8686 | | 14.9102 | 14.9614 | 14.9029 | 14.8438 | 15.0613 | 15.3134 | 15.2033 | 15.2722 | 7 | 7 | 7 | 5 | 5 | 9 | 7 | 6 |
| CLCN7 | P51798;P5 | 1.95E-19 | 1.10E-18 | -0.213616 | | 0.708825 | -1.45649 | | 16.3606 | 16.4147 | 16.253 | 16.3265 | 16.511 | 16.4242 | 16.9512 | 16.5689 | 10 | 11 | 11 | 11 | 10 | 11 | 11 | 11 |
| CAPN2 | P17655 | 1.54E-12 | 1.06E-12 | -0.213611 | | 0.934678 | -1.83469 | | 19.5788 | 19.6361 | 19.6107 | 19.8267 | 20.0896 | 19.8465 | 19.8058 | 19.5421 | 33 | 33 | 35 | 34 | 33 | 34 | 33 | 32 |
| ARMCX2 | Q7L311 | 5.16E-11 | 2.62E-11 | -0.213557 | | 0.552299 | -1.18629 | | 15.826 | 15.7359 | 15.7825 | 15.6077 | 16.125 | 16.0039 | 16.4045 | 15.8341 | 7 | 7 | 8 | 6 | 6 | 9 | 9 | 8 |
| MAP2K2 | P36507 | 5.76E-19 | 2.75E-18 | -0.213141 | + | 1.3418 | -2.51621 | | 16.8095 | 16.7939 | 16.7152 | 16.804 | 17.1381 | 17.0973 | 16.9882 | 16.9025 | 13 | 16 | 16 | 14 | 14 | 17 | 17 | 14 |
| SZRD1 | Q7Z422;Q7 | 2.10E-15 | 3.27E-15 | -0.213068 | | 0.22195 | -0.5536 | | 15.7772 | 15.5571 | 15.9653 | 16.383 | 16.166 | 14.8782 | 16.0914 | 16.3795 | 3 | 3 | 3 | 3 | 3 | 2 | 3 | 3 |
| RAB7A | P51149 | 1.39E-09 | 5.65E-10 | -0.213043 | | 1.00141 | -1.94553 | | 20.4826 | 20.5354 | 20.5105 | 21.0321 | 20.6092 | 20.5778 | 20.7326 | 20.661 | 19 | 19 | 19 | 19 | 19 | 19 | 19 | 20 |
| CHPF2 | Q9P2E5 | 0.000334 | 7.38E-05 | -0.212942 | | 0.778468 | -1.57403 | | 15.6142 | 15.6784 | 15.6059 | 16.124 | 15.8037 | 15.5048 | 15.868 | 15.9283 | 8 | 5 | 6 | 11 | 8 | 6 | 9 | 11 |
| LYPLA1 | O75608;O7 | 1.51E-05 | 3.90E-06 | -0.212809 | | 1.15888 | -2.20771 | | 17.5093 | 17.22 | 17.4751 | 17.6111 | 17.5587 | 17.5903 | 17.8097 | 17.5016 | 6 | 6 | 6 | 6 | 5 | 6 | 5 | 6 |
| EIF3G | O75821 | 1.23E-08 | 4.40E-09 | -0.212746 | + | 1.44624 | -2.69549 | | 17.5602 | 17.5839 | 17.6422 | 18.0046 | 17.6464 | 17.7726 | 17.8244 | 17.7929 | 14 | 14 | 14 | 16 | 14 | 14 | 15 | 16 |
| EIF4H | Q15056 | 3.65E-08 | 1.24E-08 | -0.212456 | + | 2.25207 | -4.21427 | | 18.3785 | 18.3295 | 18.4095 | 18.5028 | 18.5148 | 18.6527 | 18.5746 | 18.6797 | 10 | 10 | 10 | 10 | 9 | 10 | 10 | 10 |
| GOLGA4 | Q13439;Q1 | 2.18E-17 | 6.27E-17 | -0.212402 | | 1.09381 | -2.09914 | | 15.1431 | 15.213 | 15.1774 | 15.3206 | 15.3794 | 15.1857 | 15.6453 | 15.4201 | 73 | 70 | 70 | 76 | 66 | 70 | 71 | 71 |
| NDUFS8 | O00217 | 5.72E-06 | 1.54E-06 | -0.212345 | | 1.08025 | -2.07656 | | 18.7028 | 18.3944 | 18.7557 | 18.7051 | 18.9126 | 18.8211 | 18.9496 | 18.7615 | 4 | 4 | 4 | 3 | 5 | 3 | 5 | 4 |
| DHX30 | Q7L2E3;Q7 | 1.57E-11 | 8.80E-12 | -0.212107 | + | 2.12206 | -3.94859 | | 15.6646 | 15.6708 | 15.6703 | 15.8982 | 15.8721 | 15.7322 | 15.9622 | 15.9387 | 38 | 36 | 36 | 39 | 35 | 32 | 33 | 34 |
| UFC1 | Q9Y3C8 | 2.42E-08 | 8.38E-09 | -0.21187 | | 0.598766 | -1.26763 | | 17.5619 | 17.3279 | 17.6315 | 17.4638 | 17.8584 | 17.425 | 17.9903 | 17.8572 | 5 | 5 | 6 | 6 | 4 | 4 | 4 | 5 |
| GOLM1 | Q8NBJ4;Q8 | 5.44E-06 | 1.46E-06 | -0.211525 | | 0.710115 | -1.45868 | | 15.1811 | 15.3539 | 15.1557 | 15.3102 | 15.2281 | 15.4795 | 15.37 | 15.8209 | 7 | 6 | 5 | 5 | 5 | 7 | 8 | 6 |
| NCLN | Q969V3;Q9 | 1.65E-10 | 7.66E-11 | -0.211524 | + | 2.34012 | -4.39974 | | 17.4823 | 17.4397 | 17.4049 | 17.6335 | 17.5968 | 17.6265 | 17.7871 | 17.6253 | 19 | 19 | 19 | 19 | 19 | 19 | 19 | 19 |
| RWDD4 | Q6NW29 | 1.88E-11 | 1.04E-11 | -0.211497 | + | 1.40117 | -2.61779 | | 16.3838 | 16.3557 | 16.4351 | 16.6216 | 16.4149 | 16.7868 | 16.6133 | 16.5786 | 4 | 4 | 4 | 4 | 4 | 4 | 3 | 4 |

| Gene names | UniProt IDs | Protein Group P-value | Protein Group Q-value | Log2 Difference NUP214 vs Controls (n=5) | p<0.05 | -log10 p-value | T-test Test statistic | NPC Localization | Log2 MS2_quantity_NUP214_1 | Log2 MS2_quantity_NUP214_2 | Log2 MS2_quantity_NUP214_3 | Log2 MS2_quantity_Control_1 | Log2 MS2_quantity_Control_2 | Log2 MS2_quantity_Control_3 | Log2 MS2_quantity_Control_4 | Log2 MS2_quantity_Control_5 | Number Peptides for Quantitation NUP214_1 | Number Peptides for Quantitation NUP214_2 | Number Peptides for Quantitation NUP214_3 | Number Peptides for Quantitation Control_1 | Number Peptides for Quantitation Control_2 | Number Peptides for Quantitation Control_3 | Number Peptides for Quantitation Control_4 | Number Peptides for Quantitation Control_5 |
|---|---|---|---|---|---|---|---|---|---|---|---|---|---|---|---|---|---|---|---|---|---|---|---|---|
| DCAF13 | Q9NV06 | 1.21E-08 | 4.35E-09 | -0.211146 | | 0.772715 | -1.56437 | | 14.89 | 14.9639 | 14.7897 | 15.393 | 14.8969 | 14.8692 | 15.1048 | 15.198 | 11 | 11 | 11 | 12 | 7 | 12 | 9 | 8 |
| ARSA | P15289;P1 | 1.23E-14 | 1.52E-14 | -0.210921 | | 0.722602 | -1.47984 | | 17.7563 | 17.7494 | 17.7064 | 17.6462 | 18.1535 | 18.1341 | 18.0701 | 17.7377 | 8 | 8 | 8 | 9 | 8 | 9 | 8 | 9 |
| FAM8A1 | Q9UBU6 | 6.78E-10 | 2.86E-10 | -0.210738 | | 0.470074 | -1.03915 | | 15.483 | 15.2522 | 15.206 | 15.0069 | 15.6544 | 15.6533 | 15.8586 | 15.4492 | 3 | 5 | 3 | 4 | 4 | 5 | 5 | 2 |
| SLC38A1 | Q9H2H9 | 0.001393 | 0.000281 | -0.210517 | | 0.220352 | -0.55016 | | 13.9347 | 13.6891 | 13.6995 | 13.8249 | 13.2641 | 14.4056 | 14.8397 | 13.5903 | 3 | 2 | 3 | 1 | 1 | 3 | 3 | 2 |
| SLC38A5 | Q8WUX1 | 5.61E-05 | 1.37E-05 | -0.210135 | | 0.234448 | -0.61365 | | 15.3547 | 15.2518 | 15.5357 | 16.0272 | NaN | 15.1545 | NaN | NaN | 3 | 1 | 3 | 3 | NaN | 2 | NaN | NaN |
| DDX56 | Q9NY93;Q9 | 1.46E-19 | 8.55E-19 | -0.210135 | + | 2.36317 | -4.44908 | | 14.6661 | 14.7637 | 14.7393 | 14.9683 | 14.9743 | 14.8107 | 14.9348 | 14.978 | 13 | 13 | 14 | 14 | 9 | 11 | 13 | 12 |
| RAB24 | Q969Q5 | 1.20E-10 | 5.67E-11 | -0.209865 | + | 1.97296 | -3.65503 | | 15.3748 | 15.3329 | 15.3762 | 15.5459 | 15.6816 | 15.6085 | 15.594 | 15.4258 | 6 | 5 | 6 | 7 | 5 | 6 | 5 | 6 |
| YME1L1 | Q96TA2;Q9 | 1.22E-14 | 1.51E-14 | -0.209859 | | 0.918986 | -1.80861 | | 15.3554 | 15.3779 | 15.309 | 15.6635 | 15.5385 | 15.2566 | 15.5538 | 15.774 | 16 | 13 | 13 | 14 | 14 | 13 | 16 | 17 |
| NAALADL2 | Q58DX5 | 0.008997 | 0.001553 | -0.209836 | | 0.284113 | -0.68339 | | 13.616 | 13.2944 | 13.5088 | 13.6629 | 14.068 | 13.6221 | 14.1665 | 12.895 | 2 | 1 | 2 | 2 | 2 | 3 | 5 | 1 |
| ZFP91 | Q96JP5 | 0.001837 | 0.000363 | -0.2098 | | 0.253465 | -0.6385 | | 12.0346 | 12.9089 | 12.9773 | 12.834 | 13.076 | 12.6403 | NaN | NaN | 3 | 1 | 3 | 2 | 1 | 1 | NaN | NaN |
| CWC22 | Q9HCG8 | 0.000588 | 0.000125 | -0.209735 | | 0.969841 | -1.8931 | | 14.6804 | 14.3901 | 14.4389 | 14.467 | 14.7976 | 14.6722 | 14.8208 | 14.8068 | 3 | 8 | 7 | 4 | 4 | 4 | 7 | 5 |
| RPS28 | P62857 | 0.000228 | 5.15E-05 | -0.209703 | | 0.274786 | -0.66436 | | 21.8891 | 21.683 | 21.7038 | 21.9889 | 22.642 | 21.2768 | 21.6819 | 22.2521 | 4 | 4 | 4 | 4 | 4 | 4 | 4 | 4 |
| ABHD16A | O95870 | 7.62E-05 | 1.83E-05 | -0.209694 | | 1.1056 | -2.11878 | | 15.1928 | 15.2245 | 15.2507 | 15.6756 | 15.3683 | 15.4484 | 15.2208 | 15.4487 | 11 | 14 | 11 | 12 | 8 | 12 | 11 | 11 |
| PICALM | Q13492;Q1 | 2.11E-09 | 8.34E-10 | -0.209362 | + | 1.51817 | -2.82068 | | 18.0235 | 18.0994 | 18.0689 | 18.3344 | 18.1029 | 18.3625 | 18.3785 | 18.1881 | 18 | 19 | 18 | 18 | 17 | 19 | 19 | 19 |
| PDCD5 | O14737 | 0.000624 | 0.000132 | -0.209289 | | 0.162814 | -0.42253 | | 17.5019 | 16.9657 | 18.2166 | 18.3287 | 17.6253 | 16.8028 | 17.5245 | 18.5722 | 6 | 6 | 6 | 6 | 6 | 6 | 6 | 6 |
| SCAMP2 | O15127 | 3.42E-05 | 8.52E-06 | -0.209278 | | 1.20316 | -2.28187 | | 17.4931 | 17.7747 | 17.5118 | 17.7992 | 17.9558 | 17.8129 | 17.7876 | 17.6569 | 1 | 1 | 1 | 1 | 2 | 1 | 2 | 1 |
| KDELR2 | P33947 | 1.03E-08 | 3.75E-09 | -0.209139 | | 0.604263 | -1.27717 | | 17.9938 | 18.0426 | 17.796 | 17.8545 | 17.9852 | 18.1002 | 18.4908 | 18.3357 | 2 | 2 | 2 | 2 | 2 | 2 | 2 | 2 |
| ARRDC1 | Q8N5I2 | 0.000474 | 0.000102 | -0.208948 | | 0.262755 | -0.6396 | | 16.2612 | 16.7802 | 16.4793 | 16.3547 | 17.3698 | 16.2763 | 17.1784 | 16.4002 | 2 | 2 | 2 | 1 | 2 | 1 | 2 | 1 |
| OTUD7B | Q6GQQ9;Q | 0.001526 | 0.000307 | -0.208825 | | 0.857444 | -1.70616 | | 15.5564 | 15.7095 | 15.6846 | 15.8906 | 15.5177 | 15.9663 | 15.9076 | 16.0129 | 8 | 8 | 9 | 8 | 7 | 8 | 9 | 9 |
| IGFBP4 | P22692 | 1.58E-06 | 4.49E-07 | -0.208807 | | 0.56484 | -1.20835 | | 15.3419 | 15.2809 | 15.46 | 15.4689 | 15.8653 | 15.1638 | 15.5435 | 15.8071 | 5 | 5 | 5 | 6 | 5 | 6 | 4 | 6 |
| CLTC | Q00610;Q0 | 1.90E-15 | 3.00E-15 | -0.208776 | + | 2.36914 | -4.46192 | | 20.2711 | 20.3163 | 20.291 | 20.5664 | 20.5894 | 20.4284 | 20.5015 | 20.422 | 92 | 92 | 93 | 92 | 91 | 94 | 91 | 90 |
| KAT7 | O95251;O9 | 3.63E-07 | 1.11E-07 | -0.208706 | | 1.22469 | -2.31804 | | 14.6359 | 14.4315 | 14.5514 | 14.9032 | 14.6121 | 14.6162 | 14.762 | 14.848 | 5 | 4 | 6 | 3 | 1 | 3 | 2 | 2 |
| TADA3 | O75528;O7 | 1.30E-08 | 4.65E-09 | -0.208658 | | 0.542336 | -1.16869 | | 13.1267 | 13.8211 | 13.5919 | 13.7082 | 13.9197 | 13.7473 | 13.465 | 13.7693 | 1 | 2 | 1 | 2 | 2 | 1 | 1 | 2 |
| NCK2 | O43639 | 2.37E-11 | 1.28E-11 | -0.208613 | | 0.689939 | -1.42439 | | 14.9223 | 14.8413 | 15.0491 | 15.0978 | 15.495 | 14.8695 | 15.0381 | 15.2306 | 8 | 10 | 7 | 6 | 7 | 5 | 3 | 7 |
| EEF1A1 | P68104 | 5.31E-11 | 2.69E-11 | -0.208575 | + | 2.61867 | -5.01912 | | 22.8878 | 22.9941 | 22.9313 | 23.1075 | 23.2248 | 23.1893 | 23.125 | 23.085 | 34 | 35 | 36 | 35 | 35 | 35 | 34 | 33 |
| KIF1B | O60333-3 | 1.85E-11 | 1.03E-11 | -0.20838 | | 0.408768 | -0.92605 | | 15.6626 | 15.926 | 15.6586 | 16.4491 | 16.2008 | 15.6487 | 15.6109 | 15.8777 | 4 | 2 | 4 | 3 | 2 | 3 | 2 | 3 |
| EEF1G | P26641 | 7.09E-07 | 2.10E-07 | -0.208222 | + | 2.39127 | -4.50968 | | 20.6884 | 20.7251 | 20.6795 | 20.8559 | 20.8531 | 20.9228 | 21.0312 | 20.8665 | 21 | 20 | 21 | 20 | 20 | 20 | 21 | 20 |
| USP33 | Q8TEY7;Q8 | 0.000746 | 0.000157 | -0.208205 | | 1.00182 | -1.94621 | | 14.4156 | 14.0384 | 14.2059 | 14.6217 | 14.4053 | 14.4021 | 14.2918 | 14.4199 | 1 | 2 | 1 | 2 | 1 | 3 | 3 | 2 |
| MON1B | Q7L1V2;Q7 | 0.002586 | 0.000492 | -0.208169 | + | 1.47262 | -2.74124 | | 15.0777 | 15.0428 | 15.0052 | 15.055 | 15.4002 | 15.2732 | 15.2817 | 15.2403 | 5 | 5 | 4 | 4 | 4 | 5 | 4 | 4 |
| DDX10 | Q13206 | 1.21E-22 | 1.95E-21 | -0.208082 | | 1.21666 | -2.30454 | | 15.413 | 15.5028 | 15.5408 | 15.611 | 15.8166 | 15.4792 | 15.7825 | 15.7788 | 8 | 10 | 11 | 10 | 6 | 6 | 10 | 9 |
| CFAP36 | Q96G28;Q9 | 3.55E-08 | 1.21E-08 | -0.208044 | | 1.03148 | -1.99548 | | 14.8252 | 14.7018 | 14.9785 | 14.8123 | 15.1297 | 15.0431 | 15.1948 | 15.0362 | 5 | 5 | 5 | 4 | 3 | 5 | 4 | 3 |
| AKAP11 | Q9UKA4 | 4.99E-18 | 1.78E-17 | -0.20803 | | 1.25533 | -2.36963 | | 14.4604 | 14.4783 | 14.454 | 14.892 | 14.5054 | 14.718 | 14.5822 | 14.664 | 14 | 19 | 18 | 18 | 13 | 15 | 12 | 13 |
| DDX47 | Q9H0S4 | 2.11E-11 | 1.15E-11 | -0.207746 | | 1.08908 | -2.09127 | | 15.3874 | 15.5078 | 15.4544 | 15.8403 | 15.5785 | 15.4351 | 15.6557 | 15.7784 | 13 | 15 | 11 | 12 | 10 | 15 | 15 | 16 |
| RPF1 | Q9H9Y2 | 5.76E-06 | 1.54E-06 | -0.207707 | | 1.04698 | -2.02123 | | 17.0603 | 16.9072 | 16.874 | 16.9654 | 17.3571 | 17.0292 | 17.2057 | 17.217 | 3 | 3 | 3 | 3 | 3 | 3 | 3 | 3 |
| SMARCC1 | Q92922 | 1.76E-06 | 4.99E-07 | -0.207561 | | 0.936508 | -1.83773 | | 15.4446 | 15.4218 | 15.4058 | 15.6359 | 15.6483 | 15.3197 | 15.7396 | 15.8147 | 9 | 7 | 9 | 8 | 5 | 6 | 6 | 5 |
| EPS15L1 | Q9UBC2-2 | 5.17E-15 | 7.11E-15 | -0.207303 | + | 2.12463 | -3.95375 | | 16.4275 | 16.4123 | 16.4542 | 16.6111 | 16.7083 | 16.5746 | 16.7497 | 16.5494 | 30 | 31 | 32 | 29 | 25 | 30 | 30 | 28 |
| AATF | Q9NY61 | 1.05E-07 | 3.40E-08 | -0.207289 | | 0.837597 | -1.67304 | | 14.5027 | 14.6367 | 14.7098 | 15.0582 | 14.5301 | 14.8048 | 14.9176 | 14.8078 | 7 | 8 | 8 | 5 | 3 | 7 | 7 | 7 |
| KAT8 | Q9H7Z6 | 0.004409 | 0.000796 | -0.206771 | | 0.562228 | -1.22824 | | 14.7623 | 14.4862 | 14.3585 | 14.7057 | NaN | 14.4345 | 14.9477 | 14.8819 | 2 | 2 | 3 | 2 | NaN | 1 | 1 | 2 |
| HECTD1 | Q9ULT8 | 2.25E-13 | 1.94E-13 | -0.206643 | + | 1.43942 | -2.6837 | | 15.6611 | 15.7381 | 15.6619 | 15.8292 | 16.105 | 15.795 | 15.8305 | 15.9088 | 49 | 48 | 49 | 45 | 37 | 42 | 42 | 42 |
| CAMSAP2 | Q08AD1;Q0 | 6.57E-12 | 3.96E-12 | -0.206555 | | 0.81738 | -1.63926 | | 14.6067 | 14.637 | 14.6548 | 14.515 | 15.0106 | 14.744 | 14.9258 | 15.0016 | 14 | 14 | 14 | 13 | 11 | 14 | 13 | 12 |
| GRSF1 | Q12849 | 7.66E-13 | 5.69E-13 | -0.206439 | | 0.911079 | -1.79546 | | 16.3464 | 16.2726 | 16.3248 | 16.7284 | 16.4966 | 16.2768 | 16.409 | 16.6944 | 11 | 11 | 10 | 12 | 11 | 11 | 13 | 11 |
| TRA2A | Q13595;Q1 | 8.23E-08 | 2.68E-08 | -0.206262 | + | 1.43999 | -2.68469 | | 17.3204 | 17.328 | 17.2863 | 17.6039 | 17.5884 | 17.2982 | 17.5117 | 17.5869 | 8 | 8 | 8 | 8 | 8 | 8 | 8 | 8 |
| SERPINB6 | P35237 | 4.12E-12 | 2.57E-12 | -0.206213 | + | 1.34835 | -2.52737 | | 19.0005 | 18.9916 | 19.0193 | 19.4114 | 19.1923 | 19.1963 | 19.0276 | 19.2224 | 22 | 22 | 22 | 22 | 19 | 22 | 22 | 22 |
| TMED5 | Q9Y3A6 | 1.78E-14 | 2.11E-14 | -0.206143 | | 1.13805 | -2.1729 | | 16.6496 | 16.7486 | 16.7588 | 17.1439 | 16.7833 | 16.7737 | 16.9506 | 16.9742 | 6 | 6 | 7 | 7 | 6 | 6 | 7 | 7 |
| ITGA8 | P53708 | 6.80E-05 | 1.64E-05 | -0.205877 | | 0.125111 | -0.33714 | | 13.7997 | 13.85 | 13.8128 | NaN | 14.7212 | 12.4921 | 14.3976 | 14.496 | 4 | 2 | 5 | NaN | 5 | 2 | 5 | 2 |
| USP34 | Q70CQ2 | 0.002572 | 0.000489 | -0.205823 | | 0.655929 | -1.36629 | | 14.7857 | 14.6216 | 14.369 | 14.7904 | 14.9432 | 14.5341 | 14.6761 | 15.046 | 6 | 10 | 8 | 6 | 5 | 4 | 4 | 6 |
| AAMP | Q13685 | 7.94E-19 | 3.58E-18 | -0.205774 | + | 1.95182 | -3.61429 | | 15.0754 | 15.0525 | 15.0709 | 15.3152 | 15.3004 | 15.1224 | 15.3745 | 15.2476 | 7 | 8 | 8 | 8 | 8 | 6 | 6 | 8 |
| RBM19 | Q9Y4C8 | 5.17E-07 | 1.56E-07 | -0.205551 | + | 1.79622 | -3.32086 | | 14.371 | 14.3927 | 14.343 | 14.6544 | 14.4653 | 14.4597 | 14.6505 | 14.6423 | 8 | 9 | 7 | 9 | 5 | 8 | 8 | 7 |
| SF3B4 | Q15427 | 3.42E-14 | 3.70E-14 | -0.20542 | | 1.11766 | -2.13889 | | 17.2102 | 17.1127 | 17.199 | 17.293 | 17.2471 | 17.2587 | 17.571 | 17.5272 | 5 | 5 | 5 | 5 | 5 | 5 | 5 | 5 |
| AFDN | P55196;P5 | 1.82E-12 | 1.23E-12 | -0.205339 | + | 1.45356 | -2.70817 | | 14.5325 | 14.5767 | 14.7414 | 14.8037 | 14.9947 | 14.7337 | 14.7759 | 14.8029 | 11 | 14 | 15 | 15 | 8 | 12 | 11 | 11 |
| S100A13 | Q99584 | 0.000487 | 0.000105 | -0.205018 | | 0.212016 | -0.53215 | | 18.0777 | 17.6478 | 18.6686 | 18.7848 | 17.7917 | 17.7202 | 18.6106 | 18.7745 | 7 | 7 | 7 | 7 | 7 | 6 | 7 | 7 |
| RNF123 | Q5XPI4 | 6.65E-09 | 2.47E-09 | -0.20462 | + | 1.41566 | -2.64272 | | 15.6642 | 15.6639 | 15.6674 | 15.7392 | 16.0013 | 15.9482 | 15.9392 | 15.721 | 10 | 10 | 12 | 10 | 6 | 11 | 11 | 9 |
| AGO2 | Q9UKV8 | 1.12E-10 | 5.34E-11 | -0.2046 | + | 1.45428 | -2.70941 | | 15.1695 | 15.1693 | 15.2138 | 15.3856 | 15.1892 | 15.5094 | 15.3795 | 15.4803 | 25 | 23 | 23 | 20 | 18 | 22 | 21 | 21 |
| MINK1 | Q8N4C8;Q8 | 6.53E-08 | 2.15E-08 | -0.204541 | | 1.25004 | -2.36071 | | 14.943 | 15.0129 | 14.8659 | 15.2649 | 15.0217 | 15.1951 | 14.9798 | 15.2644 | 10 | 11 | 8 | 12 | 6 | 7 | 10 | 9 |
| SNX12 | Q9UMY4;Q | 1.89E-06 | 5.32E-07 | -0.204224 | | 0.926979 | -1.82189 | | 18.0334 | 18.0222 | 17.9605 | 18.2472 | 18.3296 | 17.8894 | 18.3489 | 18.2328 | 7 | 7 | 7 | 7 | 7 | 7 | 7 | 7 |
| SNIP1 | Q8TAD8 | 0.000257 | 5.75E-05 | -0.203998 | | 0.429861 | -0.96534 | | 14.3813 | 14.1696 | 14.5859 | 14.3849 | 15.1502 | 14.3834 | 14.482 | 14.514 | 4 | 5 | 4 | 3 | 2 | 1 | 3 | 3 |
| EXOC2 | Q96KP1 | 2.95E-16 | 5.86E-16 | -0.203926 | + | 2.41854 | -4.56896 | | 15.7719 | 15.8289 | 15.8261 | 16.0744 | 15.9573 | 15.9198 | 16.0786 | 16.0345 | 25 | 26 | 26 | 29 | 24 | 26 | 27 | 27 |
| MED1 | Q15648 | 1.55E-10 | 7.22E-11 | -0.203611 | | 0.972272 | -1.89713 | | 14.7603 | 14.8017 | 14.6922 | 14.8957 | 15.238 | 14.8293 | 15.0041 | 14.8079 | 5 | 5 | 6 | 4 | 3 | 4 | 5 | 4 |
| TM9SF3 | Q9HD45 | 5.44E-14 | 5.59E-14 | -0.202797 | + | 2.05386 | -3.81289 | | 17.4045 | 17.3889 | 17.3525 | 17.5551 | 17.5949 | 17.5542 | 17.7266 | 17.4932 | 15 | 15 | 16 | 14 | 12 | 15 | 14 | 13 |
| IMP4 | Q96G21 | 4.29E-19 | 2.15E-18 | -0.202695 | + | 2.07807 | -3.86078 | | 15.5216 | 15.6356 | 15.5127 | 15.8012 | 15.7722 | 15.6708 | 15.7011 | 15.8514 | 7 | 7 | 5 | 6 | 6 | 6 | 8 | 7 |
| ERP44 | Q9BS26 | 1.39E-09 | 5.64E-10 | -0.202522 | | 0.533288 | -1.15266 | | 18.9439 | 18.9358 | 18.9197 | 18.8898 | 18.9538 | 19.1448 | 19.6329 | 19.057 | 17 | 17 | 17 | 17 | 16 | 18 | 17 | 18 |
| PRDX3 | P30048;P3 | 7.66E-16 | 1.36E-15 | -0.20235 | + | 1.31249 | -2.46636 | | 20.2153 | 20.0073 | 20.1086 | 20.1327 | 20.4462 | 20.3093 | 20.3018 | 20.3738 | 12 | 12 | 12 | 12 | 12 | 12 | 12 | 12 |
| GCC2 | Q8IWJ2 | 1.43E-14 | 1.74E-14 | -0.202191 | + | 1.53953 | -2.85815 | | 14.2475 | 14.305 | 14.304 | 14.3209 | 14.5409 | 14.5382 | 14.6165 | 14.4219 | 21 | 24 | 24 | 24 | 20 | 27 | 21 | 20 |
| FAM207A | Q9NSI2;Q9 | 0.001669 | 0.000333 | -0.202158 | | 0.407077 | -0.92288 | | 14.8567 | 14.7125 | 14.9059 | 15.5043 | 14.9609 | 14.9334 | 15.2066 | 14.5306 | 2 | 4 | 2 | 2 | 2 | 3 | 3 | 1 |
| DIP2B | Q9P265 | 8.52E-16 | 1.48E-15 | -0.202031 | | 0.664746 | -1.38139 | | 15.3082 | 15.3177 | 15.285 | 15.8144 | 15.1533 | 15.5842 | 15.5714 | 15.405 | 19 | 21 | 23 | 27 | 22 | 25 | 23 | 24 |
| EML2 | O95834;O9 | 1.17E-10 | 5.55E-11 | -0.201726 | | 0.570623 | -1.2185 | | 16.0934 | 16.1207 | 16.032 | 15.8674 | 16.2868 | 16.4098 | 16.6194 | 16.2355 | 13 | 13 | 13 | 13 | 13 | 14 | 14 | 14 |
| TTC4 | O95801 | 0.00019 | 4.32E-05 | -0.201684 | | 0.734027 | -1.49917 | | 14.5911 | 14.656 | 14.6413 | 14.6198 | 15.0747 | 14.6747 | 14.7128 | 15.0737 | 8 | 11 | 10 | 10 | 6 | 8 | 7 | 9 |
| PTPN12 | Q05209 | 2.64E-17 | 7.37E-17 | -0.201614 | + | 1.37751 | -2.5772 | | 15.7926 | 15.78 | 15.8089 | 16.1712 | 15.9964 | 16.0629 | 15.8334 | 15.9134 | 21 | 23 | 24 | 23 | 18 | 23 | 20 | 23 |
| PGM3 | O95394;O9 | 1.40E-15 | 2.30E-15 | -0.201322 | | 0.962543 | -1.88097 | | 17.7739 | 17.7498 | 17.7426 | 17.7607 | 17.9584 | 18.1866 | 18.0741 | 17.8041 | 25 | 24 | 27 | 23 | 24 | 24 | 23 | 24 |
| ROCK1 | Q13464 | 4.13E-13 | 3.32E-13 | -0.201118 | | 0.973942 | -1.89991 | | 15.5463 | 15.6164 | 15.5726 | 16.0461 | 15.7767 | 15.7446 | 15.5531 | 15.7773 | 44 | 44 | 44 | 50 | 38 | 46 | 37 | 43 |
| DDHD2 | O94830 | 0.001788 | 0.000355 | -0.201111 | | 1.17519 | -2.23499 | | 15.1803 | 15.1138 | 15.1412 | 15.413 | 15.5168 | 15.3505 | 15.1112 | 15.3396 | 5 | 6 | 7 | 6 | 6 | 5 | 6 | 6 |
| RCN3 | Q96D15 | 4.05E-06 | 1.11E-06 | -0.201059 | | 0.309576 | -0.73465 | | 20.6503 | 20.3149 | 20.8362 | 20.4377 | 20.6758 | 21.2659 | 21.2258 | 20.4025 | 14 | 14 | 14 | 14 | 15 | 15 | 15 | 14 |
| PRXL2C | Q7RTV5 | 0.001706 | 0.00034 | -0.200968 | | 0.285727 | -0.68668 | | 15.7532 | 15.853 | 15.6482 | 16.2301 | 16.4022 | 15.1701 | 15.8224 | 16.1375 | 5 | 4 | 6 | 6 | 5 | 2 | 5 | 4 |
| AP4B1 | Q9Y6B7 | 1.43E-08 | 5.10E-09 | -0.200937 | | 0.657802 | -1.3695 | | 14.9495 | 15.0628 | 15.0894 | 15.0294 | 15.3707 | 15.5118 | 15.3213 | 14.9411 | 6 | 5 | 7 | 4 | 4 | 8 | 5 | 5 |
| BRI3BP | Q8WY22 | 0.002965 | 0.000553 | -0.200756 | | 0.476054 | -1.05001 | | 14.5966 | 14.5008 | 14.4577 | 14.5121 | 14.5897 | 14.555 | 15.2771 | 14.6617 | 2 | 3 | 2 | 2 | 2 | 2 | 2 | 3 |
| RNF170 | Q96K19 | 4.66E-11 | 2.38E-11 | -0.200532 | | 1.02739 | -1.98868 | | 15.4067 | 15.4193 | 15.5578 | 15.5282 | 15.5884 | 15.715 | 15.9154 | 15.5621 | 5 | 5 | 4 | 4 | 4 | 4 | 4 | 5 |
| ZFAND2B | Q8WV99;Q | 0.000221 | 5.00E-05 | -0.200473 | | 0.780259 | -1.57704 | | 16.1498 | 16.0602 | 16.1564 | 16.5778 | 16.1957 | 16.2062 | 16.1153 | 16.5182 | 2 | 2 | 2 | 3 | 2 | 2 | 2 | 2 |
| PRDX1 | Q06830 | 1.26E-13 | 1.16E-13 | -0.200291 | + | 2.45298 | -4.6445 | | 21.4636 | 21.4654 | 21.4282 | 21.6169 | 21.6669 | 21.7666 | 21.6322 | 21.5808 | 21 | 21 | 20 | 20 | 21 | 21 | 21 | 21 |

| Gene names | UniProt IDs | Protein Group P-value | Protein Group Q-value | Log2 Difference NUP214 vs Controls (n=5) | p<0.05 | -log10 p-value | T-test Test statistic | NPC Localization | Log2 MS2_quantity_NUP214_1 | Log2 MS2_quantity_NUP214_2 | Log2 MS2_quantity_NUP214_3 | Log2 MS2_quantity_Control_1 | Log2 MS2_quantity_Control_2 | Log2 MS2_quantity_Control_3 | Log2 MS2_quantity_Control_4 | Log2 MS2_quantity_Control_5 | Number Peptides for Quantitation NUP214_1 | Number Peptides for Quantitation NUP214_2 | Number Peptides for Quantitation NUP214_3 | Number Peptides for Quantitation Control_1 | Number Peptides for Quantitation Control_2 | Number Peptides for Quantitation Control_3 | Number Peptides for Quantitation Control_4 | Number Peptides for Quantitation Control_5 |
|---|---|---|---|---|---|---|---|---|---|---|---|---|---|---|---|---|---|---|---|---|---|---|---|---|
| CERS2 | Q96G23 | 9.32E-16 | 1.61E-15 | -0.200106 | | 0.856775 | -1.70505 | | 16.6238 | 16.5586 | 16.7802 | 17.0296 | 16.6839 | 16.6378 | 16.9606 | 16.9596 | 4 | 4 | 4 | 4 | 4 | 4 | 4 | 4 |
| RIOX1 | Q9H6W3 | 3.99E-05 | 9.88E-06 | -0.19992 | | 0.797273 | -1.60559 | | 14.9941 | 15.3006 | 15.1556 | 15.4226 | 15.6178 | 15.3166 | 15.1636 | 15.2295 | 5 | 4 | 5 | 5 | 5 | 3 | 4 | 5 |
| GORASP2 | Q9H8Y8;Q9 | 1.87E-08 | 6.57E-09 | -0.199864 | + | 1.49691 | -2.78352 | | 17.5444 | 17.553 | 17.6612 | 17.6301 | 17.8022 | 17.8581 | 17.9134 | 17.7265 | 9 | 10 | 9 | 9 | 8 | 10 | 9 | 9 |
| RALGAPA1 | Q6GYQ0;Q6 | 0.000588 | 0.000125 | -0.199267 | | 0.593016 | -1.25762 | | 13.9063 | 13.7104 | 13.3305 | 13.7342 | 14.0557 | 13.7946 | 13.9888 | 13.6685 | 3 | 2 | 2 | 2 | 1 | 2 | 2 | 2 |
| EPHA2 | P29317 | 3.70E-14 | 3.97E-14 | -0.199151 | | 0.579184 | -1.23348 | | 15.4111 | 15.2648 | 15.3719 | 15.5845 | 15.4465 | 15.3833 | 15.9927 | 15.3352 | 17 | 15 | 15 | 16 | 16 | 15 | 19 | 14 |
| GLRX3 | O76003 | 8.45E-12 | 4.98E-12 | -0.198982 | + | 1.8692 | -3.45713 | | 17.5412 | 17.5368 | 17.5384 | 17.7499 | 17.7906 | 17.5719 | 17.7593 | 17.8174 | 14 | 15 | 15 | 16 | 15 | 15 | 15 | 15 |
| CLEC16A | Q2KHT3 | 2.65E-05 | 6.66E-06 | -0.198906 | | 1.11239 | -2.13009 | | 14.8916 | 14.9887 | 14.9393 | 15.2922 | 15.052 | 14.9896 | 15.3154 | 15.0447 | 4 | 8 | 7 | 9 | 4 | 5 | 7 | 4 |
| PPP6R1 | Q9UPN7 | 4.25E-08 | 1.43E-08 | -0.198768 | + | 3.60779 | -7.72614 | | 15.2621 | 15.269 | 15.1832 | 15.4157 | 15.4232 | 15.4779 | 15.4169 | 15.4506 | 11 | 11 | 13 | 12 | 10 | 12 | 12 | 9 |
| PRPF39 | Q86UA1 | 3.39E-12 | 2.16E-12 | -0.198624 | | 0.393519 | -0.89736 | | 14.8258 | 14.6122 | 14.7368 | 14.7095 | 15.4913 | 14.5292 | 14.9011 | 14.9865 | 5 | 7 | 4 | 3 | 3 | 3 | 2 | 3 |
| MPHOSPH6 | Q99547 | 3.70E-07 | 1.13E-07 | -0.198504 | | 0.398952 | -0.90761 | | 13.5954 | 13.5292 | 13.5929 | 14.2046 | 13.5983 | 13.3958 | 13.5365 | 14.1199 | 2 | 2 | 2 | 2 | 1 | 2 | 1 | 2 |
| VPS52 | Q8N1B4 | 9.83E-11 | 4.71E-11 | -0.198498 | | 0.843596 | -1.68306 | | 15.6127 | 15.4041 | 15.4373 | 15.4451 | 15.8497 | 15.5606 | 15.861 | 15.6998 | 13 | 13 | 15 | 13 | 10 | 13 | 14 | 14 |
| NSD3 | Q9BZ95;Q9 | 0.025332 | 0.004185 | -0.198491 | | 0.362409 | -0.83807 | | 14.4854 | 13.9842 | 14.3788 | 14.3119 | 14.0539 | 14.373 | 14.9338 | 14.7338 | 2 | 2 | 3 | 3 | 1 | 1 | 2 | 1 |
| AMZ2 | Q86W34 | 0.003231 | 0.000597 | -0.198406 | | 0.498172 | -1.08995 | | 14.9513 | 14.9004 | 14.99 | 15.2938 | 15.428 | 15.0783 | 15.2776 | 14.6505 | 3 | 3 | 1 | 2 | 1 | 3 | 1 | 1 |
| PTCD1 | O75127 | 0.002165 | 0.000421 | -0.197914 | | 0.33542 | -0.7857 | | 13.0595 | 13.1755 | 13.2643 | 13.5908 | 13.3943 | 12.7029 | 13.3208 | 13.8128 | 2 | 3 | 3 | 3 | 2 | 1 | 2 | 1 |
| MED20 | Q9H944 | 0.003437 | 0.000631 | -0.197643 | | 1.24441 | -2.35123 | | 14.9156 | 14.8421 | 14.9045 | 14.9198 | 15.1403 | 15.0355 | 15.0405 | 15.2889 | 5 | 5 | 5 | 2 | 4 | 5 | 4 | 4 |
| MPHOSPH10 | O00566 | 8.86E-07 | 2.59E-07 | -0.19742 | + | 2.90997 | -5.72643 | | 14.6021 | 14.5864 | 14.5504 | 14.8408 | 14.7178 | 14.7832 | 14.7254 | 14.8181 | 10 | 9 | 12 | 12 | 8 | 8 | 9 | 11 |
| VPS13A | Q96RL7;Q9 | 0.01261 | 0.002138 | -0.197282 | | 0.632104 | -1.32533 | | 16.1225 | 16.1014 | 16.2473 | 16.6966 | 16.1011 | 16.3239 | 16.4877 | 16.1624 | 4 | 3 | 1 | 2 | 1 | 2 | 3 | 1 |
| CBL | P22681 | 1.33E-15 | 2.20E-15 | -0.19711 | | 0.562333 | -1.20395 | | 15.8464 | 15.7811 | 15.7517 | 16.3286 | 16.1378 | 15.6911 | 15.733 | 16.0603 | 11 | 12 | 10 | 13 | 11 | 9 | 10 | 9 |
| CYBA | P13498 | 6.46E-06 | 1.73E-06 | -0.196925 | | 0.144066 | -0.37907 | | 14.2822 | 14.2879 | 14.3135 | 13.0339 | 15.1355 | 14.9356 | 15.0158 | 14.3365 | 1 | 1 | 1 | 2 | 2 | 1 | 2 | 2 |
| TRIM4 | Q9C037;Q9 | 1.30E-09 | 5.32E-10 | -0.19687 | | 1.15274 | -2.19745 | | 14.9773 | 15.0011 | 14.9675 | 15.3114 | 15.1214 | 15.3216 | 15.1809 | 14.9587 | 13 | 13 | 15 | 13 | 8 | 14 | 12 | 11 |
| SCAF8 | Q9UPN6 | 1.12E-06 | 3.24E-07 | -0.196669 | | 0.755019 | -1.53459 | | 14.8189 | 14.6823 | 14.7463 | 14.8578 | 15.2875 | 14.7255 | 14.8986 | 14.9597 | 5 | 5 | 5 | 6 | 4 | 4 | 4 | 3 |
| UST | Q9Y2C2 | 0.000982 | 0.000203 | -0.196607 | | 0.320523 | -0.76638 | | 14.5762 | 14.5602 | 14.6602 | 15.3202 | NaN | 14.9567 | 14.3484 | 14.5566 | 1 | 2 | 2 | 2 | NaN | 2 | 1 | 1 |
| TENM2 | Q9NT68;Q9 | 7.88E-06 | 2.09E-06 | -0.196571 | | 0.341301 | -0.79719 | | 15.2075 | 15.2832 | 15.2449 | 14.8584 | 15.6064 | 15.1694 | 15.7692 | 15.8056 | 8 | 9 | 10 | 10 | 10 | 10 | 13 | 12 |
| CMC1 | Q7Z7K0 | 0.000265 | 5.93E-05 | -0.196535 | | 0.556565 | -1.1938 | | 15.8381 | 15.8283 | 16.0834 | 15.836 | 16.4349 | 16.0147 | 15.9508 | 16.3293 | 3 | 3 | 4 | 3 | 2 | 3 | 4 | 3 |
| PRRC2A | P48634 | 7.17E-18 | 2.39E-17 | -0.195985 | + | 2.38988 | -4.50667 | | 15.3747 | 15.4272 | 15.4307 | 15.721 | 15.5872 | 15.5312 | 15.5974 | 15.5973 | 23 | 23 | 26 | 22 | 18 | 19 | 20 | 23 |
| TTC7A | Q9ULT0;Q9 | 6.19E-05 | 1.50E-05 | -0.195749 | | 0.340218 | -0.79508 | | 14.8807 | 14.925 | 14.9168 | 15.0033 | 14.4632 | 15.5735 | 15.2807 | 15.1955 | 4 | 3 | 3 | 3 | 2 | 4 | 3 | 3 |
| DPP4 | P27487 | 6.20E-07 | 1.85E-07 | -0.195682 | | 0.114617 | -0.30863 | | 18.2139 | 18.2151 | 18.15 | 16.8038 | 17.799 | 19.2609 | 19.2007 | 18.8788 | 29 | 32 | 31 | 26 | 28 | 33 | 32 | 33 |
| ACP1 | P24666 | 2.03E-11 | 1.11E-11 | -0.195632 | + | 1.97948 | -3.66763 | | 18.1606 | 18.1182 | 18.1789 | 18.2674 | 18.3346 | 18.4959 | 18.314 | 18.3291 | 7 | 7 | 7 | 7 | 7 | 6 | 7 | 7 |
| POLR2M | P0CAP2;P0 | 1.73E-07 | 5.48E-08 | -0.195556 | | 0.91351 | -1.7995 | | 14.496 | 14.3288 | 14.6646 | 14.5686 | 14.9103 | 14.7399 | 14.5969 | 14.6443 | 5 | 5 | 6 | 5 | 3 | 5 | 5 | 5 |
| SARS2 | Q9NP81 | 3.22E-13 | 2.66E-13 | -0.19547 | + | 1.69335 | -3.13248 | | 15.3571 | 15.3718 | 15.4393 | 15.482 | 15.6292 | 15.7333 | 15.5141 | 15.5657 | 12 | 11 | 10 | 11 | 9 | 11 | 10 | 10 |
| KRT80 | Q6KB66;Q6 | 0.002998 | 0.000559 | -0.195448 | | 0.139093 | -0.37084 | | 12.7717 | 12.7113 | 13.1136 | 13.7298 | 12.6405 | NaN | 12.0335 | 13.8401 | 3 | 1 | 3 | 5 | 2 | NaN | 2 | 1 |
| LMF2 | Q9BU23;Q9 | 4.14E-07 | 1.26E-07 | -0.195414 | | 0.813856 | -1.63336 | | 16.9879 | 16.9321 | 16.9426 | 16.8413 | 17.2951 | 17.1017 | 17.3513 | 17.1585 | 11 | 12 | 12 | 12 | 11 | 12 | 12 | 12 |
| SELENOO | Q9BVL4 | 6.41E-12 | 3.88E-12 | -0.195403 | + | 1.30522 | -2.45402 | | 14.7475 | 14.65 | 14.7759 | 14.871 | 15.0912 | 14.7623 | 14.8852 | 14.9895 | 11 | 13 | 13 | 11 | 9 | 10 | 11 | 10 |
| GET1 | O00258;O0 | 0.007983 | 0.001387 | -0.195372 | | 1.05714 | -2.03813 | | 15.0353 | 15.0179 | 15.0124 | 15.3655 | 15.211 | 14.9474 | 15.2745 | 15.2877 | 3 | 4 | 3 | 3 | 2 | 3 | 4 | 4 |
| BIN1 | O00499;O0 | 6.40E-13 | 4.86E-13 | -0.195367 | | 0.806326 | -1.62075 | | 15.9032 | 15.8678 | 15.9174 | 15.9199 | 16.0271 | 16.4409 | 16.0394 | 16.0302 | 17 | 17 | 17 | 17 | 16 | 16 | 16 | 15 |
| SLC39A14 | Q15043;Q1 | 0.000193 | 4.39E-05 | -0.195252 | | 0.863654 | -1.71652 | | 16.3495 | 16.2684 | 16.3053 | 16.6824 | 16.2011 | 16.4513 | 16.5662 | 16.6138 | 2 | 3 | 3 | 3 | 3 | 3 | 3 | 3 |
| KIDINS220 | Q9ULH0;Q9 | 6.20E-07 | 1.85E-07 | -0.195002 | | 0.5473 | -1.17746 | | 14.012 | 14.1461 | 14.4769 | 14.6673 | 14.0698 | 14.4527 | 14.4926 | 14.3508 | 7 | 11 | 10 | 13 | 6 | 10 | 9 | 9 |
| DGKH | Q86XP1;Q8 | 0.006572 | 0.001155 | -0.194997 | | 0.198587 | -0.52971 | | 14.1238 | 13.2263 | 14.0129 | 13.9155 | NaN | 14.0499 | NaN | NaN | 1 | 1 | 1 | 1 | NaN | 1 | NaN | NaN |
| TMED1 | Q13445 | 1.46E-11 | 8.24E-12 | -0.194982 | | 0.918693 | -1.80812 | | 16.9275 | 16.8173 | 16.9254 | 17.0704 | 17.0251 | 17.0643 | 17.3716 | 16.894 | 6 | 6 | 6 | 6 | 6 | 6 | 6 | 6 |
| PIGU | Q9H490;Q9 | 5.95E-05 | 1.45E-05 | -0.194794 | | 0.87714 | -1.73898 | | 16.2644 | 16.3371 | 16.4631 | 16.5399 | 16.5239 | 16.2859 | 16.7532 | 16.6453 | 3 | 3 | 3 | 4 | 3 | 3 | 4 | 4 |
| PTRH1 | Q86Y79 | 2.28E-07 | 7.14E-08 | -0.194776 | | 0.388084 | -0.88708 | | 14.805 | 14.949 | 14.6626 | 15.2729 | 15.0447 | 14.4646 | 14.8708 | 15.3487 | 2 | 2 | 2 | 2 | 2 | 2 | 2 | 4 |
| CDC42BPA | Q5VT25;Q5 | 2.21E-11 | 1.21E-11 | -0.194707 | | 0.766983 | -1.55473 | | 15.1517 | 15.1289 | 15.1129 | 15.2687 | 15.3373 | 15.4231 | 15.5843 | 15.016 | 31 | 35 | 36 | 32 | 25 | 34 | 33 | 28 |
| TBC1D7 | Q9P0N9;Q9 | 0.015304 | 0.002574 | -0.194483 | | 0.429381 | -0.98006 | | 15.1981 | 15.0699 | 15.01 | 15.3204 | NaN | 15.2988 | 14.8667 | 15.6627 | 3 | 3 | 2 | 3 | NaN | 2 | 2 | 1 |
| NSUN2 | Q08J23 | 2.49E-10 | 1.12E-10 | -0.19443 | + | 3.7507 | -8.19856 | | 16.1354 | 16.1392 | 16.1608 | 16.3653 | 16.3074 | 16.369 | 16.2885 | 16.3675 | 29 | 31 | 30 | 31 | 27 | 31 | 28 | 28 |
| RAB2B | Q8WUD1 | 5.56E-09 | 2.09E-09 | -0.194394 | + | 2.27581 | -4.26382 | | 19.4782 | 19.542 | 19.51 | 19.7561 | 19.6371 | 19.6701 | 19.806 | 19.653 | 11 | 11 | 11 | 10 | 11 | 11 | 11 | 11 |
| GRB10 | Q13322;Q1 | 1.65E-12 | 1.12E-12 | -0.194158 | + | 1.31453 | -2.46982 | | 14.5463 | 14.4439 | 14.3743 | 14.8436 | 14.5973 | 14.6079 | 14.5385 | 14.6577 | 7 | 5 | 4 | 5 | 4 | 5 | 2 | 4 |
| GTF2H2C | Q6P1K8 | 0.004422 | 0.000798 | -0.194124 | | 0.616432 | -1.29827 | | 13.8715 | 13.9925 | 13.875 | 14.1152 | 14.1274 | 14.0234 | 14.4761 | 13.7935 | 7 | 8 | 8 | 6 | 2 | 4 | 3 | 5 |
| C1orf43 | Q9BWL3;Q9 | 0.001761 | 0.00035 | -0.193228 | | 0.379151 | -0.9028 | | NaN | 15.3266 | 15.2011 | 15.0878 | 15.4123 | NaN | 15.7428 | 15.5854 | NaN | 1 | 1 | 1 | 1 | NaN | 1 | 1 |
| NEDD1 | Q8NHV4;Q8 | 0.002388 | 0.000459 | -0.193222 | | 0.731383 | -1.4947 | | 14.7999 | 14.7932 | 14.9357 | 15.0423 | 14.8697 | 14.8666 | 15.3802 | 15.022 | 6 | 7 | 6 | 7 | 4 | 6 | 6 | 6 |
| SDCBP | O00560-2 | 1.18E-05 | 3.08E-06 | -0.1931 | | 0.67863 | -1.40512 | | 18.5486 | 18.478 | 18.4773 | 19.0226 | 18.7938 | 18.4809 | 18.4801 | 18.6945 | 11 | 11 | 11 | 9 | 11 | 9 | 10 | 10 |
| NRDC | O43847;O4 | 1.08E-16 | 2.53E-16 | -0.19271 | | 0.822192 | -1.6473 | | 16.5047 | 16.5882 | 16.578 | 16.633 | 16.7233 | 16.5044 | 16.9424 | 16.9452 | 30 | 32 | 30 | 29 | 28 | 31 | 32 | 32 |
| UTP15 | Q8TED0 | 4.96E-15 | 6.86E-15 | -0.192705 | + | 1.32704 | -2.49108 | | 15.1157 | 15.2023 | 15.169 | 15.4861 | 15.307 | 15.1652 | 15.3743 | 15.4424 | 11 | 14 | 13 | 11 | 11 | 11 | 14 | 13 |
| CASK | O14936;O1 | 5.98E-09 | 2.24E-09 | -0.19268 | | 0.457552 | -1.01631 | | 16.2581 | 16.2618 | 16.1558 | 16.0548 | 16.5462 | 16.6665 | 16.7167 | 16.1052 | 18 | 19 | 19 | 18 | 17 | 19 | 18 | 15 |
| CNPY4 | Q8N129 | 3.59E-11 | 1.87E-11 | -0.192635 | | 0.301815 | -0.71913 | | 16.7423 | 16.4011 | 17.0449 | 16.6273 | 16.6549 | 16.8856 | 17.5826 | 16.8601 | 7 | 7 | 7 | 7 | 6 | 7 | 7 | 6 |
| BAG5 | Q9UL15;Q9 | 1.23E-11 | 7.00E-12 | -0.192487 | + | 1.4232 | -2.65571 | | 14.9683 | 14.9911 | 15.0464 | 15.1936 | 15.2193 | 15.0421 | 15.1493 | 15.3677 | 9 | 9 | 8 | 8 | 8 | 8 | 8 | 8 |
| ZNF330 | Q9Y3S2 | 5.19E-14 | 5.36E-14 | -0.192442 | | 0.974025 | -1.90004 | | 14.7157 | 14.8144 | 14.9207 | 15.0186 | 14.8143 | 15.2212 | 15.0708 | 14.9221 | 5 | 5 | 5 | 5 | 5 | 5 | 5 | 5 |
| NDUFB5 | O43674;O4 | 6.48E-10 | 2.74E-10 | -0.192392 | | 0.970152 | -1.89361 | | 16.6062 | 16.5761 | 16.686 | 17.0003 | 16.8355 | 16.5821 | 16.9297 | 16.7283 | 5 | 5 | 4 | 4 | 3 | 4 | 4 | 3 |
| TBRG4 | Q969Z0 | 1.11E-11 | 6.40E-12 | -0.192152 | | 1.00413 | -1.95004 | | 15.519 | 15.4391 | 15.4288 | 15.5389 | 15.7301 | 15.4757 | 15.6425 | 15.8851 | 11 | 11 | 12 | 12 | 11 | 11 | 12 | 12 |
| TAOK1 | Q7L7X3 | 9.65E-07 | 2.81E-07 | -0.192117 | + | 1.66516 | -3.08158 | | 15.3405 | 15.3447 | 15.3448 | 15.5746 | 15.6563 | 15.3922 | 15.4683 | 15.5859 | 24 | 20 | 24 | 24 | 19 | 24 | 22 | 23 |
| NANP | Q8TBE9 | 0.000304 | 6.75E-05 | -0.192085 | | 0.714406 | -1.46596 | | 15.2649 | 15.517 | 15.4503 | 15.2983 | 15.826 | 15.636 | 15.7112 | 15.5425 | 6 | 4 | 6 | 2 | 2 | 3 | 4 | 4 |
| NT5DC1 | Q5TFE4 | 3.63E-06 | 9.95E-07 | -0.192023 | | 0.950066 | -1.86025 | | 14.8676 | 14.8412 | 14.895 | 14.9503 | 15.3318 | 15.006 | 15.115 | 14.8969 | 9 | 9 | 10 | 9 | 8 | 10 | 9 | 7 |
| ZMPSTE24 | O75844 | 3.31E-11 | 1.74E-11 | -0.191895 | | 0.938152 | -1.84046 | | 17.1448 | 17.1329 | 17.0954 | 17.1418 | 17.3575 | 17.1831 | 17.5829 | 17.3159 | 13 | 14 | 14 | 14 | 14 | 14 | 14 | 14 |
| ANKRD27 | Q96NW4 | 0.000897 | 0.000186 | -0.191713 | | 1.19852 | -2.2741 | | 14.627 | 14.3456 | 14.4277 | 14.7248 | 14.7722 | 14.5635 | 14.5523 | 14.6796 | 4 | 4 | 5 | 4 | 2 | 3 | 4 | 5 |
| PIK3R2 | O00459 | 1.60E-14 | 1.92E-14 | -0.19132 | + | 1.5827 | -2.93432 | | 15.0695 | 14.989 | 15.0921 | 15.1522 | 15.4119 | 15.2167 | 15.2506 | 15.1763 | 10 | 11 | 11 | 10 | 10 | 9 | 12 | 9 |
| SUMO1 | P63165 | 0.000141 | 3.28E-05 | -0.191132 | | 1.05387 | -2.03269 | | 16.5399 | 16.5779 | 16.6051 | 16.9002 | 16.8911 | 16.5761 | 16.6181 | 16.8415 | 4 | 3 | 4 | 3 | 3 | 3 | 3 | 3 |
| PPP3CC | P48454;P4 | 0.000845 | 0.000176 | -0.190954 | | 0.345067 | -0.80452 | | 14.8946 | 14.7634 | 14.8634 | 15.3481 | 14.3553 | 15.0502 | 15.1367 | 15.267 | 2 | 3 | 2 | 2 | 1 | 2 | 3 | 3 |
| QRSL1 | Q9H0R6 | 0.00317 | 0.000587 | -0.190935 | | 0.727466 | -1.48807 | | 13.7067 | 13.7952 | 13.4482 | 13.7133 | 13.7216 | 13.758 | 13.8867 | 14.125 | 3 | 4 | 4 | 3 | 4 | 2 | 4 | 2 |
| PPP6R2 | O75170;O7 | 1.16E-12 | 8.24E-13 | -0.190838 | + | 1.37597 | -2.57457 | | 15.2007 | 15.2796 | 15.2488 | 15.4962 | 15.5997 | 15.2974 | 15.3408 | 15.4354 | 17 | 18 | 19 | 17 | 14 | 17 | 16 | 16 |
| LIMD1 | Q9UGP4 | 1.17E-07 | 3.76E-08 | -0.190744 | | 0.410126 | -0.92859 | | 15.4095 | 15.4599 | 15.465 | 15.8844 | 16.0738 | 15.2129 | 15.4522 | 15.5545 | 8 | 9 | 6 | 10 | 9 | 7 | 7 | 8 |
| DNAJB6 | O75190 | 1.96E-08 | 6.86E-09 | -0.1907 | | 1.14934 | -2.19176 | | 16.1051 | 16.0903 | 16.0728 | 16.5043 | 16.2005 | 16.1334 | 16.2241 | 16.3383 | 5 | 6 | 6 | 6 | 4 | 4 | 6 | 5 |
| CLASP2 | O75122 | 2.49E-08 | 8.63E-09 | -0.190695 | + | 1.32524 | -2.48802 | | 15.1832 | 15.2032 | 15.1745 | 15.5245 | 15.4136 | 15.1795 | 15.4266 | 15.3441 | 24 | 23 | 19 | 24 | 15 | 18 | 21 | 20 |
| RPS6 | P62753 | 3.67E-13 | 2.99E-13 | -0.190343 | | 0.882471 | -1.74786 | | 20.4662 | 20.375 | 20.2853 | 20.4314 | 20.8574 | 20.5586 | 20.4544 | 20.5272 | 14 | 14 | 14 | 13 | 14 | 14 | 13 | 14 |
| YEATS4 | O95619 | 0.007551 | 0.001318 | -0.190304 | | 0.274672 | -0.67216 | | 13.4339 | 13.2788 | 13.1648 | 13.3591 | 13.0516 | 13.376 | NaN | 14.1446 | 2 | 2 | 3 | 3 | 3 | 3 | NaN | 2 |
| APP | P05067;P0 | 1.56E-22 | 2.37E-21 | -0.190174 | | 0.380531 | -0.87274 | | 15.6397 | 15.6431 | 15.6882 | 15.913 | 15.2859 | 15.7768 | 15.9745 | 16.2856 | 14 | 15 | 13 | 14 | 13 | 14 | 13 | 14 |
| RAB21 | Q9UL25 | 7.12E-15 | 9.36E-15 | -0.190142 | + | 2.94045 | -5.80435 | | 18.0967 | 18.0394 | 18.0056 | 18.207 | 18.2605 | 18.1749 | 18.2764 | 18.268 | 10 | 11 | 11 | 11 | 11 | 11 | 10 | 11 |
| UBAP2L | Q14157;Q1 | 3.94E-19 | 2.00E-18 | -0.190104 | | 1.10756 | -2.12205 | | 17.2456 | 17.2805 | 17.3054 | 17.6561 | 17.4672 | 17.4479 | 17.2445 | 17.5205 | 18 | 17 | 17 | 16 | 17 | 18 | 17 | 16 |
| AAGAB | Q6PD74 | 1.13E-08 | 4.07E-09 | -0.190094 | | 1.09991 | -2.1093 | | 15.1822 | 15.2472 | 15.2972 | 15.5294 | 15.226 | 15.4899 | 15.3397 | 15.5766 | 5 | 6 | 6 | 5 | 3 | 4 | 6 | 4 |
| ATG16L1 | Q676U5;Q6 | 3.38E-10 | 1.48E-10 | -0.190035 | + | 1.5725 | -2.91627 | | 15.2858 | 15.2892 | 15.2031 | 15.3082 | 15.5155 | 15.3728 | 15.5511 | 15.4993 | 13 | 14 | 12 | 10 | 10 | 13 | 12 | 11 |

| Gene names | UniProt IDs | Protein Group P-value | Protein Group Q-value | Log2 Difference NUP214 vs Controls (n=5) | p<0.05 | -log10 p-value | T-test Test statistic | NPC Localization | Log2 MS2_quantity_NUP214_1 | Log2 MS2_quantity_NUP214_2 | Log2 MS2_quantity_NUP214_3 | Log2 MS2_quantity_Control_1 | Log2 MS2_quantity_Control_2 | Log2 MS2_quantity_Control_3 | Log2 MS2_quantity_Control_4 | Log2 MS2_quantity_Control_5 | Number Peptides for Quantitation NUP214_1 | Number Peptides for Quantitation NUP214_2 | Number Peptides for Quantitation NUP214_3 | Number Peptides for Quantitation Control_1 | Number Peptides for Quantitation Control_2 | Number Peptides for Quantitation Control_3 | Number Peptides for Quantitation Control_4 | Number Peptides for Quantitation Control_5 |
|---|---|---|---|---|---|---|---|---|---|---|---|---|---|---|---|---|---|---|---|---|---|---|---|---|
| SLMAP | Q14BN4;Q | 0.000115 | 2.69E-05 | -0.190012 | | 0.410274 | -0.92887 | | 14.2751 | 14.2006 | 14.3739 | 14.5438 | 14.1991 | 14.7831 | 14.7901 | 14.05 | 4 | 4 | 5 | 5 | 3 | 4 | 4 | 3 |
| GSTZ1 | O43708;O | 3.16E-07 | 9.74E-08 | -0.190003 | | 1.19483 | -2.26789 | | 15.3848 | 15.3569 | 15.3916 | 15.8005 | 15.4734 | 15.4459 | 15.5481 | 15.571 | 2 | 2 | 2 | 3 | 1 | 3 | 3 | 2 |
| AP3B1 | O00203;O | 2.60E-10 | 1.17E-10 | -0.189925 | + | 3.09495 | -6.21149 | | 17.3032 | 17.3205 | 17.2982 | 17.4374 | 17.571 | 17.469 | 17.5171 | 17.4918 | 48 | 47 | 47 | 48 | 45 | 47 | 47 | 46 |
| DPYSL2 | Q16555 | 2.50E-12 | 1.64E-12 | -0.189719 | | 0.80735 | -1.62247 | | 20.7291 | 20.7444 | 20.677 | 20.6663 | 20.9677 | 20.9106 | 21.1872 | 20.8011 | 22 | 22 | 22 | 22 | 23 | 24 | 23 | 22 |
| NDUFAF3 | Q9BU61 | 1.79E-11 | 9.95E-12 | -0.189633 | | 0.908787 | -1.79165 | | 16.0208 | 16.0084 | 16.131 | 16.4202 | 16.041 | 16.2961 | 16.0849 | 16.373 | 4 | 5 | 4 | 5 | 4 | 5 | 5 | 5 |
| DKK3 | Q9UBP4 | 2.60E-06 | 7.24E-07 | -0.18936 | | 0.207174 | -0.52162 | | 16.7087 | 16.6286 | 16.6389 | 16.3652 | 17.4175 | 16.0747 | 17.4005 | 16.9828 | 7 | 8 | 6 | 7 | 6 | 7 | 8 | 8 |
| HSP90AB1 | P08238 | 5.57E-18 | 1.95E-17 | -0.189137 | | 0.896942 | -1.77195 | | 21.1815 | 21.2006 | 21.1932 | 21.661 | 21.3916 | 21.198 | 21.2558 | 21.3982 | 52 | 52 | 53 | 51 | 51 | 51 | 50 | 51 |
| RPS13 | P62277 | 0.000776 | 0.000163 | -0.188651 | | 0.80425 | -1.61728 | | 21.3473 | 21.1475 | 21.1205 | 21.185 | 21.6455 | 21.3801 | 21.293 | 21.4652 | 8 | 9 | 8 | 8 | 8 | 8 | 8 | 9 |
| SEC16A | O15027;O | 1.09E-13 | 1.02E-13 | -0.1886 | | 0.920326 | -1.81083 | | 16.166 | 16.1346 | 16.1547 | 16.2578 | 16.1523 | 16.5 | 16.5503 | 16.2416 | 41 | 39 | 39 | 41 | 31 | 41 | 41 | 37 |
| ZMIZ1 | Q9ULJ6 | 9.34E-05 | 2.22E-05 | -0.188439 | | 0.773848 | -1.56627 | | 15.3611 | 15.4001 | 15.1918 | 15.3897 | 15.6432 | 15.3784 | 15.7645 | 15.3548 | 5 | 3 | 3 | 3 | 3 | 3 | 4 | 3 |
| B2M | P61769 | 3.19E-11 | 1.68E-11 | -0.188377 | | 0.22539 | -0.56098 | | 18.039 | 17.5752 | 18.3319 | 17.3572 | 18.5083 | 18.0474 | 18.5321 | 18.407 | 4 | 4 | 4 | 4 | 4 | 4 | 4 | 4 |
| MAP3K3 | Q99759;Q | 0.026406 | 0.004357 | -0.188313 | | 0.296597 | -0.70865 | | 14.587 | 15.1349 | 15.014 | 15.2788 | 14.4646 | 15.0755 | 15.1465 | 15.5362 | 2 | 1 | 1 | 2 | 2 | 1 | 1 | 1 |
| SMN1 | Q16637;Q | 2.84E-07 | 8.79E-08 | -0.188027 | + | 1.57122 | -2.91401 | | 16.6466 | 16.6364 | 16.6321 | 16.7941 | 16.9236 | 16.7807 | 16.9471 | 16.6865 | 4 | 4 | 4 | 4 | 3 | 3 | 5 | 4 |
| SHISA4 | Q96DD7 | 0.00017 | 3.91E-05 | -0.18788 | | 0.836159 | -1.67064 | | 16.3192 | 16.5384 | 16.4511 | 16.8481 | 16.443 | 16.5352 | 16.7611 | 16.5331 | 2 | 2 | 2 | 2 | 1 | 2 | 1 | 1 |
| RPL6 | Q02878 | 1.54E-14 | 1.86E-14 | -0.18754 | + | 1.71229 | -3.16686 | | 19.2297 | 19.4337 | 19.2813 | 19.4498 | 19.5981 | 19.4552 | 19.542 | 19.4673 | 19 | 18 | 19 | 18 | 19 | 19 | 18 | 19 |
| WDR20 | Q8TBZ3;Q | 0.001268 | 0.000258 | -0.187504 | | 0.966164 | -1.88699 | | 14.3723 | 14.4559 | 14.478 | 14.7637 | 14.7286 | 14.5033 | 14.72 | 14.399 | 4 | 5 | 6 | 2 | 4 | 5 | 4 | 5 |
| WRAP53 | Q9BUR4 | 2.51E-15 | 3.83E-15 | -0.187024 | + | 2.54989 | -4.86132 | | 15.287 | 15.2605 | 15.1894 | 15.4484 | 15.4593 | 15.3848 | 15.4998 | 15.3711 | 3 | 2 | 3 | 3 | 2 | 3 | 2 | 3 |
| RIOK3 | O14730 | 4.94E-16 | 9.08E-16 | -0.186966 | | 0.626844 | -1.31626 | | 15.2222 | 15.3095 | 15.299 | 15.3633 | 15.5303 | 15.839 | 15.3608 | 15.226 | 5 | 7 | 6 | 8 | 3 | 7 | 6 | 4 |
| ALG11 | Q2TAA5 | 4.00E-09 | 1.53E-09 | -0.186927 | | 0.580597 | -1.23596 | | 15.4948 | 15.5424 | 15.3922 | 15.2918 | 15.7244 | 15.8712 | 15.881 | 15.5485 | 8 | 8 | 8 | 8 | 6 | 8 | 7 | 8 |
| GAK | O14976 | 1.76E-07 | 5.56E-08 | -0.186921 | | 0.92517 | -1.81888 | | 15.7717 | 15.7657 | 15.7585 | 16.2295 | 15.9544 | 15.7712 | 15.8571 | 15.949 | 18 | 21 | 19 | 22 | 16 | 17 | 21 | 18 |
| TMEM181 | Q9P2C4 | 3.90E-14 | 4.15E-14 | -0.186782 | | 0.332102 | -0.7792 | | 14.9276 | 15.0482 | 15.0399 | 15.6739 | 14.6966 | 15.3312 | 15.3865 | 14.8719 | 3 | 3 | 3 | 3 | 2 | 3 | 4 | 4 |
| HDHD2 | Q9H0R4 | 2.47E-05 | 6.22E-06 | -0.186765 | | 0.248648 | -0.61023 | | 15.7216 | 16.1513 | 16.1621 | 15.7844 | 16.7789 | 16.6477 | 15.7725 | 16.0087 | 7 | 7 | 6 | 7 | 6 | 6 | 6 | 6 |
| ATPAF1 | Q5TC12;Q | 5.16E-07 | 1.55E-07 | -0.186582 | + | 1.39068 | -2.59977 | | 15.8739 | 15.807 | 15.8111 | 15.9469 | 16.2209 | 15.9482 | 15.9545 | 16.0156 | 2 | 3 | 3 | 2 | 1 | 1 | 3 | 3 |
| IGBP1 | P78318 | 2.58E-17 | 7.21E-17 | -0.186242 | | 1.04093 | -2.01118 | | 16.4984 | 16.5356 | 16.5285 | 16.9004 | 16.6795 | 16.6507 | 16.4968 | 16.8079 | 12 | 14 | 14 | 14 | 12 | 12 | 12 | 13 |
| MAP2K4 | P45985;P | 2.13E-05 | 5.41E-06 | -0.18581 | + | 1.34076 | -2.51443 | | 15.9387 | 15.9461 | 15.9637 | 16.111 | 16.2948 | 16.2033 | 16.1038 | 15.9637 | 7 | 9 | 7 | 7 | 8 | 8 | 8 | 10 |
| ZNF148 | Q9UQR1 | 0.000919 | 0.000191 | -0.185725 | | 0.429907 | -0.96543 | | 14.4186 | 14.4793 | 14.6571 | 14.9216 | 14.9026 | 14.1861 | 14.6422 | 14.8677 | 3 | 3 | 3 | 3 | 4 | 3 | 3 | 5 |
| RRAGC | Q9HB90 | 2.67E-10 | 1.20E-10 | -0.185682 | + | 2.00565 | -3.71842 | | 17.0874 | 17.2201 | 17.2288 | 17.3787 | 17.3154 | 17.3752 | 17.4554 | 17.2975 | 12 | 12 | 12 | 12 | 10 | 12 | 12 | 12 |
| RPL9 | P32969 | 8.43E-06 | 2.23E-06 | -0.185391 | + | 1.42419 | -2.6574 | | 19.9707 | 20.1497 | 20.0418 | 20.1659 | 20.4064 | 20.2453 | 20.1776 | 20.2023 | 8 | 8 | 9 | 9 | 9 | 9 | 9 | 9 |
| PPIP5K2 | O43314;O | 1.86E-07 | 5.86E-08 | -0.185212 | + | 2.04988 | -3.80505 | | 16.3492 | 16.2651 | 16.2206 | 16.4387 | 16.5167 | 16.3914 | 16.5506 | 16.42 | 23 | 19 | 19 | 19 | 17 | 19 | 21 | 20 |
| SYNJ1 | O43426 | 6.21E-08 | 2.05E-08 | -0.185183 | | 0.831118 | -1.66222 | | 14.5465 | 14.5553 | 14.5419 | 14.9626 | 14.9035 | 14.5449 | 14.6271 | 14.6274 | 11 | 10 | 10 | 15 | 7 | 10 | 10 | 11 |
| ARL10 | Q8N8L6 | 0.006196 | 0.001092 | -0.185178 | | 0.193821 | -0.49741 | | 14.5013 | 14.5495 | 14.8697 | 14.6385 | NaN | 14.1061 | 15.5477 | 15.009 | 1 | 2 | 2 | 1 | NaN | 2 | 3 | 2 |
| YTHDF2 | Q9Y5A9 | 6.22E-09 | 2.32E-09 | -0.185176 | + | 2.30541 | -4.32608 | | 16.4085 | 16.4211 | 16.3463 | 16.639 | 16.528 | 16.4931 | 16.5849 | 16.6407 | 14 | 15 | 14 | 14 | 13 | 13 | 13 | 14 |
| BPGM | P07738 | 2.14E-10 | 9.77E-11 | -0.185018 | | 0.222023 | -0.55376 | | 15.131 | 15.012 | 14.9697 | 14.7474 | 14.8866 | 15.5922 | 16.0217 | 14.8649 | 8 | 8 | 8 | 6 | 7 | 8 | 8 | 7 |
| COG5 | Q9UP83;Q | 3.10E-14 | 3.40E-14 | -0.185003 | | 0.657775 | -1.36946 | | 15.3705 | 15.4049 | 15.4308 | 15.2737 | 15.8192 | 15.6383 | 15.7575 | 15.4465 | 15 | 17 | 16 | 14 | 15 | 18 | 16 | 16 |
| STK10 | O94804 | 2.66E-11 | 1.42E-11 | -0.184814 | | 0.708726 | -1.45632 | | 16.0011 | 15.9991 | 15.9596 | 16.2751 | 16.4133 | 15.9171 | 15.9809 | 16.2706 | 18 | 18 | 19 | 19 | 18 | 18 | 20 | 19 |
| TERF2IP | Q9NYB0 | 1.98E-10 | 9.09E-11 | -0.184797 | | 0.472376 | -1.04334 | | 14.8317 | 14.9189 | 14.8655 | 14.9569 | 15.5551 | 14.9257 | 14.7855 | 15.0609 | 8 | 6 | 8 | 9 | 3 | 7 | 7 | 7 |
| NSF | P46459 | 6.40E-20 | 4.24E-19 | -0.184756 | | 0.949836 | -1.85987 | | 17.6677 | 17.6739 | 17.6991 | 17.6171 | 17.8036 | 17.9097 | 18.0614 | 17.9333 | 38 | 40 | 42 | 39 | 41 | 39 | 41 | 40 |
| PABPC1 | P11940 | 1.54E-14 | 1.85E-14 | -0.184635 | + | 1.54136 | -2.86136 | | 19.2284 | 19.2919 | 19.26 | 19.5336 | 19.5556 | 19.3085 | 19.3678 | 19.4582 | 29 | 30 | 30 | 30 | 28 | 30 | 30 | 29 |
| IFIT5 | Q13325 | 1.80E-12 | 1.22E-12 | -0.184391 | | 1.09792 | -2.10599 | | 16.1127 | 16.0923 | 16.0943 | 16.3006 | 16.3915 | 16.0449 | 16.27 | 16.4135 | 14 | 13 | 13 | 13 | 13 | 15 | 16 | 15 |
| ALDOA | P04075 | 2.64E-11 | 1.42E-11 | -0.184371 | | 0.951979 | -1.86343 | | 21.0184 | 21.0359 | 20.9804 | 21.2795 | 21.3872 | 21.1867 | 20.9414 | 21.1849 | 35 | 34 | 34 | 35 | 29 | 31 | 32 | 31 |
| SESN3 | P58005 | 1.16E-12 | 8.30E-13 | -0.184366 | | 0.427762 | -0.96145 | | 15.7559 | 15.6965 | 15.664 | 16.3031 | 15.6793 | 16.0503 | 15.9347 | 15.4818 | 1 | 1 | 1 | 3 | 1 | 1 | 1 | 1 |
| FBXO9 | Q9UK97-2 | 0.00172 | 0.000342 | -0.184225 | | 0.375459 | -0.87572 | | 15.4443 | 15.3118 | 15.591 | 15.9616 | 15.1815 | NaN | 15.5928 | 15.797 | 4 | 6 | 4 | 3 | 3 | NaN | 5 | 3 |
| EIF4E | P06730;P | 2.55E-17 | 7.16E-17 | -0.184205 | + | 1.85323 | -3.42711 | | 18.0557 | 18.1188 | 18.1457 | 18.4216 | 18.206 | 18.2985 | 18.2992 | 18.2293 | 9 | 9 | 9 | 8 | 8 | 8 | 8 | 9 |
| COPE | O14579 | 1.11E-08 | 4.02E-09 | -0.184172 | | 0.741227 | -1.51133 | | 18.0803 | 17.8946 | 18.315 | 18.4746 | 18.1776 | 18.117 | 18.3013 | 18.3336 | 15 | 14 | 16 | 16 | 14 | 16 | 16 | 16 |
| GDPGP1 | Q6ZNW5 | 6.30E-05 | 1.53E-05 | -0.1841 | | 0.339452 | -0.79358 | | 14.4097 | 14.2242 | 14.4314 | 13.9387 | 14.8246 | 14.901 | 14.5682 | 14.4634 | 2 | 1 | 2 | 1 | 2 | 2 | 2 | 2 |
| FGGY | Q96C11;Q | 5.48E-05 | 1.34E-05 | -0.184083 | | 0.243355 | -0.59911 | | 14.1532 | 14.2866 | 14.0383 | 13.6263 | 15.0168 | 14.5059 | 14.1614 | 14.4069 | 4 | 5 | 4 | 3 | 4 | 4 | 3 | 5 |
| PYM1 | Q9BRP8;Q | 4.65E-09 | 1.76E-09 | -0.183985 | + | 1.33276 | -2.50082 | | 16.135 | 16.1756 | 16.1861 | 16.2909 | 16.5606 | 16.2499 | 16.3303 | 16.3159 | 9 | 8 | 9 | 8 | 8 | 9 | 9 | 7 |
| MOB3A | Q96BX8 | 7.03E-08 | 2.30E-08 | -0.183883 | | 0.698326 | -1.43866 | | 16.0333 | 16.1007 | 15.9836 | 16.4805 | 16.1892 | 16.2047 | 15.9106 | 16.3303 | 7 | 5 | 8 | 8 | 6 | 5 | 4 | 6 |
| PVR | P15151;P | 0.000672 | 0.000142 | -0.18382 | | 0.692933 | -1.42949 | | 15.7794 | 15.9099 | 15.7914 | 16.2615 | 15.6807 | 16.0252 | 16.0154 | 16.0707 | 6 | 6 | 6 | 6 | 6 | 6 | 6 | 6 |
| RPL7 | P18124 | 1.85E-06 | 5.21E-07 | -0.183309 | + | 1.3359 | -2.50616 | | 19.9913 | 20.268 | 19.9566 | 20.2514 | 20.2507 | 20.2784 | 20.2228 | 20.2731 | 20 | 20 | 21 | 19 | 20 | 20 | 20 | 20 |
| ADPGK | Q9BRR6;Q | 1.69E-10 | 7.82E-11 | -0.183225 | + | 1.35781 | -2.54352 | | 16.4683 | 16.4694 | 16.4534 | 16.7067 | 16.4779 | 16.6932 | 16.5737 | 16.7832 | 9 | 9 | 10 | 9 | 9 | 9 | 10 | 9 |
| RRAS | P10301 | 5.39E-16 | 9.78E-16 | -0.183194 | | 1.1526 | -2.19721 | | 17.5396 | 17.5767 | 17.6805 | 17.613 | 17.8211 | 17.9673 | 17.783 | 17.7263 | 12 | 12 | 12 | 12 | 12 | 12 | 12 | 12 |
| EIPR1 | Q53HC9 | 1.78E-10 | 8.24E-11 | -0.183044 | + | 1.75977 | -3.25361 | | 15.5524 | 15.6183 | 15.613 | 15.7803 | 15.8429 | 15.8119 | 15.832 | 15.621 | 8 | 6 | 7 | 8 | 7 | 8 | 10 | 8 |
| POLR3D | P05423 | 0.003596 | 0.000658 | -0.182775 | | 0.500058 | -1.09334 | | 14.209 | 14.1488 | 14.2526 | 14.3011 | 14.2284 | 14.1977 | 14.3299 | 14.8742 | 4 | 4 | 4 | 2 | 3 | 4 | 2 | 2 |
| EXOC1 | Q9NV70-2 | 2.35E-14 | 2.67E-14 | -0.182567 | + | 3.17303 | -6.42534 | | 17.5168 | 17.5975 | 17.487 | 17.682 | 17.7156 | 17.7531 | 17.717 | 17.7141 | 24 | 25 | 25 | 25 | 22 | 24 | 24 | 23 |
| OTUD4 | Q01804 | 1.38E-10 | 6.47E-11 | -0.182406 | + | 1.58878 | -2.9451 | | 14.895 | 14.8404 | 14.7634 | 14.9402 | 14.9051 | 15.0912 | 15.1194 | 15.021 | 13 | 16 | 16 | 14 | 11 | 14 | 14 | 15 |
| YWHAG | P61981 | 9.07E-06 | 2.39E-06 | -0.182333 | | 0.358917 | -0.83134 | | 19.3858 | 19.3621 | 19.474 | 20.0295 | 19.5044 | 19.0314 | 19.6687 | 19.7142 | 9 | 9 | 9 | 8 | 8 | 8 | 8 | 8 |
| PHKA2 | P46019 | 0.004926 | 0.000881 | -0.182292 | + | 1.56715 | -2.90682 | | 14.3623 | 14.5312 | 14.6106 | 14.6033 | 14.7422 | 14.7271 | 14.6668 | 14.6787 | 3 | 5 | 4 | 4 | 3 | 3 | 3 | 3 |
| TM7SF3 | Q9NS93 | 2.56E-08 | 8.84E-09 | -0.182238 | | 0.606377 | -1.28084 | | 15.7278 | 15.761 | 15.8212 | 16.1266 | 15.5718 | 15.9596 | 16.1705 | 15.9328 | 3 | 3 | 3 | 2 | 2 | 3 | 3 | 2 |
| GALNT1 | Q10472 | 3.38E-19 | 1.76E-18 | -0.182006 | | 0.513471 | -1.11737 | | 15.1686 | 14.9219 | 15.1527 | 15.2664 | 14.8227 | 15.4306 | 15.4294 | 15.3664 | 8 | 7 | 7 | 7 | 5 | 9 | 7 | 7 |
| WDR1 | O75083 | 4.31E-15 | 6.09E-15 | -0.181676 | | 1.01718 | -1.97171 | | 20.316 | 20.3829 | 20.2981 | 20.7151 | 20.5176 | 20.5426 | 20.2901 | 20.5046 | 31 | 31 | 31 | 32 | 30 | 32 | 30 | 32 |
| PKN2 | Q16513;Q | 2.54E-12 | 1.66E-12 | -0.181453 | | 1.06724 | -2.05492 | | 17.0659 | 17.0243 | 17.0063 | 17.3907 | 17.229 | 17.0615 | 17.0703 | 17.3164 | 23 | 22 | 21 | 22 | 19 | 20 | 21 | 23 |
| PAFAH2 | Q99487 | 0.00087 | 0.000181 | -0.181067 | | 0.57707 | -1.22979 | | 14.8272 | 14.579 | 14.9582 | 14.6538 | 15.156 | 15.1312 | 15.018 | 14.887 | 5 | 6 | 4 | 2 | 5 | 4 | 4 | 3 |
| MAN1A2 | O60476 | 9.90E-06 | 2.60E-06 | -0.180977 | + | 2.40816 | -4.54632 | | 15.5953 | 15.6843 | 15.604 | 15.7514 | 15.8463 | 15.7417 | 15.8561 | 15.8485 | 8 | 8 | 8 | 8 | 7 | 7 | 8 | 9 |
| HCFC2 | Q9Y5Z7 | 0.000773 | 0.000162 | -0.180966 | | 0.75154 | -1.52872 | | 14.9144 | 14.9784 | 15.0064 | 15.1399 | 15.4376 | 14.9421 | 14.9996 | 15.2178 | 2 | 4 | 6 | 5 | 2 | 3 | 5 | 3 |
| NOL11 | Q9H8H0 | 1.23E-06 | 3.55E-07 | -0.180645 | + | 1.34496 | -2.5216 | | 15.5096 | 15.4955 | 15.5737 | 15.7672 | 15.7262 | 15.509 | 15.7209 | 15.8112 | 11 | 10 | 9 | 10 | 8 | 10 | 11 | 10 |
| APPL2 | Q8NEU8 | 1.19E-17 | 3.67E-17 | -0.180428 | + | 1.72676 | -3.1932 | | 16.6558 | 16.6425 | 16.6605 | 16.7464 | 16.8051 | 16.9927 | 16.8338 | 16.7888 | 33 | 33 | 33 | 36 | 30 | 34 | 30 | 35 |
| TXLNA | P40222 | 4.92E-18 | 1.76E-17 | -0.180385 | | 0.568153 | -1.21417 | | 16.4442 | 16.4712 | 16.5209 | 17.0457 | 16.5487 | 16.4061 | 16.5493 | 16.746 | 22 | 23 | 23 | 23 | 21 | 23 | 22 | 23 |
| RRP7A | Q9Y3A4 | 1.82E-11 | 1.01E-11 | -0.18036 | | 0.777015 | -1.57159 | | 14.4306 | 14.5145 | 14.5662 | 14.9956 | 14.4991 | 14.6294 | 14.6799 | 14.6166 | 7 | 7 | 7 | 6 | 6 | 6 | 7 | 7 |
| POLR1G | O15446;O | 9.50E-05 | 2.25E-05 | -0.180096 | | 0.606238 | -1.2806 | | 15.739 | 15.5253 | 15.577 | 16.0746 | 15.5612 | 15.5741 | 15.9088 | 15.8504 | 4 | 4 | 4 | 4 | 3 | 3 | 3 | 3 |
| GREM1 | O60565 | 1.14E-20 | 1.00E-19 | -0.179934 | | 0.219044 | -0.54735 | | 17.8205 | 17.8871 | 17.8436 | 17.6477 | 17.7604 | 17.5625 | 18.3188 | 18.8623 | 9 | 9 | 9 | 10 | 7 | 9 | 10 | 10 |
| DCTD | P32321-2 | 1.26E-16 | 2.91E-16 | -0.179841 | | 0.856769 | -1.70504 | | 16.6899 | 16.7564 | 16.7138 | 16.8534 | 16.9566 | 17.167 | 16.8232 | 16.6992 | 10 | 10 | 10 | 9 | 9 | 9 | 10 | 9 |
| DDX20 | Q9UHI6 | 1.32E-14 | 1.62E-14 | -0.179773 | | 1.05937 | -2.04184 | | 14.3094 | 14.3658 | 14.1695 | 14.4629 | 14.4951 | 14.2423 | 14.5732 | 14.5333 | 10 | 6 | 8 | 10 | 6 | 7 | 7 | 8 |
| EI24 | O14681 | 0.001359 | 0.000275 | -0.179689 | + | 1.73029 | -3.19964 | | 15.7453 | 15.7074 | 15.7884 | 15.8799 | 15.7954 | 15.9504 | 16.015 | 15.993 | 7 | 7 | 6 | 6 | 7 | 7 | 7 | 8 |
| UBR4 | Q5T4S7;Q | 2.56E-24 | 7.58E-23 | -0.179586 | + | 2.57598 | -4.92077 | | 16.3668 | 16.3823 | 16.3753 | 16.5674 | 16.5989 | 16.4553 | 16.607 | 16.5435 | 117 | 118 | 118 | 113 | 108 | 114 | 114 | 117 |
| RAB11B | Q15907 | 8.29E-06 | 2.20E-06 | -0.179575 | + | 1.355 | -2.53872 | | 20.3988 | 20.4066 | 20.3629 | 20.7259 | 20.5622 | 20.3975 | 20.5584 | 20.6009 | 13 | 13 | 12 | 12 | 13 | 12 | 12 | 13 |
| ATG13 | O75143;O | 0.000205 | 4.65E-05 | -0.179558 | + | 1.43732 | -2.68008 | | 14.9575 | 15.0603 | 14.9692 | 15.2523 | 15.0694 | 15.3178 | 15.1293 | 15.1074 | 6 | 7 | 7 | 7 | 6 | 6 | 4 | 5 |
| SIGMAR1 | Q99720 | 3.10E-05 | 7.76E-06 | -0.179344 | | 0.596712 | -1.26405 | | 17.3848 | 17.2605 | 17.0851 | 17.2123 | 17.2661 | 17.4166 | 17.7552 | 17.4639 | 4 | 4 | 4 | 4 | 4 | 4 | 4 | 4 |

| Gene names | UniProt IDs | Protein Group P-value | Protein Group Q-value | Log2 Difference NUP214 vs Controls (n=5) | p<0.05 | -log10 p-value | T-test Test statistic | NPC Localization | Log2 MS2_quantity_ NUP214_1 | Log2 MS2_quantity_ NUP214_2 | Log2 MS2_quantity_ NUP214_3 | Log2 MS2_quantity_ Control_1 | Log2 MS2_quantity_ Control_2 | Log2 MS2_quantity_ Control_3 | Log2 MS2_quantity_ Control_4 | Log2 MS2_quantity_ Control_5 | Number Peptides for Quantitation NUP214_1 | Number Peptides for Quantitation NUP214_2 | Number Peptides for Quantitation NUP214_3 | Number Peptides for Quantitation Control_1 | Number Peptides for Quantitation Control_2 | Number Peptides for Quantitation Control_3 | Number Peptides for Quantitation Control_4 | Number Peptides for Quantitation Control_5 |
|---|---|---|---|---|---|---|---|---|---|---|---|---|---|---|---|---|---|---|---|---|---|---|---|---|
| PAPSS2 | O95340;O9 | 4.49E-10 | 1.94E-10 | -0.1793 | | 0.385454 | -0.8821 | | 17.6121 | 17.7088 | 17.6813 | 17.5776 | 17.7487 | 17.764 | 18.4389 | 17.7042 | 24 | 24 | 24 | 24 | 25 | 24 | 25 | 24 |
| SLC7A1 | P30825 | 1.01E-11 | 5.88E-12 | -0.17907 | | 0.560558 | -1.20083 | | 17.0212 | 16.9845 | 17.0227 | 17.5546 | 16.8805 | 17.2734 | 17.0787 | 17.1557 | 5 | 5 | 5 | 6 | 6 | 6 | 5 | 6 |
| NDUFV2 | P19404 | 4.97E-10 | 2.14E-10 | -0.178971 | | 0.804426 | -1.61757 | | 17.8146 | 17.5404 | 17.9003 | 17.8184 | 17.9788 | 17.8783 | 18.1349 | 17.8432 | 8 | 8 | 8 | 8 | 7 | 7 | 8 | 7 |
| MTHFD1L | Q6UB35 | 4.48E-13 | 3.56E-13 | -0.178773 | | 1.2555 | -2.36992 | | 16.5533 | 16.5828 | 16.5576 | 16.6488 | 16.7514 | 16.9496 | 16.7325 | 16.6345 | 25 | 25 | 25 | 23 | 23 | 24 | 25 | 23 |
| RRBP1 | Q9P2E9 | 2.78E-15 | 4.19E-15 | -0.178308 | + | 1.62468 | -3.00899 | | 18.9361 | 18.9111 | 18.9159 | 18.9915 | 19.1568 | 19.1475 | 19.2052 | 18.9959 | 71 | 69 | 70 | 70 | 69 | 71 | 67 | 70 |
| IMP3 | Q9NV31 | 8.08E-05 | 1.93E-05 | -0.177875 | | 0.745351 | -1.51829 | | 16.013 | 16.1457 | 16.1541 | 16.4401 | 16.3959 | 15.9638 | 16.2776 | 16.3333 | 5 | 5 | 5 | 5 | 4 | 5 | 5 | 5 |
| NABP2 | Q9BQ15 | 0.009506 | 0.001635 | -0.177863 | | 0.324593 | -0.7746 | | 14.3077 | 14.1812 | 14.2924 | 14.4559 | 14.9637 | 14.0702 | 14.2634 | NaN | 1 | 2 | 1 | 2 | 1 | 2 | 1 | NaN |
| LIN7A | O14910 | 0.000908 | 0.000188 | -0.177795 | | 0.930074 | -1.82704 | | 15.6882 | 15.5354 | 15.447 | 15.613 | 15.8848 | 15.74 | 15.8572 | 15.5785 | 5 | 4 | 4 | 4 | 4 | 5 | 5 | 4 |
| MAPKAP1 | Q9BPZ7;Q9 | 7.41E-05 | 1.78E-05 | -0.177653 | | 0.611769 | -1.29019 | | 14.4441 | 14.6162 | 14.6583 | 14.8071 | 14.8795 | 14.4676 | 15.0027 | 14.5955 | 3 | 3 | 4 | 3 | 4 | 5 | 4 | 3 |
| PFKP | Q01813 | 5.23E-15 | 7.18E-15 | -0.177555 | | 0.206639 | -0.52046 | | 18.64 | 18.5748 | 18.6135 | 19.7392 | 18.2768 | 18.4479 | 18.6359 | 18.835 | 27 | 27 | 29 | 31 | 27 | 29 | 28 | 27 |
| SLC1A4 | P43007 | 2.52E-11 | 1.36E-11 | -0.177552 | | 0.179558 | -0.46051 | | 16.535 | 16.4413 | 16.554 | 15.8277 | 16.4648 | 17.1273 | 17.497 | 16.5216 | 6 | 7 | 8 | 6 | 6 | 7 | 8 | 7 |
| RNF114 | Q9Y508 | 2.30E-10 | 1.04E-10 | -0.177478 | | 0.592073 | -1.25598 | | 16.4172 | 16.3025 | 16.3062 | 16.7582 | 16.631 | 16.4873 | 16.1424 | 16.5784 | 8 | 7 | 7 | 7 | 8 | 7 | 7 | 7 |
| P4HA3 | Q7Z4N8 | 2.37E-10 | 1.07E-10 | -0.177327 | | 0.544867 | -1.17317 | | 14.3283 | 14.4753 | 14.2256 | 14.7599 | 14.4108 | 14.6949 | 14.5675 | 14.169 | 3 | 4 | 2 | 2 | 1 | 3 | 3 | 3 |
| DNAJA3 | Q96EY1 | 1.99E-08 | 6.97E-09 | -0.177175 | + | 1.38088 | -2.58298 | | 16.5165 | 16.6578 | 16.6506 | 16.8482 | 16.8224 | 16.639 | 16.7302 | 16.8878 | 10 | 9 | 9 | 10 | 7 | 10 | 9 | 9 |
| DDX52 | Q9Y2R4 | 5.74E-08 | 1.91E-08 | -0.177162 | | 0.994805 | -1.93455 | | 15.2357 | 15.1702 | 15.118 | 15.5163 | 15.1437 | 15.2623 | 15.4282 | 15.4085 | 5 | 7 | 8 | 6 | 6 | 6 | 6 | 7 |
| PIGX | Q8TBF5;Q8 | 0.002555 | 0.000487 | -0.176726 | | 0.125138 | -0.33721 | | 14.0297 | 14.0025 | 13.9293 | 14.1203 | 13.5591 | 13.547 | 15.4291 | NaN | 2 | 2 | 2 | 2 | 2 | 1 | 3 | NaN |
| MRPL41 | Q8IXM3 | 1.40E-20 | 1.21E-19 | -0.176709 | | 0.574334 | -1.225 | | 16.325 | 16.1542 | 16.3044 | 16.4074 | 16.8396 | 16.2438 | 16.3257 | 16.3731 | 4 | 4 | 4 | 4 | 3 | 4 | 5 | 5 |
| CSNK2A1 | P68400 | 1.50E-12 | 1.03E-12 | -0.176609 | + | 2.68518 | -5.17493 | | 17.0777 | 17.1264 | 17.0802 | 17.3245 | 17.3002 | 17.1956 | 17.3013 | 17.2352 | 13 | 14 | 14 | 14 | 14 | 14 | 14 | 13 |
| TPT1 | P13693 | 7.10E-17 | 1.75E-16 | -0.176376 | | 0.926876 | -1.82172 | | 18.889 | 18.7428 | 19.0168 | 19.184 | 19.0804 | 19.037 | 18.8496 | 19.1452 | 12 | 11 | 11 | 12 | 10 | 11 | 11 | 11 |
| UBE3C | Q15386 | 2.76E-14 | 3.08E-14 | -0.17634 | + | 1.75486 | -3.2446 | | 15.8496 | 15.8614 | 15.7903 | 16.0618 | 16.0877 | 15.9227 | 15.9082 | 16.0701 | 23 | 24 | 23 | 25 | 23 | 23 | 24 | 22 |
| CTDSP1 | Q9GZU7-2; | 0.009047 | 0.001562 | -0.176247 | | 0.158352 | -0.41629 | | NaN | 16.0293 | 15.3576 | 15.6445 | 15.3493 | 15.5143 | 16.4016 | 16.4388 | NaN | 1 | 3 | 2 | 2 | 3 | 2 | 2 |
| RPS5 | P46782 | 5.53E-10 | 2.36E-10 | -0.175815 | | 0.685022 | -1.41602 | | 21.0827 | 21.1256 | 21.082 | 21.3284 | 21.0683 | 21.0799 | 21.3165 | 21.5697 | 8 | 8 | 8 | 8 | 8 | 8 | 8 | 8 |
| ARHGAP1 | Q07960 | 1.83E-10 | 8.43E-11 | -0.175479 | + | 1.30931 | -2.46095 | | 18.67 | 18.68 | 18.6868 | 18.7691 | 18.7955 | 19.0642 | 18.8129 | 18.8304 | 28 | 27 | 28 | 27 | 24 | 28 | 27 | 28 |
| P4HA1 | P13674-2 | 0.000139 | 3.22E-05 | -0.175448 | | 0.177325 | -0.45549 | | 17.0828 | 16.6575 | 16.7329 | 16.2297 | 17.9321 | 17.0758 | 16.6941 | 17.0675 | 2 | 2 | 2 | 2 | 2 | 2 | 2 | 2 |
| RHBDF1 | Q96CC6 | 0.002934 | 0.000549 | -0.175251 | | 0.890669 | -1.76151 | | 15.6213 | 15.687 | 15.5328 | 15.8625 | 15.7055 | 16.0329 | 15.6643 | 15.6796 | 4 | 4 | 4 | 5 | 3 | 4 | 5 | 4 |
| HEXB | P07686 | 1.57E-12 | 1.08E-12 | -0.175178 | | 0.389414 | -0.8896 | | 18.3784 | 18.3733 | 18.4221 | 18.3736 | 18.4596 | 18.6614 | 19.0909 | 18.2468 | 20 | 20 | 20 | 21 | 20 | 20 | 20 | 21 |
| IRAK1 | P51617;P5 | 0.000935 | 0.000194 | -0.174178 | | 0.460824 | -1.02229 | | 14.7732 | 14.8804 | 14.7298 | 15.1481 | 15.3162 | 14.7697 | 14.62 | 14.9892 | 7 | 5 | 6 | 4 | 4 | 3 | 4 | 5 |
| IBTK | Q9P2D0;Q9 | 0.002156 | 0.00042 | -0.17399 | | 0.471582 | -1.06007 | | 13.7551 | 13.7635 | 13.6897 | 13.6062 | NaN | 14.2086 | 14.0647 | 13.7609 | 4 | 4 | 4 | 4 | NaN | 4 | 6 | 1 |
| MICU1 | Q9BPX6;Q9 | 1.16E-08 | 4.19E-09 | -0.173732 | | 0.800376 | -1.61079 | | 15.4441 | 15.5302 | 15.5136 | 15.9636 | 15.5444 | 15.6115 | 15.5274 | 15.7015 | 12 | 12 | 10 | 10 | 9 | 9 | 11 | 9 |
| RPS29 | P62273 | 0.000689 | 0.000145 | -0.173644 | | 0.613778 | -1.29367 | | 20.1608 | 20.0634 | 20.012 | 20.1864 | 20.6029 | 20.0312 | 20.1396 | 20.3017 | 2 | 2 | 2 | 2 | 2 | 2 | 2 | 2 |
| CTPS1 | P17812 | 2.34E-16 | 4.88E-16 | -0.173621 | | 0.775149 | -1.56846 | | 17.1513 | 17.2399 | 17.1688 | 17.4895 | 17.4119 | 17.0953 | 17.2603 | 17.5444 | 26 | 27 | 26 | 26 | 26 | 26 | 25 | 26 |
| GRAMD1A | Q96CP6;Q9 | 0.005989 | 0.001057 | -0.173285 | | 0.231187 | -0.57335 | | 13.6613 | 13.4488 | 13.721 | 14.143 | 14.4366 | 13.2093 | 13.6175 | 13.512 | 2 | 3 | 2 | 2 | 2 | 1 | 1 | 1 |
| SCO2 | O43819 | 3.10E-05 | 7.74E-06 | -0.173153 | | 0.581613 | -1.23773 | | 15.4726 | 15.5241 | 15.4929 | 15.7303 | 15.8179 | 15.3138 | 15.9102 | 15.5764 | 3 | 3 | 3 | 3 | 1 | 3 | 2 | 3 |
| DHRS4 | Q9BTZ2 | 2.67E-06 | 7.42E-07 | -0.173004 | | 0.044587 | -0.12787 | | 17.2676 | 17.3048 | 17.22 | 15.1145 | 17.2115 | 16.8204 | 21.2282 | 16.8111 | 8 | 8 | 8 | 6 | 7 | 8 | 1 | 8 |
| WARS2 | Q9UGM6 | 1.17E-10 | 5.57E-11 | -0.172824 | | 0.689814 | -1.42418 | | 14.3376 | 14.5315 | 14.5309 | 14.7655 | 14.3208 | 14.7385 | 14.7536 | 14.6191 | 5 | 6 | 6 | 7 | 2 | 6 | 6 | 3 |
| MED14 | O60244 | 0.007319 | 0.00128 | -0.172798 | | 0.487819 | -1.0713 | | 14.7461 | 14.5849 | 14.7212 | 14.7644 | 15.2402 | 14.5476 | 14.7514 | 14.9807 | 7 | 9 | 7 | 8 | 2 | 5 | 6 | 8 |
| MOCS2 | O96007 | 0.000158 | 3.65E-05 | -0.172761 | | 0.403077 | -0.91537 | | 16.0586 | 16.1983 | 16.0168 | 16.2443 | 16.6058 | 15.8641 | 16.0765 | 16.5293 | 5 | 5 | 5 | 5 | 4 | 5 | 5 | 5 |
| SPG11 | Q96JI7 | 2.70E-05 | 6.77E-06 | -0.172483 | | 0.393348 | -0.89704 | | 14.2689 | 14.1962 | 14.1728 | 14.2669 | 14.8465 | 14.401 | 14.4498 | 13.9613 | 6 | 6 | 6 | 3 | 3 | 5 | 3 | 2 |
| SND1 | Q7KZF4 | 3.09E-13 | 2.58E-13 | -0.172192 | + | 2.69354 | -5.19473 | | 19.3617 | 19.4029 | 19.3579 | 19.5974 | 19.5287 | 19.6036 | 19.5213 | 19.4808 | 50 | 50 | 50 | 50 | 48 | 49 | 50 | 49 |
| ATP11C | Q8NB49-3 | 3.42E-06 | 9.42E-07 | -0.172117 | | 0.245491 | -0.6036 | | 15.6149 | 15.654 | 15.5498 | 16.5635 | 15.6854 | 15.8075 | 15.3101 | 15.5254 | 17 | 18 | 17 | 15 | 7 | 15 | 13 | 13 |
| FAS | P25445;P2 | 3.84E-09 | 1.47E-09 | -0.171942 | | 1.24443 | -2.35127 | | 15.308 | 15.3757 | 15.3317 | 15.3949 | 15.3919 | 15.6295 | 15.6372 | 15.4985 | 10 | 10 | 10 | 10 | 9 | 10 | 10 | 10 |
| CORO1B | Q9BR76 | 8.00E-10 | 3.35E-10 | -0.171896 | + | 2.2378 | -4.18464 | | 18.7427 | 18.7495 | 18.696 | 19.0101 | 18.9039 | 18.8349 | 18.876 | 18.8815 | 18 | 18 | 18 | 17 | 18 | 18 | 17 | 18 |
| DNAJC10 | Q8IXB1 | 9.53E-10 | 3.96E-10 | -0.171861 | | 0.978677 | -1.90777 | | 17.3435 | 17.3536 | 17.3604 | 17.3042 | 17.5782 | 17.5691 | 17.7085 | 17.4619 | 15 | 15 | 15 | 14 | 15 | 15 | 15 | 15 |
| DPH6 | Q7L8W6 | 0.007439 | 0.001299 | -0.171858 | | 0.355141 | -0.83568 | | 14.1895 | 13.7771 | 13.6935 | 14.2162 | 14.0754 | 13.6704 | NaN | 14.2723 | 3 | 2 | 2 | 3 | 2 | 2 | NaN | 2 |
| CRAT | P43155;P4 | 5.99E-12 | 3.64E-12 | -0.171855 | | 0.849571 | -1.69303 | | 16.4163 | 16.3373 | 16.3586 | 16.5432 | 16.7723 | 16.4681 | 16.3196 | 16.6097 | 16 | 16 | 17 | 16 | 14 | 16 | 16 | 15 |
| TUBB6 | Q9BUF5 | 1.71E-06 | 4.83E-07 | -0.171751 | | 1.00621 | -1.9535 | | 21.5262 | 21.5657 | 21.5389 | 21.9281 | 21.7784 | 21.6271 | 21.5456 | 21.6975 | 17 | 16 | 17 | 16 | 16 | 17 | 16 | 17 |
| MRPL10 | Q7Z7H8;Q7 | 3.40E-11 | 1.78E-11 | -0.171642 | | 0.388162 | -0.88723 | | 16.8177 | 16.765 | 16.5573 | 17.118 | 17.2458 | 16.4549 | 16.8142 | 16.792 | 2 | 2 | 3 | 3 | 2 | 2 | 2 | 2 |
| MED17 | Q9NVC6 | 0.001873 | 0.000369 | -0.171592 | | 0.466766 | -1.051 | | 16.5151 | 16.8935 | 16.6318 | 16.6038 | 16.7177 | NaN | 17.0205 | 17.065 | 8 | 8 | 6 | 7 | 5 | NaN | 5 | 5 |
| GRIPAP1 | Q4V328 | 5.92E-11 | 2.97E-11 | -0.171588 | + | 2.08814 | -3.88079 | | 14.9774 | 14.9011 | 14.9523 | 15.0349 | 15.1176 | 15.1954 | 15.1691 | 15.0588 | 26 | 28 | 25 | 23 | 18 | 24 | 22 | 23 |
| WASF2 | Q9Y6W5 | 0.00034 | 7.48E-05 | -0.171473 | | 1.20644 | -2.28738 | | 17.2654 | 17.2825 | 17.3647 | 17.329 | 17.6571 | 17.4211 | 17.4845 | 17.4865 | 10 | 10 | 10 | 10 | 10 | 9 | 10 | 10 |
| CALU | O43852;O4 | 6.37E-09 | 2.37E-09 | -0.171418 | | 0.491309 | -1.0776 | | 20.0183 | 20.1143 | 20.1606 | 20.397 | 19.9102 | 20.5406 | 20.4141 | 20.084 | 31 | 32 | 32 | 32 | 32 | 33 | 32 | 33 |
| COMMD9 | Q9P000 | 1.36E-09 | 5.55E-10 | -0.171351 | | 0.51484 | -1.11982 | | 15.946 | 15.4509 | 15.9168 | 15.8581 | 15.8484 | 15.8153 | 15.9729 | 16.2181 | 11 | 9 | 11 | 9 | 7 | 9 | 11 | 9 |
| GPD1L | Q8N335 | 7.37E-13 | 5.50E-13 | -0.17135 | | 0.549006 | -1.18048 | | 14.628 | 14.7017 | 14.5957 | 14.6625 | 14.9789 | 14.6369 | 14.6321 | 15.1554 | 7 | 6 | 8 | 5 | 5 | 4 | 4 | 7 |
| HABP4 | Q5JVS0 | 4.29E-06 | 1.17E-06 | -0.171112 | | 0.460228 | -1.0212 | | 16.4836 | 16.5988 | 16.3395 | 17.0903 | 16.5325 | 16.5408 | 16.3999 | 16.6619 | 6 | 7 | 7 | 7 | 6 | 7 | 7 | 7 |
| UTP18 | Q9Y5J1 | 1.52E-18 | 6.32E-18 | -0.170989 | | 0.866259 | -1.72086 | | 15.1873 | 15.1321 | 15.1173 | 15.4648 | 15.4306 | 15.1065 | 15.1724 | 15.4084 | 10 | 9 | 10 | 12 | 9 | 9 | 8 | 10 |
| SLC35B3 | Q9H1N7 | 1.49E-13 | 1.34E-13 | -0.170908 | | 0.396662 | -0.90329 | | 16.701 | 16.6373 | 16.5596 | 16.4379 | 17.0879 | 16.6324 | 17.1716 | 16.688 | 3 | 2 | 3 | 2 | 2 | 3 | 3 | 3 |
| SMG6 | Q86US8 | 2.31E-05 | 5.85E-06 | -0.170805 | | 1.09269 | -2.09728 | | 14.8355 | 14.9512 | 14.9399 | 14.8982 | 15.1327 | 15.1015 | 15.2438 | 15.0221 | 5 | 6 | 6 | 5 | 4 | 5 | 5 | 5 |
| KIFAP3 | Q92845 | 0.000497 | 0.000107 | -0.170694 | | 0.910734 | -1.79489 | | 15.6343 | 15.806 | 15.7797 | 15.696 | 16.045 | 16.0473 | 15.8938 | 15.8714 | 6 | 5 | 6 | 4 | 5 | 4 | 6 | 6 |
| EGFR | P00533 | 7.62E-11 | 3.72E-11 | -0.170548 | | 0.374632 | -0.8615 | | 16.1991 | 16.1874 | 16.18 | 16.7018 | 16.6445 | 16.1983 | 15.8944 | 16.3579 | 37 | 36 | 36 | 38 | 35 | 35 | 33 | 35 |
| XPO6 | Q96QU8 | 2.59E-12 | 1.69E-12 | -0.170517 | | 0.481304 | -1.05952 | | 15.3269 | 15.4085 | 15.3798 | 15.7053 | 15.7332 | 15.106 | 15.4585 | 15.7083 | 12 | 12 | 12 | 14 | 12 | 7 | 12 | 10 |
| CHMP1A | Q9HD42 | 3.69E-05 | 9.15E-06 | -0.17039 | | 0.436245 | -0.97715 | | 16.7945 | 16.693 | 16.7661 | 17.3569 | 16.715 | 16.6649 | 16.7973 | 17.0738 | 3 | 4 | 4 | 4 | 3 | 3 | 4 | 4 |
| TMEM43 | Q9BTV4 | 3.17E-13 | 2.63E-13 | -0.169928 | + | 1.58395 | -2.93654 | | 18.9046 | 18.962 | 18.9165 | 19.1133 | 19.147 | 19.2128 | 18.9648 | 19.0502 | 20 | 20 | 20 | 20 | 20 | 20 | 20 | 20 |
| EXOC7 | Q9UPT5;Q9 | 5.86E-15 | 7.90E-15 | -0.169652 | + | 2.14698 | -3.99879 | | 16.1389 | 16.2285 | 16.1699 | 16.4485 | 16.3098 | 16.2855 | 16.3322 | 16.3678 | 26 | 27 | 28 | 25 | 24 | 27 | 28 | 25 |
| HNRNPAB | Q99729-2; | 4.02E-06 | 1.10E-06 | -0.169035 | + | 1.74726 | -3.23068 | | 18.3737 | 18.509 | 18.3764 | 18.6409 | 18.5346 | 18.4966 | 18.6467 | 18.6252 | 13 | 13 | 14 | 13 | 12 | 13 | 13 | 13 |
| DYNC1LI2 | O43237 | 9.72E-07 | 2.83E-07 | -0.168976 | | 1.29336 | -2.43392 | | 17.3179 | 17.36 | 17.2962 | 17.4437 | 17.4063 | 17.6511 | 17.5759 | 17.3915 | 19 | 19 | 19 | 21 | 20 | 18 | 19 | 20 |
| HEATR1 | Q9H583 | 2.56E-24 | 7.58E-23 | -0.168581 | + | 1.33972 | -2.51266 | | 15.4275 | 15.4219 | 15.4328 | 15.6933 | 15.5177 | 15.4533 | 15.5987 | 15.7169 | 39 | 40 | 43 | 37 | 31 | 34 | 36 | 37 |
| GSTO1 | P78417 | 1.23E-08 | 4.43E-09 | -0.16855 | | 1.01813 | -1.97329 | | 20.1298 | 20.2273 | 20.2575 | 20.6093 | 20.3132 | 20.266 | 20.3422 | 20.3365 | 19 | 19 | 19 | 19 | 19 | 15 | 19 | 19 |
| ALG9 | Q9H6U8;Q9 | 9.53E-10 | 3.96E-10 | -0.167405 | | 0.894536 | -1.76794 | | 15.4408 | 15.516 | 15.6203 | 15.6679 | 15.664 | 15.6098 | 15.9446 | 15.5792 | 3 | 3 | 4 | 4 | 4 | 3 | 4 | 4 |
| DHX37 | Q8IY37 | 7.36E-11 | 3.61E-11 | -0.167363 | | 1.23564 | -2.33647 | | 14.7838 | 14.8065 | 14.649 | 14.884 | 14.7901 | 14.8527 | 15.0326 | 15.0097 | 10 | 11 | 13 | 10 | 10 | 11 | 11 | 12 |
| MT-ND2 | P03891 | 3.66E-08 | 1.24E-08 | -0.167061 | | 0.452852 | -1.00771 | | 16.822 | 16.4312 | 16.3797 | 16.6257 | 16.4116 | 16.6985 | 16.9987 | 16.8222 | 1 | 2 | 2 | 1 | 1 | 1 | 1 | 2 |
| EMC1 | Q8N766;Q8 | 8.68E-15 | 1.11E-14 | -0.166998 | + | 2.72654 | -5.27344 | | 16.8244 | 16.8552 | 16.8127 | 16.9856 | 17.0488 | 16.9166 | 17.0251 | 17.0127 | 34 | 34 | 34 | 34 | 33 | 34 | 35 | 34 |
| WDR46 | O15213 | 0.000604 | 0.000128 | -0.16694 | + | 1.96913 | -3.64762 | | 15.1272 | 15.1332 | 15.0032 | 15.2908 | 15.2345 | 15.2033 | 15.2104 | 15.3352 | 11 | 11 | 12 | 11 | 9 | 8 | 10 | 10 |
| EXOC3 | O60645 | 4.53E-14 | 4.75E-14 | -0.166872 | + | 2.16249 | -4.0302 | | 16.3311 | 16.2953 | 16.3451 | 16.5445 | 16.3868 | 16.5223 | 16.5393 | 16.4605 | 17 | 19 | 21 | 21 | 14 | 19 | 20 | 18 |
| NUDT19 | A8MXV4 | 2.83E-05 | 7.10E-06 | -0.166752 | | 0.239693 | -0.59139 | | 16.1573 | 16.2653 | 16.1434 | 15.8124 | 16.8398 | 16.5081 | 16.7134 | 15.9032 | 6 | 6 | 5 | 5 | 4 | 6 | 5 | 4 |
| PNO1 | Q9NRX1 | 6.41E-11 | 3.19E-11 | -0.16657 | | 1.29263 | -2.43268 | | 16.5742 | 16.6478 | 16.5899 | 16.7752 | 16.7803 | 16.6132 | 16.7564 | 16.9275 | 5 | 4 | 6 | 6 | 6 | 5 | 5 | 6 |
| CYHR1 | Q6ZMK1;Q6 | 1.73E-07 | 5.48E-08 | -0.166569 | | 0.402089 | -0.91351 | | 14.126 | 13.9558 | 13.8401 | 13.6784 | 14.0422 | 14.3479 | 14.3622 | 14.272 | 3 | 3 | 5 | 3 | 3 | 4 | 3 | 3 |
| TUT7 | Q5VYS8;Q5 | 0.000672 | 0.000142 | -0.166452 | | 0.834278 | -1.6675 | | 15.103 | 15.3677 | 15.1045 | 15.3346 | 15.5475 | 15.1904 | 15.3826 | 15.3357 | 17 | 16 | 12 | 13 | 11 | 11 | 18 | 13 |
| HSP90B1 | P14625 | 1.34E-11 | 7.60E-12 | -0.166177 | + | 2.30627 | -4.32789 | | 20.9369 | 20.9249 | 20.9087 | 21.1282 | 21.1706 | 21.006 | 21.0877 | 21.0558 | 68 | 66 | 67 | 66 | 64 | 67 | 66 | 67 |

| Gene names | UniProt IDs | Protein Group P-value | Protein Group Q-value | Log2 Difference NUP214 vs Controls (n=5) | p<0.05 | -log10 p-value | T-test Test statistic | NPC Localization | Log2 MS2_quantity_NUP214_1 | Log2 MS2_quantity_NUP214_2 | Log2 MS2_quantity_NUP214_3 | Log2 MS2_quantity_Control_1 | Log2 MS2_quantity_Control_2 | Log2 MS2_quantity_Control_3 | Log2 MS2_quantity_Control_4 | Log2 MS2_quantity_Control_5 | Number Peptides for Quantitation NUP214_1 | Number Peptides for Quantitation NUP214_2 | Number Peptides for Quantitation NUP214_3 | Number Peptides for Quantitation Control_1 | Number Peptides for Quantitation Control_2 | Number Peptides for Quantitation Control_3 | Number Peptides for Quantitation Control_4 | Number Peptides for Quantitation Control_5 |
|---|---|---|---|---|---|---|---|---|---|---|---|---|---|---|---|---|---|---|---|---|---|---|---|---|
| MARCKS | P29966 | 0.000297 | 6.60E-05 | -0.166165 | | 0.347865 | -0.80996 | | 17.9676 | 17.7353 | 18.3804 | 18.2516 | 17.8606 | 18.438 | 18.4164 | 18.003 | 15 | 14 | 15 | 14 | 15 | 16 | 14 | 15 |
| DNAJA1 | P31689 | 5.50E-11 | 2.77E-11 | -0.165578 | | 1.04336 | -2.01522 | | 16.9411 | 16.971 | 16.9304 | 17.1685 | 17.2357 | 16.8782 | 17.1505 | 17.1326 | 19 | 19 | 19 | 20 | 17 | 19 | 18 | 19 |
| ENOPH1 | Q9UHY7 | 6.52E-08 | 2.14E-08 | -0.165536 | | 0.911678 | -1.79646 | | 17.3908 | 17.285 | 17.4498 | 17.4315 | 17.6061 | 17.4139 | 17.7578 | 17.4944 | 6 | 6 | 6 | 6 | 6 | 6 | 6 | 6 |
| RAB22A | Q9UL26 | 8.07E-11 | 3.93E-11 | -0.165499 | | 0.926271 | -1.82072 | | 16.9737 | 16.9428 | 16.8537 | 17.0452 | 17.2731 | 16.8747 | 17.1067 | 17.1449 | 8 | 7 | 8 | 7 | 7 | 7 | 9 | 7 |
| AUP1 | Q9Y679 | 1.17E-07 | 3.74E-08 | -0.165245 | | 0.641106 | -1.34084 | | 15.5215 | 15.55 | 15.4722 | 15.7621 | 15.5341 | 15.4014 | 15.8946 | 15.8068 | 10 | 9 | 9 | 10 | 8 | 9 | 7 | 9 |
| TMED3 | Q9Y3Q3 | 2.18E-11 | 1.19E-11 | -0.1649 | | 0.340858 | -0.79633 | | 15.7258 | 15.7936 | 15.8164 | 15.5474 | 15.9306 | 16.2131 | 16.3586 | 15.668 | 5 | 6 | 5 | 6 | 6 | 6 | 6 | 6 |
| HPS3 | Q969F9;Q9 | 3.56E-07 | 1.09E-07 | -0.164851 | + | 1.39034 | -2.5992 | | 14.5976 | 14.6115 | 14.5419 | 14.8499 | 14.8427 | 14.6381 | 14.7673 | 14.6444 | 10 | 11 | 10 | 9 | 8 | 9 | 9 | 9 |
| COPB1 | P53618 | 4.37E-16 | 8.22E-16 | -0.164649 | + | 2.41982 | -4.57174 | | 18.4439 | 18.459 | 18.4158 | 18.6344 | 18.5645 | 18.6461 | 18.6547 | 18.5215 | 53 | 52 | 51 | 54 | 50 | 51 | 52 | 54 |
| CWF19L1 | Q69YN2 | 0.000244 | 5.47E-05 | -0.16437 | + | 1.97635 | -3.66157 | | 15.1853 | 15.1514 | 15.2158 | 15.2723 | 15.4602 | 15.3121 | 15.3714 | 15.3267 | 5 | 7 | 7 | 7 | 5 | 6 | 6 | 5 |
| RPS27A | P62979 | 6.60E-14 | 6.61E-14 | -0.164241 | | 0.533991 | -1.15391 | | 22.3868 | 22.4667 | 22.3385 | 22.5291 | 22.954 | 22.3254 | 22.4767 | 22.5228 | 12 | 12 | 12 | 13 | 13 | 13 | 13 | 13 |
| TMEM63A | O94886 | 7.20E-05 | 1.73E-05 | -0.164025 | | 1.24203 | -2.34722 | | 15.0507 | 15.0416 | 15.0384 | 15.1903 | 15.2365 | 15.354 | 15.2282 | 15.0291 | 4 | 5 | 5 | 4 | 6 | 5 | 5 | 4 |
| TRAPPC10 | P48553 | 0.004716 | 0.000847 | -0.163921 | | 0.330856 | -0.77675 | | 13.3912 | 13.0354 | 13.0146 | 12.9171 | 13.1918 | 13.8013 | 13.3491 | 13.2957 | 1 | 2 | 2 | 3 | 1 | 3 | 1 | 2 |
| CYTH3 | O43739;O4 | 8.89E-05 | 2.12E-05 | -0.163871 | | 0.774252 | -1.56695 | | 15.3545 | 15.3609 | 15.4516 | 15.6051 | 15.2838 | 15.7415 | 15.5121 | 15.6219 | 4 | 5 | 4 | 5 | 4 | 4 | 5 | 5 |
| VAMP3 | Q15836 | 1.07E-08 | 3.87E-09 | -0.16374 | | 0.279153 | -0.67329 | | 18.6226 | 18.4877 | 18.6172 | 18.9732 | 18.6027 | 18.1509 | 18.7479 | 19.2231 | 5 | 5 | 5 | 5 | 5 | 5 | 5 | 5 |
| SETD7 | Q8WTS6 | 2.62E-15 | 3.98E-15 | -0.163676 | + | 1.78246 | -3.2954 | | 16.8343 | 16.7599 | 16.7273 | 16.8484 | 16.9365 | 16.9267 | 17.0534 | 16.9226 | 8 | 8 | 7 | 7 | 6 | 8 | 8 | 8 |
| GPAA1 | O43292 | 0.000112 | 2.62E-05 | -0.16345 | + | 1.37447 | -2.57201 | | 16.3857 | 16.4779 | 16.3911 | 16.6858 | 16.5236 | 16.5285 | 16.6925 | 16.478 | 7 | 8 | 9 | 6 | 9 | 6 | 8 | 8 |
| STX2 | P32856;P3 | 6.94E-20 | 4.52E-19 | -0.163363 | | 0.634876 | -1.33011 | | 15.0125 | 15.0699 | 15.0306 | 14.9401 | 15.1351 | 15.3544 | 15.4565 | 15.1189 | 6 | 7 | 5 | 7 | 4 | 7 | 6 | 5 |
| CDC37L1 | Q7L3B6 | 1.39E-10 | 6.52E-11 | -0.16301 | | 0.620876 | -1.30595 | | 13.8327 | 13.89 | 13.8051 | 13.7449 | 14.1893 | 14.1171 | 14.1568 | 13.8198 | 7 | 6 | 7 | 4 | 4 | 6 | 4 | 4 |
| DDX3Y | O15523 | 2.79E-19 | 1.51E-18 | -0.162848 | + | 1.61143 | -2.98536 | | 18.7 | 18.7525 | 18.7106 | 18.9622 | 18.9703 | 18.838 | 18.7568 | 18.8921 | 30 | 30 | 31 | 34 | 30 | 31 | 35 | 30 |
| STOM | P27105 | 2.25E-15 | 3.49E-15 | -0.162804 | | 1.25367 | -2.36684 | | 18.8397 | 18.8261 | 18.841 | 18.9687 | 18.9117 | 19.0622 | 19.1648 | 18.8847 | 10 | 11 | 10 | 10 | 10 | 10 | 12 | 10 |
| SQLE | Q14534 | 1.09E-10 | 5.17E-11 | -0.162775 | | 0.212927 | -0.53413 | | 14.8772 | 14.919 | 15.0242 | 15.6433 | 15.2518 | 14.7749 | 14.3997 | 15.445 | 8 | 6 | 6 | 8 | 5 | 8 | 5 | 9 |
| VPS29 | Q9UBQ0 | 3.63E-11 | 1.89E-11 | -0.162659 | + | 4.2362 | -9.99609 | | 18.1133 | 18.1334 | 18.1174 | 18.2901 | 18.326 | 18.2792 | 18.2615 | 18.2634 | 11 | 11 | 11 | 11 | 11 | 10 | 10 | 10 |
| RAB3B | P20337 | 0.000181 | 4.13E-05 | -0.162362 | | 0.248222 | -0.60933 | | 17.6042 | 17.4359 | 17.431 | 17.9499 | 17.7556 | 17.2024 | 17.1899 | 18.166 | 8 | 7 | 8 | 7 | 7 | 7 | 6 | 7 |
| DERL2 | Q9GZP9 | 7.32E-05 | 1.76E-05 | -0.162126 | | 0.925395 | -1.81926 | | 19.3122 | 19.3391 | 19.2373 | 19.6614 | 19.4489 | 19.255 | 19.4863 | 19.4402 | 2 | 1 | 1 | 2 | 1 | 1 | 3 | 1 |
| TRIM32 | Q13049 | 5.47E-15 | 7.45E-15 | -0.161994 | | 0.94513 | -1.85205 | | 15.105 | 15.1475 | 15.0215 | 15.4733 | 15.1458 | 15.1206 | 15.2569 | 15.2701 | 20 | 19 | 19 | 21 | 20 | 19 | 19 | 20 |
| CD2AP | Q9Y5K6 | 4.61E-07 | 1.40E-07 | -0.161917 | + | 1.41336 | -2.63876 | | 15.7561 | 15.7833 | 15.809 | 15.9262 | 15.8894 | 15.8166 | 16.0652 | 16.0261 | 16 | 18 | 17 | 18 | 16 | 18 | 18 | 16 |
| MARCHF5 | Q9NX47 | 1.34E-09 | 5.48E-10 | -0.161802 | + | 1.31102 | -2.46385 | | 15.9612 | 16.002 | 16.0282 | 16.0991 | 16.0701 | 16.0937 | 16.3277 | 16.2042 | 8 | 8 | 8 | 8 | 8 | 8 | 7 | 8 |
| EEF1D | P29692 | 1.66E-08 | 5.88E-09 | -0.161801 | + | 2.15621 | -4.01745 | | 20.1721 | 20.1507 | 20.225 | 20.3673 | 20.3332 | 20.2418 | 20.3923 | 20.3875 | 17 | 15 | 16 | 15 | 15 | 16 | 17 | 16 |
| CACUL1 | Q86Y37 | 7.94E-07 | 2.34E-07 | -0.16174 | | 0.295283 | -0.706 | | 13.4104 | 12.7544 | 13.6451 | 13.4895 | 13.3904 | 13.2311 | 13.2979 | 13.7497 | 4 | 4 | 4 | 4 | 2 | 1 | 2 | 3 |
| TMEM192 | Q8IY95;Q8 | 3.48E-06 | 9.57E-07 | -0.161475 | | 0.553793 | -1.18892 | | 15.9376 | 15.9636 | 15.7309 | 15.9183 | 16.0551 | 15.8341 | 16.3811 | 16.0055 | 6 | 6 | 5 | 6 | 6 | 6 | 7 | 6 |
| GLCE | O94923 | 2.14E-12 | 1.43E-12 | -0.161314 | | 0.515701 | -1.12136 | | 15.6602 | 15.5513 | 15.2963 | 15.9666 | 15.6692 | 15.5976 | 15.4056 | 15.6805 | 2 | 4 | 5 | 4 | 2 | 3 | 3 | 5 |
| TMEM106B | Q9NUM4 | 2.35E-09 | 9.25E-10 | -0.161224 | | 0.477729 | -1.05305 | | 16.6768 | 16.787 | 16.8386 | 16.8718 | 16.7816 | 16.9034 | 17.3582 | 16.7286 | 7 | 8 | 8 | 7 | 8 | 6 | 9 | 9 |
| UBE2M | P61081 | 5.91E-08 | 1.96E-08 | -0.160993 | | 0.877967 | -1.74036 | | 18.1663 | 18.1932 | 18.1062 | 18.2668 | 18.2301 | 18.3585 | 18.5582 | 18.1675 | 8 | 8 | 8 | 7 | 7 | 8 | 7 | 8 |
| SH2B1 | Q9NRF2;Q9 | 0.00016 | 3.70E-05 | -0.160788 | | 0.201733 | -0.50973 | | 14.0332 | 13.7713 | 13.7842 | 13.2663 | 14.6877 | 13.861 | 14.1205 | 14.1828 | 3 | 4 | 3 | 3 | 1 | 3 | 2 | 2 |
| BMPR2 | Q13873 | 0.002094 | 0.000409 | -0.160335 | | 0.259285 | -0.63983 | | 14.4887 | 14.5277 | 14.1235 | 14.9158 | NaN | 14.4418 | 14.0484 | 14.7551 | 1 | 1 | 2 | 4 | NaN | 2 | 2 | 2 |
| AP4E1 | Q9UPM8 | 0.000239 | 5.38E-05 | -0.159826 | | 0.466523 | -1.03269 | | 14.0741 | 14.0349 | 14.1366 | 14.0831 | 14.0715 | 14.508 | 14.5348 | 14.0109 | 6 | 8 | 7 | 6 | 4 | 9 | 6 | 6 |
| MTAP | Q13126 | 1.75E-05 | 4.49E-06 | -0.159523 | | 0.380618 | -0.87291 | | 17.4322 | 17.4137 | 17.3906 | 17.7728 | 17.1746 | 17.907 | 17.6671 | 17.3367 | 18 | 18 | 19 | 20 | 17 | 20 | 20 | 17 |
| MED16 | Q9Y2X0;Q9 | 0.00102 | 0.00021 | -0.159471 | | 0.567497 | -1.21301 | | 13.6488 | 13.9684 | 13.9855 | 14.2385 | 14.1863 | 13.8376 | 13.9437 | 13.9291 | 6 | 5 | 5 | 4 | 3 | 5 | 5 | 5 |
| HDLBP | Q00341 | 1.29E-12 | 9.05E-13 | -0.159432 | | 1.09083 | -2.09418 | | 18.8742 | 18.9538 | 18.9078 | 18.9189 | 19.0303 | 19.2189 | 19.1788 | 19.01 | 68 | 66 | 67 | 66 | 67 | 67 | 68 | 67 |
| EIF3E | P60228 | 1.86E-10 | 8.58E-11 | -0.159375 | | 1.20953 | -2.29257 | | 18.6743 | 18.6289 | 18.6151 | 18.8644 | 18.8559 | 18.6034 | 18.79 | 18.8801 | 19 | 19 | 19 | 19 | 19 | 18 | 19 | 19 |
| MYL12B;MYL12A | O14950;P1 | 0.000354 | 7.79E-05 | -0.15926 | + | 2.70143 | -5.21348 | | 22.2757 | 22.2543 | 22.3691 | 22.451 | 22.4999 | 22.471 | 22.4283 | 22.4444 | 4 | 5 | 4 | 4 | 4 | 4 | 4 | 4 |
| TOMM34 | Q15785 | 1.48E-09 | 6.00E-10 | -0.159239 | | 0.390581 | -0.89181 | | 16.8897 | 16.8868 | 16.888 | 17.2523 | 17.1583 | 16.6323 | 16.8449 | 17.3492 | 13 | 13 | 13 | 14 | 15 | 14 | 14 | 15 |
| DENR | O43583 | 5.88E-05 | 1.43E-05 | -0.159163 | | 0.736057 | -1.5026 | | 16.9225 | 17.1062 | 17.0971 | 17.0172 | 17.179 | 17.4598 | 17.2075 | 17.142 | 9 | 9 | 9 | 9 | 7 | 9 | 9 | 9 |
| TMX3 | Q96JJ7 | 2.63E-13 | 2.24E-13 | -0.159049 | | 1.01396 | -1.96636 | | 17.686 | 17.7185 | 17.7597 | 17.8191 | 18.0007 | 17.7985 | 18.0431 | 17.7408 | 18 | 16 | 18 | 16 | 15 | 17 | 18 | 18 |
| CCNY | Q8ND76 | 8.82E-14 | 8.56E-14 | -0.158908 | | 0.48926 | -1.0739 | | 15.6389 | 15.9975 | 15.9806 | 15.8418 | 15.96 | 16.375 | 16.0204 | 15.959 | 5 | 6 | 6 | 4 | 3 | 4 | 4 | 4 |
| WIPI1 | Q5MNZ9;Q | 1.74E-09 | 6.95E-10 | -0.158845 | | 1.26493 | -2.38584 | | 16.038 | 16.1731 | 16.1374 | 16.1667 | 16.2587 | 16.4012 | 16.3521 | 16.1964 | 9 | 9 | 9 | 8 | 7 | 8 | 7 | 9 |
| BRIX1 | Q8TDN6 | 2.46E-16 | 5.07E-16 | -0.158551 | + | 1.52845 | -2.83869 | | 16.2868 | 16.3634 | 16.2876 | 16.3973 | 16.3777 | 16.465 | 16.5895 | 16.526 | 15 | 16 | 16 | 16 | 14 | 16 | 17 | 15 |
| PPP2R1B | P30154;P3 | 0.000518 | 0.000111 | -0.158257 | | 0.814848 | -1.63502 | | 18.6115 | 18.6164 | 18.7451 | 18.9387 | 18.7317 | 18.6027 | 18.8288 | 18.9776 | 9 | 8 | 8 | 11 | 10 | 10 | 10 | 11 |
| FXR1 | P51114 | 4.49E-10 | 1.94E-10 | -0.158121 | | 1.18272 | -2.2476 | | 16.5416 | 16.5567 | 16.5623 | 16.8692 | 16.6182 | 16.5939 | 16.6809 | 16.7958 | 28 | 28 | 27 | 28 | 26 | 28 | 26 | 27 |
| NLN | Q9BYT8 | 3.65E-13 | 2.98E-13 | -0.158063 | | 0.766809 | -1.55444 | | 16.2828 | 16.3068 | 16.337 | 16.6503 | 16.5609 | 16.4769 | 16.1988 | 16.4477 | 33 | 32 | 32 | 33 | 29 | 32 | 31 | 32 |
| PSMD2 | Q13200 | 7.16E-13 | 5.36E-13 | -0.157964 | + | 1.61519 | -2.99206 | | 17.9666 | 18.0259 | 18.025 | 18.2941 | 18.1266 | 18.1138 | 18.082 | 18.2025 | 40 | 41 | 42 | 42 | 37 | 42 | 41 | 41 |
| SGCE | O43556;O4 | 1.83E-06 | 5.17E-07 | -0.157882 | | 0.238512 | -0.58889 | | 15.5867 | 14.963 | 15.8752 | 15.1662 | 15.8588 | 15.9283 | 15.6957 | 15.5152 | 2 | 2 | 2 | 2 | 2 | 2 | 2 | 2 |
| TRAPPC2;TRAPPC2;TI | P0DI81;P0 | 9.06E-07 | 2.64E-07 | -0.157837 | | 0.575035 | -1.22623 | | 15.7787 | 15.912 | 16.2038 | 16.3062 | 16.1727 | 16.0905 | 15.8909 | 16.1532 | 3 | 2 | 2 | 3 | 1 | 1 | 1 | 2 |
| GPN1 | Q9HCN4;Q | 1.20E-05 | 3.12E-06 | -0.157756 | | 0.817363 | -1.63923 | | 14.98 | 14.9589 | 14.8758 | 15.2713 | 15.0579 | 15.1092 | 14.8558 | 15.1857 | 5 | 4 | 4 | 4 | 3 | 4 | 3 | 4 |
| BCR | P11274;P1 | 4.20E-06 | 1.15E-06 | -0.157729 | | 0.376289 | -0.86466 | | 15.2681 | 15.4703 | 15.2979 | 15.7869 | 15.6539 | 15.4616 | 15.0166 | 15.5968 | 6 | 4 | 7 | 7 | 3 | 7 | 3 | 8 |
| GSR | P00390;P0 | 1.92E-08 | 6.75E-09 | -0.157649 | | 0.717819 | -1.47174 | | 17.5392 | 17.5734 | 17.6209 | 17.7423 | 17.9869 | 17.7304 | 17.4856 | 17.7322 | 14 | 15 | 15 | 15 | 15 | 14 | 14 | 15 |
| RTN4IP1 | Q8WWV3;( | 0.00946 | 0.001628 | -0.157599 | | 0.33188 | -0.77876 | | 14.7161 | 14.7887 | 14.5372 | 14.638 | 15.1729 | 14.3746 | 15.0573 | 14.9484 | 3 | 3 | 2 | 2 | 1 | 2 | 2 | 2 |
| DNAJC25 | Q9H1X3 | 2.56E-05 | 6.45E-06 | -0.157467 | | 0.548634 | -1.17982 | | 13.693 | 14.0873 | 14.019 | 13.8932 | 13.9569 | 14.0908 | 14.264 | 14.2477 | 4 | 5 | 4 | 4 | 3 | 5 | 4 | 3 |
| SLC38A7 | Q9NVC3 | 2.04E-13 | 1.79E-13 | -0.157114 | | 0.408295 | -0.92516 | | 16.3023 | 16.185 | 16.237 | 16.2958 | 16.0196 | 16.6111 | 16.7356 | 16.3307 | 5 | 3 | 5 | 5 | 4 | 5 | 5 | 4 |
| TM2D1 | Q9BX74 | 2.78E-06 | 7.70E-07 | -0.156685 | | 0.200792 | -0.50766 | | 14.1955 | 13.7948 | 14.1601 | 13.6126 | 14.286 | 14.196 | 14.9574 | 13.9823 | 2 | 1 | 2 | 1 | 1 | 1 | 2 | 1 |
| RPL29 | P47914 | 8.51E-13 | 6.26E-13 | -0.156519 | | 0.674706 | -1.39842 | | 18.8657 | 18.9913 | 18.8437 | 19.0605 | 19.3321 | 19.096 | 18.908 | 18.8871 | 3 | 3 | 3 | 3 | 3 | 3 | 3 | 3 |
| PARVB | Q9HBI1 | 0.000271 | 6.04E-05 | -0.15649 | | 0.318972 | -0.75332 | | 16.6721 | 16.7041 | 16.7544 | 16.9094 | 16.8374 | 17.4188 | 16.6695 | 16.4984 | 6 | 5 | 4 | 5 | 6 | 6 | 4 | 6 |
| TIMMDC1 | Q9NPL8 | 4.31E-14 | 4.55E-14 | -0.156489 | + | 1.49246 | -2.77577 | | 14.7264 | 14.6919 | 14.6313 | 14.8012 | 14.7745 | 14.7558 | 14.9143 | 14.9527 | 9 | 8 | 9 | 10 | 8 | 9 | 9 | 9 |
| RPS9 | P46781 | 1.22E-12 | 8.65E-13 | -0.156389 | + | 2.32811 | -4.37417 | | 20.19 | 20.3162 | 20.2048 | 20.3545 | 20.4055 | 20.4464 | 20.3849 | 20.3756 | 15 | 15 | 14 | 15 | 15 | 14 | 15 | 15 |
| CAB39 | Q9Y376 | 9.45E-16 | 1.63E-15 | -0.156314 | | 0.45244 | -1.00695 | | 16.5995 | 16.6847 | 16.6109 | 17.1522 | 16.884 | 16.5248 | 16.5547 | 16.8245 | 15 | 15 | 16 | 16 | 15 | 15 | 15 | 15 |
| SEC62 | Q99442 | 0.000187 | 4.27E-05 | -0.156195 | | 0.935307 | -1.83573 | | 17.3227 | 17.3622 | 17.3333 | 17.6645 | 17.5855 | 17.3185 | 17.523 | 17.3865 | 8 | 8 | 8 | 7 | 7 | 8 | 7 | 7 |
| GSTCD | Q8NEC7 | 0.006388 | 0.001124 | -0.156154 | | 0.380002 | -0.87173 | | 14.2294 | 14.3208 | 13.6641 | 14.2987 | 14.1723 | 14.2672 | 13.9801 | 14.4198 | 3 | 3 | 4 | 2 | 1 | 3 | 3 | 2 |
| DPAGT1 | Q9H3H5;Q | 5.39E-05 | 1.32E-05 | -0.155958 | | 0.383241 | -0.87789 | | 16.0663 | 16.2978 | 16.1535 | 15.9233 | 16.3395 | 16.416 | 16.715 | 16.2487 | 3 | 3 | 3 | 3 | 3 | 3 | 3 | 3 |
| RFT1 | Q96AA3 | 0.000372 | 8.15E-05 | -0.155937 | | 0.401492 | -0.91239 | | 16.4038 | 16.2711 | 16.1663 | 16.019 | 16.3157 | 16.5851 | 16.7136 | 16.5483 | 5 | 6 | 4 | 5 | 5 | 6 | 5 | 5 |
| HMGCS1 | Q01581 | 1.61E-06 | 4.56E-07 | -0.155925 | | 0.151086 | -0.39546 | | 15.737 | 15.8378 | 15.8068 | 16.7896 | 15.9868 | 15.1162 | 15.5153 | 16.3412 | 13 | 12 | 14 | 16 | 11 | 8 | 13 | 14 |
| PMM1 | Q92871 | 2.66E-09 | 1.04E-09 | -0.155752 | | 0.47324 | -1.0449 | | 15.8326 | 15.749 | 15.8805 | 16.2717 | 16.1347 | 15.6247 | 15.9217 | 15.9296 | 8 | 7 | 7 | 8 | 8 | 6 | 9 | 9 |
| PWP2 | Q15269 | 1.90E-12 | 1.28E-12 | -0.155518 | | 0.919597 | -1.80962 | | 15.5342 | 15.4477 | 15.5314 | 15.8428 | 15.708 | 15.4869 | 15.5572 | 15.7049 | 18 | 18 | 18 | 18 | 15 | 17 | 16 | 17 |
| DYNC1H1 | Q14204 | 2.56E-24 | 7.58E-23 | -0.155489 | + | 2.0991 | -3.90264 | | 18.9069 | 18.9318 | 18.8997 | 19.0847 | 19.1724 | 18.9984 | 19.0458 | 19.0401 | 237 | 237 | 238 | 238 | 233 | 234 | 237 | 238 |
| MED4 | Q9NPJ6 | 8.68E-06 | 2.29E-06 | -0.155439 | | 0.4047 | -0.91842 | | 13.8692 | 13.8553 | 14.3445 | 14.2791 | 13.8387 | 14.178 | 14.3783 | 14.2181 | 2 | 3 | 4 | 4 | 2 | 4 | 3 | 4 |
| NDUFAF4 | Q9P032 | 1.87E-06 | 5.27E-07 | -0.155349 | | 0.50547 | -1.10305 | | 15.3085 | 15.091 | 15.2745 | 15.5811 | 15.336 | 15.0761 | 15.2949 | 15.6119 | 4 | 5 | 5 | 4 | 5 | 4 | 6 | 6 |
| ABR | Q12979;Q1 | 8.88E-10 | 3.70E-10 | -0.155036 | | 0.962072 | -1.88019 | | 16.2115 | 16.3507 | 16.2827 | 16.4288 | 16.573 | 16.5144 | 16.2314 | 16.4358 | 15 | 18 | 19 | 16 | 13 | 16 | 15 | 16 |
| RNF13 | O43567 | 0.000283 | 6.30E-05 | -0.15483 | | 0.257509 | -0.62872 | | 14.1757 | 14.1535 | 14.1782 | 13.7308 | 14.1951 | 14.36 | 14.8631 | 14.4708 | 3 | 4 | 5 | 4 | 3 | 5 | 5 | 3 |
| PDF | Q9HBH1 | 0.004254 | 0.000769 | -0.154311 | | 0.957137 | -1.872 | | 16.4755 | 16.648 | 16.3993 | 16.5697 | 16.6356 | 16.7862 | 16.5608 | 16.7573 | 4 | 3 | 4 | 4 | 3 | 2 | 4 | 3 |

| Gene names | UniProt IDs | Protein Group P-value | Protein Group Q-value | Log2 Difference NUP214 vs Controls (n=5) | p<0.05 | -log10 p-value | T-test Test statistic | NPC Localization | Log2 MS2_quantity_NUP214_1 | Log2 MS2_quantity_NUP214_2 | Log2 MS2_quantity_NUP214_3 | Log2 MS2_quantity_Control_1 | Log2 MS2_quantity_Control_2 | Log2 MS2_quantity_Control_3 | Log2 MS2_quantity_Control_4 | Log2 MS2_quantity_Control_5 | Number Peptides for Quantitation NUP214_1 | Number Peptides for Quantitation NUP214_2 | Number Peptides for Quantitation NUP214_3 | Number Peptides for Quantitation Control_1 | Number Peptides for Quantitation Control_2 | Number Peptides for Quantitation Control_3 | Number Peptides for Quantitation Control_4 | Number Peptides for Quantitation Control_5 |
|---|---|---|---|---|---|---|---|---|---|---|---|---|---|---|---|---|---|---|---|---|---|---|---|---|
| VPS33A | Q96AX1 | 6.89E-09 | 2.56E-09 | -0.154272 | + | 1.69072 | -3.12772 | | 16.0862 | 16.1557 | 16.0868 | 16.2117 | 16.358 | 16.299 | 16.29 | 16.1605 | 19 | 19 | 17 | 18 | 18 | 16 | 14 | 16 |
| FCHSD2 | O94868;O9 | 0.022098 | 0.003665 | -0.154055 | | 0.235647 | -0.58283 | | 14.5485 | 15.2745 | 14.3845 | 15.1525 | 14.8837 | 14.9819 | 14.4001 | 15.0313 | 3 | 3 | 1 | 2 | 3 | 1 | 3 | 2 |
| COPA | P53621 | 3.60E-21 | 3.71E-20 | -0.15398 | + | 2.1845 | -4.07499 | | 18.7564 | 18.806 | 18.7254 | 18.9355 | 18.8652 | 18.9604 | 18.9733 | 18.8486 | 72 | 72 | 71 | 67 | 69 | 69 | 69 | 68 |
| MGMT | P16455 | 4.89E-07 | 1.48E-07 | -0.153641 | | 0.667715 | -1.38647 | | 15.7359 | 15.5141 | 15.7674 | 16.0142 | 15.6483 | 15.7305 | 15.9694 | 15.7682 | 6 | 5 | 6 | 5 | 5 | 5 | 4 | 5 |
| RPL7L1 | Q6DKI1 | 1.04E-06 | 3.01E-07 | -0.153632 | + | 2.08323 | -3.87103 | | 15.8296 | 15.881 | 15.9112 | 15.9449 | 15.9945 | 16.0432 | 16.1014 | 16.0539 | 10 | 8 | 8 | 10 | 7 | 10 | 11 | 9 |
| WDR55 | Q9H6Y2 | 1.98E-20 | 1.61E-19 | -0.153551 | | 0.609493 | -1.28625 | | 14.7975 | 14.8765 | 14.8568 | 15.0556 | 15.1516 | 14.6508 | 15.0844 | 15.0431 | 9 | 10 | 9 | 10 | 7 | 9 | 9 | 9 |
| FNBP1 | Q96RU3-2; | 7.31E-11 | 3.59E-11 | -0.153546 | | 0.776831 | -1.57128 | | 15.1587 | 15.2622 | 15.1691 | 15.611 | 15.3845 | 15.2945 | 15.2282 | 15.2331 | 13 | 14 | 12 | 15 | 13 | 13 | 12 | 13 |
| SLC35B1 | P78383 | 2.80E-05 | 7.02E-06 | -0.153474 | | 0.313184 | -0.74183 | | 14.9121 | 14.9177 | 15.1284 | 14.715 | 14.9168 | 15.2697 | 15.5859 | 15.2104 | 3 | 3 | 3 | 3 | 3 | 2 | 3 | 3 |
| RP2 | O75695 | 1.93E-07 | 6.06E-08 | -0.153374 | + | 1.56776 | -2.9079 | | 16.6888 | 16.7679 | 16.733 | 16.9167 | 16.9976 | 16.8914 | 16.7758 | 16.8347 | 9 | 9 | 10 | 10 | 8 | 9 | 9 | 10 |
| SIRT5 | Q9NXA8;Q9 | 7.98E-13 | 5.90E-13 | -0.153352 | | 0.805015 | -1.61856 | | 15.3914 | 15.3048 | 15.5853 | 15.4675 | 15.4466 | 15.6725 | 15.5928 | 15.7233 | 4 | 4 | 5 | 5 | 3 | 5 | 4 | 4 |
| FSTL1 | Q12841 | 8.47E-15 | 1.09E-14 | -0.153204 | | 0.245665 | -0.60397 | | 17.6113 | 17.5977 | 17.6404 | 17.1741 | 17.6635 | 18.0351 | 18.2971 | 17.6785 | 11 | 11 | 11 | 10 | 11 | 11 | 11 | 11 |
| TOX4 | O94842;O9 | 7.99E-11 | 3.89E-11 | -0.1531 | + | 2.02688 | -3.75989 | | 15.4248 | 15.5011 | 15.4298 | 15.696 | 15.5787 | 15.5924 | 15.5314 | 15.6264 | 7 | 7 | 7 | 7 | 4 | 4 | 5 | 5 |
| ANKMY2 | Q8IV38 | 1.20E-09 | 4.94E-10 | -0.152955 | | 0.355547 | -0.82484 | | 14.6269 | 14.6643 | 14.6605 | 14.9421 | 15.1877 | 14.3471 | 14.7044 | 14.8363 | 9 | 8 | 8 | 9 | 6 | 8 | 9 | 7 |
| PDZD8 | Q8NEN9 | 6.01E-14 | 6.08E-14 | -0.152945 | | 0.833828 | -1.66675 | | 14.824 | 14.9249 | 14.926 | 15.0301 | 15.1582 | 14.7938 | 15.1072 | 15.1335 | 7 | 7 | 8 | 9 | 7 | 7 | 8 | 9 |
| EBNA1BP2 | Q99848 | 1.25E-16 | 2.89E-16 | -0.152939 | + | 1.42635 | -2.66114 | | 16.4262 | 16.4197 | 16.4215 | 16.5803 | 16.4544 | 16.5414 | 16.7213 | 16.5796 | 10 | 10 | 10 | 10 | 8 | 9 | 10 | 9 |
| BPNT1 | O95861 | 1.10E-11 | 6.36E-12 | -0.152864 | + | 1.99834 | -3.7042 | | 17.2904 | 17.3459 | 17.3163 | 17.5374 | 17.398 | 17.4481 | 17.5434 | 17.425 | 14 | 13 | 13 | 13 | 13 | 14 | 13 | 13 |
| PTPN11 | Q06124-1 | 1.06E-14 | 1.33E-14 | -0.152784 | | 0.778195 | -1.57357 | | 16.7665 | 16.712 | 16.7321 | 16.7367 | 16.7474 | 17.0364 | 17.0829 | 16.8449 | 27 | 29 | 27 | 26 | 26 | 27 | 28 | 26 |
| EMG1 | Q92979 | 7.49E-14 | 7.39E-14 | -0.152746 | | 1.06111 | -2.04472 | | 16.0027 | 16.1537 | 15.9786 | 16.2317 | 16.3428 | 16.0537 | 16.1556 | 16.2049 | 11 | 11 | 10 | 11 | 8 | 10 | 11 | 11 |
| MLF2 | Q15773 | 0.000504 | 0.000108 | -0.152714 | | 0.378211 | -0.86832 | | 15.6144 | 15.6646 | 15.5441 | 16.2171 | 15.5387 | 15.6736 | 15.5069 | 15.8658 | 5 | 4 | 5 | 5 | 4 | 4 | 5 | 4 |
| ZC3HAV1 | Q7Z2W4 | 9.51E-18 | 3.09E-17 | -0.15255 | + | 1.74758 | -3.23126 | | 16.5488 | 16.6603 | 16.6377 | 16.8752 | 16.7905 | 16.7057 | 16.7323 | 16.737 | 31 | 32 | 32 | 31 | 29 | 30 | 31 | 30 |
| TOR1A | O14656 | 1.05E-10 | 5.01E-11 | -0.152538 | | 1.06277 | -2.04748 | | 16.6381 | 16.6134 | 16.6413 | 16.6479 | 16.9311 | 16.6874 | 16.8927 | 16.7583 | 11 | 12 | 11 | 11 | 12 | 11 | 12 | 12 |
| COMMD3 | Q9UBI1 | 1.99E-12 | 1.34E-12 | -0.152509 | | 0.227334 | -0.56513 | | 16.4519 | 16.0985 | 16.4874 | 15.7626 | 16.5373 | 16.7052 | 16.8461 | 16.6409 | 8 | 7 | 8 | 6 | 7 | 7 | 7 | 7 |
| ITSN2 | Q9NZM3;Q | 6.55E-09 | 2.44E-09 | -0.152192 | + | 1.31554 | -2.47154 | | 15.0435 | 15.0125 | 15.0603 | 15.2736 | 15.3104 | 15.1818 | 15.0652 | 15.1239 | 11 | 13 | 10 | 12 | 8 | 11 | 10 | 10 |
| PDCD11 | Q14690 | 5.43E-13 | 4.20E-13 | -0.152035 | + | 1.3553 | -2.53924 | | 15.5411 | 15.5769 | 15.5266 | 15.7032 | 15.7228 | 15.541 | 15.7216 | 15.8127 | 40 | 42 | 42 | 42 | 33 | 41 | 42 | 38 |
| BROX | Q5VW32 | 5.78E-12 | 3.52E-12 | -0.151755 | | 0.873292 | -1.73257 | | 16.5878 | 16.4599 | 16.492 | 16.5142 | 16.8648 | 16.7055 | 16.5526 | 16.688 | 9 | 9 | 9 | 9 | 9 | 9 | 9 | 9 |
| SRSF10 | O75494;O7 | 2.82E-07 | 8.75E-08 | -0.151566 | | 0.783438 | -1.58238 | | 16.6025 | 16.5915 | 16.6151 | 16.6851 | 16.9802 | 16.5422 | 16.7671 | 16.7984 | 7 | 8 | 7 | 7 | 7 | 7 | 7 | 8 |
| RABGAP1L | Q5R372;Q5 | 1.35E-07 | 4.29E-08 | -0.15143 | | 0.480545 | -1.05815 | | 15.0546 | 14.977 | 15.0254 | 15.3795 | 15.1229 | 14.8474 | 15.0723 | 15.4301 | 13 | 14 | 12 | 13 | 12 | 10 | 11 | 14 |
| OXSR1 | O95747 | 3.24E-11 | 1.70E-11 | -0.150866 | | 0.654865 | -1.36447 | | 17.3055 | 17.2853 | 17.2966 | 17.2552 | 17.4977 | 17.3151 | 17.7306 | 17.4347 | 18 | 17 | 18 | 14 | 16 | 18 | 16 | 18 |
| UTP14A | Q9BVJ6 | 5.13E-07 | 1.55E-07 | -0.150772 | + | 1.37358 | -2.57047 | | 15.4951 | 15.5112 | 15.4716 | 15.762 | 15.6451 | 15.498 | 15.6905 | 15.6216 | 15 | 16 | 17 | 16 | 13 | 15 | 13 | 16 |
| CC2D1B | Q5T0F9-2 | 5.08E-08 | 1.69E-08 | -0.150364 | | 0.585226 | -1.24404 | | 15.79 | 15.7025 | 15.7323 | 16.2442 | 15.8618 | 15.8069 | 15.7774 | 15.7693 | 15 | 14 | 16 | 14 | 9 | 12 | 13 | 11 |
| CALHM2 | Q9HA72 | 4.22E-06 | 1.15E-06 | -0.150128 | | 0.495677 | -1.08546 | | 14.4067 | 14.236 | 14.405 | 14.1761 | 14.45 | 14.5808 | 14.7864 | 14.5036 | 3 | 3 | 4 | 3 | 4 | 3 | 5 | 4 |
| NOSIP | Q9Y314 | 1.58E-13 | 1.42E-13 | -0.150126 | | 0.825841 | -1.6534 | | 15.2032 | 15.14 | 15.2063 | 15.5452 | 15.3442 | 15.2728 | 15.3707 | 15.1336 | 9 | 8 | 9 | 8 | 7 | 9 | 11 | 8 |
| ENG | P17813;P1 | 1.17E-05 | 3.05E-06 | -0.15007 | | 0.68125 | -1.40959 | | 17.9108 | 17.9305 | 17.9833 | 17.9553 | 18.1054 | 17.8761 | 18.295 | 18.2262 | 13 | 14 | 14 | 13 | 12 | 13 | 15 | 13 |
| ORC3 | Q9UBD5;Q | 0.000106 | 2.50E-05 | -0.149978 | | 0.42202 | -0.95078 | | 14.0735 | 14.2648 | 14.19 | 14.2224 | 14.4077 | 14.0956 | 14.1704 | 14.7342 | 8 | 8 | 6 | 6 | 5 | 3 | 8 | 5 |
| MAT2A | P31153 | 4.12E-17 | 1.08E-16 | -0.149947 | | 1.14324 | -2.18157 | | 18.0955 | 18.1582 | 18.1067 | 18.445 | 18.2101 | 18.2658 | 18.1421 | 18.2874 | 14 | 14 | 14 | 13 | 14 | 13 | 13 | 14 |
| FAM160B1 | Q5W0V3 | 2.16E-09 | 8.51E-10 | -0.149908 | | 0.578839 | -1.23288 | | 16.4662 | 16.4776 | 16.4496 | 16.3361 | 16.803 | 16.563 | 16.8269 | 16.543 | 13 | 15 | 15 | 15 | 13 | 12 | 15 | 14 |
| CENPC | Q03188 | 0.005368 | 0.000954 | -0.149673 | | 0.112555 | -0.31049 | | 14.3946 | 13.9721 | 13.9525 | 13.5363 | 15.1121 | 14.1198 | NaN | NaN | 2 | 3 | 3 | 1 | 1 | 1 | NaN | NaN |
| RPS10 | P46783 | 5.90E-07 | 1.76E-07 | -0.149663 | + | 1.66438 | -3.08018 | | 21.379 | 21.2546 | 21.2973 | 21.4261 | 21.4364 | 21.3792 | 21.5492 | 21.5089 | 7 | 7 | 7 | 7 | 7 | 7 | 7 | 7 |
| NIPSNAP3A | Q9UFN0 | 5.27E-13 | 4.09E-13 | -0.149615 | | 0.564564 | -1.20787 | | 16.7758 | 16.9169 | 16.7843 | 17.2637 | 16.758 | 17.0848 | 16.9111 | 16.8587 | 11 | 11 | 11 | 11 | 10 | 10 | 12 | 11 |
| VPS39 | Q96JC1;Q9 | 2.30E-15 | 3.56E-15 | -0.149404 | + | 1.43691 | -2.67936 | | 15.5331 | 15.5074 | 15.5211 | 15.5703 | 15.7669 | 15.6856 | 15.75 | 15.5769 | 21 | 23 | 22 | 23 | 16 | 21 | 24 | 23 |
| RPS16 | P62249 | 5.61E-06 | 1.51E-06 | -0.149388 | + | 1.67922 | -3.10693 | | 21.1323 | 21.1458 | 21.1068 | 21.1558 | 21.3782 | 21.2767 | 21.2858 | 21.2921 | 11 | 11 | 11 | 11 | 11 | 11 | 11 | 11 |
| WBP4 | O75554;O7 | 2.96E-05 | 7.41E-06 | -0.149343 | | 0.369748 | -0.85216 | | 15.2433 | 15.4162 | 15.443 | 15.7726 | 15.8 | 15.4685 | 15.4354 | 15.1076 | 4 | 4 | 4 | 4 | 3 | 3 | 4 | 3 |
| TSTA3 | Q13630 | 4.49E-13 | 3.56E-13 | -0.149005 | | 0.3749 | -0.86201 | | 17.6519 | 17.6155 | 17.6082 | 17.6767 | 17.4397 | 18.0934 | 18.0603 | 17.601 | 11 | 12 | 10 | 11 | 11 | 12 | 11 | 10 |
| MTMR6 | Q9Y217 | 5.06E-13 | 3.95E-13 | -0.148887 | + | 2.24025 | -4.18971 | | 15.7027 | 15.7693 | 15.7393 | 15.8739 | 15.9159 | 15.8338 | 15.9649 | 15.8413 | 25 | 25 | 24 | 21 | 21 | 23 | 23 | 23 |
| FLOT1 | O75955 | 3.29E-07 | 1.01E-07 | -0.14886 | | 0.496641 | -1.0872 | | 17.3617 | 17.3522 | 17.3328 | 17.8822 | 17.4806 | 17.2706 | 17.4056 | 17.4498 | 21 | 23 | 22 | 23 | 22 | 22 | 20 | 22 |
| RBX1 | P62877 | 1.44E-07 | 4.59E-08 | -0.148842 | | 0.552199 | -1.18611 | | 16.6809 | 16.3911 | 16.8136 | 16.6955 | 16.9038 | 16.5618 | 16.8679 | 16.8577 | 3 | 3 | 3 | 3 | 3 | 3 | 3 | 3 |
| ABHD11 | Q8NFV4 | 4.92E-05 | 1.21E-05 | -0.14881 | | 0.412599 | -0.93321 | | 16.0415 | 16.0054 | 16.1603 | 16.344 | 16.2474 | 16.1511 | 15.8216 | 16.5255 | 7 | 7 | 8 | 8 | 6 | 6 | 7 | 7 |
| STRIP1 | Q5VSL9 | 1.85E-11 | 1.02E-11 | -0.148674 | + | 1.43633 | -2.67836 | | 15.7986 | 15.7124 | 15.7483 | 15.7991 | 16.0171 | 15.9288 | 15.9347 | 15.8292 | 17 | 17 | 17 | 15 | 11 | 15 | 16 | 15 |
| SIDT2 | Q8NBJ9;Q8 | 3.53E-05 | 8.77E-06 | -0.148519 | | 0.383495 | -0.87838 | | 15.8265 | 15.8118 | 15.6505 | 15.6122 | 15.9262 | 16.2222 | 16.139 | 15.6575 | 5 | 5 | 6 | 4 | 5 | 6 | 5 | 6 |
| RAB6A | P20340-2 | 1.04E-06 | 3.01E-07 | -0.148212 | + | 1.85321 | -3.42707 | | 19.44 | 19.443 | 19.34 | 19.61 | 19.5956 | 19.4577 | 19.5602 | 19.5558 | 9 | 10 | 9 | 9 | 9 | 9 | 9 | 9 |
| EIF3I | Q13347 | 1.99E-14 | 2.31E-14 | -0.147856 | + | 1.57085 | -2.91335 | | 17.9638 | 18.0471 | 18.0227 | 18.2711 | 18.0943 | 18.0761 | 18.1538 | 18.1999 | 17 | 17 | 17 | 17 | 16 | 16 | 17 | 16 |
| ARHGAP29 | Q52LW3 | 1.84E-16 | 4.02E-16 | -0.147839 | | 0.314762 | -0.74497 | | 15.8784 | 15.984 | 15.8893 | 16.1138 | 16.3733 | 15.526 | 16.03 | 16.2821 | 30 | 32 | 32 | 30 | 27 | 18 | 28 | 32 |
| ADI1 | Q9BV57 | 1.81E-14 | 2.13E-14 | -0.147661 | | 0.75614 | -1.53647 | | 16.6026 | 16.3762 | 16.7427 | 16.8647 | 16.7454 | 16.6831 | 16.7054 | 16.6088 | 9 | 9 | 9 | 9 | 9 | 9 | 9 | 10 |
| PPIA | P62937 | 7.45E-12 | 4.45E-12 | -0.147506 | | 0.361444 | -0.83621 | | 22.6489 | 22.5629 | 22.5517 | 22.5473 | 23.2475 | 22.6173 | 22.5561 | 22.7086 | 14 | 14 | 14 | 14 | 14 | 14 | 13 | 14 |
| RBM42 | Q9BTD8;Q9 | 0.002217 | 0.00043 | -0.147496 | | 0.858191 | -1.70741 | | 16.5369 | 16.7814 | 16.6605 | 16.7643 | 16.7455 | 16.6817 | 16.9783 | 16.8655 | 5 | 5 | 5 | 5 | 6 | 5 | 5 | 5 |
| PDXK | O00764 | 4.23E-13 | 3.38E-13 | -0.147446 | | 0.510306 | -1.11171 | | 18.397 | 18.4283 | 18.4373 | 18.3631 | 18.642 | 18.8111 | 18.718 | 18.3073 | 10 | 11 | 9 | 9 | 10 | 9 | 9 | 9 |
| TSC2 | P49815;P4 | 1.80E-11 | 1.00E-11 | -0.147291 | + | 2.91666 | -5.74346 | | 15.2959 | 15.246 | 15.2846 | 15.4513 | 15.3879 | 15.3781 | 15.4298 | 15.467 | 25 | 28 | 24 | 28 | 20 | 23 | 26 | 26 |
| COPG1 | Q9Y678 | 4.55E-17 | 1.18E-16 | -0.147233 | + | 1.7701 | -3.27262 | | 18.2162 | 18.2622 | 18.2262 | 18.4181 | 18.2846 | 18.4429 | 18.442 | 18.3229 | 48 | 47 | 47 | 46 | 45 | 47 | 46 | 46 |
| NECTIN2 | Q92692 | 5.42E-12 | 3.32E-12 | -0.147054 | | 0.89805 | -1.77379 | | 16.517 | 16.4386 | 16.3836 | 16.647 | 16.7981 | 16.5294 | 16.5134 | 16.4791 | 7 | 7 | 7 | 7 | 7 | 9 | 7 | 6 |
| YBX1 | P67809 | 0.000259 | 5.80E-05 | -0.147023 | | 0.529831 | -1.14652 | | 19.3022 | 19.3287 | 19.5298 | 19.6336 | 19.1964 | 19.6955 | 19.5483 | 19.5959 | 8 | 9 | 8 | 8 | 9 | 8 | 8 | 8 |
| LMAN1 | P49257 | 2.38E-16 | 4.94E-16 | -0.146899 | | 0.468707 | -1.03666 | | 18.5089 | 18.5011 | 18.5299 | 18.3992 | 18.606 | 18.9024 | 18.9124 | 18.481 | 19 | 18 | 20 | 18 | 18 | 20 | 18 | 17 |
| RIT1 | Q92963;Q9 | 6.06E-07 | 1.81E-07 | -0.146778 | | 0.384069 | -0.87947 | | 17.1866 | 17.1775 | 17.1771 | 17.7065 | 17.3873 | 17.4039 | 16.9535 | 17.1847 | 3 | 3 | 3 | 3 | 2 | 2 | 3 | 2 |
| LYAR | Q9NX58 | 5.98E-14 | 6.06E-14 | -0.146743 | | 0.462213 | -1.02483 | | 14.4678 | 14.6359 | 14.5736 | 15.0059 | 14.4459 | 14.4957 | 14.7934 | 14.7884 | 9 | 11 | 9 | 10 | 6 | 8 | 7 | 8 |
| MKRN2 | Q9H000 | 2.03E-09 | 8.05E-10 | -0.146739 | | 0.800319 | -1.61069 | | 16.9577 | 16.932 | 16.9024 | 16.897 | 17.0739 | 17.268 | 17.1812 | 16.967 | 7 | 9 | 10 | 10 | 7 | 10 | 8 | 9 |
| ZNF593 | O00488 | 0.001643 | 0.000328 | -0.146724 | | 0.817516 | -1.63948 | | 15.3563 | 15.3573 | 15.283 | 15.5713 | 15.3072 | 15.3319 | 15.6169 | 15.5673 | 1 | 1 | 2 | 2 | 1 | 1 | 2 | 1 |
| GOLGA7 | Q7Z5G4;Q7 | 0.001797 | 0.000356 | -0.146696 | | 0.667907 | -1.3868 | | 17.5141 | 17.6261 | 17.6197 | 17.9297 | 17.7435 | 17.4607 | 17.7293 | 17.8036 | 2 | 2 | 2 | 1 | 2 | 2 | 2 | 2 |
| TMPPE | Q6ZT21 | 0.001526 | 0.000307 | -0.146646 | | 0.275735 | -0.66631 | | 15.2991 | 15.2898 | 15.2292 | 15.5494 | 15.4688 | 15.7835 | 15.4955 | 14.7994 | 3 | 1 | 1 | 2 | 1 | 2 | 3 | 1 |
| TRMT2A | Q8IZ69;Q8 | 1.55E-08 | 5.51E-09 | -0.146541 | | 0.901359 | -1.77929 | | 15.1706 | 15.1609 | 15.1723 | 15.3583 | 15.1258 | 15.2728 | 15.3088 | 15.5067 | 7 | 8 | 11 | 8 | 5 | 6 | 8 | 9 |
| TMCO1 | Q9UM00;Q | 6.33E-11 | 3.15E-11 | -0.146396 | | 0.659787 | -1.3729 | | 18.6412 | 18.7435 | 18.5567 | 18.8128 | 18.8537 | 18.5049 | 18.8736 | 18.9227 | 3 | 3 | 3 | 3 | 3 | 3 | 3 | 3 |
| TFE3 | P19532 | 7.90E-06 | 2.10E-06 | -0.146226 | | 1.00661 | -1.95416 | | 15.8999 | 15.8124 | 15.9431 | 15.9738 | 16.1545 | 15.8639 | 16.0478 | 16.117 | 6 | 5 | 6 | 7 | 5 | 5 | 6 | 6 |
| DDAH2 | O95865 | 0.000113 | 2.66E-05 | -0.146111 | | 0.447041 | -0.99704 | | 17.2995 | 17.1087 | 17.2838 | 17.1385 | 17.3119 | 17.7689 | 17.3477 | 17.3168 | 10 | 10 | 10 | 10 | 10 | 10 | 10 | 10 |
| C1orf198 | Q9H425 | 2.29E-16 | 4.77E-16 | -0.145955 | | 0.089675 | -0.2466 | | 16.4069 | 16.3545 | 16.5149 | 15.0837 | 16.2224 | 17.6992 | 17.1123 | 16.7394 | 17 | 17 | 17 | 14 | 14 | 17 | 16 | 17 |
| GSPT1 | P15170-2; | 2.71E-14 | 3.04E-14 | -0.145938 | + | 1.51668 | -2.81806 | | 17.4627 | 17.5082 | 17.477 | 17.7663 | 17.64 | 17.6182 | 17.548 | 17.5704 | 33 | 33 | 31 | 32 | 33 | 32 | 32 | 33 |
| RIC1 | Q4ADV7 | 7.61E-08 | 2.48E-08 | -0.145793 | | 0.377758 | -0.86746 | | 14.5564 | 14.5322 | 14.6188 | 14.6667 | 14.4169 | 15.0127 | 14.9968 | 14.4814 | 6 | 7 | 5 | 6 | 5 | 8 | 6 | 6 |
| TPRN | Q4KMQ1 | 0.002637 | 0.0005 | -0.145638 | | 0.285196 | -0.6856 | | 13.3651 | 13.5323 | 13.2304 | 13.6862 | 13.8102 | 13.3845 | 12.9875 | 13.7394 | 2 | 1 | 2 | 3 | 1 | 2 | 1 | 1 |
| VPS26A | O75436 | 9.96E-12 | 5.79E-12 | -0.145499 | + | 1.57603 | -2.92252 | | 18.6427 | 18.7304 | 18.7701 | 18.9631 | 18.8478 | 18.8127 | 18.8899 | 18.786 | 16 | 16 | 16 | 16 | 16 | 16 | 16 | 16 |
| AGPAT3 | Q9NRZ7 | 1.10E-05 | 2.87E-06 | -0.145093 | | 1.24121 | -2.34584 | | 15.2875 | 15.125 | 15.1921 | 15.228 | 15.3664 | 15.4668 | 15.3194 | 15.3526 | 8 | 7 | 9 | 10 | 5 | 8 | 8 | 5 |
| APOBEC3C | Q9NRW3 | 1.44E-05 | 3.71E-06 | -0.14503 | | 0.770371 | -1.56043 | | 17.0303 | 17.0156 | 16.9857 | 17.1925 | 17.3569 | 17.1614 | 16.923 | 17.1441 | 5 | 5 | 4 | 3 | 5 | 4 | 5 | 4 |

| Gene names | UniProt IDs | Protein Group P-value | Protein Group Q-value | Log2 Difference NUP214 vs Controls (n=5) | p<0.05 | -log10 p-value | T-test Test statistic | NPC Localization | Log2 MS2_quantity_NUP214_1 | Log2 MS2_quantity_NUP214_2 | Log2 MS2_quantity_NUP214_3 | Log2 MS2_quantity_Control_1 | Log2 MS2_quantity_Control_2 | Log2 MS2_quantity_Control_3 | Log2 MS2_quantity_Control_4 | Log2 MS2_quantity_Control_5 | Number Peptides for Quantitation NUP214_1 | Number Peptides for Quantitation NUP214_2 | Number Peptides for Quantitation NUP214_3 | Number Peptides for Quantitation Control_1 | Number Peptides for Quantitation Control_2 | Number Peptides for Quantitation Control_3 | Number Peptides for Quantitation Control_4 | Number Peptides for Quantitation Control_5 |
|---|---|---|---|---|---|---|---|---|---|---|---|---|---|---|---|---|---|---|---|---|---|---|---|---|
| C1QBP | Q07021 | 2.69E-06 | 7.48E-07 | -0.144944 | | 0.997161 | -1.93847 | | 18.1642 | 18.186 | 18.2894 | 18.4649 | 18.1801 | 18.334 | 18.4593 | 18.3525 | 6 | 7 | 7 | 7 | 6 | 7 | 6 | 7 |
| NSFL1C | Q9UNZ2;Q | 3.03E-10 | 1.35E-10 | -0.14488 | + | 2.02635 | -3.75884 | | 17.6764 | 17.6523 | 17.6544 | 17.7869 | 17.915 | 17.7723 | 17.8036 | 17.7516 | 20 | 20 | 20 | 19 | 15 | 19 | 20 | 20 |
| LAMP1 | P11279 | 5.18E-07 | 1.56E-07 | -0.144659 | | 0.347373 | -0.80901 | | 20.7887 | 20.7885 | 20.7857 | 20.5237 | 20.8916 | 21.172 | 21.2733 | 20.8007 | 8 | 8 | 8 | 7 | 9 | 8 | 8 | 8 |
| GALE | Q14376 | 0.000103 | 2.44E-05 | -0.144619 | | 0.418343 | -0.94394 | | 16.775 | 16.8371 | 16.893 | 17.1302 | 16.5346 | 17.1196 | 17.1061 | 17.0078 | 8 | 8 | 8 | 7 | 8 | 7 | 8 | 8 |
| PAFAH1B3 | Q15102 | 7.72E-13 | 5.72E-13 | -0.144486 | | 0.181806 | -0.46556 | | 17.811 | 17.7426 | 17.8576 | 18.3136 | 18.6191 | 17.6611 | 17.3185 | 17.8287 | 7 | 7 | 8 | 7 | 7 | 7 | 7 | 7 |
| FAM210B | Q96KR6 | 0.001971 | 0.000387 | -0.144348 | | 0.165316 | -0.42825 | | 15.6807 | 15.8368 | 15.5952 | 16.2665 | 16.2922 | 15.2004 | 15.2761 | 16.2076 | 2 | 3 | 2 | 3 | 2 | 2 | 2 | 2 |
| OSBPL11 | Q9BXB4 | 3.21E-20 | 2.46E-19 | -0.144245 | | 0.720948 | -1.47704 | | 15.0483 | 15.1264 | 15.1155 | 15.3045 | 14.9601 | 15.2919 | 15.3708 | 15.2777 | 8 | 8 | 8 | 8 | 6 | 7 | 9 | 7 |
| SCYL1 | Q96KG9;Q | 4.42E-11 | 2.27E-11 | -0.144106 | + | 1.50511 | -2.79784 | | 16.8075 | 16.8985 | 16.8536 | 17.0172 | 16.918 | 17.0024 | 17.1181 | 16.931 | 29 | 29 | 28 | 29 | 26 | 27 | 28 | 29 |
| PDGFRA | P16234 | 2.85E-11 | 1.52E-11 | -0.144054 | | 0.149447 | -0.39165 | | 16.0378 | 16.0132 | 16.0193 | 17.1906 | 16.1366 | 15.8763 | 15.5492 | 16.0846 | 30 | 31 | 27 | 33 | 26 | 29 | 24 | 26 |
| ASAP1 | Q9ULH1;Q | 7.86E-14 | 7.72E-14 | -0.143973 | | 1.0494 | -2.02525 | | 15.7149 | 15.579 | 15.735 | 15.9353 | 15.9059 | 15.8228 | 15.6935 | 15.7441 | 19 | 19 | 20 | 20 | 19 | 19 | 19 | 18 |
| VAV2 | P52735;P | 9.11E-13 | 6.67E-13 | -0.143883 | | 0.69248 | -1.42872 | | 15.9115 | 15.8606 | 15.902 | 16.0672 | 16.299 | 15.9469 | 15.8517 | 16.0114 | 16 | 17 | 17 | 17 | 15 | 16 | 17 | 18 |
| NOP16 | Q9Y3C1 | 3.80E-05 | 9.41E-06 | -0.143577 | + | 1.53402 | -2.84847 | | 15.3686 | 15.3429 | 15.3497 | 15.5259 | 15.4209 | 15.4482 | 15.4608 | 15.6308 | 8 | 8 | 9 | 8 | 7 | 8 | 8 | 9 |
| NPRL3 | Q12980 | 1.11E-05 | 2.89E-06 | -0.143494 | | 0.37532 | -0.86281 | | 15.3625 | 15.434 | 15.2113 | 15.2012 | 15.8541 | 15.3806 | 15.6505 | 15.3108 | 2 | 1 | 2 | 1 | 1 | 1 | 1 | 1 |
| ATP2B1 | P20020;P2 | 6.59E-15 | 8.79E-15 | -0.143443 | | 0.773249 | -1.56526 | | 17.8003 | 17.8227 | 17.8105 | 17.9126 | 18.2127 | 17.8036 | 17.9005 | 17.9437 | 39 | 38 | 37 | 36 | 38 | 39 | 38 | 36 |
| EXOSC8 | Q96B26 | 0.007096 | 0.001242 | -0.143284 | | 0.256344 | -0.62629 | | 15.1921 | 15.026 | 14.7569 | 15.5161 | 15.3217 | 14.5899 | 15.0372 | 15.2099 | 3 | 1 | 2 | 2 | 1 | 1 | 1 | 1 |
| PGK1 | P00558 | 2.48E-13 | 2.12E-13 | -0.142928 | + | 1.39172 | -2.60157 | | 20.1266 | 20.0941 | 20.1289 | 20.2695 | 20.3636 | 20.3233 | 20.1364 | 20.2044 | 39 | 39 | 38 | 39 | 37 | 38 | 38 | 40 |
| CSTF2T | Q9H0L4 | 2.68E-05 | 6.73E-06 | -0.142844 | | 0.605656 | -1.27959 | | 16.3005 | 16.368 | 16.593 | 16.6024 | 16.6483 | 16.5348 | 16.3166 | 16.7145 | 6 | 6 | 8 | 5 | 6 | 4 | 3 | 7 |
| ARHGAP31 | Q2M1Z3 | 3.84E-12 | 2.42E-12 | -0.14268 | | 1.20954 | -2.29258 | | 14.1866 | 14.0953 | 14.2286 | 14.3725 | 14.3528 | 14.3405 | 14.1485 | 14.3498 | 9 | 11 | 12 | 11 | 8 | 10 | 6 | 9 |
| LETM1 | O95202 | 1.50E-18 | 6.27E-18 | -0.142182 | + | 1.63638 | -3.02991 | | 16.9321 | 17.0108 | 17.0347 | 17.2433 | 17.0517 | 17.1183 | 17.1276 | 17.1328 | 29 | 28 | 26 | 27 | 28 | 25 | 29 | 28 |
| WDFY1 | Q8IWB7 | 1.19E-09 | 4.88E-10 | -0.142155 | | 0.336096 | -0.78702 | | 16.7328 | 16.779 | 16.8479 | 17.238 | 16.5135 | 17.1428 | 17.0171 | 16.7322 | 17 | 18 | 17 | 17 | 15 | 17 | 17 | 16 |
| PIP4P1 | Q86T03;Q | 5.51E-07 | 1.65E-07 | -0.141939 | | 0.436537 | -0.97769 | | 15.9556 | 15.9287 | 15.9032 | 16.1399 | 15.6813 | 16.2104 | 16.3088 | 16.0152 | 4 | 4 | 4 | 4 | 3 | 4 | 4 | 4 |
| SUGP1 | Q8IWZ8 | 1.00E-07 | 3.23E-08 | -0.14182 | | 0.32877 | -0.77265 | | 14.6958 | 14.4652 | 14.3087 | 14.791 | 14.8968 | 14.3199 | 14.3475 | 14.8035 | 5 | 6 | 5 | 5 | 4 | 5 | 3 | 4 |
| MARK3 | P27448;P2 | 4.53E-10 | 1.96E-10 | -0.141742 | | 0.477504 | -1.05264 | | 14.2505 | 14.2671 | 14.2974 | 14.6296 | 14.6808 | 14.3159 | 14.2372 | 14.2035 | 11 | 12 | 11 | 13 | 6 | 11 | 13 | 11 |
| CXorf38 | Q8TB03 | 6.19E-06 | 1.66E-06 | -0.141719 | | 0.736229 | -1.50289 | | 15.7338 | 15.8377 | 15.778 | 15.7703 | 16.0279 | 15.8045 | 15.8867 | 16.135 | 6 | 5 | 7 | 6 | 5 | 5 | 5 | 5 |
| PKM | P14618 | 6.59E-14 | 6.61E-14 | -0.14166 | | 0.915346 | -1.80255 | | 23.1123 | 23.2115 | 23.1554 | 23.2806 | 23.4833 | 23.1313 | 23.3379 | 23.2738 | 48 | 47 | 48 | 47 | 48 | 47 | 48 | 47 |
| SLC25A22 | Q9H936 | 0.000124 | 2.90E-05 | -0.141573 | | 0.521515 | -1.13173 | | 16.5412 | 16.5714 | 16.5455 | 16.978 | 16.7621 | 16.4968 | 16.4767 | 16.7578 | 10 | 9 | 8 | 11 | 8 | 10 | 8 | 8 |
| ARHGAP10 | A1A4S6 | 2.44E-12 | 1.60E-12 | -0.141487 | | 0.326624 | -0.76843 | | 14.7051 | 14.7526 | 14.6299 | 15.3199 | 14.6256 | 14.9503 | 14.5757 | 14.7153 | 8 | 9 | 9 | 11 | 7 | 8 | 8 | 8 |
| DCTN2 | Q13561;Q | 1.46E-12 | 1.01E-12 | -0.141332 | | 1.00089 | -1.94467 | | 19.2164 | 19.1907 | 19.2051 | 19.3656 | 19.5134 | 19.181 | 19.294 | 19.3731 | 19 | 19 | 20 | 20 | 19 | 20 | 20 | 19 |
| AP3M1 | Q9Y2T2 | 7.48E-21 | 7.10E-20 | -0.141271 | | 1.22885 | -2.32504 | | 17.4269 | 17.4552 | 17.4602 | 17.5491 | 17.7527 | 17.6113 | 17.539 | 17.4914 | 15 | 14 | 14 | 11 | 13 | 12 | 14 | 13 |
| LRWD1 | Q9UFC0 | 1.88E-05 | 4.81E-06 | -0.141171 | | 0.642848 | -1.34383 | | 14.5116 | 14.5715 | 14.5287 | 14.8139 | 14.689 | 14.4137 | 14.6224 | 14.8532 | 4 | 7 | 4 | 7 | 5 | 3 | 6 | 6 |
| CARHSP1 | Q9Y2V2 | 3.19E-08 | 1.09E-08 | -0.141153 | | 0.238281 | -0.58841 | | 17.7017 | 17.8032 | 17.7201 | 18.5054 | 17.704 | 17.6273 | 17.525 | 18.0523 | 6 | 7 | 6 | 6 | 6 | 6 | 6 | 6 |
| AP1M1 | Q9BXS5;Q | 2.40E-10 | 1.08E-10 | -0.141149 | + | 1.31269 | -2.46669 | | 17.5591 | 17.5798 | 17.5755 | 17.8482 | 17.6047 | 17.6895 | 17.7652 | 17.6554 | 19 | 20 | 20 | 20 | 18 | 20 | 20 | 20 |
| DBNL | Q9UJU6-3 | 4.88E-09 | 1.85E-09 | -0.141093 | | 0.803974 | -1.61681 | | 18.321 | 18.3104 | 18.2906 | 18.6778 | 18.3597 | 18.4985 | 18.3083 | 18.3977 | 18 | 18 | 17 | 17 | 17 | 17 | 18 | 18 |
| TSC22D1 | Q15714 | 0.000483 | 0.000104 | -0.141088 | | 0.073037 | -0.20385 | | 15.1692 | 15.0403 | 14.9936 | 16.1769 | 15.3255 | 15.8041 | 13.2164 | 15.521 | 1 | 1 | 1 | 3 | 1 | 1 | 1 | 3 |
| IRGQ | Q8WZA9 | 1.30E-08 | 4.67E-09 | -0.140791 | | 0.538308 | -1.16156 | | 16.4625 | 16.4009 | 16.3794 | 16.4881 | 16.3337 | 16.56 | 16.881 | 16.5125 | 15 | 13 | 15 | 14 | 13 | 17 | 13 | 11 |
| WDR36 | Q8NI36 | 9.24E-08 | 2.99E-08 | -0.140705 | | 0.87095 | -1.72867 | | 16.1965 | 16.2121 | 16.2082 | 16.5732 | 16.2751 | 16.2151 | 16.3213 | 16.347 | 16 | 17 | 17 | 18 | 14 | 16 | 15 | 18 |
| VCP | P55072 | 7.05E-15 | 9.28E-15 | -0.140556 | | 1.03862 | -2.00733 | | 20.1596 | 20.1369 | 20.1576 | 20.3501 | 20.4054 | 20.2777 | 20.0992 | 20.3272 | 52 | 54 | 53 | 52 | 51 | 50 | 53 | 53 |
| CCDC58 | Q4VC31 | 0.00017 | 3.91E-05 | -0.14044 | | 0.150359 | -0.39377 | | 15.2284 | 14.9076 | 15.5668 | 15.6428 | 15.4225 | 14.4641 | 15.4136 | 15.9305 | 4 | 4 | 4 | 4 | 2 | 2 | 3 | 3 |
| SPAG7 | O75391 | 3.63E-05 | 9.01E-06 | -0.140148 | | 0.456784 | -1.01491 | | 14.78 | 14.8737 | 14.8975 | 15.2165 | 14.7059 | 14.7887 | 15.1235 | 15.1181 | 6 | 6 | 6 | 5 | 4 | 6 | 6 | 6 |
| ANKS1A | Q92625 | 0.000368 | 8.08E-05 | -0.140112 | | 0.581409 | -1.23737 | | 15.7584 | 15.9446 | 15.6772 | 16.2102 | 15.9328 | 15.8129 | 15.8141 | 15.8976 | 3 | 6 | 6 | 5 | 4 | 7 | 4 | 2 |
| TP53I3 | Q53FA7 | 3.91E-08 | 1.32E-08 | -0.140081 | | 1.07139 | -2.06182 | | 17.9187 | 17.873 | 17.9424 | 18.0295 | 18.0805 | 18.2274 | 17.9747 | 17.9453 | 11 | 12 | 12 | 11 | 10 | 11 | 11 | 11 |
| SLC25A24 | Q6NUK1 | 3.15E-16 | 6.18E-16 | -0.139794 | + | 1.68476 | -3.11694 | | 17.6478 | 17.628 | 17.5622 | 17.7313 | 17.7087 | 17.8714 | 17.7434 | 17.7076 | 18 | 19 | 18 | 18 | 19 | 20 | 19 | 20 |
| MTX1 | Q13505;Q | 3.07E-14 | 3.37E-14 | -0.139733 | + | 2.10464 | -3.91369 | | 15.7025 | 15.6351 | 15.6838 | 15.851 | 15.891 | 15.7666 | 15.7789 | 15.7802 | 9 | 9 | 9 | 10 | 9 | 8 | 10 | 8 |
| CHFR | Q96EP1;Q | 0.004264 | 0.000771 | -0.139574 | | 0.403802 | -0.93089 | | 13.5067 | 13.7401 | 13.7316 | 13.5715 | NaN | 13.7332 | 14.1168 | 13.7748 | 2 | 2 | 3 | 1 | NaN | 2 | 1 | 1 |
| ACBD5 | Q5T8D3-2; | 7.16E-15 | 9.41E-15 | -0.139363 | + | 1.91781 | -3.54922 | | 15.2505 | 15.3223 | 15.3336 | 15.4969 | 15.4416 | 15.3527 | 15.4879 | 15.4284 | 12 | 9 | 10 | 10 | 9 | 11 | 10 | 11 |
| RABEP2 | Q9H5N1 | 1.68E-12 | 1.15E-12 | -0.139163 | + | 2.02404 | -3.75432 | | 15.7386 | 15.6678 | 15.7596 | 15.9123 | 15.8898 | 15.8415 | 15.8822 | 15.7801 | 13 | 13 | 13 | 13 | 10 | 13 | 13 | 11 |
| SMAP1 | Q8IYB5-2;Q | 0.00014 | 3.24E-05 | -0.139008 | | 0.648684 | -1.35386 | | 16.4439 | 16.4338 | 16.365 | 16.7465 | 16.4834 | 16.3017 | 16.5929 | 16.6419 | 3 | 3 | 3 | 3 | 3 | 3 | 3 | 3 |
| RTCA | O00442;Q | 3.77E-05 | 9.34E-06 | -0.138956 | + | 1.48466 | -2.76217 | | 17.7342 | 17.8341 | 17.713 | 17.8815 | 17.8832 | 18.0073 | 17.8117 | 17.9132 | 10 | 9 | 10 | 11 | 9 | 12 | 11 | 11 |
| FAM91A1 | Q658Y4 | 2.36E-10 | 1.07E-10 | -0.138908 | | 0.997765 | -1.93947 | | 15.4904 | 15.5102 | 15.5057 | 15.5618 | 15.8515 | 15.6106 | 15.5695 | 15.6116 | 16 | 16 | 15 | 15 | 12 | 16 | 14 | 14 |
| DYNLT1 | P63172 | 4.86E-15 | 6.76E-15 | -0.138839 | | 1.00631 | -1.95366 | | 16.8948 | 16.8698 | 16.9392 | 17.0225 | 16.8511 | 17.0653 | 17.1063 | 17.1553 | 3 | 3 | 3 | 3 | 3 | 3 | 3 | 3 |
| TBC1D13 | Q9NVG8 | 1.75E-10 | 8.12E-11 | -0.138726 | | 0.822595 | -1.64798 | | 16.5356 | 16.4328 | 16.5818 | 16.6522 | 16.7863 | 16.4391 | 16.6894 | 16.7103 | 15 | 15 | 15 | 15 | 15 | 14 | 14 | 15 |
| AFTPH | Q6ULP2;Q | 0.006369 | 0.001121 | -0.13854 | | 0.1292 | -0.34387 | | 17.432 | 17.2816 | 17.289 | 18.2633 | 16.4642 | 17.237 | 17.6253 | 17.774 | 2 | 3 | 4 | 4 | 3 | 2 | 2 | 3 |
| RPL13A | P40429 | 7.30E-13 | 5.46E-13 | -0.138516 | | 1.11164 | -2.12884 | | 19.8333 | 19.9744 | 19.8273 | 19.9509 | 20.1504 | 20.075 | 19.9418 | 19.9661 | 10 | 10 | 10 | 9 | 10 | 10 | 10 | 10 |
| PMM2 | O15305 | 4.08E-10 | 1.78E-10 | -0.138492 | | 0.81957 | -1.64292 | | 17.1091 | 17.1612 | 17.1593 | 17.0447 | 17.2961 | 17.4144 | 17.3164 | 17.3368 | 11 | 12 | 11 | 11 | 12 | 11 | 12 | 12 |
| BCL9L | Q86UU0;Q | 9.32E-11 | 4.49E-11 | -0.1383 | | 0.847298 | -1.68924 | | 14.803 | 14.7184 | 14.7921 | 14.9925 | 14.9859 | 14.8248 | 14.7164 | 15.0278 | 4 | 3 | 4 | 4 | 3 | 4 | 3 | 4 |
| MCM3 | P25205;P2 | 7.04E-14 | 6.99E-14 | -0.138133 | | 0.188049 | -0.4795 | | 16.4259 | 16.4638 | 16.4674 | 17.0081 | 16.6098 | 15.8035 | 16.569 | 16.962 | 31 | 31 | 34 | 36 | 29 | 26 | 31 | 34 |
| TRAF3 | Q13114 | 0.019615 | 0.00327 | -0.138046 | | 0.408317 | -0.93961 | | 12.9359 | 12.5436 | 12.5849 | 12.6885 | NaN | 12.9019 | 12.6763 | 13.0381 | 1 | 1 | 1 | 2 | NaN | 1 | 1 | 1 |
| RPL35 | P42766 | 5.81E-05 | 1.42E-05 | -0.137914 | + | 1.43234 | -2.67147 | | 19.6753 | 19.8486 | 19.6923 | 19.8192 | 19.9035 | 19.8835 | 19.9493 | 19.8279 | 7 | 7 | 7 | 6 | 6 | 6 | 6 | 6 |
| ARHGEF10L | Q9HCE6;Q | 0.004727 | 0.000848 | -0.137877 | | 0.673389 | -1.39617 | | 14.6896 | 14.6511 | 14.8482 | 15.125 | 14.8129 | 14.7915 | 14.7551 | 14.8532 | 3 | 4 | 5 | 4 | 4 | 5 | 5 | 3 |
| RAF1 | P04049;P | 1.06E-05 | 2.77E-06 | -0.137681 | | 0.491794 | -1.07847 | | 16.2006 | 16.0614 | 16.2872 | 16.3694 | 16.5571 | 16.0736 | 16.165 | 16.4386 | 11 | 8 | 9 | 9 | 7 | 6 | 7 | 10 |
| DDX5 | P17844;P1 | 8.33E-07 | 2.44E-07 | -0.13753 | | 0.827798 | -1.65667 | | 18.3229 | 18.3096 | 18.2573 | 18.5249 | 18.4651 | 18.192 | 18.4991 | 18.4896 | 22 | 22 | 22 | 23 | 22 | 22 | 23 | 22 |
| TSN | Q15631 | 9.18E-11 | 4.43E-11 | -0.137506 | | 0.697759 | -1.4377 | | 17.9301 | 17.8909 | 18.0141 | 17.8547 | 18.0609 | 18.0552 | 18.263 | 18.179 | 13 | 12 | 12 | 12 | 12 | 12 | 12 | 12 |
| NOP9 | Q86U38 | 6.27E-07 | 1.86E-07 | -0.137436 | + | 1.38825 | -2.59562 | | 15.6901 | 15.7705 | 15.7969 | 15.8308 | 15.8183 | 15.8478 | 15.9746 | 15.9781 | 9 | 9 | 8 | 8 | 7 | 9 | 9 | 10 |
| NHEJ1 | Q9H9Q4;Q | 0.000161 | 3.73E-05 | -0.137267 | | 0.223699 | -0.55735 | | 15.2047 | 14.5832 | 15.2631 | 15.1926 | 15.2796 | 14.8608 | 14.8406 | 15.5978 | 3 | 3 | 3 | 3 | 2 | 4 | 3 | 3 |
| PACS2 | Q86VP3;Q | 5.08E-06 | 1.37E-06 | -0.137105 | | 0.662365 | -1.37732 | | 14.7324 | 14.7755 | 14.8053 | 14.8238 | 15.1827 | 14.8997 | 14.8872 | 14.7475 | 4 | 4 | 4 | 4 | 3 | 2 | 4 | 2 |
| CUL5 | Q93034 | 2.74E-10 | 1.22E-10 | -0.137085 | | 0.705425 | -1.45072 | | 16.0386 | 16.061 | 16.1005 | 16.1942 | 16.0036 | 16.2827 | 16.4167 | 16.1218 | 28 | 26 | 27 | 21 | 24 | 22 | 26 | 23 |
| RAB1A | P62820 | 1.74E-09 | 6.97E-10 | -0.137036 | + | 2.21951 | -4.14684 | | 19.8691 | 19.8226 | 19.8042 | 20.0319 | 19.9659 | 19.9457 | 20.0002 | 19.9014 | 13 | 13 | 13 | 12 | 13 | 13 | 13 | 13 |
| PIK3R4 | Q99570 | 1.64E-12 | 1.12E-12 | -0.137023 | | 1.0751 | -2.06799 | | 15.5573 | 15.6339 | 15.6208 | 15.6995 | 15.8949 | 15.6782 | 15.804 | 15.6283 | 29 | 27 | 27 | 24 | 23 | 24 | 27 | 23 |
| TUBB | P07437 | 1.24E-05 | 3.23E-06 | -0.136945 | | 1.10876 | -2.12404 | | 23.4219 | 23.3942 | 23.4653 | 23.4983 | 23.7247 | 23.5548 | 23.4495 | 23.5931 | 5 | 5 | 5 | 5 | 5 | 5 | 5 | 5 |
| TRAPPC9 | Q96Q05;Q | 0.009859 | 0.001693 | -0.136851 | | 0.27364 | -0.66201 | | 14.3069 | 13.981 | 14.0269 | 14.0907 | 13.8126 | 14.2856 | 14.3286 | 14.6913 | 3 | 4 | 3 | 2 | 1 | 2 | 2 | 3 |
| GEMIN4 | P57678 | 1.83E-15 | 2.91E-15 | -0.136808 | | 1.19099 | -2.26146 | | 14.7353 | 14.8213 | 14.8366 | 14.9481 | 14.8225 | 14.8741 | 14.9585 | 15.0696 | 14 | 14 | 14 | 14 | 10 | 11 | 15 | 11 |
| GMPPA | Q96IJ6 | 4.40E-07 | 1.34E-07 | -0.136579 | + | 1.7899 | -3.30916 | | 17.7511 | 17.8151 | 17.7094 | 17.9086 | 17.8417 | 17.9187 | 17.8334 | 17.9732 | 9 | 8 | 11 | 8 | 9 | 9 | 8 | 9 |
| MEAK7 | Q6P9B6 | 2.42E-10 | 1.10E-10 | -0.13652 | | 0.410549 | -0.92938 | | 17.0249 | 17.0173 | 17.1129 | 16.8182 | 17.2764 | 17.0829 | 17.3249 | 17.4385 | 12 | 12 | 12 | 12 | 13 | 13 | 13 | 13 |
| LIG4 | P49917 | 0.000166 | 3.83E-05 | -0.136273 | | 0.242465 | -0.59724 | | 14.4646 | 14.7749 | 14.8123 | 14.3179 | 15.0223 | 14.7819 | 15.2732 | 14.7058 | 6 | 9 | 6 | 5 | 7 | 6 | 4 | 6 |
| DDX54 | Q8TDD1;Q | 1.84E-12 | 1.25E-12 | -0.136247 | + | 1.85467 | -3.42983 | | 15.4134 | 15.5379 | 15.5201 | 15.5738 | 15.6028 | 15.606 | 15.6837 | 15.6672 | 18 | 19 | 19 | 16 | 13 | 16 | 17 | 16 |
| EXOSC9 | Q06265;Q | 6.46E-05 | 1.56E-05 | -0.13613 | | 0.445261 | -0.99377 | | 15.7974 | 15.7009 | 15.7152 | 15.8041 | 16.1587 | 15.5971 | 15.7615 | 16.0485 | 5 | 5 | 5 | 5 | 3 | 4 | 5 | 4 |
| TTLL12 | Q14166 | 3.94E-13 | 3.18E-13 | -0.136123 | | 0.196494 | -0.4982 | | 16.9257 | 16.9945 | 16.9901 | 17.4918 | 16.5795 | 17.659 | 17.0018 | 16.7992 | 21 | 20 | 22 | 19 | 16 | 20 | 18 | 18 |
| MOGS | Q13724 | 1.53E-14 | 1.84E-14 | -0.135985 | | 0.407152 | -0.92302 | | 17.8207 | 17.7719 | 17.7477 | 17.8404 | 17.6702 | 18.1598 | 18.1925 | 17.7175 | 25 | 25 | 25 | 25 | 23 | 24 | 25 | 24 |

| Gene names | UniProt IDs | Protein Group P-value | Protein Group Q-value | Log2 Difference NUP214 vs Controls (n=5) | p<0.05 | -log10 p-value | T-test Test statistic | NPC Localization | Log2 MS2_quantity_NUP214_1 | Log2 MS2_quantity_NUP214_2 | Log2 MS2_quantity_NUP214_3 | Log2 MS2_quantity_Control_1 | Log2 MS2_quantity_Control_2 | Log2 MS2_quantity_Control_3 | Log2 MS2_quantity_Control_4 | Log2 MS2_quantity_Control_5 | Number Peptides for Quantitation NUP214_1 | Number Peptides for Quantitation NUP214_2 | Number Peptides for Quantitation NUP214_3 | Number Peptides for Quantitation Control_1 | Number Peptides for Quantitation Control_2 | Number Peptides for Quantitation Control_3 | Number Peptides for Quantitation Control_4 | Number Peptides for Quantitation Control_5 |
|---|---|---|---|---|---|---|---|---|---|---|---|---|---|---|---|---|---|---|---|---|---|---|---|---|
| MAPK3 | P27361 | 2.99E-12 | 1.92E-12 | -0.135877 | | 0.919402 | -1.8093 | | 17.6247 | 17.6576 | 17.6229 | 17.8496 | 17.6607 | 17.9155 | 17.6221 | 17.8069 | 13 | 14 | 13 | 14 | 13 | 14 | 13 | 13 |
| KLC1 | Q07866-6 | 2.57E-12 | 1.68E-12 | -0.135838 | | 0.948078 | -1.85695 | | 17.0012 | 16.9923 | 17.015 | 17.1519 | 17.0135 | 17.0409 | 17.3228 | 17.1641 | 29 | 28 | 28 | 28 | 27 | 28 | 29 | 28 |
| LNPK | Q9C0E8;Q9 | 0.000217 | 4.91E-05 | -0.135733 | | 0.352391 | -0.81874 | | 17.1505 | 17.1942 | 17.2202 | 17.7271 | 17.4491 | 17.1952 | 16.9973 | 17.2515 | 10 | 10 | 9 | 10 | 8 | 10 | 9 | 9 |
| ATP6AP2 | O75787;O7 | 3.17E-07 | 9.76E-08 | -0.135731 | | 0.355209 | -0.82419 | | 16.068 | 15.9994 | 16.0926 | 16.3801 | 16.415 | 15.735 | 16.2626 | 16.1527 | 9 | 9 | 9 | 8 | 7 | 8 | 9 | 8 |
| RPAP3 | Q9H6T3 | 7.66E-11 | 3.74E-11 | -0.135509 | | 0.953506 | -1.86596 | | 17.5658 | 17.5492 | 17.5276 | 17.6475 | 17.8532 | 17.5661 | 17.7595 | 17.589 | 18 | 18 | 17 | 16 | 13 | 15 | 17 | 18 |
| NT5C3B | Q969T7;Q9 | 3.13E-17 | 8.55E-17 | -0.135448 | | 0.296714 | -0.70888 | | 15.0492 | 15.0954 | 14.975 | 14.8513 | 15.3418 | 15.4813 | 14.813 | 15.3892 | 6 | 6 | 6 | 5 | 4 | 6 | 6 | 6 |
| EIF2AK4 | Q9P2K8;Q9 | 1.72E-12 | 1.17E-12 | -0.135448 | | 0.521741 | -1.13213 | | 15.444 | 15.4422 | 15.4829 | 15.6066 | 15.6773 | 15.25 | 15.656 | 15.7692 | 36 | 34 | 38 | 35 | 30 | 32 | 34 | 37 |
| EDEM3 | Q9BZQ6;Q9 | 1.64E-12 | 1.12E-12 | -0.135427 | | 1.03181 | -1.99602 | | 14.4836 | 14.4907 | 14.4707 | 14.6561 | 14.773 | 14.4868 | 14.5269 | 14.6426 | 17 | 16 | 15 | 16 | 11 | 14 | 11 | 13 |
| NIBAN2 | Q96TA1;Q9 | 9.05E-12 | 5.30E-12 | -0.135335 | | 0.832369 | -1.66431 | | 18.7476 | 18.7047 | 18.7076 | 18.9471 | 18.9995 | 18.6631 | 18.8887 | 18.7781 | 33 | 33 | 33 | 33 | 31 | 32 | 33 | 33 |
| VAMP7 | P51809 | 1.67E-12 | 1.14E-12 | -0.1353 | | 0.55311 | -1.18771 | | 16.8037 | 16.828 | 16.7341 | 16.6707 | 16.9331 | 17.0565 | 17.1428 | 16.8164 | 8 | 8 | 8 | 8 | 8 | 8 | 8 | 9 |
| PLCD3 | Q8N3E9 | 8.24E-07 | 2.42E-07 | -0.135282 | | 0.226764 | -0.56391 | | 15.5172 | 15.513 | 15.4478 | 14.967 | 15.7169 | 15.9617 | 15.9202 | 15.5739 | 7 | 6 | 6 | 7 | 7 | 7 | 6 | 7 |
| CENPE | Q02224;Q0 | 0.008703 | 0.001503 | -0.13512 | | 0.086394 | -0.24034 | | 14.9238 | NaN | 15.053 | 15.3206 | 16.2347 | 15.2032 | 14.485 | 14.3741 | 1 | NaN | 1 | 1 | 1 | 1 | 2 | 1 |
| GSTP1 | P09211 | 8.54E-12 | 5.02E-12 | -0.135036 | | 0.361663 | -0.83663 | | 21.4083 | 21.4313 | 21.541 | 21.4289 | 21.6145 | 21.6032 | 22.015 | 21.3146 | 11 | 11 | 12 | 13 | 14 | 13 | 15 | 13 |
| EMC7 | Q9NPA0 | 8.33E-06 | 2.20E-06 | -0.134947 | | 1.20044 | -2.27732 | | 17.4078 | 17.4294 | 17.3656 | 17.5026 | 17.6528 | 17.5885 | 17.5404 | 17.395 | 6 | 6 | 6 | 6 | 6 | 6 | 6 | 6 |
| ATP5F1B | P06576 | 2.53E-12 | 1.66E-12 | -0.134935 | + | 2.68022 | -5.16318 | | 21.0308 | 21.0643 | 21.0542 | 21.1469 | 21.2567 | 21.1782 | 21.1759 | 21.1657 | 25 | 25 | 25 | 25 | 24 | 24 | 24 | 24 |
| CIAPIN1 | Q6FI81 | 1.18E-11 | 6.76E-12 | -0.134438 | | 0.792975 | -1.59838 | | 16.1988 | 16.187 | 16.1579 | 16.3366 | 16.4586 | 16.125 | 16.4318 | 16.2262 | 12 | 11 | 14 | 10 | 10 | 12 | 12 | 13 |
| SEC23A | Q15436 | 5.60E-19 | 2.69E-18 | -0.134358 | | 0.858436 | -1.70782 | | 19.1288 | 19.0729 | 19.0556 | 19.2938 | 19.0851 | 19.3808 | 19.2488 | 19.092 | 26 | 27 | 27 | 27 | 28 | 27 | 27 | 27 |
| SLC1A5 | Q15758 | 4.16E-09 | 1.59E-09 | -0.134357 | | 0.204396 | -0.51556 | | 19.0758 | 19.0798 | 19.1669 | 18.9841 | 18.7932 | 19.5908 | 19.8113 | 19.0298 | 12 | 12 | 13 | 11 | 10 | 11 | 11 | 12 |
| DUSP14 | O95147 | 0.000249 | 5.60E-05 | -0.134218 | | 0.622015 | -1.30792 | | 15.1094 | 15.0981 | 15.2126 | 15.1633 | 15.2672 | 15.1685 | 15.2102 | 15.5621 | 2 | 2 | 2 | 2 | 2 | 2 | 2 | 2 |
| TCAF1 | Q9Y4C2 | 1.69E-22 | 2.47E-21 | -0.133755 | | 0.512719 | -1.11603 | | 15.997 | 15.9788 | 15.9134 | 16.2733 | 16.0371 | 15.7786 | 16.2245 | 16.1707 | 20 | 19 | 21 | 21 | 17 | 18 | 21 | 18 |
| SEC24C | P53992 | 3.61E-14 | 3.88E-14 | -0.133656 | | 0.734738 | -1.50037 | | 17.3785 | 17.3464 | 17.359 | 17.5824 | 17.6049 | 17.4064 | 17.2743 | 17.6067 | 30 | 30 | 31 | 32 | 30 | 30 | 32 | 31 |
| TCEAL3 | Q969E4 | 1.82E-13 | 1.61E-13 | -0.133583 | | 0.271907 | -0.65846 | | 15.5042 | 15.3841 | 15.779 | 15.4293 | 15.4075 | 15.6897 | 16.1692 | 15.7509 | 7 | 8 | 7 | 7 | 7 | 8 | 8 | 6 |
| MCM6 | Q14566 | 2.56E-24 | 7.58E-23 | -0.133532 | | 0.16107 | -0.41853 | | 16.1052 | 16.1505 | 16.1093 | 16.7285 | 16.3459 | 15.3876 | 16.1695 | 16.6445 | 33 | 32 | 33 | 35 | 30 | 27 | 30 | 34 |
| SNX3 | O60493 | 4.49E-07 | 1.36E-07 | -0.133519 | | 0.734709 | -1.50032 | | 19.3041 | 19.2766 | 19.2937 | 19.5478 | 19.518 | 19.1862 | 19.4985 | 19.3745 | 10 | 10 | 10 | 10 | 11 | 10 | 10 | 10 |
| NUDT10;NUDT11 | Q8NFP7;Q9 | 6.29E-11 | 3.14E-11 | -0.133254 | | 0.227397 | -0.56527 | | 14.8289 | 15.8451 | 15.5851 | 15.6013 | 15.4808 | 15.361 | 15.6539 | 15.6679 | 2 | 1 | 2 | 3 | 2 | 2 | 2 | 2 |
| PIGK | Q92643 | 6.75E-06 | 1.80E-06 | -0.133211 | | 0.577212 | -1.23004 | | 16.6718 | 16.6468 | 16.6239 | 16.6396 | 17.0589 | 16.6092 | 16.8395 | 16.7562 | 8 | 8 | 8 | 8 | 7 | 8 | 8 | 8 |
| ITM2C | Q9NQX7;Q | 0.000123 | 2.89E-05 | -0.133118 | | 0.11741 | -0.31543 | | 15.3647 | 15.478 | 15.3906 | 15.8499 | 16.1022 | 14.323 | 15.5877 | 15.8583 | 3 | 3 | 3 | 3 | 3 | 3 | 3 | 3 |
| RELA | Q04206;Q0 | 4.93E-08 | 1.65E-08 | -0.133036 | + | 2.00513 | -3.71742 | | 17.2541 | 17.2879 | 17.1926 | 17.4158 | 17.4041 | 17.369 | 17.2955 | 17.4052 | 16 | 16 | 15 | 14 | 14 | 14 | 15 | 14 |
| UBE3A | Q05086;Q0 | 1.89E-11 | 1.05E-11 | -0.132939 | + | 1.91997 | -3.55334 | | 15.614 | 15.5779 | 15.5822 | 15.6517 | 15.7702 | 15.6736 | 15.7949 | 15.7312 | 25 | 24 | 20 | 21 | 20 | 24 | 24 | 23 |
| ERCC6L | Q2NKX8 | 4.81E-07 | 1.45E-07 | -0.132778 | | 0.416978 | -0.94139 | | 14.9724 | 15.1678 | 15.0729 | 15.4372 | 15.1826 | 14.894 | 15.096 | 15.4091 | 6 | 9 | 8 | 9 | 4 | 6 | 7 | 7 |
| HEATR6 | Q6AI08 | 0.000176 | 4.04E-05 | -0.132732 | | 0.684056 | -1.41437 | | 14.0998 | 14.2269 | 14.1479 | 14.3551 | 14.4337 | 14.2755 | 14.3497 | 14.0407 | 8 | 5 | 8 | 7 | 4 | 4 | 7 | 7 |
| VEZF1 | Q14119 | 2.17E-05 | 5.52E-06 | -0.132621 | | 0.44375 | -0.99099 | | 12.1095 | 12.2265 | 12.5589 | 12.5369 | 12.4918 | 12.5891 | 12.2663 | 12.2706 | 2 | 2 | 2 | 2 | 1 | 1 | 1 | 1 |
| PML | P29590-13 | 6.70E-05 | 1.62E-05 | -0.132432 | | 0.091169 | -0.26148 | | 13.6661 | 13.2448 | 12.3422 | 13.1516 | NaN | 13.282 | NaN | NaN | 1 | 1 | 1 | 1 | NaN | 1 | NaN | NaN |
| RNF214 | Q8ND24 | 9.00E-10 | 3.75E-10 | -0.132412 | + | 1.40477 | -2.62398 | | 14.966 | 15.0339 | 15.0111 | 15.1953 | 15.1504 | 15.033 | 15.2267 | 15.075 | 13 | 15 | 14 | 14 | 10 | 13 | 13 | 12 |
| C1orf52 | Q8N6N3;Q | 7.58E-05 | 1.82E-05 | -0.132394 | | 0.174434 | -0.44897 | | 13.3439 | 13.3944 | 13.6196 | 13.9436 | 12.9899 | 13.1376 | 13.8312 | 14.0228 | 2 | 1 | 1 | 3 | 2 | 2 | 1 | 2 |
| PEX13 | Q92968 | 0.056383 | 0.009129 | -0.132383 | | 0.062148 | -0.17896 | | 16.3575 | 16.5995 | 15.9079 | 17.0904 | NaN | 14.9987 | NaN | 17.1731 | 2 | 1 | 2 | 2 | NaN | 1 | NaN | 2 |
| PGM1 | P36871 | 4.33E-15 | 6.11E-15 | -0.132374 | | 0.338223 | -0.79118 | | 18.2012 | 18.1461 | 18.1622 | 18.033 | 18.1212 | 18.5776 | 18.3182 | 18.2812 | 28 | 28 | 29 | 28 | 27 | 28 | 29 | 29 |
| DCAF6 | Q58WW2- | 4.36E-06 | 1.19E-06 | -0.132358 | | 0.550376 | -1.18289 | | 14.5505 | 14.5728 | 14.2808 | 14.6577 | 14.8367 | 14.5065 | 14.4919 | 14.5093 | 5 | 6 | 8 | 10 | 4 | 6 | 6 | 6 |
| DECR2 | Q9NUI1 | 5.52E-10 | 2.36E-10 | -0.132146 | | 0.177568 | -0.45604 | | 16.1437 | 16.0237 | 16.1071 | 15.585 | 16.8116 | 15.8916 | 16.4178 | 16.4123 | 11 | 11 | 11 | 11 | 10 | 11 | 11 | 11 |
| ATP2B4 | P23634;P2 | 3.69E-13 | 3.00E-13 | -0.132119 | | 0.25634 | -0.62628 | | 17.9285 | 17.9477 | 17.9278 | 18.5058 | 18.1296 | 17.6554 | 17.7661 | 18.277 | 30 | 30 | 30 | 29 | 27 | 30 | 30 | 29 |
| COPS8 | Q99627 | 7.74E-06 | 2.06E-06 | -0.132118 | | 0.712699 | -1.46306 | | 17.4801 | 17.2368 | 17.4827 | 17.4209 | 17.3995 | 17.5959 | 17.6512 | 17.5924 | 5 | 5 | 5 | 5 | 5 | 5 | 5 | 5 |
| DTD1 | Q8TEA8 | 2.45E-06 | 6.83E-07 | -0.131789 | | 0.562562 | -1.20435 | | 17.6449 | 17.789 | 17.7364 | 18.1494 | 17.8016 | 17.8735 | 17.7459 | 17.7056 | 5 | 5 | 5 | 5 | 4 | 5 | 5 | 5 |
| NSUN5 | Q96P11-2 | 0.001338 | 0.000271 | -0.13174 | | 0.69625 | -1.43513 | | 13.8616 | 14.1285 | 14.0826 | 14.328 | 14.0595 | 14.1902 | 14.038 | 14.1641 | 4 | 6 | 4 | 4 | 2 | 4 | 4 | 3 |
| RCOR1 | Q9UKL0 | 3.37E-07 | 1.04E-07 | -0.131684 | | 0.196742 | -0.49875 | | 15.2295 | 15.1754 | 15.2718 | 15.2075 | 15.7932 | 14.7048 | 15.3478 | 15.7331 | 4 | 3 | 5 | 3 | 3 | 3 | 4 | 4 |
| ASPH | Q12797;Q1 | 1.20E-17 | 3.71E-17 | -0.131594 | | 0.831155 | -1.66228 | | 18.6456 | 18.7076 | 18.6602 | 18.8823 | 18.9702 | 18.6369 | 18.8023 | 18.7219 | 31 | 30 | 30 | 31 | 30 | 29 | 31 | 30 |
| SFSWAP | Q12872;Q1 | 0.002464 | 0.000472 | -0.131545 | | 0.243977 | -0.60042 | | 17.3463 | 17.532 | 17.5552 | 18.0586 | 17.5722 | 17.6561 | 17.0613 | 17.6987 | 5 | 4 | 7 | 4 | 4 | 4 | 2 | 5 |
| TM9SF2 | Q99805 | 2.03E-11 | 1.11E-11 | -0.131345 | | 0.413805 | -0.93547 | | 17.2019 | 17.2273 | 17.2656 | 17.529 | 16.9889 | 17.3666 | 17.5902 | 17.3399 | 14 | 14 | 14 | 15 | 14 | 15 | 16 | 14 |
| MGLL | Q99685 | 1.23E-13 | 1.13E-13 | -0.13134 | | 0.212995 | -0.53428 | | 17.0745 | 17.1334 | 17.1478 | 17.9163 | 17.1051 | 16.796 | 17.1745 | 17.2577 | 9 | 9 | 9 | 8 | 7 | 8 | 7 | 8 |
| USP19 | O94966-5; | 7.47E-12 | 4.45E-12 | -0.130807 | | 1.19887 | -2.27468 | | 14.7846 | 14.7849 | 14.7581 | 14.8987 | 15.0133 | 14.7739 | 14.8642 | 14.9833 | 15 | 15 | 15 | 15 | 14 | 14 | 12 | 12 |
| RAB9A | P51151 | 5.25E-12 | 3.22E-12 | -0.130757 | | 0.633596 | -1.32791 | | 16.4221 | 16.4804 | 16.4671 | 16.7691 | 16.3716 | 16.671 | 16.464 | 16.6609 | 9 | 9 | 9 | 9 | 8 | 9 | 9 | 10 |
| SHOC2 | Q9UQ13;Q | 2.26E-12 | 1.50E-12 | -0.130558 | + | 1.45474 | -2.71021 | | 16.6041 | 16.6423 | 16.5025 | 16.6956 | 16.7787 | 16.7807 | 16.6543 | 16.6582 | 8 | 7 | 8 | 8 | 7 | 8 | 8 | 8 |
| MRPL57 | Q9BQC6 | 0.000661 | 0.00014 | -0.130517 | | 0.405533 | -0.91998 | | 13.9914 | 13.7965 | 13.8887 | 14.1336 | 13.9355 | 13.6628 | 14.2183 | 14.1633 | 1 | 1 | 1 | 2 | 3 | 2 | 2 | 2 |
| RRP15 | Q9Y3B9 | 9.85E-16 | 1.69E-15 | -0.130471 | | 0.465278 | -1.03042 | | 14.7693 | 14.8742 | 14.7803 | 15.1559 | 15.1558 | 14.7641 | 14.722 | 14.8943 | 8 | 8 | 8 | 8 | 6 | 8 | 8 | 8 |
| EML1 | O00423;O0 | 2.62E-05 | 6.59E-06 | -0.130391 | | 0.468766 | -1.03677 | | 15.6247 | 15.5709 | 15.7176 | 15.409 | 15.7964 | 15.8658 | 15.8696 | 15.8998 | 8 | 13 | 10 | 8 | 7 | 10 | 9 | 11 |
| GALNT2 | Q10471 | 3.83E-13 | 3.10E-13 | -0.130382 | | 0.668348 | -1.38755 | | 17.1704 | 17.1739 | 17.1438 | 17.5535 | 17.1952 | 17.1503 | 17.2633 | 17.303 | 27 | 26 | 26 | 27 | 24 | 26 | 26 | 26 |
| RPAP1 | Q9BWH6 | 5.91E-13 | 4.53E-13 | -0.130185 | | 0.661754 | -1.37627 | | 14.9852 | 14.9925 | 15.0614 | 15.2809 | 15.188 | 14.8935 | 15.1008 | 15.2528 | 10 | 12 | 14 | 13 | 10 | 10 | 12 | 11 |
| TRIM65 | Q6PJ69 | 0.002684 | 0.000508 | -0.130123 | | 0.511938 | -1.11463 | | 15.1231 | 15.3361 | 15.2164 | 15.5893 | 15.3431 | 15.0837 | 15.3978 | 15.3626 | 5 | 4 | 5 | 5 | 6 | 3 | 4 | 6 |
| RCN1 | Q15293 | 1.88E-15 | 2.98E-15 | -0.129992 | | 0.798942 | -1.60838 | | 19.1968 | 19.2075 | 19.2987 | 19.2373 | 19.3322 | 19.5663 | 19.4074 | 19.2784 | 21 | 19 | 22 | 20 | 19 | 21 | 19 | 20 |
| HADHA | P40939 | 1.47E-22 | 2.27E-21 | -0.129983 | + | 3.14327 | -6.34316 | | 18.9229 | 18.9766 | 18.9564 | 19.0623 | 19.0511 | 19.1202 | 19.0743 | 19.1018 | 40 | 40 | 39 | 39 | 40 | 39 | 39 | 40 |
| PGRMC2 | O15173;O1 | 2.95E-07 | 9.11E-08 | -0.129967 | | 0.239377 | -0.59072 | | 19.793 | 19.7325 | 19.9058 | 19.6186 | 19.8316 | 20.5152 | 20.0592 | 19.6775 | 10 | 9 | 10 | 9 | 8 | 10 | 10 | 9 |
| GSTM3 | P21266 | 6.02E-11 | 3.01E-11 | -0.129915 | | 0.046291 | -0.13253 | | 17.3131 | 17.3363 | 17.3413 | 14.6082 | 18.4042 | 18.2524 | 18.535 | 17.5009 | 13 | 14 | 13 | 7 | 14 | 14 | 14 | 12 |
| LPGAT1 | Q92604 | 4.00E-11 | 2.06E-11 | -0.1299 | | 1.01124 | -1.96185 | | 15.1568 | 15.3218 | 15.2844 | 15.333 | 15.3783 | 15.3597 | 15.5429 | 15.3073 | 4 | 3 | 5 | 4 | 2 | 2 | 5 | 5 |
| SNTA1 | Q13424;Q1 | 0.004418 | 0.000797 | -0.129897 | | 0.218902 | -0.54704 | | 13.7355 | 13.5362 | 13.4649 | 13.1096 | 13.7828 | 14.0883 | 13.5869 | 13.9762 | 2 | 3 | 2 | 1 | 1 | 2 | 2 | 2 |
| STX16 | O14662-2 | 3.56E-09 | 1.37E-09 | -0.129864 | | 1.22526 | -2.319 | | 15.4915 | 15.5032 | 15.4844 | 15.5771 | 15.6925 | 15.642 | 15.7175 | 15.4856 | 7 | 7 | 8 | 7 | 4 | 6 | 7 | 7 |
| IRF2 | P14316;P1 | 0.01079 | 0.001845 | -0.12975 | | 0.24174 | -0.60247 | | 13.5389 | 13.5795 | 13.3173 | 13.8314 | NaN | 13.1017 | 13.8248 | 13.6753 | 2 | 2 | 2 | 1 | NaN | 1 | 1 | 1 |
| CCDC43 | Q96MW1 | 5.20E-06 | 1.40E-06 | -0.129625 | | 0.669154 | -1.38893 | | 16.0572 | 16.0623 | 16.0013 | 16.1535 | 15.9727 | 16.2708 | 16.3676 | 16.0848 | 4 | 4 | 4 | 4 | 4 | 4 | 4 | 4 |
| EMC4 | Q5J8M3;Q | 4.24E-14 | 4.49E-14 | -0.12961 | | 0.571236 | -1.21957 | | 16.6198 | 16.5135 | 16.515 | 16.5878 | 16.5378 | 16.5447 | 16.9173 | 16.8078 | 4 | 4 | 4 | 3 | 4 | 4 | 4 | 4 |
| GSK3A | P49840 | 4.32E-07 | 1.31E-07 | -0.129459 | + | 1.33765 | -2.50913 | | 16.1783 | 15.9932 | 16.0548 | 16.265 | 16.1522 | 16.2643 | 16.1766 | 16.1665 | 5 | 6 | 5 | 5 | 6 | 6 | 6 | 5 |
| NUDCD2 | Q8WVJ2 | 2.23E-16 | 4.69E-16 | -0.12941 | | 0.27457 | -0.66392 | | 16.8086 | 16.5611 | 16.8211 | 16.3192 | 17.0398 | 16.8853 | 17.0666 | 16.9874 | 6 | 5 | 7 | 6 | 5 | 5 | 5 | 5 |
| NDUFA8 | P51970 | 2.56E-09 | 9.99E-10 | -0.129258 | | 0.811835 | -1.62998 | | 16.7616 | 16.6532 | 16.8677 | 16.7628 | 16.8457 | 16.9995 | 17.0097 | 16.8328 | 9 | 9 | 9 | 8 | 8 | 8 | 9 | 8 |
| MCM7 | P33993 | 1.52E-19 | 8.82E-19 | -0.12917 | | 0.148337 | -0.38906 | | 16.7025 | 16.7793 | 16.7451 | 17.3166 | 17.0262 | 15.9677 | 16.7476 | 17.2992 | 25 | 25 | 23 | 26 | 21 | 19 | 25 | 26 |
| TWF1 | Q12792 | 3.63E-16 | 6.97E-16 | -0.129089 | + | 2.51348 | -4.7791 | | 18.0589 | 18.0724 | 18.0208 | 18.113 | 18.18 | 18.2019 | 18.1813 | 18.2225 | 22 | 22 | 22 | 20 | 18 | 20 | 22 | 20 |
| SPART | Q8N0X7 | 9.90E-09 | 3.61E-09 | -0.129013 | + | 1.90355 | -3.5221 | | 16.0005 | 16.009 | 15.9895 | 16.1595 | 16.051 | 16.1833 | 16.1743 | 16.0753 | 20 | 20 | 21 | 21 | 18 | 21 | 21 | 20 |
| MMAA | Q8IVH4 | 1.23E-05 | 3.19E-06 | -0.128775 | | 0.542537 | -1.16905 | | 14.5298 | 14.6441 | 14.692 | 14.5958 | 14.6452 | 14.9963 | 14.6419 | 14.8746 | 6 | 7 | 7 | 5 | 5 | 7 | 5 | 4 |
| MAP1S | Q66K74;Q6 | 3.57E-09 | 1.38E-09 | -0.128628 | | 0.449044 | -1.00072 | | 16.2804 | 16.308 | 16.3754 | 16.7092 | 16.2351 | 16.6155 | 16.4403 | 16.2495 | 19 | 21 | 20 | 20 | 19 | 22 | 20 | 18 |
| SRC | P12931;P1 | 6.72E-18 | 2.29E-17 | -0.128263 | | 0.344334 | -0.8031 | | 16.275 | 16.3112 | 16.2663 | 16.627 | 16.5615 | 16.4282 | 15.9531 | 16.4922 | 10 | 11 | 10 | 11 | 11 | 11 | 11 | 12 |
| PJA2 | O43164;O4 | 3.94E-18 | 1.45E-17 | -0.128222 | | 0.354828 | -0.82345 | | 14.093 | 14.0438 | 14.2075 | 14.3824 | 14.531 | 14.0017 | 13.949 | 14.3508 | 4 | 5 | 5 | 5 | 4 | 4 | 3 | 4 |
| RPP40 | O75818;O7 | 1.71E-09 | 6.85E-10 | -0.128095 | | 0.675699 | -1.40011 | | 15.2637 | 15.0037 | 14.9245 | 15.1044 | 15.2119 | 15.1548 | 15.1546 | 15.3346 | 6 | 7 | 5 | 6 | 5 | 5 | 4 | 5 |

| Gene names | UniProt IDs | Protein Group P-value | Protein Group Q-value | Log2 Difference NUP214 vs Controls (n=5) | p<0.05 | -log10 p-value | T-test Test statistic | NPC Localization | Log2 MS2_quantity_NUP214_1 | Log2 MS2_quantity_NUP214_2 | Log2 MS2_quantity_NUP214_3 | Log2 MS2_quantity_Control_1 | Log2 MS2_quantity_Control_2 | Log2 MS2_quantity_Control_3 | Log2 MS2_quantity_Control_4 | Log2 MS2_quantity_Control_5 | Number Peptides for Quantitation NUP214_1 | Number Peptides for Quantitation NUP214_2 | Number Peptides for Quantitation NUP214_3 | Number Peptides for Quantitation Control_1 | Number Peptides for Quantitation Control_2 | Number Peptides for Quantitation Control_3 | Number Peptides for Quantitation Control_4 | Number Peptides for Quantitation Control_5 |
|---|---|---|---|---|---|---|---|---|---|---|---|---|---|---|---|---|---|---|---|---|---|---|---|---|
| REEP5 | Q00765 | 2.82E-08 | 9.74E-09 | -0.128093 | | 0.299017 | -0.71352 | | 18.1214 | 18.0308 | 17.9677 | 18.0034 | 18.536 | 17.7719 | 18.3438 | 18.1852 | 6 | 6 | 6 | 6 | 6 | 6 | 6 | 6 |
| PIGT | Q969N2;Q | 3.63E-17 | 9.75E-17 | -0.127909 | | 0.679209 | -1.4061 | | 15.9595 | 16.0316 | 15.8981 | 15.9657 | 16.3266 | 15.9789 | 16.0776 | 16.1059 | 16 | 16 | 15 | 15 | 16 | 14 | 17 | 14 |
| NDE1 | Q9NXR1;Q | 1.96E-10 | 9.00E-11 | -0.127761 | | 0.738404 | -1.50656 | | 15.1131 | 15.0738 | 15.1596 | 15.2055 | 15.0633 | 15.1803 | 15.3891 | 15.378 | 9 | 11 | 12 | 9 | 10 | 9 | 12 | 10 |
| ZFAND1 | Q8TCF1 | 2.22E-10 | 1.01E-10 | -0.127657 | | 0.464623 | -1.02922 | | 14.8583 | 15.091 | 14.9516 | 14.8522 | 15.1051 | 15.3793 | 15.115 | 15.0215 | 5 | 5 | 5 | 5 | 5 | 5 | 6 | 4 |
| SEC61A1 | P61619 | 7.56E-12 | 4.50E-12 | -0.127501 | | 1.16551 | -2.21879 | | 19.4164 | 19.2763 | 19.3181 | 19.3686 | 19.4378 | 19.4699 | 19.5938 | 19.4521 | 9 | 8 | 8 | 9 | 9 | 8 | 9 | 9 |
| TFB1M | Q8WVM0 | 5.28E-07 | 1.59E-07 | -0.127491 | | 0.188064 | -0.47953 | | 15.4749 | 15.4586 | 15.5415 | 15.6309 | 16.0322 | 15.0349 | 15.3338 | 16.0639 | 5 | 3 | 5 | 5 | 3 | 3 | 2 | 4 |
| UBXN8 | O00124;O | 0.000449 | 9.72E-05 | -0.127454 | | 0.168168 | -0.43476 | | 14.2738 | 14.1683 | 14.5345 | 14.2114 | 14.0401 | 14.6521 | 15.1924 | 14.1691 | 2 | 1 | 1 | 1 | 1 | 2 | 2 | 2 |
| RPTOR | Q8N122 | 2.77E-07 | 8.60E-08 | -0.127437 | | 0.697968 | -1.43805 | | 15.6122 | 15.678 | 15.6804 | 15.6659 | 15.7599 | 15.8791 | 15.9824 | 15.6343 | 16 | 17 | 17 | 15 | 11 | 15 | 15 | 15 |
| JUND | P17535 | 2.05E-12 | 1.37E-12 | -0.127248 | | 0.36152 | -0.83636 | | 15.2274 | 15.2642 | 15.0871 | 15.5451 | 15.4545 | 14.9184 | 15.264 | 15.4186 | 4 | 4 | 4 | 2 | 2 | 2 | 2 | 3 |
| DNAJA2 | O60884 | 3.43E-13 | 2.82E-13 | -0.127154 | + | 2.16991 | -4.04527 | | 16.7673 | 16.7761 | 16.7525 | 16.9227 | 16.9151 | 16.8322 | 16.8426 | 16.9498 | 16 | 16 | 16 | 16 | 16 | 16 | 15 | 16 |
| ACTR10 | Q9NZ32 | 5.24E-13 | 4.08E-13 | -0.127116 | | 0.706853 | -1.45315 | | 16.4418 | 16.4397 | 16.4353 | 16.575 | 16.7213 | 16.339 | 16.5328 | 16.6621 | 14 | 14 | 14 | 14 | 14 | 14 | 13 | 14 |
| ABAT | P80404 | 0.001201 | 0.000245 | -0.127075 | | 0.081905 | -0.22678 | | 14.4151 | 14.5871 | 14.4378 | 14.3367 | 16.187 | 13.8797 | 13.9645 | 14.6676 | 5 | 3 | 4 | 2 | 6 | 2 | 2 | 3 |
| NLE1 | Q9NVX2 | 1.77E-06 | 5.00E-07 | -0.126961 | + | 1.63158 | -3.02133 | | 15.6437 | 15.6102 | 15.7012 | 15.8158 | 15.7354 | 15.7914 | 15.6972 | 15.8534 | 4 | 4 | 3 | 6 | 4 | 4 | 5 | 4 |
| HEXIM1 | O94992 | 8.41E-05 | 2.01E-05 | -0.12683 | | 0.583292 | -1.24066 | | 15.393 | 15.1358 | 15.3182 | 15.1996 | 15.3991 | 15.3614 | 15.5275 | 15.5582 | 6 | 5 | 5 | 4 | 4 | 5 | 6 | 4 |
| RPS23 | P62266 | 0.001262 | 0.000257 | -0.12676 | + | 1.85037 | -3.42176 | | 20.5335 | 20.4922 | 20.4817 | 20.5526 | 20.7068 | 20.5984 | 20.6624 | 20.6259 | 7 | 7 | 7 | 7 | 7 | 7 | 7 | 7 |
| POLD3 | Q15054;Q | 0.002575 | 0.00049 | -0.126619 | | 0.261481 | -0.63696 | | 13.5209 | 13.3813 | 13.0349 | 13.6186 | 13.6214 | 13.0056 | 13.2965 | 13.6527 | 1 | 2 | 2 | 3 | 1 | 1 | 1 | 1 |
| TICAM2 | Q86XR7-2 | 6.22E-15 | 8.35E-15 | -0.126576 | | 0.576691 | -1.22912 | | 18.5911 | 18.6545 | 18.6319 | 18.6468 | 18.7473 | 18.7377 | 19.0365 | 18.5938 | 8 | 7 | 8 | 7 | 7 | 7 | 7 | 8 |
| RPS18 | P62269 | 0.001065 | 0.000219 | -0.1263 | + | 1.9538 | -3.6171 | | 20.6889 | 20.6237 | 20.6316 | 20.7137 | 20.82 | 20.7586 | 20.8382 | 20.7412 | 15 | 15 | 15 | 15 | 15 | 15 | 15 | 15 |
| LRPPRC | P42704 | 4.09E-11 | 2.10E-11 | -0.126234 | | 1.049 | -2.02459 | | 17.6443 | 17.6724 | 17.6412 | 17.8454 | 17.8474 | 17.5975 | 17.8073 | 17.7968 | 72 | 72 | 73 | 72 | 71 | 69 | 70 | 70 |
| NPM1 | P06748;P0 | 2.55E-09 | 9.98E-10 | -0.126197 | | 1.14251 | -2.18035 | | 19.5774 | 19.5307 | 19.5889 | 19.7409 | 19.8008 | 19.7095 | 19.5503 | 19.6578 | 14 | 13 | 15 | 14 | 14 | 14 | 13 | 14 |
| RAB14 | P61106 | 1.45E-13 | 1.32E-13 | -0.126192 | + | 1.4349 | -2.67589 | | 18.8405 | 18.8504 | 18.8023 | 18.9863 | 19.0647 | 18.8702 | 18.9682 | 18.8969 | 13 | 14 | 13 | 13 | 13 | 13 | 13 | 13 |
| BCKDK | O14874;O | 0.000989 | 0.000204 | -0.126125 | | 0.375832 | -0.86379 | | 14.4518 | 14.688 | 14.4178 | 14.8987 | 14.6432 | 14.5279 | 14.3444 | 14.8125 | 4 | 3 | 3 | 2 | 2 | 3 | 4 | 5 |
| WDR74 | Q6RFH5;Q | 0.000325 | 7.19E-05 | -0.12603 | | 0.414366 | -0.93652 | | 16.0302 | 15.7725 | 15.9654 | 16.008 | 15.7253 | 16.0723 | 16.1807 | 16.2574 | 6 | 6 | 4 | 8 | 3 | 7 | 5 | 6 |
| DPP9 | Q86TI2;Q8 | 2.84E-10 | 1.27E-10 | -0.125838 | + | 1.78085 | -3.29244 | | 16.6322 | 16.7086 | 16.6607 | 16.8409 | 16.8285 | 16.701 | 16.8238 | 16.7708 | 21 | 21 | 23 | 23 | 23 | 21 | 23 | 22 |
| XRCC4 | Q13426-2 | 4.78E-13 | 3.76E-13 | -0.125705 | | 0.279279 | -0.67355 | | 14.794 | 14.8932 | 14.965 | 14.6657 | 15.3506 | 14.7995 | 15.311 | 14.9221 | 8 | 9 | 7 | 9 | 4 | 5 | 7 | 6 |
| TTC27 | Q6P3X3 | 4.12E-06 | 1.12E-06 | -0.125691 | | 1.20846 | -2.29077 | | 15.0546 | 14.9521 | 14.9681 | 15.1475 | 15.2274 | 15.0911 | 14.9989 | 15.1216 | 13 | 14 | 15 | 14 | 12 | 12 | 13 | 12 |
| PLAUR | Q03405 | 5.66E-12 | 3.46E-12 | -0.12566 | | 0.319932 | -0.75522 | | 15.4161 | 15.3198 | 15.3257 | 15.2615 | 15.3043 | 15.612 | 15.9064 | 15.3134 | 7 | 7 | 7 | 7 | 5 | 6 | 7 | 7 |
| GAPVD1 | Q14C86;Q | 2.20E-18 | 8.68E-18 | -0.125412 | | 0.758446 | -1.54036 | | 15.9558 | 15.9682 | 15.9625 | 16.2223 | 15.9468 | 16.1765 | 16.1584 | 15.9339 | 25 | 28 | 30 | 26 | 23 | 27 | 28 | 25 |
| TRIO | O75962 | 9.58E-14 | 9.20E-14 | -0.125319 | | 1.01907 | -1.97485 | | 16.0423 | 16.0599 | 16.0388 | 16.2291 | 16.1792 | 16.2934 | 16.009 | 16.1509 | 63 | 59 | 63 | 62 | 53 | 61 | 56 | 58 |
| NT5C2 | P49902 | 1.08E-16 | 2.53E-16 | -0.124897 | | 0.909422 | -1.79271 | | 16.4506 | 16.5216 | 16.5133 | 16.8118 | 16.5671 | 16.5816 | 16.6224 | 16.5174 | 18 | 18 | 18 | 18 | 17 | 17 | 17 | 16 |
| ATP6V1B2 | P21281 | 1.53E-21 | 1.66E-20 | -0.124807 | | 0.843152 | -1.68232 | | 18.5059 | 18.459 | 18.4235 | 18.4828 | 18.5024 | 18.6397 | 18.775 | 18.5382 | 23 | 22 | 22 | 24 | 24 | 23 | 23 | 24 |
| EIF2B2 | P49770 | 2.97E-08 | 1.02E-08 | -0.124782 | + | 1.52963 | -2.84077 | | 16.1507 | 16.1094 | 16.1034 | 16.2075 | 16.3366 | 16.154 | 16.2388 | 16.2928 | 13 | 13 | 12 | 13 | 12 | 12 | 12 | 11 |
| DPF2 | Q92785 | 2.53E-11 | 1.36E-11 | -0.124725 | | 0.526779 | -1.1411 | | 15.1441 | 15.0999 | 15.2342 | 15.2591 | 15.3476 | 14.989 | 15.4007 | 15.4243 | 8 | 7 | 8 | 7 | 6 | 8 | 9 | 8 |
| LRRC57 | Q8N9N7 | 0.001298 | 0.000263 | -0.12466 | | 0.488479 | -1.07249 | | 16.4642 | 16.2853 | 16.3703 | 16.5917 | 16.6411 | 16.1781 | 16.5615 | 16.5171 | 6 | 6 | 6 | 6 | 6 | 5 | 6 | 6 |
| DUSP23 | Q9BVJ7 | 3.81E-08 | 1.29E-08 | -0.124532 | | 0.345968 | -0.80628 | | 15.439 | 15.3681 | 15.6848 | 15.4635 | 15.7771 | 15.2926 | 15.8013 | 15.7746 | 10 | 9 | 10 | 9 | 9 | 8 | 10 | 10 |
| FAM210A | Q96ND0 | 1.09E-06 | 3.15E-07 | -0.124393 | | 0.799748 | -1.60973 | | 14.8404 | 14.9998 | 14.8163 | 15.153 | 14.935 | 14.893 | 15.0906 | 14.9778 | 4 | 5 | 4 | 5 | 3 | 4 | 4 | 4 |
| CAPZA2 | P47755 | 1.66E-05 | 4.26E-06 | -0.124272 | + | 2.0297 | -3.76541 | | 19.5708 | 19.6522 | 19.5847 | 19.7631 | 19.7527 | 19.6916 | 19.7628 | 19.6641 | 16 | 17 | 16 | 16 | 16 | 16 | 16 | 16 |
| ARHGEF7 | Q14155-1; | 2.48E-06 | 6.92E-07 | -0.12394 | + | 1.66982 | -3.08998 | | 16.6719 | 16.6722 | 16.7303 | 16.747 | 16.7907 | 16.8466 | 16.9083 | 16.7846 | 19 | 19 | 20 | 18 | 17 | 17 | 17 | 14 |
| PLSCR3 | Q9NRY6 | 0.000113 | 2.66E-05 | -0.123927 | | 0.623888 | -1.31116 | | 17.2571 | 17.2579 | 17.273 | 17.4861 | 17.2851 | 17.6157 | 17.3039 | 17.2423 | 5 | 5 | 5 | 6 | 5 | 6 | 4 | 6 |
| PIK3C2A | O00443 | 9.85E-10 | 4.09E-10 | -0.123635 | | 0.489782 | -1.07484 | | 14.7423 | 14.8099 | 14.8891 | 15.2171 | 14.8054 | 14.7641 | 15.0279 | 14.8725 | 22 | 23 | 21 | 22 | 12 | 19 | 21 | 14 |
| PIH1D1 | Q9NWS0 | 1.02E-06 | 2.95E-07 | -0.12345 | | 0.633078 | -1.32701 | | 16.2888 | 16.1348 | 16.5197 | 16.5669 | 16.4194 | 16.3876 | 16.4299 | 16.3856 | 5 | 5 | 5 | 5 | 4 | 4 | 5 | 5 |
| NAXD | Q8IW45 | 5.15E-15 | 7.10E-15 | -0.123415 | | 0.49355 | -1.08164 | | 16.9066 | 16.9578 | 16.8798 | 16.9084 | 17.1685 | 17.2992 | 16.8477 | 16.967 | 7 | 8 | 7 | 9 | 7 | 9 | 8 | 9 |
| RPL34 | P49207 | 6.92E-05 | 1.67E-05 | -0.123207 | + | 1.82727 | -3.37855 | | 20.5391 | 20.5824 | 20.4605 | 20.6546 | 20.7209 | 20.644 | 20.6134 | 20.6199 | 4 | 4 | 4 | 3 | 4 | 4 | 4 | 4 |
| ZEB2 | O60315;O | 8.12E-15 | 1.05E-14 | -0.122915 | | 0.73254 | -1.49665 | | 14.3834 | 14.2355 | 14.3943 | 14.6638 | 14.3809 | 14.4226 | 14.3571 | 14.4787 | 4 | 4 | 4 | 2 | 2 | 4 | 3 | 3 |
| NIT2 | Q9NQR4 | 4.94E-15 | 6.85E-15 | -0.12284 | | 0.771266 | -1.56193 | | 17.1508 | 17.2205 | 17.2011 | 17.2952 | 17.2822 | 17.5372 | 17.2445 | 17.2092 | 19 | 19 | 19 | 19 | 17 | 19 | 19 | 19 |
| ITCH | Q96J02;Q | 1.03E-13 | 9.80E-14 | -0.122812 | | 0.657479 | -1.36895 | | 15.5413 | 15.6117 | 15.5807 | 15.922 | 15.5165 | 15.7401 | 15.6439 | 15.6811 | 19 | 22 | 24 | 25 | 20 | 23 | 21 | 21 |
| BLOC1S3 | Q6QNY0 | 5.22E-05 | 1.28E-05 | -0.122664 | | 0.399571 | -0.90878 | | 16.1852 | 16.1458 | 16.386 | 16.18 | 16.2134 | 16.6868 | 16.4382 | 16.2897 | 6 | 6 | 7 | 6 | 6 | 7 | 7 | 7 |
| EEF1E1 | O43324 | 1.09E-06 | 3.15E-07 | -0.122466 | | 0.207833 | -0.52306 | | 17.497 | 17.452 | 17.7599 | 17.8723 | 17.4644 | 17.147 | 18.0351 | 17.9417 | 6 | 6 | 6 | 7 | 6 | 6 | 6 | 6 |
| MAN2A2 | P49641;P4 | 0.003445 | 0.000633 | -0.12243 | | 0.355839 | -0.8254 | | 16.5489 | 16.0231 | 16.0503 | 16.2272 | 16.2345 | 15.9442 | 15.9378 | 15.8057 | 3 | 1 | 3 | 3 | 2 | 2 | 3 | 3 |
| PTP4A2 | Q12974 | 1.68E-07 | 5.32E-08 | -0.122235 | | 0.528946 | -1.14495 | | 16.0793 | 16.1401 | 16.1694 | 16.5301 | 16.3087 | 16.084 | 16.1438 | 16.1926 | 6 | 6 | 6 | 6 | 5 | 6 | 6 | 6 |
| C1GALT1C1 | Q96EU7 | 3.53E-07 | 1.08E-07 | -0.122016 | | 0.855657 | -1.70318 | | 15.0041 | 15.1509 | 15.0148 | 15.0628 | 15.264 | 15.1689 | 15.092 | 15.3053 | 7 | 6 | 8 | 7 | 6 | 6 | 8 | 5 |
| YKT6 | O15498 | 1.13E-15 | 1.91E-15 | -0.121471 | + | 1.65504 | -3.06339 | | 18.0178 | 17.9845 | 18.0335 | 18.1689 | 18.0453 | 18.0982 | 18.2114 | 18.1432 | 13 | 13 | 13 | 13 | 14 | 13 | 13 | 13 |
| MINDY3 | Q9H8M7 | 1.31E-15 | 2.17E-15 | -0.120856 | | 0.590515 | -1.25326 | | 16.2862 | 16.3265 | 16.2489 | 16.5386 | 16.2888 | 16.6206 | 16.2918 | 16.3003 | 9 | 8 | 9 | 8 | 7 | 9 | 8 | 7 |
| VPS41 | P49754 | 2.19E-13 | 1.90E-13 | -0.120831 | | 1.05438 | -2.03353 | | 15.3843 | 15.4106 | 15.3497 | 15.5027 | 15.5374 | 15.4752 | 15.6321 | 15.3643 | 21 | 22 | 22 | 22 | 19 | 22 | 21 | 20 |
| RASA4B;RASA4;RASA4 | C9J798;O4 | 0.003447 | 0.000633 | -0.120765 | | 0.297157 | -0.70978 | | 16.8275 | 16.832 | 16.7409 | 16.7033 | 16.6092 | 17.3163 | 17.0605 | 16.9151 | 1 | 1 | 1 | 1 | 1 | 3 | 2 | 2 |
| RPL11 | P62913-2 | 8.63E-08 | 2.80E-08 | -0.120562 | + | 1.71278 | -3.16775 | | 20.2791 | 20.4169 | 20.3561 | 20.4506 | 20.514 | 20.5163 | 20.4272 | 20.4482 | 13 | 13 | 13 | 12 | 12 | 12 | 12 | 12 |
| SASH1 | O94885 | 2.27E-22 | 3.17E-21 | -0.120212 | | 0.739453 | -1.50833 | | 15.4945 | 15.4754 | 15.5203 | 15.7955 | 15.493 | 15.5925 | 15.4966 | 15.7071 | 29 | 27 | 28 | 28 | 25 | 27 | 27 | 30 |
| ERMP1 | Q7Z2K6 | 9.15E-06 | 2.41E-06 | -0.120201 | | 0.593393 | -1.25828 | | 14.8916 | 14.7574 | 14.7724 | 15.0819 | 14.7335 | 14.8011 | 15.0294 | 14.9908 | 11 | 10 | 11 | 13 | 8 | 9 | 10 | 9 |
| PHLDB1 | Q86UU1-2 | 1.22E-13 | 1.13E-13 | -0.120121 | | 1.19895 | -2.27481 | | 15.9625 | 15.9561 | 15.9742 | 16.0394 | 16.0599 | 16.2399 | 16.0597 | 16.0229 | 35 | 37 | 37 | 36 | 30 | 38 | 35 | 33 |
| BPNT2 | Q9NX62 | 2.56E-15 | 3.90E-15 | -0.120096 | | 0.421343 | -0.94953 | | 16.2422 | 16.4477 | 16.4092 | 16.7182 | 16.1788 | 16.5109 | 16.5662 | 16.4582 | 13 | 11 | 12 | 12 | 11 | 13 | 13 | 11 |
| ATP6V0A2 | Q9Y487 | 3.39E-07 | 1.04E-07 | -0.120003 | | 0.256085 | -0.62575 | | 15.2503 | 15.1163 | 15.2775 | 15.1413 | 15.1395 | 15.7987 | 15.5264 | 15.0676 | 9 | 8 | 11 | 9 | 8 | 11 | 9 | 8 |
| PRRC2C | Q9Y520;Q9 | 1.02E-12 | 7.35E-13 | -0.119905 | + | 1.60463 | -2.97326 | | 15.5035 | 15.5378 | 15.5803 | 15.6832 | 15.6517 | 15.5603 | 15.7259 | 15.6812 | 56 | 56 | 54 | 52 | 53 | 48 | 54 | 54 |
| KRIT1 | O00522 | 3.83E-07 | 1.17E-07 | -0.119753 | | 1.15254 | -2.19712 | | 14.5539 | 14.5296 | 14.5884 | 14.7129 | 14.8034 | 14.5618 | 14.6484 | 14.6589 | 8 | 7 | 7 | 5 | 5 | 6 | 7 | 7 |
| GLTP | Q9NZD2 | 2.33E-05 | 5.91E-06 | -0.119561 | | 0.097908 | -0.26733 | | 16.0195 | 15.6126 | 16.4775 | 16.6366 | 15.8388 | 15.2597 | 16.0372 | 17.0084 | 5 | 4 | 7 | 7 | 4 | 3 | 5 | 7 |
| KTI12 | Q96EK9 | 1.82E-10 | 8.42E-11 | -0.119533 | | 0.63452 | -1.3295 | | 15.914 | 15.8811 | 15.8021 | 16.0551 | 16.152 | 15.8672 | 15.8022 | 16.05 | 7 | 7 | 7 | 8 | 2 | 3 | 6 | 5 |
| SACM1L | Q9NTJ5 | 3.75E-14 | 4.01E-14 | -0.119462 | + | 1.86776 | -3.45443 | | 17.3223 | 17.3078 | 17.3296 | 17.4113 | 17.4149 | 17.3979 | 17.5397 | 17.433 | 26 | 26 | 27 | 26 | 28 | 28 | 29 | 29 |
| SMARCD3 | Q6STE5;Q6 | 4.89E-08 | 1.64E-08 | -0.119402 | | 0.512967 | -1.11647 | | 16.3129 | 16.3052 | 16.3404 | 16.3265 | 16.6478 | 16.2053 | 16.4453 | 16.5697 | 4 | 5 | 4 | 4 | 3 | 4 | 5 | 5 |
| PTMA | P06454;P0 | 2.45E-15 | 3.76E-15 | -0.119393 | | 0.201401 | -0.509 | | 16.0795 | 16.4988 | 16.645 | 16.5039 | 15.9635 | 16.0357 | 16.4917 | 16.641 | 4 | 3 | 4 | 4 | 3 | 4 | 4 | 4 |
| EEF2 | P13639 | 2.99E-14 | 3.29E-14 | -0.119324 | + | 3.00199 | -5.96403 | | 21.125 | 21.1565 | 21.1024 | 21.2473 | 21.2129 | 21.2768 | 21.2717 | 21.2279 | 63 | 63 | 63 | 59 | 61 | 63 | 61 | 62 |
| GTF2B | Q00403 | 2.71E-16 | 5.44E-16 | -0.119133 | | 0.425851 | -0.9579 | | 15.3508 | 15.277 | 15.2735 | 15.7345 | 15.3396 | 15.1663 | 15.4413 | 15.4163 | 11 | 12 | 10 | 11 | 8 | 11 | 10 | 12 |
| TUBA1A | Q71U36-2 | 5.32E-10 | 2.28E-10 | -0.119002 | | 0.667856 | -1.38671 | | 23.7313 | 23.7612 | 23.764 | 23.8904 | 24.0965 | 23.7942 | 23.7129 | 23.8619 | 26 | 27 | 27 | 27 | 25 | 27 | 26 | 26 |
| EXOC4 | Q96A65 | 1.05E-13 | 9.90E-14 | -0.118944 | + | 1.69174 | -3.12956 | | 16.2085 | 16.2842 | 16.2711 | 16.4441 | 16.2976 | 16.339 | 16.4046 | 16.3823 | 35 | 34 | 35 | 34 | 29 | 32 | 32 | 31 |
| KDELR3 | O43731-2 | 9.87E-06 | 2.59E-06 | -0.118834 | | 0.188764 | -0.48109 | | 18.7063 | 18.5869 | 18.7285 | 18.6924 | 18.3059 | 18.5543 | 19.3282 | 19.0829 | 5 | 5 | 5 | 5 | 3 | 5 | 5 | 4 |
| NAA50 | Q9GZZ1 | 2.13E-09 | 8.40E-10 | -0.118809 | | 0.435283 | -0.97538 | | 16.9395 | 16.9182 | 16.9677 | 17.2781 | 17.1559 | 16.7309 | 17.0543 | 17.0839 | 11 | 10 | 11 | 11 | 10 | 10 | 11 | 11 |
| REPS1 | Q96D71;Q | 5.11E-09 | 1.93E-09 | -0.118769 | | 0.419028 | -0.94521 | | 14.9134 | 14.9182 | 14.8951 | 15.0904 | 15.1794 | 14.754 | 15.2492 | 14.8652 | 10 | 8 | 7 | 6 | 6 | 6 | 8 | 5 |
| SPOUT1 | Q5T280 | 0.002644 | 0.000501 | -0.11843 | | 0.214291 | -0.53708 | | 16.5452 | 16.2796 | 16.4372 | 16.9226 | 16.66 | 16.3901 | 15.9979 | 16.7247 | 4 | 4 | 3 | 5 | 5 | 3 | 4 | 5 |
| FN1 | P02751-1; | 1.35E-12 | 9.38E-13 | -0.118327 | | 0.114184 | -0.30757 | | 19.3384 | 19.3766 | 19.3433 | 18.4727 | 19.7408 | 20.2402 | 19.4845 | 19.417 | 82 | 80 | 81 | 76 | 79 | 83 | 83 | 80 |
| EDF1 | O60869 | 1.29E-10 | 6.06E-11 | -0.118253 | | 0.607882 | -1.28345 | | 17.2484 | 17.2506 | 17.2208 | 17.4227 | 17.426 | 17.0871 | 17.3885 | 17.4667 | 9 | 9 | 9 | 9 | 9 | 9 | 9 | 9 |

| Gene names | UniProt IDs | Protein Group P-value | Protein Group Q-value | Log2 Difference NUP214 vs Controls (n=5) | p<0.05 | -log10 p-value | T-test Test statistic | NPC Localization | Log2 MS2_quantity_NUP214_1 | Log2 MS2_quantity_NUP214_2 | Log2 MS2_quantity_NUP214_3 | Log2 MS2_quantity_Control_1 | Log2 MS2_quantity_Control_2 | Log2 MS2_quantity_Control_3 | Log2 MS2_quantity_Control_4 | Log2 MS2_quantity_Control_5 | Number Peptides for Quantitation NUP214_1 | Number Peptides for Quantitation NUP214_2 | Number Peptides for Quantitation NUP214_3 | Number Peptides for Quantitation Control_1 | Number Peptides for Quantitation Control_2 | Number Peptides for Quantitation Control_3 | Number Peptides for Quantitation Control_4 | Number Peptides for Quantitation Control_5 |
|---|---|---|---|---|---|---|---|---|---|---|---|---|---|---|---|---|---|---|---|---|---|---|---|---|
| PHACTR2 | O75167;O7 | 8.77E-11 | 4.25E-11 | -0.118175 | | 0.176402 | -0.45341 | | 14.365 | 14.4628 | 14.4396 | 15.2522 | 14.3435 | 14.4235 | 14.5882 | 14.0958 | 3 | 3 | 3 | 4 | 2 | 3 | 3 | 3 |
| C2CD2 | Q9Y426;Q9 | 0.003213 | 0.000594 | -0.118134 | | 0.170684 | -0.44048 | | 14.3062 | 14.2675 | 13.9951 | 14.4447 | 14.8667 | 13.7056 | 14.4244 | 14.0974 | 4 | 5 | 6 | 6 | 3 | 4 | 6 | 5 |
| RCL1 | Q9Y2P8 | 0.000124 | 2.89E-05 | -0.118009 | + | 2.36033 | -4.44299 | | 16.2059 | 16.2298 | 16.1934 | 16.2989 | 16.2937 | 16.3168 | 16.3994 | 16.3297 | 5 | 6 | 6 | 5 | 7 | 7 | 7 | 5 |
| KIF2A | O00139;O0 | 8.24E-17 | 1.99E-16 | -0.117933 | | 1.12703 | -2.15452 | | 16.1639 | 16.1761 | 16.1536 | 16.4047 | 16.2183 | 16.1983 | 16.2364 | 16.3546 | 19 | 18 | 19 | 16 | 19 | 19 | 19 | 18 |
| PSMC4 | P43686 | 1.11E-22 | 1.84E-21 | -0.117928 | + | 2.31777 | -4.35222 | | 17.7818 | 17.8527 | 17.803 | 17.9222 | 17.8974 | 17.954 | 17.9825 | 17.896 | 26 | 25 | 26 | 28 | 25 | 26 | 26 | 26 |
| RBM14 | Q96PK6 | 8.13E-14 | 7.93E-14 | -0.117812 | | 0.679927 | -1.40733 | | 17.7175 | 17.7566 | 17.7251 | 17.965 | 17.8831 | 17.6831 | 17.7283 | 17.995 | 22 | 22 | 21 | 22 | 20 | 22 | 23 | 21 |
| DTWD1 | Q8N5C7;Q8 | 0.000118 | 2.77E-05 | -0.117731 | | 0.155871 | -0.4105 | | 13.3702 | 13.9043 | 14.0436 | 13.6906 | 14.4641 | 13.7854 | NaN | 13.6215 | 3 | 3 | 4 | 3 | 3 | 3 | NaN | 3 |
| NTMT1 | Q9BV86 | 3.98E-07 | 1.21E-07 | -0.117584 | | 0.742868 | -1.5141 | | 17.5767 | 17.5315 | 17.5152 | 17.7128 | 17.8321 | 17.5073 | 17.5614 | 17.6799 | 8 | 8 | 8 | 7 | 8 | 7 | 8 | 8 |
| PRKAR2A | P13861;P1 | 2.06E-13 | 1.80E-13 | -0.117267 | | 0.98507 | -1.91839 | | 17.4483 | 17.3991 | 17.3589 | 17.3893 | 17.4961 | 17.5321 | 17.6622 | 17.5171 | 19 | 19 | 19 | 18 | 19 | 20 | 20 | 19 |
| DIP2A | Q14689;Q1 | 4.26E-06 | 1.16E-06 | -0.117237 | | 0.997868 | -1.93964 | | 15.7725 | 15.8149 | 15.8209 | 16.0657 | 15.7898 | 15.9093 | 15.8914 | 15.9439 | 12 | 15 | 16 | 17 | 12 | 13 | 15 | 16 |
| CMAS | Q8NFW8 | 5.44E-07 | 1.63E-07 | -0.117178 | | 0.312278 | -0.74003 | | 15.7528 | 15.6894 | 15.6737 | 15.8912 | 15.5498 | 16.2363 | 15.7732 | 15.6617 | 11 | 11 | 11 | 11 | 9 | 11 | 11 | 11 |
| GPSM1 | Q86YR5;Q8 | 3.47E-11 | 1.81E-11 | -0.116786 | | 1.2446 | -2.35156 | | 16.5885 | 16.5792 | 16.557 | 16.5795 | 16.6971 | 16.6921 | 16.678 | 16.8118 | 18 | 18 | 18 | 18 | 20 | 17 | 20 | 20 |
| DCUN1D3 | Q8IWE4 | 2.92E-14 | 3.23E-14 | -0.116655 | | 0.914694 | -1.80147 | | 15.4563 | 15.4779 | 15.4728 | 15.4716 | 15.6448 | 15.6663 | 15.6813 | 15.4641 | 10 | 10 | 10 | 11 | 10 | 10 | 10 | 10 |
| NAA35 | Q5VZE5 | 7.03E-06 | 1.87E-06 | -0.116554 | | 1.25049 | -2.36148 | | 15.4105 | 15.4063 | 15.406 | 15.477 | 15.6038 | 15.4267 | 15.6167 | 15.4964 | 11 | 12 | 12 | 11 | 8 | 11 | 9 | 10 |
| AIFM2 | Q9BRQ8 | 8.59E-09 | 3.15E-09 | -0.116307 | | 0.309894 | -0.73528 | | 15.5021 | 15.4943 | 15.4874 | 15.9472 | 15.4464 | 15.8325 | 15.5002 | 15.3283 | 7 | 8 | 6 | 8 | 7 | 6 | 7 | 6 |
| FLOT2 | Q14254 | 4.29E-14 | 4.54E-14 | -0.116062 | | 0.365987 | -0.84494 | | 17.2278 | 17.2232 | 17.2018 | 17.6431 | 17.4456 | 17.0213 | 17.2742 | 17.2842 | 24 | 23 | 23 | 24 | 23 | 23 | 23 | 24 |
| LAMTOR4 | Q0VGL1 | 0.003884 | 0.000707 | -0.116025 | | 0.225055 | -0.56026 | | 16.3858 | 16.2187 | 16.4854 | 16.7592 | 15.9065 | 16.6629 | 16.5385 | 16.5294 | 2 | 2 | 2 | 2 | 2 | 2 | 2 | 2 |
| RPL37A | P61513 | 1.06E-12 | 7.61E-13 | -0.11573 | | 1.11359 | -2.1321 | | 19.2039 | 19.2745 | 19.2591 | 19.3864 | 19.4648 | 19.3753 | 19.3574 | 19.224 | 5 | 5 | 5 | 5 | 5 | 5 | 5 | 5 |
| B4GALT1 | P15291;P1 | 0.002398 | 0.00046 | -0.115655 | | 0.208945 | -0.52548 | | 14.7766 | 14.079 | 14.2543 | 14.1706 | 14.3177 | 14.8429 | 14.4518 | 14.6449 | 2 | 3 | 3 | 3 | 2 | 3 | 3 | 3 |
| NOC4L | Q9BVI4 | 1.79E-13 | 1.59E-13 | -0.115628 | | 0.707359 | -1.454 | | 15.1508 | 15.2356 | 15.2101 | 15.4625 | 15.2435 | 15.1307 | 15.3896 | 15.346 | 11 | 14 | 13 | 12 | 12 | 12 | 12 | 13 |
| EXOC6B | Q9Y2D4 | 1.26E-06 | 3.61E-07 | -0.115607 | + | 1.87375 | -3.4657 | | 16.0685 | 16.1173 | 16.1031 | 16.1356 | 16.2672 | 16.1938 | 16.2569 | 16.2059 | 20 | 22 | 20 | 21 | 17 | 20 | 19 | 21 |
| WAC | Q9BTA9;Q9 | 0.003405 | 0.000626 | -0.115471 | | 0.473985 | -1.04626 | | 14.5412 | 14.3546 | 14.7115 | 14.7699 | 14.4562 | 14.7018 | 14.5684 | 14.7598 | 5 | 5 | 5 | 4 | 4 | 5 | 4 | 4 |
| GNS | P15586 | 1.06E-07 | 3.42E-08 | -0.115368 | | 0.474223 | -1.04669 | | 18.8175 | 18.7825 | 18.8008 | 18.617 | 18.9403 | 18.9595 | 19.1252 | 18.9362 | 13 | 13 | 13 | 13 | 12 | 13 | 14 | 13 |
| NOL6 | Q9H6R4;Q9 | 1.45E-06 | 4.15E-07 | -0.115317 | + | 1.58133 | -2.93189 | | 15.5263 | 15.4658 | 15.4871 | 15.6999 | 15.5637 | 15.5678 | 15.5637 | 15.6471 | 12 | 13 | 12 | 14 | 7 | 9 | 12 | 10 |
| BRD1 | O95696;O9 | 1.89E-08 | 6.62E-09 | -0.115219 | | 0.403403 | -0.91598 | | 15.3833 | 15.7778 | 15.7644 | 15.8294 | 15.8649 | 15.518 | 15.7556 | 15.8174 | 2 | 2 | 2 | 3 | 2 | 2 | 4 | 2 |
| POMT2 | Q9UKY4 | 0.000484 | 0.000104 | -0.115034 | | 0.334238 | -0.78338 | | 15.376 | 15.2172 | 15.1823 | 15.2027 | 15.0497 | 15.4959 | 15.5213 | 15.5981 | 6 | 5 | 4 | 4 | 5 | 4 | 4 | 4 |
| GFM2 | Q969S9;Q9 | 1.06E-11 | 6.13E-12 | -0.114845 | | 0.33003 | -0.77513 | | 17.7198 | 17.9276 | 17.8533 | 18.0162 | 17.6435 | 18.213 | 17.7661 | 18.1032 | 4 | 5 | 5 | 6 | 8 | 5 | 6 | 6 |
| ATAD1 | Q8NBU5 | 2.80E-11 | 1.49E-11 | -0.114641 | | 0.650774 | -1.35745 | | 16.0508 | 15.9474 | 15.9969 | 15.9682 | 15.9918 | 16.2094 | 16.286 | 16.1094 | 15 | 15 | 14 | 15 | 13 | 14 | 15 | 14 |
| ELOA | Q14241 | 1.33E-12 | 9.30E-13 | -0.114635 | | 0.530129 | -1.14705 | | 14.2992 | 14.2414 | 14.2652 | 14.6299 | 14.1649 | 14.3809 | 14.3403 | 14.4003 | 9 | 8 | 8 | 9 | 9 | 9 | 9 | 9 |
| RNF113A | O15541 | 0.002196 | 0.000426 | -0.114546 | | 0.359657 | -0.84462 | | 15.0488 | 15.3105 | 14.9571 | 15.0743 | NaN | 15.0801 | 15.2964 | 15.4292 | 3 | 2 | 2 | 1 | NaN | 1 | 1 | 1 |
| ETF1 | P62495 | 6.54E-12 | 3.95E-12 | -0.114528 | | 0.53513 | -1.15593 | | 18.1649 | 18.3101 | 18.2979 | 18.5179 | 18.1193 | 18.4001 | 18.4766 | 18.3472 | 22 | 24 | 23 | 23 | 20 | 23 | 21 | 23 |
| SAP30 | O75446 | 0.002802 | 0.000527 | -0.114328 | | 0.132024 | -0.35388 | | 13.3987 | 13.2913 | 12.9115 | 12.597 | 13.7194 | NaN | 13.3379 | 13.605 | 2 | 3 | 1 | 1 | 2 | NaN | 3 | 2 |
| RPS14 | P62263 | 1.01E-20 | 9.09E-20 | -0.114291 | | 0.759443 | -1.54204 | | 20.9349 | 20.997 | 20.9604 | 21.0345 | 21.2895 | 21.0634 | 20.9746 | 21.03 | 9 | 9 | 9 | 9 | 9 | 9 | 9 | 9 |
| GCC1 | Q96CN9 | 0.001042 | 0.000214 | -0.114031 | | 0.725663 | -1.48502 | | 14.6204 | 14.5485 | 14.5459 | 14.5838 | 14.823 | 14.7973 | 14.6834 | 14.5406 | 8 | 10 | 10 | 8 | 7 | 10 | 11 | 9 |
| WDR13 | Q9H1Z4 | 9.01E-15 | 1.15E-14 | -0.113933 | | 0.389727 | -0.89019 | | 15.3972 | 15.437 | 15.4492 | 15.2624 | 15.8018 | 15.5479 | 15.6844 | 15.4122 | 10 | 10 | 9 | 9 | 7 | 12 | 8 | 9 |
| DCK | P27707 | 3.34E-06 | 9.19E-07 | -0.113716 | | 0.358522 | -0.83058 | | 16.5635 | 16.5203 | 16.4724 | 16.7607 | 16.7086 | 16.3198 | 16.4855 | 16.8877 | 7 | 7 | 6 | 10 | 5 | 5 | 6 | 9 |
| MED25 | Q71SY5;Q7 | 0.000109 | 2.57E-05 | -0.1137 | | 0.190133 | -0.48413 | | 15.3756 | 15.5286 | 15.3032 | 15.1499 | 15.9469 | 15.0785 | 15.6219 | 15.7836 | 1 | 1 | 1 | 1 | 1 | 1 | 1 | 1 |
| ERLIN1 | O75477 | 3.15E-16 | 6.18E-16 | -0.113623 | + | 1.52634 | -2.835 | | 17.5273 | 17.5311 | 17.5464 | 17.7114 | 17.6951 | 17.5765 | 17.5757 | 17.6841 | 11 | 11 | 11 | 11 | 11 | 11 | 11 | 11 |
| RMDN3 | Q96TC7 | 8.93E-11 | 4.33E-11 | -0.113423 | | 0.44029 | -0.98462 | | 16.4362 | 16.3665 | 16.3098 | 16.5744 | 16.5765 | 16.6131 | 16.156 | 16.5012 | 11 | 11 | 11 | 10 | 7 | 11 | 9 | 10 |
| SEC31A | O94979 | 7.27E-12 | 4.36E-12 | -0.113385 | + | 1.80059 | -3.32895 | | 18.5853 | 18.6259 | 18.6001 | 18.7581 | 18.6869 | 18.7894 | 18.6977 | 18.6538 | 45 | 45 | 45 | 43 | 43 | 44 | 43 | 44 |
| RPLP0 | P05388 | 2.75E-10 | 1.23E-10 | -0.113359 | + | 3.14455 | -6.34668 | | 20.7657 | 20.7516 | 20.713 | 20.8428 | 20.8926 | 20.8642 | 20.8337 | 20.8508 | 17 | 17 | 17 | 16 | 17 | 16 | 17 | 17 |
| TRAPPC13 | A5PLN9;A5 | 0.000126 | 2.95E-05 | -0.113315 | | 0.197661 | -0.50078 | | 18.9195 | 18.6642 | 18.8571 | 18.5963 | 19.5402 | 18.9265 | 18.8732 | 18.6983 | 6 | 8 | 7 | 5 | 2 | 4 | 6 | 6 |
| ACBD3 | Q9H3P7 | 7.12E-11 | 3.50E-11 | -0.113215 | + | 1.60086 | -2.96654 | | 17.2612 | 17.2843 | 17.2474 | 17.3098 | 17.41 | 17.3643 | 17.4677 | 17.3359 | 15 | 15 | 16 | 16 | 13 | 16 | 16 | 16 |
| TAGLN | Q01995 | 6.27E-08 | 2.07E-08 | -0.113126 | | 0.388866 | -0.88856 | | 21.4018 | 21.5042 | 21.4401 | 21.5403 | 21.4499 | 21.6619 | 21.3015 | 21.8555 | 28 | 28 | 28 | 26 | 26 | 28 | 26 | 28 |
| EIF4G3 | O43432;O4 | 7.26E-07 | 2.15E-07 | -0.113054 | + | 1.77579 | -3.28311 | | 16.2624 | 16.2375 | 16.2811 | 16.314 | 16.3837 | 16.4069 | 16.4424 | 16.3199 | 35 | 34 | 40 | 38 | 31 | 36 | 38 | 39 |
| CLPP | Q16740 | 3.08E-07 | 9.51E-08 | -0.112791 | | 0.203259 | -0.51307 | | 14.9958 | 14.6684 | 15.3306 | 15.3509 | 15.1199 | 14.8313 | 14.82 | 15.4332 | 8 | 7 | 7 | 8 | 6 | 5 | 8 | 7 |
| LTV1 | Q96GA3 | 1.26E-06 | 3.63E-07 | -0.112614 | | 0.54996 | -1.18216 | | 14.5518 | 14.4854 | 14.4529 | 14.5427 | 14.719 | 14.5946 | 14.3952 | 14.795 | 8 | 7 | 8 | 5 | 5 | 6 | 7 | 7 |
| LPIN3 | Q9BQK8;Q9 | 0.003665 | 0.000669 | -0.112593 | | 0.173137 | -0.44603 | | 15.112 | 15.1336 | 14.9802 | 15.5618 | 15.67 | 15.0196 | 14.6623 | 15.0257 | 5 | 7 | 6 | 7 | 4 | 6 | 3 | 3 |
| NFS1 | Q9Y697;Q9 | 1.65E-16 | 3.68E-16 | -0.112218 | | 0.997846 | -1.9396 | | 15.1614 | 15.1681 | 15.1417 | 15.244 | 15.4405 | 15.2319 | 15.211 | 15.2191 | 12 | 12 | 14 | 12 | 8 | 13 | 11 | 11 |
| MAP1B | P46821 | 2.76E-14 | 3.08E-14 | -0.112137 | | 0.274085 | -0.66293 | | 18.4374 | 18.4542 | 18.4741 | 18.9142 | 18.3129 | 18.5925 | 18.255 | 18.7623 | 99 | 99 | 99 | 98 | 91 | 98 | 93 | 100 |
| ZNF800 | Q2TB10 | 0.007965 | 0.001385 | -0.11196 | | 0.349764 | -0.81365 | | 14.6529 | 14.755 | 14.9956 | 15.1866 | 14.6405 | 14.8796 | 14.9422 | 14.9168 | 3 | 4 | 1 | 2 | 3 | 3 | 4 | 2 |
| CNN3 | Q15417 | 1.44E-08 | 5.15E-09 | -0.11168 | | 0.189492 | -0.48271 | | 19.8604 | 19.952 | 19.9568 | 20.265 | 19.862 | 20.5477 | 19.5403 | 19.9587 | 15 | 14 | 14 | 13 | 13 | 14 | 13 | 14 |
| DCTN6 | O00399 | 0.000179 | 4.09E-05 | -0.111542 | | 1.00021 | -1.94353 | | 16.6028 | 16.7439 | 16.6604 | 16.9095 | 16.6936 | 16.7361 | 16.7617 | 16.8019 | 5 | 5 | 5 | 5 | 5 | 5 | 5 | 5 |
| MGAT2 | Q10469 | 5.51E-12 | 3.36E-12 | -0.111047 | | 0.715352 | -1.46756 | | 16.0808 | 16.1787 | 16.0847 | 16.3873 | 16.1908 | 16.0637 | 16.2885 | 16.1986 | 12 | 14 | 13 | 14 | 12 | 12 | 14 | 13 |
| M6PR | P20645 | 4.04E-10 | 1.76E-10 | -0.110656 | | 0.302314 | -0.72013 | | 16.813 | 16.7053 | 16.6074 | 16.8954 | 16.7878 | 16.8915 | 17.0966 | 16.4249 | 8 | 8 | 8 | 8 | 7 | 8 | 9 | 8 |
| ACOT13 | Q9NPJ3;Q9 | 1.20E-15 | 2.01E-15 | -0.11059 | | 0.202191 | -0.51073 | | 17.2556 | 17.5734 | 17.5322 | 17.0707 | 17.6958 | 17.9192 | 17.769 | 17.3669 | 4 | 4 | 4 | 4 | 4 | 4 | 4 | 4 |
| GOLT1B | Q9Y3E0 | 1.74E-05 | 4.45E-06 | -0.110444 | + | 1.45007 | -2.70212 | | 16.8272 | 16.8216 | 16.8024 | 16.8959 | 16.9346 | 17.0153 | 16.9581 | 16.8339 | 4 | 4 | 4 | 4 | 4 | 4 | 4 | 4 |
| PLEC | Q15149 | 3.81E-16 | 7.27E-16 | -0.110397 | | 0.385041 | -0.88131 | | 19.8651 | 19.8824 | 19.8535 | 19.8275 | 20.1323 | 19.9603 | 19.7292 | 20.2377 | 336 | 334 | 334 | 329 | 334 | 333 | 327 | 336 |
| IFRD1 | O00458;O0 | 0.003775 | 0.000689 | -0.11039 | | 0.050853 | -0.15723 | | 13.7031 | NaN | 13.2926 | NaN | NaN | 14.2797 | 12.9368 | NaN | 1 | NaN | 2 | NaN | NaN | 2 | 1 | NaN |
| PDCD6IP | Q8WUM4 | 1.09E-11 | 6.31E-12 | -0.110337 | + | 2.34569 | -4.41163 | | 18.6054 | 18.6193 | 18.5762 | 18.7632 | 18.7046 | 18.6952 | 18.7307 | 18.6593 | 55 | 55 | 56 | 55 | 56 | 53 | 55 | 53 |
| CYRIB | Q9NUQ9 | 2.87E-14 | 3.19E-14 | -0.110251 | | 0.656851 | -1.36787 | | 16.8228 | 16.7755 | 16.7835 | 16.9144 | 17.13 | 16.8565 | 16.8285 | 16.7915 | 9 | 10 | 9 | 8 | 10 | 9 | 9 | 10 |
| LCMT2 | O60294 | 1.19E-10 | 5.67E-11 | -0.110217 | | 0.245721 | -0.60409 | | 14.7567 | 15.0471 | 15.0279 | 14.9874 | 14.8545 | 15.2849 | 15.4062 | 14.7375 | 4 | 4 | 4 | 3 | 3 | 3 | 4 | 3 |
| CTPS2 | Q9NRF8 | 2.06E-12 | 1.37E-12 | -0.110008 | | 0.292016 | -0.69941 | | 15.8956 | 15.9114 | 15.9463 | 16.3805 | 16.2168 | 15.7414 | 15.8668 | 15.9334 | 14 | 17 | 14 | 16 | 12 | 13 | 14 | 12 |
| ABCD1 | P33897 | 3.92E-15 | 5.63E-15 | -0.109938 | | 0.78802 | -1.59007 | | 15.6713 | 15.655 | 15.607 | 15.7027 | 15.8316 | 15.7568 | 15.8862 | 15.5946 | 9 | 11 | 9 | 9 | 7 | 11 | 10 | 8 |
| AKT1 | P31749 | 1.90E-16 | 4.11E-16 | -0.10987 | | 0.90108 | -1.77883 | | 15.6017 | 15.616 | 15.6188 | 15.835 | 15.7747 | 15.7543 | 15.5656 | 15.6806 | 21 | 20 | 21 | 21 | 20 | 21 | 19 | 19 |
| ELP5 | Q8TE02;Q8 | 5.76E-08 | 1.91E-08 | -0.109719 | | 0.196077 | -0.49729 | | 16.1484 | 15.6487 | 16.231 | 15.6194 | 16.3136 | 16.1235 | 16.3598 | 16.1792 | 1 | 2 | 1 | 1 | 1 | 1 | 2 | 1 |
| RPL31 | P62899;P6 | 0.00059 | 0.000125 | -0.109471 | | 1.12223 | -2.14651 | | 20.8585 | 21.014 | 20.9488 | 21.1182 | 20.974 | 21.0691 | 21.0998 | 20.9883 | 7 | 7 | 7 | 7 | 7 | 7 | 7 | 7 |
| PSMB6 | P28072 | 6.59E-08 | 2.17E-08 | -0.109404 | | 0.62504 | -1.31315 | | 19.0136 | 18.9595 | 19.0211 | 18.9239 | 19.2786 | 19.0536 | 19.2029 | 19.0785 | 8 | 8 | 8 | 8 | 8 | 8 | 8 | 8 |
| ZC2HC1A | Q96GY0 | 1.99E-11 | 1.10E-11 | -0.109379 | | 0.295649 | -0.70674 | | 16.378 | 16.3886 | 16.4623 | 16.8307 | 16.6441 | 16.1397 | 16.5491 | 16.4313 | 8 | 7 | 6 | 6 | 6 | 7 | 6 | 6 |
| CAV1 | Q03135 | 1.90E-10 | 8.76E-11 | -0.109346 | | 0.227185 | -0.56482 | | 20.8901 | 20.9565 | 21.0331 | 20.6183 | 21.4063 | 21.3391 | 20.9225 | 21.06 | 4 | 3 | 4 | 3 | 4 | 4 | 3 | 4 |
| EIF1AX | P47813 | 3.07E-07 | 9.50E-08 | -0.109305 | + | 1.56355 | -2.90047 | | 18.6117 | 18.5691 | 18.5661 | 18.672 | 18.6423 | 18.6986 | 18.6523 | 18.7929 | 8 | 7 | 8 | 7 | 8 | 8 | 7 | 8 |
| MFGE8 | Q08431 | 3.82E-11 | 1.98E-11 | -0.109295 | | 0.054592 | -0.15504 | | 17.0009 | 16.9991 | 17.0302 | 15.1728 | 17.2582 | 17.5252 | 18.3837 | 17.2568 | 11 | 12 | 11 | 10 | 11 | 11 | 13 | 12 |
| TMF1 | P82094;P8 | 2.56E-24 | 7.58E-23 | -0.109058 | | 0.756294 | -1.53673 | | 14.9623 | 14.9352 | 14.964 | 15.0967 | 14.8879 | 15.0072 | 15.1921 | 15.1306 | 26 | 27 | 27 | 26 | 19 | 24 | 27 | 25 |
| SPAST | Q9UBP0;Q9 | 4.33E-11 | 2.22E-11 | -0.109052 | | 0.653919 | -1.36284 | | 15.291 | 15.2618 | 15.1709 | 15.1889 | 15.5338 | 15.3147 | 15.3965 | 15.3176 | 5 | 5 | 4 | 4 | 2 | 5 | 5 | 5 |
| MRPL20 | Q9BYC9 | 2.53E-07 | 7.89E-08 | -0.108917 | | 0.814664 | -1.63471 | | 15.8097 | 15.9213 | 15.9146 | 16.0692 | 15.8885 | 15.8892 | 15.9931 | 16.114 | 5 | 5 | 5 | 5 | 5 | 4 | 5 | 5 |
| CAMSAP1 | Q5T5Y3;Q5 | 0.000547 | 0.000117 | -0.108864 | | 0.592099 | -1.25602 | | 14.5057 | 14.5782 | 14.4977 | 14.6382 | 14.6204 | 14.6513 | 14.4351 | 14.8353 | 7 | 8 | 8 | 5 | 6 | 6 | 5 | 7 |
| VPS53 | Q5VIR6-4 | 8.07E-16 | 1.41E-15 | -0.108821 | + | 1.60251 | -2.96949 | | 15.8323 | 15.8259 | 15.7366 | 15.9032 | 15.9248 | 15.8353 | 15.9694 | 15.9026 | 20 | 20 | 19 | 19 | 20 | 19 | 21 | 19 |
| NEMF | O60524;O6 | 1.45E-17 | 4.39E-17 | -0.108713 | + | 1.48987 | -2.77124 | | 15.8839 | 15.9366 | 15.8823 | 16.0128 | 15.962 | 15.9838 | 16.1155 | 15.9742 | 21 | 19 | 21 | 16 | 14 | 21 | 21 | 20 |

| Gene names | UniProt IDs | Protein Group P-value | Protein Group Q-value | Log2 Difference NUP214 vs Controls (n=5) | p<0.05 | -log10 p-value | T-test Test statistic | NPC Localization | Log2 MS2_quantity_NUP214_1 | Log2 MS2_quantity_NUP214_2 | Log2 MS2_quantity_NUP214_3 | Log2 MS2_quantity_Control_1 | Log2 MS2_quantity_Control_2 | Log2 MS2_quantity_Control_3 | Log2 MS2_quantity_Control_4 | Log2 MS2_quantity_Control_5 | Number Peptides for Quantitation NUP214_1 | Number Peptides for Quantitation NUP214_2 | Number Peptides for Quantitation NUP214_3 | Number Peptides for Quantitation Control_1 | Number Peptides for Quantitation Control_2 | Number Peptides for Quantitation Control_3 | Number Peptides for Quantitation Control_4 | Number Peptides for Quantitation Control_5 |
|---|---|---|---|---|---|---|---|---|---|---|---|---|---|---|---|---|---|---|---|---|---|---|---|---|
| PIGG | Q5H8A4;Q5 | 8.19E-07 | 2.41E-07 | -0.108683 | | 0.334738 | -0.78436 | | 16.0323 | 15.9573 | 15.9449 | 16.0358 | 16.1702 | 15.8223 | 16.4321 | 15.974 | 8 | 10 | 10 | 9 | 8 | 9 | 10 | 9 |
| NAV1 | Q8NEY1;Q8 | 2.31E-08 | 8.02E-09 | -0.108386 | | 0.1758 | -0.45205 | | 15.0943 | 15.0975 | 15.1407 | 15.8587 | 15.0105 | 15.0798 | 14.8176 | 15.3293 | 32 | 33 | 34 | 40 | 13 | 25 | 25 | 30 |
| ARFIP1 | P53367-2 | 6.37E-12 | 3.85E-12 | -0.108053 | + | 2.28776 | -4.28888 | | 16.2847 | 16.2557 | 16.2644 | 16.3985 | 16.414 | 16.3973 | 16.3589 | 16.3128 | 13 | 17 | 17 | 13 | 16 | 16 | 16 | 16 |
| MSI2 | Q96DH6;Q | 2.54E-10 | 1.15E-10 | -0.108 | | 0.172941 | -0.44559 | | 15.3096 | 15.4388 | 15.356 | 15.9596 | 15.5999 | 15.29 | 15.6396 | 14.8914 | 5 | 6 | 6 | 6 | 2 | 4 | 6 | 2 |
| NAA10 | P41227 | 2.68E-08 | 9.24E-09 | -0.107932 | | 0.500233 | -1.09365 | | 16.332 | 16.3827 | 16.3648 | 16.7128 | 16.2846 | 16.3819 | 16.4207 | 16.5388 | 14 | 14 | 15 | 14 | 12 | 14 | 14 | 15 |
| SORD | Q00796 | 1.25E-06 | 3.59E-07 | -0.107901 | | 0.253604 | -0.62059 | | 17.324 | 17.3803 | 17.4259 | 17.1376 | 17.9042 | 17.402 | 17.3641 | 17.6152 | 13 | 13 | 12 | 12 | 12 | 12 | 12 | 12 |
| ALG5 | Q9Y673 | 8.03E-08 | 2.61E-08 | -0.107796 | | 0.192821 | -0.49009 | | 15.9552 | 15.9767 | 15.9129 | 16.0775 | 15.586 | 16.3895 | 16.4322 | 15.7952 | 8 | 9 | 11 | 11 | 8 | 11 | 11 | 6 |
| PTPN9 | P43378 | 6.71E-13 | 5.05E-13 | -0.107683 | + | 1.78522 | -3.3005 | | 15.4514 | 15.4591 | 15.3977 | 15.477 | 15.6049 | 15.5568 | 15.566 | 15.514 | 13 | 13 | 13 | 13 | 13 | 15 | 13 | 13 |
| CCT6A | P40227 | 3.49E-13 | 2.86E-13 | -0.107494 | | 1.21605 | -2.30352 | | 19.1222 | 19.2354 | 19.13 | 19.3331 | 19.326 | 19.2277 | 19.1833 | 19.2801 | 30 | 30 | 31 | 30 | 28 | 30 | 32 | 29 |
| USP15 | Q9Y4E8;Q9 | 1.61E-19 | 9.26E-19 | -0.107311 | + | 1.39943 | -2.61479 | | 15.846 | 15.874 | 15.8371 | 16.0624 | 15.8733 | 15.9607 | 15.9552 | 15.9468 | 29 | 31 | 30 | 32 | 24 | 27 | 27 | 26 |
| UFSP2 | Q9NUQ7 | 3.64E-09 | 1.40E-09 | -0.106806 | | 0.340888 | -0.79638 | | 15.6221 | 15.6562 | 15.5551 | 15.3824 | 15.6761 | 15.869 | 15.962 | 15.7002 | 15 | 16 | 15 | 14 | 10 | 15 | 15 | 13 |
| LSM14A | Q8ND56;Q | 3.70E-09 | 1.42E-09 | -0.106778 | + | 1.5039 | -2.79572 | | 15.0309 | 15.1206 | 15.0857 | 15.1654 | 15.2127 | 15.2135 | 15.2389 | 15.0987 | 8 | 8 | 9 | 8 | 8 | 9 | 9 | 9 |
| MAN1B1 | Q9UKM7 | 2.94E-09 | 1.14E-09 | -0.106627 | | 0.318397 | -0.75218 | | 16.0702 | 16.1206 | 15.9806 | 15.9402 | 16.0778 | 16.1562 | 16.555 | 16.0896 | 13 | 13 | 13 | 13 | 12 | 13 | 12 | 12 |
| OXSM | Q9NWU1 | 0.001984 | 0.000389 | -0.106572 | | 0.299769 | -0.71503 | | 14.1168 | 14.4827 | 14.2611 | 14.2658 | 14.6116 | 14.6321 | 14.1699 | 14.2878 | 3 | 3 | 3 | 2 | 2 | 3 | 3 | 3 |
| PSMD11 | O00231 | 5.87E-17 | 1.48E-16 | -0.106452 | + | 1.42969 | -2.6669 | | 18.1005 | 18.1433 | 18.1291 | 18.2375 | 18.2934 | 18.1408 | 18.2895 | 18.1925 | 28 | 28 | 27 | 28 | 27 | 28 | 28 | 28 |
| RPL23A | P62750 | 6.95E-18 | 2.35E-17 | -0.10618 | | 0.985701 | -1.91943 | | 19.4733 | 19.5689 | 19.5545 | 19.6687 | 19.7707 | 19.5649 | 19.6197 | 19.5681 | 13 | 13 | 13 | 12 | 13 | 13 | 13 | 13 |
| DNAJC11 | Q9NVH1;Q | 2.94E-07 | 9.11E-08 | -0.106104 | | 1.01962 | -1.97577 | | 16.3443 | 16.3163 | 16.3281 | 16.4837 | 16.3192 | 16.5462 | 16.454 | 16.3754 | 14 | 14 | 14 | 15 | 12 | 13 | 11 | 13 |
| SREK1 | Q8WXA9-2 | 3.20E-05 | 7.99E-06 | -0.106052 | | 0.559768 | -1.19944 | | 16.4292 | 16.5267 | 16.5957 | 16.4259 | 16.7575 | 16.5608 | 16.7372 | 16.6348 | 6 | 5 | 5 | 5 | 6 | 5 | 5 | 4 |
| LRRC14 | Q15048 | 0.00155 | 0.000311 | -0.106026 | | 0.399817 | -0.90924 | | 15.1916 | 15.1895 | 15.3249 | 15.1954 | 15.651 | 15.24 | 15.2343 | 15.3861 | 5 | 5 | 5 | 6 | 4 | 5 | 3 | 4 |
| CHSY1 | Q86X52 | 0.001868 | 0.000369 | -0.105957 | | 0.24868 | -0.61029 | | 14.9053 | 15.2061 | 15.1641 | 15.3878 | 15.4835 | 15.2157 | 15.1031 | 14.799 | 2 | 1 | 2 | 2 | 3 | 2 | 2 | 2 |
| EVI5L | Q96CN4;Q9 | 7.48E-10 | 3.14E-10 | -0.105703 | | 0.632188 | -1.32548 | | 14.0695 | 13.9726 | 14.0879 | 14.3049 | 14.2557 | 14.0352 | 14.0289 | 14.1206 | 5 | 5 | 5 | 5 | 6 | 3 | 3 | 3 |
| MT2A | P02795 | 0.000785 | 0.000164 | -0.105685 | | 0.297404 | -0.71027 | | 19.956 | 20.0136 | 20.1697 | 19.7789 | 20.4293 | 20.2011 | 20.2256 | 20.1258 | 2 | 2 | 3 | 2 | 2 | 2 | 2 | 2 |
| LIPA | P38571;P3 | 0.00078 | 0.000163 | -0.105662 | | 0.201441 | -0.50908 | | 17.0962 | 17.1484 | 17.1382 | 17.6691 | 16.7882 | 17.0042 | 17.4368 | 17.268 | 5 | 6 | 5 | 5 | 5 | 5 | 5 | 5 |
| SLC16A3 | O15427 | 8.57E-10 | 3.58E-10 | -0.105596 | | 0.230845 | -0.57262 | | 17.6845 | 17.5685 | 17.7269 | 17.7033 | 18.0063 | 17.2976 | 18.0626 | 17.7579 | 10 | 10 | 10 | 10 | 10 | 10 | 10 | 10 |
| OPTN | Q96CV9 | 8.28E-18 | 2.73E-17 | -0.105553 | | 0.282503 | -0.68012 | | 16.5197 | 16.4765 | 16.5021 | 17.0029 | 16.6614 | 16.6032 | 16.3235 | 16.4339 | 24 | 24 | 24 | 25 | 23 | 25 | 25 | 23 |
| STRADA | Q7RTN6;Q7 | 0.001851 | 0.000366 | -0.105467 | | 0.201148 | -0.51379 | | 14.066 | 13.9484 | 13.7303 | 13.6402 | 14.3288 | 14.2308 | NaN | 13.8818 | 3 | 2 | 1 | 3 | 2 | 1 | NaN | 1 |
| DENND5A | Q6IQ26 | 0.027462 | 0.004526 | -0.105174 | | 0.089098 | -0.25597 | | NaN | 13.7401 | 13.7243 | 13.8494 | NaN | 13.2802 | NaN | 14.3824 | NaN | 1 | 1 | 3 | NaN | 2 | NaN | 1 |
| MAGI1 | Q96QZ7-2; | 2.47E-11 | 1.34E-11 | -0.105047 | | 0.183628 | -0.46964 | | 14.8539 | 14.9539 | 14.9585 | 15.4224 | 15.0156 | 15.0949 | 14.4187 | 15.1843 | 9 | 8 | 10 | 13 | 6 | 11 | 4 | 7 |
| RPL7A | P62424 | 1.02E-06 | 2.95E-07 | -0.105046 | | 1.09968 | -2.10891 | | 20.2259 | 20.2531 | 20.2409 | 20.3657 | 20.4814 | 20.3063 | 20.2889 | 20.2828 | 20 | 20 | 20 | 20 | 20 | 20 | 20 | 20 |
| DENND10 | Q8TCE6;Q8 | 1.07E-05 | 2.79E-06 | -0.104974 | | 1.02284 | -1.98111 | | 16.0456 | 16.058 | 16.0422 | 16.1631 | 16.1724 | 16.2772 | 16.1234 | 16.0318 | 11 | 12 | 13 | 11 | 9 | 10 | 11 | 7 |
| CAST | P20810-6 | 3.26E-12 | 2.08E-12 | -0.104778 | | 0.225363 | -0.56092 | | 17.532 | 17.5774 | 17.5851 | 18.1703 | 17.3859 | 17.7625 | 17.4753 | 17.5541 | 42 | 41 | 40 | 43 | 39 | 39 | 36 | 40 |
| MYO10 | Q9HD67 | 2.84E-11 | 1.51E-11 | -0.10463 | | 0.113058 | -0.30482 | | 14.5956 | 14.5709 | 14.7675 | 15.4635 | 15.1717 | 14.0302 | 14.61 | 14.4711 | 10 | 10 | 8 | 11 | 4 | 2 | 7 | 5 |
| TUBB2B | Q9BVA1 | 1.00E-07 | 3.23E-08 | -0.1046 | | 1.19284 | -2.26457 | | 21.9842 | 22.0998 | 22.0462 | 22.1424 | 22.2543 | 22.0876 | 22.1552 | 22.1004 | 8 | 8 | 8 | 8 | 8 | 8 | 8 | 8 |
| RPIA | P49247 | 1.52E-06 | 4.33E-07 | -0.104206 | | 0.200039 | -0.50601 | | 15.2975 | 15.4399 | 15.3459 | 15.856 | 15.4773 | 15.5513 | 14.9152 | 15.5269 | 4 | 5 | 4 | 5 | 3 | 3 | 4 | 5 |
| MANF | P55145 | 1.23E-09 | 5.04E-10 | -0.104163 | | 0.380113 | -0.87195 | | 17.5631 | 17.576 | 17.5633 | 17.872 | 17.7586 | 17.3378 | 17.7039 | 17.6859 | 17 | 17 | 17 | 17 | 16 | 16 | 17 | 17 |
| FTSJ1 | Q9UET6 | 3.52E-09 | 1.36E-09 | -0.104078 | | 1.19356 | -2.26577 | | 15.5132 | 15.5059 | 15.5881 | 15.6432 | 15.5363 | 15.707 | 15.6999 | 15.6124 | 5 | 5 | 5 | 5 | 3 | 5 | 4 | 6 |
| MTDH | Q86UE4 | 1.39E-12 | 9.66E-13 | -0.103866 | + | 1.32853 | -2.49361 | | 17.4316 | 17.4796 | 17.429 | 17.6151 | 17.468 | 17.5504 | 17.6178 | 17.5016 | 24 | 23 | 23 | 23 | 22 | 23 | 23 | 22 |
| SEC61G | P60059 | 0.002552 | 0.000486 | -0.103844 | | 0.201008 | -0.50813 | | 17.9616 | 17.5365 | 17.9242 | 18.0066 | 17.4911 | 17.7332 | 18.0756 | 18.2499 | 2 | 2 | 2 | 2 | 2 | 2 | 2 | 2 |
| CHM | P24386 | 2.28E-05 | 5.78E-06 | -0.103616 | | 0.807628 | -1.62293 | | 16.1661 | 16.2087 | 16.1354 | 16.2065 | 16.4337 | 16.2939 | 16.1613 | 16.2731 | 7 | 8 | 8 | 7 | 3 | 4 | 9 | 6 |
| NOP2 | P46087;P4 | 1.20E-12 | 8.53E-13 | -0.103608 | + | 1.35067 | -2.53133 | | 16.3251 | 16.3188 | 16.3299 | 16.4623 | 16.4205 | 16.3133 | 16.4578 | 16.4872 | 22 | 22 | 22 | 23 | 21 | 21 | 22 | 23 |
| JMY | Q8N9B5;Q8 | 0.000346 | 7.62E-05 | -0.103602 | | 0.177221 | -0.45526 | | 15.3394 | 15.2512 | 15.4688 | 15.0119 | 15.6774 | 15.7163 | 15.7885 | 15.0897 | 3 | 4 | 4 | 2 | 2 | 4 | 4 | 3 |
| AKT2 | P31751 | 1.22E-13 | 1.13E-13 | -0.103101 | | 0.615883 | -1.29732 | | 15.7498 | 15.7045 | 15.7051 | 15.8876 | 15.8535 | 15.7499 | 15.6401 | 15.9836 | 13 | 13 | 13 | 13 | 12 | 13 | 14 | 13 |
| SLC25A19 | Q9HC21;Q9 | 0.001846 | 0.000365 | -0.103066 | | 0.214765 | -0.53811 | | 15.0182 | 14.8168 | 14.7077 | 14.4364 | 15.1722 | 15.1664 | 14.9857 | 14.9925 | 3 | 3 | 4 | 2 | 2 | 3 | 4 | 3 |
| RMDN2 | Q96LZ7 | 2.42E-05 | 6.12E-06 | -0.102613 | | 0.382897 | -0.87724 | | 14.4778 | 14.5394 | 14.6731 | 14.49 | 14.5994 | 14.6722 | 14.9721 | 14.5965 | 4 | 6 | 5 | 2 | 2 | 5 | 4 | 4 |
| DDX59 | Q5T1V6 | 0.005539 | 0.000981 | -0.102575 | | 0.080136 | -0.22707 | | NaN | 12.5654 | 12.4112 | 11.7364 | 13.1375 | NaN | 12.7351 | 12.7544 | NaN | 1 | 2 | 2 | 1 | NaN | 1 | 1 |
| KRR1 | Q13601 | 1.57E-05 | 4.03E-06 | -0.102124 | | 0.405002 | -0.91898 | | 15.6008 | 15.6536 | 15.6743 | 16.0027 | 15.7225 | 15.5214 | 15.837 | 15.6416 | 9 | 9 | 10 | 9 | 8 | 9 | 8 | 7 |
| INF2 | Q27J81 | 2.68E-15 | 4.05E-15 | -0.102007 | | 0.265099 | -0.64444 | | 17.6273 | 17.6163 | 17.6158 | 17.8437 | 17.9486 | 17.3769 | 17.5003 | 17.9396 | 45 | 47 | 46 | 42 | 44 | 41 | 43 | 47 |
| COMMD1 | Q8N668 | 0.003592 | 0.000657 | -0.10186 | | 0.223634 | -0.55721 | | 15.449 | 15.3769 | 15.7501 | 15.731 | 15.728 | 15.1573 | 15.6582 | 15.8614 | 6 | 6 | 6 | 6 | 6 | 4 | 6 | 6 |
| TIMM50 | Q3ZCQ8;Q3 | 3.98E-07 | 1.21E-07 | -0.101854 | | 0.791564 | -1.59601 | | 16.9216 | 16.9338 | 16.909 | 17.0229 | 17.0189 | 16.8516 | 17.0977 | 17.1256 | 7 | 8 | 8 | 8 | 8 | 8 | 9 | 8 |
| DOK1 | Q99704 | 4.38E-12 | 2.72E-12 | -0.101819 | | 0.246838 | -0.60643 | | 15.771 | 15.8962 | 15.9345 | 16.1113 | 15.6232 | 16.3449 | 15.9402 | 15.8255 | 9 | 9 | 12 | 10 | 6 | 9 | 9 | 9 |
| TM9SF4 | Q92544 | 6.70E-11 | 3.32E-11 | -0.101768 | | 0.801683 | -1.61298 | | 17.338 | 17.3453 | 17.2852 | 17.4392 | 17.5196 | 17.3201 | 17.528 | 17.3161 | 15 | 15 | 14 | 15 | 13 | 15 | 15 | 13 |
| VWA8 | A3KMH1;A | 1.14E-13 | 1.07E-13 | -0.101582 | | 0.145229 | -0.3818 | | 14.5143 | 14.4669 | 14.499 | 15.2835 | 14.139 | 14.5662 | 14.7071 | 14.279 | 15 | 18 | 16 | 21 | 8 | 16 | 24 | 12 |
| COPB2 | P35606 | 9.19E-10 | 3.82E-10 | -0.101551 | | 0.493344 | -1.08126 | | 18.8944 | 18.949 | 18.8916 | 19.1614 | 18.7641 | 19.1118 | 19.0558 | 18.9731 | 50 | 50 | 50 | 49 | 48 | 50 | 48 | 50 |
| CHORDC1 | Q9UHD1 | 6.33E-11 | 3.15E-11 | -0.101508 | | 0.474618 | -1.04741 | | 16.5928 | 16.5972 | 16.58 | 16.9082 | 16.6318 | 16.5355 | 16.5673 | 16.8148 | 18 | 18 | 19 | 18 | 17 | 17 | 18 | 17 |
| RPL22 | P35268 | 1.59E-07 | 5.06E-08 | -0.101386 | | 0.167517 | -0.43327 | | 21.0497 | 20.7456 | 20.727 | 20.6208 | 21.5216 | 20.9238 | 20.6196 | 21.0251 | 7 | 7 | 7 | 7 | 7 | 7 | 7 | 7 |
| PIK3CA | P42336 | 3.84E-12 | 2.42E-12 | -0.101315 | + | 1.33864 | -2.51083 | | 14.4107 | 14.3879 | 14.4747 | 14.4386 | 14.6052 | 14.5372 | 14.5134 | 14.5343 | 10 | 11 | 12 | 11 | 8 | 9 | 8 | 8 |
| RHEB | Q15382 | 6.92E-12 | 4.16E-12 | -0.101232 | | 0.275743 | -0.66632 | | 17.7701 | 17.7871 | 17.6813 | 18.234 | 17.5423 | 17.8214 | 17.8845 | 17.7549 | 8 | 8 | 8 | 9 | 9 | 8 | 9 | 9 |
| CEBPZ | Q03701 | 3.67E-13 | 2.99E-13 | -0.101146 | | 0.714552 | -1.46621 | | 14.9641 | 14.9803 | 15.0381 | 15.0918 | 14.9514 | 15.09 | 15.0766 | 15.2668 | 14 | 15 | 16 | 16 | 9 | 11 | 13 | 14 |
| SLC9A3R1 | O14745 | 3.69E-08 | 1.25E-08 | -0.101127 | | 0.145227 | -0.3818 | | 16.2214 | 16.1223 | 16.1263 | 16.7471 | 16.5895 | 15.8517 | 15.7482 | 16.3525 | 13 | 15 | 13 | 17 | 11 | 12 | 12 | 13 |
| AP2A1 | O95782;O9 | 3.45E-14 | 3.74E-14 | -0.101108 | + | 1.72705 | -3.19372 | | 18.7004 | 18.7082 | 18.7224 | 18.8914 | 18.7871 | 18.8359 | 18.7823 | 18.7605 | 34 | 33 | 34 | 34 | 33 | 34 | 33 | 33 |
| PRKAG1 | P54619;P5 | 8.43E-13 | 6.20E-13 | -0.101069 | | 0.333941 | -0.7828 | | 16.9622 | 16.959 | 16.9947 | 16.7509 | 16.9662 | 17.2762 | 17.2352 | 17.1366 | 15 | 16 | 15 | 15 | 16 | 15 | 16 | 15 |
| KIF5B | P33176 | 1.16E-16 | 2.70E-16 | -0.10106 | | 1.28617 | -2.42175 | | 18.0783 | 18.0239 | 18.0336 | 18.104 | 18.1277 | 18.1227 | 18.2649 | 18.1124 | 60 | 61 | 59 | 56 | 59 | 57 | 59 | 57 |
| ANAPC2 | Q9UJX6;Q9 | 0.000504 | 0.000108 | -0.101002 | | 0.292006 | -0.69939 | | 14.7396 | 14.7409 | 14.7833 | 14.669 | 14.9258 | 14.8887 | 15.2047 | 14.5897 | 6 | 8 | 8 | 6 | 6 | 6 | 8 | 6 |
| MAP4K3 | Q8IVH8;Q8 | 0.015405 | 0.00259 | -0.100969 | | 0.085635 | -0.24672 | | NaN | 14.0763 | 13.7955 | 14.253 | NaN | 14.4255 | NaN | 13.4321 | NaN | 4 | 1 | 4 | NaN | 3 | NaN | 2 |
| PCNT | O95613;O9 | 0.00056 | 0.00012 | -0.100862 | | 0.2779 | -0.67073 | | 13.8844 | 13.9456 | 13.8544 | 13.8147 | 13.9148 | 13.7294 | 14.245 | 14.2744 | 6 | 9 | 7 | 4 | 6 | 6 | 7 | 8 |
| IGF2BP2 | Q9Y6M1;Q | 2.10E-14 | 2.42E-14 | -0.100806 | | 0.936675 | -1.838 | | 17.791 | 17.7473 | 17.7922 | 17.9322 | 17.9753 | 17.9173 | 17.7848 | 17.7785 | 20 | 20 | 21 | 19 | 21 | 20 | 19 | 20 |
| MORC3 | Q14149 | 2.32E-11 | 1.26E-11 | -0.100585 | | 0.372839 | -0.85807 | | 14.64 | 14.5521 | 14.611 | 14.7603 | 14.6733 | 14.4211 | 14.6921 | 14.9615 | 7 | 8 | 7 | 10 | 7 | 7 | 8 | 9 |
| PRKD1 | Q15139 | 1.62E-12 | 1.11E-12 | -0.100462 | + | 1.72987 | -3.19887 | | 15.3465 | 15.3722 | 15.3289 | 15.3631 | 15.4735 | 15.4764 | 15.4867 | 15.4486 | 17 | 18 | 17 | 17 | 13 | 16 | 16 | 16 |
| ANKRD17 | O75179;O7 | 1.46E-16 | 3.29E-16 | -0.100384 | + | 1.37795 | -2.57796 | | 15.4541 | 15.4788 | 15.5637 | 15.6871 | 15.5982 | 15.5626 | 15.5821 | 15.5663 | 24 | 27 | 26 | 24 | 19 | 27 | 24 | 25 |
| AFF4 | Q9UHB7 | 9.60E-06 | 2.53E-06 | -0.10036 | | 0.249946 | -0.61294 | | 15.1027 | 15.1119 | 14.728 | 14.9042 | 15.4646 | 14.9855 | 14.9481 | 15.1037 | 2 | 3 | 3 | 1 | 2 | 2 | 1 | 1 |
| RABL6 | Q3YEC7 | 2.51E-11 | 1.35E-11 | -0.100326 | | 0.517222 | -1.12407 | | 16.3095 | 16.2206 | 16.2639 | 16.4887 | 16.1839 | 16.4442 | 16.4787 | 16.2295 | 8 | 7 | 7 | 8 | 4 | 7 | 9 | 7 |
| ISY1 | Q9ULR0;Q9 | 1.03E-06 | 3.00E-07 | -0.100062 | | 0.211613 | -0.53128 | | 15.7045 | 15.6098 | 15.6118 | 15.6819 | 16.0986 | 15.271 | 15.7169 | 15.9419 | 3 | 5 | 5 | 4 | 3 | 3 | 4 | 5 |
| PUM3 | Q15397 | 2.69E-16 | 5.43E-16 | -0.100055 | | 0.981628 | -1.91267 | | 15.5969 | 15.636 | 15.6042 | 15.7351 | 15.563 | 15.7798 | 15.7617 | 15.7226 | 21 | 22 | 21 | 21 | 17 | 22 | 21 | 22 |
| RPS7 | P62081 | 0.000236 | 5.32E-05 | -0.0999173 | | 0.829079 | -1.65881 | | 21.1145 | 21.1284 | 21.0143 | 21.1276 | 21.335 | 21.1474 | 21.1117 | 21.2067 | 12 | 12 | 11 | 11 | 11 | 11 | 12 | 12 |
| SLC39A10 | Q9ULF5 | 0.00288 | 0.00054 | -0.0999012 | | 0.133613 | -0.36272 | | 13.3928 | 13.748 | 13.8536 | 14.0049 | NaN | 13.2896 | 13.9996 | NaN | 2 | 2 | 2 | 1 | NaN | 1 | 2 | NaN |
| EMC8 | O43402 | 7.22E-14 | 7.16E-14 | -0.0997853 | | 0.677904 | -1.40388 | | 15.9649 | 15.979 | 15.8517 | 15.939 | 16.0766 | 16.2018 | 15.9644 | 15.9765 | 8 | 8 | 8 | 8 | 6 | 9 | 7 | 8 |
| PPME1 | Q9Y570;Q9 | 2.07E-16 | 4.40E-16 | -0.0997744 | | 0.281445 | -0.67797 | | 17.1918 | 17.163 | 17.2095 | 17.2379 | 16.9523 | 17.6159 | 17.2211 | 17.412 | 14 | 14 | 13 | 13 | 10 | 13 | 13 | 14 |
| UEVLD | Q8IX04 | 6.22E-13 | 4.73E-13 | -0.0996982 | | 0.513161 | -1.11682 | | 15.3495 | 15.3382 | 15.3166 | 15.6097 | 15.5696 | 15.2847 | 15.3052 | 15.4033 | 11 | 12 | 12 | 12 | 12 | 12 | 12 | 13 |

| Gene names | UniProt IDs | Protein Group P-value | Protein Group Q-value | Log2 Difference NUP214 vs Controls (n=5) | p<0.05 | -log10 p-value | T-test Test statistic | NPC Localization | Log2 MS2_quantity_NUP214_1 | Log2 MS2_quantity_NUP214_2 | Log2 MS2_quantity_NUP214_3 | Log2 MS2_quantity_Control_1 | Log2 MS2_quantity_Control_2 | Log2 MS2_quantity_Control_3 | Log2 MS2_quantity_Control_4 | Log2 MS2_quantity_Control_5 | Number Peptides for Quantitation NUP214_1 | Number Peptides for Quantitation NUP214_2 | Number Peptides for Quantitation NUP214_3 | Number Peptides for Quantitation Control_1 | Number Peptides for Quantitation Control_2 | Number Peptides for Quantitation Control_3 | Number Peptides for Quantitation Control_4 | Number Peptides for Quantitation Control_5 |
|---|---|---|---|---|---|---|---|---|---|---|---|---|---|---|---|---|---|---|---|---|---|---|---|---|
| EXOC8 | Q8IYI6 | 1.39E-06 | 3.96E-07 | -0.0995708 | + | 1.41285 | -2.63788 | | 15.3655 | 15.3267 | 15.2708 | 15.4641 | 15.3365 | 15.4686 | 15.4071 | 15.4267 | 21 | 19 | 19 | 18 | 12 | 20 | 17 | 18 |
| STK25 | O00506 | 1.23E-11 | 7.04E-12 | -0.0994644 | | 0.79248 | -1.59755 | | 15.2286 | 15.2294 | 15.3284 | 15.3902 | 15.515 | 15.3061 | 15.2702 | 15.3266 | 7 | 9 | 9 | 9 | 5 | 7 | 8 | 8 |
| TSPAN14 | Q8NG11;Q | 2.29E-05 | 5.81E-06 | -0.0994073 | | 0.105582 | -0.28643 | | 15.1617 | 14.9612 | 15.1845 | 15.913 | 15.2871 | 14.3422 | 15.0452 | 15.4219 | 3 | 2 | 2 | 3 | 3 | 2 | 2 | 2 |
| RPS2 | P15880 | 1.11E-11 | 6.39E-12 | -0.0992404 | + | 1.32388 | -2.4857 | | 20.7776 | 20.805 | 20.7471 | 20.7901 | 20.9354 | 20.8543 | 20.9429 | 20.8562 | 18 | 18 | 18 | 18 | 17 | 18 | 18 | 18 |
| PPIL4 | Q8WUA2 | 3.38E-14 | 3.68E-14 | -0.0991995 | | 0.529336 | -1.14564 | | 15.5939 | 15.6888 | 15.6319 | 15.874 | 15.8418 | 15.628 | 15.5499 | 15.7932 | 16 | 15 | 15 | 13 | 12 | 15 | 15 | 12 |
| ACADVL | P49748;P4 | 1.72E-10 | 7.99E-11 | -0.0989852 | | 0.46353 | -1.02723 | | 18.0585 | 18.097 | 18.0777 | 18.1404 | 18.2753 | 17.9291 | 18.1853 | 18.3536 | 30 | 29 | 30 | 29 | 28 | 30 | 29 | 30 |
| USP14 | P54578;P5 | 2.62E-19 | 1.43E-18 | -0.0989407 | | 1.16996 | -2.22624 | | 17.9207 | 17.9482 | 17.9131 | 18.0531 | 17.9142 | 18.0936 | 17.9929 | 18.0774 | 24 | 26 | 27 | 26 | 23 | 25 | 25 | 26 |
| STX4 | Q12846 | 1.58E-12 | 1.09E-12 | -0.0988622 | | 0.481923 | -1.06065 | | 16.3882 | 16.4616 | 16.3741 | 16.4029 | 16.7446 | 16.5167 | 16.5231 | 16.3467 | 8 | 8 | 8 | 7 | 7 | 8 | 8 | 8 |
| RMDN1 | Q96DB5 | 1.97E-11 | 1.09E-11 | -0.0988476 | | 0.303746 | -0.723 | | 16.3568 | 16.3382 | 16.2607 | 16.4627 | 16.4211 | 16.0339 | 16.5853 | 16.5841 | 12 | 11 | 12 | 12 | 10 | 9 | 10 | 12 |
| PXDN | Q92626 | 3.47E-18 | 1.28E-17 | -0.0987938 | | 0.241491 | -0.59518 | | 16.1458 | 16.1273 | 16.115 | 15.7926 | 16.147 | 16.4238 | 16.4968 | 16.2806 | 39 | 39 | 38 | 34 | 31 | 36 | 40 | 37 |
| AIDA | Q96BJ3 | 6.22E-06 | 1.67E-06 | -0.0987396 | + | 2.12576 | -3.95601 | | 17.1907 | 17.2551 | 17.25 | 17.3751 | 17.3428 | 17.325 | 17.3277 | 17.2829 | 12 | 13 | 13 | 13 | 13 | 14 | 13 | 13 |
| AP3D1 | O14617;O1 | 7.00E-13 | 5.25E-13 | -0.0985411 | + | 1.96958 | -3.6485 | | 16.7997 | 16.8551 | 16.8681 | 16.9872 | 16.8875 | 16.9605 | 16.9315 | 16.9309 | 43 | 40 | 45 | 43 | 41 | 44 | 41 | 42 |
| TTYH3 | Q9C0H2;Q | 0.000495 | 0.000106 | -0.0984287 | | 0.316259 | -0.74794 | | 17.3654 | 17.4105 | 17.3307 | 17.7606 | 17.4594 | 17.3567 | 17.5775 | 17.1824 | 7 | 7 | 7 | 7 | 6 | 7 | 7 | 7 |
| FAM114A1 | Q8IWE2 | 9.65E-13 | 7.01E-13 | -0.0983822 | | 0.471852 | -1.04238 | | 17.8528 | 17.8624 | 17.8514 | 17.7237 | 18.1233 | 18.0138 | 18.0406 | 17.8682 | 21 | 21 | 21 | 21 | 21 | 20 | 22 | 21 |
| PSMD9 | O00233 | 1.95E-11 | 1.07E-11 | -0.098321 | | 1.13469 | -2.1673 | | 17.7615 | 17.7795 | 17.8288 | 17.9359 | 17.9676 | 17.8609 | 17.7817 | 17.8952 | 12 | 12 | 12 | 12 | 10 | 12 | 11 | 12 |
| SBDS | Q9Y3A5 | 3.28E-16 | 6.41E-16 | -0.0982441 | | 0.665477 | -1.38264 | | 17.7391 | 17.7374 | 17.6725 | 17.7146 | 17.7187 | 17.8336 | 17.9998 | 17.8061 | 19 | 20 | 18 | 19 | 18 | 19 | 19 | 19 |
| THUMPD3 | Q9BV44 | 9.03E-07 | 2.64E-07 | -0.0982233 | | 0.734842 | -1.50054 | | 15.8055 | 15.8866 | 15.7955 | 16.0307 | 15.7733 | 15.9064 | 16.017 | 15.9097 | 12 | 12 | 13 | 14 | 10 | 12 | 12 | 11 |
| TJAP1 | Q5JTD0-2 | 2.95E-14 | 3.26E-14 | -0.0980468 | | 0.366023 | -0.84501 | | 15.3328 | 15.1769 | 15.1969 | 15.2826 | 15.5734 | 15.0627 | 15.3852 | 15.3639 | 12 | 11 | 10 | 10 | 9 | 10 | 9 | 9 |
| POLR2H | P52434 | 4.69E-10 | 2.02E-10 | -0.0980124 | | 0.54378 | -1.17124 | | 15.7612 | 15.7131 | 15.8521 | 15.849 | 15.7557 | 16.0695 | 15.9316 | 15.7616 | 4 | 6 | 6 | 7 | 6 | 5 | 6 | 6 |
| QSOX2 | Q6ZRP7 | 2.56E-24 | 7.58E-23 | -0.0978319 | | 0.486109 | -1.06821 | | 15.3662 | 15.3075 | 15.3183 | 15.5477 | 15.2676 | 15.4336 | 15.6074 | 15.2862 | 16 | 16 | 16 | 16 | 9 | 14 | 15 | 11 |
| STXBP2 | Q15833;Q1 | 0.001908 | 0.000375 | -0.0978031 | | 0.108062 | -0.29518 | | 15.7321 | 15.549 | 15.4375 | 15.3497 | 16.4494 | 15.2387 | NaN | 15.6447 | 1 | 1 | 1 | 1 | 1 | 1 | NaN | 1 |
| C5orf51 | A6NDU8 | 2.79E-09 | 1.09E-09 | -0.0975503 | | 0.208528 | -0.52457 | | 14.9977 | 14.81 | 15.0241 | 14.6148 | 15.4406 | 15.0511 | 15.1511 | 14.9499 | 4 | 6 | 6 | 6 | 3 | 5 | 5 | 3 |
| AKR1B1 | P15121 | 9.01E-14 | 8.70E-14 | -0.0975235 | | 0.168701 | -0.43597 | | 18.6475 | 18.7232 | 18.6556 | 18.34 | 18.7408 | 18.6234 | 19.3629 | 18.7977 | 17 | 17 | 18 | 19 | 17 | 17 | 18 | 17 |
| RAB4A | P20338 | 1.13E-11 | 6.47E-12 | -0.0974962 | | 0.917495 | -1.80613 | | 16.5236 | 16.481 | 16.4993 | 16.5747 | 16.674 | 16.5793 | 16.6942 | 16.4717 | 9 | 9 | 10 | 9 | 8 | 9 | 10 | 9 |
| WDR59 | Q6PJI9 | 9.23E-06 | 2.43E-06 | -0.0973372 | | 0.153921 | -0.40204 | | 14.2935 | 14.3148 | 14.1787 | 13.6562 | 14.6643 | 14.4476 | 14.5648 | 14.4656 | 5 | 5 | 4 | 1 | 3 | 3 | 4 | 4 |
| RBM28 | Q9NW13 | 1.21E-22 | 1.95E-21 | -0.0972296 | + | 1.9165 | -3.54672 | | 15.697 | 15.7279 | 15.6831 | 15.8065 | 15.8065 | 15.7394 | 15.7879 | 15.8591 | 20 | 20 | 20 | 20 | 18 | 21 | 20 | 20 |
| OSMR | Q99650 | 0.000901 | 0.000187 | -0.0971974 | | 0.121342 | -0.32497 | | 14.6611 | 14.6652 | 14.8717 | 15.3757 | 14.7138 | 15.1208 | 14.0685 | 14.8705 | 6 | 6 | 6 | 7 | 4 | 4 | 4 | 4 |
| EDRF1 | Q3B7T1;Q3 | 0.005033 | 0.000899 | -0.0970466 | | 0.069983 | -0.20009 | | 11.484 | NaN | 10.0844 | 10.5191 | 11.1599 | 10.7458 | 11.1002 | NaN | 2 | NaN | 1 | 1 | 1 | 1 | 1 | NaN |
| TBPL1 | P62380 | 7.71E-05 | 1.85E-05 | -0.0970217 | | 0.319205 | -0.75378 | | 14.4547 | 14.3354 | 14.281 | 14.5126 | 14.3144 | 14.5728 | 14.1775 | 14.693 | 3 | 3 | 3 | 2 | 2 | 2 | 2 | 2 |
| GNA11 | P29992 | 1.93E-11 | 1.07E-11 | -0.0969391 | | 1.1808 | -2.24439 | | 18.2133 | 18.2676 | 18.233 | 18.2996 | 18.3022 | 18.4195 | 18.3969 | 18.2564 | 14 | 14 | 15 | 15 | 16 | 15 | 15 | 15 |
| HSPA4 | P34932 | 9.18E-18 | 2.99E-17 | -0.0965825 | | 1.27061 | -2.39542 | | 18.7509 | 18.767 | 18.7586 | 18.804 | 18.8103 | 18.8601 | 18.9693 | 18.8333 | 42 | 42 | 43 | 43 | 41 | 44 | 43 | 42 |
| RPL12 | P30050 | 9.14E-05 | 2.18E-05 | -0.0964965 | | 1.24057 | -2.34477 | | 20.9869 | 20.9808 | 20.9076 | 21.0485 | 21.148 | 21.0129 | 21.0756 | 20.9896 | 9 | 10 | 9 | 9 | 9 | 9 | 10 | 10 |
| POMT1 | Q9Y6A1;Q9 | 0.003509 | 0.000644 | -0.0963711 | | 0.456129 | -1.01371 | | 13.0977 | 13.2597 | 13.4714 | 13.3077 | 13.3382 | 13.2827 | 13.4708 | 13.4637 | 3 | 3 | 4 | 3 | 1 | 2 | 2 | 3 |
| OPA1 | O60313-2 | 9.95E-12 | 5.79E-12 | -0.0962891 | | 1.2261 | -2.32041 | | 15.7959 | 15.8323 | 15.8089 | 15.939 | 15.8408 | 15.8548 | 16.0095 | 15.8992 | 37 | 37 | 37 | 37 | 28 | 35 | 36 | 34 |
| CAPN1 | P07384 | 1.90E-19 | 1.08E-18 | -0.0958402 | | 0.276708 | -0.6683 | | 19.0705 | 19.1729 | 19.1738 | 19.6495 | 19.2073 | 19.0977 | 19.0815 | 19.1384 | 36 | 36 | 36 | 36 | 35 | 36 | 36 | 35 |
| CKAP5 | Q14008 | 2.93E-18 | 1.12E-17 | -0.0958078 | | 0.494321 | -1.08302 | | 16.2749 | 16.2629 | 16.2626 | 16.4694 | 16.4835 | 16.1225 | 16.3229 | 16.4146 | 83 | 88 | 88 | 83 | 78 | 79 | 84 | 84 |
| MCM5 | P33992 | 7.29E-14 | 7.21E-14 | -0.0956794 | | 0.112264 | -0.30288 | | 16.5787 | 16.664 | 16.6266 | 17.2569 | 16.7389 | 15.9361 | 16.5219 | 17.1403 | 25 | 27 | 29 | 28 | 24 | 22 | 27 | 27 |
| NUBP2 | Q9Y5Y2 | 0.000214 | 4.84E-05 | -0.0954025 | | 0.317072 | -0.74955 | | 17.2905 | 17.3901 | 17.378 | 17.7206 | 17.6276 | 17.282 | 17.334 | 17.2771 | 4 | 4 | 5 | 4 | 5 | 5 | 4 | 5 |
| CDC42EP1 | Q00587;Q0 | 2.03E-11 | 1.11E-11 | -0.0953732 | | 0.206145 | -0.51938 | | 16.2419 | 16.2632 | 16.3153 | 16.0062 | 16.1658 | 16.8115 | 16.4185 | 16.4423 | 9 | 8 | 10 | 9 | 9 | 9 | 9 | 9 |
| KRI1 | Q8N9T8 | 9.34E-05 | 2.22E-05 | -0.0953602 | | 0.340939 | -0.79648 | | 14.7813 | 14.7141 | 14.7584 | 15.1525 | 14.9326 | 14.7561 | 14.743 | 14.649 | 9 | 9 | 10 | 9 | 6 | 9 | 9 | 7 |
| TRIP12 | Q14669-2 | 1.54E-16 | 3.44E-16 | -0.0953136 | | 0.605322 | -1.27901 | | 15.662 | 15.6226 | 15.6106 | 15.74 | 15.8611 | 15.5306 | 15.7126 | 15.7909 | 35 | 36 | 34 | 33 | 32 | 33 | 33 | 33 |
| CCZ1B;CCZ1 | P86790;P8 | 8.11E-19 | 3.63E-18 | -0.0952975 | | 0.452451 | -1.00697 | | 16.1127 | 16.0337 | 16.1081 | 15.9697 | 16.3964 | 16.2391 | 16.158 | 16.1375 | 11 | 13 | 11 | 13 | 8 | 11 | 11 | 12 |
| SSH1 | Q8WYL5 | 1.95E-12 | 1.31E-12 | -0.0952307 | | 0.469498 | -1.0381 | | 14.9972 | 14.9123 | 14.9712 | 15.1395 | 15.183 | 14.9237 | 15.1689 | 14.8622 | 12 | 11 | 12 | 13 | 7 | 9 | 9 | 8 |
| NPTN | Q9Y639;Q9 | 7.86E-09 | 2.90E-09 | -0.0951694 | | 0.302857 | -0.72122 | | 17.4088 | 17.3412 | 17.3347 | 17.7271 | 17.1884 | 17.4069 | 17.3327 | 17.6287 | 10 | 10 | 9 | 9 | 9 | 10 | 10 | 10 |
| USO1 | O60763 | 7.01E-13 | 5.25E-13 | -0.0951065 | | 0.556684 | -1.19401 | | 17.8249 | 17.8354 | 17.8318 | 17.8291 | 17.8862 | 18.006 | 18.1157 | 17.792 | 37 | 38 | 38 | 38 | 36 | 35 | 38 | 37 |
| EIF3J | O75822 | 1.48E-10 | 6.94E-11 | -0.0950797 | | 0.34552 | -0.80541 | | 16.7044 | 16.689 | 16.7574 | 16.7194 | 16.5628 | 17.0052 | 17.0189 | 16.7539 | 18 | 18 | 18 | 18 | 18 | 19 | 18 | 18 |
| MYO9A | B2RTY4;B2 | 0.000223 | 5.04E-05 | -0.0950069 | | 0.513596 | -1.1176 | | 15.0427 | 14.894 | 15.0913 | 14.9843 | 15.3046 | 15.0379 | 15.1217 | 15.0734 | 2 | 1 | 2 | 2 | 1 | 2 | 1 | 1 |
| FBXO38 | Q6PIJ6 | 1.45E-07 | 4.60E-08 | -0.0950068 | | 0.388781 | -0.8884 | | 14.9249 | 14.9768 | 14.8637 | 15.0884 | 15.2579 | 14.78 | 14.9826 | 14.9752 | 3 | 3 | 4 | 3 | 3 | 3 | 3 | 3 |
| NAA15 | Q9BXJ9 | 1.15E-11 | 6.61E-12 | -0.0948984 | | 0.743213 | -1.51468 | | 16.9774 | 17.0019 | 16.9985 | 17.2428 | 17.0997 | 16.9501 | 17.0623 | 17.0828 | 36 | 35 | 36 | 38 | 35 | 35 | 34 | 35 |
| PAF1 | Q8N7H5;Q | 6.22E-13 | 4.73E-13 | -0.0948409 | | 0.42345 | -0.95344 | | 15.5556 | 15.5387 | 15.5677 | 15.6932 | 15.7191 | 15.3608 | 15.5806 | 15.7904 | 11 | 11 | 11 | 10 | 8 | 11 | 12 | 11 |
| UPF1 | Q92900;Q9 | 7.85E-21 | 7.33E-20 | -0.0946116 | | 0.737346 | -1.50477 | | 17.5131 | 17.548 | 17.5257 | 17.7545 | 17.7055 | 17.5037 | 17.5518 | 17.6022 | 45 | 44 | 45 | 45 | 42 | 45 | 45 | 45 |
| ARIH2 | O95376 | 1.13E-09 | 4.67E-10 | -0.0945882 | | 0.465047 | -1.03 | | 15.3492 | 15.3833 | 15.3684 | 15.5729 | 15.6145 | 15.3093 | 15.2851 | 15.526 | 8 | 10 | 9 | 10 | 8 | 9 | 9 | 9 |
| HSPA14 | Q0VDF9 | 4.15E-12 | 2.59E-12 | -0.0945491 | | 1.12427 | -2.14991 | | 15.7555 | 15.8161 | 15.7452 | 15.7541 | 15.9418 | 15.8793 | 15.8759 | 15.8829 | 13 | 13 | 14 | 12 | 10 | 12 | 15 | 13 |
| COPS2 | P61201 | 1.24E-11 | 7.06E-12 | -0.094517 | | 1.11793 | -2.13933 | | 17.3223 | 17.352 | 17.3608 | 17.4126 | 17.4767 | 17.4726 | 17.5102 | 17.3257 | 20 | 21 | 22 | 22 | 20 | 22 | 22 | 22 |
| GPAT4 | Q86UL3 | 6.77E-06 | 1.81E-06 | -0.0942717 | | 0.511478 | -1.11381 | | 14.9674 | 14.8608 | 14.8472 | 15.1502 | 14.9209 | 14.8363 | 15.1038 | 14.9193 | 7 | 7 | 7 | 8 | 7 | 5 | 5 | 7 |
| LONP2 | Q86WA8 | 0.001477 | 0.000297 | -0.0940113 | | 0.474662 | -1.04749 | | 15.0363 | 15.0696 | 14.9998 | 15.3018 | 14.9655 | 15.02 | 15.2649 | 15.094 | 5 | 4 | 5 | 4 | 4 | 5 | 5 | 6 |
| PTPN23 | Q9H3S7 | 5.08E-16 | 9.29E-16 | -0.0939552 | | 0.994417 | -1.93391 | | 16.2868 | 16.2689 | 16.2735 | 16.4407 | 16.3396 | 16.2735 | 16.4677 | 16.33 | 37 | 36 | 39 | 40 | 33 | 39 | 38 | 38 |
| TCP1 | P17987 | 2.10E-10 | 9.62E-11 | -0.0939365 | | 1.26747 | -2.39012 | | 19.3824 | 19.4588 | 19.4158 | 19.5642 | 19.5821 | 19.4358 | 19.4828 | 19.4997 | 34 | 32 | 33 | 33 | 31 | 32 | 31 | 32 |
| MCU | Q8NE86;Q8 | 1.56E-10 | 7.26E-11 | -0.0938864 | | 0.45692 | -1.01516 | | 15.8692 | 16.0001 | 15.9664 | 16.0128 | 16.0808 | 16.2478 | 16.016 | 15.838 | 7 | 7 | 10 | 8 | 7 | 7 | 9 | 7 |
| PRPSAP1 | Q14558-2 | 3.27E-11 | 1.72E-11 | -0.0938011 | | 0.303842 | -0.72319 | | 17.2265 | 17.2328 | 17.1939 | 17.4489 | 17.5257 | 17.4281 | 17.0627 | 17.0921 | 11 | 10 | 11 | 11 | 11 | 12 | 10 | 11 |
| MMGT1 | Q8N4V1 | 0.001681 | 0.000335 | -0.0937255 | | 0.363795 | -0.84073 | | 16.2131 | 16.0616 | 16.087 | 16.3138 | 16.1952 | 15.9154 | 16.3634 | 16.2837 | 2 | 2 | 3 | 3 | 1 | 2 | 2 | 2 |
| DDT | P30046 | 3.14E-08 | 1.07E-08 | -0.0937107 | | 0.358034 | -0.82964 | | 18.7894 | 18.8108 | 18.6891 | 18.5628 | 18.6178 | 18.8672 | 18.7581 | 18.8113 | 8 | 8 | 9 | 8 | 7 | 7 | 8 | 7 |
| CFAP20 | Q9Y6A4 | 0.000237 | 5.34E-05 | -0.0935481 | | 0.464597 | -1.02918 | | 16.7656 | 16.7947 | 16.808 | 16.7455 | 17.0474 | 16.7053 | 16.919 | 16.9978 | 6 | 5 | 5 | 6 | 6 | 4 | 5 | 6 |
| G6PD | P11413;P1 | 9.40E-11 | 4.53E-11 | -0.0934504 | | 0.350253 | -0.81459 | | 19.4405 | 19.4839 | 19.454 | 19.7113 | 19.5559 | 19.474 | 19.7487 | 19.2747 | 37 | 37 | 37 | 35 | 36 | 36 | 36 | 36 |
| OBSCN | Q5VST9;Q5 | 0.021192 | 0.00352 | -0.0932532 | | 0.972082 | -1.89682 | | 16.7194 | 16.6959 | 16.7945 | 16.9113 | 16.8601 | 16.8305 | 16.837 | 16.7103 | 1 | 2 | 2 | 1 | 1 | 1 | 2 | 1 |
| RPL8 | P62917 | 9.58E-05 | 2.27E-05 | -0.0931007 | | 1.15403 | -2.1996 | | 20.223 | 20.3443 | 20.2957 | 20.4136 | 20.3935 | 20.4204 | 20.3941 | 20.2822 | 11 | 11 | 11 | 11 | 10 | 11 | 11 | 11 |
| FNDC3B | Q53EP0 | 1.42E-12 | 9.87E-13 | -0.0930785 | | 0.480474 | -1.05802 | | 16.4713 | 16.4748 | 16.4188 | 16.4588 | 16.4327 | 16.7723 | 16.62 | 16.4565 | 26 | 27 | 26 | 26 | 25 | 27 | 23 | 25 |
| STK39 | Q9UEW8;Q | 2.02E-07 | 6.34E-08 | -0.092991 | | 0.237506 | -0.58677 | | 16.3401 | 16.2447 | 16.3465 | 16.7815 | 16.1366 | 16.5129 | 16.406 | 16.18 | 8 | 7 | 8 | 9 | 8 | 8 | 10 | 8 |
| BBS7 | Q8IWZ6 | 0.000231 | 5.21E-05 | -0.0929609 | | 0.126243 | -0.33678 | | 14.2728 | 14.186 | 14.2645 | 14.0363 | 14.743 | 14.7008 | 14.5121 | 13.678 | 3 | 4 | 3 | 2 | 3 | 3 | 4 | 1 |
| AK1 | P00568 | 6.37E-17 | 1.59E-16 | -0.0929187 | | 0.336689 | -0.78818 | | 18.2649 | 18.3117 | 18.2277 | 18.0894 | 18.5875 | 18.5083 | 18.3309 | 18.2891 | 12 | 13 | 13 | 13 | 12 | 13 | 12 | 12 |
| DPCD | Q9BVM2 | 0.020701 | 0.003442 | -0.0925645 | | 0.261861 | -0.63775 | | 16.4386 | 16.7206 | 16.831 | 16.5862 | 16.5772 | 16.8513 | 17.0456 | 16.7195 | 3 | 3 | 5 | 4 | 4 | 4 | 4 | 4 |
| ARID1A | O14497;O1 | 1.76E-08 | 6.21E-09 | -0.0924105 | | 0.527677 | -1.14269 | | 14.649 | 14.734 | 14.6569 | 14.9854 | 14.7086 | 14.6991 | 14.6587 | 14.8101 | 13 | 12 | 12 | 13 | 9 | 12 | 7 | 13 |
| GDI1 | P31150 | 1.57E-09 | 6.34E-10 | -0.0923705 | | 0.405108 | -0.91918 | | 18.5452 | 18.5039 | 18.4368 | 18.7678 | 18.404 | 18.7546 | 18.5152 | 18.4966 | 15 | 16 | 16 | 16 | 16 | 16 | 16 | 16 |
| PIP4K2B | P78356 | 5.36E-09 | 2.02E-09 | -0.0923335 | | 0.672974 | -1.39546 | | 16.5232 | 16.6094 | 16.6016 | 16.7395 | 16.6263 | 16.6188 | 16.8159 | 16.5515 | 10 | 11 | 12 | 12 | 10 | 10 | 12 | 12 |
| CCDC12 | Q8WUD4 | 0.002405 | 0.000462 | -0.0923246 | | 0.232636 | -0.57644 | | 15.589 | 15.6245 | 15.5999 | 15.7544 | 15.7892 | 15.2277 | 15.9065 | 15.8063 | 4 | 3 | 4 | 3 | 2 | 3 | 3 | 3 |
| FAU | P62861 | 2.75E-09 | 1.07E-09 | -0.0922863 | | 0.055668 | -0.15793 | | 21.9507 | 21.5753 | 21.7038 | 20.9276 | 23.333 | 21.5639 | 21.1392 | 22.2141 | 2 | 2 | 2 | 2 | 2 | 2 | 2 | 2 |
| NOB1 | Q9ULX3 | 8.99E-05 | 2.14E-05 | -0.0921182 | | 0.369799 | -0.85225 | | 16.3149 | 16.3115 | 16.4063 | 16.3986 | 16.7005 | 16.2212 | 16.3672 | 16.4943 | 5 | 6 | 7 | 8 | 6 | 6 | 7 | 8 |
| DCUN1D4 | Q92564;Q9 | 0.005091 | 0.000909 | -0.0919415 | | 0.373432 | -0.8592 | | 16.18 | 16.3684 | 16.347 | 16.4506 | 16.3916 | 16.1213 | 16.5646 | 16.4242 | 3 | 2 | 1 | 3 | 2 | 2 | 2 | 2 |

| Gene names | UniProt IDs | Protein Group P-value | Protein Group Q-value | Log2 Difference NUP214 vs Controls (n=5) | p<0.05 | -log10 p-value | T-test Test statistic | NPC Localization | Log2 MS2_quantity_ NUP214_1 | Log2 MS2_quantity_ NUP214_2 | Log2 MS2_quantity_ NUP214_3 | Log2 MS2_quantity_ Control_1 | Log2 MS2_quantity_ Control_2 | Log2 MS2_quantity_ Control_3 | Log2 MS2_quantity_ Control_4 | Log2 MS2_quantity_ Control_5 | Number Peptides for Quantitation NUP214_1 | Number Peptides for Quantitation NUP214_2 | Number Peptides for Quantitation NUP214_3 | Number Peptides for Quantitation Control_1 | Number Peptides for Quantitation Control_2 | Number Peptides for Quantitation Control_3 | Number Peptides for Quantitation Control_4 | Number Peptides for Quantitation Control_5 |
|---|---|---|---|---|---|---|---|---|---|---|---|---|---|---|---|---|---|---|---|---|---|---|---|---|
| STT3B | Q8TCJ2 | 1.87E-05 | 4.77E-06 | -0.0919385 | | 0.662569 | -1.37767 | | 17.4867 | 17.5092 | 17.5441 | 17.7849 | 17.5038 | 17.6261 | 17.572 | 17.5395 | 14 | 14 | 15 | 14 | 14 | 15 | 14 | 15 |
| ZMAT3 | Q9HA38;Q9 | 4.75E-09 | 1.80E-09 | -0.0917622 | | 0.261222 | -0.63642 | | 14.4835 | 14.3417 | 14.5878 | 14.3111 | 14.8174 | 14.737 | 14.5949 | 14.3534 | 2 | 3 | 2 | 3 | 2 | 2 | 2 | 3 |
| AP2M1 | Q96CW1;Q | 1.41E-12 | 9.77E-13 | -0.0916176 | | 1.14646 | -2.18695 | | 18.8012 | 18.8101 | 18.7774 | 18.8953 | 18.9483 | 18.8804 | 18.94 | 18.7752 | 25 | 25 | 26 | 25 | 26 | 26 | 25 | 25 |
| QTRT2 | Q9H974;Q9 | 1.20E-06 | 3.45E-07 | -0.0915422 | | 0.25006 | -0.61318 | | 15.4808 | 15.5628 | 15.6571 | 15.4782 | 16.0224 | 15.4868 | 15.7951 | 15.5098 | 5 | 5 | 4 | 4 | 2 | 5 | 2 | 5 |
| NMD3 | Q96D46 | 1.69E-09 | 6.75E-10 | -0.0913928 | | 0.802877 | -1.61498 | | 15.4317 | 15.4852 | 15.5318 | 15.6878 | 15.5063 | 15.5561 | 15.4806 | 15.6407 | 16 | 15 | 13 | 16 | 12 | 16 | 15 | 15 |
| DNAJB5 | O75953;O7 | 0.035851 | 0.005861 | -0.0913167 | | 0.314279 | -0.79445 | | 13.8337 | 13.8965 | 13.9502 | 13.842 | NaN | NaN | NaN | 14.1275 | 2 | 1 | 2 | 2 | NaN | NaN | NaN | 1 |
| SNUPN | O95149 | 2.29E-12 | 1.51E-12 | -0.0913005 | | 0.319545 | -0.75445 | | 15.0044 | 15.1135 | 15.0285 | 15.2406 | 15.0758 | 14.8188 | 15.2777 | 15.2877 | 4 | 3 | 3 | 2 | 3 | 2 | 2 | 2 |
| UBE2L3 | P68036;P6 | 5.71E-05 | 1.39E-05 | -0.0912278 | | 0.528644 | -1.14441 | | 19.1324 | 19.1109 | 19.1845 | 19.4226 | 19.093 | 19.1366 | 19.2938 | 19.2231 | 8 | 7 | 8 | 8 | 6 | 6 | 7 | 8 |
| NUDT1 | P36639;P3 | 3.63E-12 | 2.30E-12 | -0.0912029 | | 0.419834 | -0.94671 | | 16.0584 | 15.9349 | 16.066 | 16.1627 | 16.2213 | 15.8991 | 16.0082 | 16.2635 | 8 | 8 | 8 | 7 | 7 | 7 | 7 | 8 |
| VMP1 | Q96GC9 | 1.06E-09 | 4.39E-10 | -0.0911284 | | 0.158229 | -0.41199 | | 16.4659 | 16.6773 | 16.5779 | 17.2266 | 16.2618 | 16.7261 | 16.6599 | 16.4498 | 2 | 2 | 2 | 2 | 2 | 3 | 3 | 2 |
| MPDZ | O75970;O7 | 2.84E-08 | 9.78E-09 | -0.0909269 | | 0.205856 | -0.51875 | | 14.7702 | 14.8341 | 14.7296 | 15.0935 | 15.0981 | 14.7707 | 14.9775 | 14.4045 | 29 | 31 | 32 | 30 | 24 | 26 | 29 | 26 |
| CFAP298 | P57076 | 2.47E-09 | 9.69E-10 | -0.0908724 | | 0.515728 | -1.12141 | | 15.16 | 15.252 | 15.1024 | 15.1813 | 15.37 | 15.0877 | 15.2988 | 15.374 | 1 | 1 | 1 | 2 | 1 | 3 | 2 | 2 |
| GSPT2 | Q8IYD1 | 1.79E-07 | 5.65E-08 | -0.0906609 | + | 1.45197 | -2.70541 | | 15.5418 | 15.5162 | 15.6034 | 15.5972 | 15.614 | 15.6226 | 15.6954 | 15.6932 | 6 | 7 | 7 | 8 | 8 | 6 | 7 | 8 |
| ATP6V1E1 | P36543 | 7.41E-08 | 2.42E-08 | -0.0904112 | | 0.287524 | -0.69032 | | 18.3799 | 18.3109 | 18.3878 | 18.4958 | 18.3425 | 18.1646 | 18.7541 | 18.4927 | 9 | 9 | 9 | 9 | 9 | 8 | 10 | 8 |
| STRAP | Q9Y3F4;Q9 | 7.27E-17 | 1.78E-16 | -0.0898856 | | 0.711511 | -1.46105 | | 17.9223 | 17.932 | 17.8921 | 18.1107 | 17.8452 | 18.0015 | 18.0749 | 17.9944 | 23 | 23 | 22 | 20 | 21 | 23 | 21 | 23 |
| NOL7 | Q9UMY1 | 2.03E-06 | 5.69E-07 | -0.0898556 | | 0.458179 | -1.01746 | | 15.3392 | 15.2241 | 15.128 | 15.3393 | 15.4883 | 15.2033 | 15.3868 | 15.1837 | 3 | 3 | 3 | 4 | 2 | 3 | 4 | 3 |
| AARSD1 | Q9BTE6;Q9 | 4.14E-10 | 1.80E-10 | -0.089786 | | 0.777751 | -1.57283 | | 16.5828 | 16.6424 | 16.6195 | 16.605 | 16.7144 | 16.8439 | 16.7259 | 16.6342 | 11 | 12 | 12 | 12 | 11 | 12 | 12 | 11 |
| NME2 | P22392-2 | 2.77E-12 | 1.80E-12 | -0.0896242 | | 0.41896 | -0.94509 | | 21.5817 | 21.6455 | 21.5555 | 21.8272 | 21.4749 | 21.7967 | 21.7573 | 21.5632 | 17 | 17 | 18 | 19 | 19 | 18 | 18 | 17 |
| PLEKHG4 | Q58EX7;Q5 | 0.008128 | 0.001411 | -0.0896216 | | 0.101844 | -0.27963 | | 14.6776 | 14.6882 | 14.8618 | 15.5873 | 14.4763 | NaN | 14.4295 | 14.8355 | 2 | 2 | 3 | 5 | 2 | NaN | 1 | 2 |
| RPN2 | P04844 | 2.36E-10 | 1.07E-10 | -0.0895943 | | 0.482527 | -1.06174 | | 19.6227 | 19.6628 | 19.6189 | 19.5314 | 19.784 | 19.8221 | 19.8605 | 19.6241 | 23 | 23 | 23 | 23 | 24 | 23 | 24 | 24 |
| NAP1L5 | Q96NT1 | 0.002723 | 0.000515 | -0.0894709 | | 0.075589 | -0.21504 | | 14.3436 | 14.3339 | 14.4161 | 14.97 | NaN | NaN | 14.7596 | 13.6324 | 4 | 3 | 3 | 4 | NaN | NaN | 3 | 4 |
| METTL1 | Q9UBP6 | 3.86E-09 | 1.48E-09 | -0.0893212 | | 0.230934 | -0.57281 | | 15.6506 | 15.6555 | 15.7269 | 16.1124 | 15.442 | 15.7483 | 15.6167 | 15.9155 | 6 | 7 | 8 | 8 | 6 | 6 | 6 | 8 |
| BLOC1S6 | Q9UL45 | 2.40E-12 | 1.58E-12 | -0.0891136 | | 0.098269 | -0.26824 | | 14.5683 | 14.2722 | 14.2218 | 14.4603 | 14.8807 | 14.9936 | 13.6476 | 14.2338 | 2 | 2 | 3 | 2 | 1 | 1 | 1 | 2 |
| CALR | P27797 | 1.09E-13 | 1.02E-13 | -0.0889226 | | 0.693421 | -1.43032 | | 21.6151 | 21.7231 | 21.6766 | 21.6777 | 21.8872 | 21.7678 | 21.8166 | 21.6532 | 23 | 24 | 24 | 23 | 23 | 23 | 23 | 23 |
| CLIP2 | Q9UDT6;Q9 | 2.63E-15 | 3.99E-15 | -0.0886681 | | 0.291882 | -0.69914 | | 15.5486 | 15.6157 | 15.5392 | 15.5526 | 15.9965 | 15.488 | 15.7236 | 15.5216 | 41 | 37 | 39 | 33 | 39 | 31 | 41 | 35 |
| MINPP1 | Q9UNW1 | 3.43E-15 | 5.01E-15 | -0.088612 | | 0.275446 | -0.66571 | | 16.8504 | 16.812 | 16.8542 | 16.5775 | 16.8938 | 17.0816 | 17.1529 | 16.9316 | 8 | 9 | 10 | 10 | 8 | 10 | 10 | 8 |
| PDLIM7 | Q9NR12 | 6.63E-09 | 2.46E-09 | -0.0885899 | | 0.271021 | -0.65664 | | 19.0326 | 18.9827 | 19.007 | 18.8535 | 19.0245 | 19.4659 | 19.0432 | 19.093 | 22 | 22 | 21 | 21 | 21 | 22 | 22 | 21 |
| BMI1 | P35226 | 0.002579 | 0.000491 | -0.0885689 | | 0.497529 | -1.0888 | | 14.8626 | 14.9881 | 15.068 | 15.0356 | 15.1747 | 14.8907 | 15.0446 | 15.1618 | 3 | 3 | 3 | 2 | 2 | 3 | 3 | 3 |
| MUL1 | Q969V5 | 0.001162 | 0.000237 | -0.0885668 | | 0.304048 | -0.72361 | | 14.4713 | 14.4069 | 14.4257 | 14.3806 | 14.7351 | 14.2915 | 14.7351 | 14.4736 | 5 | 4 | 4 | 4 | 4 | 4 | 5 | 4 |
| B3GLCT | Q6Y288 | 8.49E-10 | 3.55E-10 | -0.0884186 | | 0.219879 | -0.54914 | | 15.1743 | 15.1834 | 15.125 | 15.078 | 15.292 | 15.0005 | 15.6884 | 15.1876 | 9 | 8 | 8 | 7 | 6 | 8 | 7 | 5 |
| NUMB | P49757;P4 | 3.41E-15 | 4.99E-15 | -0.0883539 | | 0.345592 | -0.80554 | | 16.9389 | 16.956 | 17.0272 | 17.3608 | 16.9071 | 17.0453 | 16.9303 | 17.0686 | 12 | 10 | 11 | 12 | 12 | 11 | 12 | 12 |
| DNAJC1 | Q96KC8 | 8.15E-13 | 6.01E-13 | -0.0883039 | | 0.226133 | -0.56257 | | 15.1198 | 15.133 | 15.1417 | 15.1612 | 15.175 | 15.3385 | 15.5708 | 14.8534 | 12 | 13 | 13 | 11 | 8 | 13 | 14 | 10 |
| LDHB | P07195 | 1.38E-08 | 4.94E-09 | -0.088172 | | 0.859081 | -1.70889 | | 20.3056 | 20.2865 | 20.2904 | 20.406 | 20.2569 | 20.4551 | 20.4591 | 20.3348 | 19 | 19 | 19 | 19 | 18 | 19 | 20 | 19 |
| SPCS2 | Q15005 | 0.000262 | 5.85E-05 | -0.0881383 | | 0.841354 | -1.67932 | | 18.5808 | 18.6155 | 18.5055 | 18.6887 | 18.5738 | 18.637 | 18.7734 | 18.604 | 12 | 12 | 12 | 12 | 12 | 12 | 12 | 12 |
| RPN1 | P04843 | 3.64E-07 | 1.11E-07 | -0.0879972 | | 0.525121 | -1.13815 | | 20.287 | 20.3316 | 20.2977 | 20.2479 | 20.4454 | 20.4863 | 20.5235 | 20.264 | 37 | 37 | 37 | 37 | 37 | 37 | 37 | 37 |
| CDK9 | P50750;P5 | 0.0002 | 4.55E-05 | -0.0873839 | | 0.726121 | -1.4858 | | 15.5028 | 15.4289 | 15.4437 | 15.6539 | 15.5891 | 15.5119 | 15.5716 | 15.4027 | 4 | 4 | 3 | 3 | 4 | 3 | 4 | 5 |
| ATIC | P31939 | 2.11E-12 | 1.41E-12 | -0.0873727 | | 0.391685 | -0.8939 | | 18.807 | 18.8452 | 18.8091 | 18.7117 | 18.9403 | 19.1239 | 18.7842 | 18.979 | 35 | 34 | 34 | 35 | 33 | 35 | 34 | 34 |
| PBDC1 | Q9BVG4 | 5.49E-12 | 3.35E-12 | -0.0872875 | | 0.509656 | -1.11055 | | 17.7402 | 17.6646 | 17.7688 | 17.6889 | 17.7372 | 17.81 | 18.0191 | 17.8039 | 9 | 10 | 11 | 9 | 8 | 9 | 9 | 9 |
| LMAN2 | Q12907 | 1.22E-13 | 1.13E-13 | -0.0871298 | | 0.279198 | -0.67338 | | 19.439 | 19.5089 | 19.5137 | 19.3982 | 19.4632 | 19.7181 | 19.8811 | 19.4111 | 15 | 14 | 15 | 15 | 14 | 15 | 15 | 15 |
| ARMT1 | Q9H993 | 6.60E-05 | 1.60E-05 | -0.0868973 | | 0.858447 | -1.70783 | | 16.6506 | 16.7062 | 16.7339 | 16.756 | 16.8378 | 16.8786 | 16.6707 | 16.7759 | 9 | 8 | 10 | 10 | 10 | 9 | 8 | 9 |
| CRELD2 | Q6UXH1;Q | 1.10E-09 | 4.55E-10 | -0.0866327 | | 0.292782 | -0.70096 | | 15.7833 | 15.7536 | 15.7805 | 15.9055 | 15.5185 | 15.8809 | 16.0835 | 15.9072 | 8 | 7 | 8 | 7 | 8 | 8 | 8 | 8 |
| CCT7 | Q99832 | 5.08E-14 | 5.25E-14 | -0.0863441 | | 0.727146 | -1.48753 | | 18.9571 | 18.9928 | 18.937 | 19.1467 | 19.135 | 18.9969 | 18.92 | 19.0446 | 39 | 37 | 39 | 39 | 38 | 37 | 39 | 36 |
| CBLL1 | Q75N03;Q | 0.000294 | 6.54E-05 | -0.08629 | | 0.196512 | -0.49825 | | 15.5259 | 15.3367 | 15.2268 | 15.3585 | 15.6634 | 15.3993 | 15.0721 | 15.7539 | 1 | 1 | 2 | 2 | 1 | 1 | 1 | 1 |
| C9orf64 | Q5T6V5 | 3.30E-11 | 1.74E-11 | -0.0862616 | | 0.409272 | -0.92699 | | 16.5358 | 16.5447 | 16.5166 | 16.6851 | 16.4867 | 16.8608 | 16.5515 | 16.509 | 14 | 14 | 14 | 15 | 12 | 15 | 15 | 13 |
| LLPH | Q9BRT6 | 3.31E-11 | 1.74E-11 | -0.0862287 | | 0.176933 | -0.45461 | | 15.5643 | 15.5678 | 15.6877 | 15.987 | 15.3548 | 15.3515 | 15.9267 | 15.8439 | 2 | 4 | 4 | 4 | 1 | 3 | 1 | 3 |
| ARL1 | P40616;P4 | 8.59E-14 | 8.35E-14 | -0.0861736 | | 0.291561 | -0.69849 | | 18.4196 | 18.4166 | 18.4389 | 18.5231 | 18.2155 | 18.6671 | 18.7346 | 18.4157 | 7 | 8 | 7 | 7 | 7 | 7 | 7 | 8 |
| RPL18 | Q07020 | 0.000326 | 7.21E-05 | -0.0860686 | | 0.794942 | -1.60168 | | 20.9931 | 21.1785 | 21.0537 | 21.1223 | 21.1384 | 21.1851 | 21.2552 | 21.1049 | 5 | 5 | 5 | 5 | 6 | 5 | 5 | 5 |
| TRUB1 | Q8WWH5 | 0.002073 | 0.000405 | -0.0859392 | | 0.527514 | -1.1424 | | 15.0895 | 15.096 | 14.927 | 15.083 | 15.18 | 14.9633 | 15.1481 | 15.2427 | 4 | 4 | 3 | 3 | 3 | 4 | 4 | 4 |
| HCCS | P53701 | 2.16E-07 | 6.77E-08 | -0.0856486 | | 0.383122 | -0.87767 | | 15.7335 | 15.7874 | 15.7512 | 15.8815 | 15.5817 | 15.9678 | 15.9802 | 15.8041 | 10 | 11 | 11 | 11 | 10 | 10 | 11 | 11 |
| LSG1 | Q9H089 | 5.87E-09 | 2.20E-09 | -0.0856342 | | 0.465855 | -1.03147 | | 15.5656 | 15.6809 | 15.5851 | 15.9258 | 15.6032 | 15.6633 | 15.6767 | 15.6118 | 13 | 14 | 16 | 18 | 12 | 15 | 15 | 15 |
| RPS11 | P62280 | 2.78E-14 | 3.10E-14 | -0.0856074 | + | 1.50229 | -2.79291 | | 20.3028 | 20.3551 | 20.275 | 20.3903 | 20.4619 | 20.381 | 20.4046 | 20.3452 | 15 | 15 | 15 | 14 | 15 | 15 | 14 | 15 |
| RPL10A | P62906 | 4.41E-16 | 8.28E-16 | -0.0855672 | | 0.789221 | -1.59208 | | 19.4868 | 19.5195 | 19.4993 | 19.5087 | 19.7318 | 19.5922 | 19.5869 | 19.5176 | 13 | 13 | 13 | 13 | 13 | 13 | 13 | 13 |
| FAM120B | Q96EK7 | 2.22E-09 | 8.74E-10 | -0.0852833 | | 1.13226 | -2.16324 | | 15.8329 | 15.7874 | 15.756 | 15.9225 | 15.8149 | 15.9331 | 15.9068 | 15.8097 | 12 | 12 | 13 | 13 | 7 | 11 | 10 | 11 |
| MCMBP | Q9BTE3;Q9 | 6.90E-12 | 4.15E-12 | -0.0848216 | | 0.234096 | -0.57954 | | 15.0536 | 15.1731 | 15.1439 | 15.408 | 15.3036 | 14.8382 | 15.0969 | 15.395 | 18 | 18 | 18 | 18 | 15 | 13 | 17 | 17 |
| ATL2 | Q8NHH9;Q | 6.52E-09 | 2.43E-09 | -0.0848206 | | 0.441461 | -0.98678 | | 14.9862 | 15.0063 | 15.0263 | 15.0753 | 14.9438 | 14.9717 | 15.1763 | 15.2882 | 5 | 7 | 7 | 8 | 5 | 6 | 8 | 6 |
| UBR3 | Q6ZT12;Q6 | 2.97E-08 | 1.02E-08 | -0.0848143 | | 0.415916 | -0.93941 | | 14.5152 | 14.4148 | 14.559 | 14.4346 | 14.7696 | 14.6398 | 14.6182 | 14.4435 | 12 | 10 | 11 | 8 | 4 | 9 | 8 | 5 |
| ATXN2L | Q8WWM7 | 2.74E-18 | 1.05E-17 | -0.0847117 | | 0.539845 | -1.16428 | | 16.6834 | 16.6934 | 16.7533 | 16.7439 | 16.6125 | 16.9079 | 16.8759 | 16.8336 | 26 | 27 | 27 | 27 | 24 | 27 | 26 | 26 |
| IKBKB | O14920 | 1.30E-14 | 1.60E-14 | -0.0847082 | | 0.319469 | -0.7543 | | 15.9744 | 15.9298 | 15.9588 | 16.1346 | 16.2758 | 15.8314 | 15.8654 | 16.0879 | 16 | 19 | 18 | 17 | 14 | 14 | 17 | 15 |
| DCAF1 | Q9Y4B6;Q9 | 0.00023 | 5.19E-05 | -0.0846369 | | 1.05739 | -2.03853 | | 13.8283 | 13.9444 | 13.844 | 13.9882 | 13.8759 | 13.96 | 13.9425 | 14.0178 | 10 | 10 | 12 | 7 | 7 | 8 | 7 | 7 |
| GNE | Q9Y223;Q9 | 2.76E-10 | 1.23E-10 | -0.0845355 | | 0.547085 | -1.17709 | | 16.1684 | 16.1972 | 16.1996 | 16.484 | 16.2475 | 16.2275 | 16.1902 | 16.2154 | 23 | 23 | 23 | 23 | 17 | 22 | 21 | 22 |
| NTAN1 | Q96AB6 | 0.000118 | 2.77E-05 | -0.0844278 | | 0.404615 | -0.91826 | | 16.0819 | 16.1585 | 16.0654 | 16.262 | 16.3869 | 16.0123 | 16.2015 | 16.0692 | 9 | 8 | 8 | 8 | 7 | 8 | 8 | 8 |
| ILVBL | A1L0T0 | 1.25E-06 | 3.60E-07 | -0.0843171 | | 0.238231 | -0.5883 | | 17.6495 | 17.734 | 17.7009 | 17.3996 | 17.7981 | 17.9834 | 17.9781 | 17.7366 | 16 | 17 | 17 | 15 | 15 | 16 | 16 | 18 |
| EIF3C | Q99613 | 1.59E-11 | 8.88E-12 | -0.0842154 | | 0.494629 | -1.08358 | | 18.3029 | 18.3336 | 18.2726 | 18.5197 | 18.4815 | 18.2035 | 18.3158 | 18.4157 | 30 | 30 | 30 | 30 | 31 | 30 | 30 | 30 |
| ASAH1 | Q13510;Q1 | 3.84E-06 | 1.05E-06 | -0.0840538 | | 0.192944 | -0.49036 | | 17.9494 | 17.9464 | 17.9056 | 17.8381 | 17.7825 | 17.8924 | 18.4879 | 18.0883 | 10 | 10 | 11 | 10 | 10 | 9 | 13 | 11 |
| STIM1 | Q13586 | 2.40E-19 | 1.32E-18 | -0.0837565 | | 0.472376 | -1.04333 | | 16.2002 | 16.2066 | 16.2326 | 16.098 | 16.4652 | 16.2784 | 16.3566 | 16.2863 | 22 | 24 | 24 | 21 | 20 | 22 | 21 | 21 |
| BAP1 | Q92560 | 0.020478 | 0.003407 | -0.0836951 | | 0.261012 | -0.65493 | | 14.3848 | 14.2598 | 14.2652 | NaN | 14.1451 | NaN | 14.5199 | 14.4958 | 2 | 1 | 1 | NaN | 1 | NaN | 3 | 1 |
| PEBP1 | P30086 | 4.75E-10 | 2.05E-10 | -0.083616 | | 0.675265 | -1.39937 | | 20.5753 | 20.6609 | 20.5626 | 20.672 | 20.6747 | 20.7683 | 20.7622 | 20.539 | 13 | 13 | 13 | 13 | 13 | 13 | 13 | 13 |
| PFN2 | P35080-2 | 9.05E-09 | 3.31E-09 | -0.0834648 | | 0.190083 | -0.48402 | | 19.8998 | 19.8066 | 19.7644 | 19.7691 | 20.4083 | 19.7504 | 19.7378 | 19.8697 | 7 | 7 | 7 | 7 | 7 | 7 | 7 | 7 |
| MLH1 | P40692 | 5.80E-07 | 1.73E-07 | -0.0833752 | | 0.128692 | -0.34265 | | 14.2238 | 14.256 | 14.1188 | 14.5011 | 13.9488 | 13.7494 | 14.5852 | 14.63 | 2 | 2 | 2 | 2 | 3 | 1 | 3 | 2 |
| BOLA1 | Q9Y3E2 | 0.001337 | 0.000271 | -0.083291 | | 0.066004 | -0.18543 | | 16.455 | 16.9231 | 16.7361 | 17.1623 | 15.6318 | 16.5021 | 17.1992 | 17.4446 | 2 | 2 | 2 | 1 | 1 | 2 | 2 | 2 |
| KIF3A | Q9Y496 | 1.12E-07 | 3.59E-08 | -0.0832155 | | 0.222628 | -0.55506 | | 14.5639 | 14.6683 | 14.7209 | 14.7669 | 14.3042 | 14.897 | 14.8551 | 14.8482 | 10 | 11 | 11 | 11 | 8 | 11 | 12 | 9 |
| PARK7 | Q99497 | 2.55E-08 | 8.82E-09 | -0.0831748 | | 0.291214 | -0.69779 | | 19.7998 | 19.6612 | 19.8107 | 19.5339 | 19.8812 | 19.9599 | 20.0261 | 19.801 | 19 | 18 | 18 | 18 | 19 | 19 | 18 | 19 |
| MFSD10 | Q14728 | 0.001378 | 0.000278 | -0.0831039 | | 0.472486 | -1.04353 | | 17.8379 | 17.9302 | 17.8276 | 17.8214 | 18.0583 | 17.9411 | 18.0954 | 17.8255 | 6 | 6 | 6 | 6 | 6 | 6 | 5 | 5 |
| UBE3B | Q7Z3V4 | 3.37E-07 | 1.04E-07 | -0.0829116 | | 0.604451 | -1.2775 | | 15.9905 | 15.906 | 15.8464 | 16.0834 | 15.8469 | 16.0472 | 15.9581 | 16.0505 | 7 | 6 | 7 | 7 | 5 | 8 | 6 | 7 |
| PSMB1 | P20618 | 1.80E-14 | 2.12E-14 | -0.0828449 | | 0.666465 | -1.38433 | | 18.9317 | 18.9051 | 18.888 | 18.8999 | 18.9062 | 19.101 | 19.0933 | 18.9553 | 15 | 15 | 15 | 15 | 15 | 15 | 15 | 15 |
| CHMP1B | Q7LBR1 | 0.001115 | 0.000229 | -0.0827477 | | 0.587614 | -1.2482 | | 16.4836 | 16.5443 | 16.3821 | 16.4643 | 16.4666 | 16.6942 | 16.5794 | 16.5592 | 4 | 4 | 4 | 4 | 4 | 4 | 4 | 4 |
| SNX17 | Q15036 | 1.75E-13 | 1.56E-13 | -0.0822378 | | 0.333791 | -0.78251 | | 17.1559 | 17.1785 | 17.131 | 17.5102 | 17.1825 | 17.0293 | 17.2666 | 17.1982 | 18 | 18 | 19 | 18 | 16 | 16 | 18 | 17 |
| MAP1A | P78559 | 8.17E-15 | 1.06E-14 | -0.0822122 | | 0.348657 | -0.8115 | | 17.5751 | 17.5922 | 17.6353 | 17.9718 | 17.5577 | 17.5689 | 17.6475 | 17.6696 | 96 | 98 | 105 | 98 | 88 | 98 | 96 | 101 |

| Gene names | UniProt IDs | Protein Group P-value | Protein Group Q-value | Log2 Difference NUP214 vs Controls (n=5) | p<0.05 | -log10 p-value | T-test Test statistic | NPC Localization | Log2 MS2_quantity_NUP214_1 | Log2 MS2_quantity_NUP214_2 | Log2 MS2_quantity_NUP214_3 | Log2 MS2_quantity_Control_1 | Log2 MS2_quantity_Control_2 | Log2 MS2_quantity_Control_3 | Log2 MS2_quantity_Control_4 | Log2 MS2_quantity_Control_5 | Number Peptides for Quantitation NUP214_1 | Number Peptides for Quantitation NUP214_2 | Number Peptides for Quantitation NUP214_3 | Number Peptides for Quantitation Control_1 | Number Peptides for Quantitation Control_2 | Number Peptides for Quantitation Control_3 | Number Peptides for Quantitation Control_4 | Number Peptides for Quantitation Control_5 |
|---|---|---|---|---|---|---|---|---|---|---|---|---|---|---|---|---|---|---|---|---|---|---|---|---|
| GALNS | P34059 | 1.62E-13 | 1.45E-13 | -0.0821636 | | 0.29641 | -0.70827 | | 16.2754 | 16.1499 | 16.2481 | 16.2392 | 16.4699 | 16.2724 | 16.5086 | 16.043 | 9 | 9 | 8 | 8 | 8 | 7 | 10 | 7 |
| MIA2 | Q96PC5;Q9 | 1.69E-20 | 1.41E-19 | -0.0820869 | | 0.385532 | -0.88224 | | 14.8446 | 14.9531 | 15.04 | 15.0569 | 14.9357 | 15.1838 | 15.1238 | 14.8398 | 15 | 15 | 18 | 18 | 10 | 18 | 17 | 15 |
| GNL3L | Q9NVN8 | 0.002625 | 0.000499 | -0.0818124 | | 0.434683 | -0.97427 | | 15.0557 | 15.1494 | 15.0424 | 15.1976 | 15.2668 | 14.936 | 15.1632 | 15.2581 | 4 | 4 | 5 | 4 | 4 | 3 | 4 | 5 |
| AHSA1 | O95433 | 5.88E-10 | 2.50E-10 | -0.0816232 | | 0.262289 | -0.63863 | | 17.8067 | 17.8511 | 17.8466 | 18.1965 | 17.6067 | 17.854 | 17.9662 | 17.9588 | 18 | 19 | 18 | 18 | 17 | 18 | 19 | 19 |
| RAB3GAP2 | Q9H2M9 | 3.01E-12 | 1.94E-12 | -0.0813956 | | 0.81692 | -1.63849 | | 16.5486 | 16.6251 | 16.5707 | 16.7871 | 16.5937 | 16.5968 | 16.6726 | 16.664 | 39 | 41 | 42 | 40 | 37 | 40 | 39 | 41 |
| C2CD5 | Q86YS7-2;( | 8.77E-07 | 2.57E-07 | -0.0813946 | | 0.356205 | -0.82611 | | 15.265 | 15.3299 | 15.2571 | 15.3555 | 15.1508 | 15.5841 | 15.4458 | 15.2906 | 13 | 12 | 15 | 12 | 8 | 11 | 11 | 11 |
| COG6 | Q9Y2V7 | 1.35E-07 | 4.30E-08 | -0.0813782 | | 0.486622 | -1.06914 | | 16.2389 | 16.1512 | 16.2366 | 16.0998 | 16.3999 | 16.373 | 16.3396 | 16.2391 | 14 | 13 | 12 | 12 | 11 | 12 | 13 | 15 |
| PRDX4 | Q13162 | 3.01E-09 | 1.17E-09 | -0.0812176 | | 0.208804 | -0.52517 | | 19.4291 | 19.5048 | 19.5315 | 19.3376 | 19.4187 | 19.928 | 19.7524 | 19.4118 | 13 | 14 | 13 | 13 | 12 | 12 | 14 | 13 |
| IMPA1 | P29218;P2 | 3.08E-10 | 1.36E-10 | -0.081213 | | 0.459881 | -1.02057 | | 17.0044 | 16.9624 | 17.0066 | 17.1976 | 17.1269 | 16.8481 | 17.1006 | 17.0885 | 13 | 14 | 13 | 14 | 13 | 13 | 13 | 13 |
| TUBGCP6 | Q96RT7;Q9 | 4.45E-10 | 1.92E-10 | -0.081074 | | 0.341218 | -0.79703 | | 14.2923 | 14.3234 | 14.1537 | 14.2306 | 14.3783 | 14.1287 | 14.528 | 14.422 | 3 | 5 | 4 | 3 | 3 | 3 | 5 | 5 |
| TRMT61A | Q96FX7 | 2.10E-09 | 8.30E-10 | -0.0810694 | | 0.383296 | -0.878 | | 15.089 | 15.0325 | 14.8489 | 15.031 | 15.2153 | 15.0654 | 14.8858 | 15.1583 | 2 | 3 | 3 | 4 | 3 | 2 | 2 | 3 |
| CHCHD3 | Q9NX63 | 1.62E-13 | 1.45E-13 | -0.0810382 | | 1.04958 | -2.02556 | | 17.0913 | 17.0562 | 17.0693 | 17.0938 | 17.2439 | 17.1274 | 17.2005 | 17.101 | 9 | 9 | 9 | 9 | 9 | 9 | 9 | 9 |
| POGK | Q9P215 | 8.41E-09 | 3.09E-09 | -0.0809135 | | 0.021759 | -0.06522 | | 13.2171 | 13.051 | 12.8258 | 12.4036 | NaN | 11.4164 | 15.5166 | NaN | 2 | 1 | 2 | 3 | NaN | 3 | 1 | NaN |
| CDC42EP4 | Q9H3Q1 | 2.99E-15 | 4.44E-15 | -0.0809071 | | 0.172585 | -0.44478 | | 15.5189 | 15.5848 | 15.5266 | 16.0374 | 15.7244 | 15.4376 | 15.2365 | 15.6859 | 9 | 9 | 10 | 9 | 10 | 7 | 8 | 9 |
| RPL35A | P18077 | 2.09E-05 | 5.31E-06 | -0.0808596 | | 0.653065 | -1.36138 | | 19.9024 | 19.9198 | 19.8134 | 19.853 | 19.9352 | 20.0378 | 19.9025 | 20.0683 | 9 | 9 | 9 | 9 | 9 | 9 | 9 | 9 |
| PSMD7 | P51665 | 1.09E-08 | 3.96E-09 | -0.0807789 | | 0.845752 | -1.68666 | | 18.3273 | 18.3601 | 18.2378 | 18.3559 | 18.4767 | 18.4375 | 18.3113 | 18.3643 | 13 | 13 | 13 | 13 | 13 | 12 | 13 | 13 |
| TRAPPC2L | Q9UL33;Q9 | 1.51E-06 | 4.30E-07 | -0.0807778 | | 0.276021 | -0.66689 | | 15.961 | 16.0579 | 15.9765 | 16.1634 | 15.9275 | 15.8233 | 16.3174 | 16.1646 | 4 | 4 | 4 | 4 | 4 | 4 | 4 | 4 |
| RDX | P35241;P3 | 6.39E-14 | 6.43E-14 | -0.0805575 | | 1.03477 | -2.00094 | | 19.8616 | 19.8774 | 19.8564 | 20.0092 | 19.9238 | 20.0181 | 19.8592 | 19.9181 | 44 | 43 | 43 | 44 | 42 | 44 | 43 | 42 |
| PRPSAP2 | O60256 | 6.55E-09 | 2.44E-09 | -0.0805397 | | 0.573104 | -1.22285 | | 16.7394 | 16.7116 | 16.6732 | 16.7215 | 16.9729 | 16.738 | 16.7144 | 16.7962 | 12 | 11 | 12 | 10 | 10 | 12 | 12 | 11 |
| SMARCD2 | Q92925;Q9 | 0.002302 | 0.000444 | -0.080507 | | 0.235228 | -0.58194 | | 15.5385 | 15.5897 | 15.6127 | 15.6718 | 15.8386 | 15.2637 | 15.7396 | 15.7903 | 6 | 6 | 5 | 6 | 4 | 5 | 5 | 4 |
| WRNIP1 | Q96S55;Q9 | 0.003078 | 0.000572 | -0.0805045 | | 0.3201 | -0.75555 | | 15.2738 | 15.4226 | 15.4324 | 15.2401 | 15.7075 | 15.4133 | 15.4704 | 15.4526 | 7 | 9 | 6 | 5 | 5 | 5 | 4 | 5 |
| DHX16 | O60231 | 9.98E-08 | 3.22E-08 | -0.0804232 | | 0.494132 | -1.08268 | | 16.4585 | 16.4969 | 16.482 | 16.5854 | 16.6187 | 16.3885 | 16.4925 | 16.7125 | 16 | 16 | 15 | 16 | 14 | 16 | 18 | 19 |
| ZNF281 | Q9Y2X9;Q9 | 0.0014 | 0.000283 | -0.0802025 | | 0.143008 | -0.38017 | | 12.8131 | 12.9195 | 12.9849 | 13.013 | NaN | 13.0129 | 13.3855 | 12.5328 | 3 | 3 | 3 | 2 | NaN | 2 | 3 | 3 |
| CAPN7 | Q9Y6W3 | 5.68E-06 | 1.53E-06 | -0.0801197 | | 0.236987 | -0.58567 | | 14.7344 | 14.7556 | 14.7452 | 14.7685 | 14.5837 | 15.0843 | 15.0458 | 14.6436 | 16 | 17 | 15 | 15 | 10 | 17 | 16 | 13 |
| CDC27 | P30260;P3 | 8.23E-10 | 3.44E-10 | -0.0800204 | | 0.648283 | -1.35317 | | 15.4888 | 15.4473 | 15.4159 | 15.406 | 15.6165 | 15.4634 | 15.5413 | 15.6263 | 12 | 12 | 12 | 13 | 11 | 11 | 12 | 11 |
| YTHDF3 | Q7Z739 | 4.57E-13 | 3.61E-13 | -0.0798506 | | 0.651089 | -1.35799 | | 16.5666 | 16.6085 | 16.5624 | 16.829 | 16.6244 | 16.6368 | 16.5851 | 16.6198 | 14 | 14 | 14 | 13 | 11 | 13 | 12 | 13 |
| WDTC1 | Q8N5D0;Q | 0.003319 | 0.000612 | -0.0796948 | | 0.427022 | -0.96008 | | 14.7225 | 14.7492 | 15.0231 | 14.9556 | 14.9224 | 14.8217 | 15.004 | 14.8526 | 3 | 2 | 3 | 3 | 3 | 3 | 3 | 4 |
| PLS3 | P13797 | 9.79E-12 | 5.70E-12 | -0.0796542 | | 0.100116 | -0.27285 | | 19.2888 | 19.2958 | 19.2995 | 19.9236 | 18.8715 | 19.8267 | 18.9312 | 19.3188 | 46 | 47 | 49 | 47 | 43 | 47 | 43 | 45 |
| NSMCE1 | Q8WV22 | 0.00113 | 0.000232 | -0.0796314 | | 0.26386 | -0.64188 | | 14.6953 | 14.9425 | 14.764 | 14.9333 | 14.7398 | 14.6705 | 14.9064 | 15.1511 | 3 | 3 | 3 | 3 | 2 | 1 | 2 | 2 |
| OXA1L | Q15070;Q1 | 1.38E-14 | 1.68E-14 | -0.079598 | | 0.255142 | -0.62379 | | 15.3776 | 15.5025 | 15.4205 | 15.7141 | 15.4903 | 15.1667 | 15.5939 | 15.6006 | 8 | 9 | 9 | 10 | 8 | 8 | 8 | 8 |
| RPS3 | P23396 | 1.51E-09 | 6.10E-10 | -0.079435 | + | 1.53009 | -2.84156 | | 21.2386 | 21.2153 | 21.179 | 21.2224 | 21.3373 | 21.2947 | 21.3001 | 21.2973 | 23 | 23 | 23 | 24 | 23 | 23 | 23 | 23 |
| LIMS1 | P48059-3 | 1.06E-12 | 7.63E-13 | -0.0792853 | | 0.15579 | -0.40637 | | 17.0879 | 17.1865 | 17.1421 | 16.9282 | 17.0531 | 17.7701 | 17.1329 | 17.2063 | 17 | 17 | 17 | 16 | 17 | 17 | 16 | 17 |
| PDIA5 | Q14554 | 1.78E-14 | 2.11E-14 | -0.0792491 | | 0.206618 | -0.52041 | | 17.095 | 17.1637 | 17.0995 | 16.9431 | 17.1091 | 17.4636 | 17.4745 | 17.003 | 26 | 27 | 26 | 22 | 21 | 26 | 26 | 24 |
| SCAMP1 | O15126 | 1.79E-08 | 6.31E-09 | -0.079018 | | 0.401942 | -0.91324 | | 16.5617 | 16.5148 | 16.6692 | 16.7303 | 16.6772 | 16.8381 | 16.5474 | 16.5115 | 5 | 6 | 5 | 5 | 4 | 5 | 5 | 5 |
| RAB10 | P61026 | 4.48E-13 | 3.56E-13 | -0.0786208 | | 0.6015 | -1.27238 | | 18.9894 | 19.0069 | 19.0231 | 19.1893 | 19.1536 | 19.1275 | 19.0083 | 18.9468 | 12 | 12 | 12 | 12 | 12 | 12 | 12 | 12 |
| ACO1 | P21399 | 4.76E-12 | 2.93E-12 | -0.0785899 | | 0.379183 | -0.87017 | | 17.4534 | 17.5257 | 17.4729 | 17.7977 | 17.5465 | 17.5557 | 17.3827 | 17.5301 | 35 | 34 | 34 | 34 | 34 | 36 | 35 | 34 |
| EFL1 | Q7Z2Z2 | 2.97E-11 | 1.58E-11 | -0.0785815 | | 0.466334 | -1.03234 | | 16.1634 | 16.2223 | 16.2743 | 16.5119 | 16.2568 | 16.2772 | 16.229 | 16.2181 | 28 | 27 | 27 | 25 | 23 | 25 | 25 | 22 |
| RAB18 | Q9NP72 | 0.000422 | 9.18E-05 | -0.0785742 | | 0.24135 | -0.59489 | | 19.0677 | 19.0994 | 19.1103 | 19.5462 | 19.0655 | 18.9794 | 19.0892 | 19.175 | 10 | 10 | 10 | 10 | 9 | 10 | 9 | 10 |
| CRLF3 | Q8IUI8 | 1.65E-13 | 1.48E-13 | -0.0785585 | | 0.179201 | -0.45971 | | 15.6956 | 15.6488 | 15.6972 | 16.0326 | 15.3212 | 15.9598 | 15.6422 | 15.8397 | 4 | 4 | 4 | 4 | 4 | 4 | 6 | 5 |
| SAR1A | Q9NR31 | 1.01E-11 | 5.88E-12 | -0.0784358 | | 0.479601 | -1.05644 | | 18.8681 | 18.7798 | 18.756 | 19.0677 | 18.7857 | 18.8096 | 18.8146 | 18.9211 | 7 | 7 | 7 | 7 | 7 | 7 | 7 | 7 |
| PPIL2 | Q13356 | 0.001014 | 0.000209 | -0.0782187 | | 0.170896 | -0.44096 | | 15.4541 | 15.4536 | 15.4088 | 15.8266 | 15.4485 | 15.0428 | 15.6356 | 15.6318 | 1 | 3 | 4 | 2 | 1 | 1 | 2 | 1 |
| ACO2 | Q99798 | 1.09E-11 | 6.27E-12 | -0.0782092 | + | 1.77665 | -3.2847 | | 17.459 | 17.4417 | 17.4414 | 17.5562 | 17.544 | 17.4626 | 17.5121 | 17.553 | 37 | 38 | 36 | 38 | 37 | 37 | 39 | 38 |
| FNTB | P49356 | 0.000142 | 3.29E-05 | -0.0781085 | | 0.208952 | -0.52549 | | 15.7963 | 15.8163 | 15.8372 | 15.5217 | 15.8957 | 15.969 | 16.215 | 15.872 | 5 | 5 | 6 | 6 | 5 | 5 | 5 | 5 |
| VRK1 | Q99986 | 1.26E-13 | 1.16E-13 | -0.0779747 | | 0.163987 | -0.42521 | | 15.27 | 15.1747 | 15.256 | 15.4614 | 15.1942 | 14.8656 | 15.3564 | 15.6802 | 6 | 8 | 6 | 6 | 6 | 7 | 7 | 6 |
| MYCBP2 | O75592;O7 | 1.53E-18 | 6.34E-18 | -0.0778529 | | 0.700683 | -1.44267 | | 14.5371 | 14.5137 | 14.5643 | 14.6529 | 14.6305 | 14.6556 | 14.6813 | 14.4608 | 42 | 40 | 40 | 39 | 26 | 34 | 35 | 29 |
| URB1 | O60287 | 0.000175 | 4.01E-05 | -0.0778177 | | 0.229702 | -0.57019 | | 14.8025 | 14.8925 | 15.0039 | 14.9392 | 14.6438 | 15.0169 | 15.2427 | 15.0447 | 6 | 7 | 9 | 8 | 9 | 7 | 10 | 8 |
| ANPEP | P15144 | 5.41E-08 | 1.80E-08 | -0.0776047 | | 0.126525 | -0.33746 | | 20.7344 | 20.7367 | 20.6931 | 20.196 | 20.718 | 21.0525 | 21.1969 | 20.8315 | 45 | 43 | 44 | 44 | 45 | 44 | 46 | 46 |
| BIRC2 | Q13490;Q1 | 0.000421 | 9.16E-05 | -0.0775968 | | 0.277983 | -0.6709 | | 14.7557 | 14.7568 | 14.6102 | 14.6527 | 15.0861 | 14.6366 | 14.7207 | 14.8294 | 5 | 5 | 6 | 4 | 5 | 4 | 4 | 4 |
| TNS1 | Q9HBL0 | 1.16E-16 | 2.70E-16 | -0.0775164 | | 0.123101 | -0.32922 | | 16.396 | 16.3658 | 16.3921 | 16.5638 | 16.16 | 17.0873 | 16.1075 | 16.3922 | 51 | 50 | 47 | 51 | 47 | 55 | 50 | 53 |
| SEPTIN2 | Q15019;Q1 | 1.25E-10 | 5.91E-11 | -0.077242 | | 0.654944 | -1.3646 | | 20.1986 | 20.2042 | 20.1771 | 20.1649 | 20.4212 | 20.2821 | 20.2468 | 20.2377 | 22 | 22 | 23 | 22 | 22 | 22 | 21 | 23 |
| BZW1 | Q7L1Q6;Q7 | 6.33E-13 | 4.81E-13 | -0.0770994 | | 0.337542 | -0.78985 | | 18.2317 | 18.2989 | 18.2429 | 18.6203 | 18.2226 | 18.2861 | 18.2879 | 18.2576 | 19 | 19 | 19 | 19 | 18 | 19 | 16 | 18 |
| TPD52L2 | O43399;O4 | 2.78E-13 | 2.34E-13 | -0.076909 | | 0.103188 | -0.28049 | | 19.1417 | 19.0776 | 19.2782 | 19.8066 | 18.9461 | 18.6446 | 19.3518 | 19.4647 | 14 | 14 | 13 | 13 | 13 | 13 | 14 | 13 |
| FYTTD1 | Q96QD9 | 1.47E-06 | 4.19E-07 | -0.0768915 | | 0.517385 | -1.12436 | | 16.2483 | 16.2245 | 16.1557 | 16.1004 | 16.3704 | 16.2937 | 16.3638 | 16.3037 | 9 | 9 | 8 | 9 | 7 | 9 | 9 | 9 |
| UBA6 | A0AVT1 | 1.63E-09 | 6.53E-10 | -0.0767643 | | 0.638417 | -1.33621 | | 16.8975 | 16.9079 | 16.867 | 17.0244 | 16.9341 | 16.8923 | 17.105 | 16.8821 | 40 | 39 | 40 | 37 | 36 | 39 | 39 | 37 |
| P4HA1 | P13674 | 6.36E-18 | 2.20E-17 | -0.0767508 | | 0.150769 | -0.39473 | | 19.0078 | 19.0226 | 19.0075 | 18.7033 | 18.9907 | 19.3597 | 19.4885 | 18.9048 | 30 | 29 | 30 | 29 | 29 | 30 | 30 | 29 |
| WDR7 | Q9Y4E6;Q9 | 1.00E-14 | 1.27E-14 | -0.0766866 | | 0.300414 | -0.71632 | | 14.5839 | 14.7393 | 14.6233 | 14.6492 | 14.6619 | 14.6402 | 15.0297 | 14.6465 | 10 | 6 | 9 | 8 | 6 | 8 | 10 | 7 |
| PYROXD1 | Q8WU10;C | 0.000297 | 6.59E-05 | -0.0765815 | | 0.055171 | -0.15991 | | NaN | 13.508 | 13.636 | 13.934 | 14.1354 | NaN | 12.7155 | 13.8096 | NaN | 1 | 2 | 5 | 2 | NaN | 2 | 1 |
| MACO1 | Q8N5G2 | 1.15E-06 | 3.33E-07 | -0.0764673 | | 0.111782 | -0.30169 | | 14.1805 | 13.5603 | 14.4692 | 14.0085 | 14.5919 | 13.8795 | 14.0967 | 14.1557 | 2 | 3 | 1 | 3 | 2 | 2 | 3 | 3 |
| PPP1R13L | Q8WUF5 | 3.38E-12 | 2.15E-12 | -0.0764394 | | 0.114131 | -0.30744 | | 15.6315 | 15.6378 | 15.646 | 15.7593 | 15.4604 | 16.4238 | 15.4931 | 15.4377 | 13 | 12 | 14 | 13 | 4 | 15 | 7 | 12 |
| VPS45 | Q9NRW7 | 6.39E-07 | 1.90E-07 | -0.0764033 | | 0.74657 | -1.52034 | | 15.8244 | 15.8651 | 15.8148 | 15.8242 | 15.8855 | 16.0246 | 15.9651 | 15.8565 | 20 | 18 | 20 | 20 | 16 | 20 | 18 | 16 |
| NOLC1 | Q14978;Q1 | 9.95E-07 | 2.89E-07 | -0.0762813 | | 0.299935 | -0.71536 | | 15.7402 | 15.5674 | 15.6082 | 15.803 | 15.4287 | 15.7345 | 15.7508 | 15.8573 | 11 | 12 | 12 | 10 | 10 | 12 | 12 | 12 |
| ACY1 | Q03154 | 0.000191 | 4.35E-05 | -0.0762408 | | 0.133548 | -0.35424 | | 16.0347 | 16.1404 | 16.0702 | 15.9189 | 15.8168 | 16.6875 | 16.3567 | 16.0101 | 14 | 13 | 14 | 12 | 10 | 14 | 12 | 13 |
| ADSL | P30566 | 2.67E-13 | 2.26E-13 | -0.0760223 | | 0.412502 | -0.93303 | | 17.2122 | 17.3193 | 17.2739 | 17.26 | 17.4755 | 17.4988 | 17.2262 | 17.2621 | 18 | 18 | 18 | 18 | 18 | 18 | 18 | 18 |
| VDAC1 | P21796 | 2.03E-07 | 6.39E-08 | -0.0760208 | | 0.499454 | -1.09226 | | 20.3437 | 20.3619 | 20.3453 | 20.3312 | 20.5606 | 20.5019 | 20.4555 | 20.2826 | 14 | 15 | 16 | 15 | 15 | 15 | 15 | 15 |
| TIMM21 | Q9BVV7 | 4.30E-12 | 2.68E-12 | -0.07547 | | 0.346112 | -0.80656 | | 14.8706 | 14.8277 | 14.8898 | 15.007 | 14.6849 | 15.0714 | 15.0291 | 14.8983 | 3 | 3 | 3 | 3 | 3 | 4 | 3 | 3 |
| CUX1 | P39880-3 | 0.000672 | 0.000142 | -0.0754455 | | 0.189861 | -0.48353 | | 14.8881 | 15.0086 | 14.8759 | 15.0346 | 15.1925 | 14.847 | 15.2771 | 14.6469 | 11 | 13 | 12 | 12 | 9 | 12 | 14 | 11 |
| EDC3 | Q96F86 | 6.81E-14 | 6.81E-14 | -0.0753932 | | 0.324114 | -0.76348 | | 15.8056 | 15.8029 | 15.794 | 16.0935 | 15.8846 | 15.6362 | 15.8353 | 15.9316 | 11 | 13 | 11 | 14 | 11 | 10 | 11 | 10 |
| CUL7 | Q14999 | 2.25E-14 | 2.58E-14 | -0.0750515 | | 1.18338 | -2.24871 | | 15.7946 | 15.8498 | 15.8149 | 15.8419 | 15.8637 | 15.936 | 15.9641 | 15.8684 | 25 | 25 | 24 | 23 | 18 | 24 | 24 | 24 |
| NR2F2 | P24468 | 0.000229 | 5.16E-05 | -0.0747777 | | 0.215319 | -0.53931 | | 14.9149 | 15.2401 | 15.0706 | 15.0126 | 14.9908 | 15.0272 | 15.4497 | 15.2695 | 8 | 5 | 7 | 2 | 4 | 7 | 6 | 7 |
| MRPL48 | Q96GC5;Q9 | 7.92E-06 | 2.10E-06 | -0.0746424 | | 0.407704 | -0.92405 | | 15.6207 | 15.6461 | 15.8475 | 15.7941 | 15.826 | 15.6293 | 15.9064 | 15.7413 | 3 | 2 | 3 | 3 | 3 | 3 | 2 | 3 |
| GIT2 | Q14161 | 5.08E-16 | 9.29E-16 | -0.0746298 | | 0.565705 | -1.20987 | | 15.5297 | 15.5717 | 15.5265 | 15.6025 | 15.7939 | 15.5628 | 15.5902 | 15.5371 | 17 | 16 | 17 | 17 | 15 | 15 | 17 | 15 |
| PEX10 | O60683;O6 | 0.004439 | 0.0008 | -0.0746166 | | 0.114607 | -0.31141 | | 15.8559 | 16.0444 | NaN | 15.7112 | 16.3411 | 16.3831 | 15.8299 | 15.8586 | 3 | 2 | NaN | 1 | 1 | 2 | 1 | 2 |
| CHST14 | Q8NCH0 | 6.51E-08 | 2.14E-08 | -0.0745831 | | 0.511867 | -1.1145 | | 16.0132 | 16.0228 | 15.9987 | 16.0249 | 16.0033 | 16.0327 | 16.2774 | 16.0925 | 10 | 9 | 10 | 8 | 10 | 10 | 10 | 9 |
| SMYD5 | Q6GMV2 | 1.45E-05 | 3.74E-06 | -0.0744877 | | 0.218435 | -0.54603 | | 15.8569 | 15.8617 | 15.8585 | 15.9844 | 16.0216 | 16.1571 | 15.5482 | 15.9565 | 5 | 5 | 5 | 5 | 4 | 5 | 5 | 5 |
| EFR3A | Q14156 | 2.94E-14 | 3.25E-14 | -0.0744819 | | 0.260494 | -0.63491 | | 15.5046 | 15.4471 | 15.4216 | 15.2773 | 15.5752 | 15.8078 | 15.4438 | 15.5571 | 20 | 18 | 19 | 18 | 14 | 21 | 16 | 15 |
| CNDP2 | Q96KP4 | 1.59E-06 | 4.53E-07 | -0.0744255 | | 0.280061 | -0.67514 | | 18.3374 | 18.3996 | 18.3421 | 18.568 | 18.2347 | 18.66 | 18.4338 | 18.2741 | 19 | 18 | 19 | 20 | 18 | 20 | 18 | 19 |
| ENDOD1 | O94919 | 9.25E-11 | 4.46E-11 | -0.0741194 | | 0.17899 | -0.45924 | | 16.7676 | 16.7352 | 16.6921 | 17.1491 | 17.0375 | 16.5877 | 16.5666 | 16.6878 | 10 | 10 | 11 | 11 | 9 | 10 | 10 | 10 |
| DDOST | P39656 | 2.39E-08 | 8.28E-09 | -0.0740739 | | 0.549051 | -1.18055 | | 20.5046 | 20.5504 | 20.6022 | 20.5283 | 20.6721 | 20.7309 | 20.6886 | 20.5123 | 16 | 16 | 16 | 16 | 16 | 16 | 16 | 16 |

| Gene names | UniProt IDs | Protein Group P-value | Protein Group Q-value | Log2 Difference NUP214 vs Controls (n=5) | p<0.05 | -log10 p-value | T-test Test statistic | NPC Localization | Log2 MS2_quantity_ NUP214_1 | Log2 MS2_quantity_ NUP214_2 | Log2 MS2_quantity_ NUP214_3 | Log2 MS2_quantity_ Control_1 | Log2 MS2_quantity_ Control_2 | Log2 MS2_quantity_ Control_3 | Log2 MS2_quantity_ Control_4 | Log2 MS2_quantity_ Control_5 | Number Peptides for Quantitation NUP214_1 | Number Peptides for Quantitation NUP214_2 | Number Peptides for Quantitation NUP214_3 | Number Peptides for Quantitation Control_1 | Number Peptides for Quantitation Control_2 | Number Peptides for Quantitation Control_3 | Number Peptides for Quantitation Control_4 | Number Peptides for Quantitation Control_5 |
|---|---|---|---|---|---|---|---|---|---|---|---|---|---|---|---|---|---|---|---|---|---|---|---|---|
| FNBP4 | Q8N3X1;Q8 | 0.000129 | 3.00E-05 | -0.0734193 | | 0.266698 | -0.64774 | | 14.5017 | 14.5782 | 14.7502 | 14.7602 | 14.3997 | 14.7745 | 14.8146 | 14.6682 | 5 | 6 | 3 | 2 | 1 | 3 | 3 | 3 |
| GATB | O75879 | 0.001895 | 0.000373 | -0.073355 | | 0.317676 | -0.75075 | | 13.5117 | 13.4532 | 13.4098 | 13.6679 | 13.7222 | 13.3345 | 13.465 | 13.4682 | 2 | 2 | 1 | 1 | 1 | 2 | 2 | 1 |
| MAP3K7 | O43318;O4 | 7.94E-12 | 4.71E-12 | -0.0732697 | | 0.760101 | -1.54315 | | 14.3028 | 14.4249 | 14.4345 | 14.4573 | 14.5596 | 14.4087 | 14.4149 | 14.4629 | 7 | 9 | 9 | 9 | 7 | 9 | 8 | 10 |
| HYOU1 | Q9Y4L1 | 1.31E-20 | 1.14E-19 | -0.0730048 | | 0.303147 | -0.7218 | | 18.4942 | 18.5308 | 18.5352 | 18.6372 | 18.3447 | 18.629 | 18.8094 | 18.545 | 34 | 35 | 35 | 34 | 36 | 36 | 36 | 35 |
| YTHDC1 | Q96MU7;Q | 2.04E-06 | 5.73E-07 | -0.0729565 | | 0.342753 | -0.80002 | | 15.5257 | 15.4558 | 15.6071 | 15.4348 | 15.5802 | 15.6948 | 15.7931 | 15.5095 | 8 | 8 | 7 | 7 | 4 | 5 | 6 | 5 |
| GOLGA5 | Q8TBA6 | 2.62E-23 | 5.17E-22 | -0.0729012 | | 0.464095 | -1.02826 | | 15.5279 | 15.5311 | 15.5778 | 15.5101 | 15.5588 | 15.7611 | 15.7288 | 15.5337 | 23 | 24 | 24 | 23 | 20 | 23 | 21 | 21 |
| MYH10 | P35580;P3 | 2.57E-21 | 2.73E-20 | -0.0728241 | | 0.133274 | -0.35359 | | 18.4479 | 18.437 | 18.4469 | 18.9854 | 18.2086 | 18.7826 | 18.3261 | 18.281 | 137 | 135 | 137 | 140 | 121 | 135 | 133 | 139 |
| SH3GLB1 | Q9Y371;Q9 | 8.21E-09 | 3.02E-09 | -0.0727861 | | 0.566448 | -1.21117 | | 17.219 | 17.2477 | 17.205 | 17.1981 | 17.3688 | 17.1943 | 17.4158 | 17.3063 | 13 | 13 | 13 | 13 | 13 | 12 | 13 | 13 |
| ALG12 | Q9BV10 | 1.36E-08 | 4.85E-09 | -0.0727367 | | 0.143153 | -0.37693 | | 15.8159 | 15.6613 | 15.7502 | 15.7806 | 15.6479 | 16.2184 | 16.0268 | 15.4024 | 3 | 2 | 3 | 3 | 3 | 2 | 3 | 2 |
| GTF2H4 | Q92759 | 0.002249 | 0.000436 | -0.0726433 | | 0.742644 | -1.51372 | | 15.3027 | 15.2474 | 15.3327 | 15.3424 | 15.2734 | 15.341 | 15.4687 | 15.409 | 4 | 5 | 3 | 4 | 3 | 4 | 5 | 3 |
| USP5 | P45974;P4 | 3.72E-11 | 1.93E-11 | -0.0726391 | + | 1.89311 | -3.50229 | | 18.8452 | 18.8044 | 18.8428 | 18.9279 | 18.8745 | 18.8843 | 18.9445 | 18.8859 | 24 | 25 | 23 | 24 | 25 | 23 | 25 | 23 |
| SLC25A6 | P12236 | 1.23E-07 | 3.94E-08 | -0.0725154 | | 1.18577 | -2.25272 | | 20.9405 | 20.97 | 20.886 | 21.0349 | 21.0683 | 20.9643 | 20.9798 | 20.9761 | 16 | 16 | 16 | 16 | 14 | 17 | 16 | 16 |
| CDC42 | P60953 | 1.66E-09 | 6.64E-10 | -0.0723639 | | 0.431735 | -0.96881 | | 20.4902 | 20.599 | 20.5166 | 20.5561 | 20.7822 | 20.4918 | 20.6738 | 20.5342 | 7 | 7 | 7 | 7 | 8 | 7 | 7 | 7 |
| PTGR1 | Q14914;Q1 | 6.56E-13 | 4.96E-13 | -0.0722664 | | 0.412204 | -0.93248 | | 18.2926 | 18.2961 | 18.3058 | 18.2602 | 18.4532 | 18.2056 | 18.5043 | 18.4288 | 16 | 15 | 15 | 14 | 15 | 14 | 14 | 15 |
| POLR3C | Q9BUI4 | 9.71E-05 | 2.30E-05 | -0.0720354 | | 0.204098 | -0.51491 | | 14.7005 | 14.8189 | 14.8198 | 14.6067 | 15.1759 | 14.6603 | 14.9446 | 14.8713 | 3 | 2 | 4 | 5 | 2 | 3 | 2 | 5 |
| MYO18A | Q92614;Q9 | 2.42E-14 | 2.73E-14 | -0.0716725 | | 0.340923 | -0.79645 | | 16.002 | 15.9984 | 16.0153 | 16.1094 | 16.2709 | 15.8593 | 16.1217 | 16.0232 | 46 | 44 | 47 | 49 | 40 | 42 | 44 | 46 |
| IBA57 | Q5T440 | 0.000169 | 3.89E-05 | -0.0715706 | | 0.388661 | -0.88817 | | 15.2934 | 15.3691 | 15.2984 | 15.2367 | 15.3553 | 15.3828 | 15.6014 | 15.3831 | 5 | 4 | 4 | 2 | 2 | 3 | 2 | 3 |
| LTA4H | P09960 | 6.96E-13 | 5.22E-13 | -0.0715074 | | 0.307542 | -0.73059 | | 17.864 | 17.86 | 17.8629 | 17.8248 | 17.9199 | 18.119 | 18.0751 | 17.7303 | 27 | 27 | 27 | 27 | 26 | 27 | 27 | 27 |
| LRRC47 | Q8N1G4 | 2.77E-12 | 1.80E-12 | -0.0712427 | | 1.01108 | -1.96158 | | 17.7333 | 17.6846 | 17.6988 | 17.7074 | 17.7755 | 17.8032 | 17.8585 | 17.7395 | 22 | 24 | 22 | 22 | 22 | 21 | 23 | 22 |
| PIGS | Q96S52 | 4.36E-13 | 3.48E-13 | -0.0712035 | + | 1.45971 | -2.71883 | | 16.5649 | 16.6026 | 16.5856 | 16.6481 | 16.6682 | 16.5956 | 16.6537 | 16.7121 | 11 | 12 | 12 | 12 | 13 | 12 | 12 | 12 |
| TKT | P29401;P2 | 7.59E-21 | 7.15E-20 | -0.0709958 | | 0.301348 | -0.7182 | | 20.0209 | 20.0481 | 20.0179 | 20.1502 | 20.3549 | 19.9399 | 19.9738 | 20.081 | 35 | 35 | 35 | 35 | 36 | 36 | 35 | 35 |
| CCDC93 | Q567U6 | 5.61E-17 | 1.42E-16 | -0.0709283 | | 0.566972 | -1.21209 | | 15.8713 | 15.8893 | 15.8635 | 15.8896 | 15.8814 | 16.0263 | 16.0741 | 15.8568 | 27 | 28 | 24 | 26 | 22 | 27 | 25 | 25 |
| GTPBP10 | A4D1E9 | 2.79E-05 | 7.01E-06 | -0.0707632 | | 0.128736 | -0.34276 | | 14.2394 | 14.3337 | 14.3114 | 14.743 | 14.4172 | 13.82 | 14.3118 | 14.5359 | 5 | 7 | 5 | 5 | 3 | 3 | 6 | 7 |
| EXOSC3 | Q9NQT5 | 2.83E-07 | 8.76E-08 | -0.0707542 | | 0.195808 | -0.49669 | | 15.0314 | 15.3647 | 15.4811 | 15.6439 | 15.2423 | 15.2438 | 15.4165 | 15.2692 | 4 | 6 | 6 | 4 | 3 | 4 | 2 | 6 |
| GNPTAB | Q3T906 | 0.000849 | 0.000177 | -0.070668 | | 0.209334 | -0.52633 | | 14.8772 | 14.7406 | 14.8029 | 14.7569 | 15.2214 | 14.8985 | 14.6322 | 14.8787 | 2 | 2 | 2 | 1 | 2 | 2 | 2 | 1 |
| TTI2 | Q6NXR4 | 8.65E-07 | 2.53E-07 | -0.0706121 | | 0.240211 | -0.59248 | | 14.2244 | 14.2318 | 14.163 | 14.493 | 14.3471 | 14.1107 | 14.0293 | 14.405 | 5 | 6 | 6 | 5 | 3 | 5 | 4 | 5 |
| SNX29 | Q8TEQ0;Q8 | 7.09E-05 | 1.71E-05 | -0.0704192 | | 0.114269 | -0.30778 | | 15.8518 | 15.9668 | 15.8104 | 16.4373 | 16.1548 | 15.7684 | 15.4368 | 15.9366 | 13 | 15 | 14 | 12 | 10 | 13 | 9 | 11 |
| TXNDC5 | Q8NBS9 | 4.92E-06 | 1.33E-06 | -0.0703506 | | 0.363281 | -0.83975 | | 20.3014 | 20.3478 | 20.3229 | 20.333 | 20.4358 | 20.5359 | 20.4835 | 20.1836 | 23 | 23 | 22 | 23 | 21 | 23 | 23 | 23 |
| ME3 | Q16798 | 0.003102 | 0.000576 | -0.0702794 | | 0.136582 | -0.36483 | | 15.1756 | 15.1271 | 15.09 | 15.2497 | NaN | 15.6405 | 14.9593 | 14.9553 | 3 | 3 | 4 | 1 | NaN | 3 | 3 | 2 |
| GSTM2 | P28161 | 3.44E-09 | 1.33E-09 | -0.0702131 | | 0.173296 | -0.4464 | | 18.2287 | 18.2115 | 18.2442 | 17.8988 | 18.2325 | 18.5741 | 18.4927 | 18.2938 | 10 | 10 | 10 | 11 | 11 | 10 | 11 | 11 |
| MVB12A | Q96EY5 | 2.73E-12 | 1.78E-12 | -0.0699231 | | 0.25391 | -0.62122 | | 16.0355 | 15.9978 | 16.0614 | 16.0153 | 16.4132 | 15.9423 | 16.0037 | 16.133 | 8 | 8 | 8 | 7 | 6 | 7 | 8 | 7 |
| AGPAT1 | Q99943 | 0.004426 | 0.000798 | -0.0698804 | | 0.159329 | -0.41453 | | 16.2284 | 16.3434 | 16.4546 | 16.5529 | 16.0076 | 16.6519 | 16.5864 | 16.261 | 2 | 2 | 2 | 2 | 2 | 3 | 2 | 2 |
| SYPL1 | Q16563-2 | 1.15E-11 | 6.58E-12 | -0.0697116 | | 0.211726 | -0.53152 | | 19.4201 | 19.3523 | 19.5134 | 19.1445 | 19.6788 | 19.4834 | 19.6411 | 19.5439 | 6 | 7 | 6 | 7 | 7 | 7 | 7 | 7 |
| TMEM109 | Q9BVC6 | 0.00013 | 3.03E-05 | -0.0696362 | | 0.214885 | -0.53837 | | 19.8308 | 19.8014 | 19.7268 | 19.6136 | 20.0553 | 19.6782 | 20.0847 | 19.8482 | 4 | 4 | 4 | 4 | 4 | 4 | 4 | 4 |
| MRPS28 | Q9Y2Q9 | 1.11E-17 | 3.49E-17 | -0.0696285 | | 0.297886 | -0.71124 | | 16.2455 | 16.3676 | 16.3416 | 16.4108 | 16.5728 | 16.1718 | 16.2968 | 16.4871 | 6 | 5 | 6 | 6 | 3 | 5 | 5 | 4 |
| GCLM | P48507 | 7.73E-11 | 3.78E-11 | -0.069578 | | 0.366524 | -0.84598 | | 16.6007 | 16.6886 | 16.6027 | 16.8397 | 16.5348 | 16.817 | 16.5977 | 16.7119 | 8 | 9 | 9 | 9 | 7 | 8 | 7 | 9 |
| P4HTM | Q9NXG6;Q | 6.00E-05 | 1.46E-05 | -0.0694506 | | 0.155912 | -0.40665 | | 16.5544 | 16.5259 | 16.5189 | 16.274 | 16.7329 | 16.5093 | 17.0233 | 16.473 | 6 | 7 | 8 | 6 | 5 | 7 | 6 | 7 |
| MT-CO3 | P00414 | 5.13E-06 | 1.38E-06 | -0.06936 | | 0.165076 | -0.4277 | | 13.7276 | 13.4445 | 13.6407 | 13.7837 | 13.3518 | 14.0261 | 13.6477 | 13.5588 | 2 | 2 | 2 | 2 | 2 | 2 | 2 | 2 |
| FKBP1A | P62942 | 4.07E-15 | 5.82E-15 | -0.0693395 | | 0.108602 | -0.29388 | | 20.6673 | 20.6785 | 20.7713 | 21.1043 | 20.211 | 21.1369 | 20.5469 | 20.8761 | 5 | 5 | 5 | 5 | 5 | 5 | 5 | 5 |
| TMEM259 | Q4ZIN3;Q4 | 2.13E-07 | 6.67E-08 | -0.0692718 | | 0.201878 | -0.51004 | | 14.2916 | 14.1954 | 14.3698 | 14.5472 | 14.1867 | 14.1219 | 14.6188 | 14.2997 | 5 | 5 | 5 | 5 | 3 | 4 | 5 | 4 |
| RNF7 | Q9UBF6 | 8.67E-07 | 2.54E-07 | -0.0689527 | | 0.296412 | -0.70828 | | 16.9999 | 16.8307 | 16.7438 | 17.0696 | 16.7782 | 16.9574 | 17.0357 | 16.7947 | 2 | 2 | 2 | 2 | 2 | 2 | 2 | 2 |
| BET1L | Q9NYM9 | 6.35E-17 | 1.59E-16 | -0.0689396 | | 0.118961 | -0.3192 | | 15.3934 | 15.5297 | 15.7872 | 15.396 | 15.7119 | 15.2431 | 15.7442 | 16.1001 | 3 | 3 | 4 | 3 | 2 | 3 | 3 | 4 |
| ZCCHC8 | Q6NZY4 | 4.06E-12 | 2.54E-12 | -0.0686635 | | 0.301168 | -0.71784 | | 15.3459 | 15.1204 | 15.1832 | 15.2239 | 15.4415 | 15.2206 | 15.4166 | 15.1232 | 5 | 7 | 7 | 8 | 5 | 7 | 5 | 8 |
| WDR43 | Q15061 | 1.71E-07 | 5.43E-08 | -0.0686523 | | 0.219525 | -0.54838 | | 15.9117 | 16.0199 | 15.9294 | 16.1137 | 15.6706 | 16.0292 | 16.0971 | 16.2009 | 12 | 10 | 12 | 14 | 11 | 12 | 11 | 12 |
| NUDT3 | O95989 | 0.000833 | 0.000174 | -0.0685666 | | 0.261661 | -0.63733 | | 17.3582 | 17.3306 | 17.3836 | 17.4322 | 17.6517 | 17.2262 | 17.5468 | 17.2734 | 3 | 2 | 3 | 2 | 2 | 2 | 2 | 2 |
| MLKL | Q8NB16 | 7.19E-11 | 3.53E-11 | -0.0685612 | | 0.132964 | -0.35285 | | 14.6266 | 14.7101 | 14.667 | 15.1774 | 14.8485 | 14.5003 | 14.3453 | 14.8107 | 10 | 11 | 10 | 12 | 7 | 12 | 7 | 10 |
| RNF34 | Q969K3;Q9 | 1.89E-14 | 2.20E-14 | -0.0685455 | | 0.294776 | -0.70498 | | 14.4366 | 14.2838 | 14.4371 | 14.477 | 14.7073 | 14.3742 | 14.3764 | 14.3371 | 3 | 3 | 3 | 3 | 3 | 3 | 3 | 3 |
| ERCC3 | P19447 | 3.60E-10 | 1.58E-10 | -0.0684593 | | 0.417243 | -0.94189 | | 14.5984 | 14.6956 | 14.5793 | 14.616 | 14.8452 | 14.7243 | 14.7286 | 14.5505 | 12 | 9 | 10 | 8 | 7 | 6 | 7 | 6 |
| ASL | P04424;P0 | 4.02E-16 | 7.58E-16 | -0.0683403 | | 0.133212 | -0.35344 | | 17.1223 | 17.15 | 17.1502 | 17.238 | 17.0002 | 17.4134 | 16.7888 | 17.6056 | 17 | 17 | 17 | 19 | 17 | 18 | 18 | 18 |
| MAP4 | P27816;P2 | 5.78E-15 | 7.82E-15 | -0.0681423 | | 0.812604 | -1.63126 | | 18.9831 | 18.964 | 19.0348 | 19.0172 | 19.0126 | 19.1586 | 19.1008 | 19.0213 | 68 | 66 | 68 | 64 | 63 | 66 | 64 | 68 |
| WDR3 | Q9UNX4 | 3.57E-08 | 1.21E-08 | -0.0680661 | | 0.317915 | -0.75122 | | 15.0903 | 15.1107 | 15.1549 | 15.3579 | 14.9855 | 15.0888 | 15.2147 | 15.2868 | 17 | 21 | 18 | 25 | 15 | 19 | 17 | 21 |
| NPR2 | P20594 | 1.96E-09 | 7.78E-10 | -0.0679811 | | 0.272052 | -0.65876 | | 15.3237 | 15.2239 | 15.1749 | 15.0379 | 15.4684 | 15.3963 | 15.3482 | 15.2932 | 15 | 15 | 16 | 13 | 11 | 13 | 11 | 13 |
| TOE1 | Q96GM8 | 1.41E-08 | 5.03E-09 | -0.0679416 | | 0.410943 | -0.93012 | | 15.402 | 15.4083 | 15.3816 | 15.3155 | 15.5835 | 15.427 | 15.4017 | 15.5985 | 6 | 4 | 5 | 6 | 3 | 4 | 5 | 4 |
| ATP5F1A | P25705 | 1.61E-09 | 6.48E-10 | -0.0678525 | | 1.26581 | -2.38732 | | 20.8265 | 20.8696 | 20.7992 | 20.8708 | 20.96 | 20.895 | 20.8569 | 20.9155 | 29 | 29 | 29 | 29 | 28 | 29 | 29 | 29 |
| CIAO3 | Q9H6Q4;Q | 8.27E-12 | 4.89E-12 | -0.0678366 | | 0.236103 | -0.5838 | | 14.8652 | 14.8697 | 14.912 | 14.6798 | 15.1973 | 15.0535 | 14.9452 | 14.8748 | 5 | 6 | 6 | 6 | 3 | 5 | 6 | 5 |
| FAM92A | A1XBS5;A1 | 0.005773 | 0.00102 | -0.0676403 | | 0.142242 | -0.39281 | | 13.608 | 13.7813 | 13.5229 | NaN | NaN | NaN | 13.8948 | 13.5152 | 1 | 2 | 2 | NaN | NaN | NaN | 2 | 2 |
| ZNF787 | Q6DD87 | 0.000385 | 8.42E-05 | -0.0675542 | | 0.186577 | -0.47622 | | 13.7135 | 13.5175 | 13.2028 | 13.5783 | 13.6666 | 13.4557 | 13.6976 | 13.3293 | 1 | 4 | 3 | 2 | 2 | 2 | 3 | 3 |
| TULP3 | O75386;O7 | 0.001595 | 0.00032 | -0.0675374 | | 0.076205 | -0.21208 | | 13.7449 | 14.048 | 13.9753 | 14.2086 | 13.0975 | 14.0597 | 14.1222 | 14.4634 | 3 | 3 | 3 | 3 | 2 | 3 | 1 | 3 |
| OSBPL9 | Q96SU4;Q9 | 0.000114 | 2.67E-05 | -0.0674184 | | 0.394614 | -0.89943 | | 14.5425 | 14.5504 | 14.4944 | 14.648 | 14.7518 | 14.4877 | 14.6425 | 14.4527 | 10 | 12 | 10 | 10 | 9 | 8 | 10 | 8 |
| TIMM22 | Q9Y584 | 1.73E-06 | 4.89E-07 | -0.0673975 | | 0.219178 | -0.54764 | | 15.3887 | 15.3979 | 15.2736 | 15.2724 | 15.3535 | 15.7283 | 15.5081 | 15.2418 | 2 | 2 | 2 | 2 | 1 | 2 | 1 | 1 |
| DDX55 | Q8NHQ9 | 0.004167 | 0.000754 | -0.0673776 | | 0.118162 | -0.32016 | | 13.5645 | 13.6506 | 13.6172 | 13.771 | 13.5154 | NaN | 13.3019 | 14.1244 | 2 | 1 | 1 | 1 | 1 | NaN | 1 | 1 |
| RAP1B | P61224 | 2.96E-15 | 4.42E-15 | -0.0671402 | | 0.356067 | -0.82584 | | 20.452 | 20.489 | 20.3319 | 20.4548 | 20.3834 | 20.581 | 20.6574 | 20.3806 | 12 | 11 | 13 | 13 | 12 | 13 | 11 | 13 |
| SEPHS2 | Q99611 | 0.001208 | 0.000246 | -0.067097 | | 0.232793 | -0.57677 | | 15.0618 | 14.8281 | 14.7986 | 14.8027 | 14.8237 | 15.1104 | 15.1663 | 14.9131 | 3 | 3 | 3 | 2 | 3 | 3 | 3 | 2 |
| SIRT6 | Q8N6T7 | 0.000257 | 5.76E-05 | -0.0670949 | | 0.412746 | -0.93349 | | 13.9648 | 14.0588 | 14.0593 | 14.2745 | 14.053 | 14.1111 | 13.9629 | 14.0722 | 4 | 4 | 3 | 3 | 3 | 2 | 4 | 3 |
| MAPK1 | P28482 | 3.87E-12 | 2.43E-12 | -0.066988 | | 0.389205 | -0.88921 | | 19.2047 | 19.2192 | 19.1721 | 19.0716 | 19.419 | 19.2898 | 19.2542 | 19.2937 | 12 | 12 | 12 | 13 | 12 | 12 | 13 | 12 |
| ERO1B | Q86YB8 | 0.001002 | 0.000207 | -0.0667825 | | 0.252933 | -0.61919 | | 18.6202 | 18.6135 | 18.2889 | 18.7078 | 18.6277 | 18.3874 | 18.5321 | 18.6163 | 1 | 1 | 2 | 2 | 1 | 1 | 2 | 1 |
| EMP3 | P54852 | 0.002034 | 0.000398 | -0.066583 | | 0.069006 | -0.19332 | | 17.1661 | 16.9286 | 17.0747 | 17.1254 | 16.3377 | 17.7432 | 17.5861 | 16.823 | 3 | 3 | 3 | 3 | 3 | 3 | 3 | 2 |
| LCMT1 | Q9UIC8;Q9 | 1.99E-11 | 1.09E-11 | -0.0662033 | | 0.400387 | -0.91031 | | 16.1727 | 16.2743 | 16.3077 | 16.3247 | 16.1529 | 16.4552 | 16.3685 | 16.2875 | 7 | 7 | 6 | 6 | 7 | 7 | 7 | 6 |
| PDLIM2 | Q96JY6;Q9 | 0.000106 | 2.51E-05 | -0.0658264 | | 0.411743 | -0.93161 | | 18.3678 | 18.3127 | 18.3708 | 18.4167 | 18.4722 | 18.5619 | 18.2471 | 18.3835 | 7 | 7 | 8 | 6 | 6 | 7 | 6 | 6 |
| VPS50 | Q96JG6 | 1.47E-13 | 1.33E-13 | -0.0657347 | | 0.554068 | -1.1894 | | 15.4598 | 15.5087 | 15.5042 | 15.5849 | 15.4717 | 15.5823 | 15.681 | 15.4631 | 20 | 20 | 20 | 19 | 18 | 20 | 19 | 18 |
| TARS2 | Q9BW92 | 8.24E-08 | 2.68E-08 | -0.0656351 | | 0.415848 | -0.93928 | | 15.1337 | 15.1398 | 15.1705 | 15.0409 | 15.2569 | 15.3342 | 15.1554 | 15.2808 | 8 | 8 | 7 | 7 | 5 | 7 | 6 | 9 |
| CBFB | Q13951 | 1.10E-14 | 1.38E-14 | -0.0654828 | | 0.209762 | -0.52726 | | 17.3573 | 17.3805 | 17.2857 | 17.7018 | 17.2476 | 17.2842 | 17.2564 | 17.5432 | 8 | 8 | 8 | 8 | 6 | 8 | 6 | 9 |
| USP24 | Q9UPU5 | 1.20E-19 | 7.24E-19 | -0.0653526 | | 0.334369 | -0.78364 | | 15.5136 | 15.5581 | 15.4891 | 15.7694 | 15.6255 | 15.4612 | 15.4359 | 15.636 | 45 | 44 | 44 | 41 | 35 | 39 | 41 | 40 |
| URI1 | O94763;O9 | 3.64E-13 | 2.97E-13 | -0.0652953 | | 0.487696 | -1.07108 | | 15.2488 | 15.2213 | 15.2887 | 15.1902 | 15.4694 | 15.3169 | 15.3101 | 15.3045 | 10 | 10 | 10 | 9 | 10 | 10 | 11 | 11 |
| PIGO | Q8TEQ8 | 0.001015 | 0.000209 | -0.0650954 | | 0.090276 | -0.2503 | | 14.5323 | 14.2896 | 14.3522 | 14.7817 | 13.8379 | 14.694 | NaN | 14.5123 | 2 | 2 | 2 | 2 | 1 | 2 | NaN | 2 |
| DOCK9 | Q9BZ29;Q9 | 0.000951 | 0.000197 | -0.0650531 | | 0.067643 | -0.18974 | | 14.9586 | 15.7452 | 15.9446 | 14.9835 | 15.3673 | 15.8226 | 16.1104 | 15.7888 | 4 | 4 | 4 | 1 | 1 | 3 | 2 | 2 |
| ZC3H14 | Q6PJT7-2;Q | 3.31E-09 | 1.28E-09 | -0.0649994 | | 0.439385 | -0.98295 | | 15.1577 | 15.0799 | 15.166 | 15.2178 | 15.2971 | 15.0414 | 15.154 | 15.2875 | 17 | 16 | 17 | 16 | 16 | 15 | 18 | 17 |
| FNDC3A | Q9Y2H6;Q9 | 4.85E-12 | 2.98E-12 | -0.0648349 | | 0.644808 | -1.3472 | | 15.2299 | 15.3034 | 15.2239 | 15.3803 | 15.2606 | 15.2281 | 15.4009 | 15.3164 | 17 | 20 | 18 | 15 | 11 | 17 | 17 | 15 |

| Gene names | UniProt IDs | Protein Group P-value | Protein Group Q-value | Log2 Difference NUP214 vs Controls (n=5) | p<0.05 | -log10 p-value | T-test Test statistic | NPC Localization | Log2 MS2_quantity_NUP214_1 | Log2 MS2_quantity_NUP214_2 | Log2 MS2_quantity_NUP214_3 | Log2 MS2_quantity_Control_1 | Log2 MS2_quantity_Control_2 | Log2 MS2_quantity_Control_3 | Log2 MS2_quantity_Control_4 | Log2 MS2_quantity_Control_5 | Number Peptides for Quantitation NUP214_1 | Number Peptides for Quantitation NUP214_2 | Number Peptides for Quantitation NUP214_3 | Number Peptides for Quantitation Control_1 | Number Peptides for Quantitation Control_2 | Number Peptides for Quantitation Control_3 | Number Peptides for Quantitation Control_4 | Number Peptides for Quantitation Control_5 |
|---|---|---|---|---|---|---|---|---|---|---|---|---|---|---|---|---|---|---|---|---|---|---|---|---|
| ABCF2 | Q9UG63;Q | 7.57E-17 | 1.84E-16 | -0.0647559 | | 0.150845 | -0.39491 | | 16.4955 | 16.5299 | 16.5085 | 16.9384 | 16.1804 | 16.5955 | 16.5007 | 16.6653 | 25 | 24 | 24 | 25 | 21 | 26 | 25 | 25 |
| SLU7 | O95391 | 0.002275 | 0.00044 | -0.064707 | | 0.173028 | -0.44579 | | 13.9675 | 13.8903 | 13.9132 | 13.9927 | 14.1176 | 14.1182 | 14.1444 | 13.5689 | 3 | 3 | 3 | 2 | 3 | 1 | 2 | 2 |
| TPP1 | O14773 | 1.11E-08 | 4.02E-09 | -0.0646826 | | 0.096593 | -0.26404 | | 18.1935 | 18.1165 | 18.1334 | 17.7455 | 18.0647 | 18.4301 | 18.802 | 18.02 | 7 | 7 | 7 | 7 | 7 | 7 | 7 | 7 |
| ATP5F1C | P36542;P3 | 8.38E-15 | 1.08E-14 | -0.064667 | | 0.799552 | -1.60941 | | 19.1173 | 19.1956 | 19.1186 | 19.1243 | 19.1847 | 19.2379 | 19.212 | 19.2834 | 10 | 10 | 10 | 10 | 10 | 10 | 10 | 10 |
| RGP1 | Q92546 | 2.88E-15 | 4.32E-15 | -0.0644568 | | 0.189349 | -0.48239 | | 14.8848 | 14.715 | 14.7533 | 14.73 | 14.9459 | 14.9956 | 15.0433 | 14.5293 | 4 | 6 | 7 | 5 | 6 | 6 | 7 | 6 |
| GPX7 | Q96SL4 | 6.76E-09 | 2.51E-09 | -0.0642949 | | 0.058251 | -0.16485 | | 17.0066 | 16.9824 | 16.9633 | 16.0737 | 17.3279 | 17.8662 | 17.0418 | 16.9324 | 5 | 6 | 6 | 3 | 5 | 5 | 5 | 6 |
| RBM12B | Q8IXT5 | 7.66E-12 | 4.55E-12 | -0.0642731 | | 0.485201 | -1.06657 | | 15.1733 | 15.1709 | 15.1182 | 15.2277 | 15.1661 | 15.0804 | 15.3269 | 15.2909 | 17 | 17 | 16 | 17 | 15 | 13 | 15 | 16 |
| SPATA5 | Q8NB90 | 3.44E-06 | 9.47E-07 | -0.0642359 | | 0.248066 | -0.60901 | | 15.2935 | 15.3023 | 15.2521 | 15.5513 | 15.1922 | 15.2454 | 15.525 | 15.2204 | 10 | 10 | 8 | 10 | 6 | 11 | 11 | 11 |
| MCM4 | P33991 | 1.02E-11 | 5.93E-12 | -0.0640509 | | 0.086919 | -0.2396 | | 16.2304 | 16.2254 | 16.2394 | 16.7595 | 16.341 | 15.6086 | 16.1628 | 16.6071 | 26 | 27 | 28 | 30 | 27 | 20 | 28 | 29 |
| MOSPD2 | Q8NHP6 | 2.41E-11 | 1.31E-11 | -0.0640062 | | 0.36374 | -0.84063 | | 16.2577 | 16.3008 | 16.3349 | 16.2569 | 16.3517 | 16.4124 | 16.5465 | 16.2416 | 15 | 14 | 16 | 13 | 14 | 16 | 15 | 15 |
| ACSS2 | Q9NR19;Q | 2.23E-08 | 7.75E-09 | -0.0639174 | | 0.219066 | -0.5474 | | 16.0944 | 16.2115 | 16.1984 | 16.5502 | 16.07 | 16.1174 | 16.2541 | 16.1685 | 16 | 16 | 17 | 17 | 13 | 16 | 15 | 16 |
| PLPP1 | O14494 | 1.40E-05 | 3.63E-06 | -0.0638549 | | 0.207 | -0.52124 | | 16.8782 | 16.8846 | 17.0265 | 16.7757 | 16.9665 | 17.3155 | 16.9559 | 16.9545 | 5 | 4 | 4 | 4 | 3 | 4 | 5 | 4 |
| KLHL12 | Q53G59;Q | 0.002524 | 0.000482 | -0.0638192 | | 0.070368 | -0.19858 | | 14.5105 | 14.3397 | 14.6227 | 14.9559 | 13.8573 | 14.4261 | NaN | 14.9799 | 2 | 2 | 1 | 2 | 2 | 1 | NaN | 2 |
| PYGM | P11217 | 8.83E-06 | 2.33E-06 | -0.0637981 | | 0.104041 | -0.28261 | | 18.7232 | 18.7202 | 18.6212 | 18.3025 | 18.7602 | 18.915 | 19.2779 | 18.5044 | 12 | 12 | 12 | 10 | 11 | 11 | 11 | 11 |
| BRAT1 | Q6PJG6 | 1.19E-12 | 8.46E-13 | -0.0637846 | | 0.128073 | -0.34117 | | 15.3048 | 15.2806 | 15.227 | 14.9614 | 15.7188 | 15.097 | 15.5506 | 15.345 | 15 | 14 | 12 | 7 | 11 | 5 | 13 | 7 |
| COIL | P38432 | 0.000138 | 3.21E-05 | -0.0635417 | | 0.207849 | -0.52309 | | 15.0283 | 14.9599 | 15.0129 | 15.2581 | 15.2061 | 14.856 | 14.8354 | 15.164 | 6 | 5 | 6 | 6 | 4 | 5 | 7 | 6 |
| CHMP4B | Q9H444 | 3.41E-07 | 1.05E-07 | -0.0635384 | | 0.104123 | -0.28281 | | 17.5293 | 17.53 | 17.5343 | 17.8978 | 17.7805 | 16.9421 | 17.6441 | 17.7091 | 8 | 9 | 9 | 9 | 8 | 7 | 8 | 9 |
| SNX27 | Q96L92 | 4.09E-09 | 1.56E-09 | -0.0634514 | | 1.27301 | -2.39949 | | 16.0976 | 16.1216 | 16.1429 | 16.2109 | 16.1652 | 16.1551 | 16.2432 | 16.1463 | 18 | 18 | 18 | 19 | 14 | 17 | 18 | 18 |
| SLC25A20 | O43772 | 3.04E-10 | 1.35E-10 | -0.0634351 | | 0.438617 | -0.98153 | | 16.3519 | 16.3337 | 16.221 | 16.3095 | 16.2401 | 16.376 | 16.411 | 16.4916 | 12 | 12 | 11 | 10 | 11 | 11 | 11 | 11 |
| CIRBP | Q14011 | 2.20E-05 | 5.58E-06 | -0.0634197 | | 0.12902 | -0.34344 | | 17.1742 | 17.2468 | 17.3318 | 17.2136 | 16.9445 | 17.7866 | 17.2888 | 17.3383 | 4 | 4 | 6 | 4 | 3 | 5 | 5 | 4 |
| COG8 | Q96MW5 | 6.00E-05 | 1.46E-05 | -0.0633609 | | 0.222498 | -0.55478 | | 15.859 | 15.8506 | 15.7678 | 15.7238 | 16.1042 | 15.7169 | 16.0748 | 15.8261 | 6 | 5 | 7 | 6 | 9 | 8 | 8 | 8 |
| SURF6 | O75683 | 0.001789 | 0.000355 | -0.0633366 | | 0.28826 | -0.69181 | | 15.1338 | 15.0703 | 14.8503 | 15.0016 | 14.9456 | 15.2227 | 15.0835 | 15.1539 | 6 | 8 | 7 | 7 | 7 | 7 | 7 | 7 |
| NPEPL1 | Q8NDH3;Q | 6.99E-11 | 3.46E-11 | -0.0632346 | | 0.214396 | -0.53731 | | 15.5935 | 15.6804 | 15.5357 | 15.4019 | 15.6137 | 15.8048 | 15.8925 | 15.6192 | 9 | 9 | 9 | 9 | 9 | 9 | 9 | 9 |
| RBBP6 | Q7Z6E9;Q7 | 0.002069 | 0.000404 | -0.0631898 | | 0.161639 | -0.41983 | | 13.4553 | 13.3623 | 13.7273 | 13.3899 | 13.6514 | 13.4327 | 13.4998 | 13.917 | 3 | 4 | 2 | 2 | 2 | 4 | 4 | 3 |
| LIN7C | Q9NUP9 | 2.89E-14 | 3.21E-14 | -0.0631879 | | 0.175064 | -0.45039 | | 16.3112 | 16.1441 | 16.1472 | 16.3804 | 15.9083 | 16.4823 | 16.3617 | 16.1872 | 6 | 5 | 5 | 4 | 5 | 4 | 4 | 5 |
| PRCC | Q92733 | 2.43E-05 | 6.12E-06 | -0.0631411 | | 0.252361 | -0.61799 | | 15.3517 | 15.3381 | 15.231 | 15.1965 | 15.5952 | 15.4629 | 15.3644 | 15.2314 | 4 | 4 | 5 | 5 | 4 | 5 | 4 | 4 |
| MAP1LC3A | Q9H492;Q | 0.000898 | 0.000186 | -0.0631368 | | 0.151802 | -0.39713 | | 19.4695 | 19.5804 | 19.4143 | 19.8502 | 19.304 | 19.3635 | 19.4255 | 19.8129 | 3 | 3 | 3 | 4 | 3 | 3 | 3 | 3 |
| MRPL14 | Q6P1L8 | 9.96E-11 | 4.77E-11 | -0.0630739 | | 0.223988 | -0.55797 | | 16.6284 | 16.6136 | 16.623 | 16.6013 | 16.94 | 16.4592 | 16.6135 | 16.8096 | 3 | 3 | 4 | 3 | 3 | 4 | 4 | 4 |
| ARMCX3 | Q9UH62 | 1.68E-11 | 9.39E-12 | -0.0628412 | | 0.594392 | -1.26002 | | 17.3843 | 17.363 | 17.4332 | 17.4071 | 17.4775 | 17.3504 | 17.5574 | 17.4894 | 15 | 14 | 15 | 14 | 14 | 14 | 15 | 15 |
| HMOX2 | P30519 | 3.10E-14 | 3.40E-14 | -0.0626628 | | 0.24761 | -0.60805 | | 16.6731 | 16.7372 | 16.7195 | 17.0311 | 16.5879 | 16.6497 | 16.792 | 16.8023 | 11 | 12 | 12 | 12 | 11 | 12 | 12 | 12 |
| DTNBP1 | Q96EV8 | 9.08E-06 | 2.39E-06 | -0.0626141 | | 0.176303 | -0.45319 | | 14.7972 | 14.971 | 14.9593 | 14.7308 | 15.0105 | 15.2535 | 15.0961 | 14.7681 | 7 | 6 | 6 | 5 | 4 | 6 | 5 | 4 |
| ACTN1 | P12814 | 5.37E-15 | 7.35E-15 | -0.0625413 | | 0.361325 | -0.83598 | | 21.345 | 21.3764 | 21.3687 | 21.3733 | 21.3018 | 21.6284 | 21.4512 | 21.3749 | 64 | 67 | 67 | 65 | 62 | 64 | 65 | 65 |
| MTPAP | Q9NVV4 | 9.50E-06 | 2.50E-06 | -0.0624781 | | 0.467793 | -1.035 | | 14.4711 | 14.5023 | 14.5344 | 14.57 | 14.5608 | 14.6297 | 14.6603 | 14.4047 | 8 | 9 | 9 | 7 | 4 | 6 | 7 | 6 |
| RPS8 | P62241 | 2.97E-18 | 1.12E-17 | -0.0624214 | | 0.649744 | -1.35568 | | 21.3673 | 21.3754 | 21.3393 | 21.4063 | 21.5549 | 21.402 | 21.3587 | 21.3934 | 14 | 14 | 14 | 14 | 14 | 14 | 14 | 14 |
| NOMO1 | Q15155 | 0.001235 | 0.000252 | -0.0623802 | | 0.090601 | -0.24895 | | 14.5899 | 14.882 | 14.5242 | 14.8909 | 15.3492 | 14.3995 | 14.4319 | 14.5673 | 2 | 2 | 2 | 2 | 1 | 2 | 2 | 1 |
| SLC2A3 | P11169 | 0.002052 | 0.000401 | -0.0623571 | | 0.159277 | -0.41441 | | 16.3041 | 16.2564 | 16.3086 | 16.6037 | 16.6323 | 16.0692 | 16.2172 | 16.2381 | 5 | 5 | 5 | 3 | 3 | 4 | 4 | 3 |
| SEPTIN9 | Q9UHD8-5 | 1.22E-11 | 7.00E-12 | -0.0623254 | | 0.25135 | -0.61588 | | 19.3692 | 19.3959 | 19.4013 | 19.5294 | 19.5622 | 19.1511 | 19.5101 | 19.5028 | 34 | 36 | 36 | 36 | 34 | 34 | 35 | 34 |
| ARCN1 | P48444 | 1.08E-15 | 1.83E-15 | -0.0622735 | | 0.455473 | -1.01251 | | 18.649 | 18.6494 | 18.6362 | 18.7602 | 18.5415 | 18.8007 | 18.7571 | 18.6763 | 34 | 34 | 34 | 35 | 34 | 35 | 34 | 35 |
| PSMD13 | Q9UNM6 | 2.58E-13 | 2.20E-13 | -0.0622103 | | 1.20339 | -2.28226 | | 18.2813 | 18.2642 | 18.2942 | 18.2947 | 18.3967 | 18.3717 | 18.3001 | 18.3473 | 24 | 24 | 24 | 24 | 23 | 23 | 23 | 23 |
| LUZP1 | Q86V48 | 2.81E-23 | 5.46E-22 | -0.0618246 | | 0.304783 | -0.72508 | | 15.957 | 15.9568 | 15.9193 | 16.1726 | 15.79 | 16.0771 | 16.0194 | 15.9717 | 43 | 43 | 46 | 45 | 41 | 43 | 44 | 40 |
| ACSL1 | P33121 | 5.75E-21 | 5.59E-20 | -0.061787 | | 0.251074 | -0.6153 | | 16.1897 | 16.1651 | 16.226 | 16.2534 | 15.9815 | 16.4221 | 16.2723 | 16.3474 | 24 | 23 | 24 | 23 | 15 | 24 | 24 | 19 |
| DRG1 | Q9Y295 | 7.60E-14 | 7.48E-14 | -0.061778 | | 1.25527 | -2.36954 | | 17.1842 | 17.1562 | 17.148 | 17.2605 | 17.2471 | 17.2172 | 17.1556 | 17.2425 | 20 | 20 | 20 | 21 | 21 | 20 | 21 | 21 |
| PLCG1 | P19174;P1 | 3.79E-13 | 3.08E-13 | -0.0616102 | | 0.161904 | -0.42044 | | 16.1231 | 16.1215 | 16.1019 | 16.49 | 16.3515 | 15.8656 | 16.0737 | 16.1047 | 25 | 25 | 27 | 31 | 25 | 21 | 24 | 24 |
| PPM1G | O15355 | 7.95E-09 | 2.93E-09 | -0.061543 | | 0.275106 | -0.66502 | | 16.5262 | 16.5271 | 16.5058 | 16.784 | 16.476 | 16.392 | 16.583 | 16.671 | 16 | 16 | 16 | 17 | 17 | 15 | 18 | 18 |
| CNOT10 | Q9H9A5;Q | 6.73E-05 | 1.63E-05 | -0.0608033 | | 0.415315 | -0.93829 | | 16.0388 | 16.1834 | 16.1106 | 16.151 | 16.2624 | 16.2812 | 16.0758 | 16.0883 | 9 | 8 | 10 | 6 | 6 | 8 | 6 | 7 |
| PSD3 | Q9NYI0;Q9 | 1.37E-06 | 3.92E-07 | -0.0607452 | | 0.053784 | -0.15286 | | 15.0001 | 15.1768 | 15.134 | 14.2709 | 16.0224 | 15.2271 | 15.4831 | 14.8185 | 8 | 11 | 11 | 6 | 13 | 11 | 12 | 5 |
| ARMC6 | Q6NXE6;Q6 | 3.24E-07 | 9.97E-08 | -0.0607002 | | 0.566871 | -1.21192 | | 15.2275 | 15.1783 | 15.2416 | 15.4181 | 15.2505 | 15.2315 | 15.2215 | 15.261 | 9 | 8 | 8 | 7 | 6 | 8 | 7 | 8 |
| HECTD3 | Q5T447 | 3.18E-11 | 1.68E-11 | -0.0606018 | | 0.221327 | -0.55226 | | 15.6861 | 15.696 | 15.6293 | 15.961 | 15.7781 | 15.4535 | 15.7116 | 15.7511 | 12 | 13 | 10 | 11 | 8 | 8 | 9 | 10 |
| DPM1 | O60762 | 2.79E-11 | 1.49E-11 | -0.0605498 | | 0.35241 | -0.81877 | | 18.0119 | 17.9662 | 17.9843 | 17.8574 | 18.0276 | 18.0982 | 18.1927 | 18.0641 | 12 | 12 | 13 | 12 | 12 | 12 | 13 | 12 |
| BYSL | Q13895 | 4.04E-13 | 3.26E-13 | -0.060545 | | 0.248655 | -0.61024 | | 16.0835 | 16.0969 | 16.0513 | 16.0772 | 16.2089 | 15.9558 | 16.0606 | 16.3864 | 16 | 17 | 17 | 17 | 13 | 18 | 15 | 18 |
| TBC1D17 | Q9HA65;Q | 2.19E-13 | 1.90E-13 | -0.0602341 | | 0.45131 | -1.00488 | | 16.6824 | 16.7545 | 16.7318 | 16.7617 | 16.7851 | 16.9468 | 16.7053 | 16.7169 | 12 | 11 | 11 | 10 | 11 | 10 | 11 | 11 |
| HFE | Q30201;Q3 | 0.000355 | 7.80E-05 | -0.0601918 | | 0.083532 | -0.23095 | | 15.1323 | 15.0345 | 15.0613 | 15.0043 | 15.5007 | 14.4478 | 15.4885 | 15.2399 | 3 | 3 | 4 | 3 | 3 | 2 | 3 | 2 |
| TBCB | Q99426 | 1.33E-07 | 4.25E-08 | -0.0600925 | | 0.299841 | -0.71517 | | 18.6098 | 18.5993 | 18.6311 | 18.8364 | 18.6242 | 18.4654 | 18.6875 | 18.7538 | 10 | 10 | 10 | 10 | 10 | 10 | 11 | 10 |
| PUF60 | Q9UHX1-2 | 4.31E-20 | 3.12E-19 | -0.0598326 | | 0.366351 | -0.84564 | | 17.2266 | 17.2348 | 17.189 | 17.38 | 17.2453 | 17.0867 | 17.3504 | 17.3208 | 23 | 24 | 23 | 24 | 21 | 23 | 23 | 23 |
| CDC42EP3 | Q9UKI2 | 4.96E-16 | 9.11E-16 | -0.059699 | | 0.106459 | -0.2886 | | 17.0349 | 16.8805 | 16.9671 | 16.9363 | 16.6545 | 17.541 | 16.8196 | 17.1513 | 8 | 8 | 8 | 8 | 7 | 8 | 8 | 8 |
| SLC44A1 | Q8WWI5;C | 5.77E-13 | 4.44E-13 | -0.0596363 | | 0.117939 | -0.31672 | | 16.9623 | 16.9357 | 16.9907 | 17.5458 | 16.7379 | 16.8526 | 16.9132 | 17.0632 | 11 | 11 | 12 | 11 | 9 | 10 | 10 | 10 |
| MLEC | Q14165 | 3.64E-12 | 2.30E-12 | -0.0595723 | | 0.283843 | -0.68285 | | 18.2124 | 18.2834 | 18.2976 | 18.2986 | 18.1664 | 18.3107 | 18.5573 | 18.287 | 12 | 12 | 12 | 12 | 12 | 12 | 12 | 12 |
| LPCAT3 | Q6P1A2 | 4.77E-05 | 1.17E-05 | -0.0595349 | | 0.189625 | -0.483 | | 15.278 | 15.4654 | 15.5168 | 15.1782 | 15.6379 | 15.4979 | 15.6294 | 15.4545 | 5 | 5 | 6 | 3 | 4 | 3 | 3 | 4 |
| RBMXL1 | Q96E39 | 2.84E-11 | 1.51E-11 | -0.0595263 | | 0.269868 | -0.65427 | | 14.8739 | 14.9687 | 14.9131 | 14.9467 | 14.7891 | 15.161 | 15.091 | 14.9026 | 3 | 3 | 2 | 3 | 2 | 2 | 3 | 2 |
| CD276 | Q5ZPR3;Q5 | 0.000139 | 3.23E-05 | -0.0594874 | | 0.340438 | -0.79551 | | 17.1478 | 17.2527 | 17.3104 | 17.2344 | 17.357 | 17.3218 | 17.4275 | 17.1416 | 8 | 9 | 8 | 8 | 7 | 8 | 7 | 8 |
| RNF2 | Q99496 | 0.000474 | 0.000102 | -0.0594103 | | 0.18312 | -0.4685 | | 14.5558 | 14.6893 | 14.8648 | 14.9554 | 14.951 | 14.5616 | 14.6299 | 14.7156 | 3 | 3 | 4 | 3 | 3 | 3 | 3 | 3 |
| CWC27 | Q6UX04 | 0.000137 | 3.18E-05 | -0.0592775 | | 0.232027 | -0.57514 | | 15.4995 | 15.3252 | 15.478 | 15.6778 | 15.3645 | 15.2979 | 15.5266 | 15.6008 | 4 | 5 | 5 | 4 | 3 | 6 | 4 | 3 |
| RPL5 | P46777 | 1.20E-11 | 6.84E-12 | -0.059264 | | 0.688407 | -1.42178 | | 19.95 | 19.9692 | 20.0252 | 20.0433 | 19.984 | 20.0943 | 20.1132 | 19.969 | 18 | 17 | 17 | 16 | 18 | 16 | 16 | 17 |
| PSMD8 | P48556 | 4.16E-09 | 1.59E-09 | -0.0591347 | | 0.350394 | -0.81487 | | 18.4208 | 18.3986 | 18.442 | 18.4628 | 18.5519 | 18.2762 | 18.5384 | 18.5688 | 12 | 12 | 13 | 12 | 12 | 13 | 12 | 13 |
| TBC1D23 | Q9NUY8 | 0.000164 | 3.78E-05 | -0.0591192 | | 0.246451 | -0.60562 | | 15.7313 | 15.7747 | 15.7062 | 15.8808 | 15.943 | 15.7663 | 15.5301 | 15.8624 | 13 | 14 | 15 | 15 | 10 | 15 | 14 | 13 |
| ECSIT | Q9BQ95 | 3.30E-06 | 9.11E-07 | -0.0591009 | | 0.171014 | -0.44122 | | 14.715 | 14.6002 | 14.6925 | 14.8836 | 14.4049 | 14.8615 | 14.5919 | 14.8999 | 9 | 10 | 9 | 10 | 7 | 10 | 7 | 9 |
| MLYCD | O95822 | 1.54E-09 | 6.22E-10 | -0.0590082 | | 0.177754 | -0.45646 | | 15.5148 | 15.4875 | 15.5314 | 15.2053 | 15.6685 | 15.7661 | 15.6463 | 15.565 | 10 | 10 | 10 | 8 | 6 | 8 | 6 | 8 |
| RPS25 | P62851 | 0.001777 | 0.000353 | -0.058954 | | 0.75584 | -1.53597 | | 21.6808 | 21.6464 | 21.6232 | 21.6811 | 21.8097 | 21.6868 | 21.6506 | 21.7173 | 6 | 6 | 6 | 6 | 6 | 6 | 6 | 6 |
| FZD7 | O75084 | 2.41E-07 | 7.52E-08 | -0.0588657 | | 0.161432 | -0.41936 | | 16.401 | 16.4685 | 16.3521 | 16.7263 | 16.2907 | 16.6885 | 16.4118 | 16.2132 | 5 | 5 | 4 | 4 | 4 | 4 | 4 | 4 |
| SUGT1 | Q9Y2Z0 | 2.46E-18 | 9.51E-18 | -0.058815 | | 0.188846 | -0.48127 | | 17.2015 | 17.2492 | 17.2318 | 17.498 | 16.9623 | 17.235 | 17.3911 | 17.345 | 19 | 20 | 21 | 21 | 19 | 18 | 20 | 20 |
| DCTN4 | Q9UJW0 | 2.19E-06 | 6.13E-07 | -0.0586745 | | 0.379438 | -0.87066 | | 16.9641 | 16.9819 | 16.9991 | 17.1272 | 17.1387 | 17.0021 | 16.8644 | 17.0696 | 19 | 19 | 19 | 18 | 17 | 18 | 16 | 18 |
| DAPK3 | O43293 | 7.75E-08 | 2.53E-08 | -0.058654 | | 0.259703 | -0.63327 | | 15.644 | 15.6033 | 15.5524 | 15.9271 | 15.5944 | 15.5533 | 15.6168 | 15.6015 | 10 | 9 | 10 | 9 | 7 | 9 | 8 | 8 |
| PSMD3 | O43242 | 2.78E-12 | 1.80E-12 | -0.0584175 | + | 2.10142 | -3.90726 | | 18.3912 | 18.4291 | 18.3865 | 18.458 | 18.4571 | 18.4869 | 18.4668 | 18.4346 | 34 | 34 | 34 | 34 | 34 | 34 | 35 | 34 |
| RAB3GAP1 | Q15042 | 1.92E-16 | 4.15E-16 | -0.0583703 | | 0.602685 | -1.27443 | | 16.0239 | 15.9521 | 15.9449 | 16.1366 | 16.0449 | 16.0081 | 15.9421 | 16.0282 | 38 | 38 | 38 | 35 | 31 | 36 | 37 | 37 |
| TRAPPC4 | Q9Y296 | 2.90E-09 | 1.13E-09 | -0.0582888 | | 0.36619 | -0.84533 | | 16.5452 | 16.6097 | 16.5531 | 16.739 | 16.63 | 16.4613 | 16.5848 | 16.7231 | 12 | 12 | 12 | 12 | 11 | 12 | 12 | 12 |
| DOCK7 | Q96N67;Q | 2.89E-13 | 2.43E-13 | -0.0582843 | | 0.3408 | -0.79621 | | 16.1921 | 16.2162 | 16.195 | 16.4096 | 16.2988 | 16.0703 | 16.2654 | 16.2527 | 49 | 52 | 52 | 56 | 48 | 52 | 50 | 53 |
| SLC41A3 | Q96GZ6;Q | 0.000552 | 0.000118 | -0.0581635 | | 0.257804 | -0.62933 | | 15.1595 | 15.1687 | 15.0572 | 14.9835 | 15.3096 | 15.0724 | 15.2919 | 15.2757 | 4 | 4 | 4 | 4 | 4 | 3 | 4 | 4 |
| SCRN3 | Q0VDG4;Q | 5.95E-11 | 2.98E-11 | -0.0576383 | | 0.17615 | -0.45284 | | 16.2096 | 16.2529 | 16.1147 | 15.9618 | 16.4544 | 16.4475 | 16.162 | 16.2244 | 7 | 7 | 7 | 5 | 6 | 5 | 6 | 7 |

| Gene names | UniProt IDs | Protein Group P-value | Protein Group Q-value | Log2 Difference NUP214 vs Controls (n=5) | p<0.05 | -log10 p-value | T-test Test statistic | NPC Localization | Log2 MS2_quantity_NUP214_1 | Log2 MS2_quantity_NUP214_2 | Log2 MS2_quantity_NUP214_3 | Log2 MS2_quantity_Control_1 | Log2 MS2_quantity_Control_2 | Log2 MS2_quantity_Control_3 | Log2 MS2_quantity_Control_4 | Log2 MS2_quantity_Control_5 | Number Peptides for Quantitation NUP214_1 | Number Peptides for Quantitation NUP214_2 | Number Peptides for Quantitation NUP214_3 | Number Peptides for Quantitation Control_1 | Number Peptides for Quantitation Control_2 | Number Peptides for Quantitation Control_3 | Number Peptides for Quantitation Control_4 | Number Peptides for Quantitation Control_5 |
|---|---|---|---|---|---|---|---|---|---|---|---|---|---|---|---|---|---|---|---|---|---|---|---|---|
| XIAP | P98170 | 0.000111 | 2.62E-05 | -0.0574689 | | 0.391992 | -0.89448 | | 15.2364 | 15.2623 | 15.1419 | 15.2942 | 15.4329 | 15.2109 | 15.2188 | 15.1982 | 11 | 11 | 11 | 7 | 6 | 9 | 8 | 8 |
| DDX24 | Q9GZR7 | 8.96E-14 | 8.66E-14 | -0.0574041 | | 0.321325 | -0.75797 | | 15.4391 | 15.4012 | 15.453 | 15.5604 | 15.4363 | 15.3157 | 15.4825 | 15.6475 | 17 | 18 | 16 | 15 | 14 | 15 | 15 | 16 |
| YES1 | P07947 | 6.63E-10 | 2.80E-10 | -0.0572631 | | 0.124633 | -0.33291 | | 16.6676 | 16.719 | 16.6912 | 17.0266 | 16.7896 | 16.8439 | 16.2616 | 16.8276 | 14 | 13 | 14 | 15 | 12 | 14 | 12 | 11 |
| GXYLT1 | Q4G148;Q | 2.45E-10 | 1.11E-10 | -0.057224 | | 0.214607 | -0.53777 | | 15.3463 | 15.3024 | 15.1995 | 15.3431 | 15.5852 | 15.4089 | 15.2013 | 15.1614 | 4 | 3 | 4 | 3 | 3 | 3 | 3 | 3 |
| TEFM | Q96QE5 | 0.001599 | 0.00032 | -0.0571449 | | 0.126333 | -0.337 | | 15.1147 | 15.1068 | 15.0044 | 14.6438 | 15.2731 | 15.1922 | 15.1984 | 15.3547 | 2 | 2 | 2 | 1 | 1 | 2 | 2 | 2 |
| PTPRK | Q15262-2; | 8.03E-13 | 5.93E-13 | -0.0570227 | | 0.182856 | -0.46791 | | 15.0557 | 15.0826 | 15.082 | 14.9573 | 15.0486 | 15.2415 | 15.434 | 14.9709 | 15 | 15 | 13 | 14 | 10 | 12 | 14 | 9 |
| VPS18 | Q9P253 | 2.13E-19 | 1.19E-18 | -0.0568446 | | 0.524308 | -1.1367 | | 16.0972 | 16.0967 | 16.0301 | 16.0415 | 16.2129 | 16.1374 | 16.205 | 16.0608 | 26 | 27 | 26 | 27 | 24 | 26 | 26 | 26 |
| SDF2 | Q99470 | 5.52E-09 | 2.07E-09 | -0.0567935 | | 0.075868 | -0.21121 | | 15.8505 | 16.0105 | 15.9339 | 16.1118 | 15.2787 | 16.4493 | 16.2264 | 15.8758 | 4 | 5 | 5 | 5 | 4 | 5 | 5 | 4 |
| PRKD2 | Q9BZL6;Q9 | 9.95E-15 | 1.27E-14 | -0.056745 | | 0.103313 | -0.2808 | | 15.0039 | 14.8606 | 14.7991 | 15.1084 | 15.2501 | 14.768 | 14.4496 | 15.1469 | 3 | 3 | 4 | 4 | 4 | 4 | 4 | 4 |
| VARS1 | P26640 | 1.39E-13 | 1.27E-13 | -0.0565881 | | 0.30703 | -0.72957 | | 18.3194 | 18.3369 | 18.337 | 18.512 | 18.2634 | 18.5437 | 18.3161 | 18.3033 | 50 | 49 | 49 | 47 | 48 | 50 | 49 | 51 |
| MECP2 | P51608;P5 | 8.99E-10 | 3.75E-10 | -0.0564366 | | 0.186085 | -0.47512 | | 15.1301 | 15.3285 | 15.3363 | 15.1395 | 15.2299 | 15.5018 | 15.5308 | 15.2051 | 8 | 8 | 8 | 8 | 7 | 7 | 8 | 7 |
| STX17 | P56962 | 0.00172 | 0.000342 | -0.056192 | | 0.072963 | -0.20541 | | 14.6104 | 14.2163 | 14.3407 | 13.8057 | NaN | 14.7483 | 14.6504 | 14.5768 | 4 | 5 | 6 | 4 | NaN | 2 | 4 | 3 |
| CLPX | O76031 | 1.72E-14 | 2.04E-14 | -0.0561224 | | 0.371451 | -0.85541 | | 15.8708 | 15.9081 | 15.8891 | 15.9153 | 16.079 | 15.7864 | 15.9409 | 16.0059 | 16 | 15 | 17 | 14 | 14 | 16 | 17 | 16 |
| PITPNA | Q00169 | 4.12E-06 | 1.12E-06 | -0.0560948 | | 0.155323 | -0.40529 | | 16.1213 | 16.2344 | 16.294 | 16.3953 | 15.9661 | 16.4674 | 16.4303 | 16.1043 | 12 | 13 | 12 | 11 | 9 | 10 | 10 | 10 |
| PURA | Q00577 | 6.34E-05 | 1.54E-05 | -0.0559696 | | 0.306059 | -0.72763 | | 17.6048 | 17.5991 | 17.5511 | 17.6089 | 17.6681 | 17.7672 | 17.7213 | 17.4393 | 7 | 7 | 6 | 7 | 6 | 6 | 6 | 7 |
| PPIF | P30405 | 1.49E-06 | 4.26E-07 | -0.0557635 | | 0.075901 | -0.21129 | | 16.7338 | 16.7916 | 16.7131 | 16.2191 | 17.2991 | 16.8824 | 16.4988 | 17.1102 | 8 | 8 | 8 | 7 | 7 | 8 | 7 | 8 |
| EMC10 | Q5UCC4;Q | 0.000107 | 2.52E-05 | -0.0557192 | | 0.442417 | -0.98854 | | 16.6736 | 16.6177 | 16.6719 | 16.7219 | 16.6579 | 16.823 | 16.762 | 16.586 | 4 | 3 | 3 | 3 | 3 | 4 | 4 | 4 |
| USP40 | Q9NVE5;Q | 4.65E-07 | 1.41E-07 | -0.0555627 | | 0.094776 | -0.25948 | | 14.3407 | 14.1959 | 14.1948 | 13.7122 | 14.4784 | 14.4852 | 14.5917 | 14.2293 | 4 | 5 | 4 | 3 | 1 | 2 | 3 | 2 |
| SH3KBP1 | Q96B97 | 2.70E-16 | 5.43E-16 | -0.0552954 | | 0.184013 | -0.4705 | | 16.352 | 16.3 | 16.3686 | 16.6044 | 16.2241 | 16.1936 | 16.5907 | 16.3646 | 22 | 23 | 22 | 23 | 20 | 21 | 21 | 21 |
| EEA1 | Q15075 | 8.30E-21 | 7.58E-20 | -0.0552841 | | 0.221096 | -0.55176 | | 16.9505 | 16.9597 | 16.9576 | 16.7352 | 17.1632 | 17.1218 | 17.0431 | 16.9927 | 81 | 80 | 79 | 75 | 80 | 81 | 80 | 80 |
| TRMT11 | Q7Z4G4;Q | 0.009556 | 0.001643 | -0.0552691 | | 0.203172 | -0.51288 | | 14.7693 | 14.8746 | 14.869 | 15.0893 | 14.9835 | 14.9053 | 14.8693 | 14.617 | 3 | 3 | 3 | 2 | 1 | 4 | 2 | 2 |
| RABEPK | Q7Z6M1 | 3.59E-07 | 1.10E-07 | -0.0551905 | | 0.098058 | -0.26771 | | 14.8676 | 14.8969 | 15.0101 | 14.4426 | 15.2007 | 15.1473 | 15.267 | 14.8426 | 10 | 9 | 12 | 8 | 7 | 11 | 10 | 8 |
| ACBD6 | Q9BR61 | 2.50E-10 | 1.13E-10 | -0.0551863 | | 0.17902 | -0.4593 | | 14.7729 | 14.9076 | 15.0524 | 14.7958 | 14.8928 | 15.1165 | 15.1888 | 14.8368 | 7 | 8 | 7 | 8 | 3 | 7 | 6 | 4 |
| TRIP4 | Q15650 | 3.36E-13 | 2.77E-13 | -0.0551409 | | 0.241766 | -0.59576 | | 15.6326 | 15.6749 | 15.5798 | 15.6524 | 15.8755 | 15.6171 | 15.7888 | 15.4873 | 13 | 16 | 17 | 14 | 15 | 14 | 15 | 15 |
| KIRREL1 | Q96J84;Q9 | 1.76E-09 | 7.02E-10 | -0.0551212 | | 0.271747 | -0.65813 | | 15.5315 | 15.4698 | 15.4821 | 15.7403 | 15.4241 | 15.6281 | 15.4137 | 15.5417 | 12 | 13 | 12 | 13 | 8 | 12 | 11 | 9 |
| RPS4X | P62701 | 4.89E-15 | 6.78E-15 | -0.0549151 | | 0.468889 | -1.03699 | | 20.4255 | 20.4647 | 20.4483 | 20.3891 | 20.5936 | 20.562 | 20.4298 | 20.5308 | 22 | 21 | 22 | 20 | 21 | 22 | 21 | 22 |
| ARAF | P10398 | 2.40E-07 | 7.50E-08 | -0.0548016 | | 0.316422 | -0.74826 | | 15.8577 | 15.9323 | 15.8965 | 15.8626 | 16.1616 | 15.9067 | 15.9022 | 15.9183 | 11 | 12 | 12 | 13 | 8 | 14 | 13 | 11 |
| CCT8 | P50990 | 5.27E-14 | 5.43E-14 | -0.0545736 | | 0.492868 | -1.08041 | | 19.7208 | 19.768 | 19.7162 | 19.9265 | 19.7794 | 19.722 | 19.7292 | 19.7908 | 38 | 36 | 37 | 36 | 36 | 37 | 36 | 37 |
| B3GALT6 | Q96L58 | 5.99E-05 | 1.46E-05 | -0.0544619 | | 0.104109 | -0.28278 | | 16.3065 | 16.2986 | 16.2783 | 16.8755 | 16.3736 | 16.0304 | 16.1604 | 16.3046 | 4 | 4 | 5 | 5 | 3 | 4 | 4 | 5 |
| RPS3A | P61247 | 3.15E-19 | 1.65E-18 | -0.0542328 | | 0.331153 | -0.77734 | | 19.7758 | 19.8018 | 19.7502 | 19.8108 | 19.9906 | 19.872 | 19.6723 | 19.8052 | 25 | 24 | 25 | 24 | 24 | 25 | 25 | 25 |
| PRMT7 | Q9NVM4;Q | 0.00098 | 0.000202 | -0.0542201 | | 0.123044 | -0.32908 | | 13.3453 | 13.5624 | 13.5479 | 13.808 | 13.7751 | 13.1672 | 13.4953 | 13.4514 | 5 | 5 | 4 | 5 | 2 | 5 | 3 | 4 |
| RPS19 | P39019 | 1.35E-06 | 3.86E-07 | -0.0542107 | | 1.09817 | -2.1064 | | 19.9144 | 19.878 | 19.845 | 19.9299 | 19.9152 | 19.9936 | 19.9266 | 19.9013 | 11 | 11 | 10 | 9 | 11 | 11 | 10 | 11 |
| MTM1 | Q13496 | 5.11E-05 | 1.25E-05 | -0.0540315 | | 0.437052 | -0.97864 | | 15.2141 | 15.2036 | 15.3284 | 15.2905 | 15.3554 | 15.3955 | 15.2824 | 15.1898 | 8 | 9 | 8 | 8 | 9 | 8 | 8 | 9 |
| MSMO1 | Q15800 | 2.38E-05 | 6.02E-06 | -0.0537919 | | 0.036691 | -0.10607 | | 17.2385 | 17.1251 | 17.21 | 18.1572 | 17.8569 | 16.6847 | 16.0916 | 17.4344 | 4 | 3 | 3 | 3 | 2 | 3 | 3 | 3 |
| ADPRS | Q9NX46 | 1.78E-07 | 5.62E-08 | -0.053668 | | 0.244644 | -0.60182 | | 16.3128 | 16.2611 | 16.3337 | 16.4599 | 16.1242 | 16.5035 | 16.3574 | 16.3361 | 9 | 9 | 8 | 10 | 9 | 10 | 10 | 10 |
| RPS20 | P60866;P6 | 1.40E-05 | 3.63E-06 | -0.0535739 | | 0.418547 | -0.94432 | | 20.4808 | 20.378 | 20.37 | 20.4565 | 20.607 | 20.4389 | 20.3858 | 20.4275 | 5 | 5 | 5 | 5 | 5 | 5 | 5 | 5 |
| TRIP11 | Q15643 | 2.82E-19 | 1.52E-18 | -0.0535446 | | 0.215933 | -0.54063 | | 15.2381 | 15.2583 | 15.2411 | 15.1435 | 15.373 | 15.2847 | 15.5421 | 15.1536 | 43 | 48 | 45 | 45 | 34 | 46 | 48 | 41 |
| AK5 | Q9Y6K8;Q | 3.30E-12 | 2.11E-12 | -0.05354 | | 0.056708 | -0.16072 | | 18.9099 | 18.9716 | 18.9055 | 19.4649 | 19.6261 | 18.2849 | 18.6673 | 18.8695 | 14 | 14 | 15 | 14 | 14 | 14 | 14 | 14 |
| ADA | P00813 | 1.05E-15 | 1.79E-15 | -0.0533849 | | 0.043397 | -0.12461 | | 15.8069 | 15.8376 | 15.8779 | 15.145 | 15.5094 | 16.9544 | 15.5948 | 16.2674 | 15 | 15 | 15 | 17 | 14 | 16 | 16 | 16 |
| CTNNA1 | P35221 | 2.28E-14 | 2.61E-14 | -0.0527851 | | 0.174512 | -0.44914 | | 17.8655 | 17.8188 | 17.8418 | 18.0616 | 17.5815 | 18.0539 | 17.9268 | 17.8503 | 47 | 46 | 47 | 46 | 43 | 47 | 45 | 46 |
| TP53BP1 | Q12888;Q | 2.56E-24 | 7.58E-23 | -0.0527414 | | 0.345623 | -0.8056 | | 16.2018 | 16.1409 | 16.1786 | 16.0513 | 16.3229 | 16.2472 | 16.3015 | 16.2095 | 32 | 33 | 37 | 37 | 28 | 35 | 36 | 31 |
| NDUFS4 | O43181 | 9.70E-08 | 3.14E-08 | -0.0519055 | | 0.154416 | -0.40319 | | 15.5568 | 15.5664 | 15.5516 | 15.6209 | 15.2931 | 15.7228 | 15.8717 | 15.5423 | 3 | 3 | 3 | 3 | 2 | 3 | 3 | 2 |
| SDF4 | Q9BRK5 | 1.82E-11 | 1.01E-11 | -0.051893 | | 0.350586 | -0.81524 | | 16.9815 | 17.0225 | 17.0107 | 16.9464 | 17.0184 | 17.2225 | 17.0068 | 17.0898 | 15 | 14 | 13 | 14 | 14 | 14 | 15 | 14 |
| MTFR1L | Q9H019;Q | 7.56E-13 | 5.63E-13 | -0.051848 | | 0.23857 | -0.58902 | | 15.7418 | 15.6466 | 15.677 | 15.6135 | 15.9109 | 15.7141 | 15.5966 | 15.8665 | 5 | 5 | 5 | 5 | 5 | 5 | 5 | 5 |
| ACTN4 | O43707 | 4.86E-19 | 2.39E-18 | -0.0518476 | | 0.13028 | -0.34645 | | 20.4024 | 20.4421 | 20.4108 | 20.0315 | 20.5978 | 20.6558 | 20.5331 | 20.5331 | 47 | 46 | 46 | 47 | 45 | 47 | 46 | 47 |
| CAMK1 | Q14012 | 3.48E-10 | 1.53E-10 | -0.0516401 | | 0.346671 | -0.80764 | | 15.4558 | 15.3825 | 15.492 | 15.5433 | 15.6138 | 15.3563 | 15.524 | 15.4381 | 10 | 10 | 10 | 10 | 10 | 9 | 10 | 9 |
| BMPER | Q8N8U9 | 1.14E-06 | 3.28E-07 | -0.0515783 | | 0.052102 | -0.14832 | | 14.2274 | 14.4008 | 14.4557 | 15.2353 | 14.49 | 13.9327 | 13.7963 | 14.61 | 8 | 10 | 9 | 11 | 6 | 5 | 4 | 6 |
| TASOR | Q9UK61;Q | 2.16E-05 | 5.50E-06 | -0.0514879 | | 0.21305 | -0.53439 | | 14.2771 | 14.4257 | 14.3767 | 14.2814 | 14.4371 | 14.2299 | 14.5245 | 14.5837 | 8 | 8 | 8 | 9 | 5 | 8 | 7 | 6 |
| PREB | Q9HCU5 | 1.23E-11 | 7.04E-12 | -0.0512512 | | 0.590539 | -1.25331 | | 16.1698 | 16.1811 | 16.1904 | 16.2904 | 16.2255 | 16.277 | 16.2471 | 16.1184 | 10 | 8 | 9 | 8 | 8 | 8 | 9 | 8 |
| CAPZB | P47756-2 | 3.44E-13 | 2.82E-13 | -0.0510146 | | 0.418657 | -0.94452 | | 19.4805 | 19.5514 | 19.5182 | 19.6797 | 19.4509 | 19.6187 | 19.5298 | 19.5594 | 27 | 27 | 27 | 25 | 25 | 25 | 26 | 25 |
| SAMD4B | Q5PRF9 | 5.29E-05 | 1.30E-05 | -0.0509498 | | 0.484729 | -1.06572 | | 15.7874 | 15.843 | 15.748 | 15.8995 | 15.8471 | 15.7386 | 15.8124 | 15.9213 | 11 | 14 | 12 | 11 | 9 | 12 | 10 | 12 |
| NCOR2 | Q9Y618;Q | 0.000256 | 5.73E-05 | -0.0505446 | | 0.305572 | -0.72666 | | 14.6976 | 14.6014 | 14.6307 | 14.5538 | 14.749 | 14.7292 | 14.6077 | 14.8289 | 12 | 15 | 13 | 12 | 13 | 10 | 14 | 13 |
| COMMD10 | Q9Y6G5 | 2.81E-14 | 3.12E-14 | -0.0504769 | | 0.06577 | -0.18481 | | 14.8908 | 14.9239 | 14.7908 | 15.1721 | 14.781 | 14.1772 | 15.2314 | 15.2333 | 8 | 7 | 7 | 8 | 2 | 3 | 6 | 7 |
| FAM50A | Q14320 | 2.00E-16 | 4.29E-16 | -0.0504524 | | 0.100182 | -0.27301 | | 15.8863 | 15.9494 | 15.9359 | 16.4025 | 15.8372 | 15.5758 | 15.9405 | 16.1154 | 11 | 12 | 11 | 12 | 11 | 11 | 12 | 12 |
| RNF31 | Q96EP0 | 1.76E-06 | 4.99E-07 | -0.0503017 | | 0.436742 | -0.97807 | | 15.4717 | 15.4442 | 15.4192 | 15.5167 | 15.6146 | 15.4892 | 15.38 | 15.4761 | 15 | 15 | 16 | 14 | 14 | 15 | 15 | 14 |
| UBR5 | O95071;O | 6.26E-15 | 8.40E-15 | -0.0501207 | | 0.190966 | -0.48598 | | 14.9564 | 15.0071 | 14.9949 | 15.076 | 15.2603 | 14.819 | 14.9176 | 15.1083 | 23 | 21 | 22 | 17 | 15 | 19 | 16 | 20 |
| P4HB | P07237 | 6.82E-15 | 9.05E-15 | -0.0498324 | | 0.207719 | -0.52281 | | 21.1854 | 21.193 | 21.1807 | 21.1823 | 21.3637 | 21.415 | 21.2078 | 21.0122 | 43 | 43 | 42 | 41 | 43 | 43 | 42 | 43 |
| SCFD1 | Q8WVM8 | 3.46E-13 | 2.83E-13 | -0.0497587 | | 0.203937 | -0.51455 | | 17.272 | 17.1979 | 17.2327 | 17.1048 | 17.3088 | 17.4586 | 17.4135 | 17.1341 | 22 | 22 | 23 | 20 | 18 | 22 | 23 | 22 |
| PSMC2 | P35998 | 6.21E-07 | 1.85E-07 | -0.0497375 | + | 1.41901 | -2.64848 | | 18.3317 | 18.3135 | 18.3224 | 18.3784 | 18.3833 | 18.3333 | 18.4138 | 18.3523 | 32 | 33 | 32 | 32 | 31 | 32 | 31 | 32 |
| UBE2G1 | P62253 | 0.000167 | 3.84E-05 | -0.0496045 | | 0.156252 | -0.40743 | | 14.9826 | 15.186 | 15.261 | 15.339 | 15.0346 | 15.0782 | 15.0861 | 15.426 | 5 | 6 | 5 | 4 | 4 | 5 | 4 | 4 |
| FKBP7 | Q9Y680;Q | 3.14E-10 | 1.39E-10 | -0.0495561 | | 0.044022 | -0.12632 | | 17.1369 | 17.1533 | 17.0726 | 16.3782 | 17.1583 | 17.5159 | 18.0615 | 16.7386 | 12 | 13 | 13 | 10 | 11 | 12 | 13 | 11 |
| PPFIA1 | Q13136;Q | 1.35E-06 | 3.88E-07 | -0.0495502 | | 0.375859 | -0.86384 | | 15.3318 | 15.3458 | 15.3429 | 15.3711 | 15.3462 | 15.4957 | 15.4738 | 15.2617 | 24 | 26 | 22 | 22 | 20 | 27 | 25 | 19 |
| NDUFA6 | P56556 | 3.17E-08 | 1.09E-08 | -0.0490765 | | 0.201298 | -0.50877 | | 16.4081 | 16.6236 | 16.4643 | 16.6093 | 16.7279 | 16.3881 | 16.5921 | 16.4213 | 4 | 4 | 4 | 4 | 3 | 4 | 5 | 5 |
| GALNT10 | Q86SR1 | 2.44E-17 | 6.92E-17 | -0.0489738 | | 0.08705 | -0.23993 | | 15.1394 | 15.0834 | 15.0921 | 14.9417 | 14.8507 | 15.3175 | 15.6772 | 14.9828 | 13 | 15 | 16 | 14 | 7 | 15 | 16 | 10 |
| BOP1 | Q14137 | 1.04E-06 | 3.02E-07 | -0.0481927 | | 0.25293 | -0.61918 | | 15.7412 | 15.707 | 15.6466 | 15.9413 | 15.6547 | 15.6211 | 15.733 | 15.7821 | 19 | 19 | 19 | 18 | 13 | 17 | 16 | 20 |
| RPF2 | Q9H7B2 | 4.17E-12 | 2.60E-12 | -0.0481809 | | 0.546206 | -1.17553 | | 16.8681 | 16.9411 | 16.9184 | 16.9097 | 17.0689 | 16.9327 | 16.9343 | 16.9413 | 9 | 9 | 9 | 8 | 6 | 9 | 8 | 7 |
| AKAP8 | O43823 | 1.43E-17 | 4.34E-17 | -0.0480942 | | 0.243752 | -0.59995 | | 14.3513 | 14.4307 | 14.6015 | 14.4629 | 14.674 | 14.4182 | 14.467 | 14.5242 | 5 | 4 | 7 | 4 | 3 | 3 | 4 | 4 |
| DPP7 | Q9UHL4 | 3.26E-10 | 1.43E-10 | -0.0479342 | | 0.068788 | -0.19275 | | 16.583 | 16.587 | 16.5963 | 16.4076 | 16.7655 | 16.4383 | 17.3069 | 16.2651 | 10 | 9 | 10 | 10 | 10 | 10 | 11 | 10 |
| NFKB2 | Q00653-4 | 2.00E-11 | 1.10E-11 | -0.0479202 | | 0.172254 | -0.44404 | | 15.3031 | 15.2948 | 15.2 | 15.2346 | 15.4391 | 15.456 | 15.0401 | 15.3996 | 19 | 21 | 20 | 17 | 14 | 20 | 20 | 21 |
| GMPR2 | Q9P2T1;Q | 9.52E-14 | 9.15E-14 | -0.0478889 | | 0.181587 | -0.46507 | | 16.3081 | 16.3489 | 16.1854 | 16.0632 | 16.4038 | 16.3651 | 16.4937 | 16.3178 | 9 | 9 | 9 | 10 | 8 | 9 | 9 | 9 |
| PRRC1 | Q96M27 | 5.90E-08 | 1.95E-08 | -0.0478294 | | 0.183818 | -0.47006 | | 18.2728 | 18.3235 | 18.3065 | 18.308 | 18.1998 | 18.5288 | 18.5252 | 18.1821 | 9 | 9 | 10 | 9 | 9 | 10 | 10 | 10 |
| HDHD3 | Q9BSH5 | 0.001855 | 0.000366 | -0.0476353 | | 0.201383 | -0.50896 | | 15.2072 | 14.9987 | 15.0995 | 15.0385 | 15.1331 | 15.2384 | 15.3353 | 15.002 | 2 | 4 | 3 | 3 | 1 | 2 | 2 | 1 |
| GOLGA2 | Q08379 | 2.45E-09 | 9.61E-10 | -0.0475353 | | 0.217045 | -0.54304 | | 15.9859 | 16.0085 | 15.9395 | 16.01 | 15.8693 | 16.0856 | 16.2382 | 15.9244 | 25 | 25 | 22 | 23 | 19 | 23 | 24 | 25 |
| METTL14 | Q9HCE5 | 1.20E-05 | 3.11E-06 | -0.0474148 | | 0.111868 | -0.30191 | | 14.9229 | 14.8615 | 15.2335 | 14.8185 | 15.3409 | 15.0094 | 14.8786 | 15.2196 | 5 | 5 | 5 | 5 | 2 | 4 | 3 | 4 |
| FKBP2 | P26885 | 0.000505 | 0.000109 | -0.0473231 | | 0.234499 | -0.58039 | | 18.1243 | 18.2381 | 18.221 | 18.2023 | 18.1301 | 18.3508 | 18.4051 | 18.1207 | 9 | 8 | 8 | 7 | 7 | 8 | 8 | 8 |
| HOOK3 | Q86VS8 | 6.73E-15 | 8.93E-15 | -0.0471658 | | 0.31991 | -0.75518 | | 16.2487 | 16.2149 | 16.2075 | 16.3606 | 16.1931 | 16.1827 | 16.4042 | 16.2136 | 27 | 25 | 27 | 26 | 23 | 25 | 27 | 24 |
| PPFIBP1 | Q86W92 | 0.000137 | 3.19E-05 | -0.0469796 | | 0.253708 | -0.6208 | | 15.7236 | 15.6266 | 15.839 | 15.6358 | 15.719 | 15.8294 | 15.9008 | 15.7985 | 3 | 4 | 4 | 4 | 3 | 3 | 3 | 3 |
| GOLGA3 | Q08378 | 3.03E-19 | 1.59E-18 | -0.0465502 | | 0.368903 | -0.85054 | | 15.7868 | 15.8272 | 15.8061 | 15.9116 | 15.7274 | 15.9275 | 15.9134 | 15.7861 | 60 | 57 | 62 | 60 | 49 | 58 | 54 | 56 |

| Gene names | UniProt IDs | Protein Group P-value | Protein Group Q-value | Log2 Difference NUP214 vs Controls (n=5) | p<0.05 | -log10 p-value | T-test Test statistic | NPC Localization | Log2 MS2_quantity_NUP214_1 | Log2 MS2_quantity_NUP214_2 | Log2 MS2_quantity_NUP214_3 | Log2 MS2_quantity_Control_1 | Log2 MS2_quantity_Control_2 | Log2 MS2_quantity_Control_3 | Log2 MS2_quantity_Control_4 | Log2 MS2_quantity_Control_5 | Number Peptides for Quantitation NUP214_1 | Number Peptides for Quantitation NUP214_2 | Number Peptides for Quantitation NUP214_3 | Number Peptides for Quantitation Control_1 | Number Peptides for Quantitation Control_2 | Number Peptides for Quantitation Control_3 | Number Peptides for Quantitation Control_4 | Number Peptides for Quantitation Control_5 |
|---|---|---|---|---|---|---|---|---|---|---|---|---|---|---|---|---|---|---|---|---|---|---|---|---|
| TAOK3 | Q9H2K8 | 2.35E-06 | 6.57E-07 | -0.0464888 | | 0.095914 | -0.26234 | | 15.6245 | 15.6852 | 15.7487 | 16.0799 | 15.9237 | 15.4535 | 15.4068 | 15.7991 | 19 | 16 | 18 | 17 | 16 | 16 | 18 | 15 |
| FUNDC2 | Q9BWH2 | 9.53E-07 | 2.78E-07 | -0.0463471 | | 0.216032 | -0.54085 | | 15.716 | 15.6173 | 15.6455 | 15.5631 | 15.6141 | 15.7584 | 15.918 | 15.6763 | 6 | 7 | 6 | 6 | 6 | 7 | 6 | 6 |
| RSF1 | Q96T23 | 2.44E-05 | 6.16E-06 | -0.0463315 | | 0.16691 | -0.43189 | | 15.6549 | 15.6448 | 15.6281 | 15.8788 | 15.7003 | 15.4421 | 15.8361 | 15.5873 | 10 | 12 | 10 | 15 | 7 | 11 | 9 | 11 |
| KPNA4 | O00629 | 9.49E-07 | 2.77E-07 | -0.0462735 | | 0.443484 | -0.9905 | | 17.54 | 17.5984 | 17.6282 | 17.6086 | 17.6105 | 17.5473 | 17.7348 | 17.6746 | 16 | 16 | 16 | 15 | 14 | 17 | 17 | 15 |
| NCDN | Q9UBB6;Q | 2.82E-12 | 1.82E-12 | -0.0461324 | | 0.34603 | -0.8064 | | 16.4426 | 16.4385 | 16.4007 | 16.6283 | 16.3818 | 16.4325 | 16.4354 | 16.489 | 18 | 16 | 19 | 17 | 18 | 18 | 19 | 18 |
| EIF5B | O60841 | 1.78E-10 | 8.21E-11 | -0.0460464 | | 0.341637 | -0.79784 | | 16.8063 | 16.8508 | 16.8618 | 16.9841 | 16.7447 | 16.9197 | 16.9399 | 16.8401 | 44 | 47 | 48 | 46 | 41 | 45 | 49 | 44 |
| VDR | P11473;P1 | 0.000742 | 0.000156 | -0.0459759 | | 0.092558 | -0.25389 | | 13.6717 | 13.6158 | 13.8225 | 13.7358 | 13.3558 | 14.1677 | 13.8326 | 13.6546 | 1 | 3 | 1 | 1 | 1 | 1 | 2 | 1 |
| THG1L | Q9NWX6 | 0.001291 | 0.000262 | -0.0459133 | | 0.100252 | -0.27319 | | 15.7847 | 15.7215 | 15.6632 | 15.9074 | 15.3477 | 16.1014 | 15.7766 | 15.7122 | 2 | 1 | 2 | 2 | 1 | 3 | 2 | 1 |
| DLGAP4 | Q9Y2H0;Q | 1.82E-12 | 1.23E-12 | -0.0458987 | | 0.233563 | -0.5784 | | 15.8967 | 15.8579 | 15.8616 | 16.1264 | 15.8797 | 15.8297 | 15.7938 | 15.9602 | 10 | 12 | 12 | 12 | 11 | 11 | 12 | 12 |
| ABCC10 | Q5T3U5;Q | 0.00279 | 0.000525 | -0.0452134 | | 0.048098 | -0.14036 | | 13.0855 | 12.6709 | 12.0987 | NaN | 12.3758 | 12.8684 | NaN | 12.7466 | 3 | 1 | 1 | NaN | 1 | 2 | NaN | 1 |
| WASHC2A | Q641Q2 | 1.95E-15 | 3.07E-15 | -0.0451043 | | 0.197867 | -0.50123 | | 15.741 | 15.6803 | 15.6608 | 15.7172 | 15.5791 | 15.853 | 15.9252 | 15.6212 | 25 | 24 | 27 | 24 | 22 | 24 | 24 | 23 |
| MLST8 | Q9BVC4 | 2.51E-11 | 1.35E-11 | -0.0449823 | | 0.174514 | -0.44915 | | 15.2623 | 15.196 | 15.0699 | 15.15 | 15.205 | 15.3286 | 15.4067 | 15.0151 | 6 | 6 | 6 | 5 | 5 | 5 | 5 | 5 |
| RPL26 | P61254 | 1.13E-05 | 2.95E-06 | -0.0448527 | | 0.274656 | -0.6641 | | 20.358 | 20.3818 | 20.2341 | 20.2059 | 20.4515 | 20.4055 | 20.354 | 20.4306 | 11 | 11 | 11 | 12 | 12 | 12 | 12 | 12 |
| EIF4ENIF1 | Q9NRA8;Q | 1.99E-05 | 5.08E-06 | -0.0447344 | | 0.15088 | -0.39499 | | 15.2602 | 15.1637 | 15.3207 | 15.5332 | 15.1763 | 15.0705 | 15.2873 | 15.3974 | 4 | 5 | 5 | 4 | 3 | 5 | 4 | 4 |
| HNRNPA1 | P09651;P0 | 1.02E-05 | 2.68E-06 | -0.0445845 | | 0.305046 | -0.7256 | | 20.443 | 20.4711 | 20.4491 | 20.5415 | 20.5475 | 20.3391 | 20.4628 | 20.6039 | 17 | 16 | 16 | 16 | 16 | 16 | 16 | 16 |
| ECE1 | P42892;P4 | 1.06E-15 | 1.80E-15 | -0.0445152 | | 0.065858 | -0.18504 | | 16.956 | 16.933 | 16.971 | 17.3923 | 17.1691 | 16.396 | 16.7895 | 17.2423 | 20 | 21 | 20 | 23 | 19 | 17 | 20 | 19 |
| GTF2E1 | P29083 | 7.09E-05 | 1.71E-05 | -0.0444607 | | 0.131464 | -0.34927 | | 15.6546 | 15.7255 | 15.5891 | 15.8786 | 15.8436 | 15.3619 | 15.6554 | 15.7647 | 4 | 5 | 4 | 4 | 4 | 3 | 4 | 4 |
| ZYG11B | Q9C0D3 | 1.32E-09 | 5.38E-10 | -0.0442539 | | 0.162261 | -0.42126 | | 14.6818 | 14.7141 | 14.6592 | 14.7129 | 15.0279 | 14.5689 | 14.6633 | 14.6735 | 7 | 7 | 7 | 7 | 7 | 5 | 7 | 7 |
| NEK7 | Q8TDX7 | 1.43E-14 | 1.74E-14 | -0.0442434 | | 0.074356 | -0.20729 | | 18.4758 | 18.5099 | 18.4251 | 18.1103 | 18.2776 | 18.9011 | 18.41 | 18.8737 | 16 | 16 | 16 | 15 | 15 | 16 | 15 | 16 |
| DNAJB2 | P25686 | 5.85E-05 | 1.43E-05 | -0.0441178 | | 0.095083 | -0.26025 | | 15.3133 | 15.3214 | 15.4541 | 15.5677 | 15.7213 | 15.1083 | 15.1172 | 15.5209 | 6 | 6 | 7 | 7 | 5 | 7 | 6 | 7 |
| C11orf54 | Q9H0W9;Q | 2.98E-13 | 2.49E-13 | -0.0436537 | | 0.039613 | -0.11418 | | 16.6192 | 16.642 | 16.6637 | 15.8997 | 16.9525 | 17.0697 | 17.3841 | 16.1206 | 8 | 7 | 9 | 7 | 6 | 9 | 9 | 7 |
| ATXN1 | P54253 | 0.001721 | 0.000342 | -0.0435584 | | 0.030106 | -0.09128 | | 14.3622 | 13.3429 | 13.6732 | NaN | NaN | 14.2097 | NaN | 13.4629 | 2 | 4 | 3 | NaN | NaN | 2 | NaN | 1 |
| DAD1 | P61803 | 0.000524 | 0.000112 | -0.0435504 | | 0.206368 | -0.51986 | | 18.7709 | 18.6715 | 18.7088 | 18.5523 | 18.7424 | 18.7782 | 18.9286 | 18.8015 | 4 | 4 | 4 | 4 | 4 | 4 | 4 | 4 |
| GCN1 | Q92616 | 5.55E-17 | 1.41E-16 | -0.0434577 | | 0.496466 | -1.08688 | | 17.4475 | 17.4585 | 17.436 | 17.5836 | 17.4393 | 17.4196 | 17.5275 | 17.4838 | 121 | 123 | 123 | 123 | 116 | 121 | 124 | 119 |
| CARD19 | Q96LW7-2 | 0.000251 | 5.63E-05 | -0.0434102 | | 0.068969 | -0.19322 | | 15.7031 | 15.3146 | 15.8363 | 15.3353 | 15.65 | 15.3516 | 15.9187 | 16.0516 | 6 | 6 | 6 | 6 | 6 | 6 | 6 | 6 |
| GSS | P48637 | 1.75E-11 | 9.72E-12 | -0.0433322 | | 0.728072 | -1.4891 | | 18.1601 | 18.225 | 18.1669 | 18.2542 | 18.2803 | 18.2293 | 18.193 | 18.1797 | 20 | 18 | 19 | 19 | 19 | 18 | 19 | 19 |
| FRG1 | Q14331 | 5.31E-11 | 2.69E-11 | -0.0432895 | | 0.118515 | -0.31812 | | 15.9916 | 16.0999 | 16.0609 | 16.2343 | 16.0871 | 15.7076 | 16.2451 | 16.1961 | 4 | 4 | 4 | 4 | 5 | 4 | 4 | 5 |
| OS9 | Q13438 | 5.46E-07 | 1.64E-07 | -0.0431826 | | 0.481133 | -1.05922 | | 15.5802 | 15.6524 | 15.5728 | 15.6279 | 15.6349 | 15.7414 | 15.6473 | 15.5734 | 10 | 9 | 9 | 8 | 7 | 11 | 10 | 9 |
| VPS16 | Q9H269 | 4.80E-07 | 1.45E-07 | -0.0431567 | | 0.246906 | -0.60657 | | 16.282 | 16.3141 | 16.333 | 16.3691 | 16.2484 | 16.4078 | 16.6524 | 16.2264 | 20 | 21 | 20 | 20 | 17 | 18 | 20 | 18 |
| NDUFS3 | O75489 | 1.82E-14 | 2.14E-14 | -0.0429751 | | 0.501024 | -1.09508 | | 17.8404 | 17.7159 | 17.7904 | 17.7985 | 17.8265 | 17.8997 | 17.8322 | 17.769 | 12 | 12 | 12 | 12 | 11 | 11 | 12 | 11 |
| RBMX2 | Q9Y388 | 4.47E-06 | 1.22E-06 | -0.0426973 | | 0.094112 | -0.25781 | | 13.3538 | 13.2958 | 13.3873 | 13.0088 | 13.5752 | 13.2279 | 13.4283 | 13.7014 | 2 | 2 | 2 | 1 | 2 | 2 | 2 | 2 |
| OSBPL8 | Q9BZF1;Q9 | 1.41E-11 | 7.95E-12 | -0.0426898 | | 0.270361 | -0.65528 | | 16.5429 | 16.5703 | 16.5904 | 16.6533 | 16.4845 | 16.7583 | 16.6281 | 16.5284 | 23 | 24 | 23 | 24 | 19 | 23 | 23 | 21 |
| PPAT | Q06203 | 5.10E-11 | 2.59E-11 | -0.0426121 | | 0.322561 | -0.76042 | | 15.08 | 15.0785 | 15.0802 | 15.2075 | 15.2151 | 14.9863 | 15.0955 | 15.1065 | 8 | 8 | 7 | 8 | 6 | 8 | 6 | 7 |
| ARF6 | P62330 | 3.76E-05 | 9.32E-06 | -0.0425559 | | 0.107837 | -0.292 | | 19.0119 | 18.9982 | 19.0767 | 19.4461 | 18.8423 | 19.1251 | 19.0716 | 18.8723 | 5 | 4 | 4 | 5 | 5 | 4 | 5 | 4 |
| ACOT7 | O00154;O | 1.44E-09 | 5.83E-10 | -0.0425036 | | 0.074935 | -0.20879 | | 18.2505 | 18.3444 | 18.2635 | 18.4999 | 18.6121 | 17.7488 | 18.331 | 18.4516 | 17 | 16 | 16 | 15 | 17 | 16 | 16 | 17 |
| COMMD2 | Q86X83;Q | 1.08E-12 | 7.76E-13 | -0.0424268 | | 0.232292 | -0.5757 | | 16.7612 | 16.641 | 16.7254 | 16.5772 | 16.8236 | 16.8778 | 16.7686 | 16.7108 | 6 | 6 | 6 | 5 | 5 | 5 | 6 | 5 |
| ASAP2 | O43150-2 | 3.92E-08 | 1.32E-08 | -0.0424024 | | 0.078281 | -0.21745 | | 15.2692 | 15.2202 | 15.2518 | 14.8574 | 15.6646 | 15.3764 | 15.4879 | 15.0611 | 13 | 13 | 14 | 8 | 10 | 12 | 9 | 10 |
| ENTPD6 | O75354;O | 0.002547 | 0.000485 | -0.0423453 | | 0.043775 | -0.12565 | | 16.2419 | 16.2145 | 15.9199 | 16.8374 | 15.7286 | 15.8951 | 15.6901 | 16.6877 | 1 | 2 | 1 | 3 | 1 | 1 | 1 | 3 |
| LAP3 | P28838;P2 | 5.01E-11 | 2.55E-11 | -0.0422791 | | 0.602853 | -1.27473 | | 18.1235 | 18.1348 | 18.1667 | 18.1518 | 18.1147 | 18.2446 | 18.2269 | 18.1816 | 29 | 29 | 29 | 30 | 27 | 28 | 29 | 29 |
| TBL2 | Q9Y4P3 | 8.70E-11 | 4.22E-11 | -0.0420572 | | 0.154466 | -0.4033 | | 16.4872 | 16.4789 | 16.4647 | 16.2685 | 16.6116 | 16.6416 | 16.6691 | 16.4041 | 23 | 23 | 23 | 22 | 22 | 23 | 23 | 22 |
| DPY19L4 | Q7Z388 | 0.002239 | 0.000434 | -0.0420436 | | 0.264514 | -0.64323 | | 15.5309 | 15.4104 | 15.5531 | 15.5192 | 15.472 | 15.5768 | 15.6858 | 15.4472 | 3 | 3 | 3 | 3 | 2 | 2 | 2 | 2 |
| PPP6R3 | Q5H9R7;Q | 1.55E-17 | 4.64E-17 | -0.0419035 | | 0.446575 | -0.99619 | | 15.5673 | 15.6091 | 15.6345 | 15.5867 | 15.7229 | 15.682 | 15.6694 | 15.5665 | 19 | 20 | 20 | 22 | 15 | 18 | 19 | 20 |
| RRP12 | Q5JTH9 | 6.85E-14 | 6.84E-14 | -0.041851 | | 0.666983 | -1.38522 | | 15.5592 | 15.5578 | 15.5024 | 15.5657 | 15.5561 | 15.6608 | 15.5751 | 15.5506 | 29 | 31 | 28 | 28 | 21 | 28 | 30 | 26 |
| ALG6 | Q9Y672 | 0.005535 | 0.000981 | -0.0418182 | | 0.066652 | -0.18713 | | 15.7689 | 15.7003 | 15.6443 | 15.846 | 15.1357 | 15.8618 | 16.1436 | 15.7445 | 3 | 3 | 3 | 2 | 1 | 3 | 3 | 2 |
| VPS35 | Q96QK1 | 5.15E-10 | 2.21E-10 | -0.0417912 | | 0.677809 | -1.40371 | | 18.9233 | 18.9626 | 18.926 | 18.9112 | 19.0406 | 18.9624 | 18.9965 | 18.9847 | 33 | 34 | 33 | 33 | 34 | 33 | 33 | 34 |
| SYVN1 | Q86TM6;Q | 7.16E-06 | 1.91E-06 | -0.0416077 | | 0.091784 | -0.25194 | | 16.0889 | 16.0748 | 15.9887 | 16.0914 | 15.6403 | 16.376 | 16.2073 | 16.147 | 6 | 6 | 7 | 6 | 4 | 6 | 6 | 6 |
| PARVA | Q9NVD7 | 4.52E-16 | 8.46E-16 | -0.0415421 | | 0.107112 | -0.29021 | | 17.8369 | 17.7807 | 17.8364 | 17.5797 | 17.704 | 18.2079 | 17.9223 | 17.8838 | 23 | 23 | 21 | 20 | 22 | 23 | 21 | 22 |
| PDIA3 | P30101 | 6.00E-19 | 2.83E-18 | -0.0414553 | | 0.35352 | -0.82092 | | 21.2748 | 21.3281 | 21.271 | 21.294 | 21.468 | 21.3419 | 21.3043 | 21.2556 | 43 | 43 | 43 | 42 | 43 | 43 | 43 | 43 |
| SEPTIN5 | Q99719 | 3.01E-05 | 7.54E-06 | -0.0414524 | | 0.223766 | -0.5575 | | 14.7128 | 14.8611 | 14.8337 | 14.813 | 14.7688 | 14.7312 | 15.0102 | 14.8967 | 6 | 6 | 7 | 2 | 7 | 4 | 9 | 6 |
| ACAP2 | Q15057 | 2.51E-18 | 9.62E-18 | -0.0413862 | | 0.145375 | -0.38214 | | 15.5537 | 15.5215 | 15.5153 | 15.4924 | 15.4259 | 15.7126 | 15.8138 | 15.4132 | 25 | 26 | 25 | 24 | 24 | 23 | 27 | 24 |
| ADAM10 | O14672 | 6.95E-18 | 2.35E-17 | -0.0412702 | | 0.074778 | -0.20838 | | 16.3553 | 16.3722 | 16.3824 | 16.7816 | 16.5458 | 16.0565 | 16.0637 | 16.6086 | 24 | 24 | 23 | 25 | 22 | 23 | 23 | 23 |
| ZC3H15 | Q8WU90 | 3.82E-14 | 4.08E-14 | -0.041254 | | 0.228911 | -0.5685 | | 16.5228 | 16.4729 | 16.4869 | 16.7151 | 16.4154 | 16.5928 | 16.4537 | 16.5002 | 14 | 16 | 15 | 14 | 12 | 14 | 13 | 14 |
| LYST | Q99698 | 0.008628 | 0.001491 | -0.0411568 | | 0.04067 | -0.1171 | | 14.2457 | 14.0865 | 14.0294 | 13.9555 | 14.2349 | 13.8882 | 15.1263 | 13.6036 | 4 | 2 | 2 | 3 | 2 | 3 | 3 | 1 |
| CCT4 | P50991 | 4.91E-16 | 9.04E-16 | -0.0410337 | | 0.4526 | -1.00725 | | 19.3816 | 19.3346 | 19.3315 | 19.4537 | 19.4433 | 19.3045 | 19.3401 | 19.4097 | 31 | 31 | 31 | 31 | 28 | 30 | 31 | 30 |
| CSNK2A2 | P19784 | 1.93E-07 | 6.06E-08 | -0.040988 | | 0.693698 | -1.43079 | | 16.6047 | 16.6103 | 16.6225 | 16.6178 | 16.7293 | 16.6513 | 16.6604 | 16.6088 | 15 | 15 | 16 | 15 | 17 | 15 | 17 | 16 |
| CASP6 | P55212 | 1.48E-20 | 1.25E-19 | -0.0409721 | | 0.161844 | -0.4203 | | 15.43 | 15.4439 | 15.4197 | 15.5009 | 15.6955 | 15.3357 | 15.2906 | 15.5381 | 8 | 5 | 7 | 8 | 7 | 6 | 6 | 8 |
| SSR3 | Q9UNL2;Q | 0.012195 | 0.002072 | -0.0406715 | | 0.13361 | -0.35438 | | 20.5364 | 20.5191 | 20.5235 | 20.4023 | 20.6974 | 20.3699 | 20.8211 | 20.5443 | 1 | 1 | 1 | 1 | 1 | 1 | 1 | 1 |
| DCAF7 | P61962 | 3.81E-11 | 1.97E-11 | -0.0404181 | | 0.452207 | -1.00653 | | 15.6563 | 15.737 | 15.6598 | 15.7982 | 15.6749 | 15.6702 | 15.7039 | 15.7767 | 10 | 9 | 9 | 9 | 7 | 10 | 9 | 9 |
| SOAT1 | P35610 | 4.30E-07 | 1.31E-07 | -0.0402815 | | 0.070402 | -0.19698 | | 16.2025 | 16.1143 | 16.1354 | 16.6147 | 16.0155 | 15.7915 | 16.0606 | 16.4727 | 9 | 9 | 11 | 11 | 9 | 8 | 11 | 9 |
| RPL17 | P18621;P1 | 7.93E-08 | 2.58E-08 | -0.0400661 | | 0.285449 | -0.68611 | | 19.4771 | 19.652 | 19.6011 | 19.6592 | 19.5689 | 19.7195 | 19.5307 | 19.6058 | 13 | 12 | 13 | 13 | 13 | 13 | 13 | 13 |
| SIPA1 | Q96FS4 | 5.28E-07 | 1.59E-07 | -0.040055 | | 0.081338 | -0.22532 | | 13.4692 | 13.7947 | 13.8252 | 14.1409 | 13.8444 | 13.467 | 13.6056 | 13.6241 | 3 | 6 | 5 | 6 | 1 | 3 | 5 | 4 |
| YWHAB | P31946 | 1.60E-10 | 7.45E-11 | -0.040016 | | 0.072878 | -0.20344 | | 20.6778 | 20.6565 | 20.7821 | 20.9704 | 20.7733 | 20.1857 | 20.8144 | 20.9837 | 15 | 15 | 14 | 14 | 15 | 14 | 14 | 14 |
| ARPC2 | O15144 | 2.56E-24 | 7.58E-23 | -0.039961 | | 0.354816 | -0.82343 | | 19.4569 | 19.526 | 19.5125 | 19.6125 | 19.4598 | 19.6298 | 19.4918 | 19.4984 | 20 | 21 | 22 | 22 | 21 | 22 | 22 | 22 |
| CPT1A | P50416 | 2.51E-15 | 3.83E-15 | -0.0397967 | | 0.125098 | -0.33403 | | 17.0424 | 17.1518 | 17.1145 | 17.4758 | 16.9705 | 17.0517 | 17.0748 | 17.1408 | 24 | 24 | 24 | 23 | 21 | 24 | 24 | 22 |
| PNPO | Q9NVS9;Q | 3.62E-12 | 2.29E-12 | -0.0396932 | | 0.073688 | -0.20555 | | 16.6492 | 16.7588 | 16.8124 | 16.7651 | 16.2697 | 17.015 | 17.0795 | 16.77 | 11 | 11 | 10 | 10 | 7 | 11 | 11 | 10 |
| CRK | P46108 | 1.90E-17 | 5.52E-17 | -0.0396926 | | 0.323786 | -0.76283 | | 17.998 | 17.9328 | 18.0268 | 18.0711 | 17.9427 | 18.0966 | 18.0838 | 17.9337 | 15 | 15 | 15 | 15 | 15 | 15 | 15 | 16 |
| PFKM | P08237;P0 | 3.35E-11 | 1.76E-11 | -0.0392836 | | 0.327884 | -0.77091 | | 16.8529 | 16.8286 | 16.8284 | 16.9902 | 16.7547 | 16.8844 | 16.8972 | 16.8528 | 20 | 20 | 20 | 19 | 21 | 20 | 21 | 19 |
| FIBP | O43427;O | 5.85E-09 | 2.19E-09 | -0.0392468 | | 0.353791 | -0.82145 | | 15.3874 | 15.2567 | 15.3085 | 15.3079 | 15.338 | 15.456 | 15.2966 | 15.3854 | 8 | 9 | 8 | 10 | 8 | 8 | 9 | 8 |
| LMBR1 | Q8WVP7;Q | 1.92E-13 | 1.69E-13 | -0.0392423 | | 0.103641 | -0.28162 | | 16.3882 | 16.1462 | 16.3209 | 16.6502 | 16.3905 | 16.0801 | 16.3055 | 16.1952 | 2 | 2 | 2 | 2 | 2 | 2 | 2 | 1 |
| CCNH | P51946 | 3.20E-05 | 8.00E-06 | -0.0390179 | | 0.114234 | -0.3077 | | 14.9244 | 15.1887 | 15.19 | 14.93 | 15.0181 | 15.1598 | 15.1819 | 15.4102 | 3 | 2 | 4 | 5 | 4 | 3 | 4 | 4 |
| LUC7L3 | O95232 | 0.000344 | 7.58E-05 | -0.0389968 | | 0.125754 | -0.33561 | | 15.9133 | 16.0111 | 15.9904 | 16.0305 | 15.7415 | 16.0472 | 16.2747 | 15.959 | 6 | 7 | 6 | 6 | 5 | 7 | 6 | 6 |
| DNM2 | P50570 | 5.16E-12 | 3.16E-12 | -0.0387211 | | 0.70051 | -1.44237 | | 17.5178 | 17.5335 | 17.4704 | 17.5577 | 17.5824 | 17.5124 | 17.4983 | 17.579 | 40 | 40 | 42 | 38 | 39 | 41 | 40 | 38 |
| SPHK2 | Q9NRA0;Q | 4.62E-08 | 1.55E-08 | -0.0385841 | | 0.041061 | -0.11818 | | 14.4019 | 14.7667 | 14.6908 | 14.6697 | 15.1071 | 13.7573 | 14.964 | 14.7937 | 4 | 3 | 3 | 4 | 4 | 1 | 4 | 4 |
| EIF3H | O15372 | 5.40E-12 | 3.30E-12 | -0.0384467 | | 0.140631 | -0.371 | | 17.6143 | 17.6772 | 17.6915 | 17.9475 | 17.538 | 17.551 | 17.6761 | 17.7846 | 16 | 16 | 16 | 17 | 16 | 17 | 17 | 17 |
| MEPCE | Q7L2J0 | 5.21E-05 | 1.28E-05 | -0.038428 | | 0.21887 | -0.54697 | | 15.3869 | 15.5966 | 15.5499 | 15.6624 | 15.6208 | 15.4517 | 15.4967 | 15.5161 | 6 | 6 | 6 | 5 | 5 | 5 | 4 | 7 |
| PPP4R1 | Q8TF05;Q8 | 1.22E-13 | 1.13E-13 | -0.0383966 | | 0.15805 | -0.41158 | | 16.1247 | 16.0897 | 16.1133 | 16.3378 | 16.2206 | 16.0052 | 15.9685 | 16.2061 | 22 | 23 | 22 | 21 | 22 | 23 | 22 | 23 |
| ARHGEF12 | Q9NZN5 | 1.92E-13 | 1.69E-13 | -0.0383156 | | 0.112457 | -0.30335 | | 15.2588 | 15.3475 | 15.3078 | 15.5508 | 15.5107 | 15.0486 | 15.218 | 15.387 | 31 | 25 | 27 | 29 | 22 | 21 | 24 | 21 |
| IQGAP1 | P46940 | 2.05E-13 | 1.80E-13 | -0.0381933 | | 0.275303 | -0.66542 | | 19.4386 | 19.468 | 19.4629 | 19.5411 | 19.4201 | 19.4505 | 19.641 | 19.4207 | 100 | 100 | 98 | 102 | 97 | 99 | 100 | 98 |

| Gene names | UniProt IDs | Protein Group P-value | Protein Group Q-value | Log2 Difference NUP214 vs Controls (n=5) | p<0.05 | -log10 p-value | T-test Test statistic | NPC Localization | Log2 MS2_quantity_NUP214_1 | Log2 MS2_quantity_NUP214_2 | Log2 MS2_quantity_NUP214_3 | Log2 MS2_quantity_Control_1 | Log2 MS2_quantity_Control_2 | Log2 MS2_quantity_Control_3 | Log2 MS2_quantity_Control_4 | Log2 MS2_quantity_Control_5 | Number Peptides for Quantitation NUP214_1 | Number Peptides for Quantitation NUP214_2 | Number Peptides for Quantitation NUP214_3 | Number Peptides for Quantitation Control_1 | Number Peptides for Quantitation Control_2 | Number Peptides for Quantitation Control_3 | Number Peptides for Quantitation Control_4 | Number Peptides for Quantitation Control_5 |
|---|---|---|---|---|---|---|---|---|---|---|---|---|---|---|---|---|---|---|---|---|---|---|---|---|
| ALG1 | Q9BT22 | 1.66E-15 | 2.67E-15 | -0.0381813 | | 0.103724 | -0.28183 | | 16.4363 | 16.4152 | 16.3759 | 16.1245 | 16.4188 | 16.5249 | 16.7517 | 16.4168 | 9 | 10 | 10 | 9 | 10 | 9 | 10 | 9 |
| BCS1L | Q9Y276 | 2.30E-09 | 9.05E-10 | -0.0379278 | | 0.195817 | -0.49671 | | 15.8455 | 15.9185 | 15.8158 | 15.9918 | 15.7727 | 15.7722 | 15.9153 | 16.0372 | 10 | 11 | 12 | 10 | 7 | 11 | 10 | 9 |
| APAF1 | O14727;O1 | 3.75E-07 | 1.15E-07 | -0.0379075 | | 0.184686 | -0.472 | | 15.0523 | 15.03 | 14.9828 | 15.1457 | 14.8529 | 15.1411 | 15.1582 | 15.0001 | 15 | 18 | 18 | 18 | 14 | 18 | 16 | 15 |
| ACSL4 | O60488;O6 | 3.20E-12 | 2.05E-12 | -0.03745 | | 0.090271 | -0.24811 | | 17.036 | 17.0758 | 17.0513 | 17.3857 | 17.3008 | 16.7652 | 16.9634 | 17.0437 | 25 | 26 | 27 | 26 | 25 | 24 | 24 | 25 |
| CDK2 | P24941;P2 | 5.49E-05 | 1.34E-05 | -0.0374236 | | 0.055656 | -0.15789 | | 15.259 | 15.2917 | 15.2779 | 15.5785 | 15.1102 | 14.7181 | 15.474 | 15.6872 | 11 | 10 | 9 | 10 | 9 | 10 | 10 | 10 |
| RPL4 | P36578 | 4.27E-09 | 1.63E-09 | -0.0371305 | | 0.140268 | -0.37015 | | 19.2827 | 19.5667 | 19.276 | 19.4423 | 19.4139 | 19.445 | 19.2156 | 19.5444 | 27 | 28 | 27 | 27 | 28 | 27 | 27 | 27 |
| ZFYVE1 | Q9HBF4;Q9 | 9.68E-13 | 7.03E-13 | -0.0367946 | | 0.214594 | -0.53774 | | 15.1544 | 15.1565 | 15.1414 | 15.2198 | 15.3623 | 15.1839 | 15.0746 | 15.097 | 11 | 13 | 12 | 12 | 10 | 13 | 13 | 10 |
| SLC9A3R2 | Q15599 | 7.20E-16 | 1.28E-15 | -0.0366891 | | 0.048426 | -0.13836 | | 15.283 | 15.2558 | 15.2174 | 15.1028 | 15.4398 | 14.6896 | 15.8986 | 15.313 | 11 | 12 | 11 | 11 | 12 | 8 | 13 | 11 |
| EMC6 | Q9BV81 | 7.89E-11 | 3.85E-11 | -0.0366355 | | 0.166905 | -0.43188 | | 17.3817 | 17.3836 | 17.4369 | 17.6002 | 17.5046 | 17.2186 | 17.4283 | 17.4354 | 2 | 2 | 2 | 2 | 2 | 2 | 2 | 2 |
| GLUD1 | P00367 | 1.11E-13 | 1.04E-13 | -0.0366075 | | 0.102384 | -0.2785 | | 18.9417 | 18.9074 | 18.9081 | 19.2254 | 18.9639 | 18.7911 | 18.6901 | 19.1078 | 24 | 24 | 24 | 25 | 23 | 24 | 23 | 24 |
| PMPCA | Q10713 | 1.49E-06 | 4.26E-07 | -0.0365827 | | 0.268215 | -0.65087 | | 16.3875 | 16.4246 | 16.43 | 16.4246 | 16.5409 | 16.4143 | 16.3272 | 16.546 | 21 | 22 | 21 | 21 | 17 | 19 | 19 | 19 |
| RPL23 | P62829 | 5.21E-05 | 1.28E-05 | -0.0364457 | | 0.183495 | -0.46934 | | 20.6146 | 20.7996 | 20.678 | 20.5715 | 20.6735 | 20.8194 | 20.844 | 20.761 | 8 | 8 | 8 | 8 | 8 | 8 | 7 | 8 |
| AGFG1 | P52594;P5 | 4.98E-15 | 6.89E-15 | -0.0364199 | | 0.108183 | -0.29285 | | 16.219 | 16.2499 | 16.1728 | 16.5321 | 16.1145 | 16.4092 | 16.0712 | 16.1247 | 9 | 9 | 9 | 10 | 9 | 9 | 9 | 9 |
| PSMD4 | P55036 | 2.68E-10 | 1.20E-10 | -0.0363819 | | 0.271148 | -0.6569 | | 17.8047 | 17.7569 | 17.7386 | 17.6959 | 17.9429 | 17.8126 | 17.773 | 17.791 | 13 | 12 | 12 | 12 | 13 | 12 | 13 | 13 |
| NRBP2 | Q9NSY0 | 7.93E-14 | 7.77E-14 | -0.0362725 | | 0.126162 | -0.33659 | | 15.4148 | 15.3409 | 15.316 | 15.423 | 15.6487 | 15.1651 | 15.3125 | 15.4183 | 10 | 8 | 9 | 10 | 9 | 10 | 8 | 8 |
| KIF13A | Q9H1H9;Q | 2.20E-10 | 1.00E-10 | -0.0359866 | | 0.1724 | -0.44437 | | 15.424 | 15.4576 | 15.4461 | 15.6057 | 15.6305 | 15.3266 | 15.3777 | 15.4521 | 29 | 29 | 29 | 29 | 26 | 27 | 28 | 29 |
| PDXDC1 | Q6P996;Q6 | 5.20E-14 | 5.37E-14 | -0.0358125 | | 0.167069 | -0.43225 | | 16.9865 | 16.9851 | 16.9992 | 16.8527 | 17.0619 | 17.0215 | 17.2307 | 16.9637 | 29 | 27 | 27 | 26 | 24 | 26 | 26 | 25 |
| WDR61 | Q9GZS3 | 1.47E-17 | 4.44E-17 | -0.0355939 | | 0.200468 | -0.50695 | | 16.1128 | 16.1094 | 16.0411 | 15.9908 | 16.1445 | 16.1386 | 16.2925 | 16.0504 | 15 | 15 | 15 | 15 | 13 | 15 | 15 | 14 |
| POLR2C | P19387 | 1.05E-05 | 2.74E-06 | -0.0355818 | | 0.247492 | -0.6078 | | 15.6897 | 15.6071 | 15.7424 | 15.6894 | 15.8137 | 15.6266 | 15.6497 | 15.7974 | 11 | 11 | 11 | 10 | 11 | 11 | 12 | 11 |
| THOP1 | P52888 | 3.56E-11 | 1.86E-11 | -0.0353926 | | 0.300568 | -0.71663 | | 16.4532 | 16.4868 | 16.441 | 16.4571 | 16.617 | 16.4017 | 16.4769 | 16.5258 | 22 | 24 | 23 | 23 | 21 | 21 | 23 | 22 |
| RWDD2B | P57060 | 1.10E-08 | 4.00E-09 | -0.035283 | | 0.30273 | -0.72097 | | 14.7917 | 14.8204 | 14.9022 | 14.8423 | 14.7682 | 14.9054 | 14.8948 | 14.9563 | 2 | 2 | 2 | 2 | 1 | 2 | 2 | 2 |
| CNBP | P62633;P6 | 8.24E-07 | 2.42E-07 | -0.0352805 | | 0.09808 | -0.26776 | | 18.5149 | 18.6911 | 18.7162 | 18.8657 | 18.9216 | 18.508 | 18.4682 | 18.6165 | 6 | 6 | 5 | 5 | 5 | 5 | 5 | 5 |
| NIFK | Q9BYG3 | 5.58E-11 | 2.81E-11 | -0.0352645 | | 0.19163 | -0.48745 | | 16.0112 | 16.013 | 15.9747 | 16.0674 | 15.8232 | 16.1132 | 16.1058 | 16.0649 | 12 | 12 | 13 | 13 | 10 | 12 | 11 | 11 |
| ACTR8 | Q9H981 | 2.82E-06 | 7.82E-07 | -0.0349288 | | 0.051237 | -0.14598 | | 14.1119 | 14.0435 | 14.0428 | 13.6788 | 14.4821 | 13.7742 | 14.0177 | 14.5524 | 4 | 5 | 5 | 3 | 5 | 2 | 3 | 5 |
| BOLA3 | Q53S33 | 0.00242 | 0.000464 | -0.034822 | | 0.044655 | -0.12806 | | 14.064 | 14.0497 | 14.0446 | 14.6922 | 14.1213 | 13.5688 | 13.7206 | 14.3349 | 3 | 3 | 2 | 4 | 2 | 2 | 2 | 2 |
| C12orf4 | Q9NQ89 | 4.49E-08 | 1.50E-08 | -0.0348029 | | 0.308081 | -0.73167 | | 15.6113 | 15.5767 | 15.5238 | 15.5461 | 15.6876 | 15.6013 | 15.6705 | 15.5215 | 9 | 8 | 8 | 6 | 4 | 7 | 8 | 6 |
| KIN | O60870 | 5.77E-09 | 2.17E-09 | -0.0347916 | | 0.109574 | -0.29627 | | 14.3033 | 14.2922 | 14.1201 | 14.4237 | 14.1063 | 14.0983 | 14.2379 | 14.5005 | 3 | 5 | 5 | 6 | 2 | 4 | 5 | 6 |
| PSMC1 | P62191 | 1.30E-12 | 9.11E-13 | -0.0347593 | | 0.368077 | -0.84895 | | 18.1575 | 18.1434 | 18.1696 | 18.2562 | 18.1024 | 18.258 | 18.1473 | 18.1941 | 22 | 23 | 23 | 23 | 23 | 23 | 23 | 22 |
| PGPEP1 | Q9NXJ5 | 3.97E-09 | 1.52E-09 | -0.0347464 | | 0.092928 | -0.25482 | | 15.8745 | 15.8137 | 15.8734 | 15.9651 | 15.5999 | 15.789 | 16.2162 | 15.8728 | 3 | 4 | 4 | 3 | 3 | 4 | 4 | 3 |
| HLCS | P50747 | 1.08E-07 | 3.47E-08 | -0.0347392 | | 0.088444 | -0.24348 | | 15.6641 | 15.6292 | 15.5993 | 15.4943 | 15.3512 | 15.8288 | 15.927 | 15.727 | 6 | 8 | 8 | 7 | 6 | 7 | 6 | 6 |
| MRPS14 | O60783 | 1.82E-08 | 6.42E-09 | -0.0345694 | | 0.093758 | -0.25692 | | 15.6914 | 15.5399 | 15.5932 | 15.7052 | 15.9663 | 15.425 | 15.6628 | 15.4544 | 2 | 2 | 2 | 2 | 3 | 2 | 3 | 2 |
| MRPL47 | Q9HD33;Q | 0.000659 | 0.00014 | -0.0345295 | | 0.15592 | -0.40667 | | 16.3193 | 16.2339 | 16.2927 | 16.3408 | 16.4154 | 16.3137 | 16.0835 | 16.4289 | 5 | 5 | 8 | 7 | 6 | 6 | 6 | 5 |
| CCT2 | P78371 | 1.49E-13 | 1.34E-13 | -0.0344307 | | 0.217699 | -0.54445 | | 19.1259 | 19.1199 | 19.0605 | 19.2255 | 19.2423 | 19.0501 | 19.0119 | 19.1529 | 38 | 38 | 38 | 38 | 39 | 38 | 38 | 38 |
| RNF14 | Q9UBS8 | 1.70E-20 | 1.41E-19 | -0.0342082 | | 0.236065 | -0.58371 | | 16.865 | 16.9477 | 16.8844 | 16.8676 | 17.0826 | 16.9424 | 16.8428 | 16.9309 | 11 | 11 | 12 | 12 | 10 | 11 | 11 | 11 |
| PDCD2L | Q9BRP1 | 4.70E-12 | 2.90E-12 | -0.0339262 | | 0.033023 | -0.09582 | | 14.473 | 14.3755 | 14.4983 | 15.0127 | 13.5633 | 14.9802 | 14.3235 | 14.5346 | 2 | 2 | 2 | 2 | 1 | 2 | 2 | 2 |
| NUB1 | Q9Y5A7;Q9 | 1.55E-18 | 6.35E-18 | -0.0338692 | | 0.126643 | -0.33774 | | 15.1349 | 15.1982 | 15.2047 | 14.944 | 15.2915 | 15.3654 | 15.1703 | 15.2945 | 11 | 9 | 9 | 9 | 7 | 10 | 8 | 10 |
| PSMB7 | Q99436 | 4.05E-06 | 1.11E-06 | -0.0337234 | | 0.174716 | -0.4496 | | 18.3707 | 18.3679 | 18.3477 | 18.361 | 18.2201 | 18.5565 | 18.4645 | 18.3772 | 7 | 7 | 8 | 7 | 7 | 7 | 8 | 7 |
| RARS2 | Q5T160 | 3.24E-16 | 6.32E-16 | -0.0337152 | | 0.226601 | -0.56357 | | 16.5796 | 16.5658 | 16.5787 | 16.5102 | 16.6276 | 16.7287 | 16.6739 | 16.5017 | 20 | 20 | 18 | 17 | 15 | 17 | 18 | 16 |
| XPNPEP1 | Q9NQW7-3 | 1.80E-13 | 1.59E-13 | -0.0334629 | | 0.125507 | -0.33501 | | 17.2868 | 17.3507 | 17.2991 | 17.3111 | 17.234 | 17.6184 | 17.3658 | 17.199 | 31 | 29 | 30 | 30 | 27 | 31 | 29 | 30 |
| HSPB1 | P04792 | 8.69E-07 | 2.54E-07 | -0.0334574 | | 0.112989 | -0.30465 | | 22.2737 | 22.281 | 22.2609 | 22.0258 | 22.2123 | 22.4003 | 22.4681 | 22.4201 | 17 | 17 | 18 | 17 | 18 | 18 | 18 | 19 |
| DAXX | Q9UER7 | 0.000587 | 0.000125 | -0.0334052 | | 0.065819 | -0.18494 | | 15.0908 | 15.2112 | 15.2464 | 15.0722 | 15.6689 | 14.8881 | 15.1253 | 15.3264 | 3 | 3 | 3 | 2 | 2 | 2 | 1 | 2 |
| FAF2 | Q96CS3 | 1.32E-12 | 9.22E-13 | -0.0333936 | | 0.20041 | -0.50682 | | 17.7415 | 17.8288 | 17.7978 | 17.803 | 17.7095 | 17.9673 | 17.8902 | 17.7436 | 14 | 14 | 14 | 14 | 14 | 14 | 14 | 14 |
| SEC24D | O94855;O9 | 3.86E-11 | 2.00E-11 | -0.0333216 | | 0.091716 | -0.25177 | | 17.8936 | 17.9605 | 17.8978 | 17.8247 | 17.7631 | 18.2542 | 18.1152 | 17.7958 | 30 | 30 | 29 | 27 | 27 | 30 | 31 | 29 |
| RIPK1 | Q13546 | 1.44E-15 | 2.37E-15 | -0.0330897 | | 0.195503 | -0.49602 | | 18.2758 | 18.24 | 18.2979 | 18.4095 | 18.3777 | 18.279 | 18.1289 | 18.3265 | 18 | 17 | 20 | 17 | 12 | 17 | 16 | 16 |
| CTSF | Q9UBX1 | 0.002746 | 0.000518 | -0.0330873 | | 0.049966 | -0.14374 | | 16.395 | 16.3742 | 16.4372 | 16.7846 | NaN | 15.9233 | 16.351 | 16.682 | 1 | 2 | 2 | 1 | NaN | 1 | 2 | 2 |
| TRAPPC1 | Q9Y5R8 | 7.42E-09 | 2.75E-09 | -0.0330711 | | 0.145224 | -0.38179 | | 16.8077 | 16.8275 | 16.8858 | 16.9963 | 16.8355 | 16.6616 | 16.8605 | 17.0132 | 7 | 7 | 7 | 6 | 5 | 6 | 6 | 7 |
| FNBP1L | Q5T0N5;Q5 | 0.000625 | 0.000133 | -0.0330286 | | 0.238021 | -0.58785 | | 14.298 | 14.2334 | 14.2912 | 14.3868 | 14.3636 | 14.3684 | 14.2217 | 14.1958 | 4 | 6 | 8 | 9 | 4 | 7 | 5 | 5 |
| SLC30A9 | Q6PML9 | 1.11E-08 | 4.00E-09 | -0.0328453 | | 0.13495 | -0.35757 | | 15.3253 | 15.2345 | 15.2529 | 15.1047 | 15.3063 | 15.4567 | 15.4403 | 15.2107 | 12 | 11 | 11 | 10 | 8 | 8 | 10 | 9 |
| ERP29 | P30040 | 2.85E-16 | 5.66E-16 | -0.0328247 | | 0.06385 | -0.17973 | | 20.2873 | 20.1962 | 20.229 | 19.856 | 20.4625 | 20.353 | 20.6101 | 20.0701 | 14 | 15 | 14 | 14 | 13 | 14 | 14 | 14 |
| RALB | P11234;P1 | 5.46E-14 | 5.60E-14 | -0.032718 | | 0.160509 | -0.41724 | | 17.7671 | 17.7709 | 17.8059 | 18.0218 | 17.8635 | 17.7086 | 17.7386 | 17.7375 | 12 | 11 | 12 | 11 | 10 | 11 | 12 | 11 |
| CACTIN | Q8WUQ7;C | 0.002366 | 0.000455 | -0.0326384 | | 0.045895 | -0.13145 | | 13.7762 | 13.6515 | 13.8441 | 14.2776 | 13.8885 | 13.1524 | 13.7176 | 13.9135 | 5 | 5 | 4 | 6 | 2 | 1 | 3 | 2 |
| PPP2R5E | Q16537;Q1 | 1.21E-13 | 1.13E-13 | -0.0325573 | | 0.277198 | -0.6693 | | 16.8083 | 16.7559 | 16.7527 | 16.827 | 16.8984 | 16.6829 | 16.7918 | 16.8241 | 11 | 11 | 11 | 11 | 10 | 9 | 11 | 11 |
| PRDX2 | P32119 | 4.91E-08 | 1.64E-08 | -0.0324975 | | 0.041405 | -0.11912 | | 19.8225 | 19.8142 | 19.7972 | 19.1447 | 20.3136 | 19.7701 | 20.1904 | 19.8001 | 13 | 13 | 14 | 13 | 13 | 13 | 13 | 14 |
| RPS26 | P62854 | 0.00136 | 0.000275 | -0.0324537 | | 0.326959 | -0.76909 | | 21.1011 | 21.1628 | 21.1077 | 21.2046 | 21.1386 | 21.0469 | 21.1918 | 21.1996 | 3 | 4 | 3 | 4 | 3 | 3 | 5 | 3 |
| MFN1 | Q8IWA4 | 8.62E-06 | 2.28E-06 | -0.0323263 | | 0.091797 | -0.25197 | | 15.504 | 15.4903 | 15.4827 | 15.4953 | 15.7917 | 15.2096 | 15.4907 | 15.6359 | 12 | 13 | 14 | 12 | 11 | 10 | 10 | 13 |
| C2orf76 | Q3KRA6 | 0.01287 | 0.002179 | -0.0321776 | | 0.067461 | -0.19332 | | 15.7666 | 15.599 | 15.7225 | NaN | 15.4239 | NaN | 15.8023 | 15.9585 | 2 | 3 | 3 | NaN | 1 | NaN | 1 | 1 |
| NME7 | Q9Y5B8;Q9 | 7.94E-10 | 3.32E-10 | -0.0320709 | | 0.239379 | -0.59073 | | 15.6788 | 15.6579 | 15.5983 | 15.545 | 15.7257 | 15.708 | 15.6421 | 15.7647 | 10 | 10 | 10 | 10 | 8 | 9 | 8 | 7 |
| COL1A2 | P08123 | 5.91E-16 | 1.06E-15 | -0.0320428 | | 0.024292 | -0.07114 | | 19.5724 | 19.5817 | 19.6104 | 18.8477 | 19.198 | 20.528 | 20.33 | 19.1973 | 59 | 60 | 60 | 59 | 52 | 62 | 59 | 56 |
| MICU2 | Q8IYU8 | 3.53E-09 | 1.36E-09 | -0.0320417 | | 0.100797 | -0.27455 | | 15.2035 | 15.1648 | 15.1897 | 15.442 | 15.2263 | 15.2074 | 14.9103 | 15.3044 | 7 | 7 | 7 | 7 | 5 | 7 | 7 | 7 |
| HADHB | P55084;P5 | 4.30E-09 | 1.64E-09 | -0.0318637 | | 0.255028 | -0.62355 | | 18.8962 | 18.9194 | 18.8817 | 18.9585 | 18.8085 | 19.0433 | 18.9141 | 18.9304 | 27 | 27 | 27 | 27 | 26 | 26 | 27 | 26 |
| TSEN34 | Q9BSV6 | 0.008494 | 0.00147 | -0.0318525 | | 0.144611 | -0.38035 | | 14.7621 | 14.7999 | 14.8004 | 14.6684 | 15.0308 | 14.7303 | 14.8012 | 14.8659 | 4 | 5 | 4 | 4 | 4 | 2 | 3 | 3 |
| TRMT6 | Q9UJA5 | 2.56E-10 | 1.15E-10 | -0.0318298 | | 0.088114 | -0.24264 | | 15.0677 | 15.1568 | 15.1033 | 15.3279 | 14.7935 | 15.2733 | 15.0659 | 15.2447 | 6 | 8 | 7 | 9 | 8 | 7 | 8 | 6 |
| ITSN1 | Q15811;Q1 | 7.99E-14 | 7.82E-14 | -0.0316484 | | 0.111012 | -0.29981 | | 15.7971 | 15.7756 | 15.8123 | 15.8918 | 15.6827 | 15.9845 | 15.9764 | 15.5978 | 18 | 20 | 19 | 20 | 13 | 18 | 18 | 17 |
| NAT10 | Q9H0A0 | 5.59E-10 | 2.39E-10 | -0.0315922 | | 0.25373 | -0.62085 | | 16.1361 | 16.1631 | 16.1525 | 16.2624 | 16.0893 | 16.1369 | 16.1403 | 16.2817 | 26 | 25 | 28 | 28 | 20 | 25 | 26 | 25 |
| VPS33B | Q9H267 | 2.86E-19 | 1.53E-18 | -0.0314171 | | 0.515516 | -1.12103 | | 15.9108 | 15.9784 | 15.9015 | 15.9683 | 15.9332 | 16.019 | 15.9603 | 15.9274 | 18 | 21 | 19 | 18 | 17 | 18 | 17 | 19 |
| OSBPL2 | Q9H1P3;Q9 | 2.69E-09 | 1.05E-09 | -0.0312848 | | 0.099235 | -0.27065 | | 15.2021 | 15.21 | 15.1509 | 15.074 | 15.0874 | 15.4562 | 15.4012 | 15.076 | 6 | 6 | 7 | 7 | 7 | 7 | 5 | 6 |
| ATG4B | Q9Y4P1;Q9 | 6.20E-21 | 5.98E-20 | -0.0311861 | | 0.232666 | -0.5765 | | 15.6245 | 15.5438 | 15.6058 | 15.5612 | 15.624 | 15.5597 | 15.6 | 15.7679 | 8 | 8 | 7 | 6 | 6 | 7 | 6 | 6 |
| CDK11B | P21127;P2 | 4.42E-08 | 1.48E-08 | -0.0309759 | | 0.152249 | -0.39817 | | 15.7983 | 15.8609 | 15.8353 | 16.0419 | 15.8648 | 15.7731 | 15.9202 | 15.7122 | 15 | 14 | 13 | 17 | 12 | 15 | 14 | 13 |
| RBCK1 | Q9BYM8 | 9.17E-11 | 4.43E-11 | -0.0308091 | | 0.256276 | -0.62615 | | 15.5532 | 15.5226 | 15.5735 | 15.5016 | 15.6247 | 15.6826 | 15.4967 | 15.5971 | 15 | 14 | 13 | 14 | 12 | 13 | 12 | 12 |
| PDHX | O00330;O0 | 3.19E-12 | 2.05E-12 | -0.0306278 | | 0.149984 | -0.3929 | | 15.6248 | 15.5135 | 15.4904 | 15.5114 | 15.7438 | 15.4403 | 15.6458 | 15.5262 | 10 | 9 | 10 | 11 | 7 | 8 | 10 | 9 |
| ZDHHC13 | Q8IUH4 | 2.21E-05 | 5.60E-06 | -0.0303492 | | 0.128578 | -0.34238 | | 15.3845 | 15.5304 | 15.5179 | 15.7008 | 15.591 | 15.3537 | 15.4421 | 15.4523 | 5 | 5 | 3 | 5 | 3 | 3 | 3 | 3 |
| MON2 | Q7Z3U7;Q7 | 6.92E-17 | 1.71E-16 | -0.0302858 | | 0.248767 | -0.61048 | | 16.6137 | 16.6115 | 16.5902 | 16.7241 | 16.5291 | 16.624 | 16.7117 | 16.5881 | 42 | 39 | 37 | 38 | 32 | 35 | 42 | 37 |
| PLXNA1 | Q9UIW2 | 1.38E-09 | 5.60E-10 | -0.0299627 | | 0.089469 | -0.24608 | | 15.5109 | 15.4695 | 15.4464 | 15.7734 | 15.3492 | 15.6652 | 15.3142 | 15.4257 | 28 | 25 | 29 | 27 | 21 | 26 | 21 | 23 |
| CLN3 | Q13286;Q1 | 1.14E-12 | 8.16E-13 | -0.0299437 | | 0.04416 | -0.1267 | | 15.6057 | 15.4168 | 15.7418 | 15.0089 | 15.5562 | 15.8448 | 15.9985 | 15.6818 | 3 | 5 | 4 | 4 | 2 | 2 | 4 | 3 |
| EPS15 | P42566 | 4.67E-13 | 3.68E-13 | -0.0297748 | | 0.166264 | -0.43042 | | 15.5892 | 15.5768 | 15.6394 | 15.7566 | 15.592 | 15.7212 | 15.6185 | 15.4697 | 27 | 29 | 27 | 27 | 23 | 27 | 26 | 26 |
| TRIM56 | Q9BRZ2 | 4.28E-15 | 6.07E-15 | -0.029773 | | 0.262026 | -0.63809 | | 15.9009 | 15.9442 | 15.9662 | 15.9583 | 15.9024 | 16.0914 | 15.9165 | 15.9655 | 11 | 12 | 12 | 11 | 11 | 12 | 11 | 14 |
| EIF2AK2 | P19525 | 1.27E-15 | 2.11E-15 | -0.0297716 | | 0.209385 | -0.52644 | | 17.1489 | 17.1878 | 17.1905 | 17.1281 | 17.2393 | 17.1792 | 17.3514 | 17.1295 | 18 | 18 | 18 | 19 | 18 | 18 | 18 | 18 |
| GET3 | O43681 | 2.42E-07 | 7.56E-08 | -0.0297462 | | 0.427827 | -0.96157 | | 18.6013 | 18.7005 | 18.6466 | 18.63 | 18.6814 | 18.7358 | 18.6836 | 18.6654 | 13 | 13 | 13 | 13 | 13 | 13 | 13 | 13 |

| Gene names | UniProt IDs | Protein Group P-value | Protein Group Q-value | Log2 Difference NUP214 vs Controls (n=5) | p<0.05 | -log10 p-value | T-test Test statistic | NPC Localization | Log2 MS2_quantity_NUP214_1 | Log2 MS2_quantity_NUP214_2 | Log2 MS2_quantity_NUP214_3 | Log2 MS2_quantity_Control_1 | Log2 MS2_quantity_Control_2 | Log2 MS2_quantity_Control_3 | Log2 MS2_quantity_Control_4 | Log2 MS2_quantity_Control_5 | Number Peptides for Quantitation NUP214_1 | Number Peptides for Quantitation NUP214_2 | Number Peptides for Quantitation NUP214_3 | Number Peptides for Quantitation Control_1 | Number Peptides for Quantitation Control_2 | Number Peptides for Quantitation Control_3 | Number Peptides for Quantitation Control_4 | Number Peptides for Quantitation Control_5 |
|---|---|---|---|---|---|---|---|---|---|---|---|---|---|---|---|---|---|---|---|---|---|---|---|---|
| SMG8 | Q8ND04;Q | 2.24E-11 | 1.22E-11 | -0.0296862 | | 0.087184 | -0.24027 | | 14.9568 | 14.9292 | 14.8998 | 14.8686 | 14.6543 | 15.1889 | 15.0797 | 14.9999 | 11 | 11 | 12 | 10 | 5 | 12 | 12 | 7 |
| EIF2B4 | Q9UI10 | 7.06E-10 | 2.97E-10 | -0.0296384 | | 0.166962 | -0.43201 | | 16.8087 | 16.7816 | 16.8077 | 16.6313 | 16.8752 | 16.8957 | 16.9114 | 16.8314 | 13 | 12 | 13 | 12 | 13 | 12 | 13 | 13 |
| TRIM26 | Q12899 | 1.34E-12 | 9.33E-13 | -0.0295666 | | 0.104665 | -0.28416 | | 14.7304 | 14.8434 | 14.8397 | 14.8768 | 14.9729 | 14.5569 | 14.9562 | 14.8076 | 11 | 13 | 12 | 12 | 8 | 10 | 9 | 9 |
| PEX1 | O43933 | 8.73E-07 | 2.56E-07 | -0.0295355 | | 0.065766 | -0.1848 | | 14.6199 | 14.6679 | 14.7252 | 14.8982 | 14.2727 | 14.8212 | 14.6153 | 14.8952 | 5 | 7 | 4 | 4 | 5 | 6 | 4 | 5 |
| NF1 | P21359;P2 | 6.09E-11 | 3.05E-11 | -0.0293259 | | 0.305624 | -0.72676 | | 14.8854 | 14.9695 | 14.9729 | 15.0745 | 14.9555 | 14.9494 | 14.9342 | 14.9458 | 28 | 30 | 24 | 26 | 21 | 23 | 21 | 21 |
| VIPAS39 | Q9H9C1 | 4.75E-18 | 1.71E-17 | -0.0292174 | | 0.133565 | -0.35428 | | 15.2366 | 15.1744 | 15.3179 | 15.2501 | 15.1168 | 15.4487 | 15.3446 | 15.2006 | 14 | 14 | 14 | 13 | 10 | 12 | 11 | 11 |
| ZNF622 | Q969S3 | 2.14E-13 | 1.86E-13 | -0.029081 | | 0.105242 | -0.28559 | | 15.1077 | 15.0979 | 15.1238 | 15.3538 | 15.0665 | 15.2882 | 14.991 | 14.9949 | 11 | 10 | 11 | 12 | 6 | 10 | 11 | 10 |
| MBD2 | Q9UBB5 | 1.56E-15 | 2.52E-15 | -0.0290457 | | 0.115861 | -0.31166 | | 15.6529 | 15.5715 | 15.5427 | 15.8186 | 15.6145 | 15.4178 | 15.6934 | 15.5461 | 5 | 6 | 5 | 5 | 4 | 4 | 4 | 4 |
| TMEM161A | Q9NX61;Q | 1.93E-12 | 1.29E-12 | -0.0290222 | | 0.128627 | -0.34249 | | 15.3046 | 15.3022 | 15.3495 | 15.406 | 15.4928 | 15.1145 | 15.3556 | 15.3701 | 1 | 2 | 2 | 2 | 2 | 2 | 2 | 2 |
| PPIG | Q13427;Q1 | 7.77E-06 | 2.06E-06 | -0.028991 | | 0.097687 | -0.26678 | | 15.3067 | 15.4922 | 15.5517 | 15.2907 | 15.6758 | 15.4992 | 15.5774 | 15.3528 | 6 | 8 | 8 | 7 | 4 | 5 | 6 | 5 |
| FCHO2 | Q0JRZ9 | 3.12E-09 | 1.21E-09 | -0.0289309 | | 0.338606 | -0.79193 | | 15.7263 | 15.7509 | 15.8233 | 15.8697 | 15.7809 | 15.8036 | 15.7311 | 15.7934 | 14 | 15 | 15 | 15 | 13 | 12 | 13 | 14 |
| MSH6 | P52701 | 1.82E-17 | 5.31E-17 | -0.0287788 | | 0.074709 | -0.2082 | | 15.3098 | 15.3842 | 15.3732 | 15.4056 | 15.4252 | 15.0405 | 15.3652 | 15.686 | 18 | 21 | 19 | 22 | 19 | 18 | 19 | 20 |
| MRPL19 | P49406 | 3.61E-05 | 8.96E-06 | -0.0286664 | | 0.162553 | -0.42193 | | 16.8237 | 16.8321 | 16.7754 | 16.9382 | 16.9351 | 16.7155 | 16.7221 | 16.8843 | 8 | 8 | 8 | 8 | 7 | 8 | 6 | 7 |
| FUBP3 | Q96I24 | 2.19E-10 | 9.97E-11 | -0.0285613 | | 0.243422 | -0.59925 | | 17.4009 | 17.3836 | 17.4034 | 17.3917 | 17.5355 | 17.358 | 17.4797 | 17.3578 | 20 | 21 | 21 | 22 | 20 | 20 | 20 | 21 |
| MFN2 | O95140 | 1.66E-06 | 4.71E-07 | -0.0284098 | | 0.122633 | -0.32809 | | 15.6194 | 15.6043 | 15.4767 | 15.765 | 15.4354 | 15.64 | 15.4835 | 15.6521 | 18 | 16 | 16 | 17 | 13 | 17 | 14 | 18 |
| CDC73 | Q6P1J9 | 1.95E-09 | 7.76E-10 | -0.0281868 | | 0.208096 | -0.52363 | | 15.6485 | 15.6452 | 15.5992 | 15.7402 | 15.6284 | 15.5215 | 15.6826 | 15.7229 | 16 | 17 | 18 | 17 | 11 | 15 | 16 | 15 |
| ASCC3 | Q8N3C0 | 4.29E-22 | 5.57E-21 | -0.0281432 | | 0.236275 | -0.58416 | | 15.9118 | 15.9614 | 16.0087 | 16.0458 | 15.9657 | 15.9258 | 16.0847 | 15.922 | 63 | 62 | 65 | 66 | 50 | 56 | 61 | 53 |
| C17orf75 | Q9HAS0 | 1.68E-05 | 4.31E-06 | -0.028133 | | 0.096873 | -0.26474 | | 15.6669 | 15.6902 | 15.72 | 15.4701 | 15.9426 | 15.765 | 15.6367 | 15.7882 | 5 | 6 | 8 | 7 | 5 | 4 | 4 | 5 |
| SLC12A9 | Q9BXP2 | 2.59E-16 | 5.30E-16 | -0.0280718 | | 0.0784 | -0.21776 | | 16.5173 | 16.4859 | 16.4702 | 16.2964 | 16.7851 | 16.3449 | 16.6999 | 16.4698 | 8 | 8 | 7 | 7 | 6 | 7 | 7 | 7 |
| PDCL | Q13371 | 7.86E-05 | 1.88E-05 | -0.027896 | | 0.051691 | -0.14721 | | 15.5521 | 15.6298 | 15.5524 | 15.6727 | 16.0802 | 15.2322 | 15.4306 | 15.6143 | 4 | 3 | 3 | 6 | 2 | 3 | 3 | 3 |
| CYP51A1 | Q16850 | 1.93E-11 | 1.07E-11 | -0.0278372 | | 0.03852 | -0.11115 | | 16.8739 | 16.9198 | 16.8879 | 17.3509 | 16.9088 | 16.2431 | 16.9405 | 17.1652 | 19 | 19 | 18 | 19 | 17 | 17 | 18 | 18 |
| HSD17B12 | Q53GQ0 | 4.01E-11 | 2.07E-11 | -0.0277122 | | 0.180421 | -0.46245 | | 18.3292 | 18.4114 | 18.366 | 18.4697 | 18.3783 | 18.3098 | 18.5205 | 18.3047 | 14 | 13 | 13 | 13 | 13 | 13 | 13 | 13 |
| POLR2G | P62487 | 6.28E-11 | 3.13E-11 | -0.027401 | | 0.107978 | -0.29234 | | 16.0229 | 16.0448 | 16.055 | 15.8941 | 16.2307 | 15.9699 | 16.0099 | 16.237 | 5 | 5 | 5 | 5 | 4 | 4 | 5 | 4 |
| BORCS5 | Q969J3 | 6.44E-12 | 3.89E-12 | -0.0273387 | | 0.055602 | -0.15775 | | 15.1532 | 14.9568 | 15.4258 | 15.2835 | 14.9382 | 14.9669 | 15.4198 | 15.4212 | 4 | 5 | 6 | 4 | 4 | 4 | 5 | 5 |
| TBC1D9B | Q66K14;Q | 1.09E-08 | 3.95E-09 | -0.0272931 | | 0.241028 | -0.59421 | | 16.185 | 16.1682 | 16.2095 | 16.1816 | 16.1376 | 16.2043 | 16.2107 | 16.34 | 27 | 27 | 27 | 28 | 20 | 25 | 25 | 27 |
| EPN1 | Q9Y6I3;Q9 | 1.07E-12 | 7.67E-13 | -0.0272926 | | 0.206814 | -0.52084 | | 16.8104 | 16.8978 | 16.8891 | 16.9987 | 16.8009 | 16.9541 | 16.8629 | 16.8488 | 9 | 8 | 9 | 9 | 8 | 8 | 8 | 9 |
| ATG101 | Q9BSB4 | 0.00137 | 0.000277 | -0.0272593 | | 0.039622 | -0.1142 | | 14.9069 | 14.8456 | 14.889 | 14.9548 | 14.2287 | 15.2736 | 15.0978 | 14.9838 | 4 | 4 | 4 | 4 | 1 | 4 | 3 | 4 |
| STAT3 | P40763;P4 | 1.64E-15 | 2.65E-15 | -0.0272086 | | 0.073817 | -0.20588 | | 16.5859 | 16.5031 | 16.4897 | 16.786 | 16.315 | 16.7571 | 16.3569 | 16.5523 | 26 | 25 | 24 | 26 | 25 | 26 | 25 | 25 |
| MRPL15 | Q9P015 | 1.55E-18 | 6.35E-18 | -0.0271177 | | 0.135035 | -0.35777 | | 16.2966 | 16.2879 | 16.2405 | 16.3112 | 16.4631 | 16.1744 | 16.1819 | 16.3798 | 13 | 12 | 12 | 13 | 10 | 13 | 12 | 12 |
| DDX41 | Q9UJV9 | 2.19E-06 | 6.13E-07 | -0.0271009 | | 0.092694 | -0.25423 | | 15.0722 | 15.0611 | 15.1134 | 14.9573 | 15.3756 | 14.9856 | 15.0221 | 15.2061 | 8 | 12 | 10 | 10 | 6 | 10 | 6 | 5 |
| RPS27L | Q71UM5 | 2.35E-12 | 1.55E-12 | -0.0270714 | | 0.077971 | -0.21665 | | 19.9251 | 19.8775 | 19.9494 | 19.7685 | 20.2777 | 20.0145 | 19.823 | 19.8383 | 5 | 5 | 5 | 5 | 5 | 5 | 5 | 5 |
| PEX16 | Q9Y5Y5;Q9 | 4.15E-06 | 1.13E-06 | -0.0270366 | | 0.101518 | -0.27634 | | 15.1298 | 15.0733 | 15.009 | 14.84 | 15.2623 | 15.1226 | 15.1762 | 15.0875 | 6 | 5 | 6 | 5 | 6 | 5 | 5 | 5 |
| SERPINB1 | P30740 | 1.26E-15 | 2.10E-15 | -0.0269245 | | 0.199818 | -0.50552 | | 17.4447 | 17.4153 | 17.4492 | 17.5757 | 17.4867 | 17.507 | 17.369 | 17.3781 | 17 | 17 | 17 | 17 | 17 | 17 | 17 | 17 |
| UNC93B1 | Q9H1C4 | 0.002747 | 0.000518 | -0.0269186 | | 0.035283 | -0.103 | | 15.504 | 15.5776 | 15.2933 | 15.0429 | 15.6386 | 16.0045 | NaN | 15.2549 | 1 | 1 | 1 | 1 | 2 | 1 | NaN | 1 |
| TTC37 | Q6PGP7 | 1.02E-17 | 3.28E-17 | -0.0268955 | | 0.093999 | -0.25752 | | 16.0444 | 16.0042 | 16.0222 | 15.8969 | 16.1218 | 16.0813 | 16.2895 | 15.863 | 42 | 45 | 44 | 38 | 33 | 42 | 42 | 38 |
| EXOC5 | O00471 | 7.01E-12 | 4.21E-12 | -0.026828 | | 0.392934 | -0.89626 | | 16.1413 | 16.177 | 16.2004 | 16.2208 | 16.1257 | 16.2035 | 16.2485 | 16.2001 | 20 | 22 | 23 | 23 | 20 | 18 | 20 | 19 |
| PES1 | O00541;O | 1.85E-09 | 7.39E-10 | -0.0264858 | | 0.193378 | -0.49132 | | 16.5812 | 16.6272 | 16.6563 | 16.7226 | 16.5542 | 16.5559 | 16.7232 | 16.6844 | 14 | 14 | 14 | 15 | 12 | 12 | 13 | 13 |
| PPP1R10 | Q96QC0 | 1.79E-08 | 6.32E-09 | -0.0260976 | | 0.096872 | -0.26474 | | 15.998 | 15.9705 | 15.9977 | 16.1105 | 16.1536 | 15.7596 | 15.9384 | 16.112 | 15 | 14 | 16 | 11 | 10 | 10 | 12 | 12 |
| PGAM1 | P18669 | 9.61E-16 | 1.66E-15 | -0.0259899 | | 0.107498 | -0.29116 | | 20.8724 | 20.9041 | 20.9172 | 21.0389 | 20.7406 | 21.1103 | 20.8702 | 20.8596 | 19 | 19 | 19 | 20 | 20 | 20 | 20 | 20 |
| GNA13 | Q14344 | 7.03E-08 | 2.30E-08 | -0.0259799 | | 0.142549 | -0.37551 | | 16.4178 | 16.4585 | 16.4842 | 16.5107 | 16.391 | 16.6342 | 16.5148 | 16.3468 | 14 | 16 | 16 | 16 | 12 | 15 | 15 | 15 |
| FBXO22 | Q8NEZ5 | 1.09E-05 | 2.84E-06 | -0.0259747 | | 0.075675 | -0.21071 | | 16.3292 | 16.3357 | 16.2965 | 16.0723 | 16.4717 | 16.2544 | 16.6114 | 16.3224 | 6 | 6 | 5 | 6 | 6 | 6 | 6 | 6 |
| SRSF11 | Q05519;Q | 3.47E-08 | 1.18E-08 | -0.0258265 | | 0.12906 | -0.34353 | | 17.8131 | 17.9394 | 17.8913 | 17.7774 | 18.0789 | 17.8272 | 17.9578 | 17.8941 | 8 | 8 | 8 | 7 | 6 | 7 | 6 | 7 |
| VCL | P18206;P1 | 2.88E-13 | 2.42E-13 | -0.0258183 | | 0.145295 | -0.38195 | | 20.2802 | 20.2538 | 20.221 | 20.4366 | 20.3405 | 20.1566 | 20.2097 | 20.2439 | 86 | 85 | 82 | 83 | 79 | 83 | 83 | 86 |
| UGDH | O60701 | 1.02E-14 | 1.29E-14 | -0.0257224 | | 0.045962 | -0.13164 | | 19.473 | 19.4985 | 19.443 | 19.3398 | 19.2602 | 20.0628 | 19.4907 | 19.3325 | 33 | 34 | 34 | 32 | 28 | 32 | 32 | 32 |
| STT3A | P46977 | 1.06E-12 | 7.60E-13 | -0.025589 | | 0.072642 | -0.20283 | | 19.1485 | 19.1295 | 19.1589 | 19.0229 | 19.0374 | 19.4379 | 19.3625 | 18.9952 | 22 | 22 | 22 | 22 | 22 | 22 | 22 | 22 |
| DDRGK1 | Q96HY6 | 6.38E-05 | 1.55E-05 | -0.0255596 | | 0.093664 | -0.25668 | | 17.885 | 17.774 | 17.8641 | 17.6716 | 17.9073 | 18.0001 | 18.03 | 17.7238 | 8 | 8 | 9 | 8 | 8 | 8 | 8 | 8 |
| SNX7 | Q9UNH6;Q | 0.0002 | 4.54E-05 | -0.0253902 | | 0.080539 | -0.22327 | | 15.1874 | 15.2658 | 15.2576 | 15.4119 | 15.1139 | 15.4923 | 15.2409 | 15.0526 | 8 | 8 | 8 | 8 | 5 | 7 | 8 | 6 |
| MICALL1 | Q8N3F8 | 8.97E-14 | 8.66E-14 | -0.0252672 | | 0.046376 | -0.13277 | | 15.0471 | 15.1657 | 15.165 | 15.6289 | 14.8897 | 15.2076 | 14.8399 | 15.1898 | 13 | 13 | 15 | 18 | 14 | 12 | 9 | 16 |
| RPL14 | P50914 | 9.27E-12 | 5.42E-12 | -0.0250484 | | 0.223779 | -0.55752 | | 21.5166 | 21.6193 | 21.5223 | 21.6135 | 21.6677 | 21.5113 | 21.5642 | 21.5322 | 5 | 5 | 5 | 5 | 5 | 5 | 6 | 5 |
| QPCTL | Q9NXS2 | 5.38E-05 | 1.32E-05 | -0.0246181 | | 0.038866 | -0.11211 | | 15.666 | 15.8067 | 15.5377 | 15.541 | 15.6963 | 15.4119 | 16.3047 | 15.5199 | 4 | 4 | 4 | 5 | 3 | 4 | 5 | 2 |
| UBFD1 | O14562 | 6.97E-09 | 2.58E-09 | -0.0245237 | | 0.049623 | -0.14161 | | 17.2226 | 17.2338 | 17.1245 | 17.0294 | 16.8522 | 17.5968 | 17.3357 | 17.2767 | 9 | 9 | 8 | 7 | 9 | 8 | 10 | 8 |
| CSNK2B | P67870 | 3.07E-07 | 9.50E-08 | -0.0244965 | | 0.05101 | -0.14537 | | 17.6595 | 17.541 | 17.7722 | 17.9094 | 17.2165 | 17.7871 | 17.7954 | 17.7019 | 4 | 4 | 4 | 5 | 5 | 5 | 5 | 5 |
| EIF2A | Q9BY44 | 7.65E-23 | 1.33E-21 | -0.0242779 | | 0.205328 | -0.51759 | | 16.9974 | 16.999 | 16.999 | 17.0322 | 17.1066 | 16.9734 | 17.0851 | 16.9165 | 26 | 27 | 27 | 26 | 24 | 25 | 28 | 25 |
| PFN1 | P07737 | 2.46E-18 | 9.51E-18 | -0.0242029 | | 0.045693 | -0.1309 | | 22.903 | 22.8689 | 22.8409 | 22.8901 | 23.3753 | 22.853 | 22.5106 | 22.8466 | 16 | 15 | 16 | 16 | 14 | 16 | 15 | 16 |
| CCDC22 | O60826 | 5.18E-11 | 2.63E-11 | -0.0241366 | | 0.130489 | -0.34695 | | 16.5658 | 16.6076 | 16.4853 | 16.4985 | 16.586 | 16.703 | 16.6562 | 16.4416 | 20 | 19 | 21 | 21 | 18 | 21 | 20 | 19 |
| CDC23 | Q9UJX2 | 8.79E-09 | 3.22E-09 | -0.0237049 | | 0.077219 | -0.21471 | | 14.663 | 14.6908 | 14.8167 | 14.5342 | 14.7797 | 14.8807 | 14.9415 | 14.5999 | 10 | 12 | 11 | 9 | 7 | 10 | 11 | 9 |
| RIOK2 | Q9BVS4 | 1.81E-15 | 2.89E-15 | -0.023651 | | 0.065964 | -0.18532 | | 15.5551 | 15.597 | 15.5017 | 15.3071 | 15.7618 | 15.3881 | 15.684 | 15.7337 | 8 | 8 | 7 | 7 | 6 | 7 | 6 | 7 |
| PCYT1A | P49585 | 6.96E-18 | 2.35E-17 | -0.023549 | | 0.268704 | -0.65187 | | 17.38 | 17.3547 | 17.3241 | 17.4328 | 17.4147 | 17.331 | 17.3009 | 17.403 | 12 | 12 | 12 | 12 | 12 | 12 | 12 | 12 |
| COG2 | Q14746;Q1 | 9.78E-16 | 1.68E-15 | -0.023361 | | 0.08012 | -0.22219 | | 15.7421 | 15.6798 | 15.6808 | 15.6081 | 15.5716 | 15.8673 | 15.9555 | 15.619 | 16 | 17 | 16 | 12 | 12 | 13 | 13 | 15 |
| POR | P16435 | 1.06E-12 | 7.60E-13 | -0.0232295 | | 0.111267 | -0.30043 | | 17.527 | 17.6434 | 17.5868 | 17.67 | 17.7511 | 17.5574 | 17.6388 | 17.4273 | 27 | 29 | 30 | 28 | 29 | 30 | 30 | 29 |
| HMG20A | Q9NP66 | 0.001061 | 0.000218 | -0.0231648 | | 0.047005 | -0.13448 | | 14.0276 | 14.1134 | 14.055 | 13.7506 | 14.526 | 13.9417 | 14.0831 | 14.1411 | 4 | 3 | 3 | 3 | 3 | 4 | 3 | 3 |
| LTN1 | O94822;O | 4.07E-15 | 5.82E-15 | -0.0231021 | | 0.155591 | -0.40591 | | 15.2569 | 15.3034 | 15.2691 | 15.336 | 15.1536 | 15.412 | 15.2986 | 15.2975 | 35 | 29 | 32 | 29 | 24 | 25 | 29 | 29 |
| CYBC1 | Q9BQA9;Q | 0.000538 | 0.000115 | -0.0230913 | | 0.134814 | -0.35724 | | 16.2724 | 16.3703 | 16.1908 | 16.1783 | 16.3178 | 16.4065 | 16.3478 | 16.2542 | 5 | 5 | 5 | 5 | 5 | 5 | 5 | 5 |
| IQSEC1 | Q6DN90;Q | 7.53E-17 | 1.84E-16 | -0.0230529 | | 0.059901 | -0.16925 | | 15.2844 | 15.263 | 15.2646 | 15.1181 | 15.6887 | 15.1672 | 15.2222 | 15.2724 | 10 | 12 | 12 | 11 | 10 | 10 | 8 | 9 |
| TELO2 | Q9Y4R8 | 1.81E-10 | 8.38E-11 | -0.0228982 | | 0.062877 | -0.17716 | | 15.055 | 15.0074 | 14.9353 | 15.2569 | 14.8788 | 14.8728 | 14.8498 | 15.2525 | 13 | 14 | 14 | 17 | 9 | 12 | 13 | 14 |
| MYD88 | Q99836;Q | 2.54E-10 | 1.14E-10 | -0.0226715 | | 0.069648 | -0.195 | | 16.1118 | 16.149 | 16.2113 | 16.2931 | 16.3155 | 15.881 | 16.0931 | 16.3175 | 9 | 10 | 10 | 11 | 9 | 10 | 12 | 10 |
| MOB1B;MOB1B;MOB | Q7L9L4;Q7 | 5.70E-06 | 1.53E-06 | -0.0226311 | | 0.137363 | -0.36329 | | 17.5364 | 17.5503 | 17.6301 | 17.7503 | 17.613 | 17.513 | 17.5095 | 17.5888 | 6 | 6 | 6 | 6 | 7 | 7 | 7 | 6 |
| SPEN | Q96T58 | 0.001361 | 0.000275 | -0.0226292 | | 0.04839 | -0.13826 | | 14.5959 | 14.3759 | 14.3546 | 14.2658 | 14.2499 | 14.376 | 14.5649 | 14.8673 | 8 | 5 | 6 | 5 | 4 | 4 | 5 | 6 |
| ZSWIM8 | A7E2V4;A7 | 0.002195 | 0.000426 | -0.0225526 | | 0.074978 | -0.2089 | | 14.8793 | 14.8718 | 15.0383 | 14.9957 | 15.1557 | 14.7235 | 15.0373 | 14.8495 | 8 | 7 | 8 | 8 | 5 | 8 | 9 | 6 |
| TRRAP | Q9Y4A5;Q | 4.02E-11 | 2.07E-11 | -0.0225515 | | 0.141524 | -0.3731 | | 15.2131 | 15.1661 | 15.1248 | 15.148 | 15.2201 | 15.0501 | 15.3059 | 15.2289 | 19 | 16 | 22 | 10 | 9 | 6 | 13 | 9 |
| UQCRFS1 | P47985 | 1.37E-09 | 5.56E-10 | -0.0224781 | | 0.082441 | -0.22816 | | 17.8864 | 17.8739 | 17.8303 | 17.8543 | 18.0679 | 17.6293 | 17.9694 | 17.9092 | 6 | 6 | 6 | 6 | 6 | 6 | 6 | 6 |
| HPS6 | Q86YV9 | 0.0023 | 0.000444 | -0.0223288 | | 0.129668 | -0.34499 | | 15.2739 | 15.2736 | 15.187 | 15.3194 | 15.4153 | 15.2028 | 15.2441 | 15.1542 | 10 | 11 | 11 | 12 | 9 | 11 | 11 | 9 |
| RSU1 | Q15404 | 3.81E-06 | 1.04E-06 | -0.0221639 | | 0.036815 | -0.10641 | | 19.4708 | 19.3888 | 19.3859 | 19.4299 | 18.9081 | 19.8628 | 19.4033 | 19.5826 | 12 | 12 | 12 | 12 | 12 | 12 | 12 | 12 |
| ETFDH | Q16134 | 6.46E-17 | 1.61E-16 | -0.0221354 | | 0.119361 | -0.32017 | | 15.9346 | 15.9498 | 15.9004 | 15.8633 | 15.8667 | 16.0232 | 16.1173 | 15.8814 | 16 | 15 | 17 | 16 | 12 | 16 | 17 | 16 |
| RALA | P11233 | 0.000293 | 6.51E-05 | -0.0221321 | | 0.046827 | -0.134 | | 18.6479 | 18.6364 | 18.5937 | 19.1258 | 18.4224 | 18.5123 | 18.5682 | 18.6121 | 3 | 3 | 3 | 3 | 2 | 3 | 3 | 3 |
| RAB1B | Q9H0U4 | 2.79E-15 | 4.20E-15 | -0.0221196 | | 0.057244 | -0.16215 | | 18.7628 | 18.77 | 18.7749 | 18.9084 | 19.1038 | 18.7284 | 18.4923 | 18.724 | 7 | 7 | 7 | 7 | 6 | 7 | 7 | 7 |
| MTOR | P42345 | 2.09E-20 | 1.69E-19 | -0.0220797 | | 0.163934 | -0.42509 | | 15.5343 | 15.5579 | 15.5369 | 15.5734 | 15.465 | 15.6336 | 15.6632 | 15.4903 | 47 | 47 | 49 | 47 | 35 | 46 | 46 | 39 |
| PSMD1 | Q99460 | 1.08E-17 | 3.40E-17 | -0.0220222 | | 0.312857 | -0.74118 | | 18.1397 | 18.2096 | 18.1808 | 18.226 | 18.2125 | 18.2467 | 18.165 | 18.1432 | 37 | 37 | 37 | 36 | 33 | 36 | 37 | 34 |

| Gene names | UniProt IDs | Protein Group P-value | Protein Group Q-value | Log2 Difference NUP214 vs Controls (n=5) | p<0.05 | -log10 p-value | T-test Test statistic | NPC Localization | Log2 MS2_quantity_NUP214_1 | Log2 MS2_quantity_NUP214_2 | Log2 MS2_quantity_NUP214_3 | Log2 MS2_quantity_Control_1 | Log2 MS2_quantity_Control_2 | Log2 MS2_quantity_Control_3 | Log2 MS2_quantity_Control_4 | Log2 MS2_quantity_Control_5 | Number Peptides for Quantitation NUP214_1 | Number Peptides for Quantitation NUP214_2 | Number Peptides for Quantitation NUP214_3 | Number Peptides for Quantitation Control_1 | Number Peptides for Quantitation Control_2 | Number Peptides for Quantitation Control_3 | Number Peptides for Quantitation Control_4 | Number Peptides for Quantitation Control_5 |
|---|---|---|---|---|---|---|---|---|---|---|---|---|---|---|---|---|---|---|---|---|---|---|---|---|
| GET4 | Q7L5D6 | 6.98E-15 | 9.20E-15 | -0.0219713 | | 0.048496 | -0.13855 | | 16.04 | 16.167 | 16.0004 | 16.5133 | 15.8915 | 16.0695 | 15.8702 | 16.1109 | 8 | 7 | 8 | 8 | 7 | 8 | 7 | 8 |
| C20orf27 | Q9GZN8;Q | 9.43E-07 | 2.75E-07 | -0.0218524 | | 0.054507 | -0.15481 | | 17.4112 | 17.4567 | 17.4008 | 17.6976 | 17.6733 | 17.3416 | 17.146 | 17.3652 | 7 | 7 | 7 | 7 | 6 | 7 | 6 | 7 |
| ATRN | O75882 | 3.06E-16 | 6.04E-16 | -0.0218389 | | 0.078108 | -0.217 | | 14.9335 | 14.9434 | 15.0237 | 15.0996 | 15.0885 | 14.7484 | 14.884 | 15.123 | 10 | 8 | 9 | 10 | 6 | 10 | 8 | 10 |
| TMEM104 | Q8NE00 | 0.000553 | 0.000118 | -0.0217051 | | 0.066695 | -0.18725 | | 16.977 | 16.9477 | 17.0082 | 17.2657 | 16.7951 | 17.0923 | 17.0112 | 16.8323 | 3 | 3 | 3 | 2 | 3 | 3 | 3 | 3 |
| GTF2E2 | P29084 | 2.48E-06 | 6.92E-07 | -0.0216695 | | 0.114737 | -0.30892 | | 14.2609 | 14.3134 | 14.3509 | 14.351 | 14.3862 | 14.1557 | 14.4579 | 14.2995 | 4 | 4 | 4 | 5 | 4 | 4 | 4 | 4 |
| BGN | P21810 | 5.92E-05 | 1.44E-05 | -0.0216583 | | 0.015997 | -0.04727 | | 16.2626 | 16.2311 | 16.3316 | 15.8319 | 16.1394 | 17.6268 | 15.7234 | 16.1623 | 9 | 10 | 9 | 8 | 7 | 9 | 8 | 10 |
| EPB41 | P11171;P1 | 0.00684 | 0.001199 | -0.0215 | | 0.090296 | -0.24817 | | 19.8546 | 20.0109 | 19.9205 | 20.1685 | 19.981 | 19.8807 | 19.8955 | 19.8251 | 1 | 1 | 1 | 2 | 2 | 1 | 1 | 1 |
| AARS2 | Q5JTZ9 | 2.58E-05 | 6.50E-06 | -0.0212961 | | 0.076735 | -0.21346 | | 15.9511 | 15.8985 | 15.9406 | 15.9841 | 16.0165 | 15.6685 | 15.9805 | 16.1071 | 16 | 16 | 15 | 14 | 14 | 13 | 15 | 16 |
| DHX32 | Q7L7V1;Q7 | 1.55E-07 | 4.93E-08 | -0.0211824 | | 0.072335 | -0.20202 | | 12.7464 | 12.812 | 12.7921 | 12.5551 | 13.0392 | 12.8309 | 12.8402 | 12.7581 | 3 | 3 | 3 | 2 | 3 | 3 | 3 | 3 |
| APOO | Q9BUR5;Q | 1.79E-09 | 7.13E-10 | -0.0210595 | | 0.242139 | -0.59655 | | 16.8226 | 16.8239 | 16.7798 | 16.7859 | 16.8397 | 16.776 | 16.9183 | 16.8291 | 5 | 5 | 5 | 5 | 5 | 5 | 5 | 5 |
| GRK2 | P25098 | 0.000972 | 0.000201 | -0.0209289 | | 0.052374 | -0.14906 | | 15.2391 | 15.1527 | 15.1126 | 15.2222 | 15.5129 | 14.8687 | 15.2162 | 15.1251 | 10 | 10 | 10 | 8 | 7 | 8 | 6 | 7 |
| PARD3 | Q8TEW0;Q | 2.37E-07 | 7.42E-08 | -0.0205747 | | 0.063254 | -0.17816 | | 14.5216 | 14.4488 | 14.4756 | 14.5748 | 14.6954 | 14.2271 | 14.3874 | 14.6283 | 12 | 9 | 10 | 11 | 6 | 5 | 6 | 10 |
| WDR18 | Q9BV38 | 0.000328 | 7.24E-05 | -0.0202784 | | 0.134108 | -0.35557 | | 16.8201 | 16.8681 | 16.8712 | 16.8476 | 16.7561 | 17.0153 | 16.8879 | 16.8603 | 11 | 11 | 12 | 12 | 8 | 12 | 11 | 11 |
| ELAVL1 | Q15717 | 1.28E-15 | 2.14E-15 | -0.0199596 | | 0.074057 | -0.20651 | | 18.6391 | 18.6555 | 18.5889 | 18.6058 | 18.9001 | 18.4975 | 18.5375 | 18.6981 | 11 | 11 | 11 | 11 | 11 | 11 | 11 | 11 |
| CHCHD6 | Q9BRQ6 | 2.04E-11 | 1.12E-11 | -0.0199264 | | 0.188377 | -0.48023 | | 14.7484 | 14.7922 | 14.8184 | 14.8942 | 14.746 | 14.7527 | 14.8531 | 14.7854 | 6 | 5 | 5 | 5 | 5 | 6 | 5 | 6 |
| ITFG2 | Q969R8 | 0.000269 | 6.02E-05 | -0.0197693 | | 0.038312 | -0.11057 | | 14.5104 | 14.4348 | 14.4565 | 14.0509 | 14.8317 | 14.6718 | 14.5075 | 14.373 | 6 | 3 | 5 | 3 | 2 | 3 | 2 | 2 |
| PRKACB | P22694;P2 | 1.77E-07 | 5.58E-08 | -0.0196499 | | 0.07701 | -0.21417 | | 17.3861 | 17.3719 | 17.3818 | 17.2 | 17.4495 | 17.4486 | 17.5996 | 17.3002 | 4 | 4 | 4 | 4 | 5 | 4 | 5 | 4 |
| AKR1C1 | Q04828 | 4.48E-11 | 2.29E-11 | -0.0195883 | | 0.018651 | -0.05495 | | 18.3518 | 18.4341 | 18.3015 | 18.4771 | 18.5785 | 18.8999 | 17.7539 | 18.6009 | 14 | 14 | 15 | 15 | 15 | 16 | 13 | 15 |
| GHITM | Q9H3K2 | 1.33E-05 | 3.45E-06 | -0.0194972 | | 0.032887 | -0.09544 | | 16.3914 | 16.4139 | 16.3634 | 16.2727 | 16.1316 | 17.0028 | 16.3635 | 16.2749 | 4 | 4 | 5 | 5 | 5 | 5 | 5 | 5 |
| CALCOCO2 | Q13137;Q1 | 3.59E-15 | 5.22E-15 | -0.0194295 | | 0.041256 | -0.11871 | | 15.3874 | 15.4443 | 15.3344 | 15.7977 | 15.0378 | 15.4378 | 15.3374 | 15.4299 | 9 | 8 | 9 | 10 | 7 | 8 | 8 | 8 |
| SELENOF | O60613 | 4.31E-07 | 1.31E-07 | -0.0193863 | | 0.038214 | -0.1103 | | 16.8747 | 16.9508 | 17.1775 | 16.8895 | 16.738 | 17.0793 | 17.4571 | 16.9379 | 4 | 4 | 4 | 4 | 4 | 4 | 4 | 4 |
| NDUFA9 | Q16795 | 1.18E-10 | 5.59E-11 | -0.0192205 | | 0.076297 | -0.21232 | | 17.4987 | 17.585 | 17.5105 | 17.3843 | 17.6301 | 17.6165 | 17.6761 | 17.352 | 16 | 17 | 16 | 17 | 16 | 16 | 15 | 16 |
| PSMA2 | P25787 | 1.30E-19 | 7.68E-19 | -0.0190735 | | 0.103084 | -0.28024 | | 18.1479 | 18.0913 | 18.118 | 17.9542 | 18.1369 | 18.2528 | 18.1962 | 18.1508 | 16 | 16 | 15 | 15 | 14 | 15 | 15 | 15 |
| DDX1 | Q92499 | 6.82E-13 | 5.13E-13 | -0.0190587 | | 0.120644 | -0.32328 | | 18.308 | 18.3895 | 18.3887 | 18.3288 | 18.3087 | 18.4178 | 18.5288 | 18.3215 | 38 | 39 | 38 | 38 | 38 | 38 | 38 | 39 |
| EMC3 | Q9P0I2 | 2.82E-11 | 1.50E-11 | -0.0189175 | | 0.18763 | -0.47857 | | 17.0163 | 17.004 | 17.0278 | 16.9844 | 17.0575 | 17.0417 | 17.1291 | 16.9619 | 9 | 8 | 9 | 10 | 8 | 10 | 9 | 9 |
| MAK16 | Q9BXY0 | 3.58E-10 | 1.57E-10 | -0.0186696 | | 0.077747 | -0.21607 | | 15.6213 | 15.5929 | 15.5786 | 15.3798 | 15.6513 | 15.7147 | 15.7416 | 15.5941 | 10 | 11 | 11 | 11 | 10 | 11 | 11 | 10 |
| RPS21 | P63220 | 2.03E-14 | 2.35E-14 | -0.0186554 | | 0.045541 | -0.13048 | | 20.5151 | 20.6309 | 20.5487 | 20.7182 | 20.2984 | 20.383 | 20.6552 | 20.8629 | 5 | 5 | 5 | 5 | 6 | 5 | 5 | 6 |
| RABL3 | Q5HYI8 | 1.45E-08 | 5.16E-09 | -0.0185785 | | 0.047234 | -0.13511 | | 17.2958 | 17.2017 | 17.2473 | 16.9815 | 17.3425 | 17.2916 | 17.5867 | 17.1319 | 6 | 5 | 5 | 5 | 4 | 4 | 5 | 5 |
| ACP2 | P11117 | 2.36E-05 | 5.98E-06 | -0.0184349 | | 0.030543 | -0.08885 | | 16.9548 | 17.1397 | 17.0873 | 16.8308 | 17.0171 | 17.2438 | 17.5778 | 16.7258 | 8 | 9 | 9 | 8 | 8 | 8 | 8 | 8 |
| PSMC5 | P62195;P6 | 1.48E-12 | 1.02E-12 | -0.0184333 | | 0.13361 | -0.35438 | | 18.1524 | 18.1219 | 18.1354 | 18.1891 | 18.0073 | 18.2192 | 18.2084 | 18.1508 | 28 | 28 | 28 | 28 | 28 | 28 | 28 | 28 |
| NUCB1 | Q02818 | 3.74E-11 | 1.94E-11 | -0.0180609 | | 0.145039 | -0.38136 | | 17.8172 | 17.852 | 17.8822 | 17.8163 | 17.8077 | 17.9467 | 17.9566 | 17.8154 | 29 | 29 | 28 | 29 | 28 | 29 | 29 | 28 |
| TRNT1 | Q96Q11;Q | 2.12E-14 | 2.44E-14 | -0.0180247 | | 0.076015 | -0.21159 | | 16.2086 | 16.3013 | 16.2416 | 16.2631 | 16.1506 | 16.4821 | 16.1409 | 16.3059 | 15 | 15 | 17 | 15 | 13 | 15 | 12 | 13 |
| GORASP1 | Q9BQQ3 | 7.77E-05 | 1.86E-05 | -0.018008 | | 0.078903 | -0.21906 | | 14.8517 | 15.01 | 14.9294 | 14.9241 | 14.8934 | 15.1568 | 14.9475 | 14.8201 | 4 | 5 | 5 | 3 | 4 | 5 | 4 | 4 |
| ZNF428 | Q96B54 | 0.001787 | 0.000355 | -0.0179262 | | 0.059572 | -0.16837 | | 16.4422 | 16.4623 | 16.7416 | 16.5168 | 16.6517 | 16.3544 | 16.6783 | 16.632 | 4 | 4 | 4 | 4 | 4 | 4 | 4 | 4 |
| GRPEL1 | Q9HAV7 | 6.93E-14 | 6.90E-14 | -0.0179012 | | 0.048412 | -0.13832 | | 16.3748 | 16.3815 | 16.5078 | 16.684 | 16.2114 | 16.2439 | 16.4533 | 16.6036 | 11 | 10 | 11 | 11 | 11 | 11 | 10 | 11 |
| MTMR9 | Q96QG7 | 6.24E-09 | 2.33E-09 | -0.0178773 | | 0.133463 | -0.35404 | | 15.6092 | 15.6933 | 15.6668 | 15.6986 | 15.6374 | 15.6873 | 15.781 | 15.5672 | 14 | 13 | 16 | 14 | 10 | 14 | 14 | 13 |
| ATXN10 | Q9UBB4 | 3.40E-14 | 3.68E-14 | -0.0178518 | | 0.043483 | -0.12484 | | 17.2947 | 17.2422 | 17.2557 | 17.4332 | 17.5206 | 16.9761 | 17.0794 | 17.4008 | 21 | 21 | 21 | 22 | 20 | 20 | 20 | 20 |
| PPP1CB | P62140 | 8.11E-10 | 3.39E-10 | -0.0177854 | | 0.141938 | -0.37408 | | 18.9391 | 18.9096 | 18.8883 | 18.8711 | 18.8483 | 19.035 | 18.9821 | 18.9139 | 6 | 6 | 6 | 6 | 6 | 6 | 6 | 6 |
| NFXL1 | Q6ZNB6 | 6.89E-05 | 1.66E-05 | -0.0177205 | | 0.087601 | -0.24133 | | 14.2764 | 14.4304 | 14.2962 | 14.2621 | 14.2401 | 14.488 | 14.3334 | 14.4366 | 9 | 8 | 10 | 5 | 7 | 10 | 9 | 5 |
| FAM193A | P78312;P7 | 0.000252 | 5.66E-05 | -0.0176888 | | 0.024916 | -0.07291 | | 12.6336 | 12.6171 | 12.4259 | 12.7324 | 12.4064 | 13.1815 | 12.1366 | 12.426 | 1 | 2 | 3 | 3 | 1 | 2 | 2 | 1 |
| NOC3L | Q8WTT2 | 7.17E-11 | 3.52E-11 | -0.0175184 | | 0.164606 | -0.42663 | | 15.3297 | 15.2946 | 15.3104 | 15.2606 | 15.3738 | 15.4056 | 15.3496 | 15.2557 | 11 | 13 | 12 | 13 | 9 | 13 | 13 | 10 |
| TRAPPC11 | Q7Z392 | 0.000128 | 2.99E-05 | -0.0174987 | | 0.093565 | -0.25643 | | 15.3997 | 15.4532 | 15.4443 | 15.3199 | 15.585 | 15.4596 | 15.5301 | 15.3547 | 19 | 18 | 20 | 17 | 14 | 20 | 19 | 15 |
| PCMT1 | P22061;P2 | 4.36E-10 | 1.89E-10 | -0.0172203 | | 0.059288 | -0.16761 | | 18.8824 | 18.8376 | 18.927 | 19.1091 | 18.6618 | 18.9959 | 18.8286 | 18.9025 | 14 | 14 | 15 | 15 | 15 | 15 | 15 | 15 |
| ENO2 | P09104 | 6.89E-08 | 2.26E-08 | -0.0172109 | | 0.043933 | -0.12608 | | 21.0908 | 21.1153 | 21.1799 | 21.2352 | 21.49 | 20.9957 | 20.9138 | 21.0948 | 14 | 14 | 15 | 16 | 15 | 15 | 15 | 15 |
| PRMT1 | Q99873;Q | 2.82E-17 | 7.79E-17 | -0.017104 | | 0.140048 | -0.36963 | | 18.3612 | 18.3916 | 18.3961 | 18.5351 | 18.3479 | 18.3658 | 18.3816 | 18.37 | 20 | 19 | 20 | 20 | 20 | 20 | 19 | 20 |
| TUBA4A | P68366;P6 | 1.01E-08 | 3.66E-09 | -0.0169647 | | 0.024146 | -0.07072 | | 21.9948 | 22.0209 | 22.0226 | 21.5744 | 22.2566 | 21.9877 | 21.7468 | 22.5832 | 6 | 6 | 6 | 6 | 6 | 6 | 6 | 6 |
| EIF2B1 | Q14232 | 5.22E-11 | 2.64E-11 | -0.0169586 | | 0.134464 | -0.35641 | | 17.0278 | 17.0067 | 16.9841 | 16.9625 | 17.0815 | 17.1259 | 17.0034 | 16.9426 | 16 | 17 | 18 | 16 | 15 | 17 | 18 | 17 |
| CCDC91 | Q7Z6B0;Q7 | 0.00357 | 0.000654 | -0.0167634 | | 0.030675 | -0.08923 | | 14.6315 | 14.3329 | 14.5819 | 14.4633 | 14.3549 | 14.8564 | 14.8107 | 14.1758 | 5 | 3 | 3 | 4 | 1 | 6 | 4 | 4 |
| CLASP2 | O75122-3 | 5.51E-06 | 1.48E-06 | -0.0167607 | | 0.016499 | -0.04872 | | 14.6414 | 14.5974 | 14.5054 | 15.1282 | 13.6196 | 14.6746 | 14.7201 | 14.8483 | 3 | 3 | 3 | 3 | 2 | 3 | 3 | 3 |
| SMAP2 | Q8WU79 | 1.33E-12 | 9.30E-13 | -0.0167208 | | 0.058838 | -0.16642 | | 16.371 | 16.457 | 16.2964 | 16.6072 | 16.4924 | 16.2288 | 16.2615 | 16.3678 | 6 | 6 | 6 | 5 | 4 | 6 | 6 | 5 |
| ATG3 | Q9NT62 | 3.94E-13 | 3.18E-13 | -0.0166292 | | 0.148689 | -0.38989 | | 17.7241 | 17.7039 | 17.7513 | 17.6695 | 17.812 | 17.7873 | 17.7801 | 17.6666 | 10 | 10 | 10 | 10 | 9 | 9 | 9 | 10 |
| DMAP1 | Q9NPF5 | 0.000161 | 3.71E-05 | -0.0165391 | | 0.031056 | -0.0903 | | 13.8011 | 13.8072 | 13.7897 | 13.9743 | 13.2703 | 13.9699 | 13.9751 | 13.8895 | 5 | 6 | 6 | 5 | 2 | 2 | 5 | 5 |
| FER | P16591 | 6.57E-05 | 1.59E-05 | -0.0163736 | | 0.033999 | -0.09856 | | 13.4849 | 13.8164 | 13.8783 | 13.7801 | 13.4419 | 14.0941 | 13.7297 | 13.6686 | 8 | 8 | 7 | 9 | 4 | 9 | 5 | 4 |
| ACTR1B | P42025 | 1.35E-07 | 4.31E-08 | -0.0163483 | | 0.085561 | -0.23614 | | 18.3517 | 18.4048 | 18.3881 | 18.3592 | 18.5781 | 18.2637 | 18.3792 | 18.4092 | 17 | 17 | 17 | 17 | 17 | 17 | 17 | 17 |
| RBM15B | Q8NDT2 | 0.001029 | 0.000212 | -0.0162877 | | 0.104097 | -0.28275 | | 15.3168 | 15.4251 | 15.4284 | 15.3652 | 15.4124 | 15.3843 | 15.5478 | 15.3224 | 8 | 8 | 8 | 7 | 5 | 7 | 9 | 5 |
| FAHD1 | Q6P587 | 2.06E-10 | 9.45E-11 | -0.0162352 | | 0.062277 | -0.17557 | | 16.7 | 16.5958 | 16.6721 | 16.5896 | 16.938 | 16.5772 | 16.6268 | 16.6295 | 9 | 9 | 9 | 9 | 6 | 8 | 9 | 9 |
| COASY | Q13057;Q1 | 4.37E-08 | 1.47E-08 | -0.0161948 | | 0.053265 | -0.15146 | | 17.0248 | 17.0042 | 16.9611 | 16.7463 | 16.9771 | 17.0903 | 17.2323 | 17.0185 | 11 | 11 | 11 | 10 | 11 | 11 | 11 | 11 |
| KDM3B | Q7LBC6 | 3.74E-12 | 2.36E-12 | -0.0160873 | | 0.091468 | -0.25114 | | 14.3281 | 14.382 | 14.3631 | 14.4643 | 14.4583 | 14.2026 | 14.3731 | 14.3705 | 15 | 11 | 15 | 13 | 8 | 7 | 12 | 10 |
| TRAM2 | Q15035 | 6.25E-08 | 2.06E-08 | -0.0160749 | | 0.026646 | -0.07784 | | 15.753 | 15.6752 | 15.9101 | 15.4488 | 15.4869 | 15.9267 | 16.259 | 15.8562 | 5 | 5 | 5 | 5 | 5 | 5 | 5 | 5 |
| GTPBP4 | Q9BZE4 | 8.53E-14 | 8.30E-14 | -0.0160634 | | 0.078078 | -0.21693 | | 16.1985 | 16.2226 | 16.2073 | 16.3295 | 16.0126 | 16.2329 | 16.2726 | 16.2801 | 30 | 29 | 27 | 28 | 25 | 29 | 30 | 29 |
| CAPN5 | O15484 | 1.82E-06 | 5.12E-07 | -0.015885 | | 0.051923 | -0.14784 | | 14.7551 | 14.6642 | 14.7566 | 14.4967 | 14.8638 | 14.8623 | 14.6096 | 14.8736 | 17 | 16 | 16 | 16 | 12 | 15 | 16 | 18 |
| CD151 | P48509 | 0.000368 | 8.06E-05 | -0.0158705 | | 0.058387 | -0.16521 | | 17.8815 | 17.8752 | 17.7989 | 17.6854 | 17.8309 | 17.7697 | 17.9761 | 18.0766 | 4 | 4 | 5 | 5 | 5 | 4 | 5 | 5 |
| SMARCB1 | Q12824 | 1.69E-09 | 6.75E-10 | -0.0156595 | | 0.042521 | -0.1222 | | 15.3015 | 15.4501 | 15.4246 | 15.6397 | 15.4683 | 15.1164 | 15.285 | 15.5292 | 9 | 8 | 8 | 8 | 6 | 7 | 7 | 8 |
| RAB27A | P51159 | 7.97E-13 | 5.89E-13 | -0.0155787 | | 0.036385 | -0.10522 | | 17.2156 | 17.236 | 17.2402 | 17.5114 | 17.4078 | 17.1357 | 16.8787 | 17.2971 | 7 | 7 | 7 | 7 | 7 | 6 | 7 | 7 |
| UNC13B | O14795;O1 | 0.000104 | 2.47E-05 | -0.0151299 | | 0.053348 | -0.15169 | | 14.9069 | 14.8245 | 14.7713 | 15.032 | 14.7872 | 14.7732 | 14.9984 | 14.6561 | 6 | 9 | 7 | 8 | 4 | 6 | 4 | 5 |
| DNASE2 | O00115;O0 | 2.54E-07 | 7.92E-08 | -0.0150394 | | 0.019506 | -0.05742 | | 16.3918 | 16.294 | 16.3968 | 15.9719 | 16.2877 | 16.6474 | 16.9843 | 15.9882 | 5 | 4 | 5 | 5 | 5 | 5 | 6 | 6 |
| RAB2A | P61019 | 0.000123 | 2.87E-05 | -0.0147111 | | 0.054586 | -0.15502 | | 18.8823 | 18.7 | 18.6369 | 18.5974 | 18.9601 | 18.7355 | 18.7162 | 18.763 | 8 | 8 | 8 | 8 | 8 | 8 | 8 | 8 |
| PGGT1B | P53609 | 1.89E-07 | 5.96E-08 | -0.0146412 | | 0.037692 | -0.10885 | | 14.597 | 14.6279 | 14.5896 | 14.7227 | 14.6375 | 14.8718 | 14.262 | 14.6032 | 4 | 4 | 5 | 3 | 4 | 4 | 5 | 4 |
| NAGLU | P54802 | 1.39E-13 | 1.27E-13 | -0.0144115 | | 0.01963 | -0.05777 | | 16.4213 | 16.4284 | 16.4044 | 16.2353 | 16.3142 | 16.7563 | 16.9489 | 15.9077 | 18 | 16 | 19 | 18 | 15 | 18 | 19 | 15 |
| MEMO1 | Q9Y316;Q | 2.42E-09 | 9.50E-10 | -0.0143684 | | 0.046418 | -0.13288 | | 17.1456 | 17.1627 | 17.1111 | 17.2789 | 17.3417 | 16.8958 | 17.0523 | 17.2022 | 11 | 11 | 11 | 8 | 9 | 9 | 10 | 9 |
| CNRIP1 | Q96F85 | 1.45E-11 | 8.20E-12 | -0.0143478 | | 0.057763 | -0.16354 | | 16.7832 | 16.8381 | 16.8713 | 17.0948 | 16.8354 | 16.7409 | 16.7678 | 16.7872 | 7 | 7 | 7 | 7 | 6 | 7 | 7 | 7 |
| SNX8 | Q9Y5X2 | 4.10E-15 | 5.84E-15 | -0.0142541 | | 0.054906 | -0.15588 | | 15.9172 | 15.8554 | 15.8322 | 16.0374 | 16.0443 | 15.7823 | 15.8317 | 15.7169 | 15 | 16 | 15 | 16 | 14 | 14 | 15 | 15 |
| C19orf53 | Q9UNZ5 | 6.15E-06 | 1.65E-06 | -0.0140114 | | 0.037124 | -0.10727 | | 15.7221 | 15.7281 | 15.6254 | 15.7106 | 15.951 | 15.3857 | 15.6406 | 15.8414 | 2 | 2 | 3 | 3 | 3 | 2 | 3 | 3 |
| ARFGAP3 | Q9NP61;Q | 2.71E-10 | 1.21E-10 | -0.0140041 | | 0.046059 | -0.1319 | | 16.6586 | 16.7403 | 16.6282 | 16.7239 | 16.7319 | 16.8713 | 16.4012 | 16.7202 | 21 | 21 | 22 | 21 | 20 | 20 | 21 | 20 |
| EPB41L1 | Q9H4G0-2 | 2.24E-05 | 5.69E-06 | -0.0139598 | | 0.013828 | -0.04095 | | 15.1926 | 15.1413 | 15.0995 | 14.2855 | 15.8577 | 15.1133 | 15.1543 | 15.3814 | 8 | 10 | 10 | 6 | 8 | 10 | 11 | 10 |
| PSMD12 | O00232 | 1.20E-08 | 4.32E-09 | -0.0139416 | | 0.190604 | -0.48518 | | 17.9435 | 17.9441 | 17.9374 | 18.0123 | 17.9358 | 18.0018 | 17.9077 | 17.9204 | 25 | 25 | 26 | 22 | 24 | 22 | 22 | 25 |
| GIGYF2 | Q6Y7W6;Q | 5.90E-18 | 2.06E-17 | -0.0139132 | | 0.073167 | -0.20419 | | 16.3724 | 16.4391 | 16.4042 | 16.5944 | 16.4471 | 16.3461 | 16.3056 | 16.4027 | 18 | 17 | 16 | 17 | 15 | 16 | 18 | 17 |
| KPNA1 | P52294 | 4.00E-17 | 1.06E-16 | -0.0138076 | | 0.061231 | -0.17279 | | 17.8521 | 17.7883 | 17.8867 | 18.0232 | 17.7944 | 17.7945 | 17.7112 | 17.9576 | 17 | 17 | 16 | 14 | 17 | 16 | 16 | 16 |

| Gene names | UniProt IDs | Protein Group P-value | Protein Group Q-value | Log2 Difference NUP214 vs Controls (n=5) | p<0.05 | -log10 p-value | T-test Test statistic | NPC Localization | Log2 MS2_quantity_ NUP214_1 | Log2 MS2_quantity_ NUP214_2 | Log2 MS2_quantity_ NUP214_3 | Log2 MS2_quantity_ Control_1 | Log2 MS2_quantity_ Control_2 | Log2 MS2_quantity_ Control_3 | Log2 MS2_quantity_ Control_4 | Log2 MS2_quantity_ Control_5 | Number Peptides for Quantitation NUP214_1 | Number Peptides for Quantitation NUP214_2 | Number Peptides for Quantitation NUP214_3 | Number Peptides for Quantitation Control_1 | Number Peptides for Quantitation Control_2 | Number Peptides for Quantitation Control_3 | Number Peptides for Quantitation Control_4 | Number Peptides for Quantitation Control_5 |
|---|---|---|---|---|---|---|---|---|---|---|---|---|---|---|---|---|---|---|---|---|---|---|---|---|
| IMPACT | Q9P2X3 | 1.87E-08 | 6.58E-09 | -0.0135998 | | 0.026366 | -0.07704 | | 15.608 | 15.7105 | 15.5751 | 15.5386 | 15.2807 | 16.0795 | 15.603 | 15.7222 | 9 | 8 | 10 | 10 | 7 | 10 | 10 | 9 |
| WDR26 | Q9H7D7;Q | 2.31E-12 | 1.52E-12 | -0.0134771 | | 0.086415 | -0.23831 | | 15.9298 | 16.0165 | 16.0134 | 16.1315 | 15.8906 | 16.002 | 16.0161 | 15.9599 | 13 | 14 | 14 | 13 | 11 | 13 | 12 | 13 |
| UBR7 | Q8N806 | 5.01E-07 | 1.51E-07 | -0.0133975 | | 0.024639 | -0.07213 | | 14.7842 | 14.8223 | 14.8204 | 15.0518 | 14.9885 | 14.2791 | 14.8751 | 14.9173 | 7 | 5 | 6 | 8 | 2 | 4 | 5 | 4 |
| EPN2 | O95208;O | 5.08E-08 | 1.70E-08 | -0.0132743 | | 0.063056 | -0.17763 | | 15.2335 | 15.2975 | 15.353 | 15.4812 | 15.2994 | 15.2344 | 15.1729 | 15.3518 | 6 | 7 | 6 | 7 | 7 | 6 | 7 | 6 |
| MBLAC2 | Q68D91 | 7.59E-07 | 2.24E-07 | -0.0132193 | | 0.042664 | -0.12259 | | 15.6568 | 15.6142 | 15.6767 | 15.4607 | 15.7119 | 15.8858 | 15.756 | 15.4981 | 8 | 8 | 8 | 8 | 7 | 8 | 9 | 7 |
| ARFGAP1 | Q8N6T3 | 1.71E-07 | 5.41E-08 | -0.0131688 | | 0.053089 | -0.15099 | | 16.3748 | 16.4052 | 16.4215 | 16.4351 | 16.2251 | 16.5762 | 16.521 | 16.3111 | 12 | 11 | 13 | 12 | 12 | 12 | 14 | 13 |
| ADAM9 | Q13443 | 1.99E-12 | 1.33E-12 | -0.0130582 | | 0.027128 | -0.0792 | | 15.6295 | 15.7449 | 15.7728 | 16.1078 | 15.7435 | 15.3443 | 15.689 | 15.7593 | 12 | 11 | 12 | 12 | 11 | 10 | 10 | 10 |
| ME2 | P23368 | 1.42E-10 | 6.67E-11 | -0.0129664 | | 0.067312 | -0.18887 | | 16.6901 | 16.6205 | 16.6498 | 16.7629 | 16.7324 | 16.5698 | 16.5207 | 16.7463 | 17 | 17 | 17 | 15 | 15 | 15 | 13 | 14 |
| CDK5 | Q00535 | 9.71E-07 | 2.82E-07 | -0.0127068 | | 0.040436 | -0.11645 | | 16.3281 | 16.172 | 16.1701 | 16.009 | 16.2595 | 16.4695 | 16.2937 | 16.1488 | 11 | 11 | 11 | 11 | 13 | 12 | 12 | 13 |
| RAB34 | P0DI83 | 0.006519 | 0.001146 | -0.0126313 | | 0.016034 | -0.04737 | | 14.8993 | 14.6543 | 14.9404 | 15.1776 | 15.0937 | 14.383 | 14.3595 | 15.2061 | 1 | 1 | 1 | 1 | 2 | 2 | 1 | 1 |
| YIF1A | O95070 | 4.99E-21 | 4.97E-20 | -0.0125014 | | 0.016654 | -0.04917 | | 17.5474 | 17.8119 | 17.8805 | 17.7969 | 17.1636 | 18.308 | 17.8261 | 17.7009 | 5 | 6 | 6 | 6 | 6 | 6 | 6 | 5 |
| PIP4K2A | P48426;P4 | 1.87E-14 | 2.19E-14 | -0.0124645 | | 0.021373 | -0.06278 | | 17.4941 | 17.5619 | 17.5793 | 17.3439 | 17.3838 | 17.6802 | 18.0859 | 17.2942 | 7 | 7 | 7 | 7 | 6 | 7 | 7 | 6 |
| NAP1L4 | Q99733;Q | 2.24E-09 | 8.83E-10 | -0.0120829 | | 0.046664 | -0.13355 | | 18.4325 | 18.3393 | 18.415 | 18.4721 | 18.5862 | 18.2242 | 18.2893 | 18.4667 | 11 | 12 | 12 | 11 | 12 | 12 | 12 | 12 |
| OSTF1 | Q92882 | 3.18E-10 | 1.40E-10 | -0.0120241 | | 0.035761 | -0.10348 | | 17.4681 | 17.4309 | 17.3936 | 17.7822 | 17.4076 | 17.3055 | 17.3564 | 17.3628 | 11 | 11 | 11 | 11 | 9 | 10 | 8 | 10 |
| RNF20 | Q5VTR2 | 1.48E-14 | 1.80E-14 | -0.0118299 | | 0.047168 | -0.13493 | | 14.6514 | 14.5597 | 14.6518 | 14.5917 | 14.6029 | 14.4396 | 14.82 | 14.7097 | 17 | 18 | 16 | 16 | 12 | 12 | 15 | 11 |
| AGA | P20933 | 7.92E-20 | 5.10E-19 | -0.01175 | | 0.018731 | -0.05518 | | 15.9541 | 15.8406 | 15.7388 | 15.799 | 16.2027 | 15.3083 | 15.8634 | 16.1078 | 6 | 6 | 5 | 4 | 5 | 3 | 3 | 4 |
| TRIM3 | O75382 | 9.61E-06 | 2.53E-06 | -0.0115964 | | 0.035051 | -0.1015 | | 15.3387 | 15.4364 | 15.3356 | 15.5659 | 15.4443 | 15.0707 | 15.4565 | 15.3717 | 13 | 12 | 12 | 13 | 11 | 10 | 12 | 12 |
| ABCE1 | P61221 | 2.56E-14 | 2.88E-14 | -0.011356 | | 0.05394 | -0.15328 | | 18.1311 | 18.1673 | 18.1005 | 18.008 | 18.0524 | 18.2564 | 18.2835 | 18.1212 | 23 | 23 | 22 | 23 | 23 | 23 | 23 | 23 |
| RETREG3 | Q86VR2 | 0.000163 | 3.76E-05 | -0.0112837 | | 0.040807 | -0.11748 | | 17.4029 | 17.5246 | 17.4782 | 17.6429 | 17.2655 | 17.6202 | 17.4405 | 17.4301 | 3 | 3 | 3 | 3 | 3 | 3 | 3 | 3 |
| MAPRE2 | Q15555;Q1 | 5.68E-06 | 1.53E-06 | -0.0112794 | | 0.014097 | -0.04174 | | 15.9044 | 15.8374 | 15.7988 | 15.9572 | 16.0492 | 15.0694 | 16.2153 | 15.9996 | 8 | 11 | 9 | 10 | 9 | 8 | 12 | 11 |
| TOR1AIP1 | Q5JTV8 | 2.02E-09 | 8.01E-10 | -0.0110897 | | 0.030624 | -0.08908 | | 17.3384 | 17.3971 | 17.3596 | 17.3659 | 17.1088 | 17.5101 | 17.6387 | 17.2572 | 17 | 18 | 18 | 20 | 17 | 20 | 19 | 19 |
| SRP68 | Q9UHB9 | 2.34E-11 | 1.27E-11 | -0.010887 | | 0.072972 | -0.20368 | | 17.7764 | 17.8011 | 17.7639 | 17.6709 | 17.8628 | 17.8859 | 17.7988 | 17.7384 | 29 | 29 | 29 | 29 | 28 | 29 | 29 | 28 |
| VPS4B | O75351 | 4.66E-13 | 3.67E-13 | -0.0107396 | | 0.053827 | -0.15298 | | 17.7936 | 17.8385 | 17.7349 | 17.7924 | 17.887 | 17.8933 | 17.6166 | 17.8094 | 20 | 20 | 19 | 19 | 19 | 19 | 19 | 19 |
| MTMR2 | Q13614;Q1 | 4.56E-05 | 1.12E-05 | -0.0107334 | | 0.03036 | -0.08834 | | 16.0187 | 16.1013 | 16.0268 | 16.2779 | 15.8114 | 16.0217 | 16.2501 | 15.9373 | 7 | 7 | 7 | 6 | 5 | 6 | 6 | 5 |
| RHOT1 | Q8IXI2;Q8I | 3.35E-11 | 1.76E-11 | -0.0106752 | | 0.043266 | -0.12425 | | 15.6314 | 15.6283 | 15.633 | 15.4887 | 15.861 | 15.6434 | 15.6752 | 15.5396 | 7 | 8 | 6 | 6 | 6 | 5 | 6 | 7 |
| IFT140 | Q96RY7 | 0.000486 | 0.000105 | -0.0106196 | | 0.008387 | -0.02499 | | 14.7585 | 14.728 | 14.8155 | 14.2876 | 15.9675 | 14.7255 | 14.7377 | 14.1714 | 5 | 5 | 6 | 1 | 2 | 6 | 4 | 3 |
| FABP5 | Q01469 | 2.54E-17 | 7.16E-17 | -0.0105139 | | 0.015252 | -0.0451 | | 17.602 | 17.6544 | 17.5763 | 18.1477 | 17.2438 | 17.5936 | 17.2605 | 17.8614 | 10 | 11 | 11 | 10 | 10 | 10 | 11 | 11 |
| ANAPC7 | Q9UJX3;Q9 | 3.74E-06 | 1.02E-06 | -0.0101947 | | 0.065683 | -0.18458 | | 15.167 | 15.1849 | 15.2547 | 15.3271 | 15.2371 | 15.0927 | 15.1729 | 15.2323 | 12 | 12 | 14 | 11 | 10 | 14 | 13 | 11 |
| TUSC3 | Q13454-2 | 4.49E-16 | 8.42E-16 | -0.010169 | | 0.043208 | -0.12409 | | 15.6095 | 15.4831 | 15.4755 | 15.6339 | 15.5689 | 15.6022 | 15.3143 | 15.5449 | 8 | 8 | 7 | 6 | 5 | 8 | 7 | 4 |
| MIA3 | Q5JRA6 | 4.71E-18 | 1.70E-17 | -0.0100637 | | 0.053882 | -0.15313 | | 15.5255 | 15.4776 | 15.5246 | 15.4558 | 15.4391 | 15.587 | 15.678 | 15.4365 | 41 | 46 | 44 | 41 | 30 | 45 | 40 | 39 |
| SEC23IP | Q9Y6Y8 | 7.88E-17 | 1.91E-16 | -0.0100595 | | 0.037936 | -0.10953 | | 16.6348 | 16.6801 | 16.6767 | 16.7768 | 16.5636 | 16.7799 | 16.7902 | 16.4591 | 26 | 28 | 27 | 27 | 24 | 27 | 27 | 25 |
| SLC66A2 | Q8N2U9;Q | 0.001847 | 0.000365 | -0.0099699 | | 0.030499 | -0.08873 | | 13.6475 | 13.8048 | 13.8592 | 13.635 | 13.6102 | 13.739 | 13.9921 | 13.9261 | 3 | 3 | 4 | 3 | 1 | 3 | 1 | 2 |
| NGDN | Q8NEJ9;Q8 | 5.10E-13 | 3.98E-13 | -0.00985425 | | 0.014189 | -0.04201 | | 13.7759 | 13.8705 | 13.8208 | 13.9455 | 13.1473 | 14.1381 | 14.0085 | 13.9219 | 6 | 6 | 4 | 5 | 4 | 4 | 4 | 4 |
| DPY19L1 | Q2PZI1 | 0.000797 | 0.000167 | -0.00985095 | | 0.028428 | -0.08288 | | 17.8778 | 17.8976 | 17.7108 | 17.5395 | 17.9095 | 18.0433 | 17.8748 | 17.8258 | 11 | 12 | 11 | 8 | 6 | 10 | 10 | 12 |
| RRAS2 | P62070;P6 | 4.23E-10 | 1.83E-10 | -0.00966504 | | 0.017766 | -0.05239 | | 18.652 | 18.6554 | 18.5884 | 18.7711 | 18.9895 | 18.4119 | 18.236 | 18.7996 | 12 | 12 | 12 | 12 | 12 | 12 | 12 | 12 |
| VHL | P40337;P4 | 0.000506 | 0.000109 | -0.00947833 | | 0.016621 | -0.04948 | | 14.4991 | 14.488 | 14.519 | 14.5933 | 14.0342 | 14.7349 | 14.6837 | NaN | 2 | 3 | 3 | 2 | 2 | 2 | 2 | NaN |
| PARD3B | Q8TEW8;Q | 6.78E-06 | 1.81E-06 | -0.00946757 | | 0.008777 | -0.02614 | | 14.5964 | 14.4595 | 14.2972 | 13.3956 | 14.6999 | 14.7788 | 14.6453 | 14.7829 | 1 | 3 | 2 | 2 | 2 | 2 | 2 | 1 |
| SDF2L1 | Q9HCN8 | 0.000688 | 0.000145 | -0.00944684 | | 0.047256 | -0.13517 | | 17.0016 | 17.0081 | 16.9588 | 17.0693 | 17.0437 | 16.8859 | 17.1279 | 16.8679 | 4 | 3 | 4 | 4 | 3 | 3 | 3 | 3 |
| PTPN2 | P17706 | 0.001014 | 0.000209 | -0.00920518 | | 0.017848 | -0.05263 | | 15.2289 | 15.3456 | 15.1849 | 15.549 | 14.8491 | 15.5224 | 15.1745 | 15.2167 | 5 | 6 | 5 | 4 | 2 | 4 | 4 | 3 |
| NPHP3 | Q7Z494 | 4.50E-11 | 2.30E-11 | -0.00914421 | | 0.034573 | -0.10016 | | 15.1012 | 14.8028 | 15.0452 | 14.9459 | 15.1348 | 14.9258 | 15.0671 | 14.8874 | 2 | 2 | 1 | 3 | 2 | 3 | 5 | 1 |
| NDUFB6 | O95139 | 1.33E-12 | 9.29E-13 | -0.00912107 | | 0.033121 | -0.0961 | | 16.6616 | 16.7598 | 16.8499 | 16.712 | 16.6593 | 16.8674 | 16.9654 | 16.6271 | 5 | 5 | 6 | 6 | 4 | 6 | 5 | 5 |
| DHX29 | Q7Z478 | 2.50E-23 | 5.02E-22 | -0.00901273 | | 0.068302 | -0.19147 | | 15.5754 | 15.5501 | 15.5329 | 15.5467 | 15.6126 | 15.4328 | 15.6186 | 15.5984 | 41 | 42 | 40 | 42 | 36 | 40 | 42 | 39 |
| SMARCA2 | P51531;P5 | 2.79E-07 | 8.65E-08 | -0.00880604 | | 0.013139 | -0.03894 | | 15.6653 | 15.6432 | 15.8032 | 15.3077 | 16.3261 | 15.6334 | 15.6996 | 15.5967 | 11 | 8 | 12 | 8 | 3 | 6 | 6 | 7 |
| PSMG1 | O95456 | 0.000159 | 3.68E-05 | -0.00869306 | | 0.021924 | -0.06436 | | 16.6841 | 16.6911 | 16.6569 | 16.4197 | 16.5479 | 16.8772 | 16.959 | 16.6266 | 7 | 7 | 8 | 7 | 7 | 8 | 7 | 8 |
| MED19 | A0JLT2;A0J | 0.014895 | 0.00251 | -0.00866246 | | 0.007507 | -0.02285 | | NaN | 13.4319 | 13.3925 | 13.4552 | 13.5697 | 12.727 | 13.9316 | NaN | NaN | 2 | 2 | 2 | 2 | 1 | 1 | NaN |
| NDUFS6 | O75380 | 2.07E-06 | 5.80E-07 | -0.00857455 | | 0.023937 | -0.07012 | | 17.0552 | 17.0291 | 17.0013 | 16.8981 | 17.3506 | 17.0401 | 17.0775 | 16.8191 | 5 | 5 | 5 | 5 | 5 | 5 | 6 | 5 |
| RNPS1 | Q15287;Q1 | 3.22E-10 | 1.42E-10 | -0.00857086 | | 0.045972 | -0.13166 | | 16.75 | 16.7372 | 16.6359 | 16.7838 | 16.7625 | 16.804 | 16.5646 | 16.6665 | 5 | 4 | 4 | 3 | 4 | 5 | 3 | 3 |
| CRKL | P46109 | 4.49E-10 | 1.94E-10 | -0.00843239 | | 0.038828 | -0.112 | | 17.3401 | 17.367 | 17.3127 | 17.4779 | 17.1494 | 17.3978 | 17.3179 | 17.3988 | 14 | 14 | 14 | 14 | 13 | 13 | 14 | 13 |
| EIF3A | Q14152 | 5.27E-14 | 5.43E-14 | -0.00830053 | | 0.0406 | -0.1169 | | 18.0211 | 18.0807 | 18.0551 | 18.2603 | 17.9833 | 17.969 | 18.0551 | 18.0353 | 68 | 69 | 67 | 68 | 66 | 66 | 66 | 66 |
| ZNFX1 | Q9P2E3 | 4.47E-09 | 1.70E-09 | -0.00820122 | | 0.05753 | -0.16292 | | 15.1546 | 15.2106 | 15.192 | 15.1203 | 15.2733 | 15.0987 | 15.271 | 15.2062 | 23 | 26 | 23 | 24 | 15 | 21 | 23 | 21 |
| TGFBRAP1 | Q8WUH2 | 7.08E-05 | 1.71E-05 | -0.00810515 | | 0.028569 | -0.08328 | | 15.5665 | 15.7393 | 15.7214 | 15.5013 | 15.8281 | 15.7883 | 15.7538 | 15.5477 | 10 | 10 | 10 | 9 | 7 | 9 | 8 | 8 |
| GOLGB1 | Q14789;Q1 | 1.00E-12 | 7.25E-13 | -0.00797354 | | 0.030909 | -0.08989 | | 15.3698 | 15.4156 | 15.419 | 15.2425 | 15.4433 | 15.3591 | 15.6401 | 15.3621 | 106 | 116 | 107 | 101 | 79 | 102 | 109 | 96 |
| PLAA | Q9Y263 | 1.90E-08 | 6.67E-09 | -0.00773849 | | 0.037606 | -0.10861 | | 16.3869 | 16.3671 | 16.3171 | 16.3187 | 16.2233 | 16.5079 | 16.4608 | 16.3132 | 26 | 26 | 25 | 28 | 24 | 26 | 26 | 27 |
| ARHGAP5 | Q13017;Q1 | 3.68E-07 | 1.13E-07 | -0.0075051 | | 0.021142 | -0.06212 | | 15.079 | 15.099 | 15.05 | 14.8002 | 15.0216 | 15.0995 | 15.3588 | 15.1374 | 28 | 26 | 26 | 25 | 20 | 24 | 23 | 22 |
| DAB2IP | Q5VWQ8;Q | 1.70E-14 | 2.02E-14 | -0.00738754 | | 0.021457 | -0.06302 | | 14.6684 | 14.8649 | 14.7517 | 14.8738 | 14.6593 | 14.5323 | 14.7742 | 15.0057 | 9 | 8 | 8 | 9 | 4 | 5 | 5 | 8 |
| FLAD1 | Q8NFF5;Q8 | 0.000788 | 0.000165 | -0.00727145 | | 0.045984 | -0.13169 | | 15.7591 | 15.8063 | 15.9251 | 15.8665 | 15.9229 | 15.838 | 15.8294 | 15.7304 | 9 | 9 | 11 | 12 | 8 | 8 | 9 | 9 |
| STRN | O43815 | 7.37E-12 | 4.41E-12 | -0.00721639 | | 0.043563 | -0.12506 | | 16.2156 | 16.2544 | 16.2481 | 16.2502 | 16.179 | 16.4041 | 16.2373 | 16.1623 | 22 | 24 | 22 | 22 | 18 | 21 | 21 | 22 |
| RPL15 | P61313 | 5.01E-05 | 1.23E-05 | -0.00717379 | | 0.046303 | -0.13257 | | 19.7443 | 19.8581 | 19.7219 | 19.8051 | 19.7694 | 19.8566 | 19.8179 | 19.6606 | 12 | 12 | 12 | 12 | 11 | 12 | 12 | 12 |
| EMC2 | Q15006 | 2.22E-10 | 1.01E-10 | -0.00716235 | | 0.054746 | -0.15545 | | 16.7821 | 16.8001 | 16.7152 | 16.7492 | 16.6965 | 16.8196 | 16.87 | 16.7295 | 9 | 9 | 10 | 10 | 9 | 10 | 10 | 10 |
| METTL5 | Q9NRN9 | 0.000107 | 2.51E-05 | -0.00713571 | | 0.025024 | -0.07322 | | 15.0203 | 15.0405 | 15.1917 | 15.3337 | 15.0994 | 14.9426 | 15.0005 | 15.0804 | 5 | 4 | 5 | 5 | 3 | 3 | 3 | 6 |
| NRF1 | Q16656;Q1 | 0.001844 | 0.000365 | -0.00709165 | | 0.009956 | -0.02961 | | 16.0493 | 15.7556 | 15.9752 | 15.7334 | 16.2342 | 15.6727 | 15.5745 | 16.4543 | 2 | 3 | 3 | 3 | 3 | 3 | 3 | 2 |
| GLT8D1 | Q68CQ7 | 4.15E-11 | 2.13E-11 | -0.0069519 | | 0.014381 | -0.04257 | | 17.1553 | 17.2265 | 17.11 | 16.9803 | 16.9081 | 17.5216 | 17.3948 | 17.0496 | 9 | 9 | 9 | 9 | 9 | 10 | 10 | 9 |
| PIK3C3 | Q8NEB9 | 3.59E-06 | 9.86E-07 | -0.00694014 | | 0.026729 | -0.07807 | | 15.9971 | 16.0802 | 16.0421 | 16.1239 | 15.9648 | 16.2479 | 15.8679 | 16.0294 | 20 | 20 | 19 | 19 | 15 | 19 | 16 | 19 |
| RASA3 | Q14644;Q1 | 7.90E-12 | 4.69E-12 | -0.00693353 | | 0.026943 | -0.07868 | | 15.5816 | 15.5026 | 15.5203 | 15.7261 | 15.6345 | 15.3988 | 15.5518 | 15.3976 | 17 | 18 | 23 | 26 | 18 | 18 | 19 | 21 |
| DLST | P36957 | 8.09E-09 | 2.98E-09 | -0.00683556 | | 0.018096 | -0.05334 | | 18.8192 | 18.832 | 18.7493 | 18.9859 | 18.7896 | 18.9369 | 18.4502 | 18.8723 | 15 | 17 | 16 | 17 | 15 | 17 | 15 | 15 |
| HSPA9 | P38646 | 1.57E-14 | 1.88E-14 | -0.00670369 | | 0.024091 | -0.07056 | | 20.0043 | 19.9903 | 19.9689 | 19.8949 | 19.8695 | 20.2416 | 20.0672 | 19.8996 | 39 | 40 | 39 | 38 | 38 | 38 | 39 | 38 |
| TXNIP | Q9H3M7 | 0.000196 | 4.45E-05 | -0.00668507 | | 0.011724 | -0.0348 | | 15.1202 | 15.1361 | 15.2336 | 15.1163 | 14.8857 | 15.7106 | 15.1431 | 14.9943 | 6 | 3 | 5 | 3 | 1 | 5 | 2 | 2 |
| C11orf68 | Q9H3H3 | 3.27E-08 | 1.12E-08 | -0.00661977 | | 0.027271 | -0.07961 | | 17.8325 | 17.8678 | 17.8179 | 18.016 | 17.7536 | 17.9425 | 17.8442 | 17.6737 | 13 | 11 | 12 | 14 | 11 | 12 | 12 | 12 |
| PSMB4 | P28070 | 2.87E-07 | 8.88E-08 | -0.00656815 | | 0.025114 | -0.07348 | | 18.9101 | 18.9151 | 18.9329 | 18.9565 | 18.6694 | 18.9861 | 19.0612 | 18.9566 | 7 | 7 | 7 | 7 | 5 | 6 | 7 | 5 |
| MT-CO2 | P00403 | 1.31E-08 | 4.69E-09 | -0.00647647 | | 0.032635 | -0.09474 | | 19.8855 | 19.8253 | 19.8812 | 19.6729 | 19.9288 | 19.9046 | 19.9475 | 19.8985 | 6 | 6 | 6 | 6 | 6 | 6 | 6 | 6 |
| SLC35A3 | Q9Y2D2;Q | 0.003965 | 0.00072 | -0.00636444 | | 0.014497 | -0.0429 | | 14.4131 | 14.4859 | 14.7805 | 14.6191 | 14.282 | 14.8556 | 14.4994 | 14.575 | 1 | 1 | 1 | 1 | 1 | 1 | 1 | 1 |
| KARS1 | Q15046 | 1.80E-16 | 3.96E-16 | -0.00632858 | | 0.064523 | -0.18151 | | 17.6567 | 17.705 | 17.6839 | 17.7339 | 17.65 | 17.757 | 17.6244 | 17.6757 | 29 | 30 | 28 | 28 | 27 | 29 | 29 | 27 |
| SEPTIN7 | Q16181 | 8.81E-20 | 5.50E-19 | -0.00627569 | | 0.043029 | -0.1236 | | 18.844 | 18.782 | 18.7552 | 18.6671 | 18.8456 | 18.8067 | 18.8704 | 18.8103 | 25 | 25 | 25 | 24 | 25 | 25 | 25 | 25 |
| CGGBP1 | Q9UFW8 | 4.41E-09 | 1.68E-09 | -0.00599289 | | 0.015243 | -0.04507 | | 15.7383 | 15.7035 | 15.7477 | 15.845 | 15.88 | 15.3735 | 15.6724 | 15.9083 | 5 | 5 | 5 | 5 | 5 | 5 | 5 | 6 |
| NDUFA11 | Q86Y39 | 0.000126 | 2.95E-05 | -0.00599047 | | 0.020003 | -0.05884 | | 16.5514 | 16.6529 | 16.6594 | 16.6133 | 16.6221 | 16.8205 | 16.7083 | 16.372 | 6 | 6 | 6 | 6 | 6 | 6 | 6 | 6 |
| MYH9 | P35579 | 3.30E-15 | 4.85E-15 | -0.00594813 | | 0.028031 | -0.08176 | | 20.7433 | 20.7214 | 20.7298 | 20.5524 | 20.8533 | 20.8357 | 20.6948 | 20.7511 | 171 | 169 | 171 | 164 | 171 | 170 | 169 | 168 |
| IST1 | P53990;P5 | 3.61E-15 | 5.24E-15 | -0.00576121 | | 0.058559 | -0.16567 | | 17.0367 | 17.0531 | 17.0002 | 17.0789 | 16.9631 | 17.1001 | 17.007 | 17.0298 | 11 | 10 | 11 | 10 | 10 | 11 | 11 | 11 |
| MOCS3 | O95396 | 2.67E-15 | 4.04E-15 | -0.00569789 | | 0.014683 | -0.04345 | | 14.9316 | 14.9553 | 14.9137 | 14.6634 | 14.8688 | 15.2656 | 14.9985 | 14.8997 | 8 | 9 | 9 | 7 | 8 | 8 | 8 | 6 |

| Gene names | UniProt IDs | Protein Group P-value | Protein Group Q-value | Log2 Difference NUP214 vs Controls (n=5) | p<0.05 | -log10 p-value | T-test Test statistic | NPC Localization | Log2 MS2_quantity_ NUP214_1 | Log2 MS2_quantity_ NUP214_2 | Log2 MS2_quantity_ NUP214_3 | Log2 MS2_quantity_ Control_1 | Log2 MS2_quantity_ Control_2 | Log2 MS2_quantity_ Control_3 | Log2 MS2_quantity_ Control_4 | Log2 MS2_quantity_ Control_5 | Number Peptides for Quantitation NUP214_1 | Number Peptides for Quantitation NUP214_2 | Number Peptides for Quantitation NUP214_3 | Number Peptides for Quantitation Control_1 | Number Peptides for Quantitation Control_2 | Number Peptides for Quantitation Control_3 | Number Peptides for Quantitation Control_4 | Number Peptides for Quantitation Control_5 |
|---|---|---|---|---|---|---|---|---|---|---|---|---|---|---|---|---|---|---|---|---|---|---|---|---|
| VAPA | Q9P0L0 | 2.89E-07 | 8.95E-08 | -0.00569229 | | 0.025307 | -0.07403 | | 18.587 | 18.5599 | 18.5752 | 18.6297 | 18.406 | 18.7557 | 18.5775 | 18.5297 | 12 | 12 | 12 | 12 | 10 | 12 | 12 | 11 |
| SEPTIN6 | Q14141;Q | 0.000381 | 8.34E-05 | -0.00569146 | | 0.011935 | -0.03542 | | 14.8324 | 14.8395 | 15.0678 | 15.2 | 15.1401 | 14.7938 | 14.5891 | 14.8715 | 4 | 3 | 3 | 5 | 4 | 4 | 4 | 4 |
| MRPL22 | Q9NWU5 | 5.32E-06 | 1.43E-06 | -0.00563812 | | 0.055297 | -0.15693 | | 16.5668 | 16.5986 | 16.6083 | 16.6356 | 16.5647 | 16.5404 | 16.5644 | 16.6793 | 6 | 6 | 7 | 5 | 7 | 6 | 7 | 6 |
| HDGFL2 | Q7Z4V5 | 9.45E-15 | 1.21E-14 | -0.005535 | | 0.025573 | -0.07479 | | 17.2153 | 17.3381 | 17.3313 | 17.4164 | 17.2326 | 17.1719 | 17.2561 | 17.4252 | 14 | 14 | 12 | 13 | 13 | 13 | 13 | 13 |
| COG7 | P83436 | 1.09E-13 | 1.02E-13 | -0.00545203 | | 0.014133 | -0.04184 | | 15.8205 | 15.7663 | 15.8499 | 15.4645 | 16.0188 | 15.8162 | 15.9667 | 15.8222 | 22 | 21 | 21 | 20 | 20 | 21 | 21 | 20 |
| CLTA | P09496-2 | 2.33E-10 | 1.06E-10 | -0.0054053 | | 0.009172 | -0.02731 | | 19.1668 | 19.0779 | 19.3541 | 19.7634 | 19.1338 | 18.9851 | 19.0774 | 19.0653 | 7 | 8 | 8 | 8 | 8 | 8 | 8 | 7 |
| MBNL1 | Q9NR56 | 8.20E-07 | 2.41E-07 | -0.00536168 | | 0.032283 | -0.09375 | | 18.0882 | 18.2027 | 18.1396 | 18.2277 | 18.2474 | 18.1065 | 18.0401 | 18.1226 | 8 | 9 | 8 | 8 | 8 | 7 | 8 | 8 |
| BLOC1S5 | Q8TDH9 | 9.68E-07 | 2.82E-07 | -0.00526002 | | 0.032635 | -0.09474 | | 14.7284 | 14.6962 | 14.798 | 14.7788 | 14.7713 | 14.8584 | 14.6457 | 14.6763 | 5 | 5 | 5 | 5 | 4 | 5 | 5 | 5 |
| BCAS3 | Q9H6U6;Q | 3.09E-07 | 9.53E-08 | -0.0051473 | | 0.02167 | -0.06364 | | 15.0264 | 15.1093 | 15.0457 | 14.8793 | 15.1187 | 15.1438 | 15.2042 | 14.982 | 11 | 12 | 11 | 10 | 9 | 10 | 8 | 8 |
| ABCB10 | Q9NRK6 | 0.00215 | 0.000419 | -0.00473614 | | 0.013218 | -0.03917 | | 14.8295 | 14.9603 | 14.9579 | 15.0068 | 15.1048 | 14.8178 | 14.6279 | 15.0458 | 5 | 8 | 7 | 5 | 3 | 6 | 4 | 5 |
| MCUR1 | Q96AQ8 | 0.000323 | 7.15E-05 | -0.00454839 | | 0.005028 | -0.01504 | | 13.9064 | 14.1951 | 14.1029 | 13.8742 | 14.1737 | 13.3174 | 14.5304 | 14.4677 | 2 | 3 | 3 | 3 | 2 | 2 | 4 | 3 |
| ABL2 | P42684;P4 | 0.005747 | 0.001016 | -0.00446359 | | 0.00434 | -0.0131 | | 13.9061 | 13.5247 | 13.2153 | 14.204 | 13.0436 | NaN | 13.3052 | 13.6597 | 1 | 3 | 3 | 3 | 2 | NaN | 1 | 3 |
| CNOT7 | Q9UIV1 | 1.30E-06 | 3.72E-07 | -0.00438639 | | 0.015872 | -0.0469 | | 17.306 | 17.1559 | 17.4462 | 17.3127 | 17.1074 | 17.3829 | 17.4087 | 17.3239 | 7 | 5 | 7 | 6 | 5 | 5 | 6 | 5 |
| VPS51 | Q9UID3 | 4.67E-08 | 1.56E-08 | -0.00405413 | | 0.014223 | -0.04211 | | 15.9174 | 15.856 | 15.8189 | 15.7159 | 15.7864 | 16.0291 | 16.0477 | 15.7617 | 17 | 17 | 16 | 17 | 16 | 17 | 16 | 17 |
| CCS | O14618 | 2.17E-08 | 7.57E-09 | -0.00395088 | | 0.011488 | -0.03411 | | 17.0937 | 17.0507 | 17.1025 | 17.0358 | 16.8513 | 17.3822 | 17.1259 | 17.0361 | 5 | 5 | 5 | 5 | 5 | 5 | 5 | 5 |
| GDI2 | P50395 | 6.43E-19 | 2.96E-18 | -0.00388362 | | 0.025087 | -0.0734 | | 19.6544 | 19.6668 | 19.6456 | 19.656 | 19.5539 | 19.7951 | 19.6721 | 19.6201 | 39 | 39 | 40 | 40 | 38 | 40 | 39 | 40 |
| ASCC1 | Q8N9N2-2 | 4.13E-07 | 1.26E-07 | -0.00388323 | | 0.022093 | -0.06485 | | 16.2178 | 16.2146 | 16.272 | 16.1386 | 16.3525 | 16.2567 | 16.3078 | 16.1378 | 7 | 8 | 8 | 9 | 8 | 8 | 8 | 8 |
| ELMO2 | Q96JJ3 | 1.46E-05 | 3.77E-06 | -0.00378558 | | 0.010647 | -0.03164 | | 16.448 | 16.4474 | 16.4432 | 16.363 | 16.6243 | 16.3074 | 16.7025 | 16.2527 | 22 | 22 | 20 | 19 | 19 | 21 | 21 | 20 |
| STX5 | Q13190 | 9.14E-12 | 5.34E-12 | -0.00376511 | | 0.013305 | -0.03943 | | 15.7989 | 15.9309 | 15.8284 | 15.76 | 15.8098 | 16.0298 | 16.0012 | 15.6818 | 12 | 11 | 11 | 10 | 9 | 10 | 11 | 11 |
| ARFGEF2 | Q9Y6D5 | 7.17E-08 | 2.35E-08 | -0.00372213 | | 0.01893 | -0.05575 | | 14.9795 | 14.9656 | 14.9234 | 14.8596 | 14.9602 | 15.0502 | 15.0875 | 14.8419 | 24 | 22 | 23 | 22 | 21 | 20 | 22 | 17 |
| MT-ND5 | P03915 | 2.50E-10 | 1.13E-10 | -0.00367978 | | 0.008 | -0.02385 | | 15.1967 | 15.1887 | 15.23 | 15.1586 | 14.8899 | 15.4008 | 15.5321 | 15.0627 | 4 | 4 | 4 | 4 | 2 | 4 | 4 | 5 |
| EPM2AIP1 | Q7L775 | 4.47E-15 | 6.29E-15 | -0.00344397 | | 0.010553 | -0.03137 | | 15.9594 | 15.9163 | 15.9099 | 15.7139 | 16.1978 | 15.8496 | 15.8839 | 16.0146 | 10 | 13 | 11 | 11 | 9 | 10 | 11 | 12 |
| KPNA3 | O00505 | 2.72E-05 | 6.83E-06 | -0.00328954 | | 0.0224 | -0.06573 | | 17.6923 | 17.6641 | 17.7101 | 17.7731 | 17.7318 | 17.5861 | 17.6231 | 17.7465 | 9 | 9 | 9 | 9 | 9 | 9 | 9 | 9 |
| BNIP2 | Q12982;Q | 0.0003 | 6.66E-05 | -0.00313505 | | 0.009021 | -0.02686 | | 16.3632 | 16.3384 | 16.34 | 16.5923 | 16.2427 | 16.4072 | 16.4299 | 16.0798 | 3 | 3 | 3 | 3 | 3 | 3 | 3 | 3 |
| OGT | O15294 | 8.84E-12 | 5.19E-12 | -0.00292282 | | 0.015026 | -0.04444 | | 16.2368 | 16.2567 | 16.2207 | 16.277 | 16.2947 | 16.0825 | 16.185 | 16.3658 | 30 | 28 | 30 | 29 | 28 | 29 | 29 | 31 |
| PRKAA1 | Q13131;Q | 3.75E-13 | 3.05E-13 | -0.00260709 | | 0.00997 | -0.02966 | | 17.4763 | 17.5619 | 17.5186 | 17.3371 | 17.4481 | 17.7278 | 17.5504 | 17.5441 | 18 | 18 | 18 | 18 | 17 | 18 | 18 | 17 |
| TMEM237 | Q96Q45;Q | 5.94E-09 | 2.22E-09 | -0.00244783 | | 0.004491 | -0.01344 | | 15.4947 | 15.2472 | 15.316 | 15.0098 | 15.3946 | 15.4797 | 15.7521 | 15.1393 | 6 | 7 | 8 | 7 | 5 | 7 | 7 | 7 |
| VEZT | Q9HBM0 | 0.009934 | 0.001706 | -0.00232614 | | 0.004453 | -0.01333 | | 14.5167 | 14.3401 | 14.2198 | 14.5954 | 14.7009 | 14.0602 | 14.22 | 14.2296 | 2 | 3 | 2 | 4 | 2 | 2 | 3 | 3 |
| HEXA | P06865 | 6.39E-14 | 6.43E-14 | -0.00215696 | | 0.00246 | -0.00738 | | 17.4264 | 17.5051 | 17.4564 | 17.0003 | 17.1984 | 17.9775 | 18.0126 | 17.1353 | 15 | 16 | 15 | 16 | 16 | 15 | 16 | 15 |
| UBL3 | O95164 | 0.000245 | 5.50E-05 | -0.00199986 | | 0.003858 | -0.01156 | | 15.2733 | 15.2852 | 15.3704 | 15.0835 | 15.6146 | 14.9499 | 15.3673 | 15.543 | 5 | 5 | 5 | 5 | 3 | 4 | 5 | 5 |
| AHCYL2 | Q96HN2;Q | 0.003189 | 0.000591 | -0.00189482 | | 0.003909 | -0.01171 | | 15.2507 | 15.3786 | 15.3398 | 15.7741 | 15.3661 | 15.1395 | 15.1419 | 15.2032 | 2 | 2 | 2 | 2 | 1 | 2 | 2 | 2 |
| CHMP5 | Q9NZZ3 | 2.18E-15 | 3.39E-15 | -0.00187314 | | 0.008773 | -0.02613 | | 17.4461 | 17.4198 | 17.2889 | 17.2722 | 17.4846 | 17.2745 | 17.4488 | 17.4539 | 4 | 5 | 5 | 5 | 5 | 4 | 4 | 4 |
| GNAQ | P50148 | 0.000117 | 2.74E-05 | -0.0018144 | | 0.005532 | -0.01654 | | 17.1227 | 17.0733 | 17.0771 | 17.0698 | 16.9506 | 17.242 | 17.314 | 16.8879 | 7 | 7 | 6 | 8 | 9 | 7 | 9 | 9 |
| DGKZ | Q13574;Q | 0.001886 | 0.000372 | -0.00176487 | | 0.002284 | -0.00685 | | 14.7477 | 14.3108 | 14.3967 | 14.3828 | 14.4039 | 14.8154 | 13.9143 | 14.9177 | 3 | 3 | 4 | 3 | 4 | 2 | 2 | 1 |
| MAGT1 | Q9H0U3 | 2.90E-11 | 1.54E-11 | -0.00167834 | | 0.005791 | -0.0173 | | 17.3661 | 17.2861 | 17.3154 | 17.4438 | 17.0444 | 17.3991 | 17.3853 | 17.3484 | 8 | 8 | 8 | 8 | 8 | 8 | 8 | 8 |
| PSPC1 | Q8WXF1 | 3.04E-05 | 7.61E-06 | -0.00158208 | | 0.005432 | -0.01624 | | 16.8025 | 16.8199 | 16.7301 | 16.9421 | 16.8841 | 16.6879 | 16.5553 | 16.8594 | 15 | 13 | 16 | 14 | 11 | 12 | 11 | 14 |
| LPIN1 | Q14693-7 | 4.39E-06 | 1.19E-06 | -0.00147107 | | 0.00148 | -0.00444 | | 14.7831 | 14.788 | 14.7865 | 15.4942 | 15.1565 | 14.1131 | 14.411 | 14.7617 | 6 | 8 | 9 | 8 | 6 | 4 | 8 | 5 |
| EXOSC2 | Q13868 | 0.000136 | 3.18E-05 | -0.00143909 | | 0.003151 | -0.00944 | | 16.126 | 16.1066 | 16.2035 | 16.4387 | 16.18 | 16.0019 | 15.7993 | 16.314 | 7 | 7 | 7 | 7 | 6 | 6 | 5 | 6 |
| SLC27A4 | Q6P1M0 | 3.83E-19 | 1.95E-18 | -0.00133298 | | 0.008229 | -0.02452 | | 16.0546 | 16.0584 | 16.0721 | 16.1866 | 16.0643 | 16.022 | 16.1004 | 15.9418 | 19 | 22 | 21 | 19 | 18 | 21 | 21 | 21 |
| SOS1 | Q07889 | 3.41E-05 | 8.51E-06 | -0.00120583 | | 0.004891 | -0.01463 | | 15.2016 | 15.0357 | 14.9945 | 15.0592 | 15.2744 | 15.0412 | 15.0445 | 14.9732 | 9 | 13 | 13 | 11 | 9 | 10 | 13 | 13 |
| MAN2A1 | Q16706 | 1.25E-17 | 3.84E-17 | -0.000953039 | | 0.002934 | -0.00879 | | 17.0462 | 17.0833 | 17.0251 | 17.3077 | 16.8084 | 17.0411 | 17.1051 | 16.9999 | 35 | 35 | 36 | 39 | 35 | 34 | 34 | 35 |
| PRKAR2B | P31323 | 5.11E-21 | 5.05E-20 | -0.000894038 | | 0.003932 | -0.01177 | | 16.9724 | 17.0071 | 17.0018 | 16.906 | 17.2174 | 16.9418 | 16.9713 | 16.9368 | 9 | 9 | 10 | 7 | 9 | 8 | 7 | 7 |
| TNPO1 | Q92973;Q | 1.16E-09 | 4.76E-10 | -0.000800578 | | 0.00665 | -0.01985 | | 18.4774 | 18.4345 | 18.3989 | 18.5211 | 18.4073 | 18.3788 | 18.397 | 18.4845 | 26 | 27 | 26 | 26 | 25 | 25 | 27 | 25 |
| HINT3 | Q9NQE9 | 2.56E-24 | 7.58E-23 | -0.000796064 | | 0.00205 | -0.00615 | | 15.2801 | 15.4706 | 15.2874 | 15.0884 | 15.3221 | 15.4361 | 15.6319 | 15.2555 | 5 | 5 | 5 | 4 | 3 | 5 | 4 | 3 |
| RHOQ | P17081 | 0.000949 | 0.000196 | -0.000768471 | | 0.002232 | -0.0067 | | 14.6998 | 14.595 | 14.5871 | 14.5046 | 14.8442 | 14.7829 | 14.613 | 14.3956 | 3 | 3 | 3 | 2 | 2 | 4 | 2 | 2 |
| ZC3H18 | Q86VM9 | 0.000215 | 4.86E-05 | -0.000512822 | | 0.001165 | -0.0035 | | 13.7465 | 13.6796 | 13.7667 | 13.9954 | 13.9487 | 13.4558 | 13.5206 | 13.7369 | 6 | 6 | 5 | 5 | 5 | 5 | 5 | 4 |
| ERF | P50548;P5 | 0.014524 | 0.00245 | -0.000486294 | | 0.000741 | -0.00225 | | 14.7852 | 14.2258 | 14.9241 | 14.9238 | NaN | 14.671 | 14.446 | 14.5414 | 1 | 2 | 2 | 1 | NaN | 1 | 2 | 2 |
| MTRES1 | Q9P0P8 | 0.006841 | 0.0012 | -0.000429789 | | 0.000539 | -0.00163 | | 15.0219 | 15.959 | 15.2502 | 15.2215 | NaN | 15.6852 | 15.3508 | 15.3858 | 2 | 1 | 1 | 2 | NaN | 2 | 2 | 1 |
| PARP4 | Q9UKK3 | 3.01E-23 | 5.66E-22 | -0.000377528 | | 0.00214 | -0.00642 | | 16.7387 | 16.7711 | 16.7628 | 16.8284 | 16.8934 | 16.6936 | 16.6662 | 16.7082 | 41 | 41 | 42 | 42 | 41 | 42 | 41 | 41 |
| SMARCE1 | Q969G3;Q | 9.09E-12 | 5.32E-12 | -0.000233014 | | 0.000769 | -0.00231 | | 15.7008 | 15.6266 | 15.5971 | 15.7611 | 15.6858 | 15.3596 | 15.6471 | 15.7549 | 7 | 8 | 6 | 6 | 7 | 5 | 6 | 8 |
| VPS11 | Q9H270 | 3.06E-11 | 1.62E-11 | -9.75E-05 | | 0.000764 | -0.0023 | | 16.1982 | 16.2257 | 16.2022 | 16.0937 | 16.25 | 16.2677 | 16.2423 | 16.1904 | 26 | 30 | 28 | 25 | 28 | 28 | 28 | 25 |
| YAF2 | Q8IY57;Q8 | 0.00012 | 2.80E-05 | 0.000351747 | | 0.000174 | 0.000527 | | 12.5687 | 12.7903 | 12.3528 | 13.1796 | NaN | 10.902 | 13.0271 | 13.1723 | 2 | 2 | 2 | 2 | NaN | 2 | 2 | 2 |
| TK2 | O00142;O | 7.85E-11 | 3.83E-11 | 0.000453695 | | 0.001758 | 0.005278 | | 16.4633 | 16.4814 | 16.4271 | 16.302 | 16.6028 | 16.5934 | 16.324 | 16.4618 | 6 | 7 | 6 | 6 | 4 | 7 | 5 | 6 |
| TERF2 | Q15554 | 0.001298 | 0.000263 | 0.000774384 | | 0.004861 | 0.014542 | | 15.1485 | 15.1717 | 15.2162 | 15.2071 | 15.2908 | 15.0541 | 15.1544 | 15.1839 | 6 | 7 | 5 | 5 | 3 | 4 | 4 | 5 |
| FNTA | P49354 | 6.39E-09 | 2.38E-09 | 0.000892258 | | 0.006291 | 0.018789 | | 16.6868 | 16.6676 | 16.7115 | 16.6428 | 16.6174 | 16.8018 | 16.7354 | 16.6414 | 12 | 11 | 12 | 10 | 9 | 11 | 11 | 10 |
| AGPAT2 | O15120 | 6.06E-06 | 1.63E-06 | 0.00127881 | | 0.002567 | 0.007698 | | 15.9892 | 15.8455 | 15.9773 | 16.4036 | 15.8291 | 15.9079 | 15.6937 | 15.8459 | 4 | 5 | 4 | 4 | 2 | 4 | 3 | 4 |
| ARIH1 | Q9Y4X5 | 3.32E-16 | 6.48E-16 | 0.00143108 | | 0.013738 | 0.04069 | | 15.9258 | 15.8602 | 15.9362 | 15.916 | 15.8887 | 15.8593 | 15.9899 | 15.876 | 12 | 12 | 12 | 12 | 11 | 12 | 12 | 12 |
| XRN2 | Q9H0D6 | 4.41E-12 | 2.73E-12 | 0.00152321 | | 0.008402 | 0.025034 | | 16.1909 | 16.1524 | 16.1537 | 16.2066 | 16.2703 | 16.1919 | 16.1511 | 16.0008 | 27 | 28 | 27 | 28 | 23 | 27 | 27 | 23 |
| CACYBP | Q9HB71 | 1.93E-08 | 6.75E-09 | 0.00165532 | | 0.002302 | 0.006905 | | 18.0163 | 18.039 | 17.9372 | 18.6426 | 17.9483 | 17.7159 | 17.623 | 18.0494 | 19 | 18 | 18 | 19 | 17 | 19 | 17 | 19 |
| IPO13 | O94829 | 8.46E-13 | 6.22E-13 | 0.00187143 | | 0.007604 | 0.022676 | | 15.3944 | 15.3478 | 15.391 | 15.1725 | 15.4926 | 15.3973 | 15.5025 | 15.3144 | 14 | 15 | 15 | 14 | 10 | 13 | 12 | 12 |
| MED12 | Q93074;Q | 1.36E-05 | 3.51E-06 | 0.00187435 | | 0.007753 | 0.023118 | | 14.1323 | 14.292 | 14.4763 | 14.4037 | 14.2548 | 14.2682 | 14.2939 | 14.2709 | 7 | 5 | 3 | 5 | 4 | 4 | 5 | 4 |
| RPE | Q96AT9 | 6.63E-10 | 2.80E-10 | 0.00199674 | | 0.004394 | 0.013152 | | 17.8715 | 17.5755 | 17.7992 | 17.3901 | 17.7975 | 18.0213 | 17.7074 | 17.8173 | 5 | 5 | 5 | 4 | 5 | 5 | 5 | 5 |
| DDX50 | Q9BQ39 | 4.32E-22 | 5.57E-21 | 0.0020078 | | 0.007088 | 0.02115 | | 15.8962 | 15.895 | 15.7961 | 16.0134 | 15.8837 | 15.6178 | 15.8239 | 15.9635 | 17 | 18 | 15 | 17 | 15 | 15 | 18 | 15 |
| TMED10 | P49755 | 2.50E-11 | 1.35E-11 | 0.00204315 | | 0.011222 | 0.033332 | | 19.7157 | 19.6724 | 19.7251 | 19.7089 | 19.5963 | 19.7778 | 19.8226 | 19.6062 | 10 | 10 | 10 | 10 | 10 | 10 | 9 | 10 |
| ISOC1 | Q96CN7 | 2.03E-05 | 5.18E-06 | 0.0020462 | | 0.005366 | 0.016042 | | 15.4191 | 15.4291 | 15.2685 | 15.6365 | 15.4856 | 15.3906 | 15.1433 | 15.195 | 7 | 7 | 7 | 8 | 6 | 7 | 8 | 7 |
| GALNT5 | Q7Z7M9 | 3.06E-23 | 5.67E-22 | 0.00218849 | | 0.004931 | 0.01475 | | 16.225 | 16.346 | 16.3406 | 16.4643 | 16.1133 | 16.2624 | 16.628 | 16.0403 | 16 | 18 | 18 | 18 | 14 | 17 | 18 | 16 |
| FDPS | P14324 | 8.57E-09 | 3.14E-09 | 0.0022363 | | 0.003844 | 0.011512 | | 18.2961 | 18.3834 | 18.3095 | 18.4939 | 18.3336 | 17.7728 | 18.4414 | 18.5953 | 9 | 10 | 11 | 9 | 9 | 8 | 10 | 9 |
| PARP10 | Q53GL7 | 0.002757 | 0.00052 | 0.00227369 | | 0.005038 | 0.015068 | | 15.7681 | 15.9299 | 15.9156 | 16.1433 | 15.4729 | 15.9529 | 15.8766 | 15.8988 | 6 | 6 | 4 | 3 | 4 | 4 | 6 | 4 |
| ATP1A1 | P05023;P0 | 1.27E-16 | 2.92E-16 | 0.00244865 | | 0.008776 | 0.026137 | | 18.3972 | 18.4025 | 18.3593 | 18.6136 | 18.3062 | 18.2139 | 18.323 | 18.4627 | 46 | 45 | 48 | 48 | 46 | 48 | 47 | 47 |
| PWP1 | Q13610 | 6.24E-11 | 3.12E-11 | 0.00264626 | | 0.013867 | 0.041069 | | 16.1917 | 16.2861 | 16.2777 | 16.3941 | 16.1629 | 16.158 | 16.221 | 16.3101 | 15 | 16 | 16 | 16 | 12 | 14 | 16 | 13 |
| IDE | P14735 | 1.48E-11 | 8.30E-12 | 0.00273374 | | 0.009547 | 0.028411 | | 16.6219 | 16.6814 | 16.6914 | 16.7546 | 16.7083 | 16.6956 | 16.7695 | 16.3828 | 31 | 32 | 30 | 33 | 25 | 31 | 31 | 27 |
| UBR1 | Q8IWV7 | 2.76E-07 | 8.58E-08 | 0.00304464 | | 0.046185 | 0.132244 | | 15.4671 | 15.4581 | 15.4893 | 15.4285 | 15.5133 | 15.4928 | 15.4337 | 15.4741 | 35 | 32 | 35 | 33 | 27 | 32 | 30 | 30 |
| NUCB2 | P80303;P8 | 6.19E-10 | 2.63E-10 | 0.00314293 | | 0.006166 | 0.018419 | | 17.4443 | 17.4898 | 17.4476 | 17.2189 | 17.3881 | 17.6529 | 17.8454 | 17.182 | 23 | 23 | 23 | 24 | 22 | 23 | 24 | 24 |
| GIT1 | Q9Y2X7;Q | 1.52E-12 | 1.05E-12 | 0.00316435 | | 0.013216 | 0.039168 | | 15.5286 | 15.5305 | 15.5347 | 15.6464 | 15.5755 | 15.6072 | 15.3034 | 15.508 | 15 | 14 | 15 | 15 | 15 | 12 | 13 | 15 |
| TRADD | Q15628 | 2.83E-10 | 1.26E-10 | 0.00319023 | | 0.006478 | 0.019344 | | 16.5892 | 16.5022 | 16.6327 | 16.8442 | 16.6005 | 16.1154 | 16.6171 | 16.6805 | 7 | 7 | 7 | 7 | 7 | 7 | 7 | 7 |
| TST | Q16762 | 3.61E-12 | 2.29E-12 | 0.00328331 | | 0.005397 | 0.016135 | | 16.302 | 16.3484 | 16.3582 | 16.823 | 16.3359 | 16.1722 | 15.8995 | 16.434 | 9 | 9 | 9 | 9 | 9 | 9 | 9 | 10 |
| ENO1 | P06733 | 4.14E-10 | 1.80E-10 | 0.00342331 | | 0.00694 | 0.020713 | | 21.8641 | 21.7884 | 21.7428 | 22.0034 | 22.1591 | 21.6747 | 21.5096 | 21.6282 | 36 | 36 | 37 | 36 | 36 | 36 | 35 | 36 |
| NLRX1 | Q86UT6 | 1.82E-05 | 4.66E-06 | 0.00345408 | | 0.002619 | 0.007854 | | 14.6464 | 14.7721 | 14.7397 | 14.974 | 13.4371 | 15.0831 | 15.2843 | 14.8011 | 4 | 4 | 5 | 4 | 1 | 4 | 4 | 4 |

| Gene names | UniProt IDs | Protein Group P-value | Protein Group Q-value | Log2 Difference NUP214 vs Controls (n=5) | p<0.05 | -log10 p-value | T-test Test statistic | NPC Localization | Log2 MS2_quantity_NUP214_1 | Log2 MS2_quantity_NUP214_2 | Log2 MS2_quantity_NUP214_3 | Log2 MS2_quantity_Control_1 | Log2 MS2_quantity_Control_2 | Log2 MS2_quantity_Control_3 | Log2 MS2_quantity_Control_4 | Log2 MS2_quantity_Control_5 | Number Peptides for Quantitation NUP214_1 | Number Peptides for Quantitation NUP214_2 | Number Peptides for Quantitation NUP214_3 | Number Peptides for Quantitation Control_1 | Number Peptides for Quantitation Control_2 | Number Peptides for Quantitation Control_3 | Number Peptides for Quantitation Control_4 | Number Peptides for Quantitation Control_5 |
|---|---|---|---|---|---|---|---|---|---|---|---|---|---|---|---|---|---|---|---|---|---|---|---|---|
| CORO1C | Q9ULV4;Q9 | 1.93E-06 | 5.42E-07 | 0.00398687 | | 0.015451 | 0.04568 | | 19.903 | 19.8777 | 19.809 | 19.9713 | 19.9418 | 19.8131 | 19.6293 | 19.9404 | 25 | 23 | 24 | 23 | 25 | 23 | 24 | 25 |
| CC2D1A | Q6P1N0 | 1.62E-22 | 2.42E-21 | 0.00408045 | | 0.017012 | 0.050208 | | 16.4069 | 16.2641 | 16.4075 | 16.1681 | 16.3953 | 16.4804 | 16.4304 | 16.3031 | 23 | 20 | 21 | 20 | 16 | 19 | 20 | 20 |
| PNISR | Q8TF01 | 0.002105 | 0.000411 | 0.00414745 | | 0.008814 | 0.026467 | | 13.8736 | 14.0913 | 14.3753 | NaN | 13.9502 | 14.011 | 14.1488 | 14.3269 | 3 | 3 | 2 | NaN | 2 | 1 | 2 | 1 |
| ACTR2 | P61160 | 1.02E-14 | 1.29E-14 | 0.00415497 | | 0.020804 | 0.06115 | | 19.9462 | 19.9671 | 19.951 | 20.0419 | 20.0304 | 20.0241 | 19.799 | 19.8578 | 18 | 18 | 18 | 18 | 17 | 18 | 18 | 18 |
| ZNF852;ZKSCAN7 | Q6ZMS4;Q9 | 0.001126 | 0.000231 | 0.00420488 | | 0.005938 | 0.017742 | | 11.9571 | 11.6866 | 11.6835 | 11.7559 | 12.2292 | 11.4841 | 12.0673 | 11.3212 | 2 | 2 | 2 | 2 | 2 | 1 | 2 | 2 |
| FAF1 | Q9UNN5 | 5.33E-07 | 1.60E-07 | 0.00421371 | | 0.017858 | 0.052658 | | 16.3422 | 16.4215 | 16.4188 | 16.6013 | 16.4016 | 16.2784 | 16.2867 | 16.3819 | 12 | 12 | 13 | 12 | 10 | 12 | 11 | 12 |
| FKBP8 | Q14318 | 6.91E-14 | 6.89E-14 | 0.00426229 | | 0.012277 | 0.036421 | | 17.4009 | 17.417 | 17.3985 | 17.3722 | 17.6969 | 17.1624 | 17.3232 | 17.4513 | 15 | 14 | 14 | 14 | 15 | 15 | 15 | 15 |
| YWHAE | P62258 | 7.94E-12 | 4.71E-12 | 0.00428365 | | 0.008665 | 0.025811 | | 20.063 | 19.9813 | 20.2443 | 20.3161 | 20.0518 | 19.6592 | 20.1736 | 20.2588 | 20 | 21 | 20 | 20 | 21 | 19 | 20 | 19 |
| CCT5 | P48643 | 1.49E-16 | 3.37E-16 | 0.00431074 | | 0.029645 | 0.086323 | | 18.7892 | 18.8331 | 18.7768 | 18.9185 | 18.7334 | 18.767 | 18.7245 | 18.8336 | 37 | 37 | 38 | 36 | 34 | 35 | 38 | 38 |
| PSMG3 | Q9BT73 | 4.51E-07 | 1.37E-07 | 0.00448659 | | 0.017436 | 0.051437 | | 16.1184 | 16.2655 | 16.2968 | 16.0509 | 16.13 | 16.2461 | 16.3486 | 16.3364 | 4 | 4 | 4 | 4 | 4 | 4 | 4 | 4 |
| KLC4 | Q9NSK0;Q9 | 1.91E-13 | 1.69E-13 | 0.00462252 | | 0.013589 | 0.040257 | | 16.8441 | 16.9607 | 16.7769 | 16.5914 | 16.9164 | 16.999 | 17.0189 | 16.754 | 17 | 18 | 18 | 13 | 13 | 17 | 16 | 12 |
| YTHDF1 | Q9BYJ9 | 0.00048 | 0.000104 | 0.00486399 | | 0.007219 | 0.021539 | | 14.8526 | 15.0012 | 15.0604 | 15.2899 | 14.4454 | 15.3221 | 15.0214 | 14.7539 | 5 | 4 | 5 | 4 | 3 | 4 | 4 | 4 |
| RHOG | P84095 | 4.19E-11 | 2.15E-11 | 0.00491994 | | 0.028948 | 0.084357 | | 17.6386 | 17.7842 | 17.7463 | 17.8223 | 17.7529 | 17.713 | 17.705 | 17.5973 | 10 | 10 | 10 | 9 | 8 | 9 | 9 | 10 |
| PCBP2 | Q15366;Q1 | 5.70E-09 | 2.14E-09 | 0.00495745 | | 0.063062 | 0.177648 | | 20.0537 | 20.1109 | 20.1465 | 20.1525 | 20.0765 | 20.0822 | 20.0738 | 20.1086 | 14 | 14 | 14 | 14 | 14 | 14 | 14 | 14 |
| PSME3 | P61289;P6 | 1.15E-17 | 3.60E-17 | 0.00512098 | | 0.010002 | 0.029747 | | 17.5419 | 17.4185 | 17.5041 | 17.6903 | 17.4676 | 17.0249 | 17.4805 | 17.7519 | 14 | 14 | 14 | 14 | 14 | 14 | 14 | 14 |
| NDRG3 | Q9UGV2;Q | 1.93E-09 | 7.67E-10 | 0.00520223 | | 0.010696 | 0.031788 | | 17.4903 | 17.4586 | 17.4296 | 17.4477 | 17.8462 | 17.2399 | 17.1624 | 17.5754 | 8 | 8 | 8 | 8 | 6 | 7 | 7 | 7 |
| MIPEP | Q99797 | 2.24E-08 | 7.78E-09 | 0.0057244 | | 0.020526 | 0.060352 | | 15.9087 | 15.9291 | 15.8835 | 15.8982 | 16.1489 | 15.7248 | 15.8153 | 15.9196 | 13 | 12 | 14 | 11 | 11 | 12 | 13 | 10 |
| RPL21 | P46778 | 0.000181 | 4.15E-05 | 0.00572497 | | 0.029419 | 0.085685 | | 19.4557 | 19.585 | 19.4251 | 19.5018 | 19.3694 | 19.6318 | 19.4511 | 19.4164 | 6 | 6 | 6 | 6 | 7 | 6 | 6 | 6 |
| UBE2R2 | Q712K3 | 1.24E-10 | 5.87E-11 | 0.00574557 | | 0.041401 | 0.119112 | | 16.379 | 16.4473 | 16.3404 | 16.4957 | 16.3846 | 16.3513 | 16.3824 | 16.3018 | 6 | 6 | 7 | 7 | 6 | 7 | 7 | 7 |
| STIP1 | P31948 | 2.09E-13 | 1.83E-13 | 0.00576401 | | 0.020522 | 0.060339 | | 18.4227 | 18.466 | 18.4062 | 18.6943 | 18.3199 | 18.379 | 18.3026 | 18.4336 | 44 | 44 | 44 | 43 | 41 | 43 | 43 | 43 |
| VCPIP1 | Q96JH7 | 5.38E-16 | 9.77E-16 | 0.0058204 | | 0.020689 | 0.06082 | | 15.5663 | 15.58 | 15.561 | 15.3875 | 15.8209 | 15.5711 | 15.5461 | 15.4909 | 27 | 26 | 27 | 26 | 20 | 27 | 26 | 21 |
| POLDIP2 | Q9Y2S7 | 3.48E-19 | 1.80E-18 | 0.00592321 | | 0.030977 | 0.090076 | | 16.2248 | 16.29 | 16.2853 | 16.2153 | 16.2129 | 16.1422 | 16.3164 | 16.4172 | 13 | 13 | 13 | 13 | 11 | 12 | 12 | 10 |
| SCPEP1 | Q9HB40 | 1.04E-17 | 3.32E-17 | 0.00602786 | | 0.005373 | 0.016064 | | 15.0298 | 15.0417 | 15.157 | 15.6569 | 14.8597 | 14.1414 | 15.0544 | 15.6384 | 10 | 10 | 10 | 10 | 8 | 6 | 10 | 10 |
| STAT1 | P42224 | 4.73E-20 | 3.28E-19 | 0.00621732 | | 0.015639 | 0.046225 | | 18.8334 | 18.8251 | 18.8229 | 18.6007 | 18.8487 | 19.1165 | 18.594 | 18.9447 | 42 | 41 | 42 | 41 | 40 | 41 | 41 | 42 |
| TMED9 | Q9BVK6 | 3.14E-14 | 3.43E-14 | 0.00623525 | | 0.032267 | 0.093703 | | 19.5219 | 19.6721 | 19.5877 | 19.6736 | 19.4691 | 19.6624 | 19.6393 | 19.494 | 11 | 11 | 11 | 10 | 10 | 12 | 11 | 12 |
| TAB1 | Q15750;Q1 | 1.19E-16 | 2.76E-16 | 0.0063254 | | 0.024992 | 0.07313 | | 16.2921 | 16.2317 | 16.2101 | 16.061 | 16.4267 | 16.2268 | 16.1563 | 16.3207 | 13 | 13 | 11 | 10 | 11 | 13 | 13 | 10 |
| SLIRP | Q9GZT3;Q9 | 3.94E-11 | 2.03E-11 | 0.00637894 | | 0.013689 | 0.040547 | | 17.4183 | 17.4863 | 17.4894 | 17.7546 | 17.5879 | 17.0455 | 17.4608 | 17.4428 | 6 | 6 | 6 | 6 | 6 | 6 | 6 | 6 |
| PRKCI | P41743 | 5.96E-09 | 2.23E-09 | 0.00650584 | | 0.025373 | 0.074216 | | 16.3662 | 16.2914 | 16.216 | 16.1847 | 16.5222 | 16.1984 | 16.2604 | 16.2576 | 7 | 9 | 8 | 8 | 6 | 7 | 6 | 8 |
| CCDC88A | Q3V6T2;Q3 | 1.05E-13 | 9.96E-14 | 0.00671508 | | 0.016279 | 0.048082 | | 15.1333 | 15.2226 | 15.1703 | 15.3664 | 15.3017 | 14.8235 | 15.0356 | 15.3162 | 24 | 25 | 25 | 26 | 22 | 18 | 12 | 24 |
| ATP5IF1 | Q9UII2 | 0.00211 | 0.000412 | 0.00673205 | | 0.024938 | 0.072978 | | 17.2087 | 17.0757 | 17.0859 | 16.933 | 17.2433 | 17.0155 | 17.2742 | 17.1176 | 3 | 4 | 3 | 2 | 3 | 3 | 4 | 4 |
| SNRNP27 | Q8WVK2 | 0.002279 | 0.000441 | 0.00679239 | | 0.017346 | 0.051175 | | 15.4392 | 15.7857 | 15.7034 | 15.4874 | 15.5038 | 15.9109 | 15.5455 | 15.7324 | 4 | 2 | 4 | 2 | 3 | 2 | 4 | 2 |
| RPP30 | P78346;P7 | 2.30E-10 | 1.04E-10 | 0.00680275 | | 0.060688 | 0.171346 | | 15.3128 | 15.4391 | 15.4056 | 15.372 | 15.3323 | 15.3648 | 15.3661 | 15.46 | 12 | 13 | 12 | 10 | 11 | 11 | 11 | 12 |
| PPP1R12A | O14974 | 3.05E-18 | 1.15E-17 | 0.00685552 | | 0.055681 | 0.157962 | | 17.2707 | 17.3004 | 17.2959 | 17.1654 | 17.3303 | 17.2797 | 17.2845 | 17.3508 | 51 | 53 | 51 | 52 | 50 | 52 | 50 | 53 |
| CHERP | Q8IWX8 | 4.99E-09 | 1.88E-09 | 0.00693741 | | 0.046382 | 0.132782 | | 15.3854 | 15.4831 | 15.4917 | 15.4446 | 15.395 | 15.3533 | 15.5487 | 15.4909 | 15 | 15 | 17 | 13 | 12 | 13 | 11 | 15 |
| CSRP1 | P21291 | 5.40E-11 | 2.72E-11 | 0.00694784 | | 0.063139 | 0.177854 | | 19.2978 | 19.2403 | 19.3374 | 19.2445 | 19.2508 | 19.3782 | 19.2945 | 19.2564 | 11 | 11 | 11 | 10 | 11 | 11 | 11 | 11 |
| CCDC124 | Q96CT7 | 1.87E-14 | 2.19E-14 | 0.00703176 | | 0.038935 | 0.112297 | | 17.1855 | 17.2014 | 17.1846 | 17.0485 | 17.2042 | 17.2742 | 17.2869 | 17.1038 | 13 | 14 | 14 | 13 | 11 | 13 | 13 | 14 |
| FBXO11 | Q86XK2;Q8 | 0.001551 | 0.000311 | 0.00706522 | | 0.006467 | 0.019471 | | 13.4262 | 12.7351 | 13.7106 | NaN | 13.7308 | 13.2796 | 12.6576 | 13.4663 | 1 | 2 | 1 | NaN | 1 | 2 | 1 | 1 |
| TRIM38 | O00635 | 1.05E-10 | 5.02E-11 | 0.00708243 | | 0.020755 | 0.061007 | | 14.5497 | 14.5927 | 14.5556 | 14.4346 | 14.7879 | 14.7088 | 14.5496 | 14.3137 | 5 | 5 | 6 | 6 | 3 | 6 | 3 | 4 |
| SRM | P19623 | 3.68E-14 | 3.95E-14 | 0.00716235 | | 0.0197 | 0.057974 | | 18.7101 | 18.7635 | 18.6917 | 18.6602 | 18.4809 | 19.0466 | 18.7014 | 18.6839 | 16 | 17 | 17 | 17 | 17 | 17 | 17 | 16 |
| IMMT | Q16891-2 | 1.57E-08 | 5.57E-09 | 0.00740026 | | 0.06515 | 0.183173 | | 17.4171 | 17.4441 | 17.4189 | 17.4215 | 17.3558 | 17.4751 | 17.4953 | 17.3487 | 38 | 38 | 39 | 39 | 38 | 39 | 40 | 40 |
| EML3 | Q32P44 | 1.82E-08 | 6.41E-09 | 0.00749893 | | 0.0276 | 0.08054 | | 15.6305 | 15.7227 | 15.6423 | 15.6988 | 15.6457 | 15.8601 | 15.4349 | 15.6487 | 16 | 12 | 14 | 14 | 9 | 14 | 13 | 13 |
| FAM83G | A6ND36 | 0.02124 | 0.003527 | 0.0075078 | | 0.005851 | 0.017628 | | NaN | 16.4995 | 15.7581 | 16.5287 | 15.2446 | 16.2884 | 16.1923 | 16.3523 | NaN | 2 | 2 | 5 | 2 | 2 | 2 | 2 |
| RAB34 | Q9BZG1 | 3.40E-12 | 2.16E-12 | 0.00758069 | | 0.033306 | 0.096616 | | 17.3525 | 17.4414 | 17.4688 | 17.601 | 17.4743 | 17.2935 | 17.36 | 17.3378 | 13 | 13 | 13 | 12 | 12 | 12 | 12 | 12 |
| TTC1 | Q99614 | 4.84E-14 | 5.03E-14 | 0.0076093 | | 0.02193 | 0.064383 | | 16.5883 | 16.5542 | 16.649 | 16.6726 | 16.2987 | 16.6048 | 16.8312 | 16.5407 | 12 | 13 | 14 | 14 | 13 | 14 | 12 | 12 |
| WDR11 | Q9BZH6 | 2.68E-16 | 5.42E-16 | 0.00774568 | | 0.066326 | 0.186272 | | 15.4797 | 15.5696 | 15.5164 | 15.5551 | 15.4788 | 15.5337 | 15.5782 | 15.425 | 27 | 27 | 26 | 25 | 19 | 25 | 24 | 23 |
| TBCK | Q8TEA7 | 0.000314 | 6.95E-05 | 0.00795256 | | 0.028027 | 0.081751 | | 15.0446 | 15.0243 | 14.9236 | 14.7643 | 15.1907 | 15.0069 | 15.0502 | 14.9357 | 14 | 13 | 15 | 10 | 7 | 10 | 12 | 10 |
| GEMIN5 | Q8TEQ6 | 4.23E-14 | 4.48E-14 | 0.0079909 | | 0.051054 | 0.145486 | | 15.7183 | 15.8009 | 15.7611 | 15.7871 | 15.6934 | 15.6427 | 15.7683 | 15.8689 | 32 | 32 | 33 | 30 | 25 | 30 | 30 | 29 |
| RBM4B | Q9BQ04 | 3.48E-07 | 1.07E-07 | 0.00824153 | | 0.02146 | 0.063033 | | 15.1424 | 15.1179 | 15.1249 | 15.1496 | 15.4316 | 14.8169 | 15.074 | 15.1287 | 2 | 2 | 2 | 2 | 2 | 2 | 2 | 2 |
| COPS5 | Q92905 | 8.97E-06 | 2.37E-06 | 0.00826073 | | 0.052955 | 0.150625 | | 17.1345 | 17.2061 | 17.1346 | 17.0613 | 17.0977 | 17.2675 | 17.2155 | 17.1087 | 11 | 11 | 13 | 10 | 10 | 11 | 11 | 11 |
| VPS26C | O14972 | 4.68E-07 | 1.41E-07 | 0.00829493 | | 0.028297 | 0.082514 | | 16.6982 | 16.6248 | 16.6057 | 16.4176 | 16.81 | 16.6518 | 16.7702 | 16.5234 | 8 | 9 | 8 | 9 | 8 | 9 | 9 | 8 |
| MRPL40 | Q9NQ50 | 7.34E-07 | 2.17E-07 | 0.00833359 | | 0.054701 | 0.155328 | | 15.9462 | 15.9479 | 16.0643 | 15.9812 | 15.9921 | 15.8783 | 16.0885 | 15.9489 | 5 | 5 | 6 | 5 | 3 | 5 | 5 | 4 |
| PSMA7 | O14818 | 1.09E-17 | 3.45E-17 | 0.00843925 | | 0.0284 | 0.082806 | | 19.4951 | 19.624 | 19.6074 | 19.5284 | 19.3472 | 19.7215 | 19.7373 | 19.5008 | 15 | 15 | 15 | 14 | 14 | 14 | 15 | 14 |
| WBP2 | Q969T9 | 1.00E-06 | 2.91E-07 | 0.00863444 | | 0.036285 | 0.104938 | | 17.2344 | 17.3115 | 17.2465 | 17.1142 | 17.3237 | 17.4105 | 17.1127 | 17.3164 | 7 | 7 | 7 | 7 | 8 | 7 | 8 | 8 |
| ANTXR2 | P58335-4 | 3.36E-09 | 1.30E-09 | 0.00911045 | | 0.010696 | 0.031787 | | 15.902 | 15.9604 | 15.9222 | 16.421 | 16.0852 | 15.9647 | 15.1229 | 16.0016 | 10 | 10 | 9 | 9 | 9 | 8 | 9 | 8 |
| PSMA6 | P60900 | 3.19E-13 | 2.64E-13 | 0.00922076 | | 0.05031 | 0.143473 | | 18.962 | 19.0119 | 18.9564 | 18.8301 | 19.0032 | 19.0947 | 19.0179 | 18.8917 | 15 | 15 | 15 | 14 | 14 | 15 | 14 | 15 |
| PTBP2 | Q9UKA9;Q9 | 7.57E-06 | 2.01E-06 | 0.00935135 | | 0.022555 | 0.066172 | | 19.2822 | 19.2314 | 19.143 | 19.2715 | 19.4379 | 18.9612 | 18.9692 | 19.4079 | 3 | 4 | 3 | 3 | 4 | 3 | 4 | 4 |
| METTL16 | Q86W50 | 0.000511 | 0.00011 | 0.00938536 | | 0.016493 | 0.048704 | | 15.3968 | 15.1824 | 15.0464 | 15.0701 | 15.3475 | 14.7614 | 15.2716 | 15.5452 | 6 | 8 | 7 | 4 | 3 | 3 | 3 | 3 |
| HS1BP3 | Q53T59 | 5.20E-11 | 2.64E-11 | 0.00957692 | | 0.03158 | 0.091773 | | 16.9591 | 16.9319 | 16.904 | 16.8696 | 17.2262 | 16.8938 | 16.8154 | 16.8055 | 13 | 13 | 12 | 13 | 11 | 11 | 12 | 12 |
| CEP43 | O95684;Q9 | 3.02E-06 | 8.34E-07 | 0.009618 | | 0.031287 | 0.090949 | | 14.8011 | 14.857 | 15.028 | 15.0126 | 14.725 | 14.8641 | 14.7493 | 15.0777 | 6 | 7 | 6 | 8 | 6 | 6 | 6 | 7 |
| PNPLA8 | Q9NP80;Q9 | 0.00034 | 7.48E-05 | 0.00976206 | | 0.049849 | 0.142221 | | 15.0397 | 14.9549 | 14.7882 | 14.805 | 14.9155 | 14.9404 | 15.001 | 14.9274 | 3 | 4 | 4 | 4 | 3 | 3 | 4 | 2 |
| PARL | Q9H300 | 0.001846 | 0.000365 | 0.00978877 | | 0.027144 | 0.07925 | | 13.9992 | 14.0411 | 14.1442 | 14.0269 | 13.8583 | 14.3888 | 14.0223 | 13.9623 | 1 | 1 | 1 | 1 | 1 | 1 | 1 | 1 |
| SNX6 | Q9UNH7 | 8.53E-10 | 3.56E-10 | 0.00983531 | | 0.061743 | 0.174149 | | 18.0143 | 18.0043 | 17.9657 | 17.8876 | 18.0051 | 18.0451 | 18.0957 | 17.8912 | 25 | 25 | 25 | 23 | 21 | 23 | 24 | 24 |
| OXR1 | Q8N573-5 | 7.08E-08 | 2.32E-08 | 0.0100229 | | 0.045346 | 0.12995 | | 15.1778 | 15.2139 | 15.2315 | 15.2035 | 15.0832 | 15.301 | 15.3446 | 15.0562 | 18 | 19 | 19 | 17 | 11 | 15 | 18 | 14 |
| SMC2 | O95347 | 1.36E-18 | 5.74E-18 | 0.0100489 | | 0.017857 | 0.052653 | | 15.5728 | 15.6538 | 15.6081 | 15.956 | 15.7332 | 15.1125 | 15.4927 | 15.7133 | 45 | 44 | 46 | 46 | 38 | 30 | 42 | 40 |
| DEGS1 | O15121 | 4.31E-08 | 1.45E-08 | 0.0101231 | | 0.026217 | 0.076617 | | 16.7825 | 16.7936 | 16.7409 | 17.0065 | 16.4621 | 16.6887 | 16.952 | 16.7017 | 5 | 5 | 5 | 4 | 4 | 4 | 4 | 5 |
| CTNND1 | O60716-3; | 8.34E-17 | 2.00E-16 | 0.0102994 | | 0.040984 | 0.117963 | | 17.7079 | 17.7163 | 17.7025 | 17.7793 | 17.4868 | 17.8577 | 17.7501 | 17.6191 | 37 | 38 | 38 | 37 | 37 | 38 | 39 | 38 |
| RAB35 | Q15286 | 3.03E-08 | 1.04E-08 | 0.0106499 | | 0.064008 | 0.180155 | | 17.0294 | 17.0506 | 17.1254 | 17.1238 | 17.1095 | 17.1249 | 16.9123 | 17.0188 | 7 | 7 | 7 | 7 | 7 | 7 | 7 | 7 |
| FBXO30 | Q8TB52 | 2.45E-09 | 9.62E-10 | 0.0107365 | | 0.028115 | 0.082001 | | 15.3041 | 15.3291 | 15.2671 | 15.5728 | 14.9982 | 15.2292 | 15.2236 | 15.423 | 9 | 11 | 9 | 11 | 7 | 8 | 10 | 8 |
| ZFPL1 | O95159 | 3.57E-06 | 9.81E-07 | 0.0107903 | | 0.089784 | 0.246876 | | 16.9245 | 16.9972 | 16.9225 | 16.9614 | 16.8917 | 16.9195 | 17.0409 | 16.8729 | 8 | 8 | 8 | 8 | 8 | 8 | 8 | 8 |
| CWC15 | Q9P013 | 1.14E-14 | 1.42E-14 | 0.0110168 | | 0.046674 | 0.133579 | | 14.9517 | 14.9455 | 14.8872 | 14.9376 | 15.1335 | 14.8416 | 14.773 | 14.9 | 5 | 4 | 5 | 6 | 6 | 5 | 5 | 5 |
| PHPT1 | Q9NRX4 | 4.49E-13 | 3.57E-13 | 0.0110784 | | 0.016826 | 0.049671 | | 17.3191 | 17.1979 | 17.2705 | 17.11 | 16.9116 | 17.8405 | 17.3761 | 17.0189 | 5 | 5 | 5 | 5 | 5 | 5 | 5 | 5 |
| SAMD1 | Q6SPF0 | 0.001624 | 0.000325 | 0.0113838 | | 0.052631 | 0.149752 | | 15.5228 | 15.4583 | 15.3926 | 15.4634 | 15.5191 | 15.3972 | 15.2719 | 15.5809 | 2 | 2 | 2 | 2 | 2 | 2 | 2 | 2 |
| THOC1 | Q96FV9 | 9.57E-11 | 4.60E-11 | 0.0113842 | | 0.042484 | 0.122096 | | 15.4503 | 15.4388 | 15.4509 | 15.4468 | 15.6282 | 15.2954 | 15.2638 | 15.5421 | 16 | 14 | 14 | 15 | 12 | 13 | 12 | 13 |
| ECPAS | Q5VYK3 | 8.91E-18 | 2.91E-17 | 0.0115128 | | 0.0833 | 0.230358 | | 16.7382 | 16.7465 | 16.7369 | 16.775 | 16.7856 | 16.8065 | 16.6545 | 16.6233 | 59 | 60 | 63 | 61 | 57 | 59 | 58 | 58 |
| TRMT112 | Q9UI30 | 9.66E-09 | 3.52E-09 | 0.011651 | | 0.016124 | 0.047635 | | 18.2617 | 17.8791 | 18.353 | 18.1227 | 17.9119 | 17.6935 | 18.449 | 18.5875 | 3 | 3 | 3 | 3 | 3 | 3 | 3 | 3 |
| DDX18 | Q9NVP1 | 4.67E-12 | 2.88E-12 | 0.0117369 | | 0.11848 | 0.318034 | | 16.6491 | 16.5425 | 16.4986 | 16.5039 | 16.5635 | 16.5512 | 16.5581 | 16.5815 | 15 | 17 | 18 | 15 | 12 | 18 | 17 | 16 |
| CBR1 | P16152 | 5.58E-14 | 5.70E-14 | 0.0118144 | | 0.078348 | 0.217624 | | 19.9657 | 20.0237 | 19.9398 | 20.0312 | 20.003 | 19.9714 | 19.8158 | 20.0016 | 19 | 19 | 19 | 20 | 18 | 19 | 18 | 18 |
| ITPRIP | Q8IWB1 | 2.79E-10 | 1.25E-10 | 0.0121036 | | 0.017626 | 0.051987 | | 14.4922 | 14.4198 | 14.1988 | 14.1021 | 13.8868 | 14.8259 | 14.5949 | 14.3811 | 7 | 6 | 7 | 7 | 4 | 11 | 11 | 6 |

| Gene names | UniProt IDs | Protein Group P-value | Protein Group Q-value | Log2 Difference NUP214 vs Controls (n=5) | p<0.05 | -log10 p-value | T-test Test statistic | NPC Localization | Log2 MS2_quantity_NUP214_1 | Log2 MS2_quantity_NUP214_2 | Log2 MS2_quantity_NUP214_3 | Log2 MS2_quantity_Control_1 | Log2 MS2_quantity_Control_2 | Log2 MS2_quantity_Control_3 | Log2 MS2_quantity_Control_4 | Log2 MS2_quantity_Control_5 | Number Peptides for Quantitation NUP214_1 | Number Peptides for Quantitation NUP214_2 | Number Peptides for Quantitation NUP214_3 | Number Peptides for Quantitation Control_1 | Number Peptides for Quantitation Control_2 | Number Peptides for Quantitation Control_3 | Number Peptides for Quantitation Control_4 | Number Peptides for Quantitation Control_5 |
|---|---|---|---|---|---|---|---|---|---|---|---|---|---|---|---|---|---|---|---|---|---|---|---|---|
| XRCC1 | P18887 | 3.14E-08 | 1.07E-08 | 0.0121155 | | 0.046788 | 0.13389 | | 15.4392 | 15.3731 | 15.3697 | 15.2521 | 15.3631 | 15.2298 | 15.5742 | 15.4902 | 9 | 11 | 9 | 10 | 9 | 7 | 9 | 8 |
| RBM3 | P98179 | 1.63E-11 | 9.08E-12 | 0.0121413 | | 0.02632 | 0.076909 | | 17.2892 | 17.4446 | 17.4137 | 17.0958 | 17.6027 | 17.5874 | 17.0891 | 17.4766 | 3 | 3 | 3 | 4 | 3 | 3 | 3 | 4 |
| PPP2R3A | Q06190 | 8.08E-09 | 2.98E-09 | 0.012211 | | 0.016762 | 0.049485 | | 14.5328 | 14.4737 | 14.4723 | 14.6231 | 14.9632 | 13.8577 | 14.3357 | 14.6239 | 9 | 8 | 10 | 9 | 8 | 3 | 10 | 5 |
| GPI | P06744 | 2.87E-14 | 3.19E-14 | 0.0122344 | | 0.031867 | 0.092579 | | 18.8844 | 18.8323 | 18.7893 | 18.4315 | 18.9313 | 18.9228 | 18.9072 | 18.9226 | 25 | 25 | 25 | 23 | 24 | 25 | 25 | 25 |
| TMEM63B | Q5T3F8;Q5 | 0.000457 | 9.89E-05 | 0.0123986 | | 0.01844 | 0.05434 | | 14.8519 | 14.9836 | 14.899 | 15.469 | 14.5607 | 14.5706 | 15.0588 | 14.8363 | 2 | 3 | 4 | 5 | 2 | 4 | 4 | 3 |
| DLD | P09622 | 5.96E-11 | 2.99E-11 | 0.0125079 | | 0.11258 | 0.303649 | | 18.2423 | 18.2343 | 18.1948 | 18.2407 | 18.2502 | 18.1701 | 18.116 | 18.2794 | 17 | 16 | 16 | 16 | 17 | 15 | 16 | 17 |
| BCL7B | Q9BQE9;Q9 | 0.003724 | 0.00068 | 0.0127691 | | 0.035093 | 0.101615 | | 17.0718 | 17.1993 | 16.8578 | 17.0208 | 16.8753 | 16.9084 | 17.3116 | 17.0349 | 1 | 1 | 1 | 1 | 1 | 1 | 1 | 1 |
| TPP2 | P29144 | 2.46E-12 | 1.62E-12 | 0.0127997 | | 0.102883 | 0.279737 | | 17.0749 | 17.0945 | 17.0701 | 17.0306 | 17.0794 | 17.0086 | 17.1947 | 17.0219 | 55 | 54 | 58 | 55 | 49 | 54 | 54 | 52 |
| CAVIN3 | Q969G5 | 1.17E-06 | 3.37E-07 | 0.012853 | | 0.021737 | 0.063828 | | 19.7517 | 19.6914 | 19.7362 | 19.5777 | 20.0418 | 19.2056 | 19.9719 | 19.7711 | 9 | 9 | 9 | 9 | 9 | 9 | 10 | 10 |
| SRP54 | P61011 | 6.20E-12 | 3.76E-12 | 0.0128719 | | 0.057314 | 0.162339 | | 17.6648 | 17.622 | 17.6327 | 17.6686 | 17.444 | 17.7985 | 17.6601 | 17.5635 | 21 | 21 | 21 | 21 | 20 | 21 | 21 | 21 |
| TUBB4B | P68371 | 4.00E-15 | 5.73E-15 | 0.0129447 | | 0.063197 | 0.178007 | | 23.7833 | 23.832 | 23.7895 | 23.8946 | 23.8767 | 23.7401 | 23.6016 | 23.8305 | 24 | 24 | 24 | 24 | 24 | 24 | 24 | 24 |
| EIF5A | P63241 | 1.69E-10 | 7.83E-11 | 0.0129604 | | 0.054468 | 0.154702 | | 19.7518 | 19.7557 | 19.8183 | 19.8707 | 19.5573 | 19.9079 | 19.7367 | 19.739 | 16 | 17 | 16 | 15 | 16 | 17 | 16 | 17 |
| UBXN4 | Q92575 | 5.58E-15 | 7.57E-15 | 0.0130155 | | 0.056533 | 0.160249 | | 17.1558 | 17.0679 | 17.0444 | 16.8738 | 17.149 | 17.1494 | 17.1885 | 17.0208 | 9 | 9 | 9 | 10 | 9 | 9 | 10 | 9 |
| MTMR10 | Q9NXD2 | 3.85E-24 | 1.02E-22 | 0.0131146 | | 0.022019 | 0.064636 | | 14.9173 | 14.8517 | 14.9076 | 15.0701 | 15.2761 | 14.3789 | 14.7563 | 14.914 | 5 | 9 | 7 | 7 | 5 | 6 | 8 | 5 |
| ARID1B | Q8NFD5;Q8 | 5.75E-07 | 1.72E-07 | 0.013122 | | 0.065263 | 0.183469 | | 14.7597 | 14.8506 | 14.8192 | 14.9937 | 14.7417 | 14.6983 | 14.7952 | 14.7547 | 10 | 9 | 12 | 8 | 4 | 6 | 3 | 5 |
| NCKAP1 | Q9Y2A7;Q9 | 2.34E-20 | 1.86E-19 | 0.0132086 | | 0.07807 | 0.216907 | | 17.2082 | 17.2208 | 17.2109 | 17.3073 | 17.0402 | 17.2655 | 17.2037 | 17.1837 | 42 | 43 | 44 | 45 | 41 | 45 | 41 | 42 |
| RANBP9 | Q96S59 | 6.91E-17 | 1.71E-16 | 0.0132771 | | 0.064955 | 0.182658 | | 15.7479 | 15.6933 | 15.7272 | 15.598 | 15.8874 | 15.6041 | 15.7603 | 15.6979 | 10 | 11 | 12 | 10 | 8 | 10 | 10 | 9 |
| DES | P17661 | 4.28E-11 | 2.20E-11 | 0.0133497 | | 0.00979 | 0.029124 | | 16.4089 | 16.4975 | 16.5685 | 16.7326 | 17.2022 | 15.6303 | 15.6938 | 17.1324 | 15 | 16 | 18 | 5 | 4 | 9 | 7 | 21 |
| RUFY2 | Q8WXA3;Q | 8.04E-05 | 1.92E-05 | 0.0133686 | | 0.046598 | 0.133372 | | 15.0719 | 15.0265 | 15.0215 | 15.0365 | 15.0261 | 15.1275 | 15.1895 | 14.7533 | 9 | 11 | 9 | 12 | 9 | 12 | 13 | 9 |
| PEX5 | P50542;P5 | 7.67E-10 | 3.22E-10 | 0.013384 | | 0.029092 | 0.084762 | | 15.3113 | 15.3261 | 15.4134 | 15.4187 | 15.6037 | 15.5378 | 15.1125 | 15.0117 | 10 | 10 | 11 | 10 | 4 | 8 | 9 | 6 |
| INPP5A | Q14642 | 1.37E-18 | 5.77E-18 | 0.0134812 | | 0.040068 | 0.115433 | | 15.1022 | 15.1578 | 15.0622 | 15.3366 | 14.8285 | 15.1653 | 14.9883 | 15.151 | 6 | 6 | 7 | 6 | 2 | 6 | 6 | 7 |
| MYO6 | Q9UM54-6 | 1.54E-16 | 3.44E-16 | 0.0136224 | | 0.122272 | 0.327215 | | 16.4474 | 16.4875 | 16.4468 | 16.4427 | 16.419 | 16.458 | 16.5504 | 16.3646 | 52 | 47 | 49 | 48 | 45 | 45 | 48 | 47 |
| RDH14 | Q9HBH5 | 1.45E-11 | 8.16E-12 | 0.0136378 | | 0.088489 | 0.243589 | | 16.3709 | 16.3157 | 16.3394 | 16.2425 | 16.4828 | 16.3096 | 16.3245 | 16.2823 | 13 | 12 | 12 | 11 | 9 | 12 | 11 | 11 |
| GMPPB | Q9Y5P6;Q9 | 1.97E-07 | 6.19E-08 | 0.0136831 | | 0.092052 | 0.252612 | | 18.2346 | 18.2465 | 18.1898 | 18.2556 | 18.0718 | 18.2982 | 18.1784 | 18.2457 | 11 | 11 | 11 | 10 | 10 | 10 | 10 | 10 |
| BZW2 | Q9Y6E2 | 3.87E-08 | 1.31E-08 | 0.0138381 | | 0.048811 | 0.139402 | | 17.7879 | 17.8096 | 17.7742 | 17.9416 | 17.7577 | 17.6111 | 17.6207 | 17.9525 | 19 | 19 | 19 | 19 | 18 | 18 | 19 | 20 |
| PITHD1 | Q9GZP4;Q9 | 3.92E-07 | 1.20E-07 | 0.0140677 | | 0.123717 | 0.330701 | | 16.5486 | 16.5391 | 16.5386 | 16.5789 | 16.5733 | 16.4565 | 16.4442 | 16.5872 | 11 | 12 | 10 | 11 | 11 | 11 | 11 | 11 |
| ASCC2 | Q9H1I8 | 3.91E-08 | 1.32E-08 | 0.0141811 | | 0.073967 | 0.206274 | | 16.2897 | 16.3003 | 16.3459 | 16.3748 | 16.1285 | 16.2547 | 16.4193 | 16.3117 | 15 | 15 | 15 | 14 | 9 | 13 | 12 | 13 |
| FAM120C | Q9NX05 | 4.10E-18 | 1.50E-17 | 0.0141971 | | 0.042058 | 0.120923 | | 15.2791 | 15.2859 | 15.2173 | 15.1371 | 15.4493 | 15.2365 | 15.4235 | 14.9864 | 8 | 9 | 7 | 6 | 6 | 7 | 8 | 8 |
| MTX2 | O75431;O7 | 3.40E-08 | 1.16E-08 | 0.0142564 | | 0.10164 | 0.276646 | | 15.782 | 15.7415 | 15.8897 | 15.8424 | 15.7815 | 15.7949 | 15.85 | 15.6819 | 8 | 9 | 9 | 8 | 8 | 8 | 9 | 9 |
| SUCLA2 | Q9P2R7;Q9 | 1.16E-05 | 3.02E-06 | 0.014444 | | 0.441337 | 0.986547 | | 17.3184 | 17.298 | 17.3044 | 17.2967 | 17.3039 | 17.2877 | 17.3185 | 17.2558 | 14 | 15 | 16 | 17 | 14 | 15 | 15 | 14 |
| TRAPPC12 | Q8WVT3 | 1.71E-17 | 5.05E-17 | 0.0146287 | | 0.057881 | 0.163858 | | 16.1499 | 16.2148 | 16.1834 | 16.0423 | 16.1078 | 16.331 | 16.3234 | 16.0359 | 11 | 10 | 10 | 11 | 10 | 11 | 11 | 11 |
| DCTN1 | Q14203;Q1 | 1.38E-14 | 1.68E-14 | 0.0149731 | | 0.138786 | 0.366647 | | 17.4804 | 17.4553 | 17.4563 | 17.5262 | 17.4748 | 17.3857 | 17.3703 | 17.488 | 63 | 63 | 63 | 65 | 59 | 62 | 63 | 62 |
| KCNK2 | O95069;O9 | 1.55E-06 | 4.42E-07 | 0.0149813 | | 0.01904 | 0.056071 | | 14.2798 | 14.2874 | 14.4416 | 14.8037 | 14.2309 | 14.6976 | 13.704 | 14.1702 | 4 | 4 | 4 | 4 | 3 | 4 | 2 | 3 |
| LARP7 | Q4G0J3;Q4 | 9.35E-07 | 2.73E-07 | 0.0150263 | | 0.078414 | 0.217794 | | 15.4009 | 15.4424 | 15.5224 | 15.3749 | 15.5739 | 15.4367 | 15.5118 | 15.3037 | 8 | 7 | 9 | 8 | 5 | 7 | 6 | 6 |
| OGA | O60502;O6 | 2.02E-11 | 1.11E-11 | 0.015071 | | 0.046389 | 0.132801 | | 16.3791 | 16.3243 | 16.2773 | 16.1729 | 16.4154 | 16.5262 | 16.3766 | 16.0679 | 21 | 21 | 21 | 21 | 17 | 21 | 19 | 19 |
| CMTM3 | Q96MX0;Q | 0.009076 | 0.001566 | 0.0151159 | | 0.027223 | 0.079474 | | 18.4109 | 18.5686 | 18.4441 | 18.001 | 18.8178 | 18.3099 | 18.5609 | 18.6076 | 2 | 2 | 2 | 1 | 2 | 1 | 1 | 2 |
| PSMA1 | P25786;P2 | 6.03E-09 | 2.26E-09 | 0.0151319 | | 0.083009 | 0.229611 | | 18.9073 | 18.918 | 18.8452 | 18.7554 | 18.8248 | 19.0243 | 18.942 | 18.8286 | 17 | 17 | 17 | 16 | 17 | 17 | 17 | 17 |
| EIF2B3 | Q9NR50 | 1.53E-09 | 6.17E-10 | 0.0152275 | | 0.06817 | 0.191123 | | 16.7622 | 16.8082 | 16.8008 | 16.7789 | 16.5657 | 16.9039 | 16.8718 | 16.7553 | 18 | 19 | 16 | 18 | 17 | 17 | 16 | 16 |
| REEP3 | Q6NUK4;Q | 5.96E-11 | 2.99E-11 | 0.015448 | | 0.05369 | 0.152608 | | 15.5163 | 15.8004 | 15.5781 | 15.7652 | 15.6491 | 15.6794 | 15.5749 | 15.4122 | 5 | 5 | 5 | 4 | 4 | 5 | 5 | 3 |
| CPS1 | P31327;P3 | 4.63E-08 | 1.55E-08 | 0.0156658 | | 0.093625 | 0.256581 | | 16.3067 | 16.4071 | 16.153 | 16.251 | 16.2264 | 16.3231 | 16.2393 | 16.3266 | 2 | 2 | 3 | 2 | 5 | 4 | 3 | 4 |
| AGO1 | Q9UL18 | 1.71E-14 | 2.03E-14 | 0.0159234 | | 0.0478 | 0.136651 | | 15.8693 | 15.761 | 15.7891 | 15.5842 | 15.8517 | 15.9658 | 15.9612 | 15.5899 | 20 | 19 | 20 | 15 | 11 | 16 | 17 | 15 |
| IDH3B | O43837 | 8.75E-12 | 5.14E-12 | 0.0159568 | | 0.121949 | 0.326435 | | 16.2028 | 16.2843 | 16.2341 | 16.3348 | 16.1974 | 16.206 | 16.1281 | 16.2557 | 17 | 15 | 16 | 14 | 14 | 15 | 15 | 14 |
| SRSF9 | Q13242 | 1.01E-13 | 9.68E-14 | 0.0159822 | | 0.074345 | 0.207255 | | 17.5388 | 17.4943 | 17.4426 | 17.5383 | 17.5525 | 17.2565 | 17.492 | 17.5402 | 13 | 13 | 12 | 12 | 13 | 13 | 13 | 13 |
| MARS1 | P56192 | 3.11E-14 | 3.40E-14 | 0.0160695 | | 0.08665 | 0.238913 | | 18.01 | 18.0681 | 18.0047 | 17.9147 | 18.0153 | 18.1514 | 18.0839 | 17.8923 | 31 | 30 | 30 | 30 | 29 | 32 | 32 | 31 |
| USP47 | Q96K76 | 7.69E-17 | 1.87E-16 | 0.0160968 | | 0.16701 | 0.432119 | | 16.3486 | 16.3488 | 16.3617 | 16.2948 | 16.3713 | 16.2518 | 16.3613 | 16.4056 | 38 | 37 | 37 | 34 | 31 | 31 | 40 | 31 |
| UQCRB | P14927 | 6.13E-06 | 1.64E-06 | 0.0162663 | | 0.012795 | 0.037937 | | 16.9113 | 16.5502 | 17.1599 | 17.1029 | 16.7061 | 15.733 | 17.4015 | 17.3441 | 8 | 8 | 8 | 7 | 8 | 6 | 8 | 8 |
| TBC1D22A | Q8WUA7;Q | 0.001502 | 0.000302 | 0.0164453 | | 0.043509 | 0.124915 | | 15.0185 | 15.0264 | 15.1275 | 14.719 | 15.0285 | 15.3168 | 15.0157 | 15.1252 | 6 | 7 | 7 | 5 | 5 | 5 | 6 | 5 |
| IFT27 | Q9BW83 | 5.57E-08 | 1.85E-08 | 0.0165854 | | 0.026914 | 0.078597 | | 16.1933 | 16.0534 | 16.0819 | 16.0513 | 15.5271 | 16.4395 | 16.3068 | 16.14 | 5 | 6 | 5 | 6 | 6 | 6 | 6 | 6 |
| RBMS1 | P29558;P2 | 8.65E-19 | 3.83E-18 | 0.0166126 | | 0.072801 | 0.203238 | | 17.5817 | 17.5369 | 17.3909 | 17.6322 | 17.4357 | 17.4094 | 17.5904 | 17.3652 | 5 | 5 | 4 | 4 | 5 | 5 | 5 | 5 |
| PPP1CA | P62136 | 1.97E-08 | 6.91E-09 | 0.0167119 | | 0.126615 | 0.337671 | | 19.7028 | 19.7167 | 19.7239 | 19.6262 | 19.8028 | 19.6064 | 19.7028 | 19.7506 | 17 | 16 | 17 | 17 | 17 | 17 | 17 | 17 |
| RPL18A | Q02543 | 5.56E-07 | 1.66E-07 | 0.0168138 | | 0.124082 | 0.331582 | | 20.6917 | 20.7583 | 20.763 | 20.6801 | 20.8627 | 20.704 | 20.6704 | 20.6871 | 10 | 9 | 9 | 9 | 9 | 9 | 9 | 9 |
| NSA2 | O95478 | 3.03E-21 | 3.15E-20 | 0.0171673 | | 0.054565 | 0.154964 | | 15.3608 | 15.5313 | 15.6327 | 15.567 | 15.3961 | 15.26 | 15.6365 | 15.5958 | 10 | 9 | 10 | 11 | 8 | 9 | 9 | 8 |
| EIF4E2 | O60573 | 5.61E-06 | 1.51E-06 | 0.0172247 | | 0.066125 | 0.185742 | | 15.6967 | 15.6287 | 15.7813 | 15.9332 | 15.6032 | 15.5633 | 15.6846 | 15.6407 | 8 | 9 | 8 | 9 | 7 | 8 | 8 | 8 |
| FIP1L1 | Q6UN15 | 5.68E-06 | 1.53E-06 | 0.0173766 | | 0.076449 | 0.212714 | | 16.7753 | 16.7237 | 16.7534 | 16.9233 | 16.7249 | 16.5874 | 16.6273 | 16.8043 | 9 | 9 | 9 | 9 | 8 | 9 | 8 | 8 |
| SPRYD4 | Q8WW59 | 7.37E-08 | 2.41E-08 | 0.0174218 | | 0.075189 | 0.209447 | | 15.7607 | 15.7649 | 15.8034 | 15.6174 | 15.6099 | 15.8151 | 15.8321 | 15.9201 | 8 | 9 | 8 | 9 | 6 | 9 | 8 | 8 |
| ANXA6 | P08133 | 1.70E-12 | 1.16E-12 | 0.0174526 | | 0.049877 | 0.142298 | | 21.1732 | 21.2104 | 21.1779 | 21.4507 | 21.2534 | 21.082 | 20.8963 | 21.1661 | 53 | 53 | 53 | 52 | 54 | 53 | 53 | 56 |
| MRPS34 | P82930 | 6.86E-14 | 6.85E-14 | 0.0175659 | | 0.099079 | 0.270261 | | 16.7952 | 16.8716 | 16.8373 | 16.904 | 16.8856 | 16.8905 | 16.7232 | 16.6823 | 9 | 9 | 9 | 9 | 8 | 9 | 9 | 9 |
| ECI1 | P42126;P4 | 9.44E-05 | 2.24E-05 | 0.0176334 | | 0.079446 | 0.220456 | | 17.5515 | 17.4123 | 17.5255 | 17.5158 | 17.3951 | 17.6228 | 17.5466 | 17.3136 | 7 | 7 | 7 | 7 | 7 | 6 | 7 | 7 |
| GFM1 | Q96RP9 | 1.24E-10 | 5.88E-11 | 0.0176445 | | 0.155451 | 0.405583 | | 16.3595 | 16.3124 | 16.3456 | 16.4334 | 16.2583 | 16.3484 | 16.2804 | 16.2871 | 25 | 27 | 27 | 25 | 22 | 26 | 26 | 25 |
| PDK1 | Q15118 | 0.011624 | 0.001978 | 0.0177251 | | 0.03968 | 0.114359 | | 14.8392 | 15.229 | 14.7901 | 15.1445 | 15.1291 | 14.9088 | 14.7218 | 14.7711 | 3 | 3 | 2 | 6 | 2 | 3 | 3 | 3 |
| SYNJ2BP | P57105 | 6.28E-05 | 1.52E-05 | 0.0177494 | | 0.042833 | 0.123056 | | 16.5061 | 16.4585 | 16.5287 | 16.3039 | 16.6527 | 16.1597 | 16.7303 | 16.5536 | 5 | 4 | 4 | 5 | 4 | 4 | 5 | 5 |
| MAP3K4 | Q9Y6R4;Q9 | 0.000125 | 2.92E-05 | 0.0177994 | | 0.042723 | 0.122752 | | 14.1383 | 14.168 | 14.1786 | 13.8871 | 13.9557 | 14.5031 | 14.215 | 14.1583 | 12 | 6 | 8 | 6 | 6 | 8 | 6 | 6 |
| FBXL18 | Q96ME1-4 | 3.37E-08 | 1.15E-08 | 0.0178427 | | 0.129627 | 0.344888 | | 15.854 | 15.8368 | 15.7944 | 15.8926 | 15.8851 | 15.8219 | 15.7554 | 15.6975 | 12 | 14 | 13 | 14 | 10 | 13 | 11 | 11 |
| ZC3HAV1L | Q96H79 | 0.000298 | 6.61E-05 | 0.0178475 | | 0.025916 | 0.07576 | | 13.9855 | 14.0122 | 14.0083 | 13.4571 | 13.8534 | 14.3709 | 14.3869 | 13.8526 | 5 | 5 | 5 | 4 | 4 | 5 | 5 | 5 |
| GLMN | Q92990 | 3.71E-06 | 1.02E-06 | 0.018003 | | 0.199967 | 0.505849 | | 15.8596 | 15.8114 | 15.8611 | 15.8904 | 15.8304 | 15.7505 | 15.8665 | 15.7922 | 7 | 7 | 6 | 8 | 5 | 6 | 4 | 8 |
| PYGB | P11216 | 1.54E-15 | 2.50E-15 | 0.0180144 | | 0.048365 | 0.138188 | | 18.2555 | 18.2373 | 18.2378 | 17.908 | 18.3485 | 18.4261 | 18.3544 | 18.0906 | 35 | 35 | 36 | 34 | 35 | 36 | 35 | 36 |
| PAIP1 | Q9H074 | 3.95E-11 | 2.04E-11 | 0.0182027 | | 0.106933 | 0.289766 | | 17.5183 | 17.5027 | 17.478 | 17.6121 | 17.4612 | 17.5626 | 17.4096 | 17.3618 | 11 | 12 | 12 | 11 | 9 | 11 | 11 | 10 |
| TIGAR | Q9NQ88 | 1.81E-08 | 6.37E-09 | 0.0182645 | | 0.153873 | 0.401931 | | 17.2768 | 17.251 | 17.1101 | 17.215 | 17.2153 | 17.2345 | 17.1791 | 17.1279 | 11 | 11 | 10 | 10 | 11 | 10 | 12 | 10 |
| AP2B1 | P63010;P6 | 1.22E-13 | 1.13E-13 | 0.0186436 | | 0.086086 | 0.237474 | | 18.6504 | 18.7172 | 18.6774 | 18.7547 | 18.4621 | 18.7725 | 18.7191 | 18.6066 | 26 | 25 | 25 | 25 | 25 | 25 | 25 | 25 |
| GOLGA1 | Q92805 | 7.32E-11 | 3.59E-11 | 0.0187251 | | 0.210871 | 0.529668 | | 15.183 | 15.1101 | 15.1462 | 15.1331 | 15.0811 | 15.1896 | 15.1682 | 15.0667 | 19 | 20 | 20 | 19 | 13 | 16 | 17 | 19 |
| ERO1A | Q96HE7 | 3.02E-08 | 1.04E-08 | 0.0187522 | | 0.086232 | 0.237849 | | 18.1981 | 18.1804 | 18.1671 | 18.3436 | 17.9912 | 18.1215 | 18.1296 | 18.2297 | 19 | 18 | 18 | 18 | 18 | 18 | 18 | 18 |
| CPSF7 | Q8N684;Q8 | 4.11E-09 | 1.57E-09 | 0.0187784 | | 0.114595 | 0.308576 | | 16.2795 | 16.3742 | 16.3752 | 16.2219 | 16.4649 | 16.2561 | 16.3267 | 16.3512 | 14 | 14 | 13 | 12 | 10 | 12 | 14 | 11 |
| ARMH3 | Q5T2E6 | 4.40E-14 | 4.62E-14 | 0.0192171 | | 0.093969 | 0.257448 | | 14.956 | 15.0663 | 15.005 | 15.0135 | 15.1429 | 15.0503 | 14.8924 | 14.8504 | 14 | 12 | 13 | 12 | 6 | 11 | 10 | 10 |
| PAK4 | O96013;O9 | 6.20E-06 | 1.66E-06 | 0.0194401 | | 0.06107 | 0.172362 | | 15.4264 | 15.5126 | 15.4362 | 15.6529 | 15.5354 | 15.29 | 15.2027 | 15.5138 | 10 | 9 | 8 | 10 | 9 | 8 | 7 | 9 |
| MRPS27 | Q92552;Q9 | 2.90E-12 | 1.87E-12 | 0.0196294 | | 0.163754 | 0.424681 | | 16.4753 | 16.5119 | 16.5559 | 16.6104 | 16.4766 | 16.4668 | 16.504 | 16.416 | 14 | 13 | 14 | 16 | 14 | 13 | 14 | 16 |
| MTMR1 | Q13613 | 1.49E-15 | 2.43E-15 | 0.0197275 | | 0.0929 | 0.254752 | | 14.6679 | 14.7192 | 14.7271 | 14.5908 | 14.7989 | 14.777 | 14.7492 | 14.5091 | 10 | 9 | 11 | 9 | 9 | 9 | 9 | 8 |
| ARID2 | Q68CP9;Q6 | 1.87E-05 | 4.78E-06 | 0.0197777 | | 0.132088 | 0.350761 | | 14.4112 | 14.4436 | 14.2604 | 14.3487 | 14.3221 | 14.2622 | 14.4205 | 14.4064 | 5 | 3 | 4 | 3 | 2 | 2 | 2 | 3 |
| FAM98A | Q8NCA5 | 5.97E-08 | 1.98E-08 | 0.0199099 | | 0.132252 | 0.351151 | | 18.2118 | 18.1932 | 18.154 | 18.0816 | 18.2389 | 18.2647 | 18.1891 | 18.0578 | 12 | 12 | 12 | 11 | 11 | 11 | 11 | 11 |

| Gene names | UniProt IDs | Protein Group P-value | Protein Group Q-value | Log2 Difference NUP214 vs Controls (n=5) | p<0.05 | -log10 p-value | T-test Test statistic | NPC Localization | Log2 MS2_quantity_NUP214_1 | Log2 MS2_quantity_NUP214_2 | Log2 MS2_quantity_NUP214_3 | Log2 MS2_quantity_Control_1 | Log2 MS2_quantity_Control_2 | Log2 MS2_quantity_Control_3 | Log2 MS2_quantity_Control_4 | Log2 MS2_quantity_Control_5 | Number Peptides for Quantitation NUP214_1 | Number Peptides for Quantitation NUP214_2 | Number Peptides for Quantitation NUP214_3 | Number Peptides for Quantitation Control_1 | Number Peptides for Quantitation Control_2 | Number Peptides for Quantitation Control_3 | Number Peptides for Quantitation Control_4 | Number Peptides for Quantitation Control_5 |
|---|---|---|---|---|---|---|---|---|---|---|---|---|---|---|---|---|---|---|---|---|---|---|---|---|
| MT-CO1 | P00395 | 1.13E-08 | 4.08E-09 | 0.019912 | | 0.052423 | 0.14919 | | 16.9549 | 17.0026 | 17.1721 | 17.0562 | 16.6895 | 17.2672 | 17.071 | 17.0326 | 3 | 3 | 3 | 3 | 3 | 3 | 3 | 3 |
| ABCB7 | O75027;O | 4.94E-06 | 1.33E-06 | 0.0199343 | | 0.062059 | 0.174988 | | 15.0789 | 15.1813 | 15.1444 | 14.9588 | 15.1874 | 14.9011 | 15.161 | 15.3662 | 7 | 9 | 7 | 9 | 7 | 6 | 7 | 6 |
| BOD1L1 | Q8NFC6 | 1.35E-06 | 3.88E-07 | 0.0200742 | | 0.038809 | 0.111949 | | 14.1963 | 14.2709 | 14.1457 | 13.8766 | 14.656 | 14.2579 | 14.1032 | 14.0275 | 13 | 10 | 14 | 10 | 4 | 8 | 10 | 10 |
| UBA1 | P22314;P2 | 2.32E-13 | 2.00E-13 | 0.020208 | | 0.060923 | 0.171971 | | 20.0301 | 20.0552 | 19.9753 | 19.7613 | 20.2442 | 20.0818 | 19.8448 | 20.0678 | 50 | 50 | 50 | 47 | 47 | 49 | 48 | 49 |
| HDDC2 | Q7Z4H3 | 2.21E-12 | 1.47E-12 | 0.0203503 | | 0.056033 | 0.158908 | | 15.7507 | 15.399 | 15.9138 | 15.6471 | 15.6753 | 15.5077 | 15.8049 | 15.7024 | 6 | 4 | 5 | 3 | 3 | 4 | 4 | 4 |
| GMPS | P49915 | 2.69E-13 | 2.27E-13 | 0.020402 | | 0.10993 | 0.297147 | | 16.5897 | 16.6651 | 16.6022 | 16.7136 | 16.5339 | 16.4373 | 16.6638 | 16.6443 | 30 | 31 | 30 | 30 | 28 | 27 | 29 | 29 |
| TOMM70 | O94826 | 4.39E-12 | 2.72E-12 | 0.0206184 | | 0.220395 | 0.550256 | | 16.7084 | 16.7187 | 16.7058 | 16.6908 | 16.6139 | 16.7829 | 16.6582 | 16.706 | 23 | 23 | 22 | 23 | 22 | 23 | 22 | 22 |
| GYS1 | P13807 | 1.35E-13 | 1.24E-13 | 0.0211334 | | 0.081879 | 0.226714 | | 16.5654 | 16.5568 | 16.5612 | 16.7876 | 16.5414 | 16.3984 | 16.4135 | 16.5591 | 21 | 21 | 20 | 23 | 20 | 22 | 22 | 22 |
| SPIN1 | Q9Y657 | 4.20E-09 | 1.60E-09 | 0.0212257 | | 0.059633 | 0.168536 | | 15.4651 | 15.2861 | 15.3382 | 15.356 | 15.3167 | 15.1359 | 15.668 | 15.233 | 7 | 7 | 9 | 6 | 6 | 6 | 6 | 5 |
| PANK4 | Q9NVE7 | 3.50E-08 | 1.19E-08 | 0.0213556 | | 0.18184 | 0.465634 | | 15.3262 | 15.4588 | 15.4335 | 15.4083 | 15.3613 | 15.4173 | 15.443 | 15.2942 | 24 | 24 | 24 | 24 | 19 | 23 | 21 | 20 |
| NDST1 | P52848 | 5.32E-11 | 2.69E-11 | 0.0215473 | | 0.046703 | 0.133659 | | 15.3125 | 15.412 | 15.433 | 15.1427 | 15.4092 | 15.799 | 15.3086 | 15.1621 | 18 | 18 | 19 | 12 | 15 | 19 | 18 | 17 |
| HSD17B4 | P51659 | 1.27E-13 | 1.17E-13 | 0.0216049 | | 0.197821 | 0.50113 | | 18.4908 | 18.528 | 18.4616 | 18.3969 | 18.5727 | 18.4924 | 18.4752 | 18.4222 | 34 | 36 | 34 | 31 | 33 | 30 | 33 | 34 |
| UGGT1 | Q9NYU2;Q | 1.18E-12 | 8.41E-13 | 0.0216543 | | 0.095786 | 0.262014 | | 17.9345 | 17.9543 | 17.9373 | 17.7904 | 18.0372 | 18.0956 | 17.8724 | 17.8062 | 57 | 60 | 61 | 57 | 57 | 59 | 57 | 57 |
| ABCF1 | Q8NE71 | 2.56E-24 | 7.58E-23 | 0.0218419 | | 0.197752 | 0.500977 | | 17.0225 | 17.0864 | 17.0324 | 17.0487 | 17.0589 | 17.0748 | 17.0399 | 16.9041 | 29 | 30 | 31 | 31 | 30 | 31 | 34 | 29 |
| ACTR3 | P61158 | 2.01E-15 | 3.14E-15 | 0.0219093 | | 0.27148 | 0.657581 | | 20.6364 | 20.6426 | 20.5813 | 20.5796 | 20.6766 | 20.6168 | 20.547 | 20.5709 | 27 | 27 | 26 | 26 | 26 | 26 | 27 | 26 |
| MRPL46 | Q9H2W6 | 3.07E-11 | 1.62E-11 | 0.0219322 | | 0.101309 | 0.275824 | | 15.9847 | 16.1249 | 16.11 | 15.9283 | 16.1516 | 15.9668 | 16.0016 | 16.2078 | 6 | 7 | 7 | 7 | 6 | 7 | 4 | 6 |
| ARL6IP5 | O75915 | 0.002958 | 0.000552 | 0.0219421 | | 0.053914 | 0.15321 | | 20.0496 | 19.9073 | 20.0763 | 20.354 | 20.0202 | 19.867 | 19.7345 | 19.9699 | 5 | 5 | 4 | 5 | 5 | 4 | 5 | 5 |
| GPALPP1 | Q8IXQ4 | 9.78E-05 | 2.32E-05 | 0.0221078 | | 0.203241 | 0.513029 | | 15.1643 | 15.0805 | 15.0514 | 15.0711 | 15.1652 | 15.0468 | 15.0062 | 15.0937 | 5 | 6 | 7 | 6 | 6 | 5 | 5 | 7 |
| SMC4 | Q9NTJ3 | 1.20E-12 | 8.49E-13 | 0.0221093 | | 0.043605 | 0.125178 | | 15.4081 | 15.4441 | 15.4482 | 15.7595 | 15.3762 | 14.956 | 15.4116 | 15.5536 | 40 | 39 | 38 | 40 | 31 | 29 | 37 | 39 |
| CNOT11 | Q9UKZ1 | 2.46E-08 | 8.53E-09 | 0.0221312 | | 0.202939 | 0.512368 | | 16.0654 | 16.0262 | 15.9998 | 15.9297 | 16.0579 | 15.9865 | 15.9689 | 16.0986 | 5 | 5 | 5 | 5 | 5 | 5 | 4 | 5 |
| SETDB1 | Q15047;Q1 | 0.004286 | 0.000774 | 0.0223545 | | 0.011878 | 0.035255 | | 12.38 | 13.0989 | 12.5528 | 12.9494 | 10.8585 | 12.8256 | 13.2407 | 13.4002 | 1 | 1 | 3 | 2 | 1 | 2 | 2 | 1 |
| KPNA6 | O60684 | 3.16E-16 | 6.18E-16 | 0.0224728 | | 0.12206 | 0.326704 | | 17.6438 | 17.642 | 17.5233 | 17.6213 | 17.6328 | 17.5049 | 17.4427 | 17.7012 | 8 | 7 | 7 | 8 | 7 | 7 | 8 | 7 |
| PREPL | Q4J6C6;Q4 | 4.39E-12 | 2.72E-12 | 0.0224989 | | 0.098224 | 0.268124 | | 15.0301 | 15.1169 | 15.168 | 15.0957 | 14.9485 | 14.9744 | 15.2789 | 15.1152 | 16 | 14 | 14 | 16 | 9 | 14 | 14 | 15 |
| CAP1 | Q01518 | 2.35E-13 | 2.02E-13 | 0.0226069 | | 0.218113 | 0.545341 | | 19.8816 | 19.8849 | 19.8702 | 19.9243 | 19.916 | 19.8621 | 19.7574 | 19.8215 | 35 | 34 | 34 | 35 | 35 | 35 | 33 | 35 |
| MED23 | Q9ULK4;Q | 1.54E-15 | 2.51E-15 | 0.0227695 | | 0.115229 | 0.310122 | | 14.9097 | 14.815 | 14.8725 | 14.8652 | 14.9192 | 14.6363 | 14.9216 | 14.8726 | 17 | 15 | 17 | 14 | 12 | 14 | 17 | 17 |
| PTBP1 | P26599;P2 | 3.24E-07 | 9.97E-08 | 0.0228218 | | 0.080814 | 0.223978 | | 20.1038 | 20.1126 | 20.0251 | 19.9189 | 20.3346 | 19.9569 | 19.9873 | 20.0908 | 15 | 15 | 15 | 15 | 15 | 15 | 15 | 15 |
| IDI1 | Q13907 | 2.04E-07 | 6.42E-08 | 0.0229856 | | 0.028857 | 0.084098 | | 16.8106 | 16.6585 | 16.8548 | 17.2588 | 16.8891 | 16.1903 | 16.375 | 17.0452 | 12 | 12 | 13 | 12 | 11 | 12 | 11 | 12 |
| PSMC6 | P62333 | 1.22E-21 | 1.39E-20 | 0.0230787 | | 0.139152 | 0.367512 | | 18.8885 | 18.876 | 18.8046 | 18.7991 | 18.8985 | 18.7986 | 18.7306 | 18.8397 | 22 | 22 | 22 | 23 | 21 | 21 | 22 | 22 |
| CNN2 | Q99439 | 1.14E-16 | 2.66E-16 | 0.0233301 | | 0.032998 | 0.095753 | | 19.8114 | 19.7494 | 19.7246 | 19.8228 | 19.8638 | 20.2797 | 19.1811 | 19.5451 | 14 | 14 | 14 | 14 | 14 | 14 | 14 | 14 |
| GPKOW | Q92917 | 0.000144 | 3.33E-05 | 0.0235984 | | 0.090407 | 0.248453 | | 15.3035 | 15.3354 | 15.3983 | 15.4307 | 15.1587 | 15.1732 | 15.3361 | 15.5118 | 10 | 11 | 11 | 11 | 8 | 11 | 10 | 12 |
| MAPKAPK2 | P49137 | 4.52E-09 | 1.72E-09 | 0.0236262 | | 0.111314 | 0.300545 | | 15.5562 | 15.55 | 15.578 | 15.6191 | 15.7189 | 15.4424 | 15.3967 | 15.5119 | 14 | 14 | 14 | 14 | 11 | 12 | 11 | 14 |
| SAP30BP | Q9UHR5;Q | 1.15E-09 | 4.71E-10 | 0.0237436 | | 0.052125 | 0.148384 | | 15.1147 | 15.142 | 15.1397 | 15.5695 | 15.0146 | 14.9469 | 14.9083 | 15.1026 | 7 | 5 | 7 | 5 | 2 | 5 | 4 | 4 |
| TMX4 | Q9H1E5 | 5.91E-08 | 1.96E-08 | 0.0237685 | | 0.074651 | 0.20805 | | 17.3307 | 17.2293 | 17.2422 | 16.9269 | 17.3807 | 17.2879 | 17.3838 | 17.2389 | 4 | 4 | 4 | 4 | 3 | 4 | 4 | 3 |
| DNAJC13 | O75165 | 2.05E-13 | 1.79E-13 | 0.0238977 | | 0.15472 | 0.403892 | | 16.5903 | 16.5805 | 16.5676 | 16.5927 | 16.5158 | 16.5925 | 16.6692 | 16.4077 | 84 | 83 | 85 | 82 | 77 | 85 | 86 | 82 |
| RPL13 | P26373 | 4.48E-15 | 6.30E-15 | 0.0239306 | | 0.038811 | 0.111954 | | 20.7938 | 20.7373 | 20.5637 | 20.6012 | 21.2131 | 20.5237 | 20.2747 | 20.759 | 8 | 8 | 8 | 8 | 8 | 8 | 6 | 8 |
| IRF3 | Q14653 | 0.003111 | 0.000577 | 0.0239808 | | 0.090566 | 0.248855 | | 15.8658 | 15.6458 | 15.7469 | 15.7293 | 15.9233 | 15.5714 | 15.6182 | 15.8021 | 5 | 6 | 5 | 4 | 4 | 4 | 4 | 4 |
| DNAJC7 | Q99615 | 1.57E-14 | 1.88E-14 | 0.024042 | | 0.238169 | 0.588168 | | 16.2643 | 16.2601 | 16.2256 | 16.3242 | 16.1712 | 16.1722 | 16.1971 | 16.265 | 28 | 26 | 26 | 26 | 22 | 23 | 25 | 25 |
| GLS | O94925-3 | 8.60E-20 | 5.40E-19 | 0.0240609 | | 0.089493 | 0.246138 | | 18.4634 | 18.4343 | 18.424 | 18.3647 | 18.1807 | 18.605 | 18.5291 | 18.4031 | 3 | 3 | 3 | 3 | 3 | 3 | 3 | 3 |
| RNF167 | Q9H6Y7 | 0.000626 | 0.000133 | 0.0242559 | | 0.021465 | 0.063048 | | 14.4295 | 14.2703 | 14.1341 | 14.36 | 13.1991 | 14.3469 | 14.9299 | 14.4327 | 2 | 2 | 2 | 3 | 1 | 2 | 2 | 3 |
| STOML2 | Q9UJZ1 | 7.49E-17 | 1.84E-16 | 0.0243574 | | 0.112951 | 0.304558 | | 17.7286 | 17.5802 | 17.8516 | 17.5845 | 17.6987 | 17.6274 | 17.8184 | 17.75 | 16 | 16 | 16 | 16 | 13 | 16 | 15 | 14 |
| CZIB | Q9NWV4 | 0.000174 | 4.00E-05 | 0.0243989 | | 0.087566 | 0.241243 | | 17.978 | 18.0232 | 17.9658 | 17.7926 | 17.9209 | 18.222 | 18.0292 | 17.8582 | 5 | 5 | 6 | 5 | 5 | 6 | 5 | 5 |
| TPM1 | P09493-4 | 4.48E-13 | 3.56E-13 | 0.0245941 | | 0.033454 | 0.097031 | | 20.9011 | 20.9182 | 20.8684 | 20.3295 | 20.6406 | 21.4699 | 20.9838 | 20.9327 | 44 | 44 | 45 | 44 | 40 | 45 | 44 | 43 |
| EIF3K | Q9UBQ5;Q | 2.96E-09 | 1.15E-09 | 0.0248089 | | 0.073795 | 0.205825 | | 17.9238 | 17.7459 | 17.949 | 17.7775 | 17.9148 | 17.6058 | 17.8287 | 18.1135 | 8 | 8 | 8 | 8 | 8 | 7 | 8 | 8 |
| CDC37 | Q16543 | 3.55E-15 | 5.16E-15 | 0.0249582 | | 0.062014 | 0.17487 | | 17.5218 | 17.4493 | 17.3997 | 17.6752 | 17.24 | 17.6054 | 17.1321 | 17.5071 | 18 | 18 | 18 | 19 | 20 | 18 | 18 | 19 |
| DUSP12 | Q9UNI6 | 7.89E-09 | 2.91E-09 | 0.0250839 | | 0.074131 | 0.206699 | | 14.7418 | 14.7127 | 14.7599 | 15.0245 | 14.457 | 14.7196 | 14.6861 | 14.6783 | 10 | 9 | 9 | 11 | 9 | 9 | 9 | 11 |
| AHSG | P02765 | 2.27E-18 | 8.95E-18 | 0.0253782 | | 0.054551 | 0.154925 | | 13.7771 | 13.7913 | 13.85 | 14.1203 | 13.4054 | 13.625 | 13.8796 | 13.8736 | 3 | 3 | 3 | 2 | 2 | 2 | 2 | 3 |
| SLC6A6 | P31641 | 3.47E-05 | 8.65E-06 | 0.025379 | | 0.027662 | 0.081385 | | 14.7546 | 14.7137 | 14.8765 | 14.1128 | 14.5551 | NaN | 15.1149 | 15.2421 | 3 | 3 | 3 | 1 | 1 | NaN | 3 | 1 |
| SCRIB | Q14160;Q1 | 6.98E-15 | 9.20E-15 | 0.025503 | | 0.145406 | 0.382213 | | 16.3575 | 16.3595 | 16.3112 | 16.1814 | 16.4802 | 16.2618 | 16.3391 | 16.3235 | 34 | 37 | 38 | 34 | 32 | 36 | 37 | 34 |
| FARSB | Q9NSD9 | 1.11E-12 | 7.93E-13 | 0.0257525 | | 0.277759 | 0.670444 | | 18.1062 | 18.1171 | 18.0923 | 18.0023 | 18.0299 | 18.1351 | 18.1486 | 18.0814 | 27 | 27 | 28 | 27 | 25 | 25 | 26 | 26 |
| UTP23 | Q9BRU9 | 0.00023 | 5.19E-05 | 0.025769 | | 0.076149 | 0.211937 | | 15.2362 | 15.1218 | 15.0958 | 15.3362 | 14.8626 | 15.1548 | 14.9946 | 15.2793 | 2 | 2 | 2 | 2 | 2 | 2 | 1 | 2 |
| G3BP1 | Q13283 | 3.24E-10 | 1.43E-10 | 0.0259686 | | 0.095463 | 0.261204 | | 19.5337 | 19.5177 | 19.4674 | 19.7115 | 19.3627 | 19.4415 | 19.3065 | 19.5794 | 16 | 16 | 16 | 15 | 15 | 17 | 15 | 15 |
| DPH1 | Q9BZG8;Q | 0.000312 | 6.90E-05 | 0.0260478 | | 0.015915 | 0.047027 | | 14.6477 | 14.4862 | 14.5152 | 14.5303 | 12.9671 | 15.0971 | 15.3344 | 14.6893 | 6 | 4 | 5 | 2 | 3 | 4 | 3 | 5 |
| CFAP410 | O43822;O | 7.40E-11 | 3.62E-11 | 0.0263124 | | 0.045244 | 0.12967 | | 15.0914 | 15.1712 | 15.206 | 14.7794 | 15.1836 | 15.511 | 15.3892 | 14.7862 | 2 | 3 | 3 | 3 | 2 | 3 | 2 | 3 |
| RBM12 | Q9NTZ6 | 5.71E-14 | 5.82E-14 | 0.0263707 | | 0.119016 | 0.319334 | | 16.3096 | 16.458 | 16.4335 | 16.5407 | 16.3973 | 16.1892 | 16.3434 | 16.3995 | 18 | 19 | 19 | 19 | 15 | 18 | 19 | 18 |
| ARPC4 | P59998;P5 | 6.11E-06 | 1.64E-06 | 0.0265404 | | 0.081063 | 0.224619 | | 19.5911 | 19.5083 | 19.589 | 19.5995 | 19.545 | 19.2017 | 19.6349 | 19.7001 | 7 | 7 | 7 | 8 | 7 | 8 | 8 | 8 |
| RPL3 | P39023 | 1.04E-10 | 4.97E-11 | 0.0267873 | | 0.267525 | 0.649444 | | 19.2243 | 19.2537 | 19.1909 | 19.1943 | 19.2319 | 19.2669 | 19.0919 | 19.196 | 27 | 26 | 27 | 26 | 26 | 27 | 25 | 26 |
| EMILIN1 | Q9Y6C2 | 4.23E-19 | 2.14E-18 | 0.0268869 | | 0.043644 | 0.125285 | | 15.2547 | 15.1928 | 15.2683 | 15.3808 | 14.9213 | 14.7872 | 15.6753 | 15.294 | 11 | 12 | 13 | 12 | 13 | 11 | 14 | 14 |
| SETD1A | O15047 | 1.85E-07 | 5.84E-08 | 0.0268937 | | 0.11962 | 0.320799 | | 14.5288 | 14.4336 | 14.6068 | 14.5582 | 14.6058 | 14.598 | 14.3457 | 14.3731 | 6 | 5 | 5 | 4 | 6 | 5 | 4 | 4 |
| GLT8D2 | Q9H1C3 | 2.56E-24 | 7.58E-23 | 0.0270516 | | 0.052309 | 0.148881 | | 15.1355 | 15.3423 | 15.2875 | 15.272 | 15.3674 | 15.192 | 15.5512 | 14.7575 | 6 | 6 | 5 | 5 | 5 | 6 | 6 | 4 |
| SDE2 | Q6IQ49;Q6 | 8.29E-16 | 1.45E-15 | 0.0270602 | | 0.102303 | 0.278296 | | 15.3826 | 15.1867 | 15.2285 | 15.3305 | 15.2813 | 15.3651 | 14.9968 | 15.2206 | 1 | 1 | 1 | 1 | 1 | 1 | 1 | 1 |
| UBP1 | Q9NZI7 | 0.000266 | 5.94E-05 | 0.0270876 | | 0.057388 | 0.162538 | | 15.3689 | 15.6056 | 15.481 | 15.4917 | 15.8782 | 15.448 | 15.1718 | 15.3007 | 4 | 5 | 5 | 5 | 3 | 3 | 3 | 3 |
| SEC22B | O75396 | 2.41E-08 | 8.37E-09 | 0.0271324 | | 0.082715 | 0.228858 | | 19.687 | 19.728 | 19.7141 | 19.7659 | 19.5654 | 19.4102 | 19.7477 | 19.9237 | 10 | 10 | 10 | 9 | 10 | 9 | 9 | 10 |
| CYBRD1 | Q53TN4 | 3.85E-06 | 1.05E-06 | 0.0272535 | | 0.062325 | 0.175695 | | 17.0939 | 16.9969 | 17.0452 | 17.0361 | 16.5963 | 17.2964 | 17.1161 | 17.0455 | 6 | 6 | 5 | 4 | 5 | 6 | 5 | 5 |
| AKAP12 | Q02952 | 3.63E-12 | 2.29E-12 | 0.0273117 | | 0.033018 | 0.09581 | | 16.3292 | 16.3122 | 16.459 | 16.5971 | 15.5353 | 16.6939 | 16.584 | 16.2871 | 51 | 55 | 56 | 55 | 41 | 56 | 52 | 55 |
| SRPRA | P08240 | 2.56E-24 | 7.58E-23 | 0.0274549 | | 0.073858 | 0.205991 | | 17.2843 | 17.2888 | 17.2558 | 17.3662 | 16.8959 | 17.4919 | 17.2156 | 17.2746 | 28 | 28 | 28 | 27 | 27 | 30 | 26 | 27 |
| SENP3 | Q9H4L4 | 1.03E-14 | 1.30E-14 | 0.0276621 | | 0.075507 | 0.210273 | | 15.3417 | 15.4713 | 15.316 | 15.5149 | 14.9798 | 15.4105 | 15.378 | 15.4604 | 4 | 7 | 5 | 6 | 3 | 6 | 5 | 6 |
| ESYT2 | A0FGR8-2 | 3.23E-15 | 4.76E-15 | 0.0277785 | | 0.108697 | 0.294116 | | 17.3021 | 17.2694 | 17.3065 | 17.0433 | 17.2772 | 17.46 | 17.3496 | 17.1942 | 29 | 30 | 30 | 29 | 26 | 26 | 28 | 28 |
| RNF121 | Q9H920 | 0.006203 | 0.001093 | 0.0277955 | | 0.011279 | 0.034189 | | 13.6936 | 13.9504 | 13.984 | 13.5388 | NaN | 15.3761 | 12.6296 | NaN | 2 | 2 | 3 | 2 | NaN | 3 | 1 | NaN |
| LRRC40 | Q9H9A6 | 1.07E-14 | 1.34E-14 | 0.0278094 | | 0.100466 | 0.273724 | | 17.0164 | 17.0228 | 17.0601 | 17.2304 | 17.0461 | 16.8475 | 16.825 | 17.0775 | 23 | 25 | 22 | 25 | 21 | 23 | 21 | 24 |
| GTF2F2 | P13984 | 1.08E-12 | 7.73E-13 | 0.0280228 | | 0.051606 | 0.146982 | | 15.3009 | 15.3795 | 15.3601 | 15.2408 | 15.7334 | 14.8576 | 15.3215 | 15.4409 | 14 | 15 | 15 | 14 | 15 | 12 | 14 | 15 |
| TTL | Q8NG68 | 6.53E-06 | 1.75E-06 | 0.0280518 | | 0.100151 | 0.272937 | | 15.1377 | 15.0636 | 15.0498 | 14.9565 | 15.1924 | 14.8055 | 15.1803 | 15.1434 | 6 | 5 | 6 | 4 | 4 | 5 | 7 | 5 |
| TBC1D2B | Q9UPU7 | 4.55E-09 | 1.73E-09 | 0.0281225 | | 0.131264 | 0.348797 | | 15.4447 | 15.4113 | 15.4186 | 15.576 | 15.4573 | 15.4151 | 15.2284 | 15.3072 | 18 | 20 | 19 | 18 | 13 | 17 | 16 | 18 |
| ATG9A | Q7Z3C6 | 9.91E-13 | 7.18E-13 | 0.0284299 | | 0.103125 | 0.280339 | | 15.7092 | 15.7194 | 15.763 | 15.9423 | 15.4683 | 15.7198 | 15.6666 | 15.7135 | 15 | 14 | 14 | 13 | 10 | 12 | 12 | 12 |
| PSME1 | Q06323 | 1.93E-11 | 1.06E-11 | 0.0284903 | | 0.068787 | 0.192744 | | 18.2346 | 18.3141 | 18.3901 | 17.904 | 18.4248 | 18.5471 | 18.2768 | 18.2694 | 20 | 20 | 20 | 19 | 17 | 20 | 19 | 19 |
| COX4I1 | P13073 | 1.02E-07 | 3.28E-08 | 0.0285633 | | 0.540423 | 1.16531 | | 18.4843 | 18.4917 | 18.4918 | 18.4328 | 18.4725 | 18.5262 | 18.4238 | 18.4482 | 9 | 9 | 9 | 9 | 8 | 9 | 8 | 9 |
| PUM1 | Q14671 | 4.71E-06 | 1.28E-06 | 0.0286096 | | 0.117987 | 0.316838 | | 16.0714 | 16.068 | 16.074 | 16.292 | 15.9534 | 16.0276 | 15.8965 | 16.0431 | 16 | 17 | 17 | 16 | 14 | 15 | 16 | 15 |
| ATL3 | Q6DD88 | 4.34E-10 | 1.88E-10 | 0.0287674 | | 0.211787 | 0.531656 | | 18.8491 | 18.9096 | 18.8954 | 18.8393 | 18.775 | 18.9935 | 18.8828 | 18.7891 | 25 | 25 | 27 | 25 | 25 | 24 | 26 | 27 |
| MRPS16 | Q9Y3D3 | 0.000623 | 0.000132 | 0.0289256 | | 0.275601 | 0.666031 | | 15.8717 | 16.0343 | 15.9767 | 15.8961 | 16.0079 | 15.9135 | 15.9225 | 15.9199 | 5 | 5 | 5 | 5 | 4 | 5 | 4 | 5 |

| Gene names | UniProt IDs | Protein Group P-value | Protein Group Q-value | Log2 Difference NUP214 vs Controls (n=5) | p<0.05 | -log10 p-value | T-test Test statistic | NPC Localization | Log2 MS2_quantity_ NUP214_1 | Log2 MS2_quantity_ NUP214_2 | Log2 MS2_quantity_ NUP214_3 | Log2 MS2_quantity_ Control_1 | Log2 MS2_quantity_ Control_2 | Log2 MS2_quantity_ Control_3 | Log2 MS2_quantity_ Control_4 | Log2 MS2_quantity_ Control_5 | Number Peptides for Quantitation NUP214_1 | Number Peptides for Quantitation NUP214_2 | Number Peptides for Quantitation NUP214_3 | Number Peptides for Quantitation Control_1 | Number Peptides for Quantitation Control_2 | Number Peptides for Quantitation Control_3 | Number Peptides for Quantitation Control_4 | Number Peptides for Quantitation Control_5 |
|---|---|---|---|---|---|---|---|---|---|---|---|---|---|---|---|---|---|---|---|---|---|---|---|---|
| TEX264 | Q9Y6I9 | 3.47E-10 | 1.52E-10 | 0.0290792 | | 0.099097 | 0.270306 | | 16.3007 | 16.3347 | 16.3507 | 16.5654 | 16.0988 | 16.3805 | 16.207 | 16.2465 | 7 | 7 | 6 | 7 | 6 | 8 | 8 | 7 |
| SRSF6 | Q13247;Q1 | 4.28E-12 | 2.67E-12 | 0.0291786 | | 0.143365 | 0.37743 | | 17.7865 | 17.7144 | 17.6857 | 17.6545 | 17.7861 | 17.522 | 17.6924 | 17.8435 | 12 | 12 | 12 | 11 | 10 | 11 | 10 | 10 |
| OSBP | P22059 | 2.00E-12 | 1.34E-12 | 0.0292236 | | 0.175706 | 0.451841 | | 16.6568 | 16.6689 | 16.6457 | 16.513 | 16.6428 | 16.794 | 16.6372 | 16.5526 | 18 | 19 | 18 | 19 | 18 | 19 | 19 | 19 |
| HACD2 | Q6Y1H2 | 4.93E-11 | 2.51E-11 | 0.0292626 | | 0.039038 | 0.112582 | | 15.7191 | 15.4948 | 15.7276 | 15.0609 | 15.6083 | 15.6373 | 16.2554 | 15.5277 | 3 | 3 | 3 | 3 | 3 | 3 | 3 | 3 |
| ICMT | O60725 | 1.14E-07 | 3.67E-08 | 0.0293755 | | 0.103186 | 0.280489 | | 17.2768 | 17.2002 | 17.3289 | 17.4387 | 16.9782 | 17.2692 | 17.2018 | 17.3083 | 3 | 3 | 3 | 3 | 3 | 3 | 3 | 3 |
| ALDH18A1 | P54886;P5 | 6.58E-11 | 3.26E-11 | 0.0294277 | | 0.08231 | 0.22782 | | 18.1487 | 18.2282 | 18.1911 | 18.0464 | 18.2049 | 18.3735 | 18.3257 | 17.8492 | 33 | 33 | 35 | 34 | 32 | 34 | 35 | 34 |
| BTF3L4 | Q96K17 | 6.73E-05 | 1.63E-05 | 0.0298459 | | 0.055064 | 0.156306 | | 16.432 | 16.5 | 16.4731 | 16.0032 | 16.3321 | 16.6512 | 16.835 | 16.3711 | 7 | 7 | 7 | 7 | 5 | 6 | 7 | 6 |
| GOLPH3 | Q9H4A6 | 1.78E-06 | 5.03E-07 | 0.0300965 | | 0.05263 | 0.149748 | | 16.2188 | 16.1265 | 16.0655 | 16.4874 | 15.574 | 16.1846 | 16.1935 | 16.0947 | 7 | 7 | 7 | 6 | 7 | 6 | 7 | 7 |
| LRPAP1 | P30533 | 3.09E-14 | 3.39E-14 | 0.0302385 | | 0.104303 | 0.28326 | | 17.5324 | 17.5194 | 17.5089 | 17.3801 | 17.7852 | 17.3486 | 17.5303 | 17.4056 | 22 | 22 | 21 | 21 | 23 | 21 | 21 | 21 |
| ZC3H4 | Q9UPT8 | 0.003578 | 0.000655 | 0.0303465 | | 0.184503 | 0.471592 | | 14.0274 | 14.1856 | 14.0411 | 14.0393 | 14.1949 | 14.0077 | 14.0694 | 13.9605 | 7 | 6 | 10 | 8 | 5 | 6 | 8 | 6 |
| FRMD4A | Q9P2Q2 | 1.14E-15 | 1.92E-15 | 0.0304923 | | 0.051991 | 0.148023 | | 14.6014 | 14.5959 | 14.4979 | 15.0854 | 14.4973 | 14.5612 | 14.1664 | 14.3626 | 6 | 5 | 5 | 7 | 4 | 5 | 3 | 4 |
| UPF3B | Q9BZI7;Q9 | 2.99E-05 | 7.49E-06 | 0.0307323 | | 0.147877 | 0.38799 | | 14.88 | 14.8325 | 14.8682 | 14.8679 | 15.0098 | 14.8401 | 14.6479 | 14.7818 | 7 | 7 | 6 | 6 | 7 | 6 | 6 | 5 |
| TUBGCP2 | Q9BSJ2;Q9 | 6.51E-20 | 4.29E-19 | 0.0308021 | | 0.211879 | 0.531856 | | 15.7917 | 15.7839 | 15.7548 | 15.8431 | 15.7984 | 15.5897 | 15.7623 | 15.7364 | 27 | 27 | 27 | 28 | 26 | 24 | 27 | 24 |
| HARS1 | P12081;P1 | 3.17E-09 | 1.23E-09 | 0.0308542 | | 0.141345 | 0.372681 | | 18.5822 | 18.5934 | 18.6223 | 18.7349 | 18.3971 | 18.4972 | 18.5315 | 18.6814 | 24 | 25 | 24 | 25 | 22 | 24 | 25 | 25 |
| CD55 | P08174;P0 | 1.19E-07 | 3.82E-08 | 0.0308898 | | 0.091183 | 0.250417 | | 16.3901 | 16.2499 | 16.2586 | 16.1368 | 16.1614 | 16.6202 | 16.2173 | 16.2076 | 6 | 7 | 7 | 7 | 5 | 7 | 6 | 5 |
| DNM1L | O00429-4 | 6.53E-24 | 1.61E-22 | 0.0308921 | | 0.482458 | 1.06161 | | 17.4753 | 17.4599 | 17.4825 | 17.4441 | 17.3793 | 17.424 | 17.5124 | 17.4485 | 38 | 38 | 38 | 37 | 38 | 37 | 39 | 38 |
| MYO1C | O00159-3 | 1.61E-10 | 7.50E-11 | 0.030998 | | 0.194549 | 0.493912 | | 19.2834 | 19.3347 | 19.2814 | 19.221 | 19.2385 | 19.4349 | 19.2874 | 19.1623 | 53 | 55 | 55 | 55 | 54 | 54 | 55 | 55 |
| GLB1 | P16278;P1 | 1.59E-11 | 8.88E-12 | 0.0311497 | | 0.037218 | 0.107534 | | 18.4766 | 18.4687 | 18.4668 | 17.7327 | 18.4196 | 18.5489 | 19.0957 | 18.4009 | 17 | 16 | 17 | 17 | 16 | 17 | 17 | 17 |
| WDR75 | Q8IWA0 | 1.08E-13 | 1.02E-13 | 0.0314529 | | 0.159474 | 0.41486 | | 14.9629 | 14.9312 | 14.8937 | 15.0474 | 14.7047 | 14.8824 | 14.9098 | 14.9448 | 22 | 23 | 22 | 21 | 19 | 20 | 18 | 21 |
| POLD1 | P28340 | 2.61E-07 | 8.12E-08 | 0.031485 | | 0.066103 | 0.185684 | | 14.9451 | 14.9169 | 14.9792 | 15.0332 | 15.0517 | 14.5421 | 14.7086 | 15.2424 | 15 | 15 | 15 | 17 | 11 | 9 | 10 | 13 |
| AKAP8L | Q9ULX6 | 5.71E-07 | 1.71E-07 | 0.0315224 | | 0.052994 | 0.150731 | | 15.3179 | 15.326 | 15.4852 | 15.6172 | 15.7622 | 15.3252 | 14.9888 | 15.0307 | 8 | 7 | 6 | 7 | 1 | 5 | 7 | 7 |
| WDFY2 | Q96P53 | 3.62E-06 | 9.94E-07 | 0.0318201 | | 0.023969 | 0.070796 | | 14.3012 | 14.2381 | 14.5978 | 15.1465 | 13.3821 | NaN | 14.2382 | 14.6221 | 4 | 3 | 4 | 6 | 3 | NaN | 2 | 4 |
| PSMD6 | Q15008 | 6.82E-16 | 1.22E-15 | 0.0319327 | | 0.537445 | 1.16003 | | 18.0189 | 18.0415 | 18.0478 | 18.0627 | 17.9621 | 17.994 | 18.0379 | 17.9639 | 27 | 26 | 26 | 25 | 26 | 26 | 25 | 25 |
| RPL10 | P27635 | 3.40E-09 | 1.31E-09 | 0.0319623 | | 0.182064 | 0.466136 | | 20.355 | 20.3266 | 20.311 | 20.3223 | 20.1725 | 20.4756 | 20.2352 | 20.289 | 11 | 11 | 11 | 11 | 11 | 11 | 11 | 11 |
| MCM2 | P49736 | 4.71E-12 | 2.90E-12 | 0.0321578 | | 0.039926 | 0.11504 | | 16.7367 | 16.7173 | 16.706 | 17.1809 | 16.6748 | 15.9911 | 16.5447 | 17.0476 | 25 | 23 | 26 | 26 | 22 | 21 | 24 | 26 |
| RSL1D1 | O76021 | 1.68E-09 | 6.72E-10 | 0.0323736 | | 0.258747 | 0.631288 | | 17.2758 | 17.3368 | 17.3452 | 17.414 | 17.1986 | 17.2686 | 17.2432 | 17.3099 | 22 | 22 | 21 | 21 | 22 | 22 | 23 | 23 |
| MRPL9 | Q9BYD2 | 9.32E-05 | 2.22E-05 | 0.0324261 | | 0.265512 | 0.645293 | | 16.4938 | 16.5965 | 16.5376 | 16.5079 | 16.5175 | 16.3903 | 16.5357 | 16.5996 | 10 | 9 | 11 | 8 | 9 | 8 | 8 | 9 |
| LIX1L | Q8IVB5 | 2.56E-07 | 7.97E-08 | 0.0326772 | | 0.096671 | 0.264236 | | 15.5587 | 15.5378 | 15.4196 | 15.5499 | 15.1432 | 15.6418 | 15.4299 | 15.5985 | 4 | 6 | 3 | 5 | 3 | 7 | 4 | 6 |
| TBK1 | Q9UHD2 | 3.48E-16 | 6.72E-16 | 0.0327552 | | 0.223358 | 0.556621 | | 15.8683 | 15.8096 | 15.8561 | 15.8206 | 15.9451 | 15.842 | 15.6839 | 15.7679 | 27 | 26 | 26 | 26 | 23 | 25 | 22 | 25 |
| MESD | Q14696 | 1.92E-14 | 2.23E-14 | 0.0327648 | | 0.17399 | 0.447964 | | 17.6743 | 17.7159 | 17.7006 | 17.7659 | 17.5178 | 17.7902 | 17.6882 | 17.559 | 13 | 13 | 13 | 13 | 11 | 13 | 13 | 11 |
| CEP41 | Q9BYV8 | 4.01E-14 | 4.26E-14 | 0.0331843 | | 0.164783 | 0.427033 | | 14.9449 | 15.0976 | 15.0599 | 15.008 | 14.9554 | 14.8676 | 14.9854 | 15.1884 | 8 | 10 | 8 | 8 | 8 | 7 | 7 | 8 |
| RPRD1A | Q96P16;Q9 | 2.54E-07 | 7.91E-08 | 0.033285 | | 0.23923 | 0.590412 | | 15.2231 | 15.3126 | 15.2748 | 15.2249 | 15.1447 | 15.1581 | 15.3358 | 15.321 | 6 | 6 | 4 | 5 | 4 | 4 | 6 | 5 |
| PAFAH1B1 | P43034 | 2.56E-24 | 7.58E-23 | 0.0334176 | | 0.25762 | 0.628947 | | 18.0971 | 18.0812 | 18.072 | 18.0273 | 17.9244 | 18.1103 | 18.1558 | 18.0324 | 26 | 25 | 26 | 23 | 24 | 24 | 26 | 24 |
| TBC1D20 | Q96BZ9;Q9 | 9.97E-07 | 2.90E-07 | 0.033638 | | 0.285482 | 0.686177 | | 15.5737 | 15.5773 | 15.6472 | 15.4732 | 15.4941 | 15.625 | 15.639 | 15.5977 | 8 | 9 | 8 | 9 | 6 | 7 | 8 | 9 |
| C11orf98 | E9PRG8 | 2.11E-23 | 4.39E-22 | 0.0336436 | | 0.173095 | 0.445939 | | 15.7857 | 15.7755 | 15.6861 | 15.8234 | 15.5335 | 15.8031 | 15.7611 | 15.6562 | 2 | 2 | 4 | 3 | 3 | 4 | 4 | 3 |
| NHLRC2 | Q8NBF2 | 3.87E-06 | 1.06E-06 | 0.0338242 | | 0.145789 | 0.38311 | | 16.5113 | 16.5935 | 16.4863 | 16.4807 | 16.4097 | 16.5917 | 16.6799 | 16.3207 | 15 | 16 | 16 | 15 | 9 | 13 | 13 | 10 |
| FAR1 | Q8WVX9 | 1.88E-12 | 1.27E-12 | 0.0339101 | | 0.059136 | 0.167211 | | 15.4546 | 15.4643 | 15.4519 | 15.2824 | 15.9853 | 15.1436 | 15.2132 | 15.4908 | 14 | 13 | 13 | 12 | 13 | 13 | 10 | 13 |
| NAA16 | Q6N069 | 9.99E-12 | 5.81E-12 | 0.0341239 | | 0.095694 | 0.261785 | | 16.548 | 16.5667 | 16.5442 | 16.8285 | 16.3193 | 16.552 | 16.2992 | 16.5953 | 7 | 9 | 8 | 9 | 7 | 8 | 10 | 10 |
| SCFD2 | Q8WU76 | 1.04E-10 | 4.96E-11 | 0.0342328 | | 0.354762 | 0.823323 | | 16.2348 | 16.1697 | 16.1251 | 16.1458 | 16.2122 | 16.0621 | 16.1774 | 16.114 | 14 | 12 | 12 | 13 | 12 | 14 | 13 | 13 |
| ANAPC4 | Q9UJX5;Q9 | 0.000813 | 0.00017 | 0.0343682 | | 0.138838 | 0.366771 | | 15.2795 | 15.2772 | 15.1672 | 15.2028 | 15.2226 | 15.4273 | 15.1771 | 15.0049 | 7 | 7 | 6 | 6 | 6 | 6 | 5 | 7 |
| KNTC1 | P50748 | 2.81E-05 | 7.05E-06 | 0.0345695 | | 0.070778 | 0.197957 | | 14.8214 | 14.8844 | 14.8641 | 15.0553 | 14.9428 | 14.37 | 14.6938 | 15.0484 | 10 | 13 | 13 | 12 | 11 | 5 | 7 | 11 |
| SPATA5L1 | Q9BVQ7 | 7.53E-13 | 5.61E-13 | 0.0346033 | | 0.154533 | 0.40346 | | 15.7884 | 15.7242 | 15.7438 | 15.8485 | 15.611 | 15.7354 | 15.8559 | 15.5369 | 8 | 8 | 11 | 9 | 9 | 11 | 10 | 9 |
| TIPRL | O75663 | 9.08E-16 | 1.58E-15 | 0.0346985 | | 0.199819 | 0.505523 | | 17.1736 | 17.1167 | 17.1065 | 17.0105 | 16.9799 | 17.2106 | 17.2209 | 17.0661 | 13 | 13 | 12 | 12 | 13 | 13 | 13 | 13 |
| NOL10 | Q9BSC4;Q9 | 1.02E-11 | 5.89E-12 | 0.0347996 | | 0.17468 | 0.449523 | | 14.6645 | 14.8526 | 14.7165 | 14.8181 | 14.5238 | 14.7231 | 14.7438 | 14.7398 | 14 | 12 | 13 | 11 | 12 | 11 | 10 | 13 |
| PSMA4 | P25789 | 1.69E-08 | 5.99E-09 | 0.0348043 | | 0.16152 | 0.419562 | | 19.2495 | 19.2085 | 19.1208 | 18.9773 | 19.279 | 19.2931 | 19.106 | 19.1351 | 14 | 14 | 14 | 14 | 12 | 14 | 13 | 14 |
| NOL9 | Q5SY16 | 2.91E-10 | 1.29E-10 | 0.0348338 | | 0.290972 | 0.697301 | | 15.971 | 15.9317 | 15.9759 | 15.7997 | 16.0181 | 15.9238 | 15.909 | 15.9728 | 10 | 11 | 11 | 10 | 10 | 9 | 10 | 10 |
| BSG | P35613;P3 | 7.44E-09 | 2.75E-09 | 0.0348494 | | 0.165314 | 0.428246 | | 19.3843 | 19.3667 | 19.3368 | 19.1706 | 19.4467 | 19.2711 | 19.491 | 19.2594 | 9 | 9 | 9 | 9 | 9 | 9 | 9 | 9 |
| NVL | O15381 | 0.000158 | 3.66E-05 | 0.0349675 | | 0.158478 | 0.412567 | | 14.3235 | 14.3938 | 14.3254 | 14.2776 | 14.4507 | 14.1366 | 14.2402 | 14.4579 | 3 | 4 | 4 | 3 | 4 | 3 | 3 | 3 |
| COQ9 | O75208 | 4.98E-11 | 2.53E-11 | 0.0349906 | | 0.05323 | 0.151366 | | 14.6265 | 14.3338 | 15.0874 | 14.3404 | 14.8105 | 14.9804 | 14.3758 | 14.7308 | 5 | 5 | 5 | 5 | 3 | 4 | 4 | 4 |
| CMBL | Q96DG6 | 2.26E-12 | 1.50E-12 | 0.034992 | | 0.060115 | 0.16982 | | 17.7753 | 17.8347 | 17.7777 | 17.4805 | 17.6856 | 17.9228 | 18.2738 | 17.4418 | 12 | 12 | 11 | 9 | 9 | 11 | 11 | 9 |
| CD2BP2 | O95400 | 4.72E-08 | 1.58E-08 | 0.0350358 | | 0.094019 | 0.257573 | | 15.8654 | 15.8401 | 15.8395 | 16.1755 | 15.5638 | 15.8484 | 15.7068 | 15.7718 | 7 | 7 | 8 | 7 | 6 | 6 | 6 | 7 |
| COPS6 | Q7L5N1 | 1.31E-22 | 2.06E-21 | 0.0350911 | | 0.355024 | 0.823829 | | 16.9715 | 16.9654 | 16.9623 | 16.8179 | 16.9204 | 16.9712 | 17.007 | 16.9399 | 14 | 14 | 14 | 14 | 14 | 14 | 14 | 14 |
| C7orf50 | Q9BRJ6 | 1.26E-09 | 5.16E-10 | 0.035549 | | 0.136131 | 0.360369 | | 16.0907 | 16.0244 | 16.0315 | 16.1542 | 15.9776 | 15.7448 | 16.1068 | 16.0832 | 7 | 6 | 7 | 7 | 6 | 6 | 5 | 7 |
| MBD1 | Q9UIS9;Q9 | 0.001305 | 0.000265 | 0.0355897 | | 0.308231 | 0.741409 | | 14.2582 | 14.2903 | 14.1433 | 14.1451 | NaN | 14.217 | 14.256 | 14.1618 | 2 | 2 | 3 | 3 | NaN | 2 | 3 | 3 |
| ARMC1 | Q9NVT9 | 1.62E-05 | 4.16E-06 | 0.0355923 | | 0.145806 | 0.383149 | | 15.3234 | 15.3953 | 15.3695 | 15.4171 | 15.1176 | 15.2117 | 15.4718 | 15.4176 | 7 | 7 | 7 | 7 | 6 | 7 | 6 | 6 |
| SKIV2L | Q15477 | 6.31E-10 | 2.67E-10 | 0.0357357 | | 0.148982 | 0.390566 | | 16.3327 | 16.3592 | 16.3286 | 16.2394 | 16.1843 | 16.4299 | 16.5037 | 16.1648 | 39 | 36 | 37 | 40 | 35 | 39 | 38 | 37 |
| MRPL13 | Q9BYD1 | 6.81E-16 | 1.22E-15 | 0.035748 | | 0.208602 | 0.524734 | | 16.4733 | 16.4387 | 16.4884 | 16.4894 | 16.5743 | 16.3102 | 16.3231 | 16.4584 | 9 | 9 | 10 | 10 | 9 | 9 | 8 | 8 |
| RAB12 | Q6IQ22 | 9.27E-14 | 8.93E-14 | 0.0357608 | | 0.251157 | 0.615476 | | 16.8376 | 16.9305 | 16.9017 | 16.7685 | 16.9422 | 16.8938 | 16.9224 | 16.744 | 12 | 12 | 13 | 13 | 13 | 11 | 14 | 13 |
| UBE4A | Q14139;Q1 | 1.86E-16 | 4.05E-16 | 0.0358135 | | 0.124615 | 0.332864 | | 15.8057 | 15.849 | 15.8331 | 15.8666 | 15.4833 | 15.9454 | 15.8134 | 15.8586 | 17 | 17 | 17 | 16 | 12 | 17 | 16 | 16 |
| YTHDC2 | Q9H6S0 | 1.78E-06 | 5.04E-07 | 0.0360062 | | 0.093055 | 0.255144 | | 15.3956 | 15.4143 | 15.5493 | 15.3648 | 15.582 | 15.635 | 15.0544 | 15.4491 | 17 | 19 | 20 | 14 | 11 | 18 | 13 | 13 |
| RPL19 | P84098 | 0.000188 | 4.29E-05 | 0.0363244 | | 0.217767 | 0.544595 | | 19.3652 | 19.454 | 19.2715 | 19.4217 | 19.368 | 19.2923 | 19.1861 | 19.3682 | 9 | 9 | 9 | 7 | 9 | 8 | 8 | 9 |
| SF1 | Q15637-4 | 2.99E-16 | 5.92E-16 | 0.0364306 | | 0.4164 | 0.940313 | | 17.1736 | 17.2216 | 17.2362 | 17.2314 | 17.1705 | 17.1286 | 17.1016 | 17.2382 | 13 | 13 | 13 | 13 | 13 | 13 | 12 | 12 |
| MAIP1 | Q8WWC4 | 0.000515 | 0.00011 | 0.0364319 | | 0.138494 | 0.365958 | | 15.725 | 15.6494 | 15.5423 | 15.4647 | 15.847 | 15.5841 | 15.6345 | 15.482 | 5 | 4 | 4 | 4 | 5 | 4 | 5 | 4 |
| HTATSF1 | O43719 | 6.34E-11 | 3.16E-11 | 0.0365266 | | 0.12527 | 0.33444 | | 15.5764 | 15.7061 | 15.6673 | 15.6553 | 15.5992 | 15.3336 | 15.821 | 15.6579 | 10 | 12 | 10 | 12 | 8 | 11 | 11 | 11 |
| LACTB | P83111 | 1.08E-20 | 9.63E-20 | 0.0366034 | | 0.150837 | 0.394886 | | 16.9558 | 16.9156 | 16.9231 | 16.7435 | 17.0961 | 16.9152 | 16.7384 | 16.9812 | 16 | 19 | 18 | 17 | 14 | 20 | 18 | 17 |
| ARHGEF1 | Q92888;Q9 | 5.02E-13 | 3.93E-13 | 0.0366089 | | 0.418291 | 0.943839 | | 16.7853 | 16.7411 | 16.7604 | 16.7793 | 16.6331 | 16.7876 | 16.728 | 16.7003 | 27 | 29 | 28 | 26 | 25 | 28 | 28 | 27 |
| BARGIN | Q6ZT62;Q6 | 1.14E-07 | 3.68E-08 | 0.0366842 | | 0.078906 | 0.219065 | | 14.2398 | 14.2799 | 14.1542 | 14.2246 | 14.5348 | 13.9582 | 14.3571 | 13.8651 | 5 | 8 | 5 | 5 | 2 | 3 | 3 | 4 |
| TMEM165 | Q9HC07 | 5.70E-05 | 1.39E-05 | 0.0367836 | | 0.156346 | 0.40765 | | 17.8375 | 17.7392 | 17.6997 | 17.9299 | 17.7678 | 17.6679 | 17.5405 | 17.7039 | 5 | 5 | 5 | 5 | 5 | 5 | 5 | 5 |
| UBE2F | Q969M7;Q | 1.03E-07 | 3.32E-08 | 0.0368073 | | 0.239733 | 0.591474 | | 15.6934 | 15.7342 | 15.6409 | 15.4872 | 15.6824 | 15.7446 | 15.7021 | 15.6471 | 5 | 6 | 5 | 5 | 3 | 5 | 4 | 4 |
| AGO3 | Q9H9G7 | 9.45E-08 | 3.06E-08 | 0.036886 | | 0.070386 | 0.196933 | | 14.5824 | 14.5135 | 14.5376 | 14.0748 | 14.5601 | 14.7432 | 14.8409 | 14.3189 | 8 | 7 | 7 | 4 | 4 | 6 | 5 | 4 |
| TRIP6 | Q15654 | 1.07E-11 | 6.16E-12 | 0.0369466 | | 0.163223 | 0.423466 | | 17.6804 | 17.6793 | 17.6359 | 17.5203 | 17.8826 | 17.5561 | 17.5959 | 17.5864 | 14 | 14 | 14 | 14 | 14 | 13 | 14 | 14 |
| FOXK1 | P85037 | 1.15E-10 | 5.49E-11 | 0.0371609 | | 0.111206 | 0.300282 | | 15.2499 | 15.2485 | 15.2001 | 15.387 | 15.3062 | 15.1121 | 14.8732 | 15.3 | 11 | 12 | 13 | 14 | 9 | 12 | 11 | 10 |
| ABCD3 | P28288 | 1.64E-11 | 9.16E-12 | 0.037173 | | 0.118514 | 0.318117 | | 16.5488 | 16.5134 | 16.5075 | 16.2502 | 16.3977 | 16.5201 | 16.7827 | 16.4796 | 18 | 17 | 16 | 18 | 18 | 17 | 19 | 17 |
| MAPK9 | P45984;P4 | 8.76E-08 | 2.84E-08 | 0.0371854 | | 0.075237 | 0.209573 | | 15.6335 | 15.7472 | 15.7945 | 15.9378 | 15.1936 | 15.8606 | 15.749 | 15.6984 | 9 | 9 | 10 | 9 | 7 | 8 | 8 | 9 |
| AHNAK | Q09666 | 3.44E-24 | 9.50E-23 | 0.0372948 | | 0.169377 | 0.437505 | | 19.9207 | 19.8842 | 19.9103 | 19.9602 | 20.0464 | 19.8024 | 19.6779 | 19.8519 | 470 | 461 | 461 | 458 | 468 | 462 | 457 | 466 |
| CSE1L | P55060;P5 | 3.14E-13 | 2.61E-13 | 0.0373516 | | 0.18918 | 0.482015 | | 17.7958 | 17.7636 | 17.825 | 17.904 | 17.7445 | 17.6136 | 17.6546 | 17.8704 | 47 | 47 | 47 | 48 | 47 | 46 | 48 | 48 |
| SRP72 | O76094 | 3.30E-10 | 1.45E-10 | 0.0374587 | | 0.250681 | 0.61448 | | 17.4305 | 17.4571 | 17.4224 | 17.2707 | 17.4664 | 17.5207 | 17.4113 | 17.327 | 26 | 26 | 26 | 27 | 26 | 25 | 26 | 25 |
| DHX57 | Q6P158 | 1.46E-11 | 8.24E-12 | 0.0375971 | | 0.19419 | 0.493119 | | 15.7904 | 15.8428 | 15.826 | 16.0021 | 15.7462 | 15.7279 | 15.6791 | 15.7553 | 18 | 19 | 20 | 20 | 19 | 17 | 17 | 19 |

| Gene names | UniProt IDs | Protein Group P-value | Protein Group Q-value | Log2 Difference NUP214 vs Controls (n=5) | p<0.05 | -log10 p-value | T-test Test statistic | NPC Localization | Log2 MS2_quantity_NUP214_1 | Log2 MS2_quantity_NUP214_2 | Log2 MS2_quantity_NUP214_3 | Log2 MS2_quantity_Control_1 | Log2 MS2_quantity_Control_2 | Log2 MS2_quantity_Control_3 | Log2 MS2_quantity_Control_4 | Log2 MS2_quantity_Control_5 | Number Peptides for Quantitation NUP214_1 | Number Peptides for Quantitation NUP214_2 | Number Peptides for Quantitation NUP214_3 | Number Peptides for Quantitation Control_1 | Number Peptides for Quantitation Control_2 | Number Peptides for Quantitation Control_3 | Number Peptides for Quantitation Control_4 | Number Peptides for Quantitation Control_5 |
|---|---|---|---|---|---|---|---|---|---|---|---|---|---|---|---|---|---|---|---|---|---|---|---|---|
| SLC25A15 | Q9Y619 | 0.002893 | 0.000542 | 0.0381997 | | 0.065367 | 0.185314 | | 15.065 | 15.236 | 15.1723 | 15.2383 | NaN | 15.447 | 15.15 | 14.6431 | 3 | 2 | 2 | 2 | NaN | 3 | 2 | 1 |
| SEC63 | Q9UGP8 | 6.64E-19 | 3.04E-18 | 0.0383626 | | 0.224871 | 0.559864 | | 17.0369 | 17.0443 | 17.0188 | 16.9378 | 16.9177 | 17.1537 | 17.077 | 16.8887 | 29 | 29 | 29 | 29 | 25 | 28 | 27 | 28 |
| RPUSD2 | Q8IZ73 | 2.36E-05 | 5.96E-06 | 0.0384743 | | 0.084759 | 0.23409 | | 14.2327 | 14.0865 | 13.8397 | 13.8355 | 14.2014 | 14.2011 | 13.6889 | 14.1455 | 3 | 3 | 3 | 4 | 2 | 2 | 3 | 2 |
| PFKFB2 | O60825;O6 | 0.001531 | 0.000308 | 0.0384841 | | 0.040105 | 0.115535 | | 14.775 | 14.7034 | 14.838 | 13.887 | 14.8109 | 15.154 | 15.2815 | 14.535 | 4 | 4 | 5 | 1 | 1 | 5 | 4 | 3 |
| HDAC7 | Q8WUI4;Q | 3.45E-18 | 1.28E-17 | 0.0385136 | | 0.147293 | 0.386628 | | 15.6312 | 15.6349 | 15.5385 | 15.5875 | 15.6189 | 15.766 | 15.3198 | 15.5229 | 12 | 16 | 17 | 15 | 14 | 15 | 12 | 13 |
| OGFOD3 | Q6PK18 | 4.40E-05 | 1.09E-05 | 0.0385712 | | 0.127402 | 0.339561 | | 15.9522 | 16.1353 | 15.9743 | 16.1046 | 15.975 | 16.0236 | 16.1226 | 15.6843 | 5 | 7 | 6 | 6 | 5 | 6 | 5 | 4 |
| CD82 | P27701 | 1.41E-05 | 3.65E-06 | 0.0387989 | | 0.020085 | 0.059081 | | 16.5095 | 16.5341 | 16.6498 | 14.8089 | 16.9342 | 17.0636 | 17.6659 | 16.1558 | 3 | 3 | 3 | 1 | 2 | 2 | 5 | 2 |
| PSMA3 | P25788;P2 | 1.31E-19 | 7.68E-19 | 0.0388362 | | 0.162912 | 0.422753 | | 19.0642 | 19.2422 | 19.2043 | 18.9847 | 19.1303 | 19.2678 | 19.2734 | 19.0008 | 9 | 8 | 8 | 8 | 9 | 8 | 8 | 8 |
| DDX27 | Q96GQ7 | 3.21E-13 | 2.66E-13 | 0.0390865 | | 0.339229 | 0.793148 | | 15.9922 | 16.0559 | 16.0207 | 16.0689 | 16.0217 | 15.8804 | 15.9206 | 16.0276 | 23 | 21 | 22 | 24 | 17 | 21 | 20 | 21 |
| RO60 | P10155 | 1.94E-10 | 8.91E-11 | 0.0393678 | | 0.205247 | 0.517416 | | 17.3987 | 17.4051 | 17.3615 | 17.2364 | 17.3751 | 17.5128 | 17.4127 | 17.2084 | 20 | 19 | 20 | 20 | 17 | 20 | 20 | 19 |
| TTI1 | O43156 | 3.87E-06 | 1.06E-06 | 0.0395059 | | 0.125543 | 0.335097 | | 15.2079 | 15.4198 | 15.1685 | 15.3149 | 15.2919 | 14.9863 | 15.1158 | 15.4208 | 6 | 4 | 4 | 4 | 3 | 3 | 3 | 3 |
| ARF1 | P84077 | 1.78E-07 | 5.63E-08 | 0.0396189 | | 0.176888 | 0.454505 | | 21.0937 | 21.1918 | 21.1313 | 21.3127 | 20.9268 | 21.0571 | 21.1417 | 21.0583 | 12 | 11 | 9 | 12 | 12 | 10 | 12 | 12 |
| DDX6 | P26196 | 1.49E-14 | 1.80E-14 | 0.0396198 | | 0.269462 | 0.653433 | | 18.1159 | 18.1851 | 18.1169 | 18.1896 | 18.2047 | 18.0071 | 17.997 | 18.1 | 22 | 22 | 22 | 22 | 21 | 21 | 22 | 21 |
| TEX10 | Q9NXF1;Q9 | 1.12E-11 | 6.44E-12 | 0.039633 | | 0.317902 | 0.751199 | | 15.9433 | 15.9043 | 15.891 | 15.7449 | 15.8694 | 15.8763 | 15.9879 | 15.8875 | 20 | 17 | 19 | 19 | 15 | 18 | 19 | 18 |
| AGK | Q53H12 | 7.56E-15 | 9.86E-15 | 0.0398788 | | 0.158054 | 0.411591 | | 16.137 | 16.2102 | 16.199 | 16.1772 | 15.8587 | 16.219 | 16.2396 | 16.2166 | 12 | 12 | 12 | 11 | 9 | 12 | 10 | 11 |
| COX20 | Q5RI15;Q5 | 5.93E-10 | 2.52E-10 | 0.0399204 | | 0.155545 | 0.405801 | | 17.3194 | 17.3405 | 17.2814 | 17.1671 | 17.5499 | 17.181 | 17.1726 | 17.2986 | 4 | 4 | 4 | 4 | 4 | 4 | 4 | 4 |
| CD109 | Q6YHK3 | 8.47E-15 | 1.09E-14 | 0.039957 | | 0.064822 | 0.182304 | | 15.7253 | 15.7377 | 15.6984 | 15.8552 | 16.0507 | 15.1016 | 15.8324 | 15.5625 | 31 | 29 | 30 | 30 | 26 | 27 | 28 | 26 |
| IK | Q13123 | 8.76E-08 | 2.84E-08 | 0.0399717 | | 0.171475 | 0.44227 | | 16.2726 | 16.2905 | 16.2714 | 16.291 | 16.4414 | 16.0587 | 16.1212 | 16.2788 | 15 | 15 | 17 | 14 | 12 | 14 | 15 | 15 |
| RPS17 | P08708 | 6.60E-05 | 1.60E-05 | 0.0401766 | | 0.486545 | 1.069 | | 19.5314 | 19.4751 | 19.4603 | 19.5027 | 19.5118 | 19.4359 | 19.3794 | 19.4138 | 10 | 8 | 8 | 10 | 9 | 8 | 9 | 9 |
| KCTD18 | Q6PI47 | 0.004964 | 0.000888 | 0.0403531 | | 0.048366 | 0.139352 | | 15.1007 | 14.8153 | 15.4898 | 14.6932 | 15.644 | 14.9322 | 15.1103 | NaN | 2 | 3 | 2 | 1 | 1 | 2 | 1 | NaN |
| TFAM | Q00059 | 1.24E-07 | 3.97E-08 | 0.0405059 | | 0.225352 | 0.560893 | | 16.0442 | 16.1355 | 16.0122 | 16.038 | 16.0518 | 16.1678 | 15.8553 | 16.0043 | 11 | 10 | 11 | 11 | 8 | 11 | 9 | 11 |
| EBF1 | Q9UH73 | 0.00183 | 0.000362 | 0.0407527 | | 0.075786 | 0.210997 | | 14.9835 | 14.8952 | 14.855 | 14.5226 | 15.0333 | 15.1841 | 14.5262 | 15.0863 | 4 | 4 | 3 | 2 | 3 | 2 | 1 | 3 |
| HSPA12A | O43301 | 6.36E-11 | 3.16E-11 | 0.0408394 | | 0.052402 | 0.149133 | | 15.3401 | 15.3913 | 15.4258 | 14.7267 | 15.3763 | 15.9967 | 15.4346 | 15.1902 | 19 | 18 | 19 | 12 | 13 | 18 | 13 | 15 |
| RUVBL1 | Q9Y265 | 1.46E-20 | 1.24E-19 | 0.0408891 | | 0.474193 | 1.04663 | | 18.4232 | 18.4401 | 18.4288 | 18.3215 | 18.4942 | 18.3923 | 18.3515 | 18.3896 | 24 | 23 | 25 | 22 | 20 | 24 | 23 | 24 |
| RAB8A | P61006 | 3.74E-07 | 1.14E-07 | 0.0409013 | | 0.155066 | 0.404692 | | 18.3438 | 18.3054 | 18.2632 | 18.2764 | 18.5474 | 18.1359 | 18.1841 | 18.1725 | 8 | 8 | 8 | 8 | 8 | 8 | 8 | 7 |
| MYBBP1A | Q9BQG0;Q | 3.01E-14 | 3.31E-14 | 0.0410254 | | 0.317286 | 0.749976 | | 16.6383 | 16.6523 | 16.632 | 16.637 | 16.5884 | 16.5646 | 16.4809 | 16.7284 | 36 | 35 | 34 | 31 | 34 | 37 | 33 | 37 |
| FRMD6 | Q96NE9-2 | 7.80E-12 | 4.63E-12 | 0.0411352 | | 0.113879 | 0.306827 | | 16.174 | 16.1251 | 16.1356 | 16.4579 | 15.8752 | 15.9526 | 16.1127 | 16.1204 | 19 | 20 | 20 | 20 | 16 | 20 | 18 | 19 |
| TM9SF1 | O15321;O1 | 3.48E-16 | 6.72E-16 | 0.041423 | | 0.151498 | 0.396422 | | 16.9304 | 16.9165 | 16.9725 | 16.8209 | 16.8264 | 16.8795 | 17.201 | 16.7641 | 7 | 9 | 9 | 9 | 9 | 7 | 9 | 9 |
| TMCO3 | Q6UWJ1;Q | 0.000425 | 9.24E-05 | 0.0416368 | | 0.13457 | 0.356666 | | 14.9275 | 15.3024 | 15.3236 | 15.2444 | 15.0079 | 15.2786 | 15.0613 | 15.1223 | 4 | 5 | 3 | 4 | 3 | 4 | 3 | 4 |
| AOX1 | Q06278 | 3.60E-11 | 1.88E-11 | 0.0418084 | | 0.030524 | 0.088801 | | 14.8193 | 14.9247 | 14.9212 | 13.5849 | 14.707 | 15.6253 | 14.9643 | 15.3513 | 6 | 5 | 5 | 2 | 2 | 6 | 3 | 3 |
| PNKP | Q96T60 | 3.22E-12 | 2.06E-12 | 0.0420003 | | 0.125542 | 0.335094 | | 15.5388 | 15.7078 | 15.5913 | 15.7164 | 15.5852 | 15.2315 | 15.592 | 15.7281 | 10 | 11 | 11 | 12 | 11 | 7 | 12 | 8 |
| MRPL32 | Q9BYC8 | 0.000106 | 2.51E-05 | 0.0421602 | | 0.354887 | 0.823565 | | 14.5083 | 14.5642 | 14.5331 | 14.5405 | 14.5851 | 14.3829 | 14.4303 | 14.5263 | 3 | 4 | 4 | 3 | 4 | 4 | 4 | 4 |
| RBM4 | Q9BWF3 | 1.35E-06 | 3.88E-07 | 0.0422115 | | 0.193883 | 0.492441 | | 16.9441 | 16.9262 | 16.9731 | 16.9987 | 16.9844 | 16.6797 | 16.8483 | 17.0168 | 13 | 14 | 14 | 13 | 13 | 13 | 14 | 14 |
| OGFR | Q9NZT2;Q9 | 7.26E-14 | 7.19E-14 | 0.0422192 | | 0.253 | 0.619324 | | 15.7921 | 15.8507 | 15.8073 | 15.7471 | 15.9618 | 15.6583 | 15.7606 | 15.7446 | 13 | 14 | 13 | 13 | 12 | 12 | 10 | 11 |
| NIP7 | Q9Y221 | 7.70E-08 | 2.51E-08 | 0.0424334 | | 0.136832 | 0.362028 | | 15.663 | 15.629 | 15.6418 | 15.8299 | 15.3113 | 15.6456 | 15.5243 | 15.6997 | 5 | 6 | 6 | 6 | 5 | 6 | 6 | 6 |
| HNRNPH3 | P31942;P3 | 0.000205 | 4.66E-05 | 0.0427797 | | 0.242635 | 0.597594 | | 19.403 | 19.226 | 19.2636 | 19.1395 | 19.2584 | 19.1687 | 19.3577 | 19.3495 | 7 | 8 | 7 | 6 | 7 | 7 | 7 | 7 |
| POP4 | O95707 | 2.13E-08 | 7.43E-09 | 0.0431591 | | 0.282646 | 0.680411 | | 14.8272 | 14.7889 | 14.8508 | 14.6351 | 14.8847 | 14.875 | 14.7678 | 14.7332 | 5 | 5 | 5 | 5 | 4 | 5 | 5 | 5 |
| GTF3C5 | Q9Y5Q8;Q9 | 7.52E-15 | 9.82E-15 | 0.0432453 | | 0.305115 | 0.725742 | | 15.2278 | 15.1804 | 15.2126 | 15.2165 | 15.2675 | 15.0079 | 15.1414 | 15.1849 | 12 | 12 | 12 | 10 | 9 | 10 | 10 | 9 |
| ERI1 | Q8IV48 | 0.000122 | 2.86E-05 | 0.0432809 | | 0.111188 | 0.300236 | | 15.1447 | 15.0086 | 15.0864 | 14.7313 | 15.0261 | 14.9093 | 15.1665 | 15.3498 | 6 | 6 | 4 | 4 | 4 | 4 | 3 | 3 |
| GALT | P07902 | 4.69E-10 | 2.02E-10 | 0.043405 | | 0.165477 | 0.428621 | | 15.5134 | 15.4989 | 15.4726 | 15.2769 | 15.5473 | 15.6521 | 15.5075 | 15.274 | 6 | 4 | 4 | 4 | 5 | 4 | 3 | 3 |
| MGST3 | O14880 | 2.52E-06 | 7.03E-07 | 0.0436228 | | 0.097758 | 0.266959 | | 20.4017 | 20.397 | 20.3227 | 20.113 | 20.8004 | 20.1844 | 20.2519 | 20.301 | 5 | 5 | 5 | 5 | 5 | 5 | 5 | 5 |
| RABEP1 | Q15276 | 8.61E-17 | 2.06E-16 | 0.0437006 | | 0.343347 | 0.801176 | | 15.3349 | 15.4052 | 15.3264 | 15.2454 | 15.3031 | 15.4554 | 15.3113 | 15.2437 | 21 | 22 | 22 | 22 | 20 | 20 | 23 | 20 |
| DNTTIP1 | Q9H147 | 1.09E-08 | 3.96E-09 | 0.0439676 | | 0.08639 | 0.238251 | | 14.7285 | 14.4782 | 14.6178 | 14.3873 | 15.0579 | 14.56 | 14.2901 | 14.5257 | 4 | 5 | 5 | 3 | 2 | 3 | 5 | 3 |
| RBFOX2 | O43251-6; | 1.09E-09 | 4.50E-10 | 0.0440945 | | 0.117524 | 0.315712 | | 17.2808 | 17.3118 | 17.1756 | 17.1999 | 17.5755 | 16.9404 | 17.1695 | 17.1747 | 7 | 9 | 8 | 8 | 5 | 6 | 7 | 8 |
| GOT1 | P17174 | 1.72E-05 | 4.41E-06 | 0.0442596 | | 0.092611 | 0.254024 | | 18.3279 | 18.3592 | 18.2889 | 17.9271 | 18.3034 | 18.5844 | 18.5411 | 18.0494 | 18 | 19 | 19 | 19 | 18 | 19 | 18 | 20 |
| EIF2B5 | Q13144 | 1.14E-15 | 1.91E-15 | 0.0444866 | | 0.279902 | 0.67482 | | 16.8086 | 16.8895 | 16.9136 | 16.7252 | 16.7929 | 16.9583 | 16.9108 | 16.743 | 16 | 14 | 14 | 14 | 12 | 13 | 13 | 14 |
| SUPT6H | Q7KZ85 | 9.10E-19 | 3.98E-18 | 0.0445238 | | 0.263663 | 0.641473 | | 14.8016 | 14.7937 | 14.8392 | 14.888 | 14.7336 | 14.5947 | 14.7646 | 14.854 | 32 | 33 | 31 | 30 | 22 | 30 | 30 | 31 |
| TALDO1 | P37837 | 9.83E-16 | 1.69E-15 | 0.0445362 | | 0.097858 | 0.267209 | | 19.4351 | 19.5391 | 19.4585 | 19.8942 | 19.1583 | 19.437 | 19.3498 | 19.3258 | 19 | 18 | 18 | 18 | 18 | 18 | 19 | 19 |
| FN3K | Q9H479 | 6.94E-10 | 2.92E-10 | 0.0445552 | | 0.063317 | 0.178325 | | 15.2345 | 14.9518 | 14.9579 | 14.6589 | 14.9565 | 14.6854 | 15.6552 | 15.0616 | 5 | 3 | 3 | 2 | 5 | 3 | 4 | 3 |
| DHCR7 | Q9UBM7 | 0.000397 | 8.66E-05 | 0.0445849 | | 0.148135 | 0.388591 | | 18.5398 | 18.5552 | 18.5367 | 18.739 | 18.565 | 18.2069 | 18.5096 | 18.476 | 5 | 5 | 5 | 5 | 5 | 5 | 5 | 5 |
| POLDIP3 | Q9BY77 | 3.10E-10 | 1.37E-10 | 0.044778 | | 0.131059 | 0.348307 | | 16.1151 | 16.0412 | 15.9272 | 16.1012 | 16.1749 | 15.7518 | 15.7695 | 16.1178 | 9 | 9 | 9 | 10 | 9 | 9 | 9 | 9 |
| SPATS2 | Q86XZ4 | 1.11E-12 | 7.94E-13 | 0.0448087 | | 0.170598 | 0.44028 | | 14.2079 | 14.2286 | 14.1899 | 14.2976 | 14.2201 | 14.3358 | 13.9811 | 13.9856 | 6 | 9 | 7 | 5 | 6 | 5 | 6 | 5 |
| DHX40 | Q8IX18;Q8 | 0.000449 | 9.73E-05 | 0.0449361 | | 0.079488 | 0.220565 | | 13.3933 | 13.5862 | 13.1521 | 13.6564 | 13.5217 | 13.4682 | 13.0387 | 12.9763 | 4 | 4 | 3 | 2 | 3 | 3 | 3 | 2 |
| UBE2D2 | P62837 | 1.57E-05 | 4.04E-06 | 0.0449835 | | 0.182179 | 0.466393 | | 18.893 | 18.8604 | 18.8025 | 18.9759 | 18.8553 | 18.9235 | 18.6531 | 18.6272 | 4 | 4 | 4 | 3 | 4 | 4 | 4 | 4 |
| PRMT5 | O14744 | 9.78E-10 | 4.06E-10 | 0.0450118 | | 0.204446 | 0.515665 | | 17.195 | 17.2249 | 17.1815 | 17.098 | 16.9781 | 17.3726 | 17.1276 | 17.2009 | 22 | 22 | 22 | 21 | 18 | 21 | 20 | 21 |
| NIF3L1 | Q9GZT8;Q9 | 7.76E-06 | 2.06E-06 | 0.0452779 | | 0.538367 | 1.16167 | | 17.5926 | 17.5857 | 17.634 | 17.5904 | 17.5508 | 17.6382 | 17.5464 | 17.4682 | 12 | 11 | 12 | 12 | 9 | 11 | 12 | 11 |
| TMEM126A | Q9H061 | 3.18E-07 | 9.80E-08 | 0.045341 | | 0.348787 | 0.811751 | | 16.4124 | 16.3913 | 16.3582 | 16.2029 | 16.4349 | 16.3511 | 16.4098 | 16.3111 | 7 | 7 | 7 | 6 | 7 | 7 | 7 | 7 |
| STUB1 | Q9UNE7 | 1.19E-18 | 5.12E-18 | 0.0456451 | | 0.139092 | 0.367372 | | 17.2105 | 17.2934 | 17.2306 | 17.4433 | 17.3317 | 16.9011 | 17.14 | 17.1797 | 19 | 19 | 17 | 18 | 17 | 17 | 18 | 18 |
| ZZEF1 | O43149 | 1.34E-12 | 9.34E-13 | 0.0457003 | | 0.600994 | 1.2715 | | 14.7851 | 14.7784 | 14.7469 | 14.7271 | 14.7475 | 14.6334 | 14.7202 | 14.7941 | 29 | 29 | 30 | 26 | 17 | 25 | 26 | 26 |
| MOB4 | Q9Y3A3;Q9 | 4.33E-10 | 1.88E-10 | 0.0457219 | | 0.623677 | 1.31079 | | 16.4061 | 16.3737 | 16.4449 | 16.3082 | 16.4303 | 16.3198 | 16.4031 | 16.3513 | 7 | 7 | 7 | 7 | 7 | 7 | 7 | 6 |
| AP2A2 | O94973 | 4.56E-15 | 6.40E-15 | 0.045806 | | 0.393569 | 0.897457 | | 18.3844 | 18.3854 | 18.3546 | 18.24 | 18.3665 | 18.3622 | 18.4333 | 18.243 | 36 | 37 | 39 | 36 | 34 | 37 | 38 | 36 |
| SEC22A | Q96IW7 | 4.69E-07 | 1.42E-07 | 0.0458632 | | 0.096587 | 0.264026 | | 15.2333 | 15.271 | 14.9937 | 15.3268 | 14.6799 | 15.2397 | 15.0414 | 15.3129 | 2 | 3 | 3 | 3 | 2 | 3 | 2 | 2 |
| PHIP | Q8WWQ0 | 7.10E-09 | 2.63E-09 | 0.0459382 | | 0.329699 | 0.774478 | | 14.5936 | 14.6618 | 14.6241 | 14.6193 | 14.6796 | 14.4204 | 14.5868 | 14.5964 | 10 | 8 | 7 | 7 | 5 | 7 | 7 | 6 |
| STARD10 | Q9Y365 | 0.002859 | 0.000537 | 0.0462125 | | 0.057766 | 0.163551 | | 15.5045 | 15.3603 | 15.5348 | 15.249 | 15.9151 | 15.9038 | 15.171 | 14.8629 | 2 | 2 | 2 | 2 | 1 | 2 | 2 | 2 |
| EXD2 | Q9NVH0 | 2.29E-15 | 3.54E-15 | 0.0464643 | | 0.105831 | 0.287043 | | 14.8948 | 14.9856 | 14.9211 | 14.9993 | 15.176 | 14.4488 | 14.9336 | 14.8792 | 9 | 7 | 7 | 10 | 6 | 6 | 10 | 9 |
| CLPB | Q9H078-2 | 6.42E-15 | 8.60E-15 | 0.0465827 | | 0.228718 | 0.56809 | | 15.843 | 15.8192 | 15.8131 | 15.9178 | 15.7459 | 15.6308 | 15.6725 | 15.9256 | 16 | 16 | 15 | 16 | 12 | 14 | 14 | 15 |
| CRTAP | O75718 | 1.44E-06 | 4.12E-07 | 0.0467089 | | 0.052254 | 0.148732 | | 18.7452 | 18.6517 | 18.6512 | 17.9907 | 18.5254 | 19.0364 | 19.2889 | 18.3386 | 18 | 20 | 20 | 17 | 18 | 19 | 20 | 18 |
| IPO5 | O00410;O0 | 2.56E-24 | 7.58E-23 | 0.0468449 | | 0.79291 | 1.59827 | | 19.1058 | 19.0972 | 19.1169 | 19.0921 | 18.9965 | 19.0574 | 19.0325 | 19.1204 | 43 | 43 | 43 | 43 | 41 | 44 | 43 | 42 |
| ZNF638 | Q14966 | 1.31E-16 | 3.00E-16 | 0.0470662 | | 0.387025 | 0.885075 | | 15.4514 | 15.4939 | 15.5139 | 15.3838 | 15.5395 | 15.3248 | 15.451 | 15.4976 | 24 | 25 | 27 | 20 | 18 | 18 | 22 | 22 |
| PSMB2 | P49721 | 5.64E-17 | 1.43E-16 | 0.0471785 | | 0.20078 | 0.507634 | | 19.3146 | 19.0823 | 19.2352 | 19.0007 | 19.3215 | 19.0692 | 19.1685 | 19.2579 | 10 | 10 | 10 | 10 | 9 | 10 | 9 | 11 |
| AP1B1 | Q10567;Q1 | 1.64E-13 | 1.46E-13 | 0.0472748 | | 0.321319 | 0.757962 | | 18.319 | 18.3006 | 18.288 | 18.3935 | 18.102 | 18.2764 | 18.263 | 18.2412 | 46 | 48 | 47 | 45 | 47 | 46 | 47 | 48 |
| GALK2 | Q01415;Q0 | 0.002046 | 0.000401 | 0.0476531 | | 0.104637 | 0.284089 | | 15.5429 | 15.5981 | 15.7085 | 15.3259 | 16.0194 | 15.3982 | 15.6226 | 15.478 | 4 | 6 | 6 | 4 | 3 | 3 | 3 | 2 |
| RTRAF | Q9Y224 | 3.90E-08 | 1.32E-08 | 0.0476571 | | 0.075702 | 0.210779 | | 17.682 | 17.5538 | 17.9669 | 17.8497 | 17.6208 | 17.1109 | 17.9925 | 17.8589 | 16 | 16 | 16 | 16 | 15 | 15 | 16 | 16 |
| NMRK1 | Q9NWW6 | 0.006545 | 0.00115 | 0.0478919 | | 0.073922 | 0.207932 | | 15.758 | 15.9871 | 15.8718 | 16.2848 | 15.3873 | NaN | 15.7007 | 15.9248 | 2 | 2 | 3 | 4 | 3 | NaN | 3 | 4 |
| NR2C2 | P49116;P4 | 0.000106 | 2.50E-05 | 0.0480686 | | 0.390218 | 0.891123 | | 14.4785 | 14.6111 | 14.6334 | 14.6091 | 14.4555 | 14.4955 | 14.5891 | 14.4822 | 4 | 6 | 5 | 6 | 2 | 6 | 4 | 6 |
| GTF2H1 | P32780 | 5.48E-12 | 3.35E-12 | 0.0481088 | | 0.267423 | 0.649234 | | 14.3545 | 14.5858 | 14.4293 | 14.3727 | 14.3504 | 14.3136 | 14.5381 | 14.4673 | 6 | 6 | 10 | 7 | 4 | 3 | 8 | 5 |
| CISD2 | Q8N5K1 | 2.79E-06 | 7.73E-07 | 0.0483117 | | 0.16032 | 0.416806 | | 17.0958 | 17.1177 | 17.0473 | 17.0817 | 16.981 | 16.7588 | 17.2902 | 17.0813 | 7 | 7 | 7 | 7 | 6 | 7 | 7 | 7 |
| DIPK2A | Q8NDZ4 | 1.64E-05 | 4.23E-06 | 0.0483459 | | 0.082209 | 0.227561 | | 15.082 | 15.1898 | 15.0193 | 15.4907 | 15.0243 | 14.5105 | 15.1117 | 15.1062 | 4 | 4 | 4 | 4 | 4 | 3 | 4 | 5 |

| Gene names | UniProt IDs | Protein Group P-value | Protein Group Q-value | Log2 Difference NUP214 vs Controls (n=5) | p<0.05 | -log10 p-value | T-test Test statistic | NPC Localization | Log2 MS2_quantity_ NUP214_1 | Log2 MS2_quantity_ NUP214_2 | Log2 MS2_quantity_ NUP214_3 | Log2 MS2_quantity_ Control_1 | Log2 MS2_quantity_ Control_2 | Log2 MS2_quantity_ Control_3 | Log2 MS2_quantity_ Control_4 | Log2 MS2_quantity_ Control_5 | Number Peptides for Quantitation NUP214_1 | Number Peptides for Quantitation NUP214_2 | Number Peptides for Quantitation NUP214_3 | Number Peptides for Quantitation Control_1 | Number Peptides for Quantitation Control_2 | Number Peptides for Quantitation Control_3 | Number Peptides for Quantitation Control_4 | Number Peptides for Quantitation Control_5 |
|---|---|---|---|---|---|---|---|---|---|---|---|---|---|---|---|---|---|---|---|---|---|---|---|---|
| HSDL2 | Q6YN16 | 1.59E-17 | 4.73E-17 | 0.0483582 | | 0.107401 | 0.29092 | | 16.6942 | 16.7303 | 16.6609 | 16.9125 | 16.3575 | 16.4547 | 16.5377 | 16.9714 | 23 | 23 | 24 | 23 | 17 | 23 | 21 | 22 |
| HMGN1 | P05114 | 0.000773 | 0.000162 | 0.048359 | | 0.064534 | 0.181544 | | 15.4233 | 15.2545 | 15.5511 | 15.0004 | 15.8557 | 14.8399 | 15.4283 | 15.6821 | 7 | 7 | 6 | 5 | 5 | 5 | 5 | 3 |
| LNPEP | Q9UIQ6;Q9 | 2.46E-18 | 9.51E-18 | 0.0483912 | | 0.180931 | 0.463595 | | 16.6066 | 16.6341 | 16.6476 | 16.881 | 16.5042 | 16.5743 | 16.4384 | 16.5074 | 28 | 29 | 29 | 29 | 28 | 29 | 28 | 29 |
| NIBAN1 | Q9BZQ8 | 5.52E-16 | 1.00E-15 | 0.0485152 | | 0.064904 | 0.182523 | | 16.9088 | 16.9966 | 16.9416 | 16.4597 | 16.946 | 17.5082 | 17.1125 | 16.4759 | 28 | 28 | 28 | 27 | 26 | 28 | 28 | 28 |
| METTL2B | Q6P1Q9;Q6 | 0.000821 | 0.000171 | 0.0485238 | | 0.106743 | 0.291894 | | 14.1394 | 14.2286 | 13.9246 | 13.7845 | NaN | 14.0721 | 14.3799 | 13.9596 | 2 | 1 | 1 | 1 | NaN | 1 | 2 | 1 |
| CCDC6 | Q16204 | 1.29E-12 | 9.09E-13 | 0.0485411 | | 0.25487 | 0.623224 | | 17.0369 | 17.0906 | 17.046 | 17.111 | 17.0506 | 17.1358 | 16.8416 | 16.9074 | 19 | 18 | 18 | 19 | 16 | 19 | 18 | 19 |
| TRIM47 | Q96LD4 | 7.77E-07 | 2.29E-07 | 0.0485867 | | 0.071136 | 0.198894 | | 14.7909 | 14.7615 | 14.6748 | 14.3115 | 15.2108 | 15.0028 | 14.3034 | 14.6405 | 4 | 4 | 5 | 5 | 2 | 2 | 2 | 2 |
| NCOR1 | O75376;O7 | 2.12E-10 | 9.70E-11 | 0.0488176 | | 0.1137 | 0.306389 | | 15.0818 | 15.3978 | 15.4577 | 14.9609 | 15.482 | 15.4224 | 15.091 | 15.3618 | 6 | 6 | 6 | 2 | 2 | 2 | 2 | 1 |
| RPS15A | P62244 | 6.87E-05 | 1.66E-05 | 0.0489624 | | 0.459993 | 1.02078 | | 21.4945 | 21.5576 | 21.4382 | 21.4651 | 21.4048 | 21.5606 | 21.398 | 21.4106 | 8 | 8 | 8 | 8 | 8 | 8 | 8 | 8 |
| WASHC2C | Q9Y4E1 | 6.25E-08 | 2.06E-08 | 0.0489751 | | 0.316889 | 0.74919 | | 15.0135 | 14.9203 | 14.9012 | 14.8283 | 14.835 | 14.9754 | 15.0326 | 14.8088 | 5 | 5 | 6 | 3 | 3 | 5 | 4 | 3 |
| RB1CC1 | Q8TDY2;Q8 | 5.56E-08 | 1.85E-08 | 0.048998 | | 0.179092 | 0.459466 | | 14.0801 | 14.2253 | 14.3816 | 14.1472 | 14.4032 | 14.1889 | 14.0041 | 14.1566 | 8 | 9 | 10 | 7 | 4 | 6 | 4 | 4 |
| EPHX1 | P07099 | 2.08E-09 | 8.23E-10 | 0.0490383 | | 0.096664 | 0.264216 | | 17.7658 | 17.8184 | 17.8024 | 17.4489 | 17.5665 | 17.8885 | 18.2214 | 17.6073 | 24 | 24 | 24 | 23 | 24 | 23 | 25 | 24 |
| METTL3 | Q86U44 | 5.51E-17 | 1.40E-16 | 0.0491625 | | 0.153421 | 0.400884 | | 14.7334 | 14.7771 | 14.8716 | 14.7772 | 15.0634 | 14.5367 | 14.7136 | 14.6336 | 12 | 13 | 11 | 11 | 8 | 10 | 11 | 10 |
| DPH5 | Q9H2P9;Q9 | 1.32E-12 | 9.25E-13 | 0.0492858 | | 0.161574 | 0.419686 | | 16.1112 | 16.1654 | 16.099 | 16.3289 | 15.938 | 16.0002 | 15.8786 | 16.2339 | 6 | 5 | 6 | 6 | 6 | 5 | 7 | 6 |
| CTIF | O43310 | 9.31E-16 | 1.61E-15 | 0.0495083 | | 0.129371 | 0.344276 | | 16.1771 | 16.3189 | 16.3327 | 16.0831 | 16.1214 | 16.6397 | 16.1122 | 16.1772 | 18 | 16 | 16 | 18 | 11 | 17 | 17 | 15 |
| PURB | Q96QR8 | 4.32E-05 | 1.07E-05 | 0.0495431 | | 0.138484 | 0.365936 | | 16.9953 | 16.9266 | 16.942 | 16.6458 | 16.8229 | 17.15 | 17.1336 | 16.773 | 7 | 7 | 7 | 6 | 5 | 6 | 8 | 7 |
| SFXN1 | Q9H9B4 | 4.80E-06 | 1.30E-06 | 0.0495514 | | 0.433418 | 0.971926 | | 17.5182 | 17.5127 | 17.4299 | 17.4887 | 17.3848 | 17.3601 | 17.547 | 17.4064 | 10 | 10 | 10 | 11 | 10 | 10 | 11 | 10 |
| BLVRB | P30043 | 2.42E-10 | 1.09E-10 | 0.0495539 | | 0.122904 | 0.328742 | | 18.0947 | 18.1981 | 18.2101 | 18.2056 | 17.7663 | 18.2624 | 18.3879 | 17.968 | 10 | 10 | 10 | 10 | 10 | 10 | 10 | 10 |
| ERC1 | Q8IUD2 | 3.25E-13 | 2.69E-13 | 0.0496583 | | 0.187373 | 0.477993 | | 16.0529 | 15.9914 | 16.0546 | 16.2307 | 15.7936 | 15.9277 | 16.0786 | 15.886 | 36 | 38 | 36 | 39 | 26 | 36 | 30 | 35 |
| FKBP9 | O95302 | 3.66E-12 | 2.31E-12 | 0.0496684 | | 0.074345 | 0.207255 | | 19.8808 | 19.9104 | 19.8402 | 19.4667 | 19.9868 | 20.3736 | 19.9105 | 19.3998 | 21 | 21 | 22 | 19 | 19 | 20 | 19 | 19 |
| SRSF2 | Q01130;Q0 | 8.80E-12 | 5.17E-12 | 0.0497252 | | 0.270832 | 0.656251 | | 19.9832 | 20.1731 | 20.1225 | 20.0983 | 20.1987 | 19.9342 | 19.9702 | 20.0146 | 5 | 5 | 5 | 5 | 5 | 5 | 5 | 5 |
| MOB2 | Q70IA6;Q7 | 0.000329 | 7.26E-05 | 0.0498097 | | 0.362151 | 0.837573 | | 15.9502 | 15.8863 | 15.8507 | 15.8389 | 15.7981 | 16.0007 | 15.8374 | 15.7545 | 7 | 8 | 7 | 6 | 5 | 8 | 8 | 8 |
| SRRM2 | Q9UQ35 | 4.84E-15 | 6.75E-15 | 0.0500679 | | 0.722723 | 1.48005 | | 16.6321 | 16.6389 | 16.6413 | 16.5596 | 16.643 | 16.5066 | 16.591 | 16.6366 | 31 | 32 | 31 | 30 | 27 | 28 | 31 | 33 |
| ROCK2 | O75116 | 1.28E-12 | 9.04E-13 | 0.0501297 | | 0.481544 | 1.05996 | | 16.8211 | 16.7819 | 16.801 | 16.8333 | 16.7106 | 16.6367 | 16.7983 | 16.7772 | 58 | 57 | 57 | 54 | 50 | 54 | 52 | 52 |
| TRMT10C | Q7L0Y3 | 1.11E-13 | 1.04E-13 | 0.0501966 | | 0.22047 | 0.550417 | | 15.7247 | 15.798 | 15.7821 | 15.931 | 15.5198 | 15.6855 | 15.7755 | 15.6785 | 14 | 14 | 14 | 13 | 12 | 13 | 12 | 12 |
| PTPA | Q15257 | 3.47E-09 | 1.34E-09 | 0.0502291 | | 0.148316 | 0.389016 | | 17.9569 | 17.8889 | 17.9542 | 17.5234 | 18.0715 | 17.8923 | 18.0209 | 17.9075 | 11 | 12 | 12 | 10 | 10 | 10 | 10 | 11 |
| CREBBP | Q92793 | 9.03E-05 | 2.15E-05 | 0.0503279 | | 0.146447 | 0.38465 | | 14.5793 | 14.4848 | 14.3666 | 14.3441 | 14.7745 | 14.3634 | 14.2316 | 14.4192 | 10 | 11 | 13 | 6 | 4 | 6 | 6 | 9 |
| MAD1L1 | Q9Y6D9 | 1.68E-08 | 5.94E-09 | 0.0504522 | | 0.229852 | 0.570509 | | 15.2144 | 15.1265 | 15.2419 | 15.2948 | 15.1597 | 14.936 | 15.0818 | 15.2469 | 11 | 10 | 11 | 10 | 5 | 8 | 8 | 6 |
| KPNA5 | O15131 | 4.49E-06 | 1.22E-06 | 0.050584 | | 0.324181 | 0.763613 | | 18.197 | 18.0682 | 18.0226 | 18.1698 | 17.9383 | 18.0613 | 18.08 | 17.9773 | 3 | 4 | 3 | 3 | 3 | 3 | 4 | 3 |
| IPO11 | Q9UI26;Q9 | 8.84E-13 | 6.49E-13 | 0.0506295 | | 0.344834 | 0.80407 | | 15.43 | 15.4113 | 15.448 | 15.2673 | 15.3859 | 15.285 | 15.442 | 15.5153 | 12 | 12 | 14 | 14 | 13 | 13 | 13 | 15 |
| ZNF326 | Q5BKZ1 | 5.51E-07 | 1.65E-07 | 0.0506608 | | 0.433153 | 0.971436 | | 16.0816 | 15.9763 | 16.0157 | 15.9206 | 16.0865 | 15.9691 | 15.8837 | 16.0094 | 14 | 15 | 16 | 12 | 12 | 12 | 11 | 13 |
| TXNDC17 | Q9BRA2 | 7.94E-15 | 1.03E-14 | 0.0507657 | | 0.184015 | 0.470502 | | 19.8538 | 19.6419 | 19.7924 | 19.4568 | 19.6573 | 19.7755 | 19.8839 | 19.7863 | 6 | 6 | 6 | 6 | 5 | 6 | 6 | 6 |
| FH | P07954;P0 | 9.00E-11 | 4.35E-11 | 0.0508223 | | 0.234349 | 0.580073 | | 18.1072 | 18.1528 | 18.1216 | 18.3102 | 18.0714 | 18.0071 | 18.0774 | 17.9159 | 20 | 20 | 21 | 21 | 20 | 20 | 20 | 20 |
| GTF2H3 | Q13889;Q1 | 1.29E-11 | 7.36E-12 | 0.0509526 | | 0.207 | 0.521242 | | 15.2612 | 15.541 | 15.5703 | 15.3829 | 15.5624 | 15.2531 | 15.4348 | 15.3996 | 3 | 3 | 2 | 2 | 3 | 3 | 3 | 3 |
| RARS1 | P54136 | 1.03E-13 | 9.80E-14 | 0.0510049 | | 0.25213 | 0.617509 | | 18.4222 | 18.3845 | 18.3575 | 18.2913 | 18.1734 | 18.5509 | 18.3368 | 18.3329 | 45 | 45 | 46 | 46 | 44 | 48 | 46 | 46 |
| SEC14L2 | O76054 | 5.65E-05 | 1.38E-05 | 0.0516461 | | 0.084781 | 0.234144 | | 15.6137 | 15.5534 | 15.5808 | 15.1306 | 15.797 | 15.9349 | 15.6359 | 15.1564 | 8 | 6 | 6 | 7 | 6 | 8 | 7 | 5 |
| CFL2 | Q9Y281;Q9 | 3.14E-11 | 1.66E-11 | 0.0519814 | | 0.264513 | 0.64323 | | 19.7446 | 19.7773 | 19.7088 | 19.8369 | 19.8253 | 19.6316 | 19.6297 | 19.5344 | 7 | 7 | 7 | 6 | 6 | 6 | 7 | 7 |
| NUDT21 | O43809 | 8.21E-11 | 3.99E-11 | 0.0521502 | | 0.211526 | 0.531089 | | 17.4158 | 17.3074 | 17.4174 | 17.2826 | 17.4879 | 17.0988 | 17.3026 | 17.4684 | 15 | 15 | 15 | 14 | 14 | 14 | 15 | 15 |
| TDP1 | Q9NUW8 | 6.24E-07 | 1.85E-07 | 0.0521718 | | 0.074469 | 0.207577 | | 15.0588 | 15.2419 | 15.0878 | 15.1107 | 15.5899 | 14.4913 | 14.8933 | 15.3013 | 1 | 1 | 1 | 1 | 1 | 2 | 2 | 1 |
| KTN1 | Q86UP2 | 2.41E-23 | 4.93E-22 | 0.0524137 | | 0.266412 | 0.64715 | | 16.603 | 16.5982 | 16.604 | 16.4613 | 16.4908 | 16.5483 | 16.784 | 16.4625 | 82 | 85 | 84 | 78 | 69 | 79 | 84 | 75 |
| SYNCRIP | O60506 | 4.78E-17 | 1.24E-16 | 0.0524303 | | 0.844918 | 1.68527 | | 19.6686 | 19.7143 | 19.6823 | 19.6851 | 19.6077 | 19.6525 | 19.6702 | 19.5643 | 28 | 29 | 28 | 29 | 30 | 28 | 29 | 29 |
| GGPS1 | O95749 | 0.000354 | 7.79E-05 | 0.0524913 | | 0.161602 | 0.419749 | | 15.584 | 15.499 | 15.5414 | 15.1594 | 15.6629 | 15.614 | 15.5974 | 15.4112 | 7 | 7 | 7 | 7 | 5 | 5 | 6 | 6 |
| TMTC3 | Q6ZXV5;Q6 | 1.64E-16 | 3.66E-16 | 0.0529043 | | 0.302455 | 0.720416 | | 17.0122 | 17.0885 | 16.9921 | 17.1545 | 16.9805 | 16.9957 | 16.8312 | 16.9284 | 21 | 20 | 20 | 20 | 18 | 20 | 18 | 21 |
| PNN | Q9H307 | 1.43E-10 | 6.70E-11 | 0.0529429 | | 0.259512 | 0.632876 | | 15.4129 | 15.5466 | 15.5322 | 15.5494 | 15.2541 | 15.4582 | 15.5749 | 15.385 | 11 | 12 | 13 | 11 | 10 | 10 | 13 | 11 |
| GFER | P55789;P5 | 4.58E-07 | 1.39E-07 | 0.0530493 | | 0.05601 | 0.158847 | | 14.0396 | 13.8049 | 14.1721 | 14.4137 | 14.1335 | 13.0559 | 13.8516 | 14.3076 | 2 | 1 | 1 | 1 | 1 | 2 | 2 | 1 |
| LANCL2 | Q9NS86 | 1.02E-05 | 2.68E-06 | 0.0531795 | | 0.424439 | 0.95528 | | 15.8599 | 15.8376 | 15.8792 | 15.8107 | 15.8499 | 15.8954 | 15.6516 | 15.8207 | 10 | 11 | 10 | 11 | 8 | 11 | 9 | 8 |
| COG4 | Q9H9E3 | 1.96E-15 | 3.09E-15 | 0.0535678 | | 0.506321 | 1.10458 | | 16.0979 | 16.0914 | 16.1662 | 15.9918 | 16.0595 | 16.1922 | 16.0523 | 16.0288 | 23 | 20 | 20 | 21 | 18 | 19 | 18 | 18 |
| RFC4 | P35249 | 6.54E-14 | 6.57E-14 | 0.0535741 | | 0.142998 | 0.376568 | | 15.8159 | 15.7635 | 15.7034 | 15.8439 | 15.7166 | 15.3484 | 15.6542 | 15.9737 | 11 | 11 | 11 | 10 | 9 | 9 | 10 | 11 |
| TRAPPC8 | Q9Y2L5 | 2.08E-10 | 9.51E-11 | 0.0536101 | | 0.350191 | 0.814474 | | 14.7679 | 14.8608 | 14.8303 | 14.6595 | 14.8775 | 14.8043 | 14.8397 | 14.6494 | 24 | 27 | 26 | 18 | 15 | 20 | 21 | 15 |
| CTDP1 | Q9Y5B0 | 1.22E-07 | 3.91E-08 | 0.0538556 | | 0.73445 | 1.49988 | | 14.9319 | 15.0157 | 14.9534 | 14.959 | 14.9734 | 14.8607 | 14.8665 | 14.9061 | 11 | 10 | 11 | 9 | 9 | 9 | 9 | 10 |
| PHB | P35232 | 3.06E-11 | 1.62E-11 | 0.0539922 | | 0.26808 | 0.650587 | | 19.0625 | 18.9886 | 19.1186 | 19.0498 | 19.0102 | 18.7856 | 19.0267 | 19.1406 | 18 | 18 | 18 | 17 | 18 | 18 | 18 | 17 |
| MSTO1 | Q9BUK6;Q9 | 1.17E-12 | 8.36E-13 | 0.0542465 | | 0.155237 | 0.405088 | | 15.5171 | 15.5649 | 15.5683 | 15.6916 | 15.1588 | 15.627 | 15.6252 | 15.3766 | 7 | 12 | 9 | 9 | 5 | 9 | 9 | 9 |
| IRS1 | P35568 | 0.002836 | 0.000533 | 0.0542594 | | 0.069911 | 0.195689 | | 13.9208 | 14.2703 | 13.9757 | 13.4041 | 14.6028 | 13.7802 | 14.1512 | 14.0684 | 5 | 4 | 5 | 3 | 4 | 3 | 3 | 4 |
| SRSF7 | Q16629;Q1 | 1.10E-15 | 1.87E-15 | 0.0543359 | | 0.303472 | 0.722453 | | 19.6418 | 19.6518 | 19.5764 | 19.5734 | 19.7438 | 19.508 | 19.4124 | 19.6073 | 6 | 7 | 6 | 6 | 6 | 6 | 6 | 7 |
| RFX1 | P22670 | 0.000791 | 0.000165 | 0.0544426 | | 0.140531 | 0.370765 | | 14.6274 | 14.6678 | 14.4893 | 14.1243 | 14.605 | 14.6331 | 14.6166 | 14.723 | 6 | 6 | 5 | 5 | 3 | 4 | 4 | 6 |
| TRIM33 | Q9UPN9 | 0.003538 | 0.000648 | 0.0545151 | | 0.112415 | 0.303245 | | 14.0266 | 14.2533 | 14.2163 | 14.3982 | 14.3686 | 13.7001 | 14.1041 | 13.9836 | 7 | 7 | 6 | 7 | 4 | 5 | 3 | 5 |
| MRPL27 | Q9P0M9 | 0.00044 | 9.54E-05 | 0.0545258 | | 0.225799 | 0.56185 | | 14.1908 | 14.2011 | 14.2038 | 14.1947 | 14.3815 | 13.9349 | 14.1012 | 14.1079 | 3 | 2 | 2 | 2 | 2 | 2 | 2 | 2 |
| SCAF11 | Q99590;Q9 | 2.15E-08 | 7.49E-09 | 0.0545945 | | 0.270877 | 0.656343 | | 13.9668 | 13.9132 | 13.7928 | 13.822 | 14.0266 | 13.8754 | 13.7501 | 13.7076 | 6 | 7 | 8 | 4 | 5 | 5 | 6 | 5 |
| PPP3CA | Q08209-2 | 3.37E-09 | 1.30E-09 | 0.0546141 | | 0.461681 | 1.02386 | | 17.6733 | 17.5388 | 17.5191 | 17.4136 | 17.5705 | 17.5489 | 17.5044 | 17.5748 | 17 | 17 | 17 | 16 | 17 | 17 | 16 | 17 |
| VPS35L | Q7Z3J2 | 2.16E-20 | 1.73E-19 | 0.055269 | | 0.287434 | 0.690137 | | 16.0583 | 16.1198 | 16.1186 | 15.899 | 16.04 | 16.1231 | 16.2196 | 15.9365 | 35 | 34 | 35 | 33 | 29 | 32 | 34 | 32 |
| RGS10 | O43665 | 1.36E-12 | 9.46E-13 | 0.0553697 | | 0.107043 | 0.290037 | | 15.8189 | 15.6414 | 15.7304 | 15.9677 | 16.001 | 15.2771 | 15.4656 | 15.663 | 7 | 7 | 5 | 8 | 7 | 6 | 6 | 6 |
| YLPM1 | P49750 | 1.90E-11 | 1.05E-11 | 0.0554063 | | 0.55496 | 1.19098 | | 15.571 | 15.5981 | 15.6154 | 15.5078 | 15.4502 | 15.5733 | 15.6517 | 15.5143 | 31 | 28 | 31 | 33 | 22 | 30 | 32 | 28 |
| STAMBP | O95630 | 2.13E-08 | 7.44E-09 | 0.0554073 | | 0.094029 | 0.257597 | | 16.2628 | 16.2502 | 16.2554 | 15.6102 | 16.2837 | 16.3322 | 16.5848 | 16.1927 | 12 | 14 | 14 | 11 | 13 | 14 | 16 | 14 |
| JAM3 | Q9BX67 | 6.31E-05 | 1.53E-05 | 0.0554319 | | 0.139618 | 0.368612 | | 15.6199 | 15.3511 | 15.4743 | 15.6805 | 15.2297 | 15.1996 | 15.6678 | 15.3541 | 6 | 7 | 7 | 6 | 6 | 7 | 6 | 7 |
| BIRC6 | Q9NR09 | 5.41E-11 | 2.73E-11 | 0.055502 | | 0.270215 | 0.654982 | | 14.808 | 14.8524 | 14.8335 | 14.9635 | 14.8916 | 14.6802 | 14.6863 | 14.6575 | 34 | 33 | 33 | 32 | 23 | 26 | 28 | 28 |
| USP16 | Q9Y5T5;Q9 | 1.60E-10 | 7.46E-11 | 0.0555431 | | 0.204445 | 0.515662 | | 14.7773 | 14.8585 | 14.7896 | 14.8998 | 14.4679 | 14.9058 | 14.7308 | 14.7603 | 9 | 9 | 11 | 9 | 6 | 10 | 6 | 9 |
| RBBP9 | O75884 | 9.49E-07 | 2.77E-07 | 0.0555847 | | 0.104411 | 0.283529 | | 16.2717 | 15.9979 | 16.3141 | 15.8842 | 15.8299 | 16.4081 | 16.5068 | 16.066 | 5 | 5 | 5 | 5 | 5 | 5 | 5 | 5 |
| CCT3 | P49368 | 2.18E-10 | 9.94E-11 | 0.0557903 | | 0.351543 | 0.817094 | | 19.5437 | 19.5285 | 19.4998 | 19.6449 | 19.4644 | 19.4682 | 19.3303 | 19.4334 | 42 | 42 | 43 | 39 | 40 | 40 | 40 | 42 |
| TTC21B | Q7Z4L5 | 0.002807 | 0.000528 | 0.0558274 | | 0.07743 | 0.217114 | | 13.5315 | 13.5295 | 13.504 | 12.9336 | NaN | 13.8613 | 13.7763 | 13.292 | 4 | 4 | 2 | 4 | NaN | 2 | 1 | 2 |
| KDSR | Q06136 | 6.61E-15 | 8.80E-15 | 0.0559898 | | 0.338185 | 0.791108 | | 17.2962 | 17.2841 | 17.3335 | 17.111 | 17.186 | 17.2926 | 17.4217 | 17.2318 | 9 | 10 | 9 | 10 | 7 | 8 | 8 | 10 |
| COPS7A | Q9UBW8 | 5.56E-11 | 2.80E-11 | 0.05599 | | 0.11544 | 0.310637 | | 17.2637 | 17.2964 | 17.2586 | 17.2739 | 17.2827 | 16.6922 | 17.4406 | 17.3951 | 14 | 14 | 14 | 13 | 13 | 13 | 13 | 13 |
| ODR4 | Q5SWX8 | 4.27E-12 | 2.66E-12 | 0.0562103 | | 0.119348 | 0.32014 | | 14.8073 | 14.8713 | 14.7536 | 14.4274 | 14.5944 | 14.981 | 15.1318 | 14.6379 | 12 | 11 | 14 | 11 | 10 | 13 | 12 | 12 |
| PARS2 | Q7L3T8 | 0.004918 | 0.00088 | 0.0562338 | | 0.158846 | 0.413415 | | 13.5468 | 13.4694 | 13.3072 | 13.2254 | 13.2083 | 13.731 | 13.3504 | 13.4093 | 2 | 2 | 2 | 2 | 1 | 2 | 2 | 2 |
| APPBP2 | Q92624 | 0.00175 | 0.000348 | 0.0564006 | | 0.156378 | 0.407724 | | 13.2696 | 13.3967 | 13.1996 | 13.2043 | 13.4839 | 13.2675 | 13.3222 | 12.8832 | 3 | 3 | 1 | 2 | 2 | 1 | 2 | 1 |
| DST | Q03001-11 | 4.49E-12 | 2.78E-12 | 0.0565001 | | 0.123935 | 0.331226 | | 14.8583 | 15.0164 | 14.9073 | 14.8492 | 15.3591 | 14.6724 | 14.7343 | 14.739 | 5 | 4 | 5 | 5 | 4 | 4 | 5 | 5 |
| USP13 | Q92995 | 0.00052 | 0.000111 | 0.0566357 | | 0.155876 | 0.406565 | | 15.5369 | 15.5172 | 15.491 | 15.7357 | 15.5797 | 15.2821 | 15.1604 | 15.5341 | 13 | 11 | 12 | 12 | 7 | 11 | 11 | 12 |
| SPHK1 | Q9NYA1-2 | 0.001353 | 0.000274 | 0.0566368 | | 0.20453 | 0.515849 | | 16.3567 | 16.4421 | 16.2498 | 16.4726 | 16.0628 | 16.4508 | 16.2628 | 16.2155 | 3 | 3 | 3 | 3 | 3 | 3 | 3 | 3 |
| ARHGEF6 | Q15052;Q1 | 8.17E-13 | 6.02E-13 | 0.0566483 | | 0.06315 | 0.177882 | | 15.6655 | 15.5776 | 15.5776 | 15.818 | 15.9809 | 15.9352 | 14.7405 | 15.2768 | 6 | 6 | 4 | 6 | 3 | 5 | 3 | 5 |

| Gene names | UniProt IDs | Protein Group P-value | Protein Group Q-value | Log2 Difference NUP214 vs Controls (n=5) | p<0.05 | -log10 p-value | T-test Test statistic | NPC Localization | Log2 MS2_quantity_NUP214_1 | Log2 MS2_quantity_NUP214_2 | Log2 MS2_quantity_NUP214_3 | Log2 MS2_quantity_Control_1 | Log2 MS2_quantity_Control_2 | Log2 MS2_quantity_Control_3 | Log2 MS2_quantity_Control_4 | Log2 MS2_quantity_Control_5 | Number Peptides for Quantitation NUP214_1 | Number Peptides for Quantitation NUP214_2 | Number Peptides for Quantitation NUP214_3 | Number Peptides for Quantitation Control_1 | Number Peptides for Quantitation Control_2 | Number Peptides for Quantitation Control_3 | Number Peptides for Quantitation Control_4 | Number Peptides for Quantitation Control_5 |
|---|---|---|---|---|---|---|---|---|---|---|---|---|---|---|---|---|---|---|---|---|---|---|---|---|
| ASPSCR1 | Q9BZE9;Q9 | 1.05E-06 | 3.03E-07 | 0.0566574 | | 0.372654 | 0.857716 | | 16.6776 | 16.6389 | 16.665 | 16.452 | 16.6845 | 16.6916 | 16.6695 | 16.5218 | 14 | 15 | 14 | 13 | 12 | 11 | 14 | 13 |
| SMARCC2 | Q8TAQ2 | 3.47E-12 | 2.21E-12 | 0.0571512 | | 0.211478 | 0.530986 | | 16.3161 | 16.3342 | 16.2467 | 16.3603 | 16.3822 | 16.0163 | 16.0821 | 16.3683 | 29 | 28 | 29 | 27 | 20 | 23 | 25 | 28 |
| HK1 | P19367;P1 | 1.87E-12 | 1.26E-12 | 0.057261 | | 0.106909 | 0.289708 | | 18.9752 | 19.0111 | 18.9482 | 19.3736 | 18.9036 | 18.7881 | 18.479 | 19.0602 | 44 | 44 | 44 | 43 | 44 | 43 | 42 | 43 |
| UQCC1 | Q9NVA1;Q9 | 8.45E-08 | 2.75E-08 | 0.0573612 | | 0.345022 | 0.804436 | | 15.5465 | 15.4684 | 15.6101 | 15.5088 | 15.2918 | 15.5409 | 15.5453 | 15.5346 | 4 | 4 | 4 | 3 | 3 | 2 | 3 | 3 |
| SNX15 | Q9NRS6 | 5.86E-05 | 1.43E-05 | 0.057435 | | 0.342981 | 0.800463 | | 15.8317 | 15.8586 | 15.8954 | 15.8145 | 15.9059 | 15.6529 | 15.9287 | 15.7203 | 8 | 9 | 9 | 6 | 9 | 7 | 8 | 7 |
| STX10 | O60499 | 0.000677 | 0.000143 | 0.0575404 | | 0.107256 | 0.290564 | | 14.4315 | 14.115 | 14.3659 | 14.1068 | 14.7944 | 14.1768 | 14.0349 | 14.1201 | 4 | 4 | 4 | 4 | 4 | 4 | 5 | 4 |
| PRPF3 | O43395 | 1.32E-09 | 5.37E-10 | 0.0575734 | | 0.544795 | 1.17304 | | 15.7799 | 15.8028 | 15.7366 | 15.7635 | 15.764 | 15.5767 | 15.7342 | 15.7392 | 15 | 14 | 13 | 15 | 11 | 13 | 11 | 13 |
| TLN1 | Q9Y490 | 2.08E-16 | 4.41E-16 | 0.0577532 | | 0.447372 | 0.997651 | | 19.988 | 19.9729 | 19.9361 | 19.9288 | 19.9709 | 19.9877 | 19.7483 | 19.9038 | 142 | 140 | 141 | 135 | 136 | 139 | 138 | 137 |
| CDC42BPB | Q9Y5S2 | 4.00E-13 | 3.22E-13 | 0.0578418 | | 0.258063 | 0.629867 | | 15.7464 | 15.8661 | 15.8219 | 15.6815 | 15.6537 | 15.9872 | 15.812 | 15.6336 | 59 | 60 | 61 | 58 | 51 | 62 | 61 | 56 |
| PTGES2 | Q9H7Z7 | 3.14E-11 | 1.66E-11 | 0.0578735 | | 0.309911 | 0.735317 | | 16.5939 | 16.6451 | 16.6461 | 16.4426 | 16.671 | 16.742 | 16.5275 | 16.4695 | 11 | 10 | 10 | 9 | 10 | 11 | 9 | 10 |
| COG1 | Q8WTW3 | 4.92E-14 | 5.10E-14 | 0.0579121 | | 0.289173 | 0.69366 | | 15.4356 | 15.4331 | 15.4158 | 15.1737 | 15.4232 | 15.4497 | 15.5215 | 15.2833 | 18 | 20 | 21 | 18 | 17 | 19 | 18 | 20 |
| AP1G1 | O43747;O4 | 3.66E-10 | 1.60E-10 | 0.0579329 | | 0.4896 | 1.07451 | | 17.402 | 17.4404 | 17.3869 | 17.4823 | 17.2686 | 17.3499 | 17.2735 | 17.385 | 26 | 26 | 26 | 26 | 25 | 27 | 27 | 27 |
| ARL6IP4 | Q66PJ3;Q6 | 1.68E-05 | 4.32E-06 | 0.0579916 | | 0.4554 | 1.01237 | | 15.2234 | 15.2466 | 15.3214 | 15.2844 | 15.0536 | 15.2136 | 15.2455 | 15.2321 | 5 | 5 | 4 | 5 | 4 | 5 | 4 | 5 |
| PTPRS | Q13332;Q1 | 3.64E-14 | 3.91E-14 | 0.0580023 | | 0.087256 | 0.240456 | | 15.1938 | 15.3862 | 15.3508 | 15.9141 | 15.2342 | 14.9315 | 14.9489 | 15.2326 | 11 | 11 | 12 | 12 | 9 | 7 | 9 | 9 |
| KPNB1 | Q14974 | 1.08E-13 | 1.02E-13 | 0.0580563 | | 0.325526 | 0.766266 | | 19.7067 | 19.6968 | 19.7008 | 19.7954 | 19.6179 | 19.7024 | 19.4504 | 19.6508 | 33 | 33 | 34 | 32 | 32 | 33 | 32 | 31 |
| KATNA1 | O75449 | 0.00395 | 0.000718 | 0.0581192 | | 0.194435 | 0.49366 | | 15.4374 | 15.6298 | 15.5769 | 15.5528 | 15.1826 | 15.6801 | 15.5242 | 15.51 | 5 | 4 | 3 | 4 | 2 | 4 | 1 | 1 |
| CSNK1A1 | P48729 | 2.07E-09 | 8.19E-10 | 0.0585576 | | 0.237354 | 0.586444 | | 16.5904 | 16.6366 | 16.6218 | 16.8382 | 16.5839 | 16.4441 | 16.4522 | 16.4702 | 15 | 14 | 14 | 14 | 13 | 13 | 14 | 15 |
| PITPNB | P48739;P4 | 2.32E-14 | 2.64E-14 | 0.0585932 | | 0.149832 | 0.392548 | | 17.2049 | 17.2379 | 17.2303 | 17.3364 | 16.8129 | 17.4636 | 17.1309 | 17.0852 | 18 | 19 | 18 | 19 | 19 | 18 | 19 | 18 |
| ANAPC1 | Q9H1A4 | 1.91E-18 | 7.66E-18 | 0.0585985 | | 0.434642 | 0.974191 | | 15.2049 | 15.1189 | 15.189 | 15.04 | 15.0951 | 15.2671 | 15.1293 | 15.0303 | 17 | 19 | 19 | 16 | 10 | 14 | 12 | 13 |
| INPP4A | Q96PE3;Q9 | 5.57E-05 | 1.36E-05 | 0.0586468 | | 0.207152 | 0.521573 | | 15.2335 | 15.177 | 15.1404 | 14.901 | 15.4051 | 15.1791 | 15.0799 | 15.0599 | 7 | 7 | 6 | 6 | 4 | 6 | 5 | 6 |
| UMPS | P11172 | 1.56E-11 | 8.76E-12 | 0.0587234 | | 0.5616 | 1.20266 | | 16.4783 | 16.5124 | 16.4657 | 16.3424 | 16.3621 | 16.5318 | 16.4144 | 16.483 | 16 | 18 | 17 | 17 | 17 | 18 | 18 | 18 |
| RNPEP | Q9H4A4 | 6.10E-12 | 3.71E-12 | 0.0588661 | | 0.18885 | 0.48128 | | 18.67 | 18.6841 | 18.6875 | 18.5221 | 18.8946 | 18.6405 | 18.7057 | 18.3454 | 26 | 26 | 25 | 25 | 25 | 26 | 25 | 25 |
| UBTD2 | Q8WUN7 | 0.002347 | 0.000452 | 0.0590333 | | 0.144968 | 0.381188 | | 15.0459 | 15.026 | 14.9818 | 15.1607 | 14.7918 | 15.1637 | 15.0924 | 14.5857 | 5 | 2 | 3 | 2 | 3 | 4 | 2 | 3 |
| TMEM87A | Q8NBN3 | 3.62E-08 | 1.23E-08 | 0.0595177 | | 0.139412 | 0.368127 | | 16.1611 | 16.228 | 16.1574 | 16.2168 | 15.6888 | 16.3063 | 16.3554 | 16.0459 | 11 | 10 | 11 | 12 | 10 | 11 | 12 | 11 |
| DYM | Q7RTS9 | 3.29E-13 | 2.72E-13 | 0.0596087 | | 0.271115 | 0.656831 | | 16.2494 | 16.2917 | 16.2423 | 16.1989 | 16.306 | 16.2323 | 16.323 | 15.9474 | 7 | 7 | 8 | 5 | 7 | 6 | 7 | 7 |
| PRKAB1 | Q9Y478 | 4.81E-07 | 1.45E-07 | 0.0596404 | | 0.163524 | 0.424154 | | 15.7747 | 15.6998 | 15.7603 | 15.4644 | 15.4279 | 15.8704 | 15.7156 | 15.9481 | 5 | 4 | 5 | 4 | 4 | 5 | 5 | 4 |
| PRXL2B | Q8TBF2;Q8 | 3.06E-15 | 4.54E-15 | 0.0599616 | | 0.070481 | 0.197181 | | 15.0077 | 15.0545 | 14.9769 | 15.4403 | 15.3336 | 14.307 | 14.5158 | 15.1685 | 5 | 5 | 7 | 5 | 6 | 4 | 3 | 4 |
| RANBP3 | Q9H6Z4-2 | 7.50E-08 | 2.45E-08 | 0.0601906 | | 0.312007 | 0.739492 | | 15.718 | 15.8064 | 15.797 | 15.8805 | 15.7752 | 15.5659 | 15.5923 | 15.7541 | 10 | 8 | 9 | 9 | 4 | 10 | 7 | 9 |
| AP5Z1 | O43299 | 9.46E-13 | 6.90E-13 | 0.0601972 | | 0.270985 | 0.656564 | | 15.173 | 15.1179 | 15.0265 | 15.0993 | 15.2737 | 14.9258 | 14.9896 | 14.9397 | 8 | 8 | 9 | 9 | 9 | 9 | 7 | 8 |
| FBXO6 | Q9NRD1 | 0.000106 | 2.50E-05 | 0.0602488 | | 0.05106 | 0.145502 | | 13.9156 | 14.5371 | 14.7706 | 14.7585 | 13.3589 | 14.8789 | 14.6038 | 14.1374 | 5 | 6 | 4 | 2 | 2 | 5 | 4 | 2 |
| DDX19A | Q9NUU7 | 2.48E-07 | 7.72E-08 | 0.0602818 | | 0.444714 | 0.992765 | | 15.6659 | 15.6896 | 15.6458 | 15.4876 | 15.7532 | 15.6494 | 15.5542 | 15.5898 | 4 | 4 | 4 | 4 | 2 | 4 | 3 | 4 |
| SLC22A18 | Q96BI1 | 0.001476 | 0.000297 | 0.0603338 | | 0.116892 | 0.314173 | | 15.4437 | 15.4884 | 15.5131 | 15.1188 | 15.8239 | 15.6484 | 15.4221 | 15.0938 | 3 | 3 | 3 | 3 | 3 | 2 | 3 | 2 |
| CSK | P41240 | 1.65E-09 | 6.62E-10 | 0.060392 | | 0.447303 | 0.997525 | | 17.0333 | 17.0621 | 17.0459 | 17.065 | 17.0154 | 17.0674 | 16.8231 | 16.9626 | 22 | 21 | 22 | 22 | 20 | 20 | 21 | 23 |
| TSSC4 | Q9Y5U2;Q9 | 1.60E-09 | 6.43E-10 | 0.0605339 | | 0.10557 | 0.286397 | | 15.5132 | 15.4342 | 15.5528 | 15.2683 | 15.7018 | 15.6874 | 14.897 | 15.6431 | 4 | 4 | 5 | 5 | 5 | 4 | 4 | 5 |
| NDUFS5 | O43920 | 1.02E-10 | 4.86E-11 | 0.0606903 | | 0.141611 | 0.373307 | | 17.0132 | 16.9396 | 17.1361 | 17.1771 | 16.5232 | 17.0209 | 17.1502 | 16.9735 | 6 | 5 | 6 | 6 | 5 | 5 | 6 | 6 |
| DDX31 | Q9H8H2 | 0.000147 | 3.42E-05 | 0.0608094 | | 0.186762 | 0.476632 | | 14.9608 | 15.0555 | 15.0882 | 15.2845 | 14.7318 | 14.9611 | 15.0401 | 14.8526 | 8 | 7 | 7 | 8 | 5 | 7 | 8 | 6 |
| KIAA2013 | Q8IYS2;Q8 | 2.16E-05 | 5.50E-06 | 0.0610898 | | 0.264475 | 0.643152 | | 16.4008 | 16.4716 | 16.4429 | 16.2013 | 16.4681 | 16.2807 | 16.5984 | 16.3384 | 8 | 8 | 8 | 8 | 6 | 8 | 8 | 7 |
| ANKHD1 | Q8IWZ3;Q8 | 1.43E-08 | 5.09E-09 | 0.0613464 | | 0.281118 | 0.677298 | | 14.9239 | 14.9754 | 14.7962 | 15.0199 | 14.8 | 14.651 | 14.9076 | 14.8075 | 9 | 9 | 9 | 12 | 5 | 8 | 7 | 11 |
| MTMR14 | Q8NCE2;Q8 | 1.67E-05 | 4.30E-06 | 0.0614958 | | 0.260232 | 0.634369 | | 15.786 | 15.5263 | 15.6209 | 15.6066 | 15.7111 | 15.5761 | 15.3627 | 15.658 | 4 | 5 | 5 | 6 | 4 | 4 | 4 | 4 |
| MAN2B2 | Q9Y2E5;Q9 | 6.88E-11 | 3.41E-11 | 0.0615238 | | 0.17217 | 0.443845 | | 16.0612 | 16.0104 | 16.0873 | 15.6745 | 16.224 | 16.1354 | 16.0959 | 15.8274 | 10 | 10 | 10 | 11 | 10 | 13 | 11 | 10 |
| UROS | P10746 | 1.47E-15 | 2.40E-15 | 0.0615315 | | 0.256962 | 0.627578 | | 15.3432 | 15.2317 | 15.3022 | 15.047 | 15.4285 | 15.1115 | 15.216 | 15.3513 | 9 | 10 | 9 | 8 | 9 | 8 | 9 | 9 |
| TBCE | Q15813 | 5.26E-16 | 9.60E-16 | 0.0617972 | | 0.184002 | 0.470472 | | 16.2529 | 16.316 | 16.2513 | 16.3564 | 16.5237 | 16.0161 | 16.0639 | 16.0979 | 22 | 23 | 24 | 22 | 21 | 21 | 26 | 22 |
| EIF4G2 | P78344 | 4.53E-13 | 3.59E-13 | 0.0618032 | | 0.433633 | 0.972324 | | 18.1641 | 18.1672 | 18.1697 | 18.2453 | 18.1374 | 17.9587 | 18.0538 | 18.1308 | 40 | 39 | 40 | 40 | 40 | 41 | 39 | 40 |
| POFUT1 | Q9H488 | 1.03E-17 | 3.29E-17 | 0.0619919 | | 0.247747 | 0.608337 | | 17.1164 | 17.1168 | 17.1483 | 17.0953 | 16.8091 | 17.2862 | 17.0479 | 17.0875 | 13 | 13 | 12 | 13 | 12 | 13 | 12 | 12 |
| MLLT1 | Q03111 | 1.62E-19 | 9.26E-19 | 0.0620301 | | 0.48177 | 1.06037 | | 15.2728 | 15.2353 | 15.2978 | 15.1683 | 15.3663 | 15.1166 | 15.1692 | 15.2128 | 2 | 4 | 4 | 3 | 1 | 1 | 2 | 1 |
| BRD8 | Q9H0E9;Q9 | 0.000155 | 3.59E-05 | 0.0620924 | | 0.09993 | 0.272386 | | 14.9063 | 14.3522 | 14.6266 | 14.8461 | 14.8141 | 14.3137 | 14.1207 | 14.7367 | 4 | 5 | 4 | 4 | 2 | 1 | 1 | 3 |
| PDS5A | Q29RF7 | 2.94E-08 | 1.01E-08 | 0.0622021 | | 0.303196 | 0.721901 | | 15.5649 | 15.636 | 15.5851 | 15.6527 | 15.5738 | 15.297 | 15.5151 | 15.6271 | 19 | 22 | 22 | 20 | 17 | 14 | 18 | 18 |
| NUBP1 | P53384;P5 | 1.98E-20 | 1.61E-19 | 0.0624177 | | 0.279814 | 0.67464 | | 16.5849 | 16.4601 | 16.4544 | 16.3746 | 16.6318 | 16.4881 | 16.4568 | 16.2355 | 6 | 9 | 9 | 8 | 6 | 8 | 6 | 6 |
| PMPCB | O75439 | 3.87E-11 | 2.00E-11 | 0.0624367 | | 0.568082 | 1.21404 | | 16.9691 | 16.9622 | 16.9724 | 16.9585 | 16.9734 | 16.8451 | 16.7835 | 16.9668 | 17 | 16 | 16 | 17 | 16 | 16 | 15 | 16 |
| RPL36 | Q9Y3U8 | 0.000279 | 6.22E-05 | 0.0624701 | | 0.2276 | 0.565702 | | 21.8354 | 21.8749 | 21.9137 | 22.0896 | 21.6072 | 21.8639 | 21.7013 | 21.7991 | 3 | 3 | 3 | 3 | 3 | 3 | 3 | 3 |
| POLR2A | P24928 | 2.07E-23 | 4.39E-22 | 0.0624959 | | 0.692049 | 1.42798 | | 15.489 | 15.442 | 15.4346 | 15.3861 | 15.4375 | 15.2935 | 15.3683 | 15.4782 | 36 | 39 | 42 | 32 | 21 | 25 | 30 | 31 |
| EIF3B | P55884;P5 | 8.00E-19 | 3.59E-18 | 0.0626895 | | 0.236196 | 0.58399 | | 18.1777 | 18.2407 | 18.1836 | 18.4301 | 17.9442 | 18.0999 | 18.0844 | 18.1313 | 35 | 35 | 35 | 36 | 32 | 36 | 36 | 35 |
| RRAGA | Q7L523 | 1.75E-13 | 1.56E-13 | 0.0626905 | | 0.316236 | 0.747895 | | 17.1555 | 17.199 | 17.1894 | 17.2851 | 16.9198 | 17.1345 | 17.1988 | 17.0549 | 10 | 11 | 10 | 11 | 11 | 10 | 10 | 11 |
| SNRPB2 | P08579 | 1.62E-14 | 1.93E-14 | 0.0627522 | | 0.224364 | 0.558777 | | 17.0342 | 17.167 | 17.1176 | 17.1702 | 16.7532 | 16.9758 | 17.1834 | 17.1351 | 6 | 6 | 6 | 6 | 6 | 6 | 6 | 7 |
| WASH3P;WASH2P | C4AMC7;Q | 1.93E-08 | 6.77E-09 | 0.0628085 | | 0.207757 | 0.522893 | | 17.0234 | 16.9817 | 16.9481 | 16.9002 | 16.6421 | 17.0819 | 17.1424 | 16.8413 | 12 | 12 | 11 | 12 | 9 | 10 | 11 | 10 |
| RWDD1 | Q9H446;Q9 | 3.22E-12 | 2.06E-12 | 0.0629292 | | 0.261389 | 0.636768 | | 15.1448 | 15.064 | 15.1585 | 15.0706 | 14.8128 | 15.2585 | 15.119 | 15.0365 | 5 | 6 | 6 | 6 | 5 | 5 | 5 | 5 |
| XPO4 | Q9C0E2 | 1.21E-14 | 1.49E-14 | 0.0629896 | | 0.496018 | 1.08608 | | 15.445 | 15.4126 | 15.4288 | 15.3327 | 15.4101 | 15.4858 | 15.2252 | 15.3753 | 16 | 19 | 18 | 18 | 11 | 13 | 15 | 18 |
| HSD17B11 | Q8NBQ5 | 8.13E-09 | 2.99E-09 | 0.0630282 | | 0.40045 | 0.910429 | | 17.3874 | 17.4227 | 17.4049 | 17.2106 | 17.2343 | 17.4803 | 17.3817 | 17.4029 | 12 | 11 | 12 | 11 | 11 | 11 | 12 | 11 |
| IWS1 | Q96ST2;Q9 | 7.93E-11 | 3.86E-11 | 0.0631822 | | 0.277592 | 0.670103 | | 15.9589 | 15.9095 | 15.8805 | 15.8944 | 15.7444 | 15.9478 | 15.6471 | 16.0318 | 9 | 9 | 9 | 8 | 5 | 9 | 9 | 9 |
| LACTB2 | Q53H82 | 9.56E-07 | 2.78E-07 | 0.0632004 | | 0.189838 | 0.483475 | | 15.9005 | 15.8827 | 15.916 | 15.965 | 15.7413 | 16.1548 | 15.7 | 15.6216 | 8 | 9 | 10 | 8 | 8 | 9 | 9 | 10 |
| OLA1 | Q9NTK5 | 1.44E-08 | 5.14E-09 | 0.063306 | | 0.309959 | 0.735412 | | 17.6922 | 17.6908 | 17.7034 | 17.4984 | 17.5658 | 17.6659 | 17.867 | 17.5637 | 20 | 22 | 22 | 20 | 20 | 21 | 22 | 20 |
| ESD | P10768 | 7.29E-09 | 2.70E-09 | 0.0634219 | | 0.250863 | 0.61486 | | 19.4093 | 19.4701 | 19.4114 | 19.3696 | 19.576 | 19.4484 | 19.1109 | 19.3295 | 14 | 11 | 13 | 11 | 11 | 11 | 11 | 12 |
| DCUN1D5 | Q9BTE7 | 4.96E-05 | 1.22E-05 | 0.0635035 | | 0.579383 | 1.23383 | | 14.5963 | 14.4718 | 14.4265 | 14.4498 | 14.4888 | 14.4636 | 14.333 | 14.4385 | 6 | 4 | 5 | 5 | 1 | 3 | 5 | 5 |
| TMX1 | Q9H3N1 | 1.14E-15 | 1.91E-15 | 0.063648 | | 1.00299 | 1.94815 | | 17.6332 | 17.6924 | 17.7014 | 17.6391 | 17.5351 | 17.5941 | 17.6466 | 17.6452 | 11 | 11 | 12 | 11 | 9 | 12 | 11 | 11 |
| LRRC59 | Q96AG4 | 6.09E-08 | 2.01E-08 | 0.0637001 | | 0.667175 | 1.38555 | | 20.2448 | 20.1596 | 20.1969 | 20.1064 | 20.0811 | 20.2608 | 20.1201 | 20.1153 | 15 | 15 | 14 | 15 | 14 | 15 | 13 | 15 |
| LGALS1 | P09382 | 1.90E-09 | 7.56E-10 | 0.0638261 | | 0.249695 | 0.612417 | | 23.9439 | 23.7116 | 23.9384 | 23.6858 | 24.0449 | 23.8338 | 23.7148 | 23.7249 | 13 | 13 | 13 | 13 | 14 | 14 | 14 | 14 |
| GTF2F1 | P35269 | 6.92E-15 | 9.14E-15 | 0.0638613 | | 0.146699 | 0.385239 | | 15.9178 | 15.9217 | 15.997 | 15.9188 | 16.1527 | 15.4155 | 15.9776 | 15.9435 | 8 | 8 | 8 | 8 | 9 | 8 | 8 | 8 |
| PSMB3 | P49720 | 1.56E-06 | 4.43E-07 | 0.0638945 | | 0.199535 | 0.504899 | | 19.0032 | 19.0349 | 18.9762 | 18.965 | 18.6016 | 19.1756 | 19.0281 | 18.934 | 9 | 9 | 8 | 9 | 9 | 9 | 8 | 8 |
| LRCH3 | Q96II8;Q96 | 4.40E-10 | 1.90E-10 | 0.0640155 | | 0.174945 | 0.450123 | | 15.6363 | 15.6369 | 15.6477 | 15.6707 | 15.8801 | 15.2225 | 15.5459 | 15.5623 | 15 | 14 | 16 | 14 | 11 | 13 | 15 | 13 |
| ATE1 | O95260-2 | 0.00012 | 2.80E-05 | 0.064297 | | 0.173339 | 0.446491 | | 15.7981 | 15.7396 | 15.6176 | 15.3043 | 15.6232 | 15.8345 | 15.8964 | 15.6122 | 7 | 9 | 9 | 9 | 7 | 9 | 10 | 9 |
| CDC40 | O60508 | 6.25E-12 | 3.79E-12 | 0.0644408 | | 0.660667 | 1.37441 | | 15.7806 | 15.6748 | 15.696 | 15.5663 | 15.7497 | 15.6171 | 15.6685 | 15.6617 | 8 | 7 | 6 | 9 | 7 | 8 | 8 | 6 |
| SRP9 | P49458 | 6.65E-06 | 1.78E-06 | 0.0645046 | | 0.078662 | 0.218436 | | 18.6607 | 18.3131 | 18.878 | 18.9527 | 18.1403 | 18.0526 | 18.5777 | 19.0404 | 4 | 4 | 4 | 4 | 4 | 4 | 4 | 4 |
| SNRNP70 | P08621 | 3.88E-07 | 1.19E-07 | 0.064555 | + | 1.84446 | 3.41067 | | 18.025 | 17.9966 | 17.9874 | 17.9836 | 17.9439 | 17.9075 | 17.9332 | 17.9241 | 14 | 14 | 14 | 14 | 13 | 14 | 13 | 13 |
| LEMD2 | Q8NC56 | 1.40E-17 | 4.25E-17 | 0.0647725 | | 0.26573 | 0.645743 | | 16.6988 | 16.7952 | 16.8317 | 16.5263 | 16.7165 | 16.9205 | 16.8067 | 16.5823 | 17 | 16 | 16 | 16 | 12 | 17 | 16 | 15 |
| RBM33 | Q96EV2 | 0.008462 | 0.001465 | 0.0648337 | | 0.16718 | 0.432506 | | 14.1057 | 14.1165 | 13.995 | 13.8134 | 14.1783 | 13.8976 | 14.3535 | 13.7951 | 4 | 4 | 4 | 5 | 3 | 4 | 3 | 2 |
| AMPD2 | Q01433 | 2.13E-13 | 1.86E-13 | 0.064894 | | 0.293009 | 0.701416 | | 17.124 | 17.1663 | 17.1266 | 17.0966 | 16.9319 | 17.3051 | 17.1045 | 16.9322 | 32 | 30 | 32 | 29 | 28 | 32 | 30 | 30 |
| NAAA | Q02083;Q0 | 0.010092 | 0.00173 | 0.0651343 | | 0.274776 | 0.664341 | | 15.1083 | 14.8582 | 15.067 | 14.9895 | 14.9046 | 14.7772 | 15.1438 | 14.915 | 4 | 5 | 5 | 4 | 2 | 4 | 5 | 3 |
| SPPL2B | Q8TCT7;Q8 | 2.97E-13 | 2.49E-13 | 0.065512 | | 0.194929 | 0.494751 | | 15.4046 | 15.4384 | 15.5511 | 15.5865 | 15.2101 | 15.1281 | 15.58 | 15.4914 | 6 | 7 | 5 | 7 | 4 | 5 | 6 | 6 |

| Gene names | UniProt IDs | Protein Group P-value | Protein Group Q-value | Log2 Difference NUP214 vs Controls (n=5) | p<0.05 | -log10 p-value | T-test Test statistic | NPC Localization | Log2 MS2_quantity_NUP214_1 | Log2 MS2_quantity_NUP214_2 | Log2 MS2_quantity_NUP214_3 | Log2 MS2_quantity_Control_1 | Log2 MS2_quantity_Control_2 | Log2 MS2_quantity_Control_3 | Log2 MS2_quantity_Control_4 | Log2 MS2_quantity_Control_5 | Number Peptides for Quantitation NUP214_1 | Number Peptides for Quantitation NUP214_2 | Number Peptides for Quantitation NUP214_3 | Number Peptides for Quantitation Control_1 | Number Peptides for Quantitation Control_2 | Number Peptides for Quantitation Control_3 | Number Peptides for Quantitation Control_4 | Number Peptides for Quantitation Control_5 |
|---|---|---|---|---|---|---|---|---|---|---|---|---|---|---|---|---|---|---|---|---|---|---|---|---|
| EIF2D | P41214 | 8.21E-12 | 4.86E-12 | 0.0656335 | | 0.303949 | 0.723408 | | 15.9713 | 15.9934 | 15.9604 | 15.761 | 15.8325 | 16.0475 | 16.0966 | 15.8095 | 15 | 16 | 15 | 15 | 10 | 14 | 13 | 12 |
| DNAJC17 | Q9NVM6 | 5.38E-12 | 3.30E-12 | 0.0661992 | | 0.649316 | 1.35495 | | 15.5176 | 15.5072 | 15.4682 | 15.4241 | 15.4644 | 15.3104 | 15.4286 | 15.5299 | 9 | 8 | 9 | 9 | 5 | 8 | 8 | 8 |
| ARFGEF1 | Q9Y6D6 | 1.95E-20 | 1.61E-19 | 0.0662331 | | 1.07015 | 2.05975 | | 15.854 | 15.8904 | 15.8789 | 15.8577 | 15.8422 | 15.8381 | 15.7498 | 15.7532 | 41 | 45 | 44 | 39 | 33 | 35 | 39 | 37 |
| VASP | P50552 | 1.96E-07 | 6.18E-08 | 0.066279 | | 0.357521 | 0.828651 | | 18.0975 | 18.1343 | 18.0763 | 18.1875 | 18.076 | 18.1226 | 17.891 | 17.905 | 14 | 16 | 15 | 15 | 15 | 16 | 16 | 13 |
| AP2S1 | P53680 | 2.11E-14 | 2.43E-14 | 0.0665723 | | 0.239901 | 0.591828 | | 19.0475 | 19.2443 | 19.1456 | 19.3403 | 18.86 | 19.0031 | 19.1104 | 19.082 | 6 | 5 | 5 | 5 | 5 | 5 | 5 | 6 |
| FBXL8 | Q96CD0 | 1.11E-08 | 4.01E-09 | 0.0666396 | | 0.431863 | 0.969049 | | 15.7773 | 15.775 | 15.6626 | 15.5016 | 15.776 | 15.6831 | 15.7387 | 15.6589 | 6 | 6 | 6 | 6 | 5 | 7 | 6 | 7 |
| RRP9 | O43818 | 6.91E-06 | 1.84E-06 | 0.0667156 | | 0.546381 | 1.17584 | | 16.2484 | 16.1065 | 16.1974 | 16.0347 | 16.077 | 16.0707 | 16.2226 | 16.1819 | 12 | 11 | 12 | 11 | 10 | 9 | 11 | 11 |
| RAN | P62826 | 5.03E-07 | 1.52E-07 | 0.0668092 | | 0.292455 | 0.700297 | | 20.6426 | 20.7403 | 20.666 | 20.817 | 20.7063 | 20.5777 | 20.4023 | 20.5774 | 13 | 13 | 13 | 13 | 12 | 13 | 13 | 13 |
| ILF2 | Q12905 | 6.72E-10 | 2.84E-10 | 0.0669727 | | 0.765547 | 1.55231 | | 19.1525 | 19.1467 | 19.1632 | 19.0239 | 19.2015 | 19.0343 | 19.0665 | 19.1096 | 16 | 16 | 16 | 16 | 15 | 16 | 16 | 17 |
| GATD1 | Q8NB37 | 6.10E-08 | 2.02E-08 | 0.0669815 | | 0.20503 | 0.516941 | | 17.4586 | 17.473 | 17.5369 | 17.6133 | 17.6733 | 17.3017 | 17.162 | 17.3623 | 6 | 5 | 5 | 4 | 5 | 5 | 5 | 5 |
| OTUD6B | Q8N6M0;Q | 7.81E-10 | 3.27E-10 | 0.0672373 | | 0.472048 | 1.04274 | | 15.4707 | 15.5829 | 15.4922 | 15.5538 | 15.5389 | 15.3351 | 15.451 | 15.3614 | 3 | 3 | 3 | 3 | 4 | 3 | 3 | 3 |
| EXOSC10 | Q01780 | 4.26E-19 | 2.14E-18 | 0.0673114 | | 0.241895 | 0.596034 | | 15.5538 | 15.6074 | 15.5533 | 15.7335 | 15.4918 | 15.2647 | 15.3906 | 15.6404 | 24 | 24 | 25 | 21 | 19 | 20 | 16 | 23 |
| FECH | P22830;P2 | 4.02E-07 | 1.22E-07 | 0.0674358 | | 0.094996 | 0.260031 | | 15.6657 | 15.6958 | 15.6408 | 15.0516 | 15.6687 | 15.1536 | 15.828 | 15.2979 | 12 | 11 | 11 | 11 | 8 | 12 | 12 | 9 |
| CST3 | P01034 | 2.62E-11 | 1.41E-11 | 0.0675444 | | 0.713066 | 1.46369 | | 17.2215 | 17.3542 | 17.394 | 17.2106 | 17.2932 | 17.3005 | 17.2646 | 17.2097 | 4 | 4 | 4 | 4 | 3 | 4 | 4 | 4 |
| UBXN1 | Q04323 | 3.67E-10 | 1.60E-10 | 0.0678267 | | 0.299149 | 0.713781 | | 17.3942 | 17.2952 | 17.3176 | 17.1433 | 17.0749 | 17.3662 | 17.4474 | 17.3073 | 9 | 10 | 11 | 11 | 9 | 10 | 9 | 10 |
| PDAP1 | Q13442 | 1.56E-09 | 6.30E-10 | 0.0679284 | | 0.550117 | 1.18244 | | 19.0939 | 19.1781 | 19.0727 | 19.0009 | 19.0213 | 19.1937 | 19.053 | 18.9659 | 7 | 7 | 7 | 6 | 6 | 7 | 7 | 7 |
| LHPP | Q9H008 | 1.30E-05 | 3.36E-06 | 0.0681304 | | 0.098549 | 0.268938 | | 15.6397 | 15.5983 | 15.4851 | 15.292 | 15.4595 | 15.8294 | 16.0017 | 14.9485 | 6 | 6 | 5 | 6 | 4 | 5 | 6 | 3 |
| NT5DC2 | Q9H857-2 | 0.000128 | 2.99E-05 | 0.0682547 | | 0.132699 | 0.352215 | | 16.1541 | 16.1917 | 16.1483 | 16.605 | 15.8825 | 16.2171 | 15.7988 | 15.9787 | 18 | 18 | 19 | 19 | 16 | 18 | 16 | 15 |
| THUMPD1 | Q9NXG2 | 0.000307 | 6.80E-05 | 0.0682827 | | 0.527663 | 1.14267 | | 15.855 | 15.8545 | 15.8124 | 15.715 | 15.8608 | 15.6266 | 15.8246 | 15.8348 | 11 | 12 | 12 | 11 | 9 | 13 | 11 | 12 |
| RAB3D | O95716 | 0.000355 | 7.80E-05 | 0.0682866 | | 0.095928 | 0.262372 | | 15.88 | 15.9347 | 15.9714 | 16.2243 | 16.0211 | 15.1067 | 16.0263 | 15.9235 | 3 | 2 | 2 | 3 | 3 | 2 | 2 | 3 |
| USP8 | P40818 | 5.07E-12 | 3.11E-12 | 0.0683592 | | 0.867974 | 1.72371 | | 15.4283 | 15.4529 | 15.4369 | 15.4513 | 15.4003 | 15.2888 | 15.3956 | 15.3189 | 19 | 23 | 18 | 18 | 16 | 21 | 20 | 18 |
| UFL1 | O94874 | 1.07E-13 | 1.00E-13 | 0.0684464 | | 0.206027 | 0.51912 | | 17.5596 | 17.5698 | 17.6058 | 17.2082 | 17.5308 | 17.7201 | 17.7131 | 17.3778 | 30 | 33 | 32 | 29 | 29 | 33 | 30 | 31 |
| UBE2Z | Q9H832 | 2.10E-12 | 1.40E-12 | 0.0685298 | | 0.206457 | 0.520057 | | 16.9349 | 17.0193 | 17.0019 | 17.1333 | 16.6871 | 17.0947 | 16.682 | 16.9872 | 14 | 14 | 13 | 13 | 13 | 12 | 12 | 13 |
| CYFIP1 | Q7L576 | 4.37E-18 | 1.59E-17 | 0.0685944 | + | 1.31675 | 2.47359 | | 17.613 | 17.5856 | 17.5826 | 17.5798 | 17.4745 | 17.5634 | 17.5134 | 17.4946 | 49 | 51 | 50 | 48 | 50 | 49 | 49 | 49 |
| GOSR2 | O14653;O1 | 1.54E-10 | 7.21E-11 | 0.0686785 | | 0.364707 | 0.842486 | | 16.3937 | 16.3375 | 16.314 | 16.3681 | 16.0455 | 16.2991 | 16.3569 | 16.329 | 6 | 7 | 7 | 8 | 7 | 6 | 8 | 8 |
| WDR92 | Q96MX6 | 4.82E-09 | 1.83E-09 | 0.0687304 | | 0.543471 | 1.1707 | | 15.6509 | 15.6697 | 15.6888 | 15.609 | 15.7541 | 15.4979 | 15.5385 | 15.6058 | 11 | 12 | 10 | 10 | 9 | 11 | 11 | 10 |
| DHX15 | O43143 | 1.15E-08 | 4.16E-09 | 0.0687533 | | 0.428042 | 0.96197 | | 17.9088 | 17.9526 | 17.8841 | 17.9765 | 17.8816 | 17.6762 | 17.786 | 17.9117 | 33 | 32 | 31 | 32 | 31 | 32 | 31 | 34 |
| POLR1B | Q9H9Y6;Q | 7.94E-07 | 2.34E-07 | 0.0687553 | | 0.495614 | 1.08535 | | 14.9534 | 14.9222 | 15.005 | 14.8811 | 15.0578 | 14.7844 | 14.8915 | 14.8423 | 8 | 8 | 9 | 10 | 4 | 8 | 6 | 6 |
| INTS13 | Q9NVM9 | 3.24E-08 | 1.11E-08 | 0.068788 | | 0.308965 | 0.733431 | | 14.7728 | 14.611 | 14.6817 | 14.4176 | 14.8095 | 14.5469 | 14.6585 | 14.666 | 11 | 8 | 10 | 8 | 7 | 9 | 9 | 7 |
| NUDT9 | Q9BW91;Q | 6.23E-12 | 3.78E-12 | 0.0688313 | | 0.313502 | 0.742465 | | 16.1403 | 16.1917 | 16.1722 | 16.0653 | 15.9698 | 16.2414 | 16.2786 | 15.9409 | 10 | 11 | 11 | 10 | 10 | 10 | 11 | 10 |
| IPO8 | O15397 | 2.00E-15 | 3.14E-15 | 0.0688416 | | 0.413888 | 0.935623 | | 16.4501 | 16.5208 | 16.5248 | 16.2916 | 16.4946 | 16.3236 | 16.5775 | 16.4614 | 15 | 15 | 15 | 12 | 10 | 11 | 11 | 11 |
| PSMC3 | P17980 | 3.84E-11 | 1.99E-11 | 0.0688772 | | 0.80803 | 1.62361 | | 18.6996 | 18.7151 | 18.6511 | 18.6114 | 18.5446 | 18.7262 | 18.5894 | 18.6269 | 25 | 26 | 26 | 25 | 26 | 25 | 26 | 25 |
| NOTCH2 | Q04721 | 4.05E-09 | 1.55E-09 | 0.0689665 | | 0.087912 | 0.242124 | | 15.3965 | 15.4966 | 15.4216 | 15.9097 | 14.9881 | 15.7583 | 14.805 | 15.3852 | 18 | 18 | 19 | 23 | 14 | 20 | 17 | 18 |
| FLNB | O75369;O7 | 2.94E-23 | 5.62E-22 | 0.0690975 | | 0.22551 | 0.561232 | | 19.5029 | 19.5393 | 19.4976 | 19.8096 | 19.3745 | 19.3469 | 19.3141 | 19.3757 | 141 | 141 | 143 | 140 | 132 | 137 | 137 | 138 |
| NSUN4 | Q96CB9;Q | 0.006465 | 0.001137 | 0.0692234 | | 0.015769 | 0.046988 | | NaN | 14.7981 | 13.8201 | 14.2594 | 16.7081 | 15.3531 | 11.6618 | 13.2171 | NaN | 1 | 3 | 3 | 1 | 2 | 2 | 2 |
| GBF1 | Q92538;Q | 1.80E-14 | 2.12E-14 | 0.0693378 | | 0.674445 | 1.39797 | | 16.7312 | 16.7204 | 16.7118 | 16.699 | 16.5285 | 16.7448 | 16.6183 | 16.6684 | 50 | 50 | 50 | 49 | 45 | 48 | 51 | 49 |
| PCM1 | Q15154;Q | 4.55E-05 | 1.12E-05 | 0.069476 | | 0.263428 | 0.640986 | | 14.6895 | 14.5449 | 14.7213 | 14.7513 | 14.5038 | 14.3647 | 14.536 | 14.7563 | 11 | 13 | 11 | 11 | 6 | 10 | 10 | 10 |
| SUFU | Q9UMX1 | 0.002184 | 0.000424 | 0.0694764 | | 0.073773 | 0.20754 | | 14.3181 | 15.3596 | 15.3669 | 15.204 | 14.5624 | 15.0815 | 14.9337 | NaN | 3 | 3 | 3 | 3 | 2 | 3 | 1 | NaN |
| CTR9 | Q6PD62 | 5.36E-12 | 3.29E-12 | 0.0695901 | | 0.293147 | 0.701695 | | 15.4322 | 15.354 | 15.4081 | 15.3679 | 15.4289 | 15.0918 | 15.2445 | 15.5094 | 18 | 18 | 17 | 16 | 15 | 14 | 16 | 15 |
| CHD2 | O14647;O | 0.005229 | 0.000932 | 0.0695984 | | 0.241834 | 0.595906 | | 14.7729 | 14.7833 | 14.7765 | 14.9696 | 14.8386 | 14.5296 | 14.5198 | 14.6823 | 5 | 5 | 6 | 5 | 5 | 3 | 4 | 4 |
| PPIB | P23284 | 1.66E-06 | 4.70E-07 | 0.0696332 | | 0.13191 | 0.350338 | | 22.0023 | 22.0376 | 21.9923 | 21.6644 | 21.7062 | 22.2133 | 22.3839 | 21.7377 | 18 | 16 | 18 | 17 | 18 | 16 | 17 | 17 |
| POGLUT1 | Q8NBL1 | 2.35E-13 | 2.02E-13 | 0.0696619 | | 0.175484 | 0.451338 | | 16.2673 | 16.3408 | 16.3353 | 16.2479 | 16.4442 | 16.3507 | 16.3787 | 15.8024 | 14 | 13 | 13 | 12 | 11 | 15 | 14 | 13 |
| POLR1A | O95602 | 3.85E-12 | 2.42E-12 | 0.069676 | | 0.7242 | 1.48255 | | 14.712 | 14.5872 | 14.6736 | 14.694 | 14.5511 | 14.5966 | 14.527 | 14.5709 | 19 | 21 | 19 | 19 | 13 | 15 | 18 | 13 |
| SERBP1 | Q8NC51 | 4.18E-08 | 1.41E-08 | 0.0700326 | | 0.750498 | 1.52697 | | 18.6117 | 18.713 | 18.673 | 18.6447 | 18.5061 | 18.6611 | 18.5421 | 18.6253 | 24 | 25 | 24 | 24 | 24 | 24 | 24 | 24 |
| QARS1 | P47897 | 5.15E-17 | 1.32E-16 | 0.0702808 | + | 1.97375 | 3.65654 | | 18.079 | 18.1117 | 18.1039 | 18.0367 | 18.0087 | 17.9972 | 18.0743 | 18.0227 | 44 | 43 | 42 | 42 | 43 | 44 | 44 | 43 |
| LARP1B | Q659C4 | 1.14E-07 | 3.68E-08 | 0.0704034 | | 0.381542 | 0.874662 | | 17.3857 | 17.4468 | 17.4709 | 17.5778 | 17.2601 | 17.3888 | 17.3347 | 17.2588 | 4 | 5 | 4 | 5 | 4 | 5 | 4 | 4 |
| ISCU | Q9H1K1 | 1.02E-09 | 4.21E-10 | 0.070822 | | 0.319617 | 0.754595 | | 15.8384 | 15.7932 | 15.8233 | 15.802 | 15.8254 | 15.901 | 15.7153 | 15.4937 | 6 | 8 | 8 | 8 | 6 | 7 | 7 | 8 |
| SNX1 | Q13596 | 2.19E-15 | 3.40E-15 | 0.0708425 | | 0.61355 | 1.29328 | | 17.753 | 17.8105 | 17.7617 | 17.7665 | 17.63 | 17.7841 | 17.7533 | 17.5873 | 23 | 23 | 23 | 22 | 23 | 21 | 22 | 21 |
| ARMC10 | Q8N2F6;Q | 2.63E-11 | 1.41E-11 | 0.0708729 | | 0.208273 | 0.524018 | | 16.0255 | 16.1109 | 16.166 | 15.66 | 16.1452 | 16.2018 | 16.1509 | 15.9917 | 7 | 7 | 7 | 7 | 7 | 7 | 7 | 6 |
| SEC14L1 | Q92503;Q | 2.58E-13 | 2.20E-13 | 0.0708986 | | 0.136969 | 0.362353 | | 15.5794 | 15.7066 | 15.8344 | 15.3148 | 16.0469 | 15.3478 | 15.6267 | 15.8434 | 8 | 3 | 4 | 6 | 5 | 3 | 5 | 6 |
| RPL36A | P83881 | 0.002318 | 0.000447 | 0.0714114 | | 0.484419 | 1.06516 | | 19.6856 | 19.7988 | 19.8218 | 19.815 | 19.5615 | 19.7261 | 19.7502 | 19.6338 | 5 | 5 | 4 | 4 | 3 | 4 | 5 | 5 |
| ZNF598 | Q86UK7;Q | 2.20E-14 | 2.53E-14 | 0.0714657 | | 0.21919 | 0.547662 | | 15.2966 | 15.3837 | 15.3761 | 15.5093 | 15.0372 | 15.0889 | 15.4768 | 15.2912 | 20 | 17 | 21 | 19 | 14 | 17 | 19 | 17 |
| LAS1L | Q9Y4W2;Q | 6.10E-10 | 2.59E-10 | 0.0716859 | | 0.444637 | 0.992623 | | 16.4907 | 16.558 | 16.572 | 16.4834 | 16.3102 | 16.537 | 16.6112 | 16.401 | 11 | 13 | 12 | 13 | 9 | 12 | 12 | 12 |
| MRPL2 | Q5T653 | 5.48E-06 | 1.47E-06 | 0.0719172 | | 0.327915 | 0.77097 | | 16.6599 | 16.6017 | 16.5643 | 16.5759 | 16.7447 | 16.3773 | 16.3949 | 16.5908 | 6 | 5 | 6 | 5 | 6 | 6 | 5 | 6 |
| UBE2K | P61086 | 2.66E-17 | 7.41E-17 | 0.0719259 | | 0.264607 | 0.643425 | | 17.9988 | 18.0269 | 18.1183 | 18.2161 | 17.7289 | 18.0727 | 17.9501 | 17.9125 | 15 | 15 | 15 | 14 | 15 | 15 | 13 | 14 |
| TMOD3 | Q9NYL9 | 8.51E-09 | 3.13E-09 | 0.072029 | | 1.08716 | 2.08807 | | 18.0482 | 18.0874 | 18.1487 | 18.0662 | 17.9752 | 18.0713 | 18.02 | 17.9809 | 18 | 18 | 19 | 19 | 17 | 18 | 18 | 18 |
| POP7 | O75817 | 3.91E-08 | 1.32E-08 | 0.0722767 | | 0.329671 | 0.774424 | | 16.354 | 16.4364 | 16.3905 | 16.1708 | 16.3677 | 16.1628 | 16.5238 | 16.3817 | 2 | 2 | 2 | 2 | 2 | 2 | 2 | 2 |
| MED27 | Q6P2C8;Q | 0.004121 | 0.000747 | 0.072319 | | 0.020617 | 0.061114 | | 15.4264 | 15.5485 | 14.8556 | 15.9472 | 17.0345 | 12.4149 | NaN | 15.4216 | 1 | 3 | 2 | 3 | 1 | 2 | NaN | 2 |
| NEDD4 | P46934-4 | 7.01E-10 | 2.95E-10 | 0.0724172 | | 0.405306 | 0.919554 | | 16.0672 | 16.0291 | 16.0022 | 16.0557 | 16.1228 | 15.8165 | 15.8536 | 15.9534 | 21 | 22 | 21 | 18 | 19 | 20 | 21 | 21 |
| TOP1 | P11387 | 2.50E-18 | 9.62E-18 | 0.0724518 | | 0.207707 | 0.522784 | | 16.7126 | 16.7251 | 16.7094 | 17.0149 | 16.6091 | 16.3954 | 16.5237 | 16.673 | 23 | 27 | 26 | 27 | 23 | 23 | 23 | 24 |
| USE1 | Q9NZ43 | 1.78E-10 | 8.21E-11 | 0.0724925 | | 0.841481 | 1.67953 | | 16.0032 | 16.0766 | 16.0401 | 16.0057 | 15.9534 | 15.9636 | 16.0479 | 15.8669 | 6 | 7 | 8 | 7 | 6 | 6 | 6 | 7 |
| RUVBL2 | Q9Y230 | 4.74E-14 | 4.95E-14 | 0.0726753 | | 0.760045 | 1.54305 | | 18.455 | 18.4795 | 18.4234 | 18.3122 | 18.4813 | 18.3825 | 18.2993 | 18.4246 | 28 | 27 | 27 | 25 | 26 | 25 | 27 | 29 |
| RNF149 | Q8NC42 | 0.000158 | 3.65E-05 | 0.0727019 | | 0.097735 | 0.2669 | | 15.4536 | 15.735 | 15.7224 | 16.2283 | 15.2645 | 15.4192 | 15.132 | 15.7776 | 5 | 5 | 7 | 7 | 4 | 6 | 5 | 7 |
| GTPBP2 | Q9BX10;Q | 0.012561 | 0.00213 | 0.072764 | | 0.019355 | 0.057449 | | 13.8092 | 14.1879 | 13.9382 | 13.5808 | 16.5064 | 11.3156 | NaN | 14.2199 | 2 | 1 | 1 | 1 | 1 | 2 | NaN | 2 |
| EIF2S2 | P20042 | 7.07E-16 | 1.26E-15 | 0.0731454 | | 0.34595 | 0.806242 | | 17.6688 | 17.6364 | 17.6449 | 17.4247 | 17.488 | 17.8209 | 17.6021 | 17.5488 | 26 | 27 | 27 | 26 | 25 | 27 | 25 | 27 |
| EXOSC4 | Q9NPD3 | 1.93E-06 | 5.43E-07 | 0.0732779 | | 0.223722 | 0.557401 | | 15.8507 | 15.7737 | 15.7077 | 15.7881 | 15.8011 | 15.382 | 15.6102 | 15.9389 | 5 | 4 | 5 | 5 | 5 | 3 | 5 | 4 |
| FKBP15 | Q5T1M5 | 1.22E-19 | 7.31E-19 | 0.073439 | | 0.881018 | 1.74544 | | 15.8877 | 15.8624 | 15.8758 | 15.8304 | 15.7608 | 15.829 | 15.8848 | 15.7045 | 25 | 23 | 25 | 25 | 17 | 23 | 21 | 18 |
| TRIM25 | Q14258 | 1.09E-16 | 2.55E-16 | 0.0735401 | | 0.206239 | 0.519583 | | 17.3086 | 17.3888 | 17.3045 | 17.3208 | 17.2988 | 17.5685 | 16.919 | 17.1952 | 24 | 23 | 24 | 24 | 20 | 25 | 23 | 24 |
| WASHC4 | Q2M389 | 1.74E-14 | 2.07E-14 | 0.0735648 | | 0.421139 | 0.949143 | | 15.9877 | 15.9712 | 15.9884 | 15.8822 | 15.794 | 15.9649 | 16.1044 | 15.7987 | 33 | 31 | 31 | 28 | 22 | 30 | 28 | 29 |
| NANS | Q9NR45 | 8.53E-14 | 8.30E-14 | 0.0735822 | | 0.198507 | 0.502639 | | 18.2852 | 18.257 | 18.2213 | 17.7537 | 18.3559 | 18.3199 | 18.2286 | 18.2465 | 16 | 15 | 15 | 15 | 14 | 15 | 15 | 14 |
| RFC3 | P40938 | 4.64E-13 | 3.66E-13 | 0.0736403 | | 0.207633 | 0.522623 | | 14.0159 | 14.1018 | 14.1191 | 14.0168 | 14.1317 | 13.6223 | 14.0172 | 14.2383 | 9 | 7 | 9 | 9 | 6 | 6 | 8 | 8 |
| PYCR3 | Q53H96 | 1.42E-13 | 1.30E-13 | 0.0740546 | | 0.403597 | 0.916347 | | 16.6754 | 16.6507 | 16.6364 | 16.6995 | 16.4128 | 16.6558 | 16.6765 | 16.4558 | 8 | 8 | 8 | 8 | 8 | 7 | 8 | 8 |
| NKTR | P30414 | 0.001665 | 0.000332 | 0.0744108 | | 0.115064 | 0.312536 | | 13.7038 | 14.1091 | 13.9506 | 13.9524 | 13.8811 | 14.2128 | NaN | 13.3407 | 3 | 3 | 3 | 1 | 2 | 1 | NaN | 1 |
| ATP6V1G1 | O75348 | 4.07E-11 | 2.09E-11 | 0.0744685 | | 0.076277 | 0.21227 | | 17.6814 | 17.7221 | 17.8878 | 18.2226 | 17.4089 | 16.814 | 17.8626 | 18.1382 | 5 | 5 | 5 | 5 | 5 | 5 | 5 | 5 |
| HSPBP1 | Q9NZL4;Q | 2.91E-09 | 1.13E-09 | 0.0745979 | | 0.231404 | 0.573814 | | 18.2629 | 18.1772 | 18.2428 | 17.9744 | 18.3426 | 17.8812 | 18.2114 | 18.3555 | 9 | 10 | 10 | 9 | 9 | 9 | 8 | 10 |
| MAGOH;MAGOHB | P61326;Q | 8.98E-12 | 5.26E-12 | 0.0750058 | | 0.593212 | 1.25796 | | 18.2114 | 18.0414 | 18.201 | 17.9787 | 18.0943 | 18.0234 | 18.1278 | 18.1572 | 7 | 7 | 7 | 7 | 7 | 7 | 7 | 7 |
| MCL1 | Q07820 | 0.003376 | 0.000621 | 0.075098 | | 0.110282 | 0.298013 | | 15.2471 | 15.3921 | 15.5771 | 16.0157 | 15.2237 | 15.1636 | 14.9428 | 15.306 | 2 | 2 | 1 | 1 | 2 | 1 | 2 | 2 |
| PPP1R9B | Q96SB3 | 2.46E-18 | 9.51E-18 | 0.0754335 | | 0.569474 | 1.21648 | | 15.9561 | 15.9338 | 15.9117 | 15.7424 | 15.9251 | 15.9078 | 15.9631 | 15.7538 | 16 | 16 | 16 | 12 | 14 | 14 | 17 | 15 |

| Gene names | UniProt IDs | Protein Group P-value | Protein Group Q-value | Log2 Difference NUP214 vs Controls (n=5) | p<0.05 | -log10 p-value | T-test Test statistic | NPC Localization | Log2 MS2_quantity_NUP214_1 | Log2 MS2_quantity_NUP214_2 | Log2 MS2_quantity_NUP214_3 | Log2 MS2_quantity_Control_1 | Log2 MS2_quantity_Control_2 | Log2 MS2_quantity_Control_3 | Log2 MS2_quantity_Control_4 | Log2 MS2_quantity_Control_5 | Number Peptides for Quantitation NUP214_1 | Number Peptides for Quantitation NUP214_2 | Number Peptides for Quantitation NUP214_3 | Number Peptides for Quantitation Control_1 | Number Peptides for Quantitation Control_2 | Number Peptides for Quantitation Control_3 | Number Peptides for Quantitation Control_4 | Number Peptides for Quantitation Control_5 |
|---|---|---|---|---|---|---|---|---|---|---|---|---|---|---|---|---|---|---|---|---|---|---|---|---|
| ANAPC5 | Q9UJX4 | 1.34E-17 | 4.10E-17 | 0.0754665 | | 0.397895 | 0.905618 | | 14.865 | 14.6038 | 14.7884 | 14.6891 | 14.8263 | 14.6484 | 14.6807 | 14.5399 | 9 | 11 | 10 | 9 | 6 | 9 | 11 | 8 |
| MRPS23 | Q9Y3D9 | 1.35E-08 | 4.84E-09 | 0.0757754 | | 0.488513 | 1.07255 | | 16.7476 | 16.874 | 16.7883 | 16.8721 | 16.8187 | 16.6425 | 16.6564 | 16.648 | 7 | 7 | 7 | 7 | 5 | 7 | 7 | 5 |
| CDKN2AIP | Q9NXV6 | 3.39E-14 | 3.68E-14 | 0.075837 | | 0.636885 | 1.33357 | | 14.3514 | 14.3499 | 14.2949 | 14.2597 | 14.3832 | 14.1409 | 14.2 | 14.2973 | 11 | 12 | 12 | 10 | 7 | 8 | 9 | 10 |
| HDGFL3 | Q9Y3E1 | 0.001711 | 0.000341 | 0.0759007 | | 0.104185 | 0.282968 | | 14.0766 | 14.197 | 14.3418 | 14.8709 | 13.7698 | 14.154 | 14.0015 | 13.8499 | 3 | 3 | 3 | 3 | 1 | 3 | 2 | 3 |
| AKAP17A | Q02040 | 7.05E-08 | 2.31E-08 | 0.0760592 | | 0.341593 | 0.797759 | | 18.238 | 18.2566 | 18.268 | 17.9636 | 18.4137 | 18.1687 | 18.1645 | 18.1802 | 6 | 7 | 8 | 7 | 5 | 5 | 8 | 5 |
| ARPC1A | Q92747 | 2.42E-07 | 7.54E-08 | 0.0761531 | | 0.310612 | 0.736715 | | 18.6055 | 18.6518 | 18.6378 | 18.7855 | 18.4153 | 18.6774 | 18.5179 | 18.3815 | 18 | 18 | 18 | 18 | 18 | 18 | 18 | 18 |
| TMEM167B | Q9NRX6 | 3.64E-07 | 1.12E-07 | 0.0763204 | | 0.119583 | 0.320711 | | 15.3944 | 15.5155 | 15.3849 | 15.9123 | 14.9126 | 15.2441 | 15.1214 | 15.586 | 2 | 2 | 2 | 2 | 1 | 2 | 2 | 2 |
| DDR2 | Q16832 | 5.54E-10 | 2.36E-10 | 0.076527 | | 0.469335 | 1.03781 | | 16.5828 | 16.6464 | 16.5415 | 16.3708 | 16.5729 | 16.5462 | 16.6603 | 16.4183 | 17 | 17 | 15 | 17 | 17 | 16 | 18 | 18 |
| HAUS8 | Q9BT25;Q9 | 0.021472 | 0.003564 | 0.0765405 | | 0.07011 | 0.21321 | | 14.4163 | NaN | 14.4038 | 14.6924 | NaN | NaN | NaN | 13.9746 | 1 | NaN | 3 | 2 | NaN | NaN | NaN | 1 |
| DIAPH1 | O60610 | 1.59E-10 | 7.40E-11 | 0.076656 | | 0.248231 | 0.609352 | | 16.6532 | 16.6447 | 16.635 | 16.8667 | 16.4801 | 16.497 | 16.3158 | 16.6787 | 43 | 46 | 45 | 44 | 44 | 43 | 44 | 44 |
| DOP1B | Q9Y3R5;Q9 | 1.38E-13 | 1.26E-13 | 0.0768398 | | 0.192707 | 0.489839 | | 16.5445 | 16.566 | 16.5684 | 16.6116 | 16.8181 | 16.182 | 16.2534 | 16.549 | 16 | 15 | 14 | 15 | 13 | 11 | 8 | 14 |
| SYNE3 | Q6ZMZ3 | 2.14E-16 | 4.53E-16 | 0.0769683 | | 0.514756 | 1.11967 | | 15.4986 | 15.5205 | 15.5245 | 15.539 | 15.5526 | 15.4237 | 15.4016 | 15.2709 | 28 | 26 | 27 | 28 | 19 | 26 | 24 | 21 |
| ATG5 | Q9H1Y0 | 1.37E-05 | 3.54E-06 | 0.0770257 | | 0.864963 | 1.7187 | | 15.9393 | 15.9633 | 15.9422 | 15.7902 | 15.8155 | 15.9492 | 15.9495 | 15.8518 | 8 | 8 | 9 | 8 | 6 | 8 | 7 | 8 |
| EIF2S3 | P41091 | 4.74E-09 | 1.80E-09 | 0.0770316 | | 0.523083 | 1.13452 | | 18.7368 | 18.7676 | 18.7594 | 18.5401 | 18.7264 | 18.8369 | 18.6755 | 18.6091 | 26 | 26 | 26 | 27 | 26 | 27 | 27 | 26 |
| MFAP1 | P55081 | 3.44E-18 | 1.28E-17 | 0.0772703 | | 0.780748 | 1.57786 | | 15.1704 | 15.0888 | 15.2233 | 15.0622 | 15.0071 | 15.0434 | 15.1658 | 15.1393 | 6 | 6 | 6 | 6 | 5 | 5 | 6 | 5 |
| POGLUT3 | Q7Z4H8 | 4.54E-13 | 3.59E-13 | 0.0773029 | | 0.258666 | 0.63112 | | 17.4831 | 17.4835 | 17.4296 | 17.2314 | 17.1432 | 17.5307 | 17.6368 | 17.3983 | 19 | 18 | 18 | 19 | 18 | 19 | 19 | 19 |
| TXNL1 | O43396 | 1.16E-14 | 1.45E-14 | 0.0773356 | | 0.576367 | 1.22856 | | 18.339 | 18.3711 | 18.313 | 18.0865 | 18.327 | 18.301 | 18.3416 | 18.2625 | 19 | 20 | 19 | 17 | 17 | 19 | 18 | 18 |
| SNX30 | Q5VWJ9 | 3.93E-05 | 9.72E-06 | 0.0774741 | | 0.312899 | 0.741266 | | 15.1547 | 15.239 | 15.0405 | 15.1064 | 14.8413 | 15.18 | 15.2366 | 14.9721 | 6 | 6 | 7 | 6 | 4 | 5 | 8 | 5 |
| RPS24 | P62847;P6 | 1.75E-12 | 1.19E-12 | 0.0775956 | | 0.271889 | 0.658421 | | 20.1166 | 20.14 | 20.3441 | 20.2159 | 20.0534 | 20.2944 | 19.8476 | 20.2019 | 6 | 6 | 6 | 6 | 6 | 6 | 6 | 6 |
| EMD | P50402 | 1.14E-15 | 1.91E-15 | 0.0776623 | | 0.708269 | 1.45555 | | 18.5551 | 18.5687 | 18.5439 | 18.3982 | 18.4339 | 18.5767 | 18.5728 | 18.4096 | 11 | 11 | 11 | 11 | 10 | 11 | 10 | 10 |
| POGZ | Q7Z3K3;Q7 | 9.07E-10 | 3.77E-10 | 0.077749 | | 0.293172 | 0.701745 | | 15.2283 | 15.2425 | 15.1871 | 15.1767 | 15.4312 | 14.9453 | 15.0298 | 15.1248 | 13 | 11 | 12 | 9 | 6 | 10 | 6 | 8 |
| ALDH3B1 | P43353 | 9.99E-06 | 2.62E-06 | 0.0778008 | | 0.097923 | 0.267371 | | 16.2119 | 16.158 | 16.1793 | 15.3012 | 16.3741 | 16.5442 | 16.2857 | 16.0212 | 5 | 5 | 5 | 1 | 6 | 6 | 6 | 5 |
| ISLR | O14498 | 9.48E-11 | 4.56E-11 | 0.0780628 | | 0.087696 | 0.241574 | | 16.2691 | 16.4011 | 16.6017 | 16.4068 | 16.6403 | 16.0623 | 16.9976 | 15.6226 | 7 | 5 | 7 | 7 | 1 | 6 | 7 | 2 |
| HNRNPC | P07910 | 9.57E-05 | 2.27E-05 | 0.0780735 | | 0.914255 | 1.80074 | | 20.737 | 20.7764 | 20.7521 | 20.6999 | 20.7609 | 20.5641 | 20.6768 | 20.6838 | 19 | 20 | 19 | 18 | 20 | 17 | 17 | 19 |
| SLTM | Q9NWH9 | 7.70E-06 | 2.05E-06 | 0.0781644 | | 1.28332 | 2.41692 | | 17.188 | 17.2029 | 17.1555 | 17.1364 | 17.117 | 17.0704 | 17.1619 | 17.034 | 9 | 9 | 11 | 10 | 8 | 10 | 10 | 9 |
| USP25 | Q9UHP3 | 5.88E-06 | 1.58E-06 | 0.078166 | | 0.268856 | 0.652187 | | 15.753 | 15.8328 | 15.7605 | 15.9171 | 15.4157 | 15.8288 | 15.6004 | 15.7575 | 11 | 8 | 12 | 11 | 7 | 9 | 12 | 7 |
| CPSF1 | Q10570 | 5.17E-10 | 2.22E-10 | 0.0782688 | | 0.369742 | 0.852145 | | 15.805 | 15.8389 | 15.8174 | 15.7024 | 15.9227 | 15.5115 | 15.749 | 15.8252 | 26 | 24 | 23 | 24 | 18 | 20 | 18 | 20 |
| NFX1 | Q12986 | 0.003228 | 0.000597 | 0.0789589 | | 0.18164 | 0.465186 | | 13.1531 | 13.3313 | 13.2498 | 12.8048 | 13.4362 | 13.1763 | 13.431 | 12.9805 | 6 | 6 | 9 | 4 | 4 | 5 | 5 | 7 |
| RRP1 | P56182 | 2.56E-12 | 1.68E-12 | 0.0791507 | | 0.556955 | 1.19449 | | 15.3696 | 15.2742 | 15.3806 | 15.2165 | 15.4135 | 15.3231 | 15.1727 | 15.1858 | 8 | 8 | 10 | 7 | 7 | 10 | 9 | 7 |
| SRPRB | Q9Y5M8 | 5.45E-15 | 7.44E-15 | 0.0791737 | | 0.278548 | 0.672057 | | 18.1786 | 18.2258 | 18.2112 | 18.1064 | 17.8895 | 18.0626 | 18.4334 | 18.1383 | 15 | 15 | 15 | 15 | 15 | 15 | 16 | 15 |
| SDSL | Q96GA7 | 0.001879 | 0.00037 | 0.0795303 | | 0.098395 | 0.268551 | | 14.9994 | 14.8032 | 14.7922 | 14.5443 | 14.4967 | 15.4773 | 15.1081 | 14.3006 | 7 | 4 | 6 | 4 | 1 | 7 | 6 | 3 |
| MRPS2 | Q9Y399 | 2.46E-05 | 6.20E-06 | 0.079652 | | 0.585181 | 1.24396 | | 16.3568 | 16.4249 | 16.3587 | 16.4256 | 16.1484 | 16.2753 | 16.3608 | 16.2922 | 7 | 7 | 6 | 6 | 5 | 6 | 6 | 6 |
| MT-CYB | P00156 | 0.00027 | 6.02E-05 | 0.0797189 | | 0.18868 | 0.480903 | | 17.6371 | 17.3572 | 17.5149 | 17.7717 | 17.1063 | 17.4208 | 17.563 | 17.2549 | 1 | 1 | 1 | 2 | 1 | 1 | 1 | 1 |
| RBM22 | Q9NW64;C | 1.18E-08 | 4.23E-09 | 0.0798686 | | 0.862344 | 1.71433 | | 16.0779 | 16.068 | 16.0962 | 16.042 | 16.0697 | 15.8879 | 15.9532 | 16.0514 | 6 | 5 | 5 | 6 | 4 | 5 | 6 | 5 |
| NDUFV1 | P49821;P4 | 3.08E-17 | 8.44E-17 | 0.080368 | | 0.473878 | 1.04606 | | 17.377 | 17.4212 | 17.4015 | 17.273 | 17.2969 | 17.4723 | 17.4117 | 17.1437 | 18 | 16 | 18 | 18 | 16 | 17 | 17 | 16 |
| SLC25A1 | P53007 | 0.000199 | 4.54E-05 | 0.0803805 | | 0.845102 | 1.68557 | | 18.4858 | 18.426 | 18.3682 | 18.2764 | 18.4426 | 18.2933 | 18.3855 | 18.3337 | 9 | 9 | 11 | 10 | 9 | 9 | 8 | 9 |
| DIP2C | Q9Y2E4 | 5.62E-07 | 1.68E-07 | 0.0804413 | | 0.713846 | 1.46501 | | 14.4331 | 14.3138 | 14.2445 | 14.2287 | 14.1608 | 14.2408 | 14.3063 | 14.3136 | 8 | 9 | 9 | 8 | 7 | 7 | 9 | 6 |
| HUWE1 | Q7Z6Z7;Q7 | 4.72E-14 | 4.93E-14 | 0.0805135 | | 0.730461 | 1.49314 | | 17.0062 | 17.0068 | 16.9943 | 16.8047 | 16.9676 | 17.0449 | 16.8831 | 16.9095 | 97 | 97 | 95 | 88 | 82 | 86 | 88 | 89 |
| CTDNEP1 | O95476 | 2.91E-14 | 3.22E-14 | 0.0805164 | | 0.569789 | 1.21703 | | 16.6501 | 16.5191 | 16.568 | 16.3594 | 16.6099 | 16.4678 | 16.5829 | 16.4729 | 2 | 2 | 2 | 3 | 3 | 3 | 3 | 2 |
| NCBP3 | Q53F19 | 4.41E-06 | 1.20E-06 | 0.080529 | | 0.41533 | 0.938317 | | 15.7963 | 15.7126 | 15.6596 | 15.4279 | 15.6846 | 15.744 | 15.5975 | 15.7575 | 7 | 9 | 6 | 6 | 8 | 7 | 6 | 6 |
| TRA2B | P62995;P6 | 8.44E-05 | 2.01E-05 | 0.0805464 | | 0.933945 | 1.83347 | | 18.6136 | 18.7467 | 18.6728 | 18.5709 | 18.6695 | 18.5949 | 18.6296 | 18.5208 | 8 | 8 | 8 | 7 | 7 | 7 | 6 | 8 |
| MAN1A1 | P33908 | 1.23E-14 | 1.52E-14 | 0.0809111 | | 0.101882 | 0.277248 | | 15.8895 | 15.8379 | 15.8728 | 16.2918 | 15.9361 | 15.7215 | 14.9904 | 15.9892 | 13 | 12 | 12 | 12 | 9 | 12 | 6 | 9 |
| YIPF3 | Q9GZM5 | 1.25E-05 | 3.24E-06 | 0.0809315 | | 0.154597 | 0.403607 | | 16.7045 | 16.7057 | 16.7842 | 16.8438 | 16.0706 | 16.7867 | 16.665 | 16.8867 | 4 | 5 | 4 | 5 | 5 | 5 | 5 | 5 |
| NDUFA12 | Q9UI09 | 1.93E-08 | 6.76E-09 | 0.0809525 | | 0.375808 | 0.86374 | | 17.5005 | 17.4576 | 17.2917 | 17.3573 | 17.2204 | 17.4286 | 17.4975 | 17.1746 | 5 | 6 | 5 | 5 | 5 | 6 | 5 | 5 |
| COL1A1 | P02452 | 1.83E-13 | 1.62E-13 | 0.0809536 | | 0.053136 | 0.151114 | | 19.951 | 19.97 | 19.9794 | 19.0841 | 19.3323 | 21.1315 | 20.5412 | 19.34 | 61 | 61 | 60 | 56 | 56 | 62 | 60 | 58 |
| APOL2 | Q9BQE5 | 2.16E-06 | 6.05E-07 | 0.0810868 | | 0.138472 | 0.365907 | | 16.5043 | 16.4366 | 16.4726 | 16.5861 | 15.9519 | 16.7899 | 16.0397 | 16.5828 | 8 | 9 | 8 | 8 | 6 | 9 | 8 | 7 |
| RRP8 | O43159 | 0.000748 | 0.000157 | 0.0811647 | | 0.169838 | 0.438554 | | 14.9807 | 14.8684 | 14.9055 | 14.8766 | 15.0884 | 14.9597 | 14.3036 | 14.9569 | 9 | 9 | 9 | 9 | 9 | 8 | 8 | 8 |
| SYAP1 | Q96A49 | 2.16E-11 | 1.18E-11 | 0.0812379 | | 0.155895 | 0.406609 | | 16.3009 | 16.2878 | 16.3987 | 16.5027 | 16.5207 | 16.0241 | 15.7749 | 16.4171 | 14 | 15 | 16 | 17 | 14 | 14 | 16 | 15 |
| HSPD1 | P10809 | 5.03E-10 | 2.16E-10 | 0.0813147 | | 0.606116 | 1.28039 | | 19.5277 | 19.5177 | 19.4717 | 19.5018 | 19.4704 | 19.3336 | 19.2925 | 19.5236 | 46 | 45 | 44 | 44 | 43 | 44 | 42 | 45 |
| FKBP10 | Q96AY3 | 3.46E-15 | 5.04E-15 | 0.081325 | | 0.129301 | 0.344109 | | 20.0054 | 20.1031 | 20.0214 | 19.3994 | 19.8683 | 20.3422 | 20.3398 | 19.8601 | 31 | 31 | 30 | 30 | 28 | 31 | 31 | 28 |
| PLCD1 | P51178 | 4.17E-15 | 5.94E-15 | 0.0813429 | | 0.080835 | 0.224032 | | 16.4476 | 16.4101 | 16.4758 | 15.2946 | 16.8196 | 16.5409 | 16.6294 | 16.5313 | 17 | 18 | 17 | 14 | 16 | 16 | 14 | 16 |
| ACOX2 | Q99424 | 1.67E-05 | 4.28E-06 | 0.0813679 | | 0.165056 | 0.427659 | | 15.2272 | 15.1375 | 15.1674 | 15.1498 | 15.5912 | 15.1004 | 14.8484 | 14.79 | 9 | 8 | 8 | 9 | 8 | 8 | 7 | 5 |
| PAPOLA | P51003 | 2.78E-08 | 9.60E-09 | 0.0814054 | | 0.280163 | 0.675353 | | 15.5073 | 15.4805 | 15.5583 | 15.6482 | 15.5966 | 15.1458 | 15.3673 | 15.4119 | 15 | 16 | 15 | 14 | 12 | 12 | 14 | 16 |
| NRBF2 | Q96F24 | 6.95E-13 | 5.22E-13 | 0.0814175 | | 0.289277 | 0.693872 | | 15.0152 | 15.0638 | 15.0937 | 14.8137 | 14.9124 | 15.2922 | 14.838 | 15.0246 | 8 | 9 | 8 | 9 | 4 | 9 | 8 | 8 |
| MRPL21 | Q7Z2W9 | 0.000162 | 3.74E-05 | 0.0815871 | | 0.177154 | 0.455104 | | 16.3162 | 16.4716 | 16.3491 | 16.5381 | 16.5148 | 15.9712 | 15.9785 | 16.4842 | 5 | 4 | 4 | 4 | 4 | 4 | 4 | 5 |
| PRKCA | P17252 | 2.26E-09 | 8.90E-10 | 0.0817136 | | 0.235743 | 0.58303 | | 16.7057 | 16.6653 | 16.5718 | 16.2612 | 16.8242 | 16.5765 | 16.7438 | 16.4236 | 23 | 22 | 22 | 21 | 22 | 21 | 23 | 22 |
| PTPN13 | Q12923-3 | 1.96E-14 | 2.27E-14 | 0.0819352 | | 0.253172 | 0.619683 | | 15.0492 | 15.0247 | 15.0035 | 15.0912 | 14.8202 | 15.1723 | 15.011 | 14.6246 | 38 | 41 | 38 | 41 | 26 | 40 | 30 | 23 |
| ARGLU1 | Q9NWB6 | 1.65E-06 | 4.69E-07 | 0.082141 | | 0.68646 | 1.41847 | | 15.5097 | 15.4973 | 15.4647 | 15.4526 | 15.3582 | 15.3459 | 15.5565 | 15.3288 | 6 | 5 | 5 | 5 | 4 | 5 | 5 | 5 |
| SURF4 | O15260 | 2.00E-16 | 4.29E-16 | 0.0821691 | | 1.10884 | 2.12418 | | 20.352 | 20.4019 | 20.3072 | 20.2837 | 20.1971 | 20.264 | 20.352 | 20.261 | 7 | 7 | 7 | 8 | 8 | 8 | 8 | 7 |
| COMMD4 | Q9H0A8 | 6.39E-07 | 1.90E-07 | 0.082458 | | 0.265673 | 0.645624 | | 16.6337 | 16.6789 | 16.7084 | 16.7674 | 16.486 | 16.2669 | 16.743 | 16.6928 | 7 | 7 | 7 | 7 | 7 | 7 | 7 | 7 |
| FADS3 | Q9Y5Q0 | 1.13E-09 | 4.67E-10 | 0.0825225 | | 0.266245 | 0.646805 | | 14.9452 | 15.0632 | 15.0773 | 14.7187 | 14.7328 | 15.1497 | 15.119 | 15.0098 | 6 | 5 | 7 | 5 | 5 | 6 | 6 | 5 |
| SNF8 | Q96H20;Q9 | 9.07E-11 | 4.38E-11 | 0.0828459 | | 0.321382 | 0.758086 | | 16.7928 | 16.6836 | 16.7868 | 16.4715 | 16.7144 | 16.7024 | 16.9298 | 16.5399 | 6 | 6 | 6 | 5 | 5 | 5 | 6 | 6 |
| NSDHL | Q15738 | 1.45E-11 | 8.16E-12 | 0.0829207 | | 0.238583 | 0.589044 | | 17.9627 | 17.9619 | 17.8806 | 18.0897 | 17.9529 | 17.5471 | 17.6648 | 18.0062 | 14 | 15 | 14 | 15 | 14 | 15 | 15 | 15 |
| PPP2R5A | Q15172 | 2.15E-13 | 1.87E-13 | 0.0831385 | | 0.318722 | 0.752823 | | 15.7234 | 15.8261 | 15.6831 | 15.8186 | 15.6051 | 15.5776 | 15.4384 | 15.8657 | 9 | 12 | 11 | 9 | 8 | 9 | 8 | 10 |
| ELMOD2 | Q8IZ81 | 1.30E-12 | 9.11E-13 | 0.0831567 | | 0.5526 | 1.18682 | | 16.4313 | 16.4945 | 16.4788 | 16.2649 | 16.4916 | 16.5126 | 16.2806 | 16.3755 | 10 | 12 | 11 | 10 | 10 | 11 | 11 | 9 |
| TRAPPC5 | Q8IUR0 | 5.89E-07 | 1.76E-07 | 0.0832374 | | 0.087308 | 0.240587 | | 16.5034 | 16.4801 | 16.4716 | 16.7958 | 16.1723 | 15.4871 | 16.676 | 16.8775 | 8 | 8 | 9 | 9 | 8 | 6 | 8 | 9 |
| SNAP29 | O95721 | 5.84E-10 | 2.49E-10 | 0.0835389 | | 0.171411 | 0.442124 | | 16.5723 | 16.482 | 16.4712 | 16.6964 | 16.6043 | 15.9728 | 16.2192 | 16.632 | 12 | 9 | 11 | 13 | 11 | 10 | 11 | 11 |
| MICALL2 | Q8IY33 | 0.000188 | 4.29E-05 | 0.0836721 | | 0.402985 | 0.915197 | | 16.2675 | 16.1967 | 16.2303 | 16.1 | 16.2511 | 16.153 | 15.9217 | 16.3133 | 3 | 3 | 2 | 1 | 3 | 3 | 3 | 3 |
| CIAO1 | O76071 | 6.86E-21 | 6.56E-20 | 0.0838477 | | 0.338352 | 0.791434 | | 16.5334 | 16.5096 | 16.4574 | 16.5414 | 16.174 | 16.6167 | 16.426 | 16.3233 | 9 | 9 | 9 | 9 | 9 | 8 | 10 | 10 |
| SYNE1 | Q8NF91 | 9.68E-23 | 1.62E-21 | 0.083891 | | 0.128157 | 0.341369 | | 15.1424 | 15.0596 | 15.0585 | 14.9389 | 14.4315 | 15.5873 | 15.0545 | 15.0023 | 104 | 106 | 107 | 95 | 44 | 116 | 87 | 91 |
| MRPL39 | Q9NYK5;Q9 | 2.40E-05 | 6.07E-06 | 0.0842758 | | 0.528634 | 1.1444 | | 16.2102 | 16.2787 | 16.2603 | 16.2673 | 16.2849 | 16.094 | 15.9969 | 16.1843 | 12 | 11 | 12 | 11 | 10 | 11 | 10 | 11 |
| POLD2 | P49005 | 3.59E-10 | 1.57E-10 | 0.0846416 | | 0.181424 | 0.464701 | | 15.6135 | 15.6658 | 15.7024 | 15.6647 | 16.0046 | 15.2775 | 15.2868 | 15.6462 | 8 | 8 | 7 | 6 | 4 | 4 | 5 | 5 |
| HARS2 | P49590 | 4.47E-08 | 1.50E-08 | 0.0846783 | | 0.352935 | 0.819789 | | 15.6667 | 15.6335 | 15.6493 | 15.7738 | 15.4817 | 15.6082 | 15.3183 | 15.6439 | 11 | 11 | 11 | 10 | 7 | 7 | 8 | 9 |
| SEC61B | P60468 | 4.40E-06 | 1.20E-06 | 0.0847885 | | 0.283683 | 0.68252 | | 20.3064 | 20.2827 | 20.2325 | 20.3743 | 20.1521 | 19.8488 | 20.2648 | 20.3055 | 3 | 3 | 3 | 3 | 3 | 3 | 3 | 3 |
| GMDS | O60547;O6 | 1.33E-12 | 9.28E-13 | 0.0849185 | | 0.192419 | 0.489199 | | 15.4668 | 15.4856 | 15.4564 | 15.735 | 15.6327 | 15.3379 | 15.1097 | 15.1081 | 15 | 15 | 14 | 13 | 11 | 14 | 13 | 12 |
| EPRS1 | P07814 | 2.82E-19 | 1.52E-18 | 0.085089 | | 0.195767 | 0.496602 | | 18.259 | 18.2747 | 18.2621 | 17.9733 | 17.9771 | 18.6235 | 18.3199 | 18.007 | 84 | 80 | 83 | 80 | 78 | 81 | 80 | 78 |
| SNRPA | P09012 | 2.97E-07 | 9.18E-08 | 0.0850931 | | 0.546724 | 1.17645 | | 17.667 | 17.6729 | 17.7037 | 17.6417 | 17.5704 | 17.4047 | 17.637 | 17.7266 | 9 | 9 | 10 | 10 | 7 | 9 | 8 | 9 |
| GARS1 | P41250 | 3.58E-16 | 6.91E-16 | 0.0851376 | | 0.105931 | 0.287291 | | 19.3825 | 19.4592 | 19.4401 | 19.0821 | 18.9386 | 20.0481 | 19.6781 | 18.9639 | 38 | 36 | 37 | 35 | 36 | 37 | 36 | 36 |

| Gene names | UniProt IDs | Protein Group P-value | Protein Group Q-value | Log2 Difference NUP214 vs Controls (n=5) | p<0.05 | -log10 p-value | T-test Test statistic | NPC Localization | Log2 MS2_quantity_NUP214_1 | Log2 MS2_quantity_NUP214_2 | Log2 MS2_quantity_NUP214_3 | Log2 MS2_quantity_Control_1 | Log2 MS2_quantity_Control_2 | Log2 MS2_quantity_Control_3 | Log2 MS2_quantity_Control_4 | Log2 MS2_quantity_Control_5 | Number Peptides for Quantitation NUP214_1 | Number Peptides for Quantitation NUP214_2 | Number Peptides for Quantitation NUP214_3 | Number Peptides for Quantitation Control_1 | Number Peptides for Quantitation Control_2 | Number Peptides for Quantitation Control_3 | Number Peptides for Quantitation Control_4 | Number Peptides for Quantitation Control_5 |
|---|---|---|---|---|---|---|---|---|---|---|---|---|---|---|---|---|---|---|---|---|---|---|---|---|
| CAP2 | P40123 | 2.96E-16 | 5.87E-16 | 0.0852332 | | 0.179627 | 0.460669 | | 18.3592 | 18.3583 | 18.3675 | 18.0095 | 18.5432 | 18.6128 | 17.9173 | 18.2996 | 17 | 17 | 17 | 16 | 17 | 17 | 16 | 17 |
| NDUFB7 | P17568 | 3.73E-07 | 1.14E-07 | 0.0853464 | | 0.241972 | 0.596197 | | 16.3949 | 16.2491 | 16.3924 | 16.413 | 16.2426 | 16.3047 | 16.4658 | 15.8746 | 4 | 4 | 4 | 4 | 4 | 4 | 4 | 4 |
| ATP5PD | O75947 | 1.15E-09 | 4.72E-10 | 0.0854136 | | 0.080088 | 0.222109 | | 17.6604 | 17.2783 | 18.1774 | 18.2017 | 17.1333 | 16.9486 | 17.7287 | 18.0876 | 13 | 13 | 13 | 13 | 12 | 13 | 13 | 13 |
| NUDCD3 | Q8IVD9 | 1.16E-08 | 4.19E-09 | 0.0854384 | | 0.230902 | 0.572744 | | 16.3183 | 16.3222 | 16.2961 | 16.6174 | 15.9908 | 16.1256 | 16.0776 | 16.3224 | 17 | 17 | 17 | 18 | 16 | 18 | 18 | 18 |
| ABI1 | Q8IZP0;Q8 | 3.06E-12 | 1.96E-12 | 0.0854448 | | 0.571019 | 1.21919 | | 16.8992 | 16.8265 | 16.8913 | 16.9053 | 16.6617 | 16.9091 | 16.7487 | 16.7096 | 14 | 14 | 14 | 14 | 12 | 14 | 14 | 14 |
| SMG5 | Q9UPR3 | 6.05E-05 | 1.47E-05 | 0.0854507 | | 0.252686 | 0.618671 | | 13.8556 | 14.0559 | 13.7007 | 14.1063 | 13.8307 | 13.6362 | 13.6661 | 13.6872 | 4 | 4 | 3 | 5 | 3 | 4 | 3 | 4 |
| POLR1C | O15160 | 4.00E-14 | 4.25E-14 | 0.0856769 | | 0.224986 | 0.560111 | | 15.9548 | 15.9625 | 15.9259 | 15.8558 | 16.0913 | 16.0013 | 15.9309 | 15.431 | 7 | 7 | 7 | 7 | 7 | 7 | 7 | 7 |
| CUL2 | Q13617;Q1 | 2.69E-12 | 1.76E-12 | 0.0857759 | | 0.204133 | 0.514982 | | 16.147 | 16.2378 | 16.1912 | 16.4766 | 15.7754 | 16.2097 | 15.8925 | 16.1769 | 30 | 30 | 31 | 30 | 26 | 27 | 28 | 28 |
| ERG28 | Q9UKR5 | 0.000158 | 3.66E-05 | 0.08578 | | 0.13889 | 0.366895 | | 15.5866 | 15.3726 | 15.6752 | 15.7144 | 15.2622 | 14.9396 | 15.4813 | 15.8976 | 3 | 3 | 3 | 2 | 2 | 1 | 2 | 2 |
| CPSF3 | Q9UKF6 | 1.38E-16 | 3.14E-16 | 0.0858599 | | 0.372553 | 0.857522 | | 15.8385 | 15.9768 | 15.9477 | 15.8492 | 16.0188 | 15.6123 | 15.7521 | 15.9433 | 14 | 17 | 16 | 14 | 9 | 12 | 12 | 13 |
| ADPRH | P54922 | 0.00061 | 0.00013 | 0.0858688 | | 0.096587 | 0.264024 | | 14.0469 | 14.2331 | 14.3137 | 14.3477 | 13.3112 | 14.7826 | 14.0737 | 14.045 | 1 | 1 | 1 | 2 | 1 | 2 | 2 | 1 |
| DIS3 | Q9Y2L1 | 3.64E-16 | 6.97E-16 | 0.0858906 | + | 2.21413 | 4.13576 | | 15.7566 | 15.8353 | 15.8016 | 15.7148 | 15.7206 | 15.7366 | 15.6798 | 15.7079 | 24 | 27 | 30 | 27 | 24 | 26 | 24 | 22 |
| ETFA | P13804 | 8.36E-15 | 1.08E-14 | 0.0864704 | | 1.27173 | 2.39731 | | 18.6412 | 18.6409 | 18.6543 | 18.471 | 18.5703 | 18.5835 | 18.5369 | 18.6335 | 15 | 15 | 14 | 14 | 15 | 15 | 14 | 15 |
| VRK2 | Q86Y07 | 2.92E-15 | 4.35E-15 | 0.0865215 | | 0.291493 | 0.698354 | | 15.2836 | 15.3126 | 15.15 | 15.1141 | 15.2596 | 14.9288 | 15.4471 | 15.0613 | 9 | 10 | 11 | 9 | 5 | 7 | 8 | 5 |
| MAT2B | Q9NZL9 | 7.85E-13 | 5.81E-13 | 0.0867032 | | 0.409989 | 0.928331 | | 17.3955 | 17.463 | 17.439 | 17.5413 | 17.2359 | 17.4775 | 17.1895 | 17.2848 | 15 | 14 | 13 | 15 | 13 | 14 | 14 | 14 |
| PHF6 | Q8IWS0;Q8 | 2.56E-24 | 7.58E-23 | 0.0867917 | | 0.208261 | 0.52399 | | 16.0123 | 15.8422 | 16.0849 | 16.1197 | 16.1194 | 15.5883 | 15.6302 | 16.0075 | 8 | 8 | 8 | 8 | 7 | 8 | 8 | 8 |
| PGP | A6NDG6 | 7.00E-08 | 2.30E-08 | 0.0871099 | | 0.332456 | 0.779892 | | 17.0065 | 16.9674 | 16.9271 | 17.0451 | 16.886 | 16.5842 | 16.8563 | 17.0278 | 11 | 10 | 11 | 8 | 9 | 10 | 9 | 9 |
| TIAL1 | Q01085 | 1.86E-13 | 1.64E-13 | 0.087206 | | 0.910967 | 1.79527 | | 17.438 | 17.4536 | 17.3836 | 17.4668 | 17.2941 | 17.3504 | 17.275 | 17.3031 | 10 | 10 | 10 | 10 | 11 | 10 | 10 | 9 |
| ERCC2 | P18074 | 2.75E-10 | 1.23E-10 | 0.0873542 | | 0.703238 | 1.44701 | | 15.4779 | 15.5642 | 15.5146 | 15.422 | 15.4244 | 15.3755 | 15.3428 | 15.5931 | 8 | 8 | 8 | 8 | 6 | 8 | 8 | 7 |
| IARS2 | Q9NSE4 | 4.06E-17 | 1.08E-16 | 0.087436 | | 0.666163 | 1.38382 | | 17.1317 | 17.16 | 17.1404 | 17.1465 | 16.9343 | 17.1852 | 17.0206 | 16.9965 | 37 | 37 | 37 | 36 | 34 | 36 | 36 | 35 |
| PPP4C | P60510 | 1.49E-15 | 2.43E-15 | 0.0875768 | | 0.428034 | 0.961955 | | 16.7616 | 16.7995 | 16.8467 | 16.9171 | 16.7358 | 16.6359 | 16.5171 | 16.7692 | 6 | 6 | 6 | 7 | 6 | 6 | 6 | 6 |
| NDUFA10 | O95299 | 7.54E-09 | 2.78E-09 | 0.0879951 | | 0.469333 | 1.0378 | | 16.7907 | 16.7887 | 16.8048 | 16.6991 | 16.8022 | 16.8032 | 16.7651 | 16.4641 | 15 | 15 | 15 | 12 | 13 | 13 | 15 | 13 |
| PPA1 | Q15181 | 5.05E-10 | 2.17E-10 | 0.0881087 | | 0.219589 | 0.548521 | | 18.3474 | 18.4204 | 18.3936 | 18.7257 | 17.9885 | 18.2934 | 18.2055 | 18.2821 | 21 | 21 | 21 | 21 | 19 | 21 | 21 | 21 |
| STAT6 | P42226 | 6.43E-13 | 4.87E-13 | 0.0881912 | | 0.271948 | 0.658543 | | 16.5061 | 16.48 | 16.5116 | 16.6106 | 16.5242 | 16.4105 | 16.0315 | 16.4784 | 19 | 19 | 20 | 18 | 15 | 18 | 18 | 17 |
| CDC5L | Q99459 | 6.25E-09 | 2.33E-09 | 0.0882531 | + | 1.62175 | 3.00376 | | 16.2657 | 16.2827 | 16.2684 | 16.1997 | 16.2458 | 16.128 | 16.2059 | 16.1408 | 31 | 28 | 29 | 25 | 23 | 25 | 26 | 27 |
| GOT2 | P00505 | 1.80E-08 | 6.35E-09 | 0.0884801 | | 0.808605 | 1.62457 | | 18.8649 | 18.8852 | 18.8189 | 18.8698 | 18.738 | 18.6435 | 18.7556 | 18.8325 | 23 | 23 | 23 | 23 | 22 | 21 | 22 | 22 |
| CHD1 | O14646;O1 | 9.61E-05 | 2.28E-05 | 0.0888889 | | 0.126829 | 0.338186 | | 14.5209 | 15.0975 | 14.7543 | 14.6503 | 14.9414 | 14.6159 | 14.1333 | 15.1692 | 2 | 2 | 2 | 2 | 2 | 1 | 1 | 2 |
| RAB33B | Q9H082 | 0.000383 | 8.37E-05 | 0.0889249 | | 0.396802 | 0.903559 | | 15.6 | 15.618 | 15.7007 | 15.409 | 15.7925 | 15.4499 | 15.4658 | 15.636 | 3 | 3 | 3 | 3 | 2 | 3 | 3 | 3 |
| CCNYL1 | Q8N7R7;Q8 | 0.000254 | 5.69E-05 | 0.0889417 | | 0.052803 | 0.151483 | | 14.0624 | 14.149 | 14.1686 | 14.7856 | 12.5766 | 14.3911 | NaN | 14.3976 | 2 | 2 | 3 | 3 | 2 | 3 | NaN | 2 |
| NDUFS2 | O75306;O7 | 2.62E-10 | 1.18E-10 | 0.0890244 | | 0.474162 | 1.04658 | | 17.341 | 17.4211 | 17.4379 | 17.3752 | 17.109 | 17.4778 | 17.3342 | 17.2588 | 14 | 15 | 14 | 14 | 12 | 14 | 14 | 14 |
| RTF2 | Q9BY42 | 5.93E-14 | 6.02E-14 | 0.0890327 | | 0.506924 | 1.10566 | | 15.0064 | 15.0463 | 14.973 | 14.9269 | 14.8895 | 14.7914 | 14.851 | 15.139 | 7 | 7 | 8 | 7 | 6 | 6 | 8 | 7 |
| PARP9 | Q8IXQ6-2 | 1.93E-06 | 5.44E-07 | 0.0890921 | | 0.196271 | 0.497713 | | 14.5982 | 14.6531 | 14.5483 | 14.1064 | 14.8582 | 14.4188 | 14.7452 | 14.4251 | 7 | 7 | 6 | 4 | 5 | 6 | 2 | 4 |
| ARL15 | Q9NXU5 | 0.000149 | 3.46E-05 | 0.0891579 | | 0.101568 | 0.276469 | | 19.3009 | 19.1106 | 19.1153 | 18.1446 | 19.2967 | 19.4358 | 19.3778 | 19.1772 | 1 | 2 | 2 | 1 | 1 | 2 | 2 | 2 |
| TCF25 | Q9BQ70 | 3.76E-07 | 1.15E-07 | 0.0892548 | | 0.303415 | 0.72234 | | 15.3018 | 15.194 | 15.263 | 15.0017 | 15.3749 | 14.939 | 15.131 | 15.3719 | 10 | 12 | 13 | 11 | 9 | 12 | 12 | 12 |
| CSNK1D | P48730;P4 | 3.00E-17 | 8.23E-17 | 0.089322 | | 0.098595 | 0.269052 | | 15.8217 | 15.8391 | 15.8713 | 16.1973 | 16.2661 | 15.9548 | 14.9835 | 15.3719 | 3 | 3 | 3 | 3 | 1 | 2 | 3 | 3 |
| MDH2 | P40926 | 1.71E-13 | 1.53E-13 | 0.0894183 | | 0.507831 | 1.10728 | | 21.018 | 20.989 | 20.9661 | 21.0817 | 20.9244 | 20.9095 | 20.7046 | 20.8879 | 19 | 18 | 18 | 17 | 19 | 18 | 18 | 19 |
| SMAP | O00193 | 2.08E-10 | 9.52E-11 | 0.0896831 | | 0.391381 | 0.893322 | | 16.1646 | 15.9161 | 16.0885 | 15.9618 | 16.0311 | 16.1361 | 15.7476 | 15.957 | 5 | 6 | 5 | 6 | 5 | 6 | 6 | 6 |
| UBXN6 | Q9BZV1 | 4.54E-17 | 1.18E-16 | 0.0898197 | | 0.354752 | 0.823302 | | 16.1217 | 16.1775 | 16.1295 | 16.0061 | 16.1503 | 16.2978 | 15.8133 | 15.998 | 14 | 14 | 13 | 13 | 12 | 15 | 13 | 13 |
| HIBCH | Q6NVY1 | 4.87E-05 | 1.20E-05 | 0.0899591 | | 0.389714 | 0.890168 | | 16.6394 | 16.5615 | 16.688 | 16.4963 | 16.2911 | 16.7355 | 16.5819 | 16.5937 | 14 | 14 | 14 | 13 | 12 | 14 | 14 | 14 |
| HMGN2 | P05204 | 0.002415 | 0.000463 | 0.0900885 | | 0.886306 | 1.75425 | | 18.0207 | 18.0171 | 17.8462 | 17.9092 | 17.8147 | 17.824 | 17.9233 | 17.8851 | 4 | 5 | 4 | 4 | 4 | 4 | 4 | 4 |
| NMT1 | P30419 | 4.59E-16 | 8.55E-16 | 0.0901157 | | 0.259393 | 0.632629 | | 18.1153 | 18.0816 | 18.0484 | 18.0478 | 17.5822 | 18.2036 | 18.0744 | 18.0502 | 13 | 13 | 13 | 13 | 13 | 13 | 13 | 13 |
| PLCB3 | Q01970 | 4.85E-14 | 5.04E-14 | 0.0901568 | | 0.578134 | 1.23165 | | 16.3922 | 16.3962 | 16.4082 | 16.3893 | 16.289 | 16.1281 | 16.2866 | 16.4507 | 28 | 28 | 28 | 27 | 24 | 26 | 24 | 27 |
| BCL10 | O95999 | 3.15E-14 | 3.44E-14 | 0.0901883 | | 0.395481 | 0.901067 | | 15.6806 | 15.6705 | 15.7471 | 15.8092 | 15.5364 | 15.5203 | 15.4238 | 15.7564 | 10 | 7 | 9 | 9 | 7 | 7 | 9 | 10 |
| ATP13A1 | Q9HD20 | 8.24E-12 | 4.87E-12 | 0.0902157 | | 0.272423 | 0.659519 | | 16.558 | 16.609 | 16.5589 | 16.3019 | 16.303 | 16.7163 | 16.7517 | 16.3525 | 36 | 38 | 38 | 36 | 34 | 39 | 39 | 36 |
| FASN | P49327 | 1.28E-18 | 5.50E-18 | 0.0902448 | | 0.183097 | 0.468448 | | 19.3623 | 19.4028 | 19.3694 | 19.727 | 19.3912 | 18.9387 | 19.001 | 19.3819 | 104 | 105 | 105 | 103 | 103 | 103 | 102 | 103 |
| CANX | P27824;P2 | 4.40E-10 | 1.90E-10 | 0.0902944 | | 0.204635 | 0.516079 | | 19.5553 | 19.6061 | 19.6493 | 19.6802 | 19.0348 | 19.7078 | 19.7106 | 19.4331 | 36 | 36 | 38 | 36 | 36 | 36 | 35 | 38 |
| VPS25 | Q9BRG1 | 2.86E-05 | 7.17E-06 | 0.0906007 | | 0.238553 | 0.588981 | | 17.2253 | 17.1968 | 17.2631 | 17.0236 | 17.0889 | 17.0411 | 17.5882 | 16.9472 | 9 | 10 | 10 | 9 | 7 | 8 | 10 | 9 |
| FAM114A2 | Q9NRY5 | 1.36E-11 | 7.72E-12 | 0.0907248 | + | 1.55201 | 2.88012 | | 15.6524 | 15.705 | 15.7134 | 15.6223 | 15.5752 | 15.6721 | 15.5706 | 15.5575 | 18 | 20 | 19 | 17 | 17 | 17 | 20 | 18 |
| TPM4 | P67936 | 2.17E-05 | 5.51E-06 | 0.0907576 | | 0.263272 | 0.640663 | | 20.8617 | 20.8444 | 20.8005 | 20.4565 | 21.0449 | 20.9245 | 20.6449 | 20.6532 | 32 | 30 | 31 | 30 | 30 | 33 | 31 | 33 |
| ELP1 | O95163 | 2.56E-24 | 7.58E-23 | 0.0910213 | | 0.581172 | 1.23696 | | 15.9794 | 16.0276 | 15.9959 | 15.733 | 16.0067 | 15.8633 | 16.0397 | 15.9072 | 31 | 30 | 30 | 28 | 24 | 28 | 29 | 29 |
| PPOX | P50336 | 3.45E-13 | 2.83E-13 | 0.0911004 | | 0.319019 | 0.75341 | | 15.4894 | 15.3651 | 15.5194 | 15.5829 | 15.18 | 15.5693 | 15.2224 | 15.2795 | 3 | 4 | 3 | 4 | 4 | 5 | 4 | 4 |
| GNAI2 | P04899 | 3.77E-10 | 1.64E-10 | 0.0913003 | | 0.763742 | 1.54927 | | 19.3768 | 19.35 | 19.3032 | 19.3243 | 19.3763 | 19.1438 | 19.2097 | 19.206 | 21 | 20 | 19 | 18 | 20 | 20 | 18 | 19 |
| LIG3 | P49916;P4 | 1.56E-12 | 1.07E-12 | 0.0913195 | | 0.640318 | 1.33948 | | 15.1236 | 15.0678 | 15.1121 | 14.819 | 15.0201 | 15.0254 | 15.1013 | 15.0835 | 20 | 21 | 19 | 19 | 18 | 21 | 20 | 19 |
| SMARCA1 | P28370 | 9.67E-17 | 2.31E-16 | 0.0914602 | | 0.48159 | 1.06004 | | 16.1588 | 16.2036 | 16.1704 | 16.021 | 16.1932 | 15.875 | 16.1072 | 16.2343 | 29 | 31 | 27 | 26 | 22 | 21 | 22 | 22 |
| DDAH1 | O94760 | 2.91E-06 | 8.05E-07 | 0.0915812 | | 0.323334 | 0.761941 | | 17.9304 | 17.9527 | 17.9103 | 18.0341 | 18.0404 | 17.6407 | 17.6304 | 17.8523 | 20 | 21 | 21 | 20 | 18 | 19 | 19 | 20 |
| MTA2 | O94776 | 1.99E-11 | 1.09E-11 | 0.0919802 | | 0.306073 | 0.727657 | | 16.4701 | 16.5277 | 16.5018 | 16.6614 | 16.3128 | 16.1235 | 16.3813 | 16.5603 | 17 | 17 | 16 | 17 | 15 | 14 | 15 | 14 |
| PUS7 | Q96PZ0;Q9 | 1.33E-12 | 9.32E-13 | 0.0922417 | | 0.203589 | 0.513792 | | 14.3417 | 14.2693 | 14.3622 | 14.2007 | 14.6379 | 14.0638 | 13.8621 | 14.3962 | 16 | 14 | 13 | 11 | 10 | 9 | 5 | 11 |
| SIAE | Q9HAT2 | 1.81E-12 | 1.22E-12 | 0.092417 | | 0.186633 | 0.476345 | | 15.5836 | 15.6211 | 15.5123 | 14.9407 | 15.5805 | 15.8008 | 15.5964 | 15.4812 | 7 | 8 | 6 | 6 | 6 | 7 | 6 | 5 |
| RIC8A | Q9NPQ8;Q | 8.27E-07 | 2.43E-07 | 0.0927298 | | 0.695299 | 1.43351 | | 17.5698 | 17.5742 | 17.5543 | 17.4953 | 17.3867 | 17.6008 | 17.3404 | 17.5437 | 24 | 24 | 24 | 24 | 21 | 23 | 21 | 23 |
| PREP | P48147 | 7.56E-14 | 7.45E-14 | 0.0928113 | | 0.603679 | 1.27616 | | 17.5324 | 17.5847 | 17.5369 | 17.3952 | 17.3941 | 17.5844 | 17.5894 | 17.3294 | 31 | 32 | 32 | 31 | 30 | 30 | 32 | 31 |
| ASH2L | Q9UBL3;Q9 | 6.11E-08 | 2.02E-08 | 0.0929865 | | 0.433755 | 0.97255 | | 14.8184 | 14.9947 | 14.9343 | 15.0802 | 14.7077 | 14.7689 | 14.7938 | 14.7633 | 8 | 6 | 8 | 10 | 7 | 7 | 7 | 9 |
| DRG2 | P55039 | 8.47E-12 | 4.99E-12 | 0.093096 | | 0.513142 | 1.11679 | | 16.8244 | 16.8249 | 16.8375 | 16.7596 | 16.518 | 16.9065 | 16.7294 | 16.7657 | 17 | 17 | 18 | 17 | 17 | 17 | 17 | 18 |
| CHMP3 | Q9Y3E7 | 2.38E-05 | 6.01E-06 | 0.0937531 | | 0.861458 | 1.71286 | | 17.073 | 17.1391 | 17.0929 | 17.0793 | 17.0245 | 17.0907 | 16.8746 | 16.9704 | 8 | 8 | 8 | 8 | 6 | 8 | 7 | 8 |
| DMXL2 | Q8TDJ6;Q8 | 0.000128 | 2.99E-05 | 0.0938609 | | 0.29263 | 0.700651 | | 13.8093 | 14.1035 | 13.7728 | 14.0938 | 13.7839 | 13.6613 | 13.8377 | 13.6299 | 4 | 4 | 4 | 5 | 2 | 4 | 5 | 4 |
| AIMP1 | Q12904 | 1.71E-16 | 3.79E-16 | 0.0939676 | | 0.402991 | 0.915207 | | 17.7202 | 17.7299 | 17.6505 | 17.4324 | 17.6992 | 17.8215 | 17.6406 | 17.4375 | 17 | 16 | 16 | 16 | 16 | 19 | 18 | 17 |
| CPSF4 | O95639;O9 | 0.001865 | 0.000368 | 0.094426 | | 0.308258 | 0.732021 | | 14.0615 | 14.16 | 14.2464 | 14.2803 | 14.2657 | 13.9908 | 13.8033 | 13.9675 | 7 | 7 | 7 | 5 | 2 | 6 | 4 | 6 |
| AZI2 | Q9H6S1 | 2.06E-09 | 8.17E-10 | 0.0944618 | | 0.079548 | 0.220721 | | 14.0321 | 14.0781 | 14.0412 | 14.3121 | 12.9152 | 14.8505 | 13.7347 | 13.9675 | 3 | 3 | 3 | 3 | 2 | 2 | 1 | 2 |
| REXO2 | Q9Y3B8-3 | 8.09E-05 | 1.94E-05 | 0.094626 | | 0.427005 | 0.960046 | | 18.6624 | 18.6476 | 18.5528 | 18.6168 | 18.5142 | 18.7244 | 18.2974 | 18.4789 | 8 | 8 | 8 | 8 | 7 | 9 | 9 | 8 |
| MRTO4 | Q9UKD2 | 9.08E-09 | 3.32E-09 | 0.0946269 | | 0.323372 | 0.762017 | | 17.2009 | 17.3285 | 17.2933 | 17.4584 | 17.0055 | 17.1799 | 16.9671 | 17.2873 | 13 | 16 | 16 | 15 | 14 | 13 | 15 | 15 |
| L3HYPDH | Q96EM0 | 6.46E-11 | 3.21E-11 | 0.0948565 | | 0.136416 | 0.361044 | | 16.5381 | 16.6532 | 16.5647 | 16.1989 | 16.4833 | 17.0974 | 16.7026 | 15.97 | 11 | 11 | 11 | 11 | 11 | 11 | 11 | 11 |
| CCDC50 | Q8IVM0 | 1.40E-15 | 2.30E-15 | 0.0949126 | | 0.262996 | 0.640094 | | 16.0745 | 16.0883 | 16.1295 | 16.2637 | 15.8235 | 16.0944 | 15.6687 | 16.1624 | 10 | 10 | 11 | 10 | 9 | 10 | 10 | 10 |
| NCL | P19338 | 2.25E-16 | 4.71E-16 | 0.0952119 | + | 1.94833 | 3.6076 | | 19.7089 | 19.6966 | 19.6949 | 19.5605 | 19.56 | 19.6174 | 19.6255 | 19.661 | 37 | 35 | 36 | 35 | 38 | 36 | 35 | 35 |
| XRN1 | Q8IZH2;Q8 | 1.61E-14 | 1.93E-14 | 0.0952464 | | 0.590108 | 1.25255 | | 15.6537 | 15.6222 | 15.6002 | 15.3376 | 15.6664 | 15.5458 | 15.6092 | 15.4916 | 24 | 23 | 24 | 21 | 18 | 22 | 19 | 21 |
| ADAM17 | P78536 | 6.68E-24 | 1.62E-22 | 0.0953028 | | 0.613173 | 1.29262 | | 15.2521 | 15.2881 | 15.1759 | 15.1616 | 15.1955 | 15.3021 | 15.0361 | 15.0216 | 11 | 10 | 8 | 9 | 7 | 9 | 9 | 8 |
| DOLPP1 | Q86YN1 | 0.005117 | 0.000913 | 0.0953056 | | 0.262443 | 0.638949 | | 16.4577 | 16.5196 | 16.5755 | 16.7682 | 16.2249 | 16.1579 | 16.5439 | 16.4165 | 2 | 2 | 2 | 2 | 2 | 3 | 2 | 2 |
| NUDT16L1 | Q9BRJ7 | 0.000218 | 4.92E-05 | 0.0953976 | | 0.19468 | 0.4942 | | 15.2569 | 15.234 | 15.3086 | 15.2156 | 15.2323 | 14.6174 | 15.3526 | 15.4376 | 6 | 4 | 5 | 5 | 4 | 4 | 6 | 4 |
| MRRF | Q96E11 | 4.01E-09 | 1.53E-09 | 0.0956151 | | 0.546789 | 1.17656 | | 16.1919 | 16.1401 | 16.1383 | 16.259 | 16.0668 | 15.9556 | 15.9191 | 16.1053 | 6 | 7 | 7 | 8 | 7 | 7 | 6 | 7 |
| LUC7L | Q9NQ29;Q | 1.13E-07 | 3.63E-08 | 0.095628 | | 0.481127 | 1.0592 | | 15.557 | 15.626 | 15.5784 | 15.6989 | 15.419 | 15.3689 | 15.3717 | 15.599 | 7 | 8 | 9 | 8 | 9 | 7 | 7 | 8 |

| Gene names | UniProt IDs | Protein Group P-value | Protein Group Q-value | Log2 Difference NUP214 vs Controls (n=5) | p<0.05 | -log10 p-value | T-test Test statistic | NPC Localization | Log2 MS2_quantity_NUP214_1 | Log2 MS2_quantity_NUP214_2 | Log2 MS2_quantity_NUP214_3 | Log2 MS2_quantity_Control_1 | Log2 MS2_quantity_Control_2 | Log2 MS2_quantity_Control_3 | Log2 MS2_quantity_Control_4 | Log2 MS2_quantity_Control_5 | Number Peptides for Quantitation NUP214_1 | Number Peptides for Quantitation NUP214_2 | Number Peptides for Quantitation NUP214_3 | Number Peptides for Quantitation Control_1 | Number Peptides for Quantitation Control_2 | Number Peptides for Quantitation Control_3 | Number Peptides for Quantitation Control_4 | Number Peptides for Quantitation Control_5 |
|---|---|---|---|---|---|---|---|---|---|---|---|---|---|---|---|---|---|---|---|---|---|---|---|---|
| TDRD3 | Q9H7E2-3 | 0.00099 | 0.000204 | 0.0957396 | | 0.254321 | 0.622079 | | 16.5847 | 16.6499 | 16.2441 | 16.1601 | 16.4769 | 16.5049 | 16.6412 | 16.2027 | 3 | 5 | 5 | 1 | 3 | 4 | 2 | 3 |
| CCDC134 | Q9H6E4 | 1.55E-17 | 4.63E-17 | 0.0958726 | | 0.146583 | 0.384969 | | 15.5122 | 15.5498 | 15.5671 | 15.318 | 16.0646 | 14.9883 | 15.2235 | 15.6412 | 6 | 5 | 6 | 5 | 5 | 5 | 6 | 5 |
| RPL32 | P62910 | 5.55E-07 | 1.66E-07 | 0.0958797 | + | 1.31149 | 2.46465 | | 19.3895 | 19.532 | 19.509 | 19.4141 | 19.3841 | 19.415 | 19.3288 | 19.3628 | 8 | 8 | 8 | 8 | 8 | 8 | 8 | 8 |
| PTBP3 | O95758;O9 | 1.80E-07 | 5.69E-08 | 0.0960979 | | 0.308112 | 0.731729 | | 16.6189 | 16.7028 | 16.6369 | 16.3313 | 16.3904 | 16.7706 | 16.8039 | 16.4875 | 10 | 10 | 11 | 10 | 9 | 11 | 10 | 10 |
| FLNA | P21333;P2 | 6.18E-10 | 2.62E-10 | 0.0961709 | | 0.493255 | 1.0811 | | 21.6593 | 21.7132 | 21.672 | 21.4239 | 21.5246 | 21.8118 | 21.6398 | 21.5267 | 152 | 153 | 150 | 146 | 145 | 150 | 149 | 150 |
| PLEKHF1 | Q96S99 | 9.96E-08 | 3.22E-08 | 0.0966287 | | 0.160809 | 0.417929 | | 16.1163 | 15.9631 | 16.0143 | 15.6445 | 16.1485 | 16.448 | 15.9399 | 15.4922 | 6 | 6 | 7 | 6 | 6 | 6 | 5 | 5 |
| TGM2 | P21980 | 2.70E-16 | 5.43E-16 | 0.0966424 | | 0.053366 | 0.151734 | | 18.1312 | 18.1351 | 18.1088 | 16.5801 | 17.3416 | 18.8978 | 19.1288 | 18.1935 | 27 | 28 | 30 | 23 | 22 | 29 | 29 | 26 |
| ABHD10 | Q9NUJ1 | 2.67E-07 | 8.29E-08 | 0.0966588 | | 0.609945 | 1.28703 | | 16.4265 | 16.5782 | 16.5789 | 16.3123 | 16.5498 | 16.5228 | 16.3251 | 16.4461 | 9 | 9 | 9 | 9 | 9 | 10 | 9 | 9 |
| NBAS | A2RRP1 | 8.93E-12 | 5.24E-12 | 0.0966616 | | 0.961032 | 1.87846 | | 16.1089 | 16.0881 | 16.06 | 15.8675 | 16.0888 | 16.0233 | 16.0188 | 15.9468 | 39 | 43 | 40 | 40 | 37 | 41 | 42 | 37 |
| RBBP4 | Q09028;Q0 | 1.11E-13 | 1.04E-13 | 0.0966722 | | 0.568983 | 1.21562 | | 17.9813 | 18.0095 | 17.9948 | 18.0465 | 17.7977 | 17.7253 | 17.9423 | 17.9809 | 14 | 14 | 14 | 14 | 14 | 14 | 14 | 14 |
| PAWR | Q96IZ0 | 3.86E-08 | 1.31E-08 | 0.0966914 | | 0.173676 | 0.447253 | | 16.4947 | 16.4221 | 16.5226 | 15.8809 | 16.2944 | 16.7982 | 16.6616 | 16.2806 | 13 | 13 | 14 | 13 | 12 | 14 | 14 | 13 |
| DHX8 | Q14562 | 6.23E-07 | 1.85E-07 | 0.0967039 | | 0.289733 | 0.694795 | | 14.2494 | 14.4418 | 14.308 | 14.129 | 14.3742 | 13.9063 | 14.4701 | 14.3023 | 8 | 6 | 8 | 3 | 6 | 4 | 5 | 7 |
| AVEN | Q9NQS1 | 1.08E-12 | 7.75E-13 | 0.0968872 | | 0.326513 | 0.76821 | | 14.963 | 14.7482 | 15.0454 | 14.8462 | 14.662 | 15.0397 | 14.9468 | 14.6153 | 7 | 6 | 6 | 6 | 5 | 6 | 5 | 6 |
| NNT | Q13423 | 2.55E-12 | 1.67E-12 | 0.0971217 | | 0.487613 | 1.07093 | | 17.2642 | 17.3585 | 17.2994 | 17.2086 | 17.3029 | 17.3012 | 17.2844 | 16.9541 | 36 | 37 | 36 | 36 | 35 | 36 | 37 | 36 |
| PFKL | P17858 | 4.26E-18 | 1.55E-17 | 0.0971472 | | 0.497444 | 1.08864 | | 18.6896 | 18.6979 | 18.65 | 18.6139 | 18.5565 | 18.7613 | 18.6257 | 18.3527 | 29 | 29 | 30 | 28 | 29 | 29 | 28 | 29 |
| MRPL4 | Q9BYD3 | 4.72E-10 | 2.04E-10 | 0.0972441 | | 0.500632 | 1.09437 | | 16.3491 | 16.3008 | 16.4152 | 16.3738 | 16.3121 | 16.2629 | 16.0113 | 16.3288 | 9 | 9 | 9 | 9 | 6 | 8 | 8 | 9 |
| DIS3L2 | Q8IYB7 | 6.39E-19 | 2.96E-18 | 0.0975266 | | 0.651143 | 1.35808 | | 15.8638 | 15.9252 | 15.8343 | 15.8657 | 15.8822 | 15.6586 | 15.6445 | 15.8337 | 23 | 20 | 21 | 22 | 16 | 16 | 22 | 19 |
| TRAF6 | Q9Y4K3 | 0.00157 | 0.000315 | 0.0976561 | | 0.304618 | 0.724747 | | 14.8088 | 15.0138 | 15.0501 | 15.1721 | 14.7582 | 14.8582 | 14.8982 | 14.6128 | 6 | 5 | 5 | 5 | 3 | 4 | 3 | 5 |
| PSMD5 | Q16401 | 7.75E-13 | 5.74E-13 | 0.0977542 | + | 1.49579 | 2.78158 | | 18.3744 | 18.3829 | 18.3721 | 18.2621 | 18.3676 | 18.2278 | 18.2303 | 18.3056 | 18 | 18 | 19 | 17 | 18 | 18 | 19 | 17 |
| CHD1L | Q86WJ1;Q | 1.91E-05 | 4.88E-06 | 0.097757 | | 0.213558 | 0.535497 | | 14.7099 | 14.9081 | 14.8894 | 14.9101 | 14.2259 | 14.7957 | 14.7904 | 14.9682 | 14 | 16 | 11 | 11 | 5 | 9 | 6 | 9 |
| NFYC | Q13952;Q1 | 5.02E-07 | 1.51E-07 | 0.0977757 | | 0.6399 | 1.33876 | | 15.8176 | 15.7839 | 15.697 | 15.716 | 15.6422 | 15.555 | 15.5875 | 15.8413 | 5 | 5 | 5 | 5 | 3 | 3 | 4 | 4 |
| WDR45 | Q9Y484;Q9 | 0.002349 | 0.000452 | 0.0978024 | | 0.711835 | 1.4616 | | 15.5763 | 15.694 | 15.6187 | 15.3995 | 15.5984 | 15.6478 | 15.4493 | 15.5644 | 5 | 4 | 5 | 5 | 5 | 4 | 5 | 5 |
| RBM8A | Q9Y5S9;Q9 | 1.28E-06 | 3.66E-07 | 0.0979165 | | 0.255628 | 0.624804 | | 17.3319 | 17.3553 | 17.3688 | 17.3144 | 16.8115 | 17.2851 | 17.5115 | 17.3479 | 6 | 6 | 7 | 7 | 6 | 6 | 6 | 5 |
| TFIP11 | Q9UBB9 | 3.85E-05 | 9.52E-06 | 0.0979166 | | 0.518388 | 1.12615 | | 16.0677 | 15.9267 | 15.9942 | 15.8668 | 16.0846 | 15.703 | 15.9037 | 15.9333 | 12 | 11 | 12 | 9 | 7 | 6 | 6 | 8 |
| SF3B3 | Q15393 | 2.56E-24 | 7.58E-23 | 0.097959 | | 0.871905 | 1.73026 | | 18.0474 | 18.0936 | 18.0416 | 17.9775 | 18.0628 | 17.8451 | 17.8925 | 18.0365 | 40 | 41 | 40 | 38 | 37 | 38 | 39 | 38 |
| VPS8 | Q8N3P4;Q8 | 1.44E-11 | 8.11E-12 | 0.098095 | | 0.327791 | 0.770727 | | 14.9745 | 14.9814 | 14.9512 | 14.5353 | 15.0916 | 14.8337 | 15.0081 | 14.886 | 18 | 18 | 19 | 13 | 13 | 20 | 17 | 13 |
| OGDH | Q02218 | 2.38E-16 | 4.94E-16 | 0.098222 | | 0.398308 | 0.906398 | | 17.097 | 17.112 | 17.108 | 17.1978 | 16.7317 | 17.0996 | 16.9293 | 17.0789 | 42 | 43 | 43 | 42 | 34 | 39 | 42 | 42 |
| LDAH | Q9H6V9;Q9 | 5.08E-07 | 1.53E-07 | 0.0984278 | | 0.190621 | 0.485213 | | 16.9969 | 16.9646 | 16.9353 | 16.2989 | 17.2095 | 16.9115 | 16.9932 | 16.9229 | 4 | 4 | 4 | 4 | 3 | 4 | 4 | 4 |
| RUSF1 | Q96GQ5 | 2.38E-08 | 8.28E-09 | 0.0986933 | | 0.352383 | 0.818721 | | 15.4884 | 15.5125 | 15.4536 | 15.4708 | 15.1827 | 15.5436 | 15.576 | 15.1575 | 11 | 12 | 11 | 10 | 7 | 11 | 9 | 9 |
| MAPK7 | Q13164 | 5.41E-05 | 1.32E-05 | 0.0988686 | | 0.3415 | 0.797578 | | 15.2582 | 15.17 | 15.0641 | 15.0782 | 14.8653 | 15.297 | 14.8709 | 15.2147 | 7 | 9 | 9 | 11 | 8 | 10 | 8 | 8 |
| IGHMBP2 | P38935 | 0.000744 | 0.000156 | 0.0988998 | | 0.085218 | 0.235261 | | 13.5435 | 13.8557 | 14.4617 | 14.527 | 12.9519 | 14.1978 | 13.5147 | 14.0824 | 1 | 2 | 1 | 3 | 2 | 2 | 3 | 3 |
| EIF5 | P55010 | 1.25E-16 | 2.89E-16 | 0.0989071 | | 0.610062 | 1.28723 | | 18.0216 | 18.0621 | 18.0165 | 17.917 | 17.8838 | 18.1582 | 17.8657 | 17.8477 | 20 | 20 | 20 | 20 | 18 | 20 | 20 | 19 |
| PIP5K1A | Q99755;Q9 | 7.06E-12 | 4.24E-12 | 0.0991009 | | 0.278397 | 0.671749 | | 16.3036 | 16.3241 | 16.1998 | 16.2149 | 15.8422 | 16.5213 | 16.1894 | 16.1159 | 9 | 11 | 10 | 11 | 8 | 11 | 10 | 8 |
| TUBGCP3 | Q96CW5 | 6.54E-13 | 4.95E-13 | 0.0991072 | + | 1.36676 | 2.55881 | | 15.3136 | 15.3612 | 15.342 | 15.1531 | 15.1976 | 15.3044 | 15.2818 | 15.2623 | 26 | 27 | 27 | 24 | 22 | 23 | 24 | 23 |
| THY1 | P04216 | 0.000725 | 0.000153 | 0.0992757 | | 0.197142 | 0.499634 | | 21.8075 | 21.9911 | 21.9359 | 21.391 | 22.1917 | 22.0454 | 21.5895 | 21.8436 | 4 | 4 | 4 | 4 | 4 | 4 | 4 | 4 |
| TJP1 | Q07157 | 2.97E-18 | 1.12E-17 | 0.0992798 | | 0.364966 | 0.842984 | | 17.0486 | 17.0531 | 17.0969 | 17.2971 | 16.823 | 16.9926 | 16.8316 | 16.8901 | 50 | 52 | 52 | 50 | 37 | 49 | 46 | 45 |
| ZNF346 | Q9UL40;Q9 | 0.00089 | 0.000185 | 0.0992997 | | 0.337887 | 0.790525 | | 14.6587 | 14.8114 | 14.6058 | 14.4738 | 14.8715 | 14.7075 | 14.5321 | 14.3783 | 3 | 3 | 4 | 2 | 2 | 3 | 4 | 3 |
| DCAKD | Q8WVC6 | 3.92E-16 | 7.44E-16 | 0.0993184 | | 0.78789 | 1.58985 | | 18.4066 | 18.4161 | 18.428 | 18.198 | 18.4047 | 18.4023 | 18.3728 | 18.2101 | 7 | 8 | 8 | 7 | 7 | 8 | 9 | 7 |
| ATRAID | Q6UW56;Q | 0.007011 | 0.001228 | 0.0993614 | | 0.060535 | 0.170937 | | 16.1681 | 16.0906 | 15.9659 | 16.3658 | 14.2642 | 16.2343 | 16.6911 | 16.3223 | 2 | 2 | 2 | 2 | 1 | 2 | 2 | 2 |
| GALK1 | P51570;P5 | 2.36E-11 | 1.28E-11 | 0.0995435 | | 0.49457 | 1.08347 | | 17.5442 | 17.4916 | 17.4859 | 17.2556 | 17.4656 | 17.6435 | 17.3172 | 17.3565 | 16 | 16 | 16 | 16 | 16 | 16 | 16 | 16 |
| GNA12 | Q03113 | 0.001181 | 0.000241 | 0.0996812 | | 0.792273 | 1.5972 | | 15.629 | 15.6868 | 15.6834 | 15.7289 | 15.577 | 15.4646 | 15.497 | 15.566 | 5 | 6 | 5 | 5 | 3 | 5 | 6 | 6 |
| ATG2A | Q2TAZ0;Q2 | 0.0019 | 0.000374 | 0.0997669 | | 0.270978 | 0.656549 | | 15.1288 | 15.0696 | 14.9541 | 14.615 | 15.1701 | 14.9072 | 14.8469 | 15.2162 | 8 | 11 | 10 | 9 | 4 | 6 | 5 | 7 |
| ABCC1 | P33527 | 8.50E-19 | 3.77E-18 | 0.0997773 | | 0.419633 | 0.94634 | | 16.168 | 16.1544 | 16.1582 | 16.2743 | 15.9658 | 16.2288 | 15.9328 | 15.9003 | 37 | 36 | 35 | 32 | 27 | 38 | 31 | 33 |
| UCHL5 | Q9Y5K5;Q9 | 1.48E-09 | 5.99E-10 | 0.0997896 | | 0.969883 | 1.89316 | | 16.7018 | 16.6722 | 16.6603 | 16.6327 | 16.512 | 16.5471 | 16.4971 | 16.7025 | 14 | 15 | 14 | 14 | 13 | 14 | 14 | 14 |
| NAGPA | Q9UK23;Q9 | 0.002673 | 0.000506 | 0.0998278 | | 0.091843 | 0.252084 | | 15.8247 | 15.2746 | 14.8705 | 14.9099 | 14.8261 | 15.4484 | 14.803 | 16.13 | 1 | 3 | 2 | 1 | 1 | 1 | 1 | 1 |
| SSU72 | Q9NP77 | 5.80E-05 | 1.41E-05 | 0.0998401 | | 0.429534 | 0.964735 | | 16.1068 | 16.0668 | 16.0503 | 16.2384 | 15.9369 | 15.881 | 15.7856 | 16.032 | 7 | 7 | 6 | 7 | 7 | 6 | 7 | 7 |
| MRPL44 | Q9H9J2 | 6.59E-10 | 2.78E-10 | 0.0999217 | | 0.448745 | 1.00017 | | 16.2323 | 16.2128 | 16.1048 | 16.1674 | 16.1214 | 15.9135 | 15.9278 | 16.2866 | 10 | 12 | 9 | 12 | 11 | 10 | 9 | 11 |
| TRIM2 | Q9C040;Q9 | 1.27E-09 | 5.19E-10 | 0.100002 | | 0.542654 | 1.16925 | | 15.5855 | 15.6254 | 15.5454 | 15.5748 | 15.5067 | 15.2389 | 15.5709 | 15.5359 | 11 | 11 | 10 | 11 | 9 | 10 | 9 | 9 |
| RHBDD2 | Q6NTF9;Q6 | 0.004647 | 0.000835 | 0.100083 | | 0.15373 | 0.401601 | | 16.4514 | 16.0153 | 16.376 | 15.7456 | 16.3102 | 15.8158 | 16.6294 | 16.403 | 2 | 2 | 2 | 2 | 2 | 1 | 2 | 2 |
| MRPL54 | Q6P161 | 0.0006 | 0.000128 | 0.10027 | | 0.277076 | 0.66905 | | 17.3283 | 17.2297 | 17.3852 | 17.409 | 17.3554 | 16.8231 | 17.1233 | 17.3598 | 1 | 2 | 2 | 1 | 2 | 1 | 2 | 2 |
| TMED2 | Q15363 | 2.27E-07 | 7.11E-08 | 0.100446 | | 0.20589 | 0.518822 | | 18.3798 | 18.5617 | 18.5963 | 18.5638 | 17.8863 | 18.6727 | 18.5708 | 18.3672 | 5 | 4 | 5 | 4 | 4 | 4 | 6 | 4 |
| U2SURP | O15042;O1 | 1.01E-14 | 1.29E-14 | 0.100618 | | 1.06082 | 2.04424 | | 16.1104 | 16.1462 | 16.0888 | 16.1202 | 16.0199 | 15.8953 | 16.0105 | 16.0268 | 21 | 20 | 23 | 21 | 16 | 23 | 21 | 20 |
| ZHX3 | Q9H4I2 | 5.43E-05 | 1.33E-05 | 0.100701 | | 0.221664 | 0.552984 | | 14.1005 | 14.4523 | 14.5369 | 14.4645 | 13.8476 | 14.177 | 14.3877 | 14.4358 | 6 | 4 | 5 | 4 | 3 | 4 | 3 | 1 |
| GPR180 | Q86V85 | 3.96E-07 | 1.21E-07 | 0.100727 | | 0.416308 | 0.940142 | | 15.1741 | 15.2895 | 15.2213 | 14.8958 | 15.0624 | 15.2597 | 15.3393 | 15.0809 | 5 | 5 | 5 | 5 | 5 | 5 | 4 | 5 |
| INTS1 | Q8N201 | 2.82E-09 | 1.10E-09 | 0.101012 | | 0.582675 | 1.23959 | | 15.019 | 15.1225 | 15.072 | 14.8296 | 15.0855 | 14.8252 | 15.0349 | 15.0755 | 22 | 19 | 21 | 15 | 10 | 16 | 17 | 15 |
| DNAJC2 | Q99543 | 1.80E-08 | 6.35E-09 | 0.101076 | | 0.623276 | 1.3101 | | 15.0862 | 15.1257 | 15.1968 | 15.1382 | 14.9597 | 15.1519 | 15.0639 | 14.8621 | 13 | 13 | 12 | 10 | 12 | 13 | 12 | 12 |
| WDR82 | Q6UXN9 | 1.02E-10 | 4.88E-11 | 0.101088 | + | 1.44763 | 2.69791 | | 15.7331 | 15.8054 | 15.756 | 15.7455 | 15.6201 | 15.6766 | 15.5994 | 15.6771 | 12 | 12 | 12 | 12 | 13 | 11 | 12 | 13 |
| SH3GL1 | Q99961 | 5.69E-19 | 2.72E-18 | 0.101089 | | 0.452313 | 1.00672 | | 18.3993 | 18.392 | 18.4314 | 18.5071 | 18.108 | 18.4099 | 18.1624 | 18.3449 | 21 | 21 | 21 | 22 | 21 | 21 | 21 | 21 |
| CTBP2 | P56545;P5 | 2.02E-17 | 5.81E-17 | 0.101217 | | 0.212765 | 0.533778 | | 16.5914 | 16.6275 | 16.6677 | 16.9777 | 16.3906 | 16.1556 | 16.4157 | 16.6988 | 7 | 7 | 7 | 7 | 7 | 7 | 7 | 7 |
| CARM1 | Q86X55;Q8 | 4.33E-06 | 1.18E-06 | 0.101359 | | 0.407082 | 0.922886 | | 17.6377 | 17.663 | 17.6259 | 17.6518 | 17.8084 | 17.404 | 17.3802 | 17.4598 | 16 | 15 | 15 | 16 | 14 | 15 | 15 | 15 |
| HNRNPK | P61978-3 | 2.97E-06 | 8.23E-07 | 0.101545 | | 0.422805 | 0.952244 | | 16.2781 | 16.4047 | 16.5062 | 16.4155 | 16.3689 | 16.1353 | 16.444 | 16.1102 | 2 | 2 | 2 | 2 | 2 | 2 | 2 | 2 |
| MATR3 | P43243 | 8.95E-14 | 8.66E-14 | 0.101555 | + | 1.5651 | 2.9032 | | 18.3198 | 18.318 | 18.2773 | 18.2514 | 18.2695 | 18.1378 | 18.1638 | 18.1947 | 32 | 32 | 32 | 32 | 32 | 32 | 32 | 32 |
| LEMD3 | Q9Y2U8 | 6.81E-13 | 5.12E-13 | 0.101727 | | 0.898839 | 1.7751 | | 15.5096 | 15.5725 | 15.5147 | 15.5396 | 15.4612 | 15.3973 | 15.291 | 15.4636 | 17 | 17 | 17 | 15 | 15 | 13 | 17 | 16 |
| BCAP31 | P51572;P5 | 9.01E-11 | 4.36E-11 | 0.101937 | | 0.482891 | 1.0624 | | 17.8619 | 17.8141 | 17.8189 | 17.8344 | 17.4544 | 17.8034 | 17.8297 | 17.7266 | 16 | 16 | 16 | 15 | 15 | 16 | 16 | 15 |
| MRPS10 | P82664 | 4.03E-08 | 1.36E-08 | 0.101998 | | 0.219041 | 0.54734 | | 15.167 | 15.2167 | 15.3863 | 15.4211 | 14.7471 | 14.9192 | 15.3235 | 15.3624 | 5 | 5 | 5 | 6 | 3 | 4 | 4 | 5 |
| TUBG1 | P23258 | 2.56E-10 | 1.15E-10 | 0.102022 | | 0.628614 | 1.31932 | | 16.702 | 16.7412 | 16.7358 | 16.7056 | 16.4166 | 16.5798 | 16.7096 | 16.7101 | 15 | 15 | 16 | 14 | 11 | 16 | 16 | 14 |
| TMEM209 | Q96SK2;Q9 | 5.36E-06 | 1.44E-06 | 0.102093 | | 0.369945 | 0.852533 | | 14.387 | 14.5196 | 14.5118 | 14.4198 | 14.6171 | 14.4689 | 14.1741 | 14.1736 | 6 | 5 | 5 | 3 | 3 | 4 | 2 | 4 |
| SPG7 | Q9UQ90 | 0.000121 | 2.83E-05 | 0.102183 | | 0.205301 | 0.517535 | | 14.1921 | 14.0919 | 14.067 | 13.7116 | 13.739 | 14.1965 | 14.4872 | 13.9398 | 3 | 5 | 5 | 2 | 1 | 1 | 2 | 1 |
| RFC5 | P40937;P4 | 4.63E-05 | 1.14E-05 | 0.102184 | | 0.244874 | 0.602306 | | 15.7616 | 15.7132 | 15.7753 | 15.9053 | 15.4002 | 15.332 | 15.6468 | 15.9549 | 11 | 9 | 11 | 10 | 7 | 8 | 10 | 9 |
| FMR1 | Q06787-2 | 8.76E-13 | 6.44E-13 | 0.10234 | | 0.580877 | 1.23644 | | 16.7962 | 16.8237 | 16.7426 | 16.5279 | 16.6085 | 16.8662 | 16.6449 | 16.7783 | 16 | 15 | 15 | 13 | 12 | 15 | 14 | 12 |
| ITGA4 | P13612 | 3.92E-16 | 7.44E-16 | 0.102609 | | 0.277788 | 0.670505 | | 16.3054 | 16.3783 | 16.359 | 16.4214 | 16.1109 | 16.5407 | 16.2588 | 15.893 | 16 | 18 | 16 | 17 | 12 | 16 | 17 | 15 |
| FRRS1 | Q6ZNA5;Q6 | 0.043274 | 0.00705 | 0.102759 | | 0.1227 | 0.33583 | | 15.9873 | 15.9334 | 15.704 | 15.2811 | NaN | 16.2959 | NaN | 15.7394 | 2 | 1 | 1 | 1 | NaN | 1 | NaN | 1 |
| RRS1 | Q15050 | 3.09E-07 | 9.53E-08 | 0.102774 | + | 2.12041 | 3.94527 | | 16.3382 | 16.3028 | 16.2874 | 16.202 | 16.1548 | 16.2313 | 16.1878 | 16.2578 | 11 | 11 | 13 | 12 | 11 | 11 | 13 | 12 |
| ATP5MF | P56134;P5 | 0.002641 | 0.000501 | 0.102851 | | 0.228671 | 0.56799 | | 20.5501 | 20.3499 | 20.3996 | 20.4957 | 20.4056 | 19.8166 | 20.3864 | 20.5473 | 3 | 3 | 3 | 3 | 3 | 3 | 3 | 3 |
| AIFM1 | O95831;O9 | 3.42E-11 | 1.79E-11 | 0.103117 | + | 1.56503 | 2.90308 | | 17.3907 | 17.4497 | 17.4153 | 17.2616 | 17.3146 | 17.3988 | 17.334 | 17.2681 | 18 | 18 | 19 | 19 | 19 | 19 | 19 | 19 |
| DDX23 | Q9BUQ8 | 7.90E-16 | 1.39E-15 | 0.103136 | | 0.762415 | 1.54704 | | 16.0907 | 16.1951 | 16.2001 | 15.9722 | 16.2193 | 15.9994 | 16.104 | 15.9993 | 24 | 24 | 25 | 26 | 22 | 24 | 24 | 25 |
| TBCC | Q15814 | 1.43E-16 | 3.23E-16 | 0.103151 | | 0.635175 | 1.33063 | | 16.7362 | 16.7628 | 16.7502 | 16.7901 | 16.4443 | 16.6698 | 16.6164 | 16.7123 | 9 | 8 | 8 | 9 | 8 | 8 | 8 | 9 |
| HSP90AA1 | P07900;P0 | 4.48E-12 | 2.78E-12 | 0.10318 | | 0.836573 | 1.67133 | | 20.6816 | 20.7107 | 20.6888 | 20.617 | 20.7258 | 20.4383 | 20.5759 | 20.5954 | 32 | 32 | 31 | 32 | 32 | 32 | 33 | 32 |

| Gene names | UniProt IDs | Protein Group P-value | Protein Group Q-value | Log2 Difference NUP214 vs Controls (n=5) | p<0.05 | -log10 p-value | T-test Test statistic | NPC Localization | Log2 MS2_quantity_NUP214_1 | Log2 MS2_quantity_NUP214_2 | Log2 MS2_quantity_NUP214_3 | Log2 MS2_quantity_Control_1 | Log2 MS2_quantity_Control_2 | Log2 MS2_quantity_Control_3 | Log2 MS2_quantity_Control_4 | Log2 MS2_quantity_Control_5 | Number Peptides for Quantitation NUP214_1 | Number Peptides for Quantitation NUP214_2 | Number Peptides for Quantitation NUP214_3 | Number Peptides for Quantitation Control_1 | Number Peptides for Quantitation Control_2 | Number Peptides for Quantitation Control_3 | Number Peptides for Quantitation Control_4 | Number Peptides for Quantitation Control_5 |
|---|---|---|---|---|---|---|---|---|---|---|---|---|---|---|---|---|---|---|---|---|---|---|---|---|
| NUDCD1 | Q96RS6;Q9 | 6.27E-22 | 7.95E-21 | 0.103454 | | 0.552619 | 1.18685 | | 16.2565 | 16.3172 | 16.2761 | 16.3804 | 15.9826 | 16.2016 | 16.1246 | 16.2099 | 15 | 17 | 16 | 16 | 14 | 16 | 15 | 16 |
| ZWILCH | Q9H900 | 8.47E-12 | 4.99E-12 | 0.103476 | | 0.391837 | 0.894183 | | 14.7475 | 14.9714 | 14.8942 | 14.9694 | 14.8681 | 14.6273 | 14.5431 | 14.83 | 6 | 4 | 6 | 5 | 5 | 4 | 4 | 5 |
| COPG2 | Q9UBF2 | 3.15E-13 | 2.62E-13 | 0.103596 | | 0.793069 | 1.59854 | | 17.7364 | 17.7477 | 17.7816 | 17.7475 | 17.6264 | 17.7473 | 17.722 | 17.6849 | 27 | 26 | 28 | 25 | 27 | 26 | 27 | 25 |
| CBX8 | Q9HC52 | 8.05E-15 | 1.04E-14 | 0.103691 | | 0.165207 | 0.432222 | | 15.6952 | 15.6273 | 15.6692 | 15.6099 | NaN | 16.1043 | 15.3531 | 15.1735 | 2 | 2 | 1 | 1 | NaN | 1 | 1 | 2 |
| CASP7 | P55210;P5 | 1.69E-09 | 6.77E-10 | 0.103851 | | 0.30028 | 0.716054 | | 15.7433 | 16.0353 | 15.9232 | 15.5999 | 16.0055 | 15.98 | 15.8698 | 15.5285 | 8 | 8 | 8 | 8 | 7 | 7 | 7 | 6 |
| HDDC3 | Q8N4P3;Q | 8.96E-06 | 2.36E-06 | 0.103882 | | 0.064542 | 0.185446 | | 13.1407 | 12.8332 | 13.0739 | 13.7254 | NaN | NaN | 11.858 | 13.1528 | 2 | 2 | 2 | 2 | NaN | NaN | 2 | 2 |
| YARS2 | Q9Y2Z4 | 1.44E-11 | 8.11E-12 | 0.104064 | | 0.660949 | 1.37489 | | 15.8167 | 16.1071 | 15.9969 | 15.8898 | 15.7988 | 15.9834 | 15.8145 | 15.8611 | 8 | 10 | 9 | 10 | 9 | 9 | 10 | 9 |
| SEPTIN11 | Q9NVA2 | 9.54E-12 | 5.56E-12 | 0.104081 | | 0.754797 | 1.53421 | | 18.9899 | 19.0296 | 18.9212 | 18.7006 | 18.942 | 18.9791 | 18.8798 | 18.8794 | 25 | 25 | 25 | 25 | 21 | 25 | 25 | 25 |
| CS | O75390 | 1.70E-09 | 6.82E-10 | 0.104089 | | 0.391752 | 0.894023 | | 19.5948 | 19.6101 | 19.5862 | 19.726 | 19.6252 | 19.2669 | 19.3242 | 19.5224 | 16 | 16 | 16 | 16 | 16 | 16 | 16 | 16 |
| RNF40 | O75150 | 1.04E-13 | 9.85E-14 | 0.104102 | | 0.685815 | 1.41737 | | 14.6283 | 14.6351 | 14.5298 | 14.5743 | 14.4281 | 14.32 | 14.5838 | 14.5618 | 17 | 17 | 17 | 17 | 13 | 16 | 14 | 16 |
| PRPF40A | O75400;O7 | 1.22E-15 | 2.04E-15 | 0.104111 | | 0.980515 | 1.91082 | | 16.1937 | 16.2189 | 16.2786 | 16.1562 | 16.2258 | 16.0629 | 16.0139 | 16.1726 | 13 | 13 | 13 | 13 | 10 | 13 | 12 | 12 |
| PAK1IP1 | Q9NWT1 | 9.77E-06 | 2.57E-06 | 0.104574 | + | 1.59403 | 2.95441 | | 16.6401 | 16.7137 | 16.6432 | 16.5226 | 16.5005 | 16.5812 | 16.5695 | 16.6318 | 11 | 9 | 10 | 11 | 6 | 9 | 8 | 7 |
| MRI1 | Q9BV20 | 2.42E-09 | 9.49E-10 | 0.105225 | | 0.147942 | 0.388141 | | 15.552 | 15.5696 | 15.5365 | 15.5115 | 15.756 | 14.6917 | 15.8419 | 15.4362 | 8 | 9 | 8 | 9 | 8 | 7 | 10 | 7 |
| MRPL28 | Q13084 | 4.77E-05 | 1.17E-05 | 0.105577 | | 0.577515 | 1.23057 | | 16.2129 | 16.3047 | 16.3534 | 16.2585 | 16.2406 | 15.9598 | 16.1658 | 16.2991 | 7 | 7 | 7 | 7 | 6 | 6 | 6 | 7 |
| FMC1 | Q96HJ9-2 | 1.33E-11 | 7.56E-12 | 0.105585 | | 0.634267 | 1.32906 | | 16.5873 | 16.7641 | 16.7494 | 16.6621 | 16.5187 | 16.4986 | 16.7626 | 16.5314 | 15 | 17 | 17 | 15 | 13 | 13 | 16 | 15 |
| CTU1 | Q7Z7A3 | 5.81E-05 | 1.42E-05 | 0.105768 | | 0.125184 | 0.334233 | | 14.9644 | 15.0126 | 14.977 | 15.0657 | 13.9487 | 15.1928 | 15.2246 | 14.9626 | 5 | 5 | 5 | 3 | 2 | 3 | 3 | 5 |
| WASHC5 | Q12768 | 1.29E-15 | 2.14E-15 | 0.105996 | | 0.479352 | 1.05599 | | 16.6214 | 16.681 | 16.6267 | 16.5597 | 16.3133 | 16.6844 | 16.7006 | 16.427 | 48 | 49 | 48 | 50 | 42 | 47 | 47 | 45 |
| IRF2BP2 | Q7Z5L9-2 | 6.67E-08 | 2.19E-08 | 0.106037 | | 0.821925 | 1.64686 | | 15.6216 | 15.691 | 15.7082 | 15.5816 | 15.6783 | 15.4146 | 15.5261 | 15.6372 | 12 | 12 | 11 | 12 | 9 | 8 | 10 | 12 |
| DCAF5 | Q96JK2;Q9 | 0.000379 | 8.29E-05 | 0.106166 | | 0.241607 | 0.595428 | | 14.1922 | 14.3849 | 14.3416 | 14.3721 | 13.8342 | 14.531 | 14.2972 | 13.9658 | 3 | 5 | 4 | 3 | 4 | 3 | 3 | 3 |
| MTA1 | Q13330 | 7.18E-08 | 2.35E-08 | 0.106176 | | 0.65528 | 1.36518 | | 15.4045 | 15.3752 | 15.4058 | 15.3795 | 15.3555 | 15.063 | 15.2974 | 15.3496 | 11 | 13 | 15 | 13 | 7 | 9 | 10 | 11 |
| ALDH16A1 | Q8IZ83 | 1.07E-17 | 3.40E-17 | 0.106216 | | 0.170907 | 0.44098 | | 16.1101 | 16.0641 | 16.0335 | 15.9333 | 16.0221 | 16.3215 | 16.239 | 15.2992 | 13 | 13 | 15 | 15 | 13 | 16 | 15 | 13 |
| APEH | P13798 | 7.47E-09 | 2.76E-09 | 0.106219 | | 0.873718 | 1.73329 | | 17.9714 | 18.0402 | 17.9313 | 17.9562 | 17.9515 | 17.9035 | 17.7295 | 17.8331 | 21 | 21 | 21 | 21 | 18 | 20 | 19 | 19 |
| PARN | O95453;O9 | 1.00E-05 | 2.63E-06 | 0.106304 | | 1.1854 | 2.25209 | | 14.9086 | 14.8854 | 15.0129 | 14.8891 | 14.7661 | 14.8317 | 14.7657 | 14.8942 | 9 | 10 | 9 | 8 | 6 | 6 | 7 | 6 |
| ARHGEF11 | O15085;O1 | 7.04E-05 | 1.70E-05 | 0.106487 | | 0.45669 | 1.01474 | | 15.0192 | 15.171 | 15.0587 | 14.737 | 15.0368 | 15.1527 | 15.0766 | 14.8794 | 17 | 15 | 16 | 12 | 11 | 15 | 11 | 12 |
| ZW10 | O43264 | 4.86E-16 | 8.98E-16 | 0.106513 | + | 1.48367 | 2.76045 | | 16.0993 | 16.0605 | 16.0813 | 15.9748 | 16.0142 | 15.8759 | 15.9626 | 16.042 | 23 | 24 | 24 | 23 | 23 | 23 | 22 | 23 |
| SNRPC | P09234 | 1.58E-08 | 5.60E-09 | 0.106534 | | 0.101648 | 0.276666 | | 18.1367 | 18.0281 | 18.5768 | 18.6451 | 17.6232 | 17.3786 | 18.3091 | 18.7472 | 2 | 2 | 2 | 2 | 2 | 2 | 2 | 2 |
| MRPL12 | P52815 | 9.56E-11 | 4.60E-11 | 0.106874 | | 0.074925 | 0.208762 | | 16.9861 | 16.1258 | 17.7321 | 17.6422 | 16.4365 | 16.2636 | 16.4313 | 17.432 | 3 | 3 | 3 | 3 | 3 | 3 | 3 | 3 |
| CNP | P09543 | 1.72E-08 | 6.09E-09 | 0.107078 | + | 1.70147 | 3.1472 | | 17.2212 | 17.1945 | 17.2253 | 17.157 | 17.0514 | 17.1748 | 17.085 | 17.0647 | 27 | 26 | 27 | 26 | 27 | 26 | 26 | 27 |
| SAE1 | Q9UBE0 | 3.20E-22 | 4.22E-21 | 0.107259 | | 1.03294 | 1.9979 | | 16.6458 | 16.6077 | 16.6271 | 16.5674 | 16.3656 | 16.5515 | 16.5878 | 16.5258 | 19 | 20 | 20 | 19 | 16 | 16 | 19 | 20 |
| TTC39B | Q5VTQ0;Q5 | 0.000265 | 5.93E-05 | 0.107342 | | 0.252283 | 0.617827 | | 14.9361 | 14.9901 | 15.0013 | 14.5842 | 14.8444 | 15.3206 | 14.9401 | 14.6534 | 7 | 4 | 5 | 4 | 3 | 6 | 7 | 3 |
| TMEM222 | Q9H0R3 | 4.77E-05 | 1.17E-05 | 0.107668 | | 0.28066 | 0.676366 | | 14.5047 | 14.4651 | 14.6155 | 14.4889 | 14.3275 | 14.8446 | 14.2072 | 14.2355 | 4 | 4 | 4 | 4 | 4 | 4 | 4 | 3 |
| STIM2 | Q9P246;Q9 | 0.002558 | 0.000487 | 0.107738 | | 0.268622 | 0.651705 | | 14.9325 | 14.919 | 14.8204 | 14.9368 | 14.5659 | 15.1904 | 14.6545 | 14.5669 | 5 | 3 | 5 | 4 | 2 | 3 | 4 | 3 |
| MRPS15 | P82914 | 2.97E-14 | 3.28E-14 | 0.107872 | | 0.508943 | 1.10927 | | 15.9201 | 15.9064 | 15.9149 | 16.0281 | 15.6406 | 15.8588 | 15.8529 | 15.6491 | 8 | 8 | 8 | 8 | 6 | 8 | 6 | 8 |
| RBBP5 | Q15291 | 1.78E-10 | 8.24E-11 | 0.107893 | | 0.74986 | 1.52589 | | 15.0013 | 14.9708 | 15.03 | 15.0814 | 14.8151 | 14.7937 | 14.8483 | 14.9256 | 7 | 6 | 6 | 7 | 5 | 5 | 7 | 8 |
| RETREG2 | Q8NC44 | 1.76E-18 | 7.14E-18 | 0.107931 | | 0.192081 | 0.488451 | | 16.1835 | 16.1377 | 16.2973 | 16.2969 | 15.4544 | 16.3213 | 16.2702 | 16.1482 | 5 | 6 | 6 | 6 | 5 | 5 | 6 | 6 |
| RIN1 | Q13671 | 4.83E-13 | 3.79E-13 | 0.10814 | | 0.206434 | 0.520008 | | 16.4701 | 16.5307 | 16.5439 | 16.6548 | 16.851 | 16.1161 | 16.0336 | 16.3783 | 14 | 16 | 14 | 14 | 12 | 11 | 13 | 14 |
| RPL30 | P62888 | 1.84E-10 | 8.49E-11 | 0.108153 | + | 1.45797 | 2.7158 | | 21.1984 | 21.2645 | 21.2363 | 21.1351 | 21.2136 | 21.1203 | 21.0378 | 21.118 | 8 | 7 | 8 | 7 | 8 | 7 | 7 | 8 |
| MTIF3 | Q9H2K0 | 0.002351 | 0.000453 | 0.108229 | | 0.285093 | 0.685387 | | 15.0868 | 15.2675 | 15.21 | 15.4458 | 15.0024 | 15.2242 | 14.9307 | 14.7962 | 2 | 2 | 2 | 2 | 2 | 2 | 1 | 1 |
| NDUFA13 | Q9P0J0 | 1.20E-08 | 4.31E-09 | 0.108327 | | 0.233264 | 0.57777 | | 17.4784 | 17.4714 | 17.5788 | 17.587 | 17.353 | 16.9113 | 17.7344 | 17.4204 | 7 | 7 | 7 | 7 | 4 | 6 | 6 | 6 |
| WASL | O00401 | 9.93E-09 | 3.61E-09 | 0.108585 | | 1.19307 | 2.26496 | | 16.4852 | 16.5905 | 16.531 | 16.5138 | 16.4267 | 16.3795 | 16.4772 | 16.3377 | 11 | 12 | 12 | 11 | 10 | 11 | 10 | 12 |
| WDR77 | Q9BQA1 | 3.65E-12 | 2.31E-12 | 0.108717 | | 0.465599 | 1.031 | | 17.7511 | 17.8175 | 17.8138 | 17.7699 | 17.4329 | 17.9087 | 17.6565 | 17.659 | 11 | 11 | 11 | 11 | 10 | 11 | 11 | 11 |
| BCCIP | Q9P287 | 9.09E-20 | 5.59E-19 | 0.108856 | | 0.353403 | 0.820695 | | 16.8358 | 16.8754 | 16.859 | 17.047 | 16.6889 | 16.5067 | 16.5968 | 16.9001 | 7 | 7 | 8 | 8 | 6 | 8 | 8 | 8 |
| STK17B | O94768 | 9.58E-05 | 2.27E-05 | 0.108949 | | 0.144546 | 0.380199 | | 13.7011 | 13.5442 | 13.5113 | 13.3843 | 14.2008 | 12.9053 | 13.3298 | 13.4526 | 4 | 4 | 4 | 3 | 4 | 2 | 3 | 3 |
| B4GAT1 | O43505 | 1.71E-05 | 4.40E-06 | 0.108951 | | 0.250966 | 0.615077 | | 15.4819 | 15.7774 | 15.5092 | 15.1329 | 15.6564 | 15.3088 | 15.8246 | 15.4798 | 6 | 6 | 6 | 6 | 4 | 5 | 6 | 4 |
| MRPL55 | Q7Z7F7;Q7 | 1.69E-08 | 5.99E-09 | 0.109002 | | 0.470548 | 1.04001 | | 16.5844 | 16.6099 | 16.5449 | 16.4537 | 16.7692 | 16.3162 | 16.4145 | 16.4001 | 4 | 4 | 4 | 4 | 4 | 4 | 4 | 4 |
| FRMD8 | Q9BZ67 | 2.68E-11 | 1.43E-11 | 0.109419 | | 0.297181 | 0.709825 | | 16.5328 | 16.5469 | 16.4739 | 16.693 | 16.3957 | 16.574 | 16.3632 | 16.0164 | 5 | 5 | 5 | 4 | 4 | 6 | 5 | 5 |
| TNPO2 | O14787 | 5.44E-14 | 5.59E-14 | 0.109427 | + | 2.28374 | 4.28044 | | 16.0693 | 16.1645 | 16.1168 | 16.0375 | 16.0354 | 15.9873 | 15.9855 | 15.9915 | 11 | 10 | 10 | 12 | 10 | 11 | 10 | 11 |
| RACK1 | P63244 | 2.04E-11 | 1.12E-11 | 0.109859 | | 0.472386 | 1.04335 | | 21.4636 | 21.5463 | 21.4681 | 21.4978 | 21.1801 | 21.5983 | 21.3949 | 21.2431 | 20 | 20 | 20 | 20 | 20 | 20 | 20 | 20 |
| RANBP1 | P43487;P4 | 5.42E-09 | 2.04E-09 | 0.109882 | | 0.924785 | 1.81825 | | 18.8158 | 18.7681 | 18.6763 | 18.7826 | 18.5857 | 18.6665 | 18.6259 | 18.5568 | 10 | 10 | 10 | 10 | 10 | 10 | 9 | 11 |
| PHB2 | Q99623 | 0.000122 | 2.85E-05 | 0.11017 | | 1.14345 | 2.18192 | | 19.8082 | 19.8484 | 19.796 | 19.7178 | 19.7863 | 19.7064 | 19.5712 | 19.7553 | 13 | 13 | 13 | 13 | 13 | 13 | 13 | 13 |
| PLOD3 | O60568 | 1.32E-11 | 7.49E-12 | 0.110192 | | 0.420288 | 0.947559 | | 19.2633 | 19.2409 | 19.2104 | 19.0788 | 19.1042 | 19.1934 | 19.3996 | 18.864 | 28 | 28 | 28 | 28 | 28 | 29 | 28 | 27 |
| RPS15 | P62841 | 1.51E-06 | 4.29E-07 | 0.110636 | | 0.209402 | 0.526474 | | 18.3801 | 18.3762 | 18.5277 | 18.6405 | 17.9438 | 18.6201 | 17.952 | 18.4303 | 5 | 5 | 4 | 4 | 4 | 4 | 4 | 4 |
| PCF11 | O94913 | 3.31E-07 | 1.02E-07 | 0.110647 | | 0.11177 | 0.301664 | | 13.3267 | 13.511 | 13.9069 | 14.3225 | 13.3816 | 12.7995 | 13.1519 | 13.6989 | 3 | 5 | 5 | 3 | 2 | 4 | 1 | 2 |
| WDFY3 | Q8IZQ1;Q8 | 5.74E-05 | 1.40E-05 | 0.110857 | | 0.425157 | 0.956614 | | 14.2186 | 14.3492 | 14.3418 | 14.0981 | 14.1184 | 14.3817 | 14.3938 | 13.9698 | 17 | 19 | 17 | 12 | 10 | 14 | 13 | 12 |
| PAICS | P22234 | 4.38E-14 | 4.61E-14 | 0.110878 | | 0.562389 | 1.20404 | | 18.9703 | 18.9914 | 18.9852 | 19.127 | 18.7943 | 18.851 | 18.7183 | 18.8663 | 21 | 21 | 21 | 21 | 21 | 21 | 21 | 21 |
| MRPL3 | P09001 | 2.46E-11 | 1.33E-11 | 0.11093 | | 0.740744 | 1.51051 | | 16.6558 | 16.7875 | 16.7207 | 16.5641 | 16.7192 | 16.4392 | 16.6273 | 16.7022 | 3 | 3 | 3 | 3 | 3 | 3 | 3 | 3 |
| SMG1 | Q96Q15;Q9 | 7.15E-07 | 2.11E-07 | 0.110962 | | 0.482367 | 1.06145 | | 14.2875 | 14.2545 | 14.3537 | 14.0702 | 14.0351 | 14.4383 | 14.103 | 14.2914 | 21 | 18 | 19 | 18 | 10 | 16 | 13 | 9 |
| ARPC1B | O15143 | 1.63E-17 | 4.81E-17 | 0.111024 | | 0.487389 | 1.07052 | | 19.2682 | 19.3512 | 19.374 | 19.4094 | 19.0288 | 19.3819 | 19.1815 | 19.0988 | 14 | 13 | 14 | 13 | 13 | 15 | 14 | 13 |
| WDR48 | Q8TAF3 | 9.84E-09 | 3.58E-09 | 0.111263 | | 0.821739 | 1.64655 | | 15.3938 | 15.4117 | 15.3628 | 15.2907 | 15.1903 | 15.4659 | 15.2411 | 15.2031 | 12 | 12 | 11 | 12 | 7 | 9 | 10 | 11 |
| FAM234A | Q9H0X4 | 2.33E-12 | 1.54E-12 | 0.111285 | | 0.290466 | 0.696278 | | 14.924 | 15.157 | 15.0061 | 14.8456 | 14.7267 | 15.3131 | 14.6567 | 14.9779 | 5 | 6 | 7 | 5 | 6 | 5 | 4 | 5 |
| MYO1B | O43795 | 3.66E-15 | 5.30E-15 | 0.111306 | | 0.248961 | 0.610882 | | 18.4539 | 18.5176 | 18.4705 | 18.0394 | 18.5195 | 18.7505 | 18.4615 | 18.0758 | 46 | 46 | 45 | 44 | 46 | 47 | 47 | 47 |
| NCAPD2 | Q15021 | 4.33E-24 | 1.12E-22 | 0.111348 | | 0.199535 | 0.504899 | | 15.8618 | 15.8809 | 15.8851 | 16.1148 | 15.9046 | 15.1618 | 15.6887 | 15.9531 | 38 | 36 | 38 | 38 | 29 | 28 | 36 | 35 |
| TIA1 | P31483 | 8.79E-13 | 6.45E-13 | 0.111797 | | 0.475975 | 1.04987 | | 16.7057 | 16.7064 | 16.613 | 16.5552 | 16.8432 | 16.3694 | 16.4872 | 16.5614 | 6 | 6 | 6 | 6 | 6 | 6 | 6 | 6 |
| OBSL1 | O75147;O7 | 1.96E-06 | 5.52E-07 | 0.111961 | | 0.384946 | 0.881131 | | 14.7941 | 14.724 | 14.8109 | 14.3988 | 14.8027 | 14.4725 | 14.8244 | 14.8235 | 17 | 18 | 15 | 15 | 11 | 12 | 13 | 13 |
| CNOT3 | O75175 | 9.75E-09 | 3.55E-09 | 0.112118 | | 0.829431 | 1.6594 | | 16.0926 | 16.0983 | 16.175 | 16.1489 | 15.9319 | 16.1042 | 15.9129 | 15.9514 | 9 | 10 | 11 | 10 | 9 | 9 | 10 | 10 |
| NBN | O60934 | 1.06E-11 | 6.15E-12 | 0.112334 | | 0.295503 | 0.706445 | | 14.6385 | 14.6394 | 14.6783 | 14.0868 | 14.7516 | 14.5731 | 14.7167 | 14.5706 | 11 | 13 | 14 | 7 | 6 | 11 | 12 | 12 |
| SRSF3 | P84103;P8 | 6.44E-06 | 1.72E-06 | 0.112377 | | 0.545257 | 1.17386 | | 20.6712 | 20.5362 | 20.5774 | 20.334 | 20.6944 | 20.4306 | 20.3677 | 20.586 | 7 | 7 | 7 | 7 | 7 | 7 | 7 | 7 |
| RNASEL | Q05823 | 1.79E-07 | 5.64E-08 | 0.112576 | | 0.17699 | 0.454734 | | 14.6076 | 14.7212 | 14.577 | 14.0891 | 14.3683 | 14.8762 | 15.0359 | 14.2438 | 8 | 7 | 9 | 5 | 5 | 8 | 5 | 3 |
| ATF7 | P17544;P1 | 0.003908 | 0.000711 | 0.112599 | | 0.08139 | 0.227417 | | 14.6041 | 14.7221 | 14.7137 | 15.1575 | 13.3526 | 14.6878 | NaN | 15.0716 | 2 | 2 | 2 | 1 | 1 | 2 | NaN | 1 |
| RAC1 | P63000;P6 | 6.99E-06 | 1.86E-06 | 0.112662 | | 0.571045 | 1.21924 | | 20.049 | 20.0594 | 20.0368 | 20.0161 | 20.1366 | 19.9459 | 19.7334 | 19.8467 | 11 | 11 | 11 | 11 | 11 | 11 | 11 | 11 |
| MRPS7 | Q9Y2R9 | 4.56E-09 | 1.73E-09 | 0.112709 | | 0.657847 | 1.36958 | | 15.9823 | 16.0034 | 16.0015 | 16.0956 | 15.7281 | 15.86 | 15.813 | 15.9185 | 12 | 13 | 13 | 12 | 11 | 11 | 10 | 11 |
| AGL | P35573 | 1.92E-16 | 4.15E-16 | 0.11281 | | 0.292104 | 0.699589 | | 15.9281 | 15.9836 | 15.9518 | 15.6105 | 15.9064 | 16.2733 | 15.7861 | 15.632 | 40 | 42 | 40 | 35 | 33 | 40 | 39 | 34 |
| ABHD4 | Q8TB40 | 0.002411 | 0.000463 | 0.112859 | | 0.158421 | 0.412437 | | 15.2737 | 15.3219 | 14.9443 | 15.2153 | 14.6022 | 15.7406 | 14.8527 | 14.9249 | 4 | 2 | 4 | 2 | 1 | 4 | 2 | 2 |
| CYB5R2 | Q6BCY4 | 1.41E-09 | 5.72E-10 | 0.113058 | | 0.116253 | 0.31262 | | 14.8159 | 14.8029 | 15.0161 | 13.8062 | 14.9567 | 15.3072 | 15.1671 | 14.5889 | 10 | 8 | 8 | 5 | 7 | 9 | 8 | 6 |
| ANKZF1 | Q9H8Y5 | 1.66E-06 | 4.72E-07 | 0.11345 | + | 1.64015 | 3.03667 | | 15.2954 | 15.1971 | 15.2413 | 15.1321 | 15.1189 | 15.2181 | 15.0817 | 15.1049 | 5 | 5 | 5 | 4 | 4 | 4 | 5 | 5 |
| SLC25A12 | O75746 | 3.69E-19 | 1.89E-18 | 0.113581 | | 0.554902 | 1.19087 | | 16.9521 | 16.9581 | 16.9485 | 16.6024 | 17.0179 | 16.8199 | 16.9518 | 16.8044 | 23 | 21 | 22 | 19 | 19 | 18 | 20 | 20 |
| TTC7B | Q86TV6;Q8 | 0.008272 | 0.001434 | 0.113586 | | 0.105038 | 0.287634 | | NaN | 15.0835 | 15.0342 | 15.3525 | 14.095 | 15.11 | 14.8034 | 15.3654 | NaN | 2 | 2 | 2 | 2 | 3 | 2 | 2 |
| RGL2 | O15211 | 0.006805 | 0.001194 | 0.113669 | | 1.14416 | 2.73309 | | 14.8577 | 14.7552 | 14.8401 | 14.7144 | NaN | NaN | NaN | 14.6936 | 3 | 2 | 1 | 2 | NaN | NaN | NaN | 2 |

| Gene names | UniProt IDs | Protein Group P-value | Protein Group Q-value | Log2 Difference NUP214 vs Controls (n=5) | p<0.05 | -log10 p-value | T-test Test statistic | NPC Localization | Log2 MS2_quantity_NUP214_1 | Log2 MS2_quantity_NUP214_2 | Log2 MS2_quantity_NUP214_3 | Log2 MS2_quantity_Control_1 | Log2 MS2_quantity_Control_2 | Log2 MS2_quantity_Control_3 | Log2 MS2_quantity_Control_4 | Log2 MS2_quantity_Control_5 | Number Peptides for Quantitation NUP214_1 | Number Peptides for Quantitation NUP214_2 | Number Peptides for Quantitation NUP214_3 | Number Peptides for Quantitation Control_1 | Number Peptides for Quantitation Control_2 | Number Peptides for Quantitation Control_3 | Number Peptides for Quantitation Control_4 | Number Peptides for Quantitation Control_5 |
|---|---|---|---|---|---|---|---|---|---|---|---|---|---|---|---|---|---|---|---|---|---|---|---|---|
| EDC4 | Q6P2E9 | 5.88E-13 | 4.51E-13 | 0.113678 | | 0.762991 | 1.54801 | | 16.4308 | 16.4435 | 16.4242 | 16.5261 | 16.2521 | 16.2271 | 16.2532 | 16.3374 | 38 | 38 | 38 | 36 | 30 | 36 | 36 | 35 |
| HTT | P42858 | 2.96E-07 | 9.16E-08 | 0.114002 | | 0.656848 | 1.36787 | | 14.9543 | 14.9917 | 15.0667 | 14.9949 | 14.9957 | 16.4794 | 14.9411 | 14.8402 | 34 | 38 | 36 | 35 | 28 | 29 | 35 | 33 |
| DDX42 | Q86XP3 | 9.75E-13 | 7.06E-13 | 0.114215 | + | 1.68133 | 3.11075 | | 16.9291 | 16.9795 | 16.9537 | 16.9093 | 16.8046 | 16.762 | 16.8418 | 16.8819 | 32 | 30 | 31 | 31 | 28 | 30 | 28 | 29 |
| RINT1 | Q6NUQ1 | 9.50E-08 | 3.07E-08 | 0.114294 | | 0.782125 | 1.58017 | | 15.3964 | 15.4383 | 15.4686 | 15.2478 | 15.4928 | 15.3899 | 15.2656 | 15.2047 | 10 | 11 | 12 | 11 | 8 | 11 | 10 | 9 |
| COL4A2 | P08572 | 0.012346 | 0.002096 | 0.11437 | | 0.069128 | 0.201985 | | 14.4 | NaN | 14.2835 | 15.0744 | NaN | 13.9923 | 13.6153 | NaN | 3 | NaN | 2 | 2 | NaN | 3 | 4 | NaN |
| ATP5F1E;ATP5F1EP2 | P56381;Q5 | 0.006385 | 0.001123 | 0.114419 | | 0.28606 | 0.687351 | | 18.3595 | 18.3626 | 18.1589 | 18.291 | 18.1436 | 17.7571 | 18.2245 | 18.48 | 2 | 2 | 2 | 2 | 2 | 2 | 2 | 2 |
| GSTK1 | Q9Y2Q3 | 2.52E-14 | 2.84E-14 | 0.114807 | | 0.518179 | 1.12578 | | 17.7398 | 17.5273 | 17.7434 | 17.5826 | 17.5351 | 17.4237 | 17.4444 | 17.7911 | 13 | 13 | 12 | 12 | 12 | 12 | 13 | 13 |
| RETSAT | Q6NUM9 | 3.91E-12 | 2.45E-12 | 0.115042 | | 0.312661 | 0.740792 | | 15.9544 | 15.9545 | 15.9333 | 16.2682 | 15.8456 | 15.6364 | 15.778 | 15.6336 | 16 | 17 | 19 | 19 | 13 | 16 | 13 | 13 |
| CNOT1 | A5YKK6 | 1.01E-15 | 1.73E-15 | 0.115153 | + | 2.88251 | 5.65691 | | 16.1475 | 16.1281 | 16.1459 | 15.9905 | 16.0599 | 16.0607 | 16.0166 | 15.999 | 61 | 62 | 62 | 60 | 54 | 62 | 59 | 57 |
| ILKAP | Q9H0C8 | 4.09E-20 | 3.00E-19 | 0.115493 | | 0.374102 | 0.860482 | | 15.9159 | 16.0513 | 15.9542 | 16.0072 | 16.0037 | 15.6254 | 15.6114 | 16.0439 | 8 | 8 | 8 | 9 | 9 | 7 | 8 | 10 |
| GLG1 | Q92896;Q5 | 1.86E-16 | 4.05E-16 | 0.1155 | | 1.28613 | 2.42167 | | 17.1232 | 17.1262 | 17.1031 | 16.8974 | 16.9593 | 17.0935 | 17.0658 | 16.9938 | 47 | 47 | 47 | 47 | 40 | 46 | 44 | 45 |
| BMP1 | P13497;P1 | 2.12E-05 | 5.38E-06 | 0.115635 | | 0.119812 | 0.321265 | | 14.3298 | 14.4405 | 14.4338 | 13.3609 | 14.3134 | 14.5904 | 14.9853 | 14.1787 | 4 | 5 | 4 | 1 | 5 | 3 | 4 | 1 |
| ST13 | P50502 | 5.19E-18 | 1.83E-17 | 0.115906 | | 0.279124 | 0.673233 | | 19.3976 | 19.4623 | 19.3931 | 19.6542 | 19.0179 | 19.5642 | 19.1675 | 19.1051 | 10 | 10 | 10 | 10 | 10 | 10 | 10 | 10 |
| CPQ | Q9Y646 | 1.29E-13 | 1.19E-13 | 0.116005 | | 0.101369 | 0.275973 | | 16.8361 | 16.964 | 16.879 | 15.7802 | 17.0191 | 17.4573 | 17.2946 | 16.3339 | 12 | 12 | 11 | 8 | 10 | 12 | 11 | 10 |
| SSRP1 | Q08945 | 2.01E-13 | 1.77E-13 | 0.116123 | | 0.724391 | 1.48287 | | 17.6909 | 17.7008 | 17.7439 | 17.6773 | 17.6132 | 17.3845 | 17.5828 | 17.721 | 19 | 19 | 19 | 19 | 19 | 19 | 19 | 19 |
| HADH | Q16836;Q1 | 2.69E-13 | 2.27E-13 | 0.116159 | | 0.491324 | 1.07762 | | 17.8417 | 17.9289 | 17.8725 | 17.778 | 17.8447 | 17.956 | 17.4756 | 17.77 | 12 | 12 | 13 | 12 | 11 | 13 | 12 | 12 |
| PELP1 | Q8IZL8 | 1.51E-14 | 1.83E-14 | 0.116209 | + | 2.14135 | 3.98741 | | 16.4956 | 16.483 | 16.5149 | 16.3702 | 16.3924 | 16.4558 | 16.3619 | 16.3278 | 15 | 14 | 15 | 14 | 14 | 14 | 14 | 16 |
| TECR | Q9NZ01 | 1.49E-10 | 6.99E-11 | 0.116281 | | 0.414837 | 0.937395 | | 17.7746 | 17.8242 | 17.9246 | 17.496 | 17.6513 | 17.7303 | 18.0458 | 17.7008 | 14 | 12 | 12 | 11 | 12 | 13 | 13 | 13 |
| EIF3D | O15371;O1 | 1.55E-12 | 1.06E-12 | 0.11637 | | 0.49494 | 1.08414 | | 17.8334 | 17.815 | 17.7486 | 17.8882 | 17.755 | 17.5682 | 17.4402 | 17.7616 | 19 | 20 | 20 | 20 | 19 | 19 | 19 | 19 |
| PI4KA | P42356 | 2.14E-11 | 1.17E-11 | 0.116572 | | 0.736086 | 1.50265 | | 15.5995 | 15.6467 | 15.6394 | 15.3723 | 15.4466 | 15.7047 | 15.5701 | 15.4661 | 37 | 38 | 37 | 31 | 27 | 37 | 37 | 31 |
| WDR12 | Q9GZL7 | 7.54E-11 | 3.69E-11 | 0.116729 | | 0.458397 | 1.01786 | | 15.7554 | 15.7078 | 15.7501 | 15.88 | 15.3476 | 15.5731 | 15.6683 | 15.6362 | 11 | 12 | 11 | 13 | 11 | 11 | 13 | 11 |
| DCUN1D1 | Q96GG9 | 9.12E-07 | 2.66E-07 | 0.116859 | | 0.621741 | 1.30745 | | 16.6174 | 16.4842 | 16.53 | 16.4855 | 16.2519 | 16.6277 | 16.3474 | 16.4226 | 6 | 7 | 7 | 7 | 7 | 7 | 6 | 6 |
| ADK | P55263 | 1.79E-16 | 3.94E-16 | 0.116864 | | 0.405775 | 0.920434 | | 16.929 | 16.988 | 16.9697 | 16.5957 | 17.1238 | 16.6898 | 16.8478 | 16.9696 | 22 | 22 | 21 | 21 | 21 | 21 | 22 | 21 |
| FLII | Q13045 | 5.27E-16 | 9.60E-16 | 0.116866 | | 0.454484 | 1.0107 | | 18.013 | 18.052 | 18.0064 | 18.1349 | 17.9564 | 17.6644 | 17.7565 | 18.0224 | 43 | 44 | 43 | 46 | 43 | 40 | 44 | 43 |
| SPNS1 | Q9H2V7;Q5 | 9.11E-07 | 2.66E-07 | 0.116931 | | 0.185533 | 0.473891 | | 17.7431 | 17.7402 | 17.6584 | 17.3968 | 17.2131 | 18.0273 | 18.0584 | 17.2891 | 5 | 5 | 5 | 5 | 4 | 5 | 5 | 5 |
| DDX3X | O00571 | 4.35E-12 | 2.71E-12 | 0.116988 | | 0.304065 | 0.72364 | | 18.7628 | 18.7816 | 18.7192 | 18.3653 | 18.8713 | 18.8173 | 18.3209 | 18.8127 | 11 | 11 | 11 | 11 | 10 | 11 | 11 | 11 |
| NDUFAF2 | Q8N183 | 2.40E-08 | 8.33E-09 | 0.117097 | | 1.0846 | 2.08381 | | 16.9154 | 16.9409 | 16.984 | 16.8951 | 16.6889 | 16.9064 | 16.7893 | 16.8687 | 10 | 10 | 9 | 9 | 7 | 10 | 8 | 9 |
| HSD17B10 | Q99714 | 2.56E-24 | 7.58E-23 | 0.117365 | | 0.663998 | 1.38011 | | 18.4297 | 18.4366 | 18.3516 | 18.2847 | 18.1282 | 18.2408 | 18.2796 | 18.5095 | 15 | 16 | 14 | 15 | 14 | 14 | 15 | 15 |
| PIP4K2C | Q8TBX8 | 1.25E-12 | 8.83E-13 | 0.117614 | | 0.125332 | 0.334591 | | 15.835 | 15.8801 | 15.88 | 14.775 | 15.8614 | 16.0953 | 16.3016 | 15.7037 | 12 | 12 | 12 | 9 | 10 | 12 | 11 | 11 |
| COPS4 | Q9BT78 | 7.24E-11 | 3.56E-11 | 0.117687 | | 0.408901 | 0.926294 | | 17.6414 | 17.6558 | 17.6589 | 17.6023 | 17.5948 | 17.1573 | 17.6623 | 17.6552 | 21 | 21 | 21 | 21 | 21 | 20 | 21 | 21 |
| SAMM50 | Q9Y512 | 2.36E-10 | 1.07E-10 | 0.117956 | | 0.779605 | 1.57594 | | 17.5283 | 17.5712 | 17.52 | 17.3581 | 17.321 | 17.6362 | 17.4049 | 17.3893 | 21 | 21 | 22 | 21 | 19 | 19 | 22 | 22 |
| CORO7 | P57737-3 | 7.93E-06 | 2.10E-06 | 0.117982 | | 0.261203 | 0.636381 | | 16.0911 | 16.1098 | 16.0755 | 16.0527 | 16.3724 | 15.5293 | 15.8513 | 16.065 | 14 | 15 | 16 | 17 | 10 | 9 | 11 | 11 |
| DDX19B | Q9UMR2;Q | 2.66E-24 | 7.71E-23 | 0.117995 | | 1.03052 | 1.99388 | | 17.0479 | 17.0367 | 16.9654 | 16.9378 | 16.7435 | 16.9781 | 16.9545 | 16.8795 | 19 | 18 | 19 | 19 | 16 | 17 | 18 | 17 |
| MRPS25 | P82663 | 7.61E-13 | 5.66E-13 | 0.118045 | | 0.280243 | 0.675515 | | 15.496 | 15.5446 | 15.4152 | 15.4497 | 14.878 | 15.6496 | 15.4551 | 15.4039 | 5 | 5 | 6 | 6 | 4 | 5 | 6 | 5 |
| VIRMA | Q69YN4;Q6 | 1.52E-19 | 8.82E-19 | 0.118115 | | 0.596946 | 1.26446 | | 15.0641 | 15.0296 | 14.8866 | 14.8076 | 14.992 | 14.7427 | 14.7737 | 15.0606 | 14 | 16 | 15 | 16 | 9 | 10 | 11 | 11 |
| THOC2 | Q8NI27 | 3.09E-10 | 1.37E-10 | 0.118225 | | 0.80044 | 1.61089 | | 15.4047 | 15.3834 | 15.3478 | 15.3307 | 15.3557 | 15.0769 | 15.1949 | 15.3439 | 26 | 28 | 21 | 16 | 18 | 16 | 15 | 21 |
| SLC66A3 | Q8N755;Q | 0.000165 | 3.80E-05 | 0.118235 | | 0.333053 | 0.781062 | | 17.2896 | 17.333 | 17.3641 | 17.4675 | 17.2367 | 16.9452 | 16.9593 | 17.4445 | 2 | 2 | 2 | 2 | 2 | 2 | 2 | 2 |
| SNAP47 | Q5SQN1;Q5 | 0.001842 | 0.000364 | 0.11835 | | 0.194396 | 0.493574 | | 15.1141 | 14.4446 | 15.1612 | 14.3011 | 15.0322 | 14.9433 | 14.8515 | 14.8133 | 1 | 2 | 1 | 2 | 1 | 2 | 1 | 1 |
| DLG5 | Q8TDM6;Q | 0.001387 | 0.00028 | 0.118436 | | 1.21218 | 2.29702 | | 15.2855 | 15.3573 | 15.3731 | 15.1937 | 15.3562 | 15.1446 | 15.197 | 15.2096 | 14 | 15 | 14 | 14 | 11 | 9 | 12 | 12 |
| RBM10 | P98175;P9 | 1.11E-12 | 7.94E-13 | 0.118591 | + | 1.37501 | 2.57292 | | 15.6214 | 15.7303 | 15.7129 | 15.6448 | 15.5576 | 15.4817 | 15.5416 | 15.6221 | 18 | 18 | 20 | 18 | 13 | 17 | 15 | 17 |
| CTSD | P07339 | 8.34E-07 | 2.45E-07 | 0.118635 | | 0.189832 | 0.483462 | | 20.3017 | 20.3204 | 20.3002 | 19.6005 | 20.1448 | 20.224 | 20.7608 | 20.2139 | 18 | 18 | 18 | 17 | 18 | 18 | 18 | 18 |
| SSBP1 | Q04837 | 4.73E-13 | 3.72E-13 | 0.1187 | | 0.628751 | 1.31955 | | 18.7282 | 18.6223 | 18.5813 | 18.5568 | 18.5503 | 18.6723 | 18.2901 | 18.5566 | 7 | 7 | 7 | 7 | 7 | 7 | 7 | 7 |
| SH3RF3 | Q8TEJ3 | 0.018397 | 0.003072 | 0.11895 | | 0.172889 | 0.445472 | | 16.2228 | 16.5197 | 16.6577 | 16.9132 | 15.7738 | 16.2045 | 16.529 | 16.3183 | 2 | 2 | 2 | 2 | 1 | 2 | 2 | 2 |
| MRPL30 | Q8TCC3;Q8 | 0.000573 | 0.000122 | 0.118972 | | 0.459142 | 1.01922 | | 14.1424 | 14.3825 | 14.3324 | 14.3621 | 13.8982 | 14.2682 | 14.163 | 14.1423 | 3 | 3 | 3 | 3 | 3 | 3 | 3 | 3 |
| MAEA | Q7L5Y9 | 1.29E-06 | 3.71E-07 | 0.119096 | | 0.632963 | 1.32681 | | 14.4136 | 14.5425 | 14.4471 | 14.3632 | 14.5821 | 14.3318 | 14.2347 | 14.2314 | 6 | 5 | 4 | 6 | 2 | 5 | 4 | 5 |
| BABAM2 | Q9NXR7 | 0.000278 | 6.20E-05 | 0.119433 | | 0.494682 | 1.08367 | | 16.3468 | 16.3695 | 16.3953 | 15.9982 | 16.2284 | 16.4796 | 16.3688 | 16.1806 | 10 | 10 | 11 | 7 | 7 | 9 | 10 | 7 |
| STXBP3 | O00186 | 3.10E-16 | 6.10E-16 | 0.119456 | | 0.759605 | 1.54231 | | 16.2508 | 16.3002 | 16.299 | 16.0788 | 16.2217 | 16.3271 | 16.1945 | 15.9973 | 22 | 21 | 22 | 22 | 22 | 21 | 23 | 20 |
| ETFB | P38117 | 1.25E-09 | 5.12E-10 | 0.119548 | | 0.736983 | 1.50416 | | 18.8504 | 18.8106 | 18.7998 | 18.6603 | 18.7025 | 18.7753 | 18.507 | 18.8585 | 18 | 17 | 17 | 18 | 18 | 17 | 17 | 18 |
| ABI2 | Q9NYB9;Q5 | 4.93E-19 | 2.42E-18 | 0.119634 | | 1.26988 | 2.39419 | | 15.9254 | 15.8217 | 15.9262 | 15.8678 | 15.7007 | 15.7422 | 15.8267 | 15.7199 | 5 | 5 | 5 | 5 | 4 | 5 | 5 | 5 |
| ASF1A | Q9Y294 | 1.20E-05 | 3.12E-06 | 0.120115 | | 0.545619 | 1.1745 | | 14.932 | 15.0929 | 15.1591 | 14.9842 | 14.7761 | 14.9236 | 15.1711 | 14.8511 | 3 | 3 | 2 | 3 | 3 | 3 | 3 | 3 |
| SHC1 | P29353;P2 | 3.30E-14 | 3.58E-14 | 0.12012 | + | 1.38137 | 2.58381 | | 16.7694 | 16.7314 | 16.743 | 16.5945 | 16.5989 | 16.5662 | 16.6184 | 16.761 | 15 | 14 | 14 | 14 | 13 | 15 | 14 | 15 |
| CYP20A1 | Q6UW02 | 1.78E-13 | 1.57E-13 | 0.120386 | | 0.355648 | 0.825035 | | 15.6601 | 15.7474 | 15.7464 | 15.4782 | 15.4097 | 15.923 | 15.7871 | 15.3899 | 14 | 12 | 12 | 12 | 8 | 14 | 12 | 12 |
| NDUFS1 | P28331;P2 | 8.73E-23 | 1.48E-21 | 0.120426 | | 1.16621 | 2.21996 | | 17.9762 | 17.9923 | 17.9933 | 17.8302 | 17.8665 | 17.9912 | 17.9017 | 17.7448 | 30 | 30 | 29 | 27 | 28 | 28 | 29 | 30 |
| ACTG1 | P63261 | 2.28E-12 | 1.51E-12 | 0.12072 | | 0.331296 | 0.777614 | | 22.7382 | 22.329 | 22.4944 | 22.0631 | 22.354 | 22.4855 | 22.4495 | 22.6469 | 2 | 2 | 2 | 1 | 1 | 1 | 2 | 1 |
| MRPL38 | Q96DV4 | 1.94E-11 | 1.07E-11 | 0.120909 | | 0.662727 | 1.37794 | | 17.3369 | 17.2971 | 17.2378 | 17.1152 | 17.3484 | 17.0214 | 17.0711 | 17.2923 | 9 | 9 | 10 | 8 | 10 | 9 | 9 | 9 |
| SDHB | P21912 | 2.87E-10 | 1.28E-10 | 0.120948 | | 0.603243 | 1.2754 | | 17.9222 | 17.9855 | 17.9579 | 17.9029 | 18.0155 | 17.8975 | 17.6123 | 17.743 | 10 | 11 | 10 | 11 | 10 | 11 | 11 | 10 |
| SART1 | O43290 | 4.07E-11 | 2.09E-11 | 0.121002 | | 0.869383 | 1.72606 | | 16.0572 | 16.117 | 16.1572 | 16.1367 | 15.8747 | 15.8981 | 15.9673 | 16.0704 | 27 | 28 | 27 | 25 | 24 | 27 | 25 | 24 |
| ANXA4 | P09525 | 3.74E-11 | 1.94E-11 | 0.121013 | | 0.394963 | 0.900088 | | 19.7752 | 19.8349 | 19.8039 | 19.4796 | 19.5337 | 19.8712 | 19.9772 | 19.5568 | 25 | 25 | 24 | 25 | 24 | 25 | 24 | 24 |
| AKTIP | Q9H8T0;Q5 | 2.91E-10 | 1.29E-10 | 0.121399 | | 0.288422 | 0.692139 | | 15.0659 | 14.7076 | 14.7941 | 14.9791 | 14.4787 | 15.0398 | 14.5023 | 14.6725 | 4 | 4 | 6 | 5 | 2 | 4 | 3 | 4 |
| RTCB | Q9Y3I0 | 2.24E-16 | 4.69E-16 | 0.12167 | | 0.870395 | 1.72775 | | 18.0093 | 18.0447 | 18.0501 | 17.9054 | 17.7436 | 18.0739 | 17.9167 | 17.9255 | 29 | 30 | 28 | 29 | 28 | 30 | 29 | 29 |
| SLC25A51 | Q9H1U9 | 0.003107 | 0.000577 | 0.121673 | | 0.434792 | 0.974467 | | 15.8854 | 15.8768 | 15.654 | 15.4981 | 15.7034 | 15.9836 | 15.5567 | 15.6766 | 2 | 3 | 2 | 3 | 2 | 2 | 2 | 2 |
| NXN | Q6DKJ4 | 1.15E-17 | 3.60E-17 | 0.121705 | | 0.477455 | 1.05255 | | 17.1318 | 17.2158 | 17.1517 | 17.3158 | 17.0243 | 16.7774 | 17.0304 | 17.0757 | 19 | 20 | 20 | 20 | 18 | 18 | 18 | 19 |
| GPT2 | Q8TD30 | 8.07E-13 | 5.95E-13 | 0.121875 | | 0.09062 | 0.248992 | | 14.1272 | 14.3021 | 14.0369 | 13.2805 | 13.8198 | 15.0527 | 14.7166 | 13.298 | 8 | 7 | 9 | 6 | 3 | 11 | 10 | 4 |
| NRAS | P01111 | 1.03E-13 | 9.82E-14 | 0.121936 | | 1.03305 | 1.99808 | | 18.4331 | 18.4595 | 18.4155 | 18.2645 | 18.2697 | 18.4564 | 18.3768 | 18.2032 | 8 | 8 | 8 | 7 | 7 | 8 | 8 | 7 |
| AHCYL1 | O43865 | 4.20E-15 | 5.96E-15 | 0.121967 | | 0.487646 | 1.07099 | | 17.6501 | 17.618 | 17.5976 | 17.3339 | 17.587 | 17.786 | 17.4513 | 17.3413 | 22 | 22 | 22 | 19 | 21 | 22 | 21 | 22 |
| OSBPL5 | Q9H0X9 | 1.39E-08 | 4.97E-09 | 0.121967 | | 0.718025 | 1.47209 | | 16.2175 | 16.1588 | 16.2122 | 16.1014 | 16.287 | 16.0144 | 15.916 | 16.0523 | 15 | 17 | 17 | 17 | 13 | 15 | 14 | 14 |
| RPRD1B | Q9NQG5 | 0.000187 | 4.26E-05 | 0.122117 | + | 2.63617 | 5.05979 | | 17.2212 | 17.2597 | 17.2546 | 17.1268 | 17.1527 | 17.0726 | 17.0994 | 17.164 | 7 | 7 | 6 | 6 | 4 | 6 | 7 | 7 |
| NDUFA7 | O95182 | 4.58E-06 | 1.24E-06 | 0.122188 | | 0.34633 | 0.806979 | | 16.577 | 16.7823 | 16.7459 | 16.759 | 16.4578 | 16.3878 | 16.9123 | 16.3809 | 7 | 6 | 7 | 7 | 7 | 7 | 7 | 7 |
| C4A | P0C0L4;P0 | 8.73E-07 | 2.55E-07 | 0.122222 | | 0.474176 | 1.0466 | | 18.1633 | 18.1179 | 18.1369 | 18.2378 | 17.7364 | 18.1623 | 17.984 | 17.9653 | 11 | 11 | 10 | 11 | 11 | 11 | 11 | 11 |
| NUDC | Q9Y266 | 4.35E-11 | 2.23E-11 | 0.122314 | | 0.63781 | 1.33516 | | 17.4432 | 17.4301 | 17.4156 | 17.5592 | 17.1527 | 17.265 | 17.2388 | 17.321 | 21 | 21 | 21 | 22 | 19 | 21 | 22 | 20 |
| SBF1 | O95248;O9 | 4.74E-15 | 6.63E-15 | 0.12232 | | 0.703025 | 1.44665 | | 15.5609 | 15.55 | 15.3787 | 15.2787 | 15.3951 | 15.492 | 15.4853 | 15.22 | 21 | 23 | 22 | 19 | 16 | 19 | 22 | 18 |
| TMPO | P42166 | 3.64E-10 | 1.59E-10 | 0.122414 | | 0.459363 | 1.01962 | | 16.2061 | 16.274 | 16.2386 | 16.371 | 15.9949 | 15.8651 | 16.108 | 16.2469 | 16 | 15 | 14 | 16 | 14 | 15 | 15 | 15 |
| ZFYVE26 | Q68DK2;Q | 0.003028 | 0.000564 | 0.122547 | | 0.058762 | 0.167621 | | 16.0157 | 15.9759 | 16.2158 | 14.1932 | NaN | 16.9896 | 16.5605 | 16.0432 | 5 | 3 | 3 | 2 | NaN | 3 | 1 | 2 |
| MECR | Q9BV79 | 3.40E-05 | 8.47E-06 | 0.122797 | | 0.919871 | 1.81008 | | 15.3237 | 15.2924 | 15.3394 | 15.0697 | 15.2363 | 15.3668 | 15.1434 | 15.1623 | 5 | 3 | 4 | 4 | 1 | 4 | 3 | 3 |
| KLHL22 | Q53GT1 | 4.55E-13 | 3.60E-13 | 0.123034 | | 1.02788 | 1.98949 | | 15.8172 | 15.8142 | 15.882 | 15.6532 | 15.8558 | 15.7428 | 15.5908 | 15.7312 | 13 | 14 | 9 | 13 | 6 | 11 | 13 | 11 |
| NDUFAF7 | Q7L592 | 0.000327 | 7.22E-05 | 0.123045 | | 0.579173 | 1.23346 | | 14.7957 | 14.5688 | 14.7768 | 14.6001 | 14.7761 | 14.5218 | 14.3991 | 14.6566 | 9 | 7 | 7 | 6 | 7 | 5 | 5 | 4 |
| CDKAL1 | Q5VV42;Q5 | 8.45E-06 | 2.24E-06 | 0.123063 | | 0.652924 | 1.36114 | | 15.143 | 15.1891 | 15.0991 | 15.1033 | 15.025 | 15.0179 | 14.7817 | 15.1755 | 6 | 7 | 6 | 6 | 3 | 4 | 5 | 4 |
| USP32 | Q8NFA0 | 7.55E-07 | 2.23E-07 | 0.123314 | | 0.344873 | 0.804147 | | 14.7448 | 14.8366 | 14.7439 | 14.9751 | 14.7428 | 14.3104 | 14.4995 | 14.7311 | 10 | 11 | 13 | 14 | 7 | 11 | 9 | 8 |

| Gene names | UniProt IDs | Protein Group P-value | Protein Group Q-value | Log2 Difference NUP214 vs Controls (n=5) | p<0.05 | -log10 p-value | T-test Test statistic | NPC Localization | Log2 MS2_quantity_ NUP214_1 | Log2 MS2_quantity_ NUP214_2 | Log2 MS2_quantity_ NUP214_3 | Log2 MS2_quantity_ Control_1 | Log2 MS2_quantity_ Control_2 | Log2 MS2_quantity_ Control_3 | Log2 MS2_quantity_ Control_4 | Log2 MS2_quantity_ Control_5 | Number Peptides for Quantitation NUP214_1 | Number Peptides for Quantitation NUP214_2 | Number Peptides for Quantitation NUP214_3 | Number Peptides for Quantitation Control_1 | Number Peptides for Quantitation Control_2 | Number Peptides for Quantitation Control_3 | Number Peptides for Quantitation Control_4 | Number Peptides for Quantitation Control_5 |
|---|---|---|---|---|---|---|---|---|---|---|---|---|---|---|---|---|---|---|---|---|---|---|---|---|
| MRPL37 | Q9BZE1 | 1.76E-11 | 9.81E-12 | 0.123571 | | 0.76099 | 1.54464 | | 16.6003 | 16.5904 | 16.568 | 16.413 | 16.5941 | 16.2579 | 16.4908 | 16.5574 | 16 | 17 | 18 | 18 | 15 | 15 | 16 | 15 |
| RNF213 | Q63HN8;Q | 1.47E-13 | 1.33E-13 | 0.123595 | | 1.00602 | 1.95318 | | 15.5794 | 15.5995 | 15.575 | 15.5818 | 15.4486 | 15.5109 | 15.2954 | 15.4686 | 118 | 114 | 114 | 116 | 91 | 109 | 106 | 101 |
| MRPL45 | Q9BRJ2 | 1.04E-08 | 3.77E-09 | 0.123638 | | 1.05564 | 2.03563 | | 16.153 | 16.2483 | 16.1566 | 16.1694 | 16.0228 | 16.0654 | 15.9267 | 16.1273 | 12 | 11 | 11 | 12 | 12 | 10 | 13 | 12 |
| GORAB | Q5T7V8 | 1.57E-05 | 4.04E-06 | 0.123687 | | 0.317949 | 0.751291 | | 15.5318 | 15.6659 | 15.5339 | 15.3391 | 15.3466 | 15.118 | 15.7537 | 15.7102 | 4 | 3 | 5 | 2 | 4 | 3 | 5 | 5 |
| SIRT2 | Q8IXJ6 | 5.26E-05 | 1.29E-05 | 0.123713 | | 0.940151 | 1.84378 | | 15.9407 | 15.8877 | 15.8997 | 15.6416 | 15.7666 | 15.9359 | 15.7404 | 15.8437 | 10 | 11 | 9 | 9 | 9 | 11 | 10 | 9 |
| RAP2A | P10114 | 0.000385 | 8.41E-05 | 0.123724 | | 0.922766 | 1.81489 | | 17.1203 | 17.0967 | 17.1251 | 16.8373 | 17.015 | 17.0825 | 17.1049 | 16.9118 | 5 | 5 | 6 | 5 | 5 | 5 | 7 | 6 |
| PLEKHA2 | Q9HB19 | 2.21E-10 | 1.01E-10 | 0.123726 | | 0.282263 | 0.67963 | | 14.9782 | 14.8127 | 14.885 | 15.2462 | 14.6149 | 14.4688 | 14.8482 | 14.663 | 6 | 6 | 6 | 6 | 4 | 5 | 6 | 5 |
| MAPK14 | Q16539 | 5.27E-08 | 1.76E-08 | 0.123741 | | 1.06091 | 2.04439 | | 16.7082 | 16.6804 | 16.6623 | 16.4765 | 16.6667 | 16.519 | 16.4688 | 16.6685 | 14 | 14 | 14 | 13 | 13 | 14 | 14 | 14 |
| BLVRA | P53004 | 6.55E-10 | 2.77E-10 | 0.123839 | | 0.387639 | 0.886238 | | 18.494 | 18.5497 | 18.5449 | 18.6967 | 18.6064 | 18.1742 | 18.2243 | 18.3267 | 18 | 17 | 16 | 16 | 18 | 15 | 15 | 17 |
| PCNA | P12004 | 6.14E-07 | 1.83E-07 | 0.123844 | | 0.34429 | 0.803013 | | 18.8683 | 18.8157 | 18.827 | 18.9676 | 18.753 | 18.3651 | 18.5436 | 18.9365 | 12 | 12 | 12 | 12 | 11 | 11 | 11 | 12 |
| FBXO3 | Q9UK99;Q | 0.000614 | 0.00013 | 0.123896 | | 0.342235 | 0.79901 | | 14.8797 | 15.0662 | 14.9927 | 15.1225 | 15.0369 | 14.6326 | 14.5511 | 14.9351 | 4 | 5 | 5 | 6 | 4 | 5 | 5 | 3 |
| TWF2 | Q6IBS0 | 1.68E-13 | 1.50E-13 | 0.12425 | | 0.520387 | 1.12972 | | 18.422 | 18.3907 | 18.4153 | 18.0478 | 18.4466 | 18.3986 | 18.4069 | 18.1256 | 16 | 16 | 16 | 16 | 16 | 16 | 17 | 16 |
| LTBP2 | Q14767 | 6.72E-18 | 2.29E-17 | 0.124252 | | 0.402466 | 0.914221 | | 15.4766 | 15.4412 | 15.5586 | 15.086 | 15.364 | 15.6172 | 15.5568 | 15.2154 | 14 | 14 | 14 | 11 | 12 | 15 | 14 | 12 |
| ALG2 | Q9H553 | 8.27E-11 | 4.02E-11 | 0.124252 | | 0.650308 | 1.35665 | | 16.4433 | 16.4381 | 16.4488 | 16.2271 | 16.1597 | 16.5278 | 16.4299 | 16.2512 | 14 | 14 | 14 | 12 | 12 | 14 | 14 | 13 |
| SELENOT | P62341 | 0.000754 | 0.000158 | 0.124299 | | 0.257539 | 0.628777 | | 16.5075 | 16.1205 | 16.1858 | 15.9112 | 16.2727 | 15.7716 | 16.4984 | 16.281 | 1 | 2 | 1 | 2 | 3 | 2 | 3 | 3 |
| AMDHD2 | Q9Y303 | 4.88E-10 | 2.10E-10 | 0.124423 | | 0.360425 | 0.83425 | | 16.366 | 16.2598 | 16.2239 | 16.4401 | 16.2345 | 16.1152 | 15.7747 | 16.2297 | 9 | 10 | 10 | 10 | 8 | 9 | 9 | 10 |
| LONP1 | P36776;P3 | 7.18E-18 | 2.39E-17 | 0.124428 | | 0.162531 | 0.421881 | | 17.2555 | 17.2182 | 17.2167 | 16.5418 | 16.8664 | 17.8038 | 17.3922 | 16.9241 | 36 | 37 | 36 | 34 | 33 | 35 | 36 | 33 |
| STK38 | Q15208 | 5.85E-11 | 2.94E-11 | 0.124567 | | 0.817937 | 1.64019 | | 16.2633 | 16.2879 | 16.2681 | 16.0839 | 16.0042 | 16.3424 | 16.1864 | 16.1257 | 12 | 14 | 13 | 12 | 12 | 14 | 12 | 14 |
| RAPH1 | Q70E73 | 2.62E-12 | 1.71E-12 | 0.124594 | | 0.32466 | 0.764558 | | 15.9184 | 15.8894 | 15.9768 | 15.5695 | 15.7137 | 16.2223 | 15.5949 | 15.9177 | 17 | 19 | 18 | 19 | 16 | 19 | 16 | 18 |
| GAMT | Q14353 | 2.16E-11 | 1.18E-11 | 0.1248 | | 0.296176 | 0.707802 | | 16.6424 | 16.558 | 16.7721 | 16.642 | 16.1132 | 16.8958 | 16.5581 | 16.4544 | 6 | 6 | 5 | 6 | 6 | 6 | 6 | 6 |
| NCAPG | Q9BPX3 | 6.14E-10 | 2.61E-10 | 0.124809 | | 0.373412 | 0.859165 | | 16.0951 | 16.1565 | 16.0813 | 16.2012 | 16.0586 | 15.5993 | 15.9171 | 16.1544 | 21 | 21 | 21 | 22 | 15 | 15 | 16 | 17 |
| SMCHD1 | A6NHR9 | 1.92E-12 | 1.29E-12 | 0.124811 | | 1.05904 | 2.04128 | | 15.8372 | 15.8588 | 15.8374 | 15.6813 | 15.871 | 15.593 | 15.7017 | 15.7513 | 40 | 40 | 41 | 33 | 25 | 28 | 28 | 36 |
| ECHS1 | P30084 | 1.43E-05 | 3.69E-06 | 0.124812 | | 0.577852 | 1.23115 | | 17.1768 | 17.1593 | 17.1625 | 17.2739 | 16.942 | 17.0279 | 16.833 | 17.1302 | 15 | 15 | 15 | 15 | 15 | 15 | 15 | 15 |
| FZD2 | Q14332 | 3.75E-15 | 5.42E-15 | 0.125153 | | 0.259497 | 0.632844 | | 15.5572 | 15.4308 | 15.4379 | 14.8094 | 15.4189 | 15.6059 | 15.6066 | 15.31 | 3 | 3 | 3 | 3 | 3 | 3 | 3 | 3 |
| ETHE1 | O95571 | 5.22E-15 | 7.17E-15 | 0.12548 | | 0.328475 | 0.772072 | | 18.506 | 18.5208 | 18.529 | 18.5158 | 18.5079 | 18.025 | 18.708 | 18.2088 | 10 | 11 | 11 | 11 | 9 | 11 | 10 | 11 |
| WIZ | O95785-3 | 7.25E-06 | 1.93E-06 | 0.125577 | | 0.385537 | 0.882252 | | 14.2773 | 14.154 | 14.3913 | 14.3683 | 14.1298 | 13.7789 | 14.2226 | 14.2435 | 9 | 9 | 6 | 11 | 6 | 5 | 8 | 6 |
| RBM15 | Q96T37;Q9 | 1.26E-07 | 4.03E-08 | 0.125619 | | 0.814422 | 1.63431 | | 15.0717 | 15.0974 | 14.9775 | 14.7679 | 14.9218 | 14.9276 | 15.1061 | 14.8928 | 14 | 16 | 17 | 13 | 12 | 14 | 12 | 11 |
| RCC1L | Q96I51 | 1.18E-05 | 3.08E-06 | 0.125713 | | 0.728757 | 1.49026 | | 15.0928 | 15.048 | 15.1449 | 15.1804 | 14.8966 | 14.8807 | 14.8541 | 15.0355 | 9 | 9 | 10 | 10 | 5 | 10 | 6 | 8 |
| NUDT5 | Q9UKK9 | 5.21E-09 | 1.96E-09 | 0.125772 | | 0.474075 | 1.04642 | | 17.9239 | 17.9201 | 17.8972 | 17.648 | 17.5984 | 18.0862 | 17.8962 | 17.7111 | 13 | 13 | 13 | 13 | 11 | 13 | 13 | 13 |
| DNAAF5 | Q86Y56 | 3.87E-14 | 4.13E-14 | 0.125867 | | 0.528183 | 1.14359 | | 15.8105 | 15.8945 | 15.8187 | 15.4249 | 15.8958 | 15.7941 | 15.6663 | 15.7958 | 20 | 21 | 20 | 18 | 18 | 19 | 17 | 19 |
| TTC5 | Q8N0Z6 | 7.00E-14 | 6.96E-14 | 0.125987 | + | 1.34878 | 2.5281 | | 15.2627 | 15.3255 | 15.2496 | 15.1433 | 15.2247 | 15.215 | 15.0282 | 15.1552 | 10 | 9 | 8 | 12 | 10 | 11 | 11 | 6 |
| NIT1 | Q86X76;Q | 4.05E-14 | 4.30E-14 | 0.126284 | | 1.20734 | 2.28889 | | 17.0792 | 17.1097 | 17.1509 | 16.9097 | 16.9049 | 17.1231 | 17.0068 | 16.9903 | 14 | 13 | 13 | 12 | 12 | 12 | 12 | 12 |
| RPS12 | P25398 | 2.37E-10 | 1.07E-10 | 0.12633 | | 0.778134 | 1.57347 | | 20.2869 | 20.2362 | 20.3301 | 20.0458 | 20.3736 | 20.0965 | 20.184 | 20.0904 | 12 | 11 | 12 | 10 | 12 | 11 | 13 | 11 |
| CBX3 | Q13185 | 1.68E-10 | 7.81E-11 | 0.126447 | | 0.799401 | 1.60915 | | 17.5498 | 17.5337 | 17.6019 | 17.5464 | 17.4763 | 17.2194 | 17.5133 | 17.4212 | 8 | 8 | 8 | 8 | 8 | 7 | 8 | 8 |
| SETX | Q7Z333;Q7 | 0.005194 | 0.000926 | 0.126697 | | 0.469333 | 1.0378 | | 14.7021 | 14.7986 | 14.5024 | 14.7652 | 14.5059 | 14.6503 | 14.3079 | 14.4756 | 4 | 3 | 2 | 2 | 2 | 3 | 2 | 2 |
| CAD | P27708 | 3.88E-13 | 3.14E-13 | 0.126777 | | 1.19431 | 2.26704 | | 17.7673 | 17.7956 | 17.7261 | 17.7371 | 17.5035 | 17.6697 | 17.5916 | 17.6791 | 61 | 59 | 62 | 60 | 60 | 61 | 61 | 61 |
| DHX36 | Q9H2U1 | 2.83E-17 | 7.79E-17 | 0.126822 | | 0.820923 | 1.64518 | | 16.1285 | 16.2055 | 16.1433 | 16.1132 | 15.9094 | 16.1158 | 16.1419 | 15.881 | 24 | 26 | 24 | 26 | 20 | 25 | 22 | 23 |
| DHRSX | Q8N5I4 | 2.48E-09 | 9.73E-10 | 0.126907 | | 0.168208 | 0.434847 | | 15.2127 | 15.2209 | 15.1879 | 14.314 | 15.2945 | 14.8953 | 15.5457 | 15.3517 | 4 | 3 | 4 | 4 | 3 | 3 | 4 | 5 |
| TTC28 | Q96AY4 | 2.75E-20 | 2.14E-19 | 0.127122 | | 0.485664 | 1.06741 | | 15.0676 | 15.1084 | 15.0874 | 15.1439 | 15.1899 | 14.8128 | 14.7395 | 14.9172 | 22 | 24 | 23 | 19 | 15 | 13 | 13 | 19 |
| CRNKL1 | Q9BZJ0;Q9 | 1.36E-12 | 9.50E-13 | 0.127212 | + | 2.25294 | 4.21607 | | 15.6232 | 15.666 | 15.6113 | 15.4402 | 15.4779 | 15.5253 | 15.5343 | 15.5538 | 11 | 16 | 19 | 18 | 9 | 15 | 15 | 14 |
| MSH2 | P43246 | 6.89E-13 | 5.17E-13 | 0.127318 | | 0.393644 | 0.897599 | | 16.0068 | 16.0546 | 16.0059 | 15.9371 | 15.9475 | 15.5532 | 15.8282 | 16.2096 | 25 | 23 | 27 | 23 | 22 | 19 | 24 | 23 |
| PRPF38A | Q8NAV1 | 8.96E-07 | 2.62E-07 | 0.127446 | | 0.659057 | 1.37165 | | 15.6704 | 15.659 | 15.694 | 15.4016 | 15.7982 | 15.4427 | 15.5206 | 15.5721 | 4 | 4 | 5 | 4 | 4 | 5 | 5 | 4 |
| FAHD2A | Q96GK7 | 4.52E-11 | 2.31E-11 | 0.12751 | | 0.282663 | 0.680446 | | 17.0637 | 17.0339 | 17.0826 | 16.9729 | 16.3897 | 17.0566 | 17.049 | 17.1946 | 12 | 12 | 12 | 12 | 12 | 12 | 12 | 12 |
| NONO | Q15233 | 3.75E-13 | 3.05E-13 | 0.127517 | | 1.10517 | 2.11806 | | 18.4882 | 18.5049 | 18.483 | 18.5092 | 18.3973 | 18.2678 | 18.2698 | 18.3784 | 27 | 26 | 27 | 26 | 29 | 27 | 26 | 27 |
| MRPS36 | P82909 | 3.23E-08 | 1.10E-08 | 0.127954 | | 0.421283 | 0.949412 | | 16.4069 | 16.4706 | 16.3979 | 16.621 | 16.2952 | 16.1312 | 16.051 | 16.3875 | 5 | 5 | 5 | 5 | 5 | 5 | 5 | 5 |
| SMARCA4 | P51532;P5 | 2.96E-22 | 3.99E-21 | 0.128 | | 0.49276 | 1.08021 | | 15.3017 | 15.3534 | 15.3228 | 15.4463 | 15.2484 | 14.9803 | 15.0129 | 15.3018 | 16 | 19 | 20 | 22 | 14 | 17 | 19 | 23 |
| NMNAT1 | Q9HAN9 | 0.000285 | 6.34E-05 | 0.128196 | | 0.43525 | 0.975315 | | 16.238 | 16.2509 | 16.2453 | 16.451 | 15.8648 | 16.0162 | 16.1919 | 16.0588 | 7 | 5 | 7 | 6 | 3 | 7 | 7 | 5 |
| CBR3 | O75828 | 2.54E-11 | 1.36E-11 | 0.128313 | | 0.352163 | 0.818294 | | 17.6387 | 17.6495 | 17.6525 | 17.1697 | 17.8736 | 17.4722 | 17.4251 | 17.6523 | 14 | 14 | 14 | 14 | 14 | 14 | 14 | 14 |
| EHBP1 | Q8NDI1;Q8 | 5.18E-07 | 1.56E-07 | 0.12832 | | 0.509237 | 1.1098 | | 15.5306 | 15.4945 | 15.5015 | 15.2459 | 15.6 | 15.5177 | 15.1283 | 15.4108 | 28 | 25 | 26 | 26 | 26 | 29 | 24 | 26 |
| AIMP2 | Q13155 | 3.18E-05 | 7.93E-06 | 0.128532 | | 0.485742 | 1.06755 | | 18.5478 | 18.5159 | 18.5098 | 18.2373 | 18.2554 | 18.6576 | 18.5699 | 18.2596 | 11 | 11 | 11 | 11 | 11 | 11 | 11 | 11 |
| PPP6C | O00743 | 1.52E-18 | 6.31E-18 | 0.12871 | + | 2.4857 | 4.71699 | | 16.9628 | 16.8991 | 16.8579 | 16.787 | 16.7988 | 16.7856 | 16.7316 | 16.7862 | 9 | 9 | 10 | 9 | 10 | 10 | 10 | 11 |
| EHD4 | Q9H223 | 2.84E-10 | 1.27E-10 | 0.128731 | | 0.308406 | 0.732317 | | 18.4044 | 18.342 | 18.3005 | 18.6861 | 18.0616 | 18.288 | 17.9208 | 18.1447 | 19 | 19 | 19 | 19 | 19 | 19 | 19 | 19 |
| USP9X | Q93008 | 1.25E-19 | 7.39E-19 | 0.128742 | | 0.81966 | 1.64307 | | 16.6909 | 16.7478 | 16.732 | 16.3917 | 16.6902 | 16.7162 | 16.6211 | 16.5549 | 75 | 76 | 75 | 68 | 68 | 74 | 70 | 73 |
| RFC2 | P35250 | 2.74E-12 | 1.78E-12 | 0.128879 | | 0.400538 | 0.910595 | | 16.1419 | 16.074 | 16.0106 | 16.0263 | 16.0732 | 15.5735 | 15.8865 | 16.1736 | 10 | 10 | 10 | 10 | 7 | 8 | 7 | 8 |
| KDM5C | P41229;P4 | 0.061619 | 0.009958 | 0.128917 | | 0.170474 | 0.451019 | | 13.9594 | 14.5311 | 14.2845 | 13.6862 | 14.4757 | 14.2263 | NaN | NaN | 5 | 2 | 2 | 1 | 3 | 4 | NaN | NaN |
| SCAI | Q8N9R8;Q | 2.72E-12 | 1.77E-12 | 0.129525 | | 0.658611 | 1.37089 | | 14.9247 | 14.9187 | 14.8075 | 14.7486 | 14.9641 | 14.608 | 14.8357 | 14.6142 | 1 | 2 | 1 | 2 | 1 | 1 | 1 | 1 |
| CTNNB1 | P35222 | 9.67E-14 | 9.28E-14 | 0.129601 | | 0.289129 | 0.693572 | | 18.0634 | 18.1361 | 18.0955 | 18.1404 | 17.5394 | 18.3743 | 17.9171 | 17.8725 | 29 | 28 | 28 | 28 | 27 | 28 | 28 | 28 |
| RBM39 | Q14498;Q | 8.04E-06 | 2.13E-06 | 0.129765 | + | 1.81115 | 3.34854 | | 17.5277 | 17.6016 | 17.6078 | 17.4593 | 17.3747 | 17.4084 | 17.4972 | 17.5068 | 14 | 15 | 14 | 13 | 14 | 13 | 13 | 12 |
| RALY | Q9UKM9 | 5.29E-06 | 1.43E-06 | 0.129838 | | 1.10499 | 2.11776 | | 18.2952 | 18.2574 | 18.1828 | 18.1903 | 18.2135 | 17.9803 | 18.0685 | 18.1239 | 13 | 13 | 13 | 12 | 12 | 12 | 13 | 14 |
| MRPL16 | Q9NX20 | 2.51E-15 | 3.83E-15 | 0.130319 | + | 1.40624 | 2.62651 | | 15.5455 | 15.5733 | 15.6478 | 15.4449 | 15.3516 | 15.4851 | 15.4538 | 15.5573 | 8 | 8 | 8 | 8 | 6 | 8 | 8 | 8 |
| YBX3 | P16989;P1 | 1.91E-15 | 3.02E-15 | 0.130489 | | 0.730162 | 1.49263 | | 18.4253 | 18.5343 | 18.4675 | 18.3403 | 18.1555 | 18.5392 | 18.4016 | 18.2895 | 12 | 13 | 13 | 13 | 13 | 13 | 14 | 11 |
| NAP1L1 | P55209;P5 | 2.28E-11 | 1.24E-11 | 0.130502 | | 0.76715 | 1.55501 | | 19.905 | 19.9177 | 19.9011 | 19.9701 | 19.7151 | 19.7317 | 19.6079 | 19.8625 | 11 | 11 | 11 | 11 | 10 | 11 | 10 | 11 |
| PQBP1 | O60828;O6 | 1.26E-10 | 5.96E-11 | 0.1307 | | 0.528538 | 1.14423 | | 15.3881 | 15.3879 | 15.2353 | 15.304 | 14.884 | 15.2689 | 15.3061 | 15.269 | 5 | 6 | 6 | 7 | 4 | 5 | 7 | 7 |
| MAP3K2 | Q9Y2U5 | 1.75E-05 | 4.49E-06 | 0.130761 | | 0.660234 | 1.37367 | | 15.0697 | 15.0539 | 14.865 | 14.7137 | 14.8753 | 15.042 | 14.7454 | 14.9507 | 6 | 7 | 6 | 4 | 5 | 5 | 5 | 6 |
| TRMT1L | Q7Z2T5 | 2.81E-05 | 7.04E-06 | 0.130897 | | 1.0305 | 1.99385 | | 15.192 | 15.1048 | 15.0036 | 14.9386 | 14.8673 | 15.0871 | 14.9248 | 15.0284 | 6 | 7 | 7 | 7 | 5 | 5 | 7 | 6 |
| PCBP1 | Q15365 | 1.32E-11 | 7.49E-12 | 0.130919 | | 0.692837 | 1.42933 | | 20.4562 | 20.4363 | 20.3833 | 20.3204 | 20.5072 | 20.1661 | 20.1318 | 20.3462 | 10 | 10 | 10 | 10 | 10 | 10 | 10 | 10 |
| YWHAZ | P63104 | 5.37E-14 | 5.52E-14 | 0.131116 | | 0.156437 | 0.407861 | | 20.9381 | 20.8033 | 20.8394 | 20.7926 | 20.961 | 19.8022 | 20.9102 | 21.1799 | 21 | 20 | 20 | 20 | 20 | 19 | 19 | 21 |
| TMEM120A | Q9BXJ8;Q9 | 2.61E-05 | 6.55E-06 | 0.131349 | | 0.506114 | 1.10421 | | 15.565 | 15.511 | 15.523 | 15.502 | 15.6692 | 15.2285 | 15.1882 | 15.4205 | 5 | 5 | 6 | 5 | 5 | 4 | 6 | 4 |
| LRRC41 | Q15345 | 3.79E-08 | 1.29E-08 | 0.131466 | | 0.486616 | 1.06913 | | 15.2476 | 15.2131 | 15.0995 | 15.1152 | 15.1203 | 14.7627 | 14.9799 | 15.2982 | 7 | 7 | 5 | 6 | 5 | 5 | 5 | 5 |
| EGLN1 | Q9GZT9;Q9 | 3.58E-08 | 1.22E-08 | 0.131597 | | 0.408831 | 0.926163 | | 14.8122 | 14.8367 | 14.8287 | 14.9037 | 14.8198 | 14.6254 | 14.3092 | 14.8132 | 10 | 10 | 11 | 8 | 6 | 9 | 7 | 10 |
| ZMYND11 | Q15326;Q | 1.24E-07 | 3.96E-08 | 0.131601 | | 0.165964 | 0.429732 | | 14.2967 | 14.2888 | 13.877 | 14.1064 | 14.6665 | 13.5873 | 14.2543 | 13.4982 | 5 | 6 | 6 | 4 | 2 | 4 | 3 | 3 |
| PRPF6 | O94906 | 1.40E-12 | 9.70E-13 | 0.131967 | + | 2.22263 | 4.15326 | | 16.3583 | 16.3648 | 16.3587 | 16.2647 | 16.2546 | 16.1474 | 16.2741 | 16.2023 | 33 | 32 | 34 | 32 | 25 | 32 | 31 | 29 |
| ATP9B | O43861;O | 0.004365 | 0.000788 | 0.132069 | | 0.128223 | 0.349484 | | 12.8025 | 13.4998 | 13.4153 | NaN | 12.897 | 12.7117 | 13.7127 | NaN | 2 | 1 | 1 | NaN | 1 | 2 | 2 | NaN |
| XPO7 | Q9UIA9 | 7.20E-15 | 9.44E-15 | 0.132069 | + | 1.64573 | 3.04666 | | 16.4395 | 16.4471 | 16.4345 | 16.2337 | 16.2294 | 16.3866 | 16.3337 | 16.3583 | 35 | 37 | 34 | 35 | 32 | 35 | 36 | 35 |
| ACAA1 | P09110 | 2.04E-09 | 8.09E-10 | 0.132157 | | 0.226151 | 0.562605 | | 16.549 | 16.5624 | 16.5015 | 15.7936 | 16.6632 | 16.6144 | 16.7268 | 16.2294 | 11 | 12 | 11 | 10 | 11 | 11 | 13 | 11 |
| CISD1 | Q9NZ45 | 7.77E-09 | 2.87E-09 | 0.132264 | | 0.197608 | 0.50066 | | 14.99 | 15.1181 | 15.0444 | 15.4332 | 14.4955 | 14.4199 | 15.1496 | 15.0947 | 5 | 5 | 5 | 5 | 4 | 5 | 5 | 5 |
| SNX4 | O95219 | 5.02E-13 | 3.93E-13 | 0.132267 | | 0.920475 | 1.81108 | | 16.142 | 16.1496 | 16.1353 | 15.9023 | 15.9557 | 16.1534 | 16.1303 | 15.9085 | 19 | 16 | 17 | 13 | 11 | 18 | 15 | 14 |
| MRPS24 | Q96EL2 | 2.21E-12 | 1.47E-12 | 0.132494 | | 0.560248 | 1.20028 | | 15.8772 | 15.9711 | 15.9226 | 15.8949 | 15.4752 | 15.7949 | 15.8811 | 15.9095 | 2 | 3 | 3 | 3 | 2 | 3 | 2 | 2 |

| Gene names | UniProt IDs | Protein Group P-value | Protein Group Q-value | Log2 Difference NUP214 vs Controls (n=5) | p<0.05 | -log10 p-value | T-test Test statistic | NPC Localization | Log2 MS2_quantity_NUP214_1 | Log2 MS2_quantity_NUP214_2 | Log2 MS2_quantity_NUP214_3 | Log2 MS2_quantity_Control_1 | Log2 MS2_quantity_Control_2 | Log2 MS2_quantity_Control_3 | Log2 MS2_quantity_Control_4 | Log2 MS2_quantity_Control_5 | Number Peptides for Quantitation NUP214_1 | Number Peptides for Quantitation NUP214_2 | Number Peptides for Quantitation NUP214_3 | Number Peptides for Quantitation Control_1 | Number Peptides for Quantitation Control_2 | Number Peptides for Quantitation Control_3 | Number Peptides for Quantitation Control_4 | Number Peptides for Quantitation Control_5 |
|---|---|---|---|---|---|---|---|---|---|---|---|---|---|---|---|---|---|---|---|---|---|---|---|---|
| GAR1 | Q9NY12;Q9 | 6.45E-09 | 2.40E-09 | 0.132528 | | 0.70713 | 1.45362 | | 16.6088 | 16.5832 | 16.4781 | 16.2825 | 16.4537 | 16.2668 | 16.5894 | 16.5286 | 6 | 6 | 6 | 5 | 4 | 6 | 5 | 6 |
| SIRT3 | Q9NTG7 | 7.84E-06 | 2.08E-06 | 0.132677 | | 0.568791 | 1.21529 | | 16.005 | 16.1208 | 15.945 | 15.707 | 16.0457 | 16.0911 | 15.7524 | 15.8586 | 2 | 2 | 1 | 1 | 1 | 1 | 1 | 1 |
| EIF3M | Q7L2H7 | 1.85E-18 | 7.46E-18 | 0.132927 | | 0.594277 | 1.25982 | | 17.5533 | 17.5015 | 17.4583 | 17.476 | 17.1019 | 17.3884 | 17.3316 | 17.5593 | 13 | 13 | 15 | 13 | 13 | 13 | 13 | 14 |
| SLK | Q9H2G2-2 | 1.23E-23 | 2.86E-22 | 0.133267 | + | 2.08446 | 3.87348 | | 16.2263 | 16.2425 | 16.229 | 16.0764 | 16.1515 | 16.07 | 16.1667 | 16.0321 | 32 | 33 | 32 | 31 | 29 | 33 | 31 | 30 |
| ARFGAP2 | Q8N6H7 | 1.18E-10 | 5.60E-11 | 0.133499 | | 0.588716 | 1.25013 | | 16.6571 | 16.7111 | 16.6091 | 16.6541 | 16.405 | 16.6266 | 16.2763 | 16.6659 | 13 | 14 | 14 | 13 | 11 | 14 | 11 | 11 |
| GOLPH3L | Q9H4A5 | 3.72E-09 | 1.43E-09 | 0.133566 | + | 1.40453 | 2.62356 | | 18.1582 | 18.2288 | 18.2762 | 18.0682 | 17.9946 | 18.0737 | 18.1004 | 18.2005 | 8 | 7 | 9 | 6 | 6 | 7 | 7 | 6 |
| POLR2E | P19388 | 2.89E-09 | 1.13E-09 | 0.133604 | | 0.467056 | 1.03366 | | 16.369 | 16.4154 | 16.3651 | 16.3177 | 15.8994 | 16.215 | 16.4706 | 16.345 | 7 | 7 | 7 | 7 | 6 | 7 | 6 | 7 |
| DLAT | P10515 | 3.99E-12 | 2.50E-12 | 0.133684 | + | 2.04713 | 3.79964 | | 17.426 | 17.3872 | 17.3792 | 17.2637 | 17.3182 | 17.2693 | 17.1711 | 17.2967 | 15 | 16 | 16 | 16 | 16 | 16 | 15 | 16 |
| DHX38 | Q92620 | 1.04E-12 | 7.50E-13 | 0.133742 | | 0.835529 | 1.66959 | | 15.1368 | 15.2194 | 15.2284 | 15.2742 | 14.9504 | 14.9949 | 14.9962 | 15.0898 | 19 | 20 | 20 | 21 | 13 | 16 | 20 | 19 |
| DNAJC8 | O75937 | 4.55E-06 | 1.24E-06 | 0.133749 | | 0.698733 | 1.43935 | | 16.9299 | 16.9407 | 16.9735 | 16.9608 | 16.8496 | 16.629 | 16.6763 | 16.9558 | 9 | 9 | 9 | 9 | 9 | 8 | 9 | 9 |
| ATF2 | P15336;P1 | 0.004139 | 0.00075 | 0.133826 | | 0.228635 | 0.5742 | | 13.5972 | 13.5874 | 13.6437 | 13.7173 | 12.9293 | 13.8018 | NaN | 13.4541 | 4 | 3 | 3 | 4 | 3 | 2 | NaN | 3 |
| CIC | Q96RK0 | 0.009709 | 0.001669 | 0.134104 | | 0.394422 | 0.912718 | | 16.527 | 16.6313 | 16.5462 | 16.6552 | NaN | 16.5198 | 16.0863 | 16.475 | 6 | 3 | 4 | 3 | NaN | 3 | 2 | 3 |
| FZD1 | Q9UP38 | 7.69E-05 | 1.85E-05 | 0.134161 | | 0.129958 | 0.348893 | | 13.919 | 13.9456 | 14.044 | 14.3864 | 12.9281 | 14.1985 | NaN | 13.8284 | 2 | 2 | 2 | 2 | 1 | 1 | NaN | 1 |
| CTBS | Q01459 | 1.25E-09 | 5.13E-10 | 0.134257 | | 0.260295 | 0.634499 | | 15.466 | 15.5399 | 15.6002 | 15.0987 | 15.21 | 15.8232 | 15.7418 | 15.1319 | 4 | 3 | 5 | 3 | 4 | 3 | 4 | 4 |
| ZC3H11A | O75152 | 0.000415 | 9.05E-05 | 0.13431 | | 0.5905 | 1.25324 | | 14.6855 | 14.7839 | 14.7728 | 14.7739 | 14.5283 | 14.353 | 14.6513 | 14.7589 | 9 | 6 | 10 | 8 | 6 | 6 | 8 | 7 |
| ATG2B | Q96BY7 | 1.44E-12 | 9.98E-13 | 0.13441 | | 1.09908 | 2.10791 | | 14.9585 | 14.9445 | 14.9781 | 14.8708 | 14.9372 | 14.6532 | 14.8143 | 14.8544 | 15 | 17 | 17 | 15 | 12 | 14 | 13 | 14 |
| RBMX | P38159 | 1.85E-19 | 1.05E-18 | 0.134489 | | 0.940149 | 1.84378 | | 18.4361 | 18.4454 | 18.4558 | 18.2726 | 18.4117 | 18.3396 | 18.1193 | 18.4133 | 20 | 21 | 19 | 19 | 21 | 20 | 20 | 20 |
| CUL4A | Q13619 | 6.04E-11 | 3.02E-11 | 0.13489 | | 1.17496 | 2.23461 | | 15.9358 | 15.9555 | 15.9344 | 15.8073 | 15.9298 | 15.6821 | 15.7371 | 15.8788 | 25 | 26 | 26 | 24 | 22 | 24 | 25 | 24 |
| QDPR | P09417 | 4.10E-12 | 2.56E-12 | 0.134915 | | 0.575838 | 1.22763 | | 17.2785 | 17.2456 | 17.2553 | 17.3175 | 17.2564 | 17.1483 | 16.8512 | 17.0508 | 8 | 8 | 8 | 7 | 8 | 8 | 7 | 8 |
| UBASH3B | Q8TF42 | 1.59E-17 | 4.73E-17 | 0.134963 | | 0.217808 | 0.544683 | | 17.2535 | 17.3642 | 17.2545 | 17.5249 | 17.3522 | 16.4885 | 17.0373 | 17.3759 | 16 | 15 | 16 | 16 | 16 | 11 | 15 | 17 |
| ATAD3A | Q9NVI7-2 | 1.74E-16 | 3.82E-16 | 0.13543 | | 1.00951 | 1.95898 | | 18.0384 | 18.0542 | 18.062 | 18.004 | 17.8605 | 17.9031 | 17.7611 | 18.0519 | 27 | 26 | 27 | 25 | 25 | 24 | 24 | 25 |
| UPF2 | Q9HAU5 | 5.82E-19 | 2.76E-18 | 0.135562 | | 0.716159 | 1.46893 | | 15.6592 | 15.7031 | 15.695 | 15.7633 | 15.628 | 15.4714 | 15.3588 | 15.5296 | 24 | 24 | 24 | 25 | 17 | 22 | 21 | 22 |
| NDUFS7 | O75251;O7 | 0.000173 | 3.96E-05 | 0.135615 | | 0.286494 | 0.688231 | | 18.2348 | 18.1928 | 18.0932 | 18.2934 | 17.7305 | 18.4754 | 17.8246 | 17.866 | 3 | 3 | 3 | 3 | 4 | 3 | 4 | 3 |
| MRPS22 | P82650 | 1.19E-09 | 4.88E-10 | 0.135626 | | 1.23904 | 2.34218 | | 16.2387 | 16.3539 | 16.3265 | 16.2619 | 16.0467 | 16.2351 | 16.1878 | 16.1223 | 16 | 18 | 17 | 16 | 13 | 17 | 17 | 16 |
| SFPQ | P23246 | 9.54E-12 | 5.56E-12 | 0.135652 | + | 1.51478 | 2.81474 | | 18.4993 | 18.5472 | 18.4796 | 18.395 | 18.4686 | 18.2807 | 18.3091 | 18.4116 | 28 | 28 | 27 | 28 | 25 | 28 | 26 | 27 |
| ARPC5 | O15511 | 7.24E-08 | 2.37E-08 | 0.135815 | | 0.86384 | 1.71683 | | 19.8721 | 19.8189 | 19.7575 | 19.8412 | 19.6953 | 19.514 | 19.605 | 19.7464 | 10 | 10 | 10 | 10 | 10 | 10 | 10 | 10 |
| PRORP | O15091 | 4.44E-07 | 1.35E-07 | 0.136123 | | 0.275286 | 0.665387 | | 14.9222 | 14.9003 | 14.827 | 14.7784 | 15.0563 | 14.1741 | 14.7798 | 14.9467 | 9 | 11 | 9 | 7 | 9 | 5 | 8 | 8 |
| TMA7 | Q9Y2S6 | 0.002314 | 0.000446 | 0.136165 | | 0.220172 | 0.549777 | | 16.9198 | 17.0797 | 16.7122 | 16.969 | 17.11 | 16.1918 | 16.5267 | 17.0411 | 4 | 3 | 4 | 3 | 4 | 3 | 3 | 4 |
| WDR70 | Q9NW82 | 4.00E-13 | 3.22E-13 | 0.136198 | | 0.223602 | 0.557145 | | 15.0338 | 14.9798 | 14.9321 | 14.8896 | 15.3619 | 14.8891 | 14.2162 | 14.8717 | 11 | 9 | 14 | 12 | 6 | 8 | 5 | 7 |
| IFT22 | Q9H7X7 | 0.001837 | 0.000363 | 0.136242 | | 0.42518 | 0.956657 | | 14.7974 | 14.8229 | 14.3847 | 14.5297 | 14.5701 | 14.5778 | 14.7155 | 14.2675 | 4 | 3 | 4 | 1 | 1 | 3 | 2 | 1 |
| CAMK2G | Q13555-11 | 2.76E-14 | 3.08E-14 | 0.136299 | | 0.389002 | 0.888821 | | 16.1775 | 16.3594 | 16.2035 | 16.3948 | 16.1 | 16.1456 | 15.716 | 16.1962 | 9 | 9 | 9 | 9 | 8 | 9 | 9 | 9 |
| CLIC4 | Q9Y696 | 9.94E-08 | 3.21E-08 | 0.136304 | | 0.602111 | 1.27344 | | 20.1364 | 20.0739 | 20.0957 | 20.0515 | 19.8079 | 20.1983 | 20.003 | 19.7679 | 21 | 22 | 21 | 21 | 21 | 21 | 21 | 20 |
| PTDSS1 | P48651 | 1.18E-15 | 1.99E-15 | 0.136413 | | 1.06842 | 2.05689 | | 16.8049 | 16.8676 | 16.8644 | 16.8655 | 16.6842 | 16.6844 | 16.5675 | 16.7443 | 7 | 8 | 7 | 7 | 6 | 8 | 8 | 8 |
| ATP2A2 | P16615 | 1.67E-23 | 3.59E-22 | 0.136774 | | 0.520944 | 1.13071 | | 18.6214 | 18.6052 | 18.6364 | 18.7995 | 18.4228 | 18.5421 | 18.2614 | 18.3953 | 49 | 49 | 49 | 49 | 49 | 50 | 48 | 48 |
| USP4 | Q13107;Q1 | 6.09E-13 | 4.64E-13 | 0.136991 | | 1.27973 | 2.41085 | | 15.633 | 15.583 | 15.6094 | 15.5675 | 15.3179 | 15.4649 | 15.4942 | 15.5129 | 24 | 23 | 27 | 23 | 19 | 24 | 24 | 21 |
| MRPS18A | Q9NVS2;Q9 | 3.91E-08 | 1.32E-08 | 0.13716 | | 0.667083 | 1.38539 | | 16.037 | 15.9755 | 15.8821 | 15.9713 | 15.8991 | 15.8043 | 15.5684 | 15.8955 | 7 | 8 | 8 | 7 | 5 | 5 | 5 | 6 |
| PRKACA | P17612 | 1.57E-14 | 1.88E-14 | 0.13721 | + | 2.20073 | 4.10822 | | 17.8908 | 17.8978 | 17.8812 | 17.8106 | 17.785 | 17.7714 | 17.6688 | 17.7276 | 17 | 16 | 17 | 17 | 17 | 17 | 16 | 17 |
| CUTC | Q9NTM9 | 0.011257 | 0.00192 | 0.137408 | | 0.151565 | 0.400396 | | 14.806 | 14.7704 | 14.396 | NaN | 13.7891 | 14.6511 | 14.5243 | 15.1158 | 1 | 1 | 2 | NaN | 2 | 2 | 2 | 2 |
| RRP1B | Q14684 | 1.90E-07 | 5.97E-08 | 0.137429 | | 0.979765 | 1.90958 | | 15.0378 | 15.0479 | 14.985 | 15.0333 | 14.8892 | 14.7187 | 14.8394 | 14.9501 | 17 | 17 | 16 | 17 | 15 | 15 | 16 | 15 |
| RBM25 | P49756 | 9.44E-19 | 4.10E-18 | 0.137466 | | 1.17189 | 2.22948 | | 16.0293 | 16.1211 | 16.0729 | 16.0586 | 15.9591 | 15.7951 | 15.8963 | 15.9755 | 22 | 22 | 22 | 22 | 20 | 21 | 20 | 22 |
| PRTFDC1 | Q9NRG1 | 0.002212 | 0.000429 | 0.137525 | | 0.47711 | 1.07046 | | 16.3542 | 16.3174 | 16.4611 | NaN | 16.5007 | 16.0138 | 16.1501 | 16.2956 | 5 | 5 | 5 | NaN | 3 | 1 | 3 | 3 |
| PPM1B | O75688 | 2.83E-07 | 8.76E-08 | 0.137789 | | 0.721628 | 1.47819 | | 16.0762 | 16.1071 | 16.063 | 15.9903 | 15.9235 | 15.987 | 16.1225 | 15.6981 | 12 | 12 | 12 | 11 | 9 | 11 | 11 | 9 |
| NR3C1 | P04150 | 1.09E-18 | 4.73E-18 | 0.137901 | | 0.421972 | 0.950694 | | 16.1229 | 16.103 | 16.1528 | 16.1495 | 16.0195 | 16.1553 | 15.5678 | 16.0494 | 21 | 21 | 21 | 21 | 18 | 22 | 19 | 21 |
| TP53RK | Q96S44 | 1.47E-14 | 1.78E-14 | 0.138013 | | 0.613654 | 1.29346 | | 16.5872 | 16.5615 | 16.5672 | 16.2145 | 16.528 | 16.4472 | 16.6694 | 16.3109 | 6 | 6 | 5 | 5 | 5 | 5 | 5 | 5 |
| RPL22L1 | Q6P5R6 | 1.15E-09 | 4.72E-10 | 0.138118 | | 0.50477 | 1.1018 | | 17.5988 | 17.4395 | 17.4534 | 17.4675 | 17.4799 | 17.135 | 17.1508 | 17.5622 | 3 | 3 | 3 | 3 | 3 | 3 | 3 | 3 |
| CRTC3 | Q6UUV7;Q | 4.93E-05 | 1.21E-05 | 0.138184 | | 0.223054 | 0.555969 | | 15.6886 | 15.2095 | 15.6273 | 15.7107 | 15.5764 | 15.4735 | 14.7463 | 15.3445 | 1 | 2 | 1 | 1 | 1 | 1 | 1 | 1 |
| ALS2 | Q96Q42 | 0.007864 | 0.001369 | 0.138444 | | 0.250768 | 0.62176 | | 16.056 | 15.7785 | 15.5964 | 15.2095 | 15.9691 | NaN | 15.7216 | 15.7871 | 4 | 6 | 5 | 5 | 5 | NaN | 5 | 2 |
| ARFRP1 | Q13795 | 0.000119 | 2.80E-05 | 0.138471 | | 0.407402 | 0.923486 | | 14.7083 | 14.706 | 14.9317 | 14.5486 | 14.7943 | 14.786 | 14.8161 | 14.2727 | 4 | 4 | 4 | 4 | 3 | 4 | 3 | 3 |
| KHNYN | O15037 | 0.005294 | 0.000942 | 0.138522 | | 0.509368 | 1.11003 | | 15.5021 | 15.6802 | 15.6956 | 15.7164 | 15.1755 | 15.5149 | 15.5101 | 15.5203 | 3 | 2 | 3 | 3 | 2 | 3 | 3 | 1 |
| HEATR3 | Q7Z4Q2 | 1.00E-06 | 2.91E-07 | 0.138672 | | 0.426507 | 0.95912 | | 15.5566 | 15.4866 | 15.535 | 15.5199 | 15.6001 | 15.0207 | 15.5307 | 15.2657 | 11 | 12 | 10 | 9 | 9 | 9 | 8 | 10 |
| IFT172 | Q9UG01 | 1.23E-10 | 5.82E-11 | 0.138817 | | 0.53368 | 1.15336 | | 15.6441 | 15.446 | 15.6732 | 15.2269 | 15.6141 | 15.6434 | 15.4453 | 15.3149 | 6 | 8 | 8 | 4 | 3 | 7 | 4 | 3 |
| RUFY1 | Q96T51 | 4.86E-15 | 6.76E-15 | 0.138972 | | 0.582377 | 1.23906 | | 16.0841 | 16.0549 | 16.0131 | 15.9635 | 16.1588 | 15.9442 | 15.8444 | 15.6478 | 26 | 28 | 29 | 28 | 24 | 28 | 26 | 23 |
| MRPS18B | Q9Y676 | 5.48E-12 | 3.35E-12 | 0.139496 | | 0.922471 | 1.8144 | | 16.2537 | 16.304 | 16.1611 | 16.1329 | 15.9142 | 16.2119 | 16.18 | 16.0616 | 5 | 5 | 5 | 5 | 5 | 5 | 5 | 5 |
| SNX33 | Q8WV41 | 7.75E-05 | 1.86E-05 | 0.139877 | | 0.773422 | 1.56555 | | 15.573 | 15.5865 | 15.5567 | 15.5803 | 15.5212 | 15.5035 | 15.2196 | 15.3363 | 11 | 10 | 10 | 11 | 9 | 9 | 10 | 9 |
| API5 | Q9BZZ5 | 1.16E-17 | 3.62E-17 | 0.139977 | + | 1.37883 | 2.57947 | | 17.6266 | 17.5939 | 17.6034 | 17.4978 | 17.5378 | 17.3097 | 17.4933 | 17.5014 | 21 | 19 | 19 | 18 | 17 | 18 | 18 | 17 |
| MTCH2 | Q9Y6C9 | 7.65E-08 | 2.50E-08 | 0.140107 | | 0.974535 | 1.90089 | | 18.7069 | 18.7063 | 18.7156 | 18.4113 | 18.7287 | 18.5066 | 18.5513 | 18.6496 | 12 | 12 | 12 | 12 | 12 | 12 | 12 | 12 |
| MPST | P25325;P2 | 1.04E-06 | 3.02E-07 | 0.140389 | + | 2.12306 | 3.95059 | | 17.2257 | 17.1192 | 17.1517 | 17.045 | 17.0664 | 17.0241 | 17.0418 | 16.9485 | 12 | 12 | 12 | 12 | 12 | 12 | 12 | 11 |
| DDX39A | O00148 | 2.41E-19 | 1.32E-18 | 0.140408 | + | 1.54766 | 2.87245 | | 19.5394 | 19.6053 | 19.5872 | 19.5402 | 19.3833 | 19.3557 | 19.4083 | 19.4969 | 17 | 17 | 17 | 17 | 16 | 17 | 17 | 17 |
| TEP1 | Q99973;Q9 | 7.30E-12 | 4.37E-12 | 0.14072 | | 0.132352 | 0.35139 | | 15.0316 | 15.1012 | 14.953 | 13.7316 | 15.4658 | 15.0883 | 15.004 | 15.1496 | 11 | 13 | 12 | 5 | 8 | 8 | 8 | 11 |
| NARS1 | O43776 | 3.36E-10 | 1.48E-10 | 0.140809 | | 0.305806 | 0.727123 | | 19.1456 | 19.1865 | 19.1712 | 18.7841 | 18.8218 | 19.4886 | 19.2511 | 18.7891 | 26 | 26 | 26 | 25 | 25 | 27 | 26 | 26 |
| ADAM12 | O43184 | 1.51E-13 | 1.36E-13 | 0.141146 | | 0.114451 | 0.315253 | | 15.6594 | 15.5691 | 15.8741 | NaN | NaN | 16.4317 | 15.2048 | 15.0427 | 4 | 5 | 6 | NaN | NaN | 9 | 3 | 2 |
| HDAC2 | Q92769;Q9 | 3.88E-16 | 7.39E-16 | 0.141551 | | 0.571904 | 1.22074 | | 16.0775 | 16.0481 | 16.1095 | 16.2206 | 15.7092 | 15.867 | 16.0213 | 15.866 | 6 | 6 | 7 | 6 | 6 | 6 | 5 | 5 |
| GGT7 | Q9UJ14 | 1.66E-15 | 2.67E-15 | 0.141623 | + | 1.42749 | 2.66311 | | 15.3136 | 15.2079 | 15.3272 | 15.1602 | 15.1299 | 15.1093 | 15.2571 | 15.0498 | 7 | 8 | 8 | 8 | 7 | 8 | 8 | 8 |
| BCLAF1 | Q9NYF8;Q9 | 4.00E-12 | 2.51E-12 | 0.141912 | | 1.16166 | 2.21235 | | 15.9507 | 16.036 | 16.0128 | 16.0163 | 15.7422 | 15.8558 | 15.8772 | 15.7984 | 19 | 20 | 19 | 20 | 16 | 18 | 18 | 18 |
| DDX46 | Q7L014 | 9.14E-19 | 3.99E-18 | 0.142108 | + | 1.39226 | 2.60249 | | 16.1057 | 16.2802 | 16.2157 | 16.0665 | 16.0787 | 16.009 | 16.1551 | 15.9829 | 38 | 38 | 39 | 34 | 31 | 32 | 35 | 29 |
| UQCRC2 | P22695 | 2.37E-09 | 9.33E-10 | 0.142157 | | 1.0827 | 2.08064 | | 18.3435 | 18.2837 | 18.2787 | 18.1254 | 18.1735 | 17.9873 | 18.2639 | 18.249 | 15 | 15 | 15 | 15 | 15 | 15 | 15 | 15 |
| SUPT5H | O00267;O0 | 1.08E-12 | 7.76E-13 | 0.142224 | + | 1.86051 | 3.44079 | | 15.6991 | 15.6535 | 15.6624 | 15.5946 | 15.4282 | 15.5254 | 15.5866 | 15.5124 | 28 | 29 | 28 | 27 | 25 | 27 | 27 | 28 |
| WTAP | Q15007 | 4.55E-09 | 1.73E-09 | 0.142311 | | 0.772946 | 1.56475 | | 14.9706 | 15.0028 | 14.9517 | 14.965 | 14.9167 | 14.5765 | 14.8336 | 14.872 | 4 | 5 | 4 | 3 | 3 | 3 | 2 | 3 |
| CARNMT1 | Q8N4J0 | 6.83E-06 | 1.82E-06 | 0.142344 | | 0.281632 | 0.678346 | | 15.2115 | 15.2115 | 15.1569 | 15.5638 | 15.0717 | 14.8419 | 14.6302 | 15.1471 | 6 | 5 | 5 | 5 | 5 | 5 | 3 | 5 |
| HNRNPH1 | P31943 | 1.47E-10 | 6.89E-11 | 0.142644 | | 0.726083 | 1.48573 | | 20.2496 | 20.2378 | 20.2265 | 20.1771 | 20.3008 | 19.8837 | 19.9989 | 20.1161 | 14 | 14 | 13 | 14 | 13 | 14 | 14 | 15 |
| RBM27 | Q9P2N5 | 0.000221 | 4.99E-05 | 0.14265 | | 0.9043 | 1.78419 | | 14.1476 | 14.1881 | 14.1732 | 14.1822 | 14.1444 | 13.9044 | 13.8931 | 14.0107 | 10 | 12 | 8 | 8 | 6 | 7 | 8 | 10 |
| NCBP2 | P52298 | 6.13E-07 | 1.82E-07 | 0.142791 | | 0.859993 | 1.71041 | | 16.5448 | 16.6978 | 16.5579 | 16.4242 | 16.6552 | 16.3194 | 16.3971 | 16.491 | 6 | 5 | 6 | 5 | 4 | 5 | 5 | 5 |
| MRPS11 | P82912;P8 | 0.002047 | 0.000401 | 0.143161 | | 0.965949 | 1.88663 | | 17.4927 | 17.5447 | 17.5772 | 17.2761 | 17.5935 | 17.4289 | 17.3344 | 17.3422 | 3 | 3 | 3 | 3 | 3 | 3 | 3 | 3 |
| HIRA | P54198;P5 | 5.11E-06 | 1.38E-06 | 0.143188 | | 0.669556 | 1.38962 | | 15.1324 | 15.2889 | 15.2149 | 15.281 | 15.152 | 14.8417 | 15.0089 | 15.0608 | 3 | 4 | 4 | 3 | 2 | 2 | 4 | 2 |
| RGS12 | O14924;O1 | 9.68E-11 | 4.65E-11 | 0.143263 | | 0.187465 | 0.478198 | | 14.0715 | 13.7481 | 13.7655 | 13.7723 | 14.5101 | 13.3814 | 13.2756 | 13.653 | 1 | 2 | 2 | 1 | 1 | 1 | 1 | 1 |
| RBM5 | P52756 | 2.31E-08 | 8.01E-09 | 0.143302 | | 0.431389 | 0.968171 | | 15.3477 | 15.1904 | 15.2484 | 15.3684 | 15.0688 | 15.0253 | 14.7878 | 15.344 | 7 | 7 | 6 | 5 | 3 | 3 | 4 | 5 |
| EXOG | Q9Y2C4 | 1.21E-08 | 4.33E-09 | 0.143496 | | 0.326648 | 0.768475 | | 15.9119 | 15.9411 | 15.8895 | 15.6144 | 16.2354 | 15.4705 | 15.5928 | 15.9403 | 4 | 4 | 4 | 4 | 4 | 4 | 3 | 4 |
| TMEM11 | P17152 | 0.000463 | 0.0001 | 0.143504 | + | 1.55299 | 2.88184 | | 17.2012 | 17.2206 | 17.2288 | 16.9934 | 17.1187 | 17.0754 | 16.9936 | 17.1859 | 4 | 4 | 4 | 4 | 4 | 4 | 4 | 4 |

| Gene names | UniProt IDs | Protein Group P-value | Protein Group Q-value | Log2 Difference NUP214 vs Controls (n=5) | p<0.05 | -log10 p-value | T-test Test statistic | NPC Localization | Log2 MS2_quantity_NUP214_1 | Log2 MS2_quantity_NUP214_2 | Log2 MS2_quantity_NUP214_3 | Log2 MS2_quantity_Control_1 | Log2 MS2_quantity_Control_2 | Log2 MS2_quantity_Control_3 | Log2 MS2_quantity_Control_4 | Log2 MS2_quantity_Control_5 | Number Peptides for Quantitation NUP214_1 | Number Peptides for Quantitation NUP214_2 | Number Peptides for Quantitation NUP214_3 | Number Peptides for Quantitation Control_1 | Number Peptides for Quantitation Control_2 | Number Peptides for Quantitation Control_3 | Number Peptides for Quantitation Control_4 | Number Peptides for Quantitation Control_5 |
|---|---|---|---|---|---|---|---|---|---|---|---|---|---|---|---|---|---|---|---|---|---|---|---|---|
| TCEAL4 | Q96EI5;Q9 | 5.68E-08 | 1.89E-08 | 0.14357 | | 0.409806 | 0.927989 | | 14.6623 | 14.5859 | 14.5829 | 14.5371 | 14.0571 | 14.4111 | 14.7393 | 14.5894 | 8 | 7 | 6 | 8 | 5 | 7 | 4 | 8 |
| XRCC5 | P13010 | 2.99E-15 | 4.44E-15 | 0.143584 | + | 1.69919 | 3.14306 | | 18.8703 | 18.9179 | 18.8967 | 18.8256 | 18.7398 | 18.6388 | 18.7392 | 18.8136 | 42 | 39 | 41 | 41 | 39 | 38 | 39 | 41 |
| SUCLG2 | Q96I99 | 1.34E-16 | 3.05E-16 | 0.143589 | + | 1.45939 | 2.71827 | | 17.1815 | 17.1144 | 17.1444 | 16.9326 | 17.1381 | 16.9936 | 16.9291 | 17.0225 | 23 | 22 | 22 | 20 | 19 | 20 | 19 | 20 |
| ATXN3 | P54252;P5 | 1.19E-06 | 3.41E-07 | 0.143599 | | 0.223087 | 0.55604 | | 15.4114 | 14.9224 | 15.4986 | 14.9159 | 14.6937 | 15.6896 | 15.1536 | 15.2167 | 6 | 5 | 5 | 5 | 4 | 5 | 5 | 4 |
| PNPT1 | Q8TCS8 | 5.29E-16 | 9.62E-16 | 0.143825 | | 0.814994 | 1.63526 | | 15.8813 | 15.8554 | 15.8439 | 15.7491 | 15.4864 | 15.7715 | 15.8854 | 15.6895 | 21 | 21 | 24 | 21 | 15 | 21 | 16 | 17 |
| ZNF207 | O43670;O4 | 2.46E-06 | 6.87E-07 | 0.143887 | + | 1.91538 | 3.54459 | | 17.3932 | 17.4338 | 17.4436 | 17.2974 | 17.3534 | 17.1737 | 17.2909 | 17.2828 | 8 | 8 | 8 | 8 | 8 | 8 | 7 | 8 |
| BRD4 | O60885 | 2.39E-07 | 7.47E-08 | 0.144033 | | 0.97038 | 1.89399 | | 15.1685 | 15.2709 | 15.176 | 15.1502 | 15.2059 | 15.0503 | 14.9013 | 14.998 | 17 | 18 | 18 | 18 | 11 | 15 | 14 | 17 |
| DUSP22 | Q9NRW4;C | 0.001169 | 0.000239 | 0.144047 | | 0.651935 | 1.35944 | | 14.9914 | 14.8598 | 14.8667 | 14.7663 | 14.9566 | 14.4943 | 14.8377 | 14.7546 | 2 | 2 | 2 | 2 | 1 | 1 | 2 | 2 |
| IDH2 | P48735 | 1.54E-13 | 1.39E-13 | 0.144059 | | 0.891266 | 1.7625 | | 18.2992 | 18.2886 | 18.2485 | 17.9828 | 18.315 | 18.0891 | 18.054 | 18.2324 | 22 | 23 | 24 | 21 | 22 | 23 | 21 | 22 |
| HLTF | Q14527 | 8.05E-14 | 7.87E-14 | 0.144193 | | 0.372133 | 0.85672 | | 14.6188 | 14.6972 | 14.4198 | 14.7066 | 14.7194 | 14.221 | 14.1682 | 14.3569 | 8 | 7 | 11 | 9 | 5 | 7 | 7 | 7 |
| NOP10 | Q9NPE3 | 0.000272 | 6.06E-05 | 0.144459 | | 0.931939 | 1.83013 | | 15.0413 | 14.9316 | 15.1878 | 14.8719 | 15.0613 | 14.9047 | 14.7973 | 14.9103 | 4 | 4 | 4 | 4 | 3 | 4 | 3 | 4 |
| AQR | O60306 | 2.37E-16 | 4.92E-16 | 0.144714 | + | 1.52418 | 2.83121 | | 15.7836 | 15.7981 | 15.7768 | 15.7149 | 15.687 | 15.5405 | 15.5571 | 15.7076 | 16 | 17 | 18 | 14 | 14 | 14 | 15 | 16 |
| SIRT1 | Q96EB6 | 0.025962 | 0.004286 | 0.144802 | | 0.67288 | 1.3953 | | 13.9025 | 14.0798 | 14.0164 | 13.6678 | 14.0734 | 13.7473 | 13.8302 | 13.9552 | 4 | 2 | 4 | 6 | 2 | 3 | 2 | 4 |
| FKBP3 | Q00688 | 3.33E-10 | 1.46E-10 | 0.144877 | | 1.1576 | 2.20557 | | 18.2787 | 18.2653 | 18.2115 | 18.1393 | 18.0554 | 18.2734 | 18.0764 | 17.9901 | 14 | 14 | 14 | 13 | 14 | 14 | 14 | 14 |
| ZC3H13 | Q5T200 | 3.31E-05 | 8.26E-06 | 0.145108 | | 0.449554 | 1.00166 | | 13.684 | 13.6284 | 13.7158 | 13.4212 | 13.7986 | 13.1729 | 13.6196 | 13.6426 | 6 | 6 | 6 | 5 | 6 | 3 | 4 | 4 |
| OTULIN | Q96BN8 | 0.001712 | 0.000341 | 0.145204 | | 0.918239 | 1.80736 | | 15.6236 | 15.632 | 15.6261 | 15.4373 | 15.326 | 15.6022 | 15.6418 | 15.4029 | 10 | 10 | 8 | 7 | 5 | 9 | 9 | 8 |
| SAFB2 | Q14151 | 1.24E-19 | 7.39E-19 | 0.145214 | | 1.18339 | 2.24873 | | 16.1259 | 16.0719 | 16.1675 | 16.0976 | 15.8529 | 15.8991 | 16.0564 | 15.9767 | 22 | 23 | 23 | 23 | 20 | 23 | 22 | 22 |
| BRCC3 | P46736;P4 | 4.76E-14 | 4.96E-14 | 0.145314 | + | 1.4931 | 2.77688 | | 16.1776 | 16.107 | 16.1999 | 15.9597 | 15.9946 | 16.1412 | 16.046 | 15.9392 | 6 | 8 | 5 | 5 | 5 | 7 | 6 | 5 |
| OSGEP | Q9NPF4 | 5.82E-14 | 5.93E-14 | 0.145514 | | 0.501703 | 1.0963 | | 16.1823 | 16.2173 | 16.2124 | 15.7775 | 16.1429 | 16.1537 | 16.3296 | 15.8888 | 11 | 10 | 11 | 10 | 11 | 11 | 10 | 10 |
| ACTL6A | O96019 | 8.27E-17 | 1.99E-16 | 0.145523 | + | 1.41091 | 2.63454 | | 16.5744 | 16.5582 | 16.5321 | 16.5191 | 16.3553 | 16.4401 | 16.2832 | 16.4492 | 13 | 14 | 14 | 14 | 13 | 13 | 13 | 13 |
| GNL1 | P36915 | 5.41E-15 | 7.41E-15 | 0.146064 | | 1.04603 | 2.01965 | | 16.6632 | 16.6882 | 16.6918 | 16.5574 | 16.3924 | 16.7102 | 16.5589 | 16.456 | 15 | 16 | 14 | 15 | 14 | 15 | 15 | 13 |
| GEMIN8 | Q9NWZ8 | 0.000485 | 0.000105 | 0.146075 | | 0.262622 | 0.639319 | | 13.9127 | 14.0943 | 13.7128 | 14.0919 | 13.1734 | 13.9676 | 13.8739 | 13.696 | 2 | 2 | 3 | 3 | 3 | 3 | 3 | 3 |
| ANKRD28 | O15084;O1 | 7.53E-09 | 2.78E-09 | 0.146554 | | 0.571178 | 1.21947 | | 14.6319 | 14.75 | 14.6407 | 14.5438 | 14.826 | 14.3658 | 14.3358 | 14.5669 | 15 | 15 | 15 | 15 | 13 | 14 | 15 | 14 |
| KIF14 | Q15058 | 4.24E-07 | 1.29E-07 | 0.146667 | | 0.077972 | 0.218529 | | 13.6124 | 13.4392 | 13.6207 | 13.7559 | NaN | 11.7421 | 14.2467 | 13.8985 | 3 | 2 | 2 | 1 | NaN | 2 | 3 | 3 |
| FADS2 | O95864 | 3.93E-07 | 1.20E-07 | 0.147201 | | 0.142216 | 0.37473 | | 16.3852 | 16.382 | 16.3522 | 17.1791 | 16.3469 | 16.1922 | 15.3385 | 16.0728 | 10 | 9 | 10 | 11 | 9 | 9 | 8 | 10 |
| AHCY | P23526 | 4.66E-16 | 8.66E-16 | 0.147237 | + | 1.45936 | 2.71821 | | 19.2483 | 19.2556 | 19.2593 | 19.0633 | 18.9769 | 19.1927 | 19.1889 | 19.1141 | 22 | 22 | 22 | 21 | 22 | 22 | 22 | 22 |
| PRCP | P42785 | 2.08E-12 | 1.39E-12 | 0.147528 | | 0.470829 | 1.04052 | | 17.402 | 17.4072 | 17.412 | 16.847 | 17.363 | 17.4103 | 17.4092 | 17.2681 | 16 | 16 | 15 | 16 | 14 | 14 | 14 | 15 |
| ACOT9 | Q9Y305;Q9 | 3.71E-10 | 1.62E-10 | 0.147873 | | 1.17644 | 2.23709 | | 17.4762 | 17.4615 | 17.5103 | 17.4296 | 17.2272 | 17.4334 | 17.3753 | 17.2085 | 18 | 18 | 18 | 17 | 17 | 17 | 19 | 17 |
| DENND4C | Q5VZ89;Q5 | 6.61E-06 | 1.77E-06 | 0.14802 | + | 1.39798 | 2.61231 | | 15.7202 | 15.8122 | 15.7466 | 15.5663 | 15.736 | 15.4974 | 15.6146 | 15.6439 | 20 | 20 | 19 | 14 | 14 | 14 | 14 | 10 |
| VAC14 | Q08AM6 | 9.74E-14 | 9.32E-14 | 0.148508 | | 0.633652 | 1.328 | | 15.7327 | 15.625 | 15.6742 | 15.3255 | 15.6113 | 15.6314 | 15.7329 | 15.3429 | 24 | 25 | 26 | 23 | 22 | 23 | 23 | 23 |
| LIMD2 | Q9BT23 | 2.86E-05 | 7.16E-06 | 0.148531 | | 0.121827 | 0.326142 | | 14.9896 | 14.0651 | 14.7195 | 15.0103 | 14.7859 | 14.5174 | 13.2608 | 14.6401 | 1 | 2 | 2 | 2 | 2 | 2 | 1 | 2 |
| TMEM201 | Q5SNT2 | 0.002173 | 0.000423 | 0.148551 | | 0.495414 | 1.08499 | | 13.843 | 13.9782 | 14.1818 | 13.8784 | 14.1208 | 13.9273 | 13.6118 | 13.7238 | 7 | 6 | 6 | 2 | 2 | 6 | 6 | 2 |
| CDK19 | Q9BWU1 | 0.002633 | 0.0005 | 0.148696 | | 0.186079 | 0.475109 | | 13.2869 | 13.548 | 13.531 | 13.3769 | 12.7358 | 13.9659 | 12.8522 | 13.6022 | 3 | 3 | 3 | 3 | 2 | 1 | 3 | 3 |
| BUB3 | O43684;O4 | 3.66E-17 | 9.83E-17 | 0.148768 | | 1.17224 | 2.23006 | | 18.1398 | 18.1301 | 18.0776 | 18.1556 | 17.8917 | 17.9126 | 17.9069 | 17.9686 | 12 | 12 | 12 | 12 | 12 | 12 | 12 | 12 |
| MRPS9 | P82933 | 0.000263 | 5.89E-05 | 0.148848 | | 0.867532 | 1.72298 | | 16.3913 | 16.3302 | 16.3841 | 16.4194 | 16.2272 | 16.2634 | 16.0295 | 16.1591 | 11 | 10 | 10 | 10 | 8 | 8 | 9 | 9 |
| CDK5RAP3 | Q96JB5 | 6.06E-13 | 4.62E-13 | 0.148874 | | 0.597914 | 1.26614 | | 16.8054 | 16.8195 | 16.7848 | 16.4449 | 16.5588 | 16.9004 | 16.8247 | 16.5431 | 20 | 21 | 22 | 20 | 18 | 20 | 21 | 21 |
| ARHGAP22 | Q7Z5H3;Q7 | 1.57E-13 | 1.42E-13 | 0.149001 | | 0.27563 | 0.66609 | | 15.356 | 15.3558 | 15.3247 | 15.5386 | 15.5843 | 15.1269 | 14.6766 | 15.056 | 8 | 8 | 6 | 9 | 8 | 4 | 6 | 5 |
| SAP18 | O00422;O0 | 3.20E-12 | 2.05E-12 | 0.149481 | + | 2.5598 | 4.88385 | | 17.4202 | 17.3849 | 17.312 | 17.212 | 17.1944 | 17.202 | 17.2279 | 17.2781 | 7 | 7 | 7 | 7 | 7 | 8 | 7 | 8 |
| FARSA | Q9Y285 | 3.34E-12 | 2.13E-12 | 0.149667 | | 0.651816 | 1.35924 | | 17.9086 | 17.9283 | 17.8573 | 17.7994 | 17.479 | 17.9893 | 17.7259 | 17.7482 | 20 | 20 | 20 | 20 | 20 | 20 | 20 | 20 |
| ZCCHC24 | Q8N2G6 | 0.002237 | 0.000434 | 0.149842 | | 0.506306 | 1.10455 | | 14.6693 | 14.5423 | 14.8432 | 14.3431 | 14.827 | 14.6567 | 14.4304 | 14.4184 | 2 | 2 | 2 | 2 | 1 | 2 | 2 | 2 |
| MYL9 | P24844 | 7.05E-05 | 1.70E-05 | 0.15012 | | 0.303123 | 0.721754 | | 20.7365 | 20.7419 | 20.9045 | 20.6986 | 20.135 | 21.0982 | 20.6511 | 20.6379 | 12 | 12 | 11 | 10 | 9 | 11 | 10 | 11 |
| PHF14 | O94880 | 3.12E-05 | 7.79E-06 | 0.150244 | | 0.409024 | 0.926525 | | 14.8194 | 14.8391 | 14.8213 | 14.8816 | 15.0357 | 14.4014 | 14.4733 | 14.5897 | 6 | 5 | 6 | 4 | 3 | 2 | 4 | 4 |
| YIF1B | Q5BJH7;Q5 | 2.13E-09 | 8.41E-10 | 0.15027 | | 0.364585 | 0.842251 | | 17.5846 | 17.5734 | 17.537 | 17.7639 | 17.0007 | 17.3531 | 17.317 | 17.6388 | 5 | 5 | 5 | 5 | 5 | 5 | 5 | 5 |
| CSTF3 | Q12996 | 2.87E-19 | 1.53E-18 | 0.150435 | | 0.99706 | 1.9383 | | 15.8489 | 15.8716 | 15.7787 | 15.7081 | 15.6615 | 15.7063 | 15.494 | 15.8433 | 17 | 16 | 16 | 16 | 12 | 17 | 18 | 15 |
| PPP1R7 | Q15435 | 3.27E-14 | 3.55E-14 | 0.150509 | + | 1.38151 | 2.58405 | | 17.5702 | 17.6351 | 17.637 | 17.5 | 17.523 | 17.3063 | 17.5379 | 17.4508 | 23 | 23 | 23 | 21 | 18 | 21 | 21 | 22 |
| STX18 | Q9P2W9 | 2.60E-16 | 5.31E-16 | 0.15053 | | 0.954529 | 1.86766 | | 16.2777 | 16.3124 | 16.3 | 16.1371 | 16.1488 | 16.3293 | 15.9501 | 16.1656 | 10 | 10 | 12 | 12 | 7 | 11 | 10 | 11 |
| TRIM21 | P19474 | 3.19E-08 | 1.09E-08 | 0.15061 | | 0.682728 | 1.41211 | | 16.0481 | 16.1236 | 16.1668 | 16.1163 | 16.1714 | 15.8884 | 15.7627 | 15.8726 | 12 | 11 | 12 | 12 | 9 | 11 | 9 | 9 |
| DNAJC9 | Q8WXX5 | 1.05E-09 | 4.36E-10 | 0.150689 | | 0.405223 | 0.919399 | | 17.067 | 17.0754 | 17.0616 | 17.2534 | 16.8063 | 16.5367 | 16.8954 | 17.0948 | 9 | 10 | 10 | 11 | 8 | 9 | 11 | 9 |
| C3 | P01024 | 0.004899 | 0.000877 | 0.150794 | | 0.328858 | 0.826817 | | 15.079 | 14.9266 | 14.6774 | NaN | 14.6065 | NaN | 14.8806 | NaN | 2 | 2 | 2 | NaN | 1 | NaN | 1 | NaN |
| ZRANB2 | O95218;O9 | 7.49E-06 | 1.99E-06 | 0.150918 | | 0.580275 | 1.23539 | | 16.0112 | 16.125 | 16.2502 | 16.1266 | 15.6904 | 16.1096 | 16.0741 | 15.8887 | 8 | 9 | 9 | 9 | 5 | 7 | 7 | 5 |
| IFT74 | Q96LB3 | 7.20E-09 | 2.67E-09 | 0.150941 | | 0.553469 | 1.18835 | | 14.5961 | 14.5229 | 14.7046 | 14.2677 | 14.7851 | 14.3691 | 14.5097 | 14.353 | 3 | 5 | 5 | 3 | 3 | 3 | 4 | 4 |
| UXT | Q9UBK9;Q9 | 2.36E-18 | 9.22E-18 | 0.151029 | | 0.295368 | 0.706173 | | 15.4452 | 15.4791 | 15.5462 | 15.6298 | 15.1503 | 14.7996 | 15.525 | 15.5911 | 3 | 4 | 4 | 4 | 3 | 3 | 3 | 4 |
| GCFC2 | P16383;P1 | 0.003572 | 0.000654 | 0.151197 | | 0.404533 | 0.918103 | | 15.0937 | 14.9032 | 15.0898 | 14.8004 | 15.2494 | 14.5219 | 14.8422 | 14.9747 | 4 | 4 | 3 | 2 | 3 | 2 | 3 | 3 |
| NCK1 | P16333 | 1.54E-12 | 1.06E-12 | 0.151208 | | 0.520296 | 1.12955 | | 15.9211 | 15.9927 | 16.0127 | 15.9868 | 15.5177 | 16.0624 | 15.8651 | 15.6894 | 16 | 16 | 16 | 17 | 15 | 17 | 16 | 15 |
| GNPDA1 | P46926 | 1.26E-06 | 3.61E-07 | 0.151442 | | 0.888546 | 1.75797 | | 18.0956 | 18.1435 | 18.1161 | 17.8079 | 18.0193 | 18.1857 | 17.9205 | 17.9015 | 12 | 12 | 11 | 11 | 11 | 11 | 11 | 11 |
| NCAPH | Q15003 | 1.42E-09 | 5.76E-10 | 0.151541 | | 0.280843 | 0.676739 | | 15.7347 | 15.6892 | 15.7136 | 15.9327 | 15.7693 | 15.0016 | 15.3656 | 15.7356 | 14 | 12 | 15 | 14 | 12 | 7 | 13 | 14 |
| SLC15A4 | Q8N697 | 0.000793 | 0.000166 | 0.151762 | | 1.23615 | 2.33732 | | 16.4155 | 16.3978 | 16.3925 | 16.0968 | 16.188 | 16.3116 | 16.3725 | 16.2819 | 6 | 6 | 7 | 8 | 6 | 5 | 6 | 6 |
| TSNAX | Q99598 | 3.89E-15 | 5.60E-15 | 0.151848 | | 0.588936 | 1.25051 | | 16.8374 | 16.864 | 16.8616 | 16.7259 | 16.5485 | 17.0442 | 16.5641 | 16.63 | 11 | 10 | 11 | 11 | 11 | 11 | 11 | 11 |
| ARHGEF18 | Q6ZSZ5;Q6 | 5.78E-08 | 1.92E-08 | 0.151985 | | 0.304239 | 0.723989 | | 15.1421 | 15.2576 | 15.258 | 15.0591 | 15.4633 | 14.5826 | 14.9031 | 15.3281 | 9 | 9 | 8 | 7 | 8 | 7 | 10 | 9 |
| CHID1 | Q9BWS9 | 4.54E-13 | 3.59E-13 | 0.152111 | | 0.416093 | 0.939741 | | 16.2553 | 16.2608 | 16.2528 | 15.8879 | 15.7924 | 16.4249 | 16.3227 | 16.093 | 12 | 12 | 12 | 12 | 10 | 12 | 12 | 12 |
| TMEM147 | Q9BVK8;Q9 | 3.70E-12 | 2.33E-12 | 0.152206 | | 0.608826 | 1.28509 | | 16.9378 | 17.0644 | 17.2496 | 17.0633 | 16.7532 | 16.7992 | 17.1349 | 16.9081 | 3 | 3 | 3 | 3 | 2 | 3 | 3 | 3 |
| DVL2 | O14641 | 0.00011 | 2.60E-05 | 0.152262 | | 0.773903 | 1.56636 | | 15.6245 | 15.7309 | 15.6952 | 15.7948 | 15.3949 | 15.5574 | 15.4529 | 15.4565 | 9 | 10 | 9 | 10 | 7 | 9 | 8 | 8 |
| HNRNPA2B1 | P22626 | 6.97E-07 | 2.06E-07 | 0.152307 | + | 1.6059 | 2.97553 | | 20.6699 | 20.7392 | 20.6657 | 20.5797 | 20.5898 | 20.397 | 20.5521 | 20.5777 | 21 | 21 | 21 | 21 | 20 | 20 | 21 | 21 |
| ENY2 | Q9NPA8;Q9 | 2.25E-05 | 5.70E-06 | 0.152579 | | 0.155642 | 0.406024 | | 16.1707 | 15.6992 | 16.3666 | 16.4984 | 15.6256 | 15.1662 | 15.8252 | 16.5159 | 6 | 4 | 7 | 4 | 2 | 2 | 4 | 6 |
| STK4 | Q13043 | 1.36E-13 | 1.24E-13 | 0.152747 | | 0.59487 | 1.26085 | | 16.2487 | 16.2389 | 16.2086 | 16.3392 | 15.9821 | 16.0545 | 15.8141 | 16.2068 | 11 | 11 | 10 | 12 | 9 | 10 | 9 | 12 |
| CROCC | Q5TZA2 | 0.001078 | 0.000221 | 0.1528 | | 0.498685 | 1.09087 | | 15.3362 | 15.3185 | 15.4124 | 14.8151 | 15.392 | 15.3734 | 15.2257 | 15.2082 | 9 | 9 | 8 | 6 | 2 | 6 | 4 | 5 |
| ALDH2 | P05091 | 6.13E-19 | 2.87E-18 | 0.153107 | | 0.319731 | 0.754821 | | 16.6944 | 16.7303 | 16.7354 | 16.4207 | 16.3023 | 17.1269 | 16.6436 | 16.3411 | 21 | 20 | 20 | 19 | 18 | 20 | 21 | 20 |
| U2AF1L5;U2AF1 | P0DN76;Q0 | 1.60E-08 | 5.67E-09 | 0.153145 | + | 2.2219 | 4.15175 | | 18.003 | 18.0094 | 17.9492 | 17.8343 | 17.8281 | 17.7429 | 17.8889 | 17.876 | 9 | 9 | 10 | 10 | 9 | 9 | 9 | 9 |
| CHMP2A | O43633 | 7.09E-11 | 3.49E-11 | 0.153345 | | 1.28906 | 2.42664 | | 17.1543 | 17.1083 | 17.0521 | 16.9718 | 17.1028 | 16.952 | 16.8945 | 16.8368 | 8 | 8 | 8 | 8 | 7 | 8 | 8 | 8 |
| RPS6KA4 | O75676-2 | 2.15E-12 | 1.43E-12 | 0.153361 | | 0.344829 | 0.804061 | | 14.7315 | 14.6706 | 14.7852 | 14.9381 | 14.7948 | 14.1715 | 14.3384 | 14.6358 | 9 | 9 | 8 | 10 | 6 | 6 | 5 | 5 |
| IAH1 | Q2TAA2 | 1.83E-08 | 6.45E-09 | 0.15367 | | 0.31937 | 0.754106 | | 17.6081 | 17.411 | 17.4905 | 17.3381 | 17.4916 | 17.5475 | 17.5938 | 16.7765 | 7 | 7 | 7 | 7 | 7 | 8 | 6 | 8 |
| CDIPT | O14735 | 2.09E-05 | 5.31E-06 | 0.154183 | | 0.976345 | 1.9039 | | 17.6568 | 17.3686 | 17.5588 | 17.273 | 17.3469 | 17.3186 | 17.4584 | 17.4725 | 5 | 5 | 5 | 5 | 5 | 5 | 5 | 5 |
| MPG | P29372;P2 | 9.07E-14 | 8.74E-14 | 0.154195 | | 0.588491 | 1.24974 | | 16.249 | 16.1812 | 16.2241 | 16.2192 | 16.1826 | 15.7718 | 15.9226 | 16.2232 | 8 | 8 | 7 | 8 | 8 | 8 | 8 | 8 |
| RBM7 | Q9Y580 | 1.17E-09 | 4.81E-10 | 0.154788 | | 0.138086 | 0.364995 | | 14.8782 | 15.0866 | 15.089 | 15.5489 | 13.7112 | 15.2459 | 14.7523 | 15.0574 | 5 | 4 | 4 | 4 | 1 | 2 | 2 | 3 |
| PABPN1 | Q86U42;Q8 | 4.76E-16 | 8.81E-16 | 0.154797 | | 0.650761 | 1.35743 | | 16.9296 | 17.187 | 17.1775 | 17.0353 | 17.1737 | 16.7902 | 16.8119 | 16.9051 | 9 | 8 | 9 | 8 | 6 | 6 | 8 | 6 |
| ZFYVE27 | Q5T4F4;Q5 | 0.001726 | 0.000343 | 0.154815 | | 0.443428 | 1.00684 | | 13.6153 | 13.3863 | 13.6037 | NaN | 13.1253 | 13.2848 | 13.4254 | 13.6856 | 3 | 3 | 3 | NaN | 1 | 3 | 2 | 2 |
| SSB | P05455 | 4.88E-15 | 6.78E-15 | 0.154976 | | 1.04912 | 2.02478 | | 17.1942 | 17.2134 | 17.1975 | 17.0258 | 16.8953 | 17.1732 | 17.1821 | 16.9571 | 34 | 33 | 34 | 33 | 30 | 33 | 33 | 33 |
| PPIH | O43447 | 8.69E-08 | 2.82E-08 | 0.155353 | + | 2.77175 | 5.38266 | | 16.8071 | 16.9031 | 16.8301 | 16.6347 | 16.693 | 16.7089 | 16.7032 | 16.7173 | 7 | 8 | 7 | 7 | 7 | 7 | 7 | 7 |

| Gene names | UniProt IDs | Protein Group P-value | Protein Group Q-value | Log2 Difference NUP214 vs Controls (n=5) | p<0.05 | -log10 p-value | T-test Test statistic | NPC Localization | Log2 MS2_quantity_NUP214_1 | Log2 MS2_quantity_NUP214_2 | Log2 MS2_quantity_NUP214_3 | Log2 MS2_quantity_Control_1 | Log2 MS2_quantity_Control_2 | Log2 MS2_quantity_Control_3 | Log2 MS2_quantity_Control_4 | Log2 MS2_quantity_Control_5 | Number Peptides for Quantitation NUP214_1 | Number Peptides for Quantitation NUP214_2 | Number Peptides for Quantitation NUP214_3 | Number Peptides for Quantitation Control_1 | Number Peptides for Quantitation Control_2 | Number Peptides for Quantitation Control_3 | Number Peptides for Quantitation Control_4 | Number Peptides for Quantitation Control_5 |
|---|---|---|---|---|---|---|---|---|---|---|---|---|---|---|---|---|---|---|---|---|---|---|---|---|
| CERS6 | Q6ZMG9;Q | 0.000381 | 8.34E-05 | 0.155496 | | 0.114913 | 0.312164 | | 14.8762 | 14.4598 | 14.5745 | NaN | 14.8131 | 13.2973 | 14.6255 | 15.1895 | 3 | 4 | 5 | NaN | 3 | 2 | 2 | 2 |
| MTREX | P42285 | 2.55E-13 | 2.18E-13 | 0.155609 | | 1.2221 | 2.31368 | | 16.1139 | 16.1896 | 16.1147 | 16.1195 | 15.8884 | 15.8721 | 15.9718 | 16.0673 | 34 | 35 | 33 | 34 | 27 | 31 | 30 | 32 |
| PPP1R37 | O75864 | 5.08E-07 | 1.53E-07 | 0.155725 | | 0.271044 | 0.656687 | | 15.2239 | 15.0381 | 14.9998 | 14.8024 | 15.4088 | 14.5211 | 14.6553 | 15.2702 | 5 | 6 | 4 | 4 | 4 | 2 | 2 | 4 |
| NAPA | P54920 | 2.43E-17 | 6.91E-17 | 0.156068 | | 0.341603 | 0.797778 | | 18.3252 | 18.3795 | 18.3707 | 18.5534 | 17.9401 | 17.7871 | 18.2914 | 18.44 | 19 | 18 | 20 | 19 | 19 | 18 | 19 | 19 |
| CORO2B | Q9UQ03;Q | 8.28E-09 | 3.04E-09 | 0.156083 | | 0.39412 | 0.898498 | | 16.6243 | 16.6098 | 16.5645 | 16.8248 | 16.391 | 16.3225 | 16.0628 | 16.6162 | 8 | 10 | 12 | 9 | 3 | 7 | 4 | 7 |
| GLOD4 | Q9HC38-2 | 3.44E-09 | 1.33E-09 | 0.156146 | | 0.763285 | 1.54851 | | 17.8309 | 17.794 | 17.8457 | 17.6754 | 17.4692 | 17.9014 | 17.5509 | 17.7402 | 19 | 18 | 18 | 18 | 17 | 18 | 18 | 18 |
| PARP12 | Q9H0J9 | 0.014333 | 0.002419 | 0.156308 | | 0.25986 | 0.641044 | | 14.7274 | 15.0268 | 14.707 | 14.2078 | NaN | 15.0634 | 14.4972 | 14.8878 | 3 | 2 | 3 | 1 | NaN | 2 | 1 | 1 |
| CREB1 | P16220;P1 | 1.87E-14 | 2.19E-14 | 0.157084 | | 0.896284 | 1.77085 | | 17.5021 | 17.462 | 17.4624 | 17.3276 | 17.1726 | 17.2216 | 17.3138 | 17.5566 | 4 | 4 | 4 | 3 | 4 | 4 | 4 | 4 |
| CNPY3 | Q9BT09 | 2.08E-15 | 3.26E-15 | 0.157246 | | 0.373729 | 0.85977 | | 17.1676 | 17.1713 | 17.2291 | 16.8108 | 16.8826 | 17.1677 | 17.5078 | 16.7915 | 11 | 11 | 11 | 11 | 11 | 11 | 10 | 10 |
| CDC16 | Q13042 | 5.60E-20 | 3.77E-19 | 0.157341 | | 0.783808 | 1.583 | | 15.7084 | 15.5622 | 15.6686 | 15.4894 | 15.2953 | 15.6112 | 15.6743 | 15.3752 | 13 | 12 | 15 | 10 | 11 | 13 | 10 | 10 |
| LMNB2 | Q03252 | 5.34E-09 | 2.01E-09 | 0.157522 | + | 2.0772 | 3.85907 | | 18.0188 | 17.9817 | 17.9388 | 17.8586 | 17.7471 | 17.8226 | 17.7789 | 17.904 | 45 | 44 | 46 | 46 | 43 | 45 | 44 | 45 |
| NDRG1 | Q92597 | 1.73E-12 | 1.18E-12 | 0.157574 | | 0.179576 | 0.460553 | | 18.4067 | 18.3951 | 18.4436 | 18.6514 | 18.1289 | 18.9636 | 17.4771 | 18.0667 | 7 | 7 | 7 | 7 | 8 | 7 | 8 | 7 |
| EIF4A3 | P38919 | 1.25E-08 | 4.49E-09 | 0.157577 | + | 2.49475 | 4.73718 | | 17.8276 | 17.9028 | 17.8719 | 17.7219 | 17.7262 | 17.6598 | 17.6639 | 17.7774 | 19 | 19 | 19 | 17 | 20 | 18 | 19 | 18 |
| SAR1B | Q9Y6B6 | 4.59E-06 | 1.24E-06 | 0.157625 | | 0.79649 | 1.60427 | | 19.7363 | 19.5394 | 19.7457 | 19.766 | 19.4086 | 19.465 | 19.4515 | 19.4897 | 7 | 8 | 7 | 7 | 8 | 7 | 8 | 7 |
| LYRM4 | Q9HD34 | 1.49E-05 | 3.84E-06 | 0.157707 | | 0.385137 | 0.894648 | | 14.5059 | 14.232 | 14.3552 | 14.4891 | 14.1638 | NaN | 13.8436 | 14.3303 | 1 | 3 | 3 | 2 | 1 | NaN | 2 | 3 |
| CORO1A | P31146 | 0.00384 | 0.0007 | 0.15776 | | 0.20155 | 0.509323 | | 14.3972 | 14.375 | 14.6308 | 14.7648 | 14.8592 | 14.0863 | 13.623 | 14.2163 | 3 | 3 | 4 | 3 | 2 | 2 | 1 | 3 |
| NUCKS1 | Q9H1E3 | 1.87E-10 | 8.59E-11 | 0.15777 | | 0.418438 | 0.944113 | | 17.2745 | 17.125 | 17.2713 | 16.7601 | 17.4376 | 17.2512 | 16.9709 | 16.9093 | 7 | 6 | 9 | 7 | 6 | 8 | 7 | 7 |
| DAP3 | P51398;P5 | 3.94E-15 | 5.65E-15 | 0.158253 | + | 2.10198 | 3.90838 | | 16.3765 | 16.3655 | 16.3456 | 16.2642 | 16.2462 | 16.2289 | 16.0963 | 16.1858 | 12 | 13 | 14 | 13 | 10 | 10 | 11 | 12 |
| NUMBL | Q9Y6R0 | 9.12E-09 | 3.33E-09 | 0.158288 | | 0.913873 | 1.80011 | | 16.1956 | 16.1143 | 16.1922 | 16.1829 | 15.9556 | 16.0547 | 15.796 | 16.056 | 9 | 9 | 9 | 8 | 6 | 8 | 7 | 9 |
| FBXL20 | Q96IG2 | 7.82E-08 | 2.55E-08 | 0.158335 | | 0.479715 | 1.05665 | | 16.0039 | 15.9196 | 15.8884 | 15.4175 | 15.9407 | 15.8157 | 16.0528 | 15.6682 | 4 | 5 | 5 | 4 | 4 | 4 | 4 | 4 |
| XXYLT1 | Q8NBI6 | 1.45E-13 | 1.32E-13 | 0.15846 | | 0.34506 | 0.80451 | | 15.5523 | 15.4312 | 15.4474 | 15.2582 | 15.1637 | 15.5529 | 15.7243 | 14.8935 | 11 | 11 | 11 | 9 | 8 | 10 | 10 | 9 |
| DGAT1 | O75907 | 4.26E-09 | 1.63E-09 | 0.15852 | | 0.258943 | 0.631695 | | 14.5976 | 14.6607 | 14.5407 | 13.7461 | 14.431 | 14.7773 | 14.7617 | 14.4896 | 3 | 3 | 4 | 4 | 4 | 5 | 5 | 4 |
| TMEM214 | Q6NUQ4 | 1.45E-15 | 2.38E-15 | 0.15868 | | 1.27059 | 2.39539 | | 17.2515 | 17.2293 | 17.2348 | 16.9884 | 16.9677 | 17.0551 | 17.2211 | 17.1669 | 24 | 23 | 22 | 22 | 19 | 24 | 23 | 23 |
| NAT14 | Q8WUY8 | 6.24E-08 | 2.06E-08 | 0.158823 | | 0.766973 | 1.55471 | | 16.917 | 16.8202 | 16.7542 | 16.4988 | 16.7488 | 16.8969 | 16.674 | 16.5397 | 4 | 5 | 5 | 4 | 3 | 5 | 4 | 3 |
| MRC2 | Q9UBG0 | 3.62E-17 | 9.75E-17 | 0.158979 | | 0.502129 | 1.09706 | | 17.8929 | 17.9951 | 18.0253 | 17.8342 | 17.5746 | 18.1611 | 17.885 | 17.6056 | 28 | 32 | 31 | 30 | 31 | 29 | 30 | 31 |
| CNOT2 | Q9NZN8 | 2.73E-13 | 2.31E-13 | 0.158997 | + | 1.88412 | 3.48528 | | 16.2432 | 16.1615 | 16.0768 | 15.9971 | 15.9958 | 16.0575 | 15.927 | 16.0301 | 7 | 9 | 7 | 9 | 5 | 6 | 6 | 6 |
| ZNF512 | Q96ME7;Q | 0.001326 | 0.000269 | 0.159153 | + | 1.68761 | 3.12209 | | 15.1257 | 15.1658 | 15.1527 | 14.8596 | 14.9689 | 15.0887 | 15.021 | 15.0062 | 8 | 8 | 8 | 7 | 5 | 7 | 7 | 6 |
| CEBPB | P17676;P1 | 0.000129 | 3.01E-05 | 0.159162 | | 0.454289 | 1.01034 | | 15.6303 | 15.5646 | 15.5963 | 15.541 | 15.7552 | 15.4495 | 15.0318 | 15.4119 | 4 | 4 | 5 | 5 | 4 | 4 | 3 | 4 |
| ACIN1 | Q9UKV3 | 9.40E-16 | 1.63E-15 | 0.159397 | + | 2.14028 | 3.98525 | | 16.0461 | 16.0963 | 16.111 | 15.9441 | 16.0054 | 15.8587 | 15.9527 | 15.8644 | 25 | 26 | 27 | 25 | 20 | 24 | 24 | 24 |
| FBXO7 | Q9Y3I1 | 3.71E-08 | 1.26E-08 | 0.159609 | | 1.09075 | 2.09405 | | 16.72 | 16.6609 | 16.7501 | 16.6828 | 16.5843 | 16.6188 | 16.5071 | 16.3606 | 7 | 7 | 6 | 5 | 6 | 6 | 5 | 6 |
| GTF3C2 | Q8WUA4 | 0.000606 | 0.000129 | 0.159779 | | 0.656238 | 1.36682 | | 14.637 | 14.4064 | 14.5443 | 14.2711 | 14.6728 | 14.2313 | 14.3797 | 14.2924 | 7 | 6 | 8 | 7 | 2 | 5 | 6 | 6 |
| SDHA | P31040 | 4.20E-13 | 3.36E-13 | 0.160014 | + | 1.59182 | 2.9505 | | 18.0237 | 18.0622 | 18.0542 | 17.9289 | 17.964 | 17.9473 | 17.7487 | 17.8445 | 23 | 23 | 23 | 23 | 20 | 23 | 23 | 21 |
| QRICH1 | Q2TAL8 | 2.56E-11 | 1.38E-11 | 0.160029 | | 1.12493 | 2.15101 | | 15.4654 | 15.4643 | 15.3818 | 15.3015 | 15.1631 | 15.1539 | 15.4393 | 15.328 | 10 | 7 | 10 | 8 | 7 | 7 | 9 | 10 |
| PRKRA | O75569 | 1.52E-11 | 8.55E-12 | 0.16021 | | 0.762671 | 1.54747 | | 16.9113 | 16.8854 | 16.9408 | 16.6174 | 16.6536 | 16.9513 | 16.9291 | 16.61 | 9 | 10 | 9 | 8 | 9 | 9 | 9 | 8 |
| DDB1 | Q16531 | 1.16E-14 | 1.44E-14 | 0.160501 | + | 3.03133 | 6.04131 | | 18.0508 | 18.0914 | 18.0745 | 17.8477 | 17.9431 | 17.9433 | 17.9349 | 17.8897 | 47 | 51 | 49 | 46 | 44 | 47 | 47 | 46 |
| FUBP1 | Q96AE4-2 | 6.31E-07 | 1.87E-07 | 0.160663 | + | 2.28089 | 4.27447 | | 18.5139 | 18.5267 | 18.5417 | 18.4653 | 18.3619 | 18.3067 | 18.3217 | 18.3782 | 25 | 24 | 25 | 23 | 24 | 23 | 24 | 25 |
| KIF1B | O60333-2 | 3.02E-10 | 1.34E-10 | 0.160785 | | 0.352246 | 0.818455 | | 15.547 | 15.5751 | 15.5374 | 15.9298 | 15.2546 | 15.1975 | 15.1075 | 15.4726 | 19 | 18 | 18 | 19 | 12 | 13 | 13 | 18 |
| FBXW5 | Q969U6;Q | 0.000446 | 9.67E-05 | 0.160906 | | 0.22153 | 0.567936 | | 14.5988 | 14.4045 | 14.5396 | 14.4848 | NaN | NaN | 13.8209 | 14.7545 | 3 | 3 | 4 | 3 | NaN | NaN | 2 | 2 |
| VAMP4 | O75379;O | 1.98E-13 | 1.74E-13 | 0.161063 | | 0.594502 | 1.26021 | | 15.2584 | 15.0591 | 15.1972 | 15.2609 | 14.8291 | 15.0398 | 14.7859 | 15.1369 | 3 | 3 | 3 | 3 | 3 | 3 | 3 | 3 |
| GSK3B | P49841-2 | 8.01E-16 | 1.41E-15 | 0.161264 | | 1.1265 | 2.15363 | | 16.4944 | 16.3399 | 16.3915 | 16.1349 | 16.3856 | 16.2 | 16.1674 | 16.3488 | 13 | 14 | 14 | 13 | 13 | 13 | 14 | 14 |
| CTSC | P53634 | 2.34E-14 | 2.66E-14 | 0.161302 | | 0.084399 | 0.233168 | | 15.7602 | 15.7778 | 15.6625 | 13.669 | 16.2562 | 15.7369 | 16.6923 | 15.5065 | 10 | 10 | 10 | 8 | 11 | 10 | 11 | 10 |
| GOSR1 | O95249 | 3.98E-12 | 2.50E-12 | 0.161303 | | 0.539226 | 1.16319 | | 16.4913 | 16.5408 | 16.4909 | 16.5202 | 16.0123 | 16.5713 | 16.2136 | 16.4144 | 6 | 6 | 7 | 6 | 6 | 8 | 8 | 7 |
| NDUFB10 | O96000 | 9.00E-13 | 6.59E-13 | 0.161383 | | 0.58729 | 1.24764 | | 16.9933 | 16.9029 | 17.0127 | 16.9868 | 16.7395 | 16.4667 | 16.9495 | 16.8988 | 8 | 8 | 8 | 8 | 7 | 6 | 7 | 7 |
| CTCF | P49711 | 1.72E-11 | 9.59E-12 | 0.16144 | | 1.24366 | 2.34997 | | 14.1492 | 14.2218 | 14.1825 | 14.065 | 14.131 | 13.8328 | 14.0474 | 14.0392 | 5 | 5 | 6 | 5 | 4 | 5 | 5 | 4 |
| MDH1 | P40925;P4 | 2.90E-09 | 1.13E-09 | 0.161535 | | 0.792647 | 1.59783 | | 19.9792 | 19.9928 | 20.0025 | 19.6593 | 19.7916 | 20.0304 | 19.9825 | 19.6861 | 18 | 19 | 19 | 18 | 17 | 18 | 17 | 19 |
| DHX9 | Q08211 | 2.75E-16 | 5.48E-16 | 0.16169 | + | 1.68121 | 3.11054 | | 18.7266 | 18.7281 | 18.7048 | 18.6655 | 18.5629 | 18.4333 | 18.5268 | 18.6022 | 56 | 53 | 55 | 55 | 54 | 53 | 53 | 55 |
| POU2F2 | P09086;P0 | 1.12E-07 | 3.61E-08 | 0.161986 | | 0.495703 | 1.08551 | | 14.5039 | 14.3422 | 14.1976 | 14.2769 | 14.4921 | 14.1626 | 13.8743 | 14.1237 | 3 | 2 | 3 | 2 | 3 | 2 | 2 | 2 |
| RASA1 | P20936;P2 | 1.30E-13 | 1.20E-13 | 0.162386 | | 0.694325 | 1.43186 | | 16.654 | 16.7311 | 16.6873 | 16.3684 | 16.8103 | 16.3428 | 16.5498 | 16.5707 | 37 | 38 | 38 | 37 | 35 | 36 | 36 | 37 |
| DERA | Q9Y315 | 3.57E-20 | 2.68E-19 | 0.162513 | | 0.309081 | 0.733663 | | 16.0115 | 15.9072 | 15.8882 | 15.8304 | 15.8042 | 16.1248 | 15.9519 | 15.154 | 13 | 13 | 14 | 13 | 12 | 13 | 13 | 11 |
| SNRPGP15;SNRPG | A8MWD9;I | 0.000114 | 2.68E-05 | 0.162911 | | 0.266006 | 0.646312 | | 19.2167 | 19.3041 | 19.5061 | 19.5127 | 18.8914 | 18.785 | 18.9934 | 19.7147 | 3 | 3 | 3 | 3 | 3 | 3 | 3 | 3 |
| PPP2CB | P62714 | 4.84E-11 | 2.47E-11 | 0.163156 | | 0.528681 | 1.14448 | | 19.2082 | 19.2475 | 19.1631 | 19.3368 | 18.7171 | 19.1959 | 19.0136 | 18.9522 | 13 | 13 | 13 | 13 | 13 | 13 | 13 | 13 |
| SMARCAD1 | Q9H4L7;Q | 0.000157 | 3.63E-05 | 0.163197 | | 0.587667 | 1.2483 | | 14.5222 | 14.4742 | 14.5279 | 14.5485 | 14.1374 | 14.0804 | 14.4716 | 14.4864 | 7 | 6 | 8 | 7 | 3 | 5 | 6 | 7 |
| MAPKAPK3 | Q16644 | 0.005206 | 0.000928 | 0.163218 | | 0.361197 | 0.835736 | | 14.4652 | 14.2099 | 14.4492 | 14.0138 | 13.8041 | 14.2241 | 14.4476 | 14.5682 | 1 | 3 | 2 | 2 | 2 | 2 | 2 | 1 |
| PRKCD | Q05655;Q | 5.47E-10 | 2.34E-10 | 0.163249 | | 0.37808 | 0.868072 | | 15.6395 | 15.6961 | 15.6362 | 15.9132 | 15.6428 | 15.3289 | 15.0812 | 15.5041 | 11 | 14 | 11 | 14 | 12 | 12 | 11 | 13 |
| SRA1 | Q9HD15 | 0.000715 | 0.000151 | 0.163423 | | 0.167715 | 0.433723 | | 15.8125 | 15.5749 | 16.0325 | 16.2161 | 15.0651 | 14.9481 | 15.768 | 16.2188 | 7 | 7 | 6 | 6 | 5 | 6 | 6 | 6 |
| METTL15 | A6NJ78 | 0.00216 | 0.00042 | 0.163514 | | 0.56253 | 1.20429 | | 13.7393 | 13.9756 | 13.9263 | 14.067 | 13.7194 | 13.5107 | 13.6283 | 13.6589 | 5 | 4 | 2 | 3 | 2 | 3 | 3 | 4 |
| RANBP6 | O60518 | 5.55E-06 | 1.49E-06 | 0.163847 | | 0.646694 | 1.35044 | | 15.0119 | 14.8834 | 15.0408 | 14.8843 | 14.722 | 14.5208 | 14.9478 | 14.9992 | 14 | 14 | 14 | 11 | 11 | 6 | 10 | 9 |
| TACC2 | O95359-2; | 0.00253 | 0.000483 | 0.164095 | | 0.484899 | 1.06603 | | 14.8106 | 14.6609 | 14.8801 | 14.4099 | 14.9093 | 14.3212 | 14.7451 | 14.7133 | 5 | 6 | 8 | 3 | 5 | 3 | 4 | 3 |
| OAT | P04181 | 4.62E-13 | 3.64E-13 | 0.164298 | + | 1.39804 | 2.61242 | | 18.9456 | 18.991 | 19.0031 | 18.7383 | 18.7361 | 18.7539 | 18.9613 | 18.8885 | 21 | 20 | 21 | 20 | 20 | 20 | 21 | 20 |
| PRPF8 | Q6P2Q9 | 6.87E-15 | 9.09E-15 | 0.164638 | + | 2.34494 | 4.41003 | | 17.1047 | 17.1379 | 17.0583 | 16.9799 | 16.8441 | 16.9619 | 16.9711 | 16.9214 | 83 | 85 | 84 | 83 | 77 | 81 | 82 | 83 |
| PPP4R2 | Q9NY27 | 2.47E-11 | 1.34E-11 | 0.165136 | | 0.672983 | 1.39548 | | 14.7278 | 14.8063 | 14.8064 | 14.832 | 14.3482 | 14.5021 | 14.7642 | 14.6286 | 10 | 10 | 10 | 9 | 3 | 8 | 7 | 10 |
| ARHGAP12 | Q8IWW6;Q | 8.68E-12 | 5.10E-12 | 0.165507 | | 0.723303 | 1.48103 | | 14.9035 | 14.9371 | 14.9286 | 14.7353 | 15.0083 | 14.5357 | 14.6375 | 14.871 | 18 | 20 | 18 | 16 | 10 | 12 | 16 | 13 |
| DKC1 | O60832 | 8.87E-13 | 6.51E-13 | 0.165786 | | 0.662355 | 1.3773 | | 16.097 | 16.2084 | 16.1274 | 15.8078 | 15.7835 | 16.2374 | 16.1226 | 15.941 | 22 | 21 | 23 | 22 | 22 | 25 | 24 | 23 |
| SLC25A5 | P05141 | 5.86E-08 | 1.94E-08 | 0.166017 | + | 1.89706 | 3.50978 | | 20.2311 | 20.3314 | 20.2185 | 20.1092 | 20.1042 | 20.0292 | 20.1925 | 20.0367 | 6 | 6 | 6 | 6 | 6 | 6 | 6 | 6 |
| MPHOSPH8 | Q99549 | 7.74E-06 | 2.06E-06 | 0.166282 | | 0.929167 | 1.82553 | | 15.1322 | 15.1567 | 15.229 | 14.8875 | 15.2623 | 14.9723 | 14.9218 | 14.9879 | 5 | 7 | 5 | 5 | 6 | 6 | 5 | 6 |
| P4HA2 | O15460-2 | 2.66E-13 | 2.26E-13 | 0.166481 | | 0.315959 | 0.747345 | | 19.7888 | 19.8706 | 19.8003 | 19.1502 | 19.6251 | 20.0217 | 20.0101 | 19.4599 | 29 | 28 | 28 | 27 | 28 | 28 | 27 | 26 |
| SLC25A10 | Q9UBX3;Q | 6.70E-15 | 8.91E-15 | 0.166596 | | 1.03472 | 2.00086 | | 16.0585 | 16.0858 | 16.0829 | 15.8384 | 16.1389 | 15.7784 | 15.8614 | 15.9287 | 11 | 10 | 11 | 10 | 10 | 10 | 11 | 11 |
| LAMA4 | Q16363;Q | 1.90E-14 | 2.22E-14 | 0.166648 | | 0.275113 | 0.665032 | | 15.2398 | 15.1731 | 15.2084 | 14.7375 | 15.3616 | 14.8921 | 14.6162 | 15.5948 | 18 | 17 | 19 | 18 | 15 | 16 | 15 | 19 |
| TNPO3 | Q9Y5L0 | 3.92E-13 | 3.17E-13 | 0.166729 | | 1.08489 | 2.08429 | | 15.9087 | 15.9266 | 15.9202 | 15.8597 | 15.8642 | 15.5444 | 15.7925 | 15.6982 | 23 | 22 | 25 | 22 | 19 | 21 | 21 | 22 |
| CPSF2 | Q9P2I0 | 1.28E-14 | 1.57E-14 | 0.166967 | | 1.16438 | 2.21691 | | 15.9071 | 15.9307 | 15.8778 | 15.8327 | 15.7186 | 15.5463 | 15.7282 | 15.8653 | 19 | 20 | 19 | 17 | 16 | 17 | 17 | 16 |
| MRPL1 | Q9BYD6 | 3.47E-07 | 1.06E-07 | 0.167083 | + | 2.13154 | 3.96764 | | 16.0069 | 15.9222 | 15.9358 | 15.8444 | 15.8597 | 15.7129 | 15.7469 | 15.7754 | 8 | 7 | 7 | 7 | 7 | 7 | 7 | 7 |
| HNRNPLL | Q8WVV9;Q | 8.03E-22 | 9.72E-21 | 0.167142 | + | 2.23861 | 4.1863 | | 15.3893 | 15.4199 | 15.4074 | 15.2791 | 15.1746 | 15.1758 | 15.2358 | 15.3268 | 19 | 21 | 20 | 21 | 17 | 17 | 17 | 16 |
| ILK | Q13418 | 2.47E-08 | 8.54E-09 | 0.167178 | | 0.375928 | 0.863968 | | 18.4675 | 18.4634 | 18.4481 | 18.1509 | 18.0314 | 18.8423 | 18.1197 | 18.3182 | 21 | 21 | 22 | 23 | 20 | 23 | 20 | 23 |
| MPDU1 | O75352;O | 0.00011 | 2.59E-05 | 0.167199 | | 0.596728 | 1.26408 | | 18.756 | 18.574 | 18.5262 | 18.2304 | 18.6818 | 18.2733 | 18.6346 | 18.4374 | 3 | 3 | 3 | 3 | 3 | 3 | 3 | 3 |
| MRPL58 | Q14197 | 5.08E-09 | 1.92E-09 | 0.167343 | + | 1.31462 | 2.46998 | | 15.9238 | 15.976 | 15.9611 | 15.9334 | 15.8229 | 15.7807 | 15.622 | 15.7723 | 5 | 6 | 8 | 7 | 4 | 7 | 6 | 6 |
| SMARCA5 | O60264 | 1.17E-07 | 3.75E-08 | 0.167797 | | 0.567027 | 1.21219 | | 15.6048 | 15.56 | 15.5896 | 15.7788 | 15.2713 | 15.3133 | 15.2102 | 15.5114 | 19 | 21 | 21 | 20 | 18 | 23 | 20 | 20 |
| CPOX | P36551 | 3.85E-15 | 5.56E-15 | 0.167824 | + | 1.45379 | 2.70857 | | 16.586 | 16.6578 | 16.675 | 16.5504 | 16.4033 | 16.3717 | 16.4338 | 16.5998 | 15 | 15 | 15 | 15 | 13 | 15 | 14 | 15 |
| COA7 | Q96BR5 | 4.76E-14 | 4.96E-14 | 0.167926 | + | 1.3326 | 2.50055 | | 15.0104 | 14.9536 | 15.0444 | 14.9072 | 14.9041 | 14.6515 | 14.824 | 14.8875 | 4 | 3 | 3 | 2 | 3 | 3 | 3 | 3 |

| Gene names | UniProt IDs | Protein Group P-value | Protein Group Q-value | Log2 Difference NUP214 vs Controls (n=5) | p<0.05 | -log10 p-value | T-test Test statistic | NPC Localization | Log2 MS2_quantity_ NUP214_1 | Log2 MS2_quantity_ NUP214_2 | Log2 MS2_quantity_ NUP214_3 | Log2 MS2_quantity_ Control_1 | Log2 MS2_quantity_ Control_2 | Log2 MS2_quantity_ Control_3 | Log2 MS2_quantity_ Control_4 | Log2 MS2_quantity_ Control_5 | Number Peptides for Quantitation NUP214_1 | Number Peptides for Quantitation NUP214_2 | Number Peptides for Quantitation NUP214_3 | Number Peptides for Quantitation Control_1 | Number Peptides for Quantitation Control_2 | Number Peptides for Quantitation Control_3 | Number Peptides for Quantitation Control_4 | Number Peptides for Quantitation Control_5 |
|---|---|---|---|---|---|---|---|---|---|---|---|---|---|---|---|---|---|---|---|---|---|---|---|---|
| JMJD8 | Q96S16;Q9 | 0.011057 | 0.001888 | 0.167954 | | 0.257092 | 0.62785 | | 15.4589 | 15.6666 | 15.5269 | 15.5638 | 14.8609 | 15.8586 | 15.6634 | 14.9676 | 3 | 3 | 2 | 1 | 1 | 3 | 4 | 1 |
| TOP2B | Q02880;Q0 | 2.22E-15 | 3.45E-15 | 0.168175 | | 1.2556 | 2.37009 | | 16.2765 | 16.3078 | 16.2863 | 16.1005 | 16.3259 | 16.0232 | 16.0597 | 16.1008 | 51 | 50 | 50 | 49 | 44 | 47 | 47 | 48 |
| ABL1 | P00519;P0 | 0.000691 | 0.000146 | 0.168214 | | 0.546091 | 1.17533 | | 14.8328 | 14.7511 | 14.8465 | 14.7544 | 14.8857 | 14.5337 | 14.2828 | 14.753 | 5 | 6 | 5 | 6 | 4 | 5 | 3 | 3 |
| ZFY;ZFY;ZFY;ZFX;ZFX;Z | P08048;P0 | 0.002385 | 0.000458 | 0.16849 | | 0.251888 | 0.617003 | | 14.1381 | 14.0182 | 13.9683 | 13.6322 | 14.5967 | 13.882 | 13.3812 | 13.8733 | 2 | 2 | 2 | 2 | 1 | 2 | 1 | 2 |
| TMLHE | Q9NVH6;Q | 0.001149 | 0.000235 | 0.168565 | | 0.239331 | 0.590625 | | 14.4016 | 14.3067 | 14.1991 | 14.0198 | 13.5529 | 14.867 | 14.0552 | 14.1746 | 3 | 2 | 4 | 2 | 2 | 4 | 3 | 2 |
| ELP6 | Q0PNE2 | 0.000824 | 0.000172 | 0.168588 | | 0.434109 | 0.973205 | | 14.7811 | 14.7482 | 14.7714 | 14.5611 | 14.74 | 14.8332 | 14.1102 | 14.7472 | 7 | 8 | 7 | 4 | 4 | 4 | 5 | 7 |
| VBP1 | P61758 | 1.31E-09 | 5.33E-10 | 0.168797 | | 0.6531 | 1.36144 | | 17.487 | 17.5163 | 17.4921 | 17.5845 | 17.0782 | 17.1573 | 17.4026 | 17.4258 | 10 | 10 | 10 | 10 | 8 | 10 | 10 | 10 |
| ATRX | P46100 | 7.68E-08 | 2.51E-08 | 0.168863 | | 1.0178 | 1.97275 | | 15.0705 | 15.0718 | 14.9971 | 14.9443 | 15.0639 | 14.684 | 14.8615 | 14.8344 | 12 | 12 | 10 | 7 | 7 | 7 | 8 | 9 |
| TCERG1 | O14776;O1 | 7.36E-13 | 5.50E-13 | 0.16912 | + | 2.43916 | 4.61409 | | 16.353 | 16.4134 | 16.3978 | 16.2156 | 16.2862 | 16.1371 | 16.1984 | 16.2575 | 29 | 34 | 30 | 30 | 27 | 26 | 29 | 24 |
| EXOSC1 | Q9Y3B2 | 9.62E-06 | 2.53E-06 | 0.169258 | | 0.59353 | 1.25851 | | 15.9696 | 15.9495 | 15.9577 | 15.8152 | 16.0158 | 15.5271 | 15.592 | 15.9982 | 6 | 7 | 6 | 7 | 4 | 6 | 5 | 6 |
| BABAM1 | Q9NWV8 | 1.38E-10 | 6.50E-11 | 0.169658 | | 0.484575 | 1.06544 | | 16.007 | 15.9732 | 16.0605 | 15.9226 | 15.4732 | 16.1991 | 15.8818 | 15.7429 | 9 | 8 | 8 | 7 | 8 | 9 | 8 | 8 |
| UBA5 | Q9GZZ9 | 6.76E-08 | 2.22E-08 | 0.170293 | | 1.12733 | 2.15501 | | 17.2406 | 17.2571 | 17.2991 | 17.0308 | 16.9651 | 17.3101 | 17.107 | 17.0635 | 8 | 8 | 8 | 7 | 8 | 7 | 7 | 8 |
| SNRPD1 | P62314 | 3.77E-11 | 1.96E-11 | 0.170439 | + | 1.37258 | 2.56877 | | 18.5892 | 18.6511 | 18.565 | 18.3729 | 18.6129 | 18.3647 | 18.3632 | 18.4428 | 4 | 4 | 4 | 5 | 5 | 4 | 5 | 5 |
| XAB2 | Q9HCS7 | 4.58E-09 | 1.74E-09 | 0.170468 | + | 1.8005 | 3.32878 | | 14.9647 | 14.9379 | 14.9893 | 14.8247 | 14.8577 | 14.6794 | 14.7326 | 14.8733 | 15 | 15 | 16 | 13 | 11 | 10 | 14 | 13 |
| RPA2 | P15927;P1 | 1.02E-10 | 4.86E-11 | 0.170556 | | 0.919705 | 1.8098 | | 16.6823 | 16.803 | 16.8404 | 16.5648 | 16.5334 | 16.4555 | 16.8431 | 16.6266 | 5 | 5 | 5 | 5 | 5 | 5 | 5 | 5 |
| IARS1 | P41252 | 7.16E-11 | 3.52E-11 | 0.170658 | | 0.303393 | 0.722295 | | 18.7584 | 18.7818 | 18.7302 | 18.2591 | 18.3556 | 19.1061 | 18.9176 | 18.2922 | 56 | 57 | 56 | 54 | 56 | 56 | 56 | 56 |
| DARS1 | P14868 | 1.48E-08 | 5.27E-09 | 0.170721 | | 1.05846 | 2.04031 | | 18.8522 | 18.8994 | 18.8027 | 18.5218 | 18.696 | 18.8951 | 18.653 | 18.6377 | 36 | 35 | 36 | 35 | 37 | 35 | 36 | 35 |
| GGCX | P38435 | 9.14E-15 | 1.17E-14 | 0.170867 | | 0.638593 | 1.33651 | | 16.8604 | 16.8005 | 16.7838 | 16.6249 | 16.6671 | 16.3541 | 16.9525 | 16.6215 | 10 | 9 | 9 | 10 | 9 | 9 | 10 | 10 |
| GNAI3 | P08754 | 2.18E-14 | 2.51E-14 | 0.171209 | + | 2.4936 | 4.73462 | | 16.8256 | 16.892 | 16.7631 | 16.701 | 16.69 | 16.6136 | 16.6182 | 16.6557 | 7 | 7 | 7 | 7 | 7 | 7 | 7 | 7 |
| DRAP1 | Q14919;Q1 | 4.00E-06 | 1.09E-06 | 0.171214 | | 0.993008 | 1.93157 | | 17.2566 | 17.3968 | 17.3262 | 17.2382 | 17.2217 | 16.9571 | 17.0642 | 17.2956 | 6 | 6 | 6 | 6 | 3 | 5 | 6 | 6 |
| UBA2 | Q9UBT2 | 4.23E-13 | 3.38E-13 | 0.171243 | + | 1.89316 | 3.50238 | | 17.0183 | 17.0682 | 17.1075 | 17.0001 | 16.8036 | 16.9333 | 16.8535 | 16.8766 | 25 | 24 | 26 | 25 | 26 | 25 | 26 | 23 |
| RBM26 | Q5T8P6;Q5 | 8.80E-10 | 3.67E-10 | 0.17142 | + | 3.65084 | 7.86596 | | 14.7815 | 14.8211 | 14.8175 | 14.6489 | 14.6097 | 14.6064 | 14.6863 | 14.6251 | 18 | 18 | 19 | 16 | 14 | 19 | 16 | 16 |
| MMS19 | Q96T76;Q9 | 2.51E-14 | 2.82E-14 | 0.171424 | + | 3.19507 | 6.48674 | | 16.3345 | 16.3197 | 16.3114 | 16.1842 | 16.1631 | 16.1194 | 16.0919 | 16.1938 | 28 | 29 | 29 | 28 | 27 | 27 | 28 | 28 |
| PITRM1 | Q5JRX3 | 5.07E-19 | 2.48E-18 | 0.171608 | | 0.684361 | 1.41489 | | 16.9126 | 16.9094 | 16.8963 | 16.984 | 16.4867 | 16.695 | 16.613 | 16.8938 | 37 | 36 | 37 | 37 | 35 | 39 | 37 | 38 |
| FAM126A | Q9BYI3;Q9 | 2.34E-11 | 1.27E-11 | 0.171794 | | 0.511268 | 1.11343 | | 15.7088 | 15.7143 | 15.5875 | 15.0753 | 15.6076 | 15.7527 | 15.5368 | 15.5197 | 6 | 7 | 7 | 6 | 4 | 6 | 5 | 6 |
| RDH11 | Q8TC12 | 1.03E-08 | 3.75E-09 | 0.172059 | + | 1.40101 | 2.61751 | | 17.5513 | 17.5362 | 17.5464 | 17.4188 | 17.5014 | 17.3114 | 17.4144 | 17.2168 | 11 | 11 | 11 | 11 | 11 | 11 | 11 | 11 |
| MRPL34 | Q9BQ48 | 0.002287 | 0.000442 | 0.172361 | | 0.23029 | 0.57144 | | 16.0169 | 15.9571 | 16.0455 | 16.1581 | 15.8565 | 14.9557 | 16.08 | 16.1203 | 2 | 2 | 2 | 2 | 2 | 2 | 2 | 2 |
| GTPBP1 | O00178 | 3.58E-11 | 1.87E-11 | 0.172447 | + | 1.74005 | 3.21747 | | 16.5578 | 16.5754 | 16.5593 | 16.3436 | 16.5229 | 16.3321 | 16.4461 | 16.3137 | 12 | 12 | 12 | 12 | 11 | 12 | 12 | 12 |
| YY1 | P25490 | 0.000309 | 6.84E-05 | 0.17255 | | 0.674649 | 1.39832 | | 16.5381 | 16.7524 | 16.3943 | 16.6006 | 16.4561 | 16.1783 | 16.2832 | 16.4272 | 4 | 3 | 2 | 2 | 3 | 3 | 3 | 3 |
| RMND1 | Q9NWS8 | 1.37E-12 | 9.56E-13 | 0.17266 | | 0.563513 | 1.20602 | | 16.1125 | 16.0868 | 16.0922 | 15.5281 | 16.1168 | 16.0434 | 15.8713 | 16.0628 | 5 | 6 | 6 | 4 | 5 | 6 | 5 | 4 |
| MXRA8 | Q9BRK3;Q9 | 3.00E-06 | 8.28E-07 | 0.172681 | | 0.455518 | 1.01259 | | 17.4113 | 17.4594 | 17.41 | 17.6953 | 17.093 | 17.0287 | 17.0647 | 17.3894 | 16 | 16 | 16 | 16 | 14 | 15 | 15 | 16 |
| FBL | P22087 | 4.18E-12 | 2.61E-12 | 0.172774 | + | 1.34945 | 2.52925 | | 17.5644 | 17.5536 | 17.4431 | 17.1639 | 17.3739 | 17.4226 | 17.3929 | 17.3847 | 17 | 18 | 17 | 17 | 15 | 17 | 16 | 16 |
| PPIE | Q9UNP9;Q | 0.000139 | 3.24E-05 | 0.17288 | | 0.828949 | 1.6586 | | 16.0159 | 16.1599 | 16.1902 | 15.989 | 15.6969 | 15.8989 | 16.0438 | 16.117 | 3 | 5 | 5 | 6 | 3 | 5 | 4 | 2 |
| SMAD3 | P84022;P8 | 3.23E-14 | 3.52E-14 | 0.172905 | | 0.650042 | 1.35619 | | 16.8189 | 16.8081 | 16.8717 | 16.8692 | 16.7607 | 16.7272 | 16.3117 | 16.6311 | 9 | 8 | 8 | 8 | 5 | 7 | 7 | 7 |
| EEF1AKNMT | Q8N6R0 | 2.22E-09 | 8.75E-10 | 0.173004 | | 0.679031 | 1.4058 | | 15.3766 | 15.5042 | 15.4092 | 15.3559 | 15.5133 | 15.2867 | 14.9899 | 15.1391 | 17 | 18 | 17 | 14 | 11 | 14 | 15 | 14 |
| NCKIPSD | Q9NZQ3;Q9 | 3.05E-12 | 1.96E-12 | 0.173047 | | 1.22551 | 2.31942 | | 15.4835 | 15.5028 | 15.5185 | 15.1947 | 15.2117 | 15.336 | 15.4697 | 15.4307 | 9 | 9 | 8 | 7 | 8 | 7 | 7 | 6 |
| TFCP2 | Q12800;Q1 | 2.39E-09 | 9.39E-10 | 0.173093 | + | 1.42514 | 2.65904 | | 15.8176 | 15.8716 | 15.7536 | 15.5599 | 15.807 | 15.5694 | 15.661 | 15.6086 | 11 | 10 | 10 | 10 | 7 | 10 | 10 | 8 |
| CLIC1 | O00299 | 1.20E-12 | 8.49E-13 | 0.173162 | | 0.804378 | 1.61749 | | 21.146 | 21.1309 | 21.2224 | 21.0901 | 21.204 | 20.7457 | 20.9031 | 21.0236 | 15 | 15 | 15 | 14 | 15 | 15 | 14 | 15 |
| PSMD14 | O00487 | 4.03E-08 | 1.36E-08 | 0.173206 | | 0.809202 | 1.62557 | | 18.0143 | 18.0452 | 17.9697 | 18.0394 | 17.6797 | 18.0116 | 17.6813 | 17.7707 | 8 | 9 | 8 | 9 | 8 | 8 | 10 | 9 |
| GRHPR | Q9UBQ7 | 2.56E-24 | 7.58E-23 | 0.173251 | | 0.231709 | 0.574462 | | 17.9854 | 18.0533 | 18.023 | 16.9869 | 18.2248 | 18.2087 | 17.9113 | 17.905 | 15 | 14 | 13 | 11 | 13 | 12 | 13 | 13 |
| KHDRBS1 | Q07666 | 5.30E-09 | 2.00E-09 | 0.173257 | | 0.739906 | 1.5091 | | 17.9697 | 17.9544 | 17.9758 | 18.046 | 17.5471 | 17.8746 | 17.6685 | 17.8306 | 12 | 11 | 10 | 11 | 12 | 10 | 10 | 11 |
| NME3 | Q13232 | 1.37E-10 | 6.43E-11 | 0.173293 | | 0.470647 | 1.04019 | | 16.3276 | 16.3205 | 16.3078 | 15.8816 | 16.1107 | 16.0432 | 16.6201 | 16.0711 | 6 | 7 | 6 | 5 | 4 | 4 | 6 | 4 |
| BCAS2 | O75934 | 4.88E-08 | 1.63E-08 | 0.173648 | | 1.14839 | 2.19017 | | 15.7721 | 15.7333 | 15.8672 | 15.729 | 15.7271 | 15.4283 | 15.5866 | 15.6151 | 10 | 10 | 10 | 9 | 7 | 9 | 10 | 10 |
| LRSAM1 | Q6UWE0;Q | 4.88E-16 | 9.01E-16 | 0.173736 | | 0.966148 | 1.88696 | | 15.5029 | 15.5319 | 15.4469 | 15.4349 | 15.0838 | 15.3913 | 15.4357 | 15.2552 | 20 | 20 | 19 | 19 | 12 | 16 | 16 | 17 |
| PRPF4 | O43172;O4 | 7.47E-09 | 2.76E-09 | 0.173838 | + | 1.51395 | 2.8133 | | 16.0081 | 16.0191 | 16 | 15.961 | 15.894 | 15.6838 | 15.8146 | 15.8227 | 19 | 18 | 19 | 18 | 17 | 18 | 18 | 18 |
| ESF1 | Q9H501 | 8.23E-09 | 3.03E-09 | 0.173866 | | 0.238482 | 0.588829 | | 13.9565 | 14.0982 | 14.193 | 14.4074 | 13.3991 | 13.8906 | 14.394 | 13.4523 | 3 | 5 | 3 | 6 | 3 | 2 | 5 | 1 |
| BTAF1 | O14981 | 3.94E-11 | 2.03E-11 | 0.173968 | + | 1.362 | 2.55068 | | 14.7702 | 14.7651 | 14.7919 | 14.5396 | 14.5473 | 14.7853 | 14.636 | 14.5005 | 19 | 20 | 22 | 19 | 8 | 18 | 14 | 11 |
| AHDC1 | Q5TGY3 | 0.007469 | 0.001304 | 0.174059 | | 0.087673 | 0.243629 | | 15.4708 | 15.4249 | 15.2276 | 14.2096 | 16.7333 | 14.2723 | NaN | 15.5863 | 1 | 2 | 1 | 1 | 1 | 2 | NaN | 1 |
| CMTR1 | Q8N1G2 | 2.45E-11 | 1.32E-11 | 0.174451 | | 1.12061 | 2.14381 | | 15.1572 | 15.2289 | 15.1521 | 15.1447 | 14.9221 | 14.8899 | 14.9128 | 15.1555 | 14 | 17 | 19 | 16 | 11 | 16 | 11 | 11 |
| FIG4 | Q92562 | 0.000799 | 0.000167 | 0.174571 | | 0.453952 | 1.00972 | | 15.4951 | 15.2738 | 15.4062 | 15.2649 | 14.78 | 15.2834 | 15.554 | 15.2032 | 6 | 7 | 6 | 4 | 3 | 7 | 5 | 5 |
| APIP | Q96GX9 | 1.25E-09 | 5.13E-10 | 0.174599 | | 0.225438 | 0.561079 | | 16.1502 | 16.1759 | 16.2064 | 16.7228 | 15.635 | 16.3599 | 15.482 | 15.8146 | 8 | 6 | 7 | 7 | 5 | 7 | 5 | 4 |
| KCTD3 | Q9Y597 | 0.004989 | 0.000892 | 0.174661 | | 0.187133 | 0.47746 | | 14.6025 | 14.5169 | 14.5198 | 14.9284 | 13.3623 | 14.7064 | 14.2679 | 14.5935 | 6 | 5 | 5 | 5 | 4 | 4 | 4 | 4 |
| SUGP2 | Q8IX01;Q8 | 5.07E-13 | 3.96E-13 | 0.174778 | + | 1.74857 | 3.23307 | | 15.3972 | 15.4636 | 15.4672 | 15.3479 | 15.1656 | 15.2628 | 15.2024 | 15.3606 | 20 | 20 | 21 | 19 | 16 | 17 | 19 | 18 |
| CCM2 | Q9BSQ5 | 5.56E-09 | 2.09E-09 | 0.174972 | | 1.15704 | 2.20463 | | 14.7727 | 14.7285 | 14.7278 | 14.7576 | 14.5295 | 14.3904 | 14.5918 | 14.5707 | 11 | 10 | 11 | 11 | 11 | 10 | 12 | 9 |
| CTBP1 | Q13363 | 3.23E-06 | 8.91E-07 | 0.175057 | | 1.10235 | 2.11336 | | 17.8008 | 17.7958 | 17.8368 | 17.8408 | 17.6113 | 17.4625 | 17.6748 | 17.5911 | 10 | 10 | 9 | 9 | 9 | 8 | 8 | 9 |
| EARS2 | Q5JPH6;Q5 | 3.79E-11 | 1.97E-11 | 0.175124 | | 0.914493 | 1.80114 | | 15.2814 | 15.382 | 15.2399 | 14.9864 | 15.2103 | 15.3486 | 14.9899 | 15.0945 | 9 | 10 | 8 | 8 | 8 | 8 | 9 | 9 |
| MAF1 | Q9H063 | 2.27E-06 | 6.36E-07 | 0.175132 | | 0.425645 | 0.95752 | | 14.6157 | 14.7738 | 14.8114 | 14.2578 | 15.0073 | 14.3516 | 14.4919 | 14.6839 | 2 | 3 | 3 | 3 | 3 | 3 | 3 | 3 |
| GPD2 | P43304 | 1.18E-11 | 6.77E-12 | 0.175397 | | 0.402822 | 0.91489 | | 17.4968 | 17.5651 | 17.5268 | 17.8231 | 17.4396 | 17.1037 | 17.0086 | 17.3958 | 34 | 35 | 36 | 35 | 33 | 31 | 34 | 33 |
| XPO1 | O14980 | 4.94E-14 | 5.12E-14 | 0.175516 | + | 1.89691 | 3.50949 | | 18.0414 | 18.0477 | 18.0879 | 17.9817 | 17.7663 | 17.9081 | 17.8428 | 17.9186 | 45 | 45 | 45 | 44 | 41 | 44 | 42 | 42 |
| SMPD4 | Q9NXE4;Q9 | 1.66E-12 | 1.13E-12 | 0.175707 | + | 1.55028 | 2.87707 | | 15.4301 | 15.3615 | 15.4111 | 15.3644 | 15.2624 | 15.2066 | 15.092 | 15.2006 | 13 | 12 | 13 | 13 | 9 | 12 | 9 | 13 |
| PLXNB2 | O15031 | 4.21E-13 | 3.37E-13 | 0.175798 | + | 4.17554 | 9.75388 | | 16.7499 | 16.7741 | 16.7553 | 16.5564 | 16.5744 | 16.6283 | 16.5958 | 16.5648 | 50 | 48 | 50 | 47 | 46 | 49 | 50 | 50 |
| PML | P29590 | 5.70E-20 | 3.80E-19 | 0.175804 | | 0.75595 | 1.53616 | | 16.9541 | 16.939 | 16.9628 | 16.7614 | 16.7855 | 17.0686 | 16.5325 | 16.7328 | 31 | 29 | 29 | 28 | 28 | 28 | 28 | 26 |
| XRCC6 | P12956 | 1.27E-16 | 2.92E-16 | 0.175902 | + | 1.84941 | 3.41994 | | 19.237 | 19.2685 | 19.241 | 19.1188 | 19.1395 | 18.9371 | 19.0403 | 19.129 | 36 | 35 | 36 | 35 | 34 | 35 | 36 | 34 |
| JAK1 | P23458 | 9.13E-11 | 4.41E-11 | 0.175982 | | 0.185592 | 0.474023 | | 15.4855 | 15.4818 | 15.4605 | 16.3823 | 15.0219 | 15.0988 | 14.7995 | 15.1974 | 30 | 34 | 33 | 36 | 18 | 30 | 26 | 28 |
| CROT | Q9UKG9 | 0.000424 | 9.22E-05 | 0.176154 | | 0.968037 | 1.8901 | | 15.1631 | 15.1793 | 15.2377 | 15.0202 | 15.1086 | 14.764 | 15.1656 | 15.0275 | 7 | 7 | 6 | 7 | 5 | 6 | 7 | 8 |
| SF3A1 | Q15459 | 1.30E-12 | 9.15E-13 | 0.176326 | + | 2.19064 | 4.08754 | | 16.8378 | 16.8317 | 16.8302 | 16.7224 | 16.6542 | 16.5463 | 16.6407 | 16.721 | 35 | 34 | 33 | 33 | 32 | 34 | 32 | 34 |
| MRPL23 | Q16540 | 3.50E-11 | 1.83E-11 | 0.176506 | | 1.02553 | 1.98558 | | 16.7721 | 16.899 | 16.8832 | 16.7779 | 16.4971 | 16.5695 | 16.695 | 16.8349 | 5 | 5 | 5 | 5 | 5 | 5 | 5 | 5 |
| SNRPA1 | P09661 | 2.35E-07 | 7.37E-08 | 0.177065 | | 1.18277 | 2.2477 | | 18.149 | 18.2608 | 18.2227 | 18.1275 | 18.1316 | 17.8291 | 18.0013 | 18.0792 | 12 | 13 | 12 | 13 | 11 | 13 | 11 | 12 |
| OSBPL3 | Q9H4L5 | 4.49E-20 | 3.17E-19 | 0.177114 | + | 1.89672 | 3.50913 | | 16.0834 | 16.1224 | 16.125 | 16.0267 | 15.9416 | 15.996 | 15.8752 | 15.8261 | 32 | 33 | 32 | 33 | 32 | 31 | 32 | 31 |
| DYRK1A | Q13627;Q1 | 1.18E-13 | 1.10E-13 | 0.177136 | | 1.09845 | 2.10686 | | 15.2054 | 15.0381 | 15.079 | 14.9707 | 14.7918 | 15.1258 | 14.8644 | 14.8993 | 6 | 6 | 5 | 4 | 4 | 4 | 3 | 4 |
| VPS28 | Q9UK41;Q9 | 8.17E-11 | 3.97E-11 | 0.177745 | | 0.208014 | 0.523453 | | 16.3797 | 16.07 | 16.6724 | 16.7119 | 15.6438 | 15.6261 | 16.3673 | 16.6324 | 8 | 8 | 10 | 8 | 8 | 7 | 9 | 10 |
| KHSRP | Q92945 | 4.83E-06 | 1.31E-06 | 0.177867 | + | 1.77961 | 3.29014 | | 18.7506 | 18.7623 | 18.7405 | 18.5374 | 18.7262 | 18.4934 | 18.5337 | 18.5759 | 25 | 26 | 25 | 25 | 22 | 25 | 23 | 24 |
| NCOA7 | Q8NI08;Q8 | 0.000146 | 3.38E-05 | 0.17826 | | 0.180586 | 0.462822 | | 14.177 | 14.3512 | 14.2526 | 14.1414 | 13.9283 | 14.7532 | 14.5041 | 13.0831 | 2 | 4 | 3 | 3 | 2 | 4 | 3 | 1 |
| THOC5 | Q13769 | 2.39E-11 | 1.29E-11 | 0.178479 | | 1.14386 | 2.18261 | | 14.4687 | 14.5742 | 14.5035 | 14.488 | 14.3069 | 14.1711 | 14.4521 | 14.2668 | 7 | 9 | 8 | 9 | 7 | 8 | 7 | 7 |
| NFATC4 | Q14934;Q1 | 1.40E-14 | 1.71E-14 | 0.178565 | | 0.444628 | 0.992607 | | 15.4511 | 15.4159 | 15.4492 | 14.8834 | 15.6972 | 15.3414 | 15.1065 | 15.2723 | 11 | 11 | 10 | 8 | 11 | 9 | 8 | 8 |
| LRCH1 | Q9Y2L9-2 | 0.0001 | 2.36E-05 | 0.17862 | | 0.483704 | 1.06387 | | 15.3452 | 15.2935 | 15.2583 | 14.9144 | 15.3256 | 14.7303 | 15.3075 | 15.3243 | 9 | 8 | 9 | 5 | 5 | 5 | 7 | 5 |
| BST1 | Q10588 | 5.86E-05 | 1.43E-05 | 0.179054 | | 0.220707 | 0.550926 | | 16.3039 | 16.4159 | 16.443 | 16.4784 | 15.4095 | 16.8322 | 15.9708 | 16.352 | 7 | 7 | 7 | 6 | 5 | 8 | 7 | 7 |
| GANAB | Q14697 | 4.12E-10 | 1.79E-10 | 0.179143 | | 0.442197 | 0.988131 | | 20.2775 | 20.3324 | 20.2718 | 20.0648 | 19.6845 | 20.4715 | 20.3266 | 20.0264 | 43 | 41 | 44 | 44 | 40 | 42 | 44 | 40 |

| Gene names | UniProt IDs | Protein Group P-value | Protein Group Q-value | Log2 Difference NUP214 vs Controls (n=5) | p<0.05 | -log10 p-value | T-test Test statistic | NPC Localization | Log2 MS2_quantity_NUP214_1 | Log2 MS2_quantity_NUP214_2 | Log2 MS2_quantity_NUP214_3 | Log2 MS2_quantity_Control_1 | Log2 MS2_quantity_Control_2 | Log2 MS2_quantity_Control_3 | Log2 MS2_quantity_Control_4 | Log2 MS2_quantity_Control_5 | Number Peptides for Quantitation NUP214_1 | Number Peptides for Quantitation NUP214_2 | Number Peptides for Quantitation NUP214_3 | Number Peptides for Quantitation Control_1 | Number Peptides for Quantitation Control_2 | Number Peptides for Quantitation Control_3 | Number Peptides for Quantitation Control_4 | Number Peptides for Quantitation Control_5 |
|---|---|---|---|---|---|---|---|---|---|---|---|---|---|---|---|---|---|---|---|---|---|---|---|---|
| TRIM28 | Q13263 | 1.51E-09 | 6.12E-10 | 0.179203 | + | 1.44466 | 2.69276 | | 18.2853 | 18.2874 | 18.2603 | 18.0779 | 18.2662 | 17.9557 | 18.0819 | 18.1106 | 27 | 25 | 27 | 24 | 24 | 24 | 23 | 25 |
| MAP7D3 | Q8IWC1;Q8 | 1.26E-06 | 3.61E-07 | 0.179303 | | 0.703203 | 1.44695 | | 14.8475 | 14.9434 | 14.7617 | 14.768 | 14.6342 | 14.5753 | 14.4298 | 14.9505 | 8 | 10 | 9 | 8 | 5 | 9 | 6 | 10 |
| GPX4 | P36969;P3 | 1.93E-08 | 6.77E-09 | 0.179715 | | 0.295608 | 0.706657 | | 16.7925 | 16.7798 | 16.7291 | 16.4209 | 16.7797 | 17.1919 | 16.4921 | 16.0525 | 12 | 12 | 12 | 12 | 12 | 12 | 11 | 12 |
| C2orf49 | Q9BVC5;Q9 | 1.24E-05 | 3.22E-06 | 0.179981 | | 0.466933 | 1.03343 | | 14.1384 | 14.1778 | 14.2069 | 14.1684 | 13.7743 | 13.5983 | 14.1634 | 14.2677 | 2 | 2 | 2 | 2 | 1 | 2 | 2 | 2 |
| GGH | Q92820 | 1.24E-10 | 5.87E-11 | 0.180017 | | 0.355314 | 0.824389 | | 16.5951 | 16.5003 | 16.5245 | 16.5223 | 16.8989 | 16.1417 | 16.2772 | 15.9596 | 11 | 11 | 11 | 9 | 9 | 10 | 9 | 9 |
| STK11 | Q15831;Q1 | 0.000518 | 0.000111 | 0.180208 | | 0.770859 | 1.56125 | | 14.9798 | 14.8534 | 14.9014 | 14.7543 | 14.9431 | 14.8557 | 14.4613 | 14.642 | 5 | 6 | 6 | 5 | 4 | 5 | 2 | 3 |
| SARNP | P82979 | 1.59E-07 | 5.05E-08 | 0.181 | | 0.774066 | 1.56664 | | 18.0497 | 18.0567 | 18.0594 | 17.9748 | 18.1254 | 17.7079 | 17.6549 | 17.9084 | 10 | 9 | 10 | 9 | 10 | 9 | 9 | 9 |
| ACAA2 | P42765 | 6.00E-10 | 2.55E-10 | 0.181056 | | 0.722488 | 1.47965 | | 17.7339 | 17.7225 | 17.6915 | 17.659 | 17.3523 | 17.7288 | 17.2775 | 17.6568 | 14 | 14 | 14 | 13 | 13 | 15 | 14 | 14 |
| PAXBP1 | Q9Y5B6 | 0.002781 | 0.000524 | 0.181115 | | 0.952813 | 1.86481 | | 14.6119 | 14.7546 | 14.7278 | 14.7112 | 14.653 | 14.4435 | 14.3852 | 14.392 | 5 | 4 | 3 | 5 | 3 | 3 | 3 | 3 |
| HNRNPA3 | P51991 | 1.39E-09 | 5.65E-10 | 0.181212 | | 1.11896 | 2.14106 | | 19.2867 | 19.3937 | 19.4378 | 19.2465 | 18.9607 | 19.2856 | 19.2332 | 19.2318 | 20 | 20 | 20 | 20 | 19 | 20 | 19 | 19 |
| FAM120A | Q9NZB2;Q9 | 1.55E-18 | 6.35E-18 | 0.181767 | + | 2.17597 | 4.0576 | | 17.2523 | 17.3054 | 17.2631 | 17.0153 | 17.1061 | 17.157 | 17.1635 | 17.0173 | 40 | 41 | 40 | 39 | 38 | 40 | 38 | 39 |
| TAF15 | Q92804;Q9 | 1.11E-07 | 3.59E-08 | 0.181788 | | 0.528362 | 1.14391 | | 18.668 | 18.7343 | 18.7987 | 18.6955 | 18.1703 | 18.8033 | 18.6969 | 18.3935 | 8 | 8 | 7 | 8 | 7 | 8 | 8 | 7 |
| GLUL | P15104 | 1.26E-10 | 5.94E-11 | 0.18191 | | 0.327298 | 0.769755 | | 16.1526 | 16.1723 | 16.2082 | 16.4499 | 15.9165 | 15.6644 | 15.5846 | 16.3635 | 10 | 10 | 9 | 10 | 8 | 8 | 9 | 8 |
| BAZ1B | Q9UIG0;Q9 | 3.18E-10 | 1.40E-10 | 0.18216 | | 1.10229 | 2.11327 | | 15.4937 | 15.4925 | 15.5253 | 15.5162 | 15.3382 | 15.1211 | 15.269 | 15.3639 | 29 | 27 | 30 | 29 | 19 | 21 | 22 | 22 |
| HTATIP2 | Q9BUP3;Q9 | 2.05E-08 | 7.15E-09 | 0.18225 | | 0.238324 | 0.588495 | | 15.6937 | 15.8989 | 15.8785 | 15.3388 | 15.5541 | 16.0802 | 16.2337 | 15.0004 | 8 | 9 | 9 | 6 | 4 | 8 | 9 | 6 |
| NME4 | O00746 | 0.000282 | 6.29E-05 | 0.18241 | | 0.399629 | 0.908884 | | 14.1804 | 14.0572 | 13.966 | 13.4871 | 14.2684 | 13.6341 | 14.1327 | 13.905 | 3 | 4 | 5 | 4 | 4 | 3 | 4 | 4 |
| NPM3 | O75607 | 9.00E-08 | 2.91E-08 | 0.182538 | | 0.983292 | 1.91543 | | 16.5348 | 16.5466 | 16.5381 | 16.4712 | 16.4904 | 16.4594 | 16.1803 | 16.1851 | 2 | 2 | 2 | 2 | 2 | 2 | 2 | 2 |
| HMGN4 | O00479 | 0.009462 | 0.001628 | 0.182843 | | 0.343537 | 0.801547 | | 16.7996 | 16.5001 | 16.2817 | 16.1747 | 16.7697 | 16.4566 | 16.6432 | 15.8772 | 1 | 1 | 2 | 1 | 1 | 1 | 1 | 1 |
| CAND1 | Q86VP6 | 1.06E-13 | 9.98E-14 | 0.182889 | | 0.805394 | 1.61919 | | 18.7257 | 18.7212 | 18.6873 | 18.277 | 18.4654 | 18.7382 | 18.6948 | 18.4671 | 53 | 53 | 54 | 51 | 50 | 52 | 53 | 54 |
| NDUFC2 | O95298 | 9.59E-06 | 2.52E-06 | 0.182938 | | 0.318682 | 0.752744 | | 16.228 | 16.1013 | 16.3932 | 16.4908 | 15.846 | 15.4885 | 16.1856 | 16.2786 | 8 | 8 | 8 | 8 | 6 | 8 | 7 | 8 |
| NADK2 | Q4G0N4 | 7.06E-13 | 5.29E-13 | 0.183062 | | 0.492441 | 1.07964 | | 15.3341 | 15.308 | 15.2278 | 14.8119 | 15.0822 | 15.5177 | 15.23 | 14.8928 | 14 | 15 | 15 | 13 | 11 | 16 | 11 | 13 |
| SNX2 | O60749 | 2.26E-13 | 1.95E-13 | 0.183236 | | 1.27825 | 2.40835 | | 17.4565 | 17.5008 | 17.4715 | 17.1652 | 17.3332 | 17.4376 | 17.3732 | 17.1559 | 25 | 25 | 25 | 24 | 20 | 25 | 25 | 22 |
| AIP | O00170 | 3.38E-16 | 6.56E-16 | 0.183555 | + | 1.5685 | 2.90921 | | 17.4514 | 17.4765 | 17.4975 | 17.1558 | 17.3908 | 17.3703 | 17.3353 | 17.2058 | 14 | 15 | 15 | 14 | 14 | 15 | 14 | 14 |
| DIAPH2 | O60879;O6 | 1.41E-12 | 9.81E-13 | 0.183906 | | 0.560925 | 1.20147 | | 16.1864 | 16.2096 | 16.1404 | 15.7515 | 16.3447 | 16.0822 | 15.7351 | 16.061 | 24 | 25 | 27 | 20 | 15 | 20 | 16 | 21 |
| SCLY | Q96I15 | 0.009409 | 0.001619 | 0.184094 | | 0.163463 | 0.428182 | | 16.0048 | 15.5721 | 15.8393 | 16.0888 | 14.6059 | 16.1067 | 15.6838 | NaN | 3 | 3 | 3 | 3 | 2 | 2 | 2 | NaN |
| GATD3B;GATD3A | A0A0B4J2D | 5.45E-07 | 1.64E-07 | 0.184129 | | 1.20364 | 2.28269 | | 16.6573 | 16.6774 | 16.6614 | 16.5815 | 16.3151 | 16.6486 | 16.3977 | 16.4633 | 13 | 13 | 14 | 11 | 11 | 14 | 11 | 11 |
| STMN1 | P16949 | 4.94E-09 | 1.87E-09 | 0.184152 | | 0.270784 | 0.656151 | | 20.8157 | 20.767 | 20.7305 | 20.7298 | 21.1402 | 19.868 | 20.4499 | 20.7294 | 10 | 9 | 9 | 7 | 10 | 8 | 8 | 10 |
| PXMP4 | Q9Y6I8 | 0.004118 | 0.000746 | 0.184294 | | 0.450467 | 1.00333 | | 16.1845 | 16.1141 | 15.8826 | 15.492 | 16.0217 | 15.648 | 16.1112 | 16.1076 | 4 | 4 | 4 | 3 | 3 | 3 | 4 | 4 |
| BUD31 | P41223 | 1.05E-13 | 9.92E-14 | 0.184619 | + | 4.92169 | 13.1424 | | 16.7279 | 16.7482 | 16.7449 | 16.5289 | 16.5716 | 16.5616 | 16.5364 | 16.5802 | 9 | 9 | 9 | 8 | 9 | 9 | 10 | 8 |
| MRE11 | P49959;P4 | 9.97E-05 | 2.36E-05 | 0.184674 | | 0.915173 | 1.80227 | | 16.4715 | 16.4875 | 16.4949 | 16.0748 | 16.5068 | 16.2268 | 16.2596 | 16.4318 | 16 | 16 | 16 | 16 | 13 | 17 | 17 | 15 |
| SET | Q01105 | 0.000343 | 7.55E-05 | 0.184704 | + | 2.58922 | 4.95114 | | 19.2947 | 19.3103 | 19.3679 | 19.1956 | 19.1124 | 19.1085 | 19.2034 | 19.0782 | 10 | 10 | 11 | 8 | 6 | 9 | 8 | 10 |
| PPP2R1A | P30153 | 2.25E-13 | 1.94E-13 | 0.184821 | | 1.16345 | 2.21535 | | 19.0019 | 18.9547 | 18.9933 | 19.0308 | 18.6885 | 18.754 | 18.705 | 18.8143 | 24 | 24 | 24 | 24 | 24 | 24 | 24 | 24 |
| AGTPBP1 | Q9UPW5;Q | 0.00976 | 0.001677 | 0.184885 | | 0.266896 | 0.648149 | | 13.6443 | 13.2764 | 13.4075 | 13.8258 | 12.8893 | 13.3774 | 12.697 | 13.4999 | 1 | 2 | 1 | 1 | 1 | 1 | 1 | 1 |
| SARAF | Q96BY9 | 1.12E-07 | 3.60E-08 | 0.184962 | | 0.523081 | 1.13452 | | 14.3971 | 14.3051 | 14.2427 | 13.7748 | 14.3791 | 13.9347 | 14.1937 | 14.3678 | 4 | 3 | 4 | 2 | 4 | 2 | 3 | 4 |
| HS2ST1 | Q7LGA3 | 1.36E-07 | 4.34E-08 | 0.185231 | | 0.942298 | 1.84735 | | 16.145 | 15.9262 | 15.8855 | 15.8306 | 15.5962 | 15.8334 | 15.7695 | 15.9721 | 4 | 7 | 5 | 5 | 5 | 5 | 4 | 5 |
| FOSL2 | P15408;P1 | 0.001848 | 0.000365 | 0.185242 | | 0.269694 | 0.661741 | | 13.683 | 13.8298 | 13.8438 | 13.3873 | NaN | 13.0663 | 13.8214 | 14.126 | 2 | 1 | 2 | 1 | NaN | 1 | 1 | 2 |
| CBX1 | P83916 | 5.96E-10 | 2.53E-10 | 0.185343 | | 0.868448 | 1.7245 | | 16.0942 | 16.2737 | 16.2559 | 16.0175 | 16.059 | 15.7593 | 16.2186 | 16.0585 | 5 | 5 | 5 | 6 | 6 | 5 | 4 | 5 |
| USP48 | Q86UV5 | 6.29E-12 | 3.81E-12 | 0.185446 | | 0.811442 | 1.62932 | | 15.0364 | 15.0554 | 15.0637 | 14.7925 | 15.0288 | 14.5804 | 14.887 | 15.0433 | 14 | 14 | 13 | 12 | 11 | 7 | 13 | 10 |
| POLR2K | P53803 | 0.006907 | 0.00121 | 0.185569 | | 0.079663 | 0.222932 | | 13.1507 | 12.5621 | 13.2174 | 12.5564 | 14.6222 | NaN | 11.287 | 12.699 | 1 | 1 | 1 | 1 | 1 | NaN | 1 | 1 |
| PHLDA1 | Q8WV24 | 8.93E-09 | 3.27E-09 | 0.185638 | | 0.209248 | 0.526139 | | 16.6499 | 16.5052 | 16.4355 | 17.0403 | 16.3668 | 15.4917 | 16.6909 | 16.1332 | 2 | 2 | 2 | 2 | 2 | 2 | 2 | 2 |
| KDM1A | O60341;O6 | 1.45E-12 | 1.00E-12 | 0.185742 | | 1.08842 | 2.09016 | | 15.6086 | 15.6639 | 15.5733 | 15.4706 | 15.582 | 15.2173 | 15.3536 | 15.5241 | 19 | 20 | 20 | 17 | 17 | 16 | 18 | 17 |
| GNAS | P63092;P6 | 9.89E-18 | 3.19E-17 | 0.185903 | | 0.917556 | 1.80623 | | 18.2985 | 18.2486 | 18.2577 | 17.8946 | 18.0497 | 18.3033 | 18.2097 | 17.9543 | 16 | 15 | 16 | 15 | 14 | 15 | 15 | 14 |
| TIMM44 | O43615 | 2.89E-15 | 4.32E-15 | 0.185908 | | 1.11321 | 2.13147 | | 17.1425 | 17.1317 | 17.119 | 16.8553 | 16.8073 | 17.1714 | 17.0052 | 16.8865 | 21 | 21 | 22 | 21 | 19 | 19 | 20 | 19 |
| TARDBP | Q13148 | 5.43E-06 | 1.46E-06 | 0.186282 | + | 1.74376 | 3.22426 | | 18.5573 | 18.6895 | 18.6501 | 18.561 | 18.3609 | 18.4316 | 18.3768 | 18.4997 | 8 | 9 | 7 | 8 | 7 | 7 | 8 | 7 |
| HAUS6 | Q7Z4H7 | 0.010023 | 0.001719 | 0.18629 | | 0.270554 | 0.655678 | | 14.5925 | 14.1621 | 14.5707 | 13.8815 | 14.7866 | 13.713 | 14.4517 | 14.4445 | 3 | 4 | 3 | 2 | 3 | 2 | 2 | 2 |
| SNW1 | Q13573 | 2.16E-16 | 4.57E-16 | 0.1866 | + | 2.25002 | 4.21001 | | 16.1986 | 16.1955 | 16.1523 | 16.0191 | 15.9974 | 15.9496 | 15.9112 | 16.1003 | 20 | 20 | 20 | 17 | 18 | 18 | 17 | 19 |
| SAFB | Q15424;Q1 | 8.34E-05 | 1.99E-05 | 0.186777 | | 0.705481 | 1.45082 | | 16.1876 | 16.1428 | 16.0417 | 16.1179 | 16.0568 | 16.0008 | 15.5848 | 15.9259 | 10 | 9 | 10 | 8 | 5 | 7 | 9 | 6 |
| SRSF4 | Q08170 | 7.77E-15 | 1.01E-14 | 0.186795 | | 0.85526 | 1.70252 | | 15.4173 | 15.4151 | 15.3249 | 15.0576 | 15.3006 | 15.3877 | 15.287 | 14.962 | 5 | 4 | 5 | 5 | 4 | 5 | 5 | 4 |
| HNRNPK | P61978;P6 | 3.27E-08 | 1.12E-08 | 0.186827 | + | 1.66311 | 3.0779 | | 20.7257 | 20.654 | 20.6731 | 20.4718 | 20.6053 | 20.4401 | 20.3783 | 20.5919 | 26 | 25 | 27 | 26 | 25 | 25 | 25 | 25 |
| TRIR | Q9BQ61 | 0.001461 | 0.000295 | 0.186842 | | 0.157143 | 0.40949 | | 14.8338 | 14.9519 | 14.9544 | 15.2503 | 15.1999 | 14.4257 | 13.512 | 15.2448 | 4 | 4 | 4 | 5 | 4 | 4 | 2 | 5 |
| MRPS30 | Q9NP92 | 1.48E-21 | 1.64E-20 | 0.186988 | + | 2.75062 | 5.33142 | | 15.5914 | 15.5008 | 15.5097 | 15.3535 | 15.2738 | 15.3351 | 15.3757 | 15.3968 | 9 | 7 | 8 | 9 | 8 | 9 | 8 | 8 |
| P3H4 | Q92791 | 2.63E-16 | 5.35E-16 | 0.186996 | | 0.496519 | 1.08698 | | 17.8704 | 17.8907 | 17.8998 | 17.3399 | 17.6089 | 17.9609 | 18.0278 | 17.5622 | 14 | 14 | 14 | 14 | 13 | 14 | 14 | 13 |
| FSCN1 | Q16658 | 3.21E-09 | 1.25E-09 | 0.187023 | + | 1.51705 | 2.81871 | | 20.3395 | 20.3576 | 20.2875 | 20.2917 | 20.2155 | 20.1004 | 20.0559 | 20.0423 | 29 | 27 | 26 | 29 | 27 | 28 | 26 | 27 |
| PBRM1 | Q86U86;Q8 | 3.02E-06 | 8.34E-07 | 0.187142 | | 1.12524 | 2.15152 | | 14.8755 | 14.8645 | 14.8279 | 14.7965 | 14.6534 | 14.4294 | 14.7015 | 14.7634 | 20 | 23 | 23 | 16 | 14 | 11 | 15 | 14 |
| MARCHF6 | O60337 | 0.008606 | 0.001488 | 0.187402 | | 0.084531 | 0.233506 | | 14.6552 | 14.3579 | 14.7677 | 15.117 | 12.019 | 15.049 | 14.8733 | 14.9728 | 1 | 3 | 1 | 2 | 1 | 2 | 2 | 2 |
| CUL1 | Q13616 | 4.45E-10 | 1.92E-10 | 0.18759 | + | 2.34872 | 4.41811 | | 15.9068 | 15.956 | 15.9386 | 15.8029 | 15.6756 | 15.8344 | 15.6967 | 15.7215 | 33 | 31 | 33 | 30 | 29 | 31 | 29 | 31 |
| IDH3A | P50213 | 1.25E-10 | 5.91E-11 | 0.18795 | | 1.21245 | 2.29747 | | 17.5465 | 17.6595 | 17.594 | 17.5603 | 17.2506 | 17.4941 | 17.3011 | 17.4541 | 13 | 13 | 13 | 13 | 13 | 13 | 13 | 13 |
| SYMPK | Q92797 | 2.98E-11 | 1.58E-11 | 0.188035 | + | 1.76345 | 3.26039 | | 15.324 | 15.3164 | 15.3656 | 15.1774 | 15.1628 | 15.0103 | 15.1157 | 15.2702 | 22 | 26 | 23 | 22 | 19 | 22 | 21 | 21 |
| NOP56 | O00567 | 5.66E-13 | 4.36E-13 | 0.188135 | + | 1.59036 | 2.94791 | | 16.7308 | 16.7114 | 16.7466 | 16.4302 | 16.5876 | 16.699 | 16.5236 | 16.4669 | 30 | 33 | 28 | 26 | 26 | 29 | 29 | 28 |
| ILF3 | Q12906-7 | 4.47E-08 | 1.50E-08 | 0.18851 | + | 1.4388 | 2.68264 | | 19.0333 | 19.0891 | 19.0928 | 19.0062 | 18.6941 | 18.9253 | 18.9089 | 18.8816 | 29 | 28 | 33 | 32 | 29 | 30 | 30 | 30 |
| ACADS | P16219 | 2.51E-05 | 6.32E-06 | 0.188734 | | 0.715814 | 1.46835 | | 15.6613 | 15.58 | 15.6751 | 15.5248 | 15.684 | 15.5149 | 15.1111 | 15.4155 | 6 | 6 | 6 | 6 | 3 | 4 | 2 | 5 |
| METAP2 | P50579 | 7.25E-15 | 9.50E-15 | 0.189105 | | 0.680905 | 1.409 | | 16.3973 | 16.4026 | 16.344 | 15.964 | 15.9856 | 16.3144 | 16.4934 | 16.2035 | 14 | 14 | 15 | 14 | 12 | 14 | 13 | 13 |
| USP39 | Q53GS9 | 3.16E-10 | 1.40E-10 | 0.189196 | + | 2.20825 | 4.12365 | | 16.7458 | 16.6881 | 16.6745 | 16.4934 | 16.6249 | 16.4876 | 16.43 | 16.5322 | 18 | 18 | 16 | 17 | 17 | 16 | 15 | 15 |
| LLGL1 | Q15334 | 7.01E-14 | 6.96E-14 | 0.189416 | | 1.1345 | 2.16698 | | 16.016 | 15.9778 | 15.9748 | 15.814 | 15.9566 | 15.9212 | 15.6151 | 15.6937 | 19 | 20 | 19 | 17 | 18 | 17 | 15 | 17 |
| MCTS1 | Q9ULC4 | 5.98E-08 | 1.98E-08 | 0.189503 | + | 1.58389 | 2.93644 | | 17.7538 | 17.7141 | 17.7909 | 17.5042 | 17.5496 | 17.7472 | 17.496 | 17.5201 | 9 | 10 | 10 | 10 | 9 | 10 | 8 | 10 |
| NEMP1 | O14524 | 0.002412 | 0.000463 | 0.18952 | | 0.441671 | 0.987163 | | 14.5839 | 14.6237 | 14.5558 | 14.1139 | 14.8574 | 14.0878 | 14.553 | 14.3791 | 5 | 6 | 6 | 3 | 3 | 3 | 5 | 5 |
| PAPSS1 | O43252 | 7.14E-11 | 3.51E-11 | 0.189762 | | 0.87756 | 1.73968 | | 17.1948 | 17.2232 | 17.2179 | 17.2084 | 16.7227 | 17.0291 | 17.0347 | 17.116 | 19 | 18 | 18 | 18 | 17 | 20 | 19 | 18 |
| PATL1 | Q86TB9;Q8 | 6.43E-14 | 6.47E-14 | 0.1902 | + | 1.9357 | 3.58338 | | 15.8526 | 15.8273 | 15.737 | 15.6389 | 15.7187 | 15.6388 | 15.5653 | 15.5153 | 10 | 11 | 10 | 10 | 7 | 11 | 9 | 11 |
| ACTA2 | P62736 | 1.67E-08 | 5.90E-09 | 0.19038 | | 0.370033 | 0.852701 | | 22.4843 | 22.4874 | 22.5045 | 21.8782 | 21.9392 | 22.7242 | 22.4323 | 22.5346 | 11 | 11 | 11 | 11 | 11 | 11 | 11 | 11 |
| SERPINH1 | P50454 | 5.87E-12 | 3.57E-12 | 0.190467 | | 0.290944 | 0.697243 | | 22.0342 | 22.0703 | 21.9909 | 21.4281 | 21.723 | 22.5154 | 22.0759 | 21.4642 | 29 | 29 | 28 | 27 | 28 | 28 | 27 | 28 |
| P3H1 | Q32P28 | 6.13E-13 | 4.67E-13 | 0.190476 | | 0.234126 | 0.5796 | | 19.0361 | 19.0441 | 19.0372 | 18.216 | 18.697 | 19.2575 | 19.5628 | 18.5102 | 26 | 26 | 26 | 26 | 27 | 27 | 26 | 26 |
| SMNDC1 | O75940 | 3.47E-14 | 3.74E-14 | 0.190811 | | 0.409691 | 0.927773 | | 15.5108 | 15.3484 | 15.3979 | 15.5757 | 14.6951 | 15.19 | 15.4636 | 15.2169 | 4 | 5 | 5 | 5 | 4 | 5 | 5 | 5 |
| SRGAP2 | O75044 | 5.93E-18 | 2.06E-17 | 0.19101 | | 1.10363 | 2.1155 | | 15.418 | 15.4407 | 15.4131 | 15.1461 | 15.4163 | 15.0344 | 15.2316 | 15.3362 | 25 | 26 | 27 | 23 | 18 | 22 | 20 | 24 |
| GLIPR2 | Q9H4G4 | 2.00E-16 | 4.29E-16 | 0.191377 | | 0.468961 | 1.03712 | | 18.4407 | 18.3891 | 18.3888 | 18.2916 | 18.0263 | 18.7246 | 17.9961 | 18.0355 | 7 | 7 | 7 | 7 | 6 | 7 | 7 | 7 |
| TNFAIP8 | O95379 | 7.59E-07 | 2.24E-07 | 0.191459 | | 0.419215 | 0.945561 | | 14.5057 | 14.3878 | 14.3623 | 13.9366 | 14.616 | 14.5666 | 13.9707 | 14.0458 | 5 | 4 | 4 | 2 | 3 | 3 | 2 | 2 |
| HMGB3 | O15347 | 6.04E-19 | 2.84E-18 | 0.192683 | | 0.614859 | 1.29554 | | 17.1207 | 17.1513 | 17.1453 | 17.0607 | 17.1842 | 16.5322 | 16.9264 | 17.0285 | 9 | 8 | 9 | 8 | 7 | 9 | 7 | 8 |
| MVD | P53602 | 7.74E-13 | 5.74E-13 | 0.192758 | | 0.513388 | 1.11723 | | 16.7834 | 16.7728 | 16.8204 | 17.058 | 16.7083 | 16.4148 | 16.3624 | 16.4537 | 15 | 15 | 14 | 14 | 10 | 11 | 13 | 11 |
| ARL2 | P36404 | 1.72E-06 | 4.86E-07 | 0.192831 | + | 2.10723 | 3.91888 | | 17.6001 | 17.623 | 17.7069 | 17.5414 | 17.512 | 17.4098 | 17.3728 | 17.4163 | 8 | 7 | 8 | 9 | 8 | 7 | 8 | 8 |

| Gene names | UniProt IDs | Protein Group P-value | Protein Group Q-value | Log2 Difference NUP214 vs Controls (n=5) | p<0.05 | -log10 p-value | T-test Test statistic | NPC Localization | Log2 MS2_quantity_NUP214_1 | Log2 MS2_quantity_NUP214_2 | Log2 MS2_quantity_NUP214_3 | Log2 MS2_quantity_Control_1 | Log2 MS2_quantity_Control_2 | Log2 MS2_quantity_Control_3 | Log2 MS2_quantity_Control_4 | Log2 MS2_quantity_Control_5 | Number Peptides for Quantitation NUP214_1 | Number Peptides for Quantitation NUP214_2 | Number Peptides for Quantitation NUP214_3 | Number Peptides for Quantitation Control_1 | Number Peptides for Quantitation Control_2 | Number Peptides for Quantitation Control_3 | Number Peptides for Quantitation Control_4 | Number Peptides for Quantitation Control_5 |
|---|---|---|---|---|---|---|---|---|---|---|---|---|---|---|---|---|---|---|---|---|---|---|---|---|
| PDE6D | O43924 | 6.28E-10 | 2.66E-10 | 0.192849 | | 0.444096 | 0.991628 | | 16.479 | 16.4084 | 16.5272 | 16.7982 | 15.9932 | 16.3107 | 16.0222 | 16.2692 | 3 | 3 | 3 | 3 | 3 | 3 | 3 | 3 |
| COL2A1 | P02458 | 0.000164 | 3.79E-05 | 0.193011 | | 0.116312 | 0.312763 | | 18.3773 | 18.4035 | 18.506 | 17.5775 | 17.3157 | 19.6141 | 19.0694 | 17.6029 | 5 | 5 | 5 | 8 | 6 | 5 | 7 | 7 |
| METAP1 | P53582 | 5.67E-14 | 5.79E-14 | 0.193167 | | 0.721526 | 1.47802 | | 17.0234 | 17.0141 | 16.9834 | 17.0426 | 16.597 | 16.8324 | 16.585 | 17.0119 | 13 | 13 | 13 | 13 | 13 | 13 | 13 | 13 |
| PPM1A | P35813;P3 | 2.19E-22 | 3.09E-21 | 0.193189 | | 0.701563 | 1.44416 | | 15.9564 | 16.0335 | 15.974 | 15.856 | 15.4454 | 16.0549 | 15.8546 | 15.7631 | 11 | 9 | 11 | 9 | 9 | 10 | 10 | 10 |
| STK3 | Q13188;Q1 | 2.39E-08 | 8.30E-09 | 0.193194 | | 0.703875 | 1.44809 | | 15.5988 | 15.5017 | 15.5736 | 15.3329 | 15.0204 | 15.5322 | 15.5826 | 15.3562 | 11 | 12 | 11 | 11 | 9 | 11 | 10 | 11 |
| FERMT2 | Q96AC1;Q9 | 5.54E-20 | 3.76E-19 | 0.193211 | | 0.287394 | 0.690055 | | 18.1254 | 18.1075 | 18.1224 | 17.802 | 17.299 | 18.5591 | 18.1647 | 17.8014 | 34 | 34 | 35 | 33 | 34 | 35 | 33 | 32 |
| TACC1 | O75410;O7 | 3.06E-06 | 8.46E-07 | 0.193254 | + | 1.3019 | 2.44839 | | 15.2644 | 15.2628 | 15.3037 | 15.0165 | 15.0187 | 14.961 | 15.291 | 15.1313 | 21 | 23 | 22 | 17 | 18 | 19 | 18 | 20 |
| ITPR2 | Q14571 | 0.000182 | 4.16E-05 | 0.193581 | | 0.433921 | 0.988732 | | 14.8332 | 14.6795 | 14.9372 | 14.9708 | NaN | 14.2427 | 14.7594 | 14.5192 | 8 | 9 | 8 | 5 | NaN | 3 | 5 | 5 |
| ACADSB | P45954 | 2.35E-09 | 9.25E-10 | 0.193626 | | 0.700668 | 1.44264 | | 15.7464 | 15.6967 | 15.6555 | 15.2255 | 15.8464 | 15.444 | 15.4993 | 15.5145 | 11 | 12 | 12 | 11 | 11 | 10 | 11 | 9 |
| EIF2S1 | P05198 | 2.36E-17 | 6.74E-17 | 0.193843 | | 1.01387 | 1.96621 | | 18.5851 | 18.5735 | 18.5703 | 18.3877 | 18.1634 | 18.6154 | 18.431 | 18.3146 | 25 | 25 | 25 | 26 | 25 | 24 | 25 | 25 |
| EXOSC6 | Q5RKV6 | 0.00081 | 0.000169 | 0.193924 | | 0.400458 | 0.910445 | | 15.7854 | 15.6571 | 15.7729 | 15.821 | 15.6234 | 14.9692 | 15.4778 | 15.8314 | 10 | 9 | 10 | 10 | 9 | 6 | 10 | 9 |
| CPSF6 | Q16630;Q1 | 7.99E-08 | 2.60E-08 | 0.194161 | + | 2.49873 | 4.74607 | | 17.1694 | 17.205 | 17.1874 | 17.0448 | 17.0663 | 16.9949 | 16.8971 | 16.9624 | 12 | 11 | 11 | 12 | 10 | 11 | 11 | 10 |
| NELFA | Q9H3P2 | 3.23E-08 | 1.11E-08 | 0.194246 | | 0.812618 | 1.63129 | | 15.4341 | 15.3857 | 15.4296 | 15.2651 | 15.4815 | 15.0091 | 15.0368 | 15.3183 | 6 | 6 | 8 | 6 | 5 | 5 | 6 | 5 |
| STARD5 | Q9NSY2 | 5.76E-06 | 1.55E-06 | 0.19442 | | 0.272638 | 0.659959 | | 15.7378 | 15.782 | 15.8227 | 14.7952 | 16.0928 | 15.5939 | 15.5641 | 15.886 | 3 | 3 | 3 | 2 | 3 | 3 | 2 | 3 |
| PRAF2 | O60831 | 3.09E-10 | 1.37E-10 | 0.194478 | | 0.406862 | 0.922474 | | 18.7822 | 18.7189 | 18.7018 | 18.3105 | 18.1122 | 18.709 | 19.0218 | 18.5456 | 5 | 5 | 5 | 5 | 5 | 4 | 5 | 5 |
| PPP3CB | P16298;P1 | 0.00033 | 7.28E-05 | 0.194536 | + | 1.494 | 2.77845 | | 16.3499 | 16.4744 | 16.3717 | 16.3443 | 16.2107 | 16.1544 | 16.057 | 16.254 | 4 | 4 | 4 | 4 | 5 | 4 | 6 | 6 |
| POLR2B | P30876 | 2.94E-13 | 2.47E-13 | 0.194682 | + | 2.63779 | 5.06356 | | 15.9249 | 15.9781 | 15.97 | 15.7878 | 15.827 | 15.7675 | 15.662 | 15.7704 | 36 | 37 | 39 | 34 | 31 | 36 | 33 | 34 |
| UBE2L6 | O14933 | 1.07E-07 | 3.46E-08 | 0.194751 | | 0.635305 | 1.33085 | | 16.67 | 16.3353 | 16.6934 | 16.0498 | 16.4006 | 16.5846 | 16.4734 | 16.3489 | 8 | 8 | 6 | 6 | 5 | 6 | 7 | 6 |
| GNB1L | Q9BYB4 | 6.18E-07 | 1.84E-07 | 0.194828 | + | 2.0749 | 3.85449 | | 15.2967 | 15.1802 | 15.1135 | 14.9674 | 15.0398 | 14.9333 | 15.0667 | 15.0027 | 10 | 10 | 9 | 9 | 9 | 10 | 10 | 9 |
| RPRD2 | Q5VT52;Q5 | 2.56E-24 | 7.58E-23 | 0.195137 | + | 2.36909 | 4.4618 | | 14.972 | 14.9154 | 14.9044 | 14.7202 | 14.6927 | 14.6529 | 14.8131 | 14.7986 | 12 | 11 | 11 | 10 | 11 | 9 | 11 | 9 |
| SBF2 | Q86WG5 | 1.47E-05 | 3.78E-06 | 0.195469 | | 0.954999 | 1.86844 | | 15.1245 | 15.1268 | 15.155 | 14.7624 | 14.8547 | 15.0682 | 14.8367 | 15.178 | 11 | 11 | 13 | 7 | 4 | 8 | 8 | 4 |
| VDAC3 | Q9Y277 | 2.95E-10 | 1.31E-10 | 0.195623 | + | 2.07015 | 3.84509 | | 19.0123 | 19.0907 | 19.0658 | 18.8516 | 18.808 | 18.9983 | 18.8474 | 18.798 | 10 | 10 | 10 | 10 | 9 | 10 | 10 | 10 |
| SPG21 | Q9NZD8 | 2.37E-06 | 6.61E-07 | 0.195645 | | 1.2115 | 2.29588 | | 16.3365 | 16.3855 | 16.3635 | 16.132 | 16.1536 | 16.3222 | 16.2679 | 15.9553 | 7 | 6 | 9 | 6 | 5 | 6 | 8 | 6 |
| LAMTOR2 | Q9Y2Q5;Q9 | 2.46E-07 | 7.68E-08 | 0.195738 | | 0.439819 | 0.983749 | | 16.5147 | 16.3963 | 16.4133 | 16.2312 | 16.2914 | 15.7585 | 16.2574 | 16.69 | 5 | 5 | 5 | 4 | 4 | 3 | 5 | 5 |
| GDE1 | Q9NZC3 | 2.28E-14 | 2.61E-14 | 0.195743 | | 0.650872 | 1.35762 | | 15.3177 | 15.1911 | 15.2806 | 14.7507 | 15.1961 | 15.1053 | 15.3613 | 14.9235 | 7 | 5 | 5 | 3 | 2 | 4 | 3 | 4 |
| LYRM7 | Q5U5X0 | 0.000167 | 3.83E-05 | 0.195798 | | 0.152667 | 0.399135 | | 14.9908 | 15.0708 | 15.1662 | 15.6677 | 14.7608 | 13.6475 | 14.7271 | 15.5975 | 6 | 5 | 5 | 4 | 3 | 2 | 4 | 3 |
| NCOA5 | Q9HCD5 | 9.87E-06 | 2.59E-06 | 0.195961 | | 0.837548 | 1.67296 | | 15.6657 | 15.7144 | 15.677 | 15.6091 | 15.6587 | 15.275 | 15.2778 | 15.6283 | 9 | 7 | 9 | 9 | 7 | 9 | 8 | 6 |
| NTPCR | Q9BSD7 | 6.82E-09 | 2.53E-09 | 0.196804 | | 0.404715 | 0.918446 | | 17.0753 | 17.1168 | 17.1158 | 16.7193 | 17.276 | 16.7916 | 17.2763 | 16.4661 | 9 | 10 | 9 | 9 | 9 | 9 | 9 | 8 |
| PCIF1 | Q9H4Z3 | 3.08E-22 | 4.11E-21 | 0.196851 | | 0.978828 | 1.90802 | | 14.5485 | 14.6947 | 14.6761 | 14.436 | 14.6547 | 14.4969 | 14.427 | 14.2 | 8 | 7 | 7 | 5 | 4 | 6 | 6 | 5 |
| DYNLT3 | P51808 | 4.63E-21 | 4.69E-20 | 0.197193 | | 0.577278 | 1.23015 | | 17.0212 | 17.0271 | 16.9851 | 16.7332 | 17.1834 | 16.4423 | 16.8136 | 16.8972 | 4 | 3 | 3 | 3 | 3 | 3 | 4 | 3 |
| SUCLG1 | P53597 | 2.19E-10 | 1.00E-10 | 0.197276 | + | 2.66253 | 5.12149 | | 17.6995 | 17.7913 | 17.6943 | 17.5563 | 17.5716 | 17.5719 | 17.4541 | 17.5015 | 9 | 10 | 9 | 8 | 9 | 9 | 9 | 9 |
| PHF8 | Q9UPP1;Q9 | 0.00276 | 0.00052 | 0.19743 | | 0.715446 | 1.46772 | | 14.3156 | 14.3356 | 14.4082 | 14.2349 | 14.496 | 13.917 | 14.024 | 14.1067 | 2 | 1 | 2 | 1 | 2 | 2 | 2 | 1 |
| TMA16 | Q96EY4 | 0.001731 | 0.000344 | 0.197653 | | 0.300221 | 0.715935 | | 14.8558 | 15.0785 | 14.8355 | 14.603 | 14.0024 | 15.1059 | 15.0809 | 14.8359 | 4 | 4 | 4 | 2 | 2 | 3 | 4 | 3 |
| KLHL42 | Q9P2K6 | 6.05E-14 | 6.12E-14 | 0.197669 | | 1.05086 | 2.02768 | | 16.2906 | 16.1825 | 16.3716 | 15.8267 | 16.1006 | 16.1951 | 16.1778 | 16.1193 | 6 | 6 | 5 | 3 | 4 | 3 | 5 | 4 |
| MBOAT2 | Q6ZWT7 | 2.40E-07 | 7.50E-08 | 0.197721 | | 0.195867 | 0.496823 | | 15.1341 | 15.4149 | 15.3855 | 15.8721 | 14.6757 | 15.3116 | 14.2248 | 15.4848 | 6 | 5 | 6 | 7 | 5 | 6 | 3 | 6 |
| SARM1 | Q6SZW1 | 8.16E-07 | 2.40E-07 | 0.197782 | | 0.460525 | 1.02175 | | 15.2826 | 15.3124 | 15.3586 | 14.98 | 14.8471 | 15.3777 | 15.5485 | 14.8471 | 10 | 12 | 12 | 12 | 4 | 12 | 11 | 9 |
| SRPK2 | P78362;P7 | 6.03E-10 | 2.56E-10 | 0.197821 | + | 1.60914 | 2.98129 | | 15.8699 | 15.7974 | 15.8085 | 15.7158 | 15.7709 | 15.5419 | 15.548 | 15.5606 | 12 | 12 | 12 | 12 | 11 | 12 | 11 | 11 |
| PRRX2 | Q99811 | 0.001017 | 0.000209 | 0.197923 | | 0.133844 | 0.35494 | | 13.4058 | 13.3535 | 13.8306 | 14.1994 | 11.7984 | 13.4492 | 13.3916 | 13.8215 | 2 | 2 | 2 | 2 | 1 | 1 | 1 | 2 |
| STK38L | Q9Y2H1 | 0.000881 | 0.000183 | 0.197945 | | 0.685867 | 1.41746 | | 15.5402 | 15.6503 | 15.6012 | 15.0647 | 15.5539 | 15.6669 | 15.387 | 15.324 | 5 | 5 | 5 | 6 | 4 | 7 | 4 | 4 |
| SRSF1 | Q07955 | 3.83E-15 | 5.54E-15 | 0.198142 | + | 4.03554 | 9.21488 | | 20.106 | 20.1509 | 20.1384 | 19.966 | 19.884 | 19.9217 | 19.9533 | 19.9431 | 15 | 15 | 15 | 16 | 15 | 15 | 16 | 15 |
| DNAJC21 | Q5F1R6;Q5 | 3.67E-14 | 3.95E-14 | 0.198207 | | 1.23585 | 2.33682 | | 14.9731 | 14.9776 | 14.9675 | 14.9881 | 14.6142 | 14.6921 | 14.8234 | 14.7549 | 7 | 7 | 9 | 8 | 5 | 7 | 9 | 7 |
| ANKFY1 | Q9P2R3 | 1.67E-13 | 1.49E-13 | 0.198499 | + | 2.6451 | 5.08063 | | 17.0217 | 17.0683 | 17.0577 | 16.7733 | 16.8389 | 16.8778 | 16.822 | 16.9417 | 32 | 32 | 33 | 33 | 29 | 30 | 31 | 34 |
| LCLAT1 | Q6UWP7;Q | 4.09E-07 | 1.24E-07 | 0.198695 | | 0.942378 | 1.84748 | | 16.5563 | 16.6184 | 16.6633 | 16.1881 | 16.4599 | 16.2858 | 16.5076 | 16.6285 | 8 | 8 | 9 | 6 | 5 | 8 | 7 | 8 |
| EFTUD2 | Q15029 | 3.54E-17 | 9.63E-17 | 0.198828 | + | 3.74376 | 8.17505 | | 18.4345 | 18.4716 | 18.4237 | 18.2771 | 18.2846 | 18.197 | 18.2236 | 18.2399 | 43 | 43 | 41 | 43 | 42 | 41 | 39 | 42 |
| DHTKD1 | Q96HY7 | 8.42E-16 | 1.47E-15 | 0.199231 | + | 1.71127 | 3.16499 | | 15.4086 | 15.3615 | 15.2865 | 14.9911 | 15.2189 | 15.1416 | 15.2233 | 15.19 | 6 | 6 | 6 | 4 | 4 | 3 | 4 | 3 |
| CCDC71L | Q8N9Z2 | 0.009089 | 0.001568 | 0.199318 | | 0.645914 | 1.3491 | | 14.4325 | 14.6593 | 14.7555 | 14.5451 | 14.4385 | 14.0423 | 14.4629 | 14.5936 | 1 | 1 | 1 | 1 | 1 | 1 | 1 | 2 |
| SLC35D1 | Q9NTN3 | 1.68E-08 | 5.95E-09 | 0.199356 | | 0.249082 | 0.611134 | | 14.9529 | 14.6496 | 15.0719 | 15.1717 | 13.8465 | 15.0836 | 14.7214 | 14.6374 | 1 | 1 | 1 | 1 | 1 | 1 | 1 | 1 |
| PRMT3 | O60678 | 0.002128 | 0.000415 | 0.19941 | | 1.13444 | 2.16689 | | 15.0062 | 14.9488 | 14.9201 | 14.7363 | 14.9367 | 14.7039 | 14.8691 | 14.5488 | 7 | 8 | 8 | 6 | 5 | 6 | 6 | 6 |
| PHF3 | Q92576;Q9 | 0.000152 | 3.53E-05 | 0.199469 | + | 1.78781 | 3.30529 | | 14.0515 | 13.9931 | 14.1602 | 13.8721 | 13.8146 | 13.9843 | 13.9005 | 13.7724 | 5 | 4 | 5 | 4 | 2 | 2 | 2 | 4 |
| CCDC90B | Q9GZT6;Q9 | 0.002163 | 0.000421 | 0.199475 | | 0.521699 | 1.13206 | | 14.6616 | 14.6482 | 14.5891 | 14.6045 | 14.0817 | 14.157 | 14.5832 | 14.7409 | 9 | 9 | 8 | 9 | 2 | 4 | 8 | 9 |
| SELENBP1 | Q13228;Q1 | 2.87E-09 | 1.12E-09 | 0.199659 | | 0.206151 | 0.519391 | | 16.5711 | 16.504 | 16.5153 | 15.2715 | 17.0306 | 16.4854 | 16.3908 | 16.4741 | 11 | 11 | 11 | 5 | 8 | 10 | 8 | 9 |
| LGALS3 | P17931 | 4.53E-07 | 1.37E-07 | 0.199766 | | 0.429198 | 0.964112 | | 20.6032 | 20.3519 | 20.66 | 19.953 | 20.7606 | 20.3962 | 20.5092 | 20.0739 | 9 | 9 | 8 | 8 | 9 | 8 | 8 | 8 |
| SRRT | Q9BXP5;Q9 | 7.33E-15 | 9.60E-15 | 0.200149 | + | 4.02618 | 9.17982 | | 16.6235 | 16.6646 | 16.6326 | 16.4932 | 16.4294 | 16.4048 | 16.4264 | 16.4469 | 28 | 30 | 29 | 27 | 24 | 28 | 27 | 29 |
| ARHGEF10 | O15013;O1 | 8.28E-14 | 8.08E-14 | 0.200442 | | 0.941018 | 1.84522 | | 15.6307 | 15.585 | 15.6513 | 15.5897 | 15.3651 | 15.6201 | 15.1899 | 15.3445 | 21 | 21 | 23 | 22 | 18 | 20 | 21 | 19 |
| HDGF | P51858 | 1.90E-08 | 6.68E-09 | 0.200516 | + | 1.30578 | 2.45496 | | 18.1232 | 18.1531 | 18.1474 | 17.8773 | 17.786 | 18.156 | 17.953 | 17.9311 | 17 | 17 | 18 | 18 | 17 | 19 | 17 | 18 |
| PPP2R5C | Q13362;Q1 | 4.76E-05 | 1.17E-05 | 0.200711 | + | 1.43032 | 2.66799 | | 15.7551 | 15.6905 | 15.8227 | 15.6701 | 15.377 | 15.5453 | 15.5327 | 15.652 | 7 | 8 | 7 | 9 | 5 | 5 | 7 | 7 |
| SLC25A32 | Q9H2D1 | 4.68E-07 | 1.41E-07 | 0.200786 | | 0.913975 | 1.80027 | | 14.7746 | 14.8269 | 14.6382 | 14.6129 | 14.2747 | 14.6993 | 14.4759 | 14.6662 | 7 | 6 | 7 | 5 | 5 | 5 | 5 | 6 |
| PPP1R8 | Q12972 | 1.54E-09 | 6.21E-10 | 0.200886 | + | 1.85489 | 3.43022 | | 15.1953 | 15.1974 | 15.2185 | 14.9343 | 15.1085 | 14.9273 | 14.9328 | 15.1114 | 12 | 12 | 14 | 12 | 10 | 13 | 12 | 13 |
| SF3B1 | O75533 | 5.36E-13 | 4.15E-13 | 0.201056 | + | 2.16713 | 4.03963 | | 17.292 | 17.3306 | 17.3224 | 17.2245 | 17.0816 | 17.0192 | 17.0739 | 17.1705 | 52 | 54 | 55 | 53 | 53 | 53 | 54 | 55 |
| HNRNPDL | O14979;O1 | 9.17E-08 | 2.97E-08 | 0.201129 | + | 1.68169 | 3.1114 | | 17.9688 | 18.025 | 17.9793 | 17.7525 | 17.9684 | 17.7624 | 17.6844 | 17.7817 | 11 | 10 | 11 | 9 | 12 | 10 | 11 | 11 |
| SORBS3 | O60504;O6 | 1.07E-08 | 3.89E-09 | 0.201661 | | 0.41998 | 0.946987 | | 17.1716 | 17.3219 | 17.3445 | 17.3688 | 16.6911 | 17.5208 | 16.931 | 16.8766 | 17 | 16 | 17 | 16 | 14 | 16 | 15 | 16 |
| TFRC | P02786 | 1.54E-11 | 8.65E-12 | 0.201903 | | 0.77588 | 1.56969 | | 18.5161 | 18.5409 | 18.5089 | 18.5039 | 18.4007 | 18.5171 | 18.0897 | 18.089 | 31 | 32 | 31 | 32 | 32 | 31 | 31 | 31 |
| CHD4 | Q14839;Q1 | 1.03E-16 | 2.42E-16 | 0.202152 | | 0.819407 | 1.64265 | | 15.865 | 15.9523 | 15.8938 | 16.0007 | 15.609 | 15.5139 | 15.5636 | 15.8205 | 35 | 37 | 37 | 41 | 24 | 35 | 32 | 35 |
| UQCRC1 | P31930 | 4.44E-17 | 1.16E-16 | 0.202169 | + | 2.62039 | 5.02309 | | 17.8468 | 17.8479 | 17.8218 | 17.6125 | 17.6597 | 17.5333 | 17.705 | 17.6728 | 17 | 18 | 18 | 18 | 15 | 18 | 16 | 17 |
| BBS1 | Q8NFJ9;Q8 | 0.000273 | 6.09E-05 | 0.202232 | | 0.167889 | 0.43412 | | 13.9887 | 14.1445 | 14.0654 | 12.7308 | 14.379 | 14.4895 | 14.3544 | 13.366 | 4 | 4 | 4 | 1 | 3 | 3 | 2 | 1 |
| DPYSL3 | Q14195-2 | 4.95E-20 | 3.41E-19 | 0.20238 | | 0.413589 | 0.935064 | | 19.033 | 19.1545 | 19.1124 | 19.0261 | 18.8289 | 19.4531 | 18.6021 | 18.5777 | 33 | 33 | 33 | 33 | 30 | 33 | 28 | 32 |
| PSMG2 | Q969U7;Q9 | 5.48E-15 | 7.46E-15 | 0.203163 | | 0.648032 | 1.35274 | | 15.8578 | 15.9979 | 15.876 | 15.596 | 15.3536 | 15.9667 | 15.9002 | 15.7205 | 6 | 6 | 6 | 6 | 5 | 6 | 6 | 6 |
| RTF1 | Q92541 | 7.54E-11 | 3.69E-11 | 0.203211 | | 0.692874 | 1.42939 | | 14.6839 | 14.7197 | 14.7189 | 14.6423 | 14.1441 | 14.4501 | 14.5073 | 14.7778 | 11 | 13 | 11 | 12 | 6 | 10 | 10 | 11 |
| ZBTB47 | Q9UFB7;Q9 | 0.002241 | 0.000434 | 0.203509 | | 0.423801 | 0.969382 | | NaN | 13.3692 | 13.1521 | 12.6083 | 13.1859 | 13.0207 | 13.1714 | 13.2996 | NaN | 2 | 1 | 1 | 2 | 1 | 1 | 2 |
| UNC119B | A6NIH7 | 2.33E-12 | 1.54E-12 | 0.203588 | | 1.27799 | 2.4079 | | 15.386 | 15.3394 | 15.2967 | 15.3356 | 15.1206 | 15.1201 | 14.9477 | 15.1614 | 7 | 5 | 7 | 6 | 6 | 7 | 7 | 6 |
| NIPBL | Q6KC79;Q6 | 2.14E-09 | 8.43E-10 | 0.203949 | | 0.796306 | 1.60397 | | 14.3033 | 14.2424 | 14.2182 | 13.8361 | 14.0788 | 13.8479 | 14.3299 | 14.1607 | 9 | 11 | 9 | 7 | 6 | 8 | 5 | 7 |
| TUFM | P49411 | 3.58E-12 | 2.27E-12 | 0.204387 | + | 1.73488 | 3.20802 | | 18.5565 | 18.666 | 18.6214 | 18.4864 | 18.2698 | 18.5095 | 18.3503 | 18.4354 | 28 | 28 | 29 | 29 | 27 | 29 | 28 | 28 |
| MRPL35 | Q9NZE8 | 0.00336 | 0.000619 | 0.204399 | | 0.911587 | 1.95269 | | 14.6534 | 14.8281 | 14.7823 | 14.4825 | NaN | 14.7298 | NaN | 14.4384 | 3 | 3 | 3 | 3 | NaN | 2 | NaN | 3 |
| TOP3B | O95985;O9 | 0.001341 | 0.000272 | 0.204508 | | 0.607263 | 1.28238 | | 14.9941 | 14.9604 | 14.9522 | 14.5044 | 14.9534 | 14.9135 | 15.0052 | 14.4456 | 5 | 5 | 5 | 4 | 5 | 5 | 5 | 5 |
| RBM17 | Q96I25 | 6.70E-09 | 2.49E-09 | 0.204705 | | 0.899003 | 1.77538 | | 15.0419 | 15.0147 | 15.1266 | 15.1473 | 14.6556 | 14.7482 | 14.8111 | 14.9197 | 8 | 8 | 7 | 9 | 7 | 8 | 9 | 8 |
| TBC1D8B | Q0IIM8 | 7.97E-12 | 4.73E-12 | 0.205005 | | 0.480098 | 1.05734 | | 15.2873 | 15.2768 | 15.2556 | 14.7961 | 14.937 | 15.4335 | 15.4011 | 14.7735 | 14 | 18 | 14 | 12 | 11 | 15 | 15 | 12 |
| PHC2 | Q8IXK0;Q8 | 6.60E-06 | 1.76E-06 | 0.205239 | | 0.511541 | 1.11392 | | 14.895 | 14.9005 | 14.8853 | 14.9751 | 14.8703 | 14.3612 | 14.3443 | 14.891 | 3 | 4 | 6 | 3 | 1 | 3 | 3 | 3 |
| ATG7 | O95352 | 4.86E-08 | 1.63E-08 | 0.205324 | | 1.15281 | 2.19757 | | 16.586 | 16.5812 | 16.539 | 16.4235 | 16.3769 | 16.5459 | 16.3514 | 16.1193 | 20 | 20 | 19 | 20 | 18 | 20 | 18 | 19 |

| Gene names | UniProt IDs | Protein Group P-value | Protein Group Q-value | Log2 Difference NUP214 vs Controls (n=5) | p<0.05 | -log10 p-value | T-test Test statistic | NPC Localization | Log2 MS2_quantity_NUP214_1 | Log2 MS2_quantity_NUP214_2 | Log2 MS2_quantity_NUP214_3 | Log2 MS2_quantity_Control_1 | Log2 MS2_quantity_Control_2 | Log2 MS2_quantity_Control_3 | Log2 MS2_quantity_Control_4 | Log2 MS2_quantity_Control_5 | Number Peptides for Quantitation NUP214_1 | Number Peptides for Quantitation NUP214_2 | Number Peptides for Quantitation NUP214_3 | Number Peptides for Quantitation Control_1 | Number Peptides for Quantitation Control_2 | Number Peptides for Quantitation Control_3 | Number Peptides for Quantitation Control_4 | Number Peptides for Quantitation Control_5 |
|---|---|---|---|---|---|---|---|---|---|---|---|---|---|---|---|---|---|---|---|---|---|---|---|---|
| IDH3G | P51553;P5 | 2.97E-11 | 1.58E-11 | 0.205527 | + | 2.54714 | 4.85508 | | 16.5753 | 16.5821 | 16.527 | 16.3693 | 16.4415 | 16.309 | 16.2695 | 16.3903 | 12 | 11 | 12 | 12 | 11 | 12 | 12 | 12 |
| ANXA7 | P20073;P2 | 5.06E-11 | 2.57E-11 | 0.205892 | + | 1.50244 | 2.79317 | | 19.1239 | 19.2157 | 19.1639 | 18.9112 | 19.0283 | 19.1046 | 18.7905 | 18.9752 | 19 | 19 | 18 | 19 | 17 | 19 | 19 | 19 |
| AFG3L2 | Q9Y4W6 | 5.00E-14 | 5.18E-14 | 0.206181 | + | 1.58145 | 2.93211 | | 15.8199 | 15.8215 | 15.7967 | 15.4421 | 15.5599 | 15.7486 | 15.6826 | 15.5996 | 25 | 28 | 27 | 25 | 26 | 25 | 23 | 23 |
| SNRNP200 | O75643 | 8.42E-19 | 3.75E-18 | 0.206234 | + | 2.72705 | 5.27467 | | 16.9431 | 16.9999 | 16.999 | 16.8038 | 16.6758 | 16.755 | 16.8278 | 16.8099 | 79 | 80 | 80 | 82 | 73 | 73 | 80 | 76 |
| RIDA | P52758 | 2.73E-10 | 1.22E-10 | 0.206414 | | 0.546673 | 1.17636 | | 17.0551 | 17.0025 | 16.9813 | 17.1067 | 16.3601 | 16.7381 | 16.799 | 17.0288 | 3 | 3 | 4 | 4 | 3 | 3 | 3 | 3 |
| BCKDHA | P12694 | 0.000309 | 6.86E-05 | 0.206416 | | 0.427582 | 0.961116 | | 16.3301 | 16.3963 | 16.3492 | 15.8324 | 15.8513 | 16.6716 | 16.3591 | 16.0463 | 6 | 7 | 6 | 5 | 2 | 5 | 5 | 5 |
| RNASEH2A | O75792 | 4.77E-06 | 1.29E-06 | 0.206571 | + | 1.42493 | 2.65868 | | 16.3623 | 16.2658 | 16.3938 | 16.1713 | 16.318 | 16.005 | 16.1261 | 16.05 | 6 | 6 | 8 | 7 | 7 | 5 | 6 | 6 |
| SCAF1 | Q9H7N4 | 1.18E-12 | 8.41E-13 | 0.206645 | | 0.651728 | 1.35909 | | 14.4635 | 14.6432 | 14.7605 | 14.8106 | 14.2248 | 14.27 | 14.3825 | 14.3907 | 3 | 3 | 5 | 3 | 3 | 4 | 3 | 3 |
| NOC2L | Q9Y3T9 | 4.07E-13 | 3.27E-13 | 0.206824 | + | 2.27186 | 4.25555 | | 16.1232 | 16.0699 | 16.0892 | 15.9243 | 15.824 | 15.966 | 15.783 | 15.939 | 15 | 15 | 15 | 12 | 12 | 13 | 14 | 12 |
| USP7 | Q93009;Q9 | 1.64E-11 | 9.17E-12 | 0.207114 | + | 2.15445 | 4.01389 | | 16.4533 | 16.5185 | 16.5004 | 16.4324 | 16.2432 | 16.2445 | 16.2491 | 16.2489 | 42 | 43 | 43 | 40 | 37 | 41 | 42 | 40 |
| MACROH2A1 | O75367;O7 | 1.90E-09 | 7.56E-10 | 0.207291 | | 1.15479 | 2.20087 | | 19.2346 | 19.2272 | 19.1948 | 18.7509 | 19.1746 | 19.0156 | 19.06 | 19.0568 | 13 | 15 | 13 | 13 | 13 | 13 | 13 | 13 |
| PRKRIP1 | Q9H875 | 0.003196 | 0.000592 | 0.207776 | | 0.383756 | 0.878871 | | 14.3355 | 14.303 | 14.1323 | 14.3563 | 14.2337 | 14.1702 | 13.3725 | 14.113 | 2 | 2 | 2 | 2 | 2 | 2 | 1 | 2 |
| PLBD2 | Q8NHP8 | 1.46E-12 | 1.01E-12 | 0.207809 | | 0.509805 | 1.11082 | | 17.8688 | 17.7816 | 17.8576 | 17.1865 | 17.4863 | 17.6972 | 18.0211 | 17.75 | 15 | 15 | 14 | 15 | 13 | 14 | 14 | 14 |
| TRIP13 | Q15645 | 6.90E-14 | 6.88E-14 | 0.207873 | | 0.565749 | 1.20995 | | 16.6096 | 16.598 | 16.5531 | 16.7674 | 16.3737 | 16.0735 | 16.1364 | 16.5441 | 15 | 15 | 15 | 15 | 14 | 14 | 15 | 15 |
| NT5C3A | Q9H0P0 | 3.86E-08 | 1.31E-08 | 0.208216 | | 0.819289 | 1.64245 | | 15.4955 | 15.6484 | 15.681 | 15.7185 | 15.2306 | 15.4449 | 15.3749 | 15.2315 | 11 | 9 | 11 | 12 | 10 | 9 | 9 | 8 |
| ME1 | P48163 | 1.80E-22 | 2.60E-21 | 0.208372 | | 0.810207 | 1.62725 | | 17.0475 | 16.9729 | 17.0425 | 16.9608 | 16.558 | 17.0413 | 16.8825 | 16.6203 | 14 | 15 | 17 | 14 | 14 | 15 | 13 | 14 |
| SCD5 | Q86SK9;Q8 | 4.50E-07 | 1.36E-07 | 0.208465 | | 0.310301 | 0.736094 | | 16.0261 | 15.927 | 16.2253 | 15.6401 | 16.4267 | 15.2504 | 15.7549 | 16.1829 | 3 | 4 | 2 | 2 | 1 | 1 | 1 | 2 |
| ARHGAP24 | Q8N264 | 3.09E-15 | 4.58E-15 | 0.208826 | | 0.526123 | 1.13993 | | 15.7131 | 15.7868 | 15.8341 | 16.0806 | 15.5184 | 15.565 | 15.3112 | 15.3706 | 12 | 14 | 13 | 13 | 9 | 7 | 7 | 10 |
| INTS7 | Q9NVH2;Q | 2.19E-08 | 7.64E-09 | 0.209425 | | 0.989896 | 1.9264 | | 16.0226 | 15.9163 | 16.0284 | 15.567 | 15.9532 | 15.932 | 15.8232 | 15.623 | 6 | 7 | 6 | 7 | 4 | 7 | 4 | 3 |
| CFL1 | P23528 | 2.01E-08 | 7.01E-09 | 0.209475 | + | 2.668 | 5.13435 | | 22.0979 | 22.1192 | 22.121 | 21.9726 | 21.8755 | 21.7991 | 21.9305 | 21.9383 | 22 | 22 | 22 | 22 | 22 | 22 | 22 | 22 |
| ARHGEF2 | Q92974;Q9 | 4.38E-16 | 8.24E-16 | 0.209543 | | 0.674813 | 1.3986 | | 17.271 | 17.3115 | 17.2894 | 16.8976 | 17.0124 | 17.4326 | 17.2374 | 16.8255 | 40 | 40 | 40 | 38 | 35 | 38 | 40 | 38 |
| INTS11 | Q5TA45;Q5 | 2.41E-07 | 7.53E-08 | 0.209661 | | 0.598823 | 1.26772 | | 15.8791 | 15.849 | 15.8569 | 15.7205 | 16.033 | 15.6694 | 15.2635 | 15.5737 | 6 | 5 | 6 | 6 | 2 | 4 | 5 | 5 |
| PPIL1 | Q9Y3C6 | 1.33E-13 | 1.22E-13 | 0.209773 | | 0.482161 | 1.06108 | | 16.6305 | 16.758 | 16.6683 | 16.8276 | 15.9766 | 16.7091 | 16.429 | 16.4367 | 6 | 6 | 6 | 6 | 6 | 6 | 6 | 6 |
| MRPL33 | O75394 | 0.002108 | 0.000411 | 0.210924 | | 0.613455 | 1.29311 | | 15.6767 | 15.7384 | 15.741 | 15.6047 | 15.6735 | 15.0412 | 15.5064 | 15.713 | 2 | 2 | 2 | 2 | 2 | 2 | 2 | 2 |
| MTIF2 | P46199 | 0.002393 | 0.00046 | 0.211024 | | 0.751196 | 1.52814 | | 15.1277 | 15.1676 | 15.3223 | 15.0895 | 14.6879 | 15.2592 | 15.0663 | 14.8713 | 6 | 7 | 7 | 4 | 5 | 6 | 7 | 5 |
| AACS | Q86V21 | 1.64E-09 | 6.58E-10 | 0.211296 | | 0.95011 | 1.86032 | | 16.2507 | 16.2523 | 16.2113 | 16.2613 | 16.1979 | 15.9459 | 15.8474 | 15.8816 | 12 | 12 | 12 | 13 | 12 | 12 | 11 | 12 |
| EP300 | Q09472 | 0.002734 | 0.000517 | 0.211406 | | 0.425373 | 0.972393 | | 14.4275 | 14.5933 | 14.5855 | 14.1802 | NaN | 14.2625 | 14.8403 | 14.0132 | 2 | 1 | 3 | 1 | NaN | 2 | 2 | 1 |
| TMEM231 | Q9H6L2 | 1.21E-10 | 5.72E-11 | 0.211685 | | 0.217355 | 0.543704 | | 15.4481 | 15.4293 | 15.4485 | 14.2448 | 15.2577 | 15.5411 | 16.0161 | 15.0918 | 2 | 2 | 2 | 3 | 3 | 3 | 2 | 3 |
| RALBP1 | Q15311 | 0.001112 | 0.000228 | 0.211774 | | 0.219352 | 0.553971 | | 16.6644 | 16.6683 | 16.649 | 16.385 | 15.6247 | NaN | 17.1886 | 16.5967 | 3 | 4 | 3 | 3 | 1 | NaN | 2 | 3 |
| BCL2 | P10415;P1 | 0.002515 | 0.00048 | 0.212053 | | 0.263856 | 0.661102 | | 14.5207 | 14.4248 | 14.6104 | NaN | 14.874 | NaN | 13.7808 | 14.265 | 1 | 2 | 1 | NaN | 2 | NaN | 1 | 1 |
| THRAP3 | Q9Y2W1 | 5.17E-14 | 5.35E-14 | 0.212075 | + | 4.72736 | 12.1682 | | 16.9535 | 17.0052 | 16.9671 | 16.766 | 16.7818 | 16.7831 | 16.7289 | 16.7559 | 26 | 26 | 26 | 25 | 24 | 25 | 26 | 26 |
| SLC25A3 | Q00325-2 | 1.19E-05 | 3.10E-06 | 0.212464 | | 0.651665 | 1.35898 | | 20.1171 | 20.0738 | 20.0932 | 19.4232 | 19.942 | 20.0809 | 19.9745 | 19.9905 | 15 | 15 | 15 | 14 | 14 | 15 | 15 | 15 |
| TAOK2 | Q9UL54 | 0.000496 | 0.000107 | 0.212944 | | 0.851813 | 1.69677 | | 15.1439 | 14.8792 | 15.1032 | 14.6655 | 14.7937 | 14.6824 | 15.1186 | 14.8857 | 3 | 6 | 5 | 5 | 5 | 3 | 6 | 4 |
| MORC2 | Q9Y6X9 | 0.001348 | 0.000273 | 0.212946 | | 1.15273 | 2.19743 | | 14.6806 | 14.7643 | 14.8474 | 14.7401 | 14.5676 | 14.3313 | 14.6188 | 14.498 | 7 | 7 | 7 | 6 | 4 | 5 | 6 | 6 |
| PEAK1 | Q9H792 | 2.39E-13 | 2.05E-13 | 0.212998 | | 0.762755 | 1.54761 | | 15.3551 | 15.3959 | 15.4073 | 15.3285 | 15.0887 | 15.4932 | 15.0045 | 14.9507 | 33 | 34 | 35 | 31 | 26 | 33 | 32 | 28 |
| ELAC2 | Q9BQ52 | 2.10E-09 | 8.31E-10 | 0.213026 | | 0.848259 | 1.69084 | | 16.0744 | 16.1853 | 16.1424 | 16.1505 | 16.0628 | 15.7024 | 15.703 | 15.9862 | 21 | 21 | 20 | 20 | 14 | 19 | 18 | 19 |
| NEK9 | Q8TD19 | 4.37E-12 | 2.72E-12 | 0.213489 | | 1.21426 | 2.30051 | | 16.7063 | 16.71 | 16.7186 | 16.3735 | 16.4182 | 16.7631 | 16.5058 | 16.4301 | 30 | 30 | 30 | 30 | 23 | 30 | 29 | 28 |
| SPTBN1 | Q01082 | 1.85E-21 | 1.99E-20 | 0.213502 | | 1.20329 | 2.28209 | | 18.3751 | 18.3898 | 18.3736 | 18.2338 | 18.2317 | 18.2944 | 17.8961 | 18.174 | 142 | 140 | 142 | 137 | 132 | 141 | 133 | 135 |
| ALDH4A1 | P30038 | 9.53E-15 | 1.22E-14 | 0.213503 | + | 1.50082 | 2.79034 | | 16.5906 | 16.6187 | 16.5909 | 16.4527 | 16.5476 | 16.2945 | 16.2256 | 16.4123 | 14 | 13 | 13 | 12 | 11 | 10 | 11 | 13 |
| PRPF38B | Q5VTL8 | 9.71E-05 | 2.30E-05 | 0.213941 | | 0.278843 | 0.672658 | | 15.2832 | 15.6498 | 15.1939 | 15.1886 | 14.6913 | 15.9483 | 14.7434 | 15.2368 | 4 | 3 | 4 | 3 | 3 | 2 | 2 | 2 |
| DDX17 | Q92841 | 1.17E-08 | 4.22E-09 | 0.214151 | + | 1.9072 | 3.52903 | | 19.3131 | 19.333 | 19.3238 | 19.2502 | 19.1302 | 18.9943 | 19.0281 | 19.143 | 33 | 32 | 32 | 31 | 33 | 33 | 31 | 33 |
| HNRNPU | Q00839 | 3.08E-18 | 1.16E-17 | 0.214187 | + | 1.44877 | 2.69987 | | 19.3312 | 19.4004 | 19.319 | 19.1856 | 19.2757 | 19.0218 | 18.9778 | 19.219 | 42 | 38 | 41 | 39 | 39 | 40 | 40 | 39 |
| ZEB1 | P37275;P3 | 0.000168 | 3.86E-05 | 0.214636 | | 0.643934 | 1.3457 | | 14.03 | 14.0924 | 14.3501 | 14.1448 | 14.1359 | 13.5997 | 14.0425 | 13.7914 | 5 | 4 | 4 | 3 | 3 | 2 | 2 | 2 |
| NELFB | Q8WX92-2 | 1.88E-05 | 4.80E-06 | 0.214647 | + | 1.64402 | 3.0436 | | 15.7999 | 15.7264 | 15.784 | 15.5042 | 15.6473 | 15.4957 | 15.427 | 15.7032 | 12 | 11 | 10 | 9 | 9 | 8 | 8 | 8 |
| SRSF5 | Q13243 | 2.40E-05 | 6.05E-06 | 0.214781 | + | 2.28327 | 4.27946 | | 16.5241 | 16.5686 | 16.503 | 16.3661 | 16.4016 | 16.3192 | 16.31 | 16.1887 | 8 | 8 | 8 | 8 | 7 | 7 | 8 | 8 |
| TAF9B | Q9HBM6 | 0.002456 | 0.00047 | 0.215148 | | 0.733775 | 1.49874 | | 14.9468 | 15.1945 | 15.095 | 15.2336 | 14.675 | 14.7005 | 14.8267 | 14.8823 | 3 | 2 | 4 | 2 | 2 | 2 | 1 | 1 |
| PDPR | Q8NCN5 | 2.49E-10 | 1.12E-10 | 0.215416 | + | 1.83451 | 3.39207 | | 16.0202 | 16.0622 | 16.0735 | 15.6842 | 15.968 | 15.798 | 15.8618 | 15.8708 | 21 | 23 | 21 | 16 | 17 | 18 | 19 | 18 |
| TUBGCP4 | Q9UGJ1;Q9 | 4.71E-06 | 1.28E-06 | 0.215858 | + | 1.5386 | 2.85651 | | 15.119 | 15.2314 | 15.2 | 15.0054 | 15.1119 | 14.8917 | 14.8053 | 15.0238 | 11 | 11 | 10 | 9 | 5 | 9 | 8 | 8 |
| MSRB3 | Q8IXL7-2 | 6.87E-15 | 9.09E-15 | 0.216055 | | 1.05531 | 2.03509 | | 16.5007 | 16.5624 | 16.5518 | 16.1947 | 16.3009 | 16.5014 | 16.5012 | 16.113 | 8 | 8 | 9 | 8 | 7 | 9 | 8 | 8 |
| DMAC2L | Q99766 | 0.003861 | 0.000703 | 0.216213 | | 0.91866 | 1.80806 | | 15.0195 | 14.7746 | 15.0272 | 14.6805 | 14.7423 | 14.4753 | 14.9556 | 14.7674 | 4 | 1 | 4 | 4 | 3 | 1 | 4 | 1 |
| ORC4 | O43929 | 0.000312 | 6.90E-05 | 0.216534 | | 0.682396 | 1.41154 | | 14.5611 | 14.2334 | 14.0485 | 14.089 | 14.169 | 13.7867 | 14.0161 | 14.2617 | 1 | 2 | 3 | 3 | 2 | 2 | 1 | 3 |
| FBXO44 | Q9H4M3 | 9.25E-06 | 2.44E-06 | 0.217199 | | 0.378385 | 0.868653 | | 14.5825 | 15.0328 | 14.1631 | 14.2684 | 14.196 | 14.8809 | 14.2985 | 14.2342 | 2 | 1 | 2 | 3 | 1 | 2 | 3 | 2 |
| XPO5 | Q9HAV4 | 4.90E-13 | 3.84E-13 | 0.218 | + | 1.89413 | 3.50422 | | 16.2119 | 16.217 | 16.1753 | 16.0973 | 15.8216 | 16.0027 | 16.0335 | 15.9618 | 26 | 28 | 27 | 24 | 17 | 23 | 20 | 23 |
| PPP4R3A | Q6IN85;Q6 | 1.07E-19 | 6.50E-19 | 0.218051 | | 1.0953 | 2.10163 | | 15.2507 | 15.2591 | 15.3317 | 15.2326 | 14.8493 | 14.9122 | 15.1314 | 15.1867 | 11 | 10 | 9 | 8 | 6 | 8 | 8 | 9 |
| PYCR2 | Q96C36 | 1.66E-16 | 3.68E-16 | 0.218139 | + | 1.64816 | 3.05103 | | 16.8078 | 16.8363 | 16.7737 | 16.5856 | 16.4564 | 16.5485 | 16.7786 | 16.57 | 13 | 13 | 13 | 11 | 13 | 11 | 12 | 12 |
| WAPL | Q7Z5K2;Q7 | 1.31E-09 | 5.33E-10 | 0.219005 | | 0.924304 | 1.81745 | | 14.1836 | 14.3753 | 14.2483 | 14.1897 | 14.0191 | 13.7695 | 14.2593 | 14.0127 | 7 | 7 | 11 | 7 | 8 | 5 | 4 | 6 |
| ZFR | Q96KR1 | 2.56E-24 | 7.58E-23 | 0.219203 | + | 2.474 | 4.691 | | 15.9547 | 15.9761 | 15.9388 | 15.8493 | 15.6396 | 15.7038 | 15.7623 | 15.7317 | 24 | 26 | 28 | 27 | 22 | 23 | 25 | 22 |
| GTF2I | P78347;P7 | 4.61E-14 | 4.82E-14 | 0.219531 | | 0.952034 | 1.86352 | | 16.3642 | 16.3726 | 16.4191 | 16.3018 | 16.2936 | 15.8288 | 16.1616 | 16.2432 | 38 | 39 | 40 | 36 | 31 | 33 | 33 | 35 |
| GPS1 | Q13098-7 | 1.50E-07 | 4.75E-08 | 0.219864 | + | 2.72268 | 5.26421 | | 17.4233 | 17.4549 | 17.4125 | 17.2012 | 17.1136 | 17.3055 | 17.2091 | 17.2225 | 18 | 16 | 18 | 19 | 15 | 16 | 17 | 19 |
| SNRNP40 | Q96DI7 | 1.77E-05 | 4.52E-06 | 0.219972 | + | 2.66958 | 5.13807 | | 16.4081 | 16.4267 | 16.4178 | 16.215 | 16.0814 | 16.271 | 16.232 | 16.1885 | 12 | 13 | 13 | 11 | 11 | 12 | 12 | 11 |
| TSR1 | Q2NL82 | 9.94E-21 | 9.00E-20 | 0.22019 | | 0.629756 | 1.32129 | | 16.3889 | 16.4841 | 16.4807 | 16.4552 | 15.7663 | 16.4047 | 16.2015 | 16.3276 | 17 | 17 | 17 | 16 | 14 | 16 | 17 | 17 |
| MICAL1 | Q8TDZ2;Q8 | 9.70E-14 | 9.29E-14 | 0.220345 | | 1.27788 | 2.40772 | | 17.2948 | 17.3363 | 17.3231 | 17.3251 | 17.069 | 16.9999 | 16.9331 | 17.1617 | 36 | 35 | 33 | 33 | 29 | 30 | 33 | 33 |
| POGLUT2 | Q6UW63 | 1.11E-08 | 4.00E-09 | 0.22052 | | 0.429662 | 0.964972 | | 15.9534 | 15.9404 | 15.8971 | 15.5109 | 15.3219 | 16.0335 | 16.1993 | 15.4832 | 18 | 20 | 20 | 14 | 14 | 18 | 19 | 17 |
| LRBA | P50851;P5 | 6.51E-09 | 2.42E-09 | 0.220625 | | 0.871229 | 1.72914 | | 14.8704 | 14.8001 | 14.9665 | 14.6193 | 14.5295 | 14.8704 | 14.8656 | 14.4071 | 16 | 16 | 18 | 14 | 7 | 16 | 15 | 10 |
| SH3PXD2A | Q5TCZ1;Q5 | 7.18E-17 | 1.77E-16 | 0.220668 | | 0.292827 | 0.701049 | | 15.2019 | 15.1898 | 15.137 | 14.5723 | 14.6206 | 15.7452 | 15.2569 | 14.5829 | 12 | 15 | 15 | 12 | 9 | 14 | 15 | 10 |
| SRP14 | P37108 | 9.22E-07 | 2.69E-07 | 0.220953 | | 1.20188 | 2.27973 | | 18.9824 | 19.0462 | 18.9864 | 18.9686 | 18.8016 | 18.5303 | 18.8489 | 18.7708 | 8 | 8 | 8 | 8 | 8 | 8 | 7 | 7 |
| HNRNPM | P52272;P5 | 8.54E-18 | 2.80E-17 | 0.221368 | + | 2.25237 | 4.21488 | | 19.2575 | 19.2459 | 19.2267 | 19.0787 | 19.0752 | 18.9618 | 18.898 | 19.0963 | 39 | 38 | 38 | 38 | 38 | 39 | 38 | 37 |
| GSN | P06396-2 | 4.96E-11 | 2.52E-11 | 0.221398 | | 0.634842 | 1.33005 | | 20.0108 | 20.0564 | 20.0165 | 19.4615 | 20.2135 | 19.7056 | 19.9122 | 19.7398 | 35 | 34 | 35 | 32 | 35 | 35 | 34 | 34 |
| HSCB | Q8IWL3 | 0.003054 | 0.000568 | 0.2218 | | 0.420759 | 0.948436 | | 14.5093 | 14.1286 | 14.2812 | 14.2979 | 14.3039 | 14.4301 | 13.5814 | 13.8095 | 1 | 2 | 1 | 2 | 1 | 1 | 1 | 1 |
| PDXP | Q96GD0 | 2.74E-11 | 1.46E-11 | 0.222102 | | 0.758559 | 1.54055 | | 15.385 | 15.3016 | 15.1958 | 14.9314 | 14.9787 | 14.8521 | 15.4331 | 15.1648 | 6 | 5 | 5 | 3 | 4 | 5 | 5 | 5 |
| DCAF11 | Q8TEB1;Q8 | 6.03E-05 | 1.47E-05 | 0.222291 | | 0.776689 | 1.57104 | | 14.3797 | 14.1619 | 14.0504 | 13.7107 | 14.2617 | 13.8724 | 14.0559 | 13.9746 | 3 | 3 | 2 | 2 | 2 | 2 | 2 | 1 |
| SIN3A | Q96ST3 | 7.53E-06 | 2.00E-06 | 0.222383 | + | 1.54077 | 2.86032 | | 15.0829 | 15.1661 | 15.2237 | 14.9491 | 14.8607 | 14.7834 | 15.0991 | 14.9836 | 19 | 17 | 18 | 16 | 14 | 14 | 15 | 16 |
| SNAP23 | O00161 | 7.46E-12 | 4.45E-12 | 0.222605 | | 0.889362 | 1.75933 | | 16.9063 | 16.8997 | 17.0559 | 16.8719 | 16.5325 | 16.9454 | 16.4998 | 16.8072 | 7 | 7 | 7 | 9 | 7 | 8 | 8 | 7 |
| TAGLN2 | P37802;P3 | 3.86E-17 | 1.03E-16 | 0.222754 | | 0.569128 | 1.21588 | | 21.2522 | 21.0228 | 21.3454 | 21.292 | 21.0036 | 20.5464 | 20.9174 | 21.1609 | 14 | 14 | 15 | 14 | 13 | 13 | 13 | 14 |
| CETN2 | P41208 | 5.28E-13 | 4.10E-13 | 0.223236 | | 0.555943 | 1.19271 | | 15.5296 | 15.613 | 15.7333 | 15.7312 | 14.916 | 15.5085 | 15.3381 | 15.5165 | 9 | 9 | 7 | 8 | 7 | 8 | 7 | 9 |
| GNPAT | O15228 | 1.27E-10 | 5.99E-11 | 0.223304 | + | 2.58185 | 4.93422 | | 15.3733 | 15.3637 | 15.3423 | 15.1319 | 15.1207 | 15.037 | 15.2473 | 15.1454 | 16 | 18 | 17 | 17 | 14 | 15 | 18 | 17 |
| UTRN | P46939 | 1.13E-21 | 1.30E-20 | 0.223859 | + | 1.34925 | 2.52892 | | 16.5448 | 16.5242 | 16.5508 | 16.1578 | 16.3749 | 16.4245 | 16.4657 | 16.1576 | 131 | 133 | 132 | 132 | 123 | 131 | 133 | 122 |
| EEF2K | O00418 | 3.36E-15 | 4.93E-15 | 0.224082 | + | 1.34472 | 2.52119 | | 14.883 | 14.9105 | 14.9961 | 14.4909 | 14.781 | 14.8758 | 14.6718 | 14.7094 | 13 | 15 | 15 | 11 | 8 | 11 | 12 | 11 |

| Gene names | UniProt IDs | Protein Group P-value | Protein Group Q-value | Log2 Difference NUP214 vs Controls (n=5) | p<0.05 | -log10 p-value | T-test Test statistic | NPC Localization | Log2 MS2_quantity_NUP214_1 | Log2 MS2_quantity_NUP214_2 | Log2 MS2_quantity_NUP214_3 | Log2 MS2_quantity_Control_1 | Log2 MS2_quantity_Control_2 | Log2 MS2_quantity_Control_3 | Log2 MS2_quantity_Control_4 | Log2 MS2_quantity_Control_5 | Number Peptides for Quantitation NUP214_1 | Number Peptides for Quantitation NUP214_2 | Number Peptides for Quantitation NUP214_3 | Number Peptides for Quantitation Control_1 | Number Peptides for Quantitation Control_2 | Number Peptides for Quantitation Control_3 | Number Peptides for Quantitation Control_4 | Number Peptides for Quantitation Control_5 |
|---|---|---|---|---|---|---|---|---|---|---|---|---|---|---|---|---|---|---|---|---|---|---|---|---|
| JTB | O76095;O7 | 1.18E-05 | 3.08E-06 | 0.224255 | | 1.26997 | 2.39435 | | 15.5268 | 15.5829 | 15.5667 | 15.5954 | 15.1817 | 15.3322 | 15.2857 | 15.2778 | 2 | 2 | 2 | 2 | 2 | 2 | 2 | 2 |
| PCNP | Q8WW12 | 5.29E-10 | 2.27E-10 | 0.224331 | | 0.887676 | 1.75653 | | 17.4131 | 17.4472 | 17.3904 | 17.3968 | 17.3477 | 17.0305 | 16.9064 | 17.2815 | 8 | 7 | 8 | 7 | 8 | 7 | 7 | 8 |
| RBBP7 | Q16576 | 1.77E-06 | 5.01E-07 | 0.22457 | | 0.576456 | 1.22871 | | 16.6892 | 16.7413 | 16.7162 | 16.9014 | 16.3259 | 16.3144 | 16.1872 | 16.7262 | 9 | 10 | 9 | 9 | 7 | 8 | 9 | 10 |
| HMBS | P08397;P0 | 9.38E-13 | 6.85E-13 | 0.224691 | | 0.647871 | 1.35246 | | 15.7067 | 15.6648 | 15.6989 | 15.8352 | 15.5453 | 15.4165 | 15.0605 | 15.4696 | 13 | 14 | 14 | 14 | 11 | 14 | 11 | 13 |
| ATP5PO | P48047 | 0.000188 | 4.28E-05 | 0.225072 | | 0.433148 | 0.971427 | | 19.3582 | 19.3922 | 19.3533 | 19.3403 | 19.1854 | 18.4632 | 19.3191 | 19.406 | 10 | 9 | 9 | 9 | 9 | 9 | 9 | 9 |
| CHAMP1 | Q96JM3 | 1.10E-08 | 3.96E-09 | 0.225219 | | 1.03044 | 1.99374 | | 14.9172 | 14.9783 | 14.9832 | 14.7931 | 14.9933 | 14.5266 | 14.5765 | 14.7822 | 10 | 13 | 11 | 8 | 7 | 9 | 7 | 6 |
| COG3 | Q96JB2 | 7.57E-10 | 3.17E-10 | 0.225773 | | 0.690166 | 1.42478 | | 14.6521 | 14.6512 | 14.7391 | 14.5087 | 14.2543 | 14.6911 | 14.706 | 14.1151 | 22 | 24 | 23 | 17 | 17 | 18 | 20 | 14 |
| ANKS3 | Q6ZW76;Q | 0.002316 | 0.000447 | 0.225871 | | 0.546919 | 1.17679 | | 14.4751 | 14.0975 | 14.3314 | 13.94 | 14.3683 | 14.4093 | 13.777 | 13.8828 | 1 | 1 | 2 | 1 | 2 | 1 | 1 | 1 |
| APPL1 | Q9UKG1 | 4.73E-12 | 2.91E-12 | 0.225891 | + | 2.69487 | 5.19788 | | 16.3009 | 16.2769 | 16.2761 | 15.977 | 16.1195 | 16.1387 | 15.9949 | 16.0635 | 20 | 21 | 20 | 18 | 13 | 18 | 18 | 17 |
| GNB1 | P62873 | 9.47E-05 | 2.25E-05 | 0.225916 | + | 3.07863 | 6.16747 | | 19.9575 | 19.9354 | 19.9571 | 19.6457 | 19.807 | 19.7073 | 19.7568 | 19.7038 | 8 | 8 | 8 | 8 | 7 | 8 | 8 | 8 |
| PHF2 | O75151 | 1.92E-16 | 4.15E-16 | 0.226 | | 0.81379 | 1.63325 | | 14.2968 | 14.4691 | 14.1434 | 14.3356 | 14.1374 | 13.8474 | 13.9 | 14.1651 | 4 | 5 | 5 | 3 | 4 | 3 | 3 | 4 |
| PPFIBP1 | Q86W92-2 | 5.68E-12 | 3.46E-12 | 0.226226 | + | 3.15985 | 6.38884 | | 16.7708 | 16.7751 | 16.7754 | 16.6178 | 16.4905 | 16.5212 | 16.5036 | 16.6048 | 42 | 42 | 43 | 36 | 33 | 40 | 39 | 40 |
| RAD50 | Q92878;Q9 | 1.53E-14 | 1.84E-14 | 0.226292 | + | 2.89519 | 5.68895 | | 15.8411 | 15.8926 | 15.8616 | 15.5769 | 15.6834 | 15.6534 | 15.5681 | 15.7124 | 42 | 44 | 44 | 35 | 30 | 33 | 36 | 36 |
| MALT1 | Q9UDY8 | 4.63E-07 | 1.40E-07 | 0.226474 | | 0.632412 | 1.32587 | | 15.5879 | 15.6092 | 15.5674 | 15.014 | 15.6879 | 15.2001 | 15.2841 | 15.6224 | 17 | 16 | 16 | 13 | 13 | 13 | 15 | 16 |
| ELP2 | Q6IA86;Q6 | 4.47E-15 | 6.29E-15 | 0.226939 | | 0.859778 | 1.71005 | | 15.2589 | 15.3246 | 15.237 | 14.7862 | 15.3535 | 14.9138 | 15.1625 | 15.0168 | 13 | 15 | 14 | 12 | 13 | 12 | 12 | 14 |
| YWHAH | Q04917 | 1.95E-13 | 1.72E-13 | 0.227407 | | 0.296114 | 0.707677 | | 18.73 | 18.6326 | 18.6662 | 18.9432 | 18.3036 | 17.5798 | 18.6894 | 18.7283 | 16 | 16 | 16 | 16 | 16 | 15 | 16 | 16 |
| THBS1 | P07996 | 2.78E-13 | 2.34E-13 | 0.227556 | | 0.283351 | 0.681845 | | 19.6778 | 19.7153 | 19.7007 | 19.517 | 18.5509 | 19.8443 | 19.9849 | 19.4548 | 47 | 47 | 48 | 46 | 45 | 47 | 47 | 48 |
| ATAD3B | Q5T9A4 | 0.00086 | 0.000179 | 0.22795 | | 0.242771 | 0.59788 | | 15.7724 | 15.8305 | 15.9598 | 15.7574 | 16.1955 | 15.411 | 14.6382 | 16.1293 | 6 | 6 | 7 | 5 | 5 | 7 | 3 | 7 |
| SAMD9 | Q5K651 | 2.60E-14 | 2.92E-14 | 0.228068 | | 0.360036 | 0.833501 | | 15.3037 | 15.1989 | 15.2318 | 14.423 | 15.07 | 15.5813 | 15.2895 | 14.7199 | 17 | 15 | 17 | 13 | 10 | 16 | 14 | 12 |
| CCDC25 | Q86WR0 | 0.000202 | 4.59E-05 | 0.228087 | + | 1.45178 | 2.70509 | | 15.4212 | 15.4998 | 15.4606 | 15.3915 | 15.011 | 15.2629 | 15.2248 | 15.2721 | 11 | 11 | 11 | 11 | 10 | 10 | 11 | 11 |
| HLA-A | P04439 | 2.15E-12 | 1.43E-12 | 0.228599 | | 0.855458 | 1.70285 | | 19.4597 | 19.6135 | 19.5449 | 19.234 | 19.0861 | 19.6304 | 19.175 | 19.4285 | 10 | 9 | 9 | 15 | 10 | 15 | 9 | 15 |
| SLC9A1 | P19634 | 2.79E-12 | 1.80E-12 | 0.228873 | | 0.465671 | 1.03113 | | 14.987 | 15.1199 | 15.1093 | 14.8929 | 14.2585 | 15.1683 | 15.1369 | 14.7594 | 5 | 5 | 5 | 5 | 2 | 5 | 5 | 4 |
| HNRNPR | O43390 | 3.79E-09 | 1.45E-09 | 0.228905 | + | 2.89893 | 5.69842 | | 18.2831 | 18.3299 | 18.2856 | 17.9898 | 18.0636 | 18.0713 | 18.1707 | 18.0577 | 21 | 23 | 21 | 20 | 17 | 19 | 21 | 19 |
| ECM1 | Q16610;Q1 | 3.55E-11 | 1.86E-11 | 0.22897 | + | 1.45026 | 2.70245 | | 17.1974 | 17.3287 | 17.2838 | 17.1823 | 17.0943 | 17.0031 | 17.0955 | 16.8297 | 16 | 17 | 18 | 14 | 14 | 16 | 16 | 15 |
| TRIM16 | O95361 | 1.68E-11 | 9.37E-12 | 0.229112 | | 1.15344 | 2.19861 | | 15.6818 | 15.7372 | 15.7226 | 15.3399 | 15.6678 | 15.4839 | 15.6468 | 15.2854 | 15 | 15 | 16 | 14 | 15 | 13 | 15 | 13 |
| KRAS | P01116-2 | 5.52E-07 | 1.65E-07 | 0.229314 | + | 2.17166 | 4.04882 | | 16.7442 | 16.8564 | 16.8087 | 16.5464 | 16.5205 | 16.6391 | 16.6858 | 16.4771 | 5 | 5 | 5 | 4 | 4 | 5 | 4 | 4 |
| CARS1 | P49589-3 | 2.12E-10 | 9.70E-11 | 0.229446 | | 0.543255 | 1.17032 | | 18.0482 | 18.0556 | 18.09 | 17.5141 | 17.8164 | 18.2108 | 18.1224 | 17.5121 | 34 | 34 | 33 | 32 | 32 | 34 | 35 | 31 |
| TPMT | P51580 | 3.00E-13 | 2.50E-13 | 0.229733 | | 0.734424 | 1.49984 | | 16.8199 | 16.9787 | 16.8765 | 16.3134 | 16.9914 | 16.7135 | 16.5515 | 16.74 | 11 | 10 | 11 | 10 | 9 | 10 | 10 | 9 |
| RNASEH2B | Q5TBB1;Q5 | 1.38E-09 | 5.61E-10 | 0.229958 | + | 1.33141 | 2.49851 | | 14.3698 | 14.343 | 14.3425 | 14.0664 | 14.1926 | 13.8765 | 14.2448 | 14.2286 | 4 | 4 | 5 | 3 | 3 | 4 | 4 | 3 |
| ABCB6 | Q9NP58;Q9 | 6.66E-10 | 2.81E-10 | 0.230886 | | 0.317962 | 0.751318 | | 17.513 | 17.6134 | 17.5432 | 16.9352 | 16.7455 | 17.8392 | 17.8703 | 17.2382 | 11 | 12 | 10 | 8 | 7 | 12 | 10 | 8 |
| HNRNPA0 | Q13151 | 1.35E-06 | 3.86E-07 | 0.231221 | | 0.927947 | 1.8235 | | 18.9019 | 18.8887 | 18.8441 | 18.3423 | 18.5399 | 18.8454 | 18.8331 | 18.6742 | 9 | 9 | 8 | 8 | 9 | 8 | 8 | 9 |
| TJP2 | Q9UDY2 | 1.87E-12 | 1.26E-12 | 0.231474 | | 0.283426 | 0.681998 | | 15.6505 | 15.6652 | 15.7088 | 16.0865 | 15.6041 | 14.5366 | 15.3784 | 15.6111 | 26 | 25 | 27 | 31 | 22 | 19 | 25 | 26 |
| TNS2 | Q63HR2;Q6 | 8.84E-06 | 2.33E-06 | 0.23163 | | 0.457593 | 1.01639 | | 15.7642 | 15.7053 | 15.6838 | 15.164 | 15.2962 | 16.1423 | 15.413 | 15.4151 | 13 | 12 | 14 | 8 | 7 | 13 | 7 | 8 |
| ARHGEF17 | Q96PE2 | 2.47E-16 | 5.07E-16 | 0.232386 | | 0.623034 | 1.30968 | | 15.8162 | 15.8139 | 15.8355 | 15.0689 | 15.7526 | 15.637 | 15.6896 | 15.7993 | 29 | 29 | 28 | 23 | 24 | 26 | 25 | 26 |
| SCRN2 | Q96FV2;Q9 | 5.60E-11 | 2.82E-11 | 0.232634 | | 0.245247 | 0.60309 | | 15.5123 | 15.7162 | 15.5742 | 15.4329 | 15.98 | 15.9875 | 14.88 | 14.5609 | 7 | 7 | 7 | 7 | 7 | 6 | 6 | 5 |
| CHMP4A | Q9BY43;Q9 | 4.77E-14 | 4.96E-14 | 0.232769 | | 0.29556 | 0.70656 | | 16.6416 | 16.6273 | 16.5953 | 16.717 | 16.5461 | 15.4081 | 16.5946 | 16.6773 | 6 | 5 | 6 | 6 | 5 | 4 | 6 | 6 |
| TPCN1 | Q9ULQ1;Q9 | 0.024115 | 0.003991 | 0.232882 | | 0.605408 | 1.27916 | | 14.2883 | 14.1467 | 14.2828 | 13.8474 | 13.9125 | 14.4485 | 14.149 | 13.6744 | 2 | 2 | 2 | 2 | 1 | 1 | 1 | 1 |
| ELP4 | Q96EB1;Q9 | 4.95E-11 | 2.52E-11 | 0.233065 | | 1.18972 | 2.25933 | | 14.4653 | 14.7014 | 14.7378 | 14.2267 | 14.3253 | 14.4806 | 14.3924 | 14.5838 | 6 | 6 | 6 | 6 | 4 | 6 | 5 | 6 |
| SUPV3L1 | Q8IYB8 | 3.55E-14 | 3.83E-14 | 0.233182 | | 1.12128 | 2.14492 | | 15.9013 | 15.8002 | 15.7567 | 15.5263 | 15.3859 | 15.8652 | 15.5872 | 15.5667 | 14 | 14 | 13 | 11 | 12 | 14 | 12 | 13 |
| SLC7A6 | Q92536 | 0.001973 | 0.000388 | 0.233388 | | 0.586756 | 1.24671 | | 15.2177 | 15.2377 | 15.3776 | 15.2251 | 14.5719 | 15.2904 | 14.8915 | 15.2425 | 1 | 1 | 1 | 2 | 1 | 1 | 1 | 1 |
| PLPBP | O94903 | 1.77E-07 | 5.58E-08 | 0.233579 | | 1.17783 | 2.23942 | | 17.4951 | 17.4902 | 17.4535 | 17.1037 | 17.1478 | 17.4019 | 17.4666 | 17.1101 | 10 | 10 | 10 | 10 | 10 | 10 | 10 | 10 |
| ADRM1 | Q16186 | 1.62E-06 | 4.61E-07 | 0.233633 | | 0.646167 | 1.34954 | | 17.6922 | 17.7106 | 17.6069 | 17.6781 | 16.9736 | 17.6777 | 17.4248 | 17.427 | 6 | 6 | 6 | 6 | 5 | 5 | 6 | 6 |
| SUPT16H | Q9Y5B9 | 1.95E-17 | 5.63E-17 | 0.233655 | + | 1.49293 | 2.77657 | | 17.0213 | 17.0141 | 16.9874 | 16.866 | 16.7005 | 16.6524 | 16.6752 | 16.9756 | 36 | 37 | 37 | 36 | 32 | 37 | 39 | 37 |
| TNRC6B | Q9UPQ9;Q | 2.43E-05 | 6.12E-06 | 0.233817 | | 0.261586 | 0.637174 | | 14.7677 | 14.5837 | 14.8642 | 14.9802 | 13.4856 | 14.7426 | 14.888 | 14.4272 | 6 | 4 | 4 | 7 | 2 | 4 | 3 | 5 |
| PPCS | Q9HAB8 | 1.75E-06 | 4.94E-07 | 0.233869 | | 0.624209 | 1.31171 | | 16.5125 | 16.5544 | 16.5406 | 15.9203 | 16.0805 | 16.495 | 16.6496 | 16.3644 | 12 | 12 | 12 | 10 | 10 | 12 | 12 | 11 |
| STXBP4 | Q6ZWJ1 | 0.00768 | 0.001339 | 0.234054 | | 0.487779 | 1.09045 | | 13.4705 | 13.9254 | 13.6943 | 13.8044 | NaN | 13.2241 | 13.6482 | 13.174 | 2 | 2 | 3 | 2 | NaN | 2 | 2 | 1 |
| CALHM5 | Q8N5C1 | 6.85E-07 | 2.03E-07 | 0.234518 | | 0.350587 | 0.815241 | | 15.2353 | 15.2412 | 15.2642 | 14.2509 | 15.2374 | 15.5072 | 15.1981 | 14.8683 | 5 | 5 | 6 | 3 | 5 | 5 | 5 | 3 |
| KANK2 | Q63ZY3;Q6 | 4.07E-13 | 3.27E-13 | 0.235241 | + | 1.52201 | 2.8274 | | 17.5238 | 17.5194 | 17.4984 | 17.0368 | 17.3817 | 17.3363 | 17.349 | 17.2895 | 29 | 27 | 31 | 27 | 22 | 25 | 27 | 27 |
| AUH | Q13825 | 0.00342 | 0.000629 | 0.235279 | | 0.539826 | 1.16425 | | 15.445 | 15.5019 | 15.5074 | 14.7613 | 15.4785 | 15.4514 | 15.0292 | 15.527 | 4 | 3 | 4 | 1 | 1 | 2 | 3 | 2 |
| PHF5A | Q7RTV0 | 0.000259 | 5.80E-05 | 0.235466 | + | 2.4659 | 4.67304 | | 18.8368 | 18.8194 | 18.7872 | 18.5548 | 18.6665 | 18.5149 | 18.493 | 18.6659 | 5 | 6 | 5 | 6 | 5 | 5 | 6 | 5 |
| DNPEP | Q9ULA0 | 1.39E-10 | 6.53E-11 | 0.235753 | | 1.28983 | 2.42793 | | 16.8426 | 16.8539 | 16.8081 | 16.5296 | 16.7386 | 16.8044 | 16.4446 | 16.4782 | 12 | 12 | 12 | 14 | 12 | 13 | 13 | 13 |
| B3GNT9 | Q6UX72 | 0.002232 | 0.000433 | 0.235852 | | 0.157234 | 0.4097 | | 13.8569 | 14.0839 | 14.2663 | 14.4014 | 13.6958 | 12.2977 | 14.7944 | 13.9768 | 1 | 2 | 1 | 1 | 2 | 1 | 2 | 1 |
| THOC6 | Q86W42 | 1.26E-14 | 1.55E-14 | 0.235893 | | 1.09064 | 2.09386 | | 16.2832 | 16.083 | 16.0123 | 15.9385 | 15.9843 | 15.7749 | 15.6787 | 16.0748 | 8 | 7 | 9 | 7 | 6 | 7 | 7 | 7 |
| WDR19 | Q8NEZ3 | 0.000542 | 0.000116 | 0.235908 | | 0.585947 | 1.2453 | | 13.8812 | 14.0448 | 13.9633 | 13.5084 | 13.9632 | 13.4462 | 14.1532 | 13.5649 | 8 | 8 | 6 | 2 | 3 | 4 | 4 | 3 |
| NELFE | P18615 | 7.09E-10 | 2.98E-10 | 0.236151 | | 1.0074 | 1.95546 | | 15.4625 | 15.385 | 15.4989 | 15.4549 | 15.1099 | 15.3382 | 14.9438 | 15.2162 | 7 | 9 | 9 | 6 | 5 | 6 | 6 | 8 |
| SCAF4 | O95104;O9 | 2.87E-06 | 7.96E-07 | 0.236325 | + | 1.69074 | 3.12775 | | 16.5396 | 16.6446 | 16.5959 | 16.53 | 16.3185 | 16.271 | 16.236 | 16.4295 | 7 | 7 | 7 | 7 | 5 | 7 | 6 | 6 |
| POSTN | Q15063-5 | 5.69E-17 | 1.44E-16 | 0.236348 | | 0.114032 | 0.307201 | | 15.2887 | 15.3472 | 15.3818 | 16.9926 | 13.7473 | 15.2705 | 15.4491 | 14.0549 | 21 | 23 | 23 | 27 | 3 | 19 | 22 | 9 |
| KCNAB2 | Q13303;Q1 | 1.81E-08 | 6.39E-09 | 0.236365 | | 0.324411 | 0.764067 | | 14.6867 | 14.7691 | 14.8291 | 14.8596 | 15.1481 | 14.6235 | 14.0024 | 13.9926 | 4 | 3 | 3 | 4 | 4 | 2 | 4 | 4 |
| PRPF4B | Q13523 | 1.20E-12 | 8.52E-13 | 0.236467 | + | 2.02174 | 3.74983 | | 15.3947 | 15.4576 | 15.4094 | 15.1666 | 15.0507 | 15.2775 | 15.2968 | 15.129 | 13 | 12 | 15 | 11 | 10 | 10 | 12 | 13 |
| AK2 | P54819;P5 | 6.41E-18 | 2.21E-17 | 0.23665 | + | 3.13187 | 6.31191 | | 18.0678 | 18.0143 | 18.0965 | 17.8813 | 17.8252 | 17.8297 | 17.8465 | 17.7317 | 14 | 14 | 14 | 13 | 15 | 14 | 15 | 14 |
| NCBP1 | Q09161 | 9.68E-12 | 5.64E-12 | 0.236749 | + | 1.39368 | 2.60493 | | 16.6537 | 16.6789 | 16.6495 | 16.5315 | 16.3024 | 16.2402 | 16.4429 | 16.6027 | 17 | 20 | 20 | 18 | 17 | 17 | 18 | 18 |
| TIMM23;TIMM23B | O14925;Q5 | 9.35E-11 | 4.50E-11 | 0.236931 | | 0.829137 | 1.65891 | | 15.7211 | 15.4833 | 15.6742 | 15.5137 | 14.9924 | 15.474 | 15.4838 | 15.4825 | 4 | 4 | 4 | 4 | 4 | 4 | 4 | 4 |
| ALYREF | Q86V81 | 1.07E-08 | 3.89E-09 | 0.237634 | + | 2.02474 | 3.75569 | | 18.2597 | 18.2757 | 18.1953 | 18.1533 | 17.8891 | 18.0403 | 18.0105 | 17.9363 | 8 | 8 | 8 | 8 | 7 | 8 | 8 | 8 |
| HMOX1 | P09601 | 5.57E-23 | 1.00E-21 | 0.237855 | | 0.370687 | 0.853953 | | 17.7325 | 17.7161 | 17.7707 | 18.0928 | 17.2787 | 16.9009 | 17.4139 | 17.8232 | 11 | 11 | 12 | 12 | 11 | 13 | 13 | 12 |
| MCM3AP | O60318 | 3.73E-09 | 1.43E-09 | 0.238309 | + | 1.65166 | 3.05731 | | 15.0832 | 14.9841 | 15.0679 | 14.9305 | 14.8971 | 14.8099 | 14.6099 | 14.7864 | 9 | 8 | 8 | 7 | 6 | 8 | 6 | 6 |
| DECR1 | Q16698;Q1 | 6.22E-09 | 2.32E-09 | 0.238396 | + | 2.63101 | 5.04777 | | 18.2471 | 18.2767 | 18.2338 | 17.9996 | 18.1035 | 18.086 | 17.9398 | 17.9417 | 17 | 17 | 17 | 17 | 15 | 16 | 17 | 17 |
| VPS37B | Q9H9H4 | 0.001062 | 0.000218 | 0.238516 | | 0.218555 | 0.546292 | | 15.4236 | 14.9733 | 15.7146 | 15.7941 | 15.3569 | 14.5342 | 14.2883 | 15.6864 | 2 | 2 | 4 | 4 | 1 | 1 | 1 | 2 |
| PA2G4 | Q9UQ80 | 6.24E-16 | 1.12E-15 | 0.23883 | + | 2.72739 | 5.27548 | | 18.5139 | 18.5529 | 18.5214 | 18.3837 | 18.209 | 18.3299 | 18.2203 | 18.31 | 21 | 22 | 22 | 21 | 21 | 21 | 21 | 22 |
| HMGCL | P35914 | 5.31E-08 | 1.77E-08 | 0.238909 | + | 1.75057 | 3.23674 | | 17.5196 | 17.4837 | 17.4939 | 17.0877 | 17.3296 | 17.4155 | 17.2173 | 17.2507 | 10 | 10 | 9 | 10 | 9 | 10 | 9 | 10 |
| ARPIN | Q7Z6K5;Q7 | 2.03E-05 | 5.17E-06 | 0.239071 | + | 1.6146 | 2.99101 | | 16.7871 | 16.7157 | 16.7651 | 16.3682 | 16.6036 | 16.5625 | 16.3884 | 16.6618 | 7 | 9 | 8 | 8 | 8 | 8 | 7 | 8 |
| UROD | P06132 | 8.21E-06 | 2.18E-06 | 0.239268 | + | 1.9389 | 3.5895 | | 18.4005 | 18.4745 | 18.47 | 18.2619 | 18.3554 | 18.1354 | 18.0792 | 18.2135 | 9 | 9 | 9 | 9 | 9 | 9 | 9 | 9 |
| KYAT3 | Q6YP21;Q6 | 2.52E-08 | 8.73E-09 | 0.23938 | | 0.413362 | 0.93464 | | 16.7491 | 16.7635 | 16.7155 | 15.8666 | 16.975 | 16.8141 | 16.4015 | 16.4594 | 15 | 15 | 15 | 14 | 15 | 15 | 15 | 14 |
| FDXR | P22570;P2 | 2.31E-18 | 9.07E-18 | 0.239439 | | 0.834577 | 1.668 | | 17.2342 | 17.2751 | 17.2458 | 16.7969 | 17.0466 | 17.3399 | 17.1196 | 16.7583 | 19 | 19 | 19 | 17 | 17 | 18 | 18 | 18 |
| RAB29 | O14966 | 1.44E-05 | 3.71E-06 | 0.239529 | + | 2.61854 | 5.01881 | | 18.3091 | 18.2871 | 18.2885 | 17.9663 | 18.1134 | 18.1343 | 18.089 | 17.9738 | 8 | 9 | 9 | 6 | 7 | 9 | 8 | 6 |
| NAE1 | Q13564;Q1 | 1.52E-13 | 1.37E-13 | 0.23956 | + | 2.27655 | 4.26537 | | 17.0214 | 16.9931 | 17.0234 | 16.682 | 16.7242 | 16.8959 | 16.8487 | 16.7145 | 16 | 16 | 16 | 15 | 15 | 16 | 16 | 15 |
| ADNP | Q9H2P0 | 3.65E-09 | 1.41E-09 | 0.239949 | | 1.15068 | 2.194 | | 14.6855 | 14.6906 | 14.6293 | 14.5399 | 14.6302 | 14.1589 | 14.3541 | 14.4592 | 11 | 15 | 16 | 15 | 9 | 9 | 12 | 12 |
| HIP1 | O00291 | 2.27E-12 | 1.50E-12 | 0.240357 | | 0.639422 | 1.33794 | | 16.3382 | 16.3807 | 16.3928 | 16.4808 | 16.3569 | 15.9136 | 15.756 | 16.1439 | 29 | 29 | 26 | 29 | 27 | 23 | 23 | 25 |
| TMEM205 | Q6UW68 | 0.000228 | 5.15E-05 | 0.240506 | | 0.623011 | 1.30964 | | 16.5984 | 16.641 | 16.6915 | 15.9941 | 16.3163 | 16.2877 | 16.7161 | 16.7013 | 3 | 3 | 3 | 3 | 3 | 3 | 3 | 3 |

| Gene names | UniProt IDs | Protein Group P-value | Protein Group Q-value | Log2 Difference NUP214 vs Controls (n=5) | p<0.05 | -log10 p-value | T-test Test statistic | NPC Localization | Log2 MS2_quantity_ NUP214_1 | Log2 MS2_quantity_ NUP214_2 | Log2 MS2_quantity_ NUP214_3 | Log2 MS2_quantity_ Control_1 | Log2 MS2_quantity_ Control_2 | Log2 MS2_quantity_ Control_3 | Log2 MS2_quantity_ Control_4 | Log2 MS2_quantity_ Control_5 | Number Peptides for Quantitation NUP214_1 | Number Peptides for Quantitation NUP214_2 | Number Peptides for Quantitation NUP214_3 | Number Peptides for Quantitation Control_1 | Number Peptides for Quantitation Control_2 | Number Peptides for Quantitation Control_3 | Number Peptides for Quantitation Control_4 | Number Peptides for Quantitation Control_5 |
|---|---|---|---|---|---|---|---|---|---|---|---|---|---|---|---|---|---|---|---|---|---|---|---|---|
| HP1BP3 | Q5SSJ5 | 6.27E-08 | 2.07E-08 | 0.241059 | | 1.18843 | 2.25717 | | 18.0765 | 18.0892 | 18.0661 | 17.5607 | 17.9002 | 17.9919 | 17.9696 | 17.7586 | 22 | 22 | 22 | 20 | 21 | 22 | 22 | 22 |
| MAX | P61244;P6 | 1.29E-06 | 3.71E-07 | 0.241225 | | 0.486161 | 1.06831 | | 15.5077 | 15.4664 | 15.2797 | 15.4571 | 14.6193 | 15.5327 | 15.0255 | 15.249 | 3 | 3 | 2 | 3 | 2 | 3 | 2 | 3 |
| PTCD3 | Q96EY7 | 1.97E-09 | 7.80E-10 | 0.24138 | + | 2.08973 | 3.88396 | | 15.5626 | 15.6147 | 15.613 | 15.4452 | 15.3121 | 15.4065 | 15.1961 | 15.417 | 18 | 18 | 17 | 16 | 14 | 16 | 15 | 14 |
| FMNL3 | Q8IVF7 | 7.62E-08 | 2.49E-08 | 0.241681 | | 0.829958 | 1.66028 | | 15.9263 | 15.9046 | 15.7975 | 15.9148 | 15.4925 | 15.7237 | 15.3013 | 15.7401 | 13 | 17 | 15 | 13 | 10 | 9 | 12 | 13 |
| NGLY1 | Q96IV0 | 9.02E-06 | 2.38E-06 | 0.241783 | + | 2.09538 | 3.89521 | | 14.9831 | 14.9433 | 15.0318 | 14.8476 | 14.6296 | 14.8356 | 14.6593 | 14.7494 | 8 | 9 | 6 | 7 | 5 | 6 | 5 | 5 |
| NARS2 | Q96I59 | 0.003203 | 0.000593 | 0.242033 | | 0.725734 | 1.48514 | | 15.1694 | 15.212 | 15.3063 | 14.7426 | 15.1125 | 15.3116 | 14.6759 | 15.0934 | 6 | 6 | 5 | 4 | 4 | 4 | 4 | 3 |
| UBE2O | Q9C0C9 | 5.45E-08 | 1.81E-08 | 0.24218 | | 1.0742 | 2.0665 | | 15.7149 | 15.717 | 15.6878 | 15.7592 | 15.4899 | 15.3678 | 15.2259 | 15.4791 | 27 | 26 | 26 | 26 | 22 | 26 | 26 | 26 |
| NEK1 | Q96PY6-3;( | 3.41E-09 | 1.32E-09 | 0.242724 | | 0.658323 | 1.37039 | | 14.3195 | 14.2071 | 14.3857 | 13.747 | 13.8489 | 14.4817 | 14.1864 | 14.0427 | 6 | 5 | 6 | 5 | 4 | 6 | 6 | 4 |
| SF3A3 | Q12874 | 2.88E-11 | 1.53E-11 | 0.24292 | + | 3.19161 | 6.47705 | | 17.5771 | 17.574 | 17.5303 | 17.4071 | 17.319 | 17.2599 | 17.3367 | 17.2651 | 17 | 17 | 17 | 17 | 17 | 17 | 17 | 17 |
| PRKAR1A | P10644 | 1.41E-10 | 6.63E-11 | 0.242943 | | 1.12932 | 2.15834 | | 18.5291 | 18.5711 | 18.562 | 18.5237 | 18.383 | 18.2691 | 18.0166 | 18.3631 | 19 | 19 | 19 | 19 | 19 | 19 | 19 | 19 |
| UBXN7 | O94888 | 1.30E-10 | 6.11E-11 | 0.243016 | + | 1.72603 | 3.19187 | | 16.295 | 16.1641 | 16.2042 | 15.8654 | 16.0632 | 15.8821 | 15.9411 | 16.1384 | 11 | 11 | 13 | 12 | 12 | 12 | 11 | 12 |
| EML4 | Q9HC35 | 4.64E-17 | 1.20E-16 | 0.243154 | | 0.683094 | 1.41273 | | 16.2532 | 16.288 | 16.2763 | 16.4307 | 16.0502 | 16.0813 | 15.6249 | 15.9596 | 27 | 28 | 27 | 26 | 23 | 25 | 24 | 25 |
| SGPP1 | Q9BX95 | 2.12E-07 | 6.65E-08 | 0.24339 | | 0.462799 | 1.0259 | | 15.4165 | 15.3067 | 15.3336 | 15.4603 | 15.2162 | 15.4718 | 14.5922 | 14.8039 | 5 | 4 | 5 | 6 | 3 | 3 | 4 | 4 |
| CPNE3 | O75131 | 1.88E-09 | 7.47E-10 | 0.243471 | | 0.758086 | 1.53975 | | 18.3027 | 18.3079 | 18.2991 | 17.9043 | 18.4594 | 17.8418 | 17.8892 | 18.2039 | 20 | 20 | 18 | 18 | 19 | 18 | 18 | 16 |
| NXF1 | Q9UBU9 | 1.74E-09 | 6.95E-10 | 0.243535 | + | 2.44353 | 4.62369 | | 16.2508 | 16.3371 | 16.3221 | 16.0902 | 16.0303 | 15.9314 | 16.1064 | 16.1406 | 15 | 16 | 15 | 16 | 13 | 16 | 13 | 14 |
| GNPNAT1 | Q96EK6 | 3.94E-10 | 1.72E-10 | 0.243967 | | 1.01937 | 1.97535 | | 17.8841 | 17.896 | 17.8918 | 17.508 | 17.3613 | 17.8462 | 17.8131 | 17.7046 | 8 | 8 | 9 | 8 | 8 | 8 | 9 | 8 |
| CSTF1 | Q05048 | 1.69E-15 | 2.72E-15 | 0.244226 | + | 1.93146 | 3.57527 | | 16.3075 | 16.325 | 16.2964 | 16.075 | 16.1185 | 16.0079 | 15.9115 | 16.2143 | 13 | 13 | 13 | 13 | 12 | 13 | 13 | 13 |
| TARS1 | P26639 | 6.62E-08 | 2.17E-08 | 0.24474 | | 0.58721 | 1.2475 | | 18.8638 | 18.9188 | 18.8555 | 18.526 | 18.3175 | 19.0401 | 18.9219 | 18.3676 | 44 | 44 | 44 | 43 | 43 | 44 | 45 | 43 |
| ALKBH5 | Q6P6C2;Q6 | 1.23E-07 | 3.93E-08 | 0.244749 | + | 2.23451 | 4.17782 | | 14.754 | 14.8046 | 14.8376 | 14.4324 | 14.6923 | 14.5388 | 14.5748 | 14.5318 | 8 | 7 | 9 | 8 | 8 | 8 | 9 | 8 |
| TOMM20 | Q15388 | 0.003205 | 0.000593 | 0.245117 | | 1.10988 | 2.12591 | | 17.5787 | 17.4095 | 17.5585 | 17.315 | 17.0506 | 17.2896 | 17.5351 | 17.162 | 2 | 2 | 2 | 1 | 2 | 2 | 2 | 2 |
| SF3B2 | Q13435 | 5.01E-17 | 1.29E-16 | 0.245422 | + | 2.71033 | 5.23469 | | 17.3316 | 17.3486 | 17.3751 | 17.2179 | 17.0982 | 17.022 | 17.0523 | 17.1413 | 30 | 32 | 32 | 33 | 27 | 31 | 33 | 31 |
| UBE2V2 | Q15819 | 1.26E-10 | 5.95E-11 | 0.245696 | | 0.602072 | 1.27337 | | 18.5826 | 18.0154 | 18.0549 | 17.7978 | 18.1243 | 18.2578 | 17.6847 | 17.9952 | 3 | 3 | 2 | 3 | 3 | 3 | 3 | 3 |
| CERT1 | Q9Y5P4;Q9 | 3.06E-11 | 1.62E-11 | 0.245903 | + | 1.34908 | 2.52862 | | 15.3705 | 15.4569 | 15.3636 | 15.4133 | 15.0641 | 15.0639 | 15.1883 | 15.0258 | 16 | 15 | 16 | 16 | 12 | 15 | 13 | 14 |
| GNB2 | P62879 | 2.22E-06 | 6.22E-07 | 0.246582 | + | 2.52223 | 4.79879 | | 20.2061 | 20.2219 | 20.1742 | 19.9447 | 20.0404 | 20.0214 | 19.8269 | 19.9371 | 12 | 14 | 12 | 14 | 12 | 12 | 13 | 13 |
| UACA | Q9BZF9 | 4.47E-20 | 3.17E-19 | 0.246818 | | 0.790842 | 1.5948 | | 16.6664 | 16.7379 | 16.6887 | 16.102 | 16.614 | 16.7024 | 16.5807 | 16.2552 | 68 | 69 | 69 | 63 | 66 | 66 | 65 | 62 |
| SGF29 | Q96ES7 | 6.72E-11 | 3.33E-11 | 0.247029 | | 0.753757 | 1.53246 | | 13.9591 | 13.3269 | 13.3914 | 13.2018 | 13.4971 | 13.3019 | 13.2505 | 13.3092 | 1 | 1 | 1 | 1 | 2 | 1 | 1 | 1 |
| GTF3C1 | Q12789;Q1 | 1.27E-07 | 4.06E-08 | 0.247257 | | 1.21007 | 2.29347 | | 14.9353 | 15.0166 | 14.9564 | 14.7829 | 14.8655 | 14.4472 | 14.6462 | 14.869 | 13 | 14 | 12 | 11 | 6 | 7 | 6 | 11 |
| DVL3 | Q92997;Q9 | 2.35E-06 | 6.56E-07 | 0.247492 | | 0.82701 | 1.65536 | | 15.3111 | 15.1927 | 15.3755 | 15.1768 | 15.0217 | 15.3495 | 14.9806 | 14.6994 | 3 | 3 | 3 | 4 | 4 | 4 | 4 | 3 |
| MRPS17 | Q9Y2R5 | 5.36E-10 | 2.30E-10 | 0.248272 | | 1.12853 | 2.15702 | | 16.7561 | 16.7995 | 16.7209 | 16.7007 | 16.4373 | 16.2246 | 16.6596 | 16.5307 | 4 | 4 | 4 | 4 | 4 | 4 | 4 | 4 |
| OMA1 | Q96E52 | 0.020596 | 0.003426 | 0.248356 | | 0.216495 | 0.541849 | | 13.6942 | 14.7028 | 14.6553 | 14.687 | 14.5616 | 14.308 | 13.8903 | 13.0652 | 1 | 2 | 2 | 1 | 1 | 1 | 1 | 1 |
| ITGAV | P06756;P0 | 4.38E-13 | 3.49E-13 | 0.24836 | | 0.443766 | 0.991021 | | 17.7057 | 17.7683 | 17.7155 | 17.404 | 17.2346 | 18.222 | 17.2391 | 17.3077 | 37 | 38 | 39 | 39 | 34 | 41 | 37 | 37 |
| URB2 | Q14146 | 0.000429 | 9.32E-05 | 0.248418 | | 1.15832 | 2.20677 | | 14.3768 | 14.2109 | 14.3745 | 14.0183 | 13.8865 | 13.9579 | 14.3242 | 14.1747 | 7 | 7 | 6 | 5 | 5 | 5 | 6 | 7 |
| EWSR1 | Q01844;Q0 | 0.000251 | 5.63E-05 | 0.248779 | + | 2.12567 | 3.95583 | | 18.7322 | 18.6462 | 18.7351 | 18.4291 | 18.3918 | 18.6317 | 18.4124 | 18.4137 | 4 | 5 | 5 | 5 | 3 | 5 | 5 | 5 |
| GPX1 | P07203 | 6.66E-06 | 1.78E-06 | 0.24878 | | 0.584269 | 1.24237 | | 17.5766 | 17.6234 | 17.59 | 17.3124 | 17.1911 | 17.6121 | 17.7312 | 16.8925 | 7 | 8 | 8 | 8 | 7 | 7 | 8 | 8 |
| CCAR2 | Q8N163 | 9.70E-16 | 1.67E-15 | 0.248851 | + | 2.6648 | 5.12682 | | 17.5807 | 17.5181 | 17.5626 | 17.2754 | 17.4266 | 17.2125 | 17.2948 | 17.3155 | 21 | 21 | 21 | 20 | 20 | 20 | 21 | 21 |
| WDR5 | P61964 | 4.94E-13 | 3.87E-13 | 0.248949 | + | 2.09842 | 3.90128 | | 15.9967 | 16.0203 | 15.9871 | 15.8067 | 15.8473 | 15.6205 | 15.6551 | 15.8325 | 11 | 11 | 10 | 11 | 10 | 10 | 10 | 10 |
| MTHFD1 | P11586 | 1.48E-11 | 8.30E-12 | 0.248954 | + | 2.8424 | 5.55645 | | 18.5118 | 18.5628 | 18.4988 | 18.3457 | 18.1743 | 18.3147 | 18.2292 | 18.3136 | 47 | 48 | 48 | 48 | 47 | 47 | 47 | 47 |
| RAP1GDS1 | P52306 | 8.01E-14 | 7.84E-14 | 0.248956 | | 0.90203 | 1.78041 | | 17.7037 | 17.7073 | 17.7583 | 17.8339 | 17.4304 | 17.2301 | 17.3272 | 17.5492 | 23 | 23 | 23 | 22 | 22 | 23 | 22 | 22 |
| DCAF8 | Q5TAQ9 | 5.27E-16 | 9.60E-16 | 0.249097 | + | 1.73894 | 3.21545 | | 15.855 | 15.9927 | 15.8633 | 15.5229 | 15.5926 | 15.8383 | 15.642 | 15.677 | 8 | 10 | 9 | 10 | 7 | 8 | 10 | 7 |
| YAE1 | Q9NRH1 | 0.003303 | 0.000609 | 0.249358 | | 0.203643 | 0.519338 | | NaN | 13.1846 | 12.8742 | 13.8101 | 12.3768 | 12.2849 | 12.4682 | 12.9604 | NaN | 2 | 2 | 2 | 2 | 1 | 2 | 1 |
| ORMDL3 | Q8N138;Q8 | 1.80E-11 | 1.00E-11 | 0.249405 | | 0.287 | 0.689257 | | 15.6994 | 15.5399 | 15.6991 | 15.919 | 14.6068 | 15.2325 | 15.1441 | 16.0812 | 2 | 2 | 1 | 2 | 1 | 1 | 1 | 2 |
| INTS10 | Q9NVR2 | 0.002871 | 0.000538 | 0.249478 | + | 2.91493 | 5.73908 | | 15.3263 | 15.3053 | 15.3937 | 15.1167 | 15.1763 | 15.0601 | 15.0022 | 15.1061 | 6 | 8 | 9 | 5 | 3 | 5 | 6 | 4 |
| TIMM29 | Q9BSF4 | 9.99E-15 | 1.27E-14 | 0.249767 | | 1.11588 | 2.13592 | | 14.6698 | 14.739 | 14.6874 | 14.3999 | 14.2253 | 14.7616 | 14.4405 | 14.4175 | 6 | 6 | 6 | 6 | 5 | 6 | 6 | 4 |
| EHD3 | Q9NZN3 | 3.17E-15 | 4.68E-15 | 0.249848 | + | 2.20997 | 4.12719 | | 18.7605 | 18.8022 | 18.7742 | 18.485 | 18.5374 | 18.4024 | 18.6776 | 18.5433 | 31 | 30 | 30 | 27 | 28 | 27 | 31 | 28 |
| HNRNPL | P14866 | 2.52E-11 | 1.36E-11 | 0.249879 | + | 3.39913 | 7.07749 | | 18.9425 | 18.9263 | 18.8508 | 18.6836 | 18.6626 | 18.6924 | 18.5732 | 18.6715 | 20 | 22 | 23 | 21 | 21 | 20 | 20 | 20 |
| FGD1 | P98174 | 0.038176 | 0.006234 | 0.249961 | | 0.274924 | 0.706181 | | 14.253 | 14.0172 | 14.4092 | NaN | NaN | 14.4084 | 13.5446 | NaN | 2 | 1 | 1 | NaN | NaN | 1 | 2 | NaN |
| PRPF19 | Q9UMS4 | 1.08E-09 | 4.45E-10 | 0.249984 | + | 5.02013 | 13.6634 | | 18.0827 | 18.1362 | 18.0954 | 17.849 | 17.8812 | 17.8769 | 17.8274 | 17.8394 | 16 | 15 | 17 | 15 | 15 | 16 | 16 | 15 |
| INTS14 | Q96SY0;Q9 | 5.80E-15 | 7.83E-15 | 0.250168 | + | 1.39695 | 2.61054 | | 14.6273 | 14.5943 | 14.3695 | 14.0997 | 14.4433 | 14.2422 | 14.3379 | 14.2778 | 4 | 5 | 5 | 5 | 3 | 5 | 3 | 3 |
| IRF2BPL | Q9H1B7 | 6.35E-09 | 2.37E-09 | 0.250168 | | 0.664708 | 1.38133 | | 15.4156 | 15.3029 | 15.3323 | 15.0515 | 14.7911 | 15.2822 | 15.5162 | 14.8593 | 5 | 8 | 6 | 5 | 4 | 4 | 8 | 6 |
| FDFT1 | P37268 | 7.86E-10 | 3.29E-10 | 0.250618 | | 0.180256 | 0.462081 | | 17.0682 | 17.0336 | 17.0291 | 18.0514 | 16.4853 | 16.2871 | 15.7721 | 17.3693 | 13 | 12 | 12 | 14 | 12 | 11 | 12 | 14 |
| MGST1 | P10620 | 1.98E-10 | 9.08E-11 | 0.251187 | | 0.33014 | 0.775344 | | 18.6375 | 18.4951 | 18.5756 | 18.2908 | 18.2128 | 17.5666 | 18.4421 | 19.0788 | 6 | 6 | 6 | 6 | 6 | 6 | 6 | 6 |
| IPO4 | Q8TEX9;Q8 | 1.17E-08 | 4.21E-09 | 0.251538 | + | 2.51209 | 4.77599 | | 17.0912 | 17.1218 | 17.0392 | 16.9608 | 16.7386 | 16.7861 | 16.8284 | 16.8489 | 26 | 26 | 25 | 24 | 24 | 23 | 27 | 24 |
| MFSD5 | Q6N075;Q6 | 5.76E-10 | 2.46E-10 | 0.251909 | + | 1.33905 | 2.51152 | | 17.7948 | 17.8802 | 17.9306 | 17.8149 | 17.5142 | 17.4946 | 17.7689 | 17.4906 | 4 | 3 | 4 | 4 | 3 | 3 | 3 | 4 |
| LSM2 | Q9Y333 | 2.39E-06 | 6.66E-07 | 0.253253 | | 0.706466 | 1.45249 | | 16.5568 | 16.4904 | 16.6536 | 16.3747 | 16.0972 | 15.9768 | 16.4126 | 16.707 | 5 | 6 | 6 | 5 | 5 | 5 | 5 | 5 |
| ACAT1 | P24752 | 3.85E-12 | 2.42E-12 | 0.253551 | + | 1.64033 | 3.037 | | 18.7488 | 18.761 | 18.74 | 18.5183 | 18.6711 | 18.3202 | 18.397 | 18.5755 | 24 | 24 | 23 | 23 | 24 | 24 | 23 | 24 |
| MMUT | P22033 | 3.28E-24 | 9.27E-23 | 0.253662 | + | 2.66487 | 5.12699 | | 16.6797 | 16.6263 | 16.6466 | 16.3153 | 16.3302 | 16.516 | 16.3999 | 16.4246 | 15 | 14 | 14 | 12 | 12 | 13 | 13 | 12 |
| CDK12 | Q9NYV4;Q9 | 0.017191 | 0.002876 | 0.253905 | | 0.21677 | 0.542443 | | 12.9336 | 13.0338 | 13.0378 | 13.2402 | 11.4362 | 12.6354 | 13.3694 | 13.0578 | 3 | 3 | 3 | 4 | 2 | 3 | 1 | 4 |
| ANP32B | Q92688;Q9 | 6.45E-08 | 2.12E-08 | 0.254386 | + | 1.90903 | 3.53251 | | 18.8491 | 18.7473 | 18.8083 | 18.4297 | 18.4584 | 18.7147 | 18.6025 | 18.5306 | 6 | 6 | 6 | 5 | 6 | 6 | 4 | 5 |
| LMNA | P02545 | 4.13E-20 | 3.00E-19 | 0.254562 | + | 1.35211 | 2.53378 | | 21.9227 | 21.898 | 21.8445 | 21.5746 | 21.9037 | 21.6171 | 21.4505 | 21.6233 | 72 | 72 | 72 | 70 | 72 | 72 | 71 | 72 |
| NUDT4B;NUDT4 | A0A024RB( | 0.000273 | 6.09E-05 | 0.254584 | + | 1.91332 | 3.54067 | | 16.8074 | 16.7935 | 16.7834 | 16.651 | 16.583 | 16.4165 | 16.6435 | 16.4068 | 4 | 4 | 4 | 3 | 3 | 4 | 4 | 4 |
| UBR2 | Q8IWV8 | 1.44E-14 | 1.75E-14 | 0.254752 | + | 2.64441 | 5.07902 | | 15.0038 | 15.0446 | 14.9989 | 14.6489 | 14.7462 | 14.8041 | 14.8696 | 14.7364 | 19 | 20 | 22 | 18 | 9 | 16 | 16 | 13 |
| EXOSC7 | Q15024 | 3.54E-12 | 2.25E-12 | 0.255 | | 1.02628 | 1.98684 | | 15.8955 | 15.8549 | 15.7802 | 15.8637 | 15.4726 | 15.5484 | 15.3283 | 15.7299 | 9 | 9 | 9 | 9 | 8 | 6 | 7 | 10 |
| GNL3 | Q9BVP2;Q9 | 2.66E-09 | 1.04E-09 | 0.25544 | + | 1.35854 | 2.54477 | | 16.4672 | 16.4929 | 16.4885 | 16.1183 | 16.1988 | 16.4812 | 16.2902 | 16.0488 | 18 | 20 | 19 | 19 | 19 | 19 | 18 | 18 |
| COX7A2L | O14548 | 5.06E-11 | 2.57E-11 | 0.255441 | | 0.567802 | 1.21355 | | 15.6083 | 15.5294 | 15.7106 | 15.5917 | 15.3242 | 14.8026 | 15.3846 | 15.7001 | 5 | 5 | 5 | 5 | 5 | 4 | 5 | 5 |
| RHOT2 | Q8IXI1 | 5.60E-11 | 2.82E-11 | 0.256572 | | 0.31723 | 0.749866 | | 16.0692 | 16.0726 | 16.0933 | 14.8109 | 16.2418 | 15.9951 | 16.0537 | 16.0075 | 5 | 5 | 5 | 5 | 5 | 5 | 5 | 5 |
| LZIC | Q8WZA0;Q | 3.14E-05 | 7.85E-06 | 0.256648 | | 0.37433 | 0.860919 | | 16.5547 | 16.5045 | 16.7341 | 16.6495 | 16.1422 | 15.5611 | 16.6398 | 16.7129 | 6 | 6 | 6 | 6 | 4 | 3 | 5 | 4 |
| ASS1 | P00966 | 3.51E-08 | 1.20E-08 | 0.256702 | | 0.155571 | 0.405861 | | 18.6222 | 18.6635 | 18.5524 | 16.5732 | 18.7192 | 18.263 | 19.2372 | 18.9876 | 23 | 23 | 23 | 20 | 22 | 22 | 22 | 24 |
| MAZ | P56270;P5 | 0.003742 | 0.000683 | 0.256907 | | 1.22703 | 2.43127 | | 15.1897 | 15.331 | 15.1638 | NaN | 14.7575 | 15.0041 | 15.1522 | 14.9712 | 2 | 1 | 2 | NaN | 1 | 1 | 1 | 1 |
| PLRG1 | O43660;O4 | 2.22E-14 | 2.55E-14 | 0.256969 | + | 2.46279 | 4.66616 | | 15.7338 | 15.8053 | 15.7706 | 15.3626 | 15.5199 | 15.5239 | 15.5815 | 15.5768 | 12 | 13 | 13 | 14 | 13 | 12 | 13 | 12 |
| BCAP29 | Q9UHQ4 | 1.26E-17 | 3.85E-17 | 0.257182 | + | 2.02747 | 3.76103 | | 15.8796 | 15.9855 | 15.976 | 15.8385 | 15.6162 | 15.7691 | 15.6075 | 15.618 | 9 | 9 | 11 | 9 | 9 | 10 | 9 | 10 |
| NHP2 | Q9NX24 | 6.21E-10 | 2.63E-10 | 0.25757 | + | 2.08631 | 3.87716 | | 16.6029 | 16.7582 | 16.7135 | 16.3512 | 16.3209 | 16.5321 | 16.5203 | 16.4453 | 4 | 5 | 4 | 3 | 4 | 4 | 4 | 3 |
| GART | P22102 | 2.22E-11 | 1.21E-11 | 0.257664 | + | 2.65424 | 5.10202 | | 17.6275 | 17.6899 | 17.6264 | 17.4794 | 17.404 | 17.407 | 17.258 | 17.4029 | 47 | 45 | 48 | 47 | 41 | 46 | 45 | 45 |
| SMC1A | Q14683 | 8.48E-20 | 5.38E-19 | 0.257876 | + | 2.14023 | 3.98515 | | 15.9593 | 16.0211 | 15.9877 | 15.8458 | 15.7623 | 15.6307 | 15.6093 | 15.8095 | 43 | 41 | 42 | 39 | 36 | 35 | 32 | 37 |
| RXRB | P28702;P2 | 5.47E-07 | 1.64E-07 | 0.258016 | + | 2.21782 | 4.14334 | | 14.9431 | 14.9038 | 15.0136 | 14.6407 | 14.6107 | 14.7314 | 14.646 | 14.8488 | 5 | 5 | 5 | 5 | 5 | 5 | 4 | 4 |
| ARMC8 | Q8IUR7 | 1.55E-06 | 4.41E-07 | 0.25808 | + | 1.91619 | 3.54614 | | 15.7844 | 15.7594 | 15.814 | 15.5201 | 15.3438 | 15.6441 | 15.6281 | 15.5032 | 11 | 11 | 11 | 9 | 8 | 7 | 9 | 8 |
| UAP1L1 | Q3KQV9 | 2.45E-05 | 6.18E-06 | 0.258161 | | 0.846583 | 1.68804 | | 18.3782 | 18.3535 | 18.3561 | 17.9804 | 18.3945 | 18.139 | 18.2705 | 17.7378 | 18 | 18 | 17 | 18 | 16 | 18 | 20 | 17 |
| PTPRM | P28827;P2 | 6.73E-15 | 8.93E-15 | 0.258451 | | 0.915951 | 1.80356 | | 15.3631 | 15.3127 | 15.3593 | 15.1765 | 15.2003 | 15.3789 | 14.8418 | 14.8355 | 12 | 15 | 15 | 11 | 11 | 13 | 9 | 10 |
| SRRM1 | Q8IYB3;Q8 | 5.50E-07 | 1.65E-07 | 0.258462 | | 1.04573 | 2.01916 | | 15.1879 | 15.2233 | 15.2979 | 15.2086 | 14.653 | 14.9199 | 15.1006 | 15.0074 | 7 | 8 | 8 | 7 | 5 | 7 | 7 | 6 |

| Gene names | UniProt IDs | Protein Group P-value | Protein Group Q-value | Log2 Difference NUP214 vs Controls (n=5) | p<0.05 | -log10 p-value | T-test Test statistic | NPC Localization | Log2 MS2_quantity_ NUP214_1 | Log2 MS2_quantity_ NUP214_2 | Log2 MS2_quantity_ NUP214_3 | Log2 MS2_quantity_ Control_1 | Log2 MS2_quantity_ Control_2 | Log2 MS2_quantity_ Control_3 | Log2 MS2_quantity_ Control_4 | Log2 MS2_quantity_ Control_5 | Number Peptides for Quantitation NUP214_1 | Number Peptides for Quantitation NUP214_2 | Number Peptides for Quantitation NUP214_3 | Number Peptides for Quantitation Control_1 | Number Peptides for Quantitation Control_2 | Number Peptides for Quantitation Control_3 | Number Peptides for Quantitation Control_4 | Number Peptides for Quantitation Control_5 |
|---|---|---|---|---|---|---|---|---|---|---|---|---|---|---|---|---|---|---|---|---|---|---|---|---|
| SLC39A11 | Q8N1S5;Q8 | 0.000286 | 6.36E-05 | 0.258495 | + | 2.20218 | 4.1112 | | 16.051 | 15.9993 | 16.0594 | 15.8572 | 15.7515 | 15.7489 | 15.8972 | 15.6354 | 3 | 3 | 3 | 3 | 2 | 3 | 3 | 3 |
| LARS2 | Q15031 | 3.14E-17 | 8.55E-17 | 0.258597 | + | 1.67408 | 3.09766 | | 15.041 | 15.1998 | 15.1616 | 15.0802 | 14.7906 | 14.9155 | 14.771 | 14.8204 | 15 | 18 | 18 | 17 | 8 | 13 | 10 | 12 |
| PPP1R3D | O95685 | 1.03E-06 | 3.00E-07 | 0.258787 | + | 1.58174 | 2.93263 | | 15.0536 | 15.0693 | 15.0518 | 14.8878 | 14.9745 | 14.5912 | 14.7266 | 14.8172 | 5 | 5 | 6 | 4 | 4 | 4 | 4 | 3 |
| RPL38 | P63173 | 7.99E-06 | 2.12E-06 | 0.259292 | | 0.39426 | 0.898763 | | 19.7456 | 19.9018 | 19.7615 | 19.8693 | 19.4671 | 18.7355 | 19.8138 | 19.8327 | 8 | 8 | 8 | 8 | 8 | 8 | 8 | 8 |
| ZFYVE21 | Q9BQ24;Q9 | 9.82E-05 | 2.32E-05 | 0.259606 | | 0.820743 | 1.64488 | | 16.316 | 16.3341 | 16.3018 | 16.4471 | 15.9951 | 15.8627 | 15.795 | 16.1885 | 5 | 5 | 6 | 5 | 5 | 5 | 6 | 5 |
| STAG2 | Q8N3U4;Q | 2.10E-06 | 5.90E-07 | 0.259673 | + | 1.84744 | 3.41627 | | 15.9848 | 15.9499 | 16.0121 | 15.8247 | 15.5261 | 15.7361 | 15.6902 | 15.8358 | 18 | 19 | 20 | 16 | 10 | 15 | 12 | 12 |
| NIPSNAP1 | Q9BPW8 | 2.70E-05 | 6.79E-06 | 0.259735 | | 0.966029 | 1.88676 | | 16.9157 | 16.9395 | 16.8812 | 16.7299 | 16.4435 | 16.9444 | 16.7505 | 16.3937 | 8 | 8 | 8 | 8 | 7 | 8 | 7 | 8 |
| ENGASE | Q8NFI3;Q8 | 0.01638 | 0.002747 | 0.259899 | | 0.421594 | 0.988662 | | 13.4389 | 13.4924 | 13.492 | NaN | 13.6445 | 12.7393 | NaN | 13.2599 | 2 | 2 | 1 | NaN | 2 | 1 | NaN | 1 |
| THADA | Q6YHU6 | 1.99E-08 | 6.97E-09 | 0.260738 | | 0.651255 | 1.35827 | | 15.0368 | 15.0291 | 14.8889 | 14.6012 | 14.8578 | 15.1147 | 14.2641 | 14.7833 | 13 | 13 | 14 | 12 | 7 | 14 | 11 | 11 |
| GTF3C4 | Q9UKN8 | 8.91E-08 | 2.89E-08 | 0.261115 | + | 1.93185 | 3.57601 | | 15.6852 | 15.8232 | 15.7748 | 15.3461 | 15.5627 | 15.4638 | 15.4825 | 15.6446 | 10 | 11 | 10 | 11 | 7 | 10 | 9 | 7 |
| GRWD1 | Q9BQ67 | 4.54E-06 | 1.23E-06 | 0.261263 | | 1.22772 | 2.32313 | | 16.3802 | 16.4481 | 16.3771 | 16.3388 | 16.2634 | 16.21 | 15.988 | 15.9027 | 9 | 10 | 10 | 9 | 9 | 9 | 9 | 8 |
| LRRFIP1 | Q32MZ4-2 | 9.69E-17 | 2.31E-16 | 0.261733 | | 0.939577 | 1.84282 | | 15.9572 | 15.9632 | 16.0687 | 16.0494 | 15.6601 | 15.5401 | 15.5174 | 15.9061 | 31 | 31 | 31 | 32 | 24 | 27 | 27 | 30 |
| SH3D19 | Q5HYK7 | 2.56E-11 | 1.38E-11 | 0.261881 | + | 2.01177 | 3.73035 | | 15.7606 | 15.7331 | 15.6886 | 15.3466 | 15.5812 | 15.5279 | 15.5351 | 15.3368 | 17 | 18 | 17 | 16 | 13 | 17 | 18 | 17 |
| CIAO2A | Q9H5X1 | 0.012865 | 0.002178 | 0.262215 | | 0.528272 | 1.16575 | | 15.9562 | 15.8328 | 15.9452 | 15.6819 | NaN | 15.2364 | 15.5386 | 16.1398 | 2 | 2 | 2 | 2 | NaN | 1 | 1 | 2 |
| CNNM3 | Q8NE01 | 9.82E-05 | 2.32E-05 | 0.262339 | | 0.941747 | 1.84643 | | 14.7245 | 14.784 | 14.8105 | 14.6854 | 14.5831 | 14.7486 | 14.187 | 14.3492 | 7 | 7 | 6 | 7 | 6 | 5 | 7 | 5 |
| EHHADH | Q08426 | 1.34E-14 | 1.64E-14 | 0.262658 | | 1.16397 | 2.21622 | | 15.8128 | 15.8593 | 15.8299 | 15.7156 | 15.6673 | 15.5369 | 15.2399 | 15.6972 | 22 | 23 | 22 | 21 | 18 | 18 | 19 | 17 |
| TSFM | P43897 | 3.58E-06 | 9.83E-07 | 0.263674 | | 0.708665 | 1.45622 | | 16.3646 | 16.4484 | 16.4672 | 16.2381 | 15.6475 | 16.1764 | 16.3823 | 16.371 | 12 | 12 | 12 | 11 | 7 | 10 | 11 | 11 |
| PRRX1 | P54821-2 | 2.22E-16 | 4.67E-16 | 0.264517 | | 0.324296 | 0.76384 | | 17.7969 | 17.6859 | 17.7783 | 18.3202 | 17.3741 | 16.8402 | 17.128 | 17.7834 | 6 | 6 | 6 | 6 | 6 | 5 | 6 | 6 |
| UBLCP1 | Q8WVY7 | 3.94E-12 | 2.48E-12 | 0.264881 | | 0.996273 | 1.93699 | | 16.6138 | 16.6484 | 16.575 | 16.2039 | 16.2165 | 16.7378 | 16.3637 | 16.2159 | 14 | 14 | 14 | 14 | 12 | 13 | 14 | 14 |
| IDH1 | O75874 | 1.03E-14 | 1.29E-14 | 0.265067 | + | 1.62634 | 3.01197 | | 19.1441 | 19.1071 | 19.0996 | 18.9592 | 18.7776 | 19.026 | 18.8411 | 18.6556 | 23 | 23 | 24 | 23 | 23 | 23 | 22 | 23 |
| TBC1D15 | Q8TC07;Q8 | 6.11E-12 | 3.72E-12 | 0.265483 | + | 3.10444 | 6.23717 | | 16.7366 | 16.7779 | 16.7341 | 16.4003 | 16.4686 | 16.5916 | 16.4923 | 16.4677 | 21 | 22 | 22 | 19 | 15 | 21 | 22 | 19 |
| SELENON | Q9NZV5;Q9 | 0.002648 | 0.000502 | 0.265572 | | 1.04086 | 2.01106 | | 15.62 | 15.5911 | 15.5807 | 15.3718 | 15.6388 | 15.3363 | 15.0188 | 15.2929 | 6 | 6 | 6 | 4 | 5 | 4 | 6 | 5 |
| FLCN | Q8NFG4 | 0.000608 | 0.000129 | 0.26562 | | 1.24752 | 2.35647 | | 15.2376 | 15.4514 | 15.2359 | 14.8705 | 15.1088 | 15.2892 | 14.9109 | 15.0341 | 4 | 4 | 4 | 3 | 3 | 4 | 4 | 3 |
| TBCD | Q9BTW9 | 1.31E-12 | 9.17E-13 | 0.265677 | + | 2.4188 | 4.56951 | | 16.8413 | 16.8315 | 16.8075 | 16.5135 | 16.614 | 16.6653 | 16.5943 | 16.4183 | 34 | 36 | 34 | 30 | 31 | 32 | 33 | 31 |
| SMU1 | Q2TAY7 | 4.14E-12 | 2.58E-12 | 0.265858 | + | 2.88485 | 5.66282 | | 17.3342 | 17.408 | 17.306 | 17.0798 | 17.1288 | 17.0304 | 17.0048 | 17.1739 | 15 | 16 | 14 | 14 | 15 | 14 | 15 | 14 |
| SMAD4 | Q13485 | 4.82E-21 | 4.85E-20 | 0.265875 | + | 3.10507 | 6.23889 | | 15.991 | 15.9826 | 15.9173 | 15.7034 | 15.7825 | 15.7166 | 15.6005 | 15.6858 | 7 | 6 | 5 | 5 | 4 | 6 | 4 | 4 |
| NFYB | P25208 | 0.00038 | 8.32E-05 | 0.265969 | | 0.392249 | 0.894963 | | 14.9661 | 14.9243 | 15.4403 | 15.1148 | 14.043 | 15.0961 | 15.0602 | 14.9073 | 1 | 1 | 1 | 1 | 1 | 1 | 1 | 1 |
| SPECC1 | Q5M775;Q | 0.025393 | 0.004194 | 0.266052 | | 0.277502 | 0.678064 | | 18.3998 | 18.3062 | 18.2178 | NaN | 18.9428 | 17.5777 | 17.5231 | 18.124 | 2 | 2 | 2 | NaN | 1 | 1 | 1 | 2 |
| INTS8 | Q75QN2;Q | 6.27E-05 | 1.52E-05 | 0.26649 | + | 1.68927 | 3.1251 | | 15.3107 | 15.2864 | 15.1502 | 14.9606 | 15.1443 | 15.0542 | 14.9569 | 14.7971 | 3 | 5 | 5 | 5 | 2 | 4 | 4 | 3 |
| WBP11 | Q9Y2W2 | 1.49E-10 | 6.97E-11 | 0.266603 | + | 2.31388 | 4.34398 | | 15.5493 | 15.5723 | 15.6196 | 15.4365 | 15.3383 | 15.3372 | 15.1613 | 15.2956 | 12 | 13 | 13 | 13 | 10 | 13 | 12 | 13 |
| CNOT9 | Q92600 | 1.17E-13 | 1.09E-13 | 0.266932 | | 0.485183 | 1.06654 | | 16.8379 | 16.7828 | 16.8802 | 16.6029 | 16.3071 | 16.0084 | 16.8738 | 17.0413 | 9 | 9 | 10 | 8 | 4 | 6 | 9 | 9 |
| PHRF1 | Q9P1Y6;Q9 | 0.007966 | 0.001385 | 0.267858 | | 0.45707 | 1.01543 | | 13.4402 | 13.5786 | 13.4695 | 13.7941 | 12.8512 | 13.5229 | 13.2169 | 12.7561 | 2 | 2 | 3 | 2 | 1 | 2 | 2 | 2 |
| GNPDA2 | Q8TDQ7;Q8 | 5.69E-13 | 4.38E-13 | 0.268063 | | 1.11793 | 2.13934 | | 17.7873 | 17.8806 | 17.8996 | 17.625 | 17.3404 | 17.9056 | 17.5398 | 17.5281 | 11 | 12 | 11 | 12 | 11 | 12 | 12 | 12 |
| SPTAN1 | Q13813 | 1.69E-16 | 3.74E-16 | 0.268864 | + | 1.47281 | 2.74156 | | 18.4012 | 18.4809 | 18.4387 | 18.3096 | 18.0861 | 18.33 | 17.9409 | 18.1905 | 159 | 161 | 158 | 159 | 151 | 159 | 151 | 158 |
| SNRPD3 | P62318;P6 | 8.72E-08 | 2.83E-08 | 0.269154 | | 0.774248 | 1.56694 | | 20.295 | 20.2894 | 20.2405 | 20.1365 | 19.9865 | 19.5331 | 20.0772 | 20.2958 | 4 | 4 | 4 | 3 | 4 | 4 | 4 | 4 |
| PIR | O00625 | 0.000687 | 0.000145 | 0.269319 | | 0.54228 | 1.16859 | | 15.0958 | 15.025 | 15.0881 | 14.602 | 14.6592 | 15.3612 | 15.0027 | 14.3764 | 5 | 6 | 5 | 4 | 3 | 5 | 6 | 3 |
| H6PD | O95479;O9 | 1.50E-16 | 3.38E-16 | 0.269373 | + | 1.47835 | 2.7512 | | 16.9631 | 16.983 | 16.9485 | 16.5986 | 16.8237 | 16.8217 | 16.4538 | 16.7796 | 20 | 21 | 22 | 22 | 18 | 20 | 21 | 20 |
| CYP27A1 | Q02318 | 0.000763 | 0.00016 | 0.269618 | | 0.981599 | 1.91262 | | 14.5365 | 14.5459 | 14.5679 | 14.6402 | 14.3187 | 14.2459 | 14.2108 | 13.987 | 8 | 9 | 7 | 5 | 4 | 4 | 5 | 4 |
| SCD | O00767 | 1.67E-06 | 4.73E-07 | 0.27028 | | 0.788087 | 1.59018 | | 18.8208 | 18.797 | 18.6581 | 18.814 | 18.6849 | 18.3703 | 18.1002 | 18.4723 | 4 | 5 | 5 | 5 | 3 | 4 | 3 | 3 |
| SVEP1 | Q4LDE5 | 0.011403 | 0.001943 | 0.270435 | | 0.344035 | 0.813602 | | 14.3192 | 13.8432 | 14.0027 | 14.4135 | 13.2016 | 13.5458 | NaN | 13.9776 | 3 | 3 | 6 | 6 | 2 | 2 | NaN | 2 |
| ARPC5L | Q9BPX5 | 0.001433 | 0.000289 | 0.270583 | | 0.289544 | 0.694412 | | 19.0343 | 18.9733 | 19.0589 | 19.3413 | 18.6066 | 17.7174 | 18.8317 | 19.261 | 5 | 5 | 5 | 5 | 5 | 5 | 5 | 5 |
| MICOS13 | Q5XKP0 | 2.62E-08 | 9.05E-09 | 0.270633 | | 0.460522 | 1.02174 | | 16.2935 | 16.4249 | 16.4291 | 16.6214 | 15.4871 | 16.3799 | 16.1838 | 15.8872 | 3 | 3 | 3 | 3 | 3 | 2 | 2 | 3 |
| INTS2 | Q9H0H0 | 0.000286 | 6.36E-05 | 0.27138 | + | 1.94864 | 3.60818 | | 15.5183 | 15.4841 | 15.2732 | 15.0715 | 15.2674 | 15.1961 | 15.071 | 15.163 | 4 | 5 | 5 | 4 | 2 | 3 | 3 | 3 |
| DIDO1 | Q9BTC0 | 5.46E-07 | 1.64E-07 | 0.271412 | + | 2.28531 | 4.28373 | | 15.4643 | 15.4679 | 15.4688 | 15.133 | 15.1111 | 15.2157 | 15.3723 | 15.146 | 16 | 16 | 13 | 17 | 9 | 11 | 17 | 12 |
| NOP58 | Q9Y2X3 | 1.06E-12 | 7.61E-13 | 0.271493 | + | 2.07812 | 3.86088 | | 17.6104 | 17.6731 | 17.667 | 17.421 | 17.2443 | 17.5391 | 17.3959 | 17.2929 | 23 | 22 | 22 | 22 | 18 | 22 | 21 | 20 |
| MT-ND4 | P03905 | 1.33E-18 | 5.64E-18 | 0.272147 | | 0.537379 | 1.15991 | | 15.6765 | 15.4858 | 15.6758 | 15.6277 | 14.755 | 15.726 | 15.3905 | 15.2035 | 5 | 5 | 5 | 4 | 5 | 5 | 5 | 5 |
| TOMM40 | O96008 | 1.14E-08 | 4.09E-09 | 0.272319 | | 1.03822 | 2.00667 | | 17.7947 | 17.6644 | 17.621 | 17.5657 | 17.0696 | 17.5896 | 17.3473 | 17.5331 | 9 | 9 | 9 | 8 | 8 | 9 | 9 | 10 |
| SMAD5 | Q99717 | 1.54E-11 | 8.64E-12 | 0.272853 | + | 1.7375 | 3.21282 | | 15.7039 | 15.6872 | 15.6049 | 15.4928 | 15.518 | 15.3093 | 15.1935 | 15.4488 | 5 | 7 | 7 | 2 | 4 | 4 | 4 | 4 |
| TBC1D5 | Q92609;Q9 | 1.93E-13 | 1.69E-13 | 0.273271 | + | 1.78164 | 3.29388 | | 16.0036 | 16.0835 | 16.0768 | 15.6289 | 15.9653 | 15.8672 | 15.6898 | 15.7557 | 22 | 20 | 19 | 20 | 18 | 21 | 19 | 19 |
| CRYZ | Q08257 | 3.68E-13 | 2.99E-13 | 0.273421 | | 0.412543 | 0.93311 | | 18.275 | 18.3105 | 18.2724 | 18.1016 | 18.3129 | 17.5878 | 17.4446 | 18.6159 | 16 | 15 | 16 | 15 | 15 | 15 | 14 | 16 |
| PBXIP1 | Q96AQ6;Q9 | 1.14E-14 | 1.42E-14 | 0.273983 | | 0.955472 | 1.86923 | | 16.906 | 16.9293 | 16.9455 | 16.3923 | 16.7298 | 16.5284 | 17.0352 | 16.5789 | 18 | 20 | 19 | 17 | 17 | 19 | 20 | 18 |
| PGAP4 | Q9BRR3 | 0.003159 | 0.000586 | 0.274003 | | 0.23285 | 0.57689 | | 16.4661 | 16.273 | 15.5586 | 16.4096 | 14.5695 | 15.975 | 16.13 | 16.0421 | 1 | 2 | 2 | 4 | 2 | 1 | 4 | 2 |
| CHD3 | Q12873;Q1 | 1.15E-05 | 2.99E-06 | 0.274433 | | 0.832379 | 1.66433 | | 15.6549 | 15.7644 | 15.7042 | 15.7903 | 15.1691 | 15.3766 | 15.1933 | 15.6378 | 15 | 19 | 19 | 14 | 14 | 15 | 14 | 13 |
| ECH1 | Q13011 | 5.26E-07 | 1.58E-07 | 0.274612 | | 0.915627 | 1.80302 | | 17.2521 | 17.1846 | 17.1545 | 16.656 | 17.1101 | 17.1022 | 17.1091 | 16.6349 | 12 | 10 | 12 | 10 | 10 | 12 | 12 | 9 |
| GATAD2B | Q8WXI9 | 1.81E-15 | 2.89E-15 | 0.274925 | + | 1.4602 | 2.71968 | | 15.5428 | 15.4706 | 15.4425 | 15.3399 | 15.0781 | 15.0459 | 15.163 | 15.425 | 10 | 10 | 10 | 9 | 7 | 8 | 8 | 9 |
| FUT11 | Q495W5-2 | 0.000537 | 0.000115 | 0.27568 | | 1.0768 | 2.07083 | | 15.5661 | 15.4991 | 15.5383 | 14.9012 | 15.3901 | 15.3783 | 15.4387 | 15.1857 | 5 | 4 | 5 | 5 | 6 | 4 | 6 | 4 |
| C15orf40 | Q8WUR7 | 6.38E-12 | 3.86E-12 | 0.275743 | | 0.937765 | 1.83982 | | 14.7251 | 14.9886 | 15.0174 | 14.5672 | 14.6471 | 14.2946 | 14.7964 | 14.8677 | 3 | 3 | 3 | 3 | 3 | 1 | 2 | 2 |
| C8orf82 | Q6P1X6 | 6.85E-10 | 2.89E-10 | 0.276184 | + | 2.89378 | 5.68536 | | 16.0266 | 16.0458 | 16.1255 | 15.8263 | 15.8976 | 15.7678 | 15.7211 | 15.7361 | 6 | 5 | 4 | 7 | 7 | 6 | 7 | 4 |
| GAS6 | Q14393;Q1 | 8.02E-12 | 4.75E-12 | 0.276492 | | 0.382133 | 0.875787 | | 16.3711 | 16.391 | 16.3383 | 16.8592 | 16.0331 | 15.599 | 15.6198 | 16.3405 | 17 | 16 | 16 | 16 | 12 | 14 | 11 | 15 |
| WARS1 | P23381 | 9.85E-11 | 4.72E-11 | 0.276565 | | 0.315862 | 0.747152 | | 18.9414 | 18.873 | 18.8764 | 18.0166 | 18.3604 | 19.5871 | 18.8573 | 18.2805 | 21 | 21 | 22 | 21 | 21 | 25 | 21 | 20 |
| TMEM33 | P57088 | 1.20E-07 | 3.84E-08 | 0.27664 | | 0.381071 | 0.873767 | | 17.6905 | 17.8469 | 17.599 | 17.9351 | 17.0523 | 16.8237 | 17.3647 | 18.0016 | 7 | 7 | 7 | 6 | 5 | 6 | 7 | 6 |
| NSMAF | Q92636;Q9 | 0.001364 | 0.000276 | 0.276822 | | 0.839105 | 1.67556 | | 14.3466 | 14.3777 | 14.3522 | 14.3903 | 13.6841 | 14.2845 | 14.0795 | 13.9717 | 12 | 9 | 10 | 2 | 3 | 7 | 3 | 4 |
| TXNRD2 | Q9NNW7;Q | 1.12E-06 | 3.23E-07 | 0.277242 | + | 1.34762 | 2.52612 | | 17.4434 | 17.4042 | 17.4357 | 17.0612 | 17.3086 | 17.2294 | 16.8711 | 17.2823 | 8 | 8 | 8 | 9 | 4 | 7 | 9 | 7 |
| TANC1 | Q9C0D5 | 1.54E-14 | 1.86E-14 | 0.277457 | | 1.12602 | 2.15283 | | 15.0759 | 15.1557 | 15.1234 | 14.9063 | 15.0704 | 14.9481 | 14.7708 | 14.5088 | 13 | 14 | 13 | 13 | 11 | 11 | 13 | 3 |
| WDR24 | Q96S15 | 0.02463 | 0.004072 | 0.277464 | | 1.03954 | 2.08657 | | 13.5223 | 13.222 | 13.5254 | 13.0825 | NaN | 13.0321 | 13.4049 | 13.0636 | 2 | 2 | 3 | 1 | NaN | 2 | 1 | 1 |
| SERINC3 | Q13530;Q1 | 2.99E-06 | 8.26E-07 | 0.277577 | | 0.477678 | 1.05296 | | 15.4918 | 15.609 | 15.7049 | 15.9686 | 14.9353 | 14.9257 | 15.5041 | 15.288 | 3 | 3 | 3 | 4 | 1 | 4 | 2 | 3 |
| GADD45GIP1 | Q8TAE8 | 1.99E-12 | 1.33E-12 | 0.277759 | + | 1.36022 | 2.54763 | | 16.4818 | 16.5109 | 16.4487 | 16.3932 | 16.3885 | 15.982 | 16.1348 | 16.1151 | 7 | 8 | 8 | 8 | 7 | 8 | 8 | 8 |
| SLC11A2 | P49281-2;I | 5.31E-10 | 2.28E-10 | 0.277933 | | 0.459962 | 1.03817 | | 15.4378 | 15.4899 | 15.3674 | 15.3467 | NaN | 15.445 | 14.483 | 15.3404 | 1 | 1 | 1 | 1 | NaN | 2 | 1 | 1 |
| SNX21 | Q969T3 | 2.96E-13 | 2.48E-13 | 0.278953 | + | 2.12395 | 3.95239 | | 16.0782 | 16.0994 | 16.1235 | 15.7799 | 15.9981 | 15.8781 | 15.7409 | 15.7102 | 8 | 9 | 8 | 9 | 5 | 8 | 7 | 8 |
| GUK1 | Q16774-2 | 1.61E-05 | 4.13E-06 | 0.278985 | + | 1.98605 | 3.68034 | | 16.7717 | 16.6347 | 16.6216 | 16.367 | 16.4791 | 16.2299 | 16.3889 | 16.5203 | 6 | 6 | 7 | 6 | 5 | 6 | 5 | 6 |
| IVD | P26440 | 2.69E-16 | 5.42E-16 | 0.278986 | | 0.852155 | 1.69734 | | 17.0307 | 17.0795 | 17.0075 | 16.3285 | 16.8689 | 16.881 | 16.6771 | 17.0457 | 14 | 14 | 14 | 13 | 13 | 12 | 14 | 13 |
| RAVER1 | Q8IY67-2 | 3.10E-10 | 1.37E-10 | 0.279207 | + | 1.72335 | 3.18698 | | 16.6086 | 16.5545 | 16.5939 | 16.4636 | 16.1956 | 16.2454 | 16.1656 | 16.4622 | 16 | 16 | 15 | 15 | 14 | 14 | 13 | 15 |
| MT-ATP6 | P00846 | 0.001336 | 0.000271 | 0.279706 | | 0.781035 | 1.57834 | | 18.049 | 17.9832 | 18.0045 | 18.1026 | 17.2843 | 17.6933 | 17.7423 | 17.8401 | 2 | 2 | 2 | 2 | 1 | 2 | 2 | 2 |
| COMT | P21964;P2 | 5.13E-19 | 2.49E-18 | 0.280122 | | 1.04349 | 2.01544 | | 19.5646 | 19.6515 | 19.6231 | 19.2129 | 19.3998 | 19.4504 | 19.5997 | 19.0019 | 17 | 18 | 17 | 17 | 17 | 17 | 18 | 17 |
| SNX5 | Q9Y5X3 | 2.63E-11 | 1.41E-11 | 0.28047 | + | 2.21239 | 4.13217 | | 16.3829 | 16.4022 | 16.3776 | 16.0175 | 16.149 | 16.2241 | 16.1856 | 15.9593 | 19 | 19 | 19 | 17 | 17 | 18 | 17 | 18 |
| RPL24 | P83731 | 1.33E-08 | 4.78E-09 | 0.281058 | + | 2.46762 | 4.67685 | | 20.5246 | 20.6001 | 20.4748 | 20.2974 | 20.2232 | 20.3162 | 20.1059 | 20.3178 | 7 | 6 | 7 | 7 | 7 | 7 | 7 | 7 |
| GBP2 | P32456 | 0.000514 | 0.00011 | 0.281154 | | 0.304109 | 0.723728 | | 16.0938 | 16.0707 | 16.18 | 16.7391 | 15.4981 | 16.07 | 14.9958 | 15.8653 | 6 | 7 | 6 | 6 | 2 | 6 | 3 | 4 |
| SMS | P52788 | 7.68E-09 | 2.83E-09 | 0.281159 | | 0.560954 | 1.20152 | | 17.3778 | 17.4491 | 17.3538 | 17.6961 | 16.8328 | 17.186 | 16.6832 | 17.1639 | 19 | 19 | 18 | 20 | 16 | 21 | 19 | 17 |

| Gene names | UniProt IDs | Protein Group P-value | Protein Group Q-value | Log2 Difference NUP214 vs Controls (n=5) | p<0.05 | -log10 p-value | T-test Test statistic | NPC Localization | Log2 MS2_quantity_NUP214_1 | Log2 MS2_quantity_NUP214_2 | Log2 MS2_quantity_NUP214_3 | Log2 MS2_quantity_Control_1 | Log2 MS2_quantity_Control_2 | Log2 MS2_quantity_Control_3 | Log2 MS2_quantity_Control_4 | Log2 MS2_quantity_Control_5 | Number Peptides for Quantitation NUP214_1 | Number Peptides for Quantitation NUP214_2 | Number Peptides for Quantitation NUP214_3 | Number Peptides for Quantitation Control_1 | Number Peptides for Quantitation Control_2 | Number Peptides for Quantitation Control_3 | Number Peptides for Quantitation Control_4 | Number Peptides for Quantitation Control_5 |
|---|---|---|---|---|---|---|---|---|---|---|---|---|---|---|---|---|---|---|---|---|---|---|---|---|
| ORC5 | O43913 | 6.15E-08 | 2.03E-08 | 0.28137 | | 0.478316 | 1.05411 | | 13.755 | 13.7268 | 13.5686 | 13.3671 | 13.4233 | 12.6909 | 13.839 | 13.6902 | 2 | 2 | 2 | 1 | 1 | 1 | 2 | 2 |
| CTDSPL2 | Q05D32;Q( | 0.00031 | 6.87E-05 | 0.281485 | | 1.07489 | 2.06765 | | 14.3737 | 14.2839 | 14.2354 | 13.8215 | 14.1988 | 13.7298 | 14.1447 | 14.1862 | 3 | 3 | 4 | 3 | 2 | 4 | 4 | 4 |
| CCDC9 | Q9Y3X0 | 6.77E-09 | 2.51E-09 | 0.281497 | + | 2.75487 | 5.3417 | | 15.2923 | 15.3276 | 15.4366 | 15.1862 | 14.9976 | 15.0376 | 15.0585 | 15.0736 | 11 | 10 | 12 | 10 | 10 | 9 | 10 | 9 |
| ARAP1 | Q96P48;Q9 | 3.17E-11 | 1.67E-11 | 0.281644 | | 0.730872 | 1.49383 | | 16.4366 | 16.4514 | 16.4604 | 16.5165 | 16.0173 | 16.4904 | 15.8188 | 15.9961 | 29 | 27 | 27 | 26 | 21 | 24 | 19 | 22 |
| RNMT | O43148;O4 | 3.06E-10 | 1.36E-10 | 0.281827 | + | 1.44416 | 2.6919 | | 16.035 | 16.0415 | 16.0897 | 15.5733 | 15.9377 | 15.957 | 15.7707 | 15.6292 | 12 | 13 | 12 | 11 | 12 | 11 | 11 | 13 |
| MDN1 | Q9NU22 | 7.77E-08 | 2.53E-08 | 0.28251 | + | 1.41971 | 2.64968 | | 14.3424 | 14.368 | 14.3348 | 14.2327 | 14.0012 | 14.2129 | 14.0872 | 13.7953 | 12 | 12 | 13 | 10 | 4 | 8 | 9 | 5 |
| UNK | Q9C0B0 | 0.000482 | 0.000104 | 0.282515 | + | 2.18953 | 4.08528 | | 14.1067 | 14.29 | 14.3265 | 13.8261 | 13.9731 | 13.9504 | 14.0099 | 14.0332 | 2 | 3 | 5 | 3 | 2 | 3 | 2 | 3 |
| RAP2C | Q9Y3L5 | 2.50E-05 | 6.30E-06 | 0.282996 | | 0.606941 | 1.28182 | | 16.1298 | 16.0982 | 16.0176 | 15.7401 | 15.2178 | 16.1483 | 16.0787 | 15.8095 | 2 | 2 | 2 | 3 | 2 | 2 | 2 | 3 |
| ARNT | P27540;P2 | 0.00096 | 0.000199 | 0.283208 | | 0.418471 | 0.959158 | | 15.4376 | 14.8126 | 15.1002 | 15.2696 | 14.267 | 14.7726 | NaN | 15.0251 | 3 | 3 | 3 | 2 | 2 | 2 | NaN | 1 |
| DEK | P35659 | 3.06E-09 | 1.19E-09 | 0.283367 | + | 1.73275 | 3.20413 | | 18.0472 | 18.1443 | 18.1126 | 17.9352 | 17.8991 | 17.5988 | 17.7434 | 17.9136 | 13 | 14 | 14 | 13 | 13 | 13 | 14 | 14 |
| CTNNBL1 | Q8WYA6;Q | 2.97E-12 | 1.92E-12 | 0.283902 | + | 2.12788 | 3.96028 | | 16.4495 | 16.5016 | 16.5504 | 16.3217 | 16.3112 | 16.2122 | 16.0363 | 16.2018 | 14 | 14 | 14 | 15 | 10 | 12 | 12 | 13 |
| BPHL | Q86WA6;Q | 1.57E-06 | 4.47E-07 | 0.283944 | | 1.20413 | 2.28351 | | 15.636 | 15.8235 | 15.7721 | 15.3497 | 15.72 | 15.3661 | 15.2516 | 15.6122 | 9 | 8 | 7 | 7 | 5 | 8 | 7 | 8 |
| SPTAN1 | Q13813-3 | 2.02E-08 | 7.06E-09 | 0.285122 | + | 1.31946 | 2.47819 | | 16.0207 | 16.1359 | 16.0407 | 15.7618 | 15.5811 | 16.0625 | 15.6496 | 15.8482 | 2 | 2 | 2 | 2 | 2 | 2 | 2 | 2 |
| PUS1 | Q9Y606;Q9 | 1.56E-08 | 5.54E-09 | 0.285683 | + | 2.08061 | 3.86584 | | 16.2421 | 16.2896 | 16.2643 | 16.1315 | 15.8111 | 16.0574 | 15.9275 | 15.971 | 10 | 10 | 9 | 8 | 6 | 11 | 11 | 10 |
| CCAR1 | Q8IX12;Q8 | 7.68E-13 | 5.70E-13 | 0.285931 | + | 3.63187 | 7.80409 | | 16.5304 | 16.4951 | 16.4978 | 16.2185 | 16.137 | 16.2993 | 16.2043 | 16.2502 | 23 | 24 | 24 | 22 | 20 | 23 | 23 | 21 |
| ARF5 | P84085 | 4.38E-05 | 1.08E-05 | 0.286075 | + | 1.53574 | 2.85149 | | 18.1387 | 18.1762 | 18.1567 | 17.7924 | 17.8283 | 18.1625 | 17.7353 | 17.8373 | 5 | 5 | 5 | 5 | 4 | 5 | 5 | 5 |
| ACVR1 | Q04771 | 3.90E-15 | 5.61E-15 | 0.286489 | | 0.514698 | 1.11957 | | 16.0051 | 16.02 | 16.0511 | 16.3723 | 15.1623 | 15.7423 | 15.7148 | 15.7029 | 5 | 6 | 6 | 5 | 5 | 6 | 5 | 5 |
| RPL36AL | Q969Q0 | 3.65E-07 | 1.12E-07 | 0.286605 | | 0.163529 | 0.424165 | | 19.0816 | 18.3132 | 17.7902 | 16.2787 | 18.4851 | 18.3367 | 18.7392 | 18.7022 | 2 | 2 | 2 | 1 | 2 | 2 | 2 | 2 |
| RCC1 | P18754;P1 | 2.46E-09 | 9.65E-10 | 0.286682 | + | 3.99608 | 9.06783 | | 17.3703 | 17.4065 | 17.33 | 17.0801 | 17.139 | 17.0737 | 17.0147 | 17.1036 | 12 | 13 | 13 | 11 | 11 | 13 | 12 | 11 |
| ZMYND8 | Q9ULU4;Q9 | 2.28E-14 | 2.61E-14 | 0.286759 | | 1.05276 | 2.03083 | | 14.841 | 14.863 | 14.7986 | 14.8474 | 14.7492 | 14.374 | 14.4447 | 14.3219 | 5 | 6 | 6 | 5 | 5 | 4 | 5 | 5 |
| CCDC47 | Q96A33 | 4.01E-11 | 2.07E-11 | 0.286902 | | 1.19779 | 2.27286 | | 16.666 | 16.6752 | 16.6991 | 16.5583 | 16.0719 | 16.6079 | 16.3509 | 16.377 | 18 | 16 | 16 | 17 | 14 | 16 | 17 | 16 |
| BAG1 | Q99933;Q9 | 0.009059 | 0.001563 | 0.287412 | | 0.143121 | 0.385852 | | 15.1999 | 14.487 | 14.8722 | NaN | 15.5432 | NaN | 13.1711 | 14.9826 | 1 | 1 | 1 | NaN | 1 | NaN | 2 | 1 |
| SEPHS1 | P49903 | 5.95E-08 | 1.97E-08 | 0.287618 | | 1.1608 | 2.21092 | | 16.975 | 17.097 | 17.0861 | 16.9424 | 16.4106 | 16.9097 | 16.7447 | 16.818 | 8 | 8 | 8 | 8 | 8 | 8 | 8 | 8 |
| LSM14B | Q9BX40 | 1.92E-09 | 7.64E-10 | 0.288035 | | 0.798794 | 1.60814 | | 15.8841 | 15.7682 | 15.7542 | 15.5384 | 15.7887 | 15.4246 | 15.0596 | 15.7594 | 5 | 5 | 5 | 6 | 3 | 5 | 4 | 4 |
| PCID2 | Q5JVF3;Q5 | 2.21E-10 | 1.00E-10 | 0.288556 | + | 2.05123 | 3.80771 | | 16.1595 | 16.1132 | 16.0788 | 15.8052 | 15.7548 | 15.808 | 15.7326 | 16.0424 | 16 | 15 | 16 | 15 | 13 | 16 | 13 | 15 |
| NAB2 | Q15742 | 0.000696 | 0.000147 | 0.288593 | | 0.816487 | 1.63776 | | 15.6648 | 15.7167 | 15.6409 | 14.9266 | 15.6238 | 15.2736 | 15.477 | 15.6265 | 9 | 9 | 8 | 7 | 7 | 6 | 6 | 8 |
| ABCC4 | O15439;O1 | 2.74E-12 | 1.78E-12 | 0.289341 | | 1.20055 | 2.27749 | | 15.237 | 15.2419 | 15.2055 | 15.2076 | 14.927 | 14.9663 | 14.9799 | 14.6133 | 14 | 16 | 14 | 15 | 8 | 15 | 14 | 12 |
| DOCK11 | Q5JSL3 | 1.59E-06 | 4.52E-07 | 0.289858 | | 0.784731 | 1.58455 | | 15.3354 | 15.3276 | 15.2744 | 15.1062 | 14.8935 | 15.4669 | 15.0152 | 14.6312 | 23 | 26 | 22 | 15 | 11 | 18 | 13 | 10 |
| PFAS | O15067 | 3.15E-14 | 3.44E-14 | 0.290133 | + | 2.54006 | 4.83903 | | 17.172 | 17.1505 | 17.2044 | 16.7955 | 16.9235 | 17.0371 | 16.8082 | 16.8632 | 25 | 26 | 24 | 25 | 21 | 23 | 23 | 25 |
| TPI1 | P60174;P6 | 6.49E-09 | 2.42E-09 | 0.290918 | + | 2.98266 | 5.91353 | | 21.5614 | 21.5638 | 21.606 | 21.2399 | 21.3726 | 21.3673 | 21.26 | 21.1909 | 18 | 18 | 18 | 18 | 18 | 18 | 18 | 18 |
| IMPDH1 | P20839;P2 | 6.82E-12 | 4.11E-12 | 0.291308 | + | 2.1714 | 4.0483 | | 16.9548 | 16.9339 | 16.9155 | 16.7029 | 16.5317 | 16.7621 | 16.4981 | 16.7224 | 16 | 15 | 15 | 15 | 16 | 16 | 16 | 16 |
| ITGA3 | P26006;P2 | 7.71E-16 | 1.36E-15 | 0.291449 | | 0.548797 | 1.18011 | | 17.0455 | 17.1273 | 17.1541 | 16.7092 | 17.3538 | 16.4308 | 16.4576 | 17.1359 | 19 | 20 | 20 | 18 | 18 | 19 | 19 | 19 |
| IPO9 | Q96P70 | 4.88E-10 | 2.10E-10 | 0.291838 | + | 2.97008 | 5.88084 | | 17.112 | 17.148 | 17.1428 | 16.9121 | 16.8456 | 16.8266 | 16.7134 | 16.9145 | 31 | 30 | 31 | 31 | 29 | 31 | 31 | 30 |
| TRAP1 | Q12931;Q1 | 1.23E-11 | 7.00E-12 | 0.291935 | + | 1.883 | 3.48317 | | 17.4149 | 17.4587 | 17.3943 | 17.2908 | 16.9751 | 17.2574 | 17.0994 | 17.0308 | 27 | 26 | 26 | 25 | 21 | 26 | 25 | 24 |
| BAIAP2 | Q9UQB8;Q9 | 9.56E-09 | 3.49E-09 | 0.291985 | | 0.356099 | 0.825906 | | 16.8971 | 16.9403 | 16.838 | 15.9602 | 16.2557 | 16.3444 | 17.3609 | 17.0781 | 19 | 18 | 19 | 12 | 10 | 14 | 17 | 16 |
| ANO6 | Q4KMQ2;Q | 6.56E-11 | 3.26E-11 | 0.292092 | | 0.88152 | 1.74628 | | 15.8384 | 15.8724 | 15.85 | 15.2912 | 15.5746 | 15.8797 | 15.7935 | 15.2685 | 21 | 21 | 21 | 21 | 19 | 22 | 22 | 20 |
| PLXND1 | Q9Y4D7 | 3.93E-16 | 7.44E-16 | 0.292198 | | 1.07347 | 2.06529 | | 14.8888 | 14.9325 | 14.8698 | 14.5973 | 14.6537 | 14.8331 | 14.7271 | 14.2129 | 14 | 14 | 14 | 8 | 4 | 10 | 11 | 3 |
| DIPK1A | Q5T7M9 | 5.99E-05 | 1.46E-05 | 0.292432 | | 0.643797 | 1.34546 | | 15.2224 | 15.1995 | 15.2842 | 14.7882 | 14.5273 | 15.4277 | 14.7709 | 15.2005 | 4 | 4 | 5 | 3 | 3 | 3 | 3 | 3 |
| RFTN1 | Q14699 | 3.62E-08 | 1.23E-08 | 0.293195 | | 1.11221 | 2.12979 | | 16.8159 | 16.853 | 16.8064 | 16.8221 | 16.2444 | 16.6153 | 16.3576 | 16.6202 | 13 | 14 | 14 | 15 | 13 | 14 | 14 | 15 |
| CXXC1 | Q9P0U4;Q9 | 6.65E-13 | 5.02E-13 | 0.293407 | | 0.978963 | 1.90824 | | 13.6222 | 13.6866 | 13.7555 | 13.5814 | 13.2258 | 13.6467 | 13.4764 | 13.0432 | 3 | 5 | 3 | 2 | 2 | 2 | 2 | 4 |
| STAT5B | P51692 | 1.91E-10 | 8.80E-11 | 0.293727 | + | 1.77825 | 3.28763 | | 15.0915 | 15.2037 | 15.1664 | 14.8201 | 14.8247 | 15.1101 | 14.7363 | 14.8093 | 14 | 13 | 14 | 14 | 12 | 13 | 13 | 13 |
| MRPS35 | P82673 | 5.59E-07 | 1.67E-07 | 0.293744 | + | 2.11856 | 3.94157 | | 15.6638 | 15.6834 | 15.6829 | 15.5694 | 15.2893 | 15.4118 | 15.2498 | 15.3943 | 14 | 13 | 13 | 10 | 7 | 12 | 11 | 10 |
| ECHDC1 | Q9NTX5;Q9 | 1.79E-09 | 7.16E-10 | 0.293787 | | 0.544241 | 1.17206 | | 17.1061 | 17.0577 | 17.0343 | 16.7297 | 16.8709 | 17.1096 | 17.0762 | 16.0748 | 12 | 13 | 13 | 12 | 12 | 13 | 13 | 10 |
| UQCRQ | O14949 | 5.81E-10 | 2.48E-10 | 0.293887 | | 0.298399 | 0.712274 | | 17.7856 | 17.853 | 17.7231 | 17.8081 | 17.3784 | 16.3549 | 17.7926 | 18.1327 | 3 | 3 | 3 | 3 | 3 | 2 | 3 | 3 |
| HACD4 | Q5VWC8 | 0.003472 | 0.000637 | 0.294312 | | 0.66962 | 1.38973 | | 16.0701 | 15.7992 | 15.5951 | 15.6975 | 15.9526 | 15.5267 | 15.2724 | 15.1865 | 2 | 2 | 2 | 2 | 2 | 2 | 1 | 2 |
| AKR1C2 | P52895 | 0.000126 | 2.95E-05 | 0.294642 | | 0.190872 | 0.485771 | | 14.8204 | 14.6557 | 14.7332 | 13.6423 | 14.2383 | 15.6706 | 13.3519 | 15.306 | 2 | 2 | 2 | 1 | 1 | 2 | 1 | 2 |
| SYF2 | O95926 | 0.005361 | 0.000953 | 0.295145 | + | 1.55979 | 3.07725 | | 14.6235 | 14.6352 | 14.4278 | 14.4374 | 14.3021 | 14.1762 | NaN | 14.1522 | 2 | 2 | 2 | 1 | 1 | 1 | NaN | 1 |
| COQ8B | Q96D53;Q9 | 0.000463 | 0.0001 | 0.295317 | | 1.03244 | 1.99707 | | 14.0998 | 14.1663 | 14.0278 | 13.7114 | 14.1908 | 13.644 | 13.5875 | 13.8796 | 3 | 2 | 4 | 2 | 2 | 1 | 2 | 2 |
| SART3 | Q15020 | 1.79E-17 | 5.26E-17 | 0.295875 | + | 3.66007 | 7.8962 | | 16.3951 | 16.4401 | 16.4198 | 16.0656 | 16.1736 | 16.1856 | 16.1343 | 16.0532 | 30 | 30 | 30 | 29 | 24 | 30 | 26 | 27 |
| VKORC1L1 | Q8N0U8 | 2.50E-06 | 6.98E-07 | 0.296119 | | 1.16574 | 2.21918 | | 18.4619 | 18.493 | 18.3199 | 18.4012 | 17.8783 | 17.9494 | 18.2365 | 18.1788 | 4 | 4 | 4 | 4 | 4 | 4 | 4 | 4 |
| INTS3 | Q68E01;Q6 | 2.04E-16 | 4.36E-16 | 0.296545 | + | 2.32479 | 4.36711 | | 15.5328 | 15.579 | 15.5525 | 15.3191 | 15.3765 | 15.2468 | 15.2714 | 15.0771 | 18 | 16 | 17 | 12 | 13 | 13 | 12 | 11 |
| PRPF31 | Q8WWY3 | 2.79E-12 | 1.80E-12 | 0.296696 | + | 2.99811 | 5.95387 | | 16.481 | 16.5027 | 16.4597 | 16.0915 | 16.1546 | 16.2637 | 16.2777 | 16.1344 | 17 | 16 | 16 | 16 | 16 | 16 | 16 | 16 |
| THAP11 | Q96EK4 | 0.000167 | 3.85E-05 | 0.296868 | | 0.810381 | 1.62754 | | 15.4836 | 15.6244 | 15.5587 | 15.4637 | 15.347 | 15.3352 | 14.7272 | 15.4204 | 2 | 2 | 2 | 2 | 2 | 2 | 2 | 2 |
| YARS1 | P54577 | 1.87E-14 | 2.19E-14 | 0.297342 | | 0.533674 | 1.15335 | | 18.5078 | 18.4881 | 18.4595 | 17.8292 | 17.9176 | 18.805 | 18.4799 | 17.9073 | 46 | 46 | 47 | 43 | 45 | 47 | 45 | 44 |
| TIMP2 | P16035 | 4.11E-17 | 1.08E-16 | 0.29781 | + | 2.22434 | 4.15679 | | 15.9746 | 15.894 | 15.8818 | 15.6659 | 15.5637 | 15.4913 | 15.7915 | 15.5826 | 9 | 9 | 9 | 9 | 8 | 8 | 8 | 8 |
| MTA3 | Q9BTC8;Q9 | 6.83E-07 | 2.02E-07 | 0.298391 | | 0.421159 | 0.949181 | | 14.9894 | 14.6757 | 14.7682 | 14.8673 | 13.7332 | 14.7975 | 14.2396 | 14.9259 | 3 | 4 | 3 | 2 | 2 | 2 | 3 | 2 |
| POTEJ | P0CG39 | 4.43E-08 | 1.49E-08 | 0.298416 | + | 2.24839 | 4.20661 | | 19.0885 | 18.9925 | 19.0148 | 18.7502 | 18.6253 | 18.9155 | 18.6539 | 18.7227 | 5 | 5 | 5 | 6 | 6 | 4 | 6 | 5 |
| ACSL3 | O95573 | 2.72E-16 | 5.44E-16 | 0.298451 | + | 2.95274 | 5.83598 | | 18.2012 | 18.2109 | 18.1538 | 17.9471 | 17.982 | 17.8903 | 17.8646 | 17.7668 | 27 | 27 | 27 | 27 | 26 | 27 | 27 | 27 |
| TOR2A | Q5JU69 | 1.41E-09 | 5.72E-10 | 0.29851 | | 0.53958 | 1.16381 | | 14.6723 | 14.5719 | 14.5817 | 14.8859 | 14.5574 | 14.1668 | 14.1819 | 13.7585 | 2 | 2 | 2 | 2 | 1 | 1 | 2 | 2 |
| MANBA | O00462 | 1.11E-05 | 2.89E-06 | 0.298888 | | 0.653716 | 1.3625 | | 15.5686 | 15.4546 | 15.4301 | 15.4474 | 14.785 | 15.131 | 15.6562 | 14.9082 | 14 | 14 | 16 | 15 | 7 | 14 | 15 | 6 |
| IGFBP3 | P17936;P1 | 6.04E-06 | 1.62E-06 | 0.298974 | | 0.163512 | 0.424126 | | 17.706 | 17.6841 | 17.532 | 16.447 | 16.1983 | 17.1532 | 19.1441 | 17.766 | 12 | 12 | 12 | 10 | 8 | 11 | 13 | 13 |
| PPP2R5D | Q14738 | 4.52E-13 | 3.58E-13 | 0.299032 | + | 3.58967 | 7.66791 | | 17.3418 | 17.2972 | 17.3166 | 17.0688 | 16.9613 | 17.1015 | 16.9617 | 17.0043 | 21 | 21 | 21 | 21 | 20 | 20 | 20 | 20 |
| TGFBR1 | P36897 | 3.48E-05 | 8.66E-06 | 0.29952 | | 0.858813 | 1.70844 | | 14.1344 | 14.2752 | 13.9971 | 14.1637 | 13.5309 | 14.0472 | 13.5874 | 13.851 | 4 | 4 | 4 | 5 | 2 | 5 | 4 | 3 |
| GGCT | O75223 | 8.10E-15 | 1.05E-14 | 0.299536 | | 0.975933 | 1.90321 | | 16.4101 | 16.4491 | 16.5619 | 15.9861 | 15.9207 | 16.496 | 16.4011 | 16.0668 | 8 | 8 | 8 | 7 | 8 | 7 | 8 | 7 |
| FAP | Q12884 | 3.01E-20 | 2.32E-19 | 0.299978 | | 0.30238 | 0.720265 | | 17.47 | 17.4924 | 17.4675 | 16.4003 | 16.8062 | 18.1737 | 17.5723 | 16.9307 | 31 | 32 | 33 | 27 | 27 | 34 | 31 | 29 |
| TLK2 | Q86UE8-3 | 1.14E-13 | 1.06E-13 | 0.300269 | | 0.571834 | 1.28542 | | 14.1154 | 13.5798 | 13.6535 | 13.8081 | NaN | NaN | 13.3165 | 13.3234 | 1 | 2 | 2 | 2 | NaN | NaN | 1 | 2 |
| LSS | P48449 | 3.25E-15 | 4.79E-15 | 0.300689 | | 0.845549 | 1.68632 | | 18.2171 | 18.2157 | 18.2013 | 18.2735 | 17.9437 | 17.7879 | 17.4805 | 18.0677 | 29 | 29 | 27 | 28 | 28 | 27 | 27 | 29 |
| TBC1D4 | O60343;O6 | 7.20E-16 | 1.28E-15 | 0.30102 | | 0.57027 | 1.21788 | | 15.9291 | 15.9018 | 15.9166 | 15.9841 | 16.0464 | 15.104 | 15.2948 | 15.6447 | 24 | 25 | 22 | 21 | 16 | 16 | 16 | 18 |
| ACADM | P11310;P1 | 9.12E-08 | 2.95E-08 | 0.301475 | | 1.11941 | 2.14181 | | 17.1431 | 17.194 | 17.1099 | 17.0916 | 16.5548 | 16.966 | 16.643 | 16.9824 | 13 | 12 | 12 | 12 | 11 | 12 | 12 | 12 |
| YWHAQ | P27348 | 8.35E-08 | 2.71E-08 | 0.301628 | | 0.420046 | 0.947108 | | 19.8941 | 19.7079 | 19.932 | 19.9084 | 19.6308 | 18.6521 | 19.5601 | 19.9639 | 16 | 16 | 16 | 15 | 16 | 15 | 14 | 15 |
| TAF12 | Q16514;Q1 | 0.002936 | 0.000549 | 0.301756 | | 0.86676 | 1.77592 | | 14.7181 | 14.8911 | 14.8041 | 14.5479 | NaN | 14.1049 | 14.6079 | 14.75 | 1 | 1 | 1 | 1 | NaN | 1 | 1 | 1 |
| PPP1R21 | Q6ZMI0 | 5.49E-11 | 2.77E-11 | 0.301846 | + | 1.87824 | 3.47418 | | 15.4196 | 15.4465 | 15.391 | 14.9313 | 15.3263 | 15.1399 | 15.1362 | 15.0524 | 17 | 17 | 18 | 15 | 15 | 18 | 15 | 15 |
| SLC2A1 | P11166 | 9.43E-11 | 4.54E-11 | 0.301979 | | 1.21319 | 2.29871 | | 17.9966 | 18.0446 | 18.0311 | 17.4939 | 17.5978 | 17.6043 | 17.9785 | 17.936 | 7 | 7 | 7 | 6 | 6 | 7 | 7 | 7 |
| INTS4 | Q96HW7 | 4.56E-05 | 1.12E-05 | 0.302178 | + | 1.73755 | 3.21291 | | 14.5923 | 14.5202 | 14.5426 | 14.1468 | 14.5177 | 14.1485 | 14.1853 | 14.2494 | 13 | 12 | 10 | 11 | 6 | 8 | 11 | 10 |
| MRPS26 | Q9BYN8 | 0.000359 | 7.88E-05 | 0.303754 | + | 1.30324 | 2.45065 | | 16.6025 | 16.6635 | 16.5888 | 16.5747 | 16.0634 | 16.4511 | 16.1722 | 16.311 | 7 | 7 | 7 | 7 | 5 | 7 | 7 | 6 |
| LARS1 | Q9P2J5 | 2.16E-15 | 3.37E-15 | 0.303771 | | 0.748717 | 1.52397 | | 17.9011 | 17.8887 | 17.8827 | 17.2995 | 17.3929 | 18.0719 | 17.7993 | 17.3718 | 55 | 54 | 56 | 55 | 51 | 55 | 53 | 52 |
| RABGAP1 | Q9Y3P9 | 3.93E-12 | 2.47E-12 | 0.304038 | + | 1.78516 | 3.3004 | | 16.0646 | 16.015 | 16.0176 | 15.7118 | 15.7631 | 15.9386 | 15.5089 | 15.7194 | 32 | 31 | 31 | 29 | 25 | 30 | 30 | 27 |
| MPI | P34949 | 1.13E-10 | 5.36E-11 | 0.30428 | | 1.0658 | 2.05252 | | 16.4593 | 16.4396 | 16.4513 | 15.7172 | 16.1599 | 16.3437 | 16.2713 | 16.2367 | 12 | 12 | 11 | 11 | 10 | 11 | 11 | 12 |

| Gene names | UniProt IDs | Protein Group P-value | Protein Group Q-value | Log2 Difference NUP214 vs Controls (n=5) | p<0.05 | -log10 p-value | T-test Test statistic | NPC Localization | Log2 MS2_quantity_NUP214_1 | Log2 MS2_quantity_NUP214_2 | Log2 MS2_quantity_NUP214_3 | Log2 MS2_quantity_Control_1 | Log2 MS2_quantity_Control_2 | Log2 MS2_quantity_Control_3 | Log2 MS2_quantity_Control_4 | Log2 MS2_quantity_Control_5 | Number Peptides for Quantitation NUP214_1 | Number Peptides for Quantitation NUP214_2 | Number Peptides for Quantitation NUP214_3 | Number Peptides for Quantitation Control_1 | Number Peptides for Quantitation Control_2 | Number Peptides for Quantitation Control_3 | Number Peptides for Quantitation Control_4 | Number Peptides for Quantitation Control_5 |
|---|---|---|---|---|---|---|---|---|---|---|---|---|---|---|---|---|---|---|---|---|---|---|---|---|
| SCP2 | P22307 | 6.40E-19 | 2.96E-18 | 0.30484 | + | 1.74515 | 3.22682 | | 17.8705 | 17.8991 | 17.8722 | 17.7217 | 17.3591 | 17.7165 | 17.4723 | 17.6091 | 26 | 26 | 25 | 26 | 25 | 25 | 25 | 25 |
| PRKCSH | P14314-2 | 2.70E-06 | 7.50E-07 | 0.304952 | | 0.419396 | 0.945898 | | 19.0193 | 19.0768 | 19.0897 | 18.8898 | 17.8518 | 19.2136 | 19.1036 | 18.7261 | 24 | 25 | 24 | 24 | 23 | 25 | 25 | 25 |
| MIOS | Q9NXC5 | 1.54E-05 | 3.97E-06 | 0.304983 | | 0.822557 | 1.64791 | | 15.3483 | 15.4257 | 15.4479 | 14.816 | 14.7916 | 15.4159 | 15.4213 | 15.0669 | 17 | 13 | 15 | 15 | 12 | 16 | 19 | 15 |
| VPS26B | Q4G0F5 | 1.12E-09 | 4.60E-10 | 0.30504 | + | 1.61133 | 2.98519 | | 15.9902 | 16.0776 | 15.99 | 15.74 | 15.4939 | 15.8734 | 15.8682 | 15.5956 | 12 | 12 | 12 | 12 | 9 | 12 | 13 | 10 |
| IPO7 | O95373 | 1.87E-16 | 4.06E-16 | 0.305306 | | 1.07717 | 2.07144 | | 18.206 | 18.2062 | 18.2127 | 18.1907 | 17.5952 | 18.0234 | 17.699 | 18.0067 | 30 | 32 | 31 | 31 | 29 | 30 | 31 | 32 |
| MBD3 | O95983;O9 | 2.45E-12 | 1.61E-12 | 0.30593 | | 0.445196 | 0.993652 | | 14.5017 | 14.3479 | 14.4995 | 14.57 | 14.1469 | 13.404 | 13.929 | 14.6689 | 5 | 5 | 5 | 4 | 4 | 4 | 3 | 4 |
| MAP4K4 | O95819-6 | 1.50E-17 | 4.51E-17 | 0.306126 | | 0.723707 | 1.48171 | | 17.0301 | 17.0113 | 17.0497 | 17.2293 | 16.7706 | 16.7868 | 16.2951 | 16.5395 | 35 | 32 | 33 | 35 | 30 | 33 | 30 | 32 |
| ASNS | P08243;P0 | 3.39E-13 | 2.79E-13 | 0.306842 | | 0.321016 | 0.757363 | | 18.0273 | 18.0646 | 18.0621 | 17.2391 | 17.2563 | 18.7609 | 18.1321 | 17.334 | 28 | 28 | 28 | 25 | 26 | 28 | 27 | 25 |
| ADAR | P55265;P5 | 2.40E-11 | 1.30E-11 | 0.306881 | + | 4.50666 | 11.1435 | | 16.3937 | 16.3945 | 16.4062 | 16.0374 | 16.1138 | 16.1514 | 16.0546 | 16.0991 | 36 | 37 | 35 | 36 | 33 | 34 | 34 | 35 |
| ERI3 | O43414;O4 | 3.57E-10 | 1.56E-10 | 0.30724 | | 1.18511 | 2.25162 | | 17.1946 | 17.2067 | 17.0692 | 16.6256 | 16.7128 | 17.1515 | 17.0134 | 16.7447 | 5 | 5 | 5 | 5 | 5 | 5 | 4 | 4 |
| MRPL17 | Q9NRX2 | 0.001241 | 0.000253 | 0.307259 | | 1.20815 | 2.29026 | | 16.4453 | 16.397 | 16.3692 | 16.3176 | 16.0626 | 15.8024 | 15.9811 | 16.3194 | 7 | 7 | 7 | 6 | 6 | 7 | 7 | 6 |
| ATP5MD | Q96IX5 | 7.52E-13 | 5.61E-13 | 0.30827 | | 0.403256 | 0.915706 | | 18.3897 | 18.1492 | 18.2667 | 18.5769 | 17.9719 | 17.296 | 17.5179 | 18.4386 | 3 | 3 | 3 | 3 | 3 | 3 | 3 | 3 |
| PMVK | Q15126 | 1.51E-11 | 8.50E-12 | 0.308391 | + | 2.73363 | 5.29048 | | 17.0815 | 17.1635 | 17.1821 | 16.9135 | 16.938 | 16.7548 | 16.7392 | 16.8243 | 12 | 13 | 12 | 11 | 12 | 12 | 11 | 12 |
| RING1 | Q06587 | 6.45E-10 | 2.73E-10 | 0.308573 | + | 2.87725 | 5.64366 | | 15.8459 | 15.7472 | 15.7523 | 15.5527 | 15.5555 | 15.4145 | 15.4748 | 15.3687 | 8 | 8 | 8 | 8 | 6 | 8 | 7 | 7 |
| SMC3 | Q9UQE7 | 3.35E-20 | 2.54E-19 | 0.308639 | + | 3.03485 | 6.05064 | | 15.9817 | 16.0171 | 15.9419 | 15.7568 | 15.626 | 15.5685 | 15.6563 | 15.7506 | 46 | 48 | 47 | 43 | 34 | 41 | 39 | 41 |
| MRPS31 | Q92665 | 1.40E-09 | 5.69E-10 | 0.308892 | + | 1.9521 | 3.61483 | | 15.8562 | 15.7931 | 15.7808 | 15.6637 | 15.3833 | 15.6452 | 15.4061 | 15.4074 | 9 | 9 | 9 | 9 | 8 | 8 | 8 | 8 |
| INPP1 | P49441 | 1.14E-13 | 1.06E-13 | 0.308917 | | 1.02304 | 1.98146 | | 16.0124 | 16.0536 | 16.0674 | 15.5363 | 15.5557 | 15.8937 | 16.1202 | 15.5718 | 8 | 8 | 8 | 6 | 5 | 6 | 5 | 7 |
| THOC7 | Q6I9Y2 | 3.66E-09 | 1.41E-09 | 0.309165 | | 1.0814 | 2.07849 | | 14.8464 | 14.9451 | 14.894 | 15.0096 | 14.4047 | 14.4275 | 14.5047 | 14.5834 | 6 | 6 | 6 | 6 | 5 | 6 | 3 | 5 |
| HDAC1 | Q13547 | 9.73E-13 | 7.05E-13 | 0.309503 | | 1.08132 | 2.07835 | | 16.7111 | 16.7866 | 16.7314 | 16.7942 | 16.2583 | 16.3585 | 16.1861 | 16.5706 | 10 | 11 | 11 | 12 | 10 | 11 | 11 | 11 |
| HCFC1 | P51610;P5 | 8.28E-12 | 4.89E-12 | 0.310056 | + | 2.18706 | 4.08024 | | 16.3859 | 16.3545 | 16.3143 | 16.1393 | 16.1178 | 15.9406 | 15.8743 | 16.1357 | 31 | 31 | 32 | 30 | 28 | 29 | 29 | 32 |
| COQ5 | Q5HYK3 | 0.000133 | 3.11E-05 | 0.310271 | + | 1.42733 | 2.66283 | | 14.2694 | 14.4873 | 14.2375 | 14.1168 | 14.2417 | 13.9897 | 13.9696 | 13.788 | 4 | 3 | 4 | 3 | 2 | 3 | 2 | 3 |
| FYN | P06241 | 0.001242 | 0.000253 | 0.310305 | | 0.322623 | 0.760538 | | 14.1457 | 14.1576 | 14.394 | 14.3313 | 13.2986 | 14.8423 | 13.2679 | 13.8703 | 5 | 4 | 3 | 5 | 2 | 3 | 2 | 3 |
| SEMA3C | Q99985 | 0.000181 | 4.14E-05 | 0.310969 | | 0.358503 | 0.842337 | | 14.5727 | 14.5574 | 14.5634 | 13.4557 | NaN | 14.9565 | 14.1728 | 14.4293 | 11 | 14 | 14 | 2 | NaN | 13 | 6 | 9 |
| AGPS | O00116 | 1.69E-12 | 1.15E-12 | 0.311418 | + | 2.46973 | 4.68153 | | 17.2402 | 17.2239 | 17.2373 | 16.9375 | 16.8416 | 17.0364 | 17.0179 | 16.7783 | 22 | 22 | 22 | 20 | 19 | 22 | 19 | 19 |
| TBC1D2 | Q9BYX2 | 1.03E-09 | 4.28E-10 | 0.311537 | | 0.411785 | 0.931692 | | 15.8784 | 15.8928 | 15.9132 | 14.6667 | 15.727 | 15.7739 | 16.1831 | 15.5656 | 17 | 16 | 17 | 13 | 10 | 15 | 15 | 15 |
| ANK2 | Q01484;Q0 | 5.36E-06 | 1.44E-06 | 0.311646 | + | 1.39619 | 2.60923 | | 15.3751 | 15.484 | 15.5276 | 15.0277 | 14.9329 | 15.4203 | 15.1138 | 15.2581 | 22 | 22 | 22 | 16 | 11 | 17 | 13 | 18 |
| COPS3 | Q9UNS2 | 2.72E-10 | 1.22E-10 | 0.312676 | + | 1.50801 | 2.80291 | | 16.8231 | 16.8253 | 16.8036 | 16.577 | 16.2669 | 16.7656 | 16.4053 | 16.5085 | 15 | 14 | 13 | 14 | 13 | 14 | 13 | 13 |
| PDRG1 | Q9NUG6 | 9.57E-05 | 2.27E-05 | 0.312813 | + | 1.45792 | 2.71572 | | 15.4011 | 15.4344 | 15.3467 | 15.3026 | 15.031 | 14.7892 | 15.1172 | 15.1663 | 4 | 4 | 4 | 4 | 2 | 1 | 3 | 3 |
| PCYT2 | Q99447;Q9 | 3.18E-08 | 1.09E-08 | 0.313529 | | 1.28092 | 2.41285 | | 17.165 | 17.052 | 17.1104 | 17.1375 | 16.8668 | 16.603 | 16.6756 | 16.695 | 15 | 16 | 16 | 15 | 13 | 14 | 15 | 14 |
| ITPRIPL2 | Q3MIP1 | 4.26E-16 | 8.03E-16 | 0.314312 | + | 1.42442 | 2.65781 | | 14.9867 | 14.9069 | 14.8956 | 14.652 | 14.3399 | 14.7725 | 14.8098 | 14.5029 | 5 | 5 | 6 | 4 | 3 | 5 | 4 | 3 |
| ELP3 | Q9H9T3;Q9 | 1.34E-11 | 7.60E-12 | 0.314498 | + | 1.58567 | 2.93959 | | 16.0649 | 16.117 | 16.1083 | 15.5144 | 15.9622 | 15.7328 | 15.9258 | 15.7761 | 17 | 16 | 17 | 14 | 15 | 15 | 14 | 14 |
| EBP | Q15125 | 2.81E-07 | 8.71E-08 | 0.314613 | | 0.454644 | 1.01099 | | 17.784 | 17.8661 | 18.3428 | 18.333 | 17.0416 | 17.6094 | 17.5339 | 17.897 | 2 | 2 | 2 | 2 | 2 | 2 | 2 | 2 |
| TGFB1I1 | O43294;O4 | 7.16E-10 | 3.01E-10 | 0.314843 | + | 1.87271 | 3.46376 | | 18.2491 | 18.3078 | 18.262 | 17.8972 | 17.9706 | 18.2073 | 17.808 | 17.9076 | 10 | 9 | 8 | 7 | 9 | 10 | 10 | 9 |
| ZDHHC20 | Q5W0Z9;Q | 1.61E-06 | 4.56E-07 | 0.315019 | | 0.991553 | 1.92915 | | 15.7612 | 15.9393 | 15.7992 | 15.7549 | 15.3808 | 15.1225 | 15.7254 | 15.6075 | 3 | 3 | 3 | 4 | 2 | 3 | 4 | 3 |
| CLCN6 | P51797;P5 | 7.53E-06 | 2.00E-06 | 0.316189 | | 1.09447 | 2.10024 | | 15.5679 | 15.5658 | 15.402 | 15.1127 | 15.3406 | 15.0796 | 14.9113 | 15.5342 | 5 | 6 | 5 | 4 | 3 | 3 | 2 | 3 |
| ACAD8 | Q9UKU7 | 7.06E-09 | 2.61E-09 | 0.317851 | | 1.20045 | 2.27732 | | 14.9142 | 14.9842 | 14.9585 | 14.6395 | 14.7817 | 14.8665 | 14.2589 | 14.6256 | 6 | 6 | 6 | 6 | 2 | 4 | 5 | 4 |
| FOCAD | Q5VW36 | 2.02E-10 | 9.24E-11 | 0.318011 | + | 2.35102 | 4.42303 | | 15.6429 | 15.6465 | 15.5981 | 15.3596 | 15.3743 | 15.3462 | 15.3764 | 15.0993 | 33 | 33 | 34 | 30 | 25 | 31 | 29 | 27 |
| RNH1 | P13489 | 1.34E-10 | 6.32E-11 | 0.31809 | | 1.28338 | 2.41702 | | 21.2081 | 21.1644 | 21.1807 | 21.0015 | 20.5471 | 21.1148 | 20.9044 | 20.7638 | 26 | 25 | 26 | 26 | 25 | 26 | 26 | 26 |
| SGCB | Q16585 | 1.09E-05 | 2.85E-06 | 0.318214 | | 0.831662 | 1.66313 | | 16.5551 | 16.8341 | 16.7414 | 16.072 | 16.3328 | 16.8829 | 16.432 | 16.2402 | 10 | 10 | 10 | 9 | 10 | 10 | 9 | 10 |
| SON | P18583;P1 | 3.58E-11 | 1.87E-11 | 0.318362 | | 1.02599 | 1.98635 | | 15.6021 | 15.6698 | 15.7104 | 15.5844 | 14.8989 | 15.4696 | 15.3131 | 15.446 | 35 | 34 | 33 | 30 | 18 | 28 | 31 | 30 |
| SFT2D2 | O95562 | 1.02E-12 | 7.37E-13 | 0.318523 | | 0.647605 | 1.35201 | | 16.7876 | 16.7272 | 16.8145 | 17.1341 | 16.1161 | 16.4168 | 16.2824 | 16.3403 | 2 | 2 | 1 | 1 | 1 | 2 | 1 | 1 |
| NFKB1 | P19838;P1 | 3.89E-13 | 3.15E-13 | 0.318535 | + | 1.71052 | 3.16364 | | 15.8535 | 15.8787 | 15.8247 | 15.5149 | 15.3242 | 15.7698 | 15.4507 | 15.6092 | 27 | 27 | 27 | 24 | 23 | 26 | 23 | 27 |
| NT5DC3 | Q86UY8;Q8 | 3.92E-07 | 1.20E-07 | 0.318546 | | 0.720542 | 1.47635 | | 15.7491 | 15.7652 | 15.7925 | 15.1115 | 15.9961 | 15.1487 | 15.4121 | 15.5832 | 8 | 10 | 9 | 8 | 8 | 8 | 9 | 9 |
| MYO1E | Q12965 | 3.93E-16 | 7.44E-16 | 0.318954 | | 1.06526 | 2.05162 | | 16.4667 | 16.5183 | 16.5265 | 16.4635 | 16.1588 | 16.4199 | 15.8477 | 16.0346 | 36 | 37 | 37 | 36 | 29 | 35 | 32 | 32 |
| PPID | Q08752 | 3.30E-12 | 2.11E-12 | 0.319139 | + | 2.7899 | 5.42692 | | 16.6327 | 16.6153 | 16.6054 | 16.3559 | 16.2192 | 16.3686 | 16.3818 | 16.1678 | 15 | 18 | 15 | 17 | 16 | 16 | 17 | 15 |
| LANCL1 | O43813 | 7.41E-14 | 7.33E-14 | 0.319187 | | 1.0594 | 2.04188 | | 17.816 | 17.9671 | 17.8184 | 17.186 | 17.7072 | 17.8466 | 17.57 | 17.4301 | 11 | 11 | 11 | 11 | 11 | 11 | 11 | 11 |
| NASP | P49321 | 8.31E-21 | 7.58E-20 | 0.319714 | + | 1.46164 | 2.72218 | | 16.4524 | 16.4692 | 16.4738 | 16.2557 | 16.2544 | 15.8143 | 16.1148 | 16.2879 | 22 | 23 | 23 | 23 | 19 | 19 | 23 | 23 |
| FARP2 | O94887 | 1.46E-08 | 5.19E-09 | 0.31989 | + | 1.55249 | 2.88095 | | 14.1074 | 14.0876 | 13.9596 | 13.5277 | 13.7406 | 13.9584 | 13.8426 | 13.5889 | 10 | 9 | 10 | 7 | 6 | 6 | 6 | 6 |
| INTS6 | Q9UL03;Q9 | 3.84E-05 | 9.52E-06 | 0.319966 | + | 2.16015 | 4.02546 | | 14.4385 | 14.407 | 14.3891 | 14.1387 | 14.2045 | 13.865 | 14.1504 | 14.0992 | 7 | 5 | 5 | 6 | 3 | 6 | 3 | 4 |
| RPA1 | P27694 | 1.07E-09 | 4.42E-10 | 0.320033 | + | 1.60567 | 2.97511 | | 16.3398 | 16.3463 | 16.3701 | 16.13 | 15.8385 | 15.9079 | 15.995 | 16.2886 | 23 | 24 | 22 | 22 | 19 | 18 | 21 | 19 |
| UBE2E2 | Q96LR5 | 2.79E-07 | 8.65E-08 | 0.320038 | | 0.719629 | 1.47481 | | 17.3883 | 17.3886 | 17.2652 | 17.5258 | 16.5423 | 16.9031 | 16.998 | 17.1674 | 5 | 5 | 5 | 5 | 4 | 4 | 5 | 4 |
| EP400 | Q96L91;Q9 | 8.02E-05 | 1.92E-05 | 0.32014 | + | 1.41585 | 2.64304 | | 14.6192 | 14.6019 | 14.5319 | 14.1631 | 14.6185 | 14.1788 | 14.1373 | 14.2233 | 5 | 6 | 5 | 5 | 4 | 3 | 3 | 4 |
| AKR1A1 | P14550 | 2.06E-13 | 1.80E-13 | 0.320493 | | 0.791594 | 1.59606 | | 18.7375 | 18.7214 | 18.7124 | 18.1347 | 18.3039 | 18.5632 | 18.9098 | 18.1048 | 18 | 17 | 17 | 16 | 16 | 17 | 18 | 18 |
| CAT | P04040 | 2.89E-20 | 2.24E-19 | 0.320674 | | 0.646984 | 1.35094 | | 17.3692 | 17.396 | 17.332 | 17.3489 | 17.313 | 16.8821 | 16.4178 | 17.2635 | 24 | 24 | 24 | 24 | 21 | 22 | 20 | 23 |
| SP100 | P23497 | 4.46E-14 | 4.68E-14 | 0.320867 | + | 1.58952 | 2.94642 | | 17.8625 | 17.8616 | 17.8457 | 17.795 | 17.5069 | 17.6079 | 17.3002 | 17.4688 | 18 | 18 | 19 | 17 | 17 | 18 | 18 | 18 |
| SNTB1 | Q13884 | 6.04E-13 | 4.61E-13 | 0.321107 | | 0.586622 | 1.24647 | | 15.9405 | 15.9415 | 15.9471 | 16.0771 | 15.7393 | 15.6032 | 15.7762 | 14.9138 | 11 | 12 | 11 | 13 | 12 | 12 | 11 | 8 |
| PIP5K1C | O60331;O6 | 1.49E-10 | 6.99E-11 | 0.321568 | | 0.890298 | 1.76089 | | 16.0411 | 16.0571 | 16.0347 | 15.815 | 15.2416 | 16.0405 | 15.633 | 15.8836 | 8 | 8 | 7 | 6 | 5 | 8 | 6 | 8 |
| LSM8 | O95777 | 5.15E-06 | 1.39E-06 | 0.322212 | | 0.35317 | 0.820244 | | 17.394 | 17.2286 | 17.4614 | 17.5112 | 16.4196 | 16.3092 | 17.1753 | 17.7803 | 3 | 3 | 3 | 3 | 3 | 4 | 3 | 3 |
| ESYT1 | Q9BSJ8 | 3.52E-13 | 2.88E-13 | 0.322814 | + | 1.48506 | 2.76287 | | 19.2723 | 19.2937 | 19.282 | 19.0594 | 19.1723 | 18.6493 | 18.9288 | 18.9895 | 56 | 56 | 55 | 56 | 55 | 56 | 55 | 55 |
| NAGA | P17050 | 4.23E-18 | 1.54E-17 | 0.323038 | | 0.566159 | 1.21067 | | 17.0944 | 17.0691 | 17.0046 | 16.0022 | 16.8572 | 16.9651 | 17.1684 | 16.672 | 9 | 9 | 9 | 9 | 8 | 9 | 9 | 8 |
| CUL3 | Q13618 | 8.72E-11 | 4.22E-11 | 0.32308 | + | 2.45659 | 4.65247 | | 16.397 | 16.3792 | 16.3804 | 16.0063 | 15.9152 | 16.2162 | 16.1339 | 16.0406 | 39 | 40 | 36 | 39 | 32 | 36 | 34 | 35 |
| RECQL | P46063 | 1.35E-18 | 5.74E-18 | 0.323192 | + | 3.52503 | 7.46319 | | 17.5334 | 17.5869 | 17.5571 | 17.2202 | 17.1454 | 17.283 | 17.3258 | 17.2054 | 27 | 27 | 27 | 27 | 27 | 27 | 27 | 27 |
| HNRNPD | Q14103;Q1 | 1.90E-08 | 6.66E-09 | 0.323283 | + | 2.60649 | 4.99092 | | 19.271 | 19.3105 | 19.2882 | 18.9243 | 19.0761 | 19.046 | 18.8051 | 18.9817 | 18 | 18 | 18 | 18 | 18 | 18 | 18 | 18 |
| IFT80 | Q9P2H3-3 | 2.56E-24 | 7.58E-23 | 0.323405 | | 0.656105 | 1.36659 | | 13.909 | 13.9793 | 13.8577 | 13.3413 | 13.0824 | 13.9867 | 13.9716 | 13.5775 | 6 | 6 | 5 | 5 | 2 | 4 | 5 | 3 |
| KDM5A | P29375;P2 | 0.000399 | 8.71E-05 | 0.323421 | | 0.949356 | 1.85907 | | 13.4065 | 13.1098 | 13.44 | 12.8945 | 12.996 | 12.705 | 13.4183 | 12.963 | 1 | 2 | 2 | 1 | 1 | 1 | 1 | 1 |
| BAX | Q07812;Q0 | 3.11E-13 | 2.59E-13 | 0.32492 | + | 2.3434 | 4.40674 | | 18.4674 | 18.3836 | 18.4703 | 18.0156 | 18.0152 | 18.059 | 18.2512 | 18.2366 | 12 | 12 | 12 | 11 | 12 | 12 | 12 | 12 |
| FUS | P35637;P3 | 3.01E-15 | 4.46E-15 | 0.324921 | | 1.23083 | 2.32836 | | 18.8341 | 18.8372 | 18.7346 | 18.7607 | 18.2097 | 18.5836 | 18.2717 | 18.5596 | 8 | 8 | 8 | 8 | 8 | 8 | 8 | 8 |
| VPS36 | Q86VN1 | 4.58E-13 | 3.62E-13 | 0.325032 | | 1.00408 | 1.94996 | | 16.0234 | 16.056 | 16.0826 | 15.7531 | 15.3551 | 16.0175 | 15.9661 | 15.553 | 16 | 15 | 16 | 15 | 14 | 16 | 14 | 14 |
| SH3PXD2B | A1X283 | 5.85E-13 | 4.49E-13 | 0.32584 | | 0.766183 | 1.55338 | | 16.6406 | 16.6961 | 16.7042 | 16.7853 | 16.3717 | 16.358 | 15.8087 | 16.4486 | 28 | 27 | 28 | 29 | 23 | 24 | 22 | 27 |
| EYA3 | Q99504;Q9 | 3.99E-06 | 1.09E-06 | 0.326729 | | 0.350252 | 0.814592 | | 13.1115 | 12.7788 | 12.7797 | 13.6056 | 12.0394 | 12.4902 | 11.9726 | 12.7087 | 1 | 2 | 2 | 2 | 3 | 1 | 3 | 2 |
| ZADH2 | Q8N4Q0 | 0.000699 | 0.000147 | 0.327096 | | 0.778323 | 1.57379 | | 16.3033 | 16.2828 | 16.2474 | 15.5005 | 16.1881 | 15.84 | 16.396 | 15.8292 | 3 | 3 | 3 | 1 | 2 | 1 | 4 | 1 |
| PDHA1 | P08559;P0 | 6.86E-08 | 2.25E-08 | 0.327253 | + | 1.52089 | 2.82544 | | 18.2184 | 18.2405 | 18.2671 | 18.0885 | 17.7223 | 18.0587 | 17.6879 | 18.0164 | 19 | 19 | 18 | 19 | 19 | 19 | 19 | 19 |
| RAP1A | P62834 | 1.70E-09 | 6.80E-10 | 0.327292 | | 0.535136 | 1.15594 | | 17.2202 | 17.3406 | 17.1366 | 16.9652 | 16.3338 | 17.6047 | 16.959 | 16.6633 | 4 | 4 | 4 | 4 | 4 | 4 | 4 | 4 |
| PSME2 | Q9UL46 | 7.99E-08 | 2.60E-08 | 0.327371 | | 0.560948 | 1.20151 | | 18.0241 | 18.0079 | 18.0314 | 17.8164 | 17.8062 | 16.9246 | 17.7698 | 18.1517 | 15 | 15 | 16 | 15 | 14 | 13 | 13 | 15 |
| RABL2A;RABL2A;RABI | Q9UBK7;Q9 | 9.00E-07 | 2.63E-07 | 0.327924 | | 1.16492 | 2.21782 | | 15.2681 | 15.5157 | 15.5683 | 15.3504 | 15.1629 | 15.2243 | 15.115 | 14.7612 | 5 | 5 | 5 | 4 | 5 | 4 | 4 | 4 |
| FEN1 | P39748 | 3.11E-08 | 1.07E-08 | 0.328194 | | 0.827235 | 1.65573 | | 16.2859 | 16.3374 | 16.3754 | 16.4039 | 15.9261 | 15.5534 | 15.8965 | 16.2435 | 10 | 12 | 12 | 13 | 11 | 9 | 12 | 12 |
| DARS2 | Q6PI48 | 2.00E-10 | 9.16E-11 | 0.328408 | + | 2.16575 | 4.03682 | | 16.1002 | 16.0863 | 16.0758 | 15.8289 | 15.7862 | 15.8869 | 15.5307 | 15.7625 | 16 | 17 | 18 | 16 | 11 | 14 | 14 | 15 |
| CHMP6 | Q96FZ7 | 2.65E-13 | 2.25E-13 | 0.328421 | | 0.448235 | 0.999237 | | 16.6264 | 16.4302 | 16.5564 | 16.2131 | 16.048 | 15.3841 | 16.7545 | 16.6466 | 6 | 6 | 6 | 6 | 4 | 5 | 5 | 6 |

| Gene names | UniProt IDs | Protein Group P-value | Protein Group Q-value | Log2 Difference NUP214 vs Controls (n=5) | p<0.05 | -log10 p-value | T-test Test statistic | NPC Localization | Log2 MS2_quantity_NUP214_1 | Log2 MS2_quantity_NUP214_2 | Log2 MS2_quantity_NUP214_3 | Log2 MS2_quantity_Control_1 | Log2 MS2_quantity_Control_2 | Log2 MS2_quantity_Control_3 | Log2 MS2_quantity_Control_4 | Log2 MS2_quantity_Control_5 | Number Peptides for Quantitation NUP214_1 | Number Peptides for Quantitation NUP214_2 | Number Peptides for Quantitation NUP214_3 | Number Peptides for Quantitation Control_1 | Number Peptides for Quantitation Control_2 | Number Peptides for Quantitation Control_3 | Number Peptides for Quantitation Control_4 | Number Peptides for Quantitation Control_5 |
|---|---|---|---|---|---|---|---|---|---|---|---|---|---|---|---|---|---|---|---|---|---|---|---|---|
| BDH2 | Q9BUT1 | 1.68E-09 | 6.73E-10 | 0.32862 | + | 2.5207 | 4.79533 | | 17.3511 | 17.2772 | 17.2364 | 16.9004 | 17.0914 | 16.9303 | 17.0462 | 16.8298 | 10 | 10 | 9 | 8 | 7 | 8 | 9 | 9 |
| TNKS1BP1 | Q9C0C2 | 6.47E-18 | 2.22E-17 | 0.328998 | + | 1.58129 | 2.93182 | | 17.5494 | 17.5494 | 17.5829 | 17.0359 | 17.2261 | 17.5373 | 17.2222 | 17.1365 | 63 | 66 | 66 | 64 | 59 | 62 | 64 | 59 |
| AKAP2 | Q9Y2D5-6 | 1.90E-14 | 2.22E-14 | 0.329667 | | 0.767798 | 1.5561 | | 17.7575 | 17.7735 | 17.7865 | 17.8987 | 17.4218 | 17.4295 | 16.9102 | 17.554 | 42 | 46 | 44 | 41 | 38 | 46 | 42 | 39 |
| VTA1 | Q9NP79 | 2.86E-07 | 8.85E-08 | 0.330003 | + | 1.77945 | 3.28985 | | 18.027 | 18.0493 | 18.0349 | 17.9719 | 17.525 | 17.6311 | 17.6597 | 17.7478 | 10 | 10 | 10 | 11 | 10 | 10 | 10 | 10 |
| SP1 | P08047;P0 | 0.001999 | 0.000392 | 0.330083 | | 0.299337 | 0.723216 | | 12.2608 | 12.3632 | 12.3714 | 12.6064 | 10.8817 | NaN | 12.1475 | 12.3712 | 3 | 2 | 2 | 2 | 2 | NaN | 2 | 2 |
| GGA2 | Q9UJY4 | 0.000299 | 6.65E-05 | 0.330331 | | 0.260909 | 0.635772 | | 13.8047 | 13.9343 | 13.7542 | 14.4113 | 12.0569 | 13.6474 | 13.7636 | 13.6244 | 4 | 5 | 4 | 4 | 2 | 3 | 3 | 3 |
| UBA3 | Q8TBC4 | 9.10E-13 | 6.66E-13 | 0.330423 | + | 3.03332 | 6.04657 | | 16.9665 | 16.994 | 16.9879 | 16.5939 | 16.6743 | 16.7904 | 16.5512 | 16.6521 | 17 | 16 | 15 | 15 | 15 | 15 | 16 | 16 |
| PPP5C | P53041 | 2.84E-12 | 1.84E-12 | 0.33055 | + | 4.04337 | 9.24432 | | 17.239 | 17.3322 | 17.2856 | 16.9315 | 16.9471 | 16.9928 | 16.8887 | 17.015 | 20 | 21 | 19 | 18 | 17 | 18 | 19 | 17 |
| TBCEL | Q5QJ74 | 1.43E-12 | 9.94E-13 | 0.330959 | | 1.0669 | 2.05436 | | 16.3033 | 16.4011 | 16.3381 | 15.7501 | 15.8704 | 16.3583 | 16.246 | 15.8578 | 9 | 9 | 9 | 8 | 7 | 10 | 9 | 7 |
| HACL1 | Q9UJ83;Q9 | 4.77E-10 | 2.06E-10 | 0.331663 | | 0.892994 | 1.76538 | | 14.8796 | 15.0233 | 15.0163 | 14.2047 | 14.8005 | 15.0288 | 14.6632 | 14.5098 | 6 | 5 | 7 | 3 | 2 | 4 | 4 | 3 |
| P3H3 | Q8IVL6 | 4.77E-09 | 1.81E-09 | 0.332281 | | 1.03236 | 1.99693 | | 18.6953 | 18.7994 | 18.7435 | 18.1806 | 18.3817 | 18.687 | 18.7063 | 18.1131 | 19 | 18 | 19 | 17 | 14 | 18 | 18 | 17 |
| TEAD1 | P28347 | 1.11E-08 | 4.02E-09 | 0.332627 | + | 1.45668 | 2.71357 | | 15.6702 | 15.7662 | 15.6899 | 15.4599 | 15.5939 | 15.1195 | 15.2077 | 15.4996 | 8 | 8 | 8 | 7 | 4 | 5 | 4 | 5 |
| IRF9 | Q00978 | 0.000113 | 2.65E-05 | 0.334068 | + | 1.7811 | 3.2929 | | 15.5667 | 15.4148 | 15.5047 | 15.0551 | 15.3559 | 15.3188 | 15.0389 | 15.038 | 3 | 5 | 5 | 5 | 4 | 4 | 2 | 3 |
| GTF3C3 | Q9Y5Q9 | 8.40E-11 | 4.08E-11 | 0.334442 | + | 2.05856 | 3.82217 | | 14.7704 | 14.6698 | 14.7534 | 14.2084 | 14.3877 | 14.5124 | 14.3192 | 14.5561 | 8 | 10 | 8 | 6 | 3 | 6 | 6 | 5 |
| MIF4GD | A9UHW6 | 0.003467 | 0.000636 | 0.33538 | | 0.476031 | 1.04997 | | 14.5035 | 14.0791 | 14.8918 | 13.5686 | 13.9078 | 14.1011 | 14.528 | 14.675 | 5 | 4 | 6 | 2 | 3 | 3 | 4 | 3 |
| PCBP4 | P57723 | 0.008194 | 0.001421 | 0.336178 | | 0.403038 | 0.989583 | | 14.6217 | 14.9384 | 15.0902 | 14.9353 | 14.1592 | NaN | NaN | NaN | 1 | 2 | 2 | 2 | 2 | NaN | NaN | NaN |
| AKR7A2 | O43488 | 4.92E-07 | 1.49E-07 | 0.336303 | | 1.08444 | 2.08355 | | 19.1087 | 19.116 | 19.151 | 18.3181 | 18.9387 | 18.975 | 18.9021 | 18.8108 | 12 | 12 | 11 | 11 | 12 | 11 | 12 | 12 |
| HNRNPC | P07910-2 | 0.002327 | 0.000449 | 0.337494 | | 0.749448 | 1.5252 | | 17.0992 | 16.8387 | 16.861 | 16.7618 | 16.1839 | 16.8642 | 16.2376 | 16.9299 | 2 | 3 | 3 | 2 | 2 | 3 | 2 | 2 |
| DHRS1 | Q96LJ7 | 4.54E-11 | 2.32E-11 | 0.33789 | | 0.442024 | 0.987812 | | 15.5356 | 15.4391 | 15.4561 | 14.6885 | 15.9905 | 15.124 | 14.5535 | 15.3389 | 8 | 8 | 8 | 6 | 8 | 5 | 8 | 7 |
| MRPL18 | Q9H0U6 | 0.000507 | 0.000109 | 0.338336 | | 1.02736 | 1.98863 | | 16.8801 | 16.869 | 16.9584 | 16.632 | 16.8066 | 16.2156 | 16.3231 | 16.8433 | 3 | 3 | 3 | 3 | 1 | 2 | 3 | 3 |
| RBMS3 | Q6XE24;Q6 | 0.001136 | 0.000233 | 0.338795 | | 0.441787 | 0.987377 | | 17.014 | 16.8983 | 17.0719 | 16.4461 | 17.4017 | 16.6087 | 16.9534 | 15.8699 | 3 | 3 | 3 | 2 | 2 | 3 | 1 | 2 |
| DENND2B | P78524 | 2.76E-08 | 9.52E-09 | 0.340161 | | 0.63271 | 1.35653 | | 15.2164 | 15.0421 | 15.0056 | 14.31 | NaN | 15.1246 | 14.48 | 15.0767 | 6 | 6 | 6 | 2 | NaN | 5 | 2 | 1 |
| EXOSC5 | Q9NQT4 | 3.12E-12 | 2.00E-12 | 0.340234 | | 1.16982 | 2.22602 | | 15.7039 | 15.7737 | 15.6777 | 15.5216 | 15.4735 | 15.0963 | 15.128 | 15.6717 | 5 | 5 | 4 | 5 | 5 | 5 | 5 | 5 |
| SUMF1 | Q8NBK3;Q8 | 7.61E-07 | 2.24E-07 | 0.340368 | | 0.752502 | 1.53034 | | 16.8429 | 16.8341 | 16.7578 | 16.4798 | 16.126 | 16.9611 | 16.6956 | 16.0937 | 5 | 5 | 5 | 5 | 5 | 5 | 5 | 5 |
| AS3MT | Q9HBK9 | 0.0001 | 2.37E-05 | 0.340427 | + | 1.30259 | 2.44956 | | 15.4276 | 15.491 | 15.4338 | 15.0358 | 15.321 | 14.8865 | 15.3898 | 14.9187 | 8 | 8 | 8 | 7 | 7 | 8 | 7 | 4 |
| ECI2 | O75521;O7 | 1.99E-09 | 7.91E-10 | 0.340574 | + | 1.56082 | 2.89565 | | 16.9025 | 16.9233 | 16.9495 | 16.8791 | 16.4007 | 16.623 | 16.4067 | 16.6133 | 17 | 17 | 17 | 17 | 15 | 14 | 16 | 16 |
| NUBPL | Q8TB37 | 0.000144 | 3.35E-05 | 0.341089 | | 0.911814 | 1.79668 | | 15.1377 | 15.36 | 15.1881 | 14.3902 | 15.174 | 15.1008 | 14.9279 | 14.8449 | 5 | 5 | 6 | 2 | 5 | 6 | 4 | 5 |
| TMEM263 | Q8WUH6 | 2.21E-09 | 8.69E-10 | 0.341399 | | 0.885426 | 1.75278 | | 17.2638 | 17.2794 | 17.4918 | 16.942 | 16.7061 | 17.3615 | 17.2991 | 16.7093 | 5 | 5 | 5 | 5 | 5 | 5 | 5 | 5 |
| IFT81 | Q8WYA0 | 5.96E-06 | 1.60E-06 | 0.341659 | | 0.698193 | 1.43844 | | 14.5945 | 14.4942 | 14.5003 | 13.6624 | 14.6541 | 14.424 | 13.9214 | 14.2781 | 4 | 5 | 5 | 2 | 2 | 3 | 2 | 3 |
| HIKESHI | Q53FT3 | 1.42E-09 | 5.75E-10 | 0.34181 | | 0.953788 | 1.86643 | | 16.5632 | 16.6462 | 16.509 | 16.3208 | 15.6938 | 16.4255 | 16.3829 | 16.332 | 5 | 5 | 5 | 4 | 3 | 5 | 4 | 4 |
| DOCK1 | Q14185 | 1.30E-18 | 5.55E-18 | 0.341872 | + | 1.74524 | 3.22698 | | 15.3525 | 15.3926 | 15.391 | 15.119 | 15.1687 | 15.1901 | 14.9241 | 14.7821 | 38 | 41 | 40 | 32 | 26 | 34 | 26 | 21 |
| MRPS12 | O15235 | 5.62E-07 | 1.68E-07 | 0.342266 | + | 2.9449 | 5.81579 | | 16.0937 | 16.2577 | 16.1502 | 15.871 | 15.7919 | 15.9382 | 15.7849 | 15.7387 | 3 | 3 | 3 | 3 | 3 | 3 | 3 | 3 |
| NELFCD | Q8IXH7;Q8 | 1.77E-10 | 8.18E-11 | 0.342932 | + | 2.54317 | 4.84607 | | 15.6466 | 15.7424 | 15.7497 | 15.5279 | 15.4302 | 15.3171 | 15.239 | 15.3356 | 13 | 13 | 12 | 11 | 4 | 9 | 9 | 9 |
| GCAT | O75600;O7 | 5.09E-07 | 1.53E-07 | 0.343093 | | 1.14675 | 2.28238 | | 15.085 | 15.1129 | 15.109 | 14.5803 | NaN | 14.5188 | 14.8809 | 15.0568 | 7 | 7 | 7 | 2 | NaN | 3 | 2 | 2 |
| SMAD2 | Q15796;Q1 | 2.05E-06 | 5.75E-07 | 0.343389 | + | 1.52557 | 2.83364 | | 16.2873 | 16.3182 | 16.1961 | 16.1373 | 15.9714 | 15.8733 | 15.6128 | 16.0242 | 4 | 4 | 4 | 3 | 3 | 4 | 3 | 3 |
| SRP19 | P09132 | 3.78E-08 | 1.28E-08 | 0.343659 | + | 2.07901 | 3.86265 | | 17.0229 | 17.036 | 16.937 | 16.5154 | 16.5013 | 16.831 | 16.6795 | 16.7476 | 3 | 4 | 4 | 4 | 4 | 4 | 4 | 3 |
| LZTFL1 | Q9NQ48 | 2.30E-08 | 8.00E-09 | 0.344094 | | 0.896246 | 1.77079 | | 15.4747 | 15.5084 | 15.5715 | 14.6965 | 15.1067 | 15.3269 | 15.5788 | 15.1618 | 11 | 11 | 10 | 9 | 11 | 10 | 11 | 11 |
| MYG1 | Q9HB07 | 1.59E-14 | 1.90E-14 | 0.344286 | + | 2.29969 | 4.314 | | 17.84 | 17.8812 | 17.844 | 17.524 | 17.3525 | 17.708 | 17.5354 | 17.434 | 11 | 12 | 12 | 11 | 11 | 11 | 11 | 11 |
| FYCO1 | Q9BQS8;Q9 | 2.21E-14 | 2.53E-14 | 0.344709 | + | 2.03094 | 3.76784 | | 16.3497 | 16.3419 | 16.3381 | 16.2227 | 16.0581 | 15.8635 | 15.9981 | 15.8501 | 38 | 37 | 36 | 36 | 31 | 31 | 34 | 29 |
| C1RL | Q9NZP8 | 0.028343 | 0.004666 | 0.344755 | + | 1.54996 | 3.36451 | | 13.908 | 13.5845 | 13.62 | 13.3564 | 13.3586 | 13.3632 | NaN | NaN | 1 | 2 | 2 | 2 | 1 | 2 | NaN | NaN |
| ASMTL | O95671 | 9.11E-22 | 1.08E-20 | 0.345343 | | 0.561319 | 1.20217 | | 15.7936 | 15.8466 | 15.7619 | 15.5637 | 15.5847 | 15.5944 | 15.9019 | 14.6322 | 16 | 18 | 16 | 17 | 16 | 16 | 18 | 13 |
| THOC3 | Q96J01 | 3.57E-06 | 9.81E-07 | 0.345389 | + | 3.42674 | 7.16064 | | 16.2832 | 16.2737 | 16.19 | 15.8907 | 15.8773 | 15.9832 | 15.9637 | 15.803 | 8 | 9 | 9 | 9 | 8 | 8 | 9 | 8 |
| FAM118B | Q9BPY3 | 0.002926 | 0.000547 | 0.34659 | | 0.773738 | 1.56608 | | 14.9681 | 14.6678 | 15.0608 | 14.8449 | 14.0907 | 14.9308 | 14.4106 | 14.4846 | 4 | 4 | 4 | 4 | 1 | 2 | 4 | 3 |
| PPWD1 | Q96BP3 | 4.01E-12 | 2.51E-12 | 0.347098 | + | 1.84917 | 3.4195 | | 14.9801 | 15.0803 | 15.0387 | 14.9119 | 14.5329 | 14.7974 | 14.5349 | 14.6526 | 15 | 14 | 15 | 14 | 6 | 12 | 10 | 13 |
| ZCCHC3 | Q9NUD5 | 0.002742 | 0.000518 | 0.34767 | | 0.552335 | 1.24731 | | 14.0124 | 13.6321 | 13.8662 | NaN | NaN | 12.9966 | 13.855 | 13.6161 | 3 | 2 | 3 | NaN | NaN | 1 | 2 | 1 |
| ELOVL5 | Q9NYP7;Q9 | 0.000443 | 9.61E-05 | 0.348698 | + | 1.39773 | 2.61188 | | 17.7787 | 17.9308 | 17.9141 | 17.739 | 17.274 | 17.7288 | 17.3363 | 17.551 | 2 | 2 | 2 | 2 | 2 | 2 | 2 | 2 |
| GPR89A;GPR89A;GPI | B7ZAQ6;B7 | 2.80E-09 | 1.09E-09 | 0.348859 | | 1.21266 | 2.29782 | | 15.7335 | 15.5272 | 15.6384 | 15.0271 | 15.0326 | 15.5048 | 15.5242 | 15.3322 | 4 | 5 | 4 | 4 | 4 | 4 | 4 | 4 |
| UQCC2 | Q9BRT2 | 5.80E-09 | 2.17E-09 | 0.348877 | | 0.465172 | 1.03023 | | 16.0258 | 15.8293 | 15.8929 | 15.9938 | 14.7094 | 15.3097 | 15.755 | 16.0678 | 3 | 4 | 4 | 3 | 3 | 2 | 4 | 4 |
| UBE2I | P63279 | 3.02E-13 | 2.53E-13 | 0.35008 | | 0.57073 | 1.21868 | | 18.1287 | 17.9733 | 18.2562 | 18.2353 | 17.537 | 17.1164 | 17.7519 | 18.2058 | 8 | 7 | 8 | 8 | 8 | 7 | 8 | 8 |
| FIS1 | Q9Y3D6 | 5.71E-08 | 1.90E-08 | 0.350303 | | 0.382871 | 0.87719 | | 18.3435 | 18.2174 | 18.4146 | 18.5071 | 17.576 | 17.0062 | 18.2431 | 18.5419 | 5 | 4 | 4 | 4 | 5 | 4 | 5 | 5 |
| MTX3 | Q5HYI7;Q5 | 2.20E-08 | 7.65E-09 | 0.350457 | | 0.78544 | 1.58574 | | 15.5147 | 15.4004 | 15.4962 | 15.4076 | 14.8398 | 15.4896 | 15.2254 | 14.6373 | 6 | 6 | 6 | 6 | 3 | 5 | 6 | 4 |
| MSH3 | P20585 | 1.84E-10 | 8.46E-11 | 0.351254 | | 0.901387 | 1.77934 | | 14.6351 | 14.5894 | 14.5766 | 14.3035 | 14.3834 | 13.8256 | 14.0435 | 14.6894 | 9 | 10 | 13 | 8 | 9 | 5 | 7 | 10 |
| DDX28 | Q9NUL7 | 0.001006 | 0.000207 | 0.351538 | | 0.667215 | 1.38562 | | 14.715 | 14.6243 | 14.5961 | 14.8923 | 14.0734 | 13.7906 | 14.1998 | 14.5118 | 2 | 1 | 1 | 1 | 1 | 1 | 1 | 2 |
| PEPD | P12955 | 4.13E-13 | 3.32E-13 | 0.351911 | | 1.05002 | 2.02628 | | 19.1821 | 19.1826 | 19.0983 | 18.6801 | 18.8883 | 19.0571 | 19.0276 | 18.3589 | 18 | 18 | 17 | 17 | 18 | 17 | 17 | 17 |
| MGME1 | Q9BQP7 | 4.16E-08 | 1.40E-08 | 0.352251 | | 0.729817 | 1.49205 | | 13.6343 | 13.549 | 13.6396 | 13.734 | 13.124 | 12.6931 | 13.2282 | 13.4975 | 1 | 2 | 2 | 1 | 2 | 1 | 2 | 2 |
| LRP1 | Q07954 | 1.37E-17 | 4.19E-17 | 0.352327 | + | 1.85534 | 3.43109 | | 18.2771 | 18.3332 | 18.2946 | 17.9978 | 18.0238 | 18.1671 | 17.8217 | 17.7363 | 133 | 131 | 134 | 131 | 126 | 131 | 132 | 130 |
| ZC3HC1 | Q86WB0 | 1.56E-10 | 7.27E-11 | 0.353075 | + | 2.47522 | 4.69369 | | 15.5995 | 15.6179 | 15.6137 | 15.3452 | 15.301 | 15.1956 | 15.0681 | 15.3765 | 10 | 10 | 10 | 10 | 7 | 10 | 10 | 9 |
| RNASEH2C | Q8TDP1 | 0.002978 | 0.000556 | 0.353076 | + | 1.53824 | 2.85588 | | 15.7784 | 15.7233 | 15.9869 | 15.3302 | 15.7752 | 15.4227 | 15.3408 | 15.5133 | 3 | 3 | 4 | 3 | 3 | 3 | 3 | 3 |
| ITPK1 | Q13572 | 2.95E-21 | 3.09E-20 | 0.353301 | + | 2.60427 | 4.98578 | | 15.9295 | 15.8886 | 15.8375 | 15.7028 | 15.5911 | 15.4859 | 15.4559 | 15.424 | 1 | 1 | 1 | 2 | 1 | 1 | 1 | 1 |
| BBS2 | Q9BXC9 | 2.06E-06 | 5.79E-07 | 0.356008 | + | 1.38446 | 2.58911 | | 14.877 | 14.8521 | 14.7901 | 14.2972 | 14.5419 | 14.8495 | 14.4307 | 14.2994 | 7 | 9 | 8 | 4 | 3 | 6 | 5 | 5 |
| SDHD | O14521;O1 | 0.000269 | 6.00E-05 | 0.357468 | | 0.489206 | 1.0738 | | 15.1063 | 14.7173 | 15.0729 | 14.3481 | 13.843 | 15.2469 | 14.8965 | 14.7056 | 2 | 2 | 2 | 2 | 1 | 2 | 2 | 2 |
| PDS5B | Q9NTI5;Q9 | 7.02E-18 | 2.36E-17 | 0.357474 | + | 2.69772 | 5.20464 | | 15.383 | 15.4573 | 15.4443 | 15.1779 | 15.0724 | 15.0376 | 14.9016 | 15.1642 | 30 | 29 | 30 | 26 | 21 | 26 | 26 | 26 |
| SLC25A13 | Q9UJS0;Q9 | 6.64E-07 | 1.97E-07 | 0.357673 | | 1.29769 | 2.44125 | | 16.278 | 16.1382 | 16.1343 | 15.5687 | 16.1002 | 16.0603 | 15.6968 | 15.7031 | 13 | 14 | 15 | 9 | 12 | 13 | 9 | 13 |
| MRPS6 | P82932 | 1.02E-13 | 9.75E-14 | 0.358031 | | 0.523104 | 1.13456 | | 15.9122 | 16.0604 | 16.2667 | 16.3455 | 15.1816 | 15.2586 | 15.7001 | 16.1231 | 4 | 5 | 4 | 5 | 4 | 2 | 4 | 5 |
| MT-ND1 | P03886 | 4.89E-08 | 1.63E-08 | 0.358507 | | 1.10061 | 2.11047 | | 17.4933 | 17.4292 | 17.4766 | 17.1744 | 16.7272 | 17.4163 | 16.9132 | 17.308 | 3 | 3 | 3 | 3 | 2 | 3 | 3 | 2 |
| CENPB | P07199 | 5.25E-05 | 1.29E-05 | 0.359723 | | 1.09769 | 2.10561 | | 15.9543 | 15.8292 | 15.877 | 15.7664 | 15.2597 | 15.4532 | 15.2774 | 15.879 | 4 | 4 | 4 | 4 | 3 | 3 | 3 | 3 |
| TATDN1 | Q6P1N9 | 4.86E-06 | 1.31E-06 | 0.359752 | | 0.944333 | 1.85073 | | 15.8981 | 15.9709 | 15.9158 | 15.6649 | 15.1464 | 15.997 | 15.6685 | 15.3656 | 8 | 8 | 6 | 5 | 3 | 7 | 4 | 4 |
| FST | P19883;P1 | 1.80E-09 | 7.17E-10 | 0.359845 | | 0.236846 | 0.585368 | | 14.3858 | 13.7278 | 14.3483 | 14.0784 | 14.5019 | 13.6464 | 12.1435 | 14.6004 | 1 | 2 | 3 | 2 | 1 | 2 | 1 | 4 |
| MRPS5 | P82675 | 3.54E-11 | 1.85E-11 | 0.360179 | | 1.29141 | 2.43061 | | 16.035 | 16.0769 | 15.9412 | 15.862 | 15.3161 | 15.921 | 15.6222 | 15.5661 | 12 | 11 | 13 | 9 | 9 | 14 | 10 | 10 |
| FN3KRP | Q9HA64 | 1.94E-12 | 1.31E-12 | 0.361049 | | 0.817482 | 1.63943 | | 15.6152 | 15.7001 | 15.8122 | 15.5854 | 15.674 | 15.5747 | 14.9679 | 14.9386 | 5 | 5 | 5 | 5 | 5 | 5 | 5 | 5 |
| PPIL3 | Q9H2H8 | 1.32E-11 | 7.52E-12 | 0.361711 | + | 1.302 | 2.44856 | | 16.9112 | 16.7638 | 16.7992 | 16.1202 | 16.6537 | 16.5536 | 16.6804 | 16.3072 | 6 | 7 | 7 | 5 | 5 | 6 | 8 | 4 |
| ACACA | Q13085;Q1 | 9.55E-13 | 6.95E-13 | 0.361735 | + | 1.62447 | 3.00862 | | 16.6437 | 16.6537 | 16.6428 | 16.5881 | 16.362 | 16.152 | 16.0705 | 16.2525 | 65 | 67 | 65 | 66 | 55 | 59 | 53 | 55 |
| PDLIM1 | O00151 | 8.68E-19 | 3.83E-18 | 0.361917 | + | 1.35519 | 2.53904 | | 18.963 | 19.0119 | 19.0201 | 18.3145 | 18.6588 | 18.9838 | 18.6323 | 18.5929 | 21 | 20 | 20 | 19 | 19 | 20 | 22 | 20 |
| CTDSP2 | O14595 | 0.009402 | 0.001619 | 0.362249 | | 0.5434 | 1.22981 | | 14.0663 | 14.0678 | 13.9491 | 13.6269 | NaN | 14.1893 | 13.1803 | NaN | 3 | 2 | 2 | 2 | NaN | 1 | 1 | NaN |
| CASP3 | P42574 | 7.00E-11 | 3.46E-11 | 0.36283 | | 0.576177 | 1.22822 | | 16.5338 | 16.6045 | 16.6392 | 16.702 | 15.5383 | 16.6982 | 15.995 | 16.2148 | 7 | 7 | 7 | 7 | 7 | 7 | 7 | 7 |
| DTYMK | P23919 | 2.06E-16 | 4.40E-16 | 0.363249 | + | 1.64662 | 3.04827 | | 17.6083 | 17.6315 | 17.5728 | 17.5108 | 17.3426 | 17.0031 | 17.1058 | 17.2423 | 13 | 13 | 13 | 13 | 13 | 12 | 11 | 13 |
| HNRNPH2 | P55795 | 1.22E-13 | 1.13E-13 | 0.363357 | + | 2.18807 | 4.0823 | | 18.8895 | 18.671 | 18.8019 | 18.3874 | 18.2538 | 18.535 | 18.3783 | 18.5662 | 5 | 5 | 5 | 5 | 5 | 5 | 5 | 5 |
| STAG1 | Q8WVM7;( | 7.58E-11 | 3.71E-11 | 0.363575 | + | 2.9837 | 5.91623 | | 14.9203 | 14.9738 | 14.9785 | 14.7326 | 14.5383 | 14.6535 | 14.476 | 14.5694 | 15 | 12 | 13 | 13 | 8 | 10 | 12 | 10 |

| Gene names | UniProt IDs | Protein Group P-value | Protein Group Q-value | Log2 Difference NUP214 vs Controls (n=5) | p<0.05 | -log10 p-value | T-test Test statistic | NPC Localization | Log2 MS2_quantity_NUP214_1 | Log2 MS2_quantity_NUP214_2 | Log2 MS2_quantity_NUP214_3 | Log2 MS2_quantity_Control_1 | Log2 MS2_quantity_Control_2 | Log2 MS2_quantity_Control_3 | Log2 MS2_quantity_Control_4 | Log2 MS2_quantity_Control_5 | Number Peptides for Quantitation NUP214_1 | Number Peptides for Quantitation NUP214_2 | Number Peptides for Quantitation NUP214_3 | Number Peptides for Quantitation Control_1 | Number Peptides for Quantitation Control_2 | Number Peptides for Quantitation Control_3 | Number Peptides for Quantitation Control_4 | Number Peptides for Quantitation Control_5 |
|---|---|---|---|---|---|---|---|---|---|---|---|---|---|---|---|---|---|---|---|---|---|---|---|---|
| YBX3 | P16989-2 | 0.003237 | 0.000598 | 0.364114 | | 0.860908 | 1.71194 | | 15.4815 | 15.1955 | 15.3731 | 14.8037 | 14.6612 | 15.5565 | 14.9773 | 14.9308 | 2 | 2 | 2 | 1 | 1 | 2 | 1 | 1 |
| TXLNG | Q9NUQ3;Q | 3.61E-06 | 9.90E-07 | 0.364182 | | 1.03496 | 2.00125 | | 15.9346 | 16.1317 | 16.1188 | 15.8807 | 15.5382 | 15.4814 | 15.4591 | 16.1285 | 5 | 5 | 5 | 5 | 3 | 5 | 1 | 5 |
| NFIC | P08651;P0 | 1.60E-16 | 3.58E-16 | 0.36452 | + | 1.82388 | 3.37222 | | 15.5342 | 15.5769 | 15.4994 | 15.1522 | 15.4011 | 15.0255 | 14.9798 | 15.3029 | 5 | 7 | 6 | 5 | 3 | 6 | 5 | 5 |
| RGPD8;RGPD5 | O14715;Q9 | 0.000171 | 3.93E-05 | 0.365036 | | 0.490027 | 1.09466 | | 15.2028 | 14.7901 | 15.3441 | 15.0334 | NaN | 14.0328 | 14.7343 | 15.1887 | 1 | 1 | 1 | 1 | NaN | 2 | 2 | 1 |
| HSD3B7 | Q9H2F3 | 5.11E-08 | 1.70E-08 | 0.365596 | | 0.347809 | 0.809852 | | 14.8107 | 14.8264 | 14.9164 | 15.7196 | 14.1726 | 14.4362 | 14.426 | 13.6736 | 5 | 6 | 7 | 6 | 3 | 4 | 2 | 1 |
| DOCK6 | Q96HP0 | 0.001595 | 0.00032 | 0.366197 | | 0.420491 | 0.947937 | | 13.85 | 13.9335 | 13.5728 | 12.5711 | 14.0302 | 13.2284 | 14.0731 | 13.1934 | 4 | 4 | 2 | 1 | 3 | 2 | 3 | 1 |
| TARBP1 | Q13395 | 3.30E-05 | 8.24E-06 | 0.366318 | | 0.869647 | 1.7265 | | 14.1523 | 14.147 | 14.1723 | 13.7824 | 14.1565 | 14.0028 | 13.794 | 13.2187 | 3 | 4 | 5 | 4 | 3 | 4 | 3 | 3 |
| WDR33 | Q9C0J8 | 4.43E-06 | 1.21E-06 | 0.366375 | + | 1.587 | 2.94195 | | 14.8084 | 14.7465 | 14.885 | 14.663 | 14.2442 | 14.5963 | 14.5095 | 14.2215 | 7 | 6 | 7 | 7 | 3 | 4 | 5 | 6 |
| SH3GLB2 | Q9NR46 | 1.17E-14 | 1.45E-14 | 0.36658 | + | 1.87826 | 3.47422 | | 16.4667 | 16.4936 | 16.4717 | 16.12 | 16.268 | 15.8506 | 16.2758 | 16.0393 | 13 | 13 | 14 | 12 | 13 | 13 | 13 | 13 |
| INTS5 | Q6P9B9 | 3.57E-06 | 9.80E-07 | 0.366992 | + | 3.06814 | 6.13932 | | 14.8091 | 14.8334 | 14.8753 | 14.3837 | 14.6153 | 14.4494 | 14.3908 | 14.5221 | 5 | 8 | 8 | 4 | 4 | 5 | 5 | 4 |
| VKORC1 | Q9BQB6;Q9 | 0.001252 | 0.000255 | 0.367459 | + | 1.3232 | 2.48455 | | 20.4716 | 20.2206 | 20.401 | 19.817 | 20.0683 | 19.9465 | 20.3592 | 19.7937 | 2 | 2 | 2 | 2 | 2 | 2 | 2 | 2 |
| OSBPL6 | Q9BZF3;Q9 | 0.001338 | 0.000271 | 0.36748 | + | 1.73527 | 3.20873 | | 14.9199 | 14.9721 | 14.9237 | 14.3602 | 14.4097 | 14.7658 | 14.7639 | 14.5559 | 6 | 7 | 9 | 4 | 4 | 4 | 7 | 4 |
| PLEKHO2 | Q8TD55 | 1.50E-08 | 5.32E-09 | 0.367856 | | 1.26201 | 2.38091 | | 16.9076 | 16.8103 | 16.862 | 16.0693 | 16.6779 | 16.7155 | 16.5159 | 16.4819 | 12 | 13 | 13 | 12 | 12 | 13 | 14 | 12 |
| BRD3 | Q15059 | 0.000749 | 0.000157 | 0.368004 | | 0.695161 | 1.43328 | | 14.8086 | 14.6571 | 14.5303 | 14.1896 | 14.9598 | 14.1833 | 13.8103 | 14.3437 | 3 | 3 | 3 | 3 | 2 | 2 | 1 | 2 |
| SLC5A3 | P53794 | 0.000859 | 0.000179 | 0.368196 | | 0.567335 | 1.2376 | | 15.6644 | 15.5383 | 15.6791 | 15.4252 | 14.5187 | NaN | 15.4909 | 15.6014 | 4 | 4 | 4 | 4 | 2 | NaN | 3 | 3 |
| PALLD | Q8WX93;Q | 5.11E-20 | 3.50E-19 | 0.368704 | | 0.764505 | 1.55056 | | 18.5236 | 18.5377 | 18.5225 | 18.0338 | 18.3142 | 18.6929 | 17.6012 | 18.1541 | 28 | 26 | 27 | 24 | 24 | 28 | 25 | 25 |
| SHMT1 | P34896;P3 | 7.86E-14 | 7.72E-14 | 0.368977 | | 0.372678 | 0.857763 | | 16.9352 | 16.9325 | 16.8671 | 16.7635 | 17.4168 | 16.4582 | 15.4258 | 16.6488 | 9 | 9 | 9 | 10 | 9 | 8 | 5 | 8 |
| ALDH9A1 | P49189 | 1.11E-15 | 1.88E-15 | 0.369046 | | 0.983696 | 1.9161 | | 18.6827 | 18.6674 | 18.6303 | 18.0447 | 18.289 | 18.733 | 18.46 | 17.9288 | 20 | 21 | 21 | 19 | 21 | 19 | 20 | 20 |
| EEFSEC | P57772 | 7.36E-11 | 3.61E-11 | 0.369169 | + | 1.86972 | 3.45812 | | 15.4437 | 15.5534 | 15.4889 | 15.0257 | 14.9766 | 15.2244 | 15.3871 | 15.017 | 17 | 18 | 18 | 15 | 12 | 16 | 16 | 15 |
| VPS13C | Q709C8;Q7 | 1.86E-22 | 2.66E-21 | 0.369264 | + | 1.36948 | 2.56346 | | 15.7585 | 15.7603 | 15.7406 | 15.2734 | 15.3747 | 15.6347 | 15.5915 | 15.0449 | 75 | 71 | 73 | 59 | 42 | 67 | 69 | 40 |
| METTL7A | Q9H8H3 | 2.65E-06 | 7.37E-07 | 0.369267 | | 0.641982 | 1.34234 | | 16.1774 | 16.1479 | 16.1037 | 15.0275 | 16.1 | 16.1031 | 16.0097 | 15.6283 | 5 | 5 | 5 | 2 | 5 | 4 | 4 | 2 |
| EZR | P15311 | 2.56E-24 | 7.58E-23 | 0.369635 | | 1.04478 | 2.01758 | | 18.8275 | 18.8624 | 18.8204 | 17.9761 | 18.7564 | 18.5364 | 18.6715 | 18.3953 | 25 | 25 | 25 | 23 | 25 | 24 | 24 | 24 |
| DAZAP1 | Q96EP5;Q9 | 1.52E-05 | 3.92E-06 | 0.369817 | + | 2.01555 | 3.73773 | | 18.0681 | 18.1596 | 18.0805 | 17.9358 | 17.5595 | 17.862 | 17.6048 | 17.7026 | 8 | 8 | 8 | 8 | 8 | 8 | 8 | 8 |
| CCDC51 | Q96ER9;Q9 | 0.002752 | 0.000519 | 0.372402 | | 0.960041 | 1.87682 | | 14.1834 | 14.4497 | 14.4184 | 14.4307 | 13.5758 | 13.8203 | 14.0713 | 13.9925 | 3 | 3 | 3 | 2 | 1 | 3 | 3 | 1 |
| TACO1 | Q9BSH4 | 4.12E-17 | 1.08E-16 | 0.373322 | + | 3.2207 | 6.5587 | | 15.9837 | 16.0639 | 15.9756 | 15.5238 | 15.7211 | 15.6525 | 15.5611 | 15.7134 | 11 | 11 | 11 | 12 | 9 | 11 | 9 | 11 |
| AGPAT4 | Q9NRZ5 | 2.21E-15 | 3.43E-15 | 0.37392 | | 0.726872 | 1.48707 | | 16.1308 | 16.1501 | 16.0072 | 15.6952 | 15.7247 | 16.1554 | 15.9798 | 15.0555 | 8 | 8 | 8 | 7 | 5 | 8 | 5 | 4 |
| APEX1 | P27695 | 1.35E-11 | 7.66E-12 | 0.374626 | + | 3.6095 | 7.73164 | | 18.2224 | 18.1954 | 18.222 | 17.791 | 17.8875 | 17.8459 | 17.9373 | 17.7315 | 21 | 21 | 21 | 20 | 19 | 20 | 21 | 20 |
| ACAD11 | Q709F0 | 6.86E-10 | 2.89E-10 | 0.374896 | | 0.782823 | 1.58135 | | 15.9591 | 16.1291 | 16.0038 | 15.1492 | 15.4629 | 15.5633 | 16.0723 | 16.0311 | 11 | 13 | 12 | 10 | 9 | 9 | 12 | 14 |
| PTPRA | P18433;P1 | 0.000551 | 0.000118 | 0.375188 | | 1.06208 | 2.04634 | | 15.3062 | 15.5046 | 15.3022 | 15.2135 | 15.2309 | 15.1585 | 14.56 | 14.816 | 7 | 7 | 5 | 7 | 4 | 4 | 3 | 3 |
| BNIP1 | Q12981 | 2.32E-05 | 5.87E-06 | 0.375994 | | 0.846439 | 1.6878 | | 15.9867 | 16.0042 | 15.9841 | 15.762 | 15.5818 | 14.9843 | 15.9076 | 15.8429 | 6 | 7 | 8 | 5 | 5 | 4 | 6 | 7 |
| SNRPD2 | P62316 | 6.03E-13 | 4.60E-13 | 0.37617 | + | 1.72321 | 3.18673 | | 19.3682 | 19.4655 | 19.4028 | 19.2537 | 19.0415 | 18.7324 | 19.0087 | 19.1435 | 10 | 10 | 10 | 10 | 10 | 10 | 10 | 10 |
| AHNAK2 | Q8IVF2-3 | 2.56E-24 | 7.58E-23 | 0.376858 | | 0.656358 | 1.36703 | | 17.3793 | 17.3427 | 17.3976 | 17.7423 | 16.7425 | 16.7983 | 16.5753 | 17.1231 | 183 | 184 | 185 | 166 | 156 | 163 | 158 | 157 |
| IMPDH2 | P12268 | 3.01E-14 | 3.31E-14 | 0.377326 | + | 1.74499 | 3.22652 | | 18.6339 | 18.6557 | 18.6477 | 18.1971 | 18.1059 | 18.5793 | 18.1238 | 18.336 | 25 | 25 | 25 | 24 | 24 | 25 | 24 | 25 |
| DBR1 | Q9UK59 | 6.45E-07 | 1.92E-07 | 0.377717 | + | 3.66188 | 7.90215 | | 15.867 | 15.8749 | 15.9638 | 15.6063 | 15.5685 | 15.424 | 15.5316 | 15.4907 | 8 | 9 | 9 | 8 | 6 | 6 | 8 | 6 |
| TUT1 | Q9H6E5 | 0.002862 | 0.000537 | 0.377738 | + | 1.39514 | 2.60744 | | 14.8923 | 14.9959 | 14.8995 | 14.7241 | 14.1637 | 14.7598 | 14.5011 | 14.6088 | 1 | 1 | 1 | 1 | 2 | 1 | 2 | 1 |
| SUOX | P51687 | 2.84E-08 | 9.78E-09 | 0.378008 | | 0.907799 | 1.79001 | | 15.1123 | 15.3975 | 15.0371 | 14.6278 | 14.6643 | 15.1659 | 15.1332 | 14.4304 | 2 | 4 | 4 | 2 | 1 | 2 | 3 | 2 |
| PDHB | P11177;P1 | 5.02E-13 | 3.93E-13 | 0.378065 | + | 1.6443 | 3.04411 | | 18.247 | 18.2333 | 18.2416 | 18.0941 | 17.6178 | 18.0456 | 17.7001 | 17.8551 | 16 | 16 | 16 | 16 | 15 | 16 | 16 | 16 |
| RPP38 | P78345 | 5.05E-05 | 1.24E-05 | 0.378192 | + | 2.19459 | 4.09564 | | 16.2223 | 16.1815 | 16.183 | 15.9371 | 15.6482 | 16.0185 | 15.7423 | 15.7409 | 7 | 7 | 5 | 4 | 6 | 5 | 5 | 5 |
| GPNMB | Q14956;Q1 | 8.11E-07 | 2.39E-07 | 0.378694 | | 0.835101 | 1.66888 | | 19.8241 | 19.9186 | 19.9368 | 19.102 | 19.5172 | 19.3428 | 20.124 | 19.4862 | 4 | 4 | 3 | 3 | 3 | 3 | 4 | 3 |
| YEATS2 | Q9ULM3 | 0.009175 | 0.001582 | 0.379149 | + | 1.33893 | 2.64279 | | 14.3748 | 14.5582 | 14.4301 | 14.2008 | 14.2538 | 13.7431 | NaN | 14.1032 | 1 | 2 | 1 | 2 | 1 | 1 | NaN | 1 |
| GTPBP6 | O43824 | 3.06E-05 | 7.65E-06 | 0.379742 | + | 3.51904 | 7.44447 | | 14.9724 | 14.8992 | 14.8533 | 14.5084 | 14.5757 | 14.4135 | 14.6066 | 14.5386 | 10 | 10 | 9 | 8 | 5 | 8 | 8 | 6 |
| FHOD1 | Q9Y613 | 5.31E-17 | 1.36E-16 | 0.380191 | + | 2.27501 | 4.26215 | | 16.2136 | 16.2108 | 16.2035 | 15.924 | 15.9989 | 15.7619 | 15.6128 | 15.848 | 24 | 25 | 24 | 23 | 23 | 23 | 22 | 25 |
| SLC27A1 | Q6PCB7 | 2.80E-10 | 1.25E-10 | 0.380693 | | 0.690198 | 1.42483 | | 15.8668 | 15.8298 | 15.7775 | 14.8055 | 15.9449 | 15.7224 | 15.2106 | 15.5367 | 11 | 13 | 13 | 9 | 13 | 14 | 12 | 10 |
| ADD1 | P35611;P3 | 9.53E-13 | 6.93E-13 | 0.381759 | + | 1.42841 | 2.66469 | | 17.7955 | 17.8356 | 17.7701 | 17.5065 | 17.3673 | 17.7159 | 17.059 | 17.4447 | 24 | 24 | 25 | 23 | 20 | 23 | 23 | 23 |
| MTHFD2 | P13995 | 2.15E-13 | 1.87E-13 | 0.382082 | | 0.43736 | 0.979211 | | 17.0583 | 17.0872 | 17.0381 | 16.0057 | 16.1529 | 17.2779 | 17.4497 | 16.5095 | 9 | 9 | 8 | 7 | 8 | 7 | 8 | 8 |
| BCAT2 | O15382 | 3.87E-11 | 2.00E-11 | 0.382222 | + | 2.15128 | 4.00748 | | 16.3032 | 16.2907 | 16.2946 | 16.1359 | 15.9167 | 15.9936 | 15.7496 | 15.7739 | 10 | 10 | 10 | 11 | 9 | 11 | 9 | 10 |
| SLC27A3 | Q5K4L6 | 4.22E-07 | 1.28E-07 | 0.382363 | | 0.992336 | 1.93045 | | 15.0182 | 14.8332 | 15.0098 | 14.1908 | 14.2518 | 14.8467 | 14.8408 | 14.7268 | 5 | 4 | 5 | 2 | 1 | 4 | 4 | 2 |
| PPT1 | P50897 | 2.54E-05 | 6.39E-06 | 0.382387 | | 1.04141 | 2.01198 | | 16.8694 | 16.8529 | 16.9424 | 16.2439 | 16.5057 | 16.3581 | 17.048 | 16.3736 | 6 | 6 | 5 | 5 | 4 | 4 | 5 | 5 |
| TCEA1 | P23193 | 9.20E-13 | 6.72E-13 | 0.382544 | + | 1.72948 | 3.19817 | | 16.7704 | 16.8187 | 16.7207 | 16.2315 | 16.2251 | 16.6668 | 16.5224 | 16.2914 | 16 | 16 | 16 | 15 | 14 | 15 | 17 | 15 |
| ARID5B | Q14865;Q1 | 0.000108 | 2.55E-05 | 0.384133 | | 0.449327 | 1.01804 | | 13.8222 | 13.4312 | 13.134 | 12.8524 | 12.377 | 13.4744 | NaN | 13.6096 | 3 | 5 | 4 | 1 | 2 | 2 | NaN | 1 |
| QTRT1 | Q9BXR0 | 4.60E-12 | 2.84E-12 | 0.384312 | | 1.12165 | 2.14553 | | 16.127 | 16.0749 | 16.017 | 15.8219 | 15.5961 | 16.0615 | 15.2538 | 15.71 | 6 | 7 | 6 | 7 | 5 | 6 | 3 | 7 |
| ADCY9 | O60503 | 1.80E-05 | 4.60E-06 | 0.384633 | + | 2.22875 | 4.16591 | | 14.958 | 14.9027 | 14.8688 | 14.4521 | 14.6872 | 14.5477 | 14.6332 | 14.3059 | 15 | 14 | 16 | 12 | 9 | 16 | 11 | 12 |
| PCCB | P05166;P0 | 6.70E-13 | 5.05E-13 | 0.385274 | + | 1.83666 | 3.39608 | | 15.4906 | 15.408 | 15.4292 | 14.8796 | 15.2911 | 15.1409 | 15.1255 | 14.8494 | 14 | 15 | 15 | 11 | 10 | 12 | 10 | 12 |
| CERCAM | Q5T4B2;Q5 | 8.27E-12 | 4.89E-12 | 0.386529 | | 0.581802 | 1.23806 | | 17.3717 | 17.4274 | 17.3733 | 16.5894 | 16.6818 | 17.5577 | 17.5938 | 16.5986 | 15 | 13 | 13 | 12 | 13 | 13 | 14 | 11 |
| TTC30A | Q86WT1 | 0.005404 | 0.00096 | 0.386578 | | 0.471608 | 1.13852 | | 14.1486 | 14.3086 | 14.9125 | NaN | NaN | 14.2826 | NaN | 13.8573 | 3 | 2 | 1 | NaN | NaN | 1 | NaN | 1 |
| PLEKHA5 | Q9HAU0;Q | 1.47E-12 | 1.02E-12 | 0.387198 | + | 6.31341 | 22.6416 | | 15.1767 | 15.1578 | 15.1693 | 14.7738 | 14.7587 | 14.8201 | 14.7976 | 14.7536 | 17 | 16 | 16 | 13 | 9 | 15 | 13 | 12 |
| SF3A2 | Q15428 | 3.73E-20 | 2.74E-19 | 0.387412 | + | 2.27672 | 4.26573 | | 16.1621 | 16.2061 | 16.1947 | 15.9771 | 15.6794 | 15.8268 | 15.6145 | 15.9034 | 8 | 8 | 8 | 8 | 7 | 7 | 8 | 8 |
| ZFC3H1 | O60293;O6 | 0.006619 | 0.001163 | 0.388614 | | 0.407733 | 0.960758 | | 13.1375 | 12.922 | 13.4443 | 12.0312 | NaN | 13.1999 | NaN | 13.1069 | 1 | 2 | 1 | 1 | NaN | 1 | NaN | 2 |
| VPS72 | Q15906;Q1 | 0.004329 | 0.000782 | 0.388666 | | 0.539008 | 1.18556 | | 14.5898 | 14.7545 | 14.5731 | 14.3339 | 13.6302 | 14.0915 | NaN | 14.9463 | 2 | 3 | 2 | 2 | 1 | 1 | NaN | 2 |
| DSTN | P60981;P6 | 7.43E-14 | 7.34E-14 | 0.388743 | + | 1.48027 | 2.75453 | | 19.6541 | 19.8285 | 19.7224 | 19.1578 | 19.0749 | 19.4887 | 19.6249 | 19.385 | 13 | 13 | 13 | 13 | 13 | 12 | 13 | 12 |
| PTEN | P60484;P6 | 1.30E-13 | 1.20E-13 | 0.388786 | | 0.954837 | 1.86818 | | 15.8104 | 15.855 | 15.8144 | 15.0045 | 15.7207 | 15.7472 | 15.5927 | 15.1239 | 7 | 6 | 6 | 5 | 6 | 6 | 5 | 6 |
| FAM98B | Q52LJ0 | 6.66E-09 | 2.48E-09 | 0.389236 | | 0.920898 | 1.81178 | | 16.7919 | 16.7216 | 16.8012 | 16.4108 | 16.3594 | 15.7981 | 16.7235 | 16.6198 | 8 | 8 | 9 | 8 | 7 | 6 | 7 | 7 |
| UBA7 | P41226 | 2.02E-11 | 1.11E-11 | 0.389646 | + | 1.33415 | 2.50318 | | 16.4292 | 16.4396 | 16.4122 | 15.7788 | 16.1887 | 16.4095 | 15.8382 | 15.9716 | 22 | 23 | 22 | 18 | 19 | 19 | 19 | 21 |
| PTPMT1 | Q8WUK0 | 0.002506 | 0.000479 | 0.390327 | + | 1.67176 | 3.09348 | | 13.2217 | 12.9278 | 13.2577 | 12.7437 | 12.5154 | 12.7158 | 12.7627 | 12.9895 | 1 | 1 | 1 | 1 | 1 | 1 | 1 | 1 |
| STON1 | Q9Y6Q2;Q9 | 0.000419 | 9.11E-05 | 0.39167 | | 0.800135 | 1.61038 | | 15.2891 | 15.3962 | 15.3442 | 14.8071 | 14.8053 | 15.2317 | 14.4376 | 15.4758 | 8 | 9 | 9 | 6 | 3 | 5 | 5 | 8 |
| PDP1 | Q9P0J1;Q9 | 1.02E-14 | 1.29E-14 | 0.392109 | + | 2.02791 | 3.7619 | | 15.4069 | 15.4664 | 15.334 | 15.1628 | 14.7372 | 15.1326 | 14.9919 | 15.0271 | 15 | 16 | 15 | 13 | 13 | 12 | 15 | 14 |
| NDUFB4 | O95168;O9 | 6.44E-07 | 1.91E-07 | 0.3923 | | 0.522106 | 1.13278 | | 17.7991 | 17.6789 | 17.9388 | 17.895 | 16.9859 | 16.654 | 17.5713 | 17.9603 | 4 | 4 | 4 | 4 | 3 | 3 | 4 | 4 |
| PGM2L1 | Q6PCE3 | 2.81E-06 | 7.80E-07 | 0.393606 | + | 5.73219 | 18.0629 | | 16.406 | 16.4844 | 16.4552 | 16.0908 | 16.0536 | 16.0493 | 16.0252 | 16.0557 | 18 | 18 | 19 | 16 | 10 | 15 | 15 | 14 |
| CBX5 | P45973 | 8.41E-20 | 5.36E-19 | 0.393952 | + | 1.7253 | 3.19054 | | 15.767 | 15.9421 | 15.905 | 15.7495 | 15.3508 | 15.2361 | 15.5537 | 15.4971 | 10 | 10 | 10 | 9 | 7 | 8 | 7 | 7 |
| RFC1 | P35251;P3 | 3.16E-12 | 2.03E-12 | 0.39408 | + | 1.84383 | 3.4095 | | 14.9269 | 14.8794 | 14.9189 | 14.6793 | 14.4196 | 14.297 | 14.4223 | 14.7533 | 13 | 13 | 14 | 12 | 9 | 7 | 9 | 7 |
| HPRT1 | P00492 | 3.80E-11 | 1.97E-11 | 0.395508 | + | 1.75023 | 3.23611 | | 18.1669 | 18.2605 | 18.2662 | 18.0654 | 17.5608 | 17.7689 | 17.9983 | 17.7849 | 11 | 11 | 11 | 11 | 10 | 11 | 11 | 11 |
| GUF1 | Q8N442 | 0.003196 | 0.000592 | 0.396272 | | 0.687847 | 1.45591 | | 14.8341 | 15.1897 | 14.9475 | 14.4829 | 14.652 | NaN | 15.1462 | 14.0957 | 3 | 3 | 2 | 1 | 2 | NaN | 4 | 1 |
| CARS2 | Q9HA77 | 1.46E-11 | 8.20E-12 | 0.396618 | | 1.06689 | 2.05434 | | 17.085 | 17.1461 | 17.083 | 16.7317 | 17.0843 | 16.1907 | 16.7693 | 16.7645 | 16 | 15 | 15 | 14 | 14 | 14 | 14 | 14 |
| HIBADH | P31937 | 1.52E-10 | 7.08E-11 | 0.396832 | + | 4.11533 | 9.51874 | | 17.8421 | 17.8838 | 17.8367 | 17.3422 | 17.4589 | 17.4822 | 17.5159 | 17.4877 | 12 | 12 | 11 | 11 | 11 | 10 | 11 | 11 |
| SCCPDH | Q8NBX0 | 3.08E-11 | 1.62E-11 | 0.397159 | + | 1.61431 | 2.9905 | | 18.6129 | 18.6094 | 18.5197 | 18.0497 | 18.3625 | 18.4755 | 18.0362 | 17.9938 | 10 | 10 | 10 | 9 | 10 | 10 | 10 | 9 |
| GPHN | Q9NQX3;Q | 7.48E-12 | 4.45E-12 | 0.397909 | + | 1.44345 | 2.69067 | | 15.4871 | 15.5801 | 15.5248 | 14.748 | 15.3301 | 15.3642 | 15.1189 | 15.1026 | 11 | 10 | 12 | 6 | 9 | 10 | 8 | 8 |
| NDUFB9 | Q9Y6M9 | 0.000205 | 4.65E-05 | 0.398566 | + | 1.3993 | 2.61458 | | 17.5733 | 17.6134 | 17.5899 | 17.4991 | 17.0312 | 17.4446 | 16.9833 | 17.0101 | 8 | 8 | 8 | 8 | 6 | 8 | 8 | 8 |
| ACTR6 | Q9GZN1 | 0.004787 | 0.000859 | 0.398786 | | 0.175283 | 0.455409 | | 14.8456 | 14.7008 | 15.0377 | 15.1428 | 15.4671 | 12.274 | NaN | 14.9664 | 1 | 1 | 2 | 1 | 1 | 2 | NaN | 1 |

| Gene names | UniProt IDs | Protein Group P-value | Protein Group Q-value | Log2 Difference NUP214 vs Controls (n=5) | p<0.05 | -log10 p-value | T-test Test statistic | NPC Localization | Log2 MS2_quantity_ NUP214_1 | Log2 MS2_quantity_ NUP214_2 | Log2 MS2_quantity_ NUP214_3 | Log2 MS2_quantity_ Control_1 | Log2 MS2_quantity_ Control_2 | Log2 MS2_quantity_ Control_3 | Log2 MS2_quantity_ Control_4 | Log2 MS2_quantity_ Control_5 | Number Peptides for Quantitation NUP214_1 | Number Peptides for Quantitation NUP214_2 | Number Peptides for Quantitation NUP214_3 | Number Peptides for Quantitation Control_1 | Number Peptides for Quantitation Control_2 | Number Peptides for Quantitation Control_3 | Number Peptides for Quantitation Control_4 | Number Peptides for Quantitation Control_5 |
|---|---|---|---|---|---|---|---|---|---|---|---|---|---|---|---|---|---|---|---|---|---|---|---|---|
| PLD3 | Q8IV08 | 1.76E-10 | 8.14E-11 | 0.399617 | + | 1.97089 | 3.65103 | | 18.693 | 18.7695 | 18.7484 | 18.413 | 18.1853 | 18.1128 | 18.5472 | 18.4286 | 11 | 10 | 12 | 10 | 10 | 9 | 10 | 10 |
| ALAD | P13716;P1 | 4.87E-18 | 1.75E-17 | 0.399825 | + | 1.56015 | 2.89445 | | 17.6527 | 17.6587 | 17.6741 | 17.5909 | 17.2344 | 17.3768 | 17.1075 | 17.0005 | 9 | 9 | 9 | 9 | 9 | 9 | 9 | 9 |
| UBTF | P17480-2 | 3.22E-13 | 2.66E-13 | 0.400061 | + | 3.35904 | 6.95816 | | 15.7409 | 15.6974 | 15.7291 | 15.4038 | 15.1921 | 15.3581 | 15.2542 | 15.4039 | 21 | 21 | 22 | 20 | 15 | 18 | 16 | 19 |
| ITIH2 | P19823 | 4.19E-09 | 1.60E-09 | 0.400139 | | 0.847501 | 1.68958 | | 15.2578 | 15.1963 | 15.241 | 15.4008 | 14.7914 | 14.6251 | 14.3473 | 14.9932 | 4 | 4 | 4 | 4 | 2 | 4 | 3 | 4 |
| DYNC2H1 | Q8NCM8;Q | 4.00E-12 | 2.50E-12 | 0.401206 | | 0.457952 | 1.01704 | | 14.4962 | 14.3495 | 14.3832 | 12.8746 | 14.1015 | 14.4716 | 14.4753 | 14.1191 | 5 | 6 | 8 | 3 | 4 | 5 | 4 | 3 |
| ZNF503 | Q96F45;Q9 | 4.62E-15 | 6.48E-15 | 0.401477 | | 1.03674 | 2.08149 | | 14.5336 | 14.3203 | 14.5883 | 13.6253 | 14.2313 | 14.1853 | NaN | 14.275 | 1 | 1 | 1 | 1 | 1 | 1 | NaN | 1 |
| WIPF1 | O43516;O4 | 1.64E-08 | 5.81E-09 | 0.401934 | | 1.19532 | 2.26872 | | 17.4469 | 17.4283 | 17.4079 | 17.4968 | 17.0809 | 16.7059 | 16.8786 | 16.9666 | 9 | 9 | 9 | 9 | 9 | 9 | 9 | 9 |
| ERBB2 | P04626;P0 | 5.13E-07 | 1.55E-07 | 0.401962 | | 0.566564 | 1.21138 | | 14.5031 | 14.7658 | 14.4379 | 14.8594 | 13.3656 | 14.3395 | 14.0236 | 14.2468 | 4 | 3 | 2 | 4 | 3 | 2 | 3 | 3 |
| PCYOX1L | Q8NBM8 | 0.000774 | 0.000162 | 0.40291 | + | 1.33328 | 2.5017 | | 15.9794 | 15.9349 | 15.8837 | 15.7855 | 15.6444 | 15.7194 | 15.1607 | 15.3388 | 5 | 5 | 5 | 5 | 5 | 5 | 4 | 5 |
| AASS | Q9UDR5 | 1.92E-11 | 1.06E-11 | 0.403795 | | 1.28769 | 2.42431 | | 15.5978 | 15.7476 | 15.6055 | 15.357 | 15.5595 | 15.3974 | 14.9599 | 14.9589 | 16 | 17 | 16 | 12 | 8 | 8 | 4 | 7 |
| FHL3 | Q13643 | 7.36E-11 | 3.61E-11 | 0.403945 | | 0.848756 | 1.69167 | | 17.0515 | 17.1017 | 17.0721 | 16.6389 | 16.2404 | 17.2874 | 16.7707 | 16.4183 | 8 | 7 | 9 | 7 | 7 | 8 | 7 | 7 |
| PFDN1 | O60925 | 2.03E-06 | 5.69E-07 | 0.40404 | | 0.425914 | 0.95802 | | 17.8356 | 17.5284 | 18.2281 | 18.1509 | 16.8571 | 16.9143 | 17.1831 | 18.1947 | 3 | 3 | 3 | 3 | 3 | 3 | 3 | 3 |
| ITGB5 | P18084 | 1.80E-17 | 5.27E-17 | 0.404307 | | 0.67436 | 1.39783 | | 16.8972 | 17.0493 | 17.0332 | 16.759 | 16.158 | 17.3472 | 16.4236 | 16.2568 | 23 | 24 | 23 | 23 | 18 | 24 | 25 | 23 |
| U2AF2 | P26368;P2 | 7.95E-18 | 2.63E-17 | 0.404809 | + | 1.5948 | 2.95578 | | 17.9274 | 17.9766 | 18.0366 | 17.7964 | 17.2836 | 17.7441 | 17.3889 | 17.6639 | 13 | 12 | 12 | 13 | 12 | 12 | 11 | 11 |
| NT5C | Q8TCD5 | 1.49E-08 | 5.31E-09 | 0.406424 | | 0.889122 | 1.75893 | | 16.6871 | 16.9214 | 16.9405 | 16.2403 | 16.9584 | 15.9671 | 16.6091 | 16.4414 | 7 | 7 | 7 | 7 | 5 | 7 | 8 | 7 |
| SFXN3 | Q9BWM7 | 1.82E-14 | 2.14E-14 | 0.406677 | + | 2.14728 | 3.99939 | | 18.4375 | 18.4477 | 18.4464 | 18.0554 | 17.984 | 17.7929 | 18.2618 | 18.0917 | 12 | 12 | 12 | 12 | 12 | 11 | 12 | 12 |
| ARL3 | P36405 | 0.000198 | 4.51E-05 | 0.407538 | + | 1.59152 | 2.94997 | | 18.7568 | 18.7626 | 18.84 | 18.1661 | 18.1517 | 18.4199 | 18.7052 | 18.4518 | 8 | 8 | 8 | 7 | 7 | 7 | 8 | 7 |
| PHLDA3 | Q9Y5J5 | 1.71E-16 | 3.79E-16 | 0.409282 | | 0.762004 | 1.54635 | | 16.2024 | 16.3151 | 16.3666 | 16.3472 | 15.1631 | 15.8899 | 16.0691 | 15.9576 | 4 | 4 | 4 | 4 | 3 | 4 | 4 | 4 |
| METTL26 | Q96S19 | 8.57E-23 | 1.47E-21 | 0.409333 | | 1.20137 | 2.27888 | | 16.4378 | 16.2339 | 16.5135 | 15.7169 | 16.449 | 16.0395 | 15.8781 | 15.8451 | 5 | 5 | 5 | 3 | 6 | 5 | 3 | 4 |
| GABPA | Q06546 | 3.71E-20 | 2.74E-19 | 0.409556 | + | 2.24262 | 4.19463 | | 15.5036 | 15.6103 | 15.6814 | 15.4423 | 15.1381 | 15.0779 | 15.0801 | 15.206 | 9 | 8 | 9 | 8 | 6 | 6 | 7 | 8 |
| RPP25L | Q8N5L8 | 0.007644 | 0.001333 | 0.410033 | | 1.08603 | 2.08619 | | 16.592 | 16.4639 | 16.4696 | 16.4988 | 16.1531 | 16.0705 | 15.5944 | 16.1755 | 2 | 2 | 2 | 1 | 1 | 2 | 1 | 1 |
| PPA2 | Q9H2U2;Q | 5.57E-15 | 7.56E-15 | 0.410808 | + | 2.102 | 3.90842 | | 16.4247 | 16.4538 | 16.4727 | 16.0053 | 15.7939 | 16.022 | 16.2806 | 16.096 | 16 | 16 | 16 | 14 | 11 | 12 | 14 | 13 |
| LRRFIP1 | Q32MZ4-4 | 7.53E-06 | 2.00E-06 | 0.411139 | | 0.937976 | 1.84017 | | 16.3028 | 16.2868 | 16.1782 | 16.2057 | 15.8532 | 15.4362 | 15.5078 | 16.2211 | 7 | 8 | 7 | 6 | 5 | 4 | 4 | 5 |
| TRAPPC6B | Q86SZ2 | 3.37E-07 | 1.04E-07 | 0.41159 | | 1.00238 | 1.94713 | | 15.2211 | 15.0664 | 15.092 | 15.1541 | 14.2449 | 14.518 | 14.9011 | 14.7563 | 7 | 8 | 8 | 7 | 4 | 5 | 5 | 6 |
| MRPL43 | Q8N983;Q8 | 0.001539 | 0.000309 | 0.411642 | | 0.490569 | 1.07626 | | 16.0466 | 15.9863 | 16.0105 | 16.0925 | 15.5023 | 14.5608 | 15.7114 | 16.1471 | 3 | 3 | 5 | 4 | 3 | 3 | 4 | 3 |
| OSBPL1A | Q9BXW6 | 7.72E-05 | 1.85E-05 | 0.411769 | + | 2.03341 | 3.77268 | | 15.6726 | 15.8014 | 15.7331 | 15.5449 | 15.3469 | 15.4364 | 15.1354 | 15.1561 | 14 | 15 | 16 | 10 | 8 | 13 | 9 | 9 |
| SARS1 | P49591 | 5.78E-07 | 1.73E-07 | 0.411896 | | 1.02268 | 1.98086 | | 18.804 | 18.8153 | 18.8146 | 18.0465 | 18.143 | 18.6983 | 18.8362 | 18.2732 | 31 | 31 | 32 | 26 | 27 | 31 | 31 | 28 |
| SERAC1 | Q96JX3 | 0.001009 | 0.000208 | 0.412792 | | 0.353797 | 0.821457 | | 13.81 | 13.523 | 13.7242 | 13.178 | 13.426 | 14.5869 | 12.3665 | 12.8073 | 1 | 1 | 1 | 1 | 1 | 1 | 1 | 1 |
| CNOT6L | Q96LI5 | 0.001019 | 0.00021 | 0.413038 | | 1.23015 | 2.32722 | | 14.6609 | 14.1578 | 14.4698 | 13.8057 | 14.0216 | 14.2445 | 14.2589 | 13.7516 | 2 | 1 | 2 | 1 | 2 | 2 | 2 | 1 |
| ROBO1 | Q9Y6N7;Q9 | 1.09E-05 | 2.86E-06 | 0.413719 | | 0.462032 | 1.0245 | | 14.7429 | 14.8374 | 14.7937 | 13.4498 | 15.0569 | 14.4518 | 14.9584 | 13.9712 | 4 | 4 | 3 | 4 | 3 | 3 | 4 | 3 |
| MRPS21 | P82921 | 1.56E-10 | 7.29E-11 | 0.413868 | + | 2.08043 | 3.86546 | | 16.2016 | 16.0306 | 16.082 | 15.8585 | 15.5688 | 15.4953 | 15.6632 | 15.8685 | 3 | 3 | 3 | 3 | 2 | 2 | 2 | 2 |
| MON1A | Q86VX9;Q8 | 0.046519 | 0.007567 | 0.414834 | | 0.167032 | 0.436442 | | 15.234 | NaN | 15.0411 | 15.3864 | 12.4788 | 15.172 | 15.5306 | 15.0458 | 1 | NaN | 2 | 2 | 2 | 1 | 2 | 2 |
| SLC16A1 | P53985 | 4.33E-07 | 1.31E-07 | 0.415015 | | 0.899775 | 1.77666 | | 15.4659 | 15.4909 | 15.5118 | 14.7752 | 14.7529 | 15.5894 | 15.4006 | 14.8546 | 9 | 9 | 9 | 7 | 7 | 9 | 9 | 8 |
| PLOD1 | Q02809;Q0 | 1.36E-06 | 3.90E-07 | 0.416611 | + | 1.52399 | 2.83087 | | 19.6652 | 19.6923 | 19.6814 | 18.9398 | 19.375 | 19.4579 | 19.4812 | 19.0612 | 34 | 34 | 34 | 34 | 34 | 35 | 34 | 34 |
| CELF1 | Q92879-4 | 3.79E-13 | 3.07E-13 | 0.419337 | | 1.1594 | 2.20859 | | 16.9187 | 16.9805 | 16.892 | 16.8413 | 16.1079 | 16.7559 | 16.2591 | 16.591 | 14 | 14 | 14 | 14 | 12 | 14 | 14 | 14 |
| SLC9A6 | Q92581-2; | 1.01E-08 | 3.66E-09 | 0.420004 | | 1.21604 | 2.30351 | | 15.6229 | 15.5628 | 15.5672 | 15.5064 | 14.6939 | 15.3255 | 15.0888 | 15.2069 | 3 | 3 | 3 | 3 | 3 | 3 | 3 | 3 |
| RAB8B | Q92930 | 1.16E-07 | 3.73E-08 | 0.420102 | + | 1.70408 | 3.15195 | | 16.7427 | 16.7404 | 16.7398 | 16.5155 | 16.3646 | 16.3903 | 15.9347 | 16.3992 | 5 | 6 | 5 | 5 | 5 | 5 | 5 | 6 |
| SNTB2 | Q13425 | 1.52E-15 | 2.48E-15 | 0.420188 | + | 2.61541 | 5.01156 | | 17.2563 | 17.2672 | 17.2042 | 16.8431 | 16.9615 | 16.9344 | 16.6251 | 16.7479 | 20 | 21 | 20 | 19 | 20 | 20 | 19 | 18 |
| TRIM5 | Q9C035 | 4.23E-07 | 1.29E-07 | 0.420209 | + | 1.78251 | 3.29549 | | 15.5667 | 15.6569 | 15.6568 | 15.496 | 15.2194 | 14.9342 | 15.0944 | 15.2889 | 11 | 10 | 9 | 8 | 6 | 7 | 8 | 8 |
| GLYR1 | Q49A26;Q4 | 1.16E-11 | 6.62E-12 | 0.421829 | + | 1.44891 | 2.70011 | | 18.0754 | 18.0421 | 18.0147 | 18.0023 | 17.4048 | 17.7689 | 17.3913 | 17.5439 | 9 | 10 | 10 | 7 | 6 | 8 | 6 | 7 |
| SGPL1 | O95470 | 8.01E-11 | 3.90E-11 | 0.422698 | + | 1.8301 | 3.38383 | | 15.4603 | 15.5329 | 15.5036 | 15.0665 | 14.9149 | 15.4074 | 15.1061 | 14.8861 | 17 | 15 | 15 | 13 | 9 | 14 | 16 | 11 |
| TCOF1 | Q13428;Q1 | 2.82E-07 | 8.75E-08 | 0.423426 | + | 3.88542 | 8.66638 | | 15.4722 | 15.521 | 15.4784 | 14.9762 | 15.0237 | 15.0972 | 15.0524 | 15.1861 | 23 | 22 | 21 | 19 | 12 | 18 | 20 | 18 |
| DBT | P11182 | 3.22E-10 | 1.42E-10 | 0.425885 | + | 1.42961 | 2.66676 | | 15.6115 | 15.5312 | 15.5592 | 15.1356 | 15.1019 | 15.5894 | 14.9502 | 14.9299 | 12 | 12 | 11 | 13 | 9 | 13 | 12 | 13 |
| NUDT16 | Q96DE0 | 1.07E-09 | 4.43E-10 | 0.426412 | | 0.750424 | 1.52684 | | 16.8523 | 16.8353 | 16.957 | 16.9012 | 16.3804 | 15.7069 | 16.5148 | 16.7724 | 10 | 11 | 11 | 11 | 7 | 9 | 10 | 10 |
| SUB1 | P53999 | 4.79E-09 | 1.81E-09 | 0.426597 | + | 1.787 | 3.3038 | | 19.2497 | 19.2736 | 19.2561 | 19.1367 | 18.5744 | 18.9531 | 18.7615 | 18.7403 | 10 | 10 | 10 | 10 | 10 | 10 | 10 | 10 |
| MAN2C1 | Q9NTJ4;Q9 | 8.47E-15 | 1.09E-14 | 0.429065 | | 1.18524 | 2.25183 | | 16.7227 | 16.8214 | 16.7938 | 16.3428 | 16.3851 | 16.8483 | 16.1764 | 15.9985 | 8 | 8 | 8 | 8 | 4 | 8 | 7 | 6 |
| AP5B1 | Q2VPB7 | 3.51E-08 | 1.19E-08 | 0.429369 | | 1.2399 | 2.34364 | | 14.4616 | 14.3921 | 14.4595 | 13.6452 | 14.3388 | 14.1601 | 14.176 | 13.7216 | 5 | 2 | 5 | 3 | 2 | 1 | 3 | 3 |
| MCCC2 | Q9HCC0;Q9 | 2.73E-12 | 1.78E-12 | 0.430652 | + | 2.32977 | 4.37771 | | 16.5774 | 16.5543 | 16.5437 | 16.0101 | 16.0235 | 16.4076 | 16.0566 | 16.1412 | 14 | 15 | 15 | 13 | 10 | 14 | 12 | 11 |
| CDH11 | P55287 | 4.93E-06 | 1.33E-06 | 0.432213 | + | 5.30777 | 15.3004 | | 15.6567 | 15.7623 | 15.6856 | 15.2559 | 15.2453 | 15.2976 | 15.3005 | 15.2473 | 7 | 9 | 10 | 8 | 5 | 8 | 7 | 9 |
| MTARC2 | Q969Z3 | 0.003422 | 0.000629 | 0.432344 | | 0.552333 | 1.18634 | | 14.8015 | 14.8527 | 14.8081 | 13.4528 | 14.411 | 15.1191 | 14.2978 | 14.6614 | 3 | 4 | 3 | 4 | 3 | 3 | 3 | 3 |
| RAP2B | P61225 | 2.53E-12 | 1.66E-12 | 0.43257 | + | 2.15605 | 4.01715 | | 17.6158 | 17.6015 | 17.6557 | 17.2782 | 17.1073 | 17.3412 | 17.3167 | 16.9155 | 6 | 6 | 6 | 6 | 6 | 6 | 6 | 6 |
| CRIPT | Q9P021 | 0.037489 | 0.006125 | 0.432687 | | 0.249859 | 0.630619 | | 14.7809 | 14.8951 | 14.5379 | 15.0975 | NaN | NaN | 12.9561 | 14.8622 | 2 | 2 | 2 | 2 | NaN | NaN | 2 | 1 |
| ARPC3 | O15145 | 0.000104 | 2.45E-05 | 0.433014 | | 0.701211 | 1.44356 | | 19.4225 | 19.3025 | 19.4376 | 19.4996 | 18.5856 | 18.313 | 19.0317 | 19.3425 | 10 | 9 | 10 | 10 | 10 | 10 | 10 | 10 |
| QKI | Q96PU8;Q9 | 8.96E-07 | 2.62E-07 | 0.433093 | + | 2.60825 | 4.99499 | | 17.3431 | 17.3827 | 17.4048 | 16.92 | 17.1914 | 16.9055 | 16.819 | 16.8829 | 12 | 11 | 10 | 10 | 10 | 9 | 10 | 10 |
| PITPNM1 | O00562;O0 | 0.00024 | 5.39E-05 | 0.433105 | + | 2.46561 | 4.67239 | | 15.3312 | 15.1796 | 15.2291 | 14.7731 | 14.7587 | 14.6649 | 15.0543 | 14.8165 | 5 | 6 | 5 | 5 | 3 | 4 | 4 | 3 |
| RHOA | P61586 | 1.57E-06 | 4.47E-07 | 0.43396 | + | 3.92469 | 8.807 | | 21.0722 | 21.101 | 21.0268 | 20.7629 | 20.5712 | 20.6161 | 20.574 | 20.6393 | 11 | 11 | 11 | 11 | 11 | 11 | 11 | 11 |
| COL5A1 | P20908 | 2.08E-05 | 5.29E-06 | 0.434657 | | 0.352186 | 0.818339 | | 17.6714 | 17.7175 | 17.6997 | 16.6075 | 16.8582 | 18.507 | 17.8794 | 16.4556 | 25 | 26 | 28 | 22 | 22 | 30 | 25 | 23 |
| COPS7B | Q9H9Q2 | 5.29E-11 | 2.68E-11 | 0.434704 | | 0.57235 | 1.22152 | | 15.6803 | 15.684 | 15.8756 | 15.9589 | 15.4183 | 14.3953 | 15.1523 | 15.6347 | 7 | 6 | 6 | 6 | 5 | 4 | 5 | 7 |
| TAPBP | O15533;O1 | 0.000429 | 9.32E-05 | 0.435069 | + | 2.1537 | 4.01237 | | 17.9853 | 18.0245 | 18.0768 | 17.408 | 17.7029 | 17.7446 | 17.7242 | 17.3892 | 5 | 5 | 5 | 5 | 5 | 5 | 5 | 5 |
| SGCD | Q92629-2 | 3.79E-10 | 1.65E-10 | 0.43576 | | 0.668245 | 1.38738 | | 17.7911 | 17.7561 | 17.7714 | 16.5668 | 17.888 | 17.7089 | 17.4377 | 17.0842 | 16 | 16 | 16 | 16 | 17 | 16 | 17 | 15 |
| GALC | P54803;P5 | 0.000846 | 0.000176 | 0.436327 | | 0.215255 | 0.539169 | | 14.8502 | 14.7789 | 14.4881 | 14.2192 | 11.961 | 14.8985 | 15.323 | 14.9454 | 1 | 1 | 2 | 1 | 2 | 2 | 3 | 3 |
| TBC1D25 | Q3MII6 | 0.000154 | 3.57E-05 | 0.437147 | | 1.11337 | 2.22107 | | 14.9222 | 14.6905 | 15.2962 | 14.62 | NaN | 14.6625 | 14.2035 | 14.644 | 1 | 2 | 1 | 1 | NaN | 1 | 2 | 1 |
| MMAB | Q96EY8 | 6.54E-07 | 1.94E-07 | 0.437428 | | 0.824017 | 1.65035 | | 15.7556 | 15.6546 | 15.2961 | 15.0518 | 14.5616 | 14.9922 | 15.6048 | 15.4462 | 3 | 5 | 4 | 2 | 3 | 4 | 5 | 3 |
| SLFN5 | Q08AF3 | 1.83E-08 | 6.45E-09 | 0.437695 | | 0.732409 | 1.49643 | | 16.2901 | 16.3646 | 16.2695 | 15.9069 | 15.8252 | 16.4279 | 16.0923 | 15.0996 | 23 | 22 | 23 | 21 | 18 | 20 | 20 | 13 |
| COL12A1 | Q99715 | 9.06E-20 | 5.59E-19 | 0.43786 | | 0.447976 | 0.99876 | | 19.2275 | 19.2962 | 19.2566 | 18.1444 | 18.6942 | 19.9977 | 18.9793 | 18.2955 | 127 | 130 | 131 | 121 | 124 | 135 | 131 | 124 |
| PSIP1 | O75475 | 8.31E-10 | 3.47E-10 | 0.438529 | + | 3.01069 | 5.98686 | | 16.2551 | 16.2363 | 16.2881 | 15.8644 | 15.628 | 15.7824 | 15.9299 | 15.9018 | 15 | 13 | 14 | 14 | 11 | 12 | 13 | 14 |
| SRR | Q9GZT4 | 9.25E-10 | 3.85E-10 | 0.439203 | | 0.615082 | 1.29593 | | 16.3067 | 16.3741 | 16.3777 | 15.2305 | 16.4442 | 16.5332 | 15.8349 | 15.5254 | 10 | 10 | 11 | 9 | 10 | 10 | 9 | 9 |
| H2BC18;H2BC18;H2E | Q5QNW6;Q | 2.26E-05 | 5.74E-06 | 0.439403 | | 1.06396 | 2.04947 | | 23.7678 | 23.5416 | 23.3941 | 22.8339 | 23.6293 | 22.9173 | 22.9519 | 23.3098 | 10 | 9 | 9 | 8 | 10 | 9 | 8 | 10 |
| MEGF6 | O75095;O7 | 0.00353 | 0.000647 | 0.43983 | | 0.614612 | 1.29512 | | 14.4174 | 14.6331 | 14.4549 | 14.1058 | 14.5954 | 13.5443 | 14.6299 | 13.4343 | 2 | 2 | 2 | 2 | 2 | 1 | 2 | 1 |
| MCUB | Q9NWR8 | 0.008094 | 0.001405 | 0.440573 | | 1.21019 | 2.29367 | | 14.7973 | 14.7832 | 14.4363 | 14.1342 | 14.4992 | 13.8306 | 14.1699 | 14.5244 | 2 | 2 | 5 | 1 | 1 | 2 | 5 | 4 |
| PJA1 | Q8NG27;Q | 6.18E-05 | 1.50E-05 | 0.441425 | | 0.636847 | 1.33351 | | 14.465 | 14.1774 | 14.2587 | 12.9785 | 14.3772 | 13.9102 | 13.7977 | 14.2312 | 3 | 3 | 3 | 3 | 3 | 3 | 3 | 3 |
| PLEKHG2 | Q9H7P9;Q9 | 0.002467 | 0.000472 | 0.442257 | | 1.01586 | 1.96953 | | 14.6075 | 14.9908 | 14.8826 | 14.5773 | 14.5882 | 14.6738 | 14.2569 | 13.8273 | 2 | 3 | 2 | 2 | 3 | 2 | 2 | 2 |
| DCTN3 | O75935;O7 | 5.50E-05 | 1.34E-05 | 0.443886 | | 0.416913 | 0.941271 | | 17.2601 | 16.9574 | 17.7448 | 17.7654 | 16.2901 | 16.1587 | 16.6107 | 17.5595 | 9 | 9 | 9 | 9 | 7 | 9 | 8 | 9 |
| KBTBD2 | Q8IY47 | 1.25E-16 | 2.89E-16 | 0.444123 | | 0.585819 | 1.24507 | | 14.3389 | 14.4095 | 14.1072 | 14.5675 | 13.2356 | 14.1027 | 13.2342 | 14.0653 | 4 | 5 | 3 | 3 | 1 | 3 | 1 | 2 |
| CPD | O75976 | 1.32E-13 | 1.21E-13 | 0.444945 | + | 3.34742 | 6.92385 | | 16.2963 | 16.3219 | 16.2602 | 15.8698 | 15.8933 | 15.6678 | 15.9445 | 15.8638 | 33 | 34 | 33 | 32 | 23 | 29 | 31 | 27 |
| PGAM5 | Q96HS1 | 9.68E-11 | 4.65E-11 | 0.445272 | + | 2.02959 | 3.7652 | | 16.8745 | 16.9637 | 16.9409 | 16.8011 | 16.344 | 16.5293 | 16.3282 | 16.4029 | 14 | 13 | 14 | 14 | 12 | 13 | 14 | 15 |
| COQ3 | Q9NZJ6 | 0.000607 | 0.000129 | 0.446078 | | 0.745022 | 1.51773 | | 15.7875 | 15.8877 | 15.8027 | 15.1973 | 16.0625 | 14.9426 | 14.9788 | 15.7183 | 2 | 1 | 2 | 1 | 1 | 1 | 1 | 2 |
| H2BC11;H2BC3;H2B( | P06899;P3 | 0.002419 | 0.000464 | 0.446833 | + | 2.48854 | 4.72333 | | 22.3509 | 22.548 | 22.5222 | 22.1165 | 22.1852 | 21.9459 | 21.8335 | 22.0531 | 2 | 2 | 2 | 2 | 2 | 2 | 2 | 2 |

| Gene names | UniProt IDs | Protein Group P-value | Protein Group Q-value | Log2 Difference NUP214 vs Controls (n=5) | p<0.05 | -log10 p-value | T-test Test statistic | NPC Localization | Log2 MS2_quantity_NUP214_1 | Log2 MS2_quantity_NUP214_2 | Log2 MS2_quantity_NUP214_3 | Log2 MS2_quantity_Control_1 | Log2 MS2_quantity_Control_2 | Log2 MS2_quantity_Control_3 | Log2 MS2_quantity_Control_4 | Log2 MS2_quantity_Control_5 | Number Peptides for Quantitation NUP214_1 | Number Peptides for Quantitation NUP214_2 | Number Peptides for Quantitation NUP214_3 | Number Peptides for Quantitation Control_1 | Number Peptides for Quantitation Control_2 | Number Peptides for Quantitation Control_3 | Number Peptides for Quantitation Control_4 | Number Peptides for Quantitation Control_5 |
|---|---|---|---|---|---|---|---|---|---|---|---|---|---|---|---|---|---|---|---|---|---|---|---|---|
| TMPO | P42167 | 2.01E-13 | 1.77E-13 | 0.446916 | + | 4.82885 | 12.6682 | | 18.4893 | 18.5395 | 18.5103 | 18.0804 | 18.1258 | 18.0305 | 17.988 | 18.106 | 21 | 21 | 20 | 20 | 21 | 20 | 19 | 21 |
| HMGB1 | P09429 | 1.62E-05 | 4.17E-06 | 0.447502 | + | 3.69714 | 8.0187 | | 19.0078 | 19.0053 | 19.0208 | 18.4683 | 18.6078 | 18.6564 | 18.4583 | 18.6282 | 15 | 14 | 15 | 15 | 12 | 12 | 10 | 14 |
| FADS1 | O60427 | 6.97E-10 | 2.93E-10 | 0.447547 | + | 2.14314 | 3.99103 | | 15.7351 | 15.6662 | 15.7398 | 15.4261 | 15.2766 | 15.386 | 14.9539 | 15.2882 | 9 | 11 | 11 | 9 | 6 | 10 | 8 | 7 |
| NUMA1 | Q14980 | 1.65E-23 | 3.59E-22 | 0.447617 | + | 3.7157 | 8.08064 | | 16.4363 | 16.4379 | 16.4442 | 15.9252 | 16.0833 | 15.8652 | 16.028 | 16.0577 | 84 | 85 | 84 | 81 | 66 | 74 | 74 | 73 |
| SNRPB;SNRPB;SNRPB | P14678;P1 | 5.53E-07 | 1.65E-07 | 0.447882 | + | 1.31768 | 2.47517 | | 19.0464 | 19.0797 | 19.0647 | 18.8417 | 18.6305 | 18.1103 | 18.6339 | 18.8621 | 8 | 8 | 8 | 8 | 8 | 8 | 8 | 8 |
| IFITM3 | Q01628 | 1.48E-11 | 8.34E-12 | 0.448089 | | 0.774091 | 1.56668 | | 15.7118 | 15.8874 | 16.0968 | 15.7076 | 14.8802 | 15.5544 | 16.0145 | 15.096 | 2 | 3 | 3 | 2 | 2 | 2 | 2 | 2 |
| MFAP4 | P55083;P5 | 0.00248 | 0.000474 | 0.448171 | | 0.20364 | 0.541662 | | 15.6224 | 15.5507 | 15.5365 | NaN | NaN | 16.2308 | 14.0126 | NaN | 2 | 2 | 2 | NaN | NaN | 2 | 2 | NaN |
| BICC1 | Q9H694 | 7.25E-17 | 1.78E-16 | 0.451652 | | 1.04991 | 2.02611 | | 15.2993 | 15.2923 | 15.2781 | 14.4454 | 14.8125 | 15.4567 | 14.7639 | 14.7127 | 21 | 18 | 20 | 15 | 11 | 20 | 17 | 17 |
| SEH1L | Q96EE3;Q9 | 1.46E-07 | 4.66E-08 | 0.455213 | + | 1.57649 | 2.92334 | | 16.3842 | 16.4153 | 16.3635 | 16.1493 | 15.6183 | 16.234 | 15.7467 | 15.914 | 11 | 12 | 12 | 12 | 9 | 12 | 10 | 12 |
| ALG8 | Q9BVK2;Q9 | 0.002919 | 0.000546 | 0.455472 | | 0.246848 | 0.606451 | | 14.6349 | 14.8818 | 15.1671 | 15.1295 | 12.2734 | 15.0475 | 15.2541 | 14.4912 | 2 | 2 | 2 | 2 | 1 | 2 | 3 | 2 |
| PTGFRN | Q9P2B2 | 4.94E-17 | 1.27E-16 | 0.457015 | | 0.304426 | 0.733639 | | 14.8773 | NaN | 15.0268 | 15.3648 | 13.2685 | 14.784 | 14.0739 | 14.9838 | 1 | NaN | 4 | 1 | 2 | 7 | 3 | 8 |
| LBR | Q14739 | 3.36E-05 | 8.38E-06 | 0.457354 | + | 1.46455 | 2.72722 | | 16.608 | 16.6167 | 16.5834 | 16.3976 | 16.1187 | 15.9758 | 15.7848 | 16.4498 | 11 | 9 | 10 | 10 | 10 | 9 | 9 | 11 |
| RAD21 | O60216 | 1.65E-15 | 2.66E-15 | 0.458028 | + | 3.21964 | 6.55569 | | 15.9028 | 15.8819 | 15.8111 | 15.5582 | 15.4444 | 15.3309 | 15.2675 | 15.4352 | 12 | 14 | 16 | 13 | 10 | 11 | 10 | 10 |
| ZNF133 | P52736;P5 | 0.015001 | 0.002526 | 0.458123 | | 0.1097 | 0.299257 | | 16.0008 | 16.0463 | 16.206 | 16.8303 | 17.0898 | 11.7513 | 16.8335 | NaN | 1 | 1 | 1 | 1 | 1 | 2 | 1 | NaN |
| HDHD5 | Q9BXW7;Q | 4.06E-08 | 1.37E-08 | 0.458561 | | 1.16363 | 2.21566 | | 16.1612 | 16.1617 | 16.2036 | 15.8667 | 15.7868 | 15.9743 | 15.1087 | 15.8481 | 9 | 9 | 10 | 10 | 8 | 9 | 8 | 9 |
| DGLUCY | Q7Z3D6;Q7 | 0.000459 | 9.93E-05 | 0.459053 | | 0.61375 | 1.29362 | | 15.7868 | 15.8205 | 15.7111 | 15.0688 | 15.7485 | 15.8107 | 14.3879 | 15.5528 | 11 | 12 | 11 | 3 | 6 | 11 | 8 | 6 |
| CRBN | Q96SW2;Q | 1.49E-10 | 6.96E-11 | 0.459077 | | 1.21587 | 2.30322 | | 16.2112 | 16.1647 | 15.9388 | 15.1976 | 15.8093 | 16.0436 | 15.6673 | 15.5112 | 3 | 3 | 3 | 3 | 1 | 3 | 3 | 3 |
| FTO | Q9C0B1 | 1.26E-16 | 2.91E-16 | 0.459135 | + | 2.1652 | 4.03569 | | 16.2443 | 16.3237 | 16.2356 | 16.075 | 15.6165 | 15.7512 | 15.6788 | 15.9222 | 17 | 18 | 17 | 17 | 13 | 17 | 17 | 16 |
| IFT46 | Q9NQC8;Q | 1.45E-12 | 1.00E-12 | 0.461184 | | 0.923269 | 1.81573 | | 14.9203 | 14.6653 | 14.6484 | 13.8047 | 14.2208 | 14.7778 | 14.6239 | 13.9901 | 1 | 2 | 2 | 2 | 1 | 1 | 2 | 1 |
| ISG15 | P05161 | 2.39E-07 | 7.46E-08 | 0.464237 | + | 1.57629 | 2.92297 | | 16.6811 | 16.7177 | 16.7231 | 16.2775 | 16.2112 | 15.9803 | 16.0751 | 16.6712 | 4 | 4 | 4 | 5 | 4 | 3 | 5 | 4 |
| NMI | Q13287 | 7.31E-12 | 4.38E-12 | 0.46449 | + | 1.66724 | 3.08532 | | 15.4788 | 15.4918 | 15.5078 | 14.9863 | 15.1586 | 15.3784 | 14.7165 | 14.9019 | 14 | 12 | 14 | 12 | 10 | 12 | 8 | 9 |
| RIOK1 | Q9BRS2 | 9.57E-15 | 1.22E-14 | 0.464879 | + | 1.595 | 2.95615 | | 15.5865 | 15.6523 | 15.7216 | 15.3778 | 14.8718 | 15.5221 | 15.101 | 15.0702 | 5 | 4 | 7 | 5 | 2 | 5 | 4 | 6 |
| CELF2 | O95319;O9 | 1.08E-08 | 3.93E-09 | 0.467244 | | 0.434812 | 0.974505 | | 15.0053 | 15.0344 | 14.9968 | 15.3477 | 15.0683 | 13.5901 | 13.7734 | 14.945 | 5 | 4 | 4 | 5 | 4 | 2 | 3 | 4 |
| FLNB | O75369-8 | 0.000422 | 9.18E-05 | 0.467489 | | 0.585323 | 1.24421 | | 14.0831 | 14.4768 | 14.5184 | 14.4121 | 14.5356 | 13.7137 | 13.0314 | 13.7669 | 2 | 2 | 1 | 1 | 1 | 1 | 1 | 1 |
| PODN | Q7Z5L7;Q7 | 6.78E-11 | 3.36E-11 | 0.467783 | | 0.400674 | 0.91085 | | 15.3232 | 15.3316 | 15.3691 | 15.3857 | 13.8048 | 15.6391 | 14.0786 | 15.4596 | 4 | 4 | 3 | 4 | 1 | 3 | 1 | 4 |
| SUN1 | O94901-9 | 3.40E-13 | 2.80E-13 | 0.467915 | + | 3.37869 | 7.01645 | | 15.8662 | 15.8046 | 15.8654 | 15.3031 | 15.4048 | 15.2911 | 15.3327 | 15.5558 | 24 | 26 | 24 | 26 | 20 | 24 | 22 | 23 |
| OPA3 | Q9H6K4 | 0.003012 | 0.000561 | 0.468423 | | 0.547535 | 1.17788 | | 16.2195 | 16.3398 | 16.3358 | 16.1024 | 15.5996 | 14.7864 | 16.1694 | 16.492 | 3 | 3 | 3 | 2 | 1 | 1 | 2 | 2 |
| MYADM | Q96S97 | 1.31E-10 | 6.16E-11 | 0.468885 | | 0.702642 | 1.44599 | | 20.1284 | 20.0938 | 20.0795 | 20.3244 | 18.8231 | 19.8293 | 19.5379 | 19.6436 | 4 | 5 | 5 | 5 | 4 | 5 | 5 | 5 |
| XPNPEP3 | Q9NQH7;Q | 0.001697 | 0.000338 | 0.469314 | + | 2.10084 | 3.9061 | | 16.0745 | 16.0627 | 16.1289 | 15.3268 | 15.8641 | 15.6998 | 15.5422 | 15.6641 | 6 | 5 | 7 | 5 | 4 | 5 | 4 | 5 |
| MRPL24 | Q96A35 | 5.57E-08 | 1.85E-08 | 0.469403 | | 1.16035 | 2.21016 | | 16.5042 | 16.4753 | 16.4132 | 16.2529 | 15.6664 | 16.2752 | 15.5522 | 16.2274 | 9 | 10 | 9 | 9 | 6 | 10 | 9 | 9 |
| RECK | O95980 | 4.26E-17 | 1.12E-16 | 0.470097 | | 0.648321 | 1.35324 | | 17.0342 | 17.0031 | 16.9308 | 16.8649 | 16.5325 | 17.0707 | 15.5572 | 16.5713 | 29 | 31 | 29 | 30 | 27 | 29 | 27 | 29 |
| TLN2 | Q9Y4G6 | 6.83E-14 | 6.83E-14 | 0.47016 | + | 3.15337 | 6.37095 | | 16.7483 | 16.7712 | 16.7334 | 16.2563 | 16.3703 | 16.4339 | 16.1233 | 16.2202 | 66 | 65 | 65 | 60 | 56 | 62 | 59 | 57 |
| HNRNPUL1 | Q9BUJ2;Q9 | 1.51E-11 | 8.49E-12 | 0.47083 | + | 2.23791 | 4.18485 | | 17.6281 | 17.6767 | 17.6225 | 17.284 | 17.1456 | 17.4026 | 16.9043 | 17.1216 | 22 | 22 | 24 | 22 | 23 | 22 | 22 | 22 |
| GNB4 | Q9HAV0 | 7.68E-07 | 2.26E-07 | 0.471523 | | 0.872041 | 1.73049 | | 17.4868 | 17.4714 | 17.3559 | 17.3663 | 16.4251 | 17.5072 | 16.7635 | 16.7704 | 7 | 6 | 7 | 7 | 6 | 7 | 6 | 5 |
| FBXO4 | Q9UKT5 | 9.35E-05 | 2.22E-05 | 0.471891 | + | 1.48133 | 2.75637 | | 15.8814 | 15.8332 | 15.8653 | 15.0967 | 15.3422 | 15.8148 | 15.1769 | 15.5098 | 7 | 7 | 7 | 4 | 2 | 6 | 6 | 2 |
| PEX11B | O96011;O9 | 2.05E-14 | 2.37E-14 | 0.473298 | | 0.863684 | 1.71656 | | 16.5723 | 16.6879 | 16.5681 | 16.3428 | 16.0637 | 15.3656 | 16.4224 | 16.4861 | 8 | 8 | 8 | 7 | 7 | 4 | 8 | 7 |
| DENND6A | Q8IWF6 | 0.011126 | 0.001899 | 0.473566 | | 0.762149 | 1.58903 | | 12.3341 | 11.8277 | 12.1303 | 12.1852 | NaN | 11.6277 | 11.6209 | 11.0615 | 1 | 2 | 2 | 2 | NaN | 2 | 1 | 1 |
| COL5A2 | P05997 | 1.41E-06 | 4.02E-07 | 0.473977 | | 0.382508 | 0.8765 | | 16.301 | 16.3311 | 16.345 | 15.3453 | 15.1198 | 17.2148 | 16.3508 | 15.2278 | 26 | 25 | 25 | 24 | 18 | 29 | 24 | 22 |
| ICAM1 | P05362 | 4.86E-15 | 6.76E-15 | 0.47414 | | 1.1913 | 2.26199 | | 15.0423 | 14.9804 | 14.9933 | 14.5269 | 14.0716 | 15.0079 | 14.3611 | 14.6884 | 5 | 3 | 5 | 3 | 2 | 4 | 4 | 4 |
| ADO | Q96SZ5 | 2.91E-13 | 2.44E-13 | 0.474524 | + | 1.30662 | 2.45639 | | 15.9413 | 15.8332 | 15.9288 | 15.0477 | 15.1946 | 15.8717 | 15.5519 | 15.467 | 8 | 6 | 8 | 6 | 7 | 6 | 6 | 7 |
| ZER1 | Q7Z7L7 | 0.000617 | 0.000131 | 0.475348 | + | 2.98484 | 5.9192 | | 14.4672 | 14.4671 | 14.391 | 13.9123 | 14.1406 | 14.0118 | 13.7845 | 13.9829 | 6 | 5 | 6 | 6 | 3 | 5 | 7 | 5 |
| CDA | P32320 | 3.15E-18 | 1.18E-17 | 0.475411 | | 0.266379 | 0.647083 | | 16.3528 | 16.2414 | 16.2445 | 14.989 | 14.7236 | 16.1788 | 17.7761 | 15.3531 | 1 | 1 | 1 | 1 | 1 | 2 | 2 | 1 |
| PALM2 | Q8IXS6-2 | 3.10E-10 | 1.37E-10 | 0.476616 | | 0.994594 | 1.9342 | | 14.6782 | 14.7985 | 14.6306 | 14.1056 | 13.6398 | 14.5874 | 14.6432 | 14.153 | 5 | 5 | 4 | 1 | 1 | 5 | 4 | 3 |
| GCDH | Q92947 | 3.58E-05 | 8.90E-06 | 0.478514 | + | 1.61037 | 2.98347 | | 16.7286 | 16.7533 | 16.7429 | 16.5341 | 16.0643 | 16.4585 | 15.9033 | 16.3552 | 8 | 8 | 8 | 8 | 8 | 8 | 8 | 8 |
| ACAT2 | Q9BWD1 | 4.29E-07 | 1.30E-07 | 0.478666 | | 1.27104 | 2.39615 | | 18.1196 | 18.2765 | 18.1397 | 18.052 | 17.8332 | 17.196 | 17.5704 | 17.848 | 12 | 14 | 12 | 12 | 11 | 13 | 13 | 12 |
| DCPS | Q96C86 | 2.36E-13 | 2.03E-13 | 0.480783 | + | 2.73299 | 5.28893 | | 16.8574 | 16.8846 | 16.8054 | 16.6045 | 16.2322 | 16.4024 | 16.3526 | 16.25 | 10 | 11 | 11 | 9 | 9 | 10 | 10 | 9 |
| H3C15 | Q71DI3 | 6.87E-15 | 9.09E-15 | 0.48092 | | 1.1308 | 2.16081 | | 23.0488 | 22.9704 | 22.9843 | 22.486 | 23.1326 | 22.3155 | 22.152 | 22.515 | 8 | 8 | 8 | 8 | 8 | 8 | 8 | 8 |
| SMYD3 | Q9H7B4;Q9 | 7.02E-05 | 1.69E-05 | 0.481219 | + | 5.51964 | 16.6243 | | 15.732 | 15.689 | 15.775 | 15.2558 | 15.3073 | 15.2367 | 15.2514 | 15.2027 | 5 | 4 | 4 | 4 | 3 | 4 | 3 | 3 |
| TES | Q9UGI8;Q9 | 9.46E-14 | 9.10E-14 | 0.481869 | | 0.3375 | 0.789769 | | 16.7198 | 16.7267 | 16.7168 | 14.459 | 16.7522 | 16.8285 | 16.8771 | 16.2792 | 23 | 23 | 23 | 19 | 21 | 23 | 23 | 23 |
| PRKDC | P78527 | 5.46E-19 | 2.63E-18 | 0.485291 | + | 3.41004 | 7.11024 | | 17.9731 | 18.0043 | 17.9715 | 17.4855 | 17.3512 | 17.6672 | 17.5181 | 17.4663 | 186 | 185 | 186 | 182 | 172 | 182 | 183 | 177 |
| CPT2 | P23786 | 7.51E-06 | 2.00E-06 | 0.485472 | + | 1.7364 | 3.2108 | | 15.5855 | 15.6262 | 15.6161 | 14.9091 | 14.8384 | 15.345 | 15.4062 | 15.1201 | 15 | 15 | 16 | 13 | 10 | 13 | 13 | 13 |
| PNP | P00491 | 5.42E-15 | 7.41E-15 | 0.485933 | | 0.448014 | 0.99883 | | 17.883 | 17.862 | 17.8595 | 17.8055 | 15.9864 | 18.0737 | 17.6155 | 17.4299 | 16 | 16 | 16 | 16 | 9 | 16 | 16 | 15 |
| DALRD3 | Q5D0E6;Q5 | 0.000289 | 6.43E-05 | 0.486307 | | 0.764411 | 1.5504 | | 14.0538 | 14.0056 | 14.0464 | 13.8766 | 13.4892 | 13.9737 | 12.6668 | 13.7386 | 2 | 3 | 3 | 1 | 1 | 2 | 1 | 1 |
| CFDP1 | Q9UEE9 | 2.04E-05 | 5.19E-06 | 0.488485 | + | 1.52725 | 2.83659 | | 17.2942 | 17.4251 | 17.1489 | 17.1342 | 16.8934 | 16.6084 | 16.4481 | 16.9205 | 3 | 4 | 3 | 3 | 2 | 3 | 3 | 3 |
| PLSCR4 | Q9NRQ2;Q | 0.002509 | 0.000479 | 0.489274 | + | 1.35423 | 2.53741 | | 17.7025 | 17.4393 | 17.5085 | 17.4228 | 16.8154 | 17.3709 | 16.8842 | 16.8109 | 1 | 1 | 2 | 1 | 1 | 2 | 1 | 1 |
| MRPL53 | Q96EL3 | 3.09E-06 | 8.52E-07 | 0.48962 | + | 1.94865 | 3.6082 | | 16.0173 | 15.8373 | 16.081 | 15.4079 | 15.4023 | 15.3078 | 15.4796 | 15.847 | 5 | 4 | 5 | 4 | 3 | 2 | 5 | 5 |
| DSE | Q9UL01 | 7.29E-11 | 3.58E-11 | 0.489637 | | 0.779722 | 1.57614 | | 15.3605 | 15.3487 | 15.2284 | 14.7154 | 14.3276 | 15.6947 | 14.7929 | 14.5839 | 6 | 4 | 5 | 4 | 3 | 6 | 3 | 4 |
| LIMS2 | Q7Z4I7;Q7 | 1.82E-06 | 5.13E-07 | 0.490032 | | 0.28828 | 0.691852 | | 14.9284 | 15.2382 | 15.0067 | 12.6124 | 14.4362 | 15.6924 | 15.1568 | 14.9409 | 3 | 3 | 3 | 2 | 2 | 3 | 3 | 3 |
| CLN5 | O75503 | 2.16E-07 | 6.76E-08 | 0.490746 | | 0.845013 | 1.68542 | | 15.8109 | 15.8441 | 15.7677 | 15.5885 | 14.9397 | 15.4294 | 15.9141 | 14.7123 | 5 | 6 | 6 | 6 | 4 | 5 | 5 | 6 |
| SACS | Q9NZJ4 | 6.59E-15 | 8.79E-15 | 0.491348 | + | 1.53937 | 2.85787 | | 15.2232 | 15.2233 | 15.2018 | 14.3322 | 14.9829 | 15.0173 | 14.7273 | 14.5638 | 66 | 63 | 67 | 38 | 33 | 53 | 37 | 34 |
| L2HGDH | Q9H9P8;Q9 | 1.53E-09 | 6.18E-10 | 0.492688 | + | 2.72492 | 5.26957 | | 14.6313 | 14.7185 | 14.7897 | 14.3297 | 14.3651 | 14.2805 | 14.0632 | 14.0638 | 2 | 3 | 3 | 1 | 1 | 1 | 1 | 1 |
| ANP32E | Q9BTT0 | 3.56E-08 | 1.21E-08 | 0.49286 | + | 3.01275 | 5.99229 | | 17.2008 | 17.2362 | 17.2831 | 16.7061 | 16.902 | 16.7108 | 16.8546 | 16.5623 | 5 | 5 | 5 | 5 | 4 | 5 | 5 | 5 |
| ATP1B1 | P05026;P0 | 1.17E-07 | 3.74E-08 | 0.493193 | + | 1.90703 | 3.5287 | | 17.0099 | 17.0592 | 17.0512 | 16.1824 | 16.7693 | 16.6092 | 16.4665 | 16.7071 | 8 | 7 | 7 | 6 | 5 | 7 | 7 | 7 |
| DNAJC19 | Q96DA6 | 0.000432 | 9.36E-05 | 0.493216 | | 0.487319 | 1.0704 | | 16.0408 | 16.1199 | 16.0598 | 16.3946 | 14.9132 | 14.629 | 15.8261 | 16.1386 | 5 | 4 | 4 | 4 | 3 | 2 | 4 | 5 |
| BAP18 | Q8IXM2;Q8 | 8.56E-05 | 2.04E-05 | 0.49418 | + | 1.47424 | 2.74405 | | 17.1288 | 16.7618 | 17.0928 | 16.3777 | 16.4769 | 16.2542 | 16.4414 | 16.9513 | 6 | 4 | 6 | 4 | 4 | 4 | 5 | 4 |
| CASP4 | P49662;P4 | 4.84E-10 | 2.08E-10 | 0.494237 | + | 2.86251 | 5.60668 | | 15.0191 | 15.0411 | 14.9808 | 14.4219 | 14.3297 | 14.5786 | 14.7061 | 14.5608 | 7 | 9 | 7 | 5 | 6 | 6 | 6 | 6 |
| NIPSNAP2 | O75323 | 0.002661 | 0.000504 | 0.495482 | + | 2.61752 | 5.01645 | | 16.4672 | 16.3507 | 16.257 | 15.8402 | 16.0331 | 15.9842 | 15.6685 | 15.7881 | 6 | 8 | 5 | 8 | 6 | 6 | 6 | 5 |
| COLGALT1 | Q8NBJ5 | 1.18E-06 | 3.39E-07 | 0.495538 | + | 1.86809 | 3.45505 | | 18.8286 | 18.837 | 18.771 | 18.3365 | 18.1598 | 18.6978 | 18.3148 | 18.0746 | 29 | 28 | 28 | 28 | 28 | 28 | 29 | 29 |
| ACSF3 | Q4G176 | 6.85E-10 | 2.89E-10 | 0.49581 | + | 1.87162 | 3.4617 | | 15.5158 | 15.4682 | 15.4452 | 14.9125 | 14.987 | 15.358 | 14.6978 | 14.9476 | 11 | 13 | 13 | 11 | 10 | 13 | 11 | 11 |
| LENG9 | Q96B70 | 0.003362 | 0.000619 | 0.496243 | | 0.878565 | 1.88664 | | 15.2432 | 15.4259 | 15.3011 | 15.1023 | 14.3127 | NaN | 15.0665 | NaN | 1 | 2 | 2 | 2 | 1 | NaN | 2 | NaN |
| FUT8 | Q9BYC5 | 4.60E-08 | 1.54E-08 | 0.499134 | + | 2.9609 | 5.85706 | | 16.3449 | 16.3175 | 16.3193 | 15.8312 | 15.9314 | 15.9792 | 15.614 | 15.7847 | 18 | 17 | 15 | 13 | 15 | 13 | 14 | 12 |
| IRS2 | Q9Y4H2 | 0.002002 | 0.000393 | 0.499211 | | 0.402339 | 0.913981 | | 14.8298 | 14.779 | 14.6168 | 13.174 | 13.8118 | 15.5455 | 14.7199 | 13.9621 | 4 | 7 | 5 | 2 | 3 | 7 | 3 | 2 |
| DNPH1 | O43598 | 0.001501 | 0.000302 | 0.499561 | + | 2.08134 | 3.86727 | | 17.7942 | 17.7484 | 17.764 | 16.9918 | 17.3086 | 17.1124 | 17.4148 | 17.5189 | 2 | 2 | 2 | 2 | 2 | 2 | 2 | 2 |
| H2AC21 | Q8IUE6 | 0.002463 | 0.000471 | 0.500738 | | 0.915943 | 1.80355 | | 18.28 | 17.7301 | 18.1436 | 17.8979 | 18.0918 | 17.2009 | 17.1762 | 17.3856 | 2 | 3 | 3 | 3 | 3 | 2 | 2 | 2 |
| PTGES3 | Q15185;Q1 | 5.85E-05 | 1.43E-05 | 0.502669 | + | 2.61627 | 5.01354 | | 19.9705 | 20.0073 | 19.9874 | 19.7741 | 19.472 | 19.3585 | 19.4438 | 19.3803 | 8 | 8 | 8 | 8 | 8 | 8 | 8 | 8 |
| HNRNPUL2 | Q1KMD3 | 3.37E-18 | 1.26E-17 | 0.503553 | + | 2.53359 | 4.82441 | | 18.0373 | 18.0433 | 18.0088 | 17.6306 | 17.284 | 17.7397 | 17.5328 | 17.4443 | 36 | 36 | 36 | 36 | 35 | 36 | 34 | 35 |
| FBLN2 | P98095;P9 | 5.51E-10 | 2.36E-10 | 0.504282 | + | 1.80778 | 3.34229 | | 15.2713 | 15.3544 | 15.2479 | 14.5854 | 14.8205 | 14.7058 | 15.2034 | 14.6194 | 10 | 9 | 9 | 2 | 8 | 6 | 9 | 7 |
| ACTN1 | P12814-2 | 0.000358 | 7.86E-05 | 0.505623 | | 0.410239 | 0.9288 | | 16.4196 | 16.5157 | 16.4776 | 17.2446 | 14.7098 | 15.9342 | 15.7289 | 16.2093 | 3 | 3 | 3 | 3 | 2 | 3 | 3 | 3 |

| Gene names | UniProt IDs | Protein Group P-value | Protein Group Q-value | Log2 Difference NUP214 vs Controls (n=5) | p<0.05 | -log10 p-value | T-test Test statistic | NPC Localization | Log2 MS2_quantity_NUP214_1 | Log2 MS2_quantity_NUP214_2 | Log2 MS2_quantity_NUP214_3 | Log2 MS2_quantity_Control_1 | Log2 MS2_quantity_Control_2 | Log2 MS2_quantity_Control_3 | Log2 MS2_quantity_Control_4 | Log2 MS2_quantity_Control_5 | Number Peptides for Quantitation NUP214_1 | Number Peptides for Quantitation NUP214_2 | Number Peptides for Quantitation NUP214_3 | Number Peptides for Quantitation Control_1 | Number Peptides for Quantitation Control_2 | Number Peptides for Quantitation Control_3 | Number Peptides for Quantitation Control_4 | Number Peptides for Quantitation Control_5 |
|---|---|---|---|---|---|---|---|---|---|---|---|---|---|---|---|---|---|---|---|---|---|---|---|---|
| CPNE2 | Q96FN4 | 9.65E-10 | 4.00E-10 | 0.506675 | + | 2.73127 | 5.28479 | | 16.1289 | 16.186 | 16.1645 | 15.8849 | 15.7358 | 15.4965 | 15.5283 | 15.6201 | 11 | 13 | 9 | 11 | 9 | 6 | 10 | 8 |
| RXRA | P19793;P1 | 0.000218 | 4.92E-05 | 0.507059 | + | 1.42769 | 2.66344 | | 13.9101 | 14.2778 | 13.8753 | 13.55 | 13.2067 | 13.5202 | 13.3487 | 13.9443 | 5 | 4 | 5 | 5 | 4 | 3 | 3 | 4 |
| BLMH | Q13867 | 1.06E-13 | 1.00E-13 | 0.50809 | + | 2.58523 | 4.94197 | | 17.2765 | 17.3319 | 17.3351 | 16.7187 | 16.7587 | 17.0665 | 16.8692 | 16.6188 | 15 | 15 | 14 | 13 | 12 | 14 | 14 | 14 |
| EXTL2 | Q9UBQ6 | 4.44E-08 | 1.49E-08 | 0.508113 | | 0.851598 | 1.69641 | | 14.7823 | 14.8779 | 14.7101 | 14.819 | 13.656 | 13.8576 | 14.5 | 14.5773 | 3 | 3 | 3 | 3 | 1 | 1 | 2 | 2 |
| TSPYL5 | Q86VY4 | 9.00E-10 | 3.75E-10 | 0.508379 | + | 1.47155 | 2.73937 | | 15.7074 | 15.6482 | 15.6642 | 14.7747 | 15.0986 | 15.5207 | 14.9947 | 15.4357 | 6 | 6 | 7 | 7 | 5 | 6 | 5 | 5 |
| STAT2 | P52630;P5 | 3.68E-14 | 3.95E-14 | 0.509469 | + | 2.24189 | 4.19311 | | 17.5524 | 17.5597 | 17.534 | 16.9105 | 17.133 | 17.3418 | 16.8249 | 16.9858 | 25 | 25 | 27 | 26 | 25 | 26 | 23 | 24 |
| CNTNAP1 | P78357 | 2.56E-13 | 2.18E-13 | 0.510297 | | 0.787486 | 1.58917 | | 16.8959 | 16.9335 | 16.8981 | 17.013 | 16.7179 | 15.7699 | 15.9016 | 16.592 | 32 | 34 | 32 | 33 | 26 | 26 | 26 | 26 |
| DDX39B | Q13838;Q1 | 3.14E-11 | 1.66E-11 | 0.512206 | + | 2.80248 | 5.45777 | | 19.0628 | 19.1297 | 19.0428 | 18.5951 | 18.5371 | 18.7748 | 18.3436 | 18.5804 | 8 | 8 | 8 | 8 | 8 | 8 | 8 | 8 |
| ALDH7A1 | P49419-2 | 1.12E-21 | 1.30E-20 | 0.512812 | | 1.14605 | 2.18626 | | 18.4895 | 18.5202 | 18.4989 | 17.8254 | 17.7004 | 18.6283 | 18.102 | 17.6942 | 29 | 29 | 29 | 28 | 26 | 29 | 29 | 28 |
| CYC1 | P08574 | 6.55E-08 | 2.15E-08 | 0.513672 | + | 2.27808 | 4.26857 | | 18.0144 | 17.9755 | 18.0004 | 17.388 | 17.5609 | 17.1759 | 17.6497 | 17.641 | 10 | 11 | 10 | 10 | 9 | 10 | 10 | 10 |
| INTS9 | Q9NV88;Q9 | 0.000799 | 0.000167 | 0.513829 | | 0.36991 | 0.852466 | | 14.3248 | 14.1773 | 14.3026 | 14.5826 | 12.1076 | 14.4726 | 13.5255 | 14.0839 | 3 | 4 | 3 | 2 | 1 | 2 | 3 | 2 |
| ACAD9 | Q9H845 | 1.77E-13 | 1.57E-13 | 0.51477 | | 1.00641 | 1.95382 | | 16.7681 | 16.7727 | 16.8038 | 16.6323 | 15.5132 | 16.5326 | 16.3103 | 16.3455 | 23 | 23 | 23 | 23 | 19 | 23 | 24 | 23 |
| MANEA | Q5SRI9 | 0.003827 | 0.000697 | 0.516089 | | 0.340806 | 0.807156 | | 14.2552 | 14.1949 | 14.2169 | 14.5815 | NaN | 12.1417 | 13.8911 | 14.2107 | 2 | 2 | 2 | 2 | NaN | 2 | 1 | 1 |
| NNMT | P40261 | 1.11E-06 | 3.21E-07 | 0.519253 | | 0.69951 | 1.44067 | | 20.6485 | 20.5835 | 20.6101 | 19.5218 | 19.9125 | 21.0977 | 20.146 | 19.7959 | 10 | 11 | 10 | 10 | 10 | 10 | 10 | 10 |
| PARP1 | P09874 | 6.93E-20 | 4.52E-19 | 0.519803 | + | 2.43114 | 4.59649 | | 16.7146 | 16.697 | 16.705 | 16.3065 | 16.2353 | 15.9768 | 16.0022 | 16.4079 | 47 | 46 | 48 | 48 | 42 | 43 | 42 | 47 |
| UGP2 | Q16851 | 1.06E-10 | 5.04E-11 | 0.520099 | + | 2.31611 | 4.3487 | | 19.163 | 19.2373 | 19.1924 | 18.592 | 18.5821 | 19.0239 | 18.6577 | 18.5314 | 35 | 35 | 35 | 33 | 33 | 35 | 34 | 34 |
| ELF1 | P32519;P3 | 0.003051 | 0.000568 | 0.523086 | + | 1.43378 | 3.59735 | | 14.6557 | 14.6769 | 14.3817 | NaN | NaN | 13.9437 | 14.1529 | NaN | 2 | 2 | 2 | NaN | NaN | 1 | 1 | NaN |
| TAP2 | Q03519 | 1.68E-12 | 1.14E-12 | 0.52309 | + | 2.88584 | 5.66531 | | 16.1868 | 16.2105 | 16.0899 | 15.4487 | 15.7299 | 15.8161 | 15.668 | 15.5338 | 12 | 12 | 11 | 11 | 12 | 11 | 11 | 12 |
| HAUS5 | O94927 | 0.003559 | 0.000652 | 0.523376 | | 0.662052 | 1.37678 | | 14.2469 | 13.8253 | 14.4669 | 14.125 | 14.2076 | 12.7968 | 13.3136 | 13.8387 | 3 | 3 | 4 | 4 | 2 | 1 | 2 | 4 |
| C16orf70 | Q9BSU1 | 3.74E-06 | 1.03E-06 | 0.525526 | + | 2.18324 | 4.07243 | | 15.4573 | 15.1689 | 15.2774 | 14.7874 | 14.6407 | 15.0917 | 14.7428 | 14.6158 | 6 | 5 | 5 | 5 | 4 | 5 | 5 | 5 |
| PRPF18 | Q99633 | 1.69E-06 | 4.79E-07 | 0.527465 | | 1.1848 | 2.25108 | | 13.2991 | 13.5774 | 13.3528 | 13.3249 | 12.277 | 12.9527 | 12.9652 | 12.8917 | 2 | 2 | 2 | 2 | 1 | 1 | 3 | 1 |
| MMP14 | P50281 | 7.90E-16 | 1.39E-15 | 0.528083 | + | 1.79657 | 3.3215 | | 18.8987 | 18.9093 | 18.8349 | 18.7934 | 18.1737 | 18.3819 | 18.1301 | 18.2854 | 19 | 18 | 19 | 18 | 17 | 18 | 17 | 17 |
| PXK | Q7Z7A4 | 1.68E-05 | 4.30E-06 | 0.528852 | + | 2.21428 | 4.13605 | | 16.4329 | 16.4936 | 16.4534 | 16.2167 | 16.0952 | 15.8372 | 15.7798 | 15.7267 | 14 | 15 | 14 | 14 | 12 | 15 | 12 | 12 |
| PCDH18 | Q9HCL0;Q9 | 2.97E-17 | 8.17E-17 | 0.529036 | + | 1.63026 | 3.01897 | | 15.093 | 14.9101 | 14.9587 | 14.5581 | 14.1607 | 14.7566 | 14.6676 | 14.1482 | 6 | 7 | 7 | 6 | 2 | 5 | 4 | 5 |
| AARS1 | P49588 | 8.75E-09 | 3.21E-09 | 0.529593 | + | 1.49156 | 2.77419 | | 19.1066 | 19.035 | 19.0752 | 18.2622 | 18.4198 | 19.0798 | 18.6326 | 18.3999 | 44 | 44 | 44 | 43 | 43 | 43 | 44 | 44 |
| TRAF2 | Q12933;Q1 | 0.000621 | 0.000132 | 0.530819 | | 1.04865 | 2.024 | | 14.2415 | 14.0988 | 14.1441 | 13.6227 | 12.8964 | 13.8039 | 13.7888 | 14.0416 | 3 | 4 | 3 | 1 | 2 | 3 | 3 | 4 |
| TIMM17B | O60830;O6 | 5.65E-11 | 2.84E-11 | 0.531315 | | 1.26925 | 2.39314 | | 15.5078 | 15.5669 | 15.6051 | 14.6582 | 14.601 | 15.2031 | 15.3897 | 15.2911 | 1 | 2 | 2 | 1 | 1 | 1 | 2 | 2 |
| SERPINB9 | P50453 | 0.000939 | 0.000195 | 0.531746 | | 0.496336 | 1.08665 | | 13.8663 | 13.7874 | 13.9256 | 13.7232 | 13.1844 | 13.6234 | 11.989 | 14.12 | 4 | 6 | 4 | 4 | 2 | 4 | 2 | 4 |
| ADD3 | Q9UEY8-2 | 4.61E-19 | 2.28E-18 | 0.532519 | + | 1.76013 | 3.25427 | | 18.9338 | 18.9366 | 18.9107 | 18.3345 | 18.3031 | 18.8518 | 18.1131 | 18.3701 | 21 | 21 | 23 | 20 | 17 | 21 | 20 | 21 |
| MPC2 | O95563 | 1.46E-11 | 8.20E-12 | 0.533081 | + | 1.6078 | 2.9789 | | 16.7383 | 16.7295 | 16.7516 | 16.5173 | 15.7129 | 16.2995 | 16.1929 | 16.311 | 4 | 4 | 4 | 4 | 3 | 4 | 4 | 4 |
| OARD1 | Q9Y530 | 8.48E-05 | 2.02E-05 | 0.533205 | + | 1.94854 | 3.608 | | 14.0876 | 14.304 | 14.1915 | 13.7705 | 13.6812 | 13.4963 | 13.3771 | 13.9806 | 4 | 4 | 5 | 3 | 4 | 3 | 3 | 5 |
| RUNX1 | Q01196;Q0 | 0.001292 | 0.000262 | 0.533986 | | 0.988165 | 1.9937 | | 15.0669 | 15.178 | 15.0573 | NaN | 14.2707 | 15.1241 | 14.1416 | 14.7305 | 3 | 3 | 3 | NaN | 3 | 4 | 3 | 3 |
| INPP5K | Q9BT40;Q9 | 2.73E-14 | 3.05E-14 | 0.535615 | + | 2.33223 | 4.38293 | | 15.9047 | 15.8741 | 15.9876 | 15.2385 | 15.2711 | 15.7209 | 15.427 | 15.275 | 6 | 5 | 5 | 5 | 4 | 5 | 5 | 5 |
| SEC23B | Q15437 | 3.45E-12 | 2.19E-12 | 0.535625 | | 1.12547 | 2.15192 | | 15.9217 | 15.9016 | 15.8949 | 14.7824 | 15.4881 | 15.1178 | 15.8086 | 15.6553 | 14 | 14 | 13 | 10 | 10 | 11 | 14 | 11 |
| PFDN2 | Q9UHV9 | 1.26E-14 | 1.55E-14 | 0.535917 | | 0.720747 | 1.4767 | | 17.9654 | 17.9172 | 18.108 | 18.1365 | 17.0993 | 16.653 | 17.4843 | 17.9317 | 8 | 8 | 8 | 8 | 6 | 8 | 8 | 8 |
| MTCL1 | Q9Y4B5 | 8.61E-20 | 5.40E-19 | 0.536081 | + | 1.99192 | 3.69173 | | 15.5219 | 15.5583 | 15.5372 | 14.6685 | 15.2933 | 15.1315 | 14.8601 | 15.0619 | 31 | 37 | 36 | 27 | 22 | 30 | 25 | 26 |
| COL6A1 | P12109 | 3.00E-10 | 1.33E-10 | 0.536713 | + | 1.70974 | 3.16221 | | 20.4538 | 20.5385 | 20.4972 | 19.8498 | 20.3644 | 20.1304 | 19.7876 | 19.6667 | 43 | 42 | 43 | 40 | 37 | 43 | 37 | 39 |
| OXCT1 | P55809 | 5.47E-11 | 2.76E-11 | 0.539391 | + | 2.86708 | 5.61812 | | 17.83 | 17.8324 | 17.8193 | 17.5221 | 17.0968 | 17.3578 | 17.2094 | 17.2531 | 16 | 16 | 16 | 16 | 16 | 15 | 15 | 16 |
| ADARB1 | P78563;P7 | 0.001454 | 0.000293 | 0.54043 | | 0.95186 | 1.86323 | | 14.7949 | 14.8148 | 14.6491 | 13.8948 | 13.7424 | 14.9913 | 14.2452 | 14.1888 | 1 | 1 | 2 | 1 | 1 | 2 | 1 | 1 |
| SLC31A1 | O15431 | 7.57E-05 | 1.82E-05 | 0.542391 | | 0.567583 | 1.21317 | | 15.2387 | 15.2117 | 15.2011 | 14.1818 | 13.9557 | 15.6151 | 15.343 | 14.2784 | 3 | 3 | 3 | 3 | 2 | 3 | 3 | 2 |
| HNRNPF | P52597 | 5.44E-10 | 2.33E-10 | 0.54305 | + | 2.26742 | 4.24627 | | 18.7288 | 18.6925 | 18.6449 | 18.3734 | 17.8636 | 18.2711 | 17.9861 | 18.2342 | 9 | 9 | 9 | 9 | 9 | 9 | 9 | 10 |
| PAXX | Q9BUH6 | 2.16E-12 | 1.44E-12 | 0.543903 | + | 3.11726 | 6.27201 | | 16.7809 | 16.7117 | 16.8508 | 16.0392 | 16.3242 | 16.3997 | 16.1967 | 16.2263 | 8 | 8 | 8 | 8 | 8 | 7 | 8 | 7 |
| HLA-E | P13747 | 1.59E-13 | 1.43E-13 | 0.544276 | + | 1.88553 | 3.48796 | | 14.4452 | 14.219 | 14.5128 | 13.7949 | 13.4991 | 13.8722 | 13.9145 | 14.1595 | 4 | 3 | 3 | 4 | 1 | 4 | 3 | 4 |
| CHTOP | Q9Y3Y2;Q9 | 3.70E-05 | 9.18E-06 | 0.548119 | + | 1.52188 | 2.82717 | | 15.9681 | 16.1879 | 15.9335 | 15.875 | 15.2512 | 15.284 | 15.2372 | 15.7612 | 3 | 3 | 3 | 3 | 3 | 3 | 3 | 3 |
| CLIC6;CLIC6;CLIC5;CL | Q96NY7;Q9 | 5.29E-09 | 1.99E-09 | 0.54953 | | 0.593167 | 1.25788 | | 18.8919 | 18.8586 | 19.4201 | 19.2365 | 18.3927 | 17.804 | 17.879 | 19.2245 | 2 | 2 | 2 | 2 | 2 | 1 | 1 | 2 |
| SUN2 | Q9UH99;Q | 5.75E-14 | 5.86E-14 | 0.550512 | + | 1.52723 | 2.83656 | | 17.2213 | 17.2908 | 17.2759 | 17.1669 | 16.2836 | 16.8419 | 16.6626 | 16.6057 | 19 | 20 | 20 | 20 | 19 | 20 | 20 | 19 |
| LMNB1 | P20700 | 2.33E-15 | 3.59E-15 | 0.551229 | + | 5.38849 | 15.7924 | | 18.0027 | 18.0113 | 17.9857 | 17.3727 | 17.4933 | 17.5007 | 17.4017 | 17.4749 | 35 | 34 | 35 | 33 | 35 | 34 | 34 | 35 |
| GUSB | P08236;P0 | 6.54E-08 | 2.15E-08 | 0.551817 | + | 1.50257 | 2.7934 | | 16.382 | 16.38 | 16.43 | 15.7466 | 15.4481 | 16.3643 | 15.8224 | 15.8461 | 6 | 6 | 6 | 5 | 2 | 6 | 3 | 4 |
| FLYWCH2 | Q96CP2 | 0.00051 | 0.000109 | 0.55209 | | 1.13044 | 2.1602 | | 14.9115 | 14.7601 | 15.1142 | 14.6931 | 14.5916 | 14.199 | 13.7352 | 14.6635 | 5 | 6 | 6 | 6 | 6 | 3 | 5 | 6 |
| MPC1 | Q9Y5U8 | 0.005466 | 0.00097 | 0.552946 | | 0.531611 | 1.26876 | | 15.1864 | 14.4659 | 14.4045 | 14.5236 | NaN | NaN | NaN | 13.7418 | 2 | 2 | 2 | 2 | NaN | NaN | NaN | 2 |
| DDB2 | Q92466 | 0.000104 | 2.46E-05 | 0.554936 | + | 2.22219 | 4.15236 | | 15.9904 | 16.0698 | 16.0262 | 15.4128 | 15.6531 | 15.7305 | 15.1729 | 15.4 | 9 | 11 | 10 | 7 | 7 | 9 | 8 | 7 |
| CHKB | Q9Y259 | 9.01E-05 | 2.15E-05 | 0.555063 | + | 2.3699 | 4.46356 | | 15.5899 | 15.6465 | 15.628 | 14.8672 | 15.3701 | 15.1879 | 14.9578 | 14.9489 | 6 | 6 | 6 | 4 | 6 | 6 | 6 | 5 |
| XPOT | O43592 | 1.81E-11 | 1.00E-11 | 0.5575 | | 1.05463 | 2.03395 | | 17.8203 | 17.8936 | 17.8468 | 16.9538 | 16.9796 | 18.0207 | 17.4843 | 17.042 | 28 | 28 | 28 | 27 | 27 | 28 | 28 | 28 |
| SLC35C2 | Q9NQQ7;Q | 0.010602 | 0.001814 | 0.557782 | | 0.978733 | 2.08841 | | 16.3272 | 15.6333 | 15.682 | 15.0317 | 15.4506 | NaN | NaN | 15.4869 | 1 | 1 | 2 | 1 | 1 | NaN | NaN | 1 |
| RYBP | Q8N488 | 6.60E-12 | 3.97E-12 | 0.558215 | + | 2.1718 | 4.04911 | | 15.0819 | 15.0659 | 15.0754 | 14.1326 | 14.6688 | 14.6665 | 14.4634 | 14.6498 | 2 | 2 | 3 | 2 | 1 | 2 | 3 | 2 |
| PBX1 | P40424;P4 | 5.86E-05 | 1.43E-05 | 0.558255 | | 1.08203 | 2.07953 | | 15.1584 | 15.0348 | 15.0515 | 13.9365 | 14.8676 | 15.0163 | 14.2186 | 14.5774 | 5 | 6 | 6 | 4 | 3 | 5 | 3 | 3 |
| ALDOC | P09972 | 1.49E-17 | 4.47E-17 | 0.559029 | + | 4.81511 | 12.5994 | | 18.5737 | 18.5502 | 18.5085 | 17.9149 | 18.0463 | 18.0704 | 17.9705 | 17.9234 | 15 | 15 | 15 | 15 | 15 | 15 | 15 | 14 |
| MYH11 | P35749;P3 | 0.000927 | 0.000192 | 0.560184 | | 1.15373 | 2.19911 | | 21.4697 | 21.5542 | 21.6743 | 21.3065 | 20.353 | 21.4353 | 20.9184 | 21.016 | 2 | 3 | 3 | 3 | 2 | 3 | 3 | 3 |
| SDR39U1 | Q9NRG7 | 0.002632 | 0.0005 | 0.560666 | | 0.912586 | 1.79797 | | 13.6932 | 13.7886 | 13.555 | 13.0842 | 12.6032 | 13.8822 | 12.6999 | 13.3217 | 2 | 2 | 2 | 1 | 1 | 2 | 1 | 2 |
| HAUS3 | Q68CZ6 | 0.000664 | 0.00014 | 0.560847 | | 0.733891 | 1.60139 | | 14.4233 | 14.3481 | 14.2272 | 14.4559 | 13.3438 | NaN | 13.5164 | NaN | 3 | 3 | 2 | 2 | 2 | NaN | 1 | NaN |
| GJA1 | P17302 | 2.78E-10 | 1.24E-10 | 0.560856 | | 0.604039 | 1.27679 | | 15.8887 | 15.9191 | 15.9264 | 15.1109 | 15.8342 | 15.5361 | 14.1998 | 16.0717 | 10 | 9 | 11 | 9 | 11 | 11 | 8 | 10 |
| THYN1 | Q9P016 | 1.59E-05 | 4.08E-06 | 0.561467 | + | 2.92419 | 5.7627 | | 15.946 | 16.0622 | 16.0147 | 15.3277 | 15.3368 | 15.6481 | 15.5873 | 15.331 | 8 | 8 | 8 | 8 | 6 | 7 | 7 | 6 |
| HTRA1 | Q92743 | 1.28E-12 | 9.02E-13 | 0.561714 | | 1.12405 | 2.14954 | | 17.3597 | 17.55 | 17.4664 | 16.4259 | 16.7486 | 16.8837 | 17.6043 | 16.8224 | 9 | 10 | 9 | 8 | 7 | 9 | 9 | 9 |
| SRBD1 | Q8N5C6 | 0.000162 | 3.74E-05 | 0.561932 | + | 2.17702 | 4.05975 | | 16.1771 | 16.1058 | 16.2572 | 15.3205 | 15.9133 | 15.617 | 15.5008 | 15.7389 | 8 | 6 | 8 | 4 | 4 | 5 | 4 | 4 |
| TAP1 | Q03518 | 2.92E-13 | 2.45E-13 | 0.564179 | + | 2.37178 | 4.4676 | | 16.7956 | 16.7863 | 16.7869 | 16.018 | 16.13 | 16.4629 | 16.4434 | 16.0728 | 12 | 12 | 11 | 11 | 12 | 12 | 12 | 12 |
| RAB3IL1 | Q8TBN0 | 3.08E-08 | 1.06E-08 | 0.565563 | + | 3.33288 | 6.88114 | | 15.8831 | 15.8686 | 15.948 | 15.2763 | 15.1555 | 15.4681 | 15.4691 | 15.3027 | 4 | 5 | 5 | 3 | 2 | 3 | 4 | 2 |
| CMSS1 | Q9BQ75;Q9 | 4.84E-05 | 1.19E-05 | 0.565623 | | 0.698344 | 1.43869 | | 14.8756 | 15.3225 | 14.2457 | 13.6487 | 13.9403 | 14.2473 | 15.0807 | 14.3279 | 3 | 2 | 3 | 1 | 3 | 2 | 2 | 3 |
| PCOLCE | Q15113 | 1.20E-12 | 8.53E-13 | 0.567756 | + | 2.00835 | 3.72369 | | 18.3814 | 18.3737 | 18.4281 | 17.6338 | 18.0409 | 18.1585 | 17.6957 | 17.6043 | 13 | 13 | 13 | 14 | 13 | 13 | 13 | 13 |
| ATP5PB | P24539 | 6.13E-12 | 3.72E-12 | 0.57121 | | 0.572417 | 1.22164 | | 17.8103 | 17.815 | 17.8322 | 17.8489 | 16.7013 | 16.153 | 17.5968 | 17.9399 | 14 | 14 | 14 | 12 | 11 | 12 | 12 | 13 |
| KHDRBS3 | O75525;O7 | 1.98E-12 | 1.33E-12 | 0.572286 | + | 2.10359 | 3.9116 | | 15.81 | 15.7673 | 15.7468 | 15.1911 | 15.4751 | 14.8188 | 15.194 | 15.3331 | 6 | 7 | 6 | 6 | 4 | 4 | 5 | 5 |
| COQ6 | Q9Y2Z9;Q9 | 2.72E-06 | 7.56E-07 | 0.574093 | | 1.27238 | 2.39842 | | 16.0625 | 16.1303 | 16.0038 | 15.8325 | 15.5332 | 15.6591 | 14.8046 | 15.6278 | 2 | 2 | 3 | 2 | 2 | 2 | 2 | 2 |
| CD81 | P60033 | 1.05E-05 | 2.76E-06 | 0.574742 | + | 1.54363 | 2.86537 | | 20.6165 | 20.6586 | 20.6988 | 19.5141 | 20.2484 | 20.1365 | 20.3883 | 20.1287 | 5 | 3 | 3 | 3 | 3 | 5 | 4 | 4 |
| SMC6 | Q96SB8;Q9 | 0.006825 | 0.001197 | 0.576344 | | 0.333176 | 0.836372 | | NaN | 12.8555 | 12.3727 | 13.0437 | NaN | NaN | 11.3409 | 11.7288 | NaN | 1 | 2 | 1 | NaN | NaN | 2 | 2 |
| ANTXR1 | Q9H6X2;Q9 | 1.71E-18 | 6.96E-18 | 0.576736 | + | 1.82907 | 3.38191 | | 15.9894 | 16.0825 | 16.0305 | 15.3004 | 15.1846 | 15.7682 | 15.7619 | 15.2719 | 6 | 6 | 7 | 6 | 6 | 6 | 5 | 6 |
| HDAC6 | Q9UBN7 | 7.35E-07 | 2.17E-07 | 0.577007 | + | 2.11115 | 3.92671 | | 15.8352 | 15.8334 | 15.8366 | 15.0871 | 15.1658 | 15.6469 | 15.347 | 15.0434 | 8 | 8 | 8 | 7 | 7 | 8 | 9 | 7 |
| ANGPTL2 | Q9UKU9 | 8.36E-07 | 2.45E-07 | 0.577438 | + | 2.44648 | 4.63017 | | 16.0977 | 15.9987 | 16.0677 | 15.4794 | 15.769 | 15.4166 | 15.1966 | 15.5246 | 11 | 12 | 11 | 6 | 8 | 6 | 7 | 7 |
| LAMB1 | P07942 | 2.34E-14 | 2.66E-14 | 0.578273 | | 0.847687 | 1.68989 | | 16.954 | 16.9548 | 16.9739 | 16.5133 | 16.2993 | 15.5554 | 17.1629 | 16.3823 | 37 | 36 | 35 | 35 | 35 | 33 | 37 | 34 |
| SHPK | Q9UHJ6 | 4.29E-14 | 4.54E-14 | 0.579628 | + | 3.1201 | 6.27975 | | 15.865 | 15.8689 | 15.8403 | 15.341 | 15.3875 | 15.307 | 15.007 | 15.3496 | 10 | 9 | 10 | 8 | 7 | 8 | 5 | 7 |

| Gene names | UniProt IDs | Protein Group P-value | Protein Group Q-value | Log2 Difference NUP214 vs Controls (n=5) | p<0.05 | -log10 p-value | T-test Test statistic | NPC Localization | Log2 MS2_quantity_ NUP214_1 | Log2 MS2_quantity_ NUP214_2 | Log2 MS2_quantity_ NUP214_3 | Log2 MS2_quantity_ Control_1 | Log2 MS2_quantity_ Control_2 | Log2 MS2_quantity_ Control_3 | Log2 MS2_quantity_ Control_4 | Log2 MS2_quantity_ Control_5 | Number Peptides for Quantitation NUP214_1 | Number Peptides for Quantitation NUP214_2 | Number Peptides for Quantitation NUP214_3 | Number Peptides for Quantitation Control_1 | Number Peptides for Quantitation Control_2 | Number Peptides for Quantitation Control_3 | Number Peptides for Quantitation Control_4 | Number Peptides for Quantitation Control_5 |
|---|---|---|---|---|---|---|---|---|---|---|---|---|---|---|---|---|---|---|---|---|---|---|---|---|
| EDEM2 | Q9BV94;Q9 | 0.000487 | 0.000105 | 0.580056 | | 0.655589 | 1.36571 | | 15.3283 | 15.356 | 14.7523 | 15.2035 | 13.4765 | 14.4611 | 14.703 | 14.9831 | 1 | 2 | 2 | 2 | 1 | 2 | 2 | 1 |
| FBXO17 | Q96EF6 | 0.000395 | 8.63E-05 | 0.583043 | | 0.599253 | 1.26847 | | 14.8928 | 14.8187 | 14.8391 | 14.1752 | 14.4849 | 15.0402 | 14.6291 | 13.0064 | 3 | 3 | 2 | 1 | 3 | 2 | 2 | 1 |
| DTX3L | Q8TDB6 | 2.39E-16 | 4.96E-16 | 0.585952 | | 1.29908 | 2.44361 | | 14.849 | 14.8634 | 14.8104 | 14.1246 | 14.5376 | 14.7485 | 13.7125 | 14.1519 | 12 | 14 | 13 | 9 | 9 | 12 | 8 | 8 |
| ZNF706 | Q9Y5V0 | 1.71E-07 | 5.41E-08 | 0.586163 | | 1.05665 | 2.03731 | | 18.2803 | 18.5953 | 18.3155 | 18.1478 | 17.573 | 17.1408 | 17.8846 | 18.308 | 2 | 1 | 1 | 1 | 1 | 1 | 1 | 2 |
| RCE1 | Q9Y256 | 9.84E-06 | 2.59E-06 | 0.586449 | + | 3.19399 | 6.48371 | | 15.7039 | 15.6911 | 15.5903 | 15.0769 | 15.307 | 14.9757 | 14.9318 | 15.0853 | 2 | 2 | 2 | 2 | 1 | 1 | 1 | 2 |
| PCCA | P05165;P0 | 3.91E-08 | 1.32E-08 | 0.586518 | + | 3.1192 | 6.27731 | | 15.2785 | 15.2796 | 15.2265 | 14.5847 | 14.7905 | 14.8889 | 14.5725 | 14.5386 | 14 | 14 | 13 | 12 | 8 | 12 | 11 | 6 |
| TMEM14C | Q9P0S9 | 4.05E-09 | 1.55E-09 | 0.586543 | + | 1.35005 | 2.53027 | | 18.0543 | 17.9768 | 17.9975 | 17.6708 | 16.8789 | 17.8342 | 17.1808 | 17.5501 | 3 | 3 | 3 | 3 | 3 | 3 | 3 | 3 |
| ALDH3A2 | P51648;P5 | 1.93E-17 | 5.60E-17 | 0.587737 | + | 1.45404 | 2.70899 | | 16.3154 | 16.2168 | 16.2915 | 15.0773 | 16.0011 | 15.6698 | 15.8928 | 15.7931 | 10 | 10 | 10 | 8 | 10 | 9 | 9 | 9 |
| HAUS4 | Q9H6D7 | 0.054554 | 0.008843 | 0.58881 | | 0.369691 | 0.883474 | | 13.9351 | 13.2167 | 13.6469 | 13.5817 | NaN | 11.7469 | NaN | 13.7038 | 2 | 1 | 4 | 1 | NaN | 2 | NaN | 2 |
| ALDH1L2 | Q3SY69 | 8.85E-19 | 3.89E-18 | 0.590265 | | 0.513527 | 1.11747 | | 17.9835 | 17.9966 | 17.9748 | 16.2438 | 17.1518 | 18.4592 | 18.0921 | 17.0265 | 44 | 44 | 45 | 39 | 38 | 45 | 44 | 42 |
| GGACT | Q9BVM4 | 1.52E-05 | 3.92E-06 | 0.590309 | | 0.873168 | 1.78738 | | 14.247 | 14.9813 | 14.952 | 14.4091 | NaN | 14.5348 | 14.0571 | 13.5449 | 2 | 1 | 1 | 2 | NaN | 1 | 1 | 1 |
| GAB1 | Q13480;Q1 | 5.96E-10 | 2.54E-10 | 0.591267 | + | 1.79833 | 3.32476 | | 14.2813 | 14.1837 | 14.2163 | 13.4437 | 13.4752 | 13.9371 | 13.3484 | 13.9746 | 3 | 3 | 3 | 1 | 1 | 3 | 3 | 2 |
| TTC26 | A0AVF1;A0 | 0.016369 | 0.002745 | 0.592596 | | 1.03745 | 2.44929 | | 14.4877 | 14.6389 | 14.5446 | NaN | NaN | 14.28 | NaN | 13.649 | 1 | 2 | 2 | NaN | NaN | 1 | NaN | 1 |
| WDR91 | A4D1P6 | 1.06E-11 | 6.11E-12 | 0.593339 | + | 2.2077 | 4.12253 | | 15.6048 | 15.7441 | 15.6093 | 14.7122 | 15.255 | 15.1793 | 15.2281 | 14.9222 | 9 | 10 | 5 | 8 | 6 | 5 | 7 | 4 |
| PLGRKT | Q9HBL7 | 4.60E-06 | 1.25E-06 | 0.594197 | | 1.18862 | 2.52846 | | 16.4507 | 16.4177 | 16.5095 | 16.153 | NaN | 15.4028 | NaN | 16.0394 | 4 | 4 | 4 | 4 | NaN | 3 | NaN | 4 |
| CDK6 | Q00534 | 3.20E-10 | 1.41E-10 | 0.594855 | + | 4.01649 | 9.14364 | | 17.9599 | 17.9559 | 17.897 | 17.2728 | 17.2494 | 17.4157 | 17.4932 | 17.2828 | 10 | 10 | 10 | 9 | 6 | 8 | 8 | 7 |
| PLCB1 | Q9NQ66;Q | 2.30E-10 | 1.04E-10 | 0.596115 | | 0.659272 | 1.37202 | | 14.674 | 14.652 | 14.6443 | 13.5658 | 14.4246 | 13.1169 | 14.9707 | 14.2252 | 4 | 4 | 3 | 4 | 3 | 3 | 5 | 2 |
| RAB31 | Q13636 | 4.98E-08 | 1.66E-08 | 0.598969 | + | 2.20395 | 4.11482 | | 18.093 | 18.2556 | 18.1201 | 17.6758 | 17.1519 | 17.6548 | 17.5587 | 17.7452 | 11 | 11 | 11 | 11 | 9 | 10 | 11 | 11 |
| ROR1 | Q01973 | 3.51E-05 | 8.74E-06 | 0.602681 | | 0.838334 | 1.72513 | | 14.5442 | 14.9985 | 14.1264 | 13.4077 | 13.727 | 14.2456 | NaN | 14.4344 | 4 | 2 | 2 | 1 | 1 | 1 | NaN | 2 |
| GANAB | Q14697-2 | 3.40E-08 | 1.16E-08 | 0.604391 | | 0.401387 | 0.912192 | | 17.2199 | 17.4548 | 17.3453 | 17.1491 | 14.8007 | 17.4264 | 17.4458 | 16.856 | 2 | 2 | 2 | 2 | 2 | 2 | 2 | 2 |
| ACOT1 | Q86TX2 | 4.08E-17 | 1.08E-16 | 0.605093 | + | 2.04748 | 3.80033 | | 17.289 | 17.3672 | 17.3167 | 16.3595 | 16.5503 | 16.8429 | 16.8058 | 17.0375 | 17 | 17 | 16 | 18 | 14 | 16 | 17 | 17 |
| MEF2A | Q02078;Q0 | 0.001497 | 0.000301 | 0.60572 | | 0.734909 | 1.50066 | | 13.9971 | 14.0199 | 13.7215 | 13.6417 | 14.1585 | 12.3512 | 13.1739 | 13.2103 | 2 | 2 | 2 | 2 | 1 | 2 | 2 | 2 |
| NRM | Q8IXM6 | 0.00041 | 8.94E-05 | 0.605733 | + | 2.17645 | 4.05857 | | 16.7527 | 16.5142 | 16.6837 | 16.2404 | 15.8394 | 15.7433 | 16.2238 | 16.1755 | 4 | 4 | 4 | 3 | 3 | 3 | 4 | 4 |
| H2AC11;H2AC7;H2AC | P0C0S8;P2 | 0.001195 | 0.000244 | 0.609915 | + | 2.43663 | 4.60855 | | 24.9007 | 24.7748 | 24.7815 | 24.191 | 24.4023 | 24.039 | 23.9612 | 24.4519 | 5 | 5 | 5 | 5 | 5 | 5 | 5 | 5 |
| COL6A2 | P12110 | 6.37E-13 | 4.83E-13 | 0.613457 | + | 2.87775 | 5.64492 | | 19.8016 | 19.9377 | 19.8631 | 19.2075 | 19.5202 | 19.3325 | 19.1068 | 19.1032 | 38 | 38 | 38 | 35 | 36 | 36 | 32 | 35 |
| MPV17 | P39210 | 0.001027 | 0.000211 | 0.613799 | + | 1.49571 | 2.94861 | | 15.2215 | 15.5351 | 15.2665 | 15.0411 | NaN | 14.3377 | 14.9395 | 14.5907 | 2 | 1 | 3 | 1 | NaN | 1 | 2 | 1 |
| RAB30 | Q15771 | 0.01307 | 0.002212 | 0.613875 | | 0.134946 | 0.391952 | | 15.218 | 15.3229 | NaN | NaN | 16.2219 | NaN | 13.0913 | NaN | 1 | 1 | NaN | NaN | 1 | NaN | 2 | NaN |
| TXNL4A | P83876 | 7.02E-10 | 2.95E-10 | 0.614296 | + | 1.46171 | 2.7223 | | 15.6292 | 15.4516 | 15.6974 | 15.2245 | 14.467 | 14.7095 | 15.2038 | 15.2873 | 2 | 3 | 2 | 2 | 3 | 2 | 3 | 3 |
| KNOP1 | Q1ED39 | 1.18E-05 | 3.08E-06 | 0.615667 | | 0.295311 | 0.72857 | | 13.482 | 13.8357 | 13.2442 | 14.3657 | NaN | 11.5013 | NaN | 12.8478 | 3 | 2 | 3 | 2 | NaN | 2 | NaN | 3 |
| LTF | P02788;P0 | 0.000425 | 9.24E-05 | 0.615724 | | 0.890913 | 1.76191 | | 18.7768 | 18.847 | 18.8231 | 18.8794 | 18.1261 | 18.6783 | 17.4484 | 17.8674 | 4 | 5 | 4 | 8 | 5 | 6 | 6 | 7 |
| ARMC9 | Q7Z3E5 | 1.86E-09 | 7.42E-10 | 0.618355 | | 0.773918 | 1.56639 | | 17.453 | 17.4442 | 17.4526 | 16.7394 | 16.6173 | 16.2493 | 17.9705 | 16.5815 | 25 | 26 | 25 | 24 | 21 | 23 | 27 | 24 |
| SPOCK1 | Q08629 | 0.001326 | 0.000269 | 0.618721 | | 0.63231 | 1.32569 | | 16.208 | 16.4238 | 16.3838 | 15.546 | 14.7058 | 16.6425 | 16.3419 | 15.3628 | 3 | 3 | 3 | 1 | 1 | 3 | 2 | 1 |
| PSPH | P78330 | 1.29E-12 | 9.05E-13 | 0.620734 | | 0.766269 | 1.55353 | | 18.166 | 18.2532 | 18.2552 | 16.9827 | 17.4047 | 18.3057 | 18.3215 | 17.0057 | 8 | 8 | 8 | 8 | 7 | 8 | 9 | 7 |
| COL18A1 | P39060;P3 | 5.36E-11 | 2.71E-11 | 0.622431 | | 0.49057 | 1.07627 | | 17.1063 | 17.1077 | 17.1163 | 15.6431 | 17.444 | 15.5559 | 17.5797 | 16.2154 | 17 | 17 | 16 | 16 | 16 | 14 | 18 | 15 |
| PXMP2 | Q9NR77 | 8.28E-08 | 2.69E-08 | 0.623411 | + | 5.84552 | 18.8784 | | 16.6619 | 16.7475 | 16.6122 | 16.0207 | 16.0287 | 16.0751 | 16.0468 | 16.0809 | 5 | 5 | 5 | 4 | 5 | 5 | 5 | 5 |
| PYCR1 | P32322;P3 | 2.11E-19 | 1.18E-18 | 0.624273 | | 1.01808 | 1.97321 | | 18.2634 | 18.3379 | 18.2705 | 17.0231 | 17.7495 | 18.2397 | 18.0918 | 17.2279 | 14 | 13 | 14 | 13 | 14 | 13 | 13 | 13 |
| DHODH | Q02127 | 0.000375 | 8.22E-05 | 0.625371 | | 1.26742 | 2.39005 | | 14.9326 | 14.8228 | 14.887 | 13.6772 | 14.439 | 13.9148 | 14.5532 | 14.6929 | 6 | 9 | 7 | 3 | 2 | 4 | 4 | 6 |
| SDHC | Q99643;Q9 | 2.16E-05 | 5.49E-06 | 0.626813 | + | 2.65714 | 5.10883 | | 16.8692 | 16.846 | 17.0818 | 16.5454 | 16.039 | 16.3333 | 16.2491 | 16.3609 | 2 | 2 | 2 | 2 | 2 | 2 | 2 | 2 |
| LRRC32 | Q14392 | 2.61E-16 | 5.33E-16 | 0.626964 | | 1.07203 | 2.14559 | | 15.4562 | 15.3375 | 15.4204 | 14.8789 | 14.8002 | 15.31 | NaN | 14.1217 | 10 | 9 | 9 | 6 | 2 | 9 | NaN | 3 |
| MTHFS | P49914;P4 | 0.002703 | 0.000511 | 0.628807 | + | 1.60508 | 2.97406 | | 14.9892 | 14.8964 | 15.059 | 13.9873 | 14.1441 | 14.1841 | 14.7892 | 14.6587 | 2 | 3 | 3 | 3 | 1 | 2 | 2 | 3 |
| PARP14 | Q460N5 | 1.76E-07 | 5.55E-08 | 0.630368 | | 1.22337 | 2.31583 | | 15.012 | 14.9592 | 14.8713 | 14.2418 | 14.7757 | 14.6591 | 13.6172 | 14.2919 | 18 | 19 | 20 | 14 | 13 | 13 | 11 | 11 |
| PPP1R11 | O60927 | 1.42E-09 | 5.76E-10 | 0.630477 | | 0.557286 | 1.19507 | | 14.4353 | 14.5962 | 14.5366 | 14.7339 | 12.6437 | 14.7302 | 13.51 | 13.8434 | 3 | 3 | 3 | 3 | 3 | 3 | 3 | 3 |
| H1-2 | P16403 | 3.61E-10 | 1.58E-10 | 0.631669 | | 1.01578 | 1.96938 | | 21.6968 | 21.5634 | 21.5037 | 20.6679 | 21.8379 | 20.8012 | 20.4568 | 21.0177 | 11 | 10 | 11 | 10 | 11 | 10 | 10 | 10 |
| SNU13 | P55769 | 1.24E-20 | 1.09E-19 | 0.633113 | + | 1.49576 | 2.78151 | | 18.0708 | 18.0833 | 18.0583 | 17.6945 | 17.0995 | 16.9872 | 17.5335 | 17.8737 | 7 | 7 | 7 | 7 | 7 | 7 | 7 | 7 |
| H1-10 | Q92522 | 0.000529 | 0.000113 | 0.633795 | + | 3.7978 | 8.35957 | | 17.7554 | 17.7699 | 17.6 | 17.0248 | 17.0677 | 17.1868 | 17.17 | 16.9238 | 4 | 5 | 5 | 3 | 4 | 4 | 4 | 4 |
| PTMS | P20962 | 1.84E-08 | 6.48E-09 | 0.634846 | | 1.19393 | 2.2664 | | 19.3546 | 20.1039 | 19.6037 | 19.3546 | 19.3332 | 18.406 | 19.0574 | 19.1115 | 3 | 3 | 3 | 2 | 3 | 2 | 2 | 2 |
| GSDMD | P57764 | 2.24E-08 | 7.79E-09 | 0.635143 | | 1.13858 | 2.1738 | | 16.2222 | 16.3344 | 16.36 | 15.1831 | 16.1203 | 16.252 | 15.2901 | 15.5064 | 10 | 10 | 10 | 8 | 8 | 10 | 7 | 8 |
| LSM6 | P62312 | 2.80E-06 | 7.76E-07 | 0.639148 | + | 1.7467 | 3.22964 | | 17.7382 | 17.7437 | 17.6398 | 17.4357 | 16.7878 | 16.7565 | 16.9589 | 17.4014 | 5 | 5 | 5 | 5 | 5 | 5 | 5 | 5 |
| CTSZ | Q9UBR2 | 3.96E-06 | 1.08E-06 | 0.640915 | | 0.788348 | 1.59062 | | 19.1478 | 19.2447 | 19.1993 | 17.7409 | 19.1944 | 18.4305 | 19.2944 | 18.1215 | 10 | 10 | 10 | 10 | 10 | 9 | 10 | 8 |
| GBP1 | P32455 | 4.46E-19 | 2.21E-18 | 0.645102 | + | 2.79451 | 5.43822 | | 18.2327 | 18.2361 | 18.2265 | 17.4888 | 17.5679 | 17.9262 | 17.4112 | 17.5392 | 21 | 21 | 21 | 22 | 18 | 20 | 21 | 21 |
| PTK7 | Q13308;Q1 | 9.56E-09 | 3.49E-09 | 0.649229 | + | 1.5688 | 2.90974 | | 18.0648 | 18.0766 | 18.0684 | 16.7649 | 17.5722 | 17.699 | 17.5783 | 17.4891 | 40 | 43 | 41 | 35 | 39 | 38 | 40 | 39 |
| ACE | P12821;P1 | 9.78E-06 | 2.57E-06 | 0.649468 | + | 1.70321 | 3.15036 | | 15.5223 | 15.7014 | 15.5923 | 14.5189 | 14.6757 | 15.2022 | 15.0893 | 15.2933 | 11 | 12 | 11 | 4 | 5 | 6 | 8 | 8 |
| PSMB8 | P28062;P2 | 9.07E-14 | 8.74E-14 | 0.653397 | + | 1.86497 | 3.44917 | | 17.7071 | 17.7684 | 17.755 | 16.6551 | 17.1717 | 17.5305 | 17.1099 | 16.9832 | 9 | 9 | 9 | 8 | 9 | 9 | 9 | 9 |
| IFI35 | P80217;P8 | 3.58E-13 | 2.93E-13 | 0.655854 | + | 1.49829 | 2.78593 | | 16.8422 | 16.7275 | 16.8161 | 16.2248 | 16.1947 | 16.7238 | 15.7495 | 15.8042 | 10 | 10 | 10 | 8 | 8 | 9 | 8 | 9 |
| SSPN | Q14714;Q1 | 2.30E-08 | 8.00E-09 | 0.657429 | | 1.24123 | 2.34588 | | 15.5831 | 15.4286 | 15.5444 | 14.3944 | 14.6982 | 14.9693 | 15.6112 | 14.6332 | 2 | 2 | 2 | 2 | 1 | 2 | 2 | 1 |
| RASSF2 | P50749 | 0.00365 | 0.000667 | 0.663578 | | 0.727722 | 1.48851 | | 14.8479 | 14.7301 | 14.7312 | 14.3829 | 14.0108 | 14.4081 | 12.8814 | 14.8476 | 4 | 5 | 5 | 4 | 4 | 4 | 3 | 4 |
| PC | P11498 | 4.62E-16 | 8.61E-16 | 0.664171 | + | 3.59454 | 7.68353 | | 16.5452 | 16.5225 | 16.516 | 15.9832 | 15.9394 | 15.9804 | 15.6812 | 15.7346 | 37 | 38 | 39 | 37 | 27 | 34 | 28 | 32 |
| MRPL11 | Q9Y3B7 | 9.64E-06 | 2.53E-06 | 0.664634 | | 0.921361 | 1.81255 | | 16.7387 | 16.7459 | 16.6428 | 16.5877 | 15.7673 | 15.12 | 16.1968 | 16.5507 | 12 | 12 | 12 | 11 | 11 | 12 | 12 | 12 |
| ATP5MG | O75964 | 3.57E-12 | 2.26E-12 | 0.669291 | | 0.811345 | 1.62916 | | 19.1341 | 19.1493 | 19.1367 | 19.2556 | 18.0035 | 17.7174 | 18.2403 | 19.1368 | 5 | 5 | 5 | 5 | 4 | 5 | 5 | 5 |
| SLIT2 | O94813;O9 | 1.95E-06 | 5.48E-07 | 0.669566 | + | 2.26007 | 4.23093 | | 15.8558 | 15.7719 | 15.7437 | 15.252 | 15.4904 | 15.0329 | 14.7955 | 15.0337 | 16 | 19 | 18 | 14 | 12 | 11 | 9 | 14 |
| MVK | Q03426 | 1.09E-07 | 3.50E-08 | 0.670402 | + | 1.77469 | 3.28108 | | 15.9057 | 15.8284 | 15.7968 | 15.3715 | 15.5172 | 14.9609 | 14.6872 | 15.3294 | 11 | 11 | 11 | 10 | 12 | 10 | 7 | 11 |
| PGM2 | Q96G03 | 7.55E-14 | 7.45E-14 | 0.670817 | | 1.16908 | 2.22477 | | 17.0742 | 17.1995 | 17.2126 | 16.7228 | 16.1015 | 17.2336 | 16.4045 | 15.9942 | 28 | 27 | 27 | 29 | 21 | 27 | 25 | 22 |
| IFI16 | Q16666;Q1 | 4.41E-13 | 3.51E-13 | 0.672401 | + | 1.88588 | 3.48861 | | 16.6573 | 16.7024 | 16.6737 | 16.2624 | 15.7823 | 16.4275 | 15.8747 | 15.68 | 29 | 30 | 31 | 29 | 24 | 30 | 31 | 26 |
| SIRPA | P78324 | 5.88E-14 | 5.98E-14 | 0.673197 | + | 1.46466 | 2.72741 | | 16.4233 | 16.3298 | 16.4535 | 16.3258 | 15.294 | 15.7626 | 15.8673 | 15.3953 | 13 | 13 | 13 | 13 | 13 | 12 | 13 | 7 |
| H1-5 | P16401 | 1.59E-11 | 8.88E-12 | 0.675104 | + | 2.37425 | 4.4729 | | 20.9047 | 20.8681 | 20.8317 | 20.144 | 20.4744 | 20.0471 | 19.8788 | 20.421 | 10 | 10 | 10 | 10 | 10 | 10 | 10 | 10 |
| HMGB2 | P26583 | 2.47E-17 | 6.98E-17 | 0.675218 | + | 2.75865 | 5.35084 | | 17.9823 | 18.0488 | 17.9914 | 17.5622 | 17.4035 | 17.2177 | 17.0292 | 17.4488 | 9 | 9 | 8 | 8 | 9 | 9 | 8 | 8 |
| RBL2 | Q08999 | 0.002632 | 0.0005 | 0.683532 | | 0.438458 | 1.0666 | | 14.0346 | 13.5411 | 13.6808 | NaN | NaN | 13.8899 | 12.2473 | NaN | 3 | 3 | 3 | NaN | NaN | 2 | 1 | NaN |
| TMEM97 | Q5BJF2 | 0.000211 | 4.79E-05 | 0.684042 | + | 2.19027 | 4.08679 | | 18.0223 | 17.9828 | 18.1675 | 17.3353 | 17.6742 | 16.9533 | 17.3647 | 17.5401 | 2 | 2 | 2 | 2 | 1 | 2 | 2 | 2 |
| LPCAT2 | Q7L5N7 | 1.30E-08 | 4.65E-09 | 0.684664 | + | 1.66794 | 3.08659 | | 16.9943 | 17.0004 | 16.9815 | 16.6277 | 16.7238 | 16.2197 | 16.1549 | 15.811 | 15 | 15 | 15 | 15 | 14 | 14 | 14 | 12 |
| ZFP36L1 | Q07352 | 2.50E-09 | 9.80E-10 | 0.684818 | | 1.10539 | 2.11844 | | 16.8065 | 16.8039 | 16.7405 | 15.8494 | 15.4993 | 16.9529 | 16.2005 | 15.992 | 5 | 6 | 5 | 5 | 5 | 6 | 5 | 5 |
| SF3B6 | Q9Y3B4 | 9.32E-06 | 2.46E-06 | 0.690674 | | 0.837941 | 1.67362 | | 17.7243 | 17.6738 | 17.6741 | 17.515 | 16.7981 | 15.8947 | 17.2058 | 17.5867 | 5 | 5 | 5 | 5 | 4 | 4 | 5 | 5 |
| AP1S1 | P61966 | 3.32E-08 | 1.13E-08 | 0.691673 | + | 1.44298 | 2.68985 | | 17.7565 | 17.8221 | 17.6014 | 17.402 | 16.6381 | 16.5417 | 17.1452 | 17.4479 | 4 | 4 | 4 | 4 | 4 | 4 | 4 | 4 |
| ADGRE5 | P48960;P4 | 1.49E-09 | 6.01E-10 | 0.692418 | + | 2.7711 | 5.38106 | | 17.0295 | 17.0392 | 17.0816 | 16.2418 | 16.6059 | 16.3094 | 16.5435 | 16.0878 | 14 | 13 | 14 | 14 | 10 | 13 | 11 | 11 |
| DNM1 | Q05193 | 6.50E-09 | 2.42E-09 | 0.693888 | + | 1.76519 | 3.26358 | | 16.8298 | 16.8344 | 16.7592 | 15.851 | 15.6284 | 16.403 | 16.4181 | 16.2691 | 27 | 25 | 28 | 22 | 17 | 25 | 25 | 24 |
| COX11 | Q9Y6N1 | 2.59E-06 | 7.21E-07 | 0.697041 | + | 2.2108 | 4.1289 | | 15.312 | 15.1729 | 15.0588 | 14.0933 | 14.4441 | 14.4624 | 14.8353 | 14.5857 | 1 | 1 | 2 | 2 | 1 | 1 | 1 | 1 |
| TSR2 | Q969E8 | 5.85E-11 | 2.94E-11 | 0.69803 | | 0.339308 | 0.793301 | | 16.3019 | 16.278 | 16.3281 | 16.5598 | 13.0043 | 16.2343 | 15.8649 | 16.3598 | 2 | 2 | 2 | 2 | 2 | 2 | 2 | 2 |
| PSAT1 | Q9Y617 | 7.37E-13 | 5.50E-13 | 0.698452 | | 0.456444 | 1.01429 | | 19.7865 | 19.8704 | 19.8442 | 17.8812 | 18.6396 | 20.5101 | 20.2036 | 18.4417 | 21 | 21 | 22 | 18 | 20 | 20 | 21 | 20 |
| NES | P48681 | 8.03E-14 | 7.85E-14 | 0.698811 | | 0.683238 | 1.41298 | | 17.4388 | 17.4216 | 17.4568 | 16.1767 | 17.1557 | 15.7171 | 17.8398 | 16.8121 | 51 | 50 | 51 | 45 | 48 | 37 | 52 | 43 |

| Gene names | UniProt IDs | Protein Group P-value | Protein Group Q-value | Log2 Difference NUP214 vs Controls (n=5) | p<0.05 | -log10 p-value | T-test Test statistic | NPC Localization | Log2 MS2_quantity_NUP214_1 | Log2 MS2_quantity_NUP214_2 | Log2 MS2_quantity_NUP214_3 | Log2 MS2_quantity_Control_1 | Log2 MS2_quantity_Control_2 | Log2 MS2_quantity_Control_3 | Log2 MS2_quantity_Control_4 | Log2 MS2_quantity_Control_5 | Number Peptides for Quantitation NUP214_1 | Number Peptides for Quantitation NUP214_2 | Number Peptides for Quantitation NUP214_3 | Number Peptides for Quantitation Control_1 | Number Peptides for Quantitation Control_2 | Number Peptides for Quantitation Control_3 | Number Peptides for Quantitation Control_4 | Number Peptides for Quantitation Control_5 |
|---|---|---|---|---|---|---|---|---|---|---|---|---|---|---|---|---|---|---|---|---|---|---|---|---|
| NQO1 | P15559;P1 | 0.000156 | 3.62E-05 | 0.699669 | | 0.708794 | 1.45644 | | 20.3916 | 20.465 | 20.3798 | 18.9711 | 20.4175 | 20.0071 | 20.4275 | 18.7392 | 12 | 12 | 12 | 11 | 12 | 12 | 12 | 11 |
| SERPINF1 | P36955 | 3.81E-07 | 1.16E-07 | 0.699718 | + | 2.05429 | 6.10635 | | 15.3309 | 15.518 | 15.6357 | 14.7989 | NaN | NaN | 14.7914 | NaN | 9 | 7 | 5 | 3 | NaN | NaN | 5 | NaN |
| SELENOH | Q8IZQ5 | 0.001764 | 0.00035 | 0.700048 | | 1.2466 | 2.35492 | | 15.4627 | 15.4342 | 15.3832 | 14.3547 | 14.6115 | 14.8688 | 15.5154 | 14.2829 | 4 | 6 | 4 | 5 | 3 | 3 | 4 | 3 |
| NDUFA2 | O43678 | 4.64E-05 | 1.14E-05 | 0.701171 | | 0.580407 | 1.23562 | | 16.9188 | 16.7697 | 16.989 | 16.9276 | 15.6592 | 14.9414 | 16.1384 | 17.29 | 5 | 5 | 5 | 4 | 3 | 3 | 4 | 4 |
| KYAT1 | Q16773;Q1 | 0.000255 | 5.73E-05 | 0.704074 | | 0.895511 | 1.76957 | | 17.3552 | 17.3081 | 17.5321 | 17.8035 | 16.1567 | 16.5294 | 16.2421 | 16.7402 | 1 | 2 | 2 | 2 | 2 | 3 | 2 | 2 |
| CYCS | P99999 | 3.60E-12 | 2.28E-12 | 0.706079 | + | 2.94598 | 5.81855 | | 16.818 | 16.7713 | 16.8628 | 16.0116 | 16.0331 | 15.8961 | 16.2034 | 16.4123 | 8 | 8 | 8 | 7 | 7 | 7 | 5 | 8 |
| TBL1XR1 | Q9BZK7 | 1.70E-13 | 1.51E-13 | 0.708216 | + | 3.69958 | 8.02683 | | 16.0589 | 16.1696 | 16.1646 | 15.4272 | 15.4215 | 15.6499 | 15.3363 | 15.279 | 11 | 11 | 11 | 10 | 10 | 10 | 10 | 8 |
| FBXW9 | Q5XUX1;Q5 | 0.000845 | 0.000176 | 0.710686 | | 0.688254 | 1.45664 | | 13.7449 | 14.148 | 13.5969 | 12.4119 | 12.471 | 13.9353 | 13.6588 | NaN | 2 | 2 | 2 | 1 | 1 | 1 | 3 | NaN |
| ANP32A | P39687 | 1.17E-08 | 4.22E-09 | 0.717778 | + | 4.10095 | 9.46333 | | 19.2216 | 19.2624 | 19.2484 | 18.7322 | 18.3863 | 18.5046 | 18.5189 | 18.4898 | 8 | 8 | 9 | 8 | 8 | 8 | 8 | 8 |
| IL6ST | P40189 | 1.38E-07 | 4.41E-08 | 0.719378 | | 1.11003 | 2.12617 | | 16.4245 | 16.3417 | 16.3447 | 16.4627 | 15.7679 | 15.3264 | 14.947 | 15.7506 | 17 | 17 | 17 | 18 | 10 | 14 | 11 | 16 |
| BBS9 | Q3SYG4;Q3 | 0.00022 | 4.97E-05 | 0.72481 | | 0.258249 | 0.648927 | | 14.164 | 13.2436 | 14.3664 | 11.1537 | NaN | 14.7174 | 13.7284 | NaN | 1 | 2 | 2 | 1 | NaN | 1 | 1 | NaN |
| MRTFB | Q9ULH7;Q9 | 0.047499 | 0.007722 | 0.72548 | | 0.707224 | 1.54931 | | 16.1659 | 15.6951 | 16.2403 | 15.0257 | 16.1641 | 14.7351 | NaN | NaN | 2 | 3 | 3 | 2 | 3 | 3 | NaN | NaN |
| H2AZ1;H2AZ2 | P0C0S5;Q7 | 0.000807 | 0.000169 | 0.725719 | + | 4.28137 | 10.1799 | | 20.7423 | 20.8171 | 20.7098 | 20.1442 | 20.093 | 19.9655 | 19.8659 | 20.0849 | 3 | 3 | 3 | 3 | 3 | 3 | 3 | 3 |
| VARS2 | Q5ST30;Q5 | 0.000648 | 0.000137 | 0.726182 | + | 1.60513 | 3.16969 | | 13.8771 | 13.7238 | 13.5924 | 12.6026 | 13.1158 | 13.461 | NaN | 12.8404 | 3 | 3 | 5 | 1 | 1 | 3 | NaN | 1 |
| PTPRG | P23470;P2 | 1.10E-12 | 7.84E-13 | 0.727708 | + | 2.15056 | 4.00602 | | 14.8326 | 14.8937 | 14.8711 | 13.9975 | 14.0864 | 14.4445 | 14.4321 | 13.73 | 11 | 11 | 10 | 11 | 6 | 10 | 10 | 9 |
| NFKBIE | O00221 | 0.000813 | 0.00017 | 0.728261 | | 0.099064 | 0.276249 | | NaN | 12.8084 | 14.1997 | 13.8275 | 7.62202 | 14.9193 | NaN | 14.7344 | NaN | 1 | 1 | 1 | 2 | 1 | NaN | 1 |
| TBCA | O75347 | 2.17E-19 | 1.21E-18 | 0.728827 | | 0.507468 | 1.10663 | | 17.944 | 18.0421 | 17.9705 | 18.247 | 16.5289 | 15.7302 | 17.5459 | 18.2315 | 14 | 14 | 15 | 15 | 13 | 13 | 15 | 15 |
| ADGRA2 | Q96PE1;Q9 | 4.69E-14 | 4.90E-14 | 0.729079 | + | 1.73903 | 3.21562 | | 15.9111 | 15.7519 | 15.6607 | 14.8848 | 15.1283 | 15.6476 | 14.8658 | 14.7009 | 5 | 5 | 5 | 4 | 3 | 5 | 3 | 3 |
| RCAN2 | Q14206;Q1 | 0.000218 | 4.93E-05 | 0.729082 | | 0.372372 | 0.857176 | | 17.6613 | 17.8518 | 17.7669 | 17.2218 | 14.7719 | 18.7329 | 17.1213 | 17.3067 | 2 | 2 | 2 | 1 | 2 | 1 | 2 | 2 |
| PSMB10 | P40306 | 4.73E-12 | 2.91E-12 | 0.729112 | | 1.20264 | 2.281 | | 15.9765 | 15.9511 | 15.9662 | 14.6791 | 15.7679 | 15.8449 | 14.8611 | 15.0245 | 6 | 6 | 6 | 5 | 5 | 6 | 4 | 5 |
| PSMB9 | P28065 | 0.000171 | 3.93E-05 | 0.729791 | + | 1.33462 | 2.50397 | | 17.1013 | 17.0577 | 17.0486 | 15.8692 | 16.5175 | 17.062 | 15.9162 | 16.3323 | 7 | 6 | 6 | 6 | 6 | 6 | 5 | 6 |
| UQCR10 | Q9UDW1 | 1.70E-06 | 4.82E-07 | 0.731934 | | 0.459793 | 1.02041 | | 17.7089 | 17.5995 | 17.9966 | 18.113 | 15.6966 | 15.8802 | 17.2955 | 18.1966 | 2 | 2 | 2 | 2 | 2 | 2 | 2 | 2 |
| TGFBI | Q15582 | 3.12E-15 | 4.61E-15 | 0.73885 | + | 1.34149 | 2.51568 | | 17.4583 | 17.4118 | 17.4638 | 16.4888 | 16.2138 | 16.6668 | 17.5261 | 16.6336 | 24 | 24 | 24 | 23 | 20 | 24 | 25 | 24 |
| EPB41L2 | O43491 | 7.36E-22 | 9.18E-21 | 0.740141 | + | 1.47647 | 2.74793 | | 17.5661 | 17.5784 | 17.5641 | 16.857 | 16.688 | 17.5902 | 16.4459 | 16.566 | 35 | 37 | 38 | 34 | 31 | 36 | 28 | 34 |
| MMP2 | P08253 | 2.00E-15 | 3.14E-15 | 0.741728 | + | 1.35972 | 2.54678 | | 16.1851 | 16.2702 | 16.212 | 15.4289 | 14.9118 | 16.1383 | 15.7712 | 15.1531 | 18 | 17 | 17 | 15 | 11 | 15 | 15 | 14 |
| NSMCE3 | Q96MG7 | 0.001465 | 0.000295 | 0.741949 | | 0.983516 | 1.98533 | | 14.6735 | 14.5526 | 15.0066 | 14.6293 | NaN | 13.2784 | 14.3378 | 13.7639 | 3 | 5 | 3 | 3 | NaN | 5 | 4 | 1 |
| LRRN4CL | Q8ND94 | 0.001859 | 0.000367 | 0.74244 | | 0.85834 | 1.70766 | | 16.7963 | 16.509 | 16.6185 | 14.8934 | 16.6752 | 15.5315 | 15.9158 | 16.4782 | 4 | 4 | 3 | 1 | 4 | 3 | 3 | 4 |
| CTSL | P07711 | 1.29E-11 | 7.33E-12 | 0.743133 | + | 3.11004 | 6.25237 | | 18.7825 | 18.7645 | 18.7422 | 18.0231 | 18.1488 | 17.8091 | 18.276 | 17.8426 | 8 | 9 | 8 | 8 | 6 | 8 | 8 | 8 |
| TPM4 | P67936-2 | 8.97E-11 | 4.34E-11 | 0.743349 | | 0.66143 | 1.37572 | | 16.2169 | 16.2508 | 16.2557 | 14.458 | 15.1576 | 15.6048 | 15.3389 | 16.9296 | 6 | 6 | 7 | 6 | 6 | 6 | 7 | 8 |
| TMEM168 | Q9H0V1;Q9 | 0.001391 | 0.000281 | 0.744499 | | 0.827884 | 1.65682 | | 15.3401 | 15.3198 | 15.2804 | 14.9349 | 14.2674 | 15.4128 | 14.7933 | 13.4365 | 1 | 1 | 1 | 1 | 1 | 1 | 1 | 1 |
| BCKDHB | P21953 | 0.001344 | 0.000272 | 0.752434 | + | 2.38649 | 4.49932 | | 16.4172 | 16.3533 | 16.4466 | 15.1923 | 15.9249 | 15.7964 | 15.71 | 15.6427 | 5 | 5 | 5 | 3 | 3 | 4 | 3 | 3 |
| PIGQ | Q9BRB3-2 | 0.000978 | 0.000202 | 0.75279 | | 0.966767 | 1.95517 | | 13.5437 | 13.718 | 14.1172 | 13.6485 | 13.2343 | NaN | 13.0679 | 12.21 | 1 | 1 | 2 | 1 | 1 | NaN | 1 | 1 |
| RCC2 | Q9P258 | 8.32E-15 | 1.08E-14 | 0.756774 | + | 2.47932 | 4.70281 | | 16.7497 | 16.8016 | 16.8027 | 15.7572 | 15.8112 | 16.3123 | 16.3134 | 15.9453 | 19 | 19 | 18 | 18 | 16 | 17 | 18 | 16 |
| H1-4 | P10412 | 0.000781 | 0.000163 | 0.757717 | | 0.436116 | 0.976915 | | 18.9573 | 17.974 | 18.3253 | 16.4511 | 19.493 | 17.6305 | 16.5668 | 18.1641 | 2 | 2 | 2 | 2 | 2 | 2 | 2 | 2 |
| PLOD2 | O00469-2 | 1.02E-09 | 4.24E-10 | 0.760041 | | 0.753485 | 1.532 | | 18.519 | 18.5576 | 18.4815 | 16.3696 | 18.5974 | 17.9786 | 18.0456 | 17.8052 | 25 | 26 | 26 | 22 | 25 | 24 | 28 | 25 |
| RAC2 | P15153 | 5.92E-07 | 1.76E-07 | 0.761936 | + | 2.13906 | 3.9828 | | 17.4848 | 17.4187 | 17.3485 | 16.455 | 16.8657 | 16.1917 | 16.8731 | 16.8914 | 4 | 4 | 4 | 4 | 4 | 4 | 4 | 4 |
| MYLK | Q15746 | 2.56E-24 | 7.58E-23 | 0.763674 | | 0.780268 | 1.57706 | | 17.7156 | 17.7382 | 17.7607 | 15.7396 | 16.8837 | 16.9539 | 17.9616 | 17.3336 | 61 | 65 | 62 | 57 | 60 | 63 | 67 | 62 |
| NSUN6 | Q8TEA1 | 0.001554 | 0.000312 | 0.764076 | + | 1.43685 | 2.83239 | | 14.7335 | 14.6828 | 14.5277 | 14.1554 | 13.2451 | 13.9073 | NaN | 14.228 | 3 | 3 | 3 | 1 | 1 | 2 | NaN | 2 |
| COL16A1 | Q07092;Q0 | 1.41E-11 | 7.99E-12 | 0.765358 | | 1.06009 | 2.04304 | | 16.2045 | 16.1445 | 16.1034 | 14.7768 | 15.2807 | 16.4443 | 15.2884 | 15.1371 | 11 | 11 | 11 | 9 | 8 | 12 | 9 | 8 |
| TMEM62 | Q0P6H9 | 0.006363 | 0.00112 | 0.768154 | | 0.964792 | 2.06006 | | 13.8743 | 13.7229 | 13.582 | 13.0268 | NaN | NaN | 12.2977 | 13.5503 | 1 | 2 | 2 | 1 | NaN | NaN | 2 | 2 |
| PPL | O60437 | 0.000514 | 0.00011 | 0.768631 | + | 1.37908 | 2.71998 | | 14.4616 | 14.4947 | 14.8484 | NaN | 13.9268 | 14.3031 | 13.2318 | 13.87 | 14 | 13 | 11 | NaN | 3 | 10 | 3 | 4 |
| NEK6 | Q9HC98;Q9 | 9.61E-09 | 3.50E-09 | 0.76961 | + | 2.84134 | 5.55383 | | 15.6538 | 15.4267 | 15.5647 | 14.9579 | 14.6665 | 14.5892 | 14.6147 | 15.0656 | 4 | 4 | 3 | 4 | 2 | 3 | 2 | 3 |
| STING1 | Q86WV6 | 3.40E-16 | 6.60E-16 | 0.770596 | + | 3.31343 | 6.82435 | | 18.9064 | 18.884 | 18.8309 | 18.3713 | 17.8838 | 18.1206 | 18.1646 | 17.9755 | 7 | 7 | 7 | 7 | 7 | 7 | 7 | 7 |
| AK4 | P27144 | 1.17E-14 | 1.45E-14 | 0.771568 | | 1.01446 | 1.96719 | | 16.2855 | 16.2502 | 16.3914 | 16.0214 | 15.572 | 15.6557 | 14.4229 | 16.0152 | 8 | 8 | 8 | 7 | 8 | 8 | 7 | 8 |
| ZNF22 | P17026 | 6.46E-11 | 3.21E-11 | 0.77351 | + | 1.66644 | 3.0839 | | 12.3809 | 12.2967 | 12.7695 | 12.1755 | 11.424 | 11.9803 | 11.2529 | 11.7116 | 3 | 4 | 4 | 3 | 3 | 3 | 2 | 2 |
| ALDH1B1 | P30837 | 3.59E-16 | 6.92E-16 | 0.779363 | | 0.67419 | 1.39754 | | 17.1995 | 17.2001 | 17.1935 | 16.2981 | 16.7575 | 17.3748 | 14.8916 | 16.7696 | 18 | 19 | 19 | 17 | 18 | 18 | 13 | 18 |
| OLFML3 | Q9NRN5 | 7.93E-05 | 1.90E-05 | 0.782068 | + | 2.75411 | 5.33986 | | 15.9002 | 15.8107 | 15.8886 | 14.8862 | 15.3898 | 15.3014 | 14.8753 | 14.9694 | 5 | 4 | 5 | 5 | 4 | 6 | 4 | 4 |
| BSCL2 | Q96G97;Q9 | 0.008301 | 0.001438 | 0.783183 | | 0.441383 | 0.986632 | | 15.8322 | 15.8044 | 15.7802 | 12.7421 | 15.0253 | 16.0711 | 15.7651 | 15.5086 | 2 | 2 | 2 | 1 | 2 | 2 | 2 | 1 |
| CBSL;CBS;CBS | P0DN79;P3 | 1.24E-07 | 3.95E-08 | 0.788088 | | 0.835182 | 1.66901 | | 17.3118 | 17.3014 | 17.3433 | 15.4023 | 16.2486 | 17.4145 | 17.1366 | 16.4519 | 17 | 17 | 19 | 10 | 11 | 17 | 16 | 14 |
| CCDC97 | Q96F63 | 0.002867 | 0.000538 | 0.798136 | | 1.15773 | 2.30261 | | 13.9926 | 13.7785 | 13.7763 | 13.8107 | NaN | 13.1596 | 12.4882 | 12.7455 | 2 | 1 | 1 | 1 | NaN | 1 | 1 | 1 |
| THBS2 | P35442 | 1.80E-05 | 4.61E-06 | 0.798607 | + | 1.39679 | 2.61026 | | 16.8796 | 16.8452 | 16.8852 | 15.7358 | 15.7786 | 16.418 | 16.8013 | 15.6232 | 17 | 18 | 18 | 17 | 13 | 18 | 17 | 15 |
| HNRNPA3 | P51991-2 | 4.07E-07 | 1.24E-07 | 0.804805 | + | 1.72574 | 3.19134 | | 16.7328 | 16.6889 | 16.028 | 15.9203 | 15.5399 | 15.8917 | 15.1808 | 15.8595 | 2 | 2 | 2 | 2 | 2 | 2 | 2 | 2 |
| PLEKHF2 | Q9H8W4 | 0.000787 | 0.000165 | 0.808167 | | 0.476386 | 1.05061 | | 15.3056 | 15.0912 | 15.1042 | 15.3913 | 12.1444 | 15.111 | 14.5825 | 14.565 | 1 | 2 | 2 | 2 | 1 | 2 | 2 | 2 |
| MTSS2 | Q765P7 | 3.24E-10 | 1.43E-10 | 0.814681 | + | 2.1192 | 3.94284 | | 15.6869 | 15.6843 | 15.7313 | 14.4029 | 15.305 | 14.7344 | 14.886 | 15.1027 | 12 | 12 | 12 | 7 | 10 | 8 | 11 | 9 |
| D2HGDH | Q8N465 | 8.94E-08 | 2.90E-08 | 0.81538 | + | 1.624 | 3.00778 | | 15.6008 | 15.4508 | 15.5788 | 15.0231 | 14.0814 | 15.1172 | 14.4298 | 14.9889 | 4 | 4 | 4 | 3 | 3 | 4 | 3 | 2 |
| TRAF5 | O00463;O0 | 5.87E-05 | 1.43E-05 | 0.819701 | | 0.661838 | 1.40912 | | 13.792 | 13.2818 | 13.5158 | 12.2954 | NaN | 14.134 | 12.0301 | 12.3812 | 1 | 2 | 2 | 1 | NaN | 2 | 1 | 2 |
| ILRUN | Q9H6K1;Q9 | 0.002242 | 0.000434 | 0.821146 | + | 1.32354 | 2.48514 | | 15.0268 | 14.8885 | 14.9373 | 14.5663 | 13.9615 | 14.6227 | 14.232 | 13.266 | 2 | 2 | 1 | 1 | 1 | 1 | 1 | 1 |
| PLPP3 | O14495 | 4.03E-08 | 1.36E-08 | 0.824036 | | 0.866243 | 1.72083 | | 18.582 | 18.5872 | 18.6551 | 18.5901 | 16.8675 | 18.4775 | 17.0258 | 17.9593 | 7 | 7 | 7 | 7 | 6 | 7 | 7 | 7 |
| TBL1X | O60907;O6 | 1.85E-13 | 1.63E-13 | 0.825056 | + | 3.55246 | 7.5495 | | 15.805 | 15.8301 | 15.8198 | 15.0587 | 14.721 | 15.2062 | 15.0618 | 14.9185 | 9 | 10 | 8 | 7 | 5 | 7 | 7 | 6 |
| HDAC3 | O15379;O1 | 0.002987 | 0.000557 | 0.825621 | + | 1.56391 | 3.39928 | | 14.1377 | 13.6139 | 13.8794 | 12.8328 | NaN | 12.8912 | 13.43 | NaN | 2 | 2 | 2 | 1 | NaN | 2 | 1 | NaN |
| LSM4 | Q9Y4Z0 | 1.15E-06 | 3.31E-07 | 0.826209 | + | 2.63163 | 5.04922 | | 17.4377 | 17.3518 | 17.3268 | 16.9081 | 16.2029 | 16.5751 | 16.374 | 16.6693 | 6 | 6 | 6 | 6 | 5 | 6 | 6 | 6 |
| EFNB1 | P98172 | 1.95E-09 | 7.73E-10 | 0.828859 | | 0.666284 | 1.41712 | | 15.0503 | 15.0003 | 15.2724 | 14.4907 | NaN | 13.5987 | 13.4469 | 15.5791 | 1 | 1 | 1 | 1 | NaN | 1 | 1 | 1 |
| MCAM | P43121 | 2.07E-09 | 8.20E-10 | 0.829622 | | 0.901462 | 1.77947 | | 15.6512 | 15.6255 | 15.6701 | 14.9088 | 15.0655 | 13.6428 | 14.6751 | 15.8045 | 7 | 7 | 8 | 5 | 2 | 1 | 2 | 6 |
| LOXL3 | P58215 | 0.000575 | 0.000123 | 0.833584 | + | 1.72129 | 4.62801 | | 17.3144 | 17.2671 | 17.409 | NaN | NaN | NaN | 16.7271 | 16.266 | 5 | 5 | 5 | NaN | NaN | NaN | 3 | 3 |
| MACROD1 | Q9BQ69 | 7.97E-06 | 2.11E-06 | 0.836176 | + | 1.81625 | 3.35803 | | 16.2364 | 16.3163 | 16.213 | 14.9979 | 15.6898 | 16.0024 | 15.2617 | 15.1434 | 6 | 6 | 5 | 3 | 4 | 6 | 3 | 3 |
| OVCA2 | Q8WZ82 | 9.98E-06 | 2.62E-06 | 0.836731 | + | 4.24704 | 10.0399 | | 17.4721 | 17.5243 | 17.4752 | 16.7036 | 16.6414 | 16.4256 | 16.7072 | 16.7912 | 5 | 6 | 6 | 5 | 5 | 5 | 6 | 6 |
| H4C1 | P62805 | 5.29E-13 | 4.10E-13 | 0.837524 | + | 1.57299 | 2.91714 | | 24.6943 | 24.7568 | 24.7283 | 24.2364 | 24.0108 | 23.0902 | 23.8329 | 24.2744 | 11 | 11 | 11 | 11 | 11 | 10 | 10 | 10 |
| H1-0 | P07305 | 0.000134 | 3.12E-05 | 0.850328 | + | 1.79514 | 3.31884 | | 18.1771 | 18.1115 | 18.2569 | 17.7716 | 17.0568 | 17.8153 | 17.0906 | 16.9232 | 5 | 5 | 5 | 5 | 5 | 5 | 5 | 5 |
| AEBP1 | Q8IUX7 | 1.43E-11 | 8.09E-12 | 0.850636 | + | 1.47656 | 2.74808 | | 15.6007 | 15.6485 | 15.7027 | 13.9219 | 14.9544 | 15.2325 | 15.101 | 14.7902 | 13 | 14 | 13 | 2 | 3 | 11 | 9 | 3 |
| CRIP2 | P52943;P5 | 2.69E-07 | 8.36E-08 | 0.852199 | + | 1.41743 | 2.64576 | | 19.3573 | 19.3288 | 19.4473 | 18.8355 | 18.5158 | 19.2507 | 18.0908 | 17.9353 | 7 | 8 | 10 | 7 | 6 | 6 | 4 | 5 |
| XPC | Q01831 | 2.57E-22 | 3.50E-21 | 0.856676 | + | 4.25553 | 10.0744 | | 15.9761 | 16.063 | 16.0126 | 15.0369 | 15.0935 | 15.3952 | 15.1682 | 15.109 | 18 | 17 | 16 | 15 | 10 | 14 | 12 | 12 |
| GTDC1 | Q4AE62;Q4 | 0.004412 | 0.000796 | 0.857247 | | 1.18013 | 2.83257 | | 13.8246 | 13.973 | 13.7081 | NaN | NaN | 13.3617 | 12.5943 | NaN | 1 | 1 | 1 | NaN | NaN | 1 | 2 | NaN |
| ERAP1 | Q9NZ08;Q9 | 1.10E-14 | 1.38E-14 | 0.859233 | + | 1.67805 | 3.10483 | | 17.1781 | 17.1426 | 17.1373 | 15.7178 | 15.9235 | 16.8502 | 16.5459 | 16.4299 | 32 | 33 | 33 | 29 | 24 | 33 | 32 | 30 |
| NAPRT | Q6XQN6;Q6 | 1.80E-06 | 5.09E-07 | 0.869101 | + | 1.80647 | 3.33985 | | 16.5228 | 16.5314 | 16.4839 | 15.8455 | 16.2655 | 15.4481 | 15.5504 | 15.1087 | 10 | 10 | 11 | 8 | 10 | 7 | 8 | 6 |
| TREX1 | Q9NSU2;Q9 | 0.00046 | 9.95E-05 | 0.869717 | + | 3.76898 | 8.26071 | | 14.422 | 14.4879 | 14.2777 | 13.6447 | 13.595 | 13.466 | 13.274 | 13.6509 | 3 | 2 | 2 | 3 | 2 | 3 | 2 | 2 |
| MEST | Q5EB52;Q5 | 0.000995 | 0.000205 | 0.873082 | | 0.402787 | 0.92893 | | 15.9362 | 15.8481 | 15.8362 | 13.1459 | NaN | 14.4257 | 15.5648 | 16.8653 | 2 | 2 | 2 | 1 | NaN | 1 | 2 | 2 |
| EPHB3 | P54753 | 2.56E-24 | 7.58E-23 | 0.874709 | + | 2.34683 | 4.41408 | | 16.5677 | 16.5937 | 16.5383 | 15.3941 | 15.5004 | 16.2372 | 15.5745 | 15.7531 | 17 | 17 | 16 | 10 | 10 | 16 | 11 | 12 |
| IGFBP7 | Q16270;Q1 | 9.33E-11 | 4.49E-11 | 0.879185 | | 0.333027 | 0.781012 | | 15.8956 | 15.9338 | 15.9659 | 12.3284 | 15.0144 | 17.3585 | 16.1203 | 14.4413 | 9 | 11 | 10 | 1 | 8 | 11 | 9 | 7 |

| Gene names | UniProt IDs | Protein Group P-value | Protein Group Q-value | Log2 Difference NUP214 vs Controls (n=5) | p<0.05 | -log10 p-value | T-test Test statistic | NPC Localization | Log2 MS2_quantity_NUP214_1 | Log2 MS2_quantity_NUP214_2 | Log2 MS2_quantity_NUP214_3 | Log2 MS2_quantity_Control_1 | Log2 MS2_quantity_Control_2 | Log2 MS2_quantity_Control_3 | Log2 MS2_quantity_Control_4 | Log2 MS2_quantity_Control_5 | Number Peptides for Quantitation NUP214_1 | Number Peptides for Quantitation NUP214_2 | Number Peptides for Quantitation NUP214_3 | Number Peptides for Quantitation Control_1 | Number Peptides for Quantitation Control_2 | Number Peptides for Quantitation Control_3 | Number Peptides for Quantitation Control_4 | Number Peptides for Quantitation Control_5 |
|---|---|---|---|---|---|---|---|---|---|---|---|---|---|---|---|---|---|---|---|---|---|---|---|---|
| MCCC1 | Q96RQ3 | 3.76E-10 | 1.64E-10 | 0.880792 | | 1.24601 | 2.35393 | | 16.0463 | 16.0646 | 15.9644 | 15.4886 | 14.1152 | 15.6691 | 14.9966 | 15.452 | 14 | 12 | 14 | 10 | 5 | 11 | 6 | 10 |
| SMDT1 | Q9H4I9 | 4.17E-05 | 1.03E-05 | 0.884218 | | 0.553618 | 1.18861 | | 16.3335 | 16.183 | 16.5728 | 16.8812 | 14.3321 | 14.2795 | 15.2498 | 16.6519 | 2 | 2 | 2 | 2 | 2 | 2 | 2 | 2 |
| PCK2 | Q16822 | 2.59E-11 | 1.39E-11 | 0.888278 | | 0.767495 | 1.55559 | | 18.7731 | 18.7474 | 18.7253 | 17.0406 | 17.3756 | 19.1299 | 18.6353 | 17.1201 | 27 | 27 | 27 | 26 | 25 | 26 | 26 | 26 |
| ATP5MPL | P56378 | 9.48E-13 | 6.91E-13 | 0.892833 | | 0.977713 | 1.90617 | | 16.9799 | 16.8018 | 17.1706 | 17.1717 | 15.3125 | 15.8806 | 15.5083 | 16.5832 | 2 | 3 | 2 | 2 | 2 | 2 | 2 | 2 |
| DPYD | Q12882 | 4.60E-08 | 1.54E-08 | 0.897843 | | 1.189 | 2.25813 | | 16.6898 | 16.7487 | 16.7045 | 16.3938 | 15.9687 | 16.1926 | 14.6859 | 15.8414 | 10 | 12 | 9 | 8 | 7 | 8 | 3 | 6 |
| TMEM119 | Q4V9L6 | 5.55E-05 | 1.36E-05 | 0.910461 | + | 1.76375 | 3.26093 | | 19.873 | 19.9278 | 20.0793 | 18.7119 | 19.0929 | 19.8344 | 18.835 | 18.7735 | 5 | 5 | 5 | 5 | 5 | 5 | 5 | 5 |
| NHSL2 | Q5HYW2 | 5.43E-07 | 1.63E-07 | 0.91075 | | 0.619952 | 1.37926 | | 15.3351 | 15.3499 | 15.4739 | 14.2669 | 13.4531 | 15.7066 | NaN | NaN | 5 | 5 | 5 | 4 | 2 | 1 | NaN | NaN |
| ETV6 | P41212 | 0.01341 | 0.002267 | 0.911934 | | 0.812016 | 1.75472 | | 13.7783 | 13.6953 | 13.7504 | NaN | 13.8666 | 12.2694 | 12.3522 | NaN | 3 | 2 | 2 | NaN | 1 | 2 | 2 | NaN |
| NFIX | Q14938-4 | 2.49E-16 | 5.12E-16 | 0.919287 | + | 2.33593 | 4.39081 | | 16.8165 | 16.8513 | 16.8901 | 16.0945 | 15.8895 | 16.3794 | 15.4226 | 15.8808 | 9 | 8 | 9 | 8 | 8 | 8 | 8 | 8 |
| HLA-B | P01889 | 0.002093 | 0.000409 | 0.927127 | | 0.690223 | 1.42488 | | 18.2472 | 18.2106 | 18.2923 | 15.7589 | 18.5735 | 17.5252 | 17.9711 | 16.7859 | 4 | 4 | 4 | 2 | 2 | 3 | 3 | 3 |
| SAMHD1 | Q9Y3Z3 | 3.16E-13 | 2.63E-13 | 0.928929 | + | 3.20796 | 6.52283 | | 16.1381 | 16.1669 | 16.1353 | 14.9505 | 15.2339 | 15.4973 | 15.0071 | 15.4004 | 25 | 25 | 23 | 17 | 14 | 22 | 15 | 18 |
| BTN3A3 | O00478;O( | 1.27E-08 | 4.56E-09 | 0.929362 | + | 1.60296 | 2.97029 | | 15.9092 | 15.8397 | 15.9243 | 14.4543 | 15.5883 | 15.4501 | 14.5483 | 14.7675 | 5 | 5 | 4 | 1 | 1 | 4 | 1 | 2 |
| TRIM44 | Q96DX7 | 0.004026 | 0.00073 | 0.93595 | | 0.639917 | 1.41815 | | 14.063 | 14.0759 | 13.8587 | 14.0518 | NaN | 11.8214 | NaN | 13.3166 | 1 | 1 | 1 | 1 | NaN | 2 | NaN | 2 |
| DAAM2 | Q86T65;Q8 | 0.000143 | 3.32E-05 | 0.9377 | + | 2.9605 | 6.73379 | | 15.8489 | 15.9989 | 15.9083 | NaN | 15.085 | 15.0998 | 14.6404 | 15.0988 | 7 | 5 | 8 | NaN | 3 | 6 | 4 | 5 |
| ALKBH8 | Q96BT7;Q9 | 0.024888 | 0.004114 | 0.94137 | | 1.07113 | 2.27886 | | 13.1803 | 13.4995 | 13.4749 | 13.2224 | 12.2137 | NaN | 11.8946 | NaN | 1 | 2 | 2 | 1 | 2 | NaN | 1 | NaN |
| WDR81 | Q562E7 | 2.55E-10 | 1.15E-10 | 0.947594 | + | 2.69819 | 5.20577 | | 14.9808 | 15.0076 | 15.0456 | 13.9961 | 14.1543 | 14.2955 | 14.305 | 13.5678 | 10 | 11 | 11 | 8 | 5 | 6 | 8 | 7 |
| PHGDH | O43175 | 2.00E-10 | 9.19E-11 | 0.957293 | | 1.18928 | 2.2586 | | 20.5562 | 20.5688 | 20.5479 | 18.7777 | 19.444 | 20.4753 | 20.1761 | 19.1286 | 27 | 27 | 26 | 25 | 23 | 26 | 25 | 25 |
| DHCR24 | Q15392 | 1.44E-14 | 1.75E-14 | 0.960433 | | 1.24352 | 2.34973 | | 18.4269 | 18.4204 | 18.43 | 18.2515 | 17.8576 | 16.6107 | 16.9005 | 17.7065 | 16 | 16 | 15 | 16 | 15 | 14 | 15 | 14 |
| CNEP1R1 | Q8N9A8;Q8 | 2.77E-06 | 7.70E-07 | 0.972411 | + | 2.27721 | 4.26674 | | 14.9725 | 14.8877 | 14.914 | 14.2426 | 14.0424 | 13.2895 | 14.0266 | 14.1603 | 2 | 3 | 2 | 2 | 1 | 2 | 1 | 2 |
| PHYHD1 | Q5SRE7;Q5 | 0.000362 | 7.94E-05 | 0.98158 | + | 2.93213 | 6.63816 | | 16.0105 | 16.0845 | 16.1243 | NaN | 15.1205 | 15.1115 | 15.3656 | 14.7684 | 4 | 4 | 6 | NaN | 4 | 2 | 3 | 3 |
| CEP78 | Q5JTW2;Q5 | 0.000604 | 0.000128 | 0.982733 | | 0.869946 | 1.727 | | 14.004 | 13.7555 | 13.3046 | 13.1362 | 11.3136 | 13.3115 | 13.5301 | 12.235 | 1 | 1 | 1 | 1 | 1 | 1 | 1 | 1 |
| TYMP | P19971;P1 | 4.44E-17 | 1.16E-16 | 0.989315 | | 1.11082 | 2.12749 | | 15.7299 | 15.6203 | 15.9723 | 13.8969 | 14.586 | 15.8114 | 15.307 | 14.3229 | 9 | 9 | 10 | 3 | 7 | 10 | 10 | 4 |
| TNS3 | Q68CZ2 | 3.55E-19 | 1.83E-18 | 0.992158 | + | 1.94969 | 3.61021 | | 16.9621 | 17.0299 | 17.0091 | 15.7489 | 16.003 | 16.6276 | 16.2391 | 15.4226 | 28 | 28 | 27 | 25 | 23 | 26 | 27 | 25 |
| OPLAH | O14841 | 6.75E-16 | 1.21E-15 | 1.00412 | | 0.784372 | 1.58395 | | 15.9592 | 15.9701 | 15.9905 | 13.173 | 15.1689 | 15.8231 | 15.6937 | 14.987 | 24 | 23 | 22 | 7 | 11 | 22 | 21 | 23 |
| FAM162A | Q96A26 | 2.68E-09 | 1.05E-09 | 1.01429 | + | 1.55152 | 2.87925 | | 17.0278 | 16.9628 | 17.2186 | 16.7663 | 15.4672 | 15.5402 | 15.963 | 16.5405 | 7 | 7 | 7 | 5 | 4 | 5 | 5 | 6 |
| TPST2 | O60704 | 3.63E-10 | 1.59E-10 | 1.01577 | + | 2.25579 | 4.222 | | 16.3976 | 16.4063 | 16.3805 | 14.8152 | 15.326 | 15.3893 | 15.9526 | 15.4121 | 6 | 6 | 6 | 4 | 4 | 6 | 5 | 5 |
| ALDH6A1 | Q02252;Q( | 1.79E-14 | 2.11E-14 | 1.01876 | + | 3.41576 | 7.12747 | | 17.6642 | 17.6526 | 17.6245 | 16.3152 | 16.5697 | 16.985 | 16.6268 | 16.645 | 18 | 18 | 18 | 12 | 14 | 15 | 14 | 13 |
| VAMP5 | O95183 | 0.000381 | 8.33E-05 | 1.02085 | | 0.838933 | 1.67528 | | 17.3039 | 17.2975 | 17.2427 | 17.1459 | 14.7747 | 16.3463 | 15.7954 | 17.2403 | 2 | 2 | 2 | 2 | 2 | 2 | 2 | 2 |
| SNX18 | Q96RF0-2 | 3.59E-24 | 9.67E-23 | 1.02226 | + | 3.0046 | 5.97087 | | 17.596 | 17.6026 | 17.6215 | 16.7396 | 16.4562 | 16.9884 | 16.2407 | 16.4974 | 19 | 19 | 19 | 18 | 16 | 19 | 17 | 17 |
| THEM6 | Q8WUY1 | 3.49E-13 | 2.86E-13 | 1.03889 | | 1.08633 | 2.08669 | | 15.8052 | 15.8024 | 15.8384 | 14.0098 | 15.3088 | 13.7537 | 15.6055 | 15.2044 | 8 | 8 | 8 | 4 | 8 | 3 | 8 | 8 |
| SLC35F5 | Q8WV83;Q | 0.015535 | 0.002611 | 1.03974 | | 1.01961 | 2.1721 | | 15.1651 | 15.4258 | 15.0494 | 14.6819 | NaN | 13.2439 | NaN | 14.5953 | 2 | 2 | 2 | 1 | NaN | 2 | NaN | 1 |
| SNRPF | P62306 | 1.22E-05 | 3.17E-06 | 1.0398 | | 0.689081 | 1.42293 | | 16.2707 | 16.1831 | 16.4558 | 16.0197 | 14.7482 | 13.5806 | 15.1905 | 16.7781 | 6 | 5 | 5 | 5 | 3 | 2 | 4 | 5 |
| COL7A1 | Q02388;Q( | 1.26E-05 | 3.27E-06 | 1.04216 | | 0.777809 | 1.61702 | | 16.4829 | 16.3783 | 16.397 | NaN | 15.9163 | 13.8528 | 16.3464 | 15.3935 | 13 | 15 | 12 | NaN | 7 | 5 | 14 | 5 |
| LUM | P51884 | 5.31E-11 | 2.69E-11 | 1.04258 | | 0.737174 | 1.50448 | | 18.1271 | 18.1561 | 18.1556 | 15.4264 | 16.9782 | 16.8719 | 18.5949 | 17.6472 | 10 | 10 | 10 | 7 | 9 | 9 | 10 | 10 |
| COL6A3 | P12111 | 2.57E-12 | 1.68E-12 | 1.04663 | + | 3.2106 | 6.53024 | | 20.4494 | 20.4589 | 20.442 | 19.1542 | 19.7821 | 19.2914 | 19.5827 | 19.2069 | 159 | 158 | 159 | 146 | 146 | 152 | 150 | 145 |
| SNX25 | Q9H3E2 | 0.002603 | 0.000495 | 1.05797 | | 0.427122 | 0.999758 | | 13.6393 | 13.8943 | 13.8318 | 13.1526 | NaN | 10.7283 | NaN | 14.3106 | 1 | 2 | 1 | 3 | NaN | 1 | NaN | 1 |
| C1S | P09871 | 3.69E-09 | 1.42E-09 | 1.05944 | + | 2.02471 | 4.09 | | 15.5397 | 15.6155 | 15.5875 | 14.3055 | 14.613 | 15.0884 | NaN | 14.079 | 4 | 5 | 5 | 3 | 1 | 4 | NaN | 3 |
| PDCD4 | Q53EL6;Q5 | 3.02E-19 | 1.59E-18 | 1.06032 | + | 2.58983 | 4.95254 | | 17.1497 | 17.1733 | 17.1549 | 15.9893 | 16.3526 | 16.3122 | 15.5143 | 16.3263 | 18 | 18 | 18 | 15 | 14 | 15 | 12 | 18 |
| MYO19 | Q96H55 | 0.003169 | 0.000587 | 1.06789 | | 1.04177 | 2.21785 | | 12.5728 | 12.4406 | 12.6922 | 11.8819 | NaN | 10.5546 | 12.0654 | NaN | 2 | 2 | 2 | 1 | NaN | 2 | 2 | NaN |
| SERPING1 | P05155;P0 | 6.07E-08 | 2.01E-08 | 1.07243 | | 1.11256 | 2.21959 | | 14.4867 | 14.2354 | 14.4896 | NaN | 14.2391 | 13.4686 | 13.3429 | 12.2752 | 4 | 4 | 4 | NaN | 2 | 2 | 3 | 1 |
| ITGA11 | Q9UKX5;Q9 | 1.87E-14 | 2.19E-14 | 1.07274 | | 1.11795 | 2.13937 | | 18.4726 | 18.4572 | 18.4505 | 16.728 | 16.8314 | 18.5681 | 17.9919 | 16.8174 | 34 | 33 | 35 | 28 | 26 | 32 | 32 | 29 |
| SLIT3 | O75094 | 2.28E-15 | 3.53E-15 | 1.07513 | + | 1.38915 | 2.59716 | | 16.2246 | 16.2563 | 16.29 | 14.6029 | 15.3793 | 16.2707 | 14.5875 | 15.0687 | 37 | 38 | 37 | 27 | 28 | 37 | 26 | 32 |
| DCN | P07585 | 5.85E-10 | 2.49E-10 | 1.07561 | + | 1.73507 | 3.20838 | | 16.9913 | 17.0909 | 17.0213 | 14.9694 | 16.2321 | 16.2614 | 16.2847 | 16.0471 | 8 | 8 | 8 | 5 | 7 | 7 | 8 | 6 |
| COX6C | P09669 | 4.75E-06 | 1.29E-06 | 1.0757 | | 0.857966 | 1.70703 | | 17.7519 | 17.595 | 17.8178 | 17.5784 | 15.9083 | 15.2703 | 16.7677 | 17.7045 | 10 | 10 | 10 | 10 | 7 | 9 | 10 | 10 |
| H1-3 | P16402 | 0.000223 | 5.03E-05 | 1.08944 | | 0.707971 | 1.45504 | | 18.6654 | 17.6055 | 17.9065 | 15.7946 | 18.8165 | 16.4698 | 16.312 | 17.4555 | 2 | 2 | 2 | 1 | 2 | 2 | 1 | 2 |
| PFDN6 | O15212 | 7.27E-05 | 1.75E-05 | 1.09208 | | 0.840621 | 1.67809 | | 18.0623 | 18.0993 | 18.2018 | 18.357 | 16.1005 | 16.1555 | 16.4745 | 18.0576 | 5 | 5 | 5 | 5 | 3 | 4 | 5 | 5 |
| PPP1R14B | Q96C90 | 2.56E-24 | 7.58E-23 | 1.09405 | | 0.982889 | 1.91476 | | 17.8329 | 17.072 | 18.1372 | 17.463 | 15.8245 | 15.7157 | 16.3832 | 17.5469 | 4 | 5 | 5 | 4 | 3 | 3 | 3 | 3 |
| MRPL51 | Q4U2R6 | 0.003049 | 0.000567 | 1.09854 | + | 1.46652 | 2.73064 | | 16.9647 | 16.5366 | 16.8493 | 16.7097 | 15.2614 | 15.7461 | 14.9863 | 15.7215 | 1 | 1 | 1 | 1 | 2 | 1 | 1 | 2 |
| DCXR | Q7Z4W1 | 3.33E-12 | 2.12E-12 | 1.10047 | | 1.26637 | 2.38827 | | 16.9078 | 16.9326 | 16.8336 | 16.0934 | 16.2864 | 16.1676 | 14.4243 | 15.9826 | 8 | 8 | 8 | 8 | 6 | 8 | 5 | 7 |
| HLA-C | P10321 | 1.33E-09 | 5.42E-10 | 1.11567 | + | 2.45787 | 4.65529 | | 18.8965 | 18.9182 | 18.8463 | 17.1074 | 18.1068 | 17.9144 | 18.0259 | 17.702 | 13 | 13 | 13 | 11 | 13 | 12 | 12 | 12 |
| LMCD1 | Q9NZU5 | 6.12E-11 | 3.06E-11 | 1.11614 | | 1.07653 | 2.07038 | | 17.7956 | 17.7572 | 17.7081 | 15.6814 | 16.6811 | 18.1097 | 16.4706 | 16.2446 | 23 | 23 | 23 | 18 | 18 | 24 | 20 | 20 |
| LDB1 | Q86U70;Q8 | 3.10E-06 | 8.54E-07 | 1.12316 | | 0.365594 | 0.856335 | | 15.7578 | 15.9865 | 15.9102 | 15.7933 | 11.4402 | 15.9055 | NaN | 15.9077 | 2 | 3 | 4 | 4 | 2 | 3 | NaN | 3 |
| GHDC | Q8N2G8;Q | 0.001558 | 0.000312 | 1.12394 | | 0.96877 | 1.89132 | | 15.7891 | 15.719 | 15.7461 | 13.7054 | 15.0438 | 15.1679 | 15.7621 | 13.4581 | 10 | 10 | 8 | 3 | 4 | 10 | 8 | 3 |
| TRPV2 | Q9Y5S1 | 2.35E-12 | 1.55E-12 | 1.12996 | + | 2.91818 | 5.74735 | | 17.549 | 17.49 | 17.5232 | 16.7933 | 16.1451 | 16.2129 | 16.7003 | 16.1022 | 16 | 16 | 14 | 14 | 11 | 13 | 13 | 13 |
| PDGFRB | P09619 | 2.71E-12 | 1.77E-12 | 1.13362 | + | 1.39504 | 2.60727 | | 18.3841 | 18.3585 | 18.3448 | 16.0425 | 17.6071 | 17.9626 | 17.3973 | 17.1349 | 28 | 28 | 30 | 25 | 28 | 28 | 28 | 28 |
| CASP1 | P29466;P2 | 2.43E-05 | 6.14E-06 | 1.1415 | + | 2.29475 | 4.3036 | | 16.1542 | 16.2271 | 16.1999 | 14.3094 | 15.3824 | 15.2357 | 15.3511 | 14.9824 | 11 | 11 | 11 | 6 | 9 | 10 | 10 | 8 |
| NAP1L1 | P55209-3 | 1.07E-09 | 4.41E-10 | 1.14432 | | 0.934879 | 1.83502 | | 15.4151 | 15.5684 | 15.576 | 15.8744 | 13.9607 | 14.8002 | 13.0639 | 14.1783 | 2 | 2 | 2 | 2 | 1 | 2 | 1 | 2 |
| SNRPE | P62304 | 0.000318 | 7.03E-05 | 1.14587 | + | 1.70969 | 3.16212 | | 19.5257 | 19.4508 | 19.3509 | 18.988 | 18.0182 | 17.7273 | 17.8409 | 18.9086 | 4 | 4 | 4 | 4 | 4 | 4 | 4 | 4 |
| SEMA7A | O75326 | 2.16E-16 | 4.57E-16 | 1.15602 | + | 2.4247 | 4.5824 | | 17.1538 | 17.2339 | 17.2128 | 15.5832 | 15.8887 | 16.4625 | 15.7681 | 16.5181 | 20 | 20 | 20 | 18 | 18 | 18 | 18 | 19 |
| COL3A1 | P02461 | 3.80E-16 | 7.26E-16 | 1.16987 | | 1.22185 | 2.31327 | | 18.0702 | 18.0698 | 18.0844 | 16.6569 | 16.218 | 18.1979 | 17.2773 | 16.1746 | 38 | 39 | 39 | 30 | 25 | 40 | 33 | 30 |
| VASN | Q6EMK4 | 1.15E-10 | 5.49E-11 | 1.17321 | + | 2.63478 | 5.05655 | | 17.6575 | 17.6508 | 17.7454 | 16.5439 | 16.7979 | 16.9716 | 16.1491 | 16.0943 | 12 | 12 | 12 | 11 | 8 | 11 | 10 | 10 |
| PDE1C | Q14123;Q1 | 4.79E-12 | 2.95E-12 | 1.18173 | | 0.604657 | 1.27786 | | 16.3172 | 16.3749 | 16.3786 | 12.6269 | 15.7959 | 15.8795 | 16.6516 | 14.9219 | 12 | 14 | 15 | 4 | 10 | 12 | 14 | 10 |
| HDAC5 | Q9UQL6;Q9 | 0.003994 | 0.000725 | 1.18263 | | 1.07192 | 2.14539 | | 14.6404 | 14.8658 | 14.6646 | 13.9176 | NaN | 12.1554 | 14.0855 | 14.0054 | 1 | 1 | 3 | 1 | NaN | 2 | 1 | 1 |
| THNSL1 | Q8IYQ7 | 2.75E-06 | 7.64E-07 | 1.19785 | | 0.503239 | 1.20713 | | 14.51 | 14.868 | 14.4771 | NaN | 14.734 | 12.1069 | NaN | NaN | 3 | 4 | 1 | NaN | 1 | 2 | NaN | NaN |
| HLA-H | P01893 | 6.79E-17 | 1.69E-16 | 1.21644 | + | 1.53536 | 2.85083 | | 17.8084 | 17.8924 | 17.8811 | 16.1154 | 16.5088 | 16.6136 | 17.8587 | 16.1244 | 2 | 2 | 2 | 2 | 2 | 2 | 1 | 2 |
| APOBEC3F | Q8IUX4 | 1.05E-09 | 4.33E-10 | 1.23217 | + | 1.38783 | 2.59489 | | 12.5117 | 12.8833 | 12.5341 | 11.2049 | 10.9025 | 12.1286 | 12.313 | 10.5054 | 1 | 1 | 2 | 1 | 1 | 1 | 2 | 1 |
| SLC9A9 | Q8IVB4 | 4.62E-09 | 1.75E-09 | 1.23916 | + | 3.21833 | 6.55202 | | 14.4638 | 14.3461 | 14.2481 | 13.4957 | 12.7318 | 13.1699 | 12.8819 | 13.2882 | 2 | 2 | 2 | 2 | 1 | 1 | 1 | 1 |
| TNC | P24821 | 1.26E-12 | 8.92E-13 | 1.24564 | + | 1.7077 | 3.15851 | | 16.799 | 16.8705 | 16.8492 | 14.6496 | 15.8069 | 16.4504 | 15.7166 | 15.3462 | 47 | 51 | 51 | 40 | 45 | 49 | 44 | 42 |
| GSTT2;GSTT2B | P0CG29;P0 | 1.73E-08 | 6.12E-09 | 1.24599 | | 0.708585 | 1.45608 | | 16.2659 | 16.1141 | 16.216 | 13.475 | 16.796 | 16.0802 | 14.5653 | 13.8468 | 6 | 5 | 4 | 3 | 6 | 6 | 4 | 2 |
| TMEM167A | Q8TBQ9 | 0.001994 | 0.000391 | 1.25625 | + | 4.76714 | 12.362 | | 20.2826 | 20.1853 | 20.0772 | 18.8685 | 18.752 | 18.8597 | 19.1559 | 18.9913 | 2 | 2 | 2 | 2 | 2 | 2 | 2 | 2 |
| CKB | P12277 | 4.79E-13 | 3.76E-13 | 1.26315 | + | 2.12218 | 3.94883 | | 18.812 | 18.8016 | 18.8239 | 16.8521 | 17.3725 | 18.3212 | 17.4728 | 17.7281 | 16 | 16 | 16 | 15 | 16 | 16 | 16 | 16 |
| BDH1 | Q02338 | 0.00304 | 0.000566 | 1.29405 | + | 2.45512 | 4.64923 | | 17.3101 | 17.5568 | 17.6701 | 16.1359 | 16.3827 | 16.8918 | 15.9644 | 15.7165 | 2 | 2 | 2 | 1 | 1 | 2 | 1 | 1 |
| CCDC80 | Q76M96;Q | 2.98E-06 | 8.23E-07 | 1.31611 | + | 1.42738 | 2.66291 | | 15.6102 | 15.6691 | 15.7113 | 12.8861 | 14.7281 | 14.9008 | 14.7039 | 14.5181 | 15 | 16 | 16 | 8 | 10 | 12 | 11 | 12 |
| CTHRC1 | Q96CG8 | 5.11E-10 | 2.19E-10 | 1.32352 | | 1.26218 | 2.38119 | | 19.2971 | 19.3388 | 19.2874 | 18.1903 | 16.9973 | 19.431 | 17.9185 | 17.3842 | 7 | 7 | 7 | 7 | 7 | 8 | 7 | 7 |
| TTC19 | Q6DKK2 | 7.31E-06 | 1.95E-06 | 1.34363 | | 0.985909 | 2.31773 | | 14.8597 | 14.2401 | 14.4959 | NaN | NaN | 12.4756 | NaN | 13.901 | 1 | 1 | 2 | NaN | NaN | 1 | NaN | 1 |
| WDR54 | Q9H977;Q9 | 0.003152 | 0.000584 | 1.34985 | | 0.537163 | 1.21759 | | 13.9468 | 14.3046 | 14.4617 | 14.8695 | NaN | 12.717 | 11.0771 | NaN | 3 | 3 | 3 | 1 | NaN | 1 | 2 | NaN |
| EFEMP1 | Q12805;Q1 | 4.34E-14 | 4.58E-14 | 1.35361 | + | 2.8057 | 6.22548 | | 16.5382 | 16.5794 | 16.5722 | 14.8175 | NaN | 15.7011 | 15.2051 | 15.115 | 4 | 5 | 6 | 3 | NaN | 5 | 3 | 4 |
| SLC1A3 | P43003;P4 | 0.002194 | 0.000426 | 1.37052 | + | 3.07025 | 6.14497 | | 16.1079 | 16.0698 | 15.9116 | 14.2596 | 14.6796 | 15.2474 | 14.484 | 14.6254 | 2 | 2 | 3 | 2 | 2 | 2 | 2 | 2 |

| Gene names | UniProt IDs | Protein Group P-value | Protein Group Q-value | Log2 Difference NUP214 vs Controls (n=5) | p<0.05 | -log10 p-value | T-test Test statistic | NPC Localization | Log2 MS2_quantity_NUP214_1 | Log2 MS2_quantity_NUP214_2 | Log2 MS2_quantity_NUP214_3 | Log2 MS2_quantity_Control_1 | Log2 MS2_quantity_Control_2 | Log2 MS2_quantity_Control_3 | Log2 MS2_quantity_Control_4 | Log2 MS2_quantity_Control_5 | Number Peptides for Quantitation NUP214_1 | Number Peptides for Quantitation NUP214_2 | Number Peptides for Quantitation NUP214_3 | Number Peptides for Quantitation Control_1 | Number Peptides for Quantitation Control_2 | Number Peptides for Quantitation Control_3 | Number Peptides for Quantitation Control_4 | Number Peptides for Quantitation Control_5 |
|---|---|---|---|---|---|---|---|---|---|---|---|---|---|---|---|---|---|---|---|---|---|---|---|---|
| CMTR2 | Q8IYT2 | 0.000453 | 9.81E-05 | 1.38971 | + | 2.70343 | 5.21824 | | 14.2208 | 14.1772 | 14.3557 | 13.6502 | 12.6535 | 12.6923 | 12.6877 | 12.6239 | 2 | 2 | 2 | 2 | 1 | 1 | 1 | 1 |
| ATM | Q13315 | 5.60E-05 | 1.37E-05 | 1.39781 | + | 2.44688 | 5.16367 | | 14.8677 | 14.9496 | 14.8809 | 12.993 | 13.6178 | 14.0687 | NaN | 13.3269 | 9 | 9 | 8 | 2 | 1 | 1 | NaN | 1 |
| AKR1C3 | P42330 | 3.05E-06 | 8.43E-07 | 1.41241 | | 0.954444 | 1.86752 | | 17.5066 | 17.5274 | 17.4081 | 15.4469 | 16.3946 | 17.559 | 14.2463 | 16.6947 | 7 | 9 | 7 | 5 | 5 | 7 | 5 | 6 |
| AIG1 | Q9NVV5;Q | 0.000588 | 0.000125 | 1.41609 | + | 2.91785 | 5.74651 | | 16.107 | 16.1667 | 16.3476 | 14.1702 | 14.6909 | 15.2537 | 14.9868 | 14.8534 | 2 | 2 | 2 | 2 | 1 | 2 | 2 | 2 |
| DPM3 | Q9P2X0;Q | 0.000659 | 0.00014 | 1.42489 | + | 2.56164 | 4.88803 | | 15.2434 | 14.8416 | 15.2402 | 13.4734 | 13.3132 | 13.3648 | 13.8485 | 14.4177 | 2 | 2 | 2 | 2 | 2 | 2 | 2 | 2 |
| DAG1 | Q14118 | 1.61E-07 | 5.12E-08 | 1.42594 | + | 2.13966 | 3.98401 | | 14.825 | 15.1152 | 15.0576 | 13.2598 | 13.9171 | 14.4491 | 13.1127 | 13.128 | 5 | 5 | 5 | 1 | 2 | 2 | 4 | 3 |
| CTSK | P43235 | 4.54E-08 | 1.52E-08 | 1.43325 | + | 2.74905 | 5.32761 | | 18.5267 | 18.5038 | 18.3325 | 16.3403 | 17.2134 | 17.1482 | 17.5256 | 16.8779 | 11 | 11 | 11 | 9 | 8 | 9 | 8 | 9 |
| TTC38 | Q5R3I4 | 5.97E-05 | 1.45E-05 | 1.43475 | + | 3.81 | 8.40171 | | 16.9426 | 16.9612 | 17.0577 | 15.458 | 15.3966 | 15.6973 | 15.9662 | 15.2441 | 6 | 6 | 6 | 1 | 3 | 2 | 6 | 3 |
| CSRP2 | Q16527 | 7.90E-17 | 1.91E-16 | 1.44497 | + | 1.38676 | 2.59306 | | 17.6869 | 17.6462 | 17.6273 | 14.8814 | 16.2369 | 16.5484 | 17.4476 | 15.9282 | 10 | 10 | 10 | 9 | 10 | 10 | 10 | 10 |
| INO80 | Q9ULG1 | 0.003123 | 0.000579 | 1.44725 | | 0.295552 | 0.802763 | | NaN | 12.9291 | 12.9939 | 13.3168 | NaN | NaN | NaN | 9.7117 | NaN | 2 | 1 | 1 | NaN | NaN | NaN | 2 |
| CTH | P32929 | 1.37E-06 | 3.93E-07 | 1.48202 | | 1.2963 | 2.4389 | | 15.4542 | 15.3158 | 15.4211 | 13.9642 | 13.5269 | 15.3751 | 12.5701 | 14.1385 | 9 | 10 | 10 | 3 | 4 | 8 | 1 | 2 |
| LRRC15 | Q8TF66;Q8 | 3.72E-05 | 9.24E-06 | 1.50927 | + | 3.27479 | 6.71262 | | 19.5235 | 19.5218 | 19.4713 | 17.9426 | 17.5038 | 17.854 | 18.5198 | 18.1613 | 15 | 15 | 15 | 15 | 15 | 15 | 15 | 15 |
| SLC25A40 | Q8TBP6 | 1.29E-07 | 4.13E-08 | 1.52565 | + | 2.20331 | 4.52367 | | 15.1345 | 14.9932 | 15.131 | NaN | 13.0609 | 14.3724 | 13.3576 | 13.4514 | 3 | 4 | 3 | NaN | 2 | 3 | 2 | 2 |
| GXYLT2 | A0PJZ3 | 0.002312 | 0.000446 | 1.56197 | | 1.28552 | 2.5412 | | 12.73 | 12.5159 | 12.9864 | 10.7156 | 10.7072 | 12.7114 | 10.5943 | NaN | 2 | 2 | 1 | 1 | 1 | 1 | 2 | NaN |
| C1R | P00736 | 2.34E-12 | 1.54E-12 | 1.57956 | + | 1.6373 | 3.58563 | | 15.7803 | 15.6533 | 15.6939 | 13.32 | 14.2404 | 14.8283 | NaN | NaN | 8 | 6 | 9 | 1 | 3 | 6 | NaN | NaN |
| PTGIS | Q16647 | 4.84E-09 | 1.83E-09 | 1.61259 | | 1.26953 | 2.3936 | | 17.3836 | 17.4789 | 17.4457 | 14.4579 | 16.2164 | 17.1563 | 14.8556 | 16.4312 | 14 | 16 | 16 | 4 | 13 | 15 | 11 | 14 |
| COL8A1 | P27658 | 4.53E-06 | 1.23E-06 | 1.6138 | | 1.22934 | 2.32587 | | 18.7191 | 18.6835 | 18.7068 | 16.1012 | 17.2854 | 16.2543 | 18.9937 | 16.8122 | 12 | 11 | 11 | 7 | 7 | 5 | 11 | 8 |
| CRIP1 | P50238 | 0.003536 | 0.000648 | 1.64932 | | 1.2321 | 2.44074 | | 21.0683 | 21.065 | 21.0364 | NaN | 18.5186 | 21.0787 | 18.9073 | 19.1246 | 2 | 1 | 2 | NaN | 1 | 2 | 1 | 1 |
| EDIL3 | O43854;O | 3.62E-10 | 1.58E-10 | 1.69762 | + | 2.41323 | 4.55736 | | 17.3276 | 17.3233 | 17.2733 | 16.1224 | 16.2026 | 14.6891 | 15.318 | 15.7201 | 17 | 17 | 17 | 17 | 15 | 12 | 14 | 16 |
| MYO1D | O94832 | 1.06E-16 | 2.51E-16 | 1.74564 | + | 1.74982 | 3.23537 | | 18.8596 | 18.8917 | 18.8279 | 16.0535 | 16.6978 | 18.3985 | 17.6018 | 16.8188 | 56 | 56 | 56 | 47 | 47 | 56 | 55 | 52 |
| TENM3 | Q9P273 | 8.95E-09 | 3.27E-09 | 1.88467 | + | 2.99959 | 6.86741 | | 15.8323 | 15.7289 | 15.7057 | 13.1925 | NaN | 14.189 | 13.9798 | 14.1224 | 9 | 9 | 9 | 1 | NaN | 4 | 3 | 1 |
| H1-1 | Q02539 | 0.002498 | 0.000477 | 2.05342 | + | 3.58239 | 12.1706 | | 15.5699 | 15.7096 | 15.5335 | 13.263 | 13.8156 | NaN | 13.5741 | NaN | 2 | 2 | 2 | 1 | 2 | NaN | 2 | NaN |
| COL14A1 | Q05707;Q | 1.15E-07 | 3.69E-08 | 2.19506 | + | 1.87547 | 3.46896 | | 19.3959 | 19.4173 | 19.3713 | 17.4144 | 16.6776 | 18.951 | 16.6722 | 16.2835 | 39 | 39 | 39 | 28 | 8 | 36 | 11 | 4 |
| UNC5B | Q8IZJ1;Q8I | 7.16E-05 | 1.72E-05 | 2.34053 | + | 1.32258 | 2.4835 | | 16.2475 | 16.097 | 16.063 | 11.6095 | 13.8586 | 13.8805 | 16.0606 | 13.5673 | 3 | 3 | 3 | 1 | 1 | 1 | 3 | 1 |
| COL15A1 | P39059 | 0.000163 | 3.75E-05 | 2.36979 | + | 2.50439 | 6.36128 | | 17.9591 | 17.9561 | 17.9784 | NaN | 15.8426 | 14.8625 | 16.0792 | NaN | 11 | 11 | 11 | NaN | 6 | 6 | 10 | NaN |
| ADCY7 | P51828 | 0.005411 | 0.000961 | 2.7332 | | 0.503352 | 1.11952 | | 14.7184 | 14.5964 | 14.2838 | 14.1043 | 13.5146 | 13.9527 | 5.62707 | NaN | 3 | 3 | 5 | 4 | 2 | 4 | 1 | NaN |
| TP53I11 | O14683 | 0.000388 | 8.47E-05 | 2.82776 | + | 1.98205 | 3.6726 | | 16.9087 | 16.7557 | 16.8324 | 12.0188 | 14.3597 | 13.9626 | 14.0699 | 15.6115 | 4 | 4 | 4 | 1 | 3 | 3 | 4 | 4 |
| ENPP1 | P22413 | 2.47E-15 | 3.79E-15 | 3.27885 | + | 3.61819 | 7.75972 | | 18.428 | 18.5041 | 18.4958 | 14.1924 | 14.8177 | 15.7308 | 15.3021 | 15.9427 | 25 | 26 | 25 | 16 | 15 | 22 | 22 | 21 |

**Supplementary Table 5: Clinical utility data collection instrument**

| | |
|---|---|
| Participant first name | *Pre-filled* |
| Participant surname | *Pre-filled* |
| Hospital UR number | *Pre-filled* |
| Result: | *Pre-filled*<br>☐ A diagnosis was made<br>☐ A partial diagnosis was made<br>☐ More than 1 diagnosis was made<br>☐ A diagnosis was not made |
| Date patient discharged from hospital: | |
| Date of death (if relevant): | |
| Date of discharge from ICU (if relevant): | |
| **Changes in patient management arising from result** | |
| Medication started | ☐ Yes ☐ No<br>Details:<br>____________________<br>____________________ |
| Medication stopped | ☐ Yes ☐ No<br>Details:<br>____________________<br>____________________ |
| Medication adjusted | ☐ Yes ☐ No<br>Details:<br>____________________<br>____________________ |
| Investigation cancelled | ☐ Yes ☐ No<br>Details:<br>____________________<br>____________________ |
| Additional investigation ordered | ☐ Yes ☐ No<br>Details:<br>____________________<br>____________________ |
| Subspecialist referral initiated | ☐ Yes ☐ No<br>Details:<br>____________________<br>____________________ |

| | |
|---|---|
| Prior subspecialist service no longer required | ☐ Yes ☐ No<br>Details:<br>________________________________________<br>________________________________________ |
| Surgical procedure initiated (incl biopsy) | ☐ Yes ☐ No<br>Details:<br>________________________________________<br>________________________________________ |
| Surgical procedure cancelled | ☐ Yes ☐ No<br>Details:<br>________________________________________<br>________________________________________ |
| Surgical procedure changed | ☐ Yes ☐ No<br>Details:<br>________________________________________<br>________________________________________ |
| Management redirected towards palliation | ☐ Yes ☐ No<br>Details:<br>________________________________________<br>________________________________________ |
| Decision to palliate reversed | ☐ Yes ☐ No<br>Details:<br>________________________________________<br>________________________________________ |
| Patient eligibility for a new research study affected | ☐ Yes ☐ No<br>Details:<br>________________________________________<br>________________________________________ |
| Was there a change in management as a result of the genomic testing in this patient? | ☐ Yes ☐ No<br>Details:<br>________________________________________<br>________________________________________ |
| Additional family members tested (e.g. sibs) | ☐ Yes ☐ No<br>If yes, outcome:<br>________________________________________<br>________________________________________ |
| Reproductive risk established for parents? | ☐ Yes ☐ No<br>If yes:<br>☐ <1% ☐ 25% ☐ 50% ☐ Other |

| | |
|---|---|
| How do you rate the clinical utility of genomic testing for this patient? | ☐ Neutral<br>☐ Useful<br>☐ Very useful<br>☐ Not useful at all<br>☐ Not very useful |
| Do you think the length of ICU stay was shortened by genomic testing? | ☐ Yes ☐ No<br>If so, by how many days? ___________<br>Explain: ___________________________________________________ |
| Do you think the length of ICU stay was extended by genomic testing? | ☐ Yes ☐ No<br>If so, by how many days? ___________<br>Explain: ___________________________________________________ |
| Genomic results (tick all that apply) | ☐ Enabled cessation of additional testing<br>☐ Required additional testing to confirm diagnosis<br>☐ Allowed avoidance of complications<br>☐ Required additional testing to screen for complications<br>☐ Enabled targeted treatment that may improve long-term outcomes<br>☐ Enabled improved communication of outcomes/expectations/prognosis with the family<br>☐ Decreased stress and confusion for the family<br>☐ Increased stress and confusion for the family<br>☐ Decreased confusion among medical staff<br>☐ Increased confusion among medical staff<br>☐ Resulted in a diagnosis not fully understood at this time<br>Comment:<br>____________________________________________________________<br>____________________________________________________________<br>____________________________________________________________<br>____________________________________________________________<br>____________________________________________________________ |
