# Supplementary material for: Integrated multi-omics for rapid rare disease diagnosis on a national scale
Source: Nat Med. 2023 Jun 8;29(7):1681–91. doi: 10.1038/s41591-023-02401-9 (PMC10353936; doi:10.1038/s41591-023-02401-9)
Supplement: Supplementary file 2 — Reporting Summary [file 41591_2023_2401_MOESM2_ESM.pdf]

nature portfolio

Corresponding author(s): Zornitza Stark

Last updated by author(s): 2022/12/24

Reporting Summary

Nature Portfolio wishes to improve the reproducibility of the work that we publish. This form provides structure for consistency and transparency in reporting. For further information on Nature Portfolio policies, see our Editorial Policies and the Editorial Policy Checklist.

Statistics

For all statistical analyses, confirm that the following items are present in the figure legend, table legend, main text, or Methods section.

n/a	Confirmed	
☐	☒	The exact sample size (*n*) for each experimental group/condition, given as a discrete number and unit of measurement
☒	☐	A statement on whether measurements were taken from distinct samples or whether the same sample was measured repeatedly
☐	☒	The statistical test(s) used AND whether they are one- or two-sided<br>*Only common tests should be described solely by name; describe more complex techniques in the Methods section.*
☒	☐	A description of all covariates tested
☒	☐	A description of any assumptions or corrections, such as tests of normality and adjustment for multiple comparisons
☐	☒	A full description of the statistical parameters including central tendency (e.g. means) or other basic estimates (e.g. regression coefficient) AND variation (e.g. standard deviation) or associated estimates of uncertainty (e.g. confidence intervals)
☐	☒	For null hypothesis testing, the test statistic (e.g. *F*, *t*, *r*) with confidence intervals, effect sizes, degrees of freedom and *P* value noted<br>*Give P values as exact values whenever suitable.*
☒	☐	For Bayesian analysis, information on the choice of priors and Markov chain Monte Carlo settings
☒	☐	For hierarchical and complex designs, identification of the appropriate level for tests and full reporting of outcomes
☒	☐	Estimates of effect sizes (e.g. Cohen's *d*, Pearson's *r*), indicating how they were calculated

Our web collection on statistics for biologists contains articles on many of the points above.

Software and code

Policy information about availability of computer code

Data collection	REDCap database for clinical data collection V12.5.16 (reference provided)
Data analysis	Illumina next generation sequencing software (Dragen V3.3.7); Alissa Interpret software suite V5.2.9 (Agilent); custom CNV detection tool, CxGo V1.0.8 - V1.6.0 (reference provided), PanelApp software V3.1.1 (reference provided); STRipy for STR detection V2.0 (reference provided); Manta for structural variant detection V1.6.0 (reference provided); Schism for structural variant detection V1.0 (https://github.com/ssadedin/schism); Outrider for RNA analysis V1.12.0 (reference provided); R V4.2; Variant Grid V3 (variantgrid.com; https://github.com/SACGF/variantgrid); Image Lab Version 6.0; PyMOL Molecular Graphics System version 1.7.2.1

For manuscripts utilizing custom algorithms or software that are central to the research but not yet described in published literature, software must be made available to editors and reviewers. We strongly encourage code deposition in a community repository (e.g. GitHub). See the Nature Portfolio guidelines for submitting code & software for further information.

Data

Policy information about availability of data

All manuscripts must include a data availability statement. This statement should provide the following information, where applicable:

- Accession codes, unique identifiers, or web links for publicly available datasets
- A description of any restrictions on data availability
- For clinical datasets or third party data, please ensure that the statement adheres to our policy

De-identified genomic and associated data from this study are available for ethically approved research. Data access requests are accepted via an online application form that will require approval from the Australian Genomics Data Access Committee. For access to the data, please email AG-datarequest@mcri.edu.au. Data access requests are reviewed by the committee once a month. Access to the data will require a Data Transfer Agreement (DTA). Once a DTA is signed, the data will be transferred to the requestor from AWS S3 storage. All variants reported in this study have been deposited in ClinVar (SUB13026601, SCV003921769 - SCV003922018). The mass spectrometry proteomics data have been deposited to the ProteomeXchange Consortium via the PRIDE partner repository with the dataset identifier PXD042001.

Web resources used:

ClinVar: https://www.ncbi.nlm.nih.gov/clinvar

GATK: https://gatk.broadinstitute.org

gnomAD: https://gnomad.broadinstitute.org

The Human Phenotype Ontology: https://hpo.jax.org/app

The Human Ancestry Ontology: https://www.ebi.ac.uk/ols/ontologies/hancestro

IGV: https://software.broadinstitute.org/software/igv

NCBI RefSeq: https://www.ncbi.nlm.nih.gov/refseq

Manta: https://github.com/Illumina/manta

OMIM: https://omim.org

PanelApp Australia: https://panelapp.agha.umccr.org/

Schism: https://github.com/ssadedin/schism

STRipy: https://stripy.org

UniProt: https://www.uniprot.org/

VariantGrid: https://variantgrid.com

VEP: https://ensembl.org/info/docs/tools/vep

Human research participants

Policy information about studies involving human research participants and Sex and Gender in Research.

Reporting on sex and gender	Sex and gender are reported in line with current laboratory standards. No sex or gender-based analyses of the data have been performed.
Population characteristics	Demographic information (particularly relating to age) is reported in the manuscript. Majority of participants recruited were under 1 month of age (Fig. 1C). No age-dependent analysis of the data has been performed. There was no inclusion or exclusion criteria based on this or other characteristics (e.g. ethnicity) and we expect the cohort to be representative of the Australian population.
Recruitment	Participants were recruited prospectively using patient eligibility criteria outlined in the manuscript. They were assessed as likely to have an underlying monogenic disorder by the referring clinical geneticist and a group of study investigators. The study provided formal onboarding sessions for new sites and specific guidelines for patient assessment and recruitment, as well as a national expert panel to ensure similar patients were approved from all recruiting sites. Despite efforts to have nationally consistent recruitment, there may have been some variability in individual clinician practice in terms of patient assessment which could impact diagnostic yield.
Ethics oversight	The Australian Genomics Acute Care study has Human Research Ethics Committee approval from Melbourne Health (HREC/16/MH/251).

Note that full information on the approval of the study protocol must also be provided in the manuscript.

Field-specific reporting

Please select the one below that is the best fit for your research. If you are not sure, read the appropriate sections before making your selection.

☒ Life sciences ☐ Behavioural & social sciences ☐ Ecological, evolutionary & environmental sciences

For a reference copy of the document with all sections, see nature.com/documents/nr-reporting-summary-flat.pdf

Life sciences study design

All studies must disclose on these points even when the disclosure is negative.

Sample size	Eligible patients were recruited prospectively into the study based on study eligibility criteria. Sample size was determined by the available funding.
Data exclusions	No data were excluded.
Replication	The study used clinically accredited genomic analysis, and as such replication was not required if the variant call reached QC requirements.
Randomization	All participants followed the same study pathway, so randomisation and blinding are not applicable to study design.
Blinding	All participants followed the same study pathway, so randomisation and blinding are not applicable to study design.

Reporting for specific materials, systems and methods

We require information from authors about some types of materials, experimental systems and methods used in many studies. Here, indicate whether each material, system or method listed is relevant to your study. If you are not sure if a list item applies to your research, read the appropriate section before selecting a response.

Materials & experimental systems

n/a	Involved in the study	
☐	☒	Antibodies
☐	☒	Eukaryotic cell lines
☒	☐	Palaeontology and archaeology
☒	☐	Animals and other organisms
☐	☒	Clinical data
☒	☐	Dual use research of concern

Methods

n/a	Involved in the study	
☒	☐	ChIP-seq
☒	☐	Flow cytometry
☒	☐	MRI-based neuroimaging

Antibodies

Antibodies used	anti-NUP214 antibody (rabbit, Abcam cat. #ab70497), Alexa Fluor 488 conjugated goat anti-rabbit antibody (ThermoFisher Scientific, Can. #A-11008), GAPDH (Cat. #G9545, Sigma Aldrich)
Validation	All utilised antibodies are commercially available and validated by the respective manufacturers. Relevant positive and negative controls were included as described in the manuscript. Antibodies were validated by the manufacturer against a titrated amount of control human fibroblast lysate and used according to manufacturer's protocols.

Eukaryotic cell lines

Policy information about cell lines and Sex and Gender in Research

Cell line source(s)	Patient-derived primary fibroblast cell line was established for participant A1131048 who was karyotypically female.
Authentication	The NUP214 variants were Sanger-confirmed in the patient-derived cell line to authenticate it.
Mycoplasma contamination	Not performed.
Commonly misidentified lines (See ICLAC register)	No commonly misidentified lines were used.

Clinical data

Policy information about clinical studies

All manuscripts should comply with the ICMJE guidelines for publication of clinical research and a completed CONSORT checklist must be included with all submissions.

Clinical trial registration	N/A, not a clinical trial
Study protocol	Included as Methods in the manuscript.
Data collection	Study sites and period of data collection are included as Methods in the manuscript.

Outcomes

The primary outcome is diagnostic yield.
