# Supplementary figures and images for: Integrated multi-omics for rapid rare disease diagnosis on a national scale
Source: Nat Med. 2023 Jun 8;29(7):1681–91. doi: 10.1038/s41591-023-02401-9 (PMC10353936; doi:10.1038/s41591-023-02401-9)

Source data

Fig 5b – WB NUP214 and GAPDH.

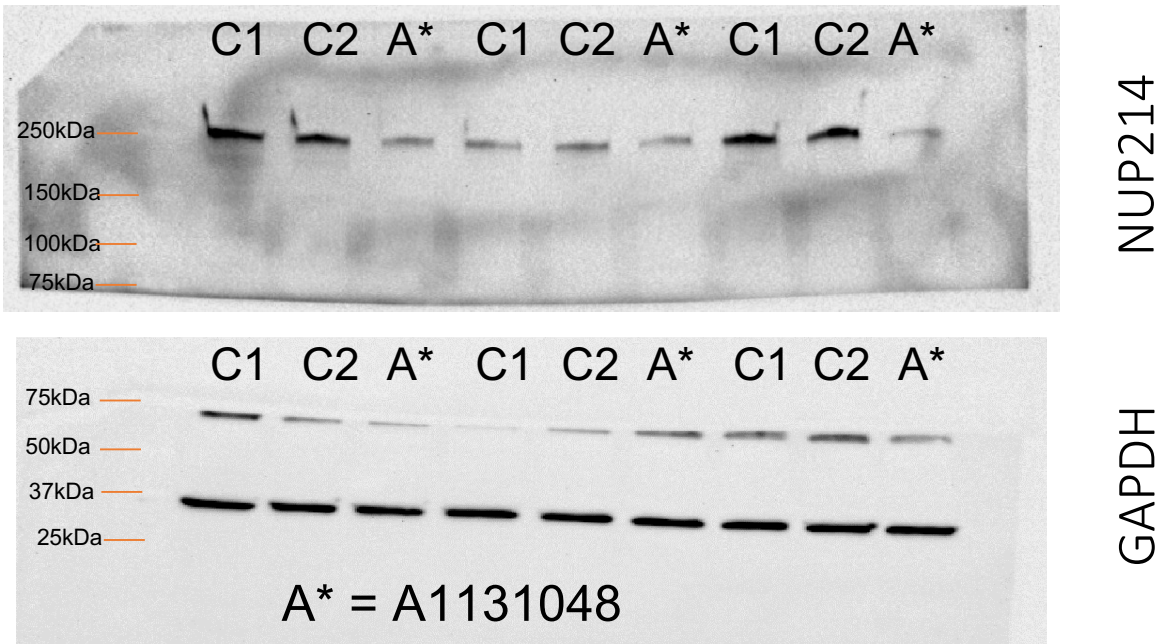

Supplement: Source Data Fig. 5 — Unprocessed western blots. [file 41591_2023_2401_MOESM3_ESM.pdf]
